# Wind energy potential assessment based on wind speed, its direction and power data

**DOI:** 10.1038/s41598-021-96376-7

**Published:** 2021-08-19

**Authors:** Zhiming Wang, Weimin Liu

**Affiliations:** 1 School of Mechanical and Electronic Engineering, Lanzhou University of Technology, Lanzhou, 730050 China; 2 Gansu Province Special Equipment Inspection and Testing Institute, Lanzhou, 730050 China

**Keywords:** Wind energy, Mechanical engineering

## Abstract

Based on wind speed, direction and power data, an assessment method of wind energy potential using finite mixture statistical distributions is proposed. Considering the correlation existing and the effect between wind speed and direction, the angular-linear modeling approach is adopted to construct the joint probability density function of wind speed and direction. For modeling the distribution of wind power density and estimating model parameters of null or low wind speed and multimodal wind speed data, based on expectation–maximization algorithm, a two-component three-parameter Weibull mixture distribution is chosen as wind speed model, and a von Mises mixture distribution with nine components and six components are selected as the models of wind direction and the correlation circular variable between wind speed and direction, respectively. A comprehensive technique of model selection, which includes Akaike information criterion, Bayesian information criterion, the coefficient of determination *R*^2^ and root mean squared error, is used to select the optimal model in all candidate models. The proposed method is applied to averaged 10-min field monitoring wind data and compared with the other estimation methods and judged by the values of *R*^2^ and root mean squared error, histogram plot and wind rose diagram. The results show that the proposed method is effective and the area under study is not suitable for wide wind turbine applications, and the estimated wind energy potential would be inaccuracy without considering the influence of wind direction.

## Introduction

Energy consumption increases dramatically with the rapid development of society and economy. Wind energy has attracted more and more attention because of its advantages such as abundance, renewability, natural cleanness, low cost and little negative impact on the environment, and has been used as an alternative to fossil fuels^1,2^. Therefore, the application of wind energy has already been selected as an important measure for the sustainable development of resources and environment all over the world. Wind energy also plays an important role in national economic growth which creates more employment opportunities^3,4^. Before developing wind power in a certain site, including the design, arrangement and condition monitoring of wind turbine systems, it is necessary to assess the wind energy potential and wind characteristics^5^.

As wind energy is proportional to the cube of wind speed, this means that even a small increase in wind speed results in a large increase in wind energy, therefore, the most important factor affecting wind energy is wind speed. But wind speed is not constant, it always fluctuates with the varying of air temperature over a period of time in different geographic locations and seasons. In this case, we can take wind speed as a random variable and describe it by a probability density function (pdf). Therefore, the pdf of wind speed becomes an important basis for evaluating wind energy potential and wind stochastic characteristics^6^. If the frequency distribution of wind speed is comprehensively expressed by an estimated pdf, the wind power density and wind energy output of wind turbines can be evaluated, which can help us make a reasonable decision whether to build a wind farm in the observed area or not, and reduce the uncertainties and the errors of wind power output estimation^7^. At the same time, the accurate estimated pdf can also help us to select an optimal wind energy conversion system and evaluate the reliability of generation system. Therefore, accurate evaluation of wind speed pdf is conducive to the prediction of wind energy potential and the selection of an optimal wind energy conversion system^8^.

There are families or groups of parametric models and nonparametric models describing wind characteristics. The parametric models are also divided into single and mixture distribution models. At present, the most widely used single distribution models include Gamma, Raleigh, Inverse Gaussian distribution, lognormal and Weibull distribution models^2–14^, etc. Among them, the two-parameter Weibull distribution model is often recognized as an effective model and is widely used in the field of wind industry to estimate wind energy potential mainly due to its simplicity. In some cases, the probability of calms (null wind speed) or the wind speed below 2 m/s is significant, and the two-parameter Weibull distribution performs poorly for a high percentage of null wind speed. While it can be observed that the three-parameter Weibull distribution gives a better result of energy calculation when the frequency of null wind speed is higher^4,13^. A wind turbine can generate electricity only when wind speed exceeds the cut-in speed. So Deep et al.^14^ pointed out that a three-parameter Weibull distribution must be used to model wind speed data between the cut-in and cut-out wind speeds, and the location parameter can be equivalent to the cut-in wind speed. On the other hand, when a distribution of wind speed is bimodal or multimodal, a single distribution model cannot perform well. In this case, some mixture distribution models^5,8,15–24^, which consist of several single distribution models (called components), are used, such as the Weibull-Weibull mixture, the Gamma-Weibull mixture, the truncated Normal-Weibull mixture, etc.

Wind direction is also an important aspect affecting the wind energy when evaluating wind characteristics in a certain area. Gugliani et al.^25^ argued that it is futile to study wind power at a particular site when wind direction was not analyzed. Han and Chu^26^ also considered that the available wind resources change with the wind direction, especially in the low-speed and complex terrain areas. Therefore, the mixture model of von Mises (voM) distribution is commonly used for modelling wind direction data^27–32^. Besides the voM distribution, the other circular statistical distributions including the uniform distribution, wrapped-normal distribution and wrapped-Cauchy distribution, etc. are also suitable for modelling analysis of wind direction. The research results showed that a mixture of voM distributions provided a flexible model for studies of wind direction that have several modes^27,28^. However, compared to wind speed model, the statistical modeling of wind direction is more difficult and complex. On the other hand, wind speed and wind direction are dependent random variables, wind speed has a directional characteristic and wind direction can complement information about wind speed in analyses of wind energy potential^28,29^. Therefore, in the past fewer decades, several bivariate distribution models for simultaneously describing wind speed and direction, which named joint distribution models of wind speed and direction, have been proposed by different authors^33–40^. Carta et al.^33^ presented a joint distribution from two marginal distributions, a single truncated from below Normal-Weibull mixture distribution for wind speed and a finite mixture of voM distributions for wind direction. Erdem and Shi^35^ given a comparison of bivariate distribution models for analyzing wind speed and direction data of multiple sites in North Dakota, USA.

The methods commonly used to estimate model parameters of wind speed or wind direction are the graphical method or least square method (LSM), maximum likelihood estimate (MLE), modified maximum likelihood estimate (MMLE), empirical method (EmM), moment method (MoM), power density method (PDM), energy pattern factor method (EPFM), equivalent energy method (EEM) and copula-based approach, etc. Non-parametric model methods were also proposed in literature. These methods include the minimum cross entropy (MCE) method^41^, maximum entropy principle method (MEPM)^42^, kernel density estimation^43–45^ and root-transformed local linear regression method^46^, etc.

In this study, to evaluate wind energy potential, the single and mixture of two-parameter and three-parameter Weibull distributions are used as candidate models for wind speed data, and a finite mixture of voM distributions is used for wind direction data. Based on MLE, the expectation–maximization (EM)^8,29,45,47^ optimization algorithm is applied to estimate model parameters of mixture distributions. As Carta et al.^27^ pointed out that although the mixture distributions enrich the modelling and have high degrees of fits, the model complexity increases with the increasing of more number of model parameters. Therefore, we use Akaike information criterion (AIC) and Bayesian information criterion (BIC) to select the optimal model, and adopt the coefficient of determination *R*^*2*^ and the root mean squared error (RMSE) to evaluate the goodness-of**-**fit of model. The number of components in mixture model does not need to be known in advance.

Therefore, the novelty and main contributions of this work can be summarized as follows: (1) For null or low wind speed and multimodal wind speed data, we use three-parameter Weibull mixture distributions to model its pdf, the number of components in mixture model can be optimally determined using a comprehensive technique of model selection. (2) Based on the wind power curve of a specified wind tribune, the wind energy output considering the effect of wind speed and direction simultaneously can be given. The effectiveness of the proposed method is verified by a real case.

The paper is organized as follows. In “Methodology” we give some details on the modelling for wind data using the Weibull and voM distributions, including parameter estimation, model selection and validation. The assessment of wind energy potential is described in “Wind power estimation”. While in “Case study” presents some information about the observed field and the statistical description for wind speed, its direction and wind power. Results and comparison with the observation data are presented with details in “Results and discussion”. Conclusions are drawn in the final and concluding section.

## Methodology

### Wind speed model with Weibull distribution

When the frequency of low wind speed, especially of null wind is significant, a three-parameter Weibull distribution can be used to model this wind speed data well and a more appropriate results can be obtained. The pdf of wind speed using the three-parameter Weibull distribution is given by^4,13^:

1 $$ f\left( {v;\eta ,\beta ,\gamma } \right) = \left( {\frac{\beta }{\eta }} \right)\left( {\frac{v - \gamma }{\eta }} \right)^{\beta - 1} \exp \left[ { - \left( {\frac{v - \gamma }{\eta }} \right)^{\beta } } \right] $$

where *v* is wind speed, *η* is the scale parameter (m/s), *η* > 0, *β* represents the shape parameter, *β* > 0, and *γ* is the position parameter, *γ* ≤ 0. When *γ* = 0, three-parameter Weibull distribution reduces to two-parameter Weibull distribution.

Mixture distributions are defined as linear combinations of two or several distributions. Therefore, there are more parameters needed to estimate for mixture distribution model than that of single distribution model. And parameter estimation of mixture distribution model is more complex and difficult. The MLE is one of the efficient methods to estimate model parameters.

Mixture model is a weighted sum with several single models^16–19,22–24^, therefore, a pdf of mixture distribution model for *m*-component three-parameter Weibull distributions can be given by

2 $$ f_{V} \left( {v;w_{i} ,\eta_{i} ,\beta_{i} ,\gamma_{i} } \right) = \sum\limits_{i = 1}^{m} {w_{i} } f\left( {v;\eta_{i} ,\beta_{i} ,\gamma_{i} } \right) = \sum\limits_{i = 1}^{m} {w_{i} } \left( {\frac{{\beta_{i} }}{{\eta_{i} }}} \right)\left( {\frac{{v - \gamma_{i} }}{{\eta_{i} }}} \right)^{{\beta_{i} - 1}} \exp \left[ { - \left( {\frac{{v - \gamma_{i} }}{{\eta_{i} }}} \right)^{{\beta_{i} }} } \right] $$

where *w*_*i*_ is the weight coefficient of the *i*th component, and must satisfy the following conditions:

3 $$0 \le w_{i} \le 1 \, \left( {i = 1,...,m} \right){\text{ and }}\sum\limits_{i = 1}^{m} {w_{i} = 1}$$

Given the *n* observed wind speed data **V** = [*v*_1_,*v*_2_,…,*v*_*n*_], the likelihood function on **V** can be obtained by

4 $$L\left( {{\mathbf{V;\Lambda }}} \right) = \prod\limits_{j = 1}^{n} {\sum\limits_{i = 1}^{m} {\left\{ {w_{i} \left( {\frac{{\beta_{i} }}{{\eta_{i} }}} \right)\left( {\frac{{v_{j} - \gamma_{i} }}{{\eta_{i} }}} \right)^{{\beta_{i} - 1}} \exp \left[ { - \left( {\frac{{v_{j} - \gamma_{i} }}{{\eta_{i} }}} \right)^{{\beta_{i} }} } \right]} \right\}} }$$

where **Λ** = [***w*, *η*, *β*, *γ***] are unknown model parameters of wind speed.

Then the log-likelihood function can be given as follows:

5 $$\ln L\left( {{\mathbf{V;\Lambda }}} \right) = \sum\limits_{j = 1}^{n} {\ln } \sum\limits_{i = 1}^{m} {\left\{ {w_{i} \left( {\frac{{\beta_{i} }}{{\eta_{i} }}} \right)\left( {\frac{{v_{j} - \gamma_{i} }}{{\eta_{i} }}} \right)^{{\beta_{i} - 1}} \exp \left[ { - \left( {\frac{{v_{j} - \gamma_{i} }}{{\eta_{i} }}} \right)^{{\beta_{i} }} } \right]} \right\}}$$

Due to the complexity of the log-likelihood function, the model parameters cannot be got by taking the partial derivatives of log-likelihood function with respect to each parameter and setting them equal to zero. Therefore, the log-likelihood function is maximized directly to estimate the model parameters. Unfortunately, there is no closed-form expression for computing them, it only can be numerically estimated. Therefore, a numerical method, such as EM algorithm is needed to find the maximum likelihood estimates of the parameters.

EM algorithm is an iterative method for finding maximum likelihood or maximum a posteriori estimates of model parameters for statistical distribution from a given data set. It proceeds iteratively in two steps, the expectation (E) step and maximization (M) step. In the E-step, a function for the expectation of the log-likelihood is created, and the hidden variables or missing data are estimated given the observed data and current estimator of model parameters. In the M-step, the likelihood function defined by the previous E-step is maximized to obtain new parameter estimations under the assumption that the hidden variables or missing data are known. It should be noted that the E-step and M-step in EM algorithm are performed iteratively until the algorithm converges. Initial values are required for the iterative procedure. In this study, the population is divided into *m* components, and the estimated parameters are assumed to have the same values with a single Weibull distribution for each component. These estimates are considered as initial values for the iterative procedure.

For an *m*-component mixture model, Eq. (6) is used to find the mean *c*_1_, variance *c*_2_, the coefficients of skewness *c*_3_ and kurtosis *c*_4_ of wind speed, respectively.

6 $$E\left( {v^{d} } \right) = \sum\limits_{i = 1}^{m} {w_{i} \int_{0}^{\infty } {v^{d} f_{V} \left( {v;w_{i} ,\eta_{i} ,\beta_{i} ,\gamma_{i} } \right)} } {\text{d}}v, \, d = 1,2,3,4$$

When the model parameters of three-parameter Weibull mixture distributions are known, based on Eq. (6), the values of *c*_1_, *c*_2_, *c*_3_ and *c*_4_ can be obtained as follows, respectively:

7 $$c_{1} = \sum\limits_{i = 1}^{m} {w_{i} \left[ {\gamma_{i} + \eta_{i} \Gamma \left( {1 + \frac{1}{{\beta_{i} }}} \right)} \right]}$$

8 $$c_{2} = \sum\limits_{i = 1}^{m} {w_{i} \left[ {\eta_{i}^{2} \left( {\Gamma \left( {1 + \frac{2}{{\beta_{i} }}} \right) - \Gamma^{2} \left( {1 + \frac{1}{{\beta_{i} }}} \right)} \right)} \right]}$$

9 $$c_{3} = \sum\limits_{i = 1}^{m} {w_{i} \left[ {\frac{{\Gamma \left( {1 + \frac{3}{{\beta_{i} }}} \right){ - }3\Gamma \left( {1 + \frac{1}{{\beta_{i} }}} \right)\Gamma \left( {1 + \frac{2}{{\beta_{i} }}} \right){ + 2}\Gamma^{3} \left( {1 + \frac{1}{{\beta_{i} }}} \right)}}{{\left[ {\Gamma \left( {1 + \frac{2}{{\beta_{i} }}} \right){ - }\Gamma^{2} \left( {1 + \frac{1}{{\beta_{i} }}} \right)} \right]^{{{3 \mathord{\left/ {\vphantom {3 2}} \right. \kern-\nulldelimiterspace} 2}}} }}} \right]}$$

10 $$c_{4} = \left( {\sum\limits_{i = 1}^{m} {w_{i} \left[ {\frac{{\Gamma \left( {1 + \frac{4}{{\beta_{i} }}} \right){ - 4}\Gamma \left( {1 + \frac{1}{{\beta_{i} }}} \right)\Gamma \left( {1 + \frac{3}{{\beta_{i} }}} \right){ + 6}\Gamma \left( {1 + \frac{2}{{\beta_{i} }}} \right)\Gamma^{2} \left( {1 + \frac{1}{{\beta_{i} }}} \right){ - 3}\Gamma^{4} \left( {1 + \frac{1}{{\beta_{i} }}} \right)}}{{\left[ {\Gamma \left( {1 + \frac{2}{{\beta_{i} }}} \right){ - }\Gamma^{2} \left( {1 + \frac{1}{{\beta_{i} }}} \right)} \right]^{2} }}} \right]} } \right){ - }3$$

### Wind direction model with von Mises distribution

For the assessment of wind direction, the voM distribution is used. Consider a random variable *θ* following the voM distribution, the corresponding pdf is^27,28^

11 $$ f\left( {\theta ;\mu ,\alpha } \right) = \frac{{\exp \left[ {\alpha \cos \left( {\theta - \mu } \right)} \right]}}{{2\pi I_{0} \left( \alpha \right)}}; \, 0 \le \theta \le 2\pi $$

where *θ* is wind direction in radians units, *μ* denotes location parameter or mean direction on the circle, 0 ≤ *μ* ≤ 2*π*, *α* represents concentration parameter, *α* ≥ 0, and *I*_0_ (*α*) is the modified Bessel function of the first kind of order zero, given by^27,28^

12 $$ I_{0} \left( \alpha \right) = \frac{1}{2\pi }\int_{0}^{2\pi } {\exp \left( {\alpha \cos \theta } \right)} {\text{d}}\theta = \sum\limits_{r = 0}^{\infty } {\frac{1}{{\left( {r!} \right)^{2} }}} \left( {\frac{\alpha }{2}} \right)^{2r} $$

When wind direction has several modes or prevailing wind directions, the distribution of wind direction comprises a finite mixture of voM distributions. Thus, based on Eq. (11), the corresponding pdf of mixture distribution model can be given by

13 $$ f_{\Theta } \left( {\theta ;p_{i} ,\mu_{i} ,\alpha_{i} } \right) = \sum\limits_{i = 1}^{k} {p_{i} } f\left( {\theta ;\mu_{i} ,\alpha_{i} } \right) = \sum\limits_{i = 1}^{k} {\frac{{p_{i} }}{{2\pi I_{0} \left( {\alpha_{i} } \right)}}\exp \left[ {\alpha_{i} \cos \left( {\theta - \mu_{i} } \right)} \right]} $$

where *k* is the number of components and *p*_*i*_ is the weight coefficient of the *i*th component that sum to one, given by

14 $$0 \le p_{i} \le 1 \, \left( {i = 1,...,k} \right){\text{ and }}\sum\limits_{i = 1}^{k} {p_{i} = 1}$$

The mixture of voM distributions corresponds to the weighted sum of several voM distributions. It is thus suitable for the statistical description of multimodal datasets. Given the *n* observed wind direction data **Θ** = [*θ*_1_, *θ*_2_, …, *θ*_*n*_], the likelihood function on **Θ** is given by

15 $$L\left( {{\mathbf{\Theta ;\Delta }}} \right) = \prod\limits_{j = 1}^{n} {\sum\limits_{i = 1}^{k} {\left\{ {\frac{{p_{i} \exp \left[ {\alpha_{i} \cos \left( {\theta_{j} - \mu_{i} } \right)} \right]}}{{2\pi I_{0} \left( {\alpha_{i} } \right)}}} \right\}} }$$

where **Δ** = [***p*, *μ*, *α***] are unknown model parameters of wind direction. Then the log-likelihood function is computed by the following expression:

16 $$\ln L\left( {{\mathbf{\Theta ;\Delta }}} \right) = \sum\limits_{j = 1}^{n} {\ln } \sum\limits_{i = 1}^{k} {\left\{ {\frac{{p_{i} \exp \left[ {\alpha_{i} \cos \left( {\theta_{j} - \mu_{i} } \right)} \right]}}{{2\pi I_{0} \left( {\alpha_{i} } \right)}}} \right\}}$$

The model parameters can also be estimated by the EM algorithm for maximum likelihood estimation^29,47^.

If the model parameters of the voM mixture distributions are known, the mean *b*_1_ and variance *b*_2_ can also be got as follows, respectively:

17 $$ b_{1} = \sum\limits_{i = 1}^{k} {p_{i} \mu_{i} } $$

18 $$b_{2} = \sum\limits_{i = 1}^{k} {p_{i} \left[ {1{ - }\frac{{I_{1} \left( {\alpha_{i} } \right)}}{{I_{0} \left( {\alpha_{i} } \right)}}} \right]}$$

### Joint distribution model of wind speed and direction

Based on Eq. (2), the corresponding cumulative distribution function (CDF) for wind speed is given as follows:

19 $$ F_{V} \left( {v;w_{i} ,\eta_{i} ,\beta_{i} ,\gamma_{i} } \right) = \int_{0}^{v} {f_{V} \left( {v^{\prime}} \right)} {\text{d}}v^{\prime} = \sum\limits_{i = 1}^{m} {w_{i} } \left\{ {1 - \exp \left[ { - \left( {\frac{{v - \gamma_{i} }}{{\eta_{i} }}} \right)^{{\beta_{i} }} } \right]} \right\} $$

Equation (13) can also be expressed as a series of Bessel functions and given by.

20 $$f_{\Theta } \left( {\theta ;p_{i} ,\mu_{i} ,\alpha_{i} } \right) = \sum\limits_{i = 1}^{k} {\left[ {\frac{{p_{i} }}{{2\pi I_{0} \left( {\alpha_{i} } \right)}}\left( {I_{0} \left( {\alpha_{i} } \right) + 2\sum\nolimits_{q = 1}^{\infty } {I_{q} \left( {\alpha_{i} } \right)\cos \left[ {q\left( {\theta - \mu_{i} } \right)} \right]} } \right)} \right]}$$

where *I*_*q*_ (*α*) is the modified Bessel function of the first kind of order *q*, whose expression is given by

21 $$I_{q} \left( \alpha \right) = \sum\limits_{r = 0}^{\infty } {\frac{1}{{r!\left( {q + r} \right)!}}} \left( {\frac{\alpha }{2}} \right)^{q + 2r} , \, q = 1,2, \cdots$$

Therefore, using Eq. (20), the CDF for wind direction can be obtained as follows:

22 $$ F_{\Theta } \left( {\theta ;p_{i} ,\mu_{i} ,\alpha_{i} } \right) = \int_{0}^{\theta } {f_{\Theta } \left( {\theta^{\prime}} \right)} {\text{d}}\theta^{\prime} = \sum\limits_{i = 1}^{k} {\frac{{p_{i} }}{{2\pi I_{0} \left( {\alpha_{i} } \right)}}} \left\{ {\theta I_{0} \left( {\alpha_{i} } \right){ + }2\sum\nolimits_{q = 1}^{\infty } {\frac{{I_{q} \left( {\alpha_{i} } \right)\sin \left[ {q\left( {\theta - \mu_{i} } \right)} \right]}}{q}} } \right\} $$

The joint angular-linear pdf of wind speed and direction is then given as^33^

23 $$f_{V,\Theta } \left( {v,\theta } \right) = 2\pi g\left( \zeta \right)f_{V} \left( v \right)f_{\Theta } \left( \theta \right); \, 0 \le \theta \le 2\pi ; \, - \infty \le v \le \infty$$

where *g*(*ζ*) is the correlation pdf of circular variable *ζ* between wind speed *v* and direction *θ*, and *ζ* given by^33^

24 $$\zeta = 2\pi \left[ {F_{V} \left( v \right) - F_{\Theta } \left( \theta \right)} \right]$$

Using the above definitions, the values of *ζ*_*j*_ can be obtained for each pair of values of wind speed *v*_*j*_ and direction *θ*_*j*_ (*j* = 1, 2, …, *n*) from a sample of size *n* given as^33^

25 $$\zeta_{j} = \left\{ \begin{gathered} 2\pi \left[ {F_{V} \left( {v_{j} } \right) - F_{\Theta } \left( {\theta_{j} } \right)} \right]{, }F_{V} \left( {v_{j} } \right) \ge F_{\Theta } \left( {\theta_{j} } \right) \hfill \\ 2\pi \left[ {F_{V} \left( {v_{j} } \right) - F_{\Theta } \left( {\theta_{j} } \right){ + }1} \right], \, F_{V} \left( {v_{j} } \right) < F_{\Theta } \left( {\theta_{j} } \right) \hfill \\ \end{gathered} \right.$$

Therefore, based on Eqs. (2), (13), (19), (22) and (24), the pdf *g***(***ζ***)** of the variable *ζ* can also be described by a mixture of voM distributions.

### Selection of optimal model

For mixture distribution model, the selection of the number of components is important. The log-likelihood cannot be directly used for selecting the number of components, since the log-likelihood value increases with the number of components. The best models of wind speed and direction are selected by two information criteria, which are named the Akaike information criterion (AIC)^12,25,39,47^ and Bayesian information criterion (BIC)^37^. The values of AIC and BIC are defined as

26 $${\text{AIC}} = - 2\max \ln L + 2l,{\text{ BIC}} = - 2\max \ln L + l\ln n$$

where *l* is the number of estimated parameters, *n* is the number of all observations, and maxln*L* is the maximized log-likelihood. In this study, the number of unknown parameters *l* = 4* m*-1 for *m* components three-parameter Weibull mixture wind speed model, and *l* = 3* m*-1 for *m* components voM mixture wind direction model. The maxln*L* can be obtained by Eqs. (5) and (16) with EM algorithm. AIC and BIC contain the penalization terms that take into account the number of model parameters and all observed values to counterbalance the maximized log-likelihood. To avoid overfitting, AIC penalizes model for its complexity only with model parameters, while BIC imposes a greater penalty for additional parameter than AIC. So, AIC and BIC give a comprehensive balance in order to find a good tradeoff between the goodness-of-fit and the complexity of the model, and avoid the risk of choosing a complex model with a poor generalization. The smaller the values of AIC and BIC are, the higher the fit accuracy of model is. Therefore, the number of components does not need to be known in advance.

### Validation of model

The coefficient of determination (*R*^2^) and the root mean squared error (RMSE) are used to judge the goodness-of**-**fit of different mixture models to wind speed and direction data, because it quantifies the correlation between the observed and the estimated probability density according to a particular distribution.

The coefficient of determination is the square of the correlation between the estimated values and observed values, and is defined by^13,16^

27 $$ R^{2} = 1 - \frac{{\sum\nolimits_{j = 1}^{n} {\left( {y_{j} - \hat{y}_{j} } \right)^{2} } }}{{\sum\nolimits_{j = 1}^{n} {\left( {y_{j} - y_{m} } \right)^{2} } }} $$

where *y*_*j*_ is the *j*th observed value, *y*_*m*_ is the mean value of all observations, and $\hat{y}_{j}$ is the *j*th estimated value, respectively. A large value of *R*^2^ indicates the proposed distribution fits the wind speed data set well in all candidate models.

Unlike the value of *R*^2^, a high RMSE value indicates a poor fit. The smaller the values of RMSE are, the better the proposed distribution function approximates the observed data. It can be given by^13,16^

28 $${\text{RMSE}} = \sqrt {\frac{{\sum\nolimits_{j = 1}^{n} {\left( {y_{j} - \hat{y}_{j} } \right)^{2} } }}{n}}$$

## Wind energy estimation

The density of air changes slightly with air temperature and with altitude at a potential site, supposed that air density is constant, based on a pdf *f*(*v*) of wind speed *v* at a height for a wind turbine, in theory, the wind power *P*(*v*) in W can be computed by^10,12^

29 $$P\left( v \right) = \frac{1}{2}\rho A\int_{0}^{\infty } {v^{3} f\left( v \right){\text{d}}v}$$

where *ρ* is air density (kg/m^3^), *A* is the sweep area of a wind turbine rotor (m^2^). Therefore, the wind power density *p*(*v*) in W/m^2^ can be given as

30 $$p\left( v \right) = \frac{P\left( v \right)}{A}{ = }\frac{1}{2}\rho \int_{0}^{\infty } {v^{3} f\left( v \right){\text{d}}v}$$

Using the real wind speed values of time series wind data, the effective wind power density, denoted as *p*_*e*_(*v*) in W/m^2^ is estimated as follows^12,22,42^:

31 $$p_{e} \left( v \right) = \frac{1}{2}\rho \sum\limits_{j = 1}^{{n_{e} }} {\frac{{v_{j}^{3} }}{{n_{e} }}}$$

where *n*_*e*_ is the number of effective wind speed, which lies between the cut-in wind speed *v*_in_ and cut-out wind speed *v*_out_ of wind turbine.

If the wind direction influence is ignored, the wind power density *p*(*v*) for a specified wind turbine can be obtained numerically by

32 $$p\left( v \right) = \frac{1}{2}\rho \int_{{v_{{{\text{in}}}} }}^{{v_{{{\text{out}}}} }} {v^{3} f\left( v \right){\text{d}}v}$$

When considering the effect of wind direction, similar to Eq. (32), the wind power density *p*(*v*,*θ*) at different wind speed and direction can be computed numerically by^37^

33 $$p\left( {v,\theta } \right) = \frac{1}{2}\rho \int_{0}^{2\pi } {\int_{{v_{{{\text{in}}}} }}^{{v_{{{\text{out}}}} }} {v^{3} } } f_{V,\Theta } \left( {v,\theta } \right){\text{d}}v{\text{d}}\theta$$

The power curve gives a relation between the output power *P*_*w*_(*v*) and wind speed *v*, and this relation can be expressed by a polynomial function of degree *u* as follows^48–50^:

34 $$ P_{w} \left( v \right) = \left\{ \begin{gathered} 0 \, v < v_{{{\text{in}}}} ,v \ge v_{{{\text{out}}}} \hfill \\ a_{0} + \sum\limits_{i = 1}^{u} {a_{i} v^{i} } \, v_{{{\text{in}}}} \le v < v_{r} \hfill \\ P_{r} \, v_{r} \le v < v_{{{\text{out}}}} \hfill \\ \end{gathered} \right. $$

where *P*_*w*_(*v*) is the output power, *P*_*r*_ denotes the rated power of wind turbine, *v*_*r*_ represent the rated speed, *a*_0_ and *a*_*i*_ are the regression constants which can be obtained using a polynomial regression method.

Based on the power curve of a specified wind tribune, the wind energy output *E*(*v*,*θ*) with a joint pdf of wind speed and direction within a period of time *t* can be calculated numerically by^33^

35 $$E\left( {v,\theta } \right) = t\int_{0}^{2\pi } {\left[ {\int_{{v_{{{\text{in}}}} }}^{{v_{{{\text{out}}}} }} {P_{w} \left( v \right)f_{V,\Theta } \left( {v,\theta } \right){\text{d}}v} } \right]} {\text{d}}\theta$$

### Case study

Wind speeds are continuously acquired for a significant time, usually not less than one year. Therefore, the data used in this study were collected in a period of one year (January 1, 2019 to December 31, 2019) and measured at a height of 30 m above the ground level from the Maling Mountain wind farm (34°31.4′ N and 118°45.7′ E) located in Jiangsu Province, China (see Supplementary Table S1 on line). The Maling Mountain wind farm is selected due to the fact that the histogram of wind speed indicates that the frequency of 0–2 m/s wind speed range is 8.25% for this station (see Supplementary Table S2 on line). The percentage of null wind speed or calms (0–0.2 m/s) at this wind farm is 0.50%. Wind direction data are circular because they are recorded in terms of degrees, from 0° clockwise to 360°. However, for modeling convenient, the data are converted into radian units. After removing some abnormal and unreasonable data such as the missing data by sensor fault, measurement error data and low temporal resolution data, a total of 47,084 wind data are collected. The statistical description of wind speed, its direction and wind power data for 1.8 MW wind turbine are shown in Table 1.

**Table 1:** Statistical description of wind speed, its direction and wind power data. The data records corresponding to the periods of averaged 10 min each and containing wind speed, wind direction and wind power data for 1.8 MW wind turbine collected from supervisory control and data acquisition (SCADA) systems.

	Wind speed (m/s)	Wind direction (rad)	Wind power (kW)
Mean	4.3902	2.7238	367.6699
Variance	3.3240	2.7547	172,406.7517
Skewness	0.8119	0.3562	2.1340
Kurtosis	2.4538	− 0.9237	1.5765
Min	0	0.0003	− 4.0816
Max	18.9878	6.2830	1842.7400
Number	47,084	47,084	47,084

## Results and discussion

The estimated parameters with different methods for the Weibull distribution wind speed model are shown in Table 2.

**Table 2:** Parameter estimation results of wind speed model with 2-parameter and 3-parameter Weibull distributions using different methods. Two-parameter and three-parameter are abbreviated as 2-p and 3-p, respectively.

Model	Method	Parameter				
		*i*	*w*_*i*_	*η*_*i*_	*β*_*i*_	*γ*_*i*_
Single model	2-p LSE	1	1	5.8556	1.3907	0
	2-p MLE	1	1	4.9040	2.4252	0
	2-p MoM	1	1	4.9436	2.5850	0
	3-p EM	1	1	5.2625	3.2919	− 0.2326
Mixture model	3-p EM (*m* = 2)	**1**	**0.6525**	**4.8655**	**3.6591**	**0**
		**2**	**0.3475**	**6.0169**	**2.3126**	**− 0.1415**
	3-p EM (*m* = 3)	1	0.0010	2.0090	1.1026	− 0.2019
		2	0.3241	5.0169	1.4774	− 0.1440
		3	0.6749	6.0114	1.5496	− 0.0010

The results of the best model are in bold

A comparison results with different methods for the Weibull distribution wind speed model are also given in Table 3. Based on the information criteria of AIC and BIC, we can see that the fit accuracy of mixture model is higher than that of single model, and the accuracy of LSE is the lowest. For single model, three-parameter model has a higher fit accuracy than that of two-parameter model. The reason is that the former considers the null wind speed. However, for mixture model, as the number of components increases, the accuracy of the model decreases. Therefore, we select two-component three-parameter Weibull mixture model as the optimal model for wind speed. This result is also confirmed by the values of RMSE and *R*^2^. Because a lower value of RMSE and a higher value of *R*^2^ indicate a better fit of the model to the data, and two-component three-parameter Weibull mixture model has the lowest value of RMSE and highest value of *R*^2^ in all candidate models. It is worth noting that in the case of multi-modal data, the fitting, modeling and analysis for a statistical distribution are more accurate than an ordinary regression analysis, a value of *R*^2^ only more than 0.7 is not sufficient^28^. In this study, this value is high, it is 0.9944.

**Table 3:** Comparison results with different methods for Weibull wind speed model. Two-parameter and three-parameter are abbreviated as 2-p and 3-p, respectively. Based on a comprehensive technique of model selection, two-component three-parameter Weibull mixture model is selected as the optimal model for wind speed.

Model	Method	ln *L*	AIC	BIC	RMSE	*R*^2^
Single model	2-p LSE	− 48,145.4007	96,294.8014	96,312.3208	0.0520	0.8505
	2-p MLE	− 21,961.6692	43,927.3384	43,944.8578	0.0211	0.9257
	2-p MoM	− 18,957.1679	37,918.3358	37,935.8552	0.0169	0.9526
	3-p EM	− 11,746.7293	23,499.4586	23,525.7377	0.0080	0.9894
Mixture model	3-p EM (*m* = 2)	**− 9309.0261**	**18,632.0522**	**18,693.3700**	**0.0058**	**0.9944**
	3-p EM (*m* = 3)	− 9450.2055	18,922.4110	19,018.7676	0.0484	0.8098

The results of the best model are in bold

**Figure 1:**
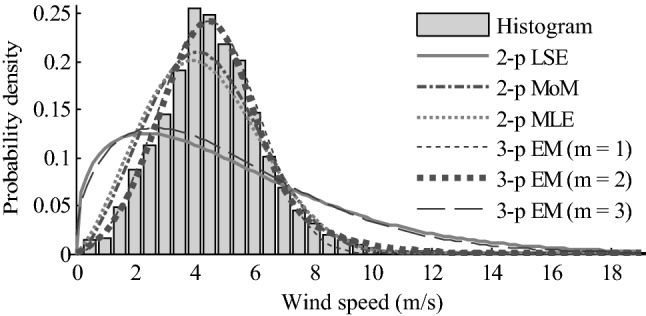
Histogram of wind speeds. The bin size of wind speed interval is 0.5 m/s. The bold dotted line of two-component three-parameter Weibull mixture distribution with EM algorithm (3-p EM, m = 2) gives the best fitting accuracy to the histogram of wind speed in all candidate distribution. Figure created using Matlab R2014a (https://www.mathworks.com).

The fit results of different models are given in Fig. 1. It also shows that two-component three-parameter Weibull mixture model adequately fits the frequency histogram of wind speed well than other models. The fit accuracy of three-component three-parameter Weibull mixture model and two-parameter Weibull single model with LSE method is the lowest in all models. To fit the sample histogram, the wind speed and direction intervals must be given. In this case, the bin size of wind speed and direction interval is selected as 0.5 m/s and 10° (0.1745 rad)^7,21,27,29,35^, which is often considered to be reasonable in wind energy analyses. The speed interval of 0.5 m/s is also close to the value of 2 km/h (approximates 0.56 m/s) used by Deep et al.^14^ and Gugliani et al^25^. They concluded that the class width of 2 km/h gives a minimum error for modelling wind speed data.

A statistical description of wind speed data can give some useful information on wind speed, such as mean, variance, symmetry and flatness. We can also calculate them using Eqs. (7)-(10) and compare them with the statistical description in Table 1 to verify the rightness and effectiveness of our proposed method. Therefore, using Eqs. (7)-(10), the estimated values of the mean, variance, the coefficients of skewness and kurtosis for wind speed can be obtained, respectively, as follows: *c*_1_ = 4.6666, *c*_2_ = 3.2406, *c*_3_ = 0.1470 and *c*_4_ = 2.8026. Compared to the real statistical values in Table 1, we can find that the relative errors of mean and variance are small, they are only 6.30% and 2.51%, respectively, and the estimated values of *c*_3_ > 0 and *c*_4_ < 3. All these indicate that the probability distribution of wind speed has a long and light right tail than a normal distribution. This result also agrees well with two-component three-parameter Weibull mixture distribution in Fig. 1.

Table 4 is the results of estimated parameters with different components for voM wind direction model.

**Table 4:** Parameter estimation results for mixture voM wind direction model. The complexity of wind direction model increases with the increasing number of components for mixture model. For example, for a wind direction mixture model with 9-component voM distributions, a total of 26 unknown parameters need to be estimated.

*k*	*p*	*α*	*μ*	*k*	*p*	*α*	*μ*	*k*	*p*	*α*	*μ*
1	1.0000	0.4329	2.0577	7	0.1952	2.7826	2.9787	9	**0.1729**	**5.8521**	**3.0405**
2	0.4725	0.0010	3.6480		0.0635	25.8134	0.5254		**0.1302**	**15.4270**	**0.5544**
	0.5275	0.8540	2.0127		0.4711	1.7217	1.6196		**0.0021**	**2.5792**	**2.2854**
3	0.7561	0.8822	2.0134		0.0010	4.3601	0.0010		**0.0010**	**4.3479**	**0.0010**
	0.0447	39.3747	0.5511		0.2218	2.1591	4.5968		**0.2297**	**2.4925**	**4.3874**
	0.1992	1.5674	4.7000		0.0464	12.1652	5.6580		**0.0969**	**6.0480**	**5.6471**
4	0.2908	1.1954	4.9078		0.0010	4.9832	1.4314		**0.1922**	**8.0584**	**2.1458**
	0.0542	38.0446	0.5274	8	0.1369	2.5621	3.1525		**0.1740**	**9.2402**	**1.3416**
	0.1466	4.0033	1.2829		0.2117	3.3017	0.7875		**0.0010**	**4.9909**	**1.5989**
	0.5084	1.2699	2.4422		0.3744	2.0779	2.0594	10	0.1709	5.8695	3.0507
5	0.4350	1.4041	2.6909		0.0010	4.3421	0.0010		0.1276	15.4501	0.5504
	0.0010	0.1277	0.0010		0.1568	2.1431	4.2825		0.0010	2.5804	2.2857
	0.2030	3.6102	1.5138		0.1172	3.4021	5.2958		0.0010	4.3455	0.0010
	0.0941	13.5594	0.5751		0.0010	4.9803	1.5391		0.2289	2.4971	4.3906
	0.2669	1.4701	4.9751		0.0010	4.9875	1.4903		0.0965	6.0483	5.6461
6	0.4295	1.5940	2.7449						0.1761	8.0646	2.1734
	0.1015	14.8627	0.5334						0.1424	9.2373	1.3224
	0.2346	3.4898	1.4698						0.0546	4.9903	1.5884
	0.0010	1.3469	0.9287						0.0010	4.9907	1.5990
	0.1467	3.6726	4.5639								
	0.0867	7.7691	5.6304								

The results of the best model are in bold

Table 5 is a comparison results with the different mixture models for wind direction. It can be observed that increasing the number of voM components from one to ten in the mixture distributions will increase the value of the *R*^2^ coefficient, which indicates a better fit to the data. However, when the number of components increases to ten, the value of the *R*^2^ coefficient does not increase any more, it is the same as the nine-component mixture model has. At the same time, a nine-component mixture model has the same value of RMSE as a ten-component mixture model. In this situation, how to select the best wind direction model? However, it is noticed that nine-component mixture model has lower values of AIC and BIC, so based on the information criteria of AIC and BIC, and the values of RMSE and *R*^2^, the best wind direction model would be selected using the comprehensive criteria of information and goodness-of-fit. It can be concluded that the most suitable model for wind direction at this station is a voM mixture model with nine-component distributions. Generally speaking, a voM mixture distribution with six components for the modelling of wind direction is enough, increasing the number of components of mixture distributions, the variations in value of *R*^2^ are not significant^27^. On the contrary, it would yield a complex model. Therefore, combining with the comprehensive criteria of model selection, an appropriate number of mixture distributions can be selected, which not only reduces the computational burden but also improves the model accuracy, and the model has a higher predictive ability.

**Table 5:** Comparison results with different mixture model for wind direction. A voM mixture model with nine-component distributions is the optimal model.

*k*	ln *L*	AIC	BIC	RMSE	*R*^2^
1	− 84,740.7450	169,485.4899	169,503.0093	0.0308	0.5798
2	− 84,706.8360	169,423.6719	169,467.4703	0.0304	0.5854
3	− 84,394.3660	168,804.7320	168,874.8095	0.0199	0.8522
4	− 84,387.5764	168,797.1529	168,893.5094	0.0169	0.8990
5	− 84,405.4189	168,838.8377	168,961.4734	0.0184	0.8774
6	− 84,410.4464	168,854.8928	169,003.8075	0.0161	0.9105
7	− 84,394.6663	168,829.3325	169,004.5263	0.0155	0.9164
8	− 84,471.9027	168,989.8054	169,191.2782	0.0227	0.7936
9	**− 84,377.1065**	**168,806.2129**	**169,033.9648**	**0.0134**	**0.9394**
10	− 84,374.6762	168,807.3524	169,061.3834	0.0134	0.9394

The results of the best model are in bold

**Figure 2:**
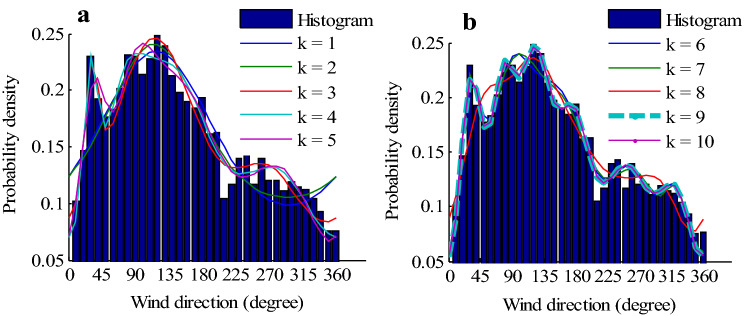
Histogram of wind directions for (**a**) *k* = 1, 2, …, 5 and (**b**) *k* = 6, 7, …, 10. The bin size of wind direction interval is 10°. A voM mixture model with nine-component distributions (**b** green bold dash line) fits the histogram of wind direction well in all candidate model with different components. Figure created using Matlab R2014a (https://www.mathworks.com).

The fit results of different models are given in Figs. 2(a) and 2(b), it also shows that nine-component mixture model fits the frequency histogram of wind direction well than other models.

In Table 4, from the estimated model parameters of nine-component mixture model of wind direction, we can see that the parameters of *μ*, which correspond to the nine main wind directions, are 0.0010, 0.5544, 1.3416, 1.5989, 2.1458, 2.2854, 3.0405, 4.3874 and 5.6471 rad (or 0.06°, 31.77°, 76.87°, 91.61°, 122.94°, 130.94°, 174.21°, 251.38° and 323.55°), respectively. As shown in Fig. 2(b), the prevailing wind directions covering from 0° to 180°.

**Figure 3:**
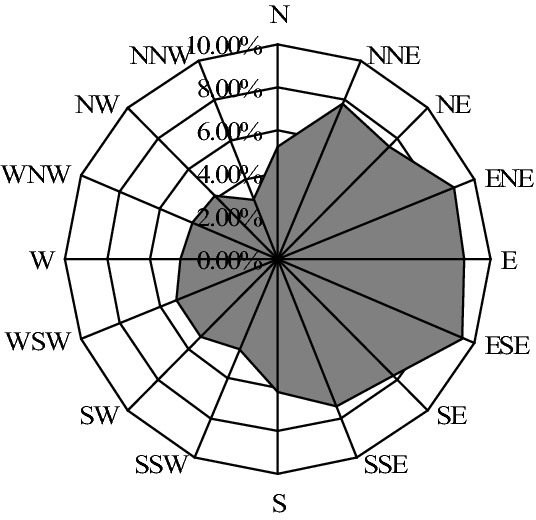
Wind rose diagram of wind direction. The wind direction is divided into 16 different sections. In the range from the direction N clockwise to S, three are seven main wind directions with 57.54% frequency totally. The other directions show an approximately uniform dispersion.

This result is also confirmed by the wind rose diagram shown in Fig. 3. According to the frequency of occurrence for wind direction, the nine main wind directions are classified into three kinds in descending order in this study: the first four, the middle three and the last two main wind directions. We can see that the first four prevailing wind directions are the ESE, ENE, E and NNE directions with 9.39%, 8.92%, 8.72% and 7.95% of frequency of occurrence, respectively. In Table 4, the four estimated parameters *μ* of nine-component mixture model are 2.1458, 1.3416, 1.5989 and 0.5544 rad, which correspond to the wind directions are 122.94°, 76.87°, 91.61° and 31.77°, respectively. This result is close to the result given by Fig. 2(b) with the ESE, ENE, E and NNE directions (112.5°, 67.5°, 90° and 22.5°). The middle three predominant directions are the SE, SSE and N directions (135°, 157.5° and 0°) with 7.74%, 7.42% and 7.40% of occurrence. This result also agrees well with the result given by the estimated parameters *μ* = 2.2854, 3.0405 and 0.0010 rad (130.94°, 174.21° and 0.06°) in nine-component mixture model of wind direction. The last two main wind directions are the WSW and NW directions (247.5° and 315°) with 5.11% and 4.20% frequency. Therefore, the yaw system of a wind turbine can be arranged to the ESE direction, since most of the wind blows from this direction, which will enable the wind turbine to be positioned in such a way as to maximize the captured energy.

Using Eqs. (17) and (18), the estimated mean and variance of wind direction can be given as follows: *b*_1_ = 2.8051 rad (160.72°) and *b*_2_ = 0.1052, respectively. The relative error of *b*_1_ is very small, it is only 2.99%.

The statistical association between the wind speed and wind direction is measured by the linear–circular correlation coefficient, *r*^2^, given by^33,37,40^

36 $$ r^{2} = \frac{{r_{vc}^{2} + r_{vs}^{2} - 2r_{vc} r_{vs} }}{{1 - r_{cs}^{2} }} $$

where *r*_*vc*_ = corr(*v*, cos*θ*), *r*_*vs*_ = corr(*v*, sin*θ*) and *r*_*cs*_ = corr(cos*θ*, sin*θ*). The correlation coefficient between wind speed and direction should satisfy the requirement of ׀*r*׀** < **1***/***3. In this study, the value of *r* is small and equals to 0.1101, therefore, it can be seen that there exists a weak correlation between wind speed and direction. The absolute value of correlation coefficient is within 1/3 satisfying the condition of use for voM wind direction model. At last, the parameter estimation results for the pdf *g*(*ξ*) of circular variable *ζ* between the wind speed and direction using a voM mixture distribution with the different components are given in Table 6.

**Table 6:** Parameter estimation results for circular variable between the wind speed and direction using different voM mixture distributions. There are 17 unknown parameters need to be estimated totally.

*k*	*p*	*α*	*μ*	*k*	*p*	*α*	*μ*	*k*	*p*	*α*	*μ*
1	1.0000	0.1283	1.7772	5	0.4786	0.6301	4.6067	7	0.0271	25.9206	5.9223
2	0.0641	4.8654	1.1485		0.0350	18.8312	1.1307		0.0235	17.4631	1.4179
	0.9359	0.0777	2.8944		0.0295	17.4734	5.8814		0.0461	16.0716	0.9755
3	0.2179	1.2932	2.0530		0.0380	19.9723	0.5709		0.0384	19.1911	0.4917
	0.0673	6.6117	0.9532		0.4189	0.9275	1.9459		0.5399	0.5717	4.6824
	0.7148	0.2678	4.8013	6	**0.0271**	**25.9203**	**5.9236**		0.2986	1.1985	2.1294
4	0.3865	0.8576	4.6962		**0.0228**	**17.4639**	**1.4361**		0.0264	1.2281	2.1992
	0.4695	0.8295	2.1480		**0.0476**	**16.0592**	**0.9895**				
	0.0324	16.4754	5.8558		**0.0395**	**19.1996**	**0.4968**				
	0.1116	4.5147	0.8659		**0.5507**	**0.5568**	**4.6757**				
					**0.3123**	**1.2454**	**2.1411**				

The results of the best model are in bold

Table 7 is a comparison results with different components for circular variable.

**Table 7:** Comparison results with different components for circular variable. A voM mixture model with six-component distributions is the optimal model for correlation circular variable between wind speed and direction.

*k*	ln *L*	AIC	BIC	RMSE	*R*^2^
1	− 86,400.2443	172,804.4887	172,822.0081	0.0280	0.9693
2	− 86,396.3473	172,802.6946	172,846.4930	0.0258	0.9739
3	− 86,419.6016	172,855.2032	172,925.2808	0.0248	0.9758
4	− 86,428.6593	172,879.3187	172,975.6753	0.0238	0.9777
5	− 86,433.5052	172,895.0104	173,017.6461	0.0236	0.9781
**6**	**− 86,446.5538**	**172,927.1075**	**173,076.0222**	**0.0233**	**0.9785**
7	− 86,445.9581	172,931.9162	173,107.1100	0.0233	0.9785

The results of the best model are in bold

From Table 7, it can be found that increasing the number of voM components from one to six in the mixture distributions will increase the value of the *R*^2^ coefficient. However, when the number of voM components increase to seven, the value of the *R*^2^ coefficient does not increase, it is the same as the six-component mixture model has. At the same time, a seven-component mixture model has the same value of RMSE as a six-component mixture model. It is also noticed that six-component mixture model has a lower value of AIC and BIC, so based on the comprehensive criteria of model selection, the most suitable model for the wind direction at this station is six-component voM mixture model.

**Figure 4:**
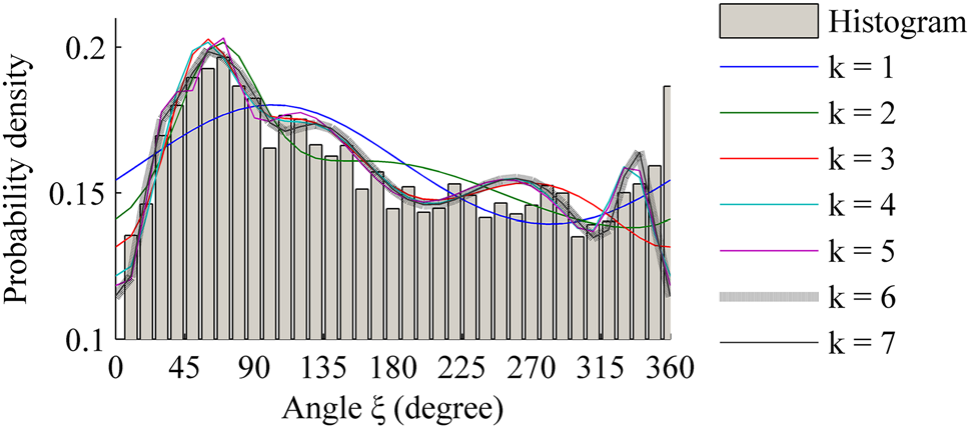
Histogram of circular variable *ξ*. A voM mixture model with six-component distributions fits the histogram of circular variable well in all candidate model with different components. Figure created using Matlab R2014a (https://www.mathworks.com).

When considering the correlation between wind speed and direction, the number of parameters of *μ*, which corresponds to the main wind directions, is decreased from 9 to 6. They are 0.4968, 0.9895, 1.4361, 2.1411, 4.6757 and 5.9236 rad (28.46°, 56.69°, 82.28°, 122.68°, 267.90° and 339.40°), respectively. Compared with the nine main wind directions (0.06°, 31.77°, 76.87°, 91.61°, 122.94°, 130.94°, 174.21°, 251.38° and 323.55°) without considering the correlation, we find an interesting phenomenon: the deleted three main wind directions (0.06°, 130.94° and 174.21°) are happened to be the middle three main wind directions analyzed in Table 4. A possible explanation is that the N and S directions, which correspond 0.06° and 174.21°, fall in the edge of the section of main wind directions ranging from the N direction clockwise to S direction, so the wind energy potential of these two directions is not significant than other parts of the section. On the other hand, 130.94° is approximately close to 122.94°, therefore, it is also deleted. At last, only six directions (31.77°, 76.87°, 91.61°, 122.94°, 251.38° and 323.55°) are left, and they are close to these six directions: 28.46°, 56.69°, 82.28°, 122.68°, 267.90° and 339.40°, as shown in Fig. 4. The maximum error does not exceed the value of one Sect. (22.5°).

**Figure 5:**
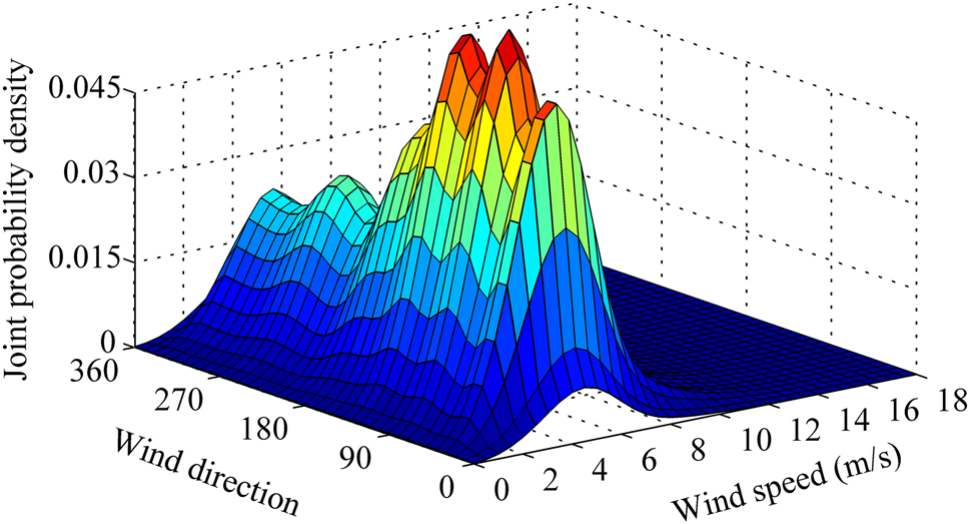
Joint probability density of wind speed and direction. Figure created using Matlab R2014a (https://www.mathworks.com).

**Figure 6:**
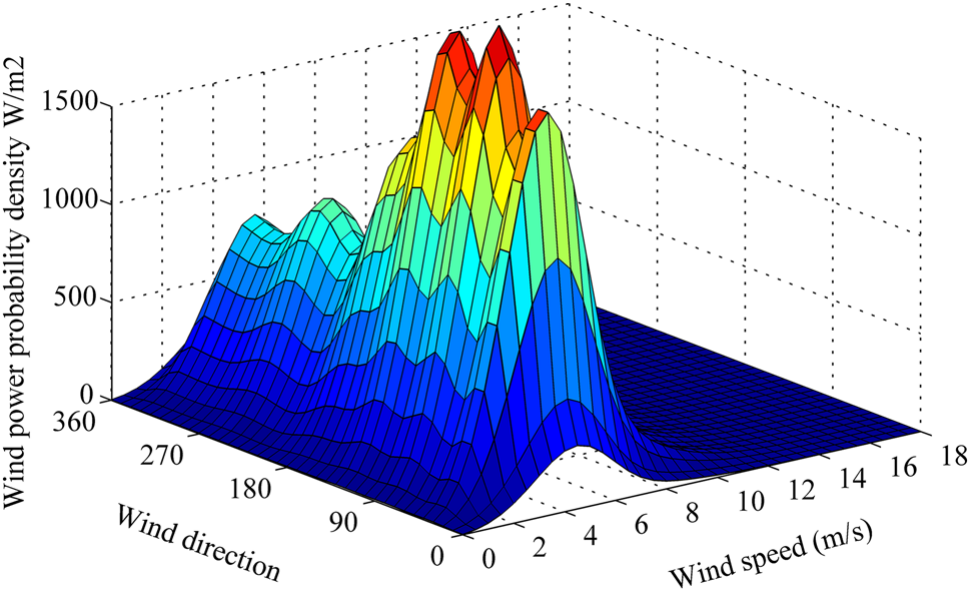
Wind power probability density. Figure created using Matlab R2014a (https://www.mathworks.com).

Therefore, based on Eqs. (23) and (33), the joint pdf of wind speed and direction and wind power probability density can be given in Figs. 5 and 6.

**Figure 7:**
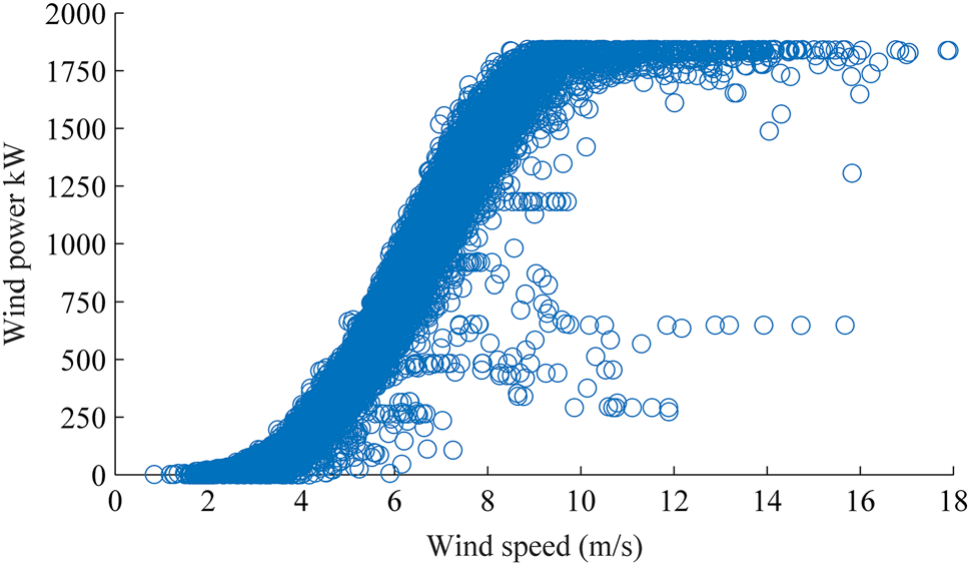
Scatter plot of wind power. There are a large number of outlier data points located far from the normal power bands. These abnormal and unreasonable data include the missing data by sensor fault, measurement error data and low temporal resolution data. Figure created using Matlab R2014a (https://www.mathworks.com).

The scatter plot of wind power output versus wind speed for 1.8 MW wind turbine is shown in Fig. 7, after pre-processing with a data-cleaning method, some abnormal and unreasonable data are discarded. The number and value of model parameters of power curve affect the fitting accuracy for wind power to speed data; more parameters can imply better performance, but can also mean time-consuming. Therefore, 4-parameter and 5-parameter logistic functions were compared to find the best performance^49^. In our study, we use a polynomial regression model to fit wind power curve because of its convenient calculating with Matlab and a high coefficient of determination 0.9932. Using a polynomial regression method to fit the data of wind power, a mathematical expression of wind power output with speed can be obtained.

The different fitting results are shown in Table 8, based on the correlation coefficient *R*^2^, we select the eight-degree polynomial as the best model for wind power output.

**Table 8:** The fitting results for wind power output with different degree *i*. The eight-degree polynomial with the highest correlation coefficient *R*^2^ = 0.9932 is selected as the best model for wind power output. For nine-degree polynomial, its correlation coefficient higher slightly than that of eight-degree polynomial, but the coefficient *a*_9_ is zero.

	*i* = 3	4	5	6	7	8	9	10
*a*_0_	190.9752	300.1130	50.3861	− 71.7432	− 29.7084	**8.2388**	0.9578	− 9.6752
*a*_1_	− 269.9311	− 382.1442	− 22.8256	233.5924	103.3182	**− 73.2569**	− 23.2012	83.4538
*a*_2_	83.2476	117.1197	− 36.9182	− 191.5619	− 85.8043	**100.0191**	34.3568	− 136.7091
*a*_3_	− 3.8966	− 7.6637	19.2781	58.9500	22.0650	**− 61.4883**	− 24.9013	91.1207
*a*_4_		0.1333	− 1.8654	− 6.6945	− 0.2396	**19.1791**	8.4253	− 33.6795
*a*_5_			0.0517	0.3245	− 0.2651	**− 2.7679**	− 0.9466	8.0854
*a*_6_				− 0.0057	0.0210	**0.2006**	0.0175	− 1.1788
*a*_7_					− 0.0005	**− 0.0072**	0.0036	0.1022
*a*_8_						**0.0001**	− 0.0002	− 0.0051
*a*_9_							0.0000	0.0001
*a*_10_								0.0000
*R*^2^	0.9851	0.9864	0.9915	0.9928	0.9930	0.9932	0.9933	0.9933

The results of the best model are in bold

**Figure 8:**
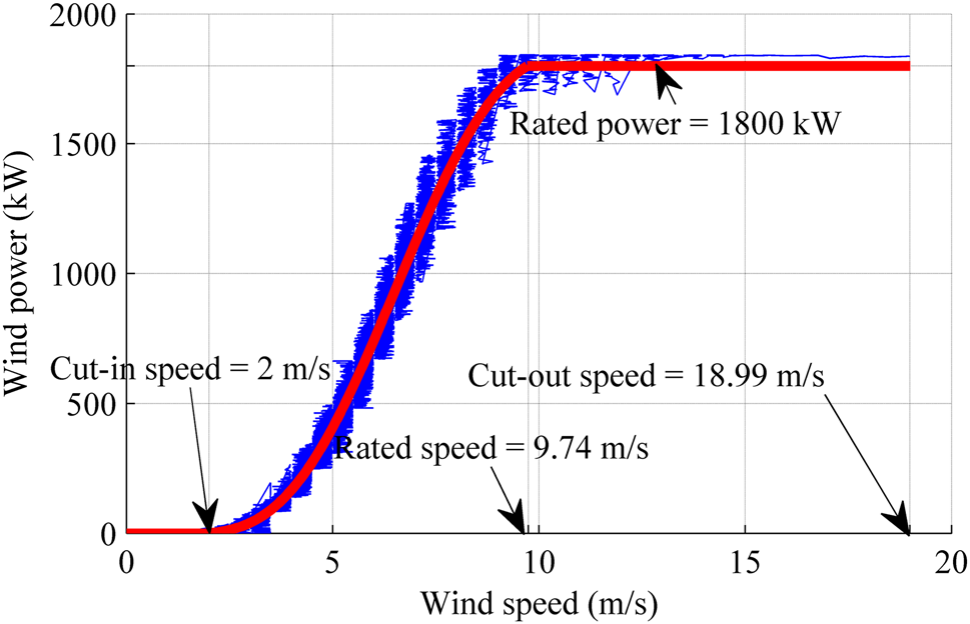
Wind power curve. The simplest data cleaning method is to delete the wind power data that lies outside the boundary. Blue zone line is wind power curve after pre-processing of raw wind power data, while red line is the fitting line for wind power data. Figure created using Matlab R2014a (https://www.mathworks.com).

The power curve of the studied wind turbine as shown in Fig. 8. The expression of wind power in W with wind speed in m/s is given as follows:

37 $$\begin{gathered} P\left( v \right) = 8238.7825 - 73256.9343v + 100019.0726v^{2} - 61488.3128v^{3} + 19179.0859v^{4} \hfill \\ \, \quad- 2767.8827v^{5} + 200.6144v^{6} - 7.1645v^{7} + 0.1006v^{8} {\text{ W}} \hfill \\ \end{gathered}$$

In this study, the cut-out wind speed (maximum allowable speed) and rated output power are known, they are 18.99 m/s and 1800 kW, respectively. The rated wind speed can be found by examining the power curve, i.e., the lowest wind speed, where the wind turbine first reaches its rated power, is the rated wind speed. Therefore, set Eq. (37) equals to 1.8 × 10^6^ and 0, we can get the rated output speed, *v*_*r*_ = 9.74 m/s, and the cut-in speed, *v*_in_ = 2 m/s, respectively, as shown in Fig. 8.

To estimate the wind power output of a wind turbine, it is necessary to know wind speed and the number of hours of the year, in which the wind blows at velocity *v*. The period *t* of one year, i.e., approximates 8760 h and the air density of studied area is 1.216 kg/m^3^ are used. Subsequently, the estimated values of wind power density at this wind farm were calculated using Eq. (33) is 93.27 W/m^2^. This value is close to the reference value of wind power density of 88.14 W/m^2^ which is obtained using Eq. (31). It indicates that the region under study stands in class 1^10^, which belongs to the low-wind speed wind power development area, and is generally not suitable for wide wind turbine establishment and wind farm investment. However, it would be possible to exploit the wind power applications for small scale wind turbines at this area. Based on Eq. (32), a comparison result without taking into account of wind direction is 125.78 W/m^2^, is also presented. It is clearly shown that the wind energy potential is overestimated without considering the effect of wind direction. Using Eq. (35), the annual energy output can also be obtained, it is 2.21 GWh.

## Conclusions

In this paper, the wind energy potential of the Maling Mountain in China is studied by using the measured wind data for a period of one year at a height of 30 m. Based on EM algorithm, an assessment method of wind energy potential using finite mixture statistical distribution model is proposed, the probability density function of wind power density and the annual energy output are given for use in wind energy analyses. The suitability of the model is judged using a comprehensive technique of model selection including AIC, BIC information criteria, coefficient of determination *R*^2^ and RMSE. Field monitoring wind data are used to verify the effectiveness and validity of the proposed method by comparing it with other estimation methods in accordance with the value of *R*^2^ and RMSE, the histogram plot and wind rose diagram. Wind direction is also important for a given wind farm when the orography and wake effects are going to be studied, so the orography and wake effects will be carried on in our future research, in addition we will also use Kato Jones distribution as candidate wind direction model and compare it with von Mises distribution. The further study to extend the modelling of direction-dependent power curve for wind turbine is also needed. The main conclusions are drawn from the study as follows:

1. The proposed method takes into account the effect of wind speed and direction simultaneously, the correlation existing between both variables, as well as the bimodal or multimodal distributions of them. The mixture distribution model provides a better fitting result for wind data than single distribution model, and it can therefore be used in the assessment of wind energy at a potential site and assessment result is more accuracy and close to the reality. On the other hand, three-parameter Weibull distribution considers the frequency of calm winds, it shows a good fit to wind speed data with a significant null wind speed or high percentage of low wind speed and is particularly suitable for a skewed data with a long tail in histogram plot.
2. The best mixture model with the lowest AIC and BIC values is selected as the optimal model from all candidate models with a finite component number, therefore it is not necessary to know the number of components in mixture model in advance. Increasing the number of components of the mixture distributions, the variations in value of *R*^2^ are not significant. On the contrary, it might yield complex. Therefore, combining with the comprehensive technique of model selection, an appropriate number of mixture distributions can be selected, which not only reduces the computational burden but also improves the model accuracy, and the model has a higher predictive ability.
3. Compared with the real wind power density of time series wind speed data, it also shown that when there exists a correlation between wind speed and its direction, the estimated results of wind energy potential is more close to the real situation when considering the influence of wind direction.

## Supplementary Information

Supplementary Table 1.

Supplementary Table 2.
