# Supplementary material for: Wind energy potential assessment based on wind speed, its direction and power data
Source: Sci Rep. 2021 Aug 19;11:16879. doi: 10.1038/s41598-021-96376-7 (PMC8377008; doi:10.1038/s41598-021-96376-7)
Supplement: Supplementary file 2 — Supplementary Table 2. [file 41598_2021_96376_MOESM2_ESM.doc]

| **No.** | **Speed Interval** | **Interval number** | **Frequency (%)** |
| --- | --- | --- | --- |
| 1 | 0-0.5 | 329 | 0.70 |
| 2 | 0.5-1 | 371 | 0.79 |
| 3 | 1-1.5 | 1133 | 2.41 |
| 4 | 1.5-2 | 2046 | 4.35 |
| 5 | 2-2.5 | 2647 | 5.62 |
| 6 | 2.5-3 | 3424 | 7.27 |
| 7 | 3-3.5 | 4508 | 9.57 |
| 8 | 3.5-4 | 6011 | 12.77 |
| 9 | 4-4.5 | 5849 | 12.42 |
| 10 | 4.5-5 | 5157 | 10.95 |
| 11 | 5-5.5 | 4747 | 10.08 |
| 12 | 5.5-6 | 3470 | 7.37 |
| 13 | 6-6.5 | 2400 | 5.10 |
| 14 | 6.5-7 | 1608 | 3.41 |
| 15 | 7-7.5 | 1069 | 2.27 |
| 16 | 7.5-8 | 745 | 1.58 |
| 17 | 8-8.5 | 478 | 1.02 |
| 18 | 8.5-9 | 328 | 0.70 |
| 19 | 9-9.5 | 219 | 0.47 |
| 20 | 9.5-10 | 133 | 0.28 |
| 21 | 10-10.5 | 75 | 0.16 |
| 22 | 10.5-11 | 62 | 0.13 |
| 23 | 11-11.5 | 64 | 0.14 |
| 24 | 11.5-12 | 52 | 0.11 |
| 25 | 12-12.5 | 40 | 0.08 |
| 26 | 12.5-13 | 36 | 0.08 |
| 27 | 13-13.5 | 23 | 0.05 |
| 28 | 13.5-14 | 31 | 0.07 |
| 29 | 14-14.5 | 9 | 0.02 |
| 30 | 14.5-15 | 6 | 0.01 |
| 31 | 15-15.5 | 5 | 0.01 |
| 32 | 15.5-16 | 2 | 0.00 |
| 33 | 16-16.5 | 1 | 0.00 |
| 34 | 16.5-17 | 1 | 0.00 |
| 35 | 17-17.5 | 1 | 0.00 |
| 36 | 17.5-18 | 2 | 0.00 |
| 37 | 18-18.5 | 1 | 0.00 |
| 38 | 18.5-19 | 1 | 0.00 |
|  |  | 47084 | 100.00 |

Table S2. Range and frequency of wind speed. The speed interval is selected as 0.5 m/s which gives a minimum error for modelling wind speed data. The frequency of 0-2 m/s wind speed range is 8.25% indicates that low wind speed is significant.
