# Supplementary material for: Wind energy potential assessment based on wind speed, its direction and power data
Source: Sci Rep. 2021 Aug 19;11:16879. doi: 10.1038/s41598-021-96376-7 (PMC8377008; doi:10.1038/s41598-021-96376-7)
Supplement: Supplementary file 1 — Supplementary Table 1. [file 41598_2021_96376_MOESM1_ESM.pdf]

Wind energy potential assessment based on wind speed, its direction and power data

Zhiming Wang1,* and Weimin Liu1,2
1School of Mechanical and Electronic Engineering, Lanzhou University of Technology, Lanzhou 730050, China.
2Gansu Province Special Equipment Inspection and Testing Institute, Lanzhou 730050, China
*corresponding. wangzhiming301@sohu.com

No.	Wind Speed	Wind Power	Wind Direction
1	0	-2.8975	147.508
2	0	-2.9206	54.4047
3	0	-2.9387	38.1313
4	0	-4.2134	32.4478
5	0	-3.2457	45.3879
6	0	-2.9312	46.094
7	0	-2.9432	65.3568
8	0	-3.4702	132.164
9	0	-2.9508	106.654
10	0	-2.9453	42.5074
11	0	-3.0074	104.962
12	0	-2.9631	166.616
13	0	-3.9559	196.947
14	0	-3.1035	8.264
15	0	-3.0954	289.576
16	0	-3.8363	282.246
17	0	-2.9801	262.266
18	0	-2.9876	28.5737
19	0	-4.0029	59.2997
20	0	-2.9757	138.09
21	0	-2.9521	137.904
22	0	-3.0723	146.542
23	0	-2.8863	50.3785
24	0	-4.2166	130.918
25	0	-2.9763	109.118
26	0	-2.9281	116.089
27	0	-3.0026	117.744
28	0	-4.2295	117.757
29	0	-2.9193	117.747
30	0	-2.9458	117.755
31	0	-4.2506	101.865
32	0	-2.9322	85.7062
33	0	-2.9282	65.1407
34	0	-2.874	189.99
35	0	-2.7763	316.536
36	0	-2.7825	60.3881
37	0	-2.903	359.558

38	0	-3.097	211.502
39	0	-3.1992	10.3135
40	0	-3.5791	8.4178
41	0	-3.5661	112.695
42	0	-2.8022	148.909
43	0	-2.8125	146.935
44	0	-3.143	156.229
45	0	-3.8648	10.6265
46	0	-2.8045	282.083
47	0	-2.8171	255.588
48	0	-2.8318	247.242
49	0	-2.8076	20.8241
50	0	-3.2744	30.9536
51	0	-2.8248	76.6847
52	0	-2.8267	75.4196
53	0	-4.0917	301.906
54	0	-2.8259	324.504
55	0	-2.8047	355.767
56	0	-3.5848	14.1908
57	0	-3.7691	172.823
58	0	-3.8715	164.53
59	0	-2.9921	79.7527
60	0	-4.1196	79.7271
61	0	-2.8358	79.7395
62	0	-3.0056	127.73
63	0	-4.2038	183.273
64	0	-2.8427	296.536
65	0	-2.8469	296.464
66	0	-4.3764	291.678
67	0	-2.7469	68.5225
68	0	-2.8087	148.326
69	0	-2.8213	155.875
70	0	-2.8351	234.17
71	0	-3.1508	326.478
72	0	-2.7717	228.198
73	0	-2.8069	226.019
74	0	-3.9169	233.836
75	0	-2.8074	208.131
76	0	-2.8079	209.669
77	0	-4.0216	163.762
78	0	-4.0168	205.386
79	0	-2.8219	203.405
80	0	-2.8195	346.627
81	0	-4.1311	346.59
82	0	-2.833	346.598
83	0	-2.8293	346.607
84	0	-3.4369	346.586
85	0	-3.5309	346.574

86	0	-2.8717	1.6682
87	0	-3.2447	1.6347
88	0	-3.9153	1.614
89	0	-2.8914	320.183
90	0	-2.892	223.385
91	0	-4.1337	101.064
92	0	-2.9687	96.809
93	0	-2.9516	96.7925
94	0	-2.8823	295.317
95	0	-2.902	225.203
96	0	-2.8665	275.694
97	0	-2.886	275.731
98	0	-4.2585	275.731
99	0	-2.8926	275.724
100	0	-2.89	275.672
101	0	-3.0473	236.77
102	0	-4.2077	246.182
103	0.0001	-2.9946	276.496
104	0.0001	-2.8928	89.1467
105	0.0001	-2.8425	204.382
106	0.0001	-3.8073	113.099
107	0.0001	-2.7099	104.03
108	0.0001	-3.8944	118.934
109	0.0001	-3.1969	116.279
110	0.0001	-3.5979	157.709
111	0.0001	-2.7941	326.466
112	0.0001	-2.7958	121.776
113	0.0001	-3.0878	256.844
114	0.0001	-3.0502	131.137
115	0.0001	-3.9441	192.234
116	0.0001	-2.8308	188.49
117	0.0001	-2.8125	216.823
118	0.0002	-2.9056	295.075
119	0.0002	-2.7868	120.923
120	0.0002	-2.8006	358.406
121	0.0002	-2.7945	117.683
122	0.0002	-2.801	307.177
123	0.0002	-2.8353	158.461
124	0.0002	-2.89	161.19
125	0.0003	-3.5164	188.943
126	0.0003	-2.7929	16.7572
127	0.0003	-2.7931	29.1397
128	0.0003	-2.8105	333.127
129	0.0003	-3.4552	0.7882
130	0.0003	-3.0905	340.902
131	0.0003	-2.8039	118.935
132	0.0003	-3.9269	143.703
133	0.0003	-2.8197	3.0402

134	0.0003	-4.0022	213.594
135	0.0003	-2.8077	307.115
136	0.0003	-2.8505	161.107
137	0.0003	-2.7417	50.6328
138	0.0003	-3.3116	277.491
139	0.0004	-2.7036	21.1196
140	0.0004	-2.7949	39.5129
141	0.0004	-3.2434	55.8314
142	0.0004	-3.574	45.1649
143	0.0004	-2.8048	5.5668
144	0.0004	-4.0116	4.1894
145	0.0004	-3.6341	306.92
146	0.0004	-2.6956	357.324
147	0.0004	-3.1798	331.45
148	0.0004	-2.8105	125.522
149	0.0005	-3.1014	78.021
150	0.0005	-2.923	13.6581
151	0.0005	-2.7008	155.007
152	0.0005	-2.7515	248.319
153	0.0005	-3.5949	193.573
154	0.0005	-2.7981	7.6673
155	0.0005	-3.2381	325.939
156	0.0005	-2.8095	311.888
157	0.0006	-2.7155	10.8686
158	0.0006	-3.9305	344.086
159	0.0007	-2.7043	56.6443
160	0.001	-2.925	125.531
161	0.001	-3.9689	15.2158
162	0.0012	-4.0055	137.554
163	0.0012	-2.6847	317.625
164	0.0016	-3.8687	275.473
165	0.0016	-3.8944	13.5618
166	0.0017	-2.935	23.1999
167	0.0018	-3.4213	156.53
168	0.0018	-3.1459	313.119
169	0.0018	-2.8052	140.715
170	0.002	-2.7004	312.629
171	0.0023	-2.7808	238.607
172	0.0024	-2.6949	4.1919
173	0.0026	-3.0375	200.617
174	0.0027	-2.8207	221.372
175	0.0029	-2.8115	39.4538
176	0.003	-3.376	353.591
177	0.0031	-2.6875	75.6682
178	0.004	-2.7072	283.593
179	0.0043	-3.771	341.372
180	0.0049	-2.929	123.191
181	0.0052	-3.6192	142.154

182	0.0052	-3.4716	204.96
183	0.0053	-2.7464	194.464
184	0.0055	-3.2786	306.433
185	0.0055	-3.1192	314.254
186	0.0066	-2.7473	209.386
187	0.0095	-2.7189	267.573
188	0.0097	-2.7353	131.732
189	0.0098	-2.7361	9.0914
190	0.0106	-2.9674	132.779
191	0.0107	-2.7581	78.033
192	0.0114	-2.9211	283.933
193	0.0114	-2.7661	343.858
194	0.0125	-2.7782	176.687
195	0.0147	-3.2255	15.9212
196	0.0151	-2.774	296.851
197	0.0173	-2.8374	124.05
198	0.018	-2.7335	305.6
199	0.0182	-2.7236	331.802
200	0.019	-3.4055	319.844
201	0.0193	-3.1653	272.076
202	0.0201	-2.7725	337.745
203	0.0208	-3.9061	220.035
204	0.0213	-3.0731	209.03
205	0.0223	-2.8676	210.419
206	0.0238	-3.8144	217.951
207	0.024	-2.9697	326.677
208	0.0257	-3.7542	30.3656
209	0.0265	-2.6847	103.93
210	0.0322	-2.89	91.7789
211	0.0404	-2.8069	204.769
212	0.0453	-2.7653	39.9402
213	0.056	-2.866	104.896
214	0.0598	-4.0292	234.169
215	0.0642	-3.2629	1.6667
216	0.0703	-3.8652	185.18
217	0.0704	-3.317	321.281
218	0.0705	-4.0179	294.255
219	0.0711	-2.9308	47.4015
220	0.0714	-3.1206	330.641
221	0.0732	-2.8438	89.9363
222	0.08	-2.804	46.8776
223	0.0809	-4.3392	338.516
224	0.0821	-2.8639	23.2824
225	0.0824	-2.9838	65.6886
226	0.0848	-3.4901	343.838
227	0.0894	-3.1431	47.3623
228	0.091	-2.9738	82.2835
229	0.0925	-2.783	124.043

230	0.0934	-3.2854	358.322
231	0.1003	-2.9335	309.838
232	0.1018	-4.2274	305.253
233	0.1082	-3.1985	342.406
234	0.1084	-2.9454	137.861
235	0.1092	-3.791	227.914
236	0.1096	-4.3083	247.781
237	0.1189	-2.8961	320.595
238	0.1227	-2.949	60.0902
239	0.1243	-3.3424	76.7756
240	0.1264	-2.7929	13.5178
241	0.1282	-2.9481	349.306
242	0.1363	-2.8376	79.7806
243	0.1375	-3.497	29.0005
244	0.1382	-3.2546	322.09
245	0.1395	-4.081	180.341
246	0.1426	-2.8489	128.59
247	0.1461	-2.7759	9.2528
248	0.1473	-3.2221	75.452
249	0.1484	-3.8094	340.64
250	0.1499	-2.7764	148.528
251	0.158	-2.8009	102.624
252	0.1595	-3.0823	343.89
253	0.1636	-2.9699	39.9365
254	0.1642	-2.9276	47.9247
255	0.1657	-2.9461	195.836
256	0.1703	-2.8243	355.618
257	0.1738	-2.8582	336.105
258	0.1753	-2.8897	1.6713
259	0.1778	-2.8065	118.967
260	0.1778	-3.9578	172.627
261	0.1784	-2.8706	184.022
262	0.1817	-2.7836	255.817
263	0.1829	-3.5112	338.219
264	0.186	-2.6971	193.951
265	0.1862	-2.9059	275.788
266	0.1873	-3.2584	319.253
267	0.1882	-2.9537	246.202
268	0.1892	-2.8149	172.416
269	0.1938	-4.2949	92.4041
270	0.2003	-4.209	13.2167
271	0.212	-4.2764	100.496
272	0.2166	-3.9727	268.798
273	0.2192	-2.8822	27.8253
274	0.2232	-3.6112	218.068
275	0.2263	-4.2258	72.737
276	0.2291	-3.4146	212.879
277	0.2294	-4.186	333.001

278	0.2302	-2.941	334.943
279	0.2355	-2.9979	204.24
280	0.2373	-2.9293	147.683
281	0.2443	-3.3289	16.1526
282	0.2452	-3.2684	99.7484
283	0.2459	-2.7787	227.979
284	0.2462	-2.822	111.657
285	0.2474	-4.0601	133.838
286	0.2515	-2.9503	14.594
287	0.2522	-2.7907	170.071
288	0.2548	-2.9767	25.921
289	0.2554	-2.7743	302.608
290	0.2557	-2.978	53.6821
291	0.2586	-2.9787	351.808
292	0.2597	-2.8224	352.036
293	0.2695	-2.9218	320.398
294	0.2744	-4.0615	119.575
295	0.2756	-4.4812	20.8537
296	0.2855	-2.6853	125.598
297	0.2908	-3.9096	306.3
298	0.2915	-2.7944	349.586
299	0.2951	-2.8603	337.409
300	0.2992	-2.7718	27.1216
301	0.3007	-2.9582	105.534
302	0.3026	-3.7852	321.758
303	0.3027	-2.809	46.6195
304	0.3027	-2.8616	147.447
305	0.3076	-2.8212	185.105
306	0.3087	-2.6916	134.514
307	0.3154	-3.9866	271.097
308	0.3182	-2.7555	14.6635
309	0.3196	-2.7724	329.998
310	0.3202	-3.7323	277.91
311	0.3229	-2.7419	133.143
312	0.3237	-3.913	55.8716
313	0.3297	-3.455	6.8168
314	0.3309	-2.8218	9.1612
315	0.3312	-2.9394	236.027
316	0.3394	-2.9524	60.0315
317	0.341	-4.1325	241.488
318	0.343	-3.9572	299.205
319	0.3456	-2.9824	240.393
320	0.3464	-2.9661	103.596
321	0.3496	-2.8951	124.138
322	0.3591	-2.8667	289.127
323	0.3594	-2.8781	1.2011
324	0.3615	-3.3645	354.181
325	0.3618	-3.2152	250.144

326	0.3618	-2.7286	5.4731
327	0.3636	-2.8338	346.643
328	0.3647	-2.9712	300.583
329	0.3677	-2.7959	289.319
330	0.3697	-3.5038	35.762
331	0.3719	-3.8319	99.676
332	0.3737	-3.8432	183.371
333	0.3761	-2.7877	275.898
334	0.3772	-2.7749	353.531
335	0.3783	-2.8536	328.206
336	0.3813	-2.9306	183.012
337	0.3834	-2.7582	22.2892
338	0.3876	-2.9464	2.9259
339	0.3882	-2.9921	33.2574
340	0.3931	-2.7659	343.495
341	0.397	-3.6119	80.6948
342	0.3988	-3.201	339.252
343	0.3995	-4.4226	271.827
344	0.4004	-2.9802	124.174
345	0.4021	-3.7254	80.3613
346	0.4027	-3.2289	172.326
347	0.4031	-4.1279	330.128
348	0.4038	-2.7662	271.757
349	0.4046	-2.9102	141.412
350	0.4084	-2.8504	10.4124
351	0.4119	-2.8767	316.778
352	0.4125	-2.8369	2.2301
353	0.4165	-2.7413	1.3966
354	0.4174	-2.7618	234.896
355	0.4239	-2.8812	294.979
356	0.4245	-3.3854	322.637
357	0.4297	-2.757	326.803
358	0.43	-2.9669	96.7694
359	0.4328	-3.8993	316.281
360	0.4334	-5.1929	204.348
361	0.4363	-2.9355	129.707
362	0.4411	-3.9788	339.467
363	0.4422	-2.7565	217.763
364	0.4435	-2.7708	52.7518
365	0.4543	-3.7827	325.862
366	0.4606	-3.8969	127.301
367	0.4608	-2.8012	277.343
368	0.461	-3.4168	187.339
369	0.4614	-2.8196	87.2733
370	0.463	-3.0615	302.156
371	0.4644	-2.8379	334.323
372	0.4646	-2.8928	106.21
373	0.4667	-2.8201	305.666

374	0.4726	-2.7506	342.479
375	0.4754	-3.3475	93.1232
376	0.4776	-2.7631	301.728
377	0.4796	-2.9196	295.346
378	0.4833	-2.8762	14.3142
379	0.4846	-2.7888	344.493
380	0.4924	-2.9549	38.265
381	0.493	-2.7432	182.36
382	0.4959	-3.7882	146.426
383	0.4978	-4.0767	296.347
384	0.4989	-2.9945	31.2681
385	0.5031	-2.9624	349.734
386	0.5083	-3.3649	60.1143
387	0.5085	-3.3612	355.633
388	0.5086	-2.9304	61.7446
389	0.5101	-3.2214	54.4144
390	0.5126	-2.8118	285.647
391	0.5128	-2.8753	344.846
392	0.5132	-2.9541	304.723
393	0.5166	-2.6883	157.463
394	0.5215	-2.9676	275.055
395	0.5232	-2.9358	93.6543
396	0.5238	-2.878	332.151
397	0.5258	-3.0068	78.9751
398	0.5259	-2.7716	64.1253
399	0.5291	-2.9708	3.7524
400	0.5345	-2.6567	185.038
401	0.5362	-4.0064	296.718
402	0.5398	-2.7606	251.855
403	0.5423	-2.9589	310.939
404	0.5434	-2.7825	337.144
405	0.5457	-3.6177	253.611
406	0.5485	-2.8747	2.2598
407	0.5494	-3.9472	152.558
408	0.5514	-4.402	305.22
409	0.5554	-2.7135	161.976
410	0.5564	-3.8164	350.823
411	0.5567	-2.7674	282.764
412	0.5573	-2.9122	97.7238
413	0.5635	-3.0964	137.85
414	0.5635	-2.9318	8.3309
415	0.5696	-2.8402	100.271
416	0.571	-3.1866	347.897
417	0.5711	-2.7868	336.252
418	0.5719	-2.756	5.2115
419	0.5741	-2.8581	234.008
420	0.5755	-2.8512	220.608
421	0.5763	-2.7368	357.528

422	0.5773	-3.5555	359.911
423	0.5784	-2.8098	32.2232
424	0.5792	-3.101	340.944
425	0.581	-2.7551	48.9264
426	0.583	-2.8293	308.296
427	0.5867	-2.8433	305.03
428	0.5878	-2.87	298.552
429	0.5895	-2.6751	354.075
430	0.5912	-3.417	355.228
431	0.5924	-2.7503	275.119
432	0.5934	-3.1369	303.192
433	0.5947	-4.2246	1.6823
434	0.5949	-3.2291	100.475
435	0.5986	-4.0761	35.6426
436	0.5998	-4.3663	314.655
437	0.6015	-2.9051	273.071
438	0.6017	-2.7937	95.525
439	0.6019	-2.6662	321.517
440	0.6037	-2.907	352.998
441	0.6059	-2.8567	341.906
442	0.6092	-3.7161	323.31
443	0.6093	-2.9731	238.143
444	0.6103	-2.7977	242.022
445	0.6192	-4.1161	247.021
446	0.6221	-2.8	53.9714
447	0.6224	-3.4951	79.6062
448	0.6256	-3.2575	273.564
449	0.626	-2.745	21.58
450	0.627	-2.7387	226.129
451	0.6278	-2.8221	18.3234
452	0.6279	-2.9245	191.346
453	0.6287	-3.1397	13.0322
454	0.6297	-2.8952	248.943
455	0.6317	-2.6686	2.9213
456	0.6333	-2.9481	72.9552
457	0.635	-3.7252	22.6719
458	0.6353	-2.8356	75.0658
459	0.6356	-2.9381	96.844
460	0.6378	-2.8712	327.362
461	0.6384	-2.8505	306.668
462	0.6398	-3.936	39.4564
463	0.6401	-2.9267	333.924
464	0.6404	-5.3796	266.23
465	0.643	-2.6686	215.781
466	0.6432	-2.976	248.729
467	0.6432	-2.8323	126.931
468	0.6476	-2.8045	351.735
469	0.6482	-2.9883	109.477

470	0.6534	-3.4857	90.304
471	0.6546	-2.9522	322.816
472	0.6551	-3.7764	275.065
473	0.6562	-2.7316	14.6464
474	0.6619	-2.6917	288.063
475	0.663	-3.9274	245.769
476	0.6634	-2.9355	253.379
477	0.6643	-3.8595	185.76
478	0.6673	-2.9114	253.861
479	0.6677	-3.8719	284.352
480	0.6678	-3.374	28.5504
481	0.6681	-3.8816	162.915
482	0.6694	-2.7768	232.698
483	0.6699	-3.1367	153.827
484	0.6721	-4.3038	103.834
485	0.6722	-4.14	147.377
486	0.6727	-2.9304	54.9923
487	0.6778	-3.121	44.7185
488	0.6783	-2.9399	344.873
489	0.6789	-3.4385	11.8987
490	0.68	-4.0193	248.568
491	0.6815	-2.8765	1.6799
492	0.6821	-3.605	205.515
493	0.6828	-4.0454	330.699
494	0.6862	-3.3578	236.639
495	0.6887	-3.8071	156.873
496	0.6896	-2.859	285.86
497	0.6908	-2.7866	107.221
498	0.691	-3.2687	311.131
499	0.6919	-2.9348	230.044
500	0.692	-2.7703	356.65
501	0.6944	-4.2908	314.564
502	0.6976	-2.7822	105.297
503	0.7011	-3.7452	64.0067
504	0.7015	-2.7177	352.786
505	0.7033	-3.5064	319.727
506	0.7033	-3.0604	162.853
507	0.7051	-2.974	269.35
508	0.7083	-2.8299	338.737
509	0.7114	-2.9294	127.087
510	0.7135	-2.9728	36.5014
511	0.7137	-3.4455	225.936
512	0.7181	-2.7837	331.708
513	0.7184	-3.7041	229.753
514	0.7189	-3.8458	316.98
515	0.719	-2.9425	96.6851
516	0.7192	-3.1018	144.912
517	0.7209	-2.9569	131.113

518	0.7247	-2.8113	102.528
519	0.725	-2.7824	258.811
520	0.731	-3.7608	97.8739
521	0.7323	-2.9846	39.565
522	0.733	-3.0854	343.559
523	0.7343	-3.6612	302.625
524	0.7367	-2.7345	60.6897
525	0.7371	-3.2779	237.56
526	0.7377	-2.8854	185.154
527	0.7415	-3.9325	280.273
528	0.7434	-3.2136	267.154
529	0.7457	-2.7841	70.7505
530	0.7521	-3.7404	58.4497
531	0.7525	-3.7273	262.116
532	0.753	-3.7317	341.735
533	0.7566	-2.7994	299.719
534	0.76	-2.8736	313.239
535	0.7629	-2.9441	220.685
536	0.7643	-3.0379	323.719
537	0.7646	-2.9962	312.971
538	0.7657	-2.8887	41.9467
539	0.7675	-3.6173	255.959
540	0.7681	-2.8542	311.195
541	0.7683	-2.7282	354.227
542	0.7687	-3.482	311.405
543	0.7715	-3.6712	302.919
544	0.7723	-2.7655	349.107
545	0.7724	-4.2812	307.105
546	0.7731	-2.8147	91.9047
547	0.7737	-2.9638	137.53
548	0.7738	-4.285	345.018
549	0.7761	-3.2166	149.418
550	0.7764	-3.2514	294.475
551	0.7804	-2.8832	312.453
552	0.7819	-2.787	4.0566
553	0.7822	-2.7698	74.5665
554	0.7824	-2.982	141.297
555	0.7832	-2.8521	20.5276
556	0.7844	-3.2166	131.035
557	0.7854	-2.9422	255.882
558	0.7857	-4.2246	196.461
559	0.7861	-2.9377	107.183
560	0.7878	-3.1756	201.861
561	0.7887	-3.6366	21.0071
562	0.7888	-2.9115	280.246
563	0.7902	-3.7548	144.366
564	0.7904	-2.8213	234.215
565	0.7939	-3.2822	6.7075

566	0.7978	-4.258	9.747
567	0.7984	-3.4621	354.137
568	0.7986	-2.7681	22.7078
569	0.8	-3.3667	212.309
570	0.8006	-2.6834	280.994
571	0.801	-2.7397	32.3899
572	0.8016	-2.8954	13.4181
573	0.8042	-2.8886	1.7019
574	0.8055	-2.778	63.3906
575	0.8065	-2.7719	222.768
576	0.8083	-2.8578	21.1892
577	0.8111	-3.2031	202.979
578	0.8127	-3.7626	353.348
579	0.8134	-4.1282	326.28
580	0.8134	-2.8406	242.474
581	0.8142	-3.9212	347.499
582	0.8154	-2.7632	357.073
583	0.8157	-3.9697	316.643
584	0.8174	-2.7206	9.0017
585	0.8176	-2.9634	330.483
586	0.8181	-2.9699	69.0551
587	0.8199	-2.8202	5.7625
588	0.8203	-3.941	86.5526
589	0.8209	-2.835	31.7234
590	0.8217	-2.8075	89.9405
591	0.8218	-3.0505	324.227
592	0.822	-3.0075	240.997
593	0.8227	-2.8499	352.679
594	0.824	-2.9063	201.239
595	0.8246	-2.9627	292.39
596	0.8247	-2.8267	279.584
597	0.8255	-3.6046	113.853
598	0.8261	-3.5535	40.1344
599	0.8285	-3.4199	161.942
600	0.8289	-2.8946	295.456
601	0.8296	-3.2139	161.756
602	0.8301	-3.0457	194.442
603	0.8304	-3.8264	341.905
604	0.8305	-2.7333	358.344
605	0.8317	-2.8965	160.317
606	0.8338	-3.8664	10.0807
607	0.8351	-3.0341	14.3351
608	0.8358	-3.2678	44.781
609	0.836	-2.9738	300.352
610	0.8361	-3.0594	25.8856
611	0.8379	-3.9467	120.341
612	0.8381	1.0589	29.5208
613	0.8412	-2.9175	304.807

614	0.8419	-2.7854	158.866
615	0.842	-2.6697	334.733
616	0.8424	-2.7559	8.6275
617	0.8431	-4.2669	295.372
618	0.8443	-2.8134	343.579
619	0.8445	-3.9027	91.7321
620	0.845	-2.8268	22.0728
621	0.845	-2.938	348.126
622	0.8464	-4.0807	338.091
623	0.8464	-2.8329	139.488
624	0.8472	-3.6557	259.632
625	0.8474	-4.1998	270.764
626	0.8477	-3.936	345.401
627	0.8511	-2.9296	51.9915
628	0.8535	-2.9011	341.994
629	0.8541	-2.8737	157.959
630	0.8559	-3.2359	288.798
631	0.856	-2.9099	58.1652
632	0.8565	-2.7575	222.802
633	0.8568	-5.2009	202.365
634	0.8574	-2.9826	328.465
635	0.8578	-3.6062	233.849
636	0.858	-3.279	90.7785
637	0.8586	-3.4015	119.073
638	0.8587	-2.7821	196.834
639	0.8594	-2.7497	92.6053
640	0.8601	-2.9163	40.3926
641	0.8607	-3.8573	286.031
642	0.8626	-3.1974	336.499
643	0.8638	-4.2665	1.658
644	0.8642	-2.9298	328.942
645	0.8668	-3.8849	300.722
646	0.8676	-2.9592	339.088
647	0.8689	-2.7949	63.9436
648	0.8692	-2.883	1.713
649	0.8696	-2.9639	10.2615
650	0.8698	-3.7652	320.065
651	0.8732	-4.0766	82.7517
652	0.8783	-2.7855	171.942
653	0.8788	-2.9289	246.37
654	0.879	-2.8028	185.416
655	0.8798	-4.1173	132.85
656	0.8806	-2.9515	325.515
657	0.8825	-2.945	353.832
658	0.883	-2.9467	314.553
659	0.8835	-2.8818	0.1966
660	0.8859	-2.9096	314.317
661	0.8859	-3.4099	0.0442

662	0.8868	-3.1678	97.2998
663	0.8874	-2.935	140.466
664	0.8888	-3.2015	306.42
665	0.8898	-3.8625	271.309
666	0.8901	-3.9849	280.167
667	0.8909	-3.913	70.9958
668	0.8909	-2.9378	31.3915
669	0.8921	-4.1788	78.899
670	0.8933	-3.9179	1.674
671	0.8943	-2.7834	350.478
672	0.896	-2.9507	103.799
673	0.8981	-2.883	319.328
674	0.8984	-3.7983	86.9851
675	0.9028	-2.7641	356.912
676	0.9036	-2.762	33.3069
677	0.9046	-2.8571	303.241
678	0.906	-3.5027	268.383
679	0.9068	-2.8389	326.531
680	0.9084	-3.1519	285.343
681	0.9085	-2.9144	140.843
682	0.9085	-3.0593	29.5666
683	0.9104	-2.9127	265.908
684	0.911	-4.0033	356.199
685	0.9111	-2.6709	353.608
686	0.9113	-4.0227	86.1741
687	0.9115	-2.7501	346.32
688	0.9123	-3.2853	3.0966
689	0.9124	-2.9264	44.5269
690	0.9125	-2.969	357.64
691	0.914	-3.9994	177.436
692	0.9143	-2.8659	313.637
693	0.9162	-2.7465	137.804
694	0.9164	-2.8422	156.326
695	0.9174	-2.8518	290.078
696	0.9175	-2.943	129.7
697	0.9176	-2.982	323.955
698	0.9218	-3.9198	236.213
699	0.9222	-3.0898	5.5322
700	0.9225	-3.5798	313.27
701	0.924	-2.7227	101.724
702	0.9252	-4.0726	106.332
703	0.9266	-2.9131	232.907
704	0.9278	-3.0165	317.484
705	0.9284	-3.4246	128.096
706	0.929	-3.2998	177.645
707	0.93	-2.9452	344.827
708	0.9309	-2.9466	84.8237
709	0.9311	-2.8381	240.241

710	0.9327	-2.774	263.464
711	0.9329	-3.903	171.761
712	0.9341	-2.8807	181.97
713	0.9343	-3.0271	103.274
714	0.9351	-2.8754	118.327
715	0.9388	-3.6272	356.443
716	0.9389	-2.9515	268.19
717	0.9394	-2.8748	1.7174
718	0.9401	-2.716	88.1694
719	0.9403	-2.9462	45.1035
720	0.9408	-4.2846	136.475
721	0.9416	-2.8765	283.478
722	0.942	-3.1449	91.2006
723	0.9435	-2.9172	43.7983
724	0.9445	-2.7855	119.594
725	0.9455	-2.8922	346.64
726	0.9471	-2.831	230.969
727	0.9472	-2.768	357.729
728	0.9475	-3.0455	257.447
729	0.9477	-3.6831	355.389
730	0.948	-2.7623	171.433
731	0.9522	-3.2935	81.8348
732	0.9524	-2.8128	130.32
733	0.953	-2.9065	321.632
734	0.954	-2.9511	31.4001
735	0.955	-3.1272	36.0442
736	0.956	-3.9121	292.837
737	0.956	-2.7979	351.462
738	0.9561	-2.9428	249.265
739	0.9564	-4.0861	16.1057
740	0.9574	-3.4583	104.048
741	0.9601	-2.9477	251.64
742	0.9621	-2.8702	333.203
743	0.9623	-3.4982	18.8474
744	0.9623	-3.4692	150.017
745	0.9625	-2.7361	344.568
746	0.9633	-2.9384	92.2263
747	0.9649	-2.9693	100.196
748	0.965	-2.9751	62.368
749	0.9656	-2.6905	179.906
750	0.9661	-3.0928	275.071
751	0.9669	-3.1929	79.9165
752	0.9685	-2.9604	331.881
753	0.9703	-2.8451	73.0235
754	0.9708	-3.9976	350.908
755	0.9719	-2.812	223.636
756	0.9722	-2.784	106.521
757	0.9722	-3.5016	102.673

758	0.9732	-2.8608	345.012
759	0.9734	-2.8431	234.226
760	0.9754	-3.3681	51.0038
761	0.9763	-3.9504	358.749
762	0.9768	-2.7946	118.23
763	0.9771	-4.2304	87.7166
764	0.9777	-2.929	165.008
765	0.9781	-3.5358	354.496
766	0.9788	-3.3164	315.652
767	0.9805	-2.7898	22.5296
768	0.9812	-4.1735	302.049
769	0.9816	-3.0269	248.081
770	0.9818	-2.962	93.5039
771	0.9822	-2.9315	98.366
772	0.9827	-3.2075	174.126
773	0.9832	-2.7456	265.521
774	0.9836	-2.6858	76.8599
775	0.9837	-2.9388	351.799
776	0.9841	-2.8649	348.791
777	0.9854	-3.8957	40.786
778	0.9854	-3.4985	274.019
779	0.9859	-3.2906	139.93
780	0.9862	-2.887	217.817
781	0.9869	-2.8849	11.1408
782	0.9876	-3.2304	165.471
783	0.988	-3.1705	47.9027
784	0.9893	-3.0419	327.576
785	0.9898	-4.3655	140.402
786	0.991	-3.4705	141.661
787	0.9912	-3.5656	66.7148
788	0.9918	-4.0359	130.135
789	0.992	-2.9373	348.462
790	0.9927	-2.7655	158.555
791	0.9928	-2.8568	318.234
792	0.9933	-3.7538	142.768
793	0.9934	-3.6143	304.878
794	0.9934	-4.0286	316.458
795	0.9948	-2.859	22.7973
796	0.9961	-2.9496	20.1475
797	0.9964	-2.8128	197.759
798	0.9976	-3.9752	319.314
799	0.9979	-2.7295	163.804
800	0.9981	-3.9969	96.3162
801	0.9986	-2.9471	325.911
802	0.9995	-2.7905	55.9934
803	0.9996	-3.3615	42.1885
804	1.0012	-2.7551	311.828
805	1.0021	-2.7788	346.888

806	1.0027	-2.9353	251.047
807	1.0042	-2.9763	87.3258
808	1.0042	-3.297	139.734
809	1.0058	-2.9372	98.362
810	1.007	-2.7484	33.3903
811	1.0075	-4.3868	247.106
812	1.0081	-3.3935	348.37
813	1.0087	-3.4654	95.2407
814	1.0095	-4.24	97.1966
815	1.0096	-2.9431	254.203
816	1.0101	-3.1793	301.856
817	1.0106	-2.8168	202.891
818	1.0108	-3.9308	81.9295
819	1.0122	-3.2256	57.9548
820	1.0138	-3.0236	82.2084
821	1.0139	-2.8669	230.354
822	1.0141	-3.6261	22.8684
823	1.0143	-2.7517	169.074
824	1.0154	-2.9548	324.549
825	1.0155	-2.8344	104.028
826	1.0164	-2.9628	315.226
827	1.0167	-3.1613	292.496
828	1.0171	-2.9558	237.75
829	1.0175	-4.0667	149.233
830	1.0191	-2.7396	328.144
831	1.0193	-2.7342	245.804
832	1.0193	-3.8287	350.746
833	1.0194	-3.2991	324.107
834	1.021	-2.9522	317.988
835	1.0217	-3.5933	16.5677
836	1.0224	-3.8364	241.877
837	1.0228	-3.8093	146.424
838	1.0228	-2.7714	73.3348
839	1.0246	-3.8585	349.619
840	1.025	-3.0847	83.9158
841	1.0254	-3.444	314.504
842	1.0288	-2.7907	127.073
843	1.0299	-3.3551	321.444
844	1.0301	-3.0041	9.2994
845	1.0304	-2.8455	282.711
846	1.0307	-2.9313	236.041
847	1.0311	-4.0133	8.5436
848	1.0339	-2.7727	323.999
849	1.0346	-2.9249	306.335
850	1.0347	-3.1584	195.067
851	1.0347	-2.9332	276.587
852	1.0352	-2.9522	7.3357
853	1.0353	-3.0865	110.92

854	1.0353	-4.1666	151.232
855	1.0357	-3.0906	267.076
856	1.0373	-3.8865	260.813
857	1.039	-3.444	44.6956
858	1.041	-2.8393	128.376
859	1.0412	-3.0788	17.6043
860	1.0417	-3.2511	148.077
861	1.0421	-2.8633	194.169
862	1.0437	-2.8376	303.799
863	1.0441	-2.8425	23.7161
864	1.0442	-1.8275	184.911
865	1.0443	-3.6455	142.195
866	1.0445	-3.8517	252.176
867	1.0448	-2.9709	81.5324
868	1.0453	-4.5709	318.394
869	1.0464	-3.8832	58.4174
870	1.0469	-4.0482	350.454
871	1.0471	-2.7885	329.837
872	1.0476	-2.8636	105.767
873	1.0489	-4.4373	60.199
874	1.0494	-2.7647	352.588
875	1.0499	-2.8878	341.621
876	1.0502	-2.9619	288.424
877	1.0505	-2.918	7.385
878	1.051	-3.4456	10.8045
879	1.0525	-4.2285	310.675
880	1.0532	-3.4717	9.3356
881	1.0536	-2.936	24.5453
882	1.0537	-2.9825	305.012
883	1.0544	-3.6309	9.9798
884	1.0556	-2.935	176.349
885	1.057	-2.8936	272.52
886	1.0572	-2.7313	151.669
887	1.0582	-2.9354	47.264
888	1.06	-2.7837	190.968
889	1.0603	-2.898	264.877
890	1.0606	-2.8378	18.0475
891	1.0608	-3.583	74.5386
892	1.0608	-3.116	124.033
893	1.062	-4.2199	315.407
894	1.0629	-4.0424	349.238
895	1.063	-3.5765	36.8694
896	1.0641	-2.8255	204.632
897	1.0653	-2.9188	157.199
898	1.0653	-3.5523	354.439
899	1.0653	-2.7377	303.663
900	1.0656	-2.9552	11.3534
901	1.0658	-2.7305	330.214

902	1.0659	-2.9412	33.7231
903	1.0664	-4.0716	267.537
904	1.0665	-4.0419	332.918
905	1.0685	-4.0816	78.7027
906	1.0703	-2.9013	262.595
907	1.0705	-3.9498	101.417
908	1.0715	-3.2364	285.257
909	1.0725	-3.9298	14.0991
910	1.0729	-4.3461	253.714
911	1.0738	-3.3381	196.351
912	1.0739	-1.7536	12.3621
913	1.075	-2.8266	15.8626
914	1.0751	-2.785	8.3019
915	1.0763	-3.1461	306.495
916	1.077	-2.814	108.188
917	1.0779	-2.9699	319.215
918	1.078	-2.7909	68.5491
919	1.0783	-2.9524	325.006
920	1.079	-2.6783	111.89
921	1.0791	-2.9341	282.272
922	1.0793	-2.8412	351.283
923	1.0796	-2.8626	76.5171
924	1.0799	-3.7068	55.2758
925	1.0814	-2.8185	65.6115
926	1.0822	-2.9736	78.1294
927	1.083	-3.4184	30.9147
928	1.0851	-2.8903	323.067
929	1.0851	-2.8649	284.991
930	1.0865	-3.9004	100.769
931	1.0866	-2.9467	233.052
932	1.0877	-3.4943	217.572
933	1.0878	-2.7576	77.6889
934	1.0879	-2.7807	201.789
935	1.0887	-2.8885	1.6786
936	1.0912	-2.9328	326.89
937	1.0918	-4.4177	317.406
938	1.0958	-2.7779	72.5432
939	1.0959	-3.8449	31.9099
940	1.0962	-2.8489	301.071
941	1.097	-3.1742	329.384
942	1.0973	-4.1019	261.642
943	1.0978	-2.6496	72.4512
944	1.098	-2.8482	237.166
945	1.0982	-3.3555	63.1494
946	1.0994	-2.9569	351.103
947	1.0999	-3.3996	166.503
948	1.1006	-3.3145	74.0109
949	1.1014	-3.1891	138.264

950	1.1028	-3.9749	155.931
951	1.1032	-2.963	55.3737
952	1.1033	-2.8989	319.467
953	1.105	-2.9616	162.976
954	1.1066	-3.7858	5.9871
955	1.1066	-2.9345	32.2885
956	1.1085	-4.1312	280.842
957	1.1087	-3.1567	246.733
958	1.1098	-3.9633	347.863
959	1.1101	-3.0951	333.785
960	1.1112	-2.9582	84.8603
961	1.112	-2.9503	342.353
962	1.1139	-3.9154	234.728
963	1.1142	-2.9254	296.728
964	1.1159	-2.7736	231.75
965	1.1165	-2.9039	329.83
966	1.1168	-4.5436	54.7188
967	1.1174	-4.0847	56.5739
968	1.1178	-3.2696	107.96
969	1.1181	-3.0142	292.977
970	1.1182	-3.6681	349.767
971	1.1187	-3.8891	29.2528
972	1.1187	-3.0475	245.453
973	1.1187	-2.949	16.2547
974	1.1195	-2.8073	105.229
975	1.1199	-3.9026	82.3068
976	1.1199	-3.9792	118.227
977	1.1201	-2.8408	350.802
978	1.1205	-2.7696	164.977
979	1.1207	-2.8455	2.1061
980	1.1207	-2.9313	230.234
981	1.1209	-3.2375	22.4599
982	1.1213	-4.379	269.109
983	1.1218	-2.8287	203.989
984	1.1222	-4.2005	313.607
985	1.1222	-3.2606	108.73
986	1.1223	-2.7354	55.7096
987	1.1225	-2.9602	17.7822
988	1.1248	-4.2474	228.256
989	1.1248	-3.1661	48.5551
990	1.1273	-2.9334	142.688
991	1.1276	-2.951	291.072
992	1.1278	-2.7904	353.862
993	1.1283	-3.0975	9.9539
994	1.1288	-2.7355	207.906
995	1.1299	-2.7996	72.4097
996	1.1299	-2.8158	10.246
997	1.1302	-3.2165	134.845

998	1.1303	-2.7695	50.7005
999	1.1303	-3.6247	286.694
1000	1.1305	-3.0009	311.487
1001	1.1308	-3.1756	335.274
1002	1.131	-2.7937	345.424
1003	1.1314	-4.0852	341.037
1004	1.1321	-2.8648	347.996
1005	1.1325	-3.4769	36.8576
1006	1.1326	-2.9338	329.68
1007	1.1327	-3.6973	71.2623
1008	1.1331	-3.3114	104.81
1009	1.1334	-3.2465	130.45
1010	1.1338	-2.7406	164.161
1011	1.1339	-2.7569	337.95
1012	1.1347	-3.2233	135.851
1013	1.1353	-3.8666	45.4247
1014	1.1361	-3.0963	346.709
1015	1.1362	-3.8357	121.649
1016	1.1371	-2.9829	43.8331
1017	1.1374	-3.0446	95.0799
1018	1.1375	-2.9117	18.4013
1019	1.1376	-3.3277	40.2468
1020	1.1391	-2.8077	39.9852
1021	1.1399	-3.2553	163.906
1022	1.1404	-3.8114	106.634
1023	1.1407	-3.2023	275.555
1024	1.1409	-2.9963	22.261
1025	1.1409	-3.3482	147.369
1026	1.1411	-2.9532	317.523
1027	1.1411	-3.3174	164.103
1028	1.1415	-3.9729	13.7831
1029	1.142	-4.3765	160.943
1030	1.1422	-2.7463	13.0232
1031	1.1423	-2.9233	270.316
1032	1.1426	-2.7746	52.781
1033	1.1434	-2.9148	49.9293
1034	1.1451	-2.8047	3.1078
1035	1.1463	-2.8173	59.2167
1036	1.1474	-4.3462	228.582
1037	1.148	-2.9012	328.118
1038	1.1485	-2.9262	258.782
1039	1.1488	-2.9375	296.314
1040	1.149	-3.3033	64.2759
1041	1.149	-3.2819	32.4815
1042	1.1496	-4.3308	273.365
1043	1.1525	-3.8033	271.605
1044	1.1525	-2.812	133.329
1045	1.1525	-2.8618	54.4847

1046	1.1526	-3.7396	294.386
1047	1.1533	-3.2124	43.8084
1048	1.1533	-3.927	166.31
1049	1.1536	-2.9015	50.5131
1050	1.1549	-3.5138	47.4493
1051	1.1552	-3.1247	179.967
1052	1.156	-3.0246	327.917
1053	1.156	-2.794	342.059
1054	1.1573	-2.7573	122.204
1055	1.1581	-4.0744	131.366
1056	1.1581	-2.7257	334.268
1057	1.1594	-2.9179	217.535
1058	1.1594	-3.2325	188.205
1059	1.1596	-3.6909	159.283
1060	1.1598	-2.7447	51.0208
1061	1.1599	-3.4319	53.24
1062	1.16	-2.8506	112.98
1063	1.1601	-2.8266	260.586
1064	1.1602	-2.9062	190.473
1065	1.1613	-2.7566	55.5948
1066	1.1617	-2.9537	13.5049
1067	1.1619	-2.9549	34.6684
1068	1.162	-3.0989	338.626
1069	1.1628	-4.2366	319.202
1070	1.1644	-2.8099	354.278
1071	1.1649	-3.7074	88.5728
1072	1.1652	-3.3804	147.024
1073	1.1658	-2.9359	250.775
1074	1.167	-2.7918	119.797
1075	1.1671	-2.9672	296.502
1076	1.1671	-2.786	345.435
1077	1.1678	-2.9693	305.467
1078	1.1679	-2.9045	151.918
1079	1.1679	-2.9159	327.343
1080	1.1679	-2.8415	165.797
1081	1.1687	-2.7393	163.342
1082	1.1701	-3.243	233.037
1083	1.1705	-2.7995	73.4271
1084	1.1707	-2.687	352.711
1085	1.1716	-3.3503	112.289
1086	1.1718	-3.2869	154.249
1087	1.1718	-2.8526	163.596
1088	1.1723	-5.8599	352.054
1089	1.1739	-2.7628	62.5758
1090	1.174	-2.6967	162.195
1091	1.1741	-2.8987	4.9849
1092	1.1749	-2.9377	8.9002
1093	1.1755	-4.0853	353.348

1094	1.1756	-2.9906	28.814
1095	1.1757	-3.4528	313.91
1096	1.1761	-2.7447	163.917
1097	1.1765	-2.6907	0.1906
1098	1.1765	-2.7871	128.235
1099	1.1774	-2.8617	20.6236
1100	1.1776	-3.3957	183.335
1101	1.1785	-2.9377	115.738
1102	1.1787	-2.7544	12.6629
1103	1.1788	-2.8863	216.844
1104	1.1797	-4.048	8.6116
1105	1.1801	-2.7802	301.114
1106	1.1824	-3.5071	189.213
1107	1.1824	-4.0125	316.198
1108	1.1825	-3.989	193.452
1109	1.1826	-3.3222	333.47
1110	1.1827	-2.9005	216.152
1111	1.1829	-3.2976	61.884
1112	1.1832	-3.3342	330.713
1113	1.1833	-3.3225	323.423
1114	1.1835	-4.145	307.126
1115	1.1837	-2.9374	354.398
1116	1.1837	-3.8727	79.9945
1117	1.1838	-4.3585	141.948
1118	1.1838	-2.9012	338.742
1119	1.1843	-4.0921	304.305
1120	1.1848	-2.9234	305.349
1121	1.1855	1.2331	110.419
1122	1.1858	-2.9	141.572
1123	1.1861	-2.6777	157.027
1124	1.187	-2.7419	153
1125	1.1876	-3.4402	135.286
1126	1.1876	-3.9133	18.6941
1127	1.1878	-3.1441	60.3547
1128	1.1879	-3.514	257.158
1129	1.1881	-3.0721	282.151
1130	1.1882	-2.9707	87.5474
1131	1.1886	-2.8545	128.11
1132	1.1887	-3.8916	348.287
1133	1.1892	-4.1061	334.747
1134	1.1898	-3.4469	236.029
1135	1.1904	-2.7792	119.357
1136	1.1906	-2.9066	19.2035
1137	1.1908	-2.86	286.189
1138	1.1911	-2.9556	142.478
1139	1.1913	-2.9479	351.529
1140	1.1913	-2.7993	3.9585
1141	1.1917	-3.4416	165.994

1142	1.1917	-2.7857	42.8664
1143	1.1922	-2.8617	122.462
1144	1.1939	-3.3399	244.48
1145	1.194	-2.8239	60.9397
1146	1.1948	-2.7922	152.126
1147	1.195	-2.8746	251.79
1148	1.1954	-2.7821	147.684
1149	1.1957	-4.319	341.787
1150	1.1962	-2.7487	275.31
1151	1.1963	-3.1292	60.6755
1152	1.1969	-4.158	248.123
1153	1.1975	-2.9996	42.6278
1154	1.1976	-3.5352	18.6852
1155	1.1985	-2.9044	59.0046
1156	1.1985	-2.9363	356.408
1157	1.1988	-2.7559	20.4959
1158	1.1992	-3.2437	90.0695
1159	1.1998	-2.7352	347.854
1160	1.2002	-3.1172	158.467
1161	1.2006	-2.7959	22.0897
1162	1.2019	-2.7982	10.4095
1163	1.2021	-2.8563	83.7618
1164	1.2024	-3.2474	277.544
1165	1.2028	-3.3611	291.626
1166	1.2036	-2.8316	139.078
1167	1.204	-2.8283	107.941
1168	1.2045	-4.0034	256.082
1169	1.2048	-2.7821	209.029
1170	1.205	-2.7844	321.439
1171	1.2055	-4.194	331.444
1172	1.2056	-2.952	121.884
1173	1.2057	-3.5295	102.528
1174	1.2063	-3.1265	310.444
1175	1.2068	-4.1927	168.703
1176	1.2074	-2.814	56.0495
1177	1.2077	-4.1701	278.916
1178	1.2079	-2.8585	320.911
1179	1.2085	-3.1528	166.42
1180	1.2088	-3.0962	326.601
1181	1.2092	-2.7363	245.504
1182	1.2096	-2.876	82.8759
1183	1.2099	-2.81	2.6738
1184	1.2099	-2.8377	113.3
1185	1.21	-2.9329	358.524
1186	1.2101	-2.8279	130.705
1187	1.2109	-3.9951	340.314
1188	1.211	-3.9291	342.059
1189	1.211	-2.8641	293.393

1190	1.2112	-3.6676	10.6099
1191	1.2119	-2.9844	119.457
1192	1.212	-2.9621	43.6023
1193	1.2125	-2.8089	254.764
1194	1.2131	-3.9003	109.485
1195	1.214	-3.9648	68.2017
1196	1.2142	-2.802	127.694
1197	1.2143	-3.214	143.014
1198	1.2147	-2.8533	354.268
1199	1.215	-3.5286	285.667
1200	1.2154	-3.1683	317.583
1201	1.2154	-3.2316	122.468
1202	1.2161	-2.8645	171.916
1203	1.2166	-2.838	131.919
1204	1.2166	-3.9137	16.4124
1205	1.2168	-2.8351	335.897
1206	1.2173	-2.8591	131.211
1207	1.2175	-3.0456	27.9651
1208	1.2177	-3.8417	325.073
1209	1.2183	-3.7233	275.315
1210	1.2187	-2.7421	172.23
1211	1.2188	-2.7513	87.1912
1212	1.2191	-2.6554	352.451
1213	1.2193	-2.85	252.06
1214	1.2195	-3.1545	330.117
1215	1.2197	-2.7263	96.9454
1216	1.2199	-3.0531	76.9926
1217	1.2201	-3.0818	64.7486
1218	1.2206	-2.8681	348.666
1219	1.2208	-3.8956	18.707
1220	1.2216	-2.9753	22.6867
1221	1.2226	-2.7618	0.2766
1222	1.2234	-2.9074	300.283
1223	1.2243	-4.399	261.662
1224	1.2255	-3.0985	38.0688
1225	1.2259	-3.8717	294.347
1226	1.226	-3.1926	12.9054
1227	1.2263	-2.9141	196.79
1228	1.2269	-2.8039	316.404
1229	1.227	-2.85	116.878
1230	1.2275	-3.4547	19.8097
1231	1.2291	-4.01	68.0012
1232	1.2292	-2.9214	131.899
1233	1.2294	-4.1234	154.417
1234	1.23	-2.9244	141.489
1235	1.2303	-3.1935	305.389
1236	1.2317	-2.8704	194.895
1237	1.2319	-2.8446	174.389

1238	1.232	-2.7361	37.6602
1239	1.2326	-2.9939	28.1104
1240	1.233	-4.2892	307.497
1241	1.2332	-2.8054	119.257
1242	1.2344	-2.8454	31.1228
1243	1.2347	-2.9218	73.3376
1244	1.2348	-2.7868	342.176
1245	1.2349	-3.0824	48.1461
1246	1.2349	-4.2574	16.827
1247	1.2349	-2.7576	275.479
1248	1.2359	-2.8222	161.151
1249	1.2359	-2.937	191.313
1250	1.236	-2.9253	320.108
1251	1.2366	-3.9915	32.0407
1252	1.2367	-2.7035	254.665
1253	1.2371	-3.9142	50.4854
1254	1.2374	-2.7605	304.653
1255	1.2376	-2.7796	41.6356
1256	1.2379	-3.8632	171.954
1257	1.2379	-2.8573	234.154
1258	1.2382	-2.9143	223.145
1259	1.2382	-2.9125	349.629
1260	1.2385	-2.8503	303.292
1261	1.2386	-3.6792	323.673
1262	1.2387	-2.8183	268.296
1263	1.2388	-2.9527	327.298
1264	1.2388	-3.2186	301.211
1265	1.2402	-2.8095	145.241
1266	1.2404	-3.3414	324.859
1267	1.2411	-3.328	115.204
1268	1.2412	-2.9805	120.118
1269	1.2412	-2.9551	202.895
1270	1.2413	-3.6089	117.043
1271	1.2414	-2.6763	358.464
1272	1.2423	-2.7968	315.87
1273	1.2432	-2.8964	161.885
1274	1.2432	-3.065	342.608
1275	1.2433	-4.3882	320.551
1276	1.2433	-2.7756	7.8984
1277	1.2441	-2.9071	4.1838
1278	1.2444	-3.1348	91.2164
1279	1.2462	-3.8538	323.192
1280	1.2491	-2.9577	25.495
1281	1.25	-2.9426	32.6703
1282	1.2502	-2.855	34.5881
1283	1.2504	-2.9436	326.329
1284	1.2504	-3.2322	38.6642
1285	1.2509	-2.6718	165.147

1286	1.253	1.5748	98.2923
1287	1.2531	-2.9465	138.473
1288	1.2534	-2.7914	63.6388
1289	1.2535	-2.8846	190.601
1290	1.2537	-2.9276	243.732
1291	1.2541	-3.1453	358.986
1292	1.2542	-2.9322	56.3797
1293	1.2544	-4.0212	267.052
1294	1.2545	-2.7846	159.012
1295	1.2548	-2.937	343.902
1296	1.2551	-2.9203	339.6
1297	1.2553	-3.5173	352.917
1298	1.2558	-2.8044	64.8888
1299	1.2565	-2.954	345.557
1300	1.2569	-2.9261	187.516
1301	1.257	-3.6687	201.565
1302	1.2577	-3.7938	294.905
1303	1.258	-2.8038	256.674
1304	1.2581	-3.3156	31.1874
1305	1.2588	-3.0593	104.168
1306	1.2602	-2.9654	257.774
1307	1.2603	-2.9491	229.251
1308	1.2606	-3.6817	308.345
1309	1.2611	-2.9675	56.2337
1310	1.2614	-3.6853	44.912
1311	1.2615	-2.9769	312.35
1312	1.2618	-3.2321	221.7
1313	1.2622	-3.3602	4.7104
1314	1.2623	-2.823	357.702
1315	1.2632	-2.9324	11.3021
1316	1.2632	-2.9714	109.082
1317	1.2636	-3.7021	351.81
1318	1.2637	-2.7689	69.8401
1319	1.2637	-2.9494	76.6965
1320	1.2638	-2.8953	29.173
1321	1.2645	-3.5354	330.169
1322	1.2649	-4.2399	3.344
1323	1.265	-2.8575	229.194
1324	1.2656	-3.0065	199.316
1325	1.2656	-4.3198	202.607
1326	1.266	-4.2653	311.635
1327	1.2672	-2.8735	168.79
1328	1.2673	-2.8057	118.545
1329	1.2677	-2.9516	280.007
1330	1.2681	-3.2142	100.964
1331	1.2685	-3.2623	165.026
1332	1.271	-3.8918	151.018
1333	1.271	-3.9316	18.7716

1334	1.2711	-2.9164	321.945
1335	1.2715	-2.7385	39.0077
1336	1.2717	-3.4164	91.0121
1337	1.2725	-3.2497	41.3085
1338	1.2731	-3.3207	50.5952
1339	1.2732	-2.7085	278.553
1340	1.2734	-4.0335	347.856
1341	1.2741	-3.3922	56.5101
1342	1.2742	-2.8152	79.5161
1343	1.2743	-3.5519	190.942
1344	1.2747	1.2863	108.461
1345	1.2748	-2.8328	11.8854
1346	1.2758	-2.8632	190.426
1347	1.2759	-2.9238	149.159
1348	1.277	-3.1884	315.81
1349	1.2772	-2.9384	45.7389
1350	1.2773	-3.8496	92.7057
1351	1.2775	-3.3272	295.954
1352	1.2777	-2.8619	281.813
1353	1.2779	-2.8527	298.806
1354	1.2784	-2.8979	296.337
1355	1.2785	-2.8885	27.1211
1356	1.2786	-4.0062	272.43
1357	1.2788	-4.2911	290.628
1358	1.2788	-2.8492	328.688
1359	1.2791	-3.4663	153.414
1360	1.2792	-2.9137	125.575
1361	1.2794	-2.7355	3.9035
1362	1.2795	-3.787	33.6239
1363	1.2812	-2.781	331.76
1364	1.2813	-2.686	314.255
1365	1.2826	-3.3485	17.3165
1366	1.2834	-2.832	290.138
1367	1.2837	-3.9764	328.778
1368	1.2844	-4.001	11.7374
1369	1.286	-4.0169	167.917
1370	1.2862	-3.4997	124.089
1371	1.2864	-2.9551	283.669
1372	1.2872	-4.0561	318.108
1373	1.2872	-2.9423	120.597
1374	1.2874	-3.8249	317.748
1375	1.2877	-2.7843	254.088
1376	1.2884	-4.1292	33.5754
1377	1.2887	-3.9622	162.393
1378	1.2888	-2.8808	207.922
1379	1.2895	-3.9088	169.497
1380	1.2896	-3.8346	328.812
1381	1.2896	-3.0572	290.828

1382	1.2904	-2.9142	168.99
1383	1.2904	-2.8607	319.416
1384	1.291	-2.6625	320.417
1385	1.2911	-3.6365	350.593
1386	1.2914	-3.5553	261.111
1387	1.2914	-2.7728	281.422
1388	1.2914	-3.8555	248.138
1389	1.2916	-2.9743	285.895
1390	1.2917	-2.8532	272.416
1391	1.2923	-3.6961	291.288
1392	1.2923	-5.8437	253.703
1393	1.2923	-2.8105	352.374
1394	1.2932	-4.0046	90.8156
1395	1.2938	-2.9499	120.533
1396	1.2945	-2.9648	318.91
1397	1.2953	-3.6615	218.94
1398	1.2957	-2.9098	114.157
1399	1.2958	-3.1761	260.198
1400	1.2965	-2.6777	29.5145
1401	1.2968	-3.8318	177.906
1402	1.2971	-2.9203	140.433
1403	1.2977	-2.8763	53.9861
1404	1.2978	-3.8737	65.8752
1405	1.2982	-3.1292	321.648
1406	1.2984	-3.1959	146.447
1407	1.2991	-2.9595	333.867
1408	1.2995	-3.1644	263.125
1409	1.2999	-2.9702	68.7923
1410	1.3	-2.9537	46.4316
1411	1.3	-3.8307	265.778
1412	1.3001	-2.7522	273.646
1413	1.3008	-3.6951	174.068
1414	1.3016	-2.7976	233.778
1415	1.3019	-2.7912	149.862
1416	1.3022	-4.2212	283.417
1417	1.3026	-3.2598	128.566
1418	1.3027	-3.4163	276.133
1419	1.3029	-2.8761	64.5778
1420	1.3032	-2.8692	180.138
1421	1.3032	-3.1046	331.276
1422	1.3033	-2.8194	103.06
1423	1.3035	-3.4645	78.2324
1424	1.3036	-2.8655	135.145
1425	1.3041	-2.8561	109.487
1426	1.3042	-3.8778	168.864
1427	1.3042	-2.9405	39.5516
1428	1.3043	-2.9168	284.997
1429	1.3045	-3.0862	321.014

1430	1.3055	-2.9598	3.6303
1431	1.306	-2.8235	254.544
1432	1.307	-2.871	270.709
1433	1.3075	-3.2281	90.2992
1434	1.3076	-2.9672	358.904
1435	1.3078	-2.9334	323.904
1436	1.3081	-3.1284	183.54
1437	1.3081	-2.8236	226.128
1438	1.3082	-2.8624	304.923
1439	1.3089	-3.2893	64.8238
1440	1.309	-2.9344	53.8807
1441	1.309	-3.439	5.1667
1442	1.3092	-3.0071	18.807
1443	1.3096	-3.9844	41.8951
1444	1.3105	-3.679	300.275
1445	1.3119	-3.1491	192.702
1446	1.312	-2.9678	348.186
1447	1.3122	-2.9934	85.2127
1448	1.3126	-2.9411	329.347
1449	1.3126	-4.07	183.138
1450	1.313	-3.9242	115.966
1451	1.3131	-3.9417	77.2476
1452	1.3132	-2.9312	11.8065
1453	1.3134	-2.75	329.406
1454	1.3144	-2.9296	324.295
1455	1.3145	-2.9408	348.61
1456	1.3151	-2.9316	313.953
1457	1.3159	-2.973	329.362
1458	1.316	-2.8342	343.312
1459	1.3165	-2.987	75.7633
1460	1.3165	-4.3196	226.163
1461	1.3167	-2.9745	60.447
1462	1.3169	-2.8451	16.9605
1463	1.3182	-3.1999	249.829
1464	1.3184	-3.0246	318.444
1465	1.3184	-2.7867	79.7029
1466	1.3186	-2.7673	49.3214
1467	1.319	-2.7867	11.7716
1468	1.319	-2.7304	314.018
1469	1.3195	-2.742	53.1068
1470	1.3197	-2.8753	298.226
1471	1.3198	-2.7694	77.1931
1472	1.3202	-2.8217	196.645
1473	1.3212	-3.4776	313.41
1474	1.3215	-2.956	186.435
1475	1.3215	-3.6349	166.087
1476	1.3217	-3.3585	156.071
1477	1.3223	-3.201	150.56

1478	1.3223	-2.965	298.903
1479	1.3225	-3.2117	228.597
1480	1.3229	-3.878	216.109
1481	1.3238	-2.9152	183.38
1482	1.3245	-3.4096	192.441
1483	1.3251	-3.0757	298.062
1484	1.3253	-4.0037	112.775
1485	1.3255	-2.9586	22.6517
1486	1.3255	-3.3041	25.1562
1487	1.3256	-2.9183	172.143
1488	1.3257	-2.8538	340.745
1489	1.3262	-3.6625	100.072
1490	1.3264	-2.7911	59.4927
1491	1.3265	-3.9087	39.9106
1492	1.3271	-2.8449	293.968
1493	1.3279	-4.1523	16.1788
1494	1.3291	-2.7647	254.798
1495	1.3295	-3.0081	204.689
1496	1.3304	-4.177	345.265
1497	1.3315	-3.4233	359.161
1498	1.3323	-2.7437	348.971
1499	1.3334	-3.9128	73.9442
1500	1.3334	-2.7745	0.0514
1501	1.3336	-2.8556	66.0407
1502	1.3339	-2.6975	310.691
1503	1.3342	-2.853	301
1504	1.3346	-3.0186	344.533
1505	1.3346	-3.9087	145.71
1506	1.3348	-2.8767	131.405
1507	1.3352	-2.9392	185.237
1508	1.336	-2.9551	24.7061
1509	1.336	-2.8098	106.042
1510	1.3371	-3.2311	128.4
1511	1.3379	-3.5452	136.796
1512	1.3386	-3.3663	37.0358
1513	1.3391	-2.966	260.169
1514	1.3392	-2.8139	27.6835
1515	1.3399	-2.951	328.528
1516	1.3401	-2.7784	22.1686
1517	1.3408	-4.5761	316.419
1518	1.3418	-3.179	295.864
1519	1.3419	-2.9241	230.611
1520	1.3422	-3.8428	14.7929
1521	1.3428	-3.3365	254.058
1522	1.3431	-2.7606	355.906
1523	1.3437	-3.973	181.224
1524	1.3442	-2.8511	192.704
1525	1.3443	-2.8581	325.314

1526	1.3448	-3.8284	322.585
1527	1.3451	-3.1365	356.754
1528	1.3455	-4.1987	275.867
1529	1.3467	-2.8261	5.4417
1530	1.3468	-3.7818	43.3704
1531	1.3474	-3.2234	258.639
1532	1.3475	-3.0006	4.5737
1533	1.3477	-2.9366	29.6127
1534	1.3477	-3.5144	267.01
1535	1.3484	-3.0224	338.769
1536	1.3487	-4.4273	266.561
1537	1.3487	-2.8021	161.177
1538	1.3488	-2.9778	25.717
1539	1.349	-3.6021	179.535
1540	1.3493	-3.0792	315.074
1541	1.3497	-2.919	77.1002
1542	1.3498	-2.9043	149.099
1543	1.35	-2.905	86.1856
1544	1.3501	-3.0004	317.203
1545	1.3511	-3.5538	142.589
1546	1.3515	-2.7415	144.066
1547	1.3516	-2.8837	70.3721
1548	1.3519	-3.5006	354.603
1549	1.3523	-2.8644	183.846
1550	1.3524	-3.9746	262.257
1551	1.3526	-2.9525	15.1885
1552	1.353	-4.611	308.27
1553	1.3532	-2.8583	247.71
1554	1.3535	-2.8637	110.15
1555	1.3537	5.8173	97.1796
1556	1.3541	-3.2764	0.5171
1557	1.3544	-2.751	287.382
1558	1.3545	-2.8122	187.349
1559	1.3551	-3.1121	313.069
1560	1.3552	-3.8643	232.15
1561	1.3553	-3.3068	291.521
1562	1.3553	-2.8289	84.3346
1563	1.3555	-4.105	105.093
1564	1.3559	-3.3379	329.993
1565	1.3567	-2.8991	268.557
1566	1.3576	-2.9209	292.47
1567	1.358	-3.9258	53.8445
1568	1.3582	-2.9161	31.9181
1569	1.3589	-2.7679	47.4132
1570	1.3598	-3.7541	147.736
1571	1.3599	-4.1003	212.249
1572	1.3601	-3.8655	346.92
1573	1.3605	-2.956	114.41

1574	1.3615	-2.7887	310.141
1575	1.3619	-3.4188	314.713
1576	1.3622	-2.977	185.243
1577	1.3625	-2.7934	112.871
1578	1.3625	-2.7663	350.66
1579	1.3632	-3.9038	24.5766
1580	1.3632	-4.207	1.1906
1581	1.3634	-2.854	131.99
1582	1.3635	-2.8879	203.417
1583	1.3639	-3.9865	255.229
1584	1.3643	-3.9766	330.4
1585	1.3652	-2.7635	56.7456
1586	1.3653	-2.6494	276.353
1587	1.3654	-3.1295	296.363
1588	1.3658	-3.7347	0.8864
1589	1.3661	-2.9608	127.994
1590	1.3661	-2.8501	256.951
1591	1.3667	-2.7604	351.384
1592	1.3676	-2.8443	13.5811
1593	1.3677	-3.6836	182.022
1594	1.3681	-2.851	53.315
1595	1.3683	-3.1858	129.016
1596	1.3692	-3.0158	221.549
1597	1.3696	-3.2812	233.629
1598	1.3697	-2.7601	327.156
1599	1.3699	-4.3514	356.609
1600	1.3699	-2.942	296.251
1601	1.3704	-2.713	323.741
1602	1.3706	-3.123	322.033
1603	1.3716	-3.9132	6.734
1604	1.3718	-2.8876	180.635
1605	1.3718	-2.8907	123.786
1606	1.3719	-2.8288	152.816
1607	1.372	-2.7799	336.688
1608	1.3724	-2.9516	161.282
1609	1.373	-3.1611	301.78
1610	1.3732	-2.8701	72.1026
1611	1.3738	-3.653	251.834
1612	1.3739	-4.421	70.9865
1613	1.3739	-3.1886	38.7617
1614	1.3743	-2.9253	342.262
1615	1.3743	-3.0003	167.111
1616	1.3745	-2.9448	281.974
1617	1.3747	-2.7958	107.015
1618	1.3747	-2.9074	315.392
1619	1.3751	-3.4477	22.2154
1620	1.3751	-2.8432	144.225
1621	1.3753	-2.8679	58.6799

1622	1.3753	-2.9135	256.001
1623	1.376	-4.2529	250.399
1624	1.3762	-2.9811	165.966
1625	1.3765	-2.8167	288.702
1626	1.3765	-3.0031	79.2614
1627	1.3776	-3.3798	346.656
1628	1.3777	-4.4506	272.99
1629	1.3779	-2.7245	283.354
1630	1.3789	-3.1248	234.205
1631	1.3796	-3.7896	38.7341
1632	1.3805	-2.8717	331.925
1633	1.3805	-3.3157	62.9632
1634	1.3808	-2.9851	298.299
1635	1.3809	-3.0917	318.416
1636	1.3815	-2.9545	11.5097
1637	1.3819	-4.1997	181.698
1638	1.3819	-3.5463	239.097
1639	1.3821	-3.6785	122.703
1640	1.3829	-2.8766	341.518
1641	1.3829	-2.7856	117.677
1642	1.3829	-3.6707	60.559
1643	1.3833	-4.4269	263.309
1644	1.3834	-3.9383	41.022
1645	1.3836	-3.4375	348.689
1646	1.3836	-2.8742	335.651
1647	1.3838	-3.893	5.0297
1648	1.3841	-3.4006	10.0419
1649	1.3842	-2.8464	61.8059
1650	1.385	-2.9574	330.899
1651	1.3852	-4.0651	277.643
1652	1.3852	-2.8908	20.3531
1653	1.3853	-2.8455	334.276
1654	1.3856	-3.7932	174.894
1655	1.3856	-2.9536	123.35
1656	1.3857	-4.2189	279.543
1657	1.386	-2.8637	110.315
1658	1.3863	-2.8844	123.225
1659	1.3867	-3.8964	134.201
1660	1.3873	-3.3768	199.958
1661	1.3874	-3.9614	349.206
1662	1.3877	-3.4674	43.5481
1663	1.3877	-2.7797	20.8983
1664	1.388	-2.9061	26.6475
1665	1.3889	-3.9827	111.955
1666	1.3892	-2.95	66.2473
1667	1.3895	-4.0988	319.39
1668	1.39	-2.8415	95.6913
1669	1.3907	-4.186	316.941

1670	1.3914	-4.253	335.372
1671	1.3925	-3.2894	206.307
1672	1.3927	-2.959	198.955
1673	1.393	-3.9351	18.5766
1674	1.3931	-3.3445	273.108
1675	1.3931	-3.2724	124.398
1676	1.3935	-2.9202	14.6856
1677	1.3935	-3.7912	45.1229
1678	1.3944	-3.4306	136.863
1679	1.3944	-3.0987	246.783
1680	1.3945	-2.8555	324.141
1681	1.3945	-3.9406	192.034
1682	1.3951	-2.9224	290.451
1683	1.3957	-3.208	322.501
1684	1.3962	-2.8283	199.238
1685	1.3963	-2.7813	1.7654
1686	1.3964	-4.368	71.5796
1687	1.3966	-4.1903	81.91
1688	1.3978	-2.9609	264.935
1689	1.3979	-4.0018	352.102
1690	1.3981	-2.9667	79.8012
1691	1.3982	-3.9361	15.8199
1692	1.3985	-4.0722	178.245
1693	1.3995	-3.1899	172.911
1694	1.4002	-2.9435	79.9165
1695	1.4011	-3.1285	16.2645
1696	1.4011	-2.883	201.706
1697	1.4018	-3.9618	162.578
1698	1.4018	-3.2652	146.127
1699	1.4025	-2.8134	105.869
1700	1.4027	-2.9378	249.735
1701	1.4031	-3.9385	353.278
1702	1.4031	-3.5384	347.917
1703	1.4033	-2.039	80.4097
1704	1.4033	-3.5239	95.3618
1705	1.4045	-2.8104	22.349
1706	1.4047	-3.1009	348.648
1707	1.4049	-3.6223	355.779
1708	1.4056	-3.0224	83.635
1709	1.4056	-2.7976	95.0352
1710	1.4056	-3.84	342.457
1711	1.4064	-2.9594	316.949
1712	1.4067	-2.8833	86.561
1713	1.4067	-3.1632	119.926
1714	1.4091	-3.8356	42.1949
1715	1.4091	-3.9165	112.703
1716	1.4095	-3.7234	67.175
1717	1.4102	-4.6651	185.408

1718	1.4111	-2.8089	72.2291
1719	1.4119	-4.3314	351.115
1720	1.412	-2.9183	318.194
1721	1.4121	-2.8556	291.324
1722	1.4122	-2.8938	89.7211
1723	1.4125	-2.865	23.2254
1724	1.4125	-2.877	332.807
1725	1.4134	-4.0505	354.806
1726	1.4135	-2.8979	152.357
1727	1.4137	-2.9336	5.3588
1728	1.4137	-2.8082	134.334
1729	1.4137	-3.7052	102.349
1730	1.4137	-2.946	281.003
1731	1.4146	-2.7818	352.708
1732	1.4147	-2.8484	320.629
1733	1.4155	-2.9763	31.5671
1734	1.4161	-3.7562	75.2151
1735	1.4163	-2.8643	96.2913
1736	1.4164	-2.9153	170.574
1737	1.4166	-3.328	292.944
1738	1.4166	-2.9443	320.814
1739	1.4166	-4.0471	296.714
1740	1.4172	-4.1954	133.741
1741	1.4174	-2.9136	38.1717
1742	1.4182	-2.8289	124.028
1743	1.4183	-2.9178	65.431
1744	1.4183	-4.0242	347.691
1745	1.4185	-3.3429	169.132
1746	1.4186	-4.198	124.985
1747	1.4189	-2.8638	315.416
1748	1.419	-3.3126	78.7852
1749	1.4191	-4.0938	195.518
1750	1.4196	-3.3533	194.117
1751	1.42	-2.9124	123.609
1752	1.4205	-3.7788	177.994
1753	1.4205	-3.0205	167.972
1754	1.4205	-2.8187	271.307
1755	1.4207	-2.8353	122.211
1756	1.4207	-3.1677	200.148
1757	1.4207	-2.7849	142.069
1758	1.4209	-3.6264	290.894
1759	1.421	-3.431	58.1287
1760	1.4211	-2.9715	52.6258
1761	1.4219	-3.818	92.5694
1762	1.4222	-3.0736	66.5652
1763	1.4232	-3.9962	90.4725
1764	1.4237	-3.1488	294.812
1765	1.4238	-4.0187	349.449

1766	1.4242	-2.9257	40.0355
1767	1.4242	-2.7999	105.633
1768	1.4248	-2.9656	67.4245
1769	1.425	-3.173	282.096
1770	1.4251	-3.9379	261.473
1771	1.4251	-2.9417	357.207
1772	1.4253	-3.9453	85.9891
1773	1.4255	-3.9112	102.816
1774	1.4255	-2.7336	358.218
1775	1.4263	-2.9219	91.0861
1776	1.4268	-2.716	359.864
1777	1.4273	-2.8623	118.338
1778	1.4281	-2.9279	61.221
1779	1.4281	-2.9047	303.824
1780	1.4285	-3.0521	285.16
1781	1.4286	-3.6141	136.663
1782	1.4289	-3.0455	264.992
1783	1.4299	-2.8407	101.277
1784	1.4305	-2.7903	173.023
1785	1.4308	-2.8307	94.245
1786	1.4308	-3.7169	117.861
1787	1.4311	-3.5701	54.6274
1788	1.4313	-2.7625	64.9588
1789	1.4315	-3.1974	20.7002
1790	1.4316	-2.6935	267.886
1791	1.4321	-3.819	66.637
1792	1.4323	-2.77	105.274
1793	1.4326	-4.0779	225.55
1794	1.4331	-1.4382	88.7949
1795	1.4336	-4.6012	199.21
1796	1.4337	-3.1981	281.935
1797	1.4338	-3.6702	343.465
1798	1.4339	-3.4958	120.556
1799	1.4348	-3.4897	125.214
1800	1.4348	-2.8664	1.7522
1801	1.4356	-2.967	335.402
1802	1.4356	-3.9356	282.951
1803	1.4356	-2.8925	33.3348
1804	1.4356	-3.4443	339.654
1805	1.4356	-3.3301	334.003
1806	1.4357	-2.8949	30.2999
1807	1.4359	-3.5961	226.366
1808	1.4364	-2.8421	331.427
1809	1.4365	-2.9836	312.146
1810	1.4365	-2.7576	340.392
1811	1.4369	-2.9383	292.023
1812	1.4379	-3.7659	221.373
1813	1.4383	-3.8293	204.921

1814	1.4383	-2.7378	342.827
1815	1.4385	-2.8311	122.738
1816	1.4386	-2.9362	329.574
1817	1.4387	-3.8261	326.422
1818	1.4392	-3.9778	16.6776
1819	1.4402	-2.9839	243.834
1820	1.4404	-3.1788	273.572
1821	1.4407	-2.7695	21.9923
1822	1.4409	-2.775	190.793
1823	1.441	-2.7827	43.7303
1824	1.441	-2.9741	70.4332
1825	1.4414	-2.9088	270.778
1826	1.4418	-3.1825	243.575
1827	1.4423	-4.1016	268.276
1828	1.4424	-3.3534	336.754
1829	1.4434	-2.7778	11.7233
1830	1.4436	-2.8856	83.8882
1831	1.444	-2.7087	314.634
1832	1.4441	-4.5161	297.856
1833	1.4452	-2.8358	308.897
1834	1.4454	-3.6788	36.4621
1835	1.4457	-2.8841	143.372
1836	1.4464	-2.806	314.368
1837	1.4467	-3.6609	271.432
1838	1.4471	-3.8351	113.239
1839	1.4475	-3.9641	180.637
1840	1.4478	-4.2661	45.4672
1841	1.4479	-2.926	166.624
1842	1.448	-2.8794	359.361
1843	1.4481	-2.9397	16.2022
1844	1.4487	-2.9915	229.035
1845	1.449	-2.8181	350.887
1846	1.4491	-2.8563	32.9606
1847	1.4492	-3.1311	79.2112
1848	1.4496	-2.8517	308.934
1849	1.4502	-2.7965	296.311
1850	1.4503	-2.9094	300.826
1851	1.451	-2.7993	348.515
1852	1.4511	-3.0876	72.7699
1853	1.4511	-4.2483	321.537
1854	1.4513	-2.8095	303.021
1855	1.4514	-3.6684	350.113
1856	1.4516	-2.8696	272.112
1857	1.4519	-4.1153	223.657
1858	1.4521	-3.2787	176.725
1859	1.4523	-2.8854	10.0654
1860	1.4523	-2.8116	154.175
1861	1.4523	-2.8384	256.508

1862	1.4531	-2.8453	305.842
1863	1.4535	-2.9397	123.106
1864	1.4538	-2.9127	36.0331
1865	1.454	-3.2086	298.306
1866	1.4541	-3.044	226.015
1867	1.4543	-5.6325	302.502
1868	1.4549	-2.9756	271.728
1869	1.4551	-5.6671	338.848
1870	1.4551	-4.0404	1.8759
1871	1.4556	-2.9476	14.8967
1872	1.4572	-2.7123	21.2519
1873	1.4573	-2.7967	90.3344
1874	1.4579	-2.9868	89.8375
1875	1.4581	-4.327	59.5234
1876	1.4581	-3.9324	55.7238
1877	1.4583	-2.7774	40.8218
1878	1.4583	-2.8077	358.907
1879	1.4584	-2.7927	87.8231
1880	1.4584	-2.7813	166.404
1881	1.4587	-3.2024	92.7702
1882	1.4589	-5.0072	309.145
1883	1.4592	-3.5045	168.326
1884	1.4594	-3.9688	168.932
1885	1.4595	-2.8561	232.23
1886	1.4596	-3.9553	74.3723
1887	1.4596	-3.024	270.163
1888	1.4598	-2.7907	340.663
1889	1.4599	-2.9368	89.2761
1890	1.4602	-2.9636	288.313
1891	1.4604	-2.6698	319.246
1892	1.4608	-2.8621	240
1893	1.461	-2.7861	128.207
1894	1.4611	-2.8044	187.833
1895	1.4612	-3.275	304.27
1896	1.4613	-4.1028	179.537
1897	1.4615	-3.0158	4.6109
1898	1.4617	-3.5013	322.188
1899	1.462	-2.9888	118.971
1900	1.4629	-3.8604	158.645
1901	1.463	-2.8067	119.593
1902	1.4638	-2.7555	249.648
1903	1.464	-2.8501	318.203
1904	1.4641	-2.9132	332.352
1905	1.4646	-3.0865	33.3558
1906	1.4649	-2.8514	266.971
1907	1.4649	-2.9316	355.122
1908	1.4651	-2.8927	66.5912
1909	1.4651	-2.8437	34.2107

1910	1.4652	-3.2836	40.0582
1911	1.4656	-2.872	330.028
1912	1.4662	-3.1695	341.592
1913	1.4662	-3.2952	280.247
1914	1.4663	-2.9842	299.401
1915	1.4664	-3.0843	340.871
1916	1.4665	-3.3274	268.835
1917	1.4669	-3.9135	322.579
1918	1.467	-2.9614	25.8174
1919	1.4681	-3.3138	330.14
1920	1.4682	-2.9502	352.189
1921	1.4684	-2.8026	352.248
1922	1.4684	-2.782	136.545
1923	1.4685	-3.56	336.359
1924	1.4695	-2.8688	20.6668
1925	1.4696	-3.8737	343.497
1926	1.4696	-2.7779	121.241
1927	1.4697	-2.8844	165.082
1928	1.4699	-3.4567	122.955
1929	1.4701	-2.7939	294.77
1930	1.4702	-2.9697	70.1301
1931	1.4703	-3.4875	143.435
1932	1.4718	-4.6417	308.716
1933	1.4727	-4.8719	255.141
1934	1.4734	-2.7449	194.033
1935	1.4738	-3.1469	46.3895
1936	1.4738	-2.7409	349.252
1937	1.4739	-3.7571	158.824
1938	1.4741	-2.7901	0.4544
1939	1.4744	-2.8075	320.641
1940	1.4746	-2.6953	327.044
1941	1.4747	-2.957	82.3921
1942	1.4749	-2.8429	319.974
1943	1.4749	-4.5318	266.986
1944	1.4758	-2.7922	18.677
1945	1.4766	-3.8913	114.109
1946	1.4766	-2.787	70.6586
1947	1.4782	-3.4993	188.138
1948	1.4782	-3.7412	349.819
1949	1.4784	-3.9285	47.2247
1950	1.4785	-3.7192	311.475
1951	1.4787	-2.9882	43.8183
1952	1.4792	-2.9897	83.944
1953	1.4795	-2.8828	133.002
1954	1.4808	-3.8694	250.638
1955	1.4816	-3.2738	181.296
1956	1.4818	-2.8257	295.67
1957	1.4818	-2.9491	19.1976

1958	1.4819	-4.0128	128.093
1959	1.4823	-2.818	291.36
1960	1.4823	-2.8496	297.458
1961	1.4826	-3.328	98.4547
1962	1.4833	-2.9169	293.95
1963	1.4836	-4.0862	95.8356
1964	1.4836	-3.3791	50.4987
1965	1.4837	-2.8665	298.106
1966	1.4838	-2.8084	182.427
1967	1.4842	-3.3272	219.285
1968	1.4842	-2.8353	89.8785
1969	1.4848	-3.7675	316.785
1970	1.4852	-3.3034	304.087
1971	1.4853	-2.8911	59.5794
1972	1.4856	-2.8403	265.105
1973	1.4857	-4.0459	337.448
1974	1.4858	-2.7961	63.7222
1975	1.4866	-3.751	344.945
1976	1.4868	-3.8642	69.4764
1977	1.487	-3.3247	205.156
1978	1.4871	-3.8364	6.7284
1979	1.4872	-4.2834	139.978
1980	1.4872	-4.0661	274.121
1981	1.4873	-2.966	353.807
1982	1.4878	-2.8112	187.889
1983	1.488	-2.801	336.828
1984	1.4881	-2.6205	282.855
1985	1.4882	-3.1724	302.819
1986	1.4887	-2.9275	337.603
1987	1.4889	-2.7962	272.655
1988	1.4892	-2.9858	17.3035
1989	1.4892	-2.8935	301.412
1990	1.4897	-3.2737	347.849
1991	1.4902	-2.8556	183.423
1992	1.4904	-2.9862	54.244
1993	1.4904	-3.9056	16.9141
1994	1.4905	-2.9662	39.3718
1995	1.4907	-3.3859	298.05
1996	1.4909	-2.7027	217.237
1997	1.4913	5.854	111.093
1998	1.4914	-2.8089	308.452
1999	1.4921	-2.9376	314.461
2000	1.4921	-2.8291	140.275
2001	1.4924	-3.9472	276.501
2002	1.4928	-2.8581	163.269
2003	1.4928	-3.9889	355.986
2004	1.493	-4.0566	0.2341
2005	1.4935	-2.765	336.274

2006	1.4936	-4.0962	320.288
2007	1.4936	-2.9944	279.513
2008	1.4942	-2.9321	180.953
2009	1.4944	-2.7967	352.555
2010	1.4944	-2.7828	338.464
2011	1.4945	-2.7446	253.56
2012	1.495	-3.2527	338.327
2013	1.495	-2.8529	323.657
2014	1.4951	-3.9006	111.721
2015	1.4963	-3.9111	223.019
2016	1.4963	-3.7548	243.774
2017	1.4964	-4.0361	315.086
2018	1.4964	-2.8729	279.25
2019	1.4971	-3.8969	75.0291
2020	1.4976	-4.1763	142.805
2021	1.4991	-2.9309	21.2917
2022	1.4994	-2.6867	125.068
2023	1.4994	-3.1474	89.8541
2024	1.4995	-2.7447	37.4916
2025	1.4996	-2.7958	202.452
2026	1.5001	-2.8939	299.815
2027	1.5004	-2.9617	337.227
2028	1.5008	-2.7756	97.8544
2029	1.5009	-2.8984	66.1966
2030	1.5009	-2.8783	354.91
2031	1.5013	-2.7867	340.174
2032	1.5014	-2.9377	299.736
2033	1.5015	-3.1528	139.232
2034	1.5015	-3.8012	149.002
2035	1.5017	-2.8565	130.66
2036	1.5017	-3.35	171.108
2037	1.5024	-3.7782	267.954
2038	1.5028	-2.9703	32.0854
2039	1.5029	-4.5022	28.1056
2040	1.5034	-2.7995	116.459
2041	1.5034	-4.1006	173.426
2042	1.5035	-2.8953	221.905
2043	1.5035	-3.1677	107.637
2044	1.5036	-2.8709	152.232
2045	1.5043	-2.9325	245.9
2046	1.5044	-3.7265	28.9891
2047	1.5047	-2.8121	357.715
2048	1.505	-2.8013	313.14
2049	1.5054	-4.1204	323.178
2050	1.5056	-2.9332	267.425
2051	1.506	-2.9404	232.132
2052	1.5061	0.1276	326.72
2053	1.5066	-2.8814	327.163

2054	1.5067	-2.8749	319.106
2055	1.5068	-2.7452	81.6789
2056	1.5068	-3.254	313.535
2057	1.5068	-4.0662	290.681
2058	1.5069	-2.762	81.2341
2059	1.5071	-2.7203	347.249
2060	1.5071	-2.8295	152.562
2061	1.5073	-2.7887	9.9902
2062	1.5075	-2.8115	305.454
2063	1.5082	-2.9618	332.933
2064	1.5084	-4.0922	131.933
2065	1.5085	-2.8728	129.422
2066	1.5091	-6.0572	300.486
2067	1.5093	-4.1418	23.5166
2068	1.5094	-3.8984	316.938
2069	1.5095	-2.9557	66.1255
2070	1.5096	-4.2149	117.911
2071	1.5097	-2.9797	266.997
2072	1.5098	-2.8745	319.494
2073	1.51	-3.9629	136.366
2074	1.5102	-2.9748	314.534
2075	1.5107	-3.9888	91.194
2076	1.5107	-2.7724	297.879
2077	1.5107	-3.0736	295.489
2078	1.5108	-3.9106	161.296
2079	1.5112	-3.9274	329.291
2080	1.5114	-2.9222	316.293
2081	1.5122	-3.1663	172.753
2082	1.5123	-3.2223	279.45
2083	1.5129	-2.7993	337.371
2084	1.5134	-2.8166	352.991
2085	1.5137	-3.7255	70.578
2086	1.5137	-2.9204	248.607
2087	1.5138	-2.9117	20.0584
2088	1.5138	-3.4906	351.51
2089	1.5138	-2.8423	170.051
2090	1.5142	-2.7762	313.921
2091	1.5148	-2.7594	179.896
2092	1.515	-4.1261	131.977
2093	1.5151	-2.9775	258.64
2094	1.5153	-3.3858	40.7157
2095	1.5155	-2.9346	160.158
2096	1.5161	-3.9945	303.674
2097	1.5162	-2.7702	351.397
2098	1.5165	-2.8292	310.334
2099	1.5175	-3.3333	18.0638
2100	1.5175	-3.1393	68.5595
2101	1.5177	-2.9088	317.715

2102	1.5188	-2.785	66.7851
2103	1.519	-3.2741	72.0942
2104	1.5195	-2.9552	52.6914
2105	1.5202	-2.9238	89.7975
2106	1.5203	-2.94	276.809
2107	1.5203	-3.3355	318.435
2108	1.5204	-2.8782	178.364
2109	1.5206	-2.825	170.021
2110	1.5221	-2.8603	7.8429
2111	1.5224	-3.5781	137.971
2112	1.5225	-3.1403	330.491
2113	1.5226	-2.7857	307.105
2114	1.5227	-2.8295	207.246
2115	1.5245	-3.2868	356.248
2116	1.5247	-2.9464	67.8441
2117	1.5256	-2.9261	104.118
2118	1.5258	-2.8636	188.552
2119	1.5259	-2.8294	247.722
2120	1.5264	-2.7884	273.927
2121	1.5266	-2.973	25.2329
2122	1.5274	-2.9513	321.356
2123	1.5275	-2.9442	319.393
2124	1.5275	-1.7744	176.107
2125	1.5276	-2.7711	221.75
2126	1.528	-2.9075	27.3503
2127	1.528	-2.8629	313.821
2128	1.528	-2.8746	69.158
2129	1.5283	-3.9304	26.1764
2130	1.5289	-2.8377	226.887
2131	1.5292	-2.8955	339.849
2132	1.5293	-4.162	283.643
2133	1.5293	-4.1148	211.929
2134	1.5294	-2.7955	220.193
2135	1.5297	-2.8333	272.248
2136	1.5302	-3.1864	355.285
2137	1.5303	-3.0842	29.2722
2138	1.5307	-2.8209	82.3441
2139	1.5312	-4.346	282.196
2140	1.5313	-3.4479	108.212
2141	1.5316	-3.6987	75.6786
2142	1.5316	-3.5821	128.945
2143	1.5318	-2.8105	8.5191
2144	1.532	-2.8958	283.332
2145	1.5333	-4.2496	237.064
2146	1.5338	-2.9912	284.459
2147	1.5341	-2.7672	60.3216
2148	1.5342	-3.121	311.418
2149	1.5345	-2.6814	122.379

2150	1.5349	-2.943	179.972
2151	1.535	-3.0655	266.329
2152	1.5352	-2.8734	250.568
2153	1.5355	-2.9418	270.229
2154	1.5359	-2.9706	315.075
2155	1.5362	-3.2802	159.442
2156	1.5364	-2.7879	149.081
2157	1.5366	-3.9122	131.99
2158	1.5367	-3.9114	143.591
2159	1.5386	-3.7927	286.245
2160	1.5387	-3.8765	29.4887
2161	1.5389	-3.829	280.065
2162	1.5389	-2.8309	231.609
2163	1.5391	-2.7666	169.357
2164	1.5391	-3.232	332.771
2165	1.5396	-4.0386	308.489
2166	1.54	-3.4775	29.6656
2167	1.54	-2.7291	351.69
2168	1.5402	-2.8516	242.284
2169	1.5406	-2.6715	223.405
2170	1.5408	-2.8441	99.929
2171	1.5409	-3.3694	235.007
2172	1.5411	-2.9121	88.595
2173	1.5412	-4.063	74.3576
2174	1.5414	-2.9664	26.7319
2175	1.5415	-2.8291	108.576
2176	1.5415	-2.8575	233.913
2177	1.5419	-3.4154	42.5701
2178	1.5422	-3.2715	149.413
2179	1.5423	-2.9707	324.786
2180	1.5427	-2.8916	350.808
2181	1.5428	-3.9342	355.558
2182	1.5429	-2.9439	260.34
2183	1.543	-2.7358	78.002
2184	1.5439	-4.2465	6.7104
2185	1.5439	-2.9825	333.471
2186	1.5441	-2.9182	140.035
2187	1.5444	-3.5664	23.0435
2188	1.5445	-2.7359	69.794
2189	1.5445	-3.3495	91.0101
2190	1.5447	-3.719	348.092
2191	1.5448	-3.3654	315.735
2192	1.545	-2.7947	20.9298
2193	1.545	-3.8473	328.26
2194	1.5451	-2.8156	349.237
2195	1.5451	-3.983	87.2762
2196	1.5452	-2.9609	23.7164
2197	1.5453	-2.9459	304.152

2198	1.5453	-3.9958	23.1086
2199	1.5455	-2.6635	89.1003
2200	1.5456	-4.1538	344.495
2201	1.5457	-2.7015	129.835
2202	1.5458	-2.9063	136.274
2203	1.5464	-2.8115	145.881
2204	1.5466	-3.2515	336.809
2205	1.5467	-2.7516	325.944
2206	1.5471	-2.7088	158.775
2207	1.5475	-4.1258	80.203
2208	1.548	-3.4867	160.829
2209	1.548	-4.0328	163.129
2210	1.5484	-2.8663	57.2785
2211	1.5485	-2.6755	26.1504
2212	1.5488	-3.2726	25.9291
2213	1.5492	-2.8882	159.176
2214	1.5496	-4.7062	305.008
2215	1.5499	-2.9034	256.725
2216	1.55	-2.7972	348.859
2217	1.5502	-3.7328	333.394
2218	1.5502	-4.1485	17.9465
2219	1.5503	-2.9035	279.245
2220	1.5505	-3.2347	195.666
2221	1.5511	-4.4659	46.405
2222	1.5513	-2.9005	123.462
2223	1.5515	-2.8511	289.121
2224	1.5516	-2.8641	310.647
2225	1.5519	-3.0601	290.777
2226	1.5522	-3.3695	271.145
2227	1.5522	-3.0734	57.7097
2228	1.5527	-3.2427	275.281
2229	1.5532	-2.9923	122.172
2230	1.5542	-2.7847	356.014
2231	1.5547	-3.9666	8.7648
2232	1.5549	-2.8329	203.143
2233	1.5553	-3.1358	277.166
2234	1.5556	-2.7767	165.129
2235	1.5559	-2.9842	116.913
2236	1.5562	-3.2431	131.651
2237	1.5563	-2.8121	120.275
2238	1.5565	-2.8795	163.143
2239	1.5565	-2.9758	326.759
2240	1.5573	-2.8162	151.285
2241	1.5574	-2.8983	324.511
2242	1.5575	-3.261	105.135
2243	1.5579	-3.6829	1.8612
2244	1.5583	-2.8781	283.251
2245	1.5586	-4.0447	301.189

2246	1.5589	-2.7067	172.461
2247	1.5592	-2.8009	229.417
2248	1.56	-2.9108	324.336
2249	1.5602	-4.4116	126.066
2250	1.5602	-2.8182	49.4577
2251	1.5609	-2.5394	156.068
2252	1.5612	-3.9297	205.522
2253	1.5612	-2.7083	146.979
2254	1.5618	-2.6822	88.8078
2255	1.5619	-2.8333	109.373
2256	1.5623	-3.3362	201.642
2257	1.5624	-2.81	205.428
2258	1.5625	-2.9591	325.907
2259	1.5626	-2.8365	344.375
2260	1.5629	-3.2156	331.405
2261	1.5631	-4.0992	290.206
2262	1.5633	-2.8475	124.001
2263	1.5633	-3.8972	118.302
2264	1.5635	-3.0832	325.729
2265	1.5636	-3.0012	295.371
2266	1.5637	-3.5612	29.7183
2267	1.5638	-3.1292	93.5084
2268	1.5638	-2.803	10.6242
2269	1.5641	-3.8705	337.583
2270	1.5643	-3.7216	79.6141
2271	1.5645	-3.2397	306.163
2272	1.5646	-4.6353	37.431
2273	1.5646	-3.8653	152.183
2274	1.5649	-3.827	225.026
2275	1.5651	-2.7878	85.2872
2276	1.5653	-2.907	274.722
2277	1.5654	-2.8824	27.2872
2278	1.5655	-2.9441	315.277
2279	1.5657	-2.9001	11.4022
2280	1.5658	-2.8229	276.187
2281	1.5661	-4.1258	268.58
2282	1.5663	-3.459	20.5746
2283	1.5666	-2.8633	347.799
2284	1.5666	-2.8622	31.7913
2285	1.5669	-2.9643	269.532
2286	1.5671	-2.9872	17.9478
2287	1.5675	-2.7947	348.408
2288	1.5678	-3.6884	91.5083
2289	1.5681	-2.7305	126.841
2290	1.5682	-2.8103	317.805
2291	1.5684	-2.6572	325.03
2292	1.5684	-2.8572	140.332
2293	1.5685	-4.4156	291.177

2294	1.5687	-3.0406	167.517
2295	1.5688	-4.0024	338.327
2296	1.5689	-3.0947	100.34
2297	1.569	-3.5301	73.3077
2298	1.5692	-2.7735	173.555
2299	1.57	-2.8852	107.625
2300	1.5702	-3.9365	319.683
2301	1.5702	-3.5664	331.115
2302	1.5702	-3.474	52.117
2303	1.5708	-2.9307	77.6635
2304	1.5711	-2.9065	317.815
2305	1.5711	-2.973	36.4408
2306	1.5711	-2.8019	112.504
2307	1.5713	-2.8229	304.612
2308	1.5714	-3.3293	336.163
2309	1.5716	-2.8039	256.654
2310	1.572	-2.936	344.579
2311	1.5725	-3.7997	31.3807
2312	1.5725	-3.4559	115.589
2313	1.5726	-3.3557	285.02
2314	1.5727	-3.8088	124.375
2315	1.5727	-3.1488	35.5233
2316	1.5727	-3.9567	13.5428
2317	1.5727	-2.9415	132.652
2318	1.5728	-2.702	352.41
2319	1.5734	-3.9331	327.343
2320	1.5741	-4.1048	81.1624
2321	1.5743	-4.3124	110.885
2322	1.5744	-2.8176	157.346
2323	1.5745	-3.1025	3.1594
2324	1.5747	-2.6616	345.193
2325	1.5748	-2.9223	27.9073
2326	1.5748	-3.4494	216.77
2327	1.5749	-3.4612	327.986
2328	1.5755	-3.7241	280.347
2329	1.5756	-3.11	41.9353
2330	1.5758	-3.729	21.8399
2331	1.5761	-3.4509	271.003
2332	1.5762	-2.7542	183.289
2333	1.5765	-2.7605	242.686
2334	1.5766	-2.9915	79.049
2335	1.5767	-2.9525	295.589
2336	1.5769	-2.7696	321.16
2337	1.5771	-2.8638	78.2739
2338	1.5784	-2.7442	354.761
2339	1.5785	-3.0547	264.569
2340	1.5787	-2.985	139.03
2341	1.5788	-2.8288	346.925

2342	1.5788	-3.6766	234.192
2343	1.5789	-2.9442	136.998
2344	1.5794	-3.3756	353.941
2345	1.5796	1.9698	148.588
2346	1.5797	-3.9296	315.455
2347	1.5798	-3.9041	331.84
2348	1.5798	-2.8066	285.638
2349	1.5799	-3.299	78.9551
2350	1.5802	-3.4163	171.816
2351	1.5804	-2.9497	302.711
2352	1.5805	-3.3827	284.174
2353	1.5806	-2.7136	337.838
2354	1.5807	-3.0518	25.9563
2355	1.581	-2.9023	172.447
2356	1.5817	-2.8315	225.272
2357	1.5821	-2.8182	30.3092
2358	1.5823	-2.7924	321.147
2359	1.5828	-3.8756	91.4448
2360	1.5835	-4.0852	200.095
2361	1.5838	-3.8902	229.548
2362	1.5839	-3.0248	20.0318
2363	1.5844	-2.9579	320.196
2364	1.5849	-4.1102	321.784
2365	1.585	-3.4088	59.983
2366	1.5852	-3.2449	321.507
2367	1.5856	-2.7736	66.8583
2368	1.5856	-2.9699	11.3351
2369	1.5858	-2.913	252.568
2370	1.5859	-3.2615	173.602
2371	1.586	-2.9308	172.318
2372	1.5865	-3.2494	152.066
2373	1.5866	-3.1393	248.79
2374	1.5869	-3.1871	185.647
2375	1.5872	-2.7578	25.3136
2376	1.5872	-4.186	356.632
2377	1.5875	-3.9902	83.1381
2378	1.5877	-4.0189	61.2997
2379	1.5883	-2.8991	128.411
2380	1.5885	-2.8774	272.258
2381	1.5887	-4.1688	321.245
2382	1.5887	-3.2254	205.81
2383	1.5894	-3.2212	90.0912
2384	1.5896	-2.7905	8.8543
2385	1.5897	-2.9679	142.686
2386	1.5898	-2.8636	351.847
2387	1.59	-2.7976	284.601
2388	1.5914	-2.8296	82.2405
2389	1.5917	-4.2662	23.5313

2390	1.592	-3.2624	121.122
2391	1.5922	-2.9524	176.528
2392	1.5923	-3.1767	359.145
2393	1.5923	-2.8538	21.2734
2394	1.5927	-2.3617	233.232
2395	1.5936	-2.9943	171.887
2396	1.5939	-2.8221	104.62
2397	1.594	-3.6304	111.539
2398	1.5941	-3.209	19.3552
2399	1.5943	-3.8174	7.5242
2400	1.5944	-3.4977	335.067
2401	1.5947	-3.8794	7.0481
2402	1.5947	-3.3918	299.565
2403	1.5947	-2.8159	268.769
2404	1.5949	-4.6694	229.219
2405	1.5952	-2.8684	174.758
2406	1.5952	-0.9535	280.293
2407	1.5952	-3.1832	290.624
2408	1.5955	-2.8073	341.287
2409	1.5955	-2.8257	307.738
2410	1.5955	-2.7706	353.594
2411	1.5956	-4.3042	103.103
2412	1.5957	-3.7416	331.806
2413	1.5959	-3.0421	102.832
2414	1.5963	-2.8278	81.3877
2415	1.5963	-2.8157	136.642
2416	1.5966	-2.9204	163.266
2417	1.5968	-2.7815	347.357
2418	1.597	-4.2268	194.742
2419	1.5975	-3.6154	68.9762
2420	1.5978	-2.9344	268.296
2421	1.5979	-2.821	319.154
2422	1.5981	-2.9919	319.949
2423	1.5981	-2.8647	150.244
2424	1.5987	-3.0098	294.723
2425	1.599	-2.8708	7.9155
2426	1.5995	-2.9191	38.4326
2427	1.5998	-3.3543	320.903
2428	1.6001	-2.6959	30.2151
2429	1.6001	-2.7669	236.971
2430	1.6003	-2.9243	270.377
2431	1.6009	-4.0798	225.854
2432	1.6014	-2.8896	333.781
2433	1.6025	-2.8363	71.8625
2434	1.6027	-2.8219	21.9235
2435	1.6028	-3.3891	271.626
2436	1.6034	-2.8696	132.287
2437	1.6035	-2.9552	127.306

2438	1.6037	-1.9871	213.608
2439	1.6037	-2.8608	145.541
2440	1.604	-2.9785	44.2887
2441	1.604	-0.6656	179.926
2442	1.6041	-2.8611	279.661
2443	1.6049	-4.1467	120.518
2444	1.605	-2.9386	292.577
2445	1.6052	-2.9632	308.022
2446	1.6052	-4.0346	327.868
2447	1.6054	-3.1423	77.8324
2448	1.6058	-2.8707	263.511
2449	1.6061	-1.7465	311.941
2450	1.6061	-3.7273	344.082
2451	1.6063	-1.2797	134.61
2452	1.6064	-2.9639	261.994
2453	1.6067	-2.9915	28.2014
2454	1.6071	-2.9094	256.176
2455	1.6074	-2.8551	336.279
2456	1.6076	-2.9922	89.9224
2457	1.6078	-3.8843	305.796
2458	1.608	-2.8803	256.755
2459	1.6081	-2.9476	308.496
2460	1.6087	-3.6511	86.741
2461	1.6087	-2.8291	347.444
2462	1.6087	-2.7639	3.9214
2463	1.609	-2.9091	276.245
2464	1.609	-3.5637	107.523
2465	1.6091	-2.8176	234.211
2466	1.6093	-2.7216	40.0738
2467	1.6095	-2.868	142.123
2468	1.6099	-4.081	126.787
2469	1.61	-3.0536	275.407
2470	1.6103	-2.7902	35.7611
2471	1.6106	-2.8439	17.3368
2472	1.6107	-2.9157	282.861
2473	1.6108	-2.8948	335.123
2474	1.6111	-4.1024	25.8937
2475	1.6116	-2.7739	26.3106
2476	1.6118	-4.0651	221.397
2477	1.6118	-2.8701	306.342
2478	1.6121	-4.2975	356.62
2479	1.6121	-4.0536	30.463
2480	1.6122	-3.5152	164.409
2481	1.6123	-3.8653	348.118
2482	1.6128	-2.8157	238.472
2483	1.613	-4.0487	146.772
2484	1.613	-3.5572	280.205
2485	1.613	-1.5784	355.069

2486	1.613	-2.8539	65.2835
2487	1.6133	-3.9086	91.5159
2488	1.6134	-3.1703	326.2
2489	1.6136	-2.6532	238.902
2490	1.6137	-2.7789	335.825
2491	1.6137	-3.1754	133.529
2492	1.6148	1.104	93.2061
2493	1.6154	-3.3829	32.8966
2494	1.6156	-2.9521	76.415
2495	1.6157	-4.162	53.7879
2496	1.616	-2.8262	91.8094
2497	1.616	3.1977	204.075
2498	1.616	-4.3665	127.767
2499	1.6163	-4.0271	302.055
2500	1.6166	-2.8312	322.173
2501	1.6166	-3.9291	59.7289
2502	1.6172	-3.8894	225.648
2503	1.6173	-3.5209	62.3094
2504	1.6185	-3.5472	333.899
2505	1.6186	-2.803	188.413
2506	1.6187	-4.0324	315.482
2507	1.619	-3.2374	315.998
2508	1.6195	-2.9735	260.448
2509	1.6198	-2.8353	157.998
2510	1.62	-2.7608	194.986
2511	1.6201	-2.9372	276.453
2512	1.6207	-2.7753	268.642
2513	1.6207	-2.8464	290.153
2514	1.6208	-3.0122	119.274
2515	1.6209	-2.7772	212.118
2516	1.6213	-2.9725	20.6631
2517	1.6218	-2.749	176.624
2518	1.6224	-2.6986	322.45
2519	1.6228	-4.0019	223.765
2520	1.6228	-2.7723	330.19
2521	1.6229	-3.0769	63.921
2522	1.6238	-2.7439	308.891
2523	1.6238	-3.1841	329.899
2524	1.6239	-3.9929	6.4525
2525	1.624	-4.0611	131.98
2526	1.6249	-2.6672	332.382
2527	1.625	0.3676	49.1695
2528	1.625	-2.8378	221.466
2529	1.6253	-2.9009	69.8301
2530	1.6253	-2.8803	349.99
2531	1.6259	-3.5077	45.0556
2532	1.626	-3.3305	74.4836
2533	1.626	-3.8825	320.194

2534	1.6264	-5.7467	187.561
2535	1.6269	-2.6951	312.153
2536	1.627	-3.6408	98.6756
2537	1.627	-2.6599	337.314
2538	1.6271	-2.7972	37.5595
2539	1.6271	-2.802	108.76
2540	1.6274	-3.9421	167.123
2541	1.6277	-3.9156	10.7642
2542	1.6277	-2.9158	303.841
2543	1.6278	-3.3233	152.348
2544	1.628	-2.9885	344.273
2545	1.6282	-3.6485	12.244
2546	1.6284	-2.7746	132.635
2547	1.6288	-3.4547	148.436
2548	1.6295	-3.3545	234.189
2549	1.6301	-2.7183	44.3743
2550	1.6302	-2.8628	49.3068
2551	1.6303	-2.638	276.428
2552	1.6303	-2.8252	321.531
2553	1.6304	-2.9525	337.135
2554	1.6304	-3.0101	240.206
2555	1.6306	-3.9929	150.395
2556	1.6307	-2.9468	354.536
2557	1.6312	-3.1749	316.62
2558	1.6313	-3.752	52.0568
2559	1.6313	-2.8074	61.3318
2560	1.6314	-3.8644	347.023
2561	1.6325	-3.8921	118.621
2562	1.6327	-2.7779	66.5384
2563	1.6327	-3.8093	319.182
2564	1.6327	-2.8839	74.6151
2565	1.6329	-2.6941	335.991
2566	1.633	-3.7594	264.262
2567	1.6334	-2.7853	234.458
2568	1.6338	-2.9009	297.092
2569	1.6339	-3.9125	125.701
2570	1.6342	-2.5312	192.397
2571	1.6342	-2.8859	280.649
2572	1.6343	-2.8222	338.57
2573	1.6344	-3.0163	314.54
2574	1.6344	-3.4746	70.6756
2575	1.6345	-2.9474	29.9672
2576	1.6352	-2.8012	77.2525
2577	1.6356	-2.9948	318.561
2578	1.6357	-3.7789	98.3872
2579	1.6357	-2.8712	266.954
2580	1.6358	-3.1271	332.972
2581	1.636	-3.5022	279.129

2582	1.6361	-2.9429	290.692
2583	1.6365	-3.1934	330.451
2584	1.6366	-2.9895	12.1477
2585	1.6371	-3.7402	294.699
2586	1.6371	0.6534	180.638
2587	1.6374	-3.127	338.661
2588	1.6376	-3.4455	274.754
2589	1.6376	-2.8972	308.288
2590	1.638	-2.9494	114.635
2591	1.638	-2.9118	272.582
2592	1.6384	-2.9327	301.207
2593	1.6386	-2.9039	59.925
2594	1.6388	-2.9216	21.3902
2595	1.6391	-2.8474	50.9662
2596	1.6395	-4.5725	326.762
2597	1.6396	-4.1503	285.894
2598	1.6399	-3.3928	184.527
2599	1.6403	-2.9235	100.353
2600	1.6408	-2.8132	72.0195
2601	1.6415	-2.7773	82.7654
2602	1.6418	-2.7662	52.22
2603	1.6419	-2.7817	215.224
2604	1.6422	-1.9143	339.631
2605	1.6426	-3.0858	352.479
2606	1.6434	-2.7926	64.4159
2607	1.6438	-2.0382	257.935
2608	1.6438	-2.7687	127.809
2609	1.6438	-2.9997	149.977
2610	1.6439	-3.2482	282.668
2611	1.6441	-2.6094	273.691
2612	1.6441	-3.4167	330.876
2613	1.6444	-3.3622	309.155
2614	1.6452	-3.8747	78.08
2615	1.6458	-2.7849	347.138
2616	1.6461	-3.7474	344.504
2617	1.6462	-2.8525	276.142
2618	1.6464	-3.7927	272.185
2619	1.6468	-2.7884	349.166
2620	1.647	-4.0558	172.813
2621	1.6476	-3.9333	92.9158
2622	1.6478	-3.9653	190.584
2623	1.6479	-3.8964	128.672
2624	1.6479	-3.5049	246.092
2625	1.648	-3.3205	301.39
2626	1.648	-2.9169	0.0444
2627	1.6483	-3.035	353.251
2628	1.6487	-4.4191	307.424
2629	1.6488	-4.0464	347.384

2630	1.6488	-3.9372	104.608
2631	1.6489	-3.9487	188.6
2632	1.6492	-2.9837	210.972
2633	1.6493	-2.83	129.771
2634	1.6494	-2.9235	72.8279
2635	1.6495	-3.2665	293.135
2636	1.6502	-3.3932	318.298
2637	1.6511	-2.8412	126.768
2638	1.6514	-2.7951	342.682
2639	1.6519	-4.0034	31.9833
2640	1.6519	-2.8228	129.851
2641	1.6522	-3.9078	20.0137
2642	1.6522	-2.7923	359.957
2643	1.6524	-2.7862	172.627
2644	1.6528	-4.2639	236.608
2645	1.6534	-3.2439	243.593
2646	1.6535	-2.8338	90.2722
2647	1.6536	-2.8503	298.701
2648	1.6537	-3.0904	158.816
2649	1.654	-4.005	80.4271
2650	1.6544	-3.2955	143.506
2651	1.6546	-3.2213	81.4943
2652	1.6547	-2.3305	86.3184
2653	1.6547	-2.8678	18.2478
2654	1.6547	-2.7767	323.141
2655	1.6554	-3.3456	354.315
2656	1.656	-2.943	195.954
2657	1.656	-2.7855	275.941
2658	1.6567	-2.8635	119.668
2659	1.6569	-2.7525	136.552
2660	1.6573	-4.2356	359.167
2661	1.6574	-3.6275	322.123
2662	1.6576	-3.1607	304.712
2663	1.6576	-2.9859	57.3868
2664	1.6579	-2.9116	239.302
2665	1.6582	-4.0801	278.051
2666	1.6592	-2.8675	182.036
2667	1.6595	-4.0825	45.5967
2668	1.6596	-3.6502	172.661
2669	1.6598	-1.9682	136.402
2670	1.6599	-2.8297	24.4045
2671	1.66	-3.6444	280.831
2672	1.6602	-2.7317	168.923
2673	1.6602	-3.0473	226.138
2674	1.6605	-2.7843	284.494
2675	1.6606	-2.8491	195.84
2676	1.6606	-3.9636	298.432
2677	1.6606	-3.0142	270.949

2678	1.6614	-2.8252	285.486
2679	1.6615	-3.823	322.352
2680	1.6615	-2.9131	337.008
2681	1.6617	-2.9915	140.143
2682	1.6619	-3.0191	181.078
2683	1.6622	-2.9803	169.522
2684	1.6623	-2.953	157.062
2685	1.6625	-3.5338	220.887
2686	1.6626	-2.85	325.361
2687	1.6628	-4.4066	29.9757
2688	1.6631	-4.317	276.114
2689	1.6632	-3.7085	126.583
2690	1.6634	-3.123	285.756
2691	1.6634	-4.3554	250.195
2692	1.6635	-3.8628	28.2101
2693	1.6643	-2.8167	244.598
2694	1.6644	-2.7733	64.6063
2695	1.6654	-3.39	75.6992
2696	1.6654	-4.2554	240.218
2697	1.6655	-3.2071	13.1776
2698	1.666	-3.0949	340.812
2699	1.6662	-3.9455	46.9635
2700	1.6664	-2.9697	159.565
2701	1.6664	-2.8697	224.959
2702	1.6672	-3.0894	83.1896
2703	1.6673	-2.724	136.547
2704	1.6674	-2.7685	21.6056
2705	1.668	-3.5049	300.619
2706	1.6689	-2.7128	67.7446
2707	1.669	-3.8831	52.7576
2708	1.6695	-4.4816	128.593
2709	1.6696	-3.5934	51.5319
2710	1.6699	-3.3646	280.229
2711	1.67	-3.3676	194.357
2712	1.6703	-3.9623	323.039
2713	1.6705	-4.1573	289.538
2714	1.6708	-3.2457	1.7242
2715	1.6711	-3.2386	162.45
2716	1.6713	-4.1701	354.857
2717	1.6714	-2.7111	169.004
2718	1.6722	-2.9096	200.926
2719	1.6723	-2.8968	332.209
2720	1.6728	-2.9674	125.44
2721	1.6729	-3.2961	68.6709
2722	1.6731	-2.9547	116.139
2723	1.6732	-2.7468	207.513
2724	1.6733	-3.1847	296.412
2725	1.6736	-2.789	232.214

2726	1.6743	-3.8333	354.1
2727	1.6745	-3.2516	178.236
2728	1.6746	-2.7184	44.3983
2729	1.6749	-2.8858	123.314
2730	1.675	-4.0411	278.231
2731	1.6751	-3.4858	312.192
2732	1.6753	-2.7406	102.278
2733	1.6755	-2.856	330.591
2734	1.6759	-2.954	136.291
2735	1.6761	-3.9998	202.725
2736	1.6762	-2.9337	65.5265
2737	1.6762	-2.9265	314.661
2738	1.6764	-2.7987	282.675
2739	1.6764	-3.2302	46.6513
2740	1.6765	-3.3249	286.486
2741	1.6773	-3.6749	322.934
2742	1.6773	-4.0791	123.45
2743	1.6779	-0.5305	42.501
2744	1.6783	-2.8781	292.265
2745	1.6787	-4.2336	315.666
2746	1.6793	-3.2695	233.135
2747	1.6797	-3.7057	14.1763
2748	1.6798	-3.1504	104.512
2749	1.6799	-3.0221	149.203
2750	1.6801	-3.7827	157.369
2751	1.6802	-3.3444	261.413
2752	1.6803	-3.3851	135.589
2753	1.6805	-2.9461	323.029
2754	1.6806	-2.8077	343.038
2755	1.6812	-2.9361	157.148
2756	1.6812	-2.8192	228.503
2757	1.6815	-0.8676	104.5
2758	1.6818	-4.5029	261.122
2759	1.682	-2.8105	293.757
2760	1.6822	-2.7946	223.961
2761	1.6822	-3.8846	164.618
2762	1.6824	-2.843	171.062
2763	1.6824	-2.7962	0.3866
2764	1.6825	-2.9457	63.3816
2765	1.6827	-2.7391	15.2647
2766	1.6827	0.6296	85.7856
2767	1.6831	-3.2792	304.432
2768	1.6832	-2.9094	344.2
2769	1.6832	-2.7669	301.375
2770	1.6834	-3.3182	344.132
2771	1.6836	-2.9027	351.129
2772	1.6846	-4.0267	282.027
2773	1.6847	-3.2698	162.29

2774	1.6849	-3.3914	64.8767
2775	1.6853	-3.6816	260.962
2776	1.6854	-2.7864	288.078
2777	1.6856	-3.0909	85.466
2778	1.6864	-3.1061	26.8486
2779	1.6864	-2.6417	149.007
2780	1.687	-3.2167	135.472
2781	1.6873	-3.7453	182.352
2782	1.6874	-3.6355	160.787
2783	1.6875	-3.0363	6.0871
2784	1.6879	3.1999	156.445
2785	1.6881	-2.9643	274.058
2786	1.6882	-2.892	74.9821
2787	1.6883	-2.829	341.097
2788	1.6883	-3.5307	127.059
2789	1.6884	-2.9176	312.359
2790	1.6888	-2.9206	209.148
2791	1.6889	-2.7595	69.8472
2792	1.6889	-3.9113	12.6235
2793	1.6897	-4.0408	321.347
2794	1.6899	-3.4511	149.695
2795	1.6901	-2.6672	296.551
2796	1.6903	-3.9427	351.23
2797	1.6904	-4.194	184.468
2798	1.6906	-3.8891	130.764
2799	1.6907	-3.0365	290.398
2800	1.6913	-2.8594	244.799
2801	1.6917	0.2782	242.151
2802	1.6917	-2.7913	4.4653
2803	1.6922	-3.1557	229.178
2804	1.6922	-2.8521	335.373
2805	1.6923	-2.8594	238.182
2806	1.6923	-2.8651	284.674
2807	1.6924	-2.7814	168.323
2808	1.6924	-2.9274	253.032
2809	1.6925	-2.6958	252.163
2810	1.6927	-2.7589	318.495
2811	1.6927	-2.7784	171.464
2812	1.693	-2.8147	61.623
2813	1.6933	-2.9586	83.6932
2814	1.6938	-4.3474	61.4196
2815	1.6939	-2.9025	209.814
2816	1.6943	-3.553	134.298
2817	1.6944	-3.5391	287.313
2818	1.6952	-2.8131	354.15
2819	1.696	-2.9763	163.709
2820	1.6961	-2.9081	175.66
2821	1.6964	-3.9528	350.673

2822	1.6966	-3.7574	117.441
2823	1.6968	-3.868	267.891
2824	1.6971	-2.8515	113.55
2825	1.6972	-2.911	157.57
2826	1.6974	-3.0476	128.884
2827	1.6974	-3.5579	328.156
2828	1.6981	-2.8764	279.698
2829	1.6984	-2.7364	327.094
2830	1.6986	-2.7994	251.3
2831	1.6986	-3.8719	326.972
2832	1.6989	-3.8653	352.835
2833	1.6989	-4.0934	279.436
2834	1.6992	-2.9495	26.9414
2835	1.7004	-2.9213	176.466
2836	1.7017	-2.9037	348.125
2837	1.7018	-2.9697	220.697
2838	1.7025	-2.8023	73.8896
2839	1.7026	-3.1069	34.1987
2840	1.7026	-3.1638	176.703
2841	1.7029	-3.9039	212.824
2842	1.7034	-2.7611	266.207
2843	1.7034	-3.9665	125.638
2844	1.7034	-3.5619	59.9749
2845	1.7036	-2.8396	86.2835
2846	1.7038	-2.9616	87.9913
2847	1.7043	-3.262	6.3474
2848	1.7044	-2.9749	29.2868
2849	1.7045	-3.746	104.084
2850	1.7046	-3.5846	62.138
2851	1.7047	-3.6592	16.624
2852	1.7052	-3.9281	345
2853	1.7055	0.0569	79.1715
2854	1.7055	-3.1563	305.893
2855	1.7055	-4.2065	241.417
2856	1.7056	0.4772	215.357
2857	1.7056	-3.8364	237.624
2858	1.7058	-2.8479	141.18
2859	1.7059	-4.201	307.673
2860	1.7061	-3.3475	265.346
2861	1.7062	-2.7659	295.77
2862	1.7065	-3.6072	69.9195
2863	1.7071	-2.9442	279.06
2864	1.7072	-2.9233	32.1458
2865	1.7072	-2.7998	248.563
2866	1.7073	-2.8532	296.788
2867	1.7077	-3.339	37.5774
2868	1.7077	-2.9084	241.226
2869	1.708	-2.9327	116.528

2870	1.708	-3.0308	164.606
2871	1.7083	-2.786	171.022
2872	1.7089	-3.322	12.8958
2873	1.7091	-2.8792	185.955
2874	1.7092	-3.9921	14.7402
2875	1.7096	-2.7419	303.975
2876	1.7098	-2.9439	302.145
2877	1.7101	-2.7867	269.044
2878	1.7102	-2.9149	249.385
2879	1.7102	-3.8232	132.084
2880	1.7103	-4.1246	208.497
2881	1.7103	-2.8062	133.373
2882	1.7105	-2.8015	192.311
2883	1.7105	-2.7984	21.9433
2884	1.7106	-2.9125	286.393
2885	1.7114	-2.9775	208.396
2886	1.7119	-3.5354	157.992
2887	1.7122	-2.8143	338.638
2888	1.7124	-3.9729	357.729
2889	1.7128	-2.9711	268.16
2890	1.7132	-2.7815	316.359
2891	1.7132	2.3265	196.192
2892	1.7134	-2.7723	330.742
2893	1.7135	-4.4244	274.211
2894	1.7136	-2.9559	220.048
2895	1.7137	-3.1944	316.252
2896	1.7143	-2.9975	63.0828
2897	1.7151	-0.3866	184.276
2898	1.7151	-2.8346	124.064
2899	1.7153	-3.0049	8.3792
2900	1.7156	-3.1949	272.391
2901	1.7157	-3.5047	51.1309
2902	1.7157	-3.96	118.653
2903	1.7162	-3.1006	226.649
2904	1.7163	-3.1473	188.176
2905	1.7164	-3.2979	350.425
2906	1.7165	-3.9252	287.912
2907	1.7165	-3.0503	273.791
2908	1.7168	-2.94	90.8611
2909	1.7171	-2.703	137.73
2910	1.7175	-2.8476	53.6712
2911	1.7188	-3.2347	31.651
2912	1.7193	-4.0729	319.401
2913	1.7195	-3.3305	160.847
2914	1.7196	-2.0071	293.866
2915	1.7196	-2.9437	63.7134
2916	1.7199	-2.8289	100.988
2917	1.7202	-3.2731	95.4141

2918	1.7207	-3.1934	22.4962
2919	1.7211	-3.3537	334.33
2920	1.7212	-3.1697	277.634
2921	1.7213	-3.5349	123.838
2922	1.7215	-2.388	118.707
2923	1.7216	-2.7761	71.1565
2924	1.7219	-2.9143	255.072
2925	1.7221	-2.8649	317.647
2926	1.7226	-3.6435	244.176
2927	1.7228	-2.8778	260.348
2928	1.7229	-2.7348	72.3134
2929	1.7231	-3.4745	206.703
2930	1.7233	-3.194	206.423
2931	1.7237	-2.8533	0.2656
2932	1.7238	-2.9051	45.5278
2933	1.7238	-4.456	13.0831
2934	1.7243	-3.7273	313.589
2935	1.7246	-4.1321	151.838
2936	1.7247	-3.5721	170.948
2937	1.7247	-4.0493	57.6266
2938	1.7247	-2.8507	103.642
2939	1.7248	-1.2088	292.166
2940	1.7248	-2.1539	314.249
2941	1.726	-3.4114	114.876
2942	1.7263	-3.9657	53.8237
2943	1.7265	-2.7826	269.017
2944	1.7271	-1.4982	299.753
2945	1.7272	-2.7996	349.269
2946	1.7281	15.0397	78.5281
2947	1.7284	-4.155	110.38
2948	1.7286	-2.8237	188.855
2949	1.729	-4.023	302.865
2950	1.7295	-3.042	309.969
2951	1.7296	-1.3625	125.157
2952	1.7298	-2.8295	25.405
2953	1.7299	-3.9945	0.0851
2954	1.7299	-2.8024	327.469
2955	1.73	-2.9855	307.484
2956	1.7303	-1.472	295.456
2957	1.7305	-3.6079	161.722
2958	1.7311	-1.1146	190.15
2959	1.7313	-4.0081	206.03
2960	1.7313	-2.8468	20.9145
2961	1.7317	-2.8282	181.015
2962	1.7319	-2.7948	53.8558
2963	1.7324	-1.0248	311.924
2964	1.7326	-3.9538	302.494
2965	1.7326	-3.9	350.01

2966	1.7327	-2.7601	120.292
2967	1.7332	-3.0131	337.606
2968	1.7335	-2.7545	45.0321
2969	1.7337	-2.805	251.672
2970	1.7339	-2.755	190.431
2971	1.734	-2.8272	26.9436
2972	1.7341	-4.1661	163.043
2973	1.7342	-3.4913	64.14
2974	1.7347	-2.8159	316.874
2975	1.7351	-3.8847	151.618
2976	1.7354	-2.8757	170.372
2977	1.7358	-2.7413	128.358
2978	1.736	-2.8903	11.3952
2979	1.736	-3.2536	238.186
2980	1.7361	-4.0183	346.079
2981	1.7361	-4.0886	119.514
2982	1.7364	-3.2505	88.2248
2983	1.7365	-2.9092	329.565
2984	1.7365	-2.9139	121.152
2985	1.7378	-2.9265	291.638
2986	1.7379	-2.77	224.084
2987	1.738	-3.1772	288.563
2988	1.7383	-1.3485	265.241
2989	1.7385	-3.077	187.121
2990	1.7386	-2.9363	213.212
2991	1.7388	-2.7455	107.395
2992	1.739	-3.7283	137.525
2993	1.7392	-3.8792	69.5274
2994	1.7393	-2.7707	21.054
2995	1.7393	-3.1496	216.023
2996	1.7396	-3.2957	330.43
2997	1.7399	-3.6715	256.939
2998	1.7399	-4.0409	335.898
2999	1.7402	-2.9277	231.266
3000	1.7405	-3.1411	63.8599
3001	1.7407	-2.8319	15.2562
3002	1.7409	-3.2182	267.64
3003	1.7409	-4.7326	182.251
3004	1.7411	-3.081	168.502
3005	1.7412	-3.5868	289.451
3006	1.7413	-3.1333	131.369
3007	1.7414	-3.0851	109.95
3008	1.7417	-2.7678	61.5325
3009	1.742	-3.2086	321.468
3010	1.7421	5.2573	207.572
3011	1.7422	1.3772	154.021
3012	1.7422	-4.938	124.177
3013	1.7428	-2.9602	252.568

3014	1.7431	-2.9312	155.869
3015	1.7431	-2.8443	106.389
3016	1.7433	-2.8686	117.666
3017	1.7439	-2.8496	240.2
3018	1.7439	-2.7715	28.7969
3019	1.7442	-2.7689	342.716
3020	1.7444	-2.7778	88.9408
3021	1.7446	-1.5417	358.122
3022	1.7448	-2.8399	283.484
3023	1.7449	-4.0057	206.248
3024	1.7451	-2.9626	288.966
3025	1.7455	-3.4027	347.399
3026	1.7455	-2.7723	23.2692
3027	1.7455	-3.0274	307.094
3028	1.7457	-2.7959	293.964
3029	1.7464	-2.8283	291.598
3030	1.7466	0.2519	126.632
3031	1.7469	-2.9033	319.561
3032	1.747	-2.2574	178.4
3033	1.7471	-4.0337	346.508
3034	1.7472	-2.83	0.8893
3035	1.7473	-2.2597	352.923
3036	1.7473	0.3977	218.097
3037	1.7474	-2.8111	279.541
3038	1.7474	-4.0907	313.696
3039	1.7475	-2.6236	267.348
3040	1.7485	-3.8373	141.16
3041	1.7487	-3.2988	306.644
3042	1.7488	-4.1081	334.719
3043	1.7492	-2.9471	324.82
3044	1.7493	-3.0886	153.181
3045	1.7494	-3.7269	317.262
3046	1.7497	-2.9492	19.0744
3047	1.7498	-2.6124	248.521
3048	1.7499	-3.8926	195.365
3049	1.7499	-2.9604	141.065
3050	1.7502	-2.9766	76.5062
3051	1.7508	-3.1913	254.803
3052	1.751	-3.848	251.225
3053	1.7511	-2.7722	218.68
3054	1.7512	-2.9874	297.756
3055	1.7516	-2.9659	17.3318
3056	1.7516	-2.8414	31.9744
3057	1.7518	-3.3635	169.572
3058	1.7521	-3.7508	64.9736
3059	1.7521	-2.7854	137.209
3060	1.7522	-4.0547	327.696
3061	1.7524	-2.9456	50.523

3062	1.7532	-2.772	352.703
3063	1.7532	-2.7703	328.57
3064	1.7532	4.7132	155.374
3065	1.7534	-2.9269	243.733
3066	1.7536	-2.8284	144.844
3067	1.7537	-2.7973	183.507
3068	1.7538	-4.4507	340.953
3069	1.7541	-2.8995	74.7963
3070	1.7544	-4.3096	258.244
3071	1.7546	-3.7643	0.4111
3072	1.7551	-3.9963	135.231
3073	1.7551	-2.7355	86.6411
3074	1.7552	-2.8784	16.0055
3075	1.7558	-3.031	181.447
3076	1.7564	-3.0533	307.134
3077	1.7566	-2.8934	103.014
3078	1.7575	-2.8668	258.772
3079	1.7579	-3.1079	23.524
3080	1.7582	-3.8971	249.775
3081	1.7582	-4.0186	277.594
3082	1.7582	-4.0256	302.141
3083	1.7583	-3.1317	115.128
3084	1.7585	-3.0067	40.3898
3085	1.7585	-2.9958	118.151
3086	1.759	-3.2366	84.8392
3087	1.7592	-2.9388	69.2116
3088	1.7594	-3.4444	302.28
3089	1.7594	-2.8678	223.247
3090	1.7595	-3.8538	216.292
3091	1.76	-2.8549	120.786
3092	1.76	-2.8585	233.913
3093	1.7604	-3.2038	103.123
3094	1.7605	-1.997	198.399
3095	1.761	-2.9413	281.031
3096	1.7612	-2.851	146.766
3097	1.7615	-3.1053	289.88
3098	1.7624	-2.8066	312.234
3099	1.7634	-3.9295	187.165
3100	1.7634	-1.3569	34.4567
3101	1.7636	-2.727	330.792
3102	1.7637	-4.0675	120.475
3103	1.7639	1.4121	240.174
3104	1.764	-3.339	225.723
3105	1.7647	0.0189	82.5785
3106	1.7647	-2.9013	270.38
3107	1.7649	-2.9764	37.8036
3108	1.7649	-2.9284	309.743
3109	1.7655	1.8287	27.878

3110	1.7657	-3.6985	0.4027
3111	1.7665	-4.2589	8.0414
3112	1.7665	-2.8027	196.023
3113	1.7665	-3.5222	176.407
3114	1.7668	-3.3468	14.3834
3115	1.768	-2.9432	158.64
3116	1.7686	-2.8506	43.3577
3117	1.7686	-2.7906	333.678
3118	1.7687	-3.8269	16.3245
3119	1.7688	-3.0916	122.635
3120	1.769	-2.9518	339.13
3121	1.7701	-3.0078	148.171
3122	1.7701	-3.1663	336.507
3123	1.7702	2.588	117.547
3124	1.7703	-2.8166	157.542
3125	1.7705	-3.6662	49.0157
3126	1.7707	6.0941	205.635
3127	1.7709	-5.2183	133.473
3128	1.7712	0.1035	245.346
3129	1.7712	-3.4233	341.264
3130	1.7714	-4.1048	108.454
3131	1.7716	-3.1258	214.178
3132	1.7716	-3.0672	331.218
3133	1.7717	-2.8353	270.76
3134	1.7722	-2.8957	260.258
3135	1.7723	-2.8707	309.37
3136	1.7724	-2.9275	60.9138
3137	1.7724	-3.5857	275.27
3138	1.7728	-2.852	303.693
3139	1.773	-3.818	335.575
3140	1.7732	-3.1326	134.144
3141	1.7735	7.6857	210.829
3142	1.7736	-4.1217	258.581
3143	1.7738	-4.1101	340.415
3144	1.774	-1.2715	79.135
3145	1.7742	-2.505	112.129
3146	1.7743	-1.0763	77.3925
3147	1.7745	0.1086	34.7388
3148	1.7748	-3.345	335.234
3149	1.7749	-3.011	264.001
3150	1.775	-3.2566	312.336
3151	1.7751	-4.108	124.195
3152	1.7751	-2.7398	339.218
3153	1.7751	-2.9367	296.777
3154	1.7758	-2.8899	117.668
3155	1.7761	-1.188	303.609
3156	1.7761	-2.9607	270.937
3157	1.7762	-2.765	95.7002

3158	1.7765	-2.7012	302.54
3159	1.7767	-2.9568	260.149
3160	1.7767	-2.6191	306.312
3161	1.7775	-3.8336	228.613
3162	1.7776	-2.9408	339.517
3163	1.7777	-3.9609	278.487
3164	1.7783	-1.9205	221.374
3165	1.7783	-2.6593	186.1
3166	1.7786	-2.9058	310.498
3167	1.7787	-2.8925	169.644
3168	1.7791	-2.8282	12.7604
3169	1.7792	-2.7619	303.024
3170	1.7797	-4.7281	130.544
3171	1.7803	-3.1073	334.969
3172	1.7808	-3.2036	170.117
3173	1.781	-2.9837	261.478
3174	1.7812	-0.6368	309.022
3175	1.7812	-2.8026	4.5377
3176	1.7814	-2.8365	277.415
3177	1.7816	15.5702	91.2692
3178	1.7817	-3.0714	315.055
3179	1.7818	-2.883	341.957
3180	1.7824	-1.347	145.259
3181	1.7824	-2.8271	325.315
3182	1.7827	-2.7457	306.512
3183	1.7828	-2.9048	324.144
3184	1.7835	-2.7264	114.66
3185	1.7838	-3.4733	279.821
3186	1.7838	-3.3087	33.6675
3187	1.7838	-2.9007	198.199
3188	1.784	-3.4392	300.808
3189	1.7841	-3.092	32.7877
3190	1.7841	-2.8044	284.846
3191	1.7843	-3.6116	332.581
3192	1.7844	-3.9049	213.165
3193	1.7845	-2.8553	64.6696
3194	1.7846	-4.0185	161.522
3195	1.7848	7.9989	203.263
3196	1.7851	-2.8943	359.277
3197	1.7852	-3.2898	140.588
3198	1.7857	-2.9409	314.347
3199	1.7858	-2.9035	333.99
3200	1.786	0.997	250.772
3201	1.7862	-2.9394	136.147
3202	1.7863	-3.4356	59.1854
3203	1.7864	-2.8494	266.519
3204	1.7868	-0.9167	121.771
3205	1.7873	-2.9783	329.082

3206	1.7876	-3.5371	308.125
3207	1.7881	-3.0562	111.422
3208	1.7883	-2.9856	16.6141
3209	1.7888	-3.9104	225.973
3210	1.7893	-3.1022	333.432
3211	1.7895	-2.7774	37.1179
3212	1.7896	-3.9035	58.3556
3213	1.7907	-2.9775	131.271
3214	1.7912	-2.9684	280.487
3215	1.792	-3.7595	46.0313
3216	1.7928	-1.8598	122.285
3217	1.7929	-3.6709	78.1929
3218	1.7934	0.0669	284.052
3219	1.7944	-0.9406	114.268
3220	1.7946	-3.7347	69.5387
3221	1.7946	-2.8977	205.863
3222	1.7949	-2.8635	186.549
3223	1.7951	-2.9566	276.523
3224	1.7951	-3.2291	92.7582
3225	1.7952	-1.8346	170.474
3226	1.7952	-3.7785	263.679
3227	1.7954	-2.9502	278.881
3228	1.7954	-2.9391	91.341
3229	1.7955	-2.7532	167.648
3230	1.7956	-2.8989	141.408
3231	1.7957	-2.7725	105.055
3232	1.7957	-2.8907	351.672
3233	1.796	-3.7856	216.42
3234	1.7961	-2.9933	332.241
3235	1.7963	-2.8017	15.3185
3236	1.7965	-3.0649	163.025
3237	1.7973	-2.8076	266.764
3238	1.7974	-3.7882	90.8287
3239	1.7975	-4.0916	233.831
3240	1.7976	-3.781	4.1828
3241	1.7977	-3.2583	30.0399
3242	1.7977	-2.5135	130.758
3243	1.798	-2.7643	143.288
3244	1.7984	-2.8671	350.188
3245	1.7984	-2.9174	47.14
3246	1.7986	1.0108	309.721
3247	1.7989	-2.8654	55.7816
3248	1.7993	-4.3403	123.815
3249	1.7993	1.6675	246.314
3250	1.7993	-2.8676	181.538
3251	1.7994	-3.3296	0.465
3252	1.8002	-3.985	343.921
3253	1.8003	-2.6867	297.553

3254	1.8003	-3.7895	315.639
3255	1.8005	-2.9466	59.3017
3256	1.8007	-2.9364	103.344
3257	1.8014	-3.0779	223.662
3258	1.8015	-1.4626	71.2329
3259	1.8017	-4.3669	277.141
3260	1.8021	-3.0667	188.138
3261	1.8022	-2.9134	329.555
3262	1.8022	-2.7428	11.1347
3263	1.8022	-2.6872	331.446
3264	1.8024	-2.7421	145.251
3265	1.8025	-2.7482	321.285
3266	1.8025	-2.8449	19.752
3267	1.8031	-3.0552	215.843
3268	1.8033	-2.8484	314.051
3269	1.8033	-3.0854	274.125
3270	1.8035	-3.1154	114.941
3271	1.804	-4.2549	142.478
3272	1.8045	-3.7696	123.373
3273	1.8045	-3.8792	1.7382
3274	1.8051	-3.7819	91.3814
3275	1.8051	-4.0023	326.861
3276	1.8053	-2.834	298.045
3277	1.806	-0.2287	190.28
3278	1.8062	-3.9262	116.319
3279	1.8062	-2.8254	62.0511
3280	1.8065	-2.8096	294.593
3281	1.8066	-2.8113	346.035
3282	1.8076	-4.0303	163.296
3283	1.808	-2.9565	328.081
3284	1.8081	-2.7362	323.691
3285	1.8082	-2.8108	69.7222
3286	1.8083	-3.7333	328.632
3287	1.8083	-2.7994	31.9652
3288	1.8084	-3.0101	36.1444
3289	1.8086	-2.8467	178.552
3290	1.8086	-3.3014	190.39
3291	1.809	-3.3327	249.708
3292	1.809	-3.312	137.655
3293	1.8095	1.24	296.124
3294	1.8104	-4.1936	75.2454
3295	1.8104	-0.4114	231
3296	1.8107	-3.4744	186.171
3297	1.8107	-2.9774	333.541
3298	1.8108	-3.784	183.551
3299	1.811	-4.0173	339.571
3300	1.811	-2.8202	228.742
3301	1.811	-3.1056	175.693

3302	1.8112	-3.0599	80.1823
3303	1.8113	-3.2538	88.7732
3304	1.8114	-4.1959	290.427
3305	1.8115	-2.9281	69.468
3306	1.8115	-3.6045	273.097
3307	1.8125	-4.0176	313.157
3308	1.8126	-0.6382	237.426
3309	1.8127	-3.2367	251.816
3310	1.8129	-3.7428	326.05
3311	1.8132	-2.7875	19.4424
3312	1.8133	-3.3274	328.152
3313	1.8135	-2.8647	189.936
3314	1.8142	0.9447	310.594
3315	1.8143	-2.8506	54.8576
3316	1.8144	-4.069	323.454
3317	1.8147	-3.9625	212.416
3318	1.8147	-2.9689	322.224
3319	1.8147	-2.8727	311.06
3320	1.8149	-3.6992	151.747
3321	1.8149	-3.6643	54.7876
3322	1.8153	5.9276	201.345
3323	1.8154	-2.7645	344.422
3324	1.8156	-4.622	72.9663
3325	1.8156	-3.2497	285.666
3326	1.8158	-3.3699	194.567
3327	1.8162	-2.7484	167.657
3328	1.8167	-2.8184	322.764
3329	1.817	-2.7673	16.6542
3330	1.8172	-2.9156	302.264
3331	1.8173	-2.7238	93.0386
3332	1.8174	-2.8457	106.058
3333	1.8176	-2.7341	155.888
3334	1.8177	-2.8499	343.336
3335	1.8182	-2.464	247.536
3336	1.8184	-2.8899	188.381
3337	1.8184	-3.0324	303.355
3338	1.8186	-5.9114	192.18
3339	1.8198	-3.5695	117.046
3340	1.8199	-2.9586	273.182
3341	1.82	-2.8253	342.778
3342	1.8201	2.4488	42.6007
3343	1.8201	-3.5376	215.262
3344	1.8204	-2.8223	324.716
3345	1.8205	-2.8789	107.534
3346	1.8205	-3.111	321.234
3347	1.8206	-2.8901	3.2049
3348	1.8211	-3.3402	301.738
3349	1.8212	-4.3322	103.994

3350	1.8215	-2.7316	127.74
3351	1.8216	-2.9001	160.99
3352	1.822	-2.7774	80.796
3353	1.8227	-2.7565	206.687
3354	1.8227	-4.4267	43.4339
3355	1.8232	-2.7784	49.7298
3356	1.8233	-3.0001	5.3653
3357	1.8234	-2.9985	291.799
3358	1.8235	-2.8546	230.87
3359	1.8236	0.6597	45.3405
3360	1.8236	-1.6638	352.028
3361	1.8237	-3.5171	132.19
3362	1.8237	-3.2245	71.8075
3363	1.8237	-2.8335	275.875
3364	1.8239	-2.8168	13.3114
3365	1.8239	-3.6405	301.346
3366	1.824	-2.8002	349.083
3367	1.8241	-2.8384	236.204
3368	1.8242	-2.8825	32.3915
3369	1.8243	-3.9059	228.428
3370	1.8245	-3.656	133.702
3371	1.8247	-2.9292	199.762
3372	1.8251	-3.605	283.488
3373	1.8254	-3.4034	139.897
3374	1.8254	-2.695	24.4862
3375	1.8255	-4.0439	177.778
3376	1.8255	-2.856	175.045
3377	1.8256	-3.2298	314.728
3378	1.8261	-2.8606	1.6062
3379	1.8262	-2.2074	87.4219
3380	1.8266	-2.968	338.631
3381	1.8267	-1.525	246.509
3382	1.8269	-3.2492	63.291
3383	1.8269	-2.894	263.365
3384	1.8272	-4.1209	155.575
3385	1.8275	-3.8231	177.092
3386	1.8275	3.0944	117.961
3387	1.8277	-4.5555	94.8324
3388	1.8281	-2.8605	185.678
3389	1.8282	-3.0758	78.7094
3390	1.8282	-2.8778	4.3352
3391	1.8286	-2.8685	109.068
3392	1.8286	-2.8061	178.358
3393	1.8287	-2.8117	287.511
3394	1.829	-2.6538	346.248
3395	1.829	-2.944	19.148
3396	1.8293	-2.84	189.418
3397	1.8295	-3.2269	351.561

3398	1.8296	-2.8366	338.139
3399	1.8298	-2.6313	280.026
3400	1.83	-2.8082	51.4692
3401	1.8302	-2.9049	70.2883
3402	1.8302	-2.7144	58.8397
3403	1.8303	-3.1089	12.5571
3404	1.8304	-2.9497	337.679
3405	1.8305	-3.9056	145.641
3406	1.8305	-3.9712	313.759
3407	1.8307	-2.7906	12.6317
3408	1.8314	2.2489	271.5
3409	1.8315	-3.3594	313.442
3410	1.8317	-2.8362	322.175
3411	1.8319	-3.3784	85.4767
3412	1.832	-2.9473	152.292
3413	1.8324	-2.9024	282.515
3414	1.8326	-3.9567	24.1171
3415	1.8327	-3.6202	285.723
3416	1.8328	-2.9295	90.9596
3417	1.8336	4.4078	63.8423
3418	1.8341	0.1226	14.9066
3419	1.8341	-2.8545	306.169
3420	1.8345	-2.7956	326.589
3421	1.8345	-2.7414	328.267
3422	1.8354	-3.883	89.8705
3423	1.8354	-3.8607	166.208
3424	1.8355	-3.0894	236.7
3425	1.8356	-2.8498	104.688
3426	1.836	-0.4471	259.98
3427	1.8362	-2.8422	274.11
3428	1.8363	-2.9977	303.895
3429	1.8368	-3.1346	349.064
3430	1.8372	-2.958	39.6972
3431	1.8373	-4.0692	207.121
3432	1.8374	0.3425	312.658
3433	1.8378	-3.2812	8.5278
3434	1.8378	-3.1509	9.8796
3435	1.8379	-3.9179	41.2672
3436	1.8383	-3.5068	46.1736
3437	1.8385	-2.9681	5.5316
3438	1.839	-2.9694	255.616
3439	1.8391	-2.7816	100.823
3440	1.8392	-2.808	35.7656
3441	1.8393	1.6076	24.8196
3442	1.8399	-3.6667	98.8295
3443	1.8399	-3.4561	85.6233
3444	1.8401	-3.5956	328.193
3445	1.8402	-2.934	274.363

3446	1.8402	-2.6824	98.9618
3447	1.8402	-3.4009	295.058
3448	1.8404	-2.8123	9.99
3449	1.8407	-2.7374	5.8036
3450	1.8407	-2.7223	143.653
3451	1.8407	-0.4596	252.952
3452	1.8409	-2.9318	30.0011
3453	1.841	-0.9644	251.253
3454	1.8411	-2.8017	105.991
3455	1.8413	-2.8249	5.0405
3456	1.8413	-3.238	300.564
3457	1.8414	-3.8195	175.297
3458	1.8416	-2.7392	123.299
3459	1.8416	-3.0685	10.8931
3460	1.8417	-2.9008	151.312
3461	1.8424	1.3187	244.599
3462	1.8432	-0.4439	195.022
3463	1.8434	-3.2164	9.8344
3464	1.8435	-3.5162	329.574
3465	1.8435	-2.9557	304.766
3466	1.8437	-3.0059	60.0847
3467	1.8441	-3.6163	195.239
3468	1.8442	-3.6343	347.951
3469	1.8445	-2.9406	82.1002
3470	1.8449	-2.794	356.064
3471	1.8452	-2.9134	84.2903
3472	1.8455	-2.8729	332.41
3473	1.8457	-2.8264	155.889
3474	1.8458	-3.9499	253.931
3475	1.846	-3.6392	114.443
3476	1.8462	-2.9196	258.174
3477	1.8465	-3.4974	272.012
3478	1.8468	-2.7842	354.067
3479	1.8471	-3.2434	75.5215
3480	1.8471	-2.8599	73.8384
3481	1.8475	-2.794	169.646
3482	1.8478	-0.4967	60.1706
3483	1.8482	-3.866	265.238
3484	1.8484	2.5236	275.04
3485	1.8484	-3.8888	134.898
3486	1.8486	-2.7717	12.547
3487	1.8486	-2.906	84.7636
3488	1.8487	-2.8106	247.47
3489	1.8489	-2.8663	167.713
3490	1.8489	-3.047	177.739
3491	1.8491	-2.8473	272.732
3492	1.8498	-3.3307	274.253
3493	1.8498	-2.9694	52.0605

3494	1.8501	-2.745	358.334
3495	1.8502	-3.7961	245.749
3496	1.8502	-3.2805	319.749
3497	1.8502	-3.2904	313.635
3498	1.8505	-2.5172	149.509
3499	1.8506	-0.1921	171.062
3500	1.8507	-2.9501	161.912
3501	1.8517	-3.4345	24.5421
3502	1.852	-4.1374	128.498
3503	1.8522	3.4396	58.9959
3504	1.8529	1.4625	247.894
3505	1.8531	-4.5358	173.809
3506	1.8533	-2.8019	9.9027
3507	1.8534	-3.1223	354.25
3508	1.8536	-2.8271	255.047
3509	1.8537	-2.9387	261.828
3510	1.8539	-2.9123	130.59
3511	1.8541	-2.6824	168.132
3512	1.8547	-3.2531	230.704
3513	1.8547	0.3882	35.151
3514	1.855	-3.8035	145.779
3515	1.8554	-4.0388	335.397
3516	1.8557	-2.8974	198.968
3517	1.8558	-1.5431	102.128
3518	1.8562	-3.9093	179.089
3519	1.8563	-1.3448	261.4
3520	1.8565	-2.9523	136.328
3521	1.8566	-3.0914	108.418
3522	1.8568	-2.8384	83.6703
3523	1.8571	-3.3965	56.2183
3524	1.8574	-2.8361	295.438
3525	1.8575	-2.2552	303.608
3526	1.8575	-3.7447	289.294
3527	1.8579	-3.2484	69.4799
3528	1.858	-2.9682	84.7533
3529	1.858	-2.7686	30.3931
3530	1.8581	-2.913	114.49
3531	1.8583	-2.987	34.4663
3532	1.8583	-2.9986	109.38
3533	1.8584	-2.9566	183.091
3534	1.8584	-2.7507	347.95
3535	1.8585	-3.9278	328.976
3536	1.8586	-2.7257	327.11
3537	1.8587	-2.8087	212.141
3538	1.8602	-3.9769	161.962
3539	1.8602	-2.9677	77.0974
3540	1.8607	-4.1396	282.881
3541	1.8611	-2.653	236.055

3542	1.8612	-2.8875	108.248
3543	1.8618	-3.9399	128.285
3544	1.8623	2.2502	223.362
3545	1.8626	-2.7568	271.294
3546	1.8628	-2.7139	167.202
3547	1.863	-3.4385	229.756
3548	1.8632	-2.7555	225.831
3549	1.8633	-3.5045	217.132
3550	1.8635	-2.9426	348.79
3551	1.8639	-3.0096	125.844
3552	1.8641	-2.8047	282.164
3553	1.8642	-2.8974	273.729
3554	1.8643	1.6732	132.389
3555	1.8647	-2.8396	133.168
3556	1.8656	-6.7877	35.9111
3557	1.8657	-2.8545	101.366
3558	1.8662	-3.3435	75.5965
3559	1.8664	-2.8101	356.867
3560	1.8665	-3.7618	26.4561
3561	1.8666	-2.9805	178.894
3562	1.8668	-3.0387	140.866
3563	1.8668	-3.4721	29.2261
3564	1.8669	-0.5245	70.6132
3565	1.8676	-3.9814	83.4885
3566	1.8679	-2.8737	248.954
3567	1.8679	-2.8269	209.345
3568	1.868	-3.8738	292.636
3569	1.8688	0.3127	341.865
3570	1.8691	-4.3907	236.769
3571	1.8691	-2.8213	213.387
3572	1.8692	-2.7831	87.5398
3573	1.8696	-2.999	262.804
3574	1.8705	-3.6817	260.329
3575	1.8705	-5.0223	142.525
3576	1.8706	-2.9897	291.033
3577	1.8707	-2.9264	260.144
3578	1.8707	-2.7231	320.792
3579	1.8707	-0.0716	232.284
3580	1.8714	-2.695	115.496
3581	1.8715	-3.8819	256.09
3582	1.8715	-2.807	94.084
3583	1.8716	-2.9371	161.131
3584	1.8719	-3.1352	321.023
3585	1.8723	-3.7172	75.8184
3586	1.8724	3.6121	63.9504
3587	1.8724	1.7009	119.103
3588	1.8724	0.5175	250.552
3589	1.8725	1.3728	272.831

3590	1.8728	-2.7891	165.71
3591	1.8728	-2.9126	246.954
3592	1.8731	-2.9389	18.8508
3593	1.8734	-2.9355	5.2216
3594	1.8736	-2.8677	129.387
3595	1.8738	-2.7473	46.4628
3596	1.874	0.9887	241.41
3597	1.8743	-2.9857	116.578
3598	1.8748	-3.8566	337.655
3599	1.8749	-3.0805	94.9054
3600	1.8753	2.2556	319.612
3601	1.8753	-3.722	218.022
3602	1.8755	-3.9991	205.332
3603	1.8755	-2.783	202.538
3604	1.8762	2.0961	191.557
3605	1.8769	-3.1761	220.946
3606	1.877	-3.5093	171.091
3607	1.8771	-0.6991	239.86
3608	1.8772	-3.7097	288.602
3609	1.8775	5.1647	354.57
3610	1.8776	-3.8998	350.492
3611	1.8777	-0.1327	181.846
3612	1.8777	-2.774	356.068
3613	1.8778	-2.9686	24.5231
3614	1.8779	-3.8902	30.6514
3615	1.8779	-3.849	80.0213
3616	1.878	-2.8131	274.502
3617	1.8782	-2.8793	291.335
3618	1.8788	-2.8437	133.691
3619	1.8789	-2.9549	311.35
3620	1.8793	-2.9708	359.713
3621	1.8796	-2.9207	302.884
3622	1.8798	-2.8536	328.061
3623	1.88	-4.0693	167.203
3624	1.8804	-4.0213	251.226
3625	1.8809	3.3185	144.581
3626	1.8811	-3.8584	349.322
3627	1.8812	-3.2105	3.8379
3628	1.8813	1.5638	146.264
3629	1.8813	-3.405	107.377
3630	1.8817	-2.9634	61.9986
3631	1.882	-2.7473	4.2936
3632	1.8821	-3.4661	253.023
3633	1.8822	-2.8584	294.331
3634	1.8825	0.7754	288.588
3635	1.8825	-2.8046	242.573
3636	1.8828	4.1949	249.078
3637	1.8829	1.5461	245.251

3638	1.8832	-4.1003	98.0851
3639	1.8835	-4.4096	29.5921
3640	1.8836	-2.9726	345.023
3641	1.8837	-2.9654	213.525
3642	1.8838	-2.9585	100.153
3643	1.884	-4.238	117.215
3644	1.8841	-2.9725	100.004
3645	1.8841	-2.1747	317.493
3646	1.8842	3.2982	45.2126
3647	1.8844	-1.291	326.475
3648	1.8849	0.5585	105.724
3649	1.8851	3.3879	315.236
3650	1.8855	-3.0874	24.5464
3651	1.8855	-2.8119	42.4122
3652	1.8856	-3.2659	307.96
3653	1.8857	-3.6942	132.44
3654	1.886	1.1875	42.1678
3655	1.8861	1.9022	309.32
3656	1.8862	-2.8447	270.703
3657	1.8863	0.1736	227.497
3658	1.8869	-2.9975	357.97
3659	1.8871	-3.1275	223.04
3660	1.8874	-2.8267	101.833
3661	1.8875	-2.8028	334.966
3662	1.8875	-4.8653	9.3816
3663	1.8884	-3.3466	297.213
3664	1.8885	-3.1685	237.837
3665	1.8885	-3.7809	186.835
3666	1.8888	-3.6418	265.836
3667	1.8889	-2.9792	54.0857
3668	1.8891	-2.7687	254.851
3669	1.8892	-2.7758	103.644
3670	1.8894	-2.9396	262.381
3671	1.8895	-4.248	124.14
3672	1.8896	-3.8401	289.95
3673	1.8897	-2.9425	204.323
3674	1.8898	6.0403	304.908
3675	1.89	-1.1985	344.873
3676	1.8902	-3.3432	224.466
3677	1.8906	-3.3198	100.584
3678	1.8909	-3.7566	110.222
3679	1.8913	-3.3696	19.3913
3680	1.8917	-3.844	336.147
3681	1.8917	-2.9043	327.218
3682	1.8919	-0.5233	333.621
3683	1.892	-4.4337	147.805
3684	1.8922	-2.816	137.872
3685	1.8923	-0.6107	106.507

3686	1.8923	2.5033	115.334
3687	1.8924	-3.8133	21.9901
3688	1.8927	-4.1404	318.071
3689	1.8929	-2.7907	13.2601
3690	1.893	-2.7647	214.772
3691	1.893	-3.4391	102.269
3692	1.8933	-3.4841	24.7648
3693	1.894	-2.8238	2.7972
3694	1.8941	1.4797	174.894
3695	1.8941	-3.2136	262.95
3696	1.8942	-2.7874	318.144
3697	1.8944	-0.9347	124.322
3698	1.8945	-2.9197	192.412
3699	1.8949	-2.7091	50.1204
3700	1.8951	-2.8586	10.2842
3701	1.8952	-3.7129	12.0963
3702	1.8952	-4.3038	335.667
3703	1.8955	-2.77	101.514
3704	1.8957	-2.8808	190.496
3705	1.8957	-2.7497	285.181
3706	1.8957	-3.5277	22.2782
3707	1.8959	3.037	86.1028
3708	1.8961	-2.8965	164.749
3709	1.8961	2.3083	203.042
3710	1.8965	-4.2815	55.1763
3711	1.8967	-4.2681	271.14
3712	1.8967	-2.7549	8.7241
3713	1.8969	-2.9123	90.6281
3714	1.897	1.4098	242.675
3715	1.8977	1.0938	185.828
3716	1.8981	-3.1338	262.162
3717	1.8984	-4.5186	156.33
3718	1.8985	-2.8028	92.1261
3719	1.8986	-3.137	220.683
3720	1.8986	-4.0334	101.807
3721	1.8988	-2.9578	18.1662
3722	1.8992	-3.5427	96.2328
3723	1.8995	-2.7138	303.418
3724	1.8999	-3.8866	45.8519
3725	1.9005	-0.373	118.492
3726	1.9007	-2.8781	185.031
3727	1.9008	6.6717	188.485
3728	1.9011	-2.834	289.219
3729	1.9012	-2.771	145.487
3730	1.9014	-2.9425	304.43
3731	1.9021	1.2801	304.965
3732	1.9022	-2.6779	31.1727
3733	1.9022	8.188	159.635

3734	1.9026	-2.7384	185.964
3735	1.9026	-2.8946	136.393
3736	1.9029	-3.5007	21.6015
3737	1.9037	-3.9422	288.369
3738	1.9037	-4.1853	73.7032
3739	1.904	-3.4678	267.546
3740	1.904	-3.3644	195.224
3741	1.9043	-2.7815	324.972
3742	1.9045	1.204	348.291
3743	1.9049	-4.1155	255.545
3744	1.9052	-4.1316	152.601
3745	1.9052	-2.8832	143.927
3746	1.9053	-3.4122	182.016
3747	1.9057	3.5735	149.1
3748	1.9061	-2.9104	260.204
3749	1.9061	-2.8217	29.6951
3750	1.9066	-3.1332	173.713
3751	1.9068	-3.0015	298.751
3752	1.9072	-3.3147	335.461
3753	1.9076	1.9538	243.272
3754	1.9078	-2.9051	292.484
3755	1.9078	-3.1876	307.833
3756	1.9079	-4.1444	274.989
3757	1.908	-3.936	47.7271
3758	1.9085	-3.6157	194.554
3759	1.9087	-3.8734	237.921
3760	1.9088	-2.9371	75.2622
3761	1.9088	-3.2154	119.459
3762	1.9093	-3.1965	335.408
3763	1.9093	-4.5953	282.247
3764	1.9096	-2.7552	126.812
3765	1.91	-3.6104	264.691
3766	1.9102	-2.7396	330.413
3767	1.9103	-4.0243	306.144
3768	1.9105	-3.8836	317.453
3769	1.9109	-3.4737	132.36
3770	1.9115	-3.827	47.0796
3771	1.912	-3.2621	224.995
3772	1.912	-2.7593	94.2255
3773	1.9127	18.4926	134.559
3774	1.9128	-2.9976	316.621
3775	1.913	3.7212	271.035
3776	1.9131	3.117	88.8298
3777	1.9132	-2.8005	36.4365
3778	1.9132	-2.2462	301.228
3779	1.9134	-2.9179	328.807
3780	1.9134	-3.9627	162.452
3781	1.9144	-2.7316	9.2816

3782	1.9144	-3.6545	302.468
3783	1.9145	-2.8249	76.0282
3784	1.915	-3.2481	203.576
3785	1.9152	-2.8064	272.077
3786	1.9153	1.7068	174.395
3787	1.9155	-3.838	46.1699
3788	1.9157	-3.2545	193.139
3789	1.9158	-2.9384	110.802
3790	1.9161	-2.9556	124.587
3791	1.9161	-3.8468	47.9216
3792	1.9161	-3.9688	322.074
3793	1.9162	-3.3163	184.705
3794	1.9168	-2.8207	253.148
3795	1.9172	-3.7469	345.359
3796	1.9178	-2.8918	347.557
3797	1.918	-3.5714	317.301
3798	1.9183	-2.8035	121.451
3799	1.9184	-4.2163	39.3387
3800	1.9187	-2.8028	64.6006
3801	1.9189	-2.8282	327.788
3802	1.9189	-3.6277	154.048
3803	1.919	-2.9481	284.744
3804	1.919	-2.8043	207.081
3805	1.9191	-4.4876	347.69
3806	1.9192	-2.8648	109.121
3807	1.9194	-2.9651	314.526
3808	1.9195	-2.9104	301.858
3809	1.9196	-2.732	158.156
3810	1.9199	-2.9526	318.678
3811	1.92	-2.8811	343.974
3812	1.9204	3.6032	64.6639
3813	1.9204	-3.1982	31.5428
3814	1.9204	-4.2553	326.876
3815	1.9205	-2.8878	307.139
3816	1.9205	-2.854	24.0076
3817	1.9206	-2.8195	253.651
3818	1.9206	10.0065	151.522
3819	1.9206	-3.2989	61.2285
3820	1.9212	14.0038	208.926
3821	1.9214	-2.9515	258.237
3822	1.9215	-2.9845	163.976
3823	1.9215	-6.3208	78.8134
3824	1.9216	2.1894	119.251
3825	1.9217	-4.0424	180.521
3826	1.9224	-3.1303	34.569
3827	1.9224	-3.99	307.068
3828	1.9227	-3.0854	74.5832
3829	1.9232	-3.7378	342.96

3830	1.9233	-3.9554	56.989
3831	1.9233	-2.976	244.94
3832	1.9234	-2.8884	343.734
3833	1.9238	-3.2562	134.268
3834	1.9239	2.5609	194.748
3835	1.924	-2.7806	89.2728
3836	1.924	4.5877	106.071
3837	1.9243	1.8226	194.443
3838	1.9244	-2.9587	94.9612
3839	1.9244	-2.8177	38.1165
3840	1.9248	-2.7626	162.473
3841	1.9248	-2.8766	274.508
3842	1.9254	-4.2364	289.954
3843	1.9255	-4.1346	326.005
3844	1.9258	-3.7172	291.519
3845	1.9259	-0.4073	356.224
3846	1.9263	-0.4248	110.541
3847	1.9266	-3.7369	123.321
3848	1.9266	-2.853	298.614
3849	1.9267	-4.0463	21.2115
3850	1.9271	-2.8133	216.699
3851	1.9273	-3.8074	339.515
3852	1.9273	-2.7734	335.356
3853	1.9275	-3.6635	235.887
3854	1.9276	-2.7323	320.84
3855	1.9277	-3.5687	59.6039
3856	1.9278	-2.778	277.084
3857	1.9278	1.2024	89.1042
3858	1.928	-2.874	60.172
3859	1.9283	11.8074	210.442
3860	1.9284	0.8825	183.901
3861	1.9285	8.1641	107.21
3862	1.9285	-4.3661	240.394
3863	1.9289	12.4978	138.035
3864	1.929	-2.7928	344.946
3865	1.9293	-2.7418	50.5358
3866	1.9295	-0.1022	269.168
3867	1.9297	-4.1455	352.408
3868	1.9298	3.5683	336.12
3869	1.9302	6.4611	318.895
3870	1.9304	-2.9303	257.604
3871	1.9305	6.1647	48.9389
3872	1.9306	-2.8978	169.773
3873	1.9307	-3.6865	229.909
3874	1.9311	-2.7629	176.038
3875	1.9313	-2.8401	180.134
3876	1.9317	-2.7546	195.274
3877	1.9317	-2.8262	222.719

3878	1.9318	-2.8336	290.449
3879	1.932	-3.7999	104.937
3880	1.9322	-3.2019	145.749
3881	1.9323	-2.7175	316.243
3882	1.9325	-2.8621	269.489
3883	1.933	-3.3028	348.17
3884	1.9334	-2.7499	16.8845
3885	1.9335	-3.0843	114.514
3886	1.9335	-2.7394	146.497
3887	1.9336	-2.8772	210.137
3888	1.9341	-3.6206	89.7234
3889	1.9341	1.4822	246.085
3890	1.9342	0.6008	37.62
3891	1.9343	-2.8084	320.532
3892	1.9344	-3.5404	79.3449
3893	1.9345	8.3789	64.6697
3894	1.9345	-2.8599	332.698
3895	1.9346	-2.9391	203.037
3896	1.9346	5.3004	111.022
3897	1.9349	-2.7204	74.2448
3898	1.9353	2.1992	219.064
3899	1.9354	-2.9512	205.596
3900	1.9355	-3.0456	205.543
3901	1.936	-2.8702	338.716
3902	1.9361	5.2172	142.084
3903	1.9361	-2.8595	275.241
3904	1.9362	0.9247	42.4056
3905	1.9362	2.0718	322.98
3906	1.9364	-2.8353	242.899
3907	1.9366	-3.0605	92.6486
3908	1.9371	-2.8776	119.243
3909	1.9374	1.5776	257.908
3910	1.9376	7.5755	339.104
3911	1.9377	-2.8603	308.286
3912	1.9378	0.2615	267.086
3913	1.9378	-3.0685	296.063
3914	1.9386	-3.0158	272.352
3915	1.9388	-2.8437	181.215
3916	1.9388	-4.5437	120.662
3917	1.9394	-2.8896	171.169
3918	1.9395	-3.2419	9.2023
3919	1.9401	-2.9598	296.053
3920	1.9402	-2.9057	146.568
3921	1.9405	-2.9408	91.3915
3922	1.9409	-3.4888	0.8849
3923	1.9412	-3.4317	297.878
3924	1.9414	-2.8352	120.461
3925	1.9415	-3.152	81.7881

3926	1.9419	-3.0802	279.973
3927	1.9421	-2.7126	52.0516
3928	1.9421	5.4889	128.057
3929	1.9422	-3.7753	4.587
3930	1.9425	-2.8406	161.966
3931	1.9428	-4.4154	258.337
3932	1.9429	-3.8303	21.9235
3933	1.9429	1.7967	331.592
3934	1.9434	-3.2307	50.9258
3935	1.9436	-0.745	129.752
3936	1.9437	-2.8401	163.953
3937	1.9441	-3.1431	2.1164
3938	1.9444	1.8484	127.486
3939	1.9446	4.1707	44.2594
3940	1.9448	-2.8694	38.6635
3941	1.9449	-2.6509	148.948
3942	1.9453	-2.9226	310.357
3943	1.9453	-3.0315	301.39
3944	1.9457	-2.7751	341.199
3945	1.9458	-2.8689	329.6
3946	1.946	4.4718	167.238
3947	1.9461	5.002	190.934
3948	1.9466	7.6914	37.4203
3949	1.9466	-2.9148	141.04
3950	1.9467	-2.8058	25.4486
3951	1.9469	-2.9729	352.893
3952	1.947	1.8122	200.375
3953	1.9471	-3.8788	356.753
3954	1.9476	-1.0694	281.681
3955	1.9477	-2.8632	102.748
3956	1.9478	-2.8056	73.205
3957	1.9481	-2.9546	59.249
3958	1.9482	-2.9307	107.858
3959	1.9483	0.3367	228.491
3960	1.9485	1.5141	119.026
3961	1.9487	2.0413	70.148
3962	1.949	-3.1025	78.6991
3963	1.9494	-2.9841	13.4491
3964	1.9498	-3.9179	53.6552
3965	1.9504	-4.076	40.2355
3966	1.9505	-2.7967	308.902
3967	1.9508	5.045	131.877
3968	1.9511	-2.9146	156.411
3969	1.9514	-2.7786	152.669
3970	1.9516	-2.8038	193.843
3971	1.9517	3.7478	292.703
3972	1.9521	-2.8293	69.8395
3973	1.9523	-2.826	322.593

3974	1.9523	-3.5409	126.375
3975	1.9526	-3.558	85.1792
3976	1.9526	-3.8095	106.592
3977	1.9528	9.4754	235.867
3978	1.9532	-3.0539	51.4163
3979	1.9533	-2.8582	165.916
3980	1.9535	-3.3001	21.3672
3981	1.9535	-2.759	11.1506
3982	1.9537	-4.3116	147.647
3983	1.9537	-3.8595	295.279
3984	1.9538	-4.0515	284.598
3985	1.9542	-2.7746	334.209
3986	1.9543	-3.9127	201.144
3987	1.9543	-3.8627	23.1518
3988	1.9546	-2.8138	139.019
3989	1.9547	-4.4027	37.6313
3990	1.9549	-2.8962	137.505
3991	1.955	-4.1292	327.381
3992	1.9552	-2.8882	167.965
3993	1.9552	4.7495	131.923
3994	1.9553	-4.0104	207.27
3995	1.9553	1.5789	242.947
3996	1.9554	-4.0177	331.724
3997	1.9556	-2.9621	126.482
3998	1.9556	-2.7986	47.2822
3999	1.9557	-2.9131	343.845
4000	1.9559	-2.7935	345.71
4001	1.9562	3.8414	185.456
4002	1.9563	3.9654	356.49
4003	1.9566	8.4364	57.4492
4004	1.9567	-0.7298	280.156
4005	1.9567	-2.8865	163.434
4006	1.9572	4.7879	248.41
4007	1.9573	-3.1596	80.7959
4008	1.9575	-3.5957	104.655
4009	1.9576	-3.8018	271.577
4010	1.9576	9.8678	313.549
4011	1.9585	-2.9762	335.63
4012	1.9585	-2.8349	70.4617
4013	1.9593	-2.7965	294.271
4014	1.9596	6.2401	209.899
4015	1.9597	-3.9447	263.346
4016	1.96	-3.1411	36.0523
4017	1.9601	-3.3613	268.093
4018	1.9601	-2.9734	49.7267
4019	1.9602	-1.9032	53.7717
4020	1.9604	-2.9068	68.3106
4021	1.9605	-3.195	269.43

4022	1.9606	3.3021	275.626
4023	1.9609	-3.1155	326.697
4024	1.9611	-2.8632	91.8443
4025	1.9612	-2.8783	313.663
4026	1.9614	-2.8344	47.9217
4027	1.9615	-0.4848	83.4248
4028	1.9619	-2.8095	13.7049
4029	1.9622	-2.7555	129.923
4030	1.9625	-2.981	154.786
4031	1.9626	3.2141	290.69
4032	1.9629	-3.0045	249.893
4033	1.963	-2.9275	165.405
4034	1.963	-2.9544	317.884
4035	1.9632	-3.1054	181.505
4036	1.9633	-4.3379	166.845
4037	1.9633	-2.8207	127.065
4038	1.9635	1.9623	280.007
4039	1.9639	-2.7939	99.9066
4040	1.9645	-2.9501	304.806
4041	1.9646	0.8344	191.27
4042	1.9649	-2.9193	71.7565
4043	1.965	-2.3054	18.9552
4044	1.9651	2.4021	155.032
4045	1.9653	-3.2828	185.307
4046	1.9655	2.9541	0.059
4047	1.9655	-2.9871	231.047
4048	1.9655	-3.3753	263.562
4049	1.9658	-3.2499	300.789
4050	1.966	-2.8763	101.701
4051	1.9662	2.5482	187.458
4052	1.9662	-2.8952	326.127
4053	1.9663	-3.4865	213.258
4054	1.9666	-0.0514	250.236
4055	1.9669	-0.3784	67.8534
4056	1.9671	-2.9497	3.932
4057	1.9674	1.1039	245.784
4058	1.9676	5.6205	262.58
4059	1.9679	-2.9389	76.668
4060	1.9683	0.8496	258.204
4061	1.9684	-3.9005	18.3097
4062	1.9686	0.9774	238.265
4063	1.9686	-3.6554	266.326
4064	1.9689	-3.3147	286.733
4065	1.9693	-2.8018	290.568
4066	1.9694	-2.726	249.581
4067	1.9694	-3.4467	68.5927
4068	1.9696	-3.2835	351.676
4069	1.9701	21.2992	112.43

4070	1.9701	-3.363	19.5799
4071	1.9703	1.1955	243.758
4072	1.9703	-2.8832	2.5238
4073	1.9703	-2.9966	285.502
4074	1.9706	2.7187	196.447
4075	1.9707	-2.8698	262.765
4076	1.971	-3.6815	325.557
4077	1.971	-3.5713	240.667
4078	1.9712	-3.4594	72.4285
4079	1.9713	-3.7485	64.9538
4080	1.9715	5.4046	278.001
4081	1.9716	2.8543	8.5284
4082	1.9717	-2.8031	81.6675
4083	1.9718	2.551	170.689
4084	1.9719	-2.8158	258.212
4085	1.9723	-0.4167	146.208
4086	1.9731	-2.9565	267.175
4087	1.9736	-2.9198	10.5605
4088	1.9737	6.3941	189.177
4089	1.974	-3.5899	138.519
4090	1.9743	6.1995	338.395
4091	1.9746	-2.8069	106.604
4092	1.9748	12.0867	203.802
4093	1.9749	3.3515	219.604
4094	1.9751	-2.8198	0.8851
4095	1.9751	-3.995	267.125
4096	1.9753	-2.8215	176.982
4097	1.9753	-2.9852	295.611
4098	1.9755	-3.2821	0.8426
4099	1.9757	-2.7419	138.196
4100	1.9758	-3.0612	8.2276
4101	1.9761	-3.3625	171.174
4102	1.9761	-4.032	330.784
4103	1.9761	-2.9631	24.2393
4104	1.9763	-2.9701	62.086
4105	1.9763	2.5048	224.297
4106	1.9766	8.2322	70.1406
4107	1.9767	5.3052	270.31
4108	1.9768	-3.1382	184.336
4109	1.9775	4.0786	344.58
4110	1.9781	-2.9854	267.204
4111	1.9781	-3.4367	6.9825
4112	1.9781	2.8947	261.336
4113	1.9782	-3.8797	99.4991
4114	1.9783	-4.2898	188.455
4115	1.9784	4.5942	57.1256
4116	1.9784	-2.757	345.952
4117	1.9784	-4.0592	158.008

4118	1.9785	-4.4854	163.819
4119	1.9787	0.8255	154.828
4120	1.9787	3.1223	358.459
4121	1.979	6.3195	242.79
4122	1.9791	4.2359	346.058
4123	1.9792	-2.9653	29.3008
4124	1.9792	3.3405	135.134
4125	1.9793	-2.8219	348.726
4126	1.9794	6.6337	312.874
4127	1.9796	-2.9011	337.135
4128	1.9799	-2.8938	118.004
4129	1.9799	-3.7615	12.9996
4130	1.9802	-3.2621	7.6399
4131	1.9807	7.2405	56.0567
4132	1.9811	7.3489	74.1332
4133	1.9811	4.5727	122.569
4134	1.9812	-2.8241	44.3889
4135	1.9812	-2.8353	152.472
4136	1.9813	-2.6258	323.529
4137	1.9814	-3.4835	114.599
4138	1.9819	-2.9413	151.639
4139	1.9821	2.7863	240.98
4140	1.9823	5.3041	188.893
4141	1.9828	-2.8631	49.7647
4142	1.9828	-2.956	20.6656
4143	1.9831	2.0043	237.903
4144	1.9832	-2.9406	147.433
4145	1.9832	-3.8698	296.195
4146	1.9835	-4.043	139.459
4147	1.9835	-2.8671	234.097
4148	1.9835	0.0641	340.605
4149	1.9835	-2.7999	307.661
4150	1.9836	-3.9461	274.112
4151	1.9846	-2.8875	116.286
4152	1.9849	-3.6554	22.6537
4153	1.9851	-2.7733	225.832
4154	1.9851	-3.3324	23.2102
4155	1.9852	-4.0471	116.262
4156	1.9854	-3.318	61.3165
4157	1.9854	-4.1082	195.233
4158	1.9855	-2.7897	215.572
4159	1.9857	4.0707	310.436
4160	1.986	-4.073	25.0007
4161	1.9864	-2.9039	282.215
4162	1.9865	-2.8969	249.334
4163	1.9867	-2.953	167.492
4164	1.9869	3.0891	99.2779
4165	1.9869	-2.928	229.753

4166	1.9871	-3.4784	128.127
4167	1.9872	-3.9996	179.235
4168	1.9873	6.5338	316.541
4169	1.9874	-4.1278	300.737
4170	1.9875	-2.734	335.578
4171	1.9876	-4.2049	358.644
4172	1.9878	-2.9889	233.801
4173	1.9879	16.533	315.605
4174	1.9882	2.3208	55.0396
4175	1.9884	-2.8142	176.313
4176	1.9884	-2.9528	305.025
4177	1.9885	-2.7516	3.1386
4178	1.9886	2.6334	180.936
4179	1.9888	-3.324	30.7933
4180	1.9892	-2.9645	358.35
4181	1.9892	-2.9088	250.274
4182	1.9893	2.2519	65.3464
4183	1.9896	-3.9504	327.037
4184	1.9897	-2.8087	4.327
4185	1.9897	-2.8948	304.022
4186	1.9898	-3.0426	335.511
4187	1.9899	-3.215	199.627
4188	1.9901	-2.8271	31.233
4189	1.9902	-3.0301	307.175
4190	1.9903	2.6906	227.61
4191	1.9907	-4.3071	143.329
4192	1.9909	8.7443	134.418
4193	1.9911	1.8291	208.396
4194	1.9912	-2.8542	14.7156
4195	1.9912	-3.6181	139.583
4196	1.9912	-3.9044	267.549
4197	1.9914	-2.8912	190.312
4198	1.9915	-4.2878	212.774
4199	1.9915	9.3821	138.004
4200	1.9919	-2.7866	48.8976
4201	1.992	-2.8957	296.385
4202	1.992	-2.9085	176.747
4203	1.992	-3.1665	291.089
4204	1.9921	-3.89	256.869
4205	1.9922	-2.7603	167.14
4206	1.9927	0.2889	192.395
4207	1.993	-3.0897	340.766
4208	1.9931	2.1479	237.338
4209	1.9932	-2.9721	210.375
4210	1.9934	-3.4358	349.462
4211	1.994	-2.7086	129.295
4212	1.994	-3.5217	210.706
4213	1.9941	-3.2071	333.155

4214	1.9943	-4.495	319.304
4215	1.9948	-2.9125	277.183
4216	1.9949	-2.9942	242.583
4217	1.995	-3.0605	152.284
4218	1.9951	-2.9283	301.805
4219	1.996	-4.3007	166.472
4220	1.9962	-3.5157	249.765
4221	1.9963	9.2525	23.2586
4222	1.9963	-2.9078	319.958
4223	1.9964	-0.14	46.4759
4224	1.9968	-2.9139	201.433
4225	1.9968	-2.8168	38.2461
4226	1.9969	-2.891	268.668
4227	1.9969	-4.3293	312.259
4228	1.997	-2.8902	137.142
4229	1.9973	-2.7607	285.402
4230	1.9974	-2.8407	28.7229
4231	1.9975	-2.8812	84.3993
4232	1.9977	-2.7637	82.115
4233	1.9979	-3.0296	221.086
4234	1.998	-2.8429	14.327
4235	1.9986	3.7214	296.608
4236	1.9987	-2.8466	280.449
4237	1.999	-3.1908	242.143
4238	1.9991	-4.3983	329.651
4239	1.9997	-3.113	267.734
4240	1.9997	-4.2602	312.597
4241	1.9999	-3.7266	320.412
4242	2	0.6631	145.793
4243	2.0002	-3.2178	32.5761
4244	2.0005	1.2397	125.702
4245	2.0006	-4.2041	240.949
4246	2.0009	-3.1174	133.033
4247	2.0014	-3.1239	344.185
4248	2.0014	-2.9056	147.115
4249	2.0015	-2.6803	173.27
4250	2.0016	-3.0113	292.234
4251	2.0018	-2.9428	349.59
4252	2.0026	-2.9401	273.064
4253	2.0027	-2.7861	133.773
4254	2.0027	-3.5873	32.9351
4255	2.0028	-2.893	177.628
4256	2.0028	4.9723	296.597
4257	2.0034	-3.8462	111.483
4258	2.0036	-4.6238	278.128
4259	2.0037	-3.6193	256.628
4260	2.0039	-3.9982	98.9094
4261	2.0039	-4.0222	52.2979

4262	2.004	-3.984	123.539
4263	2.0044	5.999	283.11
4264	2.0047	-3.256	320.633
4265	2.0051	-3.0769	86.2896
4266	2.0051	-3.5303	267.211
4267	2.0053	-0.0622	49.8933
4268	2.0054	-2.8769	243.69
4269	2.0054	-2.6766	64.7659
4270	2.0056	2.9397	272.694
4271	2.0056	-2.8602	230.739
4272	2.0057	-3.1115	21.7686
4273	2.0057	-2.7819	355.982
4274	2.0059	-3.1177	97.6053
4275	2.0062	-3.0482	293.279
4276	2.0063	-5.0507	310.255
4277	2.0069	7.982	9.5614
4278	2.0069	-3.4327	256.422
4279	2.0075	-3.5611	33.8444
4280	2.0076	-2.9092	66.1726
4281	2.0077	1.9414	143.89
4282	2.0079	-3.942	330.968
4283	2.0081	-4.0829	40.1866
4284	2.0084	-2.791	44.6906
4285	2.0085	-2.8028	175.994
4286	2.0087	-2.9181	267.6
4287	2.0088	-2.9119	127.923
4288	2.0088	2.9536	38.8161
4289	2.0094	3.5091	122.091
4290	2.0096	1.4596	322.194
4291	2.0098	-4.422	248.561
4292	2.0098	-2.8223	241.629
4293	2.0102	-3.9973	20.9219
4294	2.0107	3.0033	150.717
4295	2.0108	-2.9364	325.494
4296	2.0108	-1.3602	138.195
4297	2.0109	-2.8629	91.5378
4298	2.011	-3.9835	209.395
4299	2.0111	-4.5321	54.9899
4300	2.0112	2.7575	103.37
4301	2.0113	-3.057	91.9369
4302	2.0114	-3.1189	95.829
4303	2.0114	-3.8282	332.057
4304	2.0116	-4.3868	272.277
4305	2.0116	-2.7246	230.536
4306	2.0117	-3.3621	71.23
4307	2.0122	-3.2857	150.059
4308	2.0123	11.9591	203.132
4309	2.0123	-3.5482	130.849

4310	2.0124	-2.8698	330.648
4311	2.0126	7.9106	108.969
4312	2.0131	-3.5601	170.445
4313	2.0132	2.5094	136.08
4314	2.0133	-2.9022	251.859
4315	2.0136	2.3344	287.623
4316	2.0138	-4.1877	65.836
4317	2.0141	-4.2234	120.606
4318	2.0142	-2.7921	139.402
4319	2.0143	3.8945	316.011
4320	2.0143	-4.0014	40.7808
4321	2.0147	1.1682	281.146
4322	2.0147	-2.7986	298.259
4323	2.0152	-2.8205	138.951
4324	2.0153	-2.7814	342.953
4325	2.0153	-3.8991	106.696
4326	2.0157	2.5017	319.343
4327	2.0158	-2.8498	265.172
4328	2.0161	-2.7764	184.023
4329	2.0162	-3.9863	117.595
4330	2.0163	-2.916	88.034
4331	2.0163	-2.8827	308.016
4332	2.0167	3.7882	309.872
4333	2.0171	-1.8983	333.974
4334	2.0171	-2.8349	46.9648
4335	2.0178	-2.931	153.435
4336	2.0183	5.1643	243.744
4337	2.0184	11.7841	44.1584
4338	2.0184	-4.1132	270.478
4339	2.019	-4.0217	134.381
4340	2.019	-2.802	86.7334
4341	2.0191	-3.9928	105.349
4342	2.0194	-2.8799	351.27
4343	2.0198	5.9516	48.2693
4344	2.0199	4.4937	175.111
4345	2.0201	-3.2232	321.761
4346	2.0202	-3.4633	144.034
4347	2.0204	17.3644	311.334
4348	2.0206	-2.6927	192.211
4349	2.0207	-3.3286	190.137
4350	2.0208	-3.9268	315.737
4351	2.0209	-2.7873	63.1709
4352	2.021	-3.6191	168.902
4353	2.0211	-3.1234	179.148
4354	2.0212	-4.3099	230.859
4355	2.0216	-3.2324	292.88
4356	2.0219	-3.0128	192.998
4357	2.0222	-3.0373	180.401

4358	2.0223	1.9832	38.3797
4359	2.0224	-4.0785	283.675
4360	2.0228	-2.7748	118.461
4361	2.0229	-2.8066	231.642
4362	2.0231	-2.7952	5.2566
4363	2.0233	-3.125	198.506
4364	2.0234	4.1564	224.819
4365	2.0234	-3.1624	312.04
4366	2.0239	-2.9696	2.2973
4367	2.024	-2.8939	297.086
4368	2.024	-3.0656	229.987
4369	2.0242	-2.6622	333.989
4370	2.0242	-3.2777	253.171
4371	2.0247	4.4506	330.842
4372	2.025	4.6342	116.118
4373	2.0252	4.3638	255.788
4374	2.0252	-3.3605	290.059
4375	2.0254	7.6135	345.437
4376	2.0256	-1.688	86.0238
4377	2.0258	6.1621	161.568
4378	2.026	-2.8654	67.6444
4379	2.0264	-4.3859	335.162
4380	2.0265	-2.8099	173.888
4381	2.0266	-2.8085	198.227
4382	2.0266	1.3618	136.59
4383	2.0268	3.3097	263.58
4384	2.0269	-3.7822	169.419
4385	2.027	-2.9531	95.5892
4386	2.0271	-3.7953	183.534
4387	2.0277	-3.5453	317.791
4388	2.0278	7.0313	120.556
4389	2.0282	5.6594	282.205
4390	2.0291	2.1734	315.685
4391	2.0292	-2.8078	209.111
4392	2.0293	6.6151	5.7522
4393	2.0297	-2.9118	231.576
4394	2.0301	-4.0599	15.4735
4395	2.0302	-3.1256	148.364
4396	2.0304	-4.0266	177.665
4397	2.0305	-3.0414	116.051
4398	2.0305	-4.245	278.502
4399	2.0306	4.3447	210.385
4400	2.0307	4.3513	269.574
4401	2.0308	-4.2451	342.859
4402	2.031	-2.9051	288.199
4403	2.0313	3.1213	170.953
4404	2.0314	8.2977	310.417
4405	2.0315	-2.7301	305.095

4406	2.0315	-4.5309	129.729
4407	2.0316	-2.9273	120.469
4408	2.0317	-2.8255	121.797
4409	2.0321	-2.7509	161.776
4410	2.0323	-2.9042	37.5174
4411	2.0323	-3.7906	335.326
4412	2.0325	-3.4432	145.719
4413	2.0326	5.4934	73.4081
4414	2.0326	-3.9423	50.1432
4415	2.0327	-3.9189	335.484
4416	2.0328	-3.1119	71.0833
4417	2.0329	-3.2718	268.045
4418	2.033	11.6982	203.754
4419	2.0333	-2.8526	293.332
4420	2.0334	-3.4778	0.8388
4421	2.0334	-4.3318	327.575
4422	2.0334	1.878	199.391
4423	2.0336	-3.7988	212.058
4424	2.0337	-4.1446	343.184
4425	2.034	5.3872	275.031
4426	2.034	-2.7213	296.21
4427	2.0342	-3.1339	353.49
4428	2.0342	2.9245	266.829
4429	2.0343	1.0115	272.965
4430	2.0344	-2.6899	140.624
4431	2.0347	-2.7728	263.014
4432	2.0347	-2.8625	324.68
4433	2.0352	-3.0969	17.6079
4434	2.0355	8.3353	67.1754
4435	2.036	-2.7335	336.229
4436	2.0361	5.533	59.4301
4437	2.0361	-2.6723	270.954
4438	2.0364	4.7012	322.013
4439	2.0365	-3.5859	125.433
4440	2.0366	-3.7629	346.865
4441	2.0367	7.6938	299.342
4442	2.0368	-3.0114	25.8906
4443	2.037	-3.0046	164.344
4444	2.037	-2.8966	282.437
4445	2.0372	-3.8904	273.569
4446	2.0374	2.0982	20.3247
4447	2.0375	-3.5947	313.071
4448	2.0379	-3.0531	52.8222
4449	2.0384	-2.9723	143.909
4450	2.0384	-3.4297	230.135
4451	2.0387	-2.799	221.854
4452	2.0389	1.5295	237.34
4453	2.0391	-2.3681	215.723

4454	2.0393	-2.9042	340.862
4455	2.0394	6.3925	303.688
4456	2.0394	-2.6582	202.471
4457	2.0394	-5.0058	350.921
4458	2.0398	-2.9123	114.509
4459	2.0398	-3.1567	107.925
4460	2.0398	-4.1555	176.356
4461	2.04	-2.8989	196.799
4462	2.0401	9.5195	20.9063
4463	2.0401	4.833	310.31
4464	2.0401	-3.9604	255.266
4465	2.0402	7.8953	40.3064
4466	2.0403	8.7919	57.3614
4467	2.0405	-3.3613	141.016
4468	2.0407	-3.1689	232.476
4469	2.0411	-3.005	147.788
4470	2.0414	-2.9343	24.5681
4471	2.0415	2.0478	278.948
4472	2.0417	-2.7904	209.369
4473	2.0419	-5.4417	303.609
4474	2.0421	-2.8097	50.3609
4475	2.0424	-3.0196	356.101
4476	2.0424	1.9714	24.3101
4477	2.0427	9.9873	88.5863
4478	2.0429	-2.983	225.116
4479	2.0429	0.1204	127.484
4480	2.0431	-3.9934	355.446
4481	2.0432	-4.1504	186.556
4482	2.0432	2.5918	248.708
4483	2.0436	-2.9956	6.4217
4484	2.0437	-3.1212	288.341
4485	2.0439	-3.9598	335.908
4486	2.0439	-3.8553	142.277
4487	2.0441	-2.9316	33.9177
4488	2.0446	-3.3305	226.08
4489	2.0446	7.5787	244.204
4490	2.0447	4.4868	321.643
4491	2.0451	-2.7336	305.313
4492	2.0454	-2.6846	330.179
4493	2.0454	-3.0109	0.3917
4494	2.0455	-3.2109	311.929
4495	2.0457	-2.9963	254.713
4496	2.046	-5.1615	337.645
4497	2.0461	-2.6366	158.083
4498	2.0461	0.4912	126.325
4499	2.0466	-3.0832	328.765
4500	2.0466	-3.8668	226.239
4501	2.0469	-3.0684	340.248

4502	2.0469	7.9149	253.671
4503	2.047	-2.8255	266.705
4504	2.047	6.726	215.5
4505	2.0471	-2.887	143.857
4506	2.0472	-2.9741	187.503
4507	2.0472	-3.6732	324.436
4508	2.0472	-2.7931	161.577
4509	2.0473	-3.6599	273.309
4510	2.0473	-3.6146	275.844
4511	2.0475	-3.2906	243.702
4512	2.0476	-4.1814	339.326
4513	2.0478	-3.0568	261.071
4514	2.0478	7.6343	199.133
4515	2.0478	-4.2493	221.63
4516	2.048	-2.9619	18.5246
4517	2.048	-3.3096	77.3207
4518	2.0484	-2.8563	220.384
4519	2.0488	-2.8718	182.201
4520	2.0493	-2.8771	125.693
4521	2.0494	-2.9649	71.0293
4522	2.0495	-3.0343	5.8615
4523	2.0497	4.0777	109.755
4524	2.0499	8.7785	233.158
4525	2.0501	-2.9291	323.877
4526	2.0503	-3.4141	314.865
4527	2.0504	6.6502	33.0811
4528	2.0508	-2.893	245.65
4529	2.0508	-2.7774	328.957
4530	2.0509	-3.3177	120.671
4531	2.051	-3.0785	270.254
4532	2.051	-2.8169	268.702
4533	2.0513	-3.1411	314.403
4534	2.0514	-4.4083	70.1859
4535	2.0516	5.0369	256.485
4536	2.0521	-2.8202	348.796
4537	2.0521	-3.2197	325.021
4538	2.0523	-3.7143	3.2243
4539	2.0524	-2.8239	110.075
4540	2.0526	6.1547	232.059
4541	2.0527	-2.82	10.4343
4542	2.053	-3.1393	326.084
4543	2.053	7.664	291.703
4544	2.0537	-3.2386	107.366
4545	2.0537	5.4422	195.124
4546	2.054	-2.7188	340.61
4547	2.0541	-0.8393	294.454
4548	2.0541	-2.8056	157.475
4549	2.0542	-2.9718	72.2627

4550	2.0542	-3.1336	339.325
4551	2.0544	-4.587	338.132
4552	2.0547	-2.8013	230.251
4553	2.0549	-3.8834	68.8708
4554	2.0549	-4.5297	349.749
4555	2.0549	-2.8751	267.605
4556	2.055	-3.0653	152.498
4557	2.055	-2.9725	273.096
4558	2.055	1.5198	77.8103
4559	2.0551	-2.8287	354.587
4560	2.0551	-2.6998	13.1756
4561	2.0552	-4.1995	161.408
4562	2.0552	-3.656	359.016
4563	2.0553	-2.8031	316.92
4564	2.0554	-3.9468	178.333
4565	2.0555	-3.9137	274.292
4566	2.0555	5.2514	347.319
4567	2.0555	7.7969	137.239
4568	2.0561	-2.7361	34.9972
4569	2.0562	-3.5652	37.3437
4570	2.0566	-3.3943	353.779
4571	2.0569	18.26	310.425
4572	2.057	7.3614	79.2538
4573	2.0572	-2.9007	340.885
4574	2.0572	-2.8697	149.423
4575	2.0575	-2.7719	214.543
4576	2.0576	-2.9073	142.016
4577	2.0581	-3.7451	290.095
4578	2.0583	-2.7857	221.684
4579	2.0585	-2.9458	163.745
4580	2.059	-0.0451	134.117
4581	2.0592	-2.9463	2.0999
4582	2.0593	-4.008	127.211
4583	2.0594	-3.1934	74.1818
4584	2.0595	-4.6817	296.849
4585	2.0598	-1.1235	113.305
4586	2.0598	-3.2953	348.401
4587	2.0603	13.2764	287.601
4588	2.0604	-3.9204	142.964
4589	2.0606	-3.2366	197.122
4590	2.061	-2.7787	185.784
4591	2.0611	8.7518	344.163
4592	2.0614	-3.6872	9.6927
4593	2.0617	-2.9702	244.07
4594	2.0617	-3.5048	195.331
4595	2.0618	-4.2437	74.9813
4596	2.0619	-4.2877	86.881
4597	2.062	-2.8885	170.909

4598	2.0623	-2.8542	124.532
4599	2.0625	-2.8249	127.694
4600	2.0626	-4.3054	277.627
4601	2.0627	-3.4	182.136
4602	2.0628	-2.7562	185.709
4603	2.0628	7.6554	325.721
4604	2.0631	-3.0436	242.69
4605	2.0633	-2.7891	19.9876
4606	2.0637	-3.0693	352.461
4607	2.0637	-2.756	11.7401
4608	2.0639	-3.007	343.492
4609	2.0641	-2.9011	204.392
4610	2.0642	-2.8036	6.6935
4611	2.0643	-3.3494	286.134
4612	2.0644	-3.0462	103.746
4613	2.0645	-3.9155	34.9683
4614	2.0645	5.0347	126.265
4615	2.0645	-3.5764	284.61
4616	2.0648	10.8804	73.5437
4617	2.0649	-3.0949	155.203
4618	2.065	4.8228	170.339
4619	2.0651	9.9339	258.745
4620	2.0655	-2.7218	289.587
4621	2.0665	-2.8109	314.891
4622	2.0666	-2.9568	81.6558
4623	2.0668	-3.0752	28.6981
4624	2.0672	-3.1563	140.023
4625	2.0672	-2.8892	140.802
4626	2.0676	-2.9718	88.8616
4627	2.0677	-2.9083	338.233
4628	2.0681	-3.3474	22.8205
4629	2.0682	-3.676	321.324
4630	2.0683	3.8046	279.967
4631	2.0685	-3.0975	97.6991
4632	2.0686	-2.8976	273.871
4633	2.0687	-2.8565	127.785
4634	2.0689	-2.7887	212.384
4635	2.0689	-2.8272	287.762
4636	2.069	3.3632	280.748
4637	2.069	-2.7448	318.059
4638	2.069	3.4131	135.714
4639	2.0695	7.4664	196.464
4640	2.0696	5.4088	112.673
4641	2.0697	-4.2189	250.343
4642	2.0702	-2.8623	134.951
4643	2.0703	-2.9763	311.432
4644	2.0704	-2.8301	286.369
4645	2.0705	4.1016	242.164

4646	2.0705	8.9519	316.444
4647	2.0706	-2.951	91.6311
4648	2.0707	-4.4423	328.561
4649	2.0708	4.8077	174.004
4650	2.0708	10.367	39.2255
4651	2.071	-3.6123	258.57
4652	2.0712	3.6393	200.017
4653	2.0715	1.814	286.734
4654	2.0716	7.5358	195.95
4655	2.0719	-3.4473	200.117
4656	2.072	10.946	28.7195
4657	2.0721	-3.1281	4.8444
4658	2.0723	-2.829	329.29
4659	2.0726	-3.1294	359.267
4660	2.0735	8.8751	292.032
4661	2.0738	4.405	306.403
4662	2.0738	-2.7769	26.2807
4663	2.0739	-2.9222	25.6018
4664	2.074	-3.2645	330.43
4665	2.0742	2.0168	36.6399
4666	2.0743	-4.1116	83.5688
4667	2.0745	-2.9748	346.885
4668	2.0746	-3.7787	71.9921
4669	2.0748	-2.9587	260.403
4670	2.0752	-2.8075	114.714
4671	2.0754	-3.554	38.71
4672	2.0756	-3.9261	170.447
4673	2.0759	-3.3944	29.7341
4674	2.0759	-2.7436	175.404
4675	2.076	-3.0142	174.463
4676	2.0763	8.4508	57.968
4677	2.0763	4.9171	240.969
4678	2.0763	5.2046	344.564
4679	2.0763	-4.0697	333.543
4680	2.0763	-2.8747	324.413
4681	2.0765	1.3008	129.822
4682	2.0768	-3.9473	354.894
4683	2.0768	-3.0478	19.8435
4684	2.0774	-4.052	64.0028
4685	2.0775	1.2764	137.888
4686	2.0776	-2.9941	309.939
4687	2.0784	-2.897	134.022
4688	2.0785	-3.9313	335.259
4689	2.0786	-4.2354	85.8141
4690	2.0786	10.551	99.3611
4691	2.0787	6.6958	29.6029
4692	2.0788	-4.1158	200.881
4693	2.0789	8.207	173.92

4694	2.079	-2.816	113.568
4695	2.079	6.8748	232.282
4696	2.0796	-3.2066	184.441
4697	2.0798	9.9731	28.9481
4698	2.0805	-2.8965	352.612
4699	2.0808	-3.3242	27.5091
4700	2.0812	-3.8532	47.2447
4701	2.0813	-2.8769	271.596
4702	2.0814	-2.9928	75.3947
4703	2.0815	11.5782	70.8738
4704	2.0815	-3.5934	305.845
4705	2.0817	-3.1946	349.758
4706	2.0818	-3.343	8.77
4707	2.0821	-2.8863	203.027
4708	2.0821	-2.9808	82.0073
4709	2.0825	-3.1762	261.008
4710	2.0827	-4.36	72.1426
4711	2.0827	-3.8988	196.312
4712	2.0827	-3.4794	101.593
4713	2.0828	-2.8981	155.968
4714	2.0829	-2.9766	10.3975
4715	2.0829	-4.2308	111.467
4716	2.0831	10.4035	25.4561
4717	2.0832	-3.3116	9.5956
4718	2.0833	5.7091	112.987
4719	2.0833	2.9095	11.4893
4720	2.0835	-4.3082	17.3531
4721	2.084	-4.8205	271.327
4722	2.0842	-4.0237	179.979
4723	2.0843	-2.9185	347.531
4724	2.0844	3.9101	12.13
4725	2.0845	-3.4394	150.631
4726	2.0847	-4.3122	99.6952
4727	2.0847	9.148	318.861
4728	2.0849	8.1035	78.1569
4729	2.0849	-2.7699	122.774
4730	2.0852	-2.8841	179.796
4731	2.0854	6.9385	187.016
4732	2.0854	-2.9167	26.5626
4733	2.0855	-3.9604	95.7488
4734	2.0857	-3.4431	175.48
4735	2.0861	-3.1063	157.298
4736	2.0864	10.6564	210.868
4737	2.0865	-3.8701	48.2137
4738	2.0868	-2.7899	147.771
4739	2.087	-4.4284	114.559
4740	2.0873	3.7238	327.245
4741	2.0874	-2.9449	310.762

4742	2.0876	-2.8728	292.547
4743	2.0876	-2.8489	165.062
4744	2.0878	-3.0013	18.423
4745	2.0878	-3.9532	272.964
4746	2.0878	-3.901	316.215
4747	2.0884	7.7999	41.6472
4748	2.0884	-4.2802	307.984
4749	2.0887	6.5671	187.725
4750	2.0888	-2.9151	231.959
4751	2.0889	-3.5278	9.0391
4752	2.089	-3.1551	233.944
4753	2.0891	9.87	313.469
4754	2.0891	-2.6781	184.388
4755	2.0891	-3.849	301.847
4756	2.0892	-3.2184	268.072
4757	2.0893	-2.9869	189.893
4758	2.0893	-2.8837	312.642
4759	2.0894	-2.8312	329.71
4760	2.0896	-3.1813	100.815
4761	2.0903	11.8827	320.356
4762	2.0904	-3.6875	332.286
4763	2.0904	4.6464	260.883
4764	2.0904	-2.7931	341.828
4765	2.0905	-2.8036	153.704
4766	2.0908	-2.786	346.629
4767	2.091	8.6874	229.731
4768	2.0915	7.1543	206.557
4769	2.0915	6.3847	109.997
4770	2.0916	4.5206	133.982
4771	2.0917	11.4624	59.3044
4772	2.0917	8.8912	207.351
4773	2.0918	4.5235	253.215
4774	2.0918	-4.1155	9.3559
4775	2.0918	-3.6695	60.3349
4776	2.0922	-3.9218	69.3433
4777	2.0924	-3.127	320.366
4778	2.0924	-4.0216	177.781
4779	2.0925	-3.7176	57.2232
4780	2.0925	3.3671	125.535
4781	2.0925	-2.8426	61.2087
4782	2.0928	-3.1927	140.32
4783	2.0929	-2.9912	5.0292
4784	2.093	6.4772	68.6347
4785	2.0932	5.3853	179.317
4786	2.0933	-2.9997	299.775
4787	2.0936	-3.777	57.0912
4788	2.0937	-2.9954	9.7206
4789	2.0943	-3.0673	54.2733

4790	2.0943	14.0928	210.432
4791	2.0944	11.2243	27.217
4792	2.0946	-3.1314	323.715
4793	2.0946	11.8006	14.2325
4794	2.0947	-3.0886	277.278
4795	2.0947	-3.9022	46.9746
4796	2.095	-2.8838	194.223
4797	2.095	-3.8709	91.3575
4798	2.0951	-3.2306	96.3827
4799	2.0954	-3.9491	299.927
4800	2.0955	-2.9609	170.765
4801	2.0958	-2.7907	65.3302
4802	2.096	13.862	208.817
4803	2.096	-2.7829	105.785
4804	2.0964	7.7093	326.056
4805	2.0965	4.9277	20.4336
4806	2.0967	-3.0018	315.221
4807	2.0968	7.1677	194.029
4808	2.0974	-2.8753	124.145
4809	2.0974	-2.9034	128.607
4810	2.0976	-3.393	274.032
4811	2.0976	-2.9854	193.809
4812	2.0977	10.8177	186.289
4813	2.098	-3.4457	86.3304
4814	2.0981	-2.9983	108.224
4815	2.0982	-2.8292	190.155
4816	2.0982	6.4961	223.131
4817	2.0984	7.1909	184.085
4818	2.099	-3.8949	139.121
4819	2.0991	5.3966	148.952
4820	2.0994	10.1163	233.452
4821	2.0996	-0.8055	232.652
4822	2.0997	-2.8743	132.632
4823	2.0998	-2.9373	155.704
4824	2.1001	-3.936	151.134
4825	2.1002	7.9404	235.199
4826	2.1005	-3.7892	5.7218
4827	2.1006	8.5405	111.199
4828	2.1008	-3.6803	113.083
4829	2.1008	-2.762	177.556
4830	2.1009	-2.9948	157.664
4831	2.101	-3.5063	147.764
4832	2.1016	8.0292	344.829
4833	2.1017	-2.9616	77.424
4834	2.1019	5.932	27.5927
4835	2.1021	-3.9618	145.119
4836	2.1022	10.3199	36.5531
4837	2.1022	-2.7949	29.7849

4838	2.1025	-3.4074	7.7347
4839	2.1025	-3.2678	267.511
4840	2.1028	-3.037	196.607
4841	2.1031	-3.7662	236.929
4842	2.1031	2.3758	130.249
4843	2.1033	4.332	230.797
4844	2.1033	4.5948	254.617
4845	2.1033	-2.7749	24.7412
4846	2.1036	-3.525	296.088
4847	2.1038	-3.2146	123.31
4848	2.1038	-0.3167	200.31
4849	2.104	7.848	274.415
4850	2.1044	-2.7563	143.324
4851	2.1045	-3.9846	279.435
4852	2.1046	7.1048	335.963
4853	2.1047	-2.8246	346.26
4854	2.1047	-3.3041	352.48
4855	2.1052	8.454	71.4355
4856	2.1054	10.2027	253.437
4857	2.1056	-5.0499	176.356
4858	2.1058	12.4346	203.763
4859	2.1059	-2.8176	62.1152
4860	2.1063	5.6419	143.922
4861	2.1065	-3.2436	5.6229
4862	2.1065	1.7568	297.359
4863	2.1065	-2.7819	100.557
4864	2.1073	11.0133	272.756
4865	2.1073	-2.9964	86.8777
4866	2.1073	-2.8558	206.591
4867	2.1074	8.1494	329.465
4868	2.1074	-4.0124	180.528
4869	2.1076	-3.4651	333.744
4870	2.1078	-2.906	167.38
4871	2.1078	-2.7712	160.975
4872	2.108	11.8521	74.6136
4873	2.108	-3.0753	331.445
4874	2.1081	8.4971	201.036
4875	2.1082	11.8617	303.302
4876	2.1083	-2.6173	281.954
4877	2.1087	-2.8493	228.183
4878	2.1094	-2.7961	163.66
4879	2.1094	-3.9887	335.376
4880	2.1095	0.9891	339.097
4881	2.1095	-3.3857	89.8096
4882	2.1097	-2.8561	251.904
4883	2.1099	2.3598	286.269
4884	2.1102	12.0434	245.282
4885	2.1103	7.0793	308.964

4886	2.1104	-3.7741	235.624
4887	2.1106	-2.7861	49.1391
4888	2.111	-3.536	37.8507
4889	2.111	4.0601	280.584
4890	2.111	4.355	100.409
4891	2.1115	-2.9777	62.7922
4892	2.1115	-3.5262	308.413
4893	2.1116	-4.0492	265.217
4894	2.1116	-2.9171	171.318
4895	2.1125	-3.2322	294.08
4896	2.1129	4.8878	0.0161
4897	2.1137	16.5268	241.347
4898	2.1139	-2.9641	140.061
4899	2.1139	-3.3214	325.871
4900	2.1142	-4.5292	124.425
4901	2.1145	-2.9383	260.673
4902	2.1147	-4.282	133.432
4903	2.1149	-2.7855	173.074
4904	2.1149	12.8128	316.618
4905	2.115	-4.031	337.85
4906	2.1151	-3.2266	66.121
4907	2.1154	-2.9357	330.339
4908	2.1155	-2.844	113.598
4909	2.1159	-2.8486	141.62
4910	2.1159	-3.6321	192.002
4911	2.1161	11.9306	316.558
4912	2.1163	-3.2449	91.9813
4913	2.1166	-4.5273	337.472
4914	2.1168	7.3284	65.6236
4915	2.1171	-3.8713	38.491
4916	2.1171	3.2425	107.836
4917	2.1172	-3.4547	122.205
4918	2.1176	-2.8489	64.4073
4919	2.118	-3.7962	78.1782
4920	2.1188	-2.9251	290.138
4921	2.1188	-2.8216	141.439
4922	2.1189	-5.0172	296.07
4923	2.1197	4.667	119.659
4924	2.1198	-3.891	212.361
4925	2.1198	-2.884	293.713
4926	2.1199	14.0856	205.164
4927	2.1205	-3.4954	151.166
4928	2.1206	-2.7231	141.876
4929	2.121	9.9548	224.76
4930	2.1212	-4.2148	291.318
4931	2.1213	8.0228	261.495
4932	2.1214	-2.7419	210.807
4933	2.1214	-3.782	27.7631

4934	2.1215	8.2067	217.829
4935	2.1216	-3.2492	122.191
4936	2.1217	-2.8531	311.052
4937	2.1221	-2.8644	235.052
4938	2.1223	-2.8896	163.324
4939	2.1223	-4.0614	24.1958
4940	2.1224	-4.144	337.475
4941	2.1225	5.2646	111.385
4942	2.1231	-3.5476	109.632
4943	2.1235	2.3044	217.18
4944	2.1236	-3.539	171.478
4945	2.1241	5.6544	255.033
4946	2.1242	-4.1852	67.035
4947	2.1242	-2.7164	78.5829
4948	2.1243	-2.7677	165.102
4949	2.1246	-3.1649	126.486
4950	2.1247	4.1705	124.529
4951	2.1252	5.508	7.1809
4952	2.1252	4.3695	339.281
4953	2.1252	-2.8045	142.769
4954	2.1253	-4.1153	342.734
4955	2.1253	-2.9019	266.943
4956	2.1254	-3.7438	35.3799
4957	2.1255	15.4021	309.888
4958	2.1255	-3.0147	204.877
4959	2.1255	-2.7587	357.085
4960	2.1257	-3.5863	20.4807
4961	2.1258	-3.1378	215.045
4962	2.1259	5.0362	147.193
4963	2.126	-3.2228	97.8687
4964	2.1262	-4.0426	302.056
4965	2.1264	-3.1488	342.656
4966	2.1265	-4.192	98.4965
4967	2.127	-2.9318	15.5884
4968	2.127	8.4758	81.2681
4969	2.1273	-3.7217	272.879
4970	2.1274	-3.9678	11.4021
4971	2.1274	2.3529	132.802
4972	2.1275	5.0017	217.75
4973	2.1277	-2.7872	348.355
4974	2.1283	9.3781	197.092
4975	2.1285	8.1131	203.908
4976	2.1292	8.8189	91.2827
4977	2.1298	-2.8603	175.307
4978	2.1301	8.9947	218.512
4979	2.1304	8.8234	25.1725
4980	2.1304	-3.9354	94.8115
4981	2.1304	8.0188	334.899

4982	2.1305	-3.2253	106.671
4983	2.1307	-3.4011	313.093
4984	2.1307	-3.3321	307.55
4985	2.1308	-2.7738	198.153
4986	2.131	-2.8859	234.59
4987	2.1311	8.5045	196.993
4988	2.1311	8.389	297.891
4989	2.1312	-3.7987	38.126
4990	2.1319	-4.1004	303.437
4991	2.1321	-3.2155	269.993
4992	2.1327	-3.2385	332.535
4993	2.133	-3.807	312.598
4994	2.1332	-2.7973	333.628
4995	2.1332	-4.0157	287.967
4996	2.1338	-3.8427	211.738
4997	2.1339	-3.191	341.459
4998	2.1341	-4.1557	2.5278
4999	2.1342	-3.2475	254.421
5000	2.1345	-3.7996	256.59
5001	2.1346	-2.8012	319.501
5002	2.1348	-2.8803	6.2994
5003	2.135	-2.7131	284.411
5004	2.1352	-2.6835	331.743
5005	2.1353	-4.2877	108.096
5006	2.1353	-2.9179	302.574
5007	2.136	-4.5113	145.006
5008	2.1365	-2.6398	112.04
5009	2.137	12.3977	241.013
5010	2.137	-2.9176	345.088
5011	2.1375	10.8976	195.296
5012	2.1376	6.5657	163.333
5013	2.1377	-2.7906	358.828
5014	2.1379	2.377	131.626
5015	2.138	-2.8116	200.93
5016	2.1381	10.0831	81.1965
5017	2.1383	-3.1497	299.393
5018	2.1385	-2.9557	114.989
5019	2.1386	-3.2696	0.9495
5020	2.1386	13.3583	308.269
5021	2.1388	-3.2135	118.98
5022	2.1389	-2.8722	103.776
5023	2.1389	-3.07	217.347
5024	2.1392	-2.842	150.399
5025	2.1393	-3.9131	335.797
5026	2.1397	6.2039	312.765
5027	2.1399	-2.901	156.404
5028	2.1399	-2.7971	339.081
5029	2.14	5.8452	281.768

5030	2.1403	-2.9022	88.3358
5031	2.1405	-2.9328	105.194
5032	2.1411	5.924	77.0484
5033	2.1414	13.2891	235.378
5034	2.1417	12.8424	134.495
5035	2.1422	-3.0303	161.661
5036	2.1425	-3.0786	128.233
5037	2.1425	-2.8356	149.344
5038	2.1434	-2.8492	163.542
5039	2.1436	2.656	222.068
5040	2.1438	23.9691	88.0537
5041	2.144	-4.5454	80.3357
5042	2.1441	8.8707	7.1073
5043	2.1442	8.8502	100.472
5044	2.1444	6.5983	266.988
5045	2.1446	-2.9724	137.859
5046	2.1449	8.7904	122.495
5047	2.145	-2.8	136.428
5048	2.1451	-4.1632	119.578
5049	2.1452	7.5543	203.426
5050	2.1453	-4.4827	23.8891
5051	2.1454	-3.8307	210.194
5052	2.1459	-2.9965	220.844
5053	2.146	8.9462	297.666
5054	2.1461	-2.9591	321.344
5055	2.1462	8.6018	264.813
5056	2.1463	-3.4134	352.436
5057	2.1466	-3.4516	117.034
5058	2.1467	9.8135	289.788
5059	2.1468	-2.9643	0.4953
5060	2.1468	-2.9681	74.5228
5061	2.1469	-3.3651	317.524
5062	2.1473	-3.2315	213.473
5063	2.1478	-3.0981	223.091
5064	2.1481	7.6341	105.234
5065	2.1481	-2.7638	347.681
5066	2.1485	-4.5162	204.667
5067	2.1487	-4.1507	118.309
5068	2.1488	-2.9613	83.0232
5069	2.1491	17.3274	307.796
5070	2.1493	-4.7187	293.745
5071	2.1494	-2.9493	340.467
5072	2.1494	6.2965	253.681
5073	2.1494	12.8112	209.579
5074	2.1495	12.0947	0.1761
5075	2.1496	-3.968	4.2895
5076	2.1499	-3.2538	110.096
5077	2.15	-2.9785	281.385

5078	2.1502	-2.8475	322.039
5079	2.1506	7.3054	175.704
5080	2.1506	13.2982	65.5901
5081	2.1509	-2.9667	24.794
5082	2.1512	-4.7398	198.102
5083	2.1513	-2.9575	157.655
5084	2.1514	-3.5535	312.082
5085	2.1518	-3.8764	180.392
5086	2.1521	-2.8293	211.159
5087	2.1523	4.8839	156.032
5088	2.1527	-2.7915	351.71
5089	2.153	-2.8874	295.239
5090	2.1531	-2.8645	104.141
5091	2.1532	12.6486	206.966
5092	2.1539	-4.4623	92.089
5093	2.1542	-2.852	183.205
5094	2.1542	-2.8079	230.759
5095	2.1543	-4.2556	343.719
5096	2.1548	14.2641	29.5523
5097	2.1549	-2.82	333.749
5098	2.1549	-3.0987	276.128
5099	2.1553	-4.73	264.396
5100	2.1559	-3.5724	338.42
5101	2.156	-2.8461	45.3941
5102	2.1563	-2.7767	323.147
5103	2.1565	-2.9408	332.449
5104	2.1566	-2.9358	177.715
5105	2.1567	-2.9483	338.955
5106	2.1574	-2.8241	9.4023
5107	2.1575	9.4269	100.631
5108	2.1576	13.7789	30.256
5109	2.1577	-4.027	273.637
5110	2.1577	5.5364	342.21
5111	2.1579	-3.8056	300.275
5112	2.1579	-2.7114	61.6006
5113	2.1583	-3.6214	26.9324
5114	2.1584	8.6945	138.193
5115	2.1586	-2.8112	111.213
5116	2.1587	-3.9605	316.413
5117	2.1588	-2.7687	247.593
5118	2.159	8.1502	26.1212
5119	2.1591	-3.9557	297.133
5120	2.1594	-2.8335	8.1052
5121	2.1595	-2.9633	75.9154
5122	2.1595	-2.81	133.854
5123	2.1595	-2.8473	34.9332
5124	2.1595	-3.5576	188.466
5125	2.1597	9.5387	321.791

5126	2.1598	5.5274	259.381
5127	2.1603	-4.2841	136.1
5128	2.1607	-2.9928	54.041
5129	2.1613	-3.8871	120.055
5130	2.1613	-2.7729	104.447
5131	2.1614	-2.6692	151.291
5132	2.1616	9.958	33.3394
5133	2.1618	6.2208	236.083
5134	2.1618	-2.6598	351.983
5135	2.1619	10.3272	221.171
5136	2.162	8.817	105.138
5137	2.162	-4.4283	270.662
5138	2.1622	12.8648	294.018
5139	2.1622	11.5592	140.553
5140	2.1627	9.5833	24.6503
5141	2.1629	-2.9639	174.822
5142	2.163	10.1882	274.265
5143	2.1634	9.4675	259.49
5144	2.1635	-3.213	113.588
5145	2.1637	-2.7951	119.084
5146	2.1637	4.2856	256.027
5147	2.1638	3.9441	335.097
5148	2.1638	-2.9436	49.42
5149	2.164	-2.9217	210.12
5150	2.1642	-3.1816	227.286
5151	2.1642	8.5793	280.346
5152	2.1643	-3.7571	179.768
5153	2.1645	-2.8191	277.616
5154	2.1648	-2.9462	273.596
5155	2.1649	-2.915	245.046
5156	2.1655	10.9798	39.1976
5157	2.1655	-2.9334	19.5665
5158	2.1657	12.5908	113.999
5159	2.166	-2.8386	358.448
5160	2.1661	-2.8522	93.1983
5161	2.1661	5.3932	259.181
5162	2.1663	15.8696	313.877
5163	2.1665	-2.95	167.164
5164	2.1666	-3.0361	264.005
5165	2.1667	8.7975	344.131
5166	2.1667	6.555	222.919
5167	2.1667	-2.8	338.264
5168	2.1668	8.3918	190.9
5169	2.1668	9.3123	201.164
5170	2.1671	-4.0222	292.771
5171	2.1674	17.2554	293.127
5172	2.1678	-3.2783	73.6181
5173	2.1678	-2.8852	261.152

5174	2.168	-2.6653	332.326
5175	2.168	21.2819	312.936
5176	2.1682	-2.8516	330.484
5177	2.1683	-3.8485	4.7372
5178	2.1683	-3.2996	331.701
5179	2.1684	7.1898	330.499
5180	2.1685	-4.18	331.981
5181	2.1686	11.8823	79.0173
5182	2.1689	3.0607	125.478
5183	2.1691	8.1934	108.425
5184	2.1692	8.2208	189.695
5185	2.1695	22.0949	310.746
5186	2.1697	12.4478	130.861
5187	2.17	12.1927	218.63
5188	2.17	12.7931	209.533
5189	2.1704	-2.9625	98.134
5190	2.1706	-2.9621	82.0322
5191	2.1707	-4.1425	106.934
5192	2.1709	5.8523	114.402
5193	2.171	-2.8639	307.313
5194	2.1715	-2.8586	121.001
5195	2.1719	-3.9699	176.807
5196	2.1719	-2.7519	27.8133
5197	2.1721	9.5047	282.447
5198	2.1723	-4.1213	86.0685
5199	2.173	-2.9623	310.525
5200	2.173	7.557	350.044
5201	2.1732	13.8985	297.656
5202	2.1732	-3.0162	349.753
5203	2.1732	-2.8229	110.013
5204	2.1736	-3.9157	262.782
5205	2.1737	12.404	218.035
5206	2.1737	-2.8007	46.4792
5207	2.174	-4.1214	262.975
5208	2.1742	5.9513	127.969
5209	2.1742	13.2702	82.6997
5210	2.1744	12.0929	57.9376
5211	2.1746	11.5642	91.4658
5212	2.1747	-2.8838	179.123
5213	2.1747	-2.7961	127.079
5214	2.1748	18.3579	216.706
5215	2.1751	11.505	77.5183
5216	2.1752	8.4412	38.4947
5217	2.1753	-3.7056	40.8884
5218	2.1756	-3.0685	222.03
5219	2.1756	-2.8096	144.23
5220	2.1757	-3.451	342.075
5221	2.1761	-2.8735	313.713

5222	2.1761	-2.7895	164.588
5223	2.1762	11.0354	349.266
5224	2.1763	-4.1044	18.3546
5225	2.1764	13.672	294.687
5226	2.1764	10.8677	252.29
5227	2.1767	6.4382	5.242
5228	2.1768	-3.4203	184.294
5229	2.1769	-2.8609	93.5836
5230	2.1769	-2.9636	151.263
5231	2.177	-3.1535	147.125
5232	2.1773	0.0387	37.8071
5233	2.1775	-4.2536	120.145
5234	2.1775	10.8004	246.747
5235	2.1775	-2.8415	154.059
5236	2.1777	-3.339	168.394
5237	2.1778	-3.7483	85.2551
5238	2.1778	6.5191	285.036
5239	2.1778	9.1507	34.5878
5240	2.1781	7.1097	134.237
5241	2.1781	3.5701	225.196
5242	2.1785	-2.8721	176.918
5243	2.1785	7.2895	250.131
5244	2.1789	-4.0673	125.099
5245	2.179	7.3423	33.1237
5246	2.1791	-3.0497	167.13
5247	2.1791	12.5358	232.608
5248	2.1794	-2.9704	324.713
5249	2.1797	-4.2861	173.794
5250	2.1798	-3.9643	331.413
5251	2.1801	-2.8656	125.809
5252	2.1805	-2.9599	281.714
5253	2.1805	-3.9194	184.311
5254	2.1807	12.0651	196.576
5255	2.1808	-3.1667	2.3668
5256	2.1808	-3.3719	24.3981
5257	2.181	9.7952	127.474
5258	2.1811	-4.2067	125.432
5259	2.1817	5.521	147.878
5260	2.1818	-3.9469	158.473
5261	2.1819	-3.4368	351.473
5262	2.182	-2.9207	107.195
5263	2.1821	-4.9477	262.582
5264	2.1823	17.5486	41.3554
5265	2.1823	-4.2563	334.885
5266	2.1823	-2.806	348.716
5267	2.1824	-2.8493	323.304
5268	2.1829	-2.9378	155.689
5269	2.1829	-3.3708	304.058

5270	2.183	-4.2026	171.143
5271	2.1832	11.8747	75.1064
5272	2.1832	7.6258	280.189
5273	2.1834	-3.2629	33.3006
5274	2.1834	-2.7624	257.065
5275	2.1835	-3.177	28.711
5276	2.1836	10.2817	72.0648
5277	2.1837	-4.094	39.2364
5278	2.1838	9.187	148.491
5279	2.184	5.983	244.586
5280	2.184	-2.7468	92.1466
5281	2.1841	-4.2252	151.282
5282	2.1843	15.6073	19.3563
5283	2.1847	7.3225	243.391
5284	2.1847	-3.8994	8.4259
5285	2.1848	-3.7836	46.1562
5286	2.1851	-2.6807	176.04
5287	2.1851	-3.5628	353.359
5288	2.1851	-2.8655	323.801
5289	2.1854	4.9673	300.657
5290	2.1854	3.296	265.139
5291	2.1854	-2.8088	303.257
5292	2.1858	-3.7664	72.2704
5293	2.1859	-2.9177	279.153
5294	2.1873	9.904	134.515
5295	2.1878	-2.9484	76.3423
5296	2.1881	-4.1374	229.39
5297	2.1891	-2.873	68.8633
5298	2.1891	-2.8321	318.624
5299	2.1892	4.4328	297.951
5300	2.1894	9.2234	36.8606
5301	2.1894	-2.8956	342.615
5302	2.1895	5.6692	194.244
5303	2.1895	-2.8577	341.283
5304	2.1899	8.571	284.329
5305	2.19	-3.9472	197.862
5306	2.1901	-2.7832	128.602
5307	2.1902	5.988	203.548
5308	2.1903	8.8479	152.421
5309	2.1903	12.4962	84.8274
5310	2.1904	-2.9729	336.916
5311	2.1906	13.2792	56.1921
5312	2.1906	-2.7892	270.232
5313	2.1907	-2.9452	282.74
5314	2.1912	11.8869	194.629
5315	2.1913	-4.083	330.514
5316	2.1915	6.2219	201.726
5317	2.1915	-2.7366	248.863

5318	2.1915	13.2893	208.053
5319	2.1918	-2.7995	42.0704
5320	2.1918	10.3919	117.386
5321	2.1919	-3.5303	135.748
5322	2.1922	-2.932	304.27
5323	2.1923	15.8295	192.21
5324	2.1923	9.8213	70.9111
5325	2.1923	9.3208	23.2309
5326	2.1924	13.6965	90.5841
5327	2.1925	-2.7107	142.249
5328	2.1926	-2.8107	205.697
5329	2.1927	-2.7397	56.632
5330	2.1927	-4.3552	343.621
5331	2.1927	-2.8136	325.853
5332	2.1928	-2.9281	244.544
5333	2.1929	-2.9339	320.151
5334	2.193	-2.9696	151.961
5335	2.193	8.7445	254.091
5336	2.1934	-2.876	185.879
5337	2.1937	-4.1006	306.732
5338	2.1939	-2.9774	115.423
5339	2.1939	-2.6895	295.899
5340	2.194	-2.929	40.655
5341	2.1941	-3.1094	26.8009
5342	2.1941	-2.9287	2.5921
5343	2.1941	-2.7827	81.993
5344	2.1943	-3.1942	25.1583
5345	2.1943	-2.7647	159.305
5346	2.1944	17.4211	187.99
5347	2.1946	-2.9233	155.939
5348	2.1946	6.7928	139.959
5349	2.195	16.6656	338.558
5350	2.1953	-4.5805	236.741
5351	2.1954	15.6002	289.699
5352	2.1954	-2.8119	180.484
5353	2.1956	10.0838	153.132
5354	2.1957	-2.9554	174.903
5355	2.1957	-2.8172	78.4537
5356	2.1959	-3.2044	305.948
5357	2.1959	-3.651	267.716
5358	2.1961	10.1521	259.925
5359	2.1961	-2.8494	182.422
5360	2.1962	-4.4416	274.053
5361	2.1967	-2.9749	84.2069
5362	2.1968	-2.8903	287.134
5363	2.1969	9.2013	47.4258
5364	2.1969	-2.785	316.485
5365	2.1971	-2.8117	296.323

5366	2.1972	12.0731	270.663
5367	2.1973	-4.0931	311.157
5368	2.1974	6.6829	28.2453
5369	2.1976	-3.9301	292.132
5370	2.1978	10.5476	200.864
5371	2.1978	-3.8145	27.1119
5372	2.1979	-4.0078	152.091
5373	2.1984	8.8589	276.969
5374	2.1985	-2.9463	308.982
5375	2.1986	-2.672	337.456
5376	2.1986	-2.7176	82.7625
5377	2.199	7.0256	36.106
5378	2.199	-3.9796	117.613
5379	2.1991	-2.8154	250.677
5380	2.1991	-3.9457	164.445
5381	2.1992	-3.8773	110.339
5382	2.1992	-2.8079	193.164
5383	2.1995	14.1322	176.176
5384	2.1996	-2.7615	78.6814
5385	2.1996	-2.7212	14.5558
5386	2.1996	10.8189	258.145
5387	2.1997	-3.0356	292.486
5388	2.1998	-3.3561	17.9452
5389	2.2004	28.1293	87.723
5390	2.2005	-2.9997	91.3577
5391	2.2006	-3.9349	18.0898
5392	2.2008	5.1656	256.125
5393	2.2013	-2.9723	23.2117
5394	2.2014	-3.6951	252.112
5395	2.202	-2.8195	103.724
5396	2.202	-4.3849	176.912
5397	2.2021	-3.4248	9.8868
5398	2.2021	12.9412	72.5265
5399	2.2025	12.0076	287.965
5400	2.2029	-4.5162	88.299
5401	2.2029	18.0367	206.396
5402	2.2029	-3.5475	262.211
5403	2.203	-3.2552	148.458
5404	2.2034	-3.3206	64.5417
5405	2.2036	8.9405	188.729
5406	2.2038	12.4122	303.38
5407	2.2038	-2.7893	116.311
5408	2.204	7.6646	200.219
5409	2.2041	12.2815	354.321
5410	2.2041	-2.8985	135.801
5411	2.2042	-3.7561	317.334
5412	2.2049	11.098	28.6971
5413	2.2053	-3.1956	28.9831

5414	2.2053	7.7422	180.613
5415	2.2056	10.4424	119.219
5416	2.2058	-2.9623	275.123
5417	2.2063	-4.444	317.756
5418	2.2064	-3.5854	2.5496
5419	2.2064	-3.1399	169.13
5420	2.2065	15.6664	313.682
5421	2.2066	13.1081	28.6117
5422	2.2067	-3.012	89.9839
5423	2.2071	10.9868	200.364
5424	2.2071	-4.8877	223.954
5425	2.2072	9.0916	262.563
5426	2.2074	4.0828	131.687
5427	2.2075	-3.2403	113.978
5428	2.2075	-3.7588	5.8605
5429	2.2081	-3.1314	107.516
5430	2.2081	-4.0528	91.8509
5431	2.2082	9.2302	128.522
5432	2.2083	-2.738	120.195
5433	2.2083	-3.5795	255.314
5434	2.2084	6.4847	122.687
5435	2.2088	-1.5176	315.449
5436	2.2088	12.1011	347.275
5437	2.2088	-3.2569	278.108
5438	2.2089	-2.9593	23.5885
5439	2.2091	-2.7937	10.332
5440	2.2093	12.8384	281.242
5441	2.2094	-2.0764	121.011
5442	2.2096	-2.808	351.622
5443	2.2096	-3.5089	92.5741
5444	2.2098	-3.1804	292.556
5445	2.2101	-2.7377	177.172
5446	2.2102	-2.9696	253.392
5447	2.2102	7.8357	257.37
5448	2.2102	-2.7672	319.153
5449	2.2106	-2.7376	212.189
5450	2.2106	-3.8424	283.053
5451	2.2112	8.5774	243.745
5452	2.2113	-2.9393	78.075
5453	2.2113	-2.8335	166.341
5454	2.2118	7.3504	296.839
5455	2.2121	3.53	262.28
5456	2.2121	-3.2526	330.975
5457	2.2122	-2.8481	272.634
5458	2.2128	14.3828	139.226
5459	2.2129	-3.3118	102.669
5460	2.213	-2.8181	42.747
5461	2.2133	-3.1685	6.8378

5462	2.2133	-4.1147	277.81
5463	2.2134	3.3505	312.724
5464	2.2134	-2.8001	147.583
5465	2.2137	-3.2874	36.0512
5466	2.2139	-3.6508	343.973
5467	2.2141	-3.9311	248.431
5468	2.2141	-2.8542	303.905
5469	2.2143	-2.9961	339.62
5470	2.2146	-3.3179	53.8736
5471	2.2147	11.7261	132.988
5472	2.215	-3.4865	59.137
5473	2.2151	13.892	132.372
5474	2.2152	-3.6846	191.913
5475	2.2159	-3.2146	72.1544
5476	2.2159	-5.0114	122.532
5477	2.2161	-2.8658	24.3028
5478	2.2166	-2.853	113.036
5479	2.2167	-3.7645	1.0539
5480	2.2176	8.4624	184.608
5481	2.2176	-2.943	183.478
5482	2.2176	-3.8646	160.146
5483	2.2178	-4.0103	63.9735
5484	2.2181	-2.9696	280.993
5485	2.2186	-2.7491	345.693
5486	2.2187	9.4695	102.936
5487	2.2199	10.6944	132.666
5488	2.22	7.6604	246.866
5489	2.2202	-3.51	260.106
5490	2.2203	-3.2208	312.996
5491	2.2205	-2.7832	40.5986
5492	2.2207	-2.8792	252.173
5493	2.2209	-3.5358	221.544
5494	2.2212	17.3146	26.1826
5495	2.2213	-3.3597	67.2291
5496	2.2213	-3.3166	92.2838
5497	2.2215	-2.9503	34.6767
5498	2.2216	10.4431	49.4839
5499	2.2218	7.7855	163.356
5500	2.2219	-3.1877	352.767
5501	2.222	11.4654	89.1128
5502	2.222	11.8442	196.654
5503	2.2221	-2.9581	167.018
5504	2.2221	-3.0162	273.865
5505	2.2222	-3.1983	336.175
5506	2.2223	15.2136	144.314
5507	2.2226	-3.7495	10.3018
5508	2.2226	-2.7421	137.895
5509	2.2226	-2.797	138.161

5510	2.2228	-2.9106	338.736
5511	2.2229	-3.7888	157.023
5512	2.2229	-2.9895	224.995
5513	2.2229	-3.8495	210.362
5514	2.2229	5.0962	173.689
5515	2.2231	-2.7271	236.586
5516	2.2234	-2.8323	258.408
5517	2.2236	-2.7685	311.823
5518	2.2237	-2.9383	336.577
5519	2.2241	22.9371	287.573
5520	2.2242	-2.6861	291.395
5521	2.2242	-2.7966	275.153
5522	2.2242	-3.6518	217.62
5523	2.2243	-3.8776	335.882
5524	2.2243	-3.453	229.816
5525	2.2243	-2.799	315.978
5526	2.2245	-2.7826	346.266
5527	2.2248	6.2851	261.011
5528	2.2249	-2.9682	320.882
5529	2.225	12.7535	345.735
5530	2.225	-2.8553	169.317
5531	2.2251	-3.1547	242.236
5532	2.2257	-3.9375	178.739
5533	2.2258	10.9464	339.292
5534	2.2261	-3.0257	337.061
5535	2.2262	-4.0656	291.09
5536	2.2263	-4.0739	127.058
5537	2.2264	-3.8441	112.591
5538	2.2265	-2.8596	333.927
5539	2.2267	-3.643	203.518
5540	2.2269	9.5543	223.232
5541	2.2272	-4.0508	92.9517
5542	2.2276	-2.7143	246.491
5543	2.2277	-3.9137	331.138
5544	2.2277	23.1571	282.93
5545	2.2282	9.2716	289.176
5546	2.2283	14.9134	65.6348
5547	2.2283	-2.8113	275.844
5548	2.2284	12.1728	131.238
5549	2.2284	18.9224	36.218
5550	2.2286	-3.555	103.98
5551	2.2286	-4.0548	175.225
5552	2.2291	14.5191	47.5464
5553	2.2293	14.6575	171.751
5554	2.2293	-2.8271	287.927
5555	2.2294	16.8724	223.089
5556	2.2299	-2.8022	307.576
5557	2.23	-2.804	98.1555

5558	2.23	4.3954	277.952
5559	2.2301	-2.7421	102.293
5560	2.2302	-2.7392	115.252
5561	2.2307	21.3212	313.567
5562	2.2314	11.8467	135.067
5563	2.2316	-2.8203	280.869
5564	2.2316	-2.7563	136.541
5565	2.2317	-2.8109	32.1855
5566	2.2318	14.7525	93.512
5567	2.2318	-2.9398	87.6312
5568	2.2318	-2.8146	53.7692
5569	2.2321	13.2349	149.419
5570	2.2323	6.441	325.739
5571	2.2323	-3.018	90.4318
5572	2.2323	-4.3243	203.65
5573	2.2327	-4.2312	134.533
5574	2.2327	11.0021	167.092
5575	2.2329	-2.7999	30.0831
5576	2.2334	-3.3548	334.123
5577	2.2336	-2.868	295.666
5578	2.2336	12.8674	126.785
5579	2.2337	9.548	345.583
5580	2.2338	-3.1693	320.975
5581	2.2338	-3.8665	233.763
5582	2.2339	-3.1866	106.921
5583	2.2339	-2.877	139.174
5584	2.2342	-2.8818	145.196
5585	2.2344	-2.6631	343.288
5586	2.2346	-2.7662	199.957
5587	2.2347	-2.7832	139.416
5588	2.2363	-3.4467	67.2643
5589	2.2365	-2.8748	131.08
5590	2.2366	-2.7929	27.9404
5591	2.2366	6.4793	288.149
5592	2.237	8.2478	191.793
5593	2.2373	15.5635	66.365
5594	2.2375	-4.6213	333.437
5595	2.2375	9.7683	275.012
5596	2.2376	9.3057	295.149
5597	2.2381	-2.9282	252.917
5598	2.2383	9.6182	235.495
5599	2.2383	11.1415	307.44
5600	2.2385	-4.0822	285.662
5601	2.2389	4.2116	44.6545
5602	2.2389	-3.2352	331.995
5603	2.239	12.2372	274.256
5604	2.239	-2.8225	116.117
5605	2.2391	18.6013	206.973

5606	2.2396	-2.8788	316.711
5607	2.2398	18.2432	85.5962
5608	2.24	18.1737	240.887
5609	2.2401	-2.9012	10.3062
5610	2.2402	17.3444	244.806
5611	2.2411	-2.7616	337.344
5612	2.2413	-2.9135	288.934
5613	2.2418	-3.8805	32.4064
5614	2.2419	-3.1122	233.753
5615	2.242	-3.438	103.203
5616	2.2423	-3.4386	68.196
5617	2.2424	8.1766	281.682
5618	2.2426	-2.8693	45.9251
5619	2.2427	-3.1013	341.448
5620	2.2427	-3.4375	175.762
5621	2.2432	12.2913	35.2739
5622	2.2433	11.1178	33.6496
5623	2.2434	-2.9288	91.6945
5624	2.2437	-4.01	85.2052
5625	2.2437	-4.1574	21.5761
5626	2.2438	-3.758	91.5366
5627	2.2443	-3.5344	345.891
5628	2.2445	9.9386	239.688
5629	2.2445	-4.2669	277.602
5630	2.2446	10.6834	185.717
5631	2.2447	-3.9825	334.211
5632	2.2448	-4.5284	335.439
5633	2.2449	-4.0731	269.494
5634	2.245	9.3419	261.706
5635	2.245	12.0458	28.286
5636	2.2452	5.248	316.837
5637	2.2462	-3.6789	102.867
5638	2.2462	12.056	240.644
5639	2.2464	-2.8119	122.311
5640	2.2465	-2.7949	271.499
5641	2.247	-2.9929	20.9819
5642	2.247	-2.8001	216.893
5643	2.247	-7.5255	85.8545
5644	2.2473	7.0553	57.5574
5645	2.2476	-2.9927	302.543
5646	2.2477	-2.9924	173.758
5647	2.2477	-2.7537	174.024
5648	2.2477	20.5091	308.565
5649	2.2483	-3.1237	18.4379
5650	2.2483	14.1451	47.1244
5651	2.249	-2.6611	282.663
5652	2.2491	-3.9202	267.507
5653	2.2498	17.1973	236.336

5654	2.2501	15.6065	35.957
5655	2.2501	-3.564	265.651
5656	2.2502	13.1079	69.8748
5657	2.2503	12.8155	108.773
5658	2.2507	11.8304	324.713
5659	2.2507	15.066	19.1686
5660	2.2509	10.1085	159.618
5661	2.2512	27.8683	311.676
5662	2.2516	-4.4092	348.871
5663	2.2517	9.8995	337.45
5664	2.2518	-2.9623	350.797
5665	2.2521	-2.8393	67.8257
5666	2.2522	19.9329	203.421
5667	2.2524	8.1792	237.178
5668	2.2525	12.7306	109.927
5669	2.2529	-2.7661	202.796
5670	2.253	13.134	241.746
5671	2.2533	12.3525	89.2762
5672	2.2538	-2.8221	54.658
5673	2.254	14.6247	76.0629
5674	2.2541	-3.5316	290.001
5675	2.2545	-2.7591	36.2277
5676	2.2546	-2.8506	306.048
5677	2.2547	-3.2004	342.173
5678	2.2547	-4.2778	3.5117
5679	2.2548	7.2075	153.82
5680	2.255	11.9919	196.489
5681	2.2551	8.425	292.798
5682	2.2556	-2.9757	293.571
5683	2.256	-3.1078	220.758
5684	2.2561	-3.7854	146.272
5685	2.2561	14.2365	28.3735
5686	2.2562	-5.0307	141.16
5687	2.2563	-5.3811	130.269
5688	2.2563	-3.1818	104.468
5689	2.2567	7.6007	101.874
5690	2.2567	-2.8658	258.791
5691	2.2569	-4.9674	155.832
5692	2.2569	-2.9043	269.404
5693	2.257	-2.8077	322.332
5694	2.2571	-2.9713	30.8198
5695	2.2571	-3.1963	110.673
5696	2.2572	16.8219	128.433
5697	2.2573	-3.9644	326.859
5698	2.2574	-4.0556	189.102
5699	2.2574	9.7408	147.486
5700	2.2577	-2.8435	6.6733
5701	2.2577	12.9577	208.404

5702	2.2578	-4.0124	333.775
5703	2.2579	-2.9076	262.512
5704	2.2579	9.3855	335.939
5705	2.2581	-3.7009	110.295
5706	2.2584	19.8357	309.01
5707	2.2585	-2.8621	302.902
5708	2.2587	-2.7085	208.955
5709	2.2589	-2.7756	70.6982
5710	2.259	12.8016	132.572
5711	2.2598	9.0096	354.893
5712	2.2599	14.3079	218.662
5713	2.26	-3.9742	10.9974
5714	2.2608	-3.0444	101.158
5715	2.261	-3.3762	278.278
5716	2.2611	12.3777	317.14
5717	2.2614	-3.6449	64.3299
5718	2.2618	-2.7252	186.59
5719	2.262	16.662	262.932
5720	2.2624	-3.4744	75.5909
5721	2.2625	-4.1785	211.231
5722	2.2626	-3.1177	332.162
5723	2.2627	13.0797	107.205
5724	2.263	-3.0087	133.928
5725	2.263	-2.9251	5.694
5726	2.2631	-3.1301	85.0775
5727	2.2631	-2.3984	34.4109
5728	2.2632	-2.9924	358.171
5729	2.2632	18.2116	30.4003
5730	2.2633	10.8046	319.666
5731	2.2634	-2.9634	153.212
5732	2.2634	-2.9458	255.904
5733	2.2634	-3.4343	146.337
5734	2.2638	10.1512	208.42
5735	2.2639	-3.3642	212.934
5736	2.264	-2.8489	314.786
5737	2.264	10.6853	320.944
5738	2.2641	-2.9465	154.046
5739	2.2642	12.0882	197.579
5740	2.2643	-3.7552	249.128
5741	2.2643	-2.8719	144.839
5742	2.2648	12.7299	245.891
5743	2.2649	-2.96	318.968
5744	2.2649	15.8793	270.899
5745	2.265	-2.9278	117.233
5746	2.2652	-2.7841	215.56
5747	2.2653	-3.3213	264.461
5748	2.2653	-2.7506	153.609
5749	2.2655	11.3978	235.648

5750	2.2655	13.3843	192.189
5751	2.2655	-2.8415	67.3204
5752	2.2657	-2.9015	48.7574
5753	2.2657	-2.7572	141.564
5754	2.2661	-2.8833	67.5336
5755	2.2661	9.1099	311.606
5756	2.2661	-2.8782	23.4292
5757	2.2662	-2.974	10.5446
5758	2.2663	14.9821	245.928
5759	2.2664	10.8217	104.368
5760	2.2664	13.935	319.502
5761	2.2665	16.8133	10.5855
5762	2.267	11.535	115.717
5763	2.2671	-2.9385	85.941
5764	2.2671	21.0578	210.674
5765	2.2674	9.2534	17.1619
5766	2.2678	-3.4036	239.709
5767	2.2679	-2.6814	333.779
5768	2.2681	-2.952	10.6376
5769	2.2682	11.1335	337.6
5770	2.2683	-2.819	200.135
5771	2.2683	-3.6202	133.285
5772	2.2683	-3.9532	231.67
5773	2.2685	6.4847	50.8118
5774	2.2685	-4.5234	245.146
5775	2.2687	-2.8932	347.079
5776	2.2687	9.3126	219.607
5777	2.2687	-2.9449	309.56
5778	2.2688	-2.8536	214.748
5779	2.269	-4.0991	6.2809
5780	2.2695	-2.6961	332.007
5781	2.2696	-2.8227	117.432
5782	2.2697	-2.8429	84.1788
5783	2.2699	-3.0358	36.211
5784	2.2704	13.6544	203.615
5785	2.2705	-2.9363	124.242
5786	2.2705	10.8732	83.831
5787	2.2708	-3.8739	252.763
5788	2.2708	-2.6771	354.083
5789	2.2708	-2.849	68.7755
5790	2.271	-3.6626	343.166
5791	2.271	-2.9508	125.631
5792	2.2713	-3.3448	189.201
5793	2.2713	14.3859	179.729
5794	2.2716	-4.004	332.531
5795	2.2717	13.2562	342.083
5796	2.2719	14.5986	53.3171
5797	2.2721	13.2433	293.082

5798	2.2722	11.5904	332.138
5799	2.2725	-4.2279	315.073
5800	2.2726	-2.8989	324.073
5801	2.2729	-4.2737	44.4606
5802	2.2732	-2.7094	34.6714
5803	2.2733	-4.033	222.383
5804	2.2734	-3.0885	318.783
5805	2.2734	-2.8997	80.7514
5806	2.2734	-2.7655	95.5324
5807	2.2735	-2.9197	359.496
5808	2.2735	-3.9301	16.6092
5809	2.2735	11.5732	60.5717
5810	2.2737	-2.8413	144.944
5811	2.274	-3.0514	95.0185
5812	2.2743	12.5781	153.598
5813	2.2744	21.3116	249.34
5814	2.2744	-3.8001	205.3
5815	2.275	15.5144	44.5541
5816	2.2758	-2.8015	284.333
5817	2.2759	-3.5206	320.027
5818	2.276	-2.9361	339.985
5819	2.2761	-2.9548	42.2282
5820	2.2763	19.3476	145.573
5821	2.2767	-2.8133	21.0394
5822	2.2767	-2.8243	171.223
5823	2.2769	12.2874	322.358
5824	2.2769	-2.8143	158.426
5825	2.2771	-5.0978	276.004
5826	2.2772	-2.9089	336.602
5827	2.2772	-3.3568	182.236
5828	2.2776	14.4564	231.311
5829	2.2779	-3.1457	234.165
5830	2.2779	12.4325	335.414
5831	2.2781	-2.8663	327.616
5832	2.2784	-3.0761	59.2386
5833	2.2785	11.3027	346.524
5834	2.2785	-3.8868	144.289
5835	2.279	13.8031	334.502
5836	2.2796	17.3798	208.562
5837	2.2797	-3.114	100.511
5838	2.28	-3.2554	190.474
5839	2.2806	-2.8321	12.6536
5840	2.2806	-2.8425	206.734
5841	2.2806	-3.1774	329.204
5842	2.2809	20.4354	264.436
5843	2.281	-2.9523	170.038
5844	2.281	10.9787	101.158
5845	2.2812	-2.751	346.796

5846	2.2815	15.8534	345.869
5847	2.2816	-2.7278	44.8335
5848	2.2821	-3.056	71.691
5849	2.2821	-2.7747	28.8589
5850	2.2822	-2.6744	156.218
5851	2.2823	22.2476	44.5684
5852	2.2827	12.8881	236.123
5853	2.283	-3.0154	74.2544
5854	2.2831	-2.9062	26.3338
5855	2.2834	-2.8432	182.745
5856	2.2837	-4.254	19.8183
5857	2.2837	-3.6697	324.322
5858	2.2839	-3.9371	338.882
5859	2.2841	15.9435	146.112
5860	2.2843	-2.868	253.424
5861	2.2846	-4.212	148.775
5862	2.2847	-2.9629	66.5632
5863	2.2849	-2.8935	268.432
5864	2.2849	-2.7636	296.389
5865	2.2851	21.6371	313.089
5866	2.2853	-3.9529	97.8669
5867	2.2854	-2.9269	246.227
5868	2.2855	14.7015	73.9092
5869	2.2856	11.432	151.206
5870	2.2857	-3.2189	132.285
5871	2.2863	5.3786	270.806
5872	2.2864	-2.7429	179.183
5873	2.2866	-3.8331	123.001
5874	2.2868	14.108	47.5692
5875	2.2868	-2.7025	254.693
5876	2.2874	-2.6861	319.394
5877	2.2878	7.6257	358.056
5878	2.2879	-2.8731	317.489
5879	2.2881	13.7635	76.031
5880	2.2882	-3.6149	333.21
5881	2.2882	-2.8422	114.59
5882	2.2884	6.3325	275.39
5883	2.2885	11.9284	17.6866
5884	2.2886	10.9747	348.543
5885	2.2888	-3.2473	340.217
5886	2.2888	12.4065	114.073
5887	2.2891	-3.7521	349.522
5888	2.2891	-2.9669	279.113
5889	2.2892	12.0208	264.107
5890	2.2892	-2.7852	300.827
5891	2.2892	15.1613	203.142
5892	2.2897	-2.9546	133.785
5893	2.2898	-3.543	340.435

5894	2.2898	-2.9034	167.911
5895	2.2899	-3.3234	163.932
5896	2.2899	-3.8823	179.071
5897	2.2901	-3.3586	182.856
5898	2.2905	-2.9632	138.821
5899	2.2906	-2.7816	132.723
5900	2.2907	-3.4981	144.863
5901	2.2908	16.395	83.7988
5902	2.2913	-2.9711	180.791
5903	2.2917	-3.8703	209.762
5904	2.2917	11.9324	34.692
5905	2.2917	14.6879	23.4052
5906	2.2919	-2.6761	131.547
5907	2.2919	-2.9297	167.435
5908	2.2921	19.5632	19.1387
5909	2.2921	-3.115	228.227
5910	2.2922	-2.7971	25.0145
5911	2.2924	-3.3362	74.209
5912	2.2924	-2.8682	346.83
5913	2.2929	-3.8455	186.198
5914	2.2931	-3.7606	284.201
5915	2.2932	-2.7809	263.196
5916	2.2932	-2.882	303.417
5917	2.2933	-3.3021	146.853
5918	2.2934	-2.7545	280.049
5919	2.2935	-4.0705	55.0985
5920	2.2937	-2.9069	107.489
5921	2.2937	-2.9192	172.444
5922	2.294	17.29	40.0914
5923	2.2943	13.8042	235.037
5924	2.2945	-3.2878	318.674
5925	2.2947	-2.7601	47.6851
5926	2.2948	-4.058	300.497
5927	2.2948	-3.333	337.983
5928	2.2949	-2.7874	152.709
5929	2.2949	-3.3108	117.551
5930	2.295	11.9317	212.086
5931	2.295	-2.8549	319.188
5932	2.2953	21.3362	337.27
5933	2.2953	7.9591	243.445
5934	2.2954	-2.9195	350.971
5935	2.2954	-2.7822	19.3658
5936	2.2954	-2.8423	69.4094
5937	2.2956	-4.6047	127.029
5938	2.2958	15.2875	79.738
5939	2.2958	19.0976	22.1088
5940	2.2959	13.2356	78.8053
5941	2.296	21.3037	209.51

5942	2.2961	-4.0238	160.188
5943	2.2962	-2.8685	340.406
5944	2.2964	12.7052	132.23
5945	2.2964	7.2331	175.701
5946	2.2964	-4.3428	295.115
5947	2.2965	8.6669	134.585
5948	2.2967	-2.7049	99.1402
5949	2.2969	-3.682	355.48
5950	2.2969	17.0366	75.7185
5951	2.2969	13.1453	276.705
5952	2.2969	-2.8054	167.208
5953	2.297	10.6142	178.886
5954	2.297	-2.9503	322.157
5955	2.2974	-3.7982	23.7958
5956	2.2974	-2.9412	295.713
5957	2.2975	-2.7697	94.1241
5958	2.2976	-2.9784	265.828
5959	2.298	-3.183	329.566
5960	2.298	23.6098	66.3428
5961	2.298	-2.9334	90.5584
5962	2.298	20.7981	314.344
5963	2.2983	-2.6729	341.954
5964	2.2987	-2.9959	110.266
5965	2.2987	-2.8739	166.815
5966	2.2993	14.0825	81.9081
5967	2.2993	11.3704	211.128
5968	2.2995	-2.8564	148.816
5969	2.2997	-2.8439	348.341
5970	2.2998	-2.7759	20.4703
5971	2.2998	-2.9892	324.071
5972	2.2999	-2.7941	340.988
5973	2.2999	15.2867	238.937
5974	2.3001	13.0601	279.597
5975	2.3005	11.3859	337.473
5976	2.3005	-2.9783	297.651
5977	2.3006	-3.19	7.3569
5978	2.3009	15.436	256.328
5979	2.3009	-4.1431	142.787
5980	2.3009	-2.8627	318.245
5981	2.301	16.7573	21.4141
5982	2.3011	-3.4324	175.536
5983	2.3012	10.9403	112.936
5984	2.3017	-2.8498	160.853
5985	2.3018	19.2148	241.229
5986	2.3018	-2.8675	56.4389
5987	2.3019	11.5671	321.86
5988	2.3023	25.6705	310.68
5989	2.3026	-2.9418	20.8088

5990	2.3026	10.9993	183.985
5991	2.3028	-3.6147	70.2987
5992	2.3029	-2.9708	75.8606
5993	2.3031	9.0644	169.629
5994	2.3033	-3.8019	119.633
5995	2.3035	-3.5472	153.266
5996	2.3035	-3.3359	20.9784
5997	2.3036	29.1069	240.574
5998	2.3038	9.1464	86.9424
5999	2.3039	14.2897	4.62
6000	2.3039	16.0391	244.927
6001	2.3041	-3.8656	323.715
6002	2.3041	-2.7338	19.2729
6003	2.3044	17.5557	334.841
6004	2.3047	15.7565	222.628
6005	2.3048	-2.7812	345.507
6006	2.3049	-3.1691	344.497
6007	2.3049	-4.1801	143.483
6008	2.305	-3.0431	231.658
6009	2.3051	-2.8014	315.306
6010	2.3053	17.6187	156.406
6011	2.3056	-2.7855	265.494
6012	2.306	-3.3395	327.14
6013	2.3065	-3.9448	14.0233
6014	2.3066	-2.9157	344.148
6015	2.3066	-4.3308	28.4459
6016	2.3067	-3.8038	215.399
6017	2.3068	18.2484	346.016
6018	2.3069	-4.4981	2.1142
6019	2.307	-2.9064	68.9595
6020	2.3072	-3.5725	178.444
6021	2.3073	-2.8043	212.427
6022	2.3073	-3.8662	203.598
6023	2.3073	-2.8969	269.61
6024	2.308	-3.312	282.29
6025	2.3081	-3.6553	306.34
6026	2.3082	-3.2881	335.238
6027	2.3085	18.468	66.0754
6028	2.3085	-2.7831	266.985
6029	2.3085	-3.2321	43.1881
6030	2.3087	-2.9675	153.91
6031	2.3088	-4.2733	292.393
6032	2.3088	-2.8431	195.285
6033	2.3092	-2.8437	64.9697
6034	2.3094	15.8641	297.425
6035	2.3095	6.5714	156.125
6036	2.3098	-2.7802	92.0908
6037	2.3101	-3.8826	30.6053

6038	2.3101	-4.1064	295.612
6039	2.3102	20.0893	200.754
6040	2.3105	-2.9856	37.2235
6041	2.3108	18.5012	169.343
6042	2.3111	-2.864	187.527
6043	2.3112	-2.7529	346.641
6044	2.3112	-2.894	149.218
6045	2.3113	-3.4729	124.469
6046	2.3115	18.9328	176.606
6047	2.3116	15.4741	211.703
6048	2.3118	-2.9028	194.977
6049	2.3119	13.4767	155.389
6050	2.312	-2.7684	251.799
6051	2.3122	-4.2019	262.104
6052	2.3122	12.3801	198.342
6053	2.3125	13.0302	332.618
6054	2.3128	-3.1228	233.673
6055	2.3129	17.933	10.382
6056	2.3131	-2.9162	109.221
6057	2.3133	-2.9858	24.0164
6058	2.3133	17.5976	130.793
6059	2.3133	-3.0368	239.398
6060	2.314	18.09	197.638
6061	2.3141	16.3731	235.487
6062	2.3141	10.443	130.655
6063	2.3144	16.4333	354.309
6064	2.3146	-3.9744	67.25
6065	2.3147	-2.834	345.71
6066	2.315	-2.7964	39.71
6067	2.3152	-3.853	71.6093
6068	2.3152	17.0537	113.389
6069	2.3153	18.2087	315.094
6070	2.3155	4.2455	223.194
6071	2.3157	7.3552	357.266
6072	2.3157	-2.7199	63.5905
6073	2.3157	-4.1946	267.981
6074	2.3158	-2.8175	279.59
6075	2.3162	-3.9774	85.5054
6076	2.3163	9.6945	278.01
6077	2.3163	-2.7608	121.301
6078	2.3165	6.6238	126.761
6079	2.3167	14.9455	55.3036
6080	2.3171	-2.8736	87.1223
6081	2.3176	16.3085	240.774
6082	2.3182	-3.1532	266.396
6083	2.3183	-4.2474	173.509
6084	2.3186	-2.9378	89.9346
6085	2.3188	17.6563	328.94

6086	2.3189	-3.3748	276.611
6087	2.3193	15.7932	349.107
6088	2.3194	-4.2747	357.742
6089	2.3195	-4.2667	208.636
6090	2.3197	12.7421	162.762
6091	2.3197	13.4027	210.274
6092	2.3199	11.2365	140.403
6093	2.3205	10.5818	31.1618
6094	2.3206	-2.8075	53.6011
6095	2.3206	-3.884	216.746
6096	2.3207	12.2901	195.376
6097	2.3207	-3.0106	318.527
6098	2.3207	-2.8735	266.844
6099	2.3208	14.3688	175.58
6100	2.3208	17.4919	302.756
6101	2.3209	-2.7667	332.745
6102	2.321	-2.9389	242.114
6103	2.3213	-3.5033	14.0052
6104	2.3214	9.8636	276.62
6105	2.3214	-3.501	161.236
6106	2.3216	9.0269	265.576
6107	2.3217	-3.6434	256.669
6108	2.3218	-2.9253	93.324
6109	2.3222	-4.5117	293.972
6110	2.3223	-4.0205	275.397
6111	2.3225	-3.4428	339.167
6112	2.3226	-2.9064	356.272
6113	2.3236	-2.9409	341.978
6114	2.3236	7.4947	241.803
6115	2.3237	17.4466	129.135
6116	2.3237	-3.0331	127.022
6117	2.3237	-2.701	102.009
6118	2.3238	17.3927	25.6055
6119	2.324	-2.9282	29.9892
6120	2.324	19.5106	157.211
6121	2.3245	2.0604	20.0575
6122	2.3246	16.4818	211.785
6123	2.3249	-0.1653	249.621
6124	2.3254	13.9668	311.558
6125	2.3254	-2.9341	200.876
6126	2.3254	18.0174	125.411
6127	2.3255	-2.8895	108.85
6128	2.3256	14.3431	53.959
6129	2.3258	-2.8572	333.692
6130	2.3259	13.3736	249.783
6131	2.3259	-2.8071	312.276
6132	2.326	9.1926	252.927
6133	2.3261	-4.1701	242.168

6134	2.3262	-4.346	333.485
6135	2.3262	11.5334	186.935
6136	2.3263	17.1245	27.7042
6137	2.3264	18.7819	341.071
6138	2.3265	-2.7066	122.529
6139	2.3265	15.0831	240.153
6140	2.3265	-2.8987	167.103
6141	2.3266	-3.7655	158.488
6142	2.3266	8.1005	197.651
6143	2.3267	-2.8659	311.958
6144	2.3268	13.8141	163.34
6145	2.327	21.7325	33.8922
6146	2.327	-4.201	228.113
6147	2.327	-4.0493	309.272
6148	2.3271	-2.6879	321.36
6149	2.3272	-3.193	308.782
6150	2.3273	7.8579	213.961
6151	2.3275	-2.7884	286.079
6152	2.3276	-3.341	24.4868
6153	2.3277	-2.9947	263.005
6154	2.3277	12.6248	288.614
6155	2.3278	-2.9768	318.03
6156	2.3281	13.5286	214.023
6157	2.3282	-3.2751	65.6669
6158	2.3284	-3.5634	81.2901
6159	2.3284	-3.1716	57.8936
6160	2.3287	-4.0393	285.348
6161	2.3287	-2.8504	172.136
6162	2.3289	-4.0472	160.85
6163	2.329	17.673	57.0107
6164	2.3294	11.0417	242.513
6165	2.3296	12.0368	126.299
6166	2.3296	-2.8603	118.5
6167	2.3298	-3.6458	330.02
6168	2.3299	10.0408	204.432
6169	2.33	-3.5088	348.493
6170	2.3302	17.2184	280.229
6171	2.3302	-2.9319	72.9217
6172	2.3303	14.4879	267.377
6173	2.3305	-3.7654	166.002
6174	2.3307	-2.8472	343.125
6175	2.3308	-2.9524	328.551
6176	2.3309	11.9757	23.0619
6177	2.3314	-2.9088	18.8856
6178	2.3314	15.2696	264.734
6179	2.3315	-2.7737	24.92
6180	2.3316	-2.8773	350.644
6181	2.3317	-2.8649	43.5981

6182	2.3318	-2.9488	16.3265
6183	2.3319	22.9164	215.234
6184	2.332	20.6705	308.737
6185	2.3322	-3.3024	316.89
6186	2.3322	-3.4848	125.951
6187	2.3323	-3.249	170.509
6188	2.3323	-3.2353	220.997
6189	2.3325	14.6743	186.467
6190	2.3329	10.5038	58.354
6191	2.3334	-2.9805	312.725
6192	2.3339	-2.9416	45.8894
6193	2.3342	3.1958	166.57
6194	2.3342	-2.8171	320.114
6195	2.3343	-3.3223	319.303
6196	2.3344	-2.7808	37.942
6197	2.3347	9.0148	171.655
6198	2.3349	11.7245	278.443
6199	2.3356	-3.1418	177.03
6200	2.3356	-2.9147	349.25
6201	2.3359	17.6154	305.085
6202	2.3359	-3.5255	159.242
6203	2.336	-3.8452	63.4658
6204	2.3365	11.676	262.084
6205	2.3367	12.4613	354.052
6206	2.3367	14.8262	337.853
6207	2.3369	14.0273	172.695
6208	2.337	16.1391	334.416
6209	2.3371	10.5637	77.184
6210	2.3374	-3.0668	338.029
6211	2.3375	-2.9577	2.9878
6212	2.3375	14.255	112.987
6213	2.3379	-2.8322	162.748
6214	2.338	-3.8386	333.129
6215	2.338	13.3187	58.847
6216	2.3381	18.8867	281.809
6217	2.3382	20.1222	208.726
6218	2.3388	-2.8003	25.6287
6219	2.3392	23.832	194.442
6220	2.3395	-3.6134	21.2694
6221	2.3395	17.7589	253.507
6222	2.3395	-4.1885	237.153
6223	2.3395	-3.9192	6.4707
6224	2.3397	-2.9042	23.9813
6225	2.3397	-4.658	75.653
6226	2.3399	17.5994	160.62
6227	2.34	-2.7987	152.734
6228	2.34	-3.3488	209.902
6229	2.3401	-3.2152	301.474

6230	2.3403	-2.6933	319.251
6231	2.3403	-2.6255	273.951
6232	2.3404	-3.1852	135.725
6233	2.3405	26.3382	135.678
6234	2.3405	-2.9803	33.9491
6235	2.3407	18.1263	17.5121
6236	2.3409	-3.4942	48.2463
6237	2.341	-4.5306	304.12
6238	2.3414	20.4586	48.3423
6239	2.3417	-3.3702	105.062
6240	2.3423	-2.7962	328.686
6241	2.3426	16.5103	309.409
6242	2.3427	17.099	225.299
6243	2.3429	-3.1715	67.1919
6244	2.343	-2.8145	186.755
6245	2.343	8.5409	239.232
6246	2.3432	-2.7838	121.177
6247	2.3433	-3.175	319.995
6248	2.3433	-3.2427	8.9377
6249	2.3434	-2.8912	281.374
6250	2.3436	-2.9776	354.003
6251	2.3438	16.0931	83.2424
6252	2.3438	-3.1078	216.332
6253	2.344	-2.7226	337.248
6254	2.344	15.2209	249.888
6255	2.3442	-2.8813	216.923
6256	2.3443	-2.9855	135.846
6257	2.3449	-3.2592	331.175
6258	2.3451	16.4769	167.237
6259	2.3451	-4.3349	254.879
6260	2.3451	-2.9344	119.814
6261	2.3453	29.845	67.6421
6262	2.3456	-2.8353	179.683
6263	2.3457	19.6366	235.852
6264	2.3459	-3.397	72.2951
6265	2.346	-2.938	3.5841
6266	2.3462	-2.726	171.506
6267	2.3463	19.9693	300.035
6268	2.3463	20.0905	289.328
6269	2.3466	12.7912	17.3826
6270	2.3466	14.9718	272.5
6271	2.3468	-3.248	185.558
6272	2.3472	-2.7439	84.8671
6273	2.3472	-2.7697	282.567
6274	2.3474	-3.445	322.044
6275	2.3477	-3.1284	193.696
6276	2.3483	-2.7702	110.441
6277	2.3484	15.275	232.141

6278	2.3484	-4.1841	129.197
6279	2.3485	-3.6372	168.325
6280	2.3486	13.4637	158.291
6281	2.3486	-3.0164	260.87
6282	2.3487	-2.752	78.5546
6283	2.3487	15.8326	227.777
6284	2.3491	-4.3919	263.124
6285	2.3492	9.6409	102.289
6286	2.3493	-3.2543	258.711
6287	2.3493	14.6037	48.8979
6288	2.3494	-3.3072	193.931
6289	2.3496	-4.1347	310.087
6290	2.3497	16.1592	282.879
6291	2.3497	12.7737	252.467
6292	2.3497	26.5243	289.473
6293	2.3499	14.472	13.5764
6294	2.35	-2.7775	229.402
6295	2.3501	-3.2062	96.1736
6296	2.3501	-2.8688	264.101
6297	2.3502	-2.7318	216.368
6298	2.3502	-2.7224	108.363
6299	2.3503	13.1272	235.104
6300	2.3506	-3.2484	226.144
6301	2.3514	-2.7828	158.634
6302	2.3515	-2.808	111.259
6303	2.3515	-3.4168	281.281
6304	2.3516	-2.8745	256.969
6305	2.3517	-3.5679	207.755
6306	2.3517	17.1265	290.113
6307	2.3519	-3.227	231.992
6308	2.3521	9.838	235.867
6309	2.3526	-2.8089	124.528
6310	2.353	3.0054	311.928
6311	2.3531	-3.8445	79.9575
6312	2.3532	12.9306	234.028
6313	2.3533	6.5312	323.497
6314	2.3533	-4.4256	317.035
6315	2.3535	24.6664	244.818
6316	2.3536	24.8284	314.062
6317	2.354	-2.9246	161.388
6318	2.3541	-3.1224	56.2075
6319	2.3543	-2.9042	275.342
6320	2.3544	-3.2327	153.864
6321	2.3544	19.9163	76.6142
6322	2.3545	-2.8798	262.941
6323	2.3545	19.451	209.577
6324	2.3548	-3.9282	170.025
6325	2.3548	14.1677	35.058

6326	2.3551	-4.0837	296.485
6327	2.3551	11.4207	143.246
6328	2.3551	9.0259	63.2441
6329	2.3551	18.8545	44.7855
6330	2.3553	-3.3911	246.125
6331	2.3555	27.3178	312.852
6332	2.3561	-2.9736	280.598
6333	2.3562	-2.8203	276.439
6334	2.3565	-2.9966	105.243
6335	2.3566	12.7192	330.892
6336	2.3568	-2.797	269.77
6337	2.357	-3.2878	308.82
6338	2.3573	-2.9153	345.96
6339	2.358	-2.8157	237.342
6340	2.3582	16.0426	250.792
6341	2.3586	2.4858	306.479
6342	2.3586	-3.7852	9.0635
6343	2.3587	-3.2142	334.969
6344	2.3587	-3.8108	97.5771
6345	2.3587	-2.9185	171.773
6346	2.3588	-3.9021	336.172
6347	2.3588	-3.4292	309.376
6348	2.359	-2.8246	127.684
6349	2.3596	-2.9958	17.8901
6350	2.3596	19.9603	204.441
6351	2.3601	-2.793	36.0266
6352	2.3602	-4.1217	23.1417
6353	2.3604	-3.8993	111.364
6354	2.3609	-3.029	220.24
6355	2.361	19.0953	297.506
6356	2.361	18.0705	347.759
6357	2.3612	17.422	244.764
6358	2.3613	-3.3754	347.128
6359	2.3614	-2.8887	229.928
6360	2.3614	11.3719	324.117
6361	2.3614	14.2734	150.941
6362	2.3615	14.2838	326.954
6363	2.3616	-4.2827	178.853
6364	2.3616	-4.1055	93.5589
6365	2.3616	-3.2476	295.791
6366	2.3617	-2.9768	112.839
6367	2.3617	-2.7816	113.975
6368	2.3618	19.7011	151.358
6369	2.3618	-2.8038	126.02
6370	2.3622	-2.8122	308.337
6371	2.3623	-4.0024	22.4218
6372	2.3623	13.7881	190.488
6373	2.3626	11.3131	273.589

6374	2.3627	-2.8249	267.828
6375	2.3629	14.5394	88.5641
6376	2.3633	-2.9877	19.9594
6377	2.3634	7.3667	298.529
6378	2.3635	11.4361	112.217
6379	2.3636	-2.8814	131.952
6380	2.3639	-2.7969	261.778
6381	2.364	-2.9886	264.012
6382	2.3641	-2.7554	316.334
6383	2.3642	-3.0995	258.52
6384	2.3642	13.5829	311.678
6385	2.3643	-2.9208	134.37
6386	2.3643	-2.7168	110.211
6387	2.3645	12.8577	287.929
6388	2.3646	12.2914	230.923
6389	2.3647	-4.3995	201.402
6390	2.3649	22.1009	287.02
6391	2.3649	24.8905	354.259
6392	2.365	-3.9353	194.253
6393	2.365	-2.9072	142.856
6394	2.3652	-2.7943	188.158
6395	2.3655	12.9993	333.67
6396	2.3658	10.741	140.789
6397	2.366	-3.6081	332.718
6398	2.3661	-2.8657	13.7509
6399	2.3661	-2.8014	167.046
6400	2.3666	-2.9012	349.605
6401	2.367	21.5406	252.12
6402	2.367	11.4874	119.239
6403	2.3671	-2.8411	294.354
6404	2.3674	-3.3616	135.663
6405	2.3679	15.1631	283.037
6406	2.3683	-3.9255	33.6098
6407	2.3684	15.5428	27.3145
6408	2.3685	-2.9476	70.5198
6409	2.3687	-3.3775	189.959
6410	2.369	16.0827	223.896
6411	2.3691	-2.9347	120.837
6412	2.3692	13.0017	271.782
6413	2.3695	-4.0044	321.148
6414	2.3697	-3.1611	301.297
6415	2.3698	-3.2962	114.468
6416	2.3699	17.7015	64.2557
6417	2.3699	18.2569	10.6171
6418	2.37	-3.4509	168.963
6419	2.37	9.6098	10.3188
6420	2.3703	13.1588	311.778
6421	2.3704	-4.252	235.505

6422	2.3705	23.2377	85.604
6423	2.3705	7.9223	19.6465
6424	2.3708	-2.872	251.995
6425	2.3712	-3.2033	134.617
6426	2.3713	21.014	290.154
6427	2.3713	-3.9676	208.898
6428	2.3715	19.571	119.466
6429	2.3716	-3.0099	155.974
6430	2.3718	-4.2001	114.774
6431	2.3718	-2.8826	238.905
6432	2.3718	18.1808	315.88
6433	2.372	-2.7779	8.1489
6434	2.3721	-2.7951	253.017
6435	2.3724	-2.7528	253.434
6436	2.3726	-2.9761	57.7639
6437	2.3728	12.7608	227.159
6438	2.373	18.8894	132.445
6439	2.373	13.4969	260.375
6440	2.3731	-3.0013	113.631
6441	2.3733	15.064	256.296
6442	2.3734	18.3613	157.633
6443	2.3735	19.1801	118.997
6444	2.3742	-2.8749	144.798
6445	2.3742	-3.8213	172.083
6446	2.3743	-2.9518	136.245
6447	2.3749	17.7175	140.642
6448	2.3754	-3.5565	309.943
6449	2.3756	-3.5891	293.309
6450	2.3756	-2.8184	113.286
6451	2.3756	-2.837	193.773
6452	2.3759	19.5538	86.0882
6453	2.3762	-3.2286	162.84
6454	2.3765	-3.019	106.661
6455	2.3766	19.7958	306.61
6456	2.3767	-2.9363	241.195
6457	2.377	-3.8584	154.108
6458	2.3771	16.6858	247.744
6459	2.3772	-3.548	195.904
6460	2.3773	21.4957	30.5755
6461	2.3777	15.0784	152.906
6462	2.3781	-3.1929	87.9359
6463	2.3781	-3.628	161.382
6464	2.3783	-2.7289	97.5806
6465	2.3786	12.3959	272.613
6466	2.3787	25.0685	15.2262
6467	2.3787	21.8001	203.738
6468	2.3787	13.995	314.988
6469	2.3791	20.0774	37.8044

6470	2.3791	-2.9258	100.908
6471	2.3793	-2.9486	24.9634
6472	2.3794	-3.0726	341.36
6473	2.3795	20.5309	310.968
6474	2.3795	-2.8407	327.668
6475	2.3796	-2.9917	119.18
6476	2.3796	17.8919	91.1759
6477	2.38	-2.7322	326.735
6478	2.3801	-2.973	156.565
6479	2.3802	21.9153	35.8911
6480	2.3802	19.3612	347.613
6481	2.3804	19.6318	237.515
6482	2.3806	-2.9223	177.851
6483	2.3812	24.3017	12.87
6484	2.3814	-3.6179	270.776
6485	2.3815	-3.0417	319.665
6486	2.3819	24.9804	233.257
6487	2.382	8.9386	225.476
6488	2.3822	-3.0891	253.094
6489	2.3823	-3.1278	181.276
6490	2.3824	-3.5523	256.225
6491	2.3825	-3.4244	259.753
6492	2.3825	15.7417	257.121
6493	2.3826	-2.8384	99.5803
6494	2.3826	-2.9385	297.084
6495	2.3832	-4.0218	293.662
6496	2.3833	23.0215	3.6245
6497	2.3834	-2.8782	206.358
6498	2.3835	23.0965	40.0489
6499	2.3835	21.2832	74.5727
6500	2.3836	-3.4713	166.919
6501	2.384	-3.9241	196.137
6502	2.3841	-2.8253	140.405
6503	2.3843	17.9144	66.2315
6504	2.3844	-2.924	86.4198
6505	2.3844	-3.902	261.734
6506	2.3844	-3.8716	126.221
6507	2.3845	-2.8159	279.901
6508	2.3846	-3.8457	90.162
6509	2.3846	10.4716	116.172
6510	2.3856	-2.8914	49.9867
6511	2.386	-2.8159	41.2048
6512	2.3863	-3.395	331.017
6513	2.3868	-2.9809	165.935
6514	2.3869	-2.973	17.9854
6515	2.3869	-3.9235	27.0056
6516	2.387	14.579	232.177
6517	2.3873	14.6547	5.9849

6518	2.3874	-3.8692	52.4593
6519	2.3877	-2.7806	184.352
6520	2.3878	16.42	293.491
6521	2.3881	-3.2374	328.849
6522	2.3882	-3.6529	316.705
6523	2.3883	-3.1054	72.6329
6524	2.3885	-3.5532	48.7822
6525	2.3886	9.283	129.502
6526	2.3886	-2.8305	188.123
6527	2.3887	-3.7162	241.987
6528	2.3892	26.1356	205.886
6529	2.3893	-3.2382	38.1703
6530	2.3893	-4.4261	333.969
6531	2.3893	-2.8195	215.001
6532	2.3894	-2.9351	263.388
6533	2.3895	-2.7343	169.829
6534	2.3901	-2.8259	80.034
6535	2.3902	16.1772	124.647
6536	2.3902	-3.7262	257.992
6537	2.3902	-4.1255	317.025
6538	2.3905	16.6554	116.547
6539	2.3905	-2.8008	304.903
6540	2.3906	25.2559	55.0716
6541	2.3907	-2.8554	115.336
6542	2.3907	-3.0689	342.885
6543	2.3908	13.6058	270.199
6544	2.3909	-4.0601	74.8525
6545	2.3909	-2.8506	3.2599
6546	2.3909	-2.8357	126.091
6547	2.3909	-2.9679	321.051
6548	2.391	26.3249	151.201
6549	2.3913	9.8453	158.582
6550	2.3916	-2.9093	38.6892
6551	2.3917	20.3767	122.018
6552	2.3919	10.7127	225.35
6553	2.392	13.6901	240.704
6554	2.3922	-2.8314	214.402
6555	2.3923	18.9122	46.2912
6556	2.3923	-3.3634	154.118
6557	2.3924	-3.1648	202.992
6558	2.3925	-3.1768	87.4344
6559	2.3926	12.7537	7.6289
6560	2.3931	18.2486	127.173
6561	2.3932	-2.7798	282.157
6562	2.3932	23.4836	147.633
6563	2.3936	19.3577	69.4802
6564	2.3936	-2.9245	257.805
6565	2.3936	-2.7742	326.538

6566	2.3937	-3.2742	17.8633
6567	2.3938	21.4469	135.278
6568	2.394	11.4505	7.5063
6569	2.3941	21.6699	335.25
6570	2.3944	-2.8259	85.0764
6571	2.3944	-2.6721	345.312
6572	2.3949	-3.1247	284.091
6573	2.395	24.305	201.067
6574	2.395	-2.8023	342.006
6575	2.3951	-2.7922	336.951
6576	2.3951	13.455	11.2961
6577	2.3952	15.5853	61.7944
6578	2.3957	23.1657	200.253
6579	2.3957	-2.9174	200.354
6580	2.3958	-4.2008	128.577
6581	2.396	29.8017	214.761
6582	2.3962	15.3853	304.437
6583	2.3966	14.9813	163.752
6584	2.3967	-2.821	259.339
6585	2.3968	23.5304	144.608
6586	2.3968	18.5657	198.633
6587	2.3969	-4.6609	301.799
6588	2.3969	18.2974	12.519
6589	2.3972	-3.3375	335.633
6590	2.3973	3.5187	294.351
6591	2.3974	-2.8497	156.873
6592	2.3974	-2.8206	117.654
6593	2.3976	21.5115	284.3
6594	2.3977	12.8492	225.016
6595	2.3978	19.6917	309.288
6596	2.398	-2.7523	187.19
6597	2.398	24.0391	145.445
6598	2.3982	-5.2072	44.8816
6599	2.3987	-2.8065	31.2474
6600	2.3991	16.7705	163.979
6601	2.3993	-2.8266	319.452
6602	2.3993	-2.803	139.26
6603	2.3997	14.5532	147.458
6604	2.3997	15.8338	102.304
6605	2.3998	-2.8276	11.93
6606	2.4002	-3.476	42.0333
6607	2.4002	-3.015	27.1512
6608	2.4006	-2.8634	35.3427
6609	2.4007	21.9415	120.705
6610	2.4019	18.9192	149.576
6611	2.4019	-2.9466	323.203
6612	2.4025	-4.0126	111.736
6613	2.4025	-4.0129	32.3905

6614	2.4026	13.2505	260.026
6615	2.4026	18.4256	197.361
6616	2.403	-3.7604	215.854
6617	2.4033	-3.9341	245.847
6618	2.4033	-3.9146	262.187
6619	2.4033	16.5164	192.036
6620	2.4034	-2.7692	328.102
6621	2.4039	-3.0656	67.1248
6622	2.4039	16.9356	307.243
6623	2.404	8.7562	196.529
6624	2.4042	-3.2586	13.2796
6625	2.4043	-2.9325	108.23
6626	2.4044	19.4466	33.0669
6627	2.4046	-2.6905	143.168
6628	2.4049	19.8001	210.835
6629	2.4051	-2.9096	124.363
6630	2.4052	27.3709	234.086
6631	2.4053	16.1295	94.0887
6632	2.4055	15.9086	231.569
6633	2.4058	-3.1559	119.632
6634	2.4059	-2.8331	118.875
6635	2.4062	22.2103	204.231
6636	2.4064	-3.949	302.166
6637	2.4066	16.1639	245.039
6638	2.4066	-4.0053	25.9872
6639	2.4066	18.3757	218.127
6640	2.4066	-2.8664	141.927
6641	2.4067	-3.5026	275.132
6642	2.4067	-4.4639	283.85
6643	2.4069	-2.93	21.3451
6644	2.407	12.2337	137.475
6645	2.407	16.5272	259.487
6646	2.4071	-3.5036	180.241
6647	2.4072	-2.9378	111.761
6648	2.4072	12.6734	132.463
6649	2.4073	-3.4789	1.63
6650	2.4076	-3.1743	285.47
6651	2.4076	15.988	4.5985
6652	2.4082	-3.8546	210.857
6653	2.4082	-2.9209	25.1782
6654	2.4083	22.8779	22.5764
6655	2.4083	15.497	267.074
6656	2.4086	-3.0529	35.2559
6657	2.409	-3.5814	359.572
6658	2.409	33.6933	314.246
6659	2.409	-3.3119	209.499
6660	2.4091	21.3463	57.7173
6661	2.4092	11.2364	164.53

6662	2.4092	-2.831	27.4136
6663	2.4094	-3.9598	42.7893
6664	2.4097	-3.2737	329.108
6665	2.4097	22.2666	6.5733
6666	2.4097	-3.3258	328.678
6667	2.41	12.0914	274.655
6668	2.4101	-3.1306	263.897
6669	2.4105	16.4115	299.169
6670	2.4106	-3.57	6.9493
6671	2.4107	-2.6858	179.39
6672	2.4109	-2.9404	75.1233
6673	2.4111	-2.7808	263.193
6674	2.4114	-2.7745	2.8186
6675	2.4117	-3.2495	163.208
6676	2.412	-2.9354	130.602
6677	2.412	-3.2011	227.758
6678	2.412	15.5188	173.51
6679	2.412	19.8027	305.8
6680	2.4121	-3.753	87.0975
6681	2.4122	-3.19	119.821
6682	2.4123	-4.0674	353.23
6683	2.4124	16.3957	138.13
6684	2.4129	-2.9254	304.443
6685	2.413	-2.7108	239.337
6686	2.4136	-2.9231	229.593
6687	2.4139	-3.186	274.021
6688	2.4141	22.0358	286.193
6689	2.4142	-2.7167	131.822
6690	2.4143	-3.3387	308.825
6691	2.4144	-4.8967	212.646
6692	2.4146	-2.935	100.513
6693	2.4146	-2.8192	112.808
6694	2.4147	-2.947	23.2321
6695	2.4147	18.1128	86.4521
6696	2.4149	25.632	213.005
6697	2.415	18.8172	283.015
6698	2.415	-2.8583	125.857
6699	2.4154	-2.7925	342.356
6700	2.4157	15.6896	109.569
6701	2.4158	-3.0322	295.108
6702	2.4159	14.0617	204.369
6703	2.4159	-2.9093	354.84
6704	2.416	-2.8453	318.39
6705	2.4162	-3.2174	209.392
6706	2.4166	-2.9661	217.482
6707	2.4167	-2.8764	278.77
6708	2.4172	12.6623	210.71
6709	2.4175	-4.4045	95.2467

6710	2.4176	15.2461	184.325
6711	2.4176	-3.7563	39.7639
6712	2.4176	-2.7933	337.211
6713	2.4177	-3.5622	72.7054
6714	2.4177	16.4333	93.4163
6715	2.4177	-2.8888	260.979
6716	2.4178	-4.0803	44.3936
6717	2.418	-2.7583	244.994
6718	2.4181	-2.8353	149.907
6719	2.4185	24.4533	211.834
6720	2.4186	-1.1529	34.4838
6721	2.4186	-3.8758	318.986
6722	2.4191	14.5626	329.836
6723	2.4191	-2.8496	293.142
6724	2.4192	-3.2951	169.16
6725	2.4193	20.1817	204.717
6726	2.4195	-2.962	72.5347
6727	2.4197	17.1752	97.1116
6728	2.4199	12.707	331.719
6729	2.4205	-2.9834	349.642
6730	2.4208	-3.9673	338.47
6731	2.421	-2.8047	322.862
6732	2.4213	-4.4975	339.913
6733	2.4217	20.6942	140.042
6734	2.4218	16.3561	71.9525
6735	2.4221	-2.7597	48.4728
6736	2.4223	16.1455	155.564
6737	2.4226	20.1076	81.2934
6738	2.4226	-2.8596	305.425
6739	2.4226	-4.4519	290.73
6740	2.4228	-2.8246	1.2718
6741	2.4228	25.9166	18.82
6742	2.4229	-3.3475	226.941
6743	2.4229	-3.3781	166.54
6744	2.423	13.5017	193.424
6745	2.4234	-4.3007	259.278
6746	2.4236	24.4857	37.47
6747	2.4236	-3.7128	216.641
6748	2.4236	20.8422	61.1074
6749	2.4236	-4.421	259.356
6750	2.4237	-3.5402	99.3963
6751	2.4238	-2.7278	188.477
6752	2.424	20.5296	162.606
6753	2.4242	-2.7566	77.9834
6754	2.4246	18.4095	214.002
6755	2.4247	16.4058	289.995
6756	2.4248	-2.8857	283.388
6757	2.4248	-2.8623	288.227

6758	2.4249	20.2342	59.3094
6759	2.4251	-2.865	122.997
6760	2.4251	23.0049	310.517
6761	2.4252	-2.8821	317.594
6762	2.4252	-3.6665	309.377
6763	2.4253	-4.1314	266.822
6764	2.4254	-2.9721	70.794
6765	2.4254	16.2614	146.989
6766	2.4255	-4.1894	337.053
6767	2.4256	-4.0582	268.297
6768	2.4258	-3.2242	260.948
6769	2.426	13.5203	275.896
6770	2.426	-3.8771	344.642
6771	2.4264	-3.1665	155.677
6772	2.4268	-2.6543	212.618
6773	2.4268	-3.1566	136.762
6774	2.4268	-3.3389	306.649
6775	2.4269	-3.9067	202.199
6776	2.4272	-3.4443	156.333
6777	2.4274	-2.9984	302.151
6778	2.4275	16.7583	46.4055
6779	2.4276	26.4167	34.3547
6780	2.4277	17.3814	75.0729
6781	2.4278	-3.7704	339.159
6782	2.4279	-2.7166	312.658
6783	2.4284	16.3421	271.634
6784	2.4285	23.3681	8.4236
6785	2.4285	-2.9538	337.48
6786	2.4288	19.8761	206.051
6787	2.429	17.8119	25.6095
6788	2.4293	-2.7715	9.6289
6789	2.4293	17.8701	63.2312
6790	2.4294	-3.907	129.638
6791	2.4296	-2.8778	43.6691
6792	2.4298	2.8014	90.9006
6793	2.4299	-3.3966	166.663
6794	2.43	-3.8187	108.034
6795	2.4301	20.5584	4.0918
6796	2.4302	-3.8855	4.3204
6797	2.4304	17.3403	156.382
6798	2.4304	18.7901	263.508
6799	2.4307	-2.8694	140.456
6800	2.4311	-2.9557	333.577
6801	2.4312	20.44	251.724
6802	2.4313	22.5676	296.42
6803	2.4313	20.351	13.809
6804	2.4313	-3.0031	28.366
6805	2.4315	-2.8964	130.929

6806	2.4316	-2.805	54.8574
6807	2.4317	-4.6771	147.845
6808	2.4317	23.3713	37.3411
6809	2.4317	-2.6819	246.919
6810	2.4319	-2.8311	181.982
6811	2.4319	-3.32	306.571
6812	2.4319	-2.8163	161.039
6813	2.4321	-3.0449	128.214
6814	2.4322	-4.2729	328.015
6815	2.4322	20.8443	30.2436
6816	2.4325	-3.2391	181.379
6817	2.4327	-3.0541	201.563
6818	2.4328	21.314	330.432
6819	2.4329	-3.8559	68.4645
6820	2.4331	15.6344	285.917
6821	2.4335	26.4075	274.826
6822	2.4337	24.4865	20.0304
6823	2.4339	21.3286	56.2896
6824	2.4347	-3.1597	309.162
6825	2.435	-4.0826	15.4647
6826	2.4352	-2.7792	260.128
6827	2.4352	-3.3076	168.876
6828	2.4354	-2.7918	204.841
6829	2.436	-2.764	117.07
6830	2.436	-2.8522	317.206
6831	2.4361	-3.8726	338.249
6832	2.4364	19.3833	272.636
6833	2.4369	16.3783	12.3377
6834	2.4371	24.1868	84.6533
6835	2.4371	-2.8622	283.594
6836	2.4375	25.7869	8.9007
6837	2.4375	-2.8401	122.335
6838	2.4379	16.1879	142.674
6839	2.4379	-4.5012	327.372
6840	2.438	17.6865	114.19
6841	2.4382	14.6027	293.337
6842	2.4385	30.9749	314.036
6843	2.4385	-2.8658	341.073
6844	2.4386	15.8284	213.289
6845	2.4387	16.8466	223.964
6846	2.4389	-3.7106	339.783
6847	2.439	26.928	291.627
6848	2.439	15.3643	175.292
6849	2.439	-3.0207	98.1083
6850	2.4391	21.0263	196.954
6851	2.4392	-4.2318	100.114
6852	2.4393	17.5384	29.3854
6853	2.4396	-2.8014	280.983

6854	2.4399	18.4669	56.7207
6855	2.44	-3.4841	17.7705
6856	2.4404	24.6727	327.445
6857	2.4405	-2.9352	131.233
6858	2.4406	-3.7862	83.3771
6859	2.4411	-2.7973	220.66
6860	2.4417	-2.9688	254.104
6861	2.4417	14.551	277.88
6862	2.4419	-3.3966	204.101
6863	2.4419	-3.9425	288.384
6864	2.4422	-3.6849	16.1643
6865	2.4422	-2.8066	264.165
6866	2.4423	19.378	269.382
6867	2.4425	6.4164	144.175
6868	2.4427	17.0637	106.991
6869	2.4429	-2.7532	322.868
6870	2.443	-2.8708	3.0182
6871	2.4432	18.9449	275.265
6872	2.4432	22.8376	277.059
6873	2.4434	20.6265	1.6833
6874	2.4436	-2.6707	149.622
6875	2.4438	-2.933	341.7
6876	2.4438	15.3537	318.729
6877	2.444	17.6766	232.841
6878	2.4442	27.2526	297.205
6879	2.4443	-2.9	329.935
6880	2.4444	-4.0291	174.281
6881	2.4444	19.508	242.335
6882	2.4446	-3.4757	313.999
6883	2.4447	-2.8505	222.678
6884	2.4451	-2.7381	89.0615
6885	2.4454	18.173	67.0593
6886	2.4456	14.7973	352.922
6887	2.4457	-4.1937	195.285
6888	2.4462	-2.9231	91.0433
6889	2.4462	16.814	277.391
6890	2.4462	-2.9957	211.579
6891	2.4464	-2.8266	61.5554
6892	2.4466	-4.0279	118.565
6893	2.4469	22.6229	273.463
6894	2.4471	-4.4079	149.163
6895	2.4472	12.9306	308.867
6896	2.4472	23.3264	287.711
6897	2.4474	-2.9586	98.4316
6898	2.4475	21.9081	328.168
6899	2.4481	15.6492	242.285
6900	2.4481	-2.9817	158.04
6901	2.4482	10.3805	262.733

6902	2.4482	22.3872	20.7128
6903	2.4482	18.6838	240.39
6904	2.4483	-3.4965	153.539
6905	2.4487	24.2976	11.9385
6906	2.449	-3.8338	262.927
6907	2.449	22.5525	209.525
6908	2.4492	19.237	257.906
6909	2.4492	-2.7841	209.547
6910	2.4492	15.5603	226.869
6911	2.4495	16.1657	261.215
6912	2.4495	18.7731	150.857
6913	2.4497	22.0671	53.07
6914	2.4498	-2.7864	273.34
6915	2.4502	-3.172	189.921
6916	2.4502	14.4133	333.177
6917	2.4504	-2.8553	284.172
6918	2.4506	-4.3157	191.348
6919	2.4506	-2.9065	251.844
6920	2.4507	22.9505	133.238
6921	2.4509	16.1044	257.154
6922	2.451	24.6758	218.192
6923	2.4517	23.8478	196.712
6924	2.4517	16.352	211.049
6925	2.4517	28.3489	26.9637
6926	2.4518	27.5928	290.815
6927	2.4519	-2.8164	122.116
6928	2.4521	-4.698	116.179
6929	2.4522	14.8195	32.5683
6930	2.4522	16.2707	312.143
6931	2.4522	-3.7524	162.366
6932	2.4525	12.4378	229.257
6933	2.4525	-2.7742	299.499
6934	2.4534	14.3792	228.829
6935	2.4539	20.1323	13.1003
6936	2.4541	-3.5028	170.7
6937	2.4543	17.4429	221.138
6938	2.4545	15.343	101.49
6939	2.4545	20.0197	287.913
6940	2.4547	-3.6717	269.466
6941	2.455	-3.3807	135.675
6942	2.4556	22.7931	21.9146
6943	2.456	-3.4127	176.69
6944	2.456	-3.0991	13.1284
6945	2.4561	13.0075	250.73
6946	2.4563	-2.853	8.4648
6947	2.4564	-3.2605	57.0676
6948	2.4565	-2.9253	346.602
6949	2.4566	-2.8574	108.68

6950	2.4572	24.9772	31.091
6951	2.4574	24.6312	207.027
6952	2.4578	17.3386	64.4781
6953	2.4578	22.3806	77.3617
6954	2.4579	24.5863	152.726
6955	2.4579	-2.8448	257.679
6956	2.4579	-3.2501	151.933
6957	2.458	21.0729	100.515
6958	2.4581	17.6231	122.95
6959	2.4583	-3.07	255.297
6960	2.4585	-2.9715	140.126
6961	2.4585	-3.3663	146.73
6962	2.4588	-2.9133	330.114
6963	2.4589	20.2066	267.607
6964	2.459	21.7157	232.802
6965	2.4594	18.2965	155.946
6966	2.4594	-3.0374	304.973
6967	2.4596	-2.9532	244.942
6968	2.4596	17.3449	115.451
6969	2.4599	23.922	122.663
6970	2.4601	15.6212	140.159
6971	2.4601	35.1511	34.0038
6972	2.4605	15.1029	359.047
6973	2.4608	12.7322	146.673
6974	2.461	22.5681	336.338
6975	2.4611	-4.1963	258.302
6976	2.4612	28.8709	240.573
6977	2.4614	-4.1956	123.377
6978	2.4617	18.9272	84.1917
6979	2.4617	21.8249	29.387
6980	2.4619	16.2277	285.642
6981	2.4621	13.2326	183.63
6982	2.4623	18.5323	168.188
6983	2.4624	19.4898	1.0227
6984	2.4626	-3.4606	353.756
6985	2.4627	20.2679	153.345
6986	2.4627	30.1857	197.518
6987	2.4628	-2.7806	249.427
6988	2.4632	-3.8235	55.4716
6989	2.4632	-4.3567	339.715
6990	2.4632	-2.8731	225.742
6991	2.4633	-2.9303	30.5621
6992	2.4635	17.0782	351.588
6993	2.4636	25.2924	244.344
6994	2.4637	30.38	210.461
6995	2.4637	15.4516	147.647
6996	2.4637	-2.8756	303.737
6997	2.4639	-2.8207	326.809

6998	2.4641	8.5353	141.601
6999	2.4641	-2.8889	58.6844
7000	2.4642	-2.9124	263.094
7001	2.4647	16.7548	29.738
7002	2.4648	15.9558	233.687
7003	2.4648	-3.3689	139.647
7004	2.4648	-3.059	263.684
7005	2.4651	21.8591	140.258
7006	2.4651	13.8228	316.232
7007	2.4652	21.5307	136.117
7008	2.4654	-3.198	342.725
7009	2.4657	-3.2545	335.497
7010	2.4658	16.7471	163.211
7011	2.466	27.4221	202.679
7012	2.4662	10.6927	293.832
7013	2.4662	17.4479	234.397
7014	2.4663	27.6235	15.9198
7015	2.4663	22.4319	142.807
7016	2.4664	-3.0243	3.7134
7017	2.4664	25.4581	290.41
7018	2.4664	20.7452	80.8017
7019	2.4667	-3.9068	218.494
7020	2.4668	-4.2011	180.394
7021	2.4669	-3.6453	213.856
7022	2.4669	4.7803	42.9806
7023	2.4671	-3.7249	257.069
7024	2.4671	-3.9961	253.759
7025	2.4672	-3.2146	311.321
7026	2.4673	-2.7536	95.6571
7027	2.4674	16.2453	183.401
7028	2.4677	18.153	126.528
7029	2.4677	20.7447	84.276
7030	2.4678	-3.8894	145.68
7031	2.468	-2.8518	336.116
7032	2.4681	-2.6907	18.3406
7033	2.4683	-2.941	141.008
7034	2.4683	17.9137	34.5613
7035	2.4686	-4.4033	81.9776
7036	2.4689	-3.0335	103.815
7037	2.4689	0.5246	72.656
7038	2.4689	16.7164	134.014
7039	2.4691	-2.6834	115.739
7040	2.4691	19.4236	255.394
7041	2.4695	23.0356	25.8026
7042	2.4696	24.1685	245.708
7043	2.4697	-2.8591	165.018
7044	2.4701	-2.8558	326.316
7045	2.4701	26.093	120.588

7046	2.4701	-4.0683	301.943
7047	2.4704	16.058	147.886
7048	2.4705	20.8847	120.19
7049	2.4706	27.0954	93.3575
7050	2.4706	21.432	202.017
7051	2.4708	-4.1213	259.886
7052	2.4711	-2.8923	121.215
7053	2.4711	21.2101	300.187
7054	2.4713	15.5149	223.438
7055	2.4714	-3.5411	256.381
7056	2.4715	-2.8798	312.731
7057	2.4716	-3.029	178.219
7058	2.4718	-3.528	333.57
7059	2.4719	25.5721	157.577
7060	2.4722	-4.0603	168.445
7061	2.4722	-2.8872	260.565
7062	2.4725	-3.4668	157.282
7063	2.4727	-2.7733	267.475
7064	2.4727	20.1712	213.136
7065	2.4729	-3.2132	305.012
7066	2.4729	-3.9916	108.311
7067	2.4729	-2.8368	280.829
7068	2.4731	-2.7583	145.221
7069	2.4733	-4.3043	133.457
7070	2.4735	19.1151	208.711
7071	2.4736	-2.9602	80.002
7072	2.4736	19.5686	42.903
7073	2.4736	25.7454	151.782
7074	2.4738	14.4554	269.837
7075	2.4739	24.2629	29.8478
7076	2.4741	18.9923	235.67
7077	2.4743	13.6371	251.21
7078	2.4744	-2.8327	66.6015
7079	2.4747	-2.8142	259.14
7080	2.4749	18.1974	3.4736
7081	2.4752	13.7093	179.566
7082	2.4755	-2.7401	317.28
7083	2.476	-2.6879	299.947
7084	2.4761	13.9699	271.902
7085	2.4763	35.0528	207.419
7086	2.4765	-3.1186	289.159
7087	2.4766	34.0477	359.165
7088	2.4769	14.8452	337.96
7089	2.4769	-3.6932	67.3821
7090	2.477	21.8783	240.12
7091	2.4772	21.984	353.053
7092	2.4774	23.8638	194.265
7093	2.4775	-2.8883	138.242

7094	2.4775	-2.9259	309.498
7095	2.4778	-2.9827	187.725
7096	2.4782	-4.0947	130.269
7097	2.4782	-2.8969	276.761
7098	2.4782	21.8972	339.881
7099	2.4784	-3.2318	347.121
7100	2.4784	17.3893	261.383
7101	2.4785	26.474	334.558
7102	2.4785	-3.9465	264.466
7103	2.4786	-3.4273	102.769
7104	2.4786	-2.7565	152.372
7105	2.4786	-2.9861	99.8502
7106	2.4788	-3.8585	167.404
7107	2.4792	17.8286	133.211
7108	2.4793	-4.0911	170.959
7109	2.4795	-2.8409	270.445
7110	2.4795	16.5986	163.405
7111	2.4795	18.0573	268.776
7112	2.4797	-2.8065	184.132
7113	2.48	17.9888	192.379
7114	2.4803	-2.9181	318.136
7115	2.4803	-3.3169	129.58
7116	2.4804	-2.9702	79.836
7117	2.4804	8.321	317.093
7118	2.4807	-3.3662	125.624
7119	2.4808	23.0093	343.672
7120	2.4808	24.5098	199.49
7121	2.481	-3.6785	325.19
7122	2.481	-4.7987	165.838
7123	2.4812	-3.8561	154.547
7124	2.4812	20.3653	195.637
7125	2.4813	-2.6994	175.262
7126	2.4814	18.2604	175.291
7127	2.4818	29.1847	105.246
7128	2.4819	18.1682	240.575
7129	2.482	-2.9496	177.21
7130	2.482	-2.9392	271.171
7131	2.4825	-2.992	267.282
7132	2.4825	-2.9594	304.855
7133	2.4826	24.563	16.046
7134	2.4827	-2.9307	260.67
7135	2.4827	-3.2681	16.2857
7136	2.4828	21.8769	53.5582
7137	2.4828	17.7397	161.98
7138	2.4828	21.0629	236.323
7139	2.4828	24.2088	136.604
7140	2.4831	27.7466	3.9424
7141	2.4831	24.2583	135.346

7142	2.4831	28.6722	287.735
7143	2.4833	7.5424	261.662
7144	2.4837	23.5067	281.895
7145	2.4839	16.9073	158.514
7146	2.4839	-3.6872	278.634
7147	2.4841	-2.822	313.653
7148	2.4842	-2.8978	318.064
7149	2.4842	-3.9532	185.238
7150	2.4846	17.1216	159.438
7151	2.4846	-2.819	58.9221
7152	2.4847	14.1706	116.79
7153	2.4852	-2.9067	168.648
7154	2.4858	18.5701	263.53
7155	2.4862	26.4058	170.493
7156	2.4864	10.8862	159.089
7157	2.4868	-2.8553	332.242
7158	2.4869	23.849	201.338
7159	2.4869	-2.7098	94.8037
7160	2.487	-3.838	188.492
7161	2.4872	22.2327	149.719
7162	2.4874	12.9894	190.891
7163	2.4877	21.3128	41.515
7164	2.4877	27.5371	304.764
7165	2.4881	17.7879	5.6818
7166	2.4882	25.9329	259.078
7167	2.4882	-2.7756	217.179
7168	2.4886	-3.2416	329.223
7169	2.4887	17.7042	315.246
7170	2.4887	18.4652	341.525
7171	2.4894	-2.9099	87.4513
7172	2.4894	-3.1821	5.0585
7173	2.4894	25.9456	75.5915
7174	2.4894	22.8682	20.3593
7175	2.4896	-3.9946	76.6711
7176	2.4898	-3.0622	333.385
7177	2.4899	-2.7747	214.825
7178	2.4899	20.8742	270.12
7179	2.49	-3.3198	357.61
7180	2.4902	-3.1	297.136
7181	2.4903	13.8624	145.58
7182	2.4903	25.332	305.97
7183	2.4903	-3.6834	288.174
7184	2.4904	-3.0836	95.5004
7185	2.4905	-2.9861	271.917
7186	2.4906	23.9974	255.452
7187	2.491	-5.1229	173.309
7188	2.491	18.7415	177.046
7189	2.4911	-3.2685	353.144

7190	2.4911	18.272	345.608
7191	2.4911	-3.2826	39.6117
7192	2.4911	-2.7752	51.2952
7193	2.4912	-2.7166	161.149
7194	2.4915	20.3711	145.518
7195	2.4916	22.069	287.089
7196	2.4917	27.8941	81.0755
7197	2.4917	-2.7557	162.018
7198	2.4919	16.7892	170.662
7199	2.4919	21.3	50.5074
7200	2.492	23.9844	110.678
7201	2.492	-2.8554	174.187
7202	2.492	-2.7846	336.333
7203	2.492	-2.8849	282.774
7204	2.4922	-2.9882	14.289
7205	2.4924	17.2823	90.1339
7206	2.4925	-2.9665	17.0905
7207	2.4926	-2.8043	139.143
7208	2.4927	-4.2069	100.077
7209	2.4928	-2.898	324.694
7210	2.4929	-3.2953	233.297
7211	2.4931	25.0374	6.0151
7212	2.4932	14.7218	154.638
7213	2.4934	-3.9311	42.1927
7214	2.4936	25.5722	255.679
7215	2.4936	24.1499	119.39
7216	2.494	22.5618	356.398
7217	2.4941	-3.1504	274.021
7218	2.4943	22.6396	150.406
7219	2.4943	-3.0219	296.286
7220	2.4948	-2.6888	280.654
7221	2.495	19.8635	122.605
7222	2.495	-2.8028	272.993
7223	2.495	14.5634	268.406
7224	2.4951	24.1508	158.992
7225	2.4952	18.495	238.341
7226	2.4953	-3.5777	239.837
7227	2.4953	-2.921	133.447
7228	2.4954	12.3751	211.114
7229	2.4958	-3.1948	165.981
7230	2.4961	-3.2536	180.569
7231	2.4962	-3.8334	306.839
7232	2.4962	18.5897	30.6736
7233	2.4962	-2.7608	323.09
7234	2.4965	24.2512	236.598
7235	2.4965	-3.5353	126.445
7236	2.4967	-2.9736	9.9095
7237	2.4968	-3.2523	61.3988

7238	2.4968	-4.0829	80.403
7239	2.4969	-2.8106	56.6191
7240	2.4972	27.685	19.9429
7241	2.4974	-3.1231	333.8
7242	2.4976	-3.352	119.119
7243	2.4977	-2.8116	83.7241
7244	2.4977	-2.801	355.965
7245	2.4981	25.0176	275.741
7246	2.4981	-2.8939	321.772
7247	2.4981	-2.692	340.4
7248	2.4981	25.0424	239.819
7249	2.4981	-3.2534	118.031
7250	2.4982	20.574	113.153
7251	2.4987	-3.6566	182.62
7252	2.4987	-2.8382	61.0969
7253	2.4989	-2.8315	177.904
7254	2.4989	-4.1223	221.461
7255	2.499	14.4245	275.73
7256	2.499	-2.7914	265.41
7257	2.4992	-2.9075	133.354
7258	2.4993	-2.9344	353.016
7259	2.4993	-2.9895	192.064
7260	2.4994	-3.3136	35.0076
7261	2.4995	19.1657	20.1652
7262	2.4995	-3.0172	330.823
7263	2.4995	-3.3326	78.788
7264	2.4996	27.2852	51.7694
7265	2.4997	-3.2299	293.749
7266	2.5001	20.9219	323.981
7267	2.5004	27.3982	359.7
7268	2.5008	-2.868	229.337
7269	2.5009	16.8707	338.121
7270	2.5009	-3.9627	174.832
7271	2.501	-2.877	182.835
7272	2.5012	24.4752	137.705
7273	2.5013	-4.3551	259.048
7274	2.5013	-2.9474	145.213
7275	2.5013	-2.9206	170.679
7276	2.5013	-3.1631	131.941
7277	2.5014	-2.8087	297.725
7278	2.5016	9.3731	288.243
7279	2.5018	22.8942	82.2792
7280	2.5019	-2.9914	213.14
7281	2.5019	-3.9287	291.333
7282	2.5021	19.9651	219.56
7283	2.5021	-3.6859	308.157
7284	2.5021	22.0409	160.529
7285	2.5022	26.279	129.549

7286	2.5022	-3.9641	66.1866
7287	2.5024	-3.1421	227.604
7288	2.5028	-3.0888	114.584
7289	2.5028	16.6495	358.93
7290	2.5029	19.9821	14.4184
7291	2.503	32.89	207.662
7292	2.503	14.0932	137.066
7293	2.5031	20.5492	172.394
7294	2.5031	-3.6614	324.691
7295	2.5032	25.4548	0.7395
7296	2.5032	20.4897	165.162
7297	2.5033	-2.9166	28.6304
7298	2.5033	17.6882	183.927
7299	2.5034	-3.8786	335.077
7300	2.5035	-4.4597	145.042
7301	2.5037	-3.3338	308.951
7302	2.5038	-3.869	16.643
7303	2.5039	-4.7866	258.28
7304	2.5043	-3.9658	255.628
7305	2.5045	22.8482	30.8696
7306	2.505	-2.9613	101.599
7307	2.505	-4.0238	65.8793
7308	2.5051	-3.1546	344.011
7309	2.5051	-3.8553	173.219
7310	2.5052	-2.9242	276.133
7311	2.5053	-3.0359	92.2695
7312	2.5055	26.1423	344.154
7313	2.5057	-4.1972	110.967
7314	2.5058	22.2045	204.669
7315	2.5058	26.8307	56.241
7316	2.5059	-3.0921	297.776
7317	2.506	-2.7645	5.8231
7318	2.5061	19.5906	159.829
7319	2.5062	34.6485	202.97
7320	2.5063	-2.9229	253.886
7321	2.5063	-2.8049	267.09
7322	2.5065	-3.3662	353.285
7323	2.5066	23.5261	72.6075
7324	2.5066	-3.864	201.443
7325	2.5066	-2.7735	256.785
7326	2.5067	23.5319	230.183
7327	2.5067	-3.1648	291.863
7328	2.5068	22.898	251.842
7329	2.5069	14.3059	229.724
7330	2.5072	-3.9063	61.8913
7331	2.5074	-2.9518	68.5135
7332	2.5074	-2.8495	351.897
7333	2.5075	28.8841	270.019

7334	2.5075	-2.843	90.8916
7335	2.5075	23.8726	212.553
7336	2.5075	-3.8746	162.569
7337	2.5076	-3.3139	351.6
7338	2.5077	-2.9541	275.277
7339	2.5077	12.5871	220.529
7340	2.5078	-2.8065	255.844
7341	2.508	-2.801	327.903
7342	2.5081	-3.841	54.6018
7343	2.5082	-2.7858	8.8646
7344	2.5083	-4.0579	352.037
7345	2.5083	20.6541	52.6372
7346	2.5084	-2.7675	87.1144
7347	2.5085	18.6442	315.751
7348	2.5086	-3.0887	243.924
7349	2.5086	-3.1696	321.858
7350	2.5089	-2.9549	130.629
7351	2.5089	-2.8624	345.063
7352	2.5089	22.0915	10.5387
7353	2.509	14.1999	179.185
7354	2.5091	-3.7935	332.526
7355	2.5091	-2.8506	99.9459
7356	2.5092	12.9266	338.234
7357	2.5093	-2.7966	150.807
7358	2.5094	18.5062	163.28
7359	2.5095	-3.479	72.412
7360	2.5096	-3.2759	81.962
7361	2.5096	-2.7643	137.474
7362	2.5096	-3.1749	322.648
7363	2.5099	-2.8813	120.09
7364	2.51	17.188	149.883
7365	2.51	-3.0143	223.422
7366	2.51	-2.7905	339.948
7367	2.5102	-2.9432	83.7403
7368	2.5104	23.924	287.959
7369	2.5106	16.0798	294.214
7370	2.5112	-4.1507	338.15
7371	2.5112	19.5876	316.772
7372	2.5112	-3.0083	324.83
7373	2.5113	30.2991	238.648
7374	2.5113	20.2931	191.172
7375	2.5118	17.1499	191.474
7376	2.5118	26.1044	133.46
7377	2.5119	30.0363	74.7814
7378	2.512	-4.2905	92.8365
7379	2.5121	23.0079	230.258
7380	2.5125	18.3864	214.119
7381	2.5127	-3.5516	144.187

7382	2.5128	26.9196	83.3501
7383	2.5129	-2.7748	55.3502
7384	2.5131	-4.1224	120.918
7385	2.5132	30.2421	208.02
7386	2.5134	-2.794	210.896
7387	2.5134	-2.969	169.038
7388	2.5136	-2.7735	354.355
7389	2.5137	19.4702	273.217
7390	2.5138	-2.9068	68.2821
7391	2.5139	27.3328	24.8988
7392	2.5141	-3.5921	315.522
7393	2.5143	-2.965	347.313
7394	2.5143	-2.8098	284.98
7395	2.5143	-2.9174	264.754
7396	2.5144	19.9319	166.95
7397	2.5147	27.3892	269.505
7398	2.5151	-3.903	252.037
7399	2.5152	32.1671	47.1185
7400	2.5153	-3.8444	86.229
7401	2.5155	17.0128	238.979
7402	2.5155	18.4435	255.664
7403	2.5156	27.7267	193.067
7404	2.5156	26.7537	195.306
7405	2.5157	-2.9462	117.695
7406	2.5158	22.7731	136.453
7407	2.5158	-2.86	298.62
7408	2.5161	-4.3492	55.7972
7409	2.5162	-2.9718	96.2007
7410	2.5166	22.1785	4.5522
7411	2.517	14.6759	17.1838
7412	2.5171	15.0288	215.827
7413	2.5171	17.1328	119.49
7414	2.5171	-3.9531	219.373
7415	2.5171	24.7276	307.346
7416	2.5172	-2.985	42.5662
7417	2.5173	-2.8234	94.6273
7418	2.5176	18.7029	36.0629
7419	2.5176	-3.1669	191.473
7420	2.5178	-2.8461	312.825
7421	2.5181	-3.4716	265.327
7422	2.5182	-3.8745	230.455
7423	2.5182	-4.1984	248.876
7424	2.5183	-3.8028	329.364
7425	2.5183	28.3053	47.3157
7426	2.5187	18.8761	38.3423
7427	2.5187	-2.865	256.525
7428	2.5187	24.4047	182.15
7429	2.5188	12.2118	64.1233

7430	2.5188	14.1336	148.649
7431	2.5189	24.7383	139.715
7432	2.5189	18.8979	252.321
7433	2.5191	23.405	18.5104
7434	2.5193	23.2363	259.859
7435	2.5193	-3.1259	126.908
7436	2.5195	-3.1562	36.2183
7437	2.5196	-3.0897	13.5471
7438	2.5197	19.5351	270.545
7439	2.5198	-2.7583	213.957
7440	2.52	-2.9674	288.106
7441	2.5202	23.3689	298.777
7442	2.5203	25.3394	215.682
7443	2.5205	-2.7299	253.066
7444	2.5206	-2.865	39.2564
7445	2.5206	-2.7128	127.272
7446	2.5206	24.0021	228.927
7447	2.5209	-3.1431	130.646
7448	2.5209	-3.9453	68.9829
7449	2.521	20.8966	179.836
7450	2.5212	-3.2315	20.9985
7451	2.5214	-3.9398	40.3416
7452	2.5216	20.4716	34.0965
7453	2.5217	21.4014	275.792
7454	2.5219	19.226	181.616
7455	2.5223	-3.094	352.339
7456	2.5224	20.601	69.6004
7457	2.5228	19.9745	217.389
7458	2.5229	-4.1485	67.2086
7459	2.5229	17.7355	179.263
7460	2.5229	17.806	317.936
7461	2.5229	-3.8902	268.164
7462	2.5231	17.3143	271.955
7463	2.5233	18.8698	123.216
7464	2.5236	-3.7208	52.9701
7465	2.5236	-4.0793	340.15
7466	2.5236	7.225	80.1605
7467	2.5236	-4.3033	320.025
7468	2.5238	-2.932	296.675
7469	2.524	-3.0391	325.587
7470	2.5241	-3.3459	108.368
7471	2.5242	25.026	207.204
7472	2.5243	-3.3015	66.0727
7473	2.5244	20.2293	333.603
7474	2.5246	19.6854	22.8198
7475	2.5257	-2.8097	165.588
7476	2.5257	21.5854	199.217
7477	2.5259	22.4137	163.834

7478	2.526	30.0567	55.2178
7479	2.5261	26.534	202.225
7480	2.5261	-2.8726	45.9934
7481	2.5261	-2.8679	319.413
7482	2.5264	17.5958	239.192
7483	2.5264	23.3001	221.151
7484	2.5264	-4.1371	310.941
7485	2.5265	16.2329	269.506
7486	2.5265	26.4921	159.453
7487	2.5266	26.7801	323.919
7488	2.5266	22.6397	233.62
7489	2.5272	-2.9499	152.494
7490	2.5274	26.4925	204.496
7491	2.5278	-5.5656	55.2115
7492	2.5278	-2.8451	274.708
7493	2.528	21.8587	310.678
7494	2.528	-4.1218	80.6007
7495	2.5281	26.13	56.6177
7496	2.5284	23.2486	25.1873
7497	2.5285	14.7855	176.697
7498	2.5286	-2.9641	78.5342
7499	2.5289	-4.1182	71.4926
7500	2.5291	18.6781	335.77
7501	2.5291	-2.7655	95.5284
7502	2.5293	-4.2669	343.305
7503	2.5295	13.1057	170.774
7504	2.5295	1.0878	86.8992
7505	2.5296	-3.9035	259.264
7506	2.53	-3.3784	359.002
7507	2.5301	-2.7688	25.773
7508	2.5301	-2.7714	349.114
7509	2.5302	14.3784	153.37
7510	2.5304	25.544	260.99
7511	2.5306	-2.7559	239.705
7512	2.5308	-2.8026	11.8236
7513	2.5309	23.0153	103.331
7514	2.5312	-4.4018	260.431
7515	2.5313	-3.229	262.884
7516	2.5315	-2.913	329.87
7517	2.5315	-2.8516	222.958
7518	2.5315	-2.8518	151.854
7519	2.5317	24.856	61.2533
7520	2.5319	26.5908	79.4383
7521	2.5319	20.1241	248.332
7522	2.5319	-2.7717	81.6522
7523	2.5319	-2.9592	23.9563
7524	2.532	-2.955	65.7062
7525	2.532	-2.7706	119.9

7526	2.5322	24.1742	158.025
7527	2.5323	-2.8978	282.696
7528	2.5335	-3.3831	230.801
7529	2.5336	-3.8463	179.542
7530	2.5336	-2.8145	45.875
7531	2.5337	-2.8303	289.274
7532	2.5337	-3.0954	151.768
7533	2.5338	28.9816	229.035
7534	2.5341	18.9572	293.269
7535	2.5343	-3.3585	332.455
7536	2.5344	20.638	192.944
7537	2.5345	27.0284	195.077
7538	2.5346	21.5344	243.218
7539	2.5348	-3.7466	310.807
7540	2.5349	-2.7482	112.365
7541	2.535	20.5769	112.145
7542	2.5351	-3.5538	132.382
7543	2.5353	19.4007	274.165
7544	2.5356	21.9003	316.317
7545	2.5357	29.3444	60.2829
7546	2.536	-3.7303	30.1676
7547	2.5361	-3.4162	134.982
7548	2.5363	-2.9756	35.4917
7549	2.5364	16.2418	130.674
7550	2.5366	-2.8858	245.738
7551	2.5366	20.3403	241.893
7552	2.5367	-3.953	325.659
7553	2.5367	-3.7187	92.8053
7554	2.5369	-3.1514	173.5
7555	2.5371	26.3806	327.781
7556	2.5372	-2.7528	53.793
7557	2.5375	-3.5498	133.463
7558	2.5375	24.8546	16.2012
7559	2.5377	-2.8447	213.107
7560	2.5378	-2.8416	23.5578
7561	2.5383	-4.2884	300.831
7562	2.5384	-2.9129	271.54
7563	2.5386	24.8565	95.7327
7564	2.5386	22.7878	23.9486
7565	2.5387	-2.9473	21.9648
7566	2.5387	-3.068	89.2844
7567	2.5387	21.6233	144.815
7568	2.5388	30.342	0.6507
7569	2.5388	17.3273	272.275
7570	2.539	26.0394	224.86
7571	2.5391	-2.761	22.0582
7572	2.5392	-3.1224	28.5049
7573	2.5392	-2.9391	284.157

7574	2.5392	16.1373	225.255
7575	2.5393	-3.6567	355.906
7576	2.5401	-2.8201	63.9293
7577	2.5403	21.8825	189.226
7578	2.5404	-2.8766	281.056
7579	2.5405	26.9165	62.9075
7580	2.5406	-2.8022	283.334
7581	2.5408	39.0394	123.649
7582	2.5408	-2.7445	288.2
7583	2.5409	-3.5137	41.5635
7584	2.541	11.8483	25.0449
7585	2.5412	-3.3122	246.679
7586	2.5414	19.8807	204.685
7587	2.5415	-3.6344	177.446
7588	2.5416	-4.327	103.222
7589	2.5417	22.7623	269.618
7590	2.5418	17.4554	301.478
7591	2.5419	21.259	244.927
7592	2.542	29.9845	235.8
7593	2.542	19.0823	230.258
7594	2.5421	19.2515	37.2816
7595	2.5421	-4.7639	103.895
7596	2.5422	-2.9566	54.2947
7597	2.5423	-2.9335	259.351
7598	2.5423	-2.9128	245.984
7599	2.5425	33.4797	25.848
7600	2.5425	17.1523	140.003
7601	2.5427	-3.4494	244.41
7602	2.5428	36.0255	351.664
7603	2.5431	-2.9221	39.1877
7604	2.5432	39.7814	47.5862
7605	2.5432	-2.8115	144.758
7606	2.5436	-2.8064	121.704
7607	2.5436	15.3184	115.28
7608	2.5436	-2.8801	220.832
7609	2.5439	30.2237	153.049
7610	2.5441	-2.9515	315.141
7611	2.5442	16.0789	122.188
7612	2.5442	16.5023	35.447
7613	2.5442	14.4335	178.671
7614	2.5443	-2.7971	324.433
7615	2.5445	-4.6444	109.327
7616	2.5445	-2.8821	336.996
7617	2.5446	-2.8987	119.948
7618	2.5449	24.2749	259.793
7619	2.545	-3.3523	140.928
7620	2.545	-2.8291	67.4304
7621	2.5455	-2.9191	355.021

7622	2.5457	-2.8665	322.665
7623	2.5459	-2.8444	180.334
7624	2.5459	-3.3708	347.563
7625	2.5461	-2.8433	274.474
7626	2.5461	-2.9042	23.9681
7627	2.5464	36.0591	342.506
7628	2.5464	36.6814	239.833
7629	2.5466	-2.7792	330.913
7630	2.5466	-3.5857	260.084
7631	2.5467	-3.3552	79.4688
7632	2.5469	30.6728	273.625
7633	2.547	26.4498	90.0318
7634	2.5471	19.6404	314.343
7635	2.5472	18.1877	115.646
7636	2.5472	31.6413	145.357
7637	2.5473	-3.0009	335.478
7638	2.5473	-3.824	50.7442
7639	2.5473	24.0966	93.7184
7640	2.5473	-3.1587	118.426
7641	2.5477	23.1821	199.997
7642	2.5477	-2.9878	19.131
7643	2.548	24.4616	124.17
7644	2.548	-3.434	151.573
7645	2.548	-3.8672	266.836
7646	2.5481	-3.0979	150.501
7647	2.5482	-4.2778	64.1523
7648	2.5483	26.0809	318.145
7649	2.5484	28.7325	202.16
7650	2.5484	-3.4228	271.132
7651	2.5485	20.081	161.12
7652	2.5486	18.8403	255.373
7653	2.5486	30.0635	20.3857
7654	2.5491	-3.1886	124.881
7655	2.5491	-3.8082	130.862
7656	2.5494	13.3797	329.169
7657	2.5494	23.1329	1.0384
7658	2.5494	-3.8247	29.937
7659	2.5494	17.166	250.587
7660	2.5496	-2.886	141.592
7661	2.5499	-3.3931	3.8676
7662	2.5499	23.9564	350.848
7663	2.55	-4.2148	207.068
7664	2.5503	25.1871	80.6502
7665	2.5508	21.3033	262.855
7666	2.5509	32.9994	283.033
7667	2.5511	29.6583	251.521
7668	2.5512	19.248	335.526
7669	2.5512	20.2242	37.4593

7670	2.5513	-3.3926	324.981
7671	2.5514	24.8279	98.3583
7672	2.5517	-2.8418	109.642
7673	2.5517	22.1896	0.1076
7674	2.5519	21.2133	335.216
7675	2.5521	24.2307	268.274
7676	2.5522	32.5175	314.328
7677	2.5523	-3.1565	335.753
7678	2.5524	-3.2863	301.97
7679	2.5526	-3.4438	200.332
7680	2.5526	-3.3736	220.342
7681	2.5528	-2.8169	132.83
7682	2.5528	28.4207	30.0058
7683	2.5529	-2.7794	44.4729
7684	2.5529	-3.3518	80.9412
7685	2.5529	-2.941	112.766
7686	2.553	-2.7521	351.079
7687	2.5535	-2.7977	224.761
7688	2.5536	-2.966	40.9792
7689	2.5536	-2.904	5.6688
7690	2.5541	31.5174	124.769
7691	2.5541	-3.1426	337.952
7692	2.5547	19.0474	113.151
7693	2.5547	31.9184	234.396
7694	2.5551	36.03	244.155
7695	2.5552	27.0379	7.7762
7696	2.5552	-2.8238	158.622
7697	2.5552	-3.4875	113.59
7698	2.5554	-3.3753	15.984
7699	2.5554	23.4625	180.198
7700	2.5555	-3.3545	133.308
7701	2.5557	-3.2603	29.7894
7702	2.5559	33.0621	209.347
7703	2.5559	-2.8015	4.7682
7704	2.556	16.8735	274.295
7705	2.5564	-3.8767	97.4978
7706	2.5564	-2.7787	38.656
7707	2.5567	-2.8674	250.226
7708	2.5569	23.1159	229.922
7709	2.5569	-2.8048	31.3275
7710	2.5569	-3.0068	357.112
7711	2.5572	-3.0241	272.528
7712	2.5576	-2.7747	249.767
7713	2.5578	26.9185	37.3877
7714	2.5584	28.4463	36.2305
7715	2.5584	23.7007	17.2577
7716	2.5585	22.3331	139.601
7717	2.5586	-3.3827	39.3501

7718	2.5587	-2.9565	96.5007
7719	2.5588	-2.7577	55.9584
7720	2.5589	21.83	73.6947
7721	2.5591	26.7558	131.064
7722	2.5592	-2.9478	148.288
7723	2.5592	-3.1246	226.231
7724	2.5595	-2.7491	330.836
7725	2.5595	-2.9537	318.327
7726	2.5596	26.7349	61.3689
7727	2.5597	-2.8633	142.462
7728	2.5597	29.7193	241.497
7729	2.56	-3.4362	166.2
7730	2.5605	23.7878	279.783
7731	2.5606	-3.6465	348.676
7732	2.5609	15.9362	6.3386
7733	2.5609	-3.1111	139.744
7734	2.5613	18.1793	274.969
7735	2.5613	-2.8935	261.672
7736	2.5614	12.636	254.651
7737	2.5614	21.9338	156.037
7738	2.5615	20.5744	243.567
7739	2.5616	21.3672	6.6018
7740	2.5617	-4.1304	343.773
7741	2.5618	16.7663	66.8804
7742	2.5618	-2.8087	276.055
7743	2.5618	21.3497	237.19
7744	2.5619	-2.9229	109.632
7745	2.5619	28.1159	34.7558
7746	2.5621	-2.7876	84.104
7747	2.5621	-3.5617	288.889
7748	2.5622	-2.8892	263.531
7749	2.5623	-2.7599	119.697
7750	2.5623	33.6751	356.668
7751	2.5624	38.4119	18.8449
7752	2.5624	-2.8314	140.803
7753	2.5625	-3.4103	189.799
7754	2.5627	37.0512	76.4808
7755	2.5627	-2.8939	283.417
7756	2.5631	17.2652	74.247
7757	2.5632	22.4721	92.0278
7758	2.5632	31.1033	152.39
7759	2.5635	18.9767	129.864
7760	2.5635	-2.8363	221.552
7761	2.5637	-2.8078	338.432
7762	2.5638	-2.7882	93.7617
7763	2.5638	-2.7736	193.615
7764	2.5639	30.1095	94.7905
7765	2.5639	-2.8983	104.358

7766	2.564	-2.9368	102.807
7767	2.5643	-2.7936	287.174
7768	2.5644	-2.9953	89.9264
7769	2.5644	-2.9147	136.375
7770	2.5645	-2.902	252.328
7771	2.5645	-3.3597	8.6256
7772	2.5648	-2.8565	322.732
7773	2.5649	-2.9092	273.843
7774	2.565	-3.0543	263.942
7775	2.5651	-2.6683	130.828
7776	2.5651	27.5038	347.586
7777	2.5652	21.2238	270.873
7778	2.5653	16.2023	215.474
7779	2.5658	-2.81	330.196
7780	2.5659	29.005	296.236
7781	2.5659	-2.7422	228.268
7782	2.566	-2.801	135.239
7783	2.5666	-2.9886	34.7811
7784	2.567	-2.8673	324.293
7785	2.5672	-3.9365	171.761
7786	2.5675	-2.7935	196.894
7787	2.5679	26.8923	355.678
7788	2.5679	26.0198	292.654
7789	2.5681	38.7875	20.7458
7790	2.5682	26.1466	182.233
7791	2.5682	-2.7791	82.3585
7792	2.5685	-3.1052	275.218
7793	2.5686	21.8724	146.786
7794	2.5688	21.4926	13.395
7795	2.5688	22.0986	59.6062
7796	2.5694	-3.9852	208.805
7797	2.5696	-4.5058	319.031
7798	2.5697	-3.122	46.8411
7799	2.5698	-2.8089	48.0638
7800	2.5699	23.083	197.377
7801	2.5699	-3.1433	281.87
7802	2.5701	-4.2447	290.444
7803	2.5705	27.194	71.7958
7804	2.5706	-3.5466	175.929
7805	2.5707	-3.9106	236.007
7806	2.5707	-4.5345	319.536
7807	2.5709	39.1826	14.6499
7808	2.5709	20.781	237.85
7809	2.5712	23.0238	171.319
7810	2.5714	-3.505	3.4454
7811	2.5716	-2.7449	276.985
7812	2.5718	24.5355	138.453
7813	2.5721	-3.1095	258.993

7814	2.5722	-2.9006	296.884
7815	2.5723	21.8493	69.3746
7816	2.5724	22.7724	158.552
7817	2.5724	23.522	289.351
7818	2.5728	25.6902	283.78
7819	2.5728	-3.2948	327.572
7820	2.5728	-2.9144	157.415
7821	2.573	-3.1134	140.967
7822	2.573	-3.8338	248.696
7823	2.5731	22.6883	142.39
7824	2.5733	-3.6107	356.704
7825	2.5733	25.3239	95.9084
7826	2.5738	23.5109	88.4198
7827	2.5741	-2.8268	148.91
7828	2.5742	15.1811	245.086
7829	2.5743	-2.8491	117.884
7830	2.5743	21.4939	84.7837
7831	2.5743	-2.7824	341.678
7832	2.5746	-2.9689	33.4173
7833	2.5749	24.4868	187.093
7834	2.5749	23.564	259.569
7835	2.5751	17.6134	159.919
7836	2.5752	-2.7996	301.327
7837	2.5752	-3.1506	190.507
7838	2.5752	-2.7305	16.4528
7839	2.5754	-3.4008	52.9047
7840	2.5755	-4.6076	301.598
7841	2.5756	-3.2163	37.8523
7842	2.5756	-2.8984	213.653
7843	2.5757	-3.0938	81.351
7844	2.5759	-4.4032	282.479
7845	2.5759	-4.0726	277.592
7846	2.5761	22.6975	229.778
7847	2.5761	28.8663	33.8845
7848	2.5761	27.9416	80.7861
7849	2.5763	24.5808	154.033
7850	2.5763	22.7047	351.098
7851	2.5765	-2.9969	9.7652
7852	2.5767	20.6019	129.017
7853	2.5768	-2.9849	335.506
7854	2.5769	26.9773	222.755
7855	2.5769	17.4643	209.942
7856	2.577	-3.0701	353.645
7857	2.577	-3.0075	230.449
7858	2.5771	34.1117	14.0227
7859	2.5772	-2.9533	16.9174
7860	2.5772	24.408	202.642
7861	2.5776	17.7096	253.952

7862	2.5777	-2.8926	161.129
7863	2.5777	17.1277	253.965
7864	2.578	29.2842	352.682
7865	2.578	-2.8455	73.2665
7866	2.578	-2.9229	271.092
7867	2.5781	23.6502	336.187
7868	2.5782	-3.1672	303.006
7869	2.5784	-2.979	59.8544
7870	2.5784	29.3532	153.416
7871	2.5788	20.8482	317.183
7872	2.5789	-2.9994	143.754
7873	2.5789	-3.444	82.7085
7874	2.579	-3.9377	126.8
7875	2.5791	-2.8462	333.51
7876	2.5792	28.0114	145.052
7877	2.5792	22.2037	96.1441
7878	2.5793	26.0092	181.427
7879	2.5795	18.6833	138.287
7880	2.5797	-3.0798	300.049
7881	2.5798	29.4633	243.292
7882	2.5801	30.4452	54.324
7883	2.5802	-2.8373	117.571
7884	2.5802	-2.7796	52.2044
7885	2.5803	24.4544	15.5037
7886	2.5805	10.2824	119.039
7887	2.5808	-2.739	113.82
7888	2.5808	-2.8358	292.827
7889	2.581	26.6563	47.3632
7890	2.581	-3.8126	305.783
7891	2.5812	-3.1149	216.986
7892	2.5817	35.866	8.6834
7893	2.5819	-2.8726	45.4489
7894	2.5822	24.2304	191.12
7895	2.5824	-3.3527	94.6635
7896	2.5826	-3.1342	166.514
7897	2.5826	-2.865	113.775
7898	2.5826	-2.8439	177.862
7899	2.5829	-2.9657	141.401
7900	2.583	-2.9177	68.6712
7901	2.5835	1.468	199.963
7902	2.5836	24.0136	47.893
7903	2.5838	-2.74	175.981
7904	2.584	-2.9731	38.3778
7905	2.584	-3.7928	319.591
7906	2.5844	-3.3145	255.463
7907	2.5844	23.8781	330.243
7908	2.5844	-2.8412	88.4595
7909	2.5844	21.9143	217.646

7910	2.5851	21.4541	308.969
7911	2.5852	28.2235	280.703
7912	2.5853	27.757	139.426
7913	2.5855	-2.9266	72.5631
7914	2.5855	25.1698	224.711
7915	2.5856	24.8247	257.872
7916	2.5856	-2.8403	292.983
7917	2.5857	19.5051	67.6998
7918	2.5858	25.5669	5.2729
7919	2.5859	26.6864	358.588
7920	2.5861	-3.0133	273.448
7921	2.5862	-4.5048	56.8141
7922	2.5862	-2.7568	63.8924
7923	2.5863	-3.0131	89.3802
7924	2.5865	-3.1701	177.948
7925	2.5867	28.0315	163.461
7926	2.5868	-3.4137	82.9838
7927	2.5868	-3.2615	105.524
7928	2.5868	-3.7755	244.847
7929	2.5872	-3.2497	296.433
7930	2.5876	30.7843	133.333
7931	2.5876	24.8683	114.69
7932	2.5878	24.2071	128.292
7933	2.5881	-3.1369	296.149
7934	2.5883	-2.8764	265.485
7935	2.5885	27.9012	250.51
7936	2.5885	18.1475	236.087
7937	2.5894	-3.2334	347.083
7938	2.5896	-3.2722	207
7939	2.5896	-3.4383	266.048
7940	2.5899	-2.8713	63.6893
7941	2.5899	-2.7918	8.0468
7942	2.5904	-3.0519	55.7044
7943	2.5904	-2.8134	113.923
7944	2.5904	22.0296	194.047
7945	2.5905	24.2049	155.496
7946	2.5912	20.4729	129.089
7947	2.5912	20.378	319.981
7948	2.5913	17.6227	128.811
7949	2.5915	-3.8353	159.149
7950	2.5917	27.8425	33.5821
7951	2.5918	-3.6631	229.285
7952	2.5918	-4.1656	352.088
7953	2.5919	-2.8433	97.6979
7954	2.5919	23.6407	87.1416
7955	2.5919	-2.9578	43.001
7956	2.592	-3.6816	316.204
7957	2.5921	-3.8746	111.991

7958	2.5922	-2.8886	142.829
7959	2.5923	-2.8066	17.661
7960	2.5923	-2.8172	56.1346
7961	2.5927	-2.9208	181.979
7962	2.5927	23.8753	112.521
7963	2.5927	27.545	57.9709
7964	2.5929	-3.1114	102.354
7965	2.5931	33.9258	333.132
7966	2.5932	28.6252	66.8165
7967	2.5934	-2.7525	38.9122
7968	2.5934	23.6608	236.966
7969	2.5936	23.0748	80.2439
7970	2.594	27.0347	31.1899
7971	2.5942	27.132	77.3264
7972	2.5942	-3.8635	165.037
7973	2.5943	21.6179	139.721
7974	2.5944	-3.8923	210.807
7975	2.5944	-2.8175	315.205
7976	2.5944	-2.8592	335.66
7977	2.5945	12.1899	320.31
7978	2.5946	24.5685	64.8572
7979	2.595	31.6731	81.8169
7980	2.5951	-4.1804	331.523
7981	2.5952	21.678	131.173
7982	2.5953	21.3527	17.3733
7983	2.5955	-2.8075	81.4798
7984	2.5956	-2.9098	117.863
7985	2.5956	14.2706	304.927
7986	2.5957	-3.9091	135.631
7987	2.5958	28.4718	219.32
7988	2.5959	-2.7424	101.377
7989	2.5966	24.2455	292.991
7990	2.5967	26.273	79.5406
7991	2.5967	-2.8422	100.938
7992	2.5968	28.3819	325.032
7993	2.5969	21.9621	272.098
7994	2.597	-2.9711	24.9491
7995	2.5971	33.7278	205.535
7996	2.5972	22.5986	37.723
7997	2.5972	44.4058	200.891
7998	2.5975	-2.7234	92.6643
7999	2.5981	-3.5475	19.435
8000	2.5982	-3.2523	85.3405
8001	2.5984	22.0328	195.986
8002	2.5984	-2.8502	309.342
8003	2.5985	-2.8763	42.6345
8004	2.599	30.6841	17.7931
8005	2.5991	-2.9649	23.2331

8006	2.5995	32.6789	211.018
8007	2.5999	26.8669	297.419
8008	2.5999	-3.451	330.258
8009	2.6003	14.6797	136.182
8010	2.6003	-3.4272	273.05
8011	2.6003	-3.147	329.244
8012	2.6004	-2.9782	106.503
8013	2.6006	-4.1918	169.232
8014	2.6006	-2.9553	213.006
8015	2.6006	-3.3204	328.776
8016	2.6008	22.3824	171.757
8017	2.6009	-3.0694	354.333
8018	2.601	29.0757	242.056
8019	2.601	54.842	63.1438
8020	2.6015	26.2192	250.102
8021	2.6017	27.9171	37.4636
8022	2.6019	23.2909	318.427
8023	2.6022	25.7246	153.099
8024	2.6024	-3.3121	165.811
8025	2.6024	-2.8134	51.4759
8026	2.6025	17.8948	320.984
8027	2.6026	-4.4008	74.4921
8028	2.6026	24.0581	358.448
8029	2.6026	-2.8014	216.055
8030	2.6028	-2.8702	259.389
8031	2.6029	-3.0737	251.103
8032	2.6029	-3.0663	243.019
8033	2.603	36.1208	241.157
8034	2.6032	29.2535	244.744
8035	2.6033	21.3816	22.2703
8036	2.6035	31.0864	28.9723
8037	2.6037	-3.6869	260.847
8038	2.6037	-2.7016	34.4637
8039	2.6041	27.4214	39.404
8040	2.6041	-3.1058	328.224
8041	2.6043	-4.0323	129.555
8042	2.6049	-2.704	134.034
8043	2.6049	-2.8153	32.7139
8044	2.6051	-2.9374	249.123
8045	2.6051	33.0643	47.0209
8046	2.6051	-2.9285	168.673
8047	2.6052	24.518	297.044
8048	2.6054	-3.4927	73.1583
8049	2.6054	-2.8795	95.4412
8050	2.6054	12.3262	148.617
8051	2.6057	-3.8637	160.878
8052	2.6058	25.0127	93.8527
8053	2.6059	25.5961	27.101

8054	2.6062	-2.7391	59.5349
8055	2.6065	24.8647	160.798
8056	2.6066	21.8055	326.815
8057	2.6068	28.1901	305.971
8058	2.6069	-2.9281	317.402
8059	2.6074	28.7529	291.948
8060	2.6074	28.5461	232.152
8061	2.6077	-2.1699	45.2
8062	2.6078	27.9131	197.253
8063	2.6078	-2.8506	340.164
8064	2.6078	-2.8906	339.504
8065	2.6081	-2.9401	358.667
8066	2.6081	25.1732	321.274
8067	2.6081	-2.9418	299.37
8068	2.6084	-3.1739	339.852
8069	2.6084	-3.2332	262.935
8070	2.6085	-3.386	353.889
8071	2.6089	18.9684	336.511
8072	2.609	-2.7205	191.141
8073	2.6092	-3.4372	256.438
8074	2.6095	22.7317	259.681
8075	2.6098	-3.1703	172.941
8076	2.6101	27.3826	225.247
8077	2.6104	17.5231	104.211
8078	2.6105	19.3034	196.643
8079	2.6107	-3.9393	257.184
8080	2.6109	15.4341	171.623
8081	2.611	29.7892	297.715
8082	2.611	46.3013	317.384
8083	2.611	-3.4614	334.522
8084	2.6112	-4.5951	66.6257
8085	2.6112	-2.8159	341.079
8086	2.6113	-3.8638	73.7828
8087	2.6113	36.4002	33.9989
8088	2.6114	-2.9597	252.726
8089	2.6114	27.6846	230.57
8090	2.6115	26.1381	228.195
8091	2.6116	-2.953	284.263
8092	2.6116	24.9057	129.881
8093	2.6116	29.9223	193.122
8094	2.6118	-2.9483	47.2204
8095	2.6119	-3.3317	100.075
8096	2.6128	-3.8264	274.27
8097	2.6128	23.9311	23.4998
8098	2.613	20.8606	274.788
8099	2.613	29.3663	120.647
8100	2.6131	-3.1726	150.533
8101	2.6132	-3.2193	170.445

8102	2.6133	-2.921	350.488
8103	2.6142	-3.4547	100.105
8104	2.6142	-2.8133	349.877
8105	2.6142	24.4777	28.7756
8106	2.6144	-3.9438	174.511
8107	2.6144	-2.8365	192.456
8108	2.6145	22.8886	296.213
8109	2.6149	21.5089	23.3116
8110	2.6151	-4.0488	259.182
8111	2.6151	-4.2151	165.85
8112	2.6156	39.9845	317.362
8113	2.6156	-3.8921	139.436
8114	2.6157	-3.5601	234.335
8115	2.6159	30.9189	138.827
8116	2.6159	23.8655	88.7334
8117	2.6159	-2.9715	157.61
8118	2.6162	31.1081	129.855
8119	2.6165	-2.9783	357.291
8120	2.6165	21.0885	304.468
8121	2.6166	-2.9266	211.59
8122	2.6167	28.4992	124.983
8123	2.6167	-2.8104	251.219
8124	2.6168	34.1729	168.111
8125	2.6169	23.9565	218.311
8126	2.6172	-2.6661	102.255
8127	2.6173	-3.0058	16.2429
8128	2.6173	30.1581	35.3478
8129	2.618	26.5139	230.98
8130	2.6181	-2.8685	91.8251
8131	2.6183	-2.9584	284.467
8132	2.6183	-2.8803	340.682
8133	2.6184	-3.2223	265.183
8134	2.6184	29.5047	29.4772
8135	2.6185	10.383	36.8722
8136	2.6188	20.2809	122.911
8137	2.6191	-2.9611	34.2403
8138	2.6195	-2.8766	79.3106
8139	2.6195	16.647	129.734
8140	2.6195	22.9859	177.866
8141	2.6196	-2.9453	280.83
8142	2.6197	-3.4597	179.016
8143	2.6198	15.8987	161.958
8144	2.6199	29.631	100.449
8145	2.6199	31.9976	344.603
8146	2.62	-3.9267	74.4579
8147	2.6202	-2.6921	40.1991
8148	2.6203	-3.819	266.69
8149	2.6203	-3.3412	213.066

8150	2.6205	-3.0046	79.2531
8151	2.6207	-2.7084	253.524
8152	2.6208	27.7416	95.4114
8153	2.6208	-2.9317	20.3558
8154	2.6211	22.0664	171.347
8155	2.6213	33.1426	311.138
8156	2.6214	-2.8784	209.218
8157	2.6215	-2.925	111.345
8158	2.6215	-2.7942	124.776
8159	2.6215	26.8994	223.191
8160	2.6217	17.536	102.181
8161	2.6218	28.7849	38.3388
8162	2.6218	24.1918	120.983
8163	2.622	-2.6665	243.33
8164	2.6221	-2.8648	110.819
8165	2.6221	25.0301	291.316
8166	2.6221	26.7594	185.389
8167	2.6223	25.2147	163.79
8168	2.6223	22.5661	333.938
8169	2.6224	29.0943	211.022
8170	2.6225	26.7938	0.2752
8171	2.6225	-2.8345	257.548
8172	2.6227	30.8788	201.899
8173	2.6228	18.5904	300.877
8174	2.6229	-3.8025	129.313
8175	2.6229	24.2401	322.369
8176	2.6231	-2.7886	163.059
8177	2.6231	27.2634	12.3042
8178	2.6232	27.7943	89.4009
8179	2.6233	33.5191	209.138
8180	2.6233	-3.4617	301.835
8181	2.6234	25.47	78.5425
8182	2.6234	-2.8136	279.776
8183	2.6234	27.366	129.693
8184	2.6234	25.8397	263.817
8185	2.6238	-4.0159	151.401
8186	2.624	-3.7103	284.201
8187	2.6242	-2.9358	76.2702
8188	2.6243	-2.8236	169.041
8189	2.6246	-3.1779	90.1619
8190	2.6247	-2.7879	196.798
8191	2.6247	25.0071	218.891
8192	2.6248	-3.2791	6.9838
8193	2.6249	14.4657	234.932
8194	2.6254	20.5381	103.759
8195	2.6255	31.1708	244.367
8196	2.6256	-0.2153	317.397
8197	2.6257	20.6572	303.529

8198	2.6258	15.6711	274.587
8199	2.6262	-2.8943	37.4519
8200	2.6262	-3.2923	315.156
8201	2.6264	-3.8779	200.757
8202	2.6265	-2.827	200.615
8203	2.6265	-3.0032	232.628
8204	2.6268	-2.8001	271.901
8205	2.6268	-3.3249	237.541
8206	2.6269	31.0932	104.165
8207	2.6272	26.2671	249.696
8208	2.6273	-3.0713	110.812
8209	2.6277	23.6153	158.119
8210	2.6278	35.2429	202.645
8211	2.6278	-2.7646	357.941
8212	2.6279	30.4903	290.313
8213	2.628	32.2699	171.284
8214	2.628	34.0711	349.356
8215	2.628	-4.2108	33.9861
8216	2.6282	23.5201	0.1665
8217	2.6282	-2.7818	287.483
8218	2.6286	-3.2297	334.047
8219	2.6286	26.5875	256.38
8220	2.6287	32.5134	174.563
8221	2.6287	-2.7864	317.137
8222	2.6289	-3.9825	185.541
8223	2.6293	20.9745	327.321
8224	2.6294	-2.9541	21.8633
8225	2.6294	9.8695	177.237
8226	2.6295	19.1362	236.05
8227	2.6295	28.137	229.834
8228	2.6295	22.627	315.245
8229	2.6295	-3.6432	165.866
8230	2.6296	24.4648	224.522
8231	2.6299	-3.4624	180.125
8232	2.63	-2.9537	329.414
8233	2.6301	32.2066	75.0336
8234	2.6302	-4.5265	135.576
8235	2.6304	-4.0657	88.8545
8236	2.6304	-2.8777	332.199
8237	2.6309	27.9165	66.9214
8238	2.631	-2.936	243.559
8239	2.631	17.5004	230.608
8240	2.6313	21.9719	22.1729
8241	2.6316	-2.8452	233.152
8242	2.6319	-2.7087	323.552
8243	2.6321	38.0082	225.822
8244	2.6323	15.4427	265.162
8245	2.6323	-3.1723	251.842

8246	2.6324	28.845	211.764
8247	2.633	-2.864	107.995
8248	2.633	25.5909	157.888
8249	2.633	21.8828	199.185
8250	2.6331	-3.169	26.2864
8251	2.6332	-3.8639	94.5284
8252	2.6333	-2.7705	357.449
8253	2.6335	-3.9611	164.934
8254	2.634	27.1797	85.6109
8255	2.6342	-3.9259	289.56
8256	2.6343	23.0722	264.513
8257	2.6345	-2.8858	30.8339
8258	2.6346	34.9505	204.909
8259	2.6346	26.9916	109.061
8260	2.6348	-3.6663	307.125
8261	2.6349	24.3123	268.494
8262	2.6349	-3.0215	115.224
8263	2.6351	-2.8326	129.739
8264	2.6351	-4.0114	313.953
8265	2.6352	-2.7552	149.655
8266	2.6353	-2.8642	59.2311
8267	2.6356	29.8219	150.749
8268	2.6358	26.9194	88.8353
8269	2.6358	24.7295	234.421
8270	2.636	-3.3922	275.883
8271	2.6362	21.1781	150.771
8272	2.6362	20.1978	99.7368
8273	2.6363	22.6374	187.384
8274	2.6363	26.9109	83.8542
8275	2.6364	-2.9858	325.146
8276	2.6366	-2.8917	78.1083
8277	2.6367	-3.7834	122.08
8278	2.6367	-3.5381	331.771
8279	2.6368	39.94	154.267
8280	2.6368	-2.9739	30.1276
8281	2.6369	24.6152	184.56
8282	2.6369	-3.3795	30.3923
8283	2.6372	-2.8097	79.2208
8284	2.6373	-3.268	205.969
8285	2.6373	-2.9162	168.306
8286	2.6377	22.191	241.316
8287	2.6378	-4.2697	286.633
8288	2.6379	26.513	16.6375
8289	2.638	-3.4048	178.74
8290	2.6381	21.5494	107.746
8291	2.6381	-3.2566	247.24
8292	2.6384	29.9967	59.5814
8293	2.6384	-3.3704	34.3188

8294	2.6384	-2.8924	329.712
8295	2.6387	-3.9989	130.433
8296	2.639	32.9619	258.923
8297	2.6391	-2.7501	67.5938
8298	2.6391	-2.7896	359.983
8299	2.6392	-2.7044	31.9079
8300	2.6394	30.4362	173.615
8301	2.6395	-2.8493	225.529
8302	2.6396	-2.8716	95.5016
8303	2.6399	-2.8971	119.839
8304	2.6401	-3.8756	181.87
8305	2.6402	21.2103	251.138
8306	2.6403	-3.0982	105.533
8307	2.6403	19.7118	150.507
8308	2.6403	24.8983	121.043
8309	2.6407	16.8727	50.1351
8310	2.6407	25.615	55.9557
8311	2.6409	28.1633	261.133
8312	2.6409	38.9936	186.311
8313	2.641	-4.1004	127.743
8314	2.6412	14.8305	300.811
8315	2.6413	-2.7877	353.673
8316	2.6414	-2.771	324.96
8317	2.6415	-2.9809	327.701
8318	2.6416	20.5522	193.828
8319	2.6417	-2.9586	280.465
8320	2.6417	18.3203	98.9139
8321	2.6418	28.1029	233.72
8322	2.6419	-3.3436	158.134
8323	2.6419	-4.0042	18.3109
8324	2.6421	17.0267	162.067
8325	2.6422	32.8424	156.998
8326	2.6424	-4.5026	337.475
8327	2.6425	0.3662	251.964
8328	2.6425	18.2114	271.037
8329	2.6426	26.7542	214.765
8330	2.643	26.6992	281.941
8331	2.643	-2.8557	170.889
8332	2.6431	-2.7211	51.0344
8333	2.6435	-3.7739	32.1567
8334	2.6437	23.6375	158.693
8335	2.6437	37.7117	50.8182
8336	2.6438	34.4416	326.208
8337	2.644	33.7385	269.902
8338	2.6442	29.0243	103.782
8339	2.6445	33.5542	26.9367
8340	2.6447	20.4389	311.105
8341	2.6448	25.472	5.3863

8342	2.645	-3.3765	92.273
8343	2.645	20.2264	200.858
8344	2.6451	-2.7469	359.971
8345	2.6453	28.4125	210.807
8346	2.6456	-3.5309	88.9247
8347	2.6456	-2.7492	116.497
8348	2.6458	25.2846	356.49
8349	2.6459	28.8871	331.317
8350	2.6461	-3.1146	79.6952
8351	2.6461	16.1339	86.6046
8352	2.6461	-3.953	187.977
8353	2.6461	50.0891	288.503
8354	2.6463	37.4457	0.5736
8355	2.6465	24.9509	25.2679
8356	2.6465	23.8978	40.6486
8357	2.6467	-2.9365	142.383
8358	2.647	-2.7732	122.876
8359	2.6472	29.2889	123.143
8360	2.6472	32.4461	37.1108
8361	2.6475	25.1367	320.96
8362	2.6475	-3.1949	138.681
8363	2.6475	22.1511	350.95
8364	2.6479	-3.63	81.8422
8365	2.6482	-4.3746	288.581
8366	2.6482	30.512	170.534
8367	2.6483	-3.8354	65.5861
8368	2.6485	-3.0682	268.173
8369	2.6486	23.9853	266.656
8370	2.6488	34.9672	34.9034
8371	2.649	-3.0688	347.364
8372	2.6492	-3.2946	329.266
8373	2.6493	-3.2063	331.071
8374	2.6493	-2.8435	114.505
8375	2.6495	28.9444	156.159
8376	2.6495	25.306	210.322
8377	2.6496	-2.7744	90.1332
8378	2.6496	-4.0564	295.213
8379	2.6497	32.1068	207.873
8380	2.65	26.2752	125.543
8381	2.6503	-2.8888	30.4631
8382	2.6503	31.4573	70.7869
8383	2.6504	23.9204	257.194
8384	2.6504	-2.7071	297.452
8385	2.6509	-3.2535	53.2391
8386	2.6511	-2.9611	332.609
8387	2.6512	-2.9593	188.784
8388	2.6513	28.3796	316.881
8389	2.6513	-2.7982	138.751

8390	2.6514	-2.8362	287.754
8391	2.6515	21.1774	185.106
8392	2.6515	22.4189	121.682
8393	2.6515	38.8624	266.878
8394	2.6516	25.5589	23.3563
8395	2.6519	-3.216	320.517
8396	2.6519	-2.8895	263.387
8397	2.652	22.2322	137.512
8398	2.652	-2.7939	194.77
8399	2.652	27.891	226.271
8400	2.6521	25.1405	215.117
8401	2.6522	23.0439	320.136
8402	2.6523	-3.1632	302.293
8403	2.6525	-4.6119	306.954
8404	2.6526	-2.6921	35.2765
8405	2.6527	-2.9751	260.32
8406	2.6527	20.7856	185.752
8407	2.6528	-3.8362	268.327
8408	2.6528	25.3024	81.3028
8409	2.6531	-2.8569	348.241
8410	2.6531	36.7987	109.932
8411	2.6533	-3.0324	316.255
8412	2.6533	20.6131	155.485
8413	2.6533	-2.8082	138.075
8414	2.6535	16.6995	3.9665
8415	2.6536	23.7652	173.28
8416	2.6539	31.7824	311.681
8417	2.6539	29.3823	294.773
8418	2.654	-3.9082	97.0355
8419	2.6541	-2.6942	135.997
8420	2.6541	23.1829	4.4728
8421	2.6542	-3.1277	260.169
8422	2.6542	31.6321	290.363
8423	2.6545	15.7439	293.662
8424	2.6547	27.3254	66.5848
8425	2.6549	-2.859	308.633
8426	2.6549	-2.6972	211.842
8427	2.655	-2.9647	260.268
8428	2.6551	28.8229	85.0577
8429	2.6551	34.2811	160.776
8430	2.6552	-2.8854	216.094
8431	2.6557	-2.861	260.895
8432	2.6561	23.9	122.905
8433	2.6562	-3.4074	126.414
8434	2.6562	-2.8804	196.556
8435	2.6563	19.0089	179.098
8436	2.6565	-2.8207	39.2543
8437	2.6567	51.8585	312.741

8438	2.6567	-3.7922	307.795
8439	2.6568	27.9471	235.613
8440	2.6568	-3.1347	320.964
8441	2.6569	-3.6324	233.225
8442	2.6569	-2.9619	57.3054
8443	2.6571	21.0602	125.429
8444	2.6581	-3.1715	255.491
8445	2.6585	-2.688	327.1
8446	2.6588	-2.9901	352.187
8447	2.6588	-3.1507	57.3216
8448	2.659	-2.6747	150.988
8449	2.659	29.4852	16.0952
8450	2.6592	34.4848	23.2684
8451	2.6592	-2.7645	111.459
8452	2.6593	30.1388	174.704
8453	2.6593	-2.9439	103.937
8454	2.6596	-2.8768	49.634
8455	2.6599	-3.83	35.8385
8456	2.6599	-3.1114	147.278
8457	2.66	-3.8622	46.6307
8458	2.6602	-2.9736	197.834
8459	2.6602	22.3018	120.085
8460	2.6604	27.7696	39.4929
8461	2.6604	21.9725	242.963
8462	2.6606	22.6349	243.461
8463	2.6607	29.4537	7.1361
8464	2.6608	25.0228	198.207
8465	2.6609	-2.8804	317.804
8466	2.661	26.8239	169.737
8467	2.6612	19.5006	86.0245
8468	2.6614	17.6454	5.6135
8469	2.6615	32.5514	30.5644
8470	2.6615	18.6714	56.8833
8471	2.6616	-3.2466	359.763
8472	2.6619	22.5324	293.735
8473	2.662	-3.3235	331.042
8474	2.6621	28.7409	206.957
8475	2.6622	-4.2342	258.276
8476	2.6622	-2.8022	309.625
8477	2.6622	32.4973	57.0576
8478	2.6623	-3.0908	102.093
8479	2.6623	31.0349	311.685
8480	2.6624	-3.0049	324.867
8481	2.6624	-2.7369	112.849
8482	2.6624	-3.1855	152.686
8483	2.6625	27.0322	288.816
8484	2.6626	29.4389	211.397
8485	2.6627	46.9774	305.715

8486	2.6627	28.897	242.126
8487	2.6628	30.4454	241.142
8488	2.6632	-3.156	18.099
8489	2.6632	-2.8319	73.9193
8490	2.6632	-3.4116	339.377
8491	2.6633	44.8369	102.148
8492	2.6635	-3.4433	6.7796
8493	2.6635	29.8001	20.2307
8494	2.6636	-2.7749	119.554
8495	2.6637	37.2336	126.448
8496	2.6638	44.1204	202.396
8497	2.6638	-2.7867	47.044
8498	2.6639	-2.9299	229.562
8499	2.6639	29.257	252.199
8500	2.664	-3.2707	334.337
8501	2.664	-3.1359	124.716
8502	2.6641	34.295	86.6201
8503	2.6643	-2.8134	111.597
8504	2.6645	-2.9369	67.6639
8505	2.6648	23.4508	357.324
8506	2.6649	35.5403	81.4856
8507	2.6652	33.7936	37.2924
8508	2.6655	35.6847	137.101
8509	2.6657	21.208	255.395
8510	2.6657	22.2336	176.393
8511	2.666	30.5584	327.91
8512	2.6661	-4.3715	344.134
8513	2.6662	26.4333	46.7462
8514	2.6662	-3.8607	118.746
8515	2.6663	33.9211	222.365
8516	2.6663	21.6174	172.43
8517	2.6665	-2.7633	243.967
8518	2.6666	25.5702	184.822
8519	2.6667	22.4792	62.2457
8520	2.6668	23.748	137.435
8521	2.6668	-2.8675	302.726
8522	2.667	-2.9339	78.119
8523	2.6671	-3.0317	306.319
8524	2.6673	34.5173	124.771
8525	2.6674	-2.9489	348.69
8526	2.6674	39.8235	199.321
8527	2.6674	23.35	78.0575
8528	2.6676	-3.0832	34.7578
8529	2.6676	-4.4878	117.572
8530	2.6677	20.9146	115.587
8531	2.6678	28.7929	239.635
8532	2.6678	-2.8499	150.127
8533	2.6678	34.2385	67.9864

8534	2.6678	50.0251	117.366
8535	2.668	-3.1495	238.643
8536	2.668	33.5409	64.0818
8537	2.6682	-2.9397	245.362
8538	2.6683	32.5052	304.376
8539	2.6684	-2.7323	233.871
8540	2.6685	24.7115	211.416
8541	2.6685	-3.0742	299.483
8542	2.6685	22.8425	334.394
8543	2.6685	-2.689	151.476
8544	2.6686	29.7151	98.3116
8545	2.669	-2.8454	23.8248
8546	2.6692	-3.749	16.5288
8547	2.6694	-3.9953	111.445
8548	2.6694	-2.9729	296.485
8549	2.6695	28.8992	261.317
8550	2.6698	27.4752	216.651
8551	2.6699	-2.8616	141.451
8552	2.67	-4.2146	315.013
8553	2.6703	-3.6704	153.732
8554	2.6705	-2.7967	185.788
8555	2.6707	-3.8812	342.99
8556	2.6707	-2.8855	343.633
8557	2.6711	-3.8586	322.556
8558	2.6712	-2.7125	288.198
8559	2.6714	-2.9878	353.906
8560	2.6718	23.6163	329.92
8561	2.672	0.8277	215.244
8562	2.6722	27.2678	233.511
8563	2.6723	-2.7442	141.087
8564	2.6724	-4.7139	33.4085
8565	2.6725	-3.9912	350.801
8566	2.673	28.5314	190.56
8567	2.6731	-2.7057	154.251
8568	2.6733	-2.9509	9.3906
8569	2.6737	21.0153	275.1
8570	2.6737	20.1008	257.549
8571	2.6738	21.3632	203.159
8572	2.6738	-3.4124	333.945
8573	2.6739	-3.0548	247.045
8574	2.674	-2.7999	187.602
8575	2.6741	-2.9121	45.3587
8576	2.6742	-2.8207	304.074
8577	2.6743	39.0706	134.578
8578	2.6744	-3.2487	61.6801
8579	2.6744	27.8586	89.7513
8580	2.6745	-3.0799	339.055
8581	2.6747	-2.9524	166.841

8582	2.675	-2.9573	332.829
8583	2.6751	-3.3897	40.459
8584	2.6751	-3.3008	23.4401
8585	2.6752	-4.2957	278.305
8586	2.6753	25.813	346.277
8587	2.6754	-2.9635	279.575
8588	2.6755	-3.7479	148.934
8589	2.6755	-3.505	52.8512
8590	2.6756	30.2968	34.5521
8591	2.6761	23.0738	278.248
8592	2.6762	31.7314	48.8629
8593	2.6762	27.6683	192.379
8594	2.6762	-2.7494	8.7651
8595	2.6764	25.946	140.74
8596	2.6767	39.3473	338.403
8597	2.6767	26.4037	35.3134
8598	2.6771	-3.4553	329.807
8599	2.6773	28.7444	223.597
8600	2.6774	-4.4774	235.237
8601	2.6775	-3.0044	277.998
8602	2.6776	42.2435	62.3131
8603	2.6776	23.2112	302.408
8604	2.6777	17.7057	159.823
8605	2.6779	-2.9864	280.931
8606	2.6779	-3.9832	337.973
8607	2.6781	-2.7082	341.361
8608	2.6781	31.1128	122.196
8609	2.6782	-2.8249	24.1991
8610	2.6783	33.1665	59.6549
8611	2.6783	-4.2463	205.704
8612	2.6783	22.1493	317.068
8613	2.6787	28.4412	61.1932
8614	2.6787	-3.1534	195.013
8615	2.6788	35.5982	110.598
8616	2.6789	29.1012	308.519
8617	2.6791	-2.7668	236.815
8618	2.6792	-3.3929	325.486
8619	2.6794	27.1716	348.556
8620	2.6794	28.4702	306.381
8621	2.6795	30.5434	89.0305
8622	2.6797	23.0059	156.642
8623	2.6799	28.4998	166.735
8624	2.68	-3.3854	256.095
8625	2.68	-3.0846	207.247
8626	2.6801	-2.8918	297.42
8627	2.6802	23.8863	346.561
8628	2.6802	-3.0005	108.647
8629	2.6805	24.0404	216.206

8630	2.6805	27.0933	197.578
8631	2.6808	-2.7502	338.752
8632	2.6808	27.0421	291.846
8633	2.6811	-3.8753	203.523
8634	2.6813	-4.2824	111.573
8635	2.6813	28.9851	288.748
8636	2.6814	29.2448	63.6995
8637	2.6814	-4.121	155.815
8638	2.6815	18.5543	267.706
8639	2.6815	29.5936	270.308
8640	2.6817	25.2724	183.489
8641	2.682	33.793	238.745
8642	2.6821	-2.7887	249.7
8643	2.6821	-2.7084	79.5458
8644	2.6823	30.9937	197.767
8645	2.6827	26.6458	46.1524
8646	2.6831	-4.1374	260.351
8647	2.6833	27.2404	121.202
8648	2.6834	22.6371	179.429
8649	2.6834	27.6536	24.6366
8650	2.6838	34.498	33.3919
8651	2.6839	23.2551	144.872
8652	2.6839	-2.8151	180.72
8653	2.6842	14.4001	270.995
8654	2.6842	33.38	187.098
8655	2.6845	-2.8872	259.597
8656	2.6846	-2.8672	241.759
8657	2.685	20.9307	134.027
8658	2.685	44.2437	28.7047
8659	2.6851	37.6775	295.11
8660	2.6851	-3.5858	252.347
8661	2.6852	-2.9164	87.2845
8662	2.6853	-3.2566	64.0086
8663	2.6853	-4.2964	159.625
8664	2.6855	-3.4012	108.214
8665	2.6856	26.468	276.445
8666	2.6856	23.5913	120.705
8667	2.686	22.3501	248.495
8668	2.686	25.6284	344.426
8669	2.686	-2.929	52.1072
8670	2.6864	-2.978	324.834
8671	2.6864	-2.9848	12.36
8672	2.6864	31.03	251.187
8673	2.6864	24.597	169.029
8674	2.6864	29.5459	115.266
8675	2.6865	24.6411	97.434
8676	2.6866	-2.7775	141.315
8677	2.6869	-2.839	271.978

8678	2.6869	-2.8019	312.83
8679	2.6869	-4.0944	338.57
8680	2.6869	29.3822	265.873
8681	2.6871	-2.956	97.6194
8682	2.6871	40.7471	201.722
8683	2.6875	21.8314	213.935
8684	2.6875	21.7208	322.078
8685	2.6875	29.8251	212.461
8686	2.6877	16.3346	166.558
8687	2.6878	-2.8371	149.092
8688	2.6878	33.0494	100.55
8689	2.688	28.5538	209.871
8690	2.688	-2.7627	283.023
8691	2.6882	-4.1902	256.03
8692	2.6883	29.9228	145.14
8693	2.6884	-3.0658	117.84
8694	2.6885	25.1179	183.608
8695	2.6885	-4.0097	257.075
8696	2.6886	25.0924	154.767
8697	2.689	-3.9213	167.756
8698	2.6892	24.1258	298.599
8699	2.6894	29.6481	319.985
8700	2.6897	26.5186	275.826
8701	2.6897	-3.7743	337.562
8702	2.6897	7.0812	21.0745
8703	2.6898	-2.7116	165.437
8704	2.6899	-2.7364	207.669
8705	2.6899	34.1285	42.6163
8706	2.69	-2.9598	236.502
8707	2.69	30.933	333.384
8708	2.69	-4.0326	335.945
8709	2.6901	38.5673	58.4823
8710	2.6901	27.7607	172.234
8711	2.6902	32.9669	30.8454
8712	2.6904	-3.555	323.427
8713	2.6911	-3.6089	273.607
8714	2.6913	19.8768	206.444
8715	2.6917	23.943	187.023
8716	2.6919	28.3262	121.566
8717	2.692	-2.9469	329.748
8718	2.6921	34.2678	162.842
8719	2.6921	28.8351	147.295
8720	2.6927	36.2353	312.09
8721	2.693	33.0526	178.872
8722	2.693	-3.3816	39.8411
8723	2.693	3.0579	81.6329
8724	2.693	-3.4405	135.619
8725	2.6933	-2.7101	33.3882

8726	2.6934	17.2319	7.5864
8727	2.6934	26.306	106.633
8728	2.6934	-3.2374	267.383
8729	2.6934	-2.7122	35.6374
8730	2.6934	29.6104	32.3911
8731	2.6935	-3.8122	33.5368
8732	2.6941	-4.1304	14.0428
8733	2.6943	27.8968	302.811
8734	2.6943	25.2946	170.659
8735	2.6944	-2.6947	240.164
8736	2.6944	28.1936	337.271
8737	2.6946	34.8973	207.021
8738	2.6949	26.4361	359.643
8739	2.6951	-4.0765	213.068
8740	2.6951	30.4272	214.055
8741	2.6952	-2.8921	269.665
8742	2.6952	-3.103	205.361
8743	2.6953	-2.7331	190.784
8744	2.6954	17.6196	48.9405
8745	2.6955	34.5507	14.9048
8746	2.6955	22.8965	254.3
8747	2.6956	-2.8405	253.501
8748	2.6958	32.9269	171.432
8749	2.6958	29.1699	175.369
8750	2.696	33.2813	264.946
8751	2.6964	-3.7568	92.8379
8752	2.6964	27.0161	64.8639
8753	2.6965	-3.234	332.483
8754	2.6967	35.6771	137.934
8755	2.6968	-3.695	156.411
8756	2.697	-3.189	51.8363
8757	2.697	18.3551	227.27
8758	2.697	-3.4743	103.089
8759	2.6972	25.8668	60.3356
8760	2.6974	-3.9248	142.89
8761	2.6975	28.0243	38.739
8762	2.6977	34.6509	302.269
8763	2.6978	27.1122	226.323
8764	2.6982	-2.8238	230.053
8765	2.6988	-2.9803	9.5082
8766	2.6989	24.4601	253.986
8767	2.699	41.8797	202.826
8768	2.6992	19.8141	326.52
8769	2.6993	36.0611	61.3199
8770	2.6994	30.8219	139.651
8771	2.6996	-3.3177	328.773
8772	2.6998	-3.042	22.7061
8773	2.6998	33.7883	270.501

8774	2.7002	-2.7923	87.0586
8775	2.7003	34.0918	52.1467
8776	2.7003	23.9922	200.173
8777	2.7006	-2.9596	158.661
8778	2.7006	28.8906	142.678
8779	2.7009	-5.2663	47.3577
8780	2.7011	28.2284	138.013
8781	2.7011	34.8016	148.143
8782	2.7013	21.6236	236.83
8783	2.7017	-3.9643	48.2517
8784	2.7018	30.1906	61.0286
8785	2.702	27.338	237.437
8786	2.7021	-3.4989	163.15
8787	2.7023	43.5518	86.4496
8788	2.7023	-2.7738	75.7874
8789	2.7024	26.6735	102.277
8790	2.7025	-3.2688	246.177
8791	2.7025	-3.9103	174.263
8792	2.7025	-2.8495	211.083
8793	2.7025	-3.9404	355.866
8794	2.7027	-3.0368	254.871
8795	2.7028	-2.9297	259.803
8796	2.7031	36.6916	39.5355
8797	2.7031	22.5843	327.968
8798	2.7031	37.2052	21.7139
8799	2.7034	25.134	161.034
8800	2.7034	38.0366	130.353
8801	2.7039	25.5002	340.166
8802	2.7041	-2.9419	93.9466
8803	2.7042	-3.2115	259.376
8804	2.7043	24.8161	323.065
8805	2.7043	40.6895	246.441
8806	2.7043	-2.9014	257.253
8807	2.7045	46.8735	128.13
8808	2.7046	-2.7695	38.0063
8809	2.7046	-3.6735	145.396
8810	2.7047	-2.889	22.5681
8811	2.7047	-2.741	9.8795
8812	2.7049	34.7028	351.262
8813	2.7049	35.2509	23.2393
8814	2.705	37.2649	61.2343
8815	2.7051	-2.8362	211.589
8816	2.7052	-2.9668	143.266
8817	2.7054	-4.0307	256.83
8818	2.7054	-3.1127	95.198
8819	2.7058	-3.6017	117.143
8820	2.706	37.966	208.795
8821	2.7062	-4.0107	339.405

8822	2.7062	30.628	219.177
8823	2.7064	33.8163	209.583
8824	2.7064	-2.9645	23.8147
8825	2.7065	-3.2694	20.0475
8826	2.7068	26.8985	28.8667
8827	2.7068	29.7829	134.314
8828	2.707	-2.919	339.361
8829	2.7071	24.3549	241.911
8830	2.7073	23.4494	101.637
8831	2.7074	-2.7857	8.5186
8832	2.7076	27.48	26.0055
8833	2.7077	20.048	175.975
8834	2.708	24.3483	157.829
8835	2.7081	-2.8018	118.328
8836	2.7083	25.2819	303.292
8837	2.7083	27.3574	281.227
8838	2.7084	-2.793	326.356
8839	2.7085	-2.949	172.175
8840	2.7085	30.7043	218.804
8841	2.7087	-2.7934	143.729
8842	2.709	-2.9346	22.4308
8843	2.7092	28.5523	189.18
8844	2.7093	37.5551	186.952
8845	2.7093	-4.1195	110.881
8846	2.7093	-2.9288	248.441
8847	2.7094	22.8625	196.324
8848	2.7094	-3.4947	306.818
8849	2.7095	-2.8242	153.334
8850	2.7095	27.8644	13.8766
8851	2.7096	-2.8138	260.149
8852	2.7097	-2.7686	357.71
8853	2.7097	-2.9512	247.263
8854	2.7101	-2.9377	201.871
8855	2.7103	34.2204	194.007
8856	2.7104	-2.7202	325.993
8857	2.7105	37.8276	344.338
8858	2.7105	46.6213	284.783
8859	2.711	20.2805	135.29
8860	2.7111	-3.3414	300.953
8861	2.7113	-2.9701	305.127
8862	2.7113	-2.8565	127.129
8863	2.7114	24.5733	170.258
8864	2.7114	28.7897	19.9449
8865	2.7115	-2.7132	104.358
8866	2.7116	39.5792	243.506
8867	2.7118	31.337	201.519
8868	2.7118	-4.0194	87.4304
8869	2.7119	26.7802	222.526

8870	2.712	35.5599	107.098
8871	2.7122	22.9982	156
8872	2.7122	-2.8102	257.731
8873	2.7123	-2.8279	82.2845
8874	2.7123	30.5004	107.565
8875	2.7124	32.4391	115.307
8876	2.7124	24.7163	278.12
8877	2.7125	-4.5292	327.715
8878	2.7128	24.7999	154.112
8879	2.7129	-3.2243	263.245
8880	2.713	27.1119	171.3
8881	2.713	-3.3197	351.173
8882	2.7131	20.8505	318.095
8883	2.7132	31.1361	170.021
8884	2.7133	-2.7343	48.1235
8885	2.7133	-2.9982	297.088
8886	2.7134	36.1564	173.004
8887	2.7135	-3.1592	86.6177
8888	2.7139	22.0975	297.074
8889	2.7139	30.1523	183.092
8890	2.714	43.6167	37.3519
8891	2.7148	25.9029	203.911
8892	2.7148	-2.7313	172.54
8893	2.7149	25.8235	323.228
8894	2.715	-2.7397	356.647
8895	2.7151	-3.9373	312.735
8896	2.7158	36.9941	268.57
8897	2.7159	32.5105	22.3243
8898	2.716	30.4337	334.304
8899	2.7162	28.098	186.722
8900	2.7162	35.5963	246.337
8901	2.7163	30.9981	236.189
8902	2.7164	33.4736	207.326
8903	2.7166	-3.9676	157.561
8904	2.7169	-2.8863	208.543
8905	2.7169	22.8893	155.728
8906	2.7169	-3.6466	208.707
8907	2.717	32.185	289.103
8908	2.717	-3.6148	228.259
8909	2.7171	-2.8472	344.395
8910	2.7171	-2.8782	319.828
8911	2.7173	-4.1038	61.9086
8912	2.7174	52.4736	315.301
8913	2.7175	-3.8916	359.686
8914	2.7175	-3.9109	116.399
8915	2.7176	-3.1302	116.925
8916	2.7177	32.2605	247.841
8917	2.7178	35.1889	152.484

8918	2.7181	29.8396	219.683
8919	2.7181	-2.8226	197.968
8920	2.7182	-2.8993	178.697
8921	2.7183	-2.9735	22.5603
8922	2.7183	-2.7796	349.917
8923	2.7184	-2.8544	46.2605
8924	2.7186	35.0844	204.365
8925	2.7186	-2.8982	351.139
8926	2.7189	-2.6959	140.851
8927	2.7191	55.6609	205.306
8928	2.7191	35.4375	19.0167
8929	2.7191	30.994	262.691
8930	2.7191	-3.4497	98.1842
8931	2.7193	-2.6436	281.309
8932	2.7193	23.0076	149.963
8933	2.7194	25.6042	134.619
8934	2.7197	-2.9325	62.4337
8935	2.7197	29.3797	338.684
8936	2.7198	-3.1919	321.951
8937	2.7199	32.3979	36.8904
8938	2.7199	-2.848	144.053
8939	2.72	27.7325	347.194
8940	2.7201	-3.06	91.0874
8941	2.7202	-3.3894	35.5747
8942	2.7202	-2.7883	352.428
8943	2.7207	-2.9861	102.277
8944	2.7207	-3.042	271.706
8945	2.7208	-2.9797	183.374
8946	2.7208	-3.4035	1.6098
8947	2.721	-3.0017	34.342
8948	2.721	-3.4742	254.179
8949	2.7213	-2.9287	241.178
8950	2.7214	35.2444	163.043
8951	2.7214	38.4942	198.032
8952	2.7214	-2.8261	172.241
8953	2.7218	-3.8905	32.2376
8954	2.722	-2.9056	25.3893
8955	2.722	-3.963	130.283
8956	2.7222	-3.7364	346.292
8957	2.7225	37.7368	236.108
8958	2.7229	-3.272	177.961
8959	2.723	34.981	191.265
8960	2.723	-3.4005	261.752
8961	2.7234	-2.7909	4.7233
8962	2.7238	-3.9961	324.165
8963	2.7239	-2.6992	327.12
8964	2.724	-2.9573	62.8507
8965	2.7241	-3.4166	112.006

8966	2.7241	-3.0615	284.689
8967	2.7246	34.7358	180.026
8968	2.7248	26.9199	56.0697
8969	2.725	33.4322	228.303
8970	2.7251	-2.818	279.036
8971	2.7255	11.2054	334.958
8972	2.7256	-2.8643	321.679
8973	2.7257	-2.7466	53.8153
8974	2.7259	-2.827	73.9487
8975	2.726	29.8678	255.642
8976	2.7263	8.5042	88.2664
8977	2.7265	20.4085	166.119
8978	2.7266	-2.9599	259.789
8979	2.7266	26.3214	220.166
8980	2.7267	-3.2096	137.437
8981	2.7268	-2.7487	347.657
8982	2.727	-3.3983	156.649
8983	2.7271	-3.0868	51.0212
8984	2.7272	-2.7195	152.647
8985	2.7272	-3.3558	274.875
8986	2.7272	-4.2513	302.189
8987	2.7273	19.0919	145.373
8988	2.7274	-2.7558	236.979
8989	2.7275	37.7594	16.327
8990	2.7276	35.0908	59.8582
8991	2.7276	-3.4387	289.76
8992	2.7278	-3.0896	16.3589
8993	2.7279	18.5826	249.185
8994	2.7279	-3.7201	54.5877
8995	2.728	-3.6147	78.3117
8996	2.7282	22.2721	139.478
8997	2.7283	23.905	295.923
8998	2.7283	-3.4467	88.6999
8999	2.7284	48.1616	199.192
9000	2.7284	31.6761	125.316
9001	2.7285	28.4677	272.136
9002	2.7288	38.672	243.12
9003	2.7292	-2.8882	280.71
9004	2.7293	-2.9013	257.659
9005	2.7295	-3.9757	272.922
9006	2.7295	33.0273	15.1723
9007	2.7297	-2.8835	159.082
9008	2.7299	-3.2173	48.5402
9009	2.7303	16.0398	160.106
9010	2.7304	31.4706	118.65
9011	2.7305	29.8812	177.219
9012	2.7306	31.0461	329.922
9013	2.7308	-3.3137	39.6346

9014	2.7308	38.8818	254.674
9015	2.731	32.2991	233.952
9016	2.7313	29.5354	265.098
9017	2.7314	29.1	17.678
9018	2.7315	24.7196	8.9249
9019	2.7316	-2.711	284.654
9020	2.7316	33.0245	212.68
9021	2.7317	-2.8913	21.1663
9022	2.7319	23.6412	172.009
9023	2.7321	33.9157	200.016
9024	2.7322	-3.9577	144.586
9025	2.7323	30.7242	158.256
9026	2.7326	27.0937	98.5863
9027	2.7326	-4.4629	249.985
9028	2.7328	31.9402	210.389
9029	2.7328	33.5924	149.596
9030	2.7329	-4.0869	288.697
9031	2.7331	-4.1133	292.803
9032	2.7332	7.8795	218.201
9033	2.7334	29.9477	301.329
9034	2.7334	1.1827	121.427
9035	2.7337	-2.9378	138.701
9036	2.7337	-4.0062	269.427
9037	2.7338	42.7594	38.452
9038	2.7338	-2.7214	51.324
9039	2.734	-3.9984	312.393
9040	2.7342	-4.0736	61.6428
9041	2.7342	-3.8773	155.233
9042	2.7342	-2.765	231.51
9043	2.7342	-4.5114	246.457
9044	2.7343	-3.9465	316.099
9045	2.7344	31.4879	99.5878
9046	2.7345	-2.8253	12.7639
9047	2.7345	31.6646	324.239
9048	2.7346	-2.9667	337.024
9049	2.7347	35.3119	30.0724
9050	2.7348	34.0539	192.721
9051	2.7349	32.1422	318.182
9052	2.7349	27.9991	142.769
9053	2.735	-2.8811	336.196
9054	2.7352	-3.2331	111.819
9055	2.7353	27.4531	143.033
9056	2.7354	40.4296	44.6982
9057	2.7355	28.5129	219.55
9058	2.7358	48.232	75.6006
9059	2.7358	-3.0183	182.622
9060	2.7359	-2.9599	75.5466
9061	2.736	19.4714	358.112

9062	2.736	-3.8972	321.138
9063	2.7361	-2.9348	244.121
9064	2.7364	-3.9281	269.18
9065	2.7369	28.8372	110.744
9066	2.737	-2.7946	196.463
9067	2.7371	30.1969	34.5061
9068	2.7371	-2.7665	357.552
9069	2.7374	24.5286	229.595
9070	2.7376	31.306	248.029
9071	2.7379	28.7876	295.087
9072	2.7379	-2.9462	329.457
9073	2.738	37.5207	17.7704
9074	2.738	-2.8036	331.39
9075	2.7381	45.7139	30.9871
9076	2.7382	-3.5248	204.756
9077	2.7383	54.1278	306.858
9078	2.7383	32.9561	165.188
9079	2.7383	41.2557	187.245
9080	2.7383	-2.7567	158.651
9081	2.7384	-2.8849	103.377
9082	2.7385	-4.0153	103.328
9083	2.7385	30.7463	224.03
9084	2.7386	26.5004	22.1692
9085	2.7387	-2.8982	80.1118
9086	2.7387	43.2135	243.007
9087	2.7387	-2.7611	145.769
9088	2.739	29.1491	128.903
9089	2.7391	-3.8182	161.298
9090	2.7391	48.8692	205.148
9091	2.7391	55.1071	287.655
9092	2.7394	7.8646	72.6308
9093	2.7396	29.8338	246.948
9094	2.7396	-2.9921	291.917
9095	2.7397	-3.5411	289.367
9096	2.7397	22.3064	218.31
9097	2.7399	10.2773	222.591
9098	2.74	39.1433	210.007
9099	2.7403	-3.2859	307.461
9100	2.7403	30.5558	204.275
9101	2.7406	-2.846	91.9012
9102	2.7412	37.7692	7.1918
9103	2.7415	33.6174	197.083
9104	2.7415	-2.7216	14.9972
9105	2.7416	43.2857	202.087
9106	2.7417	34.8018	157.763
9107	2.7418	-2.9192	76.61
9108	2.742	35.499	301.459
9109	2.7421	33.1874	202.315

9110	2.7422	29.6824	85.2326
9111	2.7423	35.6207	148.835
9112	2.7424	-3.2038	24.893
9113	2.7424	-2.8639	67.8423
9114	2.7424	-3.2107	140.113
9115	2.7425	-2.9209	27.1921
9116	2.7426	22.4866	173.901
9117	2.7427	35.8397	22.0353
9118	2.7427	46.4659	358.391
9119	2.7427	26.2143	265.719
9120	2.7427	-4.661	58.5081
9121	2.7428	30.6345	76.105
9122	2.7429	-2.9242	0.4853
9123	2.7429	-2.7498	228.872
9124	2.7429	-3.2435	170.893
9125	2.743	18.4696	287.303
9126	2.743	-2.8598	147.831
9127	2.743	-2.9746	25.3901
9128	2.7432	-2.9262	45.0972
9129	2.7432	36.4828	29.0415
9130	2.7433	-2.9201	281.551
9131	2.7436	37.5212	325.924
9132	2.7437	34.0019	210.361
9133	2.7442	-2.8693	256.619
9134	2.7443	-2.9244	340.084
9135	2.7444	-2.9021	333.302
9136	2.7445	35.9507	128.459
9137	2.7445	25.7514	144.978
9138	2.7445	42.4579	342.8
9139	2.7446	24.7004	293.064
9140	2.745	31.2152	123.653
9141	2.7451	-3.3472	196.563
9142	2.7451	27.4079	335.397
9143	2.7454	-4.3593	274.83
9144	2.7454	-2.8691	275.993
9145	2.7455	36.9531	61.827
9146	2.7456	34.1175	275.2
9147	2.7456	32.1886	298.216
9148	2.7456	-2.8561	182.361
9149	2.7458	27.7906	134.318
9150	2.7459	25.7904	272.204
9151	2.7459	28.3419	153.935
9152	2.7459	-4.0302	350.578
9153	2.746	34.7733	298.379
9154	2.7462	-3.3535	24.9194
9155	2.7464	-2.8474	224.066
9156	2.7465	-3.9919	88.4994
9157	2.7465	26.0069	139.779

9158	2.7466	-2.9777	167.637
9159	2.7471	32.6544	166.739
9160	2.7471	34.7088	61.4078
9161	2.7473	25.3968	272.24
9162	2.7475	-2.8504	119.845
9163	2.7476	-2.7904	231.826
9164	2.7478	37.9633	246.895
9165	2.7478	19.7962	316.582
9166	2.7478	-3.0726	244.3
9167	2.748	23.6167	198.936
9168	2.748	-2.8023	15.6376
9169	2.7482	-3.7557	330.898
9170	2.7483	51.3112	353.491
9171	2.7483	32.6881	141.228
9172	2.7483	40.289	292.143
9173	2.7484	34.8942	136.562
9174	2.7485	25.559	158.646
9175	2.7486	-2.9742	257.375
9176	2.7486	-3.45	33.9802
9177	2.7486	26.5497	10.5471
9178	2.7489	-2.6912	113.733
9179	2.7489	-3.5781	3.6944
9180	2.7493	-2.956	341.61
9181	2.7494	28.0095	207.894
9182	2.7495	-2.977	146.202
9183	2.7497	-2.7345	158.691
9184	2.75	47.7456	38.9475
9185	2.7504	-3.766	11.7906
9186	2.7505	-2.999	13.4787
9187	2.7505	-4.3051	343.363
9188	2.7506	23.2467	195.481
9189	2.7507	43.6298	39.1382
9190	2.7508	28.6556	38.1924
9191	2.751	-2.9647	261.993
9192	2.7512	-3.3958	245.62
9193	2.7515	-2.8489	41.5806
9194	2.7516	-2.77	226.011
9195	2.7516	37.1578	30.9113
9196	2.7516	-4.3699	345.011
9197	2.7517	-2.9249	105.183
9198	2.7517	-2.967	165.69
9199	2.7518	-3.0611	122.237
9200	2.7519	37.2208	34.7884
9201	2.7522	-2.7343	122.869
9202	2.7523	35.6241	179.44
9203	2.753	27.6626	314.51
9204	2.753	19.3432	241.346
9205	2.7531	-3.481	243.672

9206	2.7531	-4.2313	331.932
9207	2.7533	41.6678	104.524
9208	2.7534	40.7771	77.0892
9209	2.7535	-3.2196	64.276
9210	2.7536	-3.7195	63.5623
9211	2.754	-2.6818	151.598
9212	2.7543	31.4164	181.597
9213	2.7544	-3.0041	33.5304
9214	2.7544	-3.6153	323.514
9215	2.7545	27.5689	234.429
9216	2.7545	-2.8494	87.7329
9217	2.7546	-3.633	273.414
9218	2.7546	33.5769	290.058
9219	2.7548	-2.8959	269.095
9220	2.7548	45.266	200.349
9221	2.7549	27.5886	11.2492
9222	2.755	-2.8668	209.028
9223	2.7551	29.5358	103.449
9224	2.7551	27.1569	199.175
9225	2.7552	33.0689	265.405
9226	2.7552	32.5366	270.49
9227	2.7552	25.9827	43.7727
9228	2.7553	29.2493	126.189
9229	2.7553	27.8026	40.5397
9230	2.7554	30.7587	228.377
9231	2.7555	37.8651	277.68
9232	2.7559	-4.2139	270.928
9233	2.756	34.1396	97.7021
9234	2.756	27.1879	56.2855
9235	2.7562	-3.9437	248.867
9236	2.7562	-2.73	50.2872
9237	2.7562	-2.8788	322.502
9238	2.7565	49.2556	311.865
9239	2.7566	31.0896	140.697
9240	2.7567	-3.8616	314.158
9241	2.7567	-3.176	98.957
9242	2.7568	35.5545	103.931
9243	2.7568	34.3017	178.579
9244	2.757	-2.9011	177.674
9245	2.7571	-2.7359	347.402
9246	2.7571	35.6917	194.293
9247	2.7571	-2.7734	130.513
9248	2.7573	33.7787	268.037
9249	2.7574	31.874	121.112
9250	2.7576	-2.8584	309.255
9251	2.7577	-2.9051	99.6577
9252	2.7578	-4.1379	234.238
9253	2.7579	36.458	78.7033

9254	2.758	-3.6707	128.89
9255	2.7581	-3.2318	74.0466
9256	2.7584	-3.2454	243.244
9257	2.7585	32.1485	182.754
9258	2.7585	34.324	250.337
9259	2.7587	-3.2274	26.562
9260	2.7588	38.3645	62.4367
9261	2.7589	-4.0715	9.4152
9262	2.7592	-2.685	274.939
9263	2.7592	21.9017	170.367
9264	2.7593	34.4076	309.138
9265	2.7593	36.6088	134.059
9266	2.7593	30.7655	99.0121
9267	2.7594	-4.1717	235.694
9268	2.7596	19.1094	146.319
9269	2.7597	-2.973	22.7272
9270	2.7599	-2.9683	32.4442
9271	2.7599	30.2641	54.7157
9272	2.7599	-3.0584	301.788
9273	2.7599	16.4089	25.6061
9274	2.7599	-3.0771	330.293
9275	2.7602	37.4478	33.723
9276	2.7602	34.9261	157.298
9277	2.7602	-3.0105	160.71
9278	2.7605	-2.9799	69.2168
9279	2.7606	46.8129	199.945
9280	2.7607	32.4674	140.484
9281	2.7608	39.5272	311.021
9282	2.7609	-2.7296	24.6608
9283	2.7609	39.9715	16.8834
9284	2.761	34.4151	291.74
9285	2.7613	-2.8131	341.47
9286	2.7615	45.8929	156.155
9287	2.7616	22.1376	0.0649
9288	2.7616	41.3931	152.612
9289	2.7618	-3.2715	285.906
9290	2.7619	27.918	292.91
9291	2.7622	16.6481	291.607
9292	2.7623	33.2682	48.7698
9293	2.7624	54.5392	229.919
9294	2.7624	26.4674	195.796
9295	2.7624	-2.7664	240.49
9296	2.7624	-3.2829	329.53
9297	2.7625	31.196	138.26
9298	2.7625	-3.1335	307.191
9299	2.7629	11.6798	317.184
9300	2.7629	-3.1094	194.512
9301	2.7631	-2.6495	272.927

9302	2.7631	-3.5428	149.374
9303	2.7632	32.7431	251.489
9304	2.7632	34.2629	348.892
9305	2.7633	-2.7808	148.477
9306	2.7634	42.4237	33.0374
9307	2.7634	40.9714	70.8765
9308	2.7636	47.3445	1.7585
9309	2.7637	38.151	197.539
9310	2.7638	44.6805	2.6428
9311	2.7639	39.7617	191.605
9312	2.7645	34.3097	177.674
9313	2.7654	-3.8079	152.98
9314	2.7655	44.3932	205.15
9315	2.7655	-3.4969	39.449
9316	2.7656	23.0068	333.321
9317	2.7661	31.2609	103.713
9318	2.7661	21.7115	52.5507
9319	2.7662	26.3798	115.72
9320	2.7664	23.9179	123.329
9321	2.7666	38.2653	155.647
9322	2.7666	-2.863	287.792
9323	2.7669	25.287	62.463
9324	2.767	-2.9372	184.139
9325	2.7673	34.3646	191.418
9326	2.7673	31.1221	57.5928
9327	2.7676	-3.7867	142.472
9328	2.7678	-3.4026	333.244
9329	2.7679	30.0165	152.56
9330	2.7679	33.038	4.7506
9331	2.768	-2.9542	277.053
9332	2.7682	24.2436	126.8
9333	2.7683	6.2311	146.204
9334	2.7683	33.336	314.494
9335	2.7684	-2.7835	338.329
9336	2.7685	23.2752	128.347
9337	2.7686	-4.3073	344.905
9338	2.7686	-0.2343	333.246
9339	2.7688	-3.1344	340.55
9340	2.7689	-2.92	29.3643
9341	2.7689	-2.7369	165.909
9342	2.7689	25.7575	215.916
9343	2.7692	-3.5579	89.1781
9344	2.7692	43.0419	190.796
9345	2.7695	36.5508	168.194
9346	2.7696	30.1424	276.042
9347	2.7697	38.9629	66.6314
9348	2.7698	26.7903	129.334
9349	2.7699	-2.7571	134.77

9350	2.7699	-2.838	42.5744
9351	2.77	30.5423	278.565
9352	2.7703	32.5357	252.636
9353	2.7704	-4.4167	141.923
9354	2.7704	-3.0445	326.419
9355	2.7706	-3.5356	105.834
9356	2.7708	-3.8094	120.614
9357	2.771	39.9252	172.998
9358	2.7713	-2.9832	229.266
9359	2.7713	-2.8479	16.7875
9360	2.7715	30.7214	233.66
9361	2.7716	26.1298	272.411
9362	2.7719	-2.7788	338.561
9363	2.772	33.2581	136.693
9364	2.7721	-2.8217	194.58
9365	2.7723	30.3472	84.8742
9366	2.7724	34.4559	146.933
9367	2.7726	24.7625	164.487
9368	2.7727	-3.4786	73.8757
9369	2.7727	41.2945	229.977
9370	2.7729	-2.8108	212.54
9371	2.7729	28.003	142.313
9372	2.7736	24.0259	227.954
9373	2.7736	37.0931	74.3835
9374	2.7738	-2.9456	283.538
9375	2.7739	32.7381	199.517
9376	2.7739	34.1085	43.9231
9377	2.7741	-3.1009	89.6975
9378	2.7741	-2.8627	112.45
9379	2.7741	-2.8965	241.657
9380	2.7742	23.9225	45.7219
9381	2.7745	-2.8254	146.646
9382	2.7746	-2.8595	43.997
9383	2.7749	-3.1896	312.009
9384	2.775	25.2312	262.499
9385	2.7752	34.6597	72.7732
9386	2.7753	5.7778	145.768
9387	2.7753	39.48	239.11
9388	2.7754	-3.2209	298.613
9389	2.7756	23.0101	176.091
9390	2.7757	33.036	239.02
9391	2.7757	-2.9966	349.112
9392	2.7759	37.6231	90.5881
9393	2.776	-4.3426	46.454
9394	2.7761	40.4351	47.2041
9395	2.7762	-2.9855	140.089
9396	2.7764	31.1462	269.234
9397	2.7766	-2.8831	102.159

9398	2.7766	-2.7623	233.407
9399	2.7767	-3.5352	80.7917
9400	2.7769	-2.8978	198.917
9401	2.7769	32.4513	220.285
9402	2.7769	29.9361	133.682
9403	2.7771	-3.6229	195.073
9404	2.7774	4.8796	102.33
9405	2.7776	31.7914	131.786
9406	2.7778	-2.9778	256.539
9407	2.7778	31.8757	104.358
9408	2.778	-3.873	128.604
9409	2.7782	-2.7819	195.987
9410	2.7782	27.6566	260.509
9411	2.7785	41.1825	106.784
9412	2.7785	47.6381	318.259
9413	2.7788	49.9576	124.292
9414	2.779	38.9001	140.366
9415	2.779	30.7053	180.038
9416	2.7791	36.7874	27.523
9417	2.7791	-2.7584	106.523
9418	2.7794	-2.9624	54.1659
9419	2.7794	-2.7541	143.41
9420	2.7795	-2.7925	50.4994
9421	2.7797	33.7799	90.5425
9422	2.7798	32.1604	351.611
9423	2.78	-2.8554	110.505
9424	2.7805	-2.8059	139.443
9425	2.7807	37.9749	138.701
9426	2.7808	26.5733	220.724
9427	2.7808	-2.8857	280.725
9428	2.7809	29.5362	232.778
9429	2.7809	26.1073	297.08
9430	2.7809	-1.2081	4.0797
9431	2.781	24.8209	146.941
9432	2.781	30.1938	172.162
9433	2.781	27.406	218.967
9434	2.7811	-3.3947	348.529
9435	2.7812	31.0913	74.1488
9436	2.7813	47.0571	354.668
9437	2.7817	-2.8039	181.209
9438	2.782	33.9154	85.636
9439	2.7821	15.8864	103.946
9440	2.7821	39.6934	202.869
9441	2.7822	13.5272	296.273
9442	2.7824	43.7665	200.61
9443	2.7824	-3.0498	299.39
9444	2.7824	-3.4002	246.553
9445	2.7824	-2.9286	278.97

9446	2.7827	43.0899	183.827
9447	2.7829	5.0537	342.157
9448	2.7829	-3.775	133.231
9449	2.7831	-3.0529	158.91
9450	2.7837	-2.8529	321.392
9451	2.7838	-2.971	328.533
9452	2.7838	-4.4754	156.042
9453	2.7844	-4.4537	69.5427
9454	2.7844	23.0618	220.29
9455	2.7844	-2.7884	166.543
9456	2.7845	36.8109	225.709
9457	2.7845	35.763	77.9365
9458	2.7845	-2.9111	155.208
9459	2.7846	-2.8296	168.478
9460	2.7846	40.473	16.8939
9461	2.7849	47.1516	204.101
9462	2.785	-4.2486	334.35
9463	2.785	-3.3136	56.8705
9464	2.7851	34.9595	45.7228
9465	2.7853	26.5881	165.504
9466	2.7853	31.4958	147.84
9467	2.7853	-4.5003	293.913
9468	2.7855	-2.8322	313.099
9469	2.7856	36.8279	141.17
9470	2.7856	40.6074	213.906
9471	2.7858	33.8741	326.405
9472	2.7861	-3.2199	328.192
9473	2.7862	-2.9704	175.796
9474	2.7864	28.88	217.389
9475	2.7865	28.1636	261.359
9476	2.7867	30.3324	305.383
9477	2.7868	-2.8037	27.3411
9478	2.787	-2.9985	237.752
9479	2.787	-2.826	22.2668
9480	2.787	29.0947	59.3858
9481	2.7872	28.5425	299.228
9482	2.7874	-3.6432	281.545
9483	2.7878	33.3463	141.195
9484	2.7878	25.0208	176.841
9485	2.7878	43.505	38.9575
9486	2.7879	-2.9876	153.732
9487	2.788	-2.9539	139.296
9488	2.7881	21.0811	53.6856
9489	2.7882	-2.7839	336.088
9490	2.7882	-2.7453	352.893
9491	2.7883	39.1285	88.698
9492	2.7883	38.4096	235.071
9493	2.7887	33.7402	13.5538

9494	2.7887	32.0985	109.018
9495	2.7887	46.1695	201.859
9496	2.7889	-2.7273	129.286
9497	2.789	-3.9978	101.97
9498	2.7891	-2.9524	68.997
9499	2.7893	37.9202	210.189
9500	2.7893	40.6278	130.815
9501	2.7894	36.3278	352.409
9502	2.7895	-3.9864	131.636
9503	2.7896	-3.3401	313.73
9504	2.7898	30.2239	341.509
9505	2.7898	-3.334	79.2504
9506	2.7899	30.6454	212.103
9507	2.7901	-3.4218	342.348
9508	2.7902	-2.7689	11.4798
9509	2.7902	32.247	247.462
9510	2.7903	51.5337	357.071
9511	2.7906	-2.9083	292.903
9512	2.7907	32.0893	196.887
9513	2.7908	38.534	164.386
9514	2.7908	21.9185	308.2
9515	2.7912	34.3681	246.937
9516	2.7913	29.5486	338.763
9517	2.7913	37.1066	79.5183
9518	2.7916	-3.1886	73.0887
9519	2.7917	35.3905	92.7404
9520	2.7918	37.5229	153.441
9521	2.7918	41.8653	113.593
9522	2.7919	4.9298	117.226
9523	2.7919	27.3332	144.469
9524	2.7922	41.3228	285.965
9525	2.7925	-2.9401	239.981
9526	2.7926	-3.3404	28.2656
9527	2.7927	37.2949	131.112
9528	2.7928	-3.5375	5.6628
9529	2.7929	38.9876	221.365
9530	2.7931	-2.8382	147.645
9531	2.7931	-3.8764	173.983
9532	2.7931	-2.8561	205.981
9533	2.7935	-2.7535	251.701
9534	2.7937	-2.8318	354.693
9535	2.7938	28.8951	332.601
9536	2.7939	33.012	321.425
9537	2.7939	-2.7009	148.109
9538	2.7939	47.0499	61.875
9539	2.7942	38.1605	93.4761
9540	2.7946	-2.7722	123.733
9541	2.795	32.76	143.253

9542	2.7951	-2.7143	153.11
9543	2.7951	-3.8275	332.07
9544	2.7951	-2.8856	301.228
9545	2.7952	-2.7854	113.097
9546	2.7953	-3.1381	277.828
9547	2.7953	-3.0948	98.3343
9548	2.7956	-4.0126	351.346
9549	2.7956	-2.8819	311.407
9550	2.7958	40.2031	202.551
9551	2.7959	-3.9655	323.759
9552	2.7961	-2.7655	317.261
9553	2.7963	28.0031	301.116
9554	2.7963	-2.8366	264.335
9555	2.7964	-2.8949	330.905
9556	2.7966	-3.2778	92.3469
9557	2.7966	35.3366	166.625
9558	2.7967	-2.7515	11.291
9559	2.7968	-3.0134	342.979
9560	2.7968	52.6745	353.474
9561	2.7968	53.2144	307.968
9562	2.7969	38.8499	79.4048
9563	2.7971	-2.8188	17.8759
9564	2.7972	31.353	98.656
9565	2.7972	-2.6314	272.511
9566	2.7973	39.0875	100.001
9567	2.7974	-3.7387	108.619
9568	2.7974	23.4955	78.17
9569	2.7974	-3.457	294.538
9570	2.7975	34.514	222.637
9571	2.7976	-2.7143	305.407
9572	2.7977	53.573	306.126
9573	2.7977	27.4023	119.034
9574	2.7977	34.859	124.064
9575	2.7978	-2.9655	14.9697
9576	2.7979	28.1077	129.98
9577	2.7982	32.8186	356.17
9578	2.7982	-3.1491	248.698
9579	2.7984	33.9553	351.757
9580	2.7985	-3.2161	62.7142
9581	2.7985	35.6461	109.956
9582	2.7985	26.4487	217.787
9583	2.7988	-4.0209	172.444
9584	2.799	30.316	63.9649
9585	2.7991	56.0814	55.0205
9586	2.7992	-2.6797	17.6049
9587	2.7993	-2.8988	281.021
9588	2.7993	-2.8091	267.969
9589	2.7993	-2.8654	333.865

9590	2.7995	-3.3255	137.642
9591	2.7995	30.6934	25.9272
9592	2.7995	-2.8068	332.629
9593	2.7997	-3.2966	125.978
9594	2.7998	-4.2582	9.5407
9595	2.7998	-3.6409	351.436
9596	2.8001	-3.1155	80.0634
9597	2.8001	-3.7238	10.9326
9598	2.8002	-2.8233	98.5071
9599	2.8003	27.6075	77.2444
9600	2.8003	-3.0277	320.308
9601	2.8005	-3.813	233.711
9602	2.8006	-2.7953	178.963
9603	2.8008	-2.8853	94.7416
9604	2.8008	33.4331	170.733
9605	2.8008	43.1345	281.228
9606	2.8009	-4.0323	141.124
9607	2.801	-2.8529	140.246
9608	2.801	45.7399	211.873
9609	2.801	29.7369	33.0881
9610	2.801	-2.972	148.715
9611	2.801	37.3053	252.578
9612	2.8015	-2.857	303.146
9613	2.8015	-2.8965	81.0528
9614	2.8016	-3.9318	353.612
9615	2.802	45.0219	212.788
9616	2.8021	39.4709	235.209
9617	2.8023	39.6671	200.833
9618	2.8025	-2.9711	333.788
9619	2.8025	-3.2984	200.431
9620	2.8027	25.5386	358.648
9621	2.8031	-4.0436	73.3494
9622	2.8031	-2.8848	199.698
9623	2.8032	-3.0617	21.7661
9624	2.8032	-2.8378	128.269
9625	2.8034	32.6688	123.445
9626	2.8036	38.9308	205.349
9627	2.8036	42.5757	312.562
9628	2.8037	-2.6887	141.986
9629	2.8037	30.3494	89.7842
9630	2.8038	-2.8798	316.278
9631	2.8039	27.0686	3.2101
9632	2.804	24.7361	253.826
9633	2.804	-3.1776	295.999
9634	2.8041	-3.8932	126.413
9635	2.8041	-3.6632	206.448
9636	2.8044	39.9755	29.194
9637	2.8047	-3.8636	277.954

9638	2.8047	32.0952	140.69
9639	2.8047	71.0031	313.587
9640	2.8049	-2.7708	160.674
9641	2.8054	31.5116	121.707
9642	2.8054	-2.7834	328.643
9643	2.8059	-2.9305	354.221
9644	2.8059	-2.9124	160.396
9645	2.806	48.1347	283.862
9646	2.806	34.9708	195.777
9647	2.8065	42.5837	61.717
9648	2.8065	-2.8172	140.593
9649	2.8066	-3.9593	323.347
9650	2.8068	47.3263	55.7628
9651	2.8069	37.0022	206.446
9652	2.8069	51.5062	4.7313
9653	2.8071	37.9122	160.765
9654	2.8072	-2.9082	138.513
9655	2.8073	23.587	96.9264
9656	2.8077	-3.9369	256.467
9657	2.8077	12.0143	168.746
9658	2.8079	-2.9276	274.069
9659	2.8081	-2.7494	94.0695
9660	2.8082	20.3376	259.86
9661	2.8082	41.2495	282.497
9662	2.8082	-2.9041	272.125
9663	2.8083	8.4882	130.935
9664	2.8086	-2.8587	179.103
9665	2.809	27.2505	310.168
9666	2.8091	-4.1321	241.639
9667	2.8094	-3.3302	148.868
9668	2.8095	45.7361	210.628
9669	2.8095	35.1128	204.546
9670	2.8097	-2.995	335.73
9671	2.8098	46.5745	207.377
9672	2.8099	-2.8489	29.0981
9673	2.81	-3.1035	124.35
9674	2.8103	3.6054	300.303
9675	2.8104	-2.8273	70.5165
9676	2.8106	56.5994	235.9
9677	2.8108	-3.077	47.6374
9678	2.8108	-2.8248	179.594
9679	2.8109	40.7138	83.8479
9680	2.8109	-3.4314	311.501
9681	2.811	-3.1583	27.8435
9682	2.8111	-3.2047	152.434
9683	2.8112	32.5039	28.6221
9684	2.8114	-2.8702	81.1573
9685	2.8114	38.8099	332.427

9686	2.8115	35.6699	254.566
9687	2.8115	-2.8043	78.0447
9688	2.8115	-3.9385	7.53
9689	2.8115	36.4691	215.897
9690	2.8118	38.5938	35.8056
9691	2.8119	44.4821	88.6869
9692	2.812	29.1323	163.458
9693	2.8121	42.9375	20.5841
9694	2.8126	28.1833	137.817
9695	2.8131	-2.9583	185.537
9696	2.8132	45.3725	193.458
9697	2.8133	34.8874	233.517
9698	2.8133	32.1499	147.699
9699	2.8135	-2.895	181.763
9700	2.8135	-4.4192	53.4799
9701	2.8139	39.6965	177.515
9702	2.8141	-3.1651	356.56
9703	2.8143	33.5239	291.568
9704	2.8143	-2.8076	265.774
9705	2.8144	-4.1747	21.466
9706	2.8147	27.9189	141.932
9707	2.8147	32.1946	88.7977
9708	2.8147	-3.3489	54.0142
9709	2.8149	-2.8707	21.6231
9710	2.815	35.3271	304.519
9711	2.8152	36.3476	283.845
9712	2.8154	32.2697	86.7068
9713	2.8154	23.9061	340.794
9714	2.8154	31.4029	97.7897
9715	2.8155	10.8493	232.379
9716	2.8157	48.1107	26.4816
9717	2.8158	40.3105	304.064
9718	2.8158	27.044	35.0819
9719	2.8159	-2.8061	135.353
9720	2.8161	35.6123	315.117
9721	2.8162	40.4108	280.917
9722	2.8163	-2.792	130.233
9723	2.8164	32.4798	268.826
9724	2.8164	31.2049	97.4584
9725	2.8166	35.0071	252.375
9726	2.8166	-3.1892	37.5941
9727	2.8166	-2.7791	296.615
9728	2.8166	37.3271	204.772
9729	2.8168	39.7405	49.6796
9730	2.8168	-4.5667	218.122
9731	2.817	34.3688	169.732
9732	2.817	-2.7717	354.176
9733	2.8171	-3.5276	146.924

9734	2.8172	-2.9159	46.5336
9735	2.8172	-3.3109	344.569
9736	2.8172	-4.2547	241.546
9737	2.8173	-2.8799	230.791
9738	2.8177	-2.8715	338.789
9739	2.8178	42.123	217.612
9740	2.8179	-3.2169	82.1444
9741	2.8181	-4.5234	86.9537
9742	2.8182	-2.9918	200.241
9743	2.8183	-2.9101	56.0445
9744	2.8184	40.4556	18.7338
9745	2.8184	-3.2604	91.0265
9746	2.8185	43.8331	120.845
9747	2.8185	-2.7478	11.0833
9748	2.8189	-2.9597	188.124
9749	2.819	-2.7291	282.725
9750	2.819	38.3355	8.4297
9751	2.819	-2.831	349.513
9752	2.819	46.3528	148.829
9753	2.8193	39.9116	69.9517
9754	2.8196	-4.362	192.04
9755	2.8196	-2.8156	249.314
9756	2.8197	18.434	17.545
9757	2.8198	33.2331	312.964
9758	2.8199	-3.8993	191.972
9759	2.82	27.8189	124.935
9760	2.8201	34.4977	133.307
9761	2.8201	32.0754	350.627
9762	2.8203	-2.8564	215.974
9763	2.8204	-2.8824	72.423
9764	2.8204	41.0389	70.6681
9765	2.8204	51.2799	117.066
9766	2.8205	-4.3542	357.832
9767	2.8206	44.6486	258.355
9768	2.8206	38.1416	51.0107
9769	2.8207	-2.8649	119.81
9770	2.8207	31.2028	332.091
9771	2.8207	31.7695	35.8582
9772	2.8207	42.6017	323.153
9773	2.8208	29.0817	256.58
9774	2.8208	27.8126	336.54
9775	2.821	38.3995	340.204
9776	2.8212	23.4673	173.942
9777	2.8213	30.7043	263.84
9778	2.8217	-3.4209	50.6719
9779	2.8218	45.9572	34.4867
9780	2.8218	-3.0769	112.336
9781	2.8219	33.4712	321.956

9782	2.822	-2.6833	312.47
9783	2.8221	31.3421	224.762
9784	2.8221	37.041	2.9012
9785	2.8224	22.816	167.969
9786	2.8225	30.8359	154.181
9787	2.8226	32.7067	239.648
9788	2.8226	43.8761	304.117
9789	2.8226	39.9227	84.8244
9790	2.8228	-2.7624	120.435
9791	2.8228	40.0705	4.7986
9792	2.823	32.5459	149.82
9793	2.823	-3.2473	63.6064
9794	2.8231	34.5585	213.368
9795	2.8231	32.774	74.0859
9796	2.8233	-2.7149	241.395
9797	2.8235	-2.9966	238.574
9798	2.8238	-2.903	264.852
9799	2.8238	-3.7233	268.696
9800	2.8238	-3.0364	330.898
9801	2.824	-3.6193	349.665
9802	2.824	-3.0268	242.869
9803	2.824	-2.8188	338.058
9804	2.8241	-2.779	95.5863
9805	2.8243	24.3281	345.728
9806	2.8244	28.0525	216.936
9807	2.8244	-2.7519	63.2
9808	2.8245	21.8106	280.91
9809	2.8246	4.5902	252.608
9810	2.8247	31.1128	283.334
9811	2.8247	31.5781	80.7277
9812	2.8248	-2.9115	128.181
9813	2.8253	-3.7005	49.0911
9814	2.8254	-2.7984	279.747
9815	2.8254	35.6237	258.84
9816	2.8255	36.0928	110.383
9817	2.8256	-2.7628	31.4288
9818	2.8259	40.1942	25.4877
9819	2.826	-3.2765	271.102
9820	2.8261	34.2409	189.879
9821	2.8261	29.7222	269.499
9822	2.8262	-2.9552	61.6112
9823	2.8265	33.648	135.117
9824	2.8266	35.1222	260.765
9825	2.8266	-3.6154	254.888
9826	2.827	-3.5656	190.309
9827	2.8271	36.4598	201.937
9828	2.8275	12.4694	170.772
9829	2.8276	44.6349	306.725

9830	2.8278	-3.7217	10.7932
9831	2.8279	29.5468	4.3696
9832	2.8281	-4.1444	175.042
9833	2.8281	18.9589	262.718
9834	2.8282	-3.6586	162.196
9835	2.8283	28.0138	188.369
9836	2.8285	-3.1358	347.759
9837	2.8287	48.3345	8.3662
9838	2.8288	63.0672	115.466
9839	2.8289	-2.9696	243.468
9840	2.8289	48.6568	28.0934
9841	2.8293	53.6284	33.2758
9842	2.8293	20.9366	333.871
9843	2.8295	26.0177	41.681
9844	2.8295	20.1318	290.048
9845	2.8295	-2.908	310.178
9846	2.8296	-2.8274	146.436
9847	2.8297	6.3178	9.51
9848	2.8298	-2.9881	30.4786
9849	2.8299	32.8746	155.261
9850	2.8299	-2.8394	191.198
9851	2.8301	31.447	289.228
9852	2.8302	42.4582	282.445
9853	2.8303	-2.9297	264.024
9854	2.8303	-2.8987	65.9436
9855	2.8303	32.0433	20.7178
9856	2.8304	32.6026	291.658
9857	2.8304	32.6148	10.6956
9858	2.8306	39.6525	116.06
9859	2.8306	34.28	182.692
9860	2.8308	42.0577	271.671
9861	2.8308	-3.261	34.2397
9862	2.8309	-2.9935	110.324
9863	2.8309	46.2175	68.2427
9864	2.8311	47.5443	19.5988
9865	2.8312	-2.9443	328.32
9866	2.8314	34.6033	114.478
9867	2.8314	27.9989	98.8246
9868	2.8316	-2.9565	37.2426
9869	2.8317	-3.1286	84.4377
9870	2.8318	44.7454	241.356
9871	2.8318	-2.8294	334.903
9872	2.8319	-2.6781	144.689
9873	2.832	-3.0409	307.832
9874	2.832	46.3077	184.262
9875	2.8321	33.2067	145.328
9876	2.8323	28.5301	336.895
9877	2.8323	39.5285	174.857

9878	2.8324	-2.9535	330.294
9879	2.8324	35.6881	118.245
9880	2.8326	26.6497	145.126
9881	2.8327	-2.7952	55.9737
9882	2.8328	-2.8208	340.65
9883	2.8328	-2.7936	107.652
9884	2.8331	-2.8692	104.284
9885	2.8337	32.3873	329.672
9886	2.8337	-3.6879	265.531
9887	2.8337	36.2545	11.5043
9888	2.8338	-2.9985	50.2199
9889	2.834	-2.9712	5.5816
9890	2.834	24.9911	218.406
9891	2.834	29.4989	238.96
9892	2.834	-3.8042	83.5742
9893	2.8341	30.6121	269.749
9894	2.8341	-2.7742	194.386
9895	2.8342	43.2525	352.671
9896	2.8345	32.9564	154.351
9897	2.8347	45.9951	210.737
9898	2.8347	-2.9021	339.463
9899	2.8349	-3.0808	252.943
9900	2.835	-3.3664	177.384
9901	2.8351	-3.4675	312.124
9902	2.8353	-4.3865	352.988
9903	2.8355	28.554	176.252
9904	2.8355	-3.0149	198.36
9905	2.8357	-2.9611	326.448
9906	2.8359	41.6676	165.314
9907	2.8359	-3.1013	53.1895
9908	2.8359	-2.8847	271.224
9909	2.836	23.4401	2.9195
9910	2.8361	39.7617	113.703
9911	2.8361	-3.292	149.702
9912	2.8363	41.7444	141.79
9913	2.8364	-2.9704	63.7772
9914	2.8364	46.3274	82.1647
9915	2.8365	-3.0938	13.9777
9916	2.8365	-3.6994	150.099
9917	2.8365	43.9395	230.487
9918	2.8366	-2.8899	267.655
9919	2.8367	-2.7772	154.659
9920	2.8368	36.2875	248.87
9921	2.837	-2.8549	29.4683
9922	2.8371	-3.2765	352.163
9923	2.8373	29.6136	265.063
9924	2.8374	-2.761	118.382
9925	2.8375	30.4996	160.783

9926	2.8377	46.9684	202.432
9927	2.8378	44.2659	161.022
9928	2.8379	28.6105	219.176
9929	2.838	39.8007	206.272
9930	2.8381	66.0128	311.405
9931	2.8381	-2.7665	310.695
9932	2.8382	-3.85	159.539
9933	2.8384	29.417	157.156
9934	2.8385	22.4021	154.101
9935	2.8385	-4.2472	197.015
9936	2.8386	44.6528	235.43
9937	2.8387	-4.4288	31.1542
9938	2.8387	-3.1514	237.659
9939	2.8387	-2.8608	160.953
9940	2.8388	61.7691	9.2238
9941	2.8391	-3.1325	94.1388
9942	2.8391	-2.8388	340.555
9943	2.8392	35.8224	104.177
9944	2.8394	-2.913	121.524
9945	2.8395	40.9874	128.627
9946	2.8396	33.55	136.364
9947	2.8397	58.7374	130.793
9948	2.8398	-2.9442	184.619
9949	2.8398	47.1413	33.6389
9950	2.8398	28.6656	233.684
9951	2.8403	-2.6648	194.305
9952	2.8404	44.7144	263.173
9953	2.8404	30.3107	9.0387
9954	2.8404	26.3157	45.6359
9955	2.8408	-3.1943	329.674
9956	2.8408	36.6092	174.295
9957	2.8408	26.6887	239.833
9958	2.8409	-3.2585	163.335
9959	2.841	29.965	172.22
9960	2.8413	-2.8658	165.313
9961	2.8414	31.8145	233.929
9962	2.8414	-3.2537	344.092
9963	2.8415	40.0982	321.806
9964	2.8415	52.0889	213.29
9965	2.8416	-3.473	135.773
9966	2.8416	36.8157	350.078
9967	2.8417	50.6424	27.4315
9968	2.8419	-2.85	312.922
9969	2.842	32.4511	208.808
9970	2.8421	-2.8375	148.323
9971	2.8421	-3.6086	176.438
9972	2.8421	-3.7765	79.7729
9973	2.8422	-4.4751	45.0567

9974	2.8423	41.1764	108.026
9975	2.8423	-2.7623	149.148
9976	2.8423	-4.0378	41.0413
9977	2.8424	27.1037	312.729
9978	2.8424	-2.8223	308.473
9979	2.8424	-2.7844	285.901
9980	2.8427	-4.1084	5.3136
9981	2.8428	-3.8053	17.7648
9982	2.8428	-2.7949	329.956
9983	2.8429	-2.8276	167.248
9984	2.8431	22.4639	235.779
9985	2.8431	40.2502	63.864
9986	2.8433	47.8352	247.617
9987	2.8433	25.1183	149.933
9988	2.8433	-3.655	10.3923
9989	2.8434	34.2236	116.141
9990	2.8434	-2.7658	125.094
9991	2.8435	19.135	298.983
9992	2.8435	41.147	86.4911
9993	2.8436	-3.2609	256.26
9994	2.8437	47.9753	114.712
9995	2.8438	-2.853	35.3217
9996	2.8438	37.6713	21.7597
9997	2.8439	-2.9542	92.0634
9998	2.8439	-3.4339	286.231
9999	2.8439	-4.3145	43.7079
10000	2.8441	37.4153	1.7291
10001	2.8445	33.9544	113.438
10002	2.8445	30.0054	156.074
10003	2.8445	42.3636	266.789
10004	2.8447	31.0823	75.1207
10005	2.8448	-4.02	296.082
10006	2.8449	-2.7916	19.8484
10007	2.8452	59.2882	204.616
10008	2.8452	-2.8315	63.704
10009	2.8452	37.7719	10.0247
10010	2.8452	-3.1897	198.849
10011	2.8453	45.8789	230.987
10012	2.8455	-3.3121	295.2
10013	2.8461	32.0428	274.071
10014	2.8464	44.6904	241.976
10015	2.8465	50.2646	73.8388
10016	2.8465	-3.1632	50.9732
10017	2.8466	40.6093	278.675
10018	2.847	58.9031	54.4065
10019	2.8472	-2.7914	28.088
10020	2.8475	-2.7253	27.3435
10021	2.8476	-2.8454	131.279

10022	2.8476	39.0621	70.9516
10023	2.8476	28.913	142.34
10024	2.8476	40.1823	57.8143
10025	2.8477	17.322	154.627
10026	2.8477	-2.9522	298.426
10027	2.8479	28.6497	316.585
10028	2.8482	33.0592	308.961
10029	2.8483	-2.7297	26.3545
10030	2.8484	37.7335	23.0025
10031	2.8485	37.1475	313.762
10032	2.8485	-2.8451	144.272
10033	2.8486	53.3723	53.9963
10034	2.8486	-3.121	128.954
10035	2.8486	-3.0382	307.668
10036	2.8487	12.253	157.253
10037	2.8488	-3.2923	245.056
10038	2.8489	37.0902	52.6201
10039	2.8489	31.7588	135.197
10040	2.8489	-4.3211	290.488
10041	2.849	-2.943	303.678
10042	2.8491	-2.8765	325.271
10043	2.8492	-3.4308	329.429
10044	2.8492	26.6141	199.128
10045	2.8494	57.3478	49.7609
10046	2.8497	-3.1443	162.854
10047	2.8498	32.7349	196.542
10048	2.8499	-2.9525	182
10049	2.8499	40.9771	204.321
10050	2.8501	43.5984	279.777
10051	2.8504	39.5235	151.253
10052	2.8505	-2.7646	141.988
10053	2.8505	35.1267	25.1954
10054	2.8506	-2.8068	4.7912
10055	2.8507	41.6583	48.1831
10056	2.8509	38.5632	254.296
10057	2.851	-3.5451	82.1663
10058	2.851	-2.8674	86.7786
10059	2.851	20.9645	165.34
10060	2.8511	31.1271	201.02
10061	2.8512	40.2667	144.423
10062	2.8512	-2.7889	311.052
10063	2.8515	40.9034	79.2214
10064	2.8516	48.3107	285.303
10065	2.8518	31.9402	130.682
10066	2.852	43.7513	198.356
10067	2.8521	-3.143	21.4457
10068	2.8521	-3.0831	202.199
10069	2.8522	-3.821	133.087

10070	2.8523	-3.4199	288.246
10071	2.8524	-2.9934	66.1128
10072	2.8525	-2.7688	140.752
10073	2.8525	-4.1034	344.61
10074	2.8526	22.376	136.93
10075	2.8532	-2.956	137.022
10076	2.8533	-3.1887	263.045
10077	2.8534	46.9322	278.737
10078	2.8537	37.1342	168.098
10079	2.8538	44.6325	130.765
10080	2.854	34.9033	84.6925
10081	2.8542	19.7866	162.179
10082	2.8544	50.9147	34.4537
10083	2.8545	41.8182	311.194
10084	2.8546	-4.3122	115.233
10085	2.8549	-3.0623	258.428
10086	2.8549	-2.7537	151.982
10087	2.855	28.8693	165.286
10088	2.8553	-3.6594	97.9432
10089	2.8554	67.3417	357.904
10090	2.8554	24.8903	294.124
10091	2.8558	-3.3294	264.693
10092	2.8558	-2.8413	192.44
10093	2.856	47.4541	30.0167
10094	2.856	38.496	164.055
10095	2.8561	-2.834	312.712
10096	2.8562	38.7788	59.0368
10097	2.8562	30.1051	341.705
10098	2.8563	-2.9263	263.024
10099	2.8565	34.8395	175.422
10100	2.8567	40.1287	306.405
10101	2.8568	-4.3532	274.979
10102	2.8568	-2.7686	317.069
10103	2.8569	13.7062	119.096
10104	2.857	50.8736	264.346
10105	2.8571	40.2462	210.829
10106	2.8572	31.3586	6.6864
10107	2.8572	-2.8181	64.1345
10108	2.8573	32.5466	277.555
10109	2.8576	34.2017	329.337
10110	2.8577	38.4581	354.567
10111	2.8579	33.8815	228.945
10112	2.858	46.135	237.276
10113	2.8581	29.6241	318.244
10114	2.8582	29.1919	331.721
10115	2.8582	33.2681	200.724
10116	2.8582	31.7901	250.054
10117	2.8588	-2.9197	126.025

10118	2.8588	-2.8532	181.91
10119	2.8588	44.8445	147.682
10120	2.859	46.4001	350.002
10121	2.8592	-3.1209	226.442
10122	2.8592	-3.5934	14.16
10123	2.8593	-2.8087	184.461
10124	2.8595	44.0598	177.342
10125	2.8595	-2.7268	12.047
10126	2.8596	-4.2195	334.608
10127	2.8596	35.2418	150.469
10128	2.8596	-2.8614	253.17
10129	2.8601	-3.3315	177.367
10130	2.8604	-2.87	96.3323
10131	2.8607	36.5575	128.316
10132	2.8608	-3.6866	6.8143
10133	2.861	-2.9055	239.907
10134	2.8611	-3.0006	77.5656
10135	2.8611	33.6063	144.21
10136	2.8612	36.0833	182.527
10137	2.8614	38.1146	278.586
10138	2.8614	36.299	74.4412
10139	2.8615	54.4806	319.079
10140	2.8617	40.4919	348.675
10141	2.8618	-2.7199	24.7159
10142	2.862	-2.9837	9.8941
10143	2.862	33.1806	164.456
10144	2.8621	34.8067	160.823
10145	2.8622	-2.8702	241.302
10146	2.8624	-3.9422	326.879
10147	2.8624	-3.7008	249.393
10148	2.8625	-3.0243	22.5779
10149	2.8625	-2.7808	102.324
10150	2.8629	48.2765	115.731
10151	2.8635	49.6412	229.847
10152	2.8636	-3.3478	101.788
10153	2.8637	-2.7809	280.296
10154	2.8637	42.1595	37.8159
10155	2.8639	43.604	41.2961
10156	2.8641	32.7029	128.573
10157	2.8641	37.7169	143.226
10158	2.8642	-3.9797	2.8876
10159	2.8642	-2.8957	119.71
10160	2.8643	-2.7348	184.445
10161	2.8644	-2.9443	194.313
10162	2.8646	41.1226	18.5006
10163	2.8647	-2.9251	326.274
10164	2.8649	-3.3988	269.085
10165	2.8652	37.2775	254.412

10166	2.8656	58.1102	326.834
10167	2.8656	27.7364	151.628
10168	2.8656	-4.0586	27.4622
10169	2.866	47.0554	252.959
10170	2.866	12.1212	162.477
10171	2.8661	75.228	312.29
10172	2.8662	-3.887	197.806
10173	2.8668	51.8989	211.895
10174	2.8668	34.2059	17.1551
10175	2.867	53.1993	27.5209
10176	2.8671	-2.8028	195.62
10177	2.8672	-3.2636	163.972
10178	2.8672	41.1954	163.629
10179	2.8672	43.1738	205.81
10180	2.8673	-3.2724	250.549
10181	2.8676	35.5086	276.982
10182	2.8677	32.6295	354.724
10183	2.8678	30.2946	248.385
10184	2.8679	43.4163	294.708
10185	2.868	-2.9285	195.354
10186	2.8681	36.062	191.885
10187	2.8682	38.5915	157.683
10188	2.8682	52.3515	306.824
10189	2.8683	-3.465	84.0605
10190	2.8683	31.2804	135.323
10191	2.8684	-4.5323	339.455
10192	2.8686	41.9635	232.372
10193	2.8686	-3.5154	113.136
10194	2.8687	-4.0414	305.402
10195	2.8687	-2.693	117.324
10196	2.8687	47.5192	83.3851
10197	2.8688	1.0765	143.969
10198	2.8689	35.9361	262.997
10199	2.8694	-4.2279	83.4536
10200	2.8695	-3.7951	105.593
10201	2.8696	39.1218	1.9883
10202	2.8697	-3.0394	151.082
10203	2.8697	33.0575	179.146
10204	2.8697	-3.4797	256.587
10205	2.8698	-2.764	338.254
10206	2.8698	-4.4899	319.431
10207	2.8699	-3.1249	256.652
10208	2.87	27.0026	303.953
10209	2.8701	41.1002	110.128
10210	2.8702	-3.4436	293.162
10211	2.8703	-2.8268	329.712
10212	2.8705	-2.9913	142.334
10213	2.8705	-2.9162	259.095

10214	2.8705	24.1053	163.998
10215	2.8706	26.6341	283.245
10216	2.8711	-2.7809	119.223
10217	2.8715	54.2466	135.199
10218	2.8717	44.7033	203.19
10219	2.8718	-3.9048	50.9436
10220	2.8723	64.7963	309.403
10221	2.8726	-2.918	264.742
10222	2.8726	-2.7619	101.698
10223	2.8726	-3.2232	210.833
10224	2.8728	-2.9375	310.706
10225	2.8731	13.184	181.36
10226	2.8732	28.6584	250.157
10227	2.8733	17.473	97.3509
10228	2.8733	-2.8766	337.022
10229	2.8734	35.2982	216.387
10230	2.8735	-2.8542	354.094
10231	2.8737	52.213	46.1722
10232	2.874	29.4098	187.177
10233	2.874	-2.8932	124.452
10234	2.8745	46.9446	145.519
10235	2.8747	31.2803	210.134
10236	2.8747	31.5635	114.42
10237	2.8748	-3.2437	301.137
10238	2.8748	-2.9694	90.9225
10239	2.875	51.5591	209.078
10240	2.875	35.2472	87.2358
10241	2.875	-2.8793	100.219
10242	2.8752	42.1477	209.188
10243	2.8753	-2.8053	278.416
10244	2.8754	40.8972	158.218
10245	2.8755	46.3214	336.945
10246	2.8756	35.3196	238.197
10247	2.8759	35.1622	139.323
10248	2.8761	42.3998	328.805
10249	2.8761	-3.3634	163.295
10250	2.8766	26.6952	292.743
10251	2.8769	27.1656	172.516
10252	2.8769	37.6206	276.701
10253	2.877	32.1241	109.416
10254	2.8773	16.6148	116.515
10255	2.8774	39.3348	201.047
10256	2.8774	-2.903	238.858
10257	2.8775	46.5143	248.385
10258	2.8775	-2.7268	124.471
10259	2.8776	44.7978	212.213
10260	2.8777	32.3047	189.072
10261	2.8777	-3.9336	297.126

10262	2.8782	41.1257	118.68
10263	2.8782	-2.9522	232.366
10264	2.8782	47.2041	212.215
10265	2.8782	-3.0719	355.606
10266	2.8782	-3.4266	119.688
10267	2.8784	-2.9306	37.5856
10268	2.8787	19.1436	306.904
10269	2.8789	2.0777	110.973
10270	2.879	-2.8606	7.1515
10271	2.8792	40.0548	210.649
10272	2.8792	-2.9158	114.849
10273	2.8795	46.138	173.585
10274	2.8795	-2.943	39.9023
10275	2.8796	56.3554	4.4431
10276	2.8796	-2.8322	78.8278
10277	2.8796	-2.76	66.2168
10278	2.8798	-3.1894	122.233
10279	2.8801	34.0646	150.666
10280	2.8803	-3.911	130.064
10281	2.8804	52.5045	241.73
10282	2.8807	-3.0157	264.11
10283	2.881	47.7394	164.847
10284	2.8811	-3.0899	100.454
10285	2.8812	-3.0447	165.886
10286	2.8816	30.4184	127.738
10287	2.8818	-2.789	239.999
10288	2.8818	40.9462	195.914
10289	2.882	35.0829	109.989
10290	2.882	-4.0575	270.076
10291	2.8821	43.7822	73.3976
10292	2.8821	45.7623	71.7431
10293	2.8822	-2.859	143.762
10294	2.8823	41.7411	140.753
10295	2.8824	48.9768	130.029
10296	2.8824	-3.436	338.759
10297	2.8825	45.0687	180.055
10298	2.8826	43.161	268.124
10299	2.8829	45.1538	151.667
10300	2.883	42.8274	211.57
10301	2.8833	30.0959	196.212
10302	2.8834	56.3694	92.3273
10303	2.8836	-3.3352	135.242
10304	2.8836	37.6967	249.26
10305	2.8837	-2.859	206.048
10306	2.8837	-2.7934	55.7054
10307	2.8837	-3.1415	256.245
10308	2.8837	40.7797	334.358
10309	2.8838	-3.7221	83.3649

10310	2.8838	41.9315	70.4615
10311	2.8839	40.663	206.619
10312	2.884	-2.6983	272.03
10313	2.8841	38.5971	54.4675
10314	2.8842	-4.1859	275.179
10315	2.8844	52.6999	205.813
10316	2.8845	-4.1154	119.328
10317	2.8848	41.873	61.1291
10318	2.8851	39.8647	86.2373
10319	2.8856	-4.228	341.668
10320	2.8857	-2.9012	95.6766
10321	2.886	37.457	284.133
10322	2.8861	40.9366	252.025
10323	2.8861	41.5777	213.554
10324	2.8861	53.27	116.09
10325	2.8862	-2.878	225.15
10326	2.8863	40.697	147.93
10327	2.8863	-3.5721	121.833
10328	2.8863	-3.2111	167.393
10329	2.8865	63.4135	202.724
10330	2.8865	-2.6325	281.073
10331	2.8866	43.6577	270.473
10332	2.8868	-3.0232	184.454
10333	2.8868	-2.6957	272.788
10334	2.8869	26.5719	272.962
10335	2.887	-3.5334	165.151
10336	2.8872	16.8226	267.531
10337	2.8874	-2.8338	25.9849
10338	2.8874	-2.7358	11.8739
10339	2.8874	-4.5491	202.082
10340	2.8875	34.4141	28.9262
10341	2.8875	-2.9775	3.4809
10342	2.8878	-2.7929	150.146
10343	2.888	-4.074	139.457
10344	2.888	43.5412	346.115
10345	2.8881	49.0349	110.692
10346	2.8884	38.5573	329.411
10347	2.8885	-3.4625	285.958
10348	2.8886	36.0887	217.984
10349	2.8887	67.5618	239.336
10350	2.8889	41.0705	150.663
10351	2.889	-4.2368	202.199
10352	2.8891	37.4621	266.532
10353	2.8891	34.6865	323.845
10354	2.8891	-2.8663	330.23
10355	2.8891	-2.8148	68.6512
10356	2.8891	-2.8797	271.876
10357	2.8892	44.2683	131.915

10358	2.8892	38.5628	78.874
10359	2.8893	-2.7848	247.433
10360	2.8893	33.7188	199.182
10361	2.8897	-2.8843	318.959
10362	2.8901	-2.8116	100.006
10363	2.8904	33.0916	253.99
10364	2.8905	48.0645	6.6142
10365	2.8906	35.8528	260.256
10366	2.8907	42.4202	125.216
10367	2.8911	36.4383	219.778
10368	2.8911	31.8609	162.445
10369	2.8913	-2.8384	206.315
10370	2.8915	38.1159	220.548
10371	2.8915	38.2877	112.325
10372	2.8915	-2.8883	250.145
10373	2.8917	-3.0206	133.143
10374	2.892	-2.8804	296.153
10375	2.8923	-2.8099	269.786
10376	2.8923	-2.8547	145.687
10377	2.8924	34.7799	20.9617
10378	2.8925	47.7962	20.7275
10379	2.8928	38.1111	109.171
10380	2.8932	22.2247	331.375
10381	2.8933	-2.7969	78.6298
10382	2.8934	-2.8318	170.24
10383	2.8935	33.5122	102.862
10384	2.8938	-3.0123	66.8481
10385	2.8943	39.4673	146.118
10386	2.8946	48.14	124.889
10387	2.8947	41.4108	58.9952
10388	2.8949	-3.2892	158.508
10389	2.895	-2.9805	75.3939
10390	2.8951	-4.1656	325.02
10391	2.8955	-2.9459	99.325
10392	2.8955	51.1838	291.504
10393	2.8956	38.8068	162.17
10394	2.8956	29.8667	126.676
10395	2.8957	-2.9028	145.185
10396	2.8957	24.3913	121.381
10397	2.8957	45.3694	30.6668
10398	2.896	35.5725	149.438
10399	2.8961	43.6015	196.285
10400	2.8961	-2.8056	79.4734
10401	2.8961	-3.0464	267.982
10402	2.8962	-2.897	126.594
10403	2.8963	-3.1743	140.645
10404	2.8963	-2.9934	336.557
10405	2.8963	40.3965	41.8894

10406	2.8965	34.9748	267.336
10407	2.8965	41.8695	117.889
10408	2.8965	-2.7929	91.4421
10409	2.8968	-2.7253	206.461
10410	2.8968	56.0026	20.0841
10411	2.8969	-4.0124	261.805
10412	2.8969	34.0897	161.869
10413	2.897	52.854	24.4572
10414	2.8971	36.9022	99.6131
10415	2.8974	42.6656	194.012
10416	2.8974	-2.7131	151.266
10417	2.8974	48.3607	215.459
10418	2.8975	37.9718	212.763
10419	2.8976	33.3384	191.384
10420	2.8977	-4.2198	121.959
10421	2.8977	43.4624	85.7699
10422	2.8977	35.7993	240.47
10423	2.8977	41.64	47.3881
10424	2.8979	-5.6698	248.019
10425	2.8979	36.8635	315.783
10426	2.898	50.1797	239.746
10427	2.898	43.6932	70.5731
10428	2.8981	-2.9836	37.9651
10429	2.8982	42.856	159.843
10430	2.8983	-2.8648	210.372
10431	2.8984	-2.7966	23.6388
10432	2.8986	29.0559	51.3444
10433	2.8986	40.9036	269.18
10434	2.8987	42.9296	85.4284
10435	2.8988	-3.9031	149.49
10436	2.8989	-3.205	275.232
10437	2.899	33.3369	145.214
10438	2.899	-3.4825	103.651
10439	2.8991	32.3529	324.494
10440	2.8991	35.7823	274.168
10441	2.8991	39.6444	78.6809
10442	2.8993	-3.0477	138.457
10443	2.8994	44.4428	38.7162
10444	2.8994	36.8095	140.944
10445	2.8995	46.0638	97.0555
10446	2.8996	-2.7465	183.368
10447	2.8997	-4.0901	357.229
10448	2.8997	45.3066	241.944
10449	2.8997	-2.9323	166.254
10450	2.8998	38.6628	247.8
10451	2.8998	33.0013	195.241
10452	2.9	-3.7429	132.002
10453	2.9002	42.7399	258.319

10454	2.9003	32.2568	187.387
10455	2.9003	-2.8218	116.323
10456	2.9004	34.3963	81.2951
10457	2.9005	-2.7178	109.998
10458	2.9008	44.9319	42.9846
10459	2.9008	-2.9546	353.709
10460	2.9009	43.223	124.46
10461	2.901	35.816	289.033
10462	2.9013	47.7599	344.469
10463	2.9014	-3.6295	269.282
10464	2.9015	-3.6941	63.2652
10465	2.9016	51.0444	157.049
10466	2.9016	-3.0052	109.454
10467	2.9017	45.7093	306.854
10468	2.9018	37.5539	172.156
10469	2.9021	41.1527	238.335
10470	2.9023	43.6389	231.408
10471	2.9025	-3.2815	32.5011
10472	2.9028	-3.0367	80.0905
10473	2.9028	50.9122	49.0248
10474	2.9031	41.8034	237.862
10475	2.9032	38.6034	137.075
10476	2.9034	-3.9579	324.385
10477	2.9037	18.9022	124.175
10478	2.9038	59.9891	209.511
10479	2.904	44.6385	122.366
10480	2.9041	-2.8895	161.691
10481	2.9042	45.2577	238.625
10482	2.9046	38.6563	45.986
10483	2.9047	49.3205	97.962
10484	2.905	-2.5385	97.7515
10485	2.9051	-3.0464	232.433
10486	2.9051	34.746	148.067
10487	2.9053	47.1359	67.6662
10488	2.9054	-3.4602	30.0164
10489	2.9055	39.1174	324.367
10490	2.9055	-2.9035	53.6973
10491	2.9055	-2.9818	308.761
10492	2.9057	-3.104	190.677
10493	2.9059	37.8957	120.296
10494	2.906	48.7974	298.625
10495	2.9061	50.5755	240.047
10496	2.9061	-3.4751	127.759
10497	2.9062	39.0726	177.589
10498	2.9062	-6.7136	61.6273
10499	2.9065	53.068	44.1734
10500	2.9065	-3.3829	196.95
10501	2.9066	53.6344	319.119

10502	2.9069	50.7123	311.344
10503	2.9069	-2.9185	91.3185
10504	2.9071	-2.7842	79.6788
10505	2.9072	42.5	168.933
10506	2.9073	44.9378	192.58
10507	2.9074	-3.9334	109.12
10508	2.9074	43.4965	263.017
10509	2.9075	57.6173	280.748
10510	2.9075	-3.2334	124.918
10511	2.9075	47.5854	138.592
10512	2.9076	28.2134	141.479
10513	2.9077	48.392	60.0173
10514	2.9079	31.7639	312.27
10515	2.908	41.7713	144.603
10516	2.9082	35.1444	164.58
10517	2.9083	-3.7395	129.167
10518	2.9084	-2.7882	293.962
10519	2.9085	39.6568	4.3727
10520	2.9087	8.9432	276.181
10521	2.9087	40.2937	31.0551
10522	2.9087	52.1026	27.3792
10523	2.9091	-2.721	354.083
10524	2.9092	-2.9643	329.254
10525	2.9092	-3.1427	304.435
10526	2.9092	17.7551	215.684
10527	2.9092	42.6455	284.401
10528	2.9094	-2.8997	351.22
10529	2.9095	50.0361	285.257
10530	2.9097	48.8043	187.815
10531	2.9097	-2.8509	318.808
10532	2.9099	32.6789	87.5098
10533	2.9103	67.3408	273.002
10534	2.9103	40.103	148.274
10535	2.9103	-3.2692	163.927
10536	2.9105	39.1506	243.357
10537	2.9106	-2.7751	48.3619
10538	2.9107	42.4932	293.199
10539	2.9107	40.4198	84.668
10540	2.9108	-3.7916	87.0881
10541	2.9108	38.4186	176.085
10542	2.9109	-3.0325	137.882
10543	2.911	2.3753	127.807
10544	2.9113	38.0246	27.3361
10545	2.9114	-3.2429	168.894
10546	2.9115	36.7925	303.566
10547	2.9117	29.083	93.7503
10548	2.9118	33.2614	137.943
10549	2.9123	-2.7052	49.6386

10550	2.9126	41.8106	122.749
10551	2.9127	40.5648	153.026
10552	2.9127	51.4253	271.352
10553	2.913	31.8533	92.8199
10554	2.9133	56.8541	204.877
10555	2.9133	30.5826	262.278
10556	2.9134	37.9413	33.1377
10557	2.9137	36.2619	265.287
10558	2.9137	-4.0345	308.224
10559	2.9139	-2.8769	36.377
10560	2.914	50.6334	97.2709
10561	2.914	-2.8517	336.078
10562	2.9141	-2.8415	0.9517
10563	2.9143	-2.8511	342.943
10564	2.9143	-3.5173	47.5659
10565	2.9144	-2.9539	330.403
10566	2.9147	-2.9392	260.537
10567	2.9148	-2.7445	149.836
10568	2.9149	40.1	200.819
10569	2.915	46.2675	232.635
10570	2.9152	33.1926	281.096
10571	2.9153	-2.678	292.126
10572	2.9154	-4.234	296.604
10573	2.9154	-3.1462	318.423
10574	2.9156	-2.9551	23.4259
10575	2.9157	36.0327	158.384
10576	2.9158	37.1923	269.505
10577	2.9158	37.8857	36.167
10578	2.9158	43.4639	350.5
10579	2.9161	48.2757	278.344
10580	2.9163	-2.886	63.6094
10581	2.9163	32.2148	253.837
10582	2.9163	-2.7768	331.677
10583	2.9164	-3.2729	102.823
10584	2.9169	51.2622	127.961
10585	2.9169	38.4245	132.977
10586	2.917	34.9115	156.43
10587	2.9171	41.9023	210.072
10588	2.9171	15.5289	95.0216
10589	2.9172	-2.7724	184.42
10590	2.9172	38.7244	198.539
10591	2.9172	38.2979	77.9621
10592	2.9172	61.7834	67.638
10593	2.9174	3.9631	118.777
10594	2.9174	-2.9172	147.731
10595	2.9176	27.2492	80.3959
10596	2.9176	-2.8682	240.644
10597	2.9178	-4.2558	118.25

10598	2.918	-4.0262	339.993
10599	2.9181	48.0503	247.014
10600	2.9182	49.9054	22.7597
10601	2.9184	-2.8045	78.4377
10602	2.9186	-2.9382	306.774
10603	2.9188	28.7727	261.354
10604	2.9189	39.9468	230.005
10605	2.9189	-3.2052	285.118
10606	2.919	30.1951	213.437
10607	2.9193	-0.0134	175.002
10608	2.9193	52.8826	35.6707
10609	2.9195	-2.9449	200.865
10610	2.92	40.0289	154.26
10611	2.9201	-2.7802	331.323
10612	2.9202	48.6122	26.9983
10613	2.9208	-4.2489	60.7646
10614	2.9209	-3.2109	70.8071
10615	2.9209	44.3684	101.212
10616	2.921	-2.9067	231.596
10617	2.9211	33.0639	306.363
10618	2.9212	-3.9984	97.8054
10619	2.9212	35.3262	95.927
10620	2.9215	58.2021	334.27
10621	2.9216	-2.9131	356.728
10622	2.9217	-3.8106	15.712
10623	2.9218	53.8574	306.288
10624	2.9218	-2.7747	308.652
10625	2.9219	-4.3857	319.68
10626	2.922	30.7221	45.2564
10627	2.9224	-3.0544	282.483
10628	2.9224	-2.7863	192.041
10629	2.9224	43.2024	197.566
10630	2.9225	39.7609	192.525
10631	2.9226	31.5507	168.54
10632	2.9226	62.6142	111.684
10633	2.9227	-4.0541	169.078
10634	2.923	-3.2686	137.777
10635	2.9232	34.3992	48.0601
10636	2.9234	-3.0124	188.101
10637	2.9235	-3.3379	260.968
10638	2.9235	57.3714	240.613
10639	2.9236	-2.8621	183.923
10640	2.9236	-2.8155	130.86
10641	2.9237	-2.7011	124.493
10642	2.9237	37.7117	255.087
10643	2.9238	39.5199	182.829
10644	2.924	44.2409	194.666
10645	2.924	-3.7645	197.715

10646	2.9241	34.9058	261.912
10647	2.9242	41.501	155.283
10648	2.9242	26.4568	90.9687
10649	2.9243	41.4632	144.067
10650	2.9244	-3.3907	287.801
10651	2.9246	47.708	47.4742
10652	2.9247	-5.5345	139.009
10653	2.9248	42.9862	246.179
10654	2.9248	59.3576	51.7294
10655	2.9249	-2.963	178.162
10656	2.925	40.6958	240.862
10657	2.9252	-2.9209	223.041
10658	2.9253	38.7034	76.8578
10659	2.9253	-2.802	296.558
10660	2.9253	45.2071	242.774
10661	2.9254	19.5012	256.988
10662	2.9255	40.5494	321.819
10663	2.9257	-3.2375	349.22
10664	2.9258	41.8106	234.326
10665	2.9258	41.5311	197.99
10666	2.9259	18.3538	92.6793
10667	2.9259	44.1021	65.8541
10668	2.926	54.0392	53.9254
10669	2.926	37.1951	318.831
10670	2.9262	-2.8005	297.054
10671	2.9269	-2.8879	333.083
10672	2.927	28.304	310.683
10673	2.927	33.9743	74.4303
10674	2.927	-2.7597	139.33
10675	2.9271	-2.9542	340.245
10676	2.9271	-3.1986	330.938
10677	2.9273	41.325	219.859
10678	2.9273	35.495	274.441
10679	2.9275	41.132	166.036
10680	2.9275	35.6406	251.054
10681	2.9276	28.8466	142.762
10682	2.9277	47.4274	211.359
10683	2.9279	37.6038	83.7901
10684	2.928	28.4399	290.067
10685	2.9282	49.0178	200.567
10686	2.9283	40.5213	314.349
10687	2.9283	-2.7948	340.674
10688	2.9285	43.2155	82.9048
10689	2.9285	43.8574	62.2936
10690	2.9286	51.1984	16.2855
10691	2.9289	50.4566	115.274
10692	2.9289	49.8348	174.45
10693	2.9291	-2.7614	120.472

10694	2.9292	-3.1104	312.871
10695	2.9295	57.7072	61.4486
10696	2.9295	-2.9736	114.41
10697	2.9297	43.8976	286.38
10698	2.9297	-2.8871	252.983
10699	2.9298	51.9128	22.8632
10700	2.9298	42.7832	23.7222
10701	2.93	45.0864	172.979
10702	2.9302	55.6859	203.96
10703	2.9304	26.5371	92.205
10704	2.9305	-3.3964	103.866
10705	2.9306	32.8595	178.135
10706	2.9306	53.3689	307.271
10707	2.931	50.0065	134.497
10708	2.931	39.2795	89.4724
10709	2.9311	40.9274	141.129
10710	2.9311	43.7385	10.9111
10711	2.9312	44.6794	63.2823
10712	2.9314	-3.3299	257.677
10713	2.9315	35.9585	134.368
10714	2.9316	26.8779	134.513
10715	2.9316	-2.7463	248.806
10716	2.9317	-3.3403	136.566
10717	2.9317	-2.7704	139.864
10718	2.932	-2.9367	260.842
10719	2.932	-3.4676	187.198
10720	2.9322	-2.9712	321.487
10721	2.9323	49.7774	210.4
10722	2.9331	55.1159	304.918
10723	2.9335	39.4585	284.608
10724	2.9337	-3.3041	155.525
10725	2.9337	35.8496	80.1974
10726	2.9339	47.6271	79.0093
10727	2.9341	15.4923	122.541
10728	2.9342	33.4624	84.4841
10729	2.9342	-2.7604	124.69
10730	2.9344	-2.7693	331.725
10731	2.9345	36.0616	139.244
10732	2.9345	-3.1543	140.384
10733	2.9345	-2.7816	289.652
10734	2.9348	-3.2833	101.334
10735	2.9349	38.004	270.569
10736	2.9353	-3.2721	328.415
10737	2.9354	36.1806	359.122
10738	2.9356	55.948	207.253
10739	2.9357	-3.0913	247.575
10740	2.9357	33.4508	126.56
10741	2.9358	-3.1295	160.696

10742	2.936	-2.8795	340.661
10743	2.936	49.6012	67.3981
10744	2.936	46.588	81.1852
10745	2.9361	54.6555	79.1938
10746	2.9362	35.7457	227.367
10747	2.9362	37.0136	94.1818
10748	2.9363	57.9995	133.704
10749	2.9364	-2.6818	171.53
10750	2.9367	-4.232	135.625
10751	2.9371	-3.0863	335.008
10752	2.9372	35.651	286.774
10753	2.9372	41.3053	88.4904
10754	2.9372	-2.8361	136.614
10755	2.9372	-2.87	236.816
10756	2.9373	-2.821	214.405
10757	2.9374	60.3181	245.849
10758	2.9374	27.1294	256.614
10759	2.9376	41.2939	302.212
10760	2.9376	-3.5561	131.31
10761	2.9376	-2.8474	91.9257
10762	2.9378	47.7934	124.726
10763	2.938	-3.2485	88.1175
10764	2.938	49.6971	20.4269
10765	2.9381	37.4038	143.919
10766	2.9382	-4.0786	82.5546
10767	2.9382	15.5697	167.653
10768	2.9386	45.6312	292.24
10769	2.939	-3.8548	100.708
10770	2.939	-6.4441	339.787
10771	2.9391	-3.5522	216.973
10772	2.9394	-3.1695	286.373
10773	2.9394	39.6262	17.5605
10774	2.9395	-2.9121	141.45
10775	2.9395	-3.0061	244.09
10776	2.9395	60.514	207.767
10777	2.9396	40.6345	199.381
10778	2.9397	-3.0021	76.1773
10779	2.9399	50.0744	285.111
10780	2.9399	40.9309	249.746
10781	2.94	-3.7541	71.0059
10782	2.9401	-3.3679	355.932
10783	2.9401	-3.4841	77.741
10784	2.9402	47.651	116.283
10785	2.9404	-3.4724	102.301
10786	2.9406	48.0684	212.647
10787	2.9407	30.7844	137.184
10788	2.9411	36.681	89.619
10789	2.9411	31.4759	161.409

10790	2.9411	44.249	174.055
10791	2.9411	-2.7781	121.298
10792	2.9412	55.2088	10.3198
10793	2.9416	38.7171	234.624
10794	2.9416	-2.8409	336.54
10795	2.9417	-4.7049	251.539
10796	2.9421	-2.8454	352.124
10797	2.9421	49.3232	187.756
10798	2.9425	34.9657	96.9859
10799	2.9425	44.1841	150.095
10800	2.9426	56.9856	69.7441
10801	2.9426	47.7676	193.295
10802	2.9428	54.521	29.7299
10803	2.9428	48.6769	207.96
10804	2.9429	-2.9518	274.487
10805	2.9429	42.6157	43.9262
10806	2.943	47.3538	163.657
10807	2.943	43.7551	2.997
10808	2.9431	-3.1915	159.388
10809	2.9431	64.086	140.221
10810	2.9432	41.7441	71.7643
10811	2.9432	36.775	260.91
10812	2.9433	36.9175	168.893
10813	2.9434	39.388	193.48
10814	2.9435	-4.4696	68.4386
10815	2.9437	33.1837	202.48
10816	2.9438	35.9004	343.348
10817	2.9438	26.2268	35.3174
10818	2.9439	-3.0919	40.2953
10819	2.944	30.6245	258.449
10820	2.9442	-2.9027	314.899
10821	2.9443	63.3551	242.366
10822	2.9444	44.4576	197.702
10823	2.9444	34.5214	199.671
10824	2.9446	58.6013	27.6858
10825	2.9446	-4.3675	124.558
10826	2.9448	50.5417	207.793
10827	2.9448	-2.9932	132.866
10828	2.9448	34.0629	166.05
10829	2.945	2.4859	337.107
10830	2.9452	-3.0408	42.6423
10831	2.9453	39.5428	195.309
10832	2.9453	44.64	6.5615
10833	2.9456	-4.1675	107.33
10834	2.9459	32.8083	247.833
10835	2.946	-4.2246	249.042
10836	2.9461	-4.3026	230.516
10837	2.9462	-2.7427	26.0724

10838	2.9464	44.9187	5.6343
10839	2.9465	47.3408	289.321
10840	2.9467	-3.2071	66.3843
10841	2.9467	39.3625	126.265
10842	2.9467	48.4367	238.579
10843	2.9467	41.0659	179.508
10844	2.9467	-3.6416	277.54
10845	2.9468	43.7106	173.7
10846	2.9469	47.3838	21.9377
10847	2.9472	37.5734	228.112
10848	2.9473	40.6693	26.3165
10849	2.9476	37.6264	214.032
10850	2.9476	42.8957	146.833
10851	2.9477	32.6965	119.586
10852	2.9477	49.915	27.6779
10853	2.9478	-3.0547	217.742
10854	2.9479	61.6672	240.645
10855	2.9479	46.6537	131.236
10856	2.9482	-3.8063	85.6149
10857	2.9483	38.1545	141.128
10858	2.9484	-3.4095	68.7967
10859	2.9485	39.256	221.282
10860	2.9486	48.2438	217.368
10861	2.9486	-3.102	268.906
10862	2.9487	44.0823	250.51
10863	2.9488	-2.9423	353.35
10864	2.9489	43.1815	107.318
10865	2.9489	-2.7079	24.4088
10866	2.949	51.6504	260.653
10867	2.949	50.1372	232.309
10868	2.9493	47.4787	197.159
10869	2.9502	51.0246	30.6719
10870	2.9502	17.023	330.658
10871	2.9503	-2.9722	85.3722
10872	2.9505	48.7305	261.246
10873	2.9505	-2.796	67.9065
10874	2.9509	36.9598	289.331
10875	2.951	40.9245	34.9855
10876	2.9512	44.1859	182.766
10877	2.9513	29.1427	129.174
10878	2.9516	44.195	9.1611
10879	2.9524	-2.8541	51.2638
10880	2.9525	30.6467	322.177
10881	2.9526	34.7447	159.338
10882	2.9529	-2.97	68.8736
10883	2.9529	26.032	276.031
10884	2.953	-2.9146	99.3358
10885	2.9531	-2.78	117.94

10886	2.9532	31.2154	321.163
10887	2.9532	-3.7288	36.8446
10888	2.9535	56.0617	23.5448
10889	2.9537	39.7344	292.286
10890	2.9538	36.1153	359.228
10891	2.9538	37.8107	52.2925
10892	2.9539	50.7137	334.306
10893	2.9539	20.7115	9.7303
10894	2.9539	-3.9654	309.122
10895	2.9541	-3.8839	209.486
10896	2.9542	52.1753	266.706
10897	2.9543	-2.8478	89.4213
10898	2.9543	-4.0784	92.408
10899	2.9545	-2.9951	87.1512
10900	2.9546	41.9991	137.958
10901	2.9546	-3.9365	84.9042
10902	2.9547	54.0155	214.497
10903	2.9548	-2.8233	128.347
10904	2.9548	-3.3021	130.126
10905	2.9549	-3.368	256.725
10906	2.9549	-2.7939	5.3217
10907	2.9549	-3.8096	332.993
10908	2.955	-2.9242	44.5475
10909	2.955	-2.7232	43.5387
10910	2.9551	-2.8496	132.366
10911	2.9551	-2.7074	147.941
10912	2.9552	25.4307	67.0995
10913	2.9552	55.5839	37.8218
10914	2.9552	-2.8538	170.113
10915	2.9554	-3.7832	216.468
10916	2.9555	-2.7406	124.985
10917	2.9556	30.9554	163.67
10918	2.9558	-3.6603	96.1215
10919	2.9558	-3.2894	86.6555
10920	2.9562	-3.9169	138.406
10921	2.9562	-3.3894	105.496
10922	2.9563	40.6145	86.608
10923	2.9563	49.6193	76.3913
10924	2.9564	56.1081	6.1698
10925	2.9564	-3.2601	291.872
10926	2.9566	58.9721	231.189
10927	2.9566	42.2064	185.462
10928	2.9568	44.0504	20.2135
10929	2.9569	-2.7913	67.1023
10930	2.957	-2.8738	338.468
10931	2.9571	32.9755	196.116
10932	2.9571	-2.8168	42.1824
10933	2.9572	41.9123	182.983

10934	2.9573	46.2271	148.426
10935	2.9574	63.7849	113.382
10936	2.9576	36.7579	354.648
10937	2.9576	-4.2857	218.561
10938	2.9577	59.0141	243.299
10939	2.9577	33.7987	167.489
10940	2.9577	44.9077	77.8201
10941	2.9579	44.3368	145.761
10942	2.9581	46.0415	267.445
10943	2.9583	39.7008	274.705
10944	2.9583	47.379	106.37
10945	2.9585	-2.7191	133.54
10946	2.9588	47.9476	214.821
10947	2.9592	-2.8933	349.268
10948	2.9592	-2.83	113.569
10949	2.9596	-2.9636	121.595
10950	2.9596	41.588	41.3382
10951	2.9596	-3.0112	16.8955
10952	2.9597	-2.7931	173.8
10953	2.9598	-2.8803	81.0702
10954	2.9599	45.663	340.932
10955	2.96	-3.1776	160.413
10956	2.9602	-2.8953	87.3136
10957	2.9602	48.117	76.6417
10958	2.9603	-2.7012	359.471
10959	2.9604	19.1066	137.616
10960	2.9605	55.1703	356.032
10961	2.9606	41.861	69.5715
10962	2.9607	-2.8938	171.455
10963	2.9609	-3.0099	78.0626
10964	2.9609	53.9385	29.5969
10965	2.9611	41.4855	40.6254
10966	2.9612	-2.8471	262.278
10967	2.9612	47.8368	241.808
10968	2.9612	52.6251	184.96
10969	2.9615	-3.0306	116.42
10970	2.9617	50.5585	197.088
10971	2.962	52.2133	161.171
10972	2.9621	-4.0771	27.6783
10973	2.9622	37.9293	248.404
10974	2.9622	-3.2354	293.74
10975	2.9624	-3.2677	134.237
10976	2.9628	-3.7826	330.323
10977	2.9628	52.0504	345.819
10978	2.9632	-2.8646	203.065
10979	2.9632	49.5807	74.5901
10980	2.9633	49.2346	344.247
10981	2.9636	-2.9086	66.6188

10982	2.9638	41.513	1.0139
10983	2.9643	38.1351	247.605
10984	2.9644	49.5327	133.627
10985	2.9646	43.4265	132.543
10986	2.965	51.0772	214.276
10987	2.965	30.4437	342.649
10988	2.965	41.9373	185.064
10989	2.965	-3.419	199.336
10990	2.9653	-2.8052	324.749
10991	2.9654	-2.9589	243.839
10992	2.9654	54.6489	248.979
10993	2.9655	47.8953	206.633
10994	2.9655	44.1895	110.285
10995	2.9655	-2.9747	247.635
10996	2.9655	31.7096	238.748
10997	2.9656	52.5394	253.744
10998	2.9656	42.109	278.074
10999	2.9656	38.2066	27.0815
11000	2.9658	-2.8681	298.565
11001	2.9658	-2.916	323.524
11002	2.9658	40.169	10.6759
11003	2.9659	31.6618	126.633
11004	2.966	66.2586	29.6195
11005	2.9664	72.812	281.839
11006	2.9664	51.7296	127.208
11007	2.9664	-3.4022	264.156
11008	2.9665	56.8387	289.269
11009	2.9665	56.5439	109.628
11010	2.9667	-2.7498	303.65
11011	2.9669	63.2514	4.6255
11012	2.9669	-2.7733	354.204
11013	2.9669	48.2263	126.784
11014	2.967	-2.8976	237.656
11015	2.9672	42.7126	287.076
11016	2.9675	49.1784	276.574
11017	2.9675	31.2858	121.225
11018	2.9677	-3.6828	112.067
11019	2.9678	62.5197	297.718
11020	2.9678	41.2317	133.523
11021	2.9678	39.1265	304.859
11022	2.9678	-3.689	315.813
11023	2.9679	44.1629	284.576
11024	2.9679	-2.7933	108.097
11025	2.9682	48.5046	111.194
11026	2.9683	47.9338	56.2098
11027	2.9684	32.6805	170.289
11028	2.9686	58.447	59.9245
11029	2.9688	-2.929	130.436

11030	2.9689	-3.8439	89.5084
11031	2.9689	45.3614	170.431
11032	2.9689	38.9411	328.489
11033	2.9692	41.3466	193.997
11034	2.9692	35.2886	114.88
11035	2.9692	48.9079	245.666
11036	2.9692	-3.6969	271.443
11037	2.9696	40.0218	140.463
11038	2.9696	49.2132	138.856
11039	2.9696	-3.4246	256.999
11040	2.9697	3.6641	267.755
11041	2.9698	67.3094	205.485
11042	2.9699	79.0945	211.847
11043	2.97	63.8828	40.4315
11044	2.9701	-3.1698	31.2711
11045	2.9702	31.854	139.587
11046	2.9703	-2.8267	116.487
11047	2.9704	40.4572	304.182
11048	2.9705	-2.8773	215.459
11049	2.9705	71.6565	203.962
11050	2.9706	41.7853	12.4648
11051	2.9706	34.3843	279.651
11052	2.9707	-3.9845	190.521
11053	2.9709	52.2303	268.803
11054	2.9709	63.7521	210.414
11055	2.9709	-2.8505	38.3838
11056	2.9711	-4.0144	163.524
11057	2.9712	30.3498	165.644
11058	2.9713	-3.7045	259.496
11059	2.9713	-4.0074	10.7929
11060	2.9716	47.354	169.683
11061	2.9716	41.868	148.162
11062	2.9717	-3.1164	223.958
11063	2.9718	40.7073	149.196
11064	2.9718	-2.9175	190.367
11065	2.9718	29.6297	158.941
11066	2.972	35.1398	336.57
11067	2.972	46.7534	32.6542
11068	2.9722	34.721	144.572
11069	2.9723	-2.8312	140.035
11070	2.9724	-2.7605	175.83
11071	2.9725	58.8954	353.339
11072	2.9725	-3.961	88.6093
11073	2.9726	34.9696	308.927
11074	2.9727	57.8756	240.459
11075	2.9729	44.8797	16.0476
11076	2.9733	31.3711	317.54
11077	2.9734	-2.9266	69.6276

11078	2.9734	-2.9075	224.665
11079	2.9735	38.5353	185.018
11080	2.9736	-3.2851	212.52
11081	2.9737	36.0259	169.578
11082	2.9737	-2.832	117.574
11083	2.974	-3.0436	300.143
11084	2.9743	-3.5755	23.3873
11085	2.9746	-2.944	286.399
11086	2.9746	52.2473	78.0955
11087	2.9747	40.9689	152.159
11088	2.9747	-4.2068	0.7364
11089	2.9747	44.3156	75.9895
11090	2.9748	-3.8256	348.207
11091	2.975	43.4815	120.666
11092	2.975	46.9982	233.909
11093	2.975	47.4506	186.276
11094	2.9751	28.8264	164.068
11095	2.9751	62.5054	106.034
11096	2.9752	49.9803	78.1028
11097	2.9753	59.469	138.58
11098	2.9755	-4.2515	107.725
11099	2.9756	58.9324	32.4816
11100	2.9758	54.4044	118.85
11101	2.9763	-2.8719	170.358
11102	2.9764	48.7237	59.9456
11103	2.9765	39.4227	62.2174
11104	2.9766	-2.9966	112.013
11105	2.9768	-4.0853	85.2663
11106	2.9768	46.6835	135.132
11107	2.977	42.597	156.022
11108	2.9772	55.3361	15.4458
11109	2.9772	37.4122	233.255
11110	2.9774	51.1372	359.127
11111	2.9774	63.4008	20.6828
11112	2.9776	27.3529	167.533
11113	2.9777	54.2321	271.162
11114	2.9777	-3.2841	348.679
11115	2.9779	53.1674	19.3941
11116	2.978	-2.9384	249.028
11117	2.9784	-3.6176	237.43
11118	2.9785	48.1948	24.609
11119	2.9789	54.5113	259.589
11120	2.9789	60.875	24.3927
11121	2.9789	30.4401	195.288
11122	2.9789	41.1911	300.599
11123	2.9791	-2.8355	115.552
11124	2.9794	45.115	23.0597
11125	2.9797	28.4596	303.889

11126	2.9803	49.8116	328.145
11127	2.9805	-2.9575	70.9517
11128	2.9807	31.4142	241.578
11129	2.9808	-3.1121	16.8422
11130	2.9808	-2.8713	27.8717
11131	2.9809	30.0672	59.8198
11132	2.9809	-2.8286	77.1934
11133	2.9809	58.3955	114.499
11134	2.981	48.7681	350.062
11135	2.9811	47.3672	58.5247
11136	2.9811	49.8335	83.764
11137	2.9813	-2.8934	67.9784
11138	2.9814	-2.9756	141.944
11139	2.9814	44.404	184.94
11140	2.9815	-2.8543	127.003
11141	2.9815	-3.1044	332.766
11142	2.9818	34.9764	144.131
11143	2.9826	37.7393	104.132
11144	2.9827	-2.9257	298.454
11145	2.9827	44.0628	170.893
11146	2.9828	54.0494	175.783
11147	2.9828	-4.2518	323.04
11148	2.9829	-2.7877	194.416
11149	2.9829	54.3444	192.175
11150	2.9831	57.3541	206.452
11151	2.9833	37.3219	128.062
11152	2.9835	-2.8982	144.102
11153	2.9837	40.775	225.579
11154	2.9837	-2.8426	320.244
11155	2.9838	51.8188	74.6822
11156	2.9838	-2.6883	151.302
11157	2.9839	45.5841	175.4
11158	2.984	-2.668	80.1455
11159	2.9843	-2.8815	204.33
11160	2.9843	52.3777	167.365
11161	2.9844	60.508	342.931
11162	2.9846	41.7086	157.815
11163	2.9846	37.7126	250.126
11164	2.9848	38.9188	208.089
11165	2.9849	35.0997	316.928
11166	2.9849	40.3069	326.625
11167	2.985	46.8329	159.037
11168	2.985	75.3389	315.667
11169	2.9851	51.273	26.1239
11170	2.9852	-3.0141	347.944
11171	2.9852	47.6309	132.091
11172	2.9852	26.2703	129.365
11173	2.9856	62.4263	50.3512

11174	2.9857	60.9033	130.596
11175	2.9858	34.9258	218.473
11176	2.9859	43.7718	69.91
11177	2.986	-2.8849	272.947
11178	2.9862	57.3496	207.261
11179	2.9862	51.8262	59.6486
11180	2.9863	36.0373	162.051
11181	2.9865	41.6045	158.822
11182	2.9866	45.0174	269.81
11183	2.9869	-3.9638	339.745
11184	2.987	54.486	309.377
11185	2.987	-2.8737	45.6639
11186	2.9872	35.475	264.497
11187	2.9873	47.8687	357.706
11188	2.9873	-2.9009	44.7135
11189	2.9874	48.534	149.827
11190	2.9874	56.2775	232.99
11191	2.9875	-2.9633	135.61
11192	2.9876	38.5422	262.088
11193	2.9876	58.2597	332.31
11194	2.9876	-2.9736	331.594
11195	2.9877	37.9369	236.74
11196	2.9878	43.111	205.701
11197	2.9878	61.0709	28.206
11198	2.988	-2.9027	82.0956
11199	2.988	-3.9369	200.017
11200	2.9883	-2.9979	153.422
11201	2.9883	40.1002	266.423
11202	2.9884	38.4283	127.102
11203	2.9886	-2.9741	355.374
11204	2.9887	48.2127	172.757
11205	2.9887	47.2479	95.4489
11206	2.9889	58.138	210.333
11207	2.9889	36.8426	132.295
11208	2.989	42.8009	156.045
11209	2.989	50.5653	111.073
11210	2.9891	-4.0266	206.902
11211	2.9893	-3.3119	294.094
11212	2.9894	49.2557	240.149
11213	2.9894	-2.7381	19.9695
11214	2.9895	39.0832	108.205
11215	2.9897	-3.6995	1.4091
11216	2.99	31.231	106.905
11217	2.9902	43.947	274.278
11218	2.9905	45.5958	164.141
11219	2.9905	13.1745	107.564
11220	2.9906	-3.0948	164.011
11221	2.9907	-2.9828	137.369

11222	2.9907	-3.8133	31.5372
11223	2.9909	-4.0415	281.979
11224	2.9911	44.4309	173.353
11225	2.9911	57.1313	44.5631
11226	2.9916	46.7869	68.3172
11227	2.9917	81.7847	202.156
11228	2.9917	-3.1666	251.007
11229	2.9917	53.4074	141.297
11230	2.9919	40.3953	217.911
11231	2.9919	48.2918	114.173
11232	2.9919	47.0174	288.002
11233	2.9922	49.3888	128.719
11234	2.9922	64.042	30.3588
11235	2.9923	53.2772	296.645
11236	2.9923	-2.8398	150.461
11237	2.9924	-3.4007	225.246
11238	2.9926	58.3481	207.571
11239	2.9926	33.0413	139.883
11240	2.9926	39.9323	131.764
11241	2.9927	-2.8597	210.871
11242	2.9931	43.5677	269.943
11243	2.9931	-2.9582	225.538
11244	2.9934	-4.0714	251.223
11245	2.9935	-2.871	254.772
11246	2.9937	-3.8059	249.772
11247	2.9938	47.9993	242.705
11248	2.9939	41.7137	268.652
11249	2.9939	-3.3774	265.75
11250	2.994	-2.9047	157.105
11251	2.9941	46.4797	28.0746
11252	2.9944	45.8964	126.646
11253	2.9944	-2.913	316.355
11254	2.9947	51.6263	37.1927
11255	2.9947	-3.1094	107.37
11256	2.9949	-3.1036	84.2504
11257	2.9953	31.5494	160.283
11258	2.9955	51.4959	45.4646
11259	2.9956	-2.9062	347.986
11260	2.9956	41.6275	153.658
11261	2.9956	-2.8197	358.669
11262	2.9958	55.0263	203.606
11263	2.9959	40.2418	173.108
11264	2.996	38.66	174.979
11265	2.9961	46.4501	169.124
11266	2.9961	-2.9974	248.273
11267	2.9961	64.5034	113.674
11268	2.9961	50.1074	227.333
11269	2.9962	-2.8768	341.135

11270	2.9962	-2.9924	320.536
11271	2.9962	40.0498	130.134
11272	2.9962	35.0826	176.641
11273	2.9962	45.082	160.465
11274	2.9964	-2.9544	65.216
11275	2.9964	-2.8896	272.007
11276	2.9964	-3.2898	49.3233
11277	2.9965	61.5857	38.1929
11278	2.9966	40.944	260.25
11279	2.9967	46.6302	1.6784
11280	2.9968	70.7039	201.839
11281	2.9969	41.1641	106.237
11282	2.997	32.4438	126.354
11283	2.9971	-3.9405	340.232
11284	2.9971	43.6902	142.8
11285	2.9972	72.097	247.785
11286	2.9977	-2.8769	312.583
11287	2.998	43.3187	59.4938
11288	2.9981	-3.1682	272.008
11289	2.9984	-3.5769	79.1172
11290	2.9985	47.4899	150.717
11291	2.9986	-3.3281	314.917
11292	2.9987	48.6381	255.829
11293	2.9988	-3.1867	130.64
11294	2.9988	51.8928	147.362
11295	2.9988	63.2972	317.024
11296	2.9988	49.8112	68.496
11297	2.999	-2.8731	318.418
11298	2.999	-3.1988	308.39
11299	2.999	-2.9245	9.3509
11300	2.9991	48.893	125.385
11301	2.9991	38.1136	334.05
11302	2.9991	54.426	248.856
11303	2.9994	37.9235	97.4705
11304	2.9994	49.5941	89.8605
11305	2.9995	35.5074	274.073
11306	2.9996	46.5258	191.239
11307	2.9999	48.9507	20.5322
11308	3	2.6317	269.945
11309	3.0001	50.4999	118.483
11310	3.0001	-2.8343	39.4853
11311	3.0002	-2.7994	98.6006
11312	3.0002	47.639	81.6982
11313	3.0006	-3.5185	350.446
11314	3.0006	63.253	251.915
11315	3.001	47.707	181.798
11316	3.001	60.1754	141.04
11317	3.001	-4.165	345.942

11318	3.0011	50.6415	115.391
11319	3.0011	-3.5676	266.62
11320	3.0012	-3.8696	249.742
11321	3.0013	-3.7592	88.5105
11322	3.0013	65.9543	0.8145
11323	3.0017	-2.9162	193.15
11324	3.0017	51.8288	202.424
11325	3.0017	-2.7619	240.223
11326	3.0019	-3.3795	99.9732
11327	3.0021	-2.8845	61.0956
11328	3.0022	55.2503	132.83
11329	3.0022	42.0359	251.436
11330	3.0022	39.1864	175.392
11331	3.0022	48.8003	30.5586
11332	3.0024	38.3077	158.376
11333	3.0028	-2.902	96.6581
11334	3.0029	-2.7885	174.016
11335	3.0029	-2.8723	231.833
11336	3.003	46.546	134.554
11337	3.003	-2.6856	149.13
11338	3.0031	42.9269	103.252
11339	3.0036	-2.8957	330.101
11340	3.0037	-3.4521	297.198
11341	3.0038	19.2112	192.537
11342	3.0039	45.2431	30.8167
11343	3.0041	50.9147	275.926
11344	3.0042	-3.9118	331.78
11345	3.0043	-2.793	132.139
11346	3.0043	-2.7833	161.835
11347	3.0045	60.9249	157.862
11348	3.0045	43.0109	184.68
11349	3.0047	-2.9664	88.9482
11350	3.0048	42.0442	150.869
11351	3.005	59.4709	53.4532
11352	3.005	81.7084	311.73
11353	3.005	-3.4297	135.279
11354	3.0051	-3.3575	200.604
11355	3.0052	69.1193	35.6173
11356	3.0052	-3.8083	21.423
11357	3.0054	-3.7181	59.9402
11358	3.0055	42.47	152.461
11359	3.0055	-3.6036	35.2629
11360	3.0056	51.6086	158.709
11361	3.0057	-3.0655	357.079
11362	3.0061	-2.7273	15.6064
11363	3.0061	68.5564	126.658
11364	3.0062	-3.4403	138.085
11365	3.0062	-2.8269	334.367

11366	3.0062	40.0199	93.8552
11367	3.0064	-2.7726	253.55
11368	3.0066	47.3783	168.11
11369	3.0066	48.0457	56.3461
11370	3.0067	-4.4276	116.197
11371	3.0067	61.2437	27.9362
11372	3.0069	57.8088	277.149
11373	3.007	-2.8054	340.325
11374	3.0071	-2.9102	251.553
11375	3.0071	43.4485	324.689
11376	3.0073	52.9464	197.407
11377	3.0073	45.343	287.289
11378	3.0073	-4.5931	112.292
11379	3.0073	56.7766	36.7645
11380	3.0074	43.026	97.0388
11381	3.0074	43.0144	2.6809
11382	3.0074	41.2376	0.2429
11383	3.0076	53.481	200.392
11384	3.0076	47.7203	194.872
11385	3.0076	47.5792	280.604
11386	3.0076	41.9356	137.075
11387	3.0076	-2.8986	167.742
11388	3.0077	-2.9829	120.558
11389	3.0079	28.8977	322.265
11390	3.008	45.6357	79.1729
11391	3.0087	81.1565	314.052
11392	3.0088	-2.9468	92.8088
11393	3.0089	39.9138	31.8835
11394	3.0091	54.1577	34.9836
11395	3.0091	46.3703	81.4909
11396	3.0092	51.9647	145.109
11397	3.0093	44.2242	126.065
11398	3.0094	53.2483	215.651
11399	3.0094	41.6657	161.187
11400	3.0094	44.3467	234.875
11401	3.0096	55.0284	39.7588
11402	3.0096	0.9985	110.372
11403	3.0098	51.605	142.47
11404	3.0098	-3.1393	147.484
11405	3.0099	-2.7569	76.5031
11406	3.0099	57.281	91.0036
11407	3.01	42.9146	185.76
11408	3.01	37.703	260.929
11409	3.01	48.735	139.392
11410	3.01	-2.7394	308.908
11411	3.0102	55.6557	44.2554
11412	3.0103	44.7483	142.93
11413	3.0103	49.3368	234.349

11414	3.0105	47.528	263.853
11415	3.0106	38.1625	342.944
11416	3.0108	-2.9109	337.927
11417	3.0108	45.7956	158.463
11418	3.0108	-4.0049	26.7109
11419	3.0109	54.0007	276.157
11420	3.011	-2.8581	307.069
11421	3.0111	51.096	150.033
11422	3.0111	40.5562	155.606
11423	3.0112	-3.9636	288.976
11424	3.0115	50.2987	47.6708
11425	3.0115	41.346	339.008
11426	3.0115	-2.9759	83.321
11427	3.0118	62.9582	212.791
11428	3.012	55.1739	351.188
11429	3.0121	-3.7652	58.3642
11430	3.0122	-2.9079	356.794
11431	3.0125	20.0019	262.22
11432	3.0126	-3.0088	253.328
11433	3.0128	-2.9034	268.561
11434	3.0129	51.2768	106.576
11435	3.0134	56.2592	131.186
11436	3.0134	42.4377	158.579
11437	3.0134	47.3929	110.783
11438	3.0138	47.8884	30.633
11439	3.014	-2.9217	131.217
11440	3.0141	70.0913	208.768
11441	3.0142	-2.795	118.605
11442	3.0142	-3.6279	352.085
11443	3.0142	-2.8982	287.868
11444	3.0143	45.8561	228.969
11445	3.0145	-2.7932	234.733
11446	3.0145	-2.8849	200.775
11447	3.0147	-3.189	336.138
11448	3.0149	60.267	341.724
11449	3.015	-2.9705	149.392
11450	3.0151	55.5094	242.914
11451	3.0152	58.1128	222.159
11452	3.0152	-2.8161	168.547
11453	3.0153	43.8672	91.4658
11454	3.0154	22.7395	321.633
11455	3.0156	-3.0686	183.051
11456	3.0157	44.7753	177.204
11457	3.0158	51.5806	27.776
11458	3.0158	-2.9347	105.553
11459	3.0159	54.4144	206.881
11460	3.016	-2.7365	16.1073
11461	3.0161	56.7605	129.422

11462	3.0164	53.2109	126.911
11463	3.0165	33.43	218.698
11464	3.0166	-2.9349	110.963
11465	3.0166	48.7796	110.318
11466	3.0166	50.8149	357.077
11467	3.0167	44.7719	76.5614
11468	3.0169	31.1606	149.951
11469	3.0169	-3.6006	320.079
11470	3.0169	52.7009	24.9513
11471	3.017	63.8514	229.774
11472	3.0171	40.8335	52.1224
11473	3.0173	71.4948	35.922
11474	3.0174	56.6961	243.974
11475	3.0175	44.6991	111.554
11476	3.0177	-3.2513	111.37
11477	3.0179	37.0878	51.4967
11478	3.0179	49.8977	309.424
11479	3.018	23.2943	132.434
11480	3.0181	-3.0037	281.62
11481	3.0183	8.7855	202.402
11482	3.0185	39.0312	166.224
11483	3.0187	56.6073	183.85
11484	3.0187	59.7926	141.561
11485	3.0189	-2.6933	263.822
11486	3.019	60.8325	28.0747
11487	3.019	53.5591	95.3842
11488	3.019	61.0187	37.9511
11489	3.0191	49.8172	348.523
11490	3.0191	31.3047	244.565
11491	3.0191	-3.3961	279.305
11492	3.0192	43.9747	132.008
11493	3.0192	-2.8242	82.3102
11494	3.0193	-3.1692	194.516
11495	3.0194	60.3803	142.005
11496	3.0195	-3.3835	143.659
11497	3.0196	29.5056	241.045
11498	3.0201	50.1945	37.97
11499	3.0205	-2.7523	124.318
11500	3.0206	46.0946	64.4231
11501	3.0208	41.9864	278.764
11502	3.0214	46.5902	293.654
11503	3.0214	-2.9464	308.925
11504	3.0215	-2.9946	271.191
11505	3.0215	39.4262	68.3384
11506	3.0215	-2.8691	304.344
11507	3.0217	46.3192	17.0208
11508	3.0218	-2.879	187.324
11509	3.022	60.4052	197.86

11510	3.0223	-3.442	0.2622
11511	3.0225	17.26	268.949
11512	3.0225	47.4236	153.711
11513	3.0225	-2.9783	222.207
11514	3.0226	41.0489	228.203
11515	3.0227	-2.9349	339.205
11516	3.0227	-2.8641	320.417
11517	3.0227	-2.8809	141.257
11518	3.0228	-3.4605	293.488
11519	3.0228	53.933	202.526
11520	3.0229	44.3554	115.776
11521	3.0229	57.1366	106.097
11522	3.023	55.945	353.101
11523	3.0231	-2.8538	192.111
11524	3.0232	50.3918	315.466
11525	3.0233	45.7169	122.002
11526	3.0233	56.5551	11.0004
11527	3.0234	65.6219	252.303
11528	3.0235	71.7708	238.135
11529	3.0236	-3.0688	57.963
11530	3.0237	56.2417	208.053
11531	3.0237	72.4713	103.51
11532	3.0238	-3.2703	80.9771
11533	3.0238	-4.0069	282.078
11534	3.0238	-3.8825	328.56
11535	3.024	39.2798	154.613
11536	3.0242	55.8551	145.422
11537	3.0243	-2.8423	87.3645
11538	3.0243	67.4245	72.008
11539	3.0243	-3.8549	73.0522
11540	3.0245	52.6992	282.232
11541	3.0245	42.8782	261.768
11542	3.0245	41.9077	285.551
11543	3.0246	50.7294	202.931
11544	3.0248	50.2837	26.268
11545	3.025	46.5816	218.47
11546	3.0252	49.8604	210.01
11547	3.0253	48.4129	95.244
11548	3.0254	52.0229	69.2537
11549	3.0257	46.6198	214.546
11550	3.0257	45.4761	307.4
11551	3.0257	48.3334	50.3156
11552	3.0258	-2.8995	334.309
11553	3.0259	35.8168	159.518
11554	3.0259	36.216	124.688
11555	3.0259	-3.0551	120.615
11556	3.0259	-2.9855	320.807
11557	3.026	-2.8086	123.94

11558	3.0262	-3.3766	95.4447
11559	3.0263	90.3327	315.7
11560	3.0264	40.6691	128.85
11561	3.0265	-3.2787	311.97
11562	3.0265	50.9617	29.898
11563	3.0266	43.1842	266.992
11564	3.0266	-2.767	130.75
11565	3.0267	-2.7878	322.49
11566	3.0268	59.2282	16.5859
11567	3.0268	-2.7597	320.129
11568	3.0268	53.325	151.902
11569	3.0269	-2.8542	31.7702
11570	3.0271	63.6693	96.108
11571	3.0273	-4.5064	200.555
11572	3.0274	65.0572	226.479
11573	3.0274	43.1583	168.19
11574	3.0275	-2.9495	350.22
11575	3.0279	-2.7691	300.631
11576	3.028	-2.8977	212.201
11577	3.028	42.7823	138.94
11578	3.028	41.6539	173.718
11579	3.0282	49.4386	162.278
11580	3.0283	45.2011	155.082
11581	3.0286	51.1293	202.602
11582	3.0287	50.9013	241.872
11583	3.0287	-3.2281	3.8449
11584	3.0288	47.9568	188.375
11585	3.0289	-2.8406	326.733
11586	3.0289	47.5816	175.629
11587	3.029	-2.9679	24.8759
11588	3.0294	-2.8133	164.265
11589	3.0296	50.3085	240.275
11590	3.0297	-2.9508	47.3375
11591	3.0298	50.8853	95.8943
11592	3.0299	55.7925	240.069
11593	3.0301	-2.7369	94.2931
11594	3.0302	25.4003	121.946
11595	3.0302	51.4335	203.762
11596	3.0304	-2.8481	143.316
11597	3.0306	47.697	13.844
11598	3.0308	41.9728	154.947
11599	3.0308	-2.8269	113.42
11600	3.031	47.2445	153.024
11601	3.031	-3.8132	261.377
11602	3.0312	-3.3868	304.232
11603	3.0313	-3.8812	343.284
11604	3.0315	50.0635	175.711
11605	3.0315	65.8398	87.0596

11606	3.0315	61.9578	213.555
11607	3.0315	32.7067	212.595
11608	3.0315	57.4312	160.589
11609	3.0315	-2.8928	105.021
11610	3.0316	34.8415	59.9594
11611	3.0316	27.9094	297.308
11612	3.0316	45.698	348.814
11613	3.0317	-3.0233	320.703
11614	3.0317	-2.7463	272.185
11615	3.0317	42.8906	300.624
11616	3.0319	70.0621	290.019
11617	3.0321	47.0981	198.887
11618	3.0327	46.7957	359.904
11619	3.0328	-4.1695	122.673
11620	3.0329	-2.7911	337.723
11621	3.0331	55.646	18.2765
11622	3.0332	80.493	307.924
11623	3.0334	64.0463	9.754
11624	3.0335	31.7956	276.496
11625	3.0336	39.9461	85.7361
11626	3.0337	52.2199	247.507
11627	3.0339	46.7141	37.0581
11628	3.0339	-3.4314	183.045
11629	3.0342	-2.859	217.029
11630	3.0343	-4.6429	71.4632
11631	3.0343	-2.7699	41.9739
11632	3.0344	39.1018	193.049
11633	3.0346	55.1579	330.046
11634	3.0348	-3.5432	186.342
11635	3.0348	-2.7305	34.5696
11636	3.0348	35.3364	140.857
11637	3.0348	-2.7778	308.949
11638	3.0348	-2.9566	353.231
11639	3.0349	44.6364	130.143
11640	3.0349	52.1176	245.67
11641	3.035	48.9158	332.487
11642	3.0352	47.6004	219.273
11643	3.0354	50.2864	136.309
11644	3.0355	-2.8409	193.48
11645	3.0356	-3.4809	168.255
11646	3.0357	37.461	137.197
11647	3.0357	85.083	314.223
11648	3.0358	18.0575	234.375
11649	3.0358	62.2311	233.762
11650	3.0363	42.5258	140.463
11651	3.0364	59.1644	273.584
11652	3.0364	-4.3099	256.264
11653	3.0366	57.712	139.957

11654	3.0367	34.543	44.524
11655	3.0369	46.1509	89.0545
11656	3.037	43.7054	57.0628
11657	3.0371	43.0835	105.563
11658	3.0372	40.0116	164.057
11659	3.0373	-3.6506	136.308
11660	3.0373	-3.9634	250.035
11661	3.0375	50.2963	75.4731
11662	3.0375	-2.9509	96.4846
11663	3.0376	-3.35	172.435
11664	3.0378	50.6644	184.465
11665	3.0379	41.9552	136.343
11666	3.038	61.4047	146.929
11667	3.0383	47.7471	143.788
11668	3.0384	33.7041	164.69
11669	3.0386	43.1158	156.218
11670	3.0387	54.5923	285.972
11671	3.0387	-2.805	300.504
11672	3.0388	-3.1305	349.941
11673	3.0388	37.2237	120.255
11674	3.0389	54.6353	106.154
11675	3.0389	66.0496	177.668
11676	3.0389	74.2276	203.965
11677	3.0389	-2.8211	205.323
11678	3.0389	50.8979	345.702
11679	3.039	-3.9822	4.1941
11680	3.039	57.9748	278.267
11681	3.0391	55.0438	123.754
11682	3.0392	59.8411	8.2129
11683	3.0393	-3.3397	262.886
11684	3.0393	61.6274	45.0757
11685	3.0394	36.4945	36.1977
11686	3.0398	50.2142	152.53
11687	3.04	57.9718	6.7257
11688	3.0401	57.1003	109.446
11689	3.0401	52.4659	24.8292
11690	3.0402	54.6517	279.665
11691	3.0403	42.4595	179.154
11692	3.0404	49.4884	202.482
11693	3.0405	67.3477	87.761
11694	3.0405	55.0826	239.618
11695	3.0406	51.9451	277.229
11696	3.0406	54.1385	70.7772
11697	3.0407	-2.9774	26.693
11698	3.0407	-3.5671	167.97
11699	3.0408	-2.8035	95.6284
11700	3.041	-2.8253	208.489
11701	3.041	40.3324	136.617

11702	3.041	74.7	25.0263
11703	3.0413	-3.909	184.981
11704	3.0414	-2.8816	274.57
11705	3.0415	57.6975	249.957
11706	3.0415	41.6906	141.457
11707	3.0415	0.1051	338.915
11708	3.0415	-3.4023	353.072
11709	3.0417	11.8541	225.648
11710	3.0418	47.4649	1.6004
11711	3.0418	-3.4913	243.065
11712	3.0419	59.1648	80.4071
11713	3.0419	51.175	68.2973
11714	3.0419	58.7974	213.009
11715	3.0419	49.9606	26.4653
11716	3.0419	49.152	13.7522
11717	3.0423	20.2673	61.9789
11718	3.0424	51.9082	115.368
11719	3.0424	38.6453	130.964
11720	3.0424	-2.7633	117.715
11721	3.0425	-2.8769	205.449
11722	3.0429	-4.4511	351.879
11723	3.043	-2.7109	305.812
11724	3.043	43.4398	304.38
11725	3.0431	59.0673	86.8183
11726	3.0431	-2.9931	276.351
11727	3.0434	51.6613	165.207
11728	3.0436	56.5123	113.41
11729	3.0438	55.032	106.676
11730	3.0438	-2.8733	172.2
11731	3.0439	62.0022	0.6469
11732	3.0441	38.085	130.298
11733	3.0441	48.4197	165.418
11734	3.0443	-3.3452	169.463
11735	3.0444	-3.8311	269.285
11736	3.0445	75.0971	214.159
11737	3.0447	-2.9334	238.431
11738	3.0448	61.5729	299.745
11739	3.0451	-4.4056	216.307
11740	3.0453	-2.7766	187.407
11741	3.0455	48.9745	153.759
11742	3.0457	-2.8497	140.628
11743	3.0457	-2.8707	193.481
11744	3.0458	68.1027	71.7503
11745	3.0458	48.8101	144.339
11746	3.0461	-2.9715	116.417
11747	3.0462	63.1485	134.191
11748	3.0464	50.9951	83.5913
11749	3.0465	44.551	332.682

11750	3.0466	-2.8756	270.699
11751	3.0468	61.7193	239.623
11752	3.0469	65.8088	207.269
11753	3.047	-2.808	166.691
11754	3.0471	44.4125	59.8803
11755	3.0471	-3.9128	131.487
11756	3.0473	53.0609	76.8279
11757	3.0473	59.3471	155.302
11758	3.0473	64.8542	88.6646
11759	3.0473	64.0559	33.3098
11760	3.0474	-2.6995	10.3056
11761	3.0475	-3.9973	216.263
11762	3.0476	-2.8888	77.2228
11763	3.0477	37.17	298.876
11764	3.0477	21.0416	219.281
11765	3.048	47.2972	179.389
11766	3.048	50.996	276.379
11767	3.0483	-2.7234	327.648
11768	3.0484	52.09	73.2715
11769	3.0485	45.659	325.812
11770	3.0486	58.3705	77.4083
11771	3.0486	0.2296	241.138
11772	3.0486	-3.8463	222.319
11773	3.0488	34.1091	181.965
11774	3.0488	-2.7964	283.132
11775	3.0489	67.7572	28.1857
11776	3.0489	61.1451	341.865
11777	3.049	64.6719	287.229
11778	3.049	-4.8143	222.434
11779	3.0493	86.598	315.687
11780	3.0494	-3.9034	130.126
11781	3.0494	66.0737	67.7437
11782	3.0495	42.853	159.315
11783	3.0497	59.4547	107.29
11784	3.0498	-2.7478	131.005
11785	3.0498	56.0147	70.6425
11786	3.0499	-2.8558	158.728
11787	3.0499	-3.3456	151.915
11788	3.0499	51.9123	38.1954
11789	3.0499	-2.7606	321.424
11790	3.0499	38.8381	216.329
11791	3.0501	-3.8317	316.349
11792	3.0503	-2.7892	48.8835
11793	3.0504	49.48	38.3084
11794	3.0505	66.4721	308.307
11795	3.0505	-2.7193	263.788
11796	3.0506	43.7599	306.439
11797	3.0508	-3.3523	282.208

11798	3.0511	64.7032	239.067
11799	3.0512	51.4937	158.73
11800	3.0512	-2.9507	72.8579
11801	3.0512	-3.9975	196.163
11802	3.0515	-2.8013	277.42
11803	3.0518	49.7104	15.5145
11804	3.0518	53.7937	24.1946
11805	3.0522	41.4238	228.024
11806	3.0522	-2.813	208.153
11807	3.0524	49.3606	171.139
11808	3.0524	-3.2079	142.978
11809	3.0526	56.8318	29.7384
11810	3.0528	61.0294	95.1136
11811	3.0529	-2.9753	152.302
11812	3.0531	62.0478	269.264
11813	3.0533	61.4046	37.2261
11814	3.0534	62.6264	138.126
11815	3.0534	-3.997	210.299
11816	3.0534	-2.8585	49.704
11817	3.0534	65.6758	187.972
11818	3.0541	-4.078	213.013
11819	3.0543	-3.8352	117.615
11820	3.0544	-2.6843	170.522
11821	3.0547	-2.9004	333.016
11822	3.0549	-3	38.5486
11823	3.055	60.1372	38.4872
11824	3.0557	-3.4508	50.9765
11825	3.0558	62.901	135.973
11826	3.0559	53.3479	125.844
11827	3.0562	45.7748	219.685
11828	3.0564	46.5949	153.792
11829	3.0566	67.1982	69.5381
11830	3.0566	-2.8877	316.117
11831	3.0567	-3.8563	76.8699
11832	3.057	-3.8791	190.135
11833	3.057	44.906	295.729
11834	3.057	63.901	85.236
11835	3.057	50.3523	241.478
11836	3.0571	53.1509	40.3498
11837	3.0572	-4.4339	95.7227
11838	3.0572	46.0807	298.937
11839	3.0575	54.2959	125.903
11840	3.0575	-3.4189	313.363
11841	3.0576	-2.9848	263.615
11842	3.0577	36.2691	106.677
11843	3.0579	52.8481	206.217
11844	3.058	52.3205	196.171
11845	3.0582	-3.4495	138.828

11846	3.0583	-2.9839	23.8644
11847	3.0583	50.306	150.183
11848	3.0583	35.8072	139.941
11849	3.0584	46.1331	358.715
11850	3.0585	-3.5715	302.311
11851	3.0585	-3.1522	294.592
11852	3.0585	-3.8735	281.124
11853	3.0587	47.1388	20.5033
11854	3.0588	-3.9919	171.252
11855	3.0589	-3.5398	269.705
11856	3.0589	53.6038	22.2284
11857	3.0591	72.7839	186.745
11858	3.0592	42.385	132.147
11859	3.0592	42.8183	249.723
11860	3.0593	37.6295	210.224
11861	3.0594	44.358	96.9559
11862	3.0596	-2.837	332.844
11863	3.0599	56.0824	70.2155
11864	3.06	-2.9825	54.0506
11865	3.06	-2.8853	271.859
11866	3.0601	41.5484	81.6433
11867	3.0605	37.5345	32.8972
11868	3.0606	50.0724	292.445
11869	3.0606	52.3424	192.188
11870	3.0607	-3.3359	352.145
11871	3.0608	33.8512	278.549
11872	3.0609	-3.9697	166.206
11873	3.0609	-2.8154	213.354
11874	3.061	23	72.9519
11875	3.061	-4.0109	178.653
11876	3.0611	47.5211	262.74
11877	3.0616	54.0587	54.6648
11878	3.0618	45.5332	351.258
11879	3.0619	57.5165	230.867
11880	3.0622	47.2993	23.9409
11881	3.0624	60.2315	325.811
11882	3.0626	-4.6359	69.1654
11883	3.0628	47.3356	114.247
11884	3.0632	69.2255	210.674
11885	3.0632	56.8976	129.836
11886	3.0632	7.5607	87.4168
11887	3.0632	57.4366	64.327
11888	3.0633	72.7986	230.915
11889	3.0634	65.6736	135.989
11890	3.0635	67.613	98.0689
11891	3.0635	60.4987	291.752
11892	3.0637	67.81	313.506
11893	3.0639	54.4751	206.662

11894	3.0641	50.9942	210.36
11895	3.0642	53.8431	278.375
11896	3.0642	-2.9702	329.111
11897	3.0643	48.2863	310.625
11898	3.0644	55.7313	140.036
11899	3.0645	52.3798	220.083
11900	3.0645	-3.0425	308.149
11901	3.0645	-2.7951	117.876
11902	3.0647	55.4442	59.0999
11903	3.065	-2.6847	151.691
11904	3.0651	57.7858	148.016
11905	3.0651	57.6628	31.532
11906	3.0652	42.4545	246.889
11907	3.0653	58.7364	144.182
11908	3.0653	65.3527	118.373
11909	3.0655	72.0119	198.747
11910	3.0656	-4.2364	16.6225
11911	3.0659	58.7438	89.6543
11912	3.066	52.9018	99.5099
11913	3.066	-2.847	331.216
11914	3.0661	51.6342	357.283
11915	3.0661	58.6969	185.87
11916	3.0662	66.9455	243.798
11917	3.0662	-2.9446	326.862
11918	3.0663	48.0759	128.074
11919	3.0663	-3.7428	154.399
11920	3.0663	-2.9338	194.982
11921	3.0664	52.9724	314.084
11922	3.0665	50.0133	214.661
11923	3.0665	-2.7715	133.207
11924	3.0668	-4.2168	358.307
11925	3.0669	-2.8414	120.686
11926	3.067	57.599	328.095
11927	3.067	50.8367	111.612
11928	3.0671	-2.9731	219.579
11929	3.0671	61.0978	103.125
11930	3.0672	49.674	175.836
11931	3.0677	-2.9329	250.891
11932	3.0679	64.6155	130.576
11933	3.0679	51.5528	187.872
11934	3.0679	-2.97	21.0955
11935	3.0681	52.0452	173.093
11936	3.0686	47.6895	175.1
11937	3.0686	57.6468	292.579
11938	3.0689	72.3274	143.925
11939	3.0689	-2.9839	27.8839
11940	3.0689	72.0016	27.1766
11941	3.0689	47.3051	137.14

11942	3.0691	44.7201	340.755
11943	3.0691	49.3655	171.293
11944	3.0691	-2.7796	68.5807
11945	3.0692	95.2752	323.863
11946	3.0694	41.5002	326.447
11947	3.0696	64.0882	262.482
11948	3.0696	56.3774	242.624
11949	3.0697	68.5235	359.351
11950	3.0702	40.3112	92.3692
11951	3.0702	46.4249	170.906
11952	3.0702	60.9822	75.4338
11953	3.0706	-3.5365	33.2296
11954	3.0706	50.9424	84.5922
11955	3.0707	71.4992	202.214
11956	3.0707	-4.2566	184.541
11957	3.0709	29.704	330.858
11958	3.071	46.1933	268.659
11959	3.0711	45.5857	48.3406
11960	3.0712	85.4578	307.259
11961	3.0714	53.0839	251.561
11962	3.0716	57.4636	336.709
11963	3.0718	-2.7862	117.721
11964	3.0719	-2.8113	87.8262
11965	3.0719	-2.8431	276.754
11966	3.0719	57.1287	77.389
11967	3.0719	60.1416	114.283
11968	3.0721	43.4315	145.052
11969	3.0722	56.3787	352.113
11970	3.0723	-3.3735	146.228
11971	3.0725	53.2907	161.483
11972	3.0727	56.6195	25.7236
11973	3.0728	-3.4478	189.02
11974	3.0728	-3.4635	83.9658
11975	3.0728	57.5941	34.9572
11976	3.0729	55.6451	306.157
11977	3.0731	48.6619	137.696
11978	3.0733	80.2931	58.5738
11979	3.0734	56.3346	332.396
11980	3.0736	-4.2406	100.947
11981	3.0736	109.952	313.052
11982	3.0739	60.6474	20.3413
11983	3.074	40.6473	128.246
11984	3.074	43.7896	89.6973
11985	3.0742	40.8129	159.174
11986	3.0742	54.6683	6.6605
11987	3.0742	-2.993	278.726
11988	3.0744	-2.8745	318.863
11989	3.0746	-3.1184	0.3787

11990	3.0747	-2.8312	184.438
11991	3.0749	51.3445	199.698
11992	3.0749	-4.4774	74.4395
11993	3.075	75.6691	220.616
11994	3.075	39.4103	156.724
11995	3.075	61.4622	41.1668
11996	3.0752	-2.8476	249.1
11997	3.0753	45.9654	122.089
11998	3.0754	48.3477	331.745
11999	3.0755	41.7852	138.623
12000	3.0755	43.239	211.615
12001	3.0756	41.0321	272.493
12002	3.0756	-2.7929	114.241
12003	3.0756	49.8961	118.65
12004	3.0759	-4.2025	123.75
12005	3.0759	50.5067	99.3754
12006	3.0759	49.4972	318.725
12007	3.076	48.2993	179.056
12008	3.0761	46.2591	241.837
12009	3.0763	52.3556	149.993
12010	3.0764	-2.6785	146.898
12011	3.0764	-3.799	181.445
12012	3.0765	49.0388	225.619
12013	3.0767	-3.3915	314.776
12014	3.0768	58.1792	162.827
12015	3.0769	67.8022	205.685
12016	3.0769	-2.9896	260.99
12017	3.0771	52.2638	248.386
12018	3.0771	-2.9825	135.163
12019	3.0773	-4.1752	97.191
12020	3.0774	12.2125	156.317
12021	3.0776	48.0506	106.045
12022	3.0776	-4.1844	238.047
12023	3.0777	64.0298	98.4706
12024	3.0777	44.5168	123.75
12025	3.0777	41.1793	161.383
12026	3.0777	23.7326	158.995
12027	3.0777	-2.9327	323.268
12028	3.0778	39.48	70.9747
12029	3.0778	46.1516	159.449
12030	3.0782	-3.4381	120.373
12031	3.0783	70.2941	322.772
12032	3.0784	-2.7593	116.702
12033	3.0784	-2.846	186.23
12034	3.0785	55.999	208.813
12035	3.0787	47.6127	96.7803
12036	3.0789	44.0996	89.4023
12037	3.0792	57.0378	288.61

12038	3.0793	40.2853	280.197
12039	3.0793	59.6916	242.734
12040	3.0793	-3.2738	200.278
12041	3.0794	56.1438	89.6622
12042	3.0795	48.8711	173.585
12043	3.0797	76.5026	311.846
12044	3.0797	36.7567	289.131
12045	3.0797	-2.9378	7.4468
12046	3.0798	-3.6337	333.513
12047	3.08	-3.5863	25.5078
12048	3.0801	51.7755	265.021
12049	3.0801	-2.6965	119
12050	3.0802	0.9549	104.253
12051	3.0802	-2.7381	183.941
12052	3.0802	-2.7341	17.576
12053	3.0803	-2.9646	298.252
12054	3.0803	-2.9073	5.3624
12055	3.0804	-2.8481	14.1105
12056	3.0804	42.7673	152.251
12057	3.0805	59.9344	93.2385
12058	3.0805	-2.9815	199.335
12059	3.0806	55.7653	171.267
12060	3.0806	47.5714	153.403
12061	3.081	39.3315	230.549
12062	3.0813	49.1659	174.42
12063	3.0817	-3.6817	306.803
12064	3.0817	53.3799	31.3059
12065	3.0819	79.1681	311.077
12066	3.0819	-2.9202	99.2125
12067	3.0822	59.7729	134.857
12068	3.0822	34.8953	196.31
12069	3.0825	60.7107	239.064
12070	3.0825	-3.3023	85.7305
12071	3.0827	58.8403	157.353
12072	3.0828	48.0321	137.345
12073	3.0829	-4.0037	170.47
12074	3.0832	-2.8594	283.339
12075	3.0832	52.4743	280.898
12076	3.0834	72.5933	176.703
12077	3.0834	58.2594	153.864
12078	3.0835	-2.7643	132.681
12079	3.0835	42.185	300.987
12080	3.0835	52.4117	327.174
12081	3.0837	-3.2204	74.8117
12082	3.0837	80.5881	281.901
12083	3.0839	-2.834	280.626
12084	3.084	57.6308	57.5667
12085	3.0844	-2.8873	210.284

12086	3.0844	-2.9551	109.417
12087	3.0845	-3.0571	6.0573
12088	3.0846	53.0312	127.852
12089	3.0846	60.4182	217.217
12090	3.0847	-2.9303	204.635
12091	3.0849	59.5526	75.0089
12092	3.0852	43.7598	341.991
12093	3.0852	-2.8511	214.108
12094	3.0852	-3.0816	243.823
12095	3.0852	56.6158	252.567
12096	3.0852	23.1402	112.111
12097	3.0853	-4.1354	146.132
12098	3.0854	56.8322	147.467
12099	3.0854	49.9839	7.6214
12100	3.0856	43.7891	128.057
12101	3.0856	52.5665	241.606
12102	3.0859	-3.0419	339.307
12103	3.086	50.6908	77.8098
12104	3.086	53.0607	331.289
12105	3.0862	62.1193	20.8751
12106	3.0862	73.1229	27.1651
12107	3.0863	-2.8725	43.4198
12108	3.0863	47.8191	150.956
12109	3.0865	61.0221	206.091
12110	3.0866	55.7744	359.262
12111	3.0867	59.8692	194.613
12112	3.0868	-4.0402	84.0289
12113	3.0869	-3.0723	8.3739
12114	3.087	63.6283	35.7686
12115	3.0871	46.4034	184.557
12116	3.0871	-2.8517	88.3947
12117	3.0872	70.9429	246.417
12118	3.0872	50.8075	111.688
12119	3.0872	46.2019	306.651
12120	3.0874	-2.9261	101.155
12121	3.0875	-2.9355	348.043
12122	3.0875	50.701	157.321
12123	3.0875	65.9848	136.437
12124	3.0876	67.6998	291.741
12125	3.0877	59.6033	18.061
12126	3.0879	57.2895	280.353
12127	3.0879	50.1222	111.493
12128	3.0879	72.3839	208.833
12129	3.0879	44.9523	146.676
12130	3.0879	49.4593	56.0984
12131	3.0883	59.7065	240.991
12132	3.0883	-2.8632	208.041
12133	3.0883	-2.6781	272.277

12134	3.0884	-5.5781	63.2181
12135	3.0886	49.9582	140.496
12136	3.0889	-2.8075	183.344
12137	3.0892	43.6126	210.107
12138	3.0893	61.0103	319.897
12139	3.0894	-3.0578	210.78
12140	3.0895	47.7999	280.481
12141	3.0896	-3.755	203.427
12142	3.0898	55.9245	233.375
12143	3.0898	-2.7903	150.448
12144	3.0898	64.9534	142.021
12145	3.0898	-3.6203	333.072
12146	3.0899	53.0286	95.7483
12147	3.09	61.3011	295.687
12148	3.0903	-2.95	335.084
12149	3.0905	54.986	211.723
12150	3.0907	-2.7507	119.272
12151	3.0908	41.651	259.147
12152	3.0909	-3.9133	358.007
12153	3.091	64.0327	51.3216
12154	3.0912	64.336	49.7487
12155	3.0912	37.7728	260.405
12156	3.0914	51.7033	53.6878
12157	3.0914	61.3054	282.077
12158	3.0915	-3.5796	305.981
12159	3.0916	-2.9358	326.87
12160	3.0918	60.7321	281.105
12161	3.0918	47.1209	219.437
12162	3.0919	-2.7532	195.214
12163	3.0919	71.6557	242.715
12164	3.092	-3.595	168.894
12165	3.092	41.0366	90.0718
12166	3.092	52.5871	300.554
12167	3.092	-3.3625	164.316
12168	3.0922	59.3642	42.7212
12169	3.0923	60.4536	72.5705
12170	3.0923	35.3294	253.973
12171	3.0923	-2.8475	344.387
12172	3.0923	69.2465	26.5063
12173	3.0924	70.6161	12.9777
12174	3.0926	43.7734	135.626
12175	3.0927	62.5209	344.803
12176	3.0931	67.8107	64.37
12177	3.0932	-4.9852	38.3295
12178	3.0932	42.3223	154.459
12179	3.0936	-3.1926	244.688
12180	3.0938	84.9058	288.272
12181	3.0941	50.0321	163.546

12182	3.0942	29.0126	183.996
12183	3.0943	64.2495	39.9594
12184	3.0943	58.6753	71.795
12185	3.0944	-3.5177	124.907
12186	3.0944	75.2192	277.893
12187	3.0945	45.901	321.184
12188	3.0949	-2.7791	312.243
12189	3.0949	-2.995	10.9238
12190	3.0949	-2.915	77.7988
12191	3.095	49.4317	281.14
12192	3.0951	49.8143	211.567
12193	3.0951	56.4081	284.108
12194	3.0954	-2.776	97.7532
12195	3.0956	-3.283	0.72
12196	3.0956	-3.8972	124.55
12197	3.0956	35.3275	74.8597
12198	3.0956	54.284	21.3511
12199	3.0957	67.4353	278.222
12200	3.0957	-2.8765	251.866
12201	3.0959	-3.0257	164.278
12202	3.096	64.1322	51.2753
12203	3.0961	-2.8119	40.0723
12204	3.0962	-3.5741	23.5113
12205	3.0964	52.0781	132.317
12206	3.0965	36.2628	281.146
12207	3.0966	50.6644	166.333
12208	3.0968	-4.4787	117.689
12209	3.0968	53.1643	98.1153
12210	3.0969	-4.5844	212.403
12211	3.0971	84.0336	33.1327
12212	3.0972	57.8173	262.912
12213	3.0973	60.8743	7.5791
12214	3.0974	-2.7952	334.772
12215	3.0974	-2.9672	84.0758
12216	3.0977	42.5765	177.236
12217	3.098	-2.7953	118.856
12218	3.0981	63.641	17.1093
12219	3.0981	55.6471	151.324
12220	3.0982	87.8451	287.259
12221	3.0984	69.7205	85.6019
12222	3.0984	58.2279	243.547
12223	3.0984	58.7281	196.939
12224	3.0984	83.8697	96.1424
12225	3.0985	-3.3795	158.316
12226	3.0987	52.0594	116.616
12227	3.0987	64.6574	265.668
12228	3.0988	70.4157	35.6374
12229	3.0988	-2.9122	304.988

12230	3.0988	55.0206	201.86
12231	3.0988	58.3308	52.8888
12232	3.0988	-2.8529	18.8205
12233	3.0989	54.5778	139.995
12234	3.099	75.6371	210.242
12235	3.099	-2.761	146.688
12236	3.0991	-2.8971	74.0969
12237	3.0991	-2.8472	16.1367
12238	3.0992	49.9069	60.1308
12239	3.0992	58.3749	302.627
12240	3.0994	62.8154	203.764
12241	3.0994	42.1945	210.93
12242	3.0994	27.6119	64.2111
12243	3.0995	75.2842	237.786
12244	3.0996	44.4752	17.3248
12245	3.0996	57.1847	21.057
12246	3.0996	46.8039	77.5782
12247	3.0998	2.6359	230.844
12248	3.0998	80.3176	286.597
12249	3.0999	46.3606	319.443
12250	3.0999	-3.2489	40.5228
12251	3.1	48.1581	134.455
12252	3.1002	42.4594	263.251
12253	3.1004	63.384	82.5207
12254	3.1005	-2.9098	248.153
12255	3.1007	-2.7264	200.298
12256	3.1007	53.4062	147.71
12257	3.1009	-3.4785	30.3576
12258	3.101	-3.5621	93.4503
12259	3.101	67.3926	211.777
12260	3.1011	57.8304	178.587
12261	3.1011	-2.9941	345.747
12262	3.1012	-2.7996	144.252
12263	3.1013	60.053	69.1095
12264	3.1014	-2.8577	210.63
12265	3.1014	-2.8833	170.253
12266	3.1015	-3.2074	168.8
12267	3.102	48.5748	170.315
12268	3.1024	66.0636	72.9741
12269	3.1024	-4.0055	314.885
12270	3.1026	55.4692	236.851
12271	3.1026	49.8612	236.555
12272	3.1027	50.804	206.845
12273	3.1029	56.3791	178.41
12274	3.1029	54.2181	246.173
12275	3.1029	-2.8764	278.383
12276	3.1032	-3.2643	131.943
12277	3.1033	-2.8664	131.473

12278	3.1035	48.2554	264.844
12279	3.1035	-3.7869	269.764
12280	3.1037	-3.0485	159.432
12281	3.1039	60.5699	24.1737
12282	3.104	-4.0975	136.341
12283	3.1041	65.8207	81.8231
12284	3.1041	-4.5968	246.64
12285	3.1044	50.3319	197.824
12286	3.1045	62.7896	258.513
12287	3.1046	-2.8463	225.46
12288	3.1047	64.9308	202.847
12289	3.1049	64.464	126.479
12290	3.105	47.8499	170.105
12291	3.1051	45.2917	155.366
12292	3.1051	58.7986	90.7502
12293	3.1053	59.3673	63.7678
12294	3.1054	47.6528	199.015
12295	3.1055	82.107	55.9114
12296	3.1055	47.7639	170.809
12297	3.1055	55.7209	318.524
12298	3.1056	79.7816	310.439
12299	3.1056	19.9075	147.841
12300	3.1057	59.5816	288.574
12301	3.106	-3.2864	236.139
12302	3.106	46.7985	126.411
12303	3.1061	59.5945	60.8746
12304	3.1061	58.9041	80.712
12305	3.1061	41.0162	223.932
12306	3.1061	-2.6993	288.513
12307	3.1061	-4.0447	115.39
12308	3.1062	78.6473	202.765
12309	3.1063	-2.9368	196.905
12310	3.1065	-2.8886	33.6634
12311	3.1066	54.7359	192.48
12312	3.1068	77.1679	5.5995
12313	3.1068	66.7351	117.266
12314	3.1068	-2.8192	78.149
12315	3.1069	71.9044	47.3623
12316	3.107	74.4276	212.394
12317	3.107	76.4803	201.075
12318	3.107	-3.9026	171.082
12319	3.1072	56.3668	267.636
12320	3.1073	82.7651	355.378
12321	3.1074	64.222	38.8873
12322	3.1075	-2.9146	81.3321
12323	3.1076	57.9012	189.47
12324	3.1076	-2.8506	98.9452
12325	3.1076	60.1638	210.092

12326	3.1077	50.7931	156.788
12327	3.1077	57.842	44.8988
12328	3.1077	-2.7741	63.7109
12329	3.1077	68.2589	26.8442
12330	3.1078	62.8255	260.229
12331	3.1078	27.7081	118.403
12332	3.1083	43.7744	313.33
12333	3.1084	40.2575	131.378
12334	3.1085	60.6813	216.4
12335	3.1087	-2.9258	272.108
12336	3.1089	64.9075	199.727
12337	3.1089	-3.5817	128.343
12338	3.1092	-2.89	226.665
12339	3.1092	50.0292	174.872
12340	3.1093	-3.0351	102.33
12341	3.1093	54.0861	160.668
12342	3.1098	78.1348	257.033
12343	3.11	43.9097	135.864
12344	3.11	63.2447	37.3273
12345	3.1101	-2.9334	333.928
12346	3.1103	-2.6862	255.374
12347	3.1103	47.398	135.796
12348	3.1103	-2.8351	42.9957
12349	3.1104	64.5246	47.4065
12350	3.1104	50.34	40.1638
12351	3.1105	41.2941	172.885
12352	3.1108	48.2441	197.968
12353	3.111	-2.9556	322.839
12354	3.1112	-2.7184	93.2941
12355	3.1114	-4.2556	155.461
12356	3.1114	55.0653	257.108
12357	3.1116	-3.7336	180.94
12358	3.1116	-2.9399	310.209
12359	3.1117	64.9402	20.8159
12360	3.1117	-2.8286	222.342
12361	3.1117	-3.4065	41.9519
12362	3.1117	-2.9886	74.4052
12363	3.1119	66.1731	105.109
12364	3.1119	-2.8168	76.7011
12365	3.112	-3.3484	317.128
12366	3.1121	41.9942	107.674
12367	3.1122	49.1089	215.544
12368	3.1124	-2.8928	343.002
12369	3.1125	-2.7852	272.646
12370	3.1126	39.7601	314.581
12371	3.1126	59.1269	202.237
12372	3.1126	18.1742	104.261
12373	3.1126	51.1908	198.07

12374	3.1126	-3.1859	23.9908
12375	3.1127	-2.8607	28.1254
12376	3.1128	59.7936	16.3516
12377	3.1129	-3.8238	354.97
12378	3.113	-2.8646	88.9327
12379	3.113	50.3371	122.396
12380	3.113	56.4143	18.364
12381	3.1133	69.3133	344.522
12382	3.1135	23.6255	200.933
12383	3.1135	37.1897	266.039
12384	3.1137	61.0955	144.279
12385	3.1137	58.1655	88.8172
12386	3.1138	48.3836	104.988
12387	3.1138	65.8447	81.1317
12388	3.1139	59.2104	274.32
12389	3.114	49.927	316.713
12390	3.1141	57.5215	60.6469
12391	3.1141	-4.1443	116.658
12392	3.1141	63.6854	18.64
12393	3.1141	-3.9569	348.636
12394	3.1144	52.9668	260.773
12395	3.1146	-4.2616	196.261
12396	3.1148	55.3458	118.041
12397	3.1151	59.2065	213.514
12398	3.1151	47.6535	6.4771
12399	3.1153	69.3758	3.1063
12400	3.1153	-3.1801	146.093
12401	3.1154	49.962	218.382
12402	3.1155	61.7353	152.794
12403	3.1158	62.0107	26.8203
12404	3.1159	-4.1674	334.34
12405	3.1161	71.2157	348.78
12406	3.1162	54.3433	325.722
12407	3.1163	48.7244	131.556
12408	3.1166	58.5653	118.953
12409	3.1168	55.3391	10.4773
12410	3.1172	-4.1472	219.619
12411	3.1172	53.4009	10.749
12412	3.1172	50.8714	72.4343
12413	3.1172	-2.9139	13.2431
12414	3.1173	-3.6561	29.1854
12415	3.1175	61.8849	20.5237
12416	3.1175	-3.0679	236.99
12417	3.1176	-3.5262	62.1955
12418	3.1178	-4.0436	45.6857
12419	3.1179	-2.8998	208.699
12420	3.118	67.0527	109.82
12421	3.1182	62.455	337.073

12422	3.1183	65.4274	160.518
12423	3.1184	49.527	265.601
12424	3.1184	51.7624	152.415
12425	3.1187	74.0826	198.378
12426	3.1187	-3.9432	146.365
12427	3.1188	-2.8366	166.386
12428	3.1189	68.5876	216.575
12429	3.1189	-2.7746	83.2808
12430	3.119	83.5536	58.4762
12431	3.1191	58.0108	64.3793
12432	3.1192	48.9276	151.563
12433	3.1194	27.6624	189.12
12434	3.1195	61.7341	138.718
12435	3.1195	59.792	215.392
12436	3.1197	95.6952	309.811
12437	3.1198	61.3326	70.9031
12438	3.1198	54.6452	167.577
12439	3.1198	-3.9152	130.298
12440	3.1198	49.7921	218.342
12441	3.12	58.376	114.287
12442	3.1201	-3.1487	134.168
12443	3.1201	-2.871	335
12444	3.1203	-2.8719	245.625
12445	3.1205	-3.4989	325.748
12446	3.1205	-1.5687	117.337
12447	3.1207	34.2654	255.333
12448	3.1208	77.9943	118.49
12449	3.1212	-2.9328	62.7369
12450	3.1212	68.4563	51.0699
12451	3.1213	48.274	143.709
12452	3.1214	37.9083	77.4231
12453	3.1219	51.1435	188.471
12454	3.1219	37.4899	198.45
12455	3.122	51.4001	152.329
12456	3.122	68.5867	240.017
12457	3.1221	76.3612	295.826
12458	3.1222	68.9761	53.4776
12459	3.1223	63.4253	318.565
12460	3.1223	62.4111	64.1089
12461	3.1223	-0.2136	9.7451
12462	3.1223	-3.3057	33.8848
12463	3.1223	70.8158	211.475
12464	3.1225	68.75	229.901
12465	3.1226	40.9416	282.332
12466	3.1228	58.1091	122.562
12467	3.123	-3.1328	292.516
12468	3.123	60.8649	133.446
12469	3.123	60.789	160.495

12470	3.1232	-2.9193	170.3
12471	3.1232	58.9825	85.5804
12472	3.1234	58.9318	356.884
12473	3.1235	44.9115	102.377
12474	3.1236	-2.8729	29.8436
12475	3.1236	50.7354	237.161
12476	3.1238	69.7727	226.449
12477	3.1238	59.0545	154.357
12478	3.1239	70.5465	105.861
12479	3.124	72.5889	252.528
12480	3.1241	74.1493	75.8045
12481	3.1243	-2.7497	8.4755
12482	3.1244	49.3766	112.699
12483	3.1245	64.2263	84.6378
12484	3.1246	-4.0643	10.88
12485	3.1246	-2.8192	156.542
12486	3.1246	49.8583	74.4681
12487	3.1246	60.7851	96.4023
12488	3.1248	44.1523	131.301
12489	3.1249	52.8657	94.4924
12490	3.1249	62.9231	88.88
12491	3.125	-3.0365	190.187
12492	3.1251	72.7706	254.472
12493	3.1252	43.9563	211.904
12494	3.1252	-2.8386	330.642
12495	3.1256	-2.8969	227.341
12496	3.1258	46.5146	295.394
12497	3.126	54.1144	200.798
12498	3.126	44.2983	172.84
12499	3.1261	59.788	47.1015
12500	3.1262	59.6136	295.031
12501	3.1263	-3.2659	262.603
12502	3.1263	56.2878	234.653
12503	3.1265	54.2174	177.724
12504	3.1266	57.6331	127.658
12505	3.1267	-2.7915	172.296
12506	3.1268	67.0473	262.079
12507	3.1268	-2.7484	129.025
12508	3.127	-4.152	286.121
12509	3.1273	52.0916	191.618
12510	3.1274	58.5454	7.8729
12511	3.1276	70.6396	164.305
12512	3.1277	56.4548	194.673
12513	3.1279	54.8992	214.407
12514	3.128	51.9226	199.776
12515	3.1282	73.4059	240.183
12516	3.1282	-3.3961	74.3056
12517	3.1285	77.911	61.9756

12518	3.1285	41.111	335.169
12519	3.1286	57.5099	166.176
12520	3.1286	70.0369	45.6883
12521	3.1288	-2.9303	125.226
12522	3.1288	-2.744	320.946
12523	3.1288	51.3314	173.473
12524	3.1289	61.1395	72.1804
12525	3.1289	-3.3137	276.369
12526	3.129	-3.4179	53.7018
12527	3.129	62.5833	295.809
12528	3.1291	75.5038	198.997
12529	3.1293	-2.7619	270.311
12530	3.1295	52.2583	18.616
12531	3.1296	65.0495	160.266
12532	3.1298	73.3925	211.517
12533	3.13	27.1447	180.39
12534	3.1301	-2.8636	206.118
12535	3.1301	-2.9143	331.981
12536	3.1302	-3.9447	262.087
12537	3.1303	54.5813	121.692
12538	3.1303	52.3947	180.114
12539	3.1303	-3.636	180.714
12540	3.1303	-2.9011	263.547
12541	3.1305	60.9477	96.4509
12542	3.1307	60.1129	344.606
12543	3.1307	43.1293	157.184
12544	3.1308	107.588	344.536
12545	3.1309	69.2923	276.334
12546	3.1309	-2.7767	302.598
12547	3.131	58.4515	302.134
12548	3.1311	58.6734	156.525
12549	3.1312	47.7563	271.166
12550	3.1314	65.5787	270.852
12551	3.1315	-3.8432	119.868
12552	3.1315	19.7408	28.8765
12553	3.1317	61.4681	217.554
12554	3.1317	43.801	224.743
12555	3.1318	70.4707	286.46
12556	3.1319	50.4334	318.398
12557	3.132	54.2952	231.077
12558	3.1322	53.2193	4.5483
12559	3.1322	54.0867	109.71
12560	3.1324	54.0934	119.692
12561	3.1326	-2.7378	15.6829
12562	3.1326	65.5984	112.63
12563	3.133	41.4719	99.4541
12564	3.1331	70.9833	216.106
12565	3.1333	62.1075	91.8907

12566	3.1334	56.9087	221.041
12567	3.1335	60.936	347.455
12568	3.1335	54.4093	89.9302
12569	3.1336	69.8877	305.638
12570	3.1338	-2.6134	235.014
12571	3.1339	-3.141	144.602
12572	3.134	56.6202	95.1211
12573	3.134	-2.7952	201.717
12574	3.134	31.3348	162.244
12575	3.1343	-2.7883	80.9548
12576	3.1343	66.684	31.7353
12577	3.1344	-2.7706	116.978
12578	3.1346	65.6816	74.2174
12579	3.1347	67.4525	252.037
12580	3.1347	66.9215	88.2173
12581	3.1348	57.6183	109.271
12582	3.1349	57.7895	159.781
12583	3.135	57.5663	126.454
12584	3.135	55.3479	36.7362
12585	3.1351	50.0467	172.607
12586	3.1351	61.0073	186.917
12587	3.1356	60.3168	11.5648
12588	3.1356	60.4781	42.3587
12589	3.1358	-2.7434	11.086
12590	3.1359	52.5322	133.409
12591	3.1362	63.1334	61.1139
12592	3.1362	-3.6428	198.064
12593	3.1363	68.5811	26.0949
12594	3.1363	-4.0509	215.309
12595	3.1364	-3.598	114.2
12596	3.1366	45.7348	201.872
12597	3.1367	-3.5051	322.574
12598	3.1367	-2.7006	38.1505
12599	3.1367	60.2694	161.631
12600	3.1368	53.3015	149.544
12601	3.1369	66.5919	258.17
12602	3.137	61.761	73.8221
12603	3.1371	51.5198	19.5115
12604	3.1372	-3.58	213.827
12605	3.1375	55.7542	343.947
12606	3.1376	-2.8695	55.9699
12607	3.1379	-3.8837	102.859
12608	3.1379	-2.6704	122.071
12609	3.138	61.7872	143.668
12610	3.138	48.3185	357.332
12611	3.138	58.5159	28.7237
12612	3.138	-3.1471	249.039
12613	3.1382	54.2124	158.135

12614	3.1383	36.8569	334.331
12615	3.1384	71.0541	204.3
12616	3.1385	-3.9867	115.761
12617	3.1385	52.2917	140.754
12618	3.1386	44.0625	153.378
12619	3.1386	56.8696	186.418
12620	3.1387	-2.8475	12.8301
12621	3.1388	57.1755	195.218
12622	3.1399	-3.4625	63.6739
12623	3.1401	79.633	13.9163
12624	3.1401	46.6088	163.454
12625	3.1402	44.9946	249.228
12626	3.1404	81.087	66.0533
12627	3.1404	39	170.75
12628	3.1404	64.8306	308.242
12629	3.1406	-2.7478	312.729
12630	3.1406	58.87	111.841
12631	3.1408	-3.2631	126.245
12632	3.1413	49.2894	151.568
12633	3.1413	-3.0994	51.7737
12634	3.1414	71.5362	34.3083
12635	3.1415	58.0826	219.542
12636	3.1417	-2.9634	25.6393
12637	3.1418	52.2714	71.8926
12638	3.1419	69.1619	111.343
12639	3.142	-3.4045	88.988
12640	3.142	63.4184	336.518
12641	3.1421	-5.758	216.375
12642	3.1423	45.8398	141.753
12643	3.1424	-3.2931	186.486
12644	3.1424	63.9715	108.54
12645	3.1425	45.627	157.188
12646	3.1425	55.227	0.5112
12647	3.1425	65.218	20.7355
12648	3.1426	71.2331	336.722
12649	3.1427	67.0009	77.3068
12650	3.1428	-2.6309	275.397
12651	3.143	51.2394	212.449
12652	3.143	67.0777	48.637
12653	3.143	50.8229	320.548
12654	3.1433	60.5511	208.318
12655	3.1437	76.4806	211.113
12656	3.1437	-5.5783	58.3155
12657	3.1437	63.5042	5.399
12658	3.1438	67.7353	155.106
12659	3.1439	55.431	112.95
12660	3.144	-2.9574	5.4934
12661	3.1441	31.1663	344.087

12662	3.1441	-4.2	134.412
12663	3.1441	83.6563	68.17
12664	3.1443	66.209	208.281
12665	3.1443	-2.9432	336.839
12666	3.1443	-3.1502	284.064
12667	3.1447	47.7527	166.643
12668	3.1447	55.2858	303.284
12669	3.1448	46.0843	31.196
12670	3.1453	47.5157	256.124
12671	3.1453	56.7131	315.817
12672	3.1455	51.2374	140.927
12673	3.1455	48.7541	115.119
12674	3.1456	64.107	133.806
12675	3.1458	46.9053	356.535
12676	3.1459	61.2503	232.796
12677	3.146	42.1674	117.029
12678	3.1461	69.194	14.5332
12679	3.1461	53.042	176.577
12680	3.1461	60.3214	257.112
12681	3.1462	53.6429	70.8362
12682	3.1463	53.2355	135.291
12683	3.1463	52.762	98.8154
12684	3.1464	54.6651	334.671
12685	3.1464	-4.0679	206.698
12686	3.1465	-3.9478	156.583
12687	3.1465	83.783	346.419
12688	3.1465	-4.1081	173.162
12689	3.1466	71.5914	59.8306
12690	3.1466	64.2009	45.4076
12691	3.1467	-2.8855	143.356
12692	3.1469	56.9412	64.6683
12693	3.1469	-2.9897	266.869
12694	3.1471	-2.8839	0.2554
12695	3.1472	58.3228	96.076
12696	3.1472	73.1241	149.578
12697	3.1473	47.4531	89.5946
12698	3.1473	-2.804	340.801
12699	3.1474	-2.9261	233.813
12700	3.1474	66.5589	133.238
12701	3.1476	58.5201	99.2322
12702	3.1477	-3.5517	166.775
12703	3.1478	45.2708	200.969
12704	3.1478	-3.2827	319.004
12705	3.148	-3.8324	47.2811
12706	3.1481	60.299	166.113
12707	3.1481	-4.0002	223.227
12708	3.1481	53.1046	37.5552
12709	3.1482	50.3767	240.708

12710	3.1483	60.8309	136.805
12711	3.1484	-4.3822	192.191
12712	3.1484	71.129	80.5818
12713	3.1484	56.8005	231.1
12714	3.1486	65.9008	283.139
12715	3.1488	58.2895	12.8405
12716	3.1491	61.1422	65.5181
12717	3.1493	65.308	85.3687
12718	3.1493	63.5687	141.89
12719	3.1496	44.5966	179.585
12720	3.1497	61.3	273.278
12721	3.1497	52.2042	274.385
12722	3.15	-2.7451	14.158
12723	3.1502	62.5596	114.94
12724	3.1503	-4.3257	18.2364
12725	3.1504	58.0427	273.33
12726	3.1505	57.8806	67.9124
12727	3.1507	-3.2416	255.681
12728	3.1508	-2.7214	104.175
12729	3.1508	52.6776	12.4143
12730	3.1509	-2.9364	104.901
12731	3.151	52.1299	297.408
12732	3.151	-3.0946	89.2317
12733	3.151	-3.4715	118.339
12734	3.1511	68.2265	86.0057
12735	3.1512	-3.9648	323.715
12736	3.1512	-2.6955	109.927
12737	3.1512	-3.5436	120.521
12738	3.1515	58.8769	260.242
12739	3.1515	69.9307	212.721
12740	3.1515	58.69	195.791
12741	3.1515	62.1982	282.322
12742	3.1517	47.8902	214.702
12743	3.1517	52.6433	177.718
12744	3.1517	52.3359	69.7692
12745	3.1517	61.887	81.003
12746	3.1521	64.3212	50.2699
12747	3.1522	18.7651	199.434
12748	3.1525	57.4812	121.575
12749	3.1528	66.905	296.057
12750	3.1528	64.2207	105.313
12751	3.1528	72.0794	225.524
12752	3.1529	65.723	151.834
12753	3.1531	51.1285	214.301
12754	3.1532	56.4352	70.1777
12755	3.1532	60.9885	244.676
12756	3.1532	-2.7917	229.632
12757	3.1534	-2.9333	27.2135

12758	3.1536	87.5992	315.691
12759	3.1536	14.6055	232.167
12760	3.1539	48.7543	294.97
12761	3.1539	-2.8441	271.231
12762	3.1542	55.2309	106.325
12763	3.1542	60.13	332.98
12764	3.1543	55.1498	219.229
12765	3.1544	65.373	183.643
12766	3.1544	83.7079	207.718
12767	3.1545	72.4528	83.4925
12768	3.1545	-2.9367	196.266
12769	3.1546	-3.2209	237.084
12770	3.1546	50.1871	292.601
12771	3.1547	-3.0911	280.621
12772	3.1548	94.3603	316.967
12773	3.1549	-3.2247	295.663
12774	3.1551	-2.8403	315.236
12775	3.1551	43.5965	274.838
12776	3.1552	-3.1269	93.0428
12777	3.1553	46.2931	158.206
12778	3.1554	50.5569	78.1852
12779	3.1555	-2.812	130.66
12780	3.1555	-3.6155	347.839
12781	3.1557	-3.2049	354.034
12782	3.1557	-3.0381	328.726
12783	3.1558	-2.9254	322.614
12784	3.1558	-2.9579	238.038
12785	3.1559	-2.8646	242.057
12786	3.1561	50.2782	62.9946
12787	3.1562	66.2973	279.333
12788	3.1562	76.1599	211.961
12789	3.1563	64.1655	137.875
12790	3.1564	63.5589	99.1617
12791	3.1564	45.4645	65.8099
12792	3.1564	-4.0185	149.649
12793	3.1565	50.134	117.695
12794	3.1566	-2.9094	241.653
12795	3.1567	66.9296	112.728
12796	3.1567	48.0871	100.175
12797	3.1567	55.9439	204.06
12798	3.1568	-4.219	330.626
12799	3.1568	68.3731	115.757
12800	3.1568	59.271	269.317
12801	3.1571	66.2293	159.39
12802	3.1576	69.1024	254.863
12803	3.1576	49.6453	137.368
12804	3.1576	-3.8198	171.226
12805	3.1577	57.8482	229.661

12806	3.158	54.2023	151.025
12807	3.1582	51.04	110.907
12808	3.1584	-3.1258	177.721
12809	3.159	-3.4048	283.141
12810	3.1591	69.5702	163.543
12811	3.1591	50.2217	194.646
12812	3.1592	-2.993	1.4114
12813	3.1593	-4.464	39.2267
12814	3.1593	77.7251	309.913
12815	3.1594	-3.6535	19.0021
12816	3.1595	46.9728	267.5
12817	3.1595	-2.8619	96.56
12818	3.1596	30.1485	316.655
12819	3.1596	72.3365	211.14
12820	3.1598	54.694	108.86
12821	3.1599	63.5809	35.0578
12822	3.1599	54.7827	152.563
12823	3.1599	50.2826	34.6675
12824	3.1601	70.3404	32.1653
12825	3.1601	53.801	143.792
12826	3.1602	-2.8627	143.106
12827	3.1603	81.7602	315.513
12828	3.1603	63.465	113.885
12829	3.1605	67.1561	236.432
12830	3.1606	56.2866	168.03
12831	3.1607	68.0831	38.2087
12832	3.1608	-2.9995	165.697
12833	3.1608	52.032	121.545
12834	3.1608	-3.0883	306.573
12835	3.1609	63.4079	19.1905
12836	3.1609	44.5306	149.993
12837	3.1609	56.8679	132.407
12838	3.1609	-3.1779	22.0223
12839	3.161	59.4787	126.945
12840	3.1614	-3.2352	265.319
12841	3.1616	58.966	166.003
12842	3.1616	67.5121	11.5819
12843	3.1617	50.9341	130.763
12844	3.1618	77.3721	59.4673
12845	3.1619	-4.3044	221.304
12846	3.1621	53.6082	232.68
12847	3.1622	-2.8415	116.591
12848	3.1623	-3.3373	357.214
12849	3.1623	76.4753	253.911
12850	3.1623	79.398	115.836
12851	3.1625	-2.8594	163.848
12852	3.1626	72.4046	216.468
12853	3.1626	-3.3883	243.423

12854	3.1626	47.0933	335.045
12855	3.1627	-2.9901	72.0505
12856	3.1627	-4.0412	144.458
12857	3.1628	52.6153	202.705
12858	3.1629	41.2386	34.3009
12859	3.163	-3.1022	34.194
12860	3.1632	50.7227	167.968
12861	3.1634	65.5574	293.599
12862	3.1635	18.5833	97.7743
12863	3.1636	-2.955	87.9836
12864	3.1637	75.5817	359.025
12865	3.164	67.0314	111.491
12866	3.164	35.5355	132.898
12867	3.1642	66.2947	48.2842
12868	3.1642	55.3967	96.1263
12869	3.1642	-3.7751	5.5177
12870	3.1643	64.0013	206.186
12871	3.1644	79.7546	53.8439
12872	3.1644	51.8927	114.004
12873	3.1646	72.1742	262.387
12874	3.1647	65.13	219.142
12875	3.1647	70.3278	288
12876	3.1648	49.239	263.838
12877	3.1649	-2.9669	257.106
12878	3.165	59.1476	135.545
12879	3.1652	53.5967	192.288
12880	3.1652	62.3217	285.272
12881	3.1653	66.2648	277.637
12882	3.1656	68.5669	6.7301
12883	3.1656	61.6163	10.9847
12884	3.1656	-3.0967	315.07
12885	3.1657	37.6074	322.366
12886	3.1659	62.8897	248.718
12887	3.166	-2.8098	5.8807
12888	3.1661	68.7855	123.859
12889	3.1662	52.1913	22.9461
12890	3.1662	-2.7514	126.829
12891	3.1663	60.1157	139.931
12892	3.1664	-2.8242	109.878
12893	3.1665	-2.7735	120.255
12894	3.1665	62.1504	151.412
12895	3.1666	-2.9422	256.122
12896	3.1669	64.7911	348.92
12897	3.1669	56.5624	106.406
12898	3.1669	54.0981	93.5611
12899	3.1669	61.5586	92.7513
12900	3.1669	-2.9788	327.515
12901	3.1673	-3.92	173.564

12902	3.1673	63.855	22.7377
12903	3.1673	62.1046	236.322
12904	3.1674	71.3778	359.844
12905	3.1674	67.7744	177.609
12906	3.1675	52.6936	122.509
12907	3.1675	54.4806	134.745
12908	3.1679	66.0767	243.23
12909	3.1679	45.9928	184.219
12910	3.1679	72.8928	236.715
12911	3.168	62.5628	209.467
12912	3.1681	-2.9785	126.724
12913	3.1681	70.8546	203.226
12914	3.1681	-3.3998	310.621
12915	3.1682	-2.8763	10.671
12916	3.1683	-2.937	303.48
12917	3.1684	60.0111	94.2949
12918	3.1686	-2.9162	123.648
12919	3.1686	48.6052	288.347
12920	3.1688	61.9858	146.376
12921	3.1689	70.53	254.401
12922	3.1689	53.1545	116.954
12923	3.169	68.0966	107.066
12924	3.1691	53.1279	274.075
12925	3.1693	66.9814	89.1086
12926	3.1693	66.1099	273.593
12927	3.1694	66.076	209.886
12928	3.1695	65.3052	129.097
12929	3.1696	59.4182	348.534
12930	3.1696	-2.8057	78.4578
12931	3.1697	-2.858	302.425
12932	3.1698	50.7685	249.05
12933	3.17	-4.3144	105.635
12934	3.1702	-3.7532	344.822
12935	3.1702	-3.8199	171.926
12936	3.1702	56.0571	91.7681
12937	3.1703	-2.9701	324.902
12938	3.1703	49.3188	156.097
12939	3.1704	-2.9199	150.287
12940	3.1705	-2.8707	337.672
12941	3.1706	-3.4003	325.693
12942	3.1707	69.7993	234.391
12943	3.1709	62.4474	151.901
12944	3.1709	48.9406	170.429
12945	3.1711	-2.8198	191.132
12946	3.1711	63.7676	301.118
12947	3.1712	51.923	36.7758
12948	3.1713	60.5068	98.5271
12949	3.1713	72.0682	56.9884

12950	3.1713	-2.7151	326.07
12951	3.1718	56.5846	210.108
12952	3.172	52.7276	86.1765
12953	3.172	-2.9179	329.229
12954	3.1721	55.0861	51.9992
12955	3.1723	66.0581	160.268
12956	3.1724	58.3931	244.979
12957	3.1725	63.8917	40.5101
12958	3.1725	56.409	120.761
12959	3.1726	-2.9104	70.546
12960	3.1727	-2.9825	103.721
12961	3.173	56.5241	110.717
12962	3.1731	71.2475	205.687
12963	3.1732	57.0419	124.324
12964	3.1732	56.1766	309.718
12965	3.1733	45.9095	273.874
12966	3.1734	63.2726	304.071
12967	3.1734	58.1176	71.4226
12968	3.1734	-2.9483	198.555
12969	3.1737	90.3414	84.2017
12970	3.1741	52.3615	101.921
12971	3.1742	-3.325	261.017
12972	3.1743	-2.9215	104.669
12973	3.1746	50.4881	143.037
12974	3.1747	-2.7276	14.1081
12975	3.1747	-3.0443	132.645
12976	3.1747	-2.9384	257.75
12977	3.1748	73.8904	238.161
12978	3.1748	47.3034	74.8495
12979	3.1748	-3.6864	274.724
12980	3.1749	-2.8583	137.415
12981	3.175	63.3133	161.066
12982	3.1751	-2.9768	136.102
12983	3.1752	74.0509	29.8738
12984	3.1753	-3.0971	314.125
12985	3.1754	60.5023	60.669
12986	3.1756	59.8027	129.574
12987	3.1756	-3.354	97.1941
12988	3.1756	-2.9221	252.434
12989	3.1758	90.1851	269.923
12990	3.1759	60.9274	163.79
12991	3.1761	61.2108	185.045
12992	3.1762	60.1681	318.671
12993	3.1762	71.9014	315.335
12994	3.1762	56.2637	259.61
12995	3.1764	59.1436	164.476
12996	3.1765	67.0475	112.66
12997	3.1769	-3.0061	57.7538

12998	3.177	-3.7835	269.511
12999	3.1773	-2.911	330.331
13000	3.1774	76.4709	22.6276
13001	3.1774	-4.1839	167.774
13002	3.1775	49.0148	132.284
13003	3.1775	59.2835	18.9079
13004	3.1777	-3.4899	29.652
13005	3.1778	-2.6768	124.605
13006	3.1779	81.2878	36.5548
13007	3.1779	-2.8523	6.4392
13008	3.178	-3.0006	275.167
13009	3.1787	62.8551	282.047
13010	3.1789	54.2341	58.7535
13011	3.1791	64.882	198.823
13012	3.1791	-3.341	105.018
13013	3.1791	-3.6983	132.091
13014	3.1792	55.1009	210.463
13015	3.1792	52.2458	171.012
13016	3.1792	-3.8203	181.461
13017	3.1793	72.9921	15.4175
13018	3.1794	51.2904	131.019
13019	3.1795	45.3269	214.901
13020	3.1797	-2.9846	156.944
13021	3.1797	61.2201	139.553
13022	3.1797	58.4639	315.145
13023	3.1797	53.8094	136.64
13024	3.1797	64.6411	42.2446
13025	3.1798	-4.388	24.3885
13026	3.1798	48.0337	136.911
13027	3.1799	72.456	43.9915
13028	3.18	-3.2564	131.804
13029	3.1801	64.4466	289.242
13030	3.1801	69.4072	237.743
13031	3.1801	-3.2262	303.034
13032	3.1802	62.7905	38.7832
13033	3.1803	-3.8698	205.652
13034	3.1804	-2.945	265.041
13035	3.1805	62.3213	229.304
13036	3.1806	57.6549	191.229
13037	3.1806	86.6176	196.445
13038	3.1807	-2.9308	251.792
13039	3.1808	-2.9277	266.674
13040	3.181	61.684	312.466
13041	3.1811	45.2422	289.231
13042	3.1814	66.263	40.1424
13043	3.1816	50.4174	142.38
13044	3.1817	62.0339	278.965
13045	3.1819	58.1525	310.567

13046	3.182	52.547	91.7898
13047	3.1821	56.7163	154.246
13048	3.1822	56.5196	90.9949
13049	3.1822	52.7496	221.917
13050	3.1825	58.6863	96.016
13051	3.1826	69.0298	155.567
13052	3.1829	59.1234	158.351
13053	3.183	47.7262	322.282
13054	3.183	-2.9068	273.001
13055	3.1832	55.8024	82.0913
13056	3.1834	61.662	120.842
13057	3.1835	73.4753	314.843
13058	3.1836	88.6129	239.636
13059	3.1836	45.4557	145.528
13060	3.1836	-3.0398	275.836
13061	3.1837	-2.9231	137.061
13062	3.1838	53.1902	69.7774
13063	3.184	-2.7801	354.164
13064	3.1841	-2.9028	254.882
13065	3.1841	-2.822	41.5188
13066	3.1842	-2.799	177.939
13067	3.1843	-2.7922	242.52
13068	3.1843	48.3755	125.867
13069	3.1843	-4.0051	6.9389
13070	3.1844	-2.7768	117.051
13071	3.1845	-3.2631	15.9252
13072	3.1847	52.5035	33.5937
13073	3.185	58.3761	83.4055
13074	3.1851	68.7079	238.605
13075	3.1853	-3.2668	301.4
13076	3.1853	-2.9458	255.23
13077	3.1856	65.844	59.9818
13078	3.1856	58.495	89.2694
13079	3.1856	52.1778	254.291
13080	3.1856	63.1994	121.548
13081	3.1858	-4.1482	246.27
13082	3.1859	-3.7865	260.236
13083	3.186	-3.6823	281.669
13084	3.1863	72.1143	63.7024
13085	3.1866	51.0556	285.774
13086	3.1866	56.7369	297.527
13087	3.1871	70.607	268.35
13088	3.1872	-3.065	338.321
13089	3.1873	70.3785	231.772
13090	3.1873	66.2909	203.736
13091	3.1875	72.3042	92.2398
13092	3.1875	47.487	224.277
13093	3.1876	63.2156	42.4628

13094	3.1878	65.6256	149.174
13095	3.1878	-3.929	97.8756
13096	3.1878	62.8511	134.484
13097	3.1879	69.8833	138.143
13098	3.1879	77.5131	257.475
13099	3.188	-2.8761	253.898
13100	3.1881	62.7085	58.4341
13101	3.1882	77.4011	345.525
13102	3.1884	64.7715	274.299
13103	3.1884	-2.7982	260.45
13104	3.1887	70.4615	173.382
13105	3.1887	72.3252	261.208
13106	3.1887	-2.9448	248.446
13107	3.1888	71.4482	206.198
13108	3.189	69.263	284.96
13109	3.1891	58.7835	123.546
13110	3.1893	68.2573	321.661
13111	3.1895	63.9121	68.8012
13112	3.1895	80.184	195.646
13113	3.1896	61.2099	221.83
13114	3.1896	47.7352	258.292
13115	3.1896	81.4708	209.423
13116	3.1897	-2.7898	93.7875
13117	3.1897	61.7729	318.816
13118	3.1898	52.9721	142.55
13119	3.1899	67.0405	73.123
13120	3.1899	-2.7656	151.442
13121	3.19	58.9826	213.583
13122	3.1901	96.5626	354.208
13123	3.1901	67.5316	40.7686
13124	3.1901	71.223	28.6037
13125	3.1901	55.4499	299.435
13126	3.1902	-3.125	94.2499
13127	3.1903	62.8502	8.2173
13128	3.1903	60.2459	145.924
13129	3.1904	50.3172	115.004
13130	3.1906	57.2072	181.924
13131	3.1906	75.4674	79.5137
13132	3.1907	51.5577	299.959
13133	3.1907	59.3743	156.455
13134	3.1907	88.7907	26.591
13135	3.1908	58.5182	285.704
13136	3.1909	-3.1228	60.5122
13137	3.191	63.9834	141.906
13138	3.1911	-2.7947	358.842
13139	3.1913	57.2158	256.866
13140	3.1913	64.2682	215.13
13141	3.1913	-2.8346	14.4315

13142	3.1913	76.3596	213.253
13143	3.1915	57.3559	121.917
13144	3.1916	57.0125	133.699
13145	3.1917	-2.93	73.424
13146	3.1918	-2.9139	266.697
13147	3.1921	82.1923	68.4291
13148	3.1923	-2.9876	260.176
13149	3.1923	79.3387	61.5266
13150	3.1923	74.6337	233.385
13151	3.1925	69.7015	186.352
13152	3.1925	51.3215	285.165
13153	3.1927	66.4663	222.337
13154	3.1927	-2.8029	89.1755
13155	3.1927	54.9117	125.798
13156	3.1927	-2.995	88.1783
13157	3.1929	56.4042	83.1363
13158	3.1929	-2.7758	358.423
13159	3.193	66.3872	177.162
13160	3.193	59.1311	122.342
13161	3.193	44.99	335.118
13162	3.1931	-2.6591	167.843
13163	3.1931	59.7435	130.679
13164	3.1935	54.265	284.36
13165	3.1935	53.7093	76.8541
13166	3.1938	52.7039	239.763
13167	3.1938	54.4201	100.817
13168	3.1939	-3.2692	335.917
13169	3.1939	68.3249	77.1737
13170	3.194	55.7801	187.526
13171	3.1941	-4.1183	336.238
13172	3.1941	69.755	69.6912
13173	3.1942	64.017	125.216
13174	3.1943	48.2339	266.054
13175	3.1943	55.4457	93.796
13176	3.1944	64.0115	239.605
13177	3.1944	59.7463	191.783
13178	3.1945	92.6988	50.4228
13179	3.1945	71.5612	240.308
13180	3.1945	70.479	316.657
13181	3.1947	64.199	159.401
13182	3.1947	-2.4164	316.518
13183	3.1948	51.4358	155.467
13184	3.1948	-3.8599	79.6122
13185	3.1949	66.3544	203.919
13186	3.195	66.6571	95.6032
13187	3.195	64.8814	44.2676
13188	3.195	73.5774	119.26
13189	3.1951	66.9234	41.746

13190	3.1952	57.464	94.0358
13191	3.1953	66.6599	105.814
13192	3.1953	75.8723	51.0215
13193	3.1955	-3.0236	72.1898
13194	3.1956	94.2287	208.153
13195	3.1956	62.7293	322.269
13196	3.1958	67.3843	343.83
13197	3.1958	-4.0419	69.9535
13198	3.1961	77.5149	198.226
13199	3.1962	60.2962	216.734
13200	3.1962	65.9948	291.269
13201	3.1963	67.0851	168.444
13202	3.1963	60.6833	290.777
13203	3.1965	63.4522	44.2148
13204	3.1967	65.9702	267.345
13205	3.1969	65.9119	281.223
13206	3.1969	63.2721	192.172
13207	3.197	-2.8944	261.633
13208	3.1976	68.855	240.601
13209	3.1976	-2.7953	123.542
13210	3.1979	66.8114	157.073
13211	3.1979	76.6846	313.39
13212	3.198	-3.386	260.185
13213	3.198	59.574	261.286
13214	3.1981	86.6522	51.7952
13215	3.1981	58.3851	82.7018
13216	3.1984	62.3791	304.362
13217	3.1986	83.0229	315.266
13218	3.1989	61.5535	182.294
13219	3.199	-3.9093	49.1583
13220	3.1991	-2.7847	331.562
13221	3.1992	68.5825	23.1209
13222	3.1993	68.6826	248.191
13223	3.1995	51.9592	54.024
13224	3.1996	56.3125	34.267
13225	3.1997	24.1257	176.546
13226	3.1998	-2.7389	282.821
13227	3.1998	54.6914	164.808
13228	3.2	-2.8705	113.356
13229	3.2	18.5812	110.461
13230	3.2	48.2323	110.127
13231	3.2002	58.1664	197.479
13232	3.2002	78.4466	25.5981
13233	3.2002	-4.3899	133.345
13234	3.2003	-2.7053	127.089
13235	3.2003	64.3119	273.928
13236	3.2005	97.2731	212.23
13237	3.2005	50.2886	144.893

13238	3.2005	10.2659	88.7275
13239	3.2007	68.9451	70.1568
13240	3.2008	65.7021	119.692
13241	3.2008	-2.8633	183.407
13242	3.2009	70.4122	57.9355
13243	3.2011	56.1511	120.914
13244	3.2011	69.645	10.2684
13245	3.2012	60.49	219.798
13246	3.2012	57.0465	112.261
13247	3.2014	75.6878	19.7535
13248	3.2015	70.1367	138.441
13249	3.2016	53.2899	130.022
13250	3.2017	55.7352	164.817
13251	3.2017	61.9942	204.05
13252	3.2017	63.5815	201.411
13253	3.2018	78.5214	21.8934
13254	3.2018	-2.8701	129.148
13255	3.2018	-3.8688	141.109
13256	3.2018	92.8925	285.386
13257	3.2018	-2.8007	99.5651
13258	3.2019	58.0581	2.6497
13259	3.2019	54.2299	176.098
13260	3.2019	77.6208	358.794
13261	3.2019	66.1565	276.42
13262	3.202	76.6637	259.015
13263	3.202	37.0103	255.89
13264	3.202	44.4233	189.78
13265	3.202	59.7996	114.496
13266	3.2021	53.6986	248.604
13267	3.2021	61.8316	121.915
13268	3.2023	51.5218	32.9764
13269	3.2024	-3.0213	269.317
13270	3.2024	63.4695	212.652
13271	3.2024	53.8146	13.9559
13272	3.2025	-2.8275	136.025
13273	3.2027	56.5637	6.1951
13274	3.2027	71.0615	313.766
13275	3.2029	72.7201	255.777
13276	3.2029	76.1383	48.2372
13277	3.2032	64.4286	195.925
13278	3.2032	63.5711	268.139
13279	3.2033	80.7446	210.459
13280	3.2035	61.1167	136.878
13281	3.2035	75.0311	35.5276
13282	3.2039	89.6341	1.2929
13283	3.2039	55.5684	169.748
13284	3.2039	52.5501	118.786
13285	3.204	-2.7528	219.591

13286	3.204	-2.9046	163.187
13287	3.204	59.5825	248.589
13288	3.2041	61.9493	241.452
13289	3.2041	60.3861	199.459
13290	3.2041	57.3664	167.43
13291	3.2043	66.3204	239.201
13292	3.2043	64.4401	107.922
13293	3.2043	-2.7901	145.579
13294	3.2045	60.0151	165.559
13295	3.2045	56.8059	173.029
13296	3.2045	58.2626	240.278
13297	3.2047	62.3664	229.396
13298	3.2047	49.5329	259.491
13299	3.2048	62.278	287.011
13300	3.2049	58.2356	79.4073
13301	3.205	70.2246	177.704
13302	3.205	-2.7509	174.956
13303	3.2051	71.7138	110.831
13304	3.2053	45.3297	199.179
13305	3.2054	93.4796	317.001
13306	3.2054	66.3094	36.8753
13307	3.2055	62.5666	193.547
13308	3.2057	73.5259	260.837
13309	3.2058	66.6316	246.676
13310	3.2059	84.1317	247.559
13311	3.206	76.4689	82.7247
13312	3.2062	85.7674	247.681
13313	3.2062	65.4895	283.502
13314	3.2063	47.8951	294.759
13315	3.2064	77.08	228.254
13316	3.2064	63.4221	107.895
13317	3.2065	-2.8642	153.975
13318	3.2065	51.7779	32.2834
13319	3.2068	47.6363	155.623
13320	3.2069	68.6142	237.529
13321	3.2071	-3.968	44.314
13322	3.2073	57.222	235.676
13323	3.2074	56.6278	190.186
13324	3.2075	79.8724	48.132
13325	3.2076	60.8463	98.4066
13326	3.2077	55.9482	66.1052
13327	3.2077	-3.8292	332.013
13328	3.2078	67.0223	81.4428
13329	3.2078	61.6702	81.8437
13330	3.2078	-2.8933	136.717
13331	3.2079	-3.5744	40.986
13332	3.2079	67.0514	178.971
13333	3.208	64.1571	322.375

13334	3.208	62.521	200.481
13335	3.2081	-3.0445	239.422
13336	3.2082	-2.9039	8.5231
13337	3.2082	87.2552	48.8705
13338	3.2084	91.5641	65.3163
13339	3.2084	41.8591	350.004
13340	3.2084	-2.8609	136.878
13341	3.2085	73.9339	115.161
13342	3.2087	5.3048	66.2696
13343	3.2089	58.8316	306.301
13344	3.2091	-2.7301	36.3706
13345	3.2092	66.3145	127.983
13346	3.2094	56.1668	140.751
13347	3.2094	49.8768	101.734
13348	3.2097	47.9071	175.79
13349	3.2097	-2.787	204.915
13350	3.2097	61.7026	178.275
13351	3.21	-3.426	170.83
13352	3.2101	-3.2445	80.9775
13353	3.2104	-3.444	258.822
13354	3.2107	56.6592	99.4278
13355	3.2108	56.045	230.043
13356	3.2111	-4.2397	282.838
13357	3.2112	62.5622	278.74
13358	3.2116	-2.7602	186.488
13359	3.2116	71.5911	336.598
13360	3.2117	-3.314	168.782
13361	3.2117	-2.9096	232.403
13362	3.2117	63.0296	243.333
13363	3.2118	-2.957	64.7435
13364	3.2119	14.6298	68.5188
13365	3.2119	64.7557	104.362
13366	3.212	-2.876	305.946
13367	3.2121	69.3914	27.2815
13368	3.2125	60.9841	237.22
13369	3.2126	-2.8955	243.215
13370	3.2127	-4.4879	232.62
13371	3.2128	66.466	152.502
13372	3.213	72.116	126.629
13373	3.2131	-4.3532	348.861
13374	3.2132	-2.9292	125.675
13375	3.2133	75.2997	93.9608
13376	3.2133	71.6468	68.8316
13377	3.2133	9.0029	242.875
13378	3.2136	-4.1778	110.104
13379	3.2136	-2.8658	204.121
13380	3.2139	71.1948	74.7247
13381	3.2139	69.8198	29.7614

13382	3.2139	61.1945	163.665
13383	3.2139	41.3313	144.494
13384	3.2139	-3.6226	216.357
13385	3.2141	-2.9742	122.762
13386	3.2141	-2.9316	110.566
13387	3.2141	-2.9211	89.3848
13388	3.2143	82.7103	313.481
13389	3.2144	-5.9539	87.911
13390	3.2147	46.5988	136.491
13391	3.2147	-4.5991	322.12
13392	3.2147	-2.8225	348.914
13393	3.2148	57.5226	20.6385
13394	3.2148	-3.034	217.313
13395	3.215	57.2843	181.078
13396	3.215	-2.8056	352.531
13397	3.2152	63.1001	95.353
13398	3.2152	71.2452	188.21
13399	3.2152	-2.8972	166.52
13400	3.2154	53.1893	131.137
13401	3.2154	69.0735	135.023
13402	3.2156	-3.1679	47.5893
13403	3.2156	70.2723	63.5185
13404	3.2156	70.833	299.034
13405	3.2158	60.895	256.514
13406	3.2158	-2.9402	16.3787
13407	3.2159	77.1164	49.8436
13408	3.2161	58.9686	0.221
13409	3.2162	63.1084	206.045
13410	3.2163	56.3267	106.757
13411	3.2163	-2.845	78.6808
13412	3.2164	62.9301	124.185
13413	3.2168	61.4198	101.423
13414	3.2171	48.1595	338.136
13415	3.2171	63.9147	226.596
13416	3.2171	-3.2255	186.97
13417	3.2171	79.752	130.347
13418	3.2172	73.8131	349.286
13419	3.2173	65.7634	55.6005
13420	3.2174	79.9541	44.1382
13421	3.2174	69.2918	80.8821
13422	3.2174	-5.7054	193.852
13423	3.2176	47.7797	115.959
13424	3.2178	72.3833	305.131
13425	3.2178	-3.332	94.278
13426	3.2179	71.606	108.324
13427	3.2181	38.7567	114.766
13428	3.2181	58.646	63.0453
13429	3.2182	70.394	207.622

13430	3.2184	50.1038	167.929
13431	3.2185	67.5672	63.1252
13432	3.2186	54.9709	118.327
13433	3.2186	79.8533	56.7063
13434	3.2187	79.132	203.977
13435	3.2187	90.0552	218.738
13436	3.2189	61.8822	169.213
13437	3.2189	-2.7744	134.319
13438	3.219	43.3456	151.405
13439	3.2192	78.175	70.5881
13440	3.2193	69.8338	328.904
13441	3.2194	-2.8078	92.0924
13442	3.2194	47.0047	124.32
13443	3.2195	61.1737	108.136
13444	3.2197	-2.7923	23.6101
13445	3.2197	62.5951	357.229
13446	3.2198	59.5173	98.7149
13447	3.22	80.2272	36.7325
13448	3.2201	56.5525	178.921
13449	3.2202	36.3596	247.412
13450	3.2203	79.3406	206.974
13451	3.2203	-2.8006	343.471
13452	3.2203	52.4121	249.983
13453	3.2204	-3.4177	346.084
13454	3.2204	48.8646	124.162
13455	3.2204	-3.2329	319.337
13456	3.2205	47.3391	198.438
13457	3.2205	49.0002	343.729
13458	3.2205	60.3825	291.689
13459	3.2206	-2.9174	251.235
13460	3.2206	59.8485	131.009
13461	3.2209	54.0216	329.311
13462	3.221	52.2154	235.178
13463	3.2215	89.6695	5.0366
13464	3.2215	63.1155	113.394
13465	3.2217	61.0415	219.15
13466	3.2219	64.8029	317.389
13467	3.2219	72.5547	83.6143
13468	3.2219	56.5932	264.542
13469	3.2219	67.7806	346.966
13470	3.2221	61.5635	116.64
13471	3.2222	53.4467	130.223
13472	3.2226	69.3712	89.8852
13473	3.2226	-3.9339	80.8513
13474	3.2227	-4.2577	131.018
13475	3.2227	60.718	50.8219
13476	3.2231	53.523	292.693
13477	3.2231	30.3848	284.728

13478	3.2231	65.1675	149.004
13479	3.2233	82.979	64.3695
13480	3.2234	75.1786	153.947
13481	3.2235	65.6581	134.831
13482	3.2235	64.4066	0.1727
13483	3.2236	62.3263	66.8684
13484	3.2237	75.1361	214.761
13485	3.2239	63.8983	97.0135
13486	3.2239	-4.1126	46.1797
13487	3.2242	-3.1874	130.891
13488	3.2243	79.3547	169.435
13489	3.2243	88.3232	212.162
13490	3.2244	58.8257	124.977
13491	3.2245	90.9752	41.1804
13492	3.2245	-3.5632	110.459
13493	3.2246	57.4857	268.231
13494	3.2246	68.5269	282.514
13495	3.2248	76.4177	32.4033
13496	3.2248	55.6063	169.174
13497	3.2249	55.5412	159.234
13498	3.225	-2.7129	43.557
13499	3.225	67.1571	261.987
13500	3.2251	-2.9569	330.109
13501	3.2251	67.0846	332.243
13502	3.2252	55.743	240.376
13503	3.2253	55.2106	128.305
13504	3.2257	59.1112	162.747
13505	3.2258	69.5106	72.443
13506	3.2258	-3.3729	258.34
13507	3.2258	68.1856	65.5787
13508	3.2259	59.4152	109.614
13509	3.2259	-4.1564	263.59
13510	3.2262	73.069	316.916
13511	3.2264	60.1905	177.722
13512	3.2264	-2.78	331.557
13513	3.2266	56.2677	180.058
13514	3.2267	-3.1621	251.403
13515	3.2268	67.6018	311.576
13516	3.2268	-3.7263	87.8712
13517	3.2268	73.9809	234.833
13518	3.2271	95.1467	6.3557
13519	3.2273	-5.6596	235.905
13520	3.2274	58.8775	222.527
13521	3.2275	80.6598	288.372
13522	3.2275	49.9785	144.881
13523	3.2275	-2.69	158.284
13524	3.2276	-3.0581	287.488
13525	3.2276	61.7214	152.818

13526	3.2276	81.1933	206.253
13527	3.2276	-3.3114	323.222
13528	3.2277	68.9351	335.79
13529	3.2278	61.4806	58.2691
13530	3.2278	-2.8232	278.828
13531	3.2279	-3.0069	242.21
13532	3.228	-3.6153	91.2016
13533	3.2281	77.3075	356.543
13534	3.2282	-3.153	22.435
13535	3.2283	60.3053	191.69
13536	3.2283	68.4822	15.9778
13537	3.2285	62.9729	142.061
13538	3.2286	78.4969	206.25
13539	3.2287	-3.0778	139.857
13540	3.2287	64.5305	270.002
13541	3.2287	50.8373	141.62
13542	3.2287	61.9026	48.1372
13543	3.2287	74.1563	41.4165
13544	3.2288	60.2933	224.912
13545	3.2289	63.508	103.356
13546	3.2289	101.875	16.3358
13547	3.229	68.254	80.1744
13548	3.229	65.7395	164.072
13549	3.2293	-4.2805	49.0527
13550	3.2293	-3.9585	257.956
13551	3.2297	79.0574	172.135
13552	3.2299	68.3167	146.62
13553	3.23	-3.2914	132.162
13554	3.2301	75.6883	24.4464
13555	3.2303	44.4384	339.198
13556	3.2303	55.0329	263.788
13557	3.2305	31.3748	18.3164
13558	3.2306	83.0717	41.8506
13559	3.2307	96.2086	87.8069
13560	3.231	66.3961	221.647
13561	3.231	63.4286	82.2583
13562	3.231	63.4373	275.27
13563	3.2313	63.7034	80.9559
13564	3.2313	70.5081	221.9
13565	3.2313	70.3713	235.654
13566	3.2315	62.8959	76.3299
13567	3.2316	55.2428	215.111
13568	3.2316	70.2287	119.503
13569	3.2319	64.4937	41.5601
13570	3.232	57.6838	69.7278
13571	3.2321	62.8251	165.141
13572	3.2321	-3.9006	7.0221
13573	3.2324	84.8041	128.152

13574	3.2326	-2.848	205.642
13575	3.2327	78.7382	76.4754
13576	3.2329	67.7014	15.7144
13577	3.233	4.5022	172.815
13578	3.233	-2.8905	265.383
13579	3.2332	57.8006	300.029
13580	3.2334	78.821	43.7798
13581	3.2334	-2.8819	237.094
13582	3.2335	-3.7267	243.561
13583	3.2337	-2.8364	211.17
13584	3.2339	65.7655	86.0513
13585	3.2343	51.9397	183.505
13586	3.2345	57.7152	179.937
13587	3.2345	67.71	292.526
13588	3.2347	63.9781	272.503
13589	3.2348	-2.866	272.025
13590	3.2351	84.8283	53.6846
13591	3.2352	70.5425	146.331
13592	3.2356	66.0636	195.171
13593	3.2358	59.5821	138.068
13594	3.2359	69.8614	173.326
13595	3.236	16.0977	168.824
13596	3.2362	76.3777	303.433
13597	3.2363	67.4062	241.115
13598	3.2363	60.5637	232.1
13599	3.2363	-3.499	272.365
13600	3.2365	51.7499	117.503
13601	3.2367	77.6025	116.327
13602	3.2367	56.0525	114.036
13603	3.2368	54.0151	19.5555
13604	3.2369	70.2414	326.974
13605	3.2369	-3.5005	140.846
13606	3.237	94.1528	8.2127
13607	3.2371	51.6747	28.9408
13608	3.2372	66.0263	122.085
13609	3.2373	71.356	278.575
13610	3.2374	95.6997	8.1012
13611	3.2376	49.2898	154.999
13612	3.2378	64.0179	200.156
13613	3.2378	71.0522	151.562
13614	3.2379	25.807	342.13
13615	3.2381	-4.2919	153.652
13616	3.2382	-2.9228	357.971
13617	3.2382	70.6584	165.102
13618	3.2382	64.2957	116.3
13619	3.2384	62.603	190.501
13620	3.2387	55.8135	233.417
13621	3.2388	85.1902	85.4352

13622	3.239	68.1815	130.276
13623	3.239	60.8787	97.9625
13624	3.2391	80.7676	23.3546
13625	3.2392	-2.764	71.7849
13626	3.2393	80.5613	205.847
13627	3.2393	75.7366	358.412
13628	3.2394	48.3344	300.565
13629	3.2394	-2.7249	266.615
13630	3.2395	70.082	36.4424
13631	3.2396	64.6212	214.042
13632	3.2397	53.9061	166.354
13633	3.2398	73.6307	90.8182
13634	3.2399	61.9814	219.341
13635	3.2404	78.5571	113.852
13636	3.2405	66.8222	64.143
13637	3.2405	62.461	189.963
13638	3.2405	72.1224	76.8112
13639	3.2406	63.9658	111.752
13640	3.2408	-3.8309	82.5675
13641	3.2409	58	12.3902
13642	3.2409	60.6582	309.671
13643	3.2409	52.6294	118.645
13644	3.2409	59.1544	264.009
13645	3.2409	51.1399	146.523
13646	3.2414	72.1963	95.564
13647	3.2415	80.038	273.858
13648	3.2417	-3.8908	92.084
13649	3.2417	53.833	160.169
13650	3.2417	64.7665	65.4645
13651	3.2418	58.6631	112.556
13652	3.2418	60.3897	266.035
13653	3.2422	72.4535	245.293
13654	3.2423	51.1544	114.242
13655	3.2425	79.3972	196.954
13656	3.2426	-4.1128	133.589
13657	3.2426	67.3729	137.078
13658	3.2427	56.5356	144.06
13659	3.2427	64.5113	160.524
13660	3.2429	61.8559	254.209
13661	3.2429	63.5203	62.9674
13662	3.2429	96.3764	315.221
13663	3.243	71.1232	259.009
13664	3.243	64.1444	69.3343
13665	3.2434	67.5807	253.15
13666	3.2435	76.9652	235.96
13667	3.2435	40.2873	4.4753
13668	3.2436	57.9012	139.761
13669	3.2437	-3.1167	151.219

13670	3.2438	88.1216	131.91
13671	3.2438	73.0796	233.746
13672	3.2438	76.8397	237.321
13673	3.2438	82.7881	138.107
13674	3.2439	68.3788	103.957
13675	3.2439	-4.4256	6.5668
13676	3.244	-2.9035	111.891
13677	3.2443	82.0762	271.791
13678	3.2444	76.3388	199.39
13679	3.2445	-2.6971	336.143
13680	3.2445	61.3809	106.758
13681	3.2446	-2.8546	158.741
13682	3.2449	83.3046	169.341
13683	3.2449	66.6144	256.718
13684	3.2449	73.372	213.284
13685	3.2449	71.4744	308.846
13686	3.245	49.8791	221.171
13687	3.2451	87.8093	149.873
13688	3.2455	62.5385	266.928
13689	3.2455	-2.6691	249.502
13690	3.2455	-2.8115	97.3395
13691	3.2455	-2.9706	338.217
13692	3.2455	60.4117	298.64
13693	3.2461	58.1402	185.953
13694	3.2462	-2.6848	159.853
13695	3.2463	60.4444	137.201
13696	3.2464	84.0078	50.1958
13697	3.2464	-2.8353	209.04
13698	3.2466	-0.624	336.392
13699	3.2468	56.8323	110.236
13700	3.2468	72.2863	10.7124
13701	3.2471	55.1795	108.516
13702	3.2473	53.1946	180.526
13703	3.2473	58.6626	286.108
13704	3.2473	57.0586	106.065
13705	3.2474	45.6348	44.5771
13706	3.2475	59.4436	107.901
13707	3.2477	65.8202	326.785
13708	3.2477	-4.458	309.9
13709	3.2478	71.2414	277.113
13710	3.2479	75.9735	293.794
13711	3.2479	63.1274	93.5417
13712	3.2479	-2.7552	135.14
13713	3.248	56.2362	200.638
13714	3.2482	68.8918	301.103
13715	3.2483	85.3967	288.656
13716	3.2485	82.4631	175.877
13717	3.2485	85.4653	199.403

13718	3.2486	57.1985	235.082
13719	3.2488	83.6356	346.829
13720	3.2492	64.7436	60.1415
13721	3.2493	-3.0014	56.1602
13722	3.2493	-4.3076	305.302
13723	3.2495	72.0694	36.812
13724	3.2496	59.1142	263.471
13725	3.2497	54.1859	268.875
13726	3.2502	72.5688	214.833
13727	3.2502	62.2698	33.4783
13728	3.2504	65.3644	104.014
13729	3.2505	60.7669	197.156
13730	3.2505	80.8391	205.403
13731	3.2506	89.4202	39.5211
13732	3.2506	58.6787	171.068
13733	3.2509	69.3987	162.236
13734	3.2509	-2.7321	2.7782
13735	3.2509	25.3729	115.289
13736	3.251	77.2009	283.421
13737	3.251	67.5159	148.849
13738	3.2511	68.4536	207.801
13739	3.2512	-3.8885	322.289
13740	3.2512	-3.0177	209.641
13741	3.2512	71.4627	127.146
13742	3.2513	80.2153	117.889
13743	3.2515	-2.7831	95.2059
13744	3.2515	-3.648	8.9941
13745	3.2515	69.15	327.287
13746	3.2516	98.1084	132.107
13747	3.2517	70.1337	164.611
13748	3.2518	69.9492	21.2718
13749	3.2518	74.6696	126.039
13750	3.2519	65.8991	143.533
13751	3.252	71.9909	158.209
13752	3.2521	-2.781	128.722
13753	3.2521	88.2531	198.043
13754	3.2522	73.7008	14.7757
13755	3.2522	76.032	242.993
13756	3.2525	13.3562	228.834
13757	3.2525	-2.8097	346.202
13758	3.2527	61.1856	110.675
13759	3.2527	56.0637	121.15
13760	3.253	55.4656	125.946
13761	3.253	-2.7819	64.1652
13762	3.2532	65.3551	33.7787
13763	3.2533	68.914	272.373
13764	3.2533	67.0849	201.149
13765	3.2534	54.2572	169.904

13766	3.2536	-4.6476	129.643
13767	3.2536	-2.8335	46.9108
13768	3.2538	82.3618	99.5792
13769	3.2538	67.774	47.9044
13770	3.2539	71.0167	17.4259
13771	3.2539	42.351	178.443
13772	3.254	70.8532	249.093
13773	3.2541	70.3699	133.696
13774	3.2541	67.7175	84.9196
13775	3.2542	73.9187	270.403
13776	3.2543	67.5947	93.2734
13777	3.2547	54.9864	116.306
13778	3.2547	74.9826	37.6654
13779	3.2549	75.1103	86.3133
13780	3.2549	64.3629	192.199
13781	3.255	58.8653	130.43
13782	3.2551	74.0403	28.2061
13783	3.2552	56.9827	353.645
13784	3.2552	-4.1125	332.674
13785	3.2553	85.9874	81.577
13786	3.2554	75.1714	62.7944
13787	3.2555	-3.2516	178.669
13788	3.2555	55.6306	332.488
13789	3.2559	62.2522	248.51
13790	3.2562	60.4439	282.939
13791	3.2563	89.6335	298.966
13792	3.2563	63.1768	38.6748
13793	3.2564	71.3942	212.328
13794	3.2564	68.0139	135.97
13795	3.2565	66.9515	277.505
13796	3.2567	72.2627	298.4
13797	3.2567	75.211	297.036
13798	3.2568	-3.1063	356.545
13799	3.2568	72.6755	93.8109
13800	3.2569	79.5394	227.957
13801	3.2569	-2.8164	327.17
13802	3.2569	-3.5407	108.751
13803	3.2569	60.8787	209.268
13804	3.257	74.6183	330.072
13805	3.2571	64.5714	211.958
13806	3.2571	59.1804	184.914
13807	3.2571	79.997	148.798
13808	3.2573	75.7872	207.231
13809	3.2574	66.0239	259.597
13810	3.2574	70.8521	323.943
13811	3.2575	50.344	198.286
13812	3.2576	67.2947	180.501
13813	3.2576	-3.9528	155.056

13814	3.2577	72.2264	199.187
13815	3.2578	67.7336	124.659
13816	3.2578	58.2849	140.977
13817	3.2579	83.0902	153.007
13818	3.258	79.5232	305.105
13819	3.2581	58.0041	152.9
13820	3.2583	69.8711	92.9975
13821	3.2584	56.6216	250.467
13822	3.2584	82.1907	358.741
13823	3.2586	52.1945	333.654
13824	3.2587	76.6784	306.284
13825	3.2587	-2.9165	132.043
13826	3.2587	65.5593	282.092
13827	3.2588	62.6421	154.544
13828	3.2589	-2.8438	120.698
13829	3.2589	64.0447	22.5631
13830	3.2589	-2.8089	205.635
13831	3.2591	43.7222	143.154
13832	3.2592	69.732	127.267
13833	3.2593	38.0564	51.0682
13834	3.2597	65.5203	219.123
13835	3.2598	67.7465	29.8033
13836	3.2599	55.9537	167.503
13837	3.2599	79.5042	206.452
13838	3.2599	68.343	319.978
13839	3.26	62.9421	135.732
13840	3.26	-3.3916	331.169
13841	3.2601	73.6479	233.359
13842	3.2604	73.8485	53.477
13843	3.2604	82.5069	149.578
13844	3.2605	52.0009	134.998
13845	3.2608	71.5405	312.163
13846	3.2609	60.5973	221.543
13847	3.2609	61.7376	110.91
13848	3.2611	67.2899	272.957
13849	3.2611	64.7474	51.1856
13850	3.2614	-3.1765	137.507
13851	3.2614	72.821	228.971
13852	3.2614	74.0638	156.71
13853	3.2616	66.5959	65.9089
13854	3.2617	-4.4984	122.973
13855	3.2617	-3.9268	90.7884
13856	3.2618	60.4933	210.514
13857	3.2619	68.1556	166.438
13858	3.2619	65.6123	150.238
13859	3.2621	60.2569	149.717
13860	3.2621	-2.7793	258.259
13861	3.2624	60.1449	191.387

13862	3.2624	69.6975	311.281
13863	3.2626	58.5674	118.262
13864	3.2626	72.0405	29.8085
13865	3.2627	73.881	115.12
13866	3.2628	54.3984	262.062
13867	3.263	63.1364	108.328
13868	3.2631	79.0281	30.2034
13869	3.2631	66.5603	77.515
13870	3.2631	62.1878	192.774
13871	3.2632	-3.8437	139.983
13872	3.2633	73.9209	209.145
13873	3.2634	-3.3232	255.192
13874	3.2634	58.6453	198.089
13875	3.2638	-4.0137	90.008
13876	3.2639	59.4855	77.0811
13877	3.2643	-2.6528	170.988
13878	3.2644	63.6956	143.64
13879	3.2644	66.3881	73.6587
13880	3.2645	58.3366	94.1659
13881	3.2645	19.8274	62.7698
13882	3.2646	12.0144	123.225
13883	3.2647	61.7261	49.261
13884	3.2647	46.2836	129.912
13885	3.2648	-2.8784	250.464
13886	3.2648	60.736	18.5536
13887	3.2653	85.3566	14.5855
13888	3.2653	72.017	271.639
13889	3.2653	60.7345	126.391
13890	3.2653	60.7863	214.221
13891	3.2653	76.2379	168.735
13892	3.2653	63.0991	304.545
13893	3.2654	81.8383	271.797
13894	3.2656	67.3338	140.176
13895	3.2656	58.8941	255.954
13896	3.2656	69.7704	101.193
13897	3.2657	55.0639	333.304
13898	3.266	81.266	356.881
13899	3.2662	-2.918	221.928
13900	3.2663	50.1039	133.851
13901	3.2663	80.8774	61.3634
13902	3.2665	84.3026	21.6765
13903	3.2665	18.1786	71.6326
13904	3.2666	67.5031	114.04
13905	3.2667	50.9231	147.064
13906	3.2668	63.6999	67.6091
13907	3.2669	92.9218	208.894
13908	3.2673	54.4411	134.548
13909	3.2674	70.3897	261.548

13910	3.2675	81.2705	354.61
13911	3.2675	60.9286	190.56
13912	3.2676	70.3095	343.718
13913	3.2676	103.015	24.3955
13914	3.2677	89.9402	290.653
13915	3.2678	83.8251	63.5012
13916	3.2679	69.0787	249.329
13917	3.2679	55.6622	125.441
13918	3.2679	50.5074	186.428
13919	3.2681	61.5442	175.02
13920	3.2682	-3.9642	330.907
13921	3.2682	59.9794	135.746
13922	3.2683	66.8083	158.476
13923	3.2684	94.9463	22.0826
13924	3.2684	62.7185	199.082
13925	3.2685	61.0779	319.842
13926	3.2685	72.1372	213.544
13927	3.2687	77.4219	22.5794
13928	3.2687	53.913	173.923
13929	3.2687	73.138	25.7354
13930	3.2688	57.8349	121.107
13931	3.2689	65.0225	287.467
13932	3.2691	83.7801	242.371
13933	3.2691	82.3049	329.042
13934	3.2692	-3.3499	273.437
13935	3.2693	67.4664	280.31
13936	3.2693	-2.7965	164.525
13937	3.2695	113.18	41.874
13938	3.2696	-2.7803	325.515
13939	3.2698	91.8954	59.9407
13940	3.27	77.2651	301.639
13941	3.2703	66.6067	103.086
13942	3.2704	49.2215	25.6848
13943	3.2706	70.0063	75.0061
13944	3.2706	70.7127	145.709
13945	3.2707	75.8705	106.58
13946	3.2709	82.4161	242.595
13947	3.271	69.6964	122.035
13948	3.2713	63.8222	117.887
13949	3.2714	77.1538	295.246
13950	3.2714	-4.0205	195.461
13951	3.2715	71.8523	87.7755
13952	3.2715	-3.2696	24.11
13953	3.2717	68.561	212.936
13954	3.2719	64.2731	185.454
13955	3.2719	73.2817	127.398
13956	3.2721	-4.3567	233.203
13957	3.2722	59.7398	224.022

13958	3.2723	67.1359	135.222
13959	3.2724	65.6703	100.98
13960	3.2724	52.838	152.108
13961	3.2725	65.5758	97.9557
13962	3.2726	-3.1499	274.241
13963	3.2727	-3.0038	12.1422
13964	3.2727	66.0144	130.557
13965	3.2727	76.8725	260.909
13966	3.2728	56.9173	197.829
13967	3.2728	58.0584	244.513
13968	3.2728	67.4881	145.198
13969	3.2729	75.5655	164.184
13970	3.2729	81.1064	165.803
13971	3.273	-3.6984	7.4091
13972	3.2732	73.6669	156.82
13973	3.2734	74.789	200.718
13974	3.2736	-4.1187	22.6826
13975	3.2737	69.501	132.646
13976	3.2739	80.6875	215.418
13977	3.2739	-3.4695	87.9267
13978	3.2739	-2.9033	264.852
13979	3.274	76.2384	26.3206
13980	3.2743	67.1533	159.258
13981	3.2743	70.1855	147.405
13982	3.2743	52.6135	276.017
13983	3.2743	58.6111	354.184
13984	3.2744	92.4212	238.591
13985	3.2744	59.0012	115.35
13986	3.2744	-2.8128	282.786
13987	3.2745	86.8294	234.56
13988	3.2746	65.4237	235.974
13989	3.2746	-2.7456	16.5954
13990	3.2747	64.4014	256.413
13991	3.2749	61.5891	147.715
13992	3.275	71.0387	78.4498
13993	3.275	83.6188	19.8431
13994	3.2751	58.1843	63.1815
13995	3.2755	30.5214	53.4856
13996	3.2756	-4.0181	264.978
13997	3.2757	-2.9633	164.573
13998	3.2758	60.5422	297.003
13999	3.2761	55.4887	191.741
14000	3.2763	72.8984	181.034
14001	3.2763	62.4019	290.803
14002	3.2765	45.9942	297.932
14003	3.2765	77.3216	286.484
14004	3.2766	-3.2245	136.793
14005	3.2767	-3.8682	127.25

14006	3.2767	-3.9211	145.933
14007	3.2768	76.5611	226.693
14008	3.2769	-3.0961	79.5654
14009	3.2769	78.793	33.2605
14010	3.277	58.6406	144.975
14011	3.2771	-2.8498	140.609
14012	3.2773	77.2958	201.691
14013	3.2773	78.9636	324.71
14014	3.2775	-2.9407	42.366
14015	3.2776	-2.8787	320.381
14016	3.2777	68.5568	277.208
14017	3.2777	-2.8393	337.026
14018	3.2778	76.4555	68.1757
14019	3.278	69.7619	264.793
14020	3.278	59.2966	161.199
14021	3.278	56.7411	158.548
14022	3.278	-3.5769	262.755
14023	3.2782	-1.0894	258.812
14024	3.2784	-3.7996	54.7815
14025	3.2785	75.3711	204.121
14026	3.2786	-3.6843	67.7105
14027	3.2787	78.877	42.5205
14028	3.2789	-3.8708	328.178
14029	3.2789	74.2532	220.207
14030	3.279	85.0118	49.9538
14031	3.2791	61.1543	52.7543
14032	3.2792	74.0983	239.143
14033	3.2793	-2.8287	46.058
14034	3.2797	100.279	270.501
14035	3.2798	-2.7978	142.672
14036	3.28	18.185	274.031
14037	3.2801	82.4919	86.4117
14038	3.2802	77.6671	43.7712
14039	3.2802	66.8103	267.139
14040	3.2803	77.912	211.695
14041	3.2804	67.3978	176.861
14042	3.2806	77.7931	74.2058
14043	3.2806	75.2256	71.5223
14044	3.2807	68.1683	206.489
14045	3.2807	79.315	314.842
14046	3.2807	-3.9714	57.9071
14047	3.2809	-2.8216	113.485
14048	3.2809	77.9425	278.829
14049	3.2809	63.3778	224.752
14050	3.2809	-2.9235	144.827
14051	3.281	55.0048	161.681
14052	3.2811	63.3803	286.355
14053	3.2813	-2.8588	80.8487

14054	3.2813	66.7624	22.8407
14055	3.2813	-2.87	253.329
14056	3.2814	56.2237	198.789
14057	3.2817	-2.916	191.008
14058	3.2817	61.7804	193.773
14059	3.2818	-2.8425	317.325
14060	3.2819	75.5964	153.857
14061	3.282	69.1619	183.775
14062	3.282	-3.2421	343.751
14063	3.2821	70.5714	43.4429
14064	3.2821	-2.9653	61.4163
14065	3.2822	73.3544	26.5356
14066	3.2824	97.2388	120.265
14067	3.2824	51.0102	124.069
14068	3.2824	-3.4734	65.6473
14069	3.2825	65.9688	307.073
14070	3.2825	-2.8531	143.02
14071	3.2826	78.3267	119.202
14072	3.2827	-3.1686	98.7814
14073	3.2827	67.7572	29.9886
14074	3.2828	83.2582	37.8173
14075	3.2828	77.062	64.8617
14076	3.2831	84.9715	94.5574
14077	3.2834	56.9024	181.957
14078	3.2835	-3.0375	47.8441
14079	3.2835	84.2831	264.695
14080	3.2836	71.4945	145.793
14081	3.2836	-4.1252	264.207
14082	3.2837	-2.8878	350.693
14083	3.2837	77.0788	311.204
14084	3.2839	61.022	92.368
14085	3.2839	66.0697	341.399
14086	3.284	-3.0239	141.756
14087	3.284	70.6127	30.3787
14088	3.284	63.2619	133.566
14089	3.284	-3.283	116.145
14090	3.2841	81.2501	246.492
14091	3.2841	64.6601	237.492
14092	3.2841	110.763	314.977
14093	3.2843	85.9384	51.1214
14094	3.2843	54.5559	134.046
14095	3.2844	-2.7848	45.6994
14096	3.2845	60.2232	197.789
14097	3.2845	-4.5068	317.124
14098	3.2846	66.0455	22.8743
14099	3.2846	-3.7191	310.812
14100	3.2847	56.6109	133.217
14101	3.2848	87.2906	149.594

14102	3.2848	-3.1047	195.145
14103	3.2851	91.9178	45.7489
14104	3.2852	76.9914	254.995
14105	3.2852	72.3399	18.7771
14106	3.2853	58.4371	300.703
14107	3.2855	-3.3152	95.589
14108	3.2857	92.3799	74.5376
14109	3.2857	66.6606	196.188
14110	3.2859	75.6728	79.05
14111	3.2864	71.5569	223.952
14112	3.2865	68.7262	268.205
14113	3.2865	62.0295	127.845
14114	3.2865	70.4727	226.855
14115	3.2867	87.9978	123.231
14116	3.2867	73.0311	254.642
14117	3.2867	55.8287	155.06
14118	3.2868	75.4422	199.667
14119	3.2868	65.5703	69.9659
14120	3.2869	79.8734	206.704
14121	3.2871	50.2022	272.456
14122	3.2872	-3.916	278.788
14123	3.2874	-4.0095	223.449
14124	3.2874	-2.9453	249.66
14125	3.2876	93.0646	204.501
14126	3.2877	60.7543	110.743
14127	3.2878	61.6449	100.273
14128	3.2881	85.8688	35.0863
14129	3.2882	-3.0192	187.646
14130	3.2883	76.8707	327.586
14131	3.2884	-4.0661	117.162
14132	3.2885	-2.9673	265.553
14133	3.2887	66.0757	127.509
14134	3.2887	-4.4266	309.271
14135	3.2887	-2.8851	258.718
14136	3.2889	94.2367	240.012
14137	3.289	77.4595	308.4
14138	3.2891	62.7197	115.049
14139	3.2891	73.8437	98.3655
14140	3.2892	78.6751	215.37
14141	3.2893	57.2426	316.101
14142	3.2893	87.3114	21.3281
14143	3.2896	71.8721	263.502
14144	3.2898	-4.003	343.545
14145	3.29	74.005	63.3188
14146	3.2902	91.1824	281.505
14147	3.2902	73.4574	129.743
14148	3.2903	-3.0223	351.607
14149	3.2904	79.598	208.71

14150	3.2904	-3.2842	81.6802
14151	3.2905	58.9137	163.045
14152	3.2906	-4.1332	132.423
14153	3.2907	-2.7939	184.111
14154	3.2909	86.8519	48.9158
14155	3.2911	65.8688	90.7358
14156	3.2912	-2.899	349.717
14157	3.2912	95.0344	52.4407
14158	3.2913	80.1378	315.286
14159	3.2915	57.9123	198.611
14160	3.2916	79.9232	152.29
14161	3.2917	74.9727	67.5605
14162	3.2919	77.1565	131.419
14163	3.292	-2.8071	285.945
14164	3.292	56.8245	327.002
14165	3.292	-3.9835	338.092
14166	3.2921	63.2561	276.314
14167	3.2922	73.0402	82.8516
14168	3.2922	43.7365	107.795
14169	3.2924	64.533	121.476
14170	3.2924	61.7304	220.595
14171	3.2926	81.2999	315.706
14172	3.2927	63.1719	60.2612
14173	3.2927	65.7498	254.971
14174	3.2929	68.4039	122.464
14175	3.2929	70.7164	28.4511
14176	3.293	82.7005	273.668
14177	3.2931	69.3811	69.3937
14178	3.2931	67.7048	310.022
14179	3.2931	-4.3019	332.332
14180	3.2933	44.6043	90.0599
14181	3.2934	-2.7956	229.723
14182	3.2936	85.8955	2.6613
14183	3.2936	-2.7057	52.198
14184	3.2937	66.1278	224.073
14185	3.2938	62.7985	300.529
14186	3.294	-2.8653	16.1921
14187	3.2941	55.6998	142.221
14188	3.2941	-2.8372	10.1355
14189	3.2942	67.5417	190.9
14190	3.2942	71.0999	313.041
14191	3.2943	73.1356	184.33
14192	3.2944	82.607	1.4761
14193	3.2946	76.5012	167.008
14194	3.2946	74.6326	261.212
14195	3.295	60.7465	195.337
14196	3.2952	57.5428	323.061
14197	3.2954	45.2681	328.424

14198	3.2955	61.2944	283.057
14199	3.2959	86.986	286.755
14200	3.2959	70.1352	281.996
14201	3.296	72.8386	109.226
14202	3.2961	-3.5601	60.0591
14203	3.2961	53.273	133.483
14204	3.2961	73.3823	41.4897
14205	3.2963	-2.7198	20.2849
14206	3.2964	61.7699	278.663
14207	3.2964	67.106	98.0898
14208	3.2965	89.0995	75.7546
14209	3.2966	81.7038	144.643
14210	3.2966	73.4961	95.8556
14211	3.2967	-3.2495	79.4319
14212	3.2971	87.0967	215.473
14213	3.2971	-3.0536	302.135
14214	3.2972	-3.3874	53.6366
14215	3.2972	73.4237	49.2836
14216	3.2973	73.1758	156.855
14217	3.2977	-2.9303	100.922
14218	3.2977	68.9383	78.7501
14219	3.2978	74.5998	199.478
14220	3.2978	24.5054	289.878
14221	3.2979	63.219	175.767
14222	3.298	-4.2247	118.604
14223	3.2981	53.2676	151.068
14224	3.2982	63.657	159.494
14225	3.2984	69.8291	216.742
14226	3.2986	87.912	48.7306
14227	3.2986	64.9606	117.62
14228	3.2987	65.2976	56.4609
14229	3.2987	66.6946	350.475
14230	3.299	-3.0503	97.9951
14231	3.299	63.5541	155.623
14232	3.299	70.6666	58.917
14233	3.2991	-3.7674	358.353
14234	3.2992	69.9371	247.495
14235	3.2996	76.3655	233.596
14236	3.2997	-3.4842	230.997
14237	3.2998	-3.0453	254.573
14238	3.3	-2.9386	195.383
14239	3.3001	89.9256	201.359
14240	3.3001	-3.1185	17.5578
14241	3.3002	57.6218	268.319
14242	3.3002	-2.674	38.5416
14243	3.3003	86.803	237.711
14244	3.3004	59.7767	23.4491
14245	3.3005	91.8486	190.009

14246	3.3006	-4.3844	128.035
14247	3.3008	-3.5783	150.142
14248	3.3008	66.4289	56.9333
14249	3.3008	45.7296	322.237
14250	3.3008	82.933	148.233
14251	3.3009	63.5304	240.455
14252	3.301	66.2962	126.994
14253	3.301	71.0233	172.573
14254	3.3011	62.8883	64.1901
14255	3.3012	92.8848	57.48
14256	3.3012	65.5368	165.454
14257	3.3012	83.8515	223.961
14258	3.3012	74.46	120.183
14259	3.3013	-2.8742	75.9232
14260	3.3013	64.1028	91.6469
14261	3.3014	85.9232	23.2642
14262	3.3014	62.0566	108.723
14263	3.3014	-2.8057	272.989
14264	3.3016	56.0751	161.037
14265	3.3017	-4.0819	6.4046
14266	3.3018	76.0876	123.259
14267	3.3018	66.7538	241.375
14268	3.3019	75.9331	35.2041
14269	3.302	69.8265	167.603
14270	3.3022	90.3303	42.1097
14271	3.3022	79.197	287.79
14272	3.3022	-2.8637	253.836
14273	3.3023	73.7887	186.659
14274	3.3023	-2.8347	338.703
14275	3.3024	54.9619	247.013
14276	3.3026	30.8494	125.781
14277	3.3027	93.0096	76.2789
14278	3.3028	75.7678	74.3592
14279	3.3028	83.0044	289.86
14280	3.3029	87.109	286.957
14281	3.303	54.7913	57.4159
14282	3.303	-3.3168	11.8095
14283	3.3032	69.8225	143.011
14284	3.3033	-3.1939	245.017
14285	3.3033	70.9077	110.18
14286	3.3036	84.6034	43.5145
14287	3.3036	-2.7939	169.113
14288	3.3036	68.5355	106.279
14289	3.3036	-2.7503	116.48
14290	3.3037	-3.7324	24.3065
14291	3.3039	62.0003	119.424
14292	3.304	-3.6393	3.7278
14293	3.3041	60.4093	16.3842

14294	3.3041	70.9448	130.436
14295	3.3042	78.0016	140.873
14296	3.3042	81.3392	206.021
14297	3.3042	80.9111	38.3498
14298	3.3043	108.91	7.7343
14299	3.3045	71.6461	66.9179
14300	3.3045	85.6338	83.9226
14301	3.3048	95.3627	204.421
14302	3.305	60.3645	217.357
14303	3.3052	95.1726	286.551
14304	3.3052	72.1528	201.727
14305	3.3052	-3.9867	193.258
14306	3.3054	68.8719	258.654
14307	3.3054	77.2351	254.28
14308	3.3055	83.4699	71.9689
14309	3.3055	68.292	102.681
14310	3.3056	80.0705	314.138
14311	3.3057	31.8328	21.4862
14312	3.3059	88.8093	116.736
14313	3.306	-2.7703	6.3779
14314	3.306	74.2483	39.792
14315	3.3064	65.692	130.68
14316	3.3064	72.2123	132.306
14317	3.3065	75.0446	188.068
14318	3.3066	57.1199	259.836
14319	3.3066	90.3331	48.7674
14320	3.3067	-3.316	176.66
14321	3.3068	-2.9976	27.1468
14322	3.3068	-4.3486	273.083
14323	3.307	67.4699	105.592
14324	3.3073	-2.7984	43.943
14325	3.3074	59.0553	234.498
14326	3.3074	63.083	123.676
14327	3.3075	-2.8909	134.369
14328	3.3075	58.4166	262.241
14329	3.3076	78.4126	233.761
14330	3.3076	68.1102	179.085
14331	3.3077	77.6159	72.9118
14332	3.3077	-2.9662	71.6926
14333	3.3078	91.6739	100.176
14334	3.3078	55.8459	167.539
14335	3.3079	76.0643	161.575
14336	3.308	-3.5689	121.367
14337	3.308	87.4359	18.075
14338	3.308	67.0823	228.746
14339	3.3081	70.8772	277.754
14340	3.3081	79.9585	90.7116
14341	3.3081	-2.8924	265.913

14342	3.3082	78.7723	301.799
14343	3.3082	62.9885	187.013
14344	3.3085	64.2494	271.823
14345	3.3085	-4.01	62.2419
14346	3.3085	65.4023	161.774
14347	3.3087	54.8661	276.872
14348	3.3088	66.8753	98.2442
14349	3.3089	-2.8313	322.638
14350	3.3094	-4.2366	21.3518
14351	3.3095	-3.5875	192.958
14352	3.3096	83.8158	193.047
14353	3.3097	81.5945	87.0373
14354	3.3097	61.5545	144.685
14355	3.3098	73.1289	70.2023
14356	3.3099	-4.0798	281.72
14357	3.3099	90.7884	37.1064
14358	3.31	67.4297	64.2199
14359	3.31	62.6137	137.385
14360	3.3101	71.3428	249.287
14361	3.3102	76.839	102.786
14362	3.3102	51.3487	125.375
14363	3.3103	-2.8614	79.5224
14364	3.3104	57.2251	296.171
14365	3.3105	61.2358	139.766
14366	3.3106	65.5447	98.5793
14367	3.3107	79.7571	69.3557
14368	3.3108	80.1395	133.751
14369	3.3109	-2.8745	165.638
14370	3.3109	81.5886	205.729
14371	3.3109	107.25	70.244
14372	3.311	80.0776	130.314
14373	3.3111	-2.9246	108.586
14374	3.3111	-3.4901	105.443
14375	3.3112	82.8012	90.1259
14376	3.3112	65.4301	46.285
14377	3.3117	81.0794	107.704
14378	3.3117	62.6967	119.526
14379	3.3118	97.7783	109.757
14380	3.3118	-3.1354	236.91
14381	3.3118	76.9059	206.609
14382	3.3119	62.0691	159.061
14383	3.312	73.1966	96.9501
14384	3.3122	72.9202	27.3627
14385	3.3122	66.6455	26.7205
14386	3.3123	80.8209	182.171
14387	3.3123	57.4616	167.887
14388	3.3126	85.4644	146.835
14389	3.3127	67.223	170.591

14390	3.3127	-2.8325	153.141
14391	3.3128	71.6554	1.8378
14392	3.3128	101.026	311.349
14393	3.3131	67.2525	61.5158
14394	3.3131	94.4106	204.703
14395	3.3132	71.6306	107.214
14396	3.3133	84.4927	26.3297
14397	3.3135	70.1592	100.016
14398	3.3136	-4.1384	225.268
14399	3.3139	61.5969	87.8843
14400	3.314	64.5253	27.3117
14401	3.314	-2.8684	355.895
14402	3.3143	72.2466	103.574
14403	3.3144	5.4876	79.2258
14404	3.3144	73.7809	93.5988
14405	3.3145	-2.7829	70.4995
14406	3.3145	73.9621	55.6245
14407	3.3145	-4.7666	74.1417
14408	3.3146	46.917	157.86
14409	3.3146	46.5641	209.448
14410	3.3146	85.7823	108.502
14411	3.3146	59.6114	153.846
14412	3.3147	67.8557	148.616
14413	3.315	56.3668	178.996
14414	3.315	-2.6829	262.314
14415	3.3153	-3.7101	260.237
14416	3.3154	73.7135	126.415
14417	3.3155	78.3165	114.065
14418	3.3156	68.6674	202.786
14419	3.3157	75.4849	174.965
14420	3.3157	-3.8272	91.5316
14421	3.3157	85.485	122.294
14422	3.3158	28.9158	355.497
14423	3.3158	92.1046	204.859
14424	3.316	69.513	16.692
14425	3.3161	73.3255	286.209
14426	3.3161	74.1432	123.045
14427	3.3163	76.3392	148.519
14428	3.3164	-2.9723	277.801
14429	3.3165	108.205	243.264
14430	3.3167	83.7185	80.8097
14431	3.3167	87.9459	283.436
14432	3.3168	73.8272	56.2582
14433	3.3171	80.5077	200.722
14434	3.3172	-0.391	78.6289
14435	3.3173	80.3134	43.9003
14436	3.3173	-2.9137	195.26
14437	3.3175	92.3771	245.854

14438	3.3175	85.9303	236.664
14439	3.3175	86.2352	233.551
14440	3.3176	81.8354	200.918
14441	3.3176	54.9439	136.314
14442	3.3176	73.0835	125.787
14443	3.3177	61.577	26.4105
14444	3.3177	70.7042	349.324
14445	3.3178	87.6021	89.9175
14446	3.3179	85.3541	312.099
14447	3.3179	76.841	6.888
14448	3.318	93.2504	38.219
14449	3.318	66.7798	336.348
14450	3.318	109.921	212.697
14451	3.3181	71.8305	86.2027
14452	3.3183	-3.1884	228.623
14453	3.3184	57.8612	146.712
14454	3.3188	68.5027	216.997
14455	3.3189	118.347	316.103
14456	3.319	81.8514	98.2316
14457	3.319	-3.164	114.323
14458	3.3191	-2.8126	227.657
14459	3.3191	58.427	102.914
14460	3.3192	85.8449	122.003
14461	3.3192	74.6237	89.4604
14462	3.3192	-3.511	190.85
14463	3.3193	83.6902	214.242
14464	3.3193	55.4809	164.056
14465	3.3193	63.6284	229.949
14466	3.3195	87.1684	230.992
14467	3.3197	-2.9827	52.1031
14468	3.3198	78.2968	146.934
14469	3.3199	68.0259	156.774
14470	3.32	61.1335	347.8
14471	3.3204	71.8072	133.872
14472	3.3206	86.1156	144.399
14473	3.3207	74.1768	65.0259
14474	3.3207	20.1633	131.666
14475	3.321	63.7844	196.386
14476	3.3211	94.3246	85.78
14477	3.3214	-2.8136	276.425
14478	3.3214	88.0556	118.007
14479	3.3215	97.6892	206.953
14480	3.3215	69.5066	274.348
14481	3.3215	91.4735	146.853
14482	3.3217	81.7929	114.025
14483	3.3217	74.1998	236.366
14484	3.3217	-3.3105	153.23
14485	3.3217	59.7333	281.737

14486	3.3217	51.8243	80.9921
14487	3.3218	78.9728	9.3012
14488	3.3219	95.5792	40.8127
14489	3.3219	64.6322	92.6177
14490	3.3219	68.3659	18.9493
14491	3.3219	82.1431	271.659
14492	3.322	70.2165	183.672
14493	3.322	67.1717	252.938
14494	3.322	76.5959	58.8251
14495	3.322	87.5677	8.9069
14496	3.3221	70.0628	151.552
14497	3.3222	62.7373	88.1046
14498	3.3224	73.9817	200.269
14499	3.3224	65.5952	349.145
14500	3.3224	76.9507	66.1099
14501	3.3226	107.087	207.474
14502	3.3226	67.9839	237.139
14503	3.3228	75.5046	105.386
14504	3.3229	92.3471	23.9149
14505	3.3231	92.484	48.5036
14506	3.3231	92.8799	320.166
14507	3.3233	92.2584	288.275
14508	3.3233	-2.815	157.22
14509	3.3233	43.8064	156.218
14510	3.3234	66.1316	148.539
14511	3.3235	72.0735	70.9823
14512	3.3235	71.5918	68.9205
14513	3.3236	84.7174	317.599
14514	3.3236	76.431	303.528
14515	3.3237	86.7634	322.811
14516	3.3237	71.2886	133.886
14517	3.3237	-2.9571	215.759
14518	3.3237	-2.8949	268.116
14519	3.3239	76.7931	98.5642
14520	3.324	80.1815	23.0293
14521	3.3241	73.273	144.411
14522	3.3241	58.8568	202.35
14523	3.3241	-2.731	14.3342
14524	3.3242	70.8207	165.908
14525	3.3242	68.9558	136.36
14526	3.3246	70.1255	271.967
14527	3.3247	82.0897	4.7135
14528	3.3247	76.4424	138.985
14529	3.3249	75.9824	252.655
14530	3.3249	-3.8959	51.3275
14531	3.325	-3.8914	172.839
14532	3.3251	-2.9614	32.5171
14533	3.3252	9.5943	198.873

14534	3.3252	83.8892	192.284
14535	3.3253	-5.2452	76.214
14536	3.3254	84.1187	249.161
14537	3.3254	78.8545	209.568
14538	3.3254	71.3429	169.092
14539	3.3255	69.6597	92.6145
14540	3.3257	-2.6873	293.038
14541	3.3259	-3.4355	131.637
14542	3.3259	-4.1686	252.536
14543	3.326	80.8985	219.986
14544	3.326	48.9181	173.873
14545	3.3262	74.8825	132.916
14546	3.3262	-2.9673	116.619
14547	3.3265	80.489	261.726
14548	3.3266	73.7378	245.182
14549	3.3266	50.9385	127.044
14550	3.3267	83.9699	4.3678
14551	3.3267	100.245	14.3238
14552	3.3268	87.1425	356.46
14553	3.327	68.6007	149.237
14554	3.3271	-3.5001	140.496
14555	3.3271	55.9852	5.3279
14556	3.3273	85.4899	205.473
14557	3.3275	-2.7928	126.275
14558	3.3276	79.8667	32.1055
14559	3.3276	68.8474	129.983
14560	3.3278	56.8894	97.3081
14561	3.3278	-2.7037	276.64
14562	3.328	78.0558	286.15
14563	3.3281	65.4673	157.617
14564	3.3282	73.8511	128.313
14565	3.3283	79.7912	256.252
14566	3.3286	86.3307	80.6588
14567	3.3286	68.1891	66.4964
14568	3.3286	89.6162	115.303
14569	3.3289	-3.0659	355.411
14570	3.3289	75.0593	87.4564
14571	3.3289	-2.9706	167.261
14572	3.329	66.3432	75.4279
14573	3.3292	76.8932	189.784
14574	3.3292	69.936	230.86
14575	3.3293	62.8484	235.452
14576	3.3294	66.4599	289.356
14577	3.3297	79.7116	293.871
14578	3.3299	95.4669	85.2715
14579	3.33	41.3556	309.615
14580	3.33	57.4241	23.9334
14581	3.3302	74.7805	129.241

14582	3.3303	84.3817	72.5223
14583	3.3303	96.1913	32.6774
14584	3.3305	74.7124	85.3566
14585	3.3306	80.9704	93.5752
14586	3.3308	71.1142	124.545
14587	3.3309	-3.1055	260.407
14588	3.331	83.9961	281.219
14589	3.331	-2.9379	310.033
14590	3.3315	60.9274	102.923
14591	3.3315	84.3821	179.13
14592	3.3316	86.3825	165.746
14593	3.3317	68.9144	211.709
14594	3.332	70.4011	85.3201
14595	3.332	76.8777	208.284
14596	3.3322	90.238	71.079
14597	3.3322	-2.8378	203.46
14598	3.3322	77.0514	313.703
14599	3.3324	87.7805	90.9567
14600	3.3325	-4.4006	354.767
14601	3.3326	58.6188	174.855
14602	3.3326	62.0194	73.7137
14603	3.3326	-3.3738	40.1946
14604	3.3327	98.2935	36.0529
14605	3.3328	73.9243	111.139
14606	3.3328	-3.6841	84.5081
14607	3.3329	77.0899	331.749
14608	3.333	81.3864	192.702
14609	3.333	59.7594	126.743
14610	3.333	-3.5573	33.8445
14611	3.3331	73.348	115.271
14612	3.3332	94.0423	203.225
14613	3.3336	67.7646	190.433
14614	3.3337	80.4371	121.218
14615	3.3337	89.8147	281.866
14616	3.3338	72.9434	232.362
14617	3.3338	84.5003	308.004
14618	3.334	84.298	345.028
14619	3.3342	99.8836	244.328
14620	3.3342	-2.8673	85.6278
14621	3.3344	80.573	94.5662
14622	3.3344	-2.7821	24.5204
14623	3.3344	89.0917	269.338
14624	3.3347	72.2051	339.831
14625	3.3347	-3.8918	24.9815
14626	3.3348	81.6092	96.431
14627	3.3348	71.6931	58.5407
14628	3.3348	72.0294	186.094
14629	3.3353	76.4704	126.801

14630	3.3353	73.0169	241.981
14631	3.3354	78.322	151.169
14632	3.3354	85.7338	202.85
14633	3.3356	-3.4292	249.183
14634	3.3356	76.363	30.9387
14635	3.3357	73.7361	130.324
14636	3.3357	-3.7294	291.171
14637	3.3357	64.7812	93.7965
14638	3.3358	76.221	142.657
14639	3.336	62.4905	167.2
14640	3.3361	-3.8913	347.535
14641	3.3362	66.0752	187.104
14642	3.3362	74.0063	314.117
14643	3.3363	94.5906	43.0746
14644	3.3363	71.8851	149.392
14645	3.3364	55.3156	114.929
14646	3.3364	81.5276	328.164
14647	3.3365	62.7665	258.365
14648	3.3367	103.338	207.456
14649	3.3368	68.4388	214.343
14650	3.337	69.8808	229.738
14651	3.3371	56.5804	72.4879
14652	3.3373	85.1163	225.757
14653	3.3375	88.2397	81.8907
14654	3.3375	-2.8676	148.562
14655	3.3377	69.9744	355.189
14656	3.3378	60.1587	165.174
14657	3.338	-3.5378	232.766
14658	3.3383	-2.3381	215.75
14659	3.3384	77.7564	126.873
14660	3.3387	64.6172	160.626
14661	3.3388	117.725	238.626
14662	3.3389	-2.9245	103.59
14663	3.339	-2.7609	277.091
14664	3.339	-3.074	236.301
14665	3.339	86.1306	269.743
14666	3.3391	77.6728	102.383
14667	3.3391	72.9373	75.6276
14668	3.3392	98.4881	75.488
14669	3.3393	72.0627	157.691
14670	3.3393	71.0473	294.474
14671	3.3394	-2.9863	142.343
14672	3.3394	95.5156	316.752
14673	3.3395	70.6562	277.654
14674	3.3396	69.6976	69.7619
14675	3.3396	70.4087	246.453
14676	3.3397	-4.1827	212.266
14677	3.3399	73.1573	145.808

14678	3.3399	66.3262	148.142
14679	3.34	67.2153	53.5481
14680	3.3402	64.281	197.173
14681	3.3403	73.0611	160.791
14682	3.3404	58.9661	135.099
14683	3.3405	77.3746	162.825
14684	3.3405	-2.9281	75.1124
14685	3.3405	75.8001	162.574
14686	3.3405	59.1564	122.461
14687	3.3406	73.5316	124.575
14688	3.3407	-2.6478	220.187
14689	3.3407	-2.995	96.8125
14690	3.3409	72.9008	150.695
14691	3.341	-3.3339	191.875
14692	3.341	66.8592	197.596
14693	3.3411	84.7015	256.916
14694	3.3415	72.5687	238.708
14695	3.3416	106.542	199.455
14696	3.3416	74.6198	50.4022
14697	3.3418	-3.1577	76.9105
14698	3.3418	63.0676	92.4225
14699	3.3419	80.6378	259.241
14700	3.3419	-4.4661	96.9427
14701	3.3419	-3.3186	262.031
14702	3.342	88.352	321.649
14703	3.342	104.094	214.557
14704	3.342	-2.8285	192.61
14705	3.3421	72.9936	44.1976
14706	3.3427	75.0483	126.934
14707	3.3428	79.3651	296.316
14708	3.3428	122.249	248.83
14709	3.3429	78.9666	77.6834
14710	3.3429	-3.0359	111.085
14711	3.3429	74.5063	100.873
14712	3.343	69.8444	181.168
14713	3.343	73.5565	20.4717
14714	3.3431	71.3335	117.697
14715	3.3431	-3.3982	273.402
14716	3.3432	87.531	30.0523
14717	3.3437	98.0463	244.414
14718	3.3438	68.4285	124.675
14719	3.3438	74.5542	51.6575
14720	3.3439	76.853	76.2755
14721	3.3439	74.9615	41.0981
14722	3.344	97.1744	53.8223
14723	3.3441	-2.9973	88.8687
14724	3.3441	48.613	187.27
14725	3.3441	91.933	210.348

14726	3.3443	105.659	45.7577
14727	3.3443	-3.1339	46.8691
14728	3.3444	70.3964	249.351
14729	3.3444	66.6721	69.1175
14730	3.3447	-2.5918	42.8682
14731	3.3451	78.2389	275.887
14732	3.3451	75.7384	308.87
14733	3.3453	59.0067	81.4991
14734	3.3454	69.9876	309.428
14735	3.3456	65.4843	155.071
14736	3.3456	87.6564	72.7297
14737	3.3457	89.0253	24.3436
14738	3.3457	69.1632	82.4771
14739	3.3458	77.2758	75.3477
14740	3.3461	-3.9871	78.2092
14741	3.3462	78.9022	276.211
14742	3.3463	90.3186	98.7885
14743	3.3463	65.4337	16.7648
14744	3.3463	72.1332	87.5229
14745	3.3463	69.6079	72.5378
14746	3.3465	80.7273	123.404
14747	3.3466	64.6303	133.107
14748	3.3469	-2.6788	154.384
14749	3.347	89.44	10.003
14750	3.3472	73.9227	243.615
14751	3.3473	72.6451	295.816
14752	3.3473	-3.0142	110.496
14753	3.3474	85.2095	261.255
14754	3.3474	79.6788	320.755
14755	3.3475	85.1438	20.9424
14756	3.3475	88.9031	212.181
14757	3.3477	94.2148	14.1119
14758	3.3477	63.0843	98.2393
14759	3.3477	102.663	45.7179
14760	3.3477	94.3654	24.5593
14761	3.348	-3.2893	318.309
14762	3.3481	-3.0043	260.87
14763	3.3481	89.0567	107.832
14764	3.3481	75.9107	119.185
14765	3.3485	82.8576	182.191
14766	3.3485	58.7572	197.704
14767	3.3486	69.2626	154.206
14768	3.3488	86.2853	349.806
14769	3.3488	72.3388	262.177
14770	3.3489	95.9469	209.18
14771	3.3489	73.8484	83.1813
14772	3.349	60.0914	131.719
14773	3.3491	52.8578	123.025

14774	3.3492	68.9121	301.731
14775	3.3495	62.361	144.439
14776	3.3496	71.958	45.5859
14777	3.3496	82.3349	233.384
14778	3.3496	-2.767	120.385
14779	3.3498	43.6149	28.5644
14780	3.3498	83.3382	83.6723
14781	3.3498	58.4684	191.575
14782	3.3499	93.1822	212.58
14783	3.3502	74.4113	168.88
14784	3.3504	82.7398	119.774
14785	3.3504	77.0638	218.558
14786	3.3506	63.1668	154.08
14787	3.3507	66.9539	316.046
14788	3.3508	5.0378	267.051
14789	3.351	70.9122	135.563
14790	3.3511	85.0232	355.633
14791	3.3511	61.7689	67.1974
14792	3.3513	63.0593	194.91
14793	3.3514	-2.9608	235.399
14794	3.3515	69.9724	95.6423
14795	3.3516	-2.8914	142.029
14796	3.3517	81.2666	358.744
14797	3.3517	64.4579	127.979
14798	3.3518	94.399	41.1559
14799	3.3518	81.5279	97.3206
14800	3.3518	99.3973	238.961
14801	3.3519	68.6905	354.763
14802	3.3519	82.0562	50.2884
14803	3.3522	-3.0423	346.672
14804	3.3522	66.3778	102.56
14805	3.3523	-2.6992	177.882
14806	3.3527	89.4214	40.7669
14807	3.3527	33.5956	128.857
14808	3.3528	97.4002	227.11
14809	3.3528	72.2422	120.875
14810	3.353	74.9362	116.711
14811	3.353	71.0427	180.47
14812	3.3531	81.5817	110.649
14813	3.3531	-1.3864	185.956
14814	3.3533	71.6141	300.146
14815	3.3535	-2.985	45.7158
14816	3.3535	86.6741	76.9523
14817	3.3535	79.4493	294.649
14818	3.3536	93.6715	93.0204
14819	3.3536	79.1111	305.39
14820	3.3537	78.8352	64.1616
14821	3.3539	69.1295	115.13

14822	3.354	69.6604	213.21
14823	3.354	69.2553	129.784
14824	3.354	64.3339	162.321
14825	3.354	66.1745	51.0162
14826	3.3541	94.4914	84.9152
14827	3.3542	78.3411	228.196
14828	3.3542	83.7613	50.3985
14829	3.3542	97.0624	118.239
14830	3.3542	88.4932	208.081
14831	3.3542	79.1632	134.572
14832	3.3542	-3.3022	278.12
14833	3.3543	98.8524	307.182
14834	3.3544	103.204	95.4998
14835	3.3545	80.4164	272.992
14836	3.3545	66.0376	143.885
14837	3.3547	73.8407	235.815
14838	3.3549	80.0654	231.935
14839	3.355	74.7905	190.952
14840	3.3551	66.2305	231.79
14841	3.3551	73.4257	269.188
14842	3.3552	73.948	65.339
14843	3.3552	-2.8632	90.2389
14844	3.3552	81.633	91.484
14845	3.3552	83.8345	237.83
14846	3.3552	84.5408	144.516
14847	3.3553	-2.7724	22.736
14848	3.3554	-3.96	262.636
14849	3.3555	86.4306	266.772
14850	3.3555	94.2051	267.601
14851	3.3555	-2.9565	259.845
14852	3.3556	-3.5522	49.4807
14853	3.3557	-3.5205	194.859
14854	3.3558	89.0476	59.6906
14855	3.3561	69.1729	160.188
14856	3.3562	77.3825	39.6699
14857	3.3562	76.7751	58.1706
14858	3.3562	-2.8183	339.636
14859	3.3562	5.8456	302.062
14860	3.3563	90.7756	318.916
14861	3.3563	88.5429	291.866
14862	3.3564	74.3275	260.288
14863	3.3564	78.4515	205.947
14864	3.3568	77.3921	201.845
14865	3.3569	87.1675	153.347
14866	3.357	67.6098	133.173
14867	3.357	71.431	125.081
14868	3.3571	128.18	319.662
14869	3.3572	83.2539	214.773

14870	3.3575	73.3931	322.972
14871	3.3577	-3.3124	47.1674
14872	3.3582	75.892	153.601
14873	3.3583	86.6892	4.3328
14874	3.3584	-2.992	56.9456
14875	3.3585	84.9996	279.224
14876	3.3585	85.544	52.6121
14877	3.3586	-3.1298	4.5456
14878	3.3587	94.2932	81.9924
14879	3.3587	79.5178	64.3843
14880	3.3588	-2.942	303.273
14881	3.3589	107.967	207.915
14882	3.359	75.8359	3.1851
14883	3.359	98.8265	81.489
14884	3.3591	79.883	150.201
14885	3.3591	71.4305	148.637
14886	3.3592	64.1071	179.519
14887	3.3592	82.2724	67.4649
14888	3.3593	94.5493	63.426
14889	3.3594	102.167	202.617
14890	3.3595	82.0473	161.214
14891	3.3599	84.3077	239.617
14892	3.36	63.341	111.7
14893	3.3601	78.9295	130.935
14894	3.3602	-3.0433	150.117
14895	3.3604	76.2803	158.359
14896	3.3606	74.07	300.015
14897	3.3608	60.7751	178.428
14898	3.3608	80.7607	237.018
14899	3.3608	78.041	262.011
14900	3.361	72.8529	69.7801
14901	3.3612	70.5361	260.336
14902	3.3613	88.214	255.991
14903	3.3613	-3.0495	6.0507
14904	3.3614	-3.0292	122.62
14905	3.3614	64.1846	176.143
14906	3.3614	85.0803	153.281
14907	3.3614	55.491	142.106
14908	3.3616	71.8031	122.402
14909	3.3616	-3.2362	178.21
14910	3.3616	74.8175	85.681
14911	3.3618	-3.4269	112.706
14912	3.3619	77.2543	232.439
14913	3.3622	-3.1154	139.12
14914	3.3622	65.2676	95.4812
14915	3.3624	-2.8976	71.712
14916	3.3626	80.3048	69.6217
14917	3.3626	-2.8177	142.227

14918	3.3626	81.5075	269.788
14919	3.3628	-3.9292	74.6138
14920	3.3629	101.744	22.3347
14921	3.3629	68.0005	82.2724
14922	3.3629	61.466	138.157
14923	3.363	77.8825	87.2737
14924	3.3631	77.4731	82.2971
14925	3.3631	-3.5465	358.83
14926	3.3632	79.0792	88.4881
14927	3.3632	81.7692	338.98
14928	3.3633	47.8049	157.086
14929	3.3635	95.4062	45.5757
14930	3.3635	94.1107	73.2255
14931	3.3635	-3.9457	251.599
14932	3.3635	78.876	82.2304
14933	3.3635	74.6288	320.923
14934	3.3638	79.1672	102.761
14935	3.3639	-2.6755	159.152
14936	3.364	83.9011	26.8808
14937	3.364	-4.1439	264.768
14938	3.364	69.0615	219.503
14939	3.364	-2.8873	18.3507
14940	3.3642	87.2311	214.926
14941	3.3643	-2.9948	36.2202
14942	3.3643	78.7399	144.565
14943	3.3643	70.7568	113.88
14944	3.3644	91.6183	249.372
14945	3.3644	67.1228	272.461
14946	3.3644	108.141	310.302
14947	3.3645	71.5996	129.413
14948	3.3646	68.7905	277.744
14949	3.3648	49.1614	278.059
14950	3.365	70.639	225.866
14951	3.3652	84.4484	319.081
14952	3.3652	-2.9148	39.5686
14953	3.3652	90.9445	14.8966
14954	3.3654	83.3321	123.898
14955	3.3654	80.9377	128.549
14956	3.3656	102.478	142.009
14957	3.3657	-2.7701	331.669
14958	3.3657	86.7044	7.9991
14959	3.3658	104.896	318.062
14960	3.3658	80.2485	110.915
14961	3.3659	83.4552	136.784
14962	3.366	-2.7768	114.961
14963	3.3661	75.8362	159.036
14964	3.3661	-2.6767	262.464
14965	3.3661	67.1426	128.355

14966	3.3661	-2.9191	236.181
14967	3.3661	73.2541	140.643
14968	3.3663	86.6111	15.3824
14969	3.3663	101.092	129.166
14970	3.3665	83.8848	262.197
14971	3.3665	71.8216	99.9634
14972	3.3666	80.1882	276.133
14973	3.3666	83.7172	224.818
14974	3.3666	68.4725	290.56
14975	3.3671	73.2583	111.777
14976	3.3671	66.3221	124.932
14977	3.3672	74.7834	201.713
14978	3.3673	75.6841	236.958
14979	3.3674	74.1144	167.027
14980	3.3674	103.152	220.493
14981	3.3675	77.0834	28.6817
14982	3.3676	82.1276	130.882
14983	3.3677	112.366	203.115
14984	3.3679	72.4266	148.702
14985	3.3679	82.0043	107.259
14986	3.3679	85.4354	173.362
14987	3.3679	85.8717	47.2119
14988	3.368	-4.1292	25.4478
14989	3.368	78.3932	154.083
14990	3.368	90.9425	83.6837
14991	3.3682	80.337	86.4043
14992	3.3682	94.6859	117.767
14993	3.3682	-3.9057	136.02
14994	3.3682	-4.0516	47.502
14995	3.3683	97.5926	213.116
14996	3.3683	46.3036	157.439
14997	3.3684	68.5571	247.759
14998	3.3684	73.9661	45.5539
14999	3.3684	82.8067	275.311
15000	3.3685	83.315	316.678
15001	3.3686	81.6697	239.322
15002	3.3686	-2.7893	5.9101
15003	3.3687	-0.9522	95.1114
15004	3.3688	72.7579	268.43
15005	3.3688	68.3539	249.133
15006	3.3689	72.0058	286.898
15007	3.3691	94.0909	201.338
15008	3.3692	73.7722	357.038
15009	3.3694	87.4905	138.683
15010	3.3695	77.3342	50.0228
15011	3.3696	84.036	104.024
15012	3.3696	67.4867	80.7162
15013	3.3697	98.9399	107.335

15014	3.3697	64.6884	189.166
15015	3.3697	62.7645	164.007
15016	3.3699	82.0158	68.2342
15017	3.3699	74.017	15.9522
15018	3.37	84.8943	279.654
15019	3.37	81.9244	201.213
15020	3.3702	80.1462	84.0636
15021	3.3703	95.3849	313.423
15022	3.3704	-2.9992	37.218
15023	3.3705	95.6689	123.346
15024	3.3705	-2.8691	314.937
15025	3.3706	72.8929	153.021
15026	3.3706	84.4744	130.28
15027	3.3706	68.9551	179.894
15028	3.3708	98.3321	243.273
15029	3.3708	76.0555	129.506
15030	3.3709	72.0824	59.1294
15031	3.3709	77.2303	231.568
15032	3.3709	27.9402	158.14
15033	3.3709	102.444	51.8195
15034	3.371	72.5721	286.926
15035	3.3711	90.2064	199.912
15036	3.3711	81.5795	146.278
15037	3.3712	76.2468	26.7048
15038	3.3712	82.0874	132.717
15039	3.3713	74.1565	218.947
15040	3.3714	67.9429	184.89
15041	3.3714	-2.8774	43.4739
15042	3.3715	-2.9579	152.554
15043	3.3716	118.505	74.5947
15044	3.3717	87.7045	316.338
15045	3.3718	60.1111	43.4284
15046	3.3719	-2.7839	308.219
15047	3.3721	69.7714	109.014
15048	3.3721	69.1576	11.181
15049	3.3723	71.7984	194.8
15050	3.3723	105.958	23.5117
15051	3.3724	78.4707	270.159
15052	3.3724	95.2789	218.976
15053	3.3724	73.2802	124.511
15054	3.3724	68.2847	130.286
15055	3.3725	71.9758	160.055
15056	3.3727	109.942	43.3322
15057	3.3729	99.3829	254.975
15058	3.3729	94.8155	214.108
15059	3.373	-2.8698	308.308
15060	3.373	68.6458	127.587
15061	3.3731	-4.1437	35.7852

15062	3.3732	-3.583	264.434
15063	3.3733	83.021	266.524
15064	3.3734	91.6172	167.587
15065	3.3734	88.0804	313.344
15066	3.3734	82.9147	4.3877
15067	3.3735	-3.7548	250.768
15068	3.3735	75.2384	128.72
15069	3.3735	57.7165	251.558
15070	3.3735	75.0581	75.1277
15071	3.3736	-2.8958	282.535
15072	3.3737	78.536	86.9569
15073	3.3737	102.68	5.5098
15074	3.3737	46.3527	184.751
15075	3.3739	98.311	27.2693
15076	3.374	77.2464	149.934
15077	3.374	120.296	279.21
15078	3.3742	103.555	313.042
15079	3.3744	107.978	13.8228
15080	3.3744	77.659	135.596
15081	3.3744	80.5475	358.653
15082	3.3744	63.4447	9.0101
15083	3.3746	78.771	136.063
15084	3.3747	63.5159	332.737
15085	3.3748	62.3009	186.681
15086	3.375	122.268	222.924
15087	3.3751	83.1272	327.858
15088	3.3753	80.8468	40.1173
15089	3.3753	77.3694	224.416
15090	3.3753	59.4213	114.44
15091	3.3754	76.3266	114.98
15092	3.3756	86.9778	271.705
15093	3.3759	101.62	26.2796
15094	3.3762	-4.2369	154.675
15095	3.3763	82.0398	128.863
15096	3.3763	84.2737	188.566
15097	3.3764	98.4506	38.5327
15098	3.3764	81.8097	124.091
15099	3.3766	81.3032	238.628
15100	3.3766	-2.9595	163.933
15101	3.3767	72.0814	266.811
15102	3.3767	75.5468	323.481
15103	3.3767	-2.6241	280.419
15104	3.3768	14.439	101.602
15105	3.3772	70.8494	101.025
15106	3.3777	79.6596	206.564
15107	3.3777	63.9102	302.404
15108	3.3778	67.6666	359.851
15109	3.378	-4.5031	263.622

15110	3.3781	87.1414	260.667
15111	3.3782	100.059	125.6
15112	3.3784	58.8263	190.313
15113	3.3785	64.3167	98.3239
15114	3.3788	63.2405	311.709
15115	3.3789	77.7302	92.1306
15116	3.3789	63.7354	265.33
15117	3.379	64.7074	156.295
15118	3.3791	82.2249	97.5195
15119	3.3792	66.7605	221.537
15120	3.3792	30.0018	83.4256
15121	3.3793	78.6914	164.848
15122	3.3794	70.1593	313.502
15123	3.3794	86.8148	108.246
15124	3.3795	30.1809	104.044
15125	3.3795	72.6703	146.163
15126	3.3795	75.7211	122.463
15127	3.3795	92.8073	333.598
15128	3.3795	77.3988	97.4963
15129	3.3797	70.2345	159.469
15130	3.3797	67.3086	336.357
15131	3.3798	95.3061	54.2385
15132	3.3798	96.4734	24.6644
15133	3.3798	71.1194	131.726
15134	3.3799	91.2873	167.333
15135	3.38	75.9802	98.5538
15136	3.3802	95.7895	229.05
15137	3.3803	95.9518	47.0776
15138	3.3803	-4.1362	233.278
15139	3.3804	-2.697	29.5213
15140	3.3804	-2.7939	315.806
15141	3.3806	72.0675	193.772
15142	3.3808	94.9162	207.579
15143	3.3811	69.0856	27.0063
15144	3.3814	82.825	136.005
15145	3.3815	86.8306	357.695
15146	3.3815	55.3572	21.1139
15147	3.3816	75.6672	29.9129
15148	3.3816	-3.3175	127.556
15149	3.3816	81.7213	153.995
15150	3.3817	84.7131	172.877
15151	3.3817	-2.6804	275.341
15152	3.3818	63.7511	281.376
15153	3.382	69.7728	69.3481
15154	3.3821	-2.876	32.2316
15155	3.3822	81.8097	66.1595
15156	3.3822	65.042	288.409
15157	3.3824	91.6826	353.289

15158	3.3825	73.1798	90.4582
15159	3.3825	-3.509	227.081
15160	3.3826	99.5145	12.8538
15161	3.3826	77.2791	168.852
15162	3.3826	-3.515	199.508
15163	3.3827	67.0322	8.1687
15164	3.3828	45.5402	101.767
15165	3.3828	92.3394	212.998
15166	3.3829	63.6045	128.587
15167	3.3829	82.8401	283.006
15168	3.383	92.0907	270.981
15169	3.383	72.6826	218.003
15170	3.383	-2.7121	358.026
15171	3.3831	70.0797	116.258
15172	3.3831	87.4521	28.6898
15173	3.3832	93.7167	259.472
15174	3.3832	88.6991	142.151
15175	3.3833	101.846	249.091
15176	3.3835	-2.7676	5.9471
15177	3.3836	61.7926	310.491
15178	3.3837	55.2325	15.0291
15179	3.3838	66.3868	334.443
15180	3.3838	95.476	91.3986
15181	3.3839	95.5637	81.8756
15182	3.384	78.7995	89.0812
15183	3.3841	69.386	114.302
15184	3.3841	77.659	172.623
15185	3.3841	68.0151	289.715
15186	3.3842	65.8414	170.931
15187	3.3842	73.1511	159.465
15188	3.3842	101.657	41.9443
15189	3.3844	89.5858	91.5691
15190	3.3845	66.315	192.794
15191	3.3846	88.4854	215.213
15192	3.3847	95.7841	294.621
15193	3.385	77.9656	183.669
15194	3.3851	96.6702	33.6396
15195	3.3853	78.1718	78.6965
15196	3.3853	77.1613	143.294
15197	3.3855	8.5596	227.854
15198	3.3855	68.3707	221.329
15199	3.3856	70.3033	175.221
15200	3.3857	85.8218	42.0307
15201	3.3857	96.7914	81.9012
15202	3.3857	79.5173	65.9498
15203	3.3859	63.2558	105.55
15204	3.386	-2.8457	70.9192
15205	3.3861	60.9473	128.257

15206	3.3861	65.8776	158.67
15207	3.3861	-3.86	197.485
15208	3.3863	-3.559	202.823
15209	3.3864	81.292	163.261
15210	3.3864	13.9687	315.551
15211	3.3865	64.6961	328.467
15212	3.3866	-3.1331	267.15
15213	3.3867	62.3257	109.164
15214	3.3867	77.7028	168.338
15215	3.3869	79.5639	259.184
15216	3.387	79.9689	144.104
15217	3.3871	82.0301	207.404
15218	3.3871	77.0893	160.505
15219	3.3872	78.4794	222.959
15220	3.3874	81.1155	174.721
15221	3.3874	96.5179	204.837
15222	3.3874	109.759	53.5114
15223	3.3876	95.3906	123.543
15224	3.3877	79.9436	27.9814
15225	3.3879	-3.0186	90.432
15226	3.388	93.7545	113.218
15227	3.388	82.0965	205.279
15228	3.3881	-4.0257	267.651
15229	3.3882	100.234	69.7483
15230	3.3882	84.7334	239.11
15231	3.3883	77.2644	168.005
15232	3.3884	78.1118	127.756
15233	3.3884	71.767	117.71
15234	3.3885	-3.0229	234.538
15235	3.3885	-2.9065	285.026
15236	3.3887	78.9821	78.6212
15237	3.3888	85.7086	321.553
15238	3.3888	79.8464	69.5408
15239	3.3888	77.6252	161.46
15240	3.3888	74.9584	127.148
15241	3.3889	92.8507	261.057
15242	3.389	101.972	306.272
15243	3.3891	113.277	58.6672
15244	3.3893	114.192	92.2563
15245	3.3895	85.2725	142.024
15246	3.3897	-2.7551	6.6282
15247	3.3898	77.4259	161.04
15248	3.3899	-3.9753	202.907
15249	3.39	67.1404	103.858
15250	3.3902	-3.3907	82.2317
15251	3.3902	78.7366	309.806
15252	3.3902	78.4733	93.9558
15253	3.3902	-2.8479	314.897

15254	3.3902	91.1068	139.376
15255	3.3903	84.3081	98.8901
15256	3.3906	38.2168	351.708
15257	3.3906	80.2747	198.78
15258	3.3907	92.5894	310.969
15259	3.3909	88.7844	151.176
15260	3.3909	83.2701	299.728
15261	3.3909	79.7714	21.0309
15262	3.391	-3.0407	310.949
15263	3.3911	73.8408	225.032
15264	3.3912	75.1008	328.903
15265	3.3912	-2.789	44.4844
15266	3.3914	76.4694	260.837
15267	3.3914	85.6356	275.558
15268	3.3915	-3.2286	124.986
15269	3.3915	74.2926	163.116
15270	3.3916	97.2276	202.483
15271	3.3917	-2.7095	34.1106
15272	3.3918	13.375	151.216
15273	3.3919	65.6131	113.648
15274	3.3923	96.0297	289.9
15275	3.3923	85.7194	105.603
15276	3.3923	87.0397	8.551
15277	3.3924	70.5474	122.211
15278	3.3925	69.6565	309.347
15279	3.3928	70.1339	141.511
15280	3.393	111.904	249.861
15281	3.393	73.3691	121.517
15282	3.3933	91.8226	49.5466
15283	3.3933	62.0147	280.774
15284	3.3933	81.0629	238.057
15285	3.3934	81.8318	222.099
15286	3.3934	18.131	72.958
15287	3.3935	69.5554	247.815
15288	3.3935	79.9897	336.063
15289	3.3938	60.9046	143.119
15290	3.394	-2.9941	99.0026
15291	3.394	82.407	127.638
15292	3.3944	90.0191	130.308
15293	3.3945	78.8469	84.8096
15294	3.3946	90.9261	94.3594
15295	3.3946	82.742	74.3801
15296	3.3947	76.3033	177.195
15297	3.3947	85.4174	51.5615
15298	3.3948	53.2243	202.116
15299	3.3948	-2.7994	198.083
15300	3.3948	93.2137	121.644
15301	3.3952	79.1925	211.219

15302	3.3954	126.968	204.175
15303	3.3955	-3.4642	253.866
15304	3.3955	61.4861	119.751
15305	3.3955	86.8253	282.771
15306	3.3956	90.0858	302.149
15307	3.3956	-3.6642	42.0153
15308	3.3957	-2.7586	301.287
15309	3.3958	-3.3101	228.564
15310	3.3958	55.8498	172.667
15311	3.396	60.201	22.1065
15312	3.3963	90.8281	314.084
15313	3.3963	105.64	209.518
15314	3.3964	100.421	239.406
15315	3.3964	84.1118	97.825
15316	3.3967	75.2524	28.0447
15317	3.3969	87.7422	166.803
15318	3.3969	82.1931	328.473
15319	3.397	91.4891	79.4446
15320	3.397	85.3181	82.9728
15321	3.3971	-3.2648	31.0153
15322	3.3972	-2.9278	163.279
15323	3.3972	62.1422	183.375
15324	3.3974	91.8431	233.067
15325	3.3976	83.2098	100.559
15326	3.3976	100.554	39.3067
15327	3.3976	106.333	157.253
15328	3.3981	83.8659	71.2836
15329	3.3982	-3.1977	126.544
15330	3.3982	77.9325	251.622
15331	3.3983	59.6608	73.7906
15332	3.3984	91.123	311.026
15333	3.3984	81.8573	9.049
15334	3.3985	85.9126	156.531
15335	3.3985	67.8307	204.283
15336	3.3986	-3.0092	145.218
15337	3.3987	61.7577	167.872
15338	3.3987	90.7935	82.6651
15339	3.3988	87.8409	111.366
15340	3.3988	83.8194	14.1036
15341	3.3988	-3.9668	116.368
15342	3.3989	89.5985	17.18
15343	3.399	73.1813	270.332
15344	3.3991	105.346	54.563
15345	3.3991	73.2213	179.076
15346	3.3991	73.4341	151.131
15347	3.3991	61.8371	209.811
15348	3.3992	76.4779	234.391
15349	3.3993	54.4656	160.962

15350	3.3994	-3.1868	262.026
15351	3.3997	68.572	285.046
15352	3.3998	88.3601	70.2793
15353	3.3998	66.609	176.487
15354	3.3999	-3.8867	37.662
15355	3.4	77.0639	166.224
15356	3.4	88.0863	37.8244
15357	3.4	79.5585	84.0919
15358	3.4	71.5042	265.478
15359	3.4002	76.1792	278.148
15360	3.4002	83.7383	139.38
15361	3.4003	74.1112	282.558
15362	3.4003	87.9616	82.8057
15363	3.4005	92.6609	272.285
15364	3.4005	-2.8273	303.075
15365	3.4007	-2.825	309.461
15366	3.4008	88.2574	71.9934
15367	3.4009	67.2064	177.939
15368	3.401	63.95	83.587
15369	3.4011	86.2527	154.535
15370	3.4012	105.902	31.0134
15371	3.4012	46.5866	34.9078
15372	3.4012	97.3541	211.125
15373	3.4014	78.0926	227.903
15374	3.4014	79.3169	336.022
15375	3.4015	81.4962	119.903
15376	3.4015	63.2298	24.8005
15377	3.4017	79.9956	217.136
15378	3.4018	-3.1331	282.625
15379	3.4018	88.315	131.484
15380	3.4023	84.3874	0.6394
15381	3.4023	67.6231	137.891
15382	3.4023	99.25	287.218
15383	3.4024	99.8189	116.103
15384	3.4026	95.2002	355.168
15385	3.4026	-3.0254	127.815
15386	3.4027	80.3901	61.7475
15387	3.4028	-4.0419	185.13
15388	3.4029	111.268	3.0568
15389	3.4029	86.5071	130.702
15390	3.4029	75.6164	288.574
15391	3.4029	62.9967	128.805
15392	3.403	94.3027	314.042
15393	3.403	90.493	284.339
15394	3.403	101.11	32.1527
15395	3.4031	73.4743	91.0585
15396	3.4032	107.425	271.831
15397	3.4032	39.7652	138.531

15398	3.4034	-2.692	27.7703
15399	3.4036	91.7124	68.2829
15400	3.4036	92.4968	239.626
15401	3.4036	57.8983	333.106
15402	3.4038	65.8317	145.588
15403	3.4039	70.5018	44.604
15404	3.404	62.5347	18.9117
15405	3.404	95.6329	242.505
15406	3.404	89.2677	102.049
15407	3.4042	78.1353	98.3116
15408	3.4044	92.7023	51.9282
15409	3.4044	97.9841	42.2811
15410	3.4045	78.2103	334.582
15411	3.4046	76.5783	41.4082
15412	3.4046	76.7757	146.808
15413	3.4049	81.1256	135.034
15414	3.4049	-3.5189	43.9182
15415	3.405	74.6094	92.0291
15416	3.405	80.8173	148.104
15417	3.4051	77.7816	234.554
15418	3.4051	84.774	274.613
15419	3.4054	88.4986	341.634
15420	3.4056	65.8374	196.069
15421	3.4058	83.8703	108.308
15422	3.4059	79.8897	4.3964
15423	3.4059	75.2549	257.403
15424	3.4059	80.06	288.896
15425	3.406	89.9696	151.846
15426	3.406	77.0023	121.869
15427	3.4061	95.1395	227.201
15428	3.4063	-2.8577	44.4387
15429	3.4065	83.1339	132.93
15430	3.4065	87.9083	49.3754
15431	3.4066	81.6402	77.2298
15432	3.4067	-2.7805	130.676
15433	3.4068	64.5938	116.803
15434	3.407	-2.7336	340.49
15435	3.4072	112.532	43.1861
15436	3.4072	-2.8231	343.711
15437	3.4072	80.8444	353.417
15438	3.4072	82.4195	235.505
15439	3.4073	-2.8761	138.379
15440	3.4073	81.304	196.454
15441	3.4073	104.208	315.616
15442	3.4074	83.187	121.266
15443	3.4075	80.6855	85.1816
15444	3.4077	88.0328	114.032
15445	3.4078	70.874	245.094

15446	3.4078	-2.7629	304.266
15447	3.4079	68.9366	139.113
15448	3.408	-2.8177	5.8828
15449	3.4081	80.9287	353.693
15450	3.4081	76.9488	138.522
15451	3.4084	93.2416	152.422
15452	3.4085	88.7469	242.102
15453	3.4085	121.249	318.378
15454	3.4085	87.1129	241.192
15455	3.4086	-3.1647	343.339
15456	3.4087	85.4321	127.599
15457	3.4087	96.5947	259.734
15458	3.4088	72.3707	90.9278
15459	3.4091	-3.6513	38.4071
15460	3.4093	-2.8715	78.0069
15461	3.4093	69.8819	228.857
15462	3.4095	64.7569	334.155
15463	3.4097	78.9594	193.249
15464	3.4099	112.575	42.8383
15465	3.41	75.4726	248.965
15466	3.41	93.601	123.017
15467	3.4101	113.855	315.049
15468	3.4102	67.4278	4.1837
15469	3.4102	76.241	216.513
15470	3.4102	73.7553	275.878
15471	3.4103	95.8732	30.7115
15472	3.4104	83.1082	74.335
15473	3.4107	77.2617	248.239
15474	3.4107	64.0583	178.186
15475	3.4107	-2.727	344.925
15476	3.4107	84.5061	142.342
15477	3.4107	81.2777	227.612
15478	3.4109	82.1234	190.827
15479	3.4112	86.72	69.3365
15480	3.4112	98.459	207.23
15481	3.4115	92.2287	204.13
15482	3.4115	84.0367	53.7682
15483	3.4117	117.746	196.368
15484	3.4118	89.9266	312.151
15485	3.4118	67.7727	116.491
15486	3.4118	80.3857	200.292
15487	3.4118	90.6244	211.18
15488	3.412	98.7445	213.235
15489	3.412	68.0646	25.8815
15490	3.4121	108.892	47.1993
15491	3.4123	92.0673	198.879
15492	3.4124	75.3996	249.847
15493	3.4124	98.9114	33.3063

15494	3.4124	49.2301	2.9717
15495	3.4125	62.6311	146.269
15496	3.4125	79.4767	66.0432
15497	3.4127	-2.8662	309.317
15498	3.4128	84.3534	95.4035
15499	3.4128	64.999	198.922
15500	3.4129	96.9835	263.406
15501	3.4132	77.0325	14.8864
15502	3.4132	70.1821	317.212
15503	3.4135	106.596	283.888
15504	3.4136	-3.2512	45.0924
15505	3.4136	87.8521	87.4546
15506	3.4136	65.7541	292.904
15507	3.4137	71.0976	58.0515
15508	3.4138	106.615	124.952
15509	3.4138	84.8638	58.7394
15510	3.4139	73.9515	13.6453
15511	3.4141	-2.8171	189.622
15512	3.4141	-3.2224	355.245
15513	3.4142	79.6518	173.728
15514	3.4142	79.5248	168.658
15515	3.4143	106.186	43.8731
15516	3.4145	-2.7782	306.506
15517	3.4145	95.1988	8.7498
15518	3.4146	115.883	298.441
15519	3.4147	91.553	145.692
15520	3.4147	81.4187	187.93
15521	3.4148	87.8112	134.862
15522	3.4148	75.2574	131.455
15523	3.4148	8.2145	27.5492
15524	3.4148	84.2074	40.9738
15525	3.4149	98.6338	150.085
15526	3.4149	81.8628	90.9463
15527	3.415	73.9604	16.1592
15528	3.4152	111.679	39.4313
15529	3.4156	99.2595	74.1628
15530	3.4157	97.7844	206.121
15531	3.4159	70.314	223.447
15532	3.4159	87.2995	272.583
15533	3.416	67.6697	97.2675
15534	3.4162	73.6357	325.188
15535	3.4162	82.1227	123.408
15536	3.4163	76.1731	18.6093
15537	3.4164	67.906	130.902
15538	3.4165	104.089	35.9808
15539	3.4165	85.8607	105.896
15540	3.4165	89.6284	215.56
15541	3.4168	89.9983	111.34

15542	3.4169	67.9045	68.0755
15543	3.417	106.34	206.625
15544	3.4171	68.0145	188.434
15545	3.4171	94.2256	197.534
15546	3.4171	91.002	75.3234
15547	3.4171	-2.817	193.338
15548	3.4171	1.6633	167.602
15549	3.4173	-3.8356	131.067
15550	3.4173	92.8054	88.1725
15551	3.4173	85.4417	180.825
15552	3.4174	104.496	197.088
15553	3.4176	102.271	113.449
15554	3.4176	69.6702	189.733
15555	3.4177	62.7647	277.565
15556	3.4179	98.903	214.006
15557	3.4179	90.4766	201.016
15558	3.4179	77.7124	196.093
15559	3.418	75.0685	149.562
15560	3.4181	77.6661	263.727
15561	3.4182	92.5576	34.7523
15562	3.4185	102.451	30.7831
15563	3.4185	79.9403	178.708
15564	3.4185	71.7599	305.169
15565	3.4185	73.1036	65.751
15566	3.4188	81.9098	231.8
15567	3.4189	87.997	165.677
15568	3.419	127.68	247.097
15569	3.419	81.2446	99.3451
15570	3.419	83.0326	141.109
15571	3.4191	54.4408	166.066
15572	3.4191	62.8456	223.991
15573	3.4192	82.7712	347.931
15574	3.4193	-2.8775	99.7264
15575	3.4193	75.8416	93.5528
15576	3.4193	103.954	57.1547
15577	3.4195	69.6619	219.895
15578	3.4195	69.6843	204.542
15579	3.4195	83.1405	218.162
15580	3.4195	-3.4375	119.197
15581	3.4196	-3.2504	273.089
15582	3.4197	98.7754	74.7597
15583	3.4197	86.4244	31.8666
15584	3.4197	77.8384	127.456
15585	3.4199	94.2427	264.182
15586	3.4199	-2.7865	5.422
15587	3.42	85.4043	257.349
15588	3.42	90.6217	31.1894
15589	3.4201	93.5297	99.485

15590	3.4203	124.141	111.388
15591	3.4203	97.3196	211.548
15592	3.4204	94.0339	355.859
15593	3.4205	94.9858	68.5868
15594	3.4206	82.2413	176.032
15595	3.4208	96.5458	62.4224
15596	3.4208	74.98	129.131
15597	3.4208	68.6033	130.529
15598	3.4208	-2.9856	281.823
15599	3.4209	-2.9673	266.865
15600	3.4209	82.9034	35.614
15601	3.421	-3.3898	61.8425
15602	3.4211	65.6778	150.389
15603	3.4214	94.3515	184.259
15604	3.4214	79.8105	99.0421
15605	3.4215	77.2741	101.771
15606	3.4217	93.167	136.584
15607	3.4217	64.3233	143.346
15608	3.4218	97.906	267.538
15609	3.4218	76.4597	219.003
15610	3.4219	82.0959	334.505
15611	3.4219	75.145	115.031
15612	3.4219	81.7893	114.75
15613	3.4223	-4.2616	56.2342
15614	3.4223	68.8891	151.638
15615	3.4223	69.1844	5.3963
15616	3.4223	76.2951	165.872
15617	3.4224	55.6979	209.778
15618	3.4226	84.6809	106.15
15619	3.4227	95.1971	153.852
15620	3.4228	91.2941	245.321
15621	3.4229	80.3045	107.512
15622	3.423	-1.0028	66.0169
15623	3.4233	101.24	250.873
15624	3.4233	-2.7277	333.836
15625	3.4233	95.0149	20.7562
15626	3.4235	92.0699	100.584
15627	3.4235	97.4328	237.76
15628	3.424	77.6953	147.346
15629	3.4241	81.5764	116.133
15630	3.4241	77.5607	228.246
15631	3.4242	81.1947	101.721
15632	3.4242	78.2516	152.225
15633	3.4243	68.2323	4.8385
15634	3.4243	69.4413	217.13
15635	3.4244	106.726	41.6694
15636	3.4244	81.9642	290.488
15637	3.4245	82.95	113.832

15638	3.4246	87.9475	256.04
15639	3.4246	74.8453	122.227
15640	3.4246	100.274	20.912
15641	3.4247	81.7709	246.767
15642	3.4248	85.7927	125.457
15643	3.4248	83.4832	264.512
15644	3.4249	71.6932	93.7334
15645	3.425	89.3173	57.3517
15646	3.4252	-2.948	163.65
15647	3.4252	71.8502	23.2932
15648	3.4253	69.487	121.056
15649	3.4253	85.6311	54.7956
15650	3.4254	119.246	27.628
15651	3.4254	80.8965	148.457
15652	3.4254	81.2958	117.965
15653	3.4256	92.3362	154.568
15654	3.4256	82.8465	255.063
15655	3.4256	-2.7742	93.239
15656	3.4257	87.7587	230.528
15657	3.4257	65.9399	175.651
15658	3.4258	86.2498	33.3837
15659	3.4258	86.9305	25.1122
15660	3.4259	68.1068	303.633
15661	3.4259	76.768	173.657
15662	3.426	-3.7567	109.273
15663	3.426	97.2245	139.187
15664	3.4262	82.0836	204.517
15665	3.4262	76.7493	130.819
15666	3.4263	-3.9818	346.503
15667	3.4263	95.3257	287.115
15668	3.4264	97.1683	53.4407
15669	3.4264	75.7747	130.094
15670	3.4267	102.49	304.277
15671	3.4269	102.517	51.2728
15672	3.4269	-2.742	18.0287
15673	3.4272	111.173	356.361
15674	3.4272	84.9099	243.942
15675	3.4272	74.5595	4.801
15676	3.4273	77.492	215.953
15677	3.4273	86.5126	122.858
15678	3.4274	73.2739	93.9211
15679	3.4274	71.7065	345.27
15680	3.4276	87.8196	299.57
15681	3.4278	89.0037	121.428
15682	3.4278	73.6574	123.374
15683	3.4279	112.785	51.643
15684	3.4279	28.7697	171.581
15685	3.428	90.8339	169.367

15686	3.428	68.5377	104.486
15687	3.4281	75.1577	239.948
15688	3.4282	64.6386	136.998
15689	3.4283	89.4655	105.352
15690	3.4283	82.7681	256.938
15691	3.4284	85.5918	72.176
15692	3.4285	91.2625	199.465
15693	3.4285	80.7892	111.232
15694	3.4286	85.2121	237.471
15695	3.4287	55.4472	331.421
15696	3.4288	-3.838	56.2062
15697	3.4289	-3.9673	191.72
15698	3.4289	82.0235	70.5851
15699	3.429	73.8	209.95
15700	3.429	-4.0967	50.839
15701	3.4292	89.6923	164.536
15702	3.4292	60.3864	156.216
15703	3.4293	100.184	238.789
15704	3.4293	97.3262	145.555
15705	3.4294	-2.893	251.731
15706	3.4294	86.6317	82.7185
15707	3.4295	98.2118	313.68
15708	3.4295	63.7042	171.819
15709	3.4296	93.1403	78.4688
15710	3.4297	76.3804	182.804
15711	3.4297	89.2861	91.0235
15712	3.4298	79.155	69.5138
15713	3.4298	66.4778	327.1
15714	3.4299	-3.5253	91.344
15715	3.43	75.6178	135.472
15716	3.43	77.2557	96.6639
15717	3.4301	91.9653	30.6693
15718	3.4301	73.9148	47.7126
15719	3.4303	91.4016	206.598
15720	3.4304	88.4563	133.263
15721	3.4304	61.9448	165.689
15722	3.4304	98.4872	347.517
15723	3.4305	77.6623	104.8
15724	3.4306	73.7755	135.985
15725	3.4307	59.7818	248.869
15726	3.4308	75.1851	306.727
15727	3.4309	76.4324	154.655
15728	3.4309	107.295	83.6411
15729	3.431	85.31	34.6552
15730	3.4311	108.467	35.7072
15731	3.4312	80.2026	127.843
15732	3.4312	91.1528	63.915
15733	3.4312	82.444	123.238

15734	3.4314	90.8743	262.569
15735	3.4314	-4.1536	302.721
15736	3.4315	84.1767	143.549
15737	3.4315	88.1045	85.9002
15738	3.4317	99.2571	340.682
15739	3.4317	95.9063	53.9503
15740	3.4317	75.8663	198.31
15741	3.4317	84.5577	220.95
15742	3.4318	103.594	215.058
15743	3.4318	77.4011	97.4204
15744	3.4318	-3.2384	157.804
15745	3.4319	109.179	55.96
15746	3.4319	99.43	102.672
15747	3.4324	81.1385	257.176
15748	3.4325	79.6313	290.358
15749	3.4326	67.4718	164.701
15750	3.4326	87.5984	229.073
15751	3.4326	-2.9625	180.686
15752	3.4328	72.2241	228.42
15753	3.4328	64.369	123.914
15754	3.4329	118.504	306.346
15755	3.4329	68.4884	312.679
15756	3.433	78.2557	20.0602
15757	3.433	73.2388	120.689
15758	3.4331	91.9922	70.3187
15759	3.4333	87.352	36.7406
15760	3.4334	-2.9823	36.8638
15761	3.4335	74.8142	129.833
15762	3.4336	82.5317	231.113
15763	3.4336	94.2217	34.2543
15764	3.4337	86.0248	162.304
15765	3.4337	6.87	357.782
15766	3.4337	77.3809	152.465
15767	3.4338	83.8599	85.9944
15768	3.4339	83.3633	133.969
15769	3.4339	83.4814	106.675
15770	3.434	60.8738	158.395
15771	3.4342	78.7642	186.772
15772	3.4344	-3.5019	115.946
15773	3.4344	-3.3808	321.288
15774	3.4346	92.8996	232.065
15775	3.4348	84.6307	165.822
15776	3.4349	88.5354	347.916
15777	3.435	88.1063	64.7869
15778	3.435	83.8365	44.3799
15779	3.435	92.1645	355.658
15780	3.4351	87.4233	85.1952
15781	3.4351	-3.4771	159.919

15782	3.4352	2.6505	324.698
15783	3.4352	91.7998	319.617
15784	3.4352	91.7987	332.126
15785	3.4353	95.86	88.6136
15786	3.4353	78.4613	81.5557
15787	3.4353	74.9453	174.192
15788	3.4354	85.7319	205.746
15789	3.4354	-3.0132	56.3113
15790	3.4354	85.1111	288.395
15791	3.4355	89.2779	10.9235
15792	3.4356	81.4239	248.457
15793	3.4357	118.348	246.341
15794	3.4358	83.1565	270.352
15795	3.4358	112.272	313.506
15796	3.4359	82.156	69.1034
15797	3.436	99.3204	94.9048
15798	3.436	97.6641	290.38
15799	3.4362	94.2121	49.3251
15800	3.4362	99.3021	85.8577
15801	3.4363	83.3001	26.8135
15802	3.4363	90.4121	73.607
15803	3.4364	121.821	24.1035
15804	3.4365	57.7287	188.6
15805	3.4365	69.1295	306.471
15806	3.4366	78.9094	29.6175
15807	3.4368	80.0589	188.857
15808	3.4369	67.7676	315.28
15809	3.4369	86.8318	59.2305
15810	3.437	79.5936	181.229
15811	3.437	100.769	64.4887
15812	3.4371	78.255	115.752
15813	3.4373	68.6117	262.794
15814	3.4373	78.2988	176.65
15815	3.4373	79.082	72.8612
15816	3.4374	83.1453	278.912
15817	3.4374	86.1986	175.258
15818	3.4375	97.3828	68.3702
15819	3.4375	92.9375	107.153
15820	3.4376	99.5164	246.977
15821	3.4376	82.4511	318.631
15822	3.4377	88.9493	197.922
15823	3.4378	80.5445	305.654
15824	3.4378	-2.671	162.056
15825	3.4378	69.5161	286.017
15826	3.4378	87.1932	109.193
15827	3.4379	86.0061	152.22
15828	3.4381	66.1326	266.953
15829	3.4382	-2.8687	164.342

15830	3.4382	83.5746	131.961
15831	3.4382	11.0927	44.5681
15832	3.4383	87.7596	198.555
15833	3.4383	98.3686	127.093
15834	3.4384	85.6254	126.937
15835	3.4386	64.2626	87.0211
15836	3.4386	84.7694	195.702
15837	3.4386	97.941	290.137
15838	3.4387	95.0841	289.255
15839	3.4388	116.454	206.841
15840	3.4388	80.5044	298.808
15841	3.4389	74.3462	268.588
15842	3.439	96.6878	110.987
15843	3.439	79.6772	192.132
15844	3.439	6.2946	116.468
15845	3.4391	72.5075	137.675
15846	3.4392	77.0264	113.247
15847	3.4393	109.778	310.236
15848	3.4394	77.2549	88.2517
15849	3.4395	-2.9104	161.273
15850	3.4395	77.1767	34.8225
15851	3.4396	85.1869	127.328
15852	3.4397	80.8602	246.222
15853	3.4399	68.3907	348.873
15854	3.4399	77.9896	99.863
15855	3.44	-2.8681	198.837
15856	3.44	105.748	247.726
15857	3.4401	123.342	117.071
15858	3.4401	85.3568	164.694
15859	3.4405	78.6279	21.5237
15860	3.4406	71.7638	172.969
15861	3.4407	85.9598	108.191
15862	3.4407	-2.8403	323.889
15863	3.4407	-3.1665	323.996
15864	3.4411	-2.6587	142.663
15865	3.4412	-3.0455	96.1022
15866	3.4413	-2.9695	325.697
15867	3.4415	85.6137	170.631
15868	3.4415	94.6772	79.5911
15869	3.4416	-2.7058	34.6439
15870	3.4417	78.6944	111.737
15871	3.4418	92.2129	117.976
15872	3.4418	-3.9883	89.7421
15873	3.4418	86.834	269.11
15874	3.4419	86.4261	178.161
15875	3.4419	-2.7949	114.523
15876	3.442	104.302	129.792
15877	3.442	80.2788	41.6151

15878	3.442	87.1266	131.488
15879	3.442	-2.7864	306.408
15880	3.4421	98.893	215.286
15881	3.4422	74.1143	114.507
15882	3.4422	74.8227	295.939
15883	3.4423	77.8451	101.657
15884	3.4423	99.3405	66.217
15885	3.4424	68.8596	344.05
15886	3.4424	64.0508	191.334
15887	3.4424	82.1667	114.137
15888	3.4426	88.9832	113.337
15889	3.4429	106.488	90.5764
15890	3.443	90.9259	53.7511
15891	3.443	77.1422	300.922
15892	3.4431	73.9254	64.1735
15893	3.4431	55.0559	327.239
15894	3.4432	79.1924	226.758
15895	3.4432	93.2647	99.1895
15896	3.4433	-2.9309	132.35
15897	3.4434	73.6387	165.584
15898	3.4434	81.4839	326.959
15899	3.4434	81.0903	150.765
15900	3.4434	82.8818	228.763
15901	3.4437	93.2717	345.788
15902	3.4437	89.2771	232.044
15903	3.4441	90.1915	141.979
15904	3.4443	93.8078	87.3505
15905	3.4444	98.7331	194.376
15906	3.4444	19.7341	170.651
15907	3.4445	83.3617	1.1231
15908	3.4447	75.579	7.0721
15909	3.4448	71.8485	160.584
15910	3.4449	87.8338	231.599
15911	3.4449	104.757	95.547
15912	3.4449	67.2622	298.108
15913	3.445	70.2827	242.065
15914	3.4451	73.0032	61.73
15915	3.4453	93.548	149.263
15916	3.4455	85.6272	280.75
15917	3.4455	74.4571	165.96
15918	3.4455	61.7533	155.198
15919	3.4456	100.381	271.863
15920	3.4456	104.875	229.725
15921	3.4457	79.1014	223.593
15922	3.4457	6.225	78.0606
15923	3.4458	105.873	47.528
15924	3.4459	108.244	194.751
15925	3.4459	80.2469	112.828

15926	3.4459	71.5296	102.358
15927	3.4461	85.3959	210.103
15928	3.4463	118.002	116.459
15929	3.4463	-4.0025	19.0552
15930	3.4463	96.9254	290.648
15931	3.4464	82.5509	185.905
15932	3.4464	-3.5645	198.936
15933	3.4465	91.5672	173.177
15934	3.4465	71.8453	121.849
15935	3.4466	97.9665	168.947
15936	3.4466	-3.5249	147.953
15937	3.4467	79.6856	136.54
15938	3.4468	82.7959	267.57
15939	3.4468	80.1689	73.7648
15940	3.4469	99.4001	124.732
15941	3.4473	75.537	171.655
15942	3.4473	103.087	198.506
15943	3.4473	68.9005	122.413
15944	3.4473	86.2331	280.736
15945	3.4474	-4.9286	147.492
15946	3.4474	102.613	303.373
15947	3.4474	81.9112	10.9242
15948	3.4474	81.4842	143.199
15949	3.4474	77.1598	153.077
15950	3.4474	-3.031	112.081
15951	3.4474	87.0225	200.751
15952	3.4475	83.7543	100.173
15953	3.4475	92.8199	222.667
15954	3.4475	71.4237	117.536
15955	3.4476	102.388	85.5153
15956	3.4476	78.9768	207.988
15957	3.4477	91.3805	77.0267
15958	3.4478	91.0581	272.287
15959	3.4479	75.8101	348.846
15960	3.4481	88.2952	126.1
15961	3.4482	-3.1866	76.8221
15962	3.4482	83.7193	78.6224
15963	3.4483	90.5715	69.7249
15964	3.4483	85.9807	104.059
15965	3.4484	84.4063	300.259
15966	3.4487	108.054	84.6508
15967	3.4487	64.3103	97.2164
15968	3.4487	94.5215	10.2238
15969	3.4487	102.237	235.644
15970	3.4489	106.281	42.4955
15971	3.4489	95.7248	293.569
15972	3.449	104.286	83.481
15973	3.449	81.2977	141.086

15974	3.4491	85.3372	267.217
15975	3.4492	99.35	79.0336
15976	3.4493	80.0401	153.91
15977	3.4494	73.1812	138.771
15978	3.4495	95.2232	242.772
15979	3.4495	88.8624	136.201
15980	3.4495	79.527	155.656
15981	3.4495	87.3202	67.0621
15982	3.4498	79.176	334.089
15983	3.45	-2.7146	314.707
15984	3.4501	-4.1957	17.051
15985	3.4501	-3.8719	254.11
15986	3.4502	-3.8568	121.597
15987	3.4502	90.4031	248.058
15988	3.4504	81.4274	251.299
15989	3.4505	72.2344	5.3928
15990	3.4505	94.9143	194.243
15991	3.4506	-4.1582	242.524
15992	3.4506	-2.6966	357.768
15993	3.4507	96.2875	260.585
15994	3.4508	83.8103	195.89
15995	3.4508	95.5472	44.7537
15996	3.4509	81.7259	0.8051
15997	3.4509	88.0429	129.961
15998	3.4511	112.427	134.183
15999	3.4511	99.439	111.004
16000	3.4512	-2.9143	257.706
16001	3.4513	107	218.389
16002	3.4514	82.4799	263.439
16003	3.4515	67.6572	63.917
16004	3.4515	70.8414	291.654
16005	3.4517	84.3774	173.168
16006	3.4517	94.6539	196.347
16007	3.4519	82.383	89.158
16008	3.4519	82.9457	98.2608
16009	3.452	106.554	132.259
16010	3.452	90.6404	304.206
16011	3.4522	-4.223	309.916
16012	3.4523	106.049	307.817
16013	3.4523	93.3227	17.3307
16014	3.4523	98.2094	274.632
16015	3.4525	84.4309	41.5635
16016	3.4526	72.7194	66.0154
16017	3.4527	94.5578	173.918
16018	3.4527	82.2925	120.163
16019	3.4527	77.9119	149.723
16020	3.4529	84.1985	269.792
16021	3.453	91.4186	73.2678

16022	3.453	89.2333	69.7029
16023	3.4531	76.2851	160.801
16024	3.4532	91.3442	69.63
16025	3.4534	80.0788	122.555
16026	3.4535	102.827	92.8465
16027	3.4536	92.4	156.988
16028	3.4536	107.282	64.4022
16029	3.4536	70.4389	100.722
16030	3.4536	79.6079	122.088
16031	3.4536	101.084	138.439
16032	3.4536	78.403	353.178
16033	3.4537	105.279	209.648
16034	3.4537	82.2413	327.476
16035	3.4538	96.1779	104.949
16036	3.4539	111.441	197.949
16037	3.4539	113.48	339.592
16038	3.4539	39.276	1.1497
16039	3.454	96.6024	170.507
16040	3.4541	100.223	243.024
16041	3.4541	83.7743	306.579
16042	3.4543	79.6949	90.279
16043	3.4543	81.0668	234.719
16044	3.4544	82.3535	302.093
16045	3.4545	89.3498	250.193
16046	3.4545	-3.4624	320.102
16047	3.4545	93.7892	50.3834
16048	3.4546	81.54	118.042
16049	3.4546	93.2648	272.726
16050	3.4547	84.4191	355.318
16051	3.4548	66.6014	286.896
16052	3.4549	86.2069	168.326
16053	3.455	83.328	166.646
16054	3.455	72.1152	306.518
16055	3.4551	83.6261	183.526
16056	3.4551	76.8031	130.143
16057	3.4552	66.7524	113.363
16058	3.4553	86.3971	187.269
16059	3.4553	79.7308	351.089
16060	3.4555	89.2212	314.614
16061	3.4555	78.6039	160.809
16062	3.4555	67.8018	144.911
16063	3.4555	70.3358	133.008
16064	3.4556	82.8441	125.346
16065	3.4558	89.6122	58.8695
16066	3.456	90.8748	105.169
16067	3.4561	98.5298	23.619
16068	3.4562	98.4136	67.5972
16069	3.4562	82.1228	141.097

16070	3.4563	71.0474	229.684
16071	3.4564	-4.1097	216.908
16072	3.4564	75.9384	84.1109
16073	3.4565	67.6499	299.958
16074	3.4566	71.7233	231.262
16075	3.4566	95.1726	196.85
16076	3.4567	-3.3044	30.032
16077	3.4568	70.6294	186.137
16078	3.4569	84.9676	19.2373
16079	3.4569	89.2777	44.558
16080	3.4572	53.0801	92.4713
16081	3.4572	85.5227	81.3015
16082	3.4573	90.9006	308.196
16083	3.4574	-4.0574	32.9246
16084	3.4576	86.071	66.6597
16085	3.4576	79.9379	192.105
16086	3.4576	80.5878	286.553
16087	3.4577	-2.6832	266.155
16088	3.4578	79.2985	74.6349
16089	3.4578	83.9746	140.591
16090	3.458	63.5498	81.5447
16091	3.4582	98.5391	169.645
16092	3.4582	90.5079	65.4317
16093	3.4583	90.4325	141.306
16094	3.4583	88.5594	115.162
16095	3.4585	76.6408	11.2046
16096	3.4585	75.3806	160.016
16097	3.4585	84.8907	185.166
16098	3.4587	-2.893	184.564
16099	3.4588	117.122	49.8238
16100	3.4588	102.644	75.656
16101	3.4588	91.3921	82.7434
16102	3.4588	84.8389	337.571
16103	3.4589	59.9851	184.362
16104	3.4589	77.3977	125.345
16105	3.4592	63.9011	279.444
16106	3.4593	98.1886	95.2483
16107	3.4593	54.5927	252.686
16108	3.4593	87.6308	291.105
16109	3.4593	4.3804	318.311
16110	3.4594	87.7678	28.0997
16111	3.4594	82.8501	111.663
16112	3.4594	-2.6823	1.6203
16113	3.4596	74.37	128.033
16114	3.4599	87.3069	300.087
16115	3.4602	89.5015	86.4123
16116	3.4605	94.0769	139.715
16117	3.4605	77.5885	12.4221

16118	3.4605	-2.6876	266.554
16119	3.4606	42.1829	154.846
16120	3.4606	-2.8559	152.441
16121	3.4608	96.4465	0.7076
16122	3.4609	76.0605	100.139
16123	3.461	87.0935	0.6926
16124	3.461	62.6747	129.218
16125	3.4611	99.721	319.516
16126	3.4611	103.757	106.545
16127	3.4613	87.8639	115.716
16128	3.4613	84.0759	293.716
16129	3.4613	69.7129	119.702
16130	3.4613	106.066	21.1176
16131	3.4618	86.1853	256.132
16132	3.4619	75.7542	155.824
16133	3.4619	92.7328	308.38
16134	3.4619	86.0639	108.806
16135	3.462	67.2131	80.4368
16136	3.462	85.3259	131.56
16137	3.4621	-3.3606	25.4025
16138	3.4621	102.271	150.266
16139	3.4621	78.8438	45.473
16140	3.4625	71.6012	226.046
16141	3.4627	87.8739	164.913
16142	3.4627	66.276	141.2
16143	3.4627	84.77	266.911
16144	3.4628	93.9247	169.338
16145	3.4629	95.9242	127.432
16146	3.4629	75.4199	151.438
16147	3.4629	51.3627	150.619
16148	3.4629	86.3295	129.228
16149	3.463	76.6958	179.749
16150	3.463	86.318	125.949
16151	3.4632	93.7064	146.869
16152	3.4632	88.7681	89.3185
16153	3.4632	108.328	51.3413
16154	3.4633	70.5806	217.184
16155	3.4633	76.7398	86.396
16156	3.4634	-3.2439	225.532
16157	3.4635	56.1133	284.996
16158	3.4637	91.4523	225.941
16159	3.4637	83.665	134.89
16160	3.4638	107.308	205.439
16161	3.464	100.017	81.9889
16162	3.4641	85.7175	234.202
16163	3.4642	95.9199	321.917
16164	3.4642	86.7355	158.495
16165	3.4644	81.9728	95.9131

16166	3.4645	89.4793	114.017
16167	3.4645	87.4424	72.6568
16168	3.4646	81.1836	301.704
16169	3.4646	50.4654	158.125
16170	3.4648	104.999	210.611
16171	3.465	81.441	74.3696
16172	3.4651	97.4048	104.572
16173	3.4652	101.506	206.143
16174	3.4653	94.8596	119.369
16175	3.4653	82.1566	97.6605
16176	3.4654	118.601	62.3484
16177	3.4654	85.159	153.634
16178	3.4656	64.3777	306.128
16179	3.4657	91.1006	306.595
16180	3.4657	88.7808	149.913
16181	3.4658	84.2424	114.819
16182	3.4659	94.6513	224.074
16183	3.4659	107.429	129.248
16184	3.466	96.6119	152.744
16185	3.466	85.1728	174.557
16186	3.4661	106.939	344.433
16187	3.4663	83.293	269.632
16188	3.4664	86.186	270.281
16189	3.4664	67.7205	145.382
16190	3.4665	86.263	236.607
16191	3.4665	83.8734	325.738
16192	3.4665	83.2759	110.528
16193	3.4667	88.825	130.99
16194	3.4668	91.0474	329.491
16195	3.4669	-4.1328	163.83
16196	3.467	74.281	191.894
16197	3.4671	81.0968	167.521
16198	3.4672	71.0952	209.166
16199	3.4673	103.401	286.821
16200	3.4674	83.4138	148.328
16201	3.4679	107.335	41.6605
16202	3.4681	81.7598	146.179
16203	3.4681	-2.7734	95.1304
16204	3.4681	73.5508	118.415
16205	3.4682	88.9788	217.978
16206	3.4682	88.9139	317.942
16207	3.4685	72.7428	122.288
16208	3.4686	72.6569	271.118
16209	3.4687	97.538	63.1787
16210	3.4688	90.2695	139.748
16211	3.4689	54.0684	219.168
16212	3.4689	89.985	118.538
16213	3.469	85.1956	63.6806

16214	3.4691	86.2412	76.7164
16215	3.4691	88.1502	102.756
16216	3.4692	-2.8125	134.609
16217	3.4693	85.5731	240.553
16218	3.4693	83.5301	129.663
16219	3.4694	100.229	104.215
16220	3.4695	74.0716	162.303
16221	3.4695	107.812	68.8251
16222	3.4696	87.7919	298.841
16223	3.4697	80.4635	355.948
16224	3.4699	120.237	44.6046
16225	3.47	89.6773	71.0552
16226	3.4701	88.6583	77.6712
16227	3.4701	-3.0271	263.662
16228	3.4701	97.9279	137.497
16229	3.4702	101.366	132.577
16230	3.4702	82.7577	106.074
16231	3.4703	68.513	192.572
16232	3.4703	86.0707	16.5065
16233	3.4703	93.4138	187.111
16234	3.4704	106.926	312.813
16235	3.4705	-3.9809	148.353
16236	3.4706	95.1355	313.045
16237	3.4706	82.9569	188.866
16238	3.4707	76.9757	181.088
16239	3.4707	100.118	12.7508
16240	3.4707	78.1614	353.921
16241	3.4707	-3.3559	73.7558
16242	3.4708	110.202	17.7413
16243	3.4708	97.0128	134.768
16244	3.4709	73.591	231.711
16245	3.4709	74.7858	99.2688
16246	3.4711	114.917	197.111
16247	3.4711	-2.7988	179.337
16248	3.4712	104.374	18.3708
16249	3.4712	86.7386	99.4343
16250	3.4715	108.072	359.87
16251	3.4715	89.5631	241.215
16252	3.4716	75.4167	38.9856
16253	3.4717	62.162	190.232
16254	3.4718	86.6692	78.5233
16255	3.4718	85.1054	237.597
16256	3.4719	88.3884	203.053
16257	3.472	90.2829	30.6001
16258	3.472	46.1238	326.175
16259	3.472	94.5338	339.929
16260	3.4723	46.2606	72.0874
16261	3.4727	98.1211	245.6

16262	3.473	-3.8854	265.701
16263	3.4731	66.2291	295.765
16264	3.4731	72.5271	199.384
16265	3.4732	94.2205	83.898
16266	3.4733	114.25	242.979
16267	3.4733	84.9103	129.592
16268	3.4734	90.3451	59.6343
16269	3.4736	89.335	183.631
16270	3.4736	89.2192	165.436
16271	3.4737	92.8646	104.21
16272	3.4737	89.823	140.892
16273	3.4737	105.651	125.803
16274	3.4739	87.3125	100.074
16275	3.4739	91.718	91.4021
16276	3.474	92.8038	217.38
16277	3.474	-2.9657	116.37
16278	3.4741	85.3728	198.931
16279	3.4746	96.3884	265.326
16280	3.4746	105.605	134.146
16281	3.4746	85.2234	111.061
16282	3.4746	73.0195	67.7213
16283	3.4747	92.3997	284.852
16284	3.4747	77.1803	132.52
16285	3.4747	91.744	162.371
16286	3.4748	-3.2564	91.3273
16287	3.475	95.2494	117.054
16288	3.475	62.355	2.769
16289	3.4751	120.827	196.005
16290	3.4752	-5.7689	92.2964
16291	3.4753	16.3112	3.5991
16292	3.4754	94.0413	291.713
16293	3.4756	85.5112	71.0424
16294	3.476	88.9831	102.942
16295	3.476	79.3257	260.132
16296	3.4761	72.599	128.647
16297	3.4762	88.219	21.2205
16298	3.4762	81.0858	4.5251
16299	3.4762	84.657	135.133
16300	3.4763	99.1563	280.837
16301	3.4763	98.4553	127.744
16302	3.4763	92.4589	185.666
16303	3.4764	99.4069	39.2948
16304	3.4766	95.1092	223.157
16305	3.4766	96.7739	212.545
16306	3.4767	141.567	314.715
16307	3.4768	-4.4324	155.435
16308	3.4769	93.2651	169.526
16309	3.4769	92.118	105.062

16310	3.4771	93.2814	139.972
16311	3.4775	86.8851	196.891
16312	3.4775	127.557	216.172
16313	3.4776	33.8653	240.02
16314	3.4777	99.3351	78.5315
16315	3.4778	104.412	64.6594
16316	3.4778	82.1133	187.646
16317	3.4778	77.7333	226.398
16318	3.4779	29.519	153.08
16319	3.4779	99.2103	38.7099
16320	3.478	62.4673	191.046
16321	3.4781	88.4005	129.051
16322	3.4782	96.068	221.799
16323	3.4782	100.425	235.517
16324	3.4783	105.095	42.2779
16325	3.4783	93.7673	232.666
16326	3.4783	76.9291	169.502
16327	3.4784	86.5372	291.685
16328	3.4784	83.964	142.018
16329	3.4784	72.3157	68.5753
16330	3.4785	90.6782	265.023
16331	3.4785	73.5431	312.207
16332	3.4785	106.396	45.8902
16333	3.4787	20.4813	267.575
16334	3.4787	67.4772	181.471
16335	3.4788	115.753	63.2736
16336	3.4788	-4.422	317.645
16337	3.4788	63.7738	70.4972
16338	3.4789	97.2101	112.707
16339	3.479	91.2153	108.677
16340	3.4791	84.6587	103.291
16341	3.4791	92.7429	138.279
16342	3.4792	-3.0499	16.871
16343	3.4792	94.2282	229.089
16344	3.4794	106.727	246.446
16345	3.4794	73.3805	73.1956
16346	3.4794	-2.8861	92.9619
16347	3.4794	74.266	51.5311
16348	3.4795	107.997	147.999
16349	3.4795	88.8929	287.569
16350	3.4796	83.9662	190.198
16351	3.4797	83.6269	261.769
16352	3.4797	74.1697	177.606
16353	3.4797	68.4381	126.136
16354	3.4799	78.9453	199.921
16355	3.4799	77.794	4.3565
16356	3.4801	85.4665	98.2684
16357	3.4802	-2.919	151.248

16358	3.4803	72.0492	184.945
16359	3.4803	81.6819	122.967
16360	3.4803	101.103	336.053
16361	3.4804	68.3363	185.626
16362	3.4807	104.829	56.2723
16363	3.4807	92.3601	132.641
16364	3.4808	70.2678	306.119
16365	3.4808	106.38	340.364
16366	3.4809	97.6366	274.168
16367	3.481	108.036	62.7316
16368	3.4812	73.3325	118.844
16369	3.4812	102.814	155.211
16370	3.4814	-5.5201	129.194
16371	3.4814	99.6906	105.967
16372	3.4815	85.0399	226.628
16373	3.4815	73.8111	272.294
16374	3.4815	92.2442	336.278
16375	3.4816	90.9683	59.1731
16376	3.4816	92.3481	266.87
16377	3.4817	46.9202	32.8064
16378	3.4818	67.6437	172.86
16379	3.4818	83.8903	131.623
16380	3.4821	105.573	65.135
16381	3.4821	83.5488	158.888
16382	3.4823	95.6351	99.4404
16383	3.4824	88.4977	120.062
16384	3.4824	93.3324	121.957
16385	3.4825	73.1045	217.804
16386	3.4825	103.071	46.6107
16387	3.4826	-2.7677	197.913
16388	3.4829	108.396	42.0294
16389	3.4829	62.7779	142.393
16390	3.4829	87.6997	321.79
16391	3.483	74.0778	268.57
16392	3.4831	76.3572	90.4834
16393	3.4832	-3.7987	182.652
16394	3.4835	-3.7296	9.6299
16395	3.4836	88.3252	173.58
16396	3.4839	87.341	111.256
16397	3.4839	76.6315	69.7533
16398	3.4841	89.0103	186.296
16399	3.4842	135.867	259.594
16400	3.4843	98.1519	359.789
16401	3.4844	74.7624	107.361
16402	3.4845	88.3503	274.37
16403	3.4845	58.7462	210.893
16404	3.4845	101.325	317.146
16405	3.4845	103.43	146.714

16406	3.4846	-2.788	293.909
16407	3.4847	-3.1168	300.061
16408	3.4848	86.1952	190.472
16409	3.4852	79.9551	147.254
16410	3.4853	85.3644	90.0921
16411	3.4853	84.8877	319.152
16412	3.4854	83.7065	104.724
16413	3.4855	107.913	43.0721
16414	3.4855	33.4373	220.508
16415	3.4855	76.1672	165.09
16416	3.4856	99.9531	252.319
16417	3.4857	108.222	35.2632
16418	3.4858	107.258	245.174
16419	3.4858	-2.7181	342.507
16420	3.4858	82.8407	250.199
16421	3.4859	99.7313	160.002
16422	3.4859	83.4193	147.036
16423	3.4859	125.085	89.1931
16424	3.4859	1.7038	263.476
16425	3.4859	83.5063	336.101
16426	3.486	72.127	137.78
16427	3.4861	77.9631	228.772
16428	3.4861	96.3138	167.766
16429	3.4862	80.6896	200.582
16430	3.4863	110.523	204.883
16431	3.4865	78.78	172.48
16432	3.4866	75.3591	262.311
16433	3.4867	107.089	71.0403
16434	3.4867	95.6075	213.937
16435	3.4867	92.2374	43.9799
16436	3.4867	113.477	113.256
16437	3.4868	80.9108	263.851
16438	3.4868	103.745	265.122
16439	3.4869	85.4293	118.941
16440	3.4869	72.3845	169.257
16441	3.4871	108.192	245.17
16442	3.4871	100.563	107.078
16443	3.4871	-3.059	195.799
16444	3.4871	86.9889	213.172
16445	3.4871	98.2507	329.978
16446	3.4872	99.235	201.778
16447	3.4872	74.1425	313.419
16448	3.4872	92.4889	116.1
16449	3.4874	110.445	95.7787
16450	3.4874	142.482	204.034
16451	3.4875	106.245	260.229
16452	3.4876	95.0012	187.597
16453	3.4877	81.1126	194.101

16454	3.4877	93.8215	29.4673
16455	3.4879	-3.9347	246.797
16456	3.4881	103.08	60.3049
16457	3.4881	91.991	257.278
16458	3.4883	68.2701	156.69
16459	3.4883	86.9104	176.745
16460	3.4885	4.3629	109.914
16461	3.4885	84.5161	216.582
16462	3.4887	112.329	132.719
16463	3.4887	93.166	352.82
16464	3.4889	92.3711	153.53
16465	3.4889	95.7344	319.068
16466	3.4889	81.9397	134.999
16467	3.4891	75.2774	155.595
16468	3.4891	98.9872	279.546
16469	3.4892	100.921	83.1706
16470	3.4893	89.5699	311.366
16471	3.4893	112.114	2.4239
16472	3.4895	72.0575	157.945
16473	3.4896	82.1586	334.194
16474	3.4898	94.2938	94.4096
16475	3.49	84.599	54.4779
16476	3.4901	109.106	55.7556
16477	3.4901	87.0926	128.623
16478	3.4901	88.0129	140.605
16479	3.4902	86.4705	258.921
16480	3.4903	93.1217	50.5589
16481	3.4904	105.989	119.462
16482	3.4904	110.182	61.1566
16483	3.4905	95.3515	145.936
16484	3.4906	101.927	308.426
16485	3.4906	73.8437	144.216
16486	3.4907	93.2776	25.5071
16487	3.4907	84.5891	89.0381
16488	3.4907	109.537	119.92
16489	3.4909	114.207	248.687
16490	3.4909	94.9247	95.5486
16491	3.4909	100.429	205.929
16492	3.4909	195.329	46.305
16493	3.4909	89.5772	229.708
16494	3.4911	91.228	200.604
16495	3.4911	99.3955	92.1474
16496	3.4912	111.399	52.6043
16497	3.4912	69.3343	175.572
16498	3.4912	94.9643	62.0345
16499	3.4915	88.108	213.318
16500	3.4915	81.3883	254.896
16501	3.4915	87.0496	188.463

16502	3.4919	87.0378	119.515
16503	3.4919	10.8975	243.34
16504	3.492	154.586	55.1836
16505	3.4921	78.8709	137.213
16506	3.4921	85.0199	146.534
16507	3.4921	98.7731	333.978
16508	3.4922	81.1343	86.9606
16509	3.4922	100.354	243.66
16510	3.4922	83.8642	297.968
16511	3.4923	88.4469	308.352
16512	3.4923	76.6311	141.426
16513	3.4923	92.8734	39.4509
16514	3.4924	84.3456	94.8541
16515	3.4924	91.4408	21.6892
16516	3.4925	86.694	125.298
16517	3.4926	100.031	288.064
16518	3.4927	83.2019	66.5929
16519	3.4927	99.348	330.221
16520	3.4928	75.7426	254.551
16521	3.4929	92.4853	94.8803
16522	3.4932	-3.3757	113.605
16523	3.4933	89.4143	112.743
16524	3.4934	-3.1309	146.78
16525	3.4934	99.1977	14.3657
16526	3.4936	104.211	56.15
16527	3.4939	123.499	31.847
16528	3.4941	98.0884	200.131
16529	3.4941	74.0781	287.893
16530	3.4941	114.751	351.256
16531	3.4942	94.1628	243.447
16532	3.4944	-3.9561	164.41
16533	3.4944	85.5201	139.989
16534	3.4945	103.217	63.1319
16535	3.4945	96.8965	303.917
16536	3.4946	109.683	33.099
16537	3.4946	108.97	42.0449
16538	3.4947	90.4897	95.4824
16539	3.4947	98.1387	232.089
16540	3.4947	108.411	306.217
16541	3.4948	87.8557	1.0805
16542	3.4949	94.067	95.5428
16543	3.4952	101.779	62.9342
16544	3.4952	104.073	217.249
16545	3.4953	81.5985	281.733
16546	3.4953	105.746	142.541
16547	3.4954	-3.3659	146.726
16548	3.4955	93.4085	154.879
16549	3.4956	93.5678	136.585

16550	3.4957	91.4007	98.6067
16551	3.4957	-3.246	124.546
16552	3.4958	15.7368	257.368
16553	3.4958	90.6194	86.5956
16554	3.4959	74.9852	165.798
16555	3.496	63.8577	293.432
16556	3.496	-3.9722	40.9082
16557	3.4961	75.3974	89.6621
16558	3.4961	91.8333	318.461
16559	3.4962	71.6803	95.854
16560	3.4963	88.9496	211.861
16561	3.4965	87.6278	86.2916
16562	3.4965	90.4914	204.592
16563	3.4966	102.816	64.2134
16564	3.4966	114.058	217.424
16565	3.4966	97.1585	94.7628
16566	3.4968	76.058	63.6372
16567	3.497	77.1411	156.907
16568	3.4971	73.3862	90.5925
16569	3.4973	116.134	26.8335
16570	3.4975	96.3168	107.202
16571	3.4975	77.8366	204.124
16572	3.4975	110.37	52.9976
16573	3.4976	95.0605	335.218
16574	3.4977	81.9118	1.7919
16575	3.498	71.4466	141.295
16576	3.4981	99.0449	57.054
16577	3.4981	102.528	123.673
16578	3.4982	130.309	81.5842
16579	3.4982	91.3148	235.005
16580	3.4983	90.5341	270.406
16581	3.4984	-2.7255	27.121
16582	3.4985	-2.7529	279.28
16583	3.4985	86.2712	77.0436
16584	3.4986	87.0643	109.91
16585	3.4987	114.371	274.404
16586	3.4988	-3.8642	145.853
16587	3.4989	78.9332	207.723
16588	3.4989	73.2961	161.502
16589	3.4991	83.9659	25.0401
16590	3.4992	95.7424	42.6283
16591	3.4992	73.4126	127.151
16592	3.4992	115.709	130.422
16593	3.4993	85.9808	105.803
16594	3.4993	3.2415	108.957
16595	3.4995	93.5349	238.257
16596	3.4996	77.9045	98.7193
16597	3.4997	123.154	289.014

16598	3.4997	136.922	208.669
16599	3.4997	82.4717	336.445
16600	3.4999	92.4661	151.591
16601	3.4999	92.1942	80.8787
16602	3.4999	95.9735	0.1195
16603	3.5	99.6848	91.5224
16604	3.5001	86.9883	163.694
16605	3.5002	93.4292	40.0028
16606	3.5006	-3.4111	188.653
16607	3.5006	99.5611	266.063
16608	3.5008	84.2971	130.321
16609	3.501	113.591	174.579
16610	3.5011	109.718	35.9364
16611	3.5011	84.45	141.938
16612	3.5011	101.902	56.4597
16613	3.5011	106.868	84.5522
16614	3.5012	78.3848	195.854
16615	3.5013	100.199	275.686
16616	3.5013	6.0403	129.254
16617	3.5015	-3.3798	51.3732
16618	3.5015	86.3124	183.183
16619	3.5016	110.717	313.64
16620	3.5016	66.0613	147.275
16621	3.5016	101.551	305.066
16622	3.5017	88.7144	158.577
16623	3.5017	71.8582	81.1462
16624	3.5018	129.469	86.564
16625	3.5018	104.731	264.07
16626	3.5018	88.7721	146.351
16627	3.5018	85.4431	0.7772
16628	3.5019	104.83	246.254
16629	3.502	103.222	100.129
16630	3.502	-3.4331	335.211
16631	3.5021	28.899	90.2539
16632	3.5021	-3.1654	131.485
16633	3.5022	14.4268	265.073
16634	3.5022	89.7737	93.9626
16635	3.5023	62.8222	146.553
16636	3.5023	98.7791	268.911
16637	3.5024	96.4845	353.955
16638	3.5026	76.9154	65.1883
16639	3.5026	84.5056	91.0828
16640	3.5027	93.7742	283.931
16641	3.5027	84.6094	75.2826
16642	3.5027	121.63	204.141
16643	3.5028	137.511	115.858
16644	3.5029	111.681	28.7821
16645	3.5029	115.916	37.1028

16646	3.503	-4.5884	279.508
16647	3.503	84.9978	220.041
16648	3.5031	76.0442	22.8655
16649	3.5031	105.359	146.691
16650	3.5032	98.4629	70.8025
16651	3.5034	112.721	194.465
16652	3.5034	103.293	205.88
16653	3.5034	108.92	31.604
16654	3.5035	80.7965	4.9039
16655	3.5035	87.9783	157.701
16656	3.5036	122.937	314.293
16657	3.5036	107.847	77.2141
16658	3.5036	95.4193	141.938
16659	3.5037	95.4171	94.5277
16660	3.5037	91.4874	23.9805
16661	3.5038	111.556	213.477
16662	3.5038	99.1274	234.101
16663	3.5039	94.6997	179.297
16664	3.5039	80.9383	258.895
16665	3.5039	87.3086	196.315
16666	3.5042	111.786	97.9867
16667	3.5042	90.4196	104.187
16668	3.5044	120.487	105.416
16669	3.5045	114.157	22.1068
16670	3.5046	94.8101	171.771
16671	3.5047	91.2866	43.4839
16672	3.505	112.701	275.555
16673	3.505	98.306	95.2057
16674	3.505	-3.0289	114.32
16675	3.5052	79.1788	177.892
16676	3.5052	75.5664	18.4641
16677	3.5054	85.9808	102.126
16678	3.5055	95.4946	281.519
16679	3.5055	90.7635	299.538
16680	3.5055	101.9	31.1393
16681	3.5056	86.8786	70.676
16682	3.5056	96.1445	20.1305
16683	3.5056	122.927	90.8305
16684	3.5057	72.9672	336.181
16685	3.5057	80.2941	275.224
16686	3.5057	88.8236	91.2212
16687	3.506	105.154	77.9016
16688	3.5061	80.2775	135.221
16689	3.5061	-4.2137	16.3438
16690	3.5062	78.0514	177.296
16691	3.5063	90.1902	280.637
16692	3.5065	99.6661	359.045
16693	3.5065	77.3989	144.822

16694	3.5067	101.795	186.889
16695	3.5067	83.8145	134.813
16696	3.5067	107.166	117.293
16697	3.5068	105.329	344.552
16698	3.5068	95.1104	262.485
16699	3.5069	99.6599	20.5031
16700	3.507	93.2784	39.8635
16701	3.5071	92.356	103.128
16702	3.5072	45.158	287.751
16703	3.5072	84.6082	123.673
16704	3.5072	94.0649	103.097
16705	3.5074	85.2782	197.563
16706	3.5074	84.5115	68.5175
16707	3.5075	96.5054	103.647
16708	3.5076	115.754	68.7735
16709	3.5077	82.1175	185.781
16710	3.5078	100.099	108.188
16711	3.5078	90.2255	152.037
16712	3.5078	4.4106	341.965
16713	3.5078	82.807	23.2985
16714	3.5079	127.379	91.4353
16715	3.508	108.45	69.962
16716	3.508	-3.4253	135.217
16717	3.5081	82.5666	247.915
16718	3.5081	68.1794	70.8326
16719	3.5082	81.7254	181.044
16720	3.5082	77.9556	191.212
16721	3.5082	92.036	110.345
16722	3.5082	71.4285	321.965
16723	3.5084	82.3636	237.972
16724	3.5085	98.4242	329.851
16725	3.509	78.0363	121.524
16726	3.5091	87.7979	218.854
16727	3.5091	87.3644	83.6265
16728	3.5093	108.695	49.3752
16729	3.5093	92.3202	107.334
16730	3.5093	94.198	281.939
16731	3.5095	102.003	73.8811
16732	3.5095	81.2143	264.733
16733	3.5096	98.7396	202.539
16734	3.5097	82.126	80.254
16735	3.5097	102.632	176.344
16736	3.5098	73.8555	220.583
16737	3.5099	119.339	128.01
16738	3.5099	98.7999	122.915
16739	3.5099	89.0325	45.5411
16740	3.51	72.3019	127.319
16741	3.51	75.7293	335.728

16742	3.51	87.7762	337.305
16743	3.5101	85.3502	236.593
16744	3.5101	69.5619	129.982
16745	3.5102	91.2536	146.447
16746	3.5103	90.3574	285.253
16747	3.5105	104.703	305.533
16748	3.5107	83.5168	142.772
16749	3.5108	90.7414	1.0452
16750	3.5108	106.757	178.479
16751	3.5108	100.574	36.8009
16752	3.511	-3.3747	308.552
16753	3.511	93.926	279.687
16754	3.5111	88.9939	226.701
16755	3.5114	99.1346	231.823
16756	3.5114	108.779	315.227
16757	3.5115	88.7631	160.491
16758	3.5117	95.4206	353.892
16759	3.5117	86.7412	135.986
16760	3.5118	95.5773	175.314
16761	3.5121	97.1338	188.581
16762	3.5121	94.8198	357.048
16763	3.5122	75.8157	151.481
16764	3.5122	75.2568	169.581
16765	3.5123	99.2019	35.6657
16766	3.5124	-3.6049	117.502
16767	3.5124	83.5775	188.956
16768	3.5126	97.9914	126.316
16769	3.5126	95.3784	172.114
16770	3.5126	79.9071	137.237
16771	3.5128	110.022	240.623
16772	3.5128	105.221	178.384
16773	3.5129	94.413	54.0344
16774	3.513	94.6188	65.9875
16775	3.5132	107.41	265.481
16776	3.5132	89.927	180.223
16777	3.5132	92.4832	91.3858
16778	3.5133	67.3986	93.8216
16779	3.5133	-3.4732	314.972
16780	3.5134	85.485	248.643
16781	3.5135	114.647	20.9333
16782	3.5136	100.481	156.039
16783	3.5136	96.2655	64.9446
16784	3.5138	104.764	114.525
16785	3.5139	97.6433	59.3476
16786	3.514	86.8128	177.124
16787	3.514	83.1533	189.219
16788	3.5142	75.748	194.488
16789	3.5142	68.5855	60.3326

16790	3.5142	100.131	323.64
16791	3.5145	82.7446	161.938
16792	3.5145	103.669	78.8863
16793	3.5148	26.3368	126.882
16794	3.5148	96.9207	77.8573
16795	3.5148	-2.7133	20.4375
16796	3.5148	96.641	218.988
16797	3.515	118.303	135.806
16798	3.5151	86.726	137.864
16799	3.5151	110.647	62.524
16800	3.5152	102.106	254.676
16801	3.5152	90.0548	196.455
16802	3.5153	114.242	47.8466
16803	3.5153	114.912	72.9511
16804	3.5154	74.9379	124.713
16805	3.5155	69.5969	134.215
16806	3.5155	88.8515	117.795
16807	3.5158	78.9451	108.302
16808	3.5158	86.1187	114.192
16809	3.516	97.9974	237.747
16810	3.5161	-2.7143	17.5809
16811	3.5162	95.8241	216.643
16812	3.5162	111.907	311.217
16813	3.5162	-3.2646	190.854
16814	3.5162	78.7223	137.246
16815	3.5163	87.7434	123.692
16816	3.517	104.595	41.9776
16817	3.517	111.72	286.321
16818	3.5171	94.4388	321.534
16819	3.5175	81.1792	276.446
16820	3.5176	103.305	335.413
16821	3.5177	124.952	45.3252
16822	3.5178	110.576	0.3241
16823	3.5178	85.969	29.6623
16824	3.5179	99.5625	137.8
16825	3.5179	80.9764	261.865
16826	3.5179	110.087	14.6671
16827	3.518	90.3381	93.5245
16828	3.518	100.533	327.649
16829	3.5181	78.3631	54.5542
16830	3.5182	87.0324	36.3216
16831	3.5182	88.0716	67.5784
16832	3.5183	109.293	292.07
16833	3.5183	85.7371	177.543
16834	3.5183	83.3331	181.974
16835	3.5183	-2.7328	32.2179
16836	3.5184	123.271	58.5039
16837	3.5184	-2.6436	289.024

16838	3.5184	77.4602	190.549
16839	3.5186	105.326	117.822
16840	3.5187	-1.349	189.433
16841	3.5187	-3.1134	170.464
16842	3.5188	81.156	302.97
16843	3.5188	90.8412	55.6268
16844	3.5189	110.079	317.599
16845	3.519	99.1552	86.1323
16846	3.5193	-4.3769	146.972
16847	3.5194	121.954	204.071
16848	3.5196	101.338	332.845
16849	3.5198	89.1726	127.737
16850	3.5199	115.987	166.562
16851	3.52	103.144	110.002
16852	3.52	102.739	154.065
16853	3.5202	97.1593	218.911
16854	3.5203	132.102	284.381
16855	3.5203	69.5342	171.733
16856	3.5203	102.942	60.0851
16857	3.5205	70.3877	165.332
16858	3.5205	99.5278	125.025
16859	3.5205	15.006	116.089
16860	3.5206	111.098	116.549
16861	3.5206	94.4391	198.35
16862	3.5208	91.0985	232.103
16863	3.5208	-3.2429	139.396
16864	3.5208	90.0232	318.398
16865	3.5209	83.5546	134.141
16866	3.5209	96.0237	63.3127
16867	3.521	117.234	33.4925
16868	3.5212	122.998	50.9876
16869	3.5212	109.746	309.184
16870	3.5212	85.0866	5.5812
16871	3.5212	88.1534	231.574
16872	3.5213	107.772	78.4327
16873	3.5213	-2.7415	25.8939
16874	3.5213	120.115	113.029
16875	3.5214	94.0881	281.244
16876	3.5215	70.5177	202.908
16877	3.5217	91.2279	199.684
16878	3.5217	-2.6691	247.84
16879	3.5217	99.0163	267.625
16880	3.5217	92.5174	167.342
16881	3.522	89.9632	210.513
16882	3.5221	116.249	58.2013
16883	3.5223	105.358	162.918
16884	3.5223	-2.7872	83.4053
16885	3.5223	123.443	26.8848

16886	3.5224	93.9435	66.9356
16887	3.5225	99.2406	188.76
16888	3.5227	2.6397	225.768
16889	3.5227	98.4294	237.234
16890	3.5227	84.6008	295.082
16891	3.5227	100.791	88.0834
16892	3.5228	81.3998	236.25
16893	3.5229	115.26	196.414
16894	3.523	67.3769	132.37
16895	3.523	104.146	302.14
16896	3.5231	87.171	134.11
16897	3.5232	101.191	28.8924
16898	3.5233	95.3296	298.013
16899	3.5234	87.4591	118.18
16900	3.5236	88.072	349.382
16901	3.5237	114.958	140.18
16902	3.5238	96.9155	61.7879
16903	3.5238	72.0189	166.439
16904	3.5241	80.6374	220.493
16905	3.5242	71.669	62.7288
16906	3.5242	95.9684	318.732
16907	3.5244	101.444	34.6758
16908	3.5244	93.1996	154.896
16909	3.5244	79.7697	164.759
16910	3.5244	-2.7195	0.742
16911	3.5246	81.9059	107.001
16912	3.5246	82.7973	128.435
16913	3.5247	101.267	297.538
16914	3.5247	88.2778	124.368
16915	3.5248	69.7949	336.009
16916	3.5249	90.967	259.091
16917	3.5249	93.171	126.953
16918	3.5251	91.1538	225.11
16919	3.5251	101.35	116.154
16920	3.5252	93.6777	147.079
16921	3.5253	81.5387	135.942
16922	3.5254	97.4468	141.001
16923	3.5254	83.0301	68.034
16924	3.5254	21.5649	55.2245
16925	3.5255	99.7996	122.316
16926	3.5256	118.909	41.0812
16927	3.5258	90.7418	205.861
16928	3.526	127.693	49.2198
16929	3.5262	91.412	189.797
16930	3.5262	73.4877	186.992
16931	3.5262	103.43	341.885
16932	3.5264	103.853	79.5204
16933	3.5265	90.9951	191.606

16934	3.5266	95.5762	152.474
16935	3.5266	110.179	36.1017
16936	3.5268	105.361	106.157
16937	3.5268	90.135	16.5854
16938	3.5269	111.973	208.493
16939	3.527	104.516	91.6413
16940	3.527	-2.8617	180.336
16941	3.5271	-2.8145	355.01
16942	3.5271	70.006	220.912
16943	3.5271	23.3409	30.9836
16944	3.5272	87.4873	115.177
16945	3.5273	86.5331	254.289
16946	3.5274	68.7152	179.666
16947	3.5275	21.3924	67.3721
16948	3.5276	22.5277	80.3896
16949	3.5276	66.6898	189.101
16950	3.5278	121.714	314.842
16951	3.5278	80.168	141.973
16952	3.5279	91.0715	129.979
16953	3.5279	74.1225	135.009
16954	3.5279	104.981	266.557
16955	3.528	92.6556	275.277
16956	3.5284	101.185	87.2931
16957	3.5284	6.2178	150.756
16958	3.5284	99.0676	67.2277
16959	3.5285	113.159	23.8003
16960	3.5287	103.437	210.855
16961	3.5287	99.6937	31.7148
16962	3.5288	102.466	269.601
16963	3.5289	107.826	345.084
16964	3.5292	92.1299	86.9616
16965	3.5292	90.5339	118.41
16966	3.5292	119.497	103.944
16967	3.5293	95.5084	37.3788
16968	3.5293	84.8256	217.358
16969	3.5294	94.0617	199.597
16970	3.5294	86.5577	293.962
16971	3.5295	107.684	26.9826
16972	3.5296	126.677	61.5857
16973	3.5296	99.3328	48.622
16974	3.5296	85.7668	217.45
16975	3.5296	98.1929	213.695
16976	3.5298	96.4467	40.9693
16977	3.5298	76.1295	163.04
16978	3.53	94.8283	102.189
16979	3.5302	116.962	169.22
16980	3.5302	-3.7149	319.267
16981	3.5304	90.9174	15.5233

16982	3.5305	93.9747	29.3315
16983	3.5305	37.6115	280.724
16984	3.5306	102.259	110.879
16985	3.5306	94.1989	197.561
16986	3.5306	121.848	214.802
16987	3.5306	78.6904	234.805
16988	3.5307	65.2737	34.844
16989	3.5307	92.3823	280.993
16990	3.5307	56.2125	188.562
16991	3.5308	92.3582	100.205
16992	3.5308	98.2481	103.326
16993	3.5309	70.7149	59.2815
16994	3.5309	94.1517	110.081
16995	3.5309	104.355	135.061
16996	3.531	85.3791	281.756
16997	3.5312	101.118	142.469
16998	3.5313	101.764	155.943
16999	3.5313	106.852	87.4866
17000	3.5313	101.316	80.049
17001	3.5313	65.2223	203.764
17002	3.5314	80.54	215.628
17003	3.5314	93.1377	132.913
17004	3.5315	87.9504	271.326
17005	3.5315	115.345	236.409
17006	3.5315	78.8674	259.5
17007	3.5317	3.9634	168.639
17008	3.5317	73.0188	170.145
17009	3.532	92.9972	30.5634
17010	3.5321	68.8159	270.558
17011	3.5322	95.0221	69.6604
17012	3.5322	91.2854	3.7459
17013	3.5325	69.7047	126.255
17014	3.5326	103.243	155.223
17015	3.5327	111.576	197.673
17016	3.5329	93.1274	197
17017	3.5329	99.064	243.549
17018	3.533	103.833	236.557
17019	3.533	85.0813	142.383
17020	3.533	-3.3741	159.968
17021	3.5331	97.2541	340.22
17022	3.5331	102.83	345.535
17023	3.5332	127.547	47.8572
17024	3.5333	116.421	18.4563
17025	3.5335	89.3563	80.2929
17026	3.5335	97.7323	212.83
17027	3.5336	110.624	120.249
17028	3.5336	103.764	305.537
17029	3.5337	81.8565	181.728

17030	3.5338	102.083	46.4974
17031	3.5338	92.4884	56.097
17032	3.5339	91.1311	142.133
17033	3.534	95.4672	180.93
17034	3.534	80.168	126.278
17035	3.534	78.9378	133.068
17036	3.534	96.0457	73.1205
17037	3.5342	101.118	65.2702
17038	3.5342	63.2409	52.9433
17039	3.5343	124.314	83.8496
17040	3.5344	77.7177	201.069
17041	3.5344	99.0843	76.9513
17042	3.5344	86.8223	278.307
17043	3.5345	82.1187	214.408
17044	3.5345	67.3801	277.791
17045	3.5348	89.8402	145.068
17046	3.5348	90.7927	82.5276
17047	3.5351	102.008	112.902
17048	3.5351	77.7473	183.459
17049	3.5351	92.4372	63.2613
17050	3.5352	71.8221	325.263
17051	3.5354	84.2735	286.131
17052	3.5355	73.52	48.0832
17053	3.5356	101.279	252.728
17054	3.5356	78.6109	199.855
17055	3.5358	110.408	251.197
17056	3.5359	89.5107	229.822
17057	3.5359	82.4011	109.612
17058	3.5359	129.198	213.147
17059	3.536	94.9342	212.312
17060	3.5361	83.747	291.91
17061	3.5362	104.774	291.456
17062	3.5363	77.2699	113.166
17063	3.5364	73.826	357.731
17064	3.5364	97.369	89.5405
17065	3.5364	103.389	112.334
17066	3.5364	107.489	179.534
17067	3.5365	103.662	243.954
17068	3.5365	86.5885	134.842
17069	3.5366	-4.2011	144.819
17070	3.5366	109.987	135.408
17071	3.5367	92.4223	46.9916
17072	3.5367	88.8186	299.877
17073	3.5367	101.285	61.0956
17074	3.5369	-2.783	38.2854
17075	3.5369	100.548	290.462
17076	3.537	-2.7971	99.004
17077	3.5371	134.94	20.5937

17078	3.5371	87.1383	262.937
17079	3.5372	104.473	114.571
17080	3.5373	0.9881	329.966
17081	3.5373	100.721	298.689
17082	3.5374	93.3293	217.672
17083	3.5375	101.855	263.696
17084	3.5375	-4.018	219.537
17085	3.5375	88.9978	244.592
17086	3.5376	97.6124	329.215
17087	3.5377	-3.4203	353.881
17088	3.5378	108.647	296.338
17089	3.5378	74.7466	160.334
17090	3.5378	80.0677	195.125
17091	3.5378	88.2416	128.182
17092	3.5379	95.3195	131.072
17093	3.538	83.2164	138.149
17094	3.5381	93.0165	167.978
17095	3.5382	116.026	59.8812
17096	3.5382	99.7768	108.255
17097	3.5382	98.464	84.1512
17098	3.5383	70.3821	174.94
17099	3.5383	89.4726	114.886
17100	3.5383	94.6136	263.406
17101	3.5383	95.0961	153.512
17102	3.5387	111.245	143.298
17103	3.5389	115.056	72.4
17104	3.539	101.938	164.535
17105	3.5391	112.974	197.286
17106	3.5391	89.9321	260.879
17107	3.5391	94.5088	43.3445
17108	3.5391	89.6176	122.795
17109	3.5391	85.4396	6.7843
17110	3.5392	103.195	81.0936
17111	3.5393	123.098	83.5501
17112	3.5394	62.8861	136.032
17113	3.5395	91.3596	119.144
17114	3.5395	106.792	3.5151
17115	3.5396	139.799	358.548
17116	3.5396	107.52	74.2051
17117	3.5396	81.6061	270.134
17118	3.5396	112.374	317.705
17119	3.5396	87.6265	190.943
17120	3.5397	84.468	106.021
17121	3.5398	106.44	358.974
17122	3.5399	-2.7579	294.426
17123	3.5399	80.4727	111.378
17124	3.5401	115.327	203.798
17125	3.5401	105.73	213.247

17126	3.5402	100.825	292.514
17127	3.5405	91.6686	328.387
17128	3.5405	85.5667	122.302
17129	3.5406	93.9109	89.8723
17130	3.5407	78.2856	140.752
17131	3.5407	87.7023	150.284
17132	3.5408	96.719	21.7524
17133	3.5408	94.6602	52.424
17134	3.5409	94.8421	24.2812
17135	3.5409	88.6591	154.31
17136	3.541	126.357	239.431
17137	3.5412	104.814	304.442
17138	3.5412	180.22	288.935
17139	3.5414	91.65	247.588
17140	3.5414	96.0815	150.908
17141	3.5414	100.74	104.743
17142	3.5416	102.533	258.875
17143	3.5418	141.126	109.797
17144	3.5419	87.7005	126.654
17145	3.542	106.593	93.6334
17146	3.5421	101.349	202.452
17147	3.5423	93.5722	237.053
17148	3.5424	-4.2585	96.0817
17149	3.5424	107.584	242.121
17150	3.5424	103.522	200.601
17151	3.5426	-2.8489	144.566
17152	3.5427	105.98	201.861
17153	3.5427	83.4266	308.327
17154	3.5427	116.671	58.2816
17155	3.5428	94.1667	104.502
17156	3.5429	103.704	230.333
17157	3.543	127.385	278.674
17158	3.5431	125.524	7.2946
17159	3.5431	19.1467	123.287
17160	3.5431	100.344	23.3946
17161	3.5432	88.5213	184.816
17162	3.5432	-2.6915	259.039
17163	3.5433	8.9977	297.179
17164	3.5434	24.943	90.4403
17165	3.5435	-1.7698	174.858
17166	3.5436	88.3733	171.888
17167	3.5437	41.6986	233.799
17168	3.5437	66.5608	136.625
17169	3.5437	104.254	211.766
17170	3.5438	100.091	106.697
17171	3.5439	83.8049	342.119
17172	3.5439	-3.2854	251.344
17173	3.5439	105.485	88.2711

17174	3.5439	101.384	57.3222
17175	3.5442	86.0812	265.304
17176	3.5442	77.4628	167.725
17177	3.5442	28.0807	197.828
17178	3.5443	82.8401	98.6427
17179	3.5444	96.3367	230.592
17180	3.5444	94.4485	133.737
17181	3.5445	104.968	95.8041
17182	3.5446	76.964	181.226
17183	3.5447	120.836	47.8236
17184	3.5447	89.3168	213.671
17185	3.5449	86.2821	274.145
17186	3.5449	121.345	66.4465
17187	3.5451	79.7206	170.778
17188	3.5452	122.96	250.057
17189	3.5452	98.7593	127.464
17190	3.5453	45.9834	281.306
17191	3.5454	117.68	246.614
17192	3.5454	95.5142	265.913
17193	3.5456	88.6721	148.925
17194	3.5458	47.0838	28.2782
17195	3.5459	116.971	243.777
17196	3.546	86.2847	103.535
17197	3.546	93.6141	112.137
17198	3.546	79.6778	294.7
17199	3.5461	102.67	78.6782
17200	3.5461	94.0143	123.476
17201	3.5461	115.974	149.747
17202	3.5462	-2.7615	24.9211
17203	3.5463	132.927	211.443
17204	3.5464	134.729	212.691
17205	3.5465	83.6973	156.185
17206	3.5465	100.726	238.338
17207	3.5465	108.184	38.7856
17208	3.5465	110.328	51.2427
17209	3.5467	7.6282	149.75
17210	3.5467	103.554	86.8978
17211	3.5467	99.765	297.592
17212	3.5468	75.1526	159.805
17213	3.5468	101.525	53.7354
17214	3.5468	107.06	290.898
17215	3.5469	87.2211	246.675
17216	3.5469	102.997	73.8116
17217	3.5469	35.9194	259.708
17218	3.547	97.5091	277.224
17219	3.547	91.3278	299.244
17220	3.5471	90.5875	135.997
17221	3.5471	-3.7348	352.106

17222	3.5473	57.4786	37.6818
17223	3.5473	106.52	293.495
17224	3.5474	105.142	57.9227
17225	3.5476	116.935	4.1875
17226	3.5476	100.923	70.1684
17227	3.5476	98.9247	96.9866
17228	3.5476	81.3258	27.299
17229	3.5477	77.6313	126.477
17230	3.5477	96.4641	126.712
17231	3.5478	87.1342	78.4847
17232	3.5482	86.7679	200.169
17233	3.5482	96.4788	259.594
17234	3.5483	77.1237	147.909
17235	3.5483	95.6532	233.935
17236	3.5484	67.0193	129.179
17237	3.5486	84.7656	125.429
17238	3.5486	116.971	85.9871
17239	3.5487	94.2531	260.471
17240	3.5488	77.1351	257.467
17241	3.549	97.055	94.6824
17242	3.5491	102.04	68.1502
17243	3.5491	101.778	295.09
17244	3.5492	111.376	64.3711
17245	3.5492	114.297	310.638
17246	3.5492	101.078	50.1922
17247	3.5493	127.972	103.714
17248	3.5494	84.992	264.694
17249	3.5495	95.0047	276.143
17250	3.5495	115.39	216.801
17251	3.5495	135.325	249.063
17252	3.5496	85.0416	135.345
17253	3.5497	53.271	132.997
17254	3.5498	0.5743	295.115
17255	3.5498	107.728	130.787
17256	3.5501	102.645	125.025
17257	3.5502	78.0546	126.422
17258	3.5504	83.364	168.476
17259	3.5505	112.102	240.157
17260	3.5505	122.379	308.595
17261	3.5507	101.361	58.1318
17262	3.5507	95.9116	187.753
17263	3.5507	110.104	94.1847
17264	3.5508	104.357	104.614
17265	3.5508	103.716	155.843
17266	3.5509	85.032	334.962
17267	3.551	100.115	292.048
17268	3.551	82.781	250.572
17269	3.5513	111.074	192.945

17270	3.5513	77.6015	116.894
17271	3.5513	106.399	152.178
17272	3.5515	100.397	294.583
17273	3.5516	117.031	160.821
17274	3.5517	90.0902	213.045
17275	3.5517	104.72	193.526
17276	3.5517	101.515	70.2253
17277	3.5517	92.4149	104.197
17278	3.5518	58.7075	166.376
17279	3.5518	86.1702	173.34
17280	3.5518	54.7465	186.997
17281	3.5519	110.983	312.652
17282	3.5519	97.3894	126.763
17283	3.5519	112.51	92.7143
17284	3.552	90.0252	199.617
17285	3.552	82.2216	16.1066
17286	3.5521	113.326	222.325
17287	3.5522	105.78	31.8767
17288	3.5522	27.6905	344.974
17289	3.5524	67.8201	36.9002
17290	3.5524	85.5628	186.891
17291	3.5525	107.114	131.372
17292	3.5525	71.5999	186.364
17293	3.5527	83.0997	306.624
17294	3.5527	111.158	26.995
17295	3.5527	89.1533	46.0892
17296	3.5527	82.0426	289.856
17297	3.5529	95.4448	230.056
17298	3.553	-2.8607	92.692
17299	3.5531	91.533	159.92
17300	3.5532	101.795	207.162
17301	3.5532	37.2001	149.456
17302	3.5533	81.7086	170.875
17303	3.5534	94.0147	260.634
17304	3.5534	96.025	153.646
17305	3.5535	84.2358	229.344
17306	3.5535	89.5355	255.868
17307	3.5535	120.159	142.732
17308	3.5536	104.231	296.363
17309	3.5536	93.7934	119.122
17310	3.5536	90.2112	136.045
17311	3.5536	100.601	84.5816
17312	3.5538	93.8199	119.106
17313	3.554	86.1419	58.4409
17314	3.5541	96.07	142.663
17315	3.5542	-1.2113	235.859
17316	3.5542	89.9825	157.812
17317	3.5542	118.598	187.29

17318	3.5542	100.825	138.409
17319	3.5544	125.77	312.777
17320	3.5545	76.0943	33.3117
17321	3.5545	86.1047	189.192
17322	3.5545	110.275	138.598
17323	3.5546	83.6797	106.506
17324	3.5546	93.0479	127.003
17325	3.5548	3.344	291.827
17326	3.555	124.922	38.9634
17327	3.5551	102.887	38.8492
17328	3.5553	75.2938	213.339
17329	3.5556	102.05	154.193
17330	3.5556	93.5318	279.69
17331	3.5556	96.9422	131.997
17332	3.5557	92.874	69.3998
17333	3.5559	115.523	164.742
17334	3.5561	105.627	199.456
17335	3.5561	113.664	215.186
17336	3.5562	138.781	65.93
17337	3.5562	77.0961	111.306
17338	3.5562	-2.7767	96.645
17339	3.5564	-2.7945	120.232
17340	3.5565	89.0914	147.242
17341	3.5565	87.8561	127.441
17342	3.5565	94.0312	301.439
17343	3.5567	109.957	315.952
17344	3.5569	84.0197	216.174
17345	3.5569	115.009	186.769
17346	3.5571	94.6352	244.426
17347	3.5574	98.7486	166.8
17348	3.5575	112.071	31.4794
17349	3.5577	110.426	63.2289
17350	3.5578	99.3969	46.0337
17351	3.5578	84.8885	219.41
17352	3.5578	-4.077	139.702
17353	3.5579	118.179	200.013
17354	3.5579	99.4085	329.543
17355	3.558	-2.8185	138.289
17356	3.5582	112.094	81.9946
17357	3.5583	50.3499	279.005
17358	3.5584	116.266	56.3461
17359	3.5587	90.3922	295.101
17360	3.5587	97.5789	151.192
17361	3.5588	95.7136	57.2587
17362	3.5589	81.9755	185.657
17363	3.5589	100.978	19.8503
17364	3.5591	121.743	27.4461
17365	3.5591	82.277	183.767

17366	3.5592	96.7724	88.6642
17367	3.5593	96.2064	236.8
17368	3.5594	123.908	96.256
17369	3.5594	110.539	311.604
17370	3.5595	96.0101	74.0014
17371	3.5595	116.375	238.039
17372	3.5596	123.593	65.0872
17373	3.5597	111.176	161.259
17374	3.5597	102.778	263.637
17375	3.5597	87.2646	13.6412
17376	3.5598	99.7748	261.19
17377	3.5599	99.8254	246.412
17378	3.5601	118.2	72.0777
17379	3.5602	96.8835	203.434
17380	3.5603	100.486	328.029
17381	3.5603	94.0915	247.284
17382	3.5603	100.532	211.109
17383	3.5604	101.002	86.4931
17384	3.5605	116.497	349.529
17385	3.5605	113.36	81.8793
17386	3.5605	67.9733	196.611
17387	3.5605	92.322	117.524
17388	3.5606	93.8742	132.828
17389	3.5607	104.973	299.25
17390	3.5607	96.7741	258.817
17391	3.5607	89.2789	250.337
17392	3.5607	98.5931	204.772
17393	3.5607	88.701	293.143
17394	3.5607	103.473	298.145
17395	3.5608	26.6866	33.1535
17396	3.5608	84.2443	266.426
17397	3.5609	90.3582	137.487
17398	3.561	23.0216	166.624
17399	3.561	96.2981	267.051
17400	3.5612	87.362	247.346
17401	3.5613	99.222	301.199
17402	3.5615	107.033	229.724
17403	3.5615	93.7049	133.391
17404	3.5615	110.243	336.592
17405	3.5615	87.9953	136.441
17406	3.5616	94.0173	136.79
17407	3.5616	100.768	116.358
17408	3.5616	98.8729	12.5384
17409	3.5616	136.351	319.93
17410	3.5617	115.674	30.6627
17411	3.5617	122.822	65.5138
17412	3.5618	114.44	109.16
17413	3.5619	82.202	34.6783

17414	3.562	114.879	2.8254
17415	3.562	94.6141	206.556
17416	3.562	78.0059	147.412
17417	3.5621	99.8425	170.444
17418	3.5621	95.872	134.38
17419	3.5623	99.4518	139.1
17420	3.5624	-2.7212	18.873
17421	3.5624	103.087	139.958
17422	3.5625	122.582	249.166
17423	3.5627	114.545	190.153
17424	3.5627	79.1724	185.223
17425	3.5629	11.5105	15.8222
17426	3.563	37.5775	229.782
17427	3.5631	115.571	61.2054
17428	3.5631	124.045	205.876
17429	3.5631	94.2832	189.05
17430	3.5632	84.5464	44.5647
17431	3.5633	102.443	109.929
17432	3.5633	85.5353	313.26
17433	3.5633	100.382	197.572
17434	3.5634	124.519	236.508
17435	3.5635	113.446	25.7211
17436	3.5635	120.218	348.105
17437	3.5637	90.2375	152.867
17438	3.5638	99.2283	109.081
17439	3.564	92.7536	84.4515
17440	3.5641	105.876	134.385
17441	3.5641	89.6099	118.391
17442	3.5641	108.114	146.892
17443	3.5643	28.624	241.742
17444	3.5645	120.639	22.953
17445	3.5645	85.033	183.216
17446	3.5645	95.1012	105.645
17447	3.5645	97.4297	241.414
17448	3.5646	69.8427	222.441
17449	3.5647	118.798	82.9848
17450	3.5649	119.099	245.563
17451	3.5649	108.174	81.7592
17452	3.5649	79.4389	232.126
17453	3.565	118.395	314.047
17454	3.565	92.6787	305.898
17455	3.565	100.657	234.821
17456	3.565	102.208	197.576
17457	3.5651	96.837	233.493
17458	3.5651	92.8909	107.542
17459	3.5651	110.708	322.838
17460	3.5651	101.291	178.628
17461	3.5652	103.459	116.845

17462	3.5653	84.9036	248.4
17463	3.5653	95.9717	94.0865
17464	3.5653	99.2918	172.278
17465	3.5653	93.3573	256.351
17466	3.5654	126.629	312.569
17467	3.5654	123.193	242.731
17468	3.5654	102.828	140.967
17469	3.5654	100.943	322.837
17470	3.5655	70.7223	326.196
17471	3.5656	95.6762	300.73
17472	3.5658	77.935	61.0219
17473	3.5658	104.559	229.296
17474	3.5659	93.0911	190.729
17475	3.566	11.7432	187.521
17476	3.5661	83.889	227.156
17477	3.5661	89.873	234.539
17478	3.5662	125.332	45.7705
17479	3.5662	120.502	61.5318
17480	3.5664	105.293	90.9393
17481	3.5664	79.431	342.498
17482	3.5666	105.172	94.0789
17483	3.5667	118.107	114.711
17484	3.5667	87.2703	161.666
17485	3.5668	109.67	99.185
17486	3.5668	103.085	165.851
17487	3.5668	115.762	67.8386
17488	3.5668	88.6456	172.965
17489	3.5669	89.6144	277.955
17490	3.567	86.2682	164.822
17491	3.5671	103.953	122.444
17492	3.5672	115.472	271.51
17493	3.5672	96.2629	187.3
17494	3.5673	85.1507	239.614
17495	3.5673	88.4015	120.485
17496	3.5673	113.911	269.655
17497	3.5677	-2.9626	98.4888
17498	3.5677	99.1244	149.673
17499	3.5679	92.4469	64.1376
17500	3.5679	99.259	114.717
17501	3.568	82.3098	331.298
17502	3.5682	108.815	13.786
17503	3.5683	140.924	206.038
17504	3.5684	131.589	46.8692
17505	3.5684	84.5045	29.4902
17506	3.5684	104.413	326.86
17507	3.5686	11.1103	228.799
17508	3.5686	59.89	154.967
17509	3.5689	-2.5479	265.91

17510	3.5689	92.7341	160.12
17511	3.5692	122.659	22.6417
17512	3.5692	100.67	185.368
17513	3.5693	4.3812	346.235
17514	3.5694	89.3023	18.0782
17515	3.5694	102.73	339.573
17516	3.5694	103.208	160.135
17517	3.5695	98.4396	259.445
17518	3.5696	115.836	108.48
17519	3.57	99.3074	64.3402
17520	3.57	137.977	74.9224
17521	3.57	116.42	89.2402
17522	3.5701	97.6799	110.253
17523	3.5702	82.0578	350.078
17524	3.5703	56.9053	204.037
17525	3.5704	13.562	258.689
17526	3.5705	119.58	103.662
17527	3.5705	90.9145	71.3388
17528	3.5706	101.19	38.9226
17529	3.5706	103.462	163.786
17530	3.5707	91.0685	93.1414
17531	3.5707	131.311	52.4013
17532	3.5708	121.319	338.854
17533	3.5708	122.245	322.948
17534	3.5709	137.892	244.61
17535	3.5709	49.2255	260.825
17536	3.571	98.357	83.8715
17537	3.571	116.319	0.1667
17538	3.571	116.63	150.171
17539	3.5712	114.945	345.661
17540	3.5714	135.145	306.381
17541	3.5714	103.145	82.1361
17542	3.5714	99.472	166.575
17543	3.5714	113.012	216.381
17544	3.5715	88.6783	332.502
17545	3.5716	105.486	236.405
17546	3.5716	99.7458	53.4977
17547	3.5717	90.0659	116.003
17548	3.5717	90.611	170.923
17549	3.5717	125.942	35.2584
17550	3.5718	105.509	304.001
17551	3.5719	122.545	76.1602
17552	3.5719	77.9636	13.0267
17553	3.572	104.626	99.1222
17554	3.5721	131.297	244.106
17555	3.5722	124.79	144.06
17556	3.5723	125.397	68.1406
17557	3.5724	71.7665	143.606

17558	3.5725	108.148	313.726
17559	3.5728	95.7807	200.933
17560	3.5728	94.6262	160.629
17561	3.5729	100.167	75.9179
17562	3.5729	80.1122	167.736
17563	3.573	103.716	123.767
17564	3.5731	83.6284	136.124
17565	3.5731	97.4979	274.884
17566	3.5731	48.9522	155.963
17567	3.5733	133.276	204.624
17568	3.5734	96.7921	104.477
17569	3.5735	93.3328	53.2882
17570	3.5737	90.3157	60.2495
17571	3.5738	99.3264	113.168
17572	3.5738	95.887	261.797
17573	3.5739	104.791	37.4121
17574	3.5739	85.1069	187.856
17575	3.5739	123.784	283.421
17576	3.574	99.816	129.637
17577	3.5742	96.2235	107.092
17578	3.5742	114.888	119.037
17579	3.5743	96.4527	343.668
17580	3.5743	98.501	155.518
17581	3.5744	80.9485	79.1627
17582	3.5745	111.844	24.3057
17583	3.5747	104.828	201.303
17584	3.5747	115.895	111.16
17585	3.5747	129.128	159.216
17586	3.5749	92.1045	196.093
17587	3.5749	120.897	286.925
17588	3.5751	105.562	276.161
17589	3.5753	147.427	258
17590	3.5753	102.806	154.537
17591	3.5753	-2.7987	147.735
17592	3.5753	-2.6839	272.962
17593	3.5754	131.129	350.475
17594	3.5754	87.1939	232.849
17595	3.5754	66.8431	147.687
17596	3.5755	113.801	165.035
17597	3.5756	124.005	23.5898
17598	3.5757	125.493	241.461
17599	3.5757	89.8334	307.664
17600	3.5758	125.375	65.9984
17601	3.5758	89.5717	98.5578
17602	3.5759	116.837	31.6955
17603	3.5759	83.4981	55.9754
17604	3.576	82.8637	126.437
17605	3.5762	83.2208	57.5448

17606	3.5762	-2.6899	12.8634
17607	3.5763	-3.2172	168.733
17608	3.5763	103.783	88.1097
17609	3.5764	98.9148	348.719
17610	3.5764	110.012	53.7454
17611	3.5765	95.7685	261.248
17612	3.5765	91.9584	255.499
17613	3.5766	125.849	24.5744
17614	3.5766	-2.7532	299.524
17615	3.5767	55.5017	84.2676
17616	3.5768	100.129	209.442
17617	3.5769	169.135	30.3987
17618	3.5769	114.475	111.826
17619	3.577	81.4939	54.1354
17620	3.577	116.219	126.723
17621	3.5772	95.3694	195.136
17622	3.5772	104.731	161.736
17623	3.5773	133.797	58.5667
17624	3.5773	85.5495	158.475
17625	3.5773	-2.7849	55.7542
17626	3.5774	-1.3062	52.9418
17627	3.5774	72.3869	115.981
17628	3.5775	94.8067	53.6153
17629	3.5777	106.777	284.854
17630	3.5779	113.062	196.323
17631	3.578	105.599	311.735
17632	3.578	101.591	315.402
17633	3.5781	107.277	210.214
17634	3.5782	117.123	145.884
17635	3.5782	109.032	81.1746
17636	3.5782	111.207	109.29
17637	3.5783	110.894	308.074
17638	3.5783	110.762	4.3821
17639	3.5784	137.338	33.1283
17640	3.5784	132.363	290.724
17641	3.5785	99.6593	222.641
17642	3.5786	122.738	212.027
17643	3.5786	88.4497	149.366
17644	3.5787	98.4374	136.881
17645	3.5788	130.475	109.768
17646	3.5788	124.721	108.723
17647	3.5788	111.13	300.366
17648	3.5789	101.837	102.67
17649	3.579	98.7577	44.8431
17650	3.579	150.256	206.577
17651	3.5791	100.405	100.947
17652	3.5792	-2.7041	28.9429
17653	3.5792	106.89	117.13

17654	3.5793	126.064	68.0358
17655	3.5793	92.5854	111.278
17656	3.5795	106.564	91.7904
17657	3.5795	95.8138	107.806
17658	3.5796	86.2432	168.735
17659	3.5796	103.723	110.975
17660	3.5796	94.7924	105.078
17661	3.5798	134.475	310.041
17662	3.5799	104.567	81.0799
17663	3.58	81.345	49.4088
17664	3.58	105.163	191.284
17665	3.5801	132.697	57.5232
17666	3.5801	99.5244	256.615
17667	3.5801	101.02	122.602
17668	3.5802	17.1032	287.212
17669	3.5802	90.0425	226.149
17670	3.5803	89.6072	318.701
17671	3.5805	98.7331	72.917
17672	3.5806	90.2197	187.989
17673	3.5807	98.4941	157.564
17674	3.5807	106.916	139.704
17675	3.5808	136.079	70.2864
17676	3.5808	85.844	116.822
17677	3.5809	92.0933	143.075
17678	3.581	126.446	145.892
17679	3.581	122.643	203.68
17680	3.581	-2.7914	106.424
17681	3.5811	82.8676	13.256
17682	3.5812	122.928	57.1325
17683	3.5813	87.4813	198.513
17684	3.5813	130.213	354.344
17685	3.5813	106.521	40.5428
17686	3.5814	99.4122	269.061
17687	3.5815	123.537	340.867
17688	3.5815	110.896	103.232
17689	3.5815	98.5854	300.909
17690	3.5816	111.195	167.874
17691	3.5817	110.985	80.8214
17692	3.5817	110.696	68.8712
17693	3.5818	101.908	144.243
17694	3.5819	104.867	5.0133
17695	3.5819	81.8406	183.388
17696	3.5819	98.0432	97.6715
17697	3.5819	104.845	50.6077
17698	3.582	120.989	216.291
17699	3.582	91.902	124.316
17700	3.5823	117.283	256.556
17701	3.5823	118.653	34.5872

17702	3.5823	104.293	142.124
17703	3.5823	81.2875	90.3011
17704	3.5824	107.325	306.727
17705	3.5824	88.0906	119.966
17706	3.5824	117.898	354.36
17707	3.5825	119.785	63.8803
17708	3.5826	105.64	87.2641
17709	3.5827	113.503	325.033
17710	3.5828	118.123	226.055
17711	3.5829	92.7867	193.032
17712	3.5829	110.636	157.427
17713	3.583	96.8619	50.594
17714	3.5831	101.707	157.152
17715	3.5831	106.462	242.611
17716	3.5833	11.5468	75.6759
17717	3.5834	95.4952	102.246
17718	3.5834	77.0337	68.2979
17719	3.5835	108.54	107.868
17720	3.5837	91.1384	250.438
17721	3.5837	78.3514	162.628
17722	3.5837	109.261	94.8199
17723	3.5838	127.18	330.967
17724	3.5841	117.025	168.74
17725	3.5841	107.67	20.9974
17726	3.5841	99.7006	235.152
17727	3.5842	105.46	135.416
17728	3.5844	111.864	270.206
17729	3.5845	91.4516	105.471
17730	3.5846	100.141	134.816
17731	3.5846	86.7987	317.825
17732	3.5847	135.397	338.037
17733	3.5847	104.192	278.563
17734	3.5848	138.644	39.658
17735	3.5848	90.4805	122.195
17736	3.5848	92.2926	64.8503
17737	3.5848	82.7181	160.165
17738	3.5849	112.935	77.3471
17739	3.5849	118.113	266.1
17740	3.5849	76.5045	94.4702
17741	3.585	107.831	92.1984
17742	3.585	89.6449	92.2171
17743	3.5851	102.854	60.2239
17744	3.5851	92.4464	152.758
17745	3.5853	119.194	43.9149
17746	3.5853	120.633	268.436
17747	3.5853	86.9419	126.02
17748	3.5853	113.775	157.839
17749	3.5855	105.269	293.903

17750	3.5856	114.372	295.618
17751	3.5857	115.376	337.288
17752	3.5857	81.4047	321.04
17753	3.5858	101.669	155.145
17754	3.5858	101.416	93.3522
17755	3.5858	77.4645	140.353
17756	3.586	111.555	111.871
17757	3.586	94.9138	190.231
17758	3.5861	112.022	251.708
17759	3.5861	128.65	34.4009
17760	3.5864	97.2507	118.948
17761	3.5867	136.122	24.6664
17762	3.5867	99.9628	189.433
17763	3.5867	110.58	283.185
17764	3.5869	98.3992	121.953
17765	3.5869	81.1347	278.794
17766	3.587	102.137	82.4868
17767	3.587	82.0528	233.974
17768	3.5871	100.168	353.126
17769	3.5871	100.801	137.017
17770	3.5871	118.684	144.021
17771	3.5874	96.8146	129.002
17772	3.5874	94.9843	262.202
17773	3.5874	47.1557	135.685
17774	3.5875	124.048	130.477
17775	3.5875	95.2649	38.4367
17776	3.5876	131.738	245.396
17777	3.5876	98.9995	45.427
17778	3.5877	91.1223	226.273
17779	3.5878	114.978	188.715
17780	3.5879	103.855	211.277
17781	3.588	81.1758	148.917
17782	3.588	115.924	101.618
17783	3.5882	85.9947	108.486
17784	3.5882	109.393	294.444
17785	3.5883	134.118	22.5311
17786	3.5883	132.789	45.3013
17787	3.5884	152.621	315.059
17788	3.5885	80.3066	333.758
17789	3.5885	103.891	271.302
17790	3.5886	105.192	126.14
17791	3.5886	107.694	38.5159
17792	3.5887	109.718	50.8763
17793	3.5887	96.6862	221.541
17794	3.5887	112.229	18.0958
17795	3.5888	128.126	37.7812
17796	3.5888	99.7261	211.59
17797	3.5888	99.2528	209.601

17798	3.5889	113.146	14.7162
17799	3.5889	109.35	97.3125
17800	3.5889	88.8241	199.779
17801	3.589	134.293	39.2725
17802	3.589	100.459	157.493
17803	3.5892	107.77	314.94
17804	3.5894	80.9572	77.8201
17805	3.5895	119.146	318.102
17806	3.5895	96.6286	155.591
17807	3.5895	101.604	310.446
17808	3.5895	55.8414	98.5393
17809	3.5896	100.437	183.685
17810	3.5897	90.2633	253.466
17811	3.5898	114.811	84.1929
17812	3.5899	102.928	111.826
17813	3.59	101.941	102.524
17814	3.5901	118.574	66.3361
17815	3.5901	111.466	114.053
17816	3.5901	-2.8435	135.517
17817	3.5903	117.771	9.3829
17818	3.5905	76.2876	164.951
17819	3.5906	94.2497	227.313
17820	3.5907	89.4561	54.5068
17821	3.5908	88.389	308.468
17822	3.5909	-3.0144	193.117
17823	3.5909	72.7129	197.631
17824	3.5912	87.3921	235.652
17825	3.5914	92.3361	194.733
17826	3.5914	115.666	65.5089
17827	3.5915	116.959	312.234
17828	3.5915	112.484	97.9681
17829	3.5915	105.812	234.94
17830	3.5916	82.8406	30.5759
17831	3.5917	107.479	105.63
17832	3.5917	97.7104	188.872
17833	3.5917	105.858	65.9432
17834	3.5917	52.3931	134.545
17835	3.5917	118.368	335.119
17836	3.592	118.558	166.32
17837	3.5922	87.1826	4.5733
17838	3.5922	72.398	120.801
17839	3.5923	94.3832	195.903
17840	3.5924	114.037	281.158
17841	3.5925	118.897	37.3162
17842	3.5927	131.929	294.398
17843	3.5927	126.067	197.197
17844	3.5927	88.588	194.421
17845	3.5928	113.423	346.853

17846	3.5928	87.8034	151.475
17847	3.5928	131.985	87.5463
17848	3.5928	105.968	315.387
17849	3.5929	116.212	243.167
17850	3.5929	106.732	295.594
17851	3.593	136.374	6.2735
17852	3.593	97.2604	127.389
17853	3.593	-2.7654	289.37
17854	3.5931	109.722	51.9324
17855	3.5931	80.3439	183.411
17856	3.5933	101.133	100.202
17857	3.5934	102.098	56.1082
17858	3.5935	106.825	237.931
17859	3.594	109.206	253.114
17860	3.594	88.725	111.108
17861	3.594	93.1041	226.418
17862	3.594	100.267	300.329
17863	3.5941	107.101	37.6313
17864	3.5942	108.518	218.43
17865	3.5943	109.665	135.799
17866	3.5943	-3.9056	60.2235
17867	3.5943	119.08	31.3851
17868	3.5944	116.705	83.1937
17869	3.5945	102.19	145.275
17870	3.5945	104.776	134.909
17871	3.5947	73.148	89.0695
17872	3.5948	36.6395	215.041
17873	3.5948	106.164	98.2307
17874	3.5948	101.649	164.007
17875	3.5949	100.833	98.2444
17876	3.5951	117.96	292.228
17877	3.5951	108.187	53.0164
17878	3.5951	113.474	273.166
17879	3.5952	69.3446	65.0827
17880	3.5953	118.043	276.154
17881	3.5953	102.009	45.8011
17882	3.5954	96.5523	134.944
17883	3.5955	145.118	31.854
17884	3.5955	87.0275	134.897
17885	3.5958	113.999	73.9072
17886	3.5959	116.241	318.641
17887	3.5959	129.979	295.623
17888	3.5959	108.297	157.819
17889	3.5962	102.325	260.342
17890	3.5963	86.6772	209.612
17891	3.5963	98.6958	71.6128
17892	3.5963	99.2817	159.807
17893	3.5967	77.8414	26.9106

17894	3.5967	96.3158	89.9207
17895	3.5968	117.109	103.46
17896	3.5968	96.6164	117.229
17897	3.5968	84.439	330.964
17898	3.5969	7.0868	334.727
17899	3.5969	118.805	268.337
17900	3.5969	109.745	183.834
17901	3.597	118.901	198.057
17902	3.597	91.7473	96.3613
17903	3.597	115.005	10.3199
17904	3.5971	101.401	95.3014
17905	3.5971	82.6941	164.887
17906	3.5972	10.2554	164.805
17907	3.5973	128.618	60.4995
17908	3.5975	118.931	296.533
17909	3.5976	140.523	34.5314
17910	3.5976	129.536	200.992
17911	3.5977	90.3072	124.262
17912	3.5978	100.897	229.841
17913	3.598	93.3493	48.8947
17914	3.5981	109.187	198.901
17915	3.5981	-2.8039	283.395
17916	3.5981	103.267	42.3598
17917	3.5982	94.887	36.6657
17918	3.5982	92.8923	144.067
17919	3.5983	105.484	305.058
17920	3.5983	90.6454	143.095
17921	3.5983	113.473	146.192
17922	3.5985	118.732	67.4054
17923	3.5986	107.492	111.367
17924	3.5986	87.8357	153.506
17925	3.5987	82.5879	6.417
17926	3.5988	124.788	76.6985
17927	3.5991	95.1643	195.686
17928	3.5991	113.736	76.3217
17929	3.5991	109.248	159.614
17930	3.5992	-0.6269	160.774
17931	3.5995	107.666	91.444
17932	3.5995	119.462	112
17933	3.5996	85.9035	127.645
17934	3.5998	109.869	120.392
17935	3.6	122.699	241.87
17936	3.6	-2.748	287.344
17937	3.6	120.652	66.2339
17938	3.6001	69.1676	147.59
17939	3.6002	100.211	226.371
17940	3.6003	86.269	117.732
17941	3.6003	86.7564	60.6785

17942	3.6003	95.6124	146.816
17943	3.6003	87.6544	98.6516
17944	3.6004	95.8269	14.7053
17945	3.6004	115.963	230.906
17946	3.6005	121.069	125.42
17947	3.6006	103.476	81.8491
17948	3.6006	87.3741	183.895
17949	3.6006	98.809	273.729
17950	3.6006	95.4832	110.988
17951	3.6006	109.504	146.406
17952	3.6006	91.8402	15.8895
17953	3.6007	132.455	72.0968
17954	3.6007	104.698	294.236
17955	3.601	118.403	107.076
17956	3.601	54.9507	193.899
17957	3.601	143.362	305.803
17958	3.6011	69.8387	120.739
17959	3.6011	125.509	292.396
17960	3.6013	107.173	172.998
17961	3.6014	118.862	272.825
17962	3.6014	108.07	199.748
17963	3.6014	96.0851	117.326
17964	3.6014	94.9639	98.178
17965	3.6015	122.199	254.332
17966	3.6015	105.753	111.786
17967	3.6016	89.6517	156.392
17968	3.6016	-4.2341	131.81
17969	3.6018	67.3345	115.413
17970	3.6018	93.4441	72.117
17971	3.6019	109.995	228.974
17972	3.6019	87.0578	230.218
17973	3.6019	137.691	200.339
17974	3.602	105.975	53.4136
17975	3.602	88.3721	311.357
17976	3.6023	93.1053	5.7622
17977	3.6023	117.761	56.8188
17978	3.6024	108.627	88.1633
17979	3.6025	122.7	19.9756
17980	3.6026	76.0838	36.9113
17981	3.6028	107.984	131.684
17982	3.6029	80.3244	252.086
17983	3.6029	97.7795	166.505
17984	3.6031	131.717	317.332
17985	3.6031	95.6362	119.364
17986	3.6031	94.0937	5.8254
17987	3.6031	96.4193	34.2688
17988	3.6032	89.9126	179.411
17989	3.6033	85.7373	175.125

17990	3.6033	97.8313	125.855
17991	3.6033	112.676	305.811
17992	3.6034	105.463	252.534
17993	3.6034	102.623	293.828
17994	3.6035	113.886	231.803
17995	3.6036	106.033	125.397
17996	3.6037	143.694	37.9213
17997	3.6037	118.132	58.6629
17998	3.6038	119.001	169.088
17999	3.6038	111.161	294.797
18000	3.6039	91.7324	195.581
18001	3.6039	114.856	54.1276
18002	3.6039	95.2164	178.084
18003	3.6042	115.347	56.1653
18004	3.6042	106.53	174.445
18005	3.6042	103.249	163.555
18006	3.6043	131.064	202.419
18007	3.6043	100.713	130.648
18008	3.6044	118.587	69.756
18009	3.6044	0.9775	327.827
18010	3.6044	89.2782	111.159
18011	3.6044	97.2404	120.883
18012	3.6046	88.4788	115.216
18013	3.6047	94.329	191.775
18014	3.6047	102.915	233.357
18015	3.6048	-4.6518	313.162
18016	3.605	109.464	168.555
18017	3.605	98.1854	254.598
18018	3.6051	82.8727	132.141
18019	3.6052	88.5843	120.168
18020	3.6053	93.6516	12.2919
18021	3.6054	113.225	62.2542
18022	3.6054	96.0215	142.281
18023	3.6054	128.429	52.0724
18024	3.6055	14.5788	248.55
18025	3.6055	114.144	135.03
18026	3.6056	130.89	151.545
18027	3.6056	99.6003	299.182
18028	3.6056	95.0141	155.299
18029	3.6056	105.855	94.336
18030	3.6057	92.825	273.15
18031	3.6057	110.629	135.569
18032	3.6058	95.6038	243.933
18033	3.606	110.812	307.028
18034	3.606	94.2194	259.49
18035	3.6061	117.414	307.091
18036	3.6061	109.291	297.291
18037	3.6062	115.811	53.8835

18038	3.6062	92.0843	98.4702
18039	3.6064	119.693	336.443
18040	3.6065	101.262	58.8223
18041	3.6065	10.068	218.518
18042	3.6065	100.661	119.602
18043	3.6066	121.201	244.289
18044	3.6067	95.0375	235.859
18045	3.6068	80.9591	196.131
18046	3.6068	112.681	103.949
18047	3.607	85.9954	263.192
18048	3.6072	0.5989	35.2001
18049	3.6073	116.968	284.952
18050	3.6073	98.3256	20.2062
18051	3.6074	122.238	58.8397
18052	3.6076	-2.7907	135.584
18053	3.6077	84.2502	159.995
18054	3.6078	121.121	293.211
18055	3.6079	97.1645	82.876
18056	3.6082	114.525	88.4757
18057	3.6082	97.6992	22.4932
18058	3.6083	79.653	130.43
18059	3.6084	91.0086	150.797
18060	3.6086	110.42	135.244
18061	3.6087	109.481	230.575
18062	3.6088	118.829	23.3887
18063	3.6089	126.653	171.607
18064	3.6089	105.877	68.1636
18065	3.6089	96.9889	147.602
18066	3.6089	101.389	119.617
18067	3.6089	106.261	109.237
18068	3.6092	23.8611	145.656
18069	3.6092	112.979	94.0906
18070	3.6092	90.2439	149.062
18071	3.6092	110.274	113.91
18072	3.6093	66.639	302.859
18073	3.6094	93.6211	160.334
18074	3.6097	101.118	142.646
18075	3.6098	87.8504	131.878
18076	3.6098	110.875	339.802
18077	3.61	119.034	63.0423
18078	3.6101	130.73	251.13
18079	3.6102	118.178	263.135
18080	3.6102	110.205	166.474
18081	3.6102	114.78	338.028
18082	3.6104	132.228	93.9024
18083	3.6105	84.9099	92.2394
18084	3.6105	99.3813	119.362
18085	3.6106	101.448	162.38

18086	3.6109	125.032	21.6249
18087	3.6109	79.4294	183.233
18088	3.6109	87.5699	128.895
18089	3.6109	97.1349	100.858
18090	3.6109	84.6387	139.609
18091	3.6109	142.183	203.76
18092	3.611	111.309	110.716
18093	3.611	112.464	133.189
18094	3.6111	106.536	106.58
18095	3.6111	97.5316	153.07
18096	3.6112	105.641	131.057
18097	3.6113	112.831	263.367
18098	3.6113	101.593	51.6278
18099	3.6114	120.263	279.907
18100	3.6117	115.948	264.716
18101	3.612	88.7076	191.87
18102	3.6122	117.318	310.563
18103	3.6122	107.36	292.483
18104	3.6124	111.867	187.775
18105	3.6124	119.112	14.6415
18106	3.6124	95.2065	102.464
18107	3.6125	96.2276	185.681
18108	3.6126	-2.8533	168.117
18109	3.6127	113.235	185.584
18110	3.6127	95.3509	326.1
18111	3.6128	102.422	290.481
18112	3.6128	102.662	291.489
18113	3.6129	94.4347	159.065
18114	3.6129	110.613	183.988
18115	3.613	112.549	218.145
18116	3.613	92.6273	139.314
18117	3.613	62.7558	31.8269
18118	3.6131	143.493	44.117
18119	3.6131	119.812	87.0236
18120	3.6131	77.1807	171.212
18121	3.6131	41.0829	230.546
18122	3.6132	86.0126	187.947
18123	3.6132	99.7513	34.3093
18124	3.6133	105.979	103.705
18125	3.6134	125.072	13.1025
18126	3.6134	99.4809	7.49
18127	3.6134	98.077	30.9044
18128	3.6134	105.675	311.593
18129	3.6134	94.1842	72.457
18130	3.6135	111.454	103.927
18131	3.6136	121.893	112.385
18132	3.6137	88.1266	119.122
18133	3.6137	83.288	131.157

18134	3.6137	112.214	300.747
18135	3.6137	64.9675	175.109
18136	3.6138	93.9889	330.653
18137	3.614	110.859	131.06
18138	3.6141	-3.6592	223.591
18139	3.6141	100.231	127.386
18140	3.6141	57.8784	148.234
18141	3.6141	92.7628	188.167
18142	3.6142	87.0671	136.332
18143	3.6142	146.844	38.6389
18144	3.6142	87.762	169.249
18145	3.6142	-6.576	202.99
18146	3.6142	101.159	76.4734
18147	3.6143	90.5425	296.408
18148	3.6145	113.645	299.998
18149	3.6147	107.831	190.854
18150	3.6147	108.871	18.2659
18151	3.6147	106.671	56.3281
18152	3.6148	133.046	64.3706
18153	3.6148	124.792	149.413
18154	3.6148	109.305	96.6906
18155	3.6148	102.823	115.991
18156	3.6149	51.6487	133.228
18157	3.6149	111.819	298.656
18158	3.615	110.658	249.146
18159	3.6152	95.0311	279.168
18160	3.6154	102.021	31.8869
18161	3.6154	132.965	102.502
18162	3.6156	-2.8579	31.9767
18163	3.6156	120.252	134.265
18164	3.6157	86.2525	165.011
18165	3.6158	110.9	269.241
18166	3.6159	70.6961	13.4967
18167	3.6159	58.8826	134.084
18168	3.616	103.889	77.1213
18169	3.616	83.9067	199.981
18170	3.616	94.527	114.469
18171	3.616	114.267	117.417
18172	3.616	112.465	337.273
18173	3.6161	83.7628	162.153
18174	3.6161	4.7037	20.6074
18175	3.6161	136.433	217.979
18176	3.6161	88.3369	119.646
18177	3.6163	99.3405	316.418
18178	3.6163	65.9051	96.3325
18179	3.6163	116.409	99.6733
18180	3.6164	132.697	297.978
18181	3.6165	104.837	192.352

18182	3.6166	110.535	153.059
18183	3.6167	149.587	201.171
18184	3.6167	-2.7853	277.238
18185	3.6168	110.968	221.871
18186	3.6168	119.788	80.8576
18187	3.6168	98.521	145.545
18188	3.6168	119.806	274.685
18189	3.6169	89.1592	157.799
18190	3.617	97.9763	154.775
18191	3.617	116.034	26.3128
18192	3.6172	100.683	38.9502
18193	3.6174	113.415	76.8927
18194	3.6175	116.761	101.334
18195	3.6175	102.433	139.175
18196	3.6176	99.1815	136.232
18197	3.6176	107.799	284.411
18198	3.6176	142.285	316.528
18199	3.6177	105.019	145.871
18200	3.6177	97.8834	81.8935
18201	3.6179	-2.756	33.7871
18202	3.6179	116.123	304.84
18203	3.6183	101.767	111.732
18204	3.6183	61.5105	6.9004
18205	3.6184	111.979	111.649
18206	3.6184	96.9033	145.468
18207	3.6184	93.9518	320.422
18208	3.6185	143.519	206.228
18209	3.6185	68.9691	121.028
18210	3.6185	101.787	323.54
18211	3.6185	72.1848	142.673
18212	3.6185	131.732	45.1539
18213	3.6186	104.936	1.3838
18214	3.6192	86.1257	117.06
18215	3.6193	122.179	263.686
18216	3.6194	141.29	55.6804
18217	3.6195	111.138	77.6012
18218	3.6195	114.098	4.0455
18219	3.6196	118.44	314.705
18220	3.6198	104.748	38.3396
18221	3.62	127.103	280.092
18222	3.6203	126.853	113.209
18223	3.6203	98.5892	197.262
18224	3.6204	135.728	7.5315
18225	3.6205	109.216	291.317
18226	3.6206	90.2375	56.5268
18227	3.6207	97.5828	82.4206
18228	3.6208	95.9644	36.9144
18229	3.6209	134.011	25.4074

18230	3.621	84.9868	184.602
18231	3.621	129.216	50.1326
18232	3.621	107.979	75.0612
18233	3.6211	113.702	26.8972
18234	3.6212	119.701	172.666
18235	3.6212	107.956	112.578
18236	3.6214	96.4816	169.045
18237	3.6214	125.257	37.1875
18238	3.6215	96.1317	111.741
18239	3.6216	111.801	351.777
18240	3.6216	101.342	288.828
18241	3.6216	95.5197	333.961
18242	3.6216	99.6501	259.65
18243	3.6217	96.5641	324.666
18244	3.6217	-2.7696	191.363
18245	3.6217	78.1611	142.654
18246	3.6217	112.187	128.047
18247	3.6218	100.137	133.464
18248	3.6219	-0.8582	96.7094
18249	3.622	125.059	58.6456
18250	3.622	59.8664	357.604
18251	3.6221	132.32	29.682
18252	3.6221	127.228	5.0857
18253	3.6221	102.799	325.394
18254	3.6223	70.514	259.979
18255	3.6226	77.8049	15.198
18256	3.6226	83.7276	152.801
18257	3.6227	115.972	30.7119
18258	3.6228	119.679	61.3781
18259	3.6228	121.056	314.84
18260	3.6229	62.6208	41.1443
18261	3.6229	100.178	255.512
18262	3.6229	110.297	340.745
18263	3.6231	111.336	152.897
18264	3.6231	84.8417	93.5978
18265	3.6231	105.554	111.719
18266	3.6232	97.814	109.524
18267	3.6234	136.127	315.217
18268	3.6234	115.556	152.679
18269	3.6235	123.863	161.106
18270	3.6235	91.8223	58.8355
18271	3.6235	122.767	35.8626
18272	3.6236	104.344	41.3559
18273	3.6236	131.269	290.696
18274	3.6237	114.427	107.677
18275	3.6237	111.288	298.927
18276	3.6237	102.466	82.2837
18277	3.6238	102.247	44.9399

18278	3.6238	51.7189	66.9987
18279	3.6239	108.196	114.481
18280	3.624	128.175	212.076
18281	3.624	131.92	215.402
18282	3.6241	102.468	172.759
18283	3.6242	97.8438	332.074
18284	3.6242	-3.2349	91.4568
18285	3.6242	70.9823	260.81
18286	3.6243	-5.0706	145.309
18287	3.6244	-4.512	279.432
18288	3.6245	134.558	33.3807
18289	3.6245	120.404	243.526
18290	3.6246	111.873	95.1279
18291	3.6247	-1.8552	168.452
18292	3.6248	104.172	21.9034
18293	3.6249	79.3541	142.919
18294	3.625	117.513	107.093
18295	3.6252	133.305	194.482
18296	3.6252	112.39	115.046
18297	3.6252	115.869	57.6047
18298	3.6253	121.441	319.336
18299	3.6253	111.375	232.353
18300	3.6253	100.672	157.489
18301	3.6253	79.4409	191.823
18302	3.6256	93.3376	172.696
18303	3.6256	92.4151	157.844
18304	3.6256	95.9213	336.674
18305	3.6257	116.59	308.181
18306	3.6257	82.6516	148.529
18307	3.6258	100.73	165.942
18308	3.6258	109.935	15.098
18309	3.6261	86.9179	62.9615
18310	3.6263	101.784	109.956
18311	3.6263	96.0781	300.578
18312	3.6265	119.297	154.637
18313	3.6266	112.942	260.309
18314	3.6267	115.571	126.407
18315	3.6268	125.347	30.5139
18316	3.6268	145.437	238.993
18317	3.6268	84.5262	47.9487
18318	3.6268	85.1494	173.427
18319	3.627	71.2236	119.78
18320	3.6271	114.901	102.52
18321	3.6271	119.315	201.195
18322	3.6272	124.894	201.991
18323	3.6272	111.214	229.566
18324	3.6272	88.3131	12.2476
18325	3.6273	98.8073	114.489

18326	3.6273	130.02	126.415
18327	3.6274	100.341	241.977
18328	3.6276	89.1627	50.606
18329	3.6276	102.963	194.098
18330	3.6277	121.725	149.118
18331	3.6277	136.844	216.523
18332	3.6277	100.593	122.333
18333	3.6278	103.181	123.436
18334	3.6279	115.195	293.208
18335	3.6279	105.106	173.903
18336	3.628	73.0504	55.3776
18337	3.628	93.7417	57.9129
18338	3.6281	-2.8185	114.992
18339	3.6282	97.8511	170.4
18340	3.6284	110.331	158.551
18341	3.6284	102.749	185.69
18342	3.6284	7.5283	55.1119
18343	3.6288	-2.3426	249.351
18344	3.6288	118.556	351.021
18345	3.629	134.58	243.613
18346	3.629	102.238	136.954
18347	3.6291	109.165	272.936
18348	3.6291	118.241	300.571
18349	3.6293	100.738	275.88
18350	3.6293	100.76	57.5301
18351	3.6293	104.599	151.97
18352	3.6293	89.2172	75.8018
18353	3.6294	83.4838	200.956
18354	3.6294	113.041	262.269
18355	3.6295	110.721	42.7277
18356	3.6295	90.9475	276.163
18357	3.6296	78.2168	19.3971
18358	3.6297	148.766	53.8555
18359	3.6297	107.948	301.508
18360	3.6299	121.747	307.753
18361	3.63	118.695	215.348
18362	3.6301	128.913	47.6599
18363	3.6302	121.419	124.537
18364	3.6302	125.852	216.98
18365	3.6302	108.292	140.406
18366	3.6303	101.644	191.726
18367	3.6304	127.394	144.355
18368	3.6304	109.518	195.2
18369	3.6305	109.243	275.134
18370	3.6305	124.508	226.01
18371	3.6306	116.785	184.914
18372	3.6307	110.401	300.042
18373	3.6307	109.14	145.768

18374	3.6307	103.597	174.612
18375	3.6307	109.256	288.907
18376	3.6307	96.7308	306.4
18377	3.6308	107.114	113.199
18378	3.6309	138.902	31.7737
18379	3.631	111.295	71.525
18380	3.6312	110.909	232.168
18381	3.6313	135.229	43.3727
18382	3.6314	100.567	158.372
18383	3.6314	102.322	328.772
18384	3.6314	121.306	312.05
18385	3.6316	106.96	99.4951
18386	3.6316	127.265	151.141
18387	3.6317	105.777	251.521
18388	3.6318	109.146	305.104
18389	3.6319	-3.9252	147.057
18390	3.632	106.81	124.337
18391	3.632	115.495	86.0875
18392	3.6322	122.458	351.833
18393	3.6322	108.684	68.9769
18394	3.6323	129.773	26.2173
18395	3.6324	57.5771	191.996
18396	3.6324	89.7229	126.2
18397	3.6326	-3.5696	194.642
18398	3.6328	116.631	90.2055
18399	3.6329	126.935	242.796
18400	3.6332	107.676	140.793
18401	3.6332	75.6513	129.922
18402	3.6333	118.123	120.837
18403	3.6336	119.513	59.5177
18404	3.6336	93.6572	187.217
18405	3.6336	99.0825	163.191
18406	3.6337	132.514	260.957
18407	3.6338	109.555	220.967
18408	3.6339	86.8466	200.018
18409	3.6339	100.929	222.183
18410	3.6339	94.4226	146.884
18411	3.634	113.391	265.885
18412	3.6341	85.2951	272.229
18413	3.6341	90.8944	128.639
18414	3.6342	92.0987	166.82
18415	3.6343	95.5572	56.9032
18416	3.6344	-2.72	317.685
18417	3.6344	125.286	150.903
18418	3.6345	122.76	126.184
18419	3.6346	76.1733	152.553
18420	3.6346	110.93	246.327
18421	3.6347	115.828	18.9449

18422	3.6347	134.267	83.1183
18423	3.6347	-2.7941	115.05
18424	3.6349	148.228	102.979
18425	3.6349	122.791	149.709
18426	3.6349	110.54	121.899
18427	3.6351	112.746	231.28
18428	3.6353	110.868	354.158
18429	3.6354	76.1548	296.299
18430	3.6354	101.934	204.183
18431	3.6355	119.14	350.433
18432	3.6356	106.865	194.904
18433	3.6356	105.442	153.285
18434	3.6356	-2.7213	28.6169
18435	3.6357	138.423	24.2509
18436	3.6357	110.456	109.604
18437	3.6357	113.098	148.447
18438	3.6357	120.428	133.696
18439	3.6358	116.933	258.997
18440	3.6358	94.9885	176.209
18441	3.6359	100.995	166.234
18442	3.636	136.687	106.317
18443	3.6361	108.245	321.325
18444	3.6361	90.8265	175.464
18445	3.6362	99.0143	60.5901
18446	3.6363	120.28	289.442
18447	3.6363	123.675	43.2907
18448	3.6363	94.2764	260.131
18449	3.6364	91.8879	105.742
18450	3.6364	93.0493	234.949
18451	3.6365	127.581	266.175
18452	3.6366	91.4865	76.2244
18453	3.6369	-2.7716	123.904
18454	3.637	106.818	177.431
18455	3.6371	107.245	136.647
18456	3.6373	137.857	286.351
18457	3.6373	106.232	113.034
18458	3.6373	103.356	222.247
18459	3.6374	99.4792	73.1234
18460	3.6374	6.1269	104.784
18461	3.6375	107.845	78.6058
18462	3.6376	129.192	288.883
18463	3.6379	101.434	91.8177
18464	3.638	115.214	27.1916
18465	3.638	95.0982	160.56
18466	3.6381	111.404	118.919
18467	3.6381	103.883	166.318
18468	3.6382	134.896	56.705
18469	3.6383	97.0931	106.604

18470	3.6384	92.6253	134.837
18471	3.6386	100.666	325.036
18472	3.6386	109.477	234.036
18473	3.6386	102.794	156.701
18474	3.6386	97.2841	211.148
18475	3.6388	116.355	138.014
18476	3.6389	89.5274	268.203
18477	3.639	95.8661	219.896
18478	3.6391	129.251	41.5216
18479	3.6391	112.445	295.681
18480	3.6392	121.336	267.918
18481	3.6393	-3.1679	205.751
18482	3.6397	124.006	83.5532
18483	3.6397	124.049	308.129
18484	3.6397	114.188	273.346
18485	3.6398	144.971	42.2056
18486	3.6399	85.0508	46.9704
18487	3.64	102.784	1.1413
18488	3.6402	111.325	200.099
18489	3.6404	119.008	121.283
18490	3.6405	125.835	246.386
18491	3.6405	113.301	151.312
18492	3.6406	-2.8112	140.417
18493	3.6408	60.3218	113.905
18494	3.6408	119.733	67.3168
18495	3.6409	103.978	158.77
18496	3.6409	-2.732	34.4254
18497	3.641	114.04	104.226
18498	3.641	105.378	259.271
18499	3.6412	-2.7507	30.711
18500	3.6413	94.2663	134.631
18501	3.6414	119.601	21.2124
18502	3.6414	120.705	277.463
18503	3.6415	102.758	354.738
18504	3.6416	107.167	165.645
18505	3.6416	108.747	217.111
18506	3.6418	141.028	140.875
18507	3.6418	122.234	242.884
18508	3.6418	128.306	190.136
18509	3.642	99.9333	110.991
18510	3.642	115.623	182.844
18511	3.6421	142.562	57.4426
18512	3.6425	116.418	239.103
18513	3.6425	106.542	293.819
18514	3.6426	107.651	232.346
18515	3.6427	126.991	109.11
18516	3.6427	109.566	118.715
18517	3.6428	119.205	83.6858

18518	3.6428	111.544	274.987
18519	3.6429	98.6184	270.133
18520	3.643	82.9521	168.494
18521	3.6431	97.7035	57.4797
18522	3.6431	140.335	195.068
18523	3.6432	93.4439	161.288
18524	3.6433	118.207	290.522
18525	3.6433	109.419	187.756
18526	3.6434	80.5277	169.948
18527	3.6437	112.282	211.634
18528	3.6437	116.831	108.63
18529	3.6437	168.492	286.889
18530	3.6439	100.712	258.335
18531	3.6439	114.189	183.751
18532	3.644	-2.688	3.5378
18533	3.6442	77.7979	264.887
18534	3.6444	129.366	81.8177
18535	3.6444	116.519	125.132
18536	3.6444	127.519	46.8836
18537	3.6445	100.05	141.185
18538	3.6446	-2.8076	202.502
18539	3.6446	104.471	180.99
18540	3.6446	98.6018	282.016
18541	3.6447	104.978	146.263
18542	3.6449	107.725	53.7103
18543	3.6449	96.8716	193.482
18544	3.645	88.5578	171.611
18545	3.645	-3.4907	92.361
18546	3.6451	147.546	90.8463
18547	3.6451	110.492	48.0563
18548	3.6451	141.573	205.383
18549	3.6451	121.126	57.3969
18550	3.6452	117.35	53.9696
18551	3.6453	102.437	58.7705
18552	3.6453	117.973	104.945
18553	3.6454	100.179	164.49
18554	3.6455	132.677	117.578
18555	3.6456	106.164	99.6692
18556	3.6456	97.3211	287.944
18557	3.6457	97.1689	187.889
18558	3.6458	100.502	194.272
18559	3.6458	106.754	98.67
18560	3.6458	99.1592	270.829
18561	3.6458	99.0867	123.967
18562	3.6462	79.7991	35.5004
18563	3.6462	127.859	137.489
18564	3.6463	110.227	351.442
18565	3.6464	131.519	324.151

18566	3.6465	89.4927	179.925
18567	3.6465	111.614	74.4613
18568	3.6467	136.265	123.793
18569	3.6468	110.975	108.116
18570	3.6469	116.331	94.4064
18571	3.6469	-0.44	115.05
18572	3.6469	94.0488	94.1042
18573	3.6469	90.1976	181.765
18574	3.647	134.036	244.594
18575	3.647	117.015	118.381
18576	3.647	126.546	104.278
18577	3.6471	117.342	200.048
18578	3.6473	86.3768	178.139
18579	3.6474	138.618	70.8487
18580	3.6474	110.327	152.711
18581	3.6474	94.7229	131.975
18582	3.6475	-3.4202	107.426
18583	3.6477	100.108	175.306
18584	3.6477	109.069	139.419
18585	3.6478	107.698	112.207
18586	3.6479	55.8004	5.777
18587	3.648	106.06	152.435
18588	3.648	108.294	68.5319
18589	3.6481	107.193	193.751
18590	3.6482	115.172	95.1743
18591	3.6482	109.153	108.968
18592	3.6483	116.923	135.133
18593	3.6483	121.236	89.6753
18594	3.6484	123.675	271.271
18595	3.6484	121.955	26.8068
18596	3.6484	125.883	202.819
18597	3.6486	88.4191	60.6545
18598	3.6486	125.041	4.8873
18599	3.6487	115.765	45.1171
18600	3.6488	125.093	75.8439
18601	3.6488	92.6482	108.696
18602	3.6489	129.599	248.307
18603	3.649	86.9863	246.307
18604	3.649	60.0578	117.534
18605	3.6491	164.073	303.1
18606	3.6491	87.2451	119.287
18607	3.6491	120.248	17.6241
18608	3.6492	136.225	69.6499
18609	3.6492	112.798	240.391
18610	3.6495	72.3531	176.09
18611	3.6495	102.878	279.807
18612	3.6496	111.689	147.228
18613	3.6497	133.544	326.292

18614	3.6498	124.686	123.83
18615	3.6499	138.864	57.221
18616	3.6499	98.756	188.248
18617	3.6499	112.44	156.261
18618	3.65	128.736	144.483
18619	3.6501	90.4159	188.586
18620	3.6502	114.844	158.024
18621	3.6503	118.706	218.44
18622	3.6503	107.888	150.671
18623	3.6504	137.044	210.071
18624	3.6506	91.7565	143.482
18625	3.6508	104.407	160.482
18626	3.6508	120.633	240.481
18627	3.6508	109.467	175.864
18628	3.6509	109.055	51.0807
18629	3.6509	108.894	316.85
18630	3.6509	133.401	321.438
18631	3.651	111.271	260.066
18632	3.6511	91.5615	160.364
18633	3.6511	69.9677	112.449
18634	3.6511	108.399	105.535
18635	3.6514	127.584	93.0579
18636	3.6515	125.057	146.194
18637	3.6517	145.922	104.152
18638	3.6519	108.498	91.8607
18639	3.6519	-4.0536	202.659
18640	3.652	100.467	157.009
18641	3.652	119.202	126.601
18642	3.652	108.713	123.345
18643	3.6521	90.7	195.538
18644	3.6521	116.148	26.3651
18645	3.6522	95.2996	151.573
18646	3.6526	111.597	308.669
18647	3.6526	-2.6735	23.9972
18648	3.6527	119.469	225.31
18649	3.6527	96.4882	141.103
18650	3.6528	132.179	73.0704
18651	3.6529	119.421	94.8106
18652	3.6529	87.1691	140.556
18653	3.6529	111.471	90.6081
18654	3.6529	92.0043	171.733
18655	3.653	70.1662	33.6541
18656	3.653	55.0348	270.956
18657	3.653	107.94	67.4771
18658	3.653	94.2189	142.69
18659	3.653	136.032	211.148
18660	3.653	107.126	243.472
18661	3.6531	138.09	42.3502

18662	3.6531	99.9519	129.369
18663	3.6532	153.071	236.3
18664	3.6533	120.541	171.883
18665	3.6534	151.153	301.79
18666	3.6535	138.387	58.2921
18667	3.6535	107.307	307.248
18668	3.6537	128.93	116.645
18669	3.6537	98.3821	234.418
18670	3.6537	96.0398	135.531
18671	3.6537	126.744	344.784
18672	3.6539	120.896	117.154
18673	3.6539	50.9186	164.305
18674	3.6539	110.882	133.986
18675	3.6539	113.486	211.887
18676	3.6541	110.086	37.5035
18677	3.6542	117.439	247.591
18678	3.6543	111.511	94.8227
18679	3.6544	128.189	6.4204
18680	3.6544	107.963	100.48
18681	3.6544	133.246	90.5575
18682	3.6544	112.791	318.226
18683	3.6545	128.03	41.6895
18684	3.6546	67.7153	132.255
18685	3.6546	96.1955	164.911
18686	3.6547	124.149	103.13
18687	3.6547	106.719	159.312
18688	3.6549	116.868	330.525
18689	3.6549	103.734	141.262
18690	3.6549	111.894	161.698
18691	3.655	106.17	280.289
18692	3.655	126.52	107.521
18693	3.6552	92.2281	308.611
18694	3.6554	117.303	211.427
18695	3.6556	96.8104	211.63
18696	3.6557	104.993	274.104
18697	3.6558	90.401	135.126
18698	3.6558	97.3617	89.088
18699	3.6559	102.654	91.0188
18700	3.6559	128.741	250.361
18701	3.6559	-2.6839	35.2809
18702	3.656	102.201	168.743
18703	3.656	82.4794	172.393
18704	3.656	117.065	40.5575
18705	3.6561	125.359	72.0987
18706	3.6562	103.92	318.729
18707	3.6563	123.858	303.295
18708	3.6565	99.6174	276.966
18709	3.6566	94.9815	183.824

18710	3.6566	92.5924	308.532
18711	3.6567	90.8003	139.322
18712	3.6567	113.683	75.8051
18713	3.6568	121.768	230.693
18714	3.6568	82.8109	148.654
18715	3.657	103.543	191.359
18716	3.657	95.5458	316.06
18717	3.657	102.81	212.102
18718	3.657	116.29	134.921
18719	3.657	148.986	205.219
18720	3.6571	132.141	71.8321
18721	3.6571	-2.7232	21.0235
18722	3.6571	116.979	289.971
18723	3.6574	-2.3203	348.082
18724	3.6574	108.969	279.092
18725	3.6574	135.932	58.2616
18726	3.6575	112.603	229.465
18727	3.6579	100.059	272.752
18728	3.6579	104.855	106.948
18729	3.658	145.922	38.7639
18730	3.6581	105.568	274.647
18731	3.6583	116.229	110.124
18732	3.6583	107.243	187.886
18733	3.6583	100.819	130.792
18734	3.6585	121.609	1.0827
18735	3.6585	108.849	258.652
18736	3.6586	-2.7535	300.148
18737	3.6586	129.073	251.769
18738	3.6587	87.6454	160.563
18739	3.6587	85.982	226.018
18740	3.6588	91.236	118.045
18741	3.6589	114.707	229.57
18742	3.6589	107.635	150.59
18743	3.6589	135.143	52.446
18744	3.659	150.169	201.986
18745	3.659	140.198	223.803
18746	3.6591	98.2205	128.165
18747	3.6592	111.036	101.478
18748	3.6593	112.619	331.218
18749	3.6593	107.251	175.114
18750	3.6595	137.914	128.982
18751	3.6596	87.2339	238.429
18752	3.6597	116.851	289.957
18753	3.6598	94.8823	147.922
18754	3.6599	110.883	113.497
18755	3.66	161.999	197.895
18756	3.6601	118.313	263.098
18757	3.6601	129.92	282.612

18758	3.6602	138.133	24.2957
18759	3.6604	109.578	162.819
18760	3.6604	114.349	136.234
18761	3.6605	116.133	175.901
18762	3.6605	114.561	292.203
18763	3.6606	90.619	88.1911
18764	3.6606	119.934	292.793
18765	3.6606	123.579	68.7831
18766	3.6607	117.472	223.879
18767	3.6608	128.151	79.1563
18768	3.6609	124.353	46.775
18769	3.661	161.576	315.378
18770	3.6611	124.017	59.308
18771	3.6611	117.619	104.941
18772	3.6614	101.789	95.4733
18773	3.6614	110.273	51.6244
18774	3.6615	111.29	90.2278
18775	3.6615	97.3516	219.971
18776	3.6615	109.463	181.354
18777	3.6616	133.749	356.373
18778	3.6616	146.815	70.8523
18779	3.6618	119.733	270.076
18780	3.662	113.127	40.2741
18781	3.6621	118.713	316.382
18782	3.6621	12.3818	297.213
18783	3.6622	-2.8033	164.442
18784	3.6623	155.515	39.8416
18785	3.6623	98.4219	123.11
18786	3.6623	90.7873	183.473
18787	3.6623	87.874	161.383
18788	3.6624	102.72	102.583
18789	3.6624	103.502	210.651
18790	3.6625	120.345	44.5307
18791	3.6625	140.125	53.9772
18792	3.6626	103.662	108.776
18793	3.6628	107.565	356.866
18794	3.663	123.956	59.0769
18795	3.663	101.335	54.7003
18796	3.6632	104.062	157.367
18797	3.6632	129.171	46.5057
18798	3.6632	129.236	61.4109
18799	3.6636	143.13	59.7621
18800	3.6636	102.493	114.727
18801	3.6637	99.7169	332.059
18802	3.6637	140.403	321.419
18803	3.6639	102.177	190.571
18804	3.664	8.6937	219.611
18805	3.664	110.728	101.275

18806	3.6642	101.3	295.835
18807	3.6645	125.097	292.384
18808	3.6645	130.4	20.1286
18809	3.6645	115.59	157.423
18810	3.6646	123.918	157.22
18811	3.6646	71.5715	175.876
18812	3.6647	116.233	227.27
18813	3.6647	132.198	269.578
18814	3.6648	105.55	121.905
18815	3.6648	114.553	172.034
18816	3.6649	-3.562	180.215
18817	3.665	152.79	19.8677
18818	3.665	140.905	42.2544
18819	3.665	107.711	113.232
18820	3.665	119.847	273.372
18821	3.6651	103.26	167.296
18822	3.6651	128.736	47.3858
18823	3.6652	158.141	276.234
18824	3.6653	121.08	211.862
18825	3.6655	98.292	114.163
18826	3.6656	91.1198	197.102
18827	3.6657	118.179	293.129
18828	3.6659	93.2787	99.5052
18829	3.666	119.496	128.611
18830	3.6662	129.267	297.051
18831	3.6662	36.2744	284.419
18832	3.6662	113.047	299.825
18833	3.6663	121.74	149.927
18834	3.6666	125.866	272.035
18835	3.6668	132.84	348.512
18836	3.6668	102.133	162.21
18837	3.6669	96.263	181.022
18838	3.6669	134.173	326.212
18839	3.6669	103.529	109.797
18840	3.6669	109.963	301.645
18841	3.667	134.485	139.652
18842	3.667	118.356	57.9193
18843	3.667	143.078	286.241
18844	3.6671	138.915	312.984
18845	3.6672	92.2653	12.7342
18846	3.6674	15.0857	254.747
18847	3.6674	121.254	194.834
18848	3.6675	108.216	224.26
18849	3.6675	126.17	46.3092
18850	3.6676	112.787	206.509
18851	3.6676	79.3037	125.29
18852	3.6676	116.099	67.4142
18853	3.6677	147.343	145.568

18854	3.6677	143.944	50.9545
18855	3.6677	109.247	322.02
18856	3.6677	98.5377	156.693
18857	3.6677	88.9088	11.2662
18858	3.6679	90.2516	193.973
18859	3.6679	-3.5693	104.235
18860	3.6679	-2.6736	255.642
18861	3.668	95.1684	102.423
18862	3.6682	114.575	51.8451
18863	3.6682	126.456	297.226
18864	3.6683	109.874	175.992
18865	3.6683	106.778	126.916
18866	3.6684	94.2476	303.216
18867	3.6685	118.729	33.2531
18868	3.6687	144.737	90.8759
18869	3.6688	101.506	4.6889
18870	3.6688	-2.7369	357.107
18871	3.6688	131.556	147.795
18872	3.669	82.9457	126.564
18873	3.6691	129.977	329.493
18874	3.6692	110.478	142.391
18875	3.6692	122.017	106.177
18876	3.6693	147.311	176.016
18877	3.6693	144.427	146.399
18878	3.6693	110.695	237.112
18879	3.6695	118.006	8.1985
18880	3.6696	129.043	86.9208
18881	3.6696	99.3208	220.389
18882	3.6697	91.5797	353.056
18883	3.6697	124.297	267.158
18884	3.6698	1.147	147.051
18885	3.6699	110.593	100.073
18886	3.67	154.699	206.317
18887	3.67	112.611	150.506
18888	3.6701	78.4657	295.026
18889	3.6701	109.994	237.484
18890	3.6701	120.465	309.205
18891	3.6702	108.459	271.529
18892	3.6702	97.2345	118.749
18893	3.6702	90.018	142.132
18894	3.6703	130.259	10.6517
18895	3.6703	115.079	59.0167
18896	3.6705	136.812	134.882
18897	3.6705	-2.8093	151.249
18898	3.6705	115.401	351.469
18899	3.6707	130.143	29.7888
18900	3.6707	119.202	100.17
18901	3.6708	133.686	134.246

18902	3.6708	111.617	48.4841
18903	3.6709	129.061	27.6009
18904	3.671	89.6219	38.8902
18905	3.671	119.977	316.274
18906	3.6711	111.379	174.303
18907	3.6711	95.1669	202.218
18908	3.6712	111.702	2.0554
18909	3.6713	130.676	68.1413
18910	3.6713	106.71	297.298
18911	3.6715	138.682	50.678
18912	3.6715	98.3063	293.033
18913	3.6715	110.01	39.3116
18914	3.6716	116.396	154.801
18915	3.6716	136.034	263.487
18916	3.6717	99.7922	106.932
18917	3.6717	114.587	22.1039
18918	3.6718	127.699	97.1804
18919	3.6718	120.504	191.368
18920	3.6718	96.1996	98.9953
18921	3.6719	116.37	198.877
18922	3.6722	105.595	273.482
18923	3.6722	124.725	153.357
18924	3.6724	29.3315	119.125
18925	3.6725	145.942	28.0263
18926	3.6725	99.6864	166.821
18927	3.6727	117.415	125.762
18928	3.6728	96.4283	193.014
18929	3.673	98.9705	167.458
18930	3.6731	119.332	74.0501
18931	3.6731	120.451	39.0056
18932	3.6732	132.198	42.5778
18933	3.6732	126.378	137.988
18934	3.6733	99.5426	138.982
18935	3.6734	133.849	352.14
18936	3.6734	142.83	116.141
18937	3.6736	111.947	55.1485
18938	3.6736	104.137	102.545
18939	3.6737	161.174	115.89
18940	3.6737	113.904	38.1561
18941	3.6737	-4.2185	201.938
18942	3.6738	114.663	219.059
18943	3.6738	111.825	234.064
18944	3.6738	121.062	76.7564
18945	3.6738	117.89	137.079
18946	3.6739	152.71	18.3611
18947	3.6739	129.523	78.0697
18948	3.674	113.905	63.5151
18949	3.6741	-1.8494	133.347

18950	3.6742	-2.8229	201.55
18951	3.6744	91.965	177.966
18952	3.6744	114.675	249.805
18953	3.6744	102.614	75.6984
18954	3.6745	111.885	286.594
18955	3.6745	105.699	147.655
18956	3.6745	123.351	256.045
18957	3.6746	132.576	325.707
18958	3.6746	115.381	298.887
18959	3.6746	121.736	168.867
18960	3.6748	147.526	46.9996
18961	3.6749	103.033	161.924
18962	3.675	123.183	159.358
18963	3.6751	130.972	257.952
18964	3.6753	96.8213	136.098
18965	3.6753	105.616	180.075
18966	3.6754	-2.7176	32.9612
18967	3.6755	119.844	133.878
18968	3.6755	135.438	51.3196
18969	3.6756	122.755	141.826
18970	3.6757	109.073	269.482
18971	3.6759	91.4591	26.8617
18972	3.6759	130.832	111.125
18973	3.676	152.117	26.5044
18974	3.6762	90.3354	183.851
18975	3.6763	130.316	21.5923
18976	3.6764	104.662	80.981
18977	3.6765	200.395	312.34
18978	3.6765	96.8375	172.583
18979	3.6765	95.0596	159.405
18980	3.6766	134.307	192.108
18981	3.6766	58.1003	77.0321
18982	3.6768	122.278	48.3998
18983	3.6769	107.251	101.468
18984	3.6769	76.0737	185.248
18985	3.6769	123.288	233.661
18986	3.6769	133.182	48.0381
18987	3.6771	97.7809	164.831
18988	3.6771	94.6769	101.638
18989	3.6772	79.3083	145.929
18990	3.6774	112.979	73.8009
18991	3.6774	122.156	39.9253
18992	3.6779	108.224	129.126
18993	3.6779	93.2351	128.872
18994	3.6779	99.6511	84.9134
18995	3.6781	138.405	27.6089
18996	3.6781	110.367	103.358
18997	3.6781	-2.7975	76.4558

18998	3.6781	113.85	80.9016
18999	3.6782	122.663	266.804
19000	3.6782	96.3556	115.594
19001	3.6783	149.204	246.492
19002	3.6784	105.77	88.4543
19003	3.6785	108.98	115.532
19004	3.6786	133.309	289.923
19005	3.6786	-5.0572	330.573
19006	3.6787	132.784	359.326
19007	3.6787	103.401	249.836
19008	3.6788	86.773	108.578
19009	3.679	131.624	286.149
19010	3.6791	144.806	318.535
19011	3.6791	82.3011	126.788
19012	3.6792	98.9856	153.51
19013	3.6792	-3.3715	92.6831
19014	3.6793	93.01	146.021
19015	3.6794	137.207	76.3833
19016	3.6794	113.774	186
19017	3.6794	123.87	305.72
19018	3.6794	110.43	306.098
19019	3.6794	85.6152	150.76
19020	3.6795	95.016	98.0594
19021	3.6796	113.501	28.8076
19022	3.6796	124.75	105.297
19023	3.6797	-2.6872	9.2364
19024	3.6798	89.7599	334.292
19025	3.6799	111.453	145.464
19026	3.6799	-3.3894	92.2964
19027	3.68	95.371	279.169
19028	3.68	-3.6551	302.422
19029	3.6801	106.719	38.1208
19030	3.6801	105.434	191.445
19031	3.6801	128.656	300.367
19032	3.6802	103.565	34.6342
19033	3.6802	120.827	118.09
19034	3.6803	132.055	116.887
19035	3.6803	120.754	67.1361
19036	3.6803	111.044	149.594
19037	3.6804	120.216	122.025
19038	3.6805	105.93	65.1544
19039	3.6805	110.364	108.425
19040	3.6805	142.097	59.4887
19041	3.6806	123.206	207.094
19042	3.6806	133.004	103.477
19043	3.6806	107.645	194.081
19044	3.6806	107.241	57.228
19045	3.6806	21.9226	173.324

19046	3.6806	115.977	138.113
19047	3.6807	112.755	56.4023
19048	3.6807	105.184	233.569
19049	3.6807	89.8266	8.1755
19050	3.6808	106.705	145.336
19051	3.6809	108.728	148.193
19052	3.6809	109.653	66.6886
19053	3.681	124.746	119.109
19054	3.681	109.502	228.255
19055	3.6812	143.859	204.965
19056	3.6813	95.4596	188.461
19057	3.6814	119.793	109.722
19058	3.6814	114.645	28.5674
19059	3.6814	88.4472	190.62
19060	3.6815	124.872	278.632
19061	3.6816	133.74	58.4826
19062	3.6816	139.16	105.848
19063	3.6816	102.692	146.988
19064	3.6817	105.484	294.625
19065	3.6817	117.72	16.0814
19066	3.6818	133.215	63.5195
19067	3.6818	83.338	315.86
19068	3.6821	115.581	220.795
19069	3.6823	130.817	198.237
19070	3.6823	87.7202	150.338
19071	3.6823	99.5866	241.881
19072	3.6824	138.808	67.4464
19073	3.6825	96.6832	159.849
19074	3.6829	108.602	142.458
19075	3.6829	103.174	117.919
19076	3.6831	103.198	187.739
19077	3.6831	102.875	116.487
19078	3.6832	105.279	268.592
19079	3.6833	129.679	146.966
19080	3.6834	119.978	115.666
19081	3.6834	93.8781	77.5475
19082	3.6836	115.407	227.518
19083	3.6836	94.251	271.263
19084	3.6837	123.484	282.964
19085	3.6837	99.146	317.998
19086	3.6837	108.127	78.9684
19087	3.6839	91.5949	80.2358
19088	3.6839	131.753	353.785
19089	3.684	73.845	292.296
19090	3.684	80.3517	87.5256
19091	3.6841	119.661	278.477
19092	3.6841	153.624	205.685
19093	3.6841	95.9401	119.919

19094	3.6841	85.7618	112.7
19095	3.6842	123.617	233.878
19096	3.6842	148.126	335.177
19097	3.6843	112.178	153.313
19098	3.6843	117.82	81.6017
19099	3.6844	139.103	57.8237
19100	3.6844	124.497	294.867
19101	3.6845	120.479	38.8405
19102	3.6845	132.101	60.2522
19103	3.6846	100.796	43.6128
19104	3.6846	111.174	159.518
19105	3.6847	105.116	273.806
19106	3.6851	104.459	331.153
19107	3.6852	110.37	114.273
19108	3.6852	143.937	345.87
19109	3.6853	90.1653	190.099
19110	3.6853	121.077	168.451
19111	3.6854	116.157	301.592
19112	3.6855	123.474	158.735
19113	3.6856	153.8	33.1869
19114	3.6856	122.732	181.115
19115	3.6857	153.035	52.8786
19116	3.6857	128.927	130.713
19117	3.6857	124.166	69.0117
19118	3.6858	132.766	164.214
19119	3.6858	125.321	354.291
19120	3.6859	65.4488	5.2898
19121	3.6859	130.041	73.1396
19122	3.6862	110.069	159.09
19123	3.6863	109.859	109.083
19124	3.6864	139.83	84.9127
19125	3.6865	88.5612	181.681
19126	3.6867	158.56	209.753
19127	3.6867	138.069	77.3523
19128	3.6867	141.624	123.548
19129	3.6869	84.1453	200.162
19130	3.6869	121.446	47.6982
19131	3.6869	134.708	97.1544
19132	3.6871	115.419	84.7622
19133	3.6872	143.244	30.38
19134	3.6872	105.406	118.911
19135	3.6873	22.9006	275
19136	3.6873	123.316	261.756
19137	3.6873	123.269	252.898
19138	3.6873	106.714	190.895
19139	3.6873	126.068	51.488
19140	3.6874	97.0895	260.321
19141	3.6874	128.281	110.448

19142	3.6874	119.228	305.772
19143	3.6874	100.854	104.242
19144	3.6875	134.303	310.113
19145	3.6876	95.4322	132.125
19146	3.6876	96.4625	333.833
19147	3.6877	121.262	97.4592
19148	3.6879	110.65	227.311
19149	3.6879	109.762	191.151
19150	3.688	111.905	227.298
19151	3.688	74.3757	99.1152
19152	3.6882	109.044	111.044
19153	3.6882	119.763	127.571
19154	3.6883	133.191	43.2752
19155	3.6884	122.771	297.266
19156	3.6884	111.759	110.295
19157	3.6885	98.8819	148.435
19158	3.6886	121.961	121.436
19159	3.6886	110.633	252.286
19160	3.6886	12.4913	213.413
19161	3.6887	125.762	46.0746
19162	3.6887	118.462	284.236
19163	3.6887	-3.3452	302.607
19164	3.6889	136.923	337.195
19165	3.689	98.9827	213.948
19166	3.689	138.797	65.1394
19167	3.6891	88.6156	215.497
19168	3.6892	134.974	22.8832
19169	3.6893	98.5154	161.684
19170	3.6894	151.357	117.725
19171	3.6895	130.396	154.654
19172	3.6895	103.591	141.206
19173	3.6895	100.543	154.582
19174	3.6896	146.531	8.1389
19175	3.6896	147.087	102.579
19176	3.6896	138.127	66.5222
19177	3.6897	111.159	67.2049
19178	3.6898	140.322	346.311
19179	3.6899	111.547	272.599
19180	3.6899	92.7508	126.635
19181	3.69	119.709	129.469
19182	3.6901	128.081	137.802
19183	3.6902	126.652	279.041
19184	3.6903	114.9	144.864
19185	3.6904	87.1361	42.3735
19186	3.6905	123.901	43.3147
19187	3.6906	169.442	20.5815
19188	3.6906	115.274	304.372
19189	3.6907	109.303	112.92

19190	3.6907	133.753	88.614
19191	3.6908	104.186	225.507
19192	3.6909	131.357	175.766
19193	3.691	129.568	80.9934
19194	3.6911	128.783	248.051
19195	3.6912	102.017	180.466
19196	3.6912	158.957	113.61
19197	3.6912	116.721	153.945
19198	3.6912	127.269	26.5391
19199	3.6914	144.78	337.35
19200	3.6915	83.8711	173.983
19201	3.6915	42.2444	52.8082
19202	3.6916	89.454	167.846
19203	3.6916	146.817	259.959
19204	3.6917	105.435	121.199
19205	3.6918	137.274	185.318
19206	3.6919	108.853	123.578
19207	3.692	114.646	213.347
19208	3.692	116.853	236.509
19209	3.6921	124.744	74.8973
19210	3.6921	108.92	152.644
19211	3.6921	105.106	77.7531
19212	3.6922	124.844	72.743
19213	3.6922	114.184	269.143
19214	3.6923	101.54	192.146
19215	3.6924	89.9963	113.965
19216	3.6924	114.416	60.7798
19217	3.6925	99.4012	129.299
19218	3.6925	109.884	116.949
19219	3.6926	-4.633	184.395
19220	3.6927	136.975	53.5007
19221	3.6927	146.445	76.1602
19222	3.693	116.705	153.448
19223	3.6931	127.552	297.751
19224	3.6932	110.253	110.58
19225	3.6932	107.574	51.6585
19226	3.6933	102.25	48.196
19227	3.6934	129.807	75.8412
19228	3.6936	147.214	202.518
19229	3.6936	121.429	149.184
19230	3.6937	102.222	204.558
19231	3.6938	88.7213	126.887
19232	3.6938	105.077	128.007
19233	3.6938	103.403	198.275
19234	3.6939	118.676	246.074
19235	3.6941	95.3115	66.7106
19236	3.6941	129.849	82.6632
19237	3.6941	114.585	72.3439

19238	3.6942	106.593	16.1568
19239	3.6943	124.792	88.3563
19240	3.6946	137.838	262.958
19241	3.6947	111.024	142.08
19242	3.6948	118.022	229.696
19243	3.6949	139.793	317.068
19244	3.6949	116.269	43.031
19245	3.695	109.972	102.446
19246	3.695	127.511	96.2173
19247	3.6951	94.2285	167.568
19248	3.6952	114.424	118.307
19249	3.6952	114.932	101.247
19250	3.6952	117.624	64.5874
19251	3.6953	126.079	41.7372
19252	3.6953	94.6769	267.926
19253	3.6953	93.9555	274.46
19254	3.6953	133.358	27.133
19255	3.6953	-2.9969	158.978
19256	3.6956	101.18	256.95
19257	3.6957	20.5013	239.918
19258	3.6958	124.902	130.088
19259	3.6961	120.855	345.724
19260	3.6961	109.46	286.855
19261	3.6963	153.204	199.396
19262	3.6963	144.191	298.746
19263	3.6963	136.047	100.838
19264	3.6963	135.397	355.003
19265	3.6964	94.8685	158.617
19266	3.6964	106.471	256.18
19267	3.6965	111.084	332.197
19268	3.6966	111.686	88.7799
19269	3.6967	-2.7089	35.8325
19270	3.6969	160.037	56.1565
19271	3.697	140.991	29.7022
19272	3.697	115.406	134.169
19273	3.6971	129.872	346.065
19274	3.6971	80.1537	124.651
19275	3.6971	87.8318	67.2899
19276	3.6974	113.756	242.291
19277	3.6974	118.339	66.1996
19278	3.6975	135.503	205.986
19279	3.6976	104.088	173.928
19280	3.6976	95.8086	307.147
19281	3.6977	116.265	222.151
19282	3.6977	124.41	227.123
19283	3.6978	121.776	88.245
19284	3.6978	100.861	221.135
19285	3.6979	113.71	107.48

19286	3.6979	82.4179	199.589
19287	3.6979	116.633	277.841
19288	3.698	136.311	206.629
19289	3.698	133.988	98.9155
19290	3.6981	105.472	157.511
19291	3.6982	112.187	136.763
19292	3.6982	-2.8046	150.747
19293	3.6983	125.119	232.879
19294	3.6985	82.7924	119.009
19295	3.6986	110.512	85.1678
19296	3.6987	83.5171	181.92
19297	3.6987	116.463	165.653
19298	3.6989	140.296	268.242
19299	3.6989	108.886	244.828
19300	3.699	118.839	85.3491
19301	3.699	-2.7222	30.681
19302	3.699	117.239	83.6718
19303	3.699	73.417	74.1442
19304	3.6991	146.984	258.007
19305	3.6991	106.456	80.6403
19306	3.6992	144.139	90.2188
19307	3.6992	130.182	112.013
19308	3.6992	128.779	111.435
19309	3.6993	138.297	289.711
19310	3.6995	130.231	158.354
19311	3.6995	127.792	145.696
19312	3.6995	82.3446	157.821
19313	3.6996	99.8655	129.792
19314	3.6997	142.816	63.3869
19315	3.6997	129.162	273.565
19316	3.6997	110.619	226.146
19317	3.6997	134.366	132.3
19318	3.6997	116.605	92.2967
19319	3.7	101.855	72.5692
19320	3.7	115.702	274.584
19321	3.7	102.358	125.231
19322	3.7001	131.097	38.033
19323	3.7001	105.774	194.995
19324	3.7002	128.62	312.536
19325	3.7002	118.067	254.936
19326	3.7002	138.107	241.701
19327	3.7002	133.399	304.843
19328	3.7003	130.677	41.9741
19329	3.7003	108.695	173.169
19330	3.7003	83.675	159.608
19331	3.7005	116.092	195.673
19332	3.7005	106.41	122.661
19333	3.7006	129.842	143.812

19334	3.7007	131.051	153.274
19335	3.7007	126.574	177.788
19336	3.7007	-4.1595	203.746
19337	3.7007	121.033	270.663
19338	3.7008	97.5513	243.194
19339	3.7009	102.304	113.772
19340	3.7009	111.792	188.236
19341	3.701	153.633	42.1765
19342	3.701	103.216	181.667
19343	3.7011	88.9499	157.084
19344	3.7011	134.7	327.288
19345	3.7012	120.307	211.274
19346	3.7012	104.837	117.423
19347	3.7012	124.567	307.443
19348	3.7013	151.035	120.562
19349	3.7014	127.901	247.203
19350	3.7015	123.814	123.689
19351	3.7016	81.466	174.326
19352	3.7016	109.161	264.321
19353	3.7017	130.937	147.86
19354	3.7019	120.529	112.101
19355	3.7019	113.398	115.183
19356	3.702	96.249	95.527
19357	3.7021	108.171	323.701
19358	3.7022	117.636	67.9855
19359	3.7023	165.772	19.5431
19360	3.7023	129.722	181.415
19361	3.7023	110.318	171.043
19362	3.7025	130.549	30.8177
19363	3.7025	91.3901	224.911
19364	3.7026	132.201	74.3097
19365	3.7027	124.648	277.426
19366	3.7029	94.7055	71.1425
19367	3.7029	122.347	116.462
19368	3.703	106.151	187.271
19369	3.7032	115.553	183.957
19370	3.7032	72.4499	180.127
19371	3.7034	131.644	246.691
19372	3.7034	112.787	102.359
19373	3.7035	102.631	170.581
19374	3.7036	130.596	130.032
19375	3.7038	-2.7919	153.856
19376	3.7038	122.464	85.4047
19377	3.7038	101.851	186.923
19378	3.704	102.789	216.77
19379	3.7043	121.087	250.539
19380	3.7043	132.457	128.436
19381	3.7044	124.676	350.794

19382	3.7044	-3.6356	54.1779
19383	3.7044	103.377	181.589
19384	3.7044	110.404	235.765
19385	3.7046	115.324	145.947
19386	3.7046	127.657	81.6233
19387	3.7047	178.695	288.491
19388	3.7048	116.607	132.43
19389	3.7049	121.772	218.626
19390	3.7049	111.2	71.1191
19391	3.7049	118.166	137.957
19392	3.705	121.054	303.432
19393	3.7051	142.815	298.451
19394	3.7051	106.541	248.767
19395	3.7051	122.933	119.863
19396	3.7053	134.321	67.3291
19397	3.7053	134.816	330.634
19398	3.7054	138.386	128.799
19399	3.7055	139.098	211.53
19400	3.7055	110.811	90.4942
19401	3.7056	114.69	274.858
19402	3.7056	121.976	62.2568
19403	3.7056	119.791	272.558
19404	3.7058	164.194	81.3496
19405	3.7059	130.534	314.254
19406	3.706	119.201	41.2999
19407	3.706	130.818	200.384
19408	3.706	70.6846	280.42
19409	3.7061	119.625	105.1
19410	3.7063	125.793	202.068
19411	3.7063	103.779	130.141
19412	3.7065	140.93	68.4287
19413	3.7066	100.765	351.16
19414	3.7066	130.021	38.1746
19415	3.7067	168.545	94.2781
19416	3.7067	124.198	234.802
19417	3.7068	111.986	163.399
19418	3.7068	113.507	159.459
19419	3.7068	123.926	118.726
19420	3.7069	129.724	71.8951
19421	3.7069	94.8935	142.65
19422	3.7069	117.422	171.721
19423	3.7072	124.549	60.6902
19424	3.7072	108.389	154.153
19425	3.7073	92.7185	320.428
19426	3.7075	125.561	43.455
19427	3.7076	90.3165	205.483
19428	3.7076	35.5817	200.275
19429	3.7076	102.67	128.221

19430	3.7077	128.954	287.69
19431	3.7077	132.803	209.63
19432	3.7077	115.91	138.951
19433	3.7078	121.918	90.5599
19434	3.7079	124.44	109.551
19435	3.7079	104.946	125.621
19436	3.708	109.301	219.846
19437	3.708	126.608	214.299
19438	3.7082	88.2024	130.129
19439	3.7082	122.725	154.468
19440	3.7082	117.727	92.2362
19441	3.7082	106.028	135.921
19442	3.7083	153.199	254.953
19443	3.7083	103.703	211.458
19444	3.7083	121.157	136.101
19445	3.7083	113.117	104.15
19446	3.7084	126.648	193.186
19447	3.7084	107.371	120.665
19448	3.7085	-2.9936	65.5512
19449	3.7087	100.983	296.173
19450	3.7087	107.322	115.55
19451	3.7087	128.789	53.7549
19452	3.7087	137.315	327.713
19453	3.7088	124.108	105.101
19454	3.7088	95.1008	358.499
19455	3.7089	135.36	234.96
19456	3.7089	119.841	159.09
19457	3.709	87.0516	138.173
19458	3.7092	117.119	308.567
19459	3.7093	99.8888	274.888
19460	3.7093	-3.215	114.389
19461	3.7094	166.157	204.398
19462	3.7094	111.851	157.422
19463	3.7096	118.602	314.825
19464	3.7096	131.565	328.782
19465	3.7097	112.231	301.951
19466	3.7097	123.829	55.091
19467	3.7097	78.295	36.4483
19468	3.7097	98.0306	80.7368
19469	3.7099	131.402	125.903
19470	3.7099	76.4982	82.0681
19471	3.7099	131.329	149.589
19472	3.71	112.584	299.097
19473	3.71	86.0759	158.037
19474	3.7101	90.0983	166.898
19475	3.7101	125.031	63.8201
19476	3.7101	105.701	135.216
19477	3.7102	105.546	122.247

19478	3.7103	107.036	294.679
19479	3.7104	142.512	24.4339
19480	3.7105	165.117	16.519
19481	3.7105	134.536	65.8107
19482	3.7105	21.3262	250.001
19483	3.7106	114.609	270.784
19484	3.7107	121.095	291.584
19485	3.7107	133.49	259.301
19486	3.7109	113.245	115.671
19487	3.7109	122.751	300.083
19488	3.711	102.104	169.436
19489	3.7113	151.715	191.738
19490	3.7113	94.8784	146.752
19491	3.7114	107.136	157.325
19492	3.7115	96.4798	137.964
19493	3.7115	118.179	65.9989
19494	3.7116	124.573	113.473
19495	3.7117	96.0852	171.488
19496	3.7117	120.148	146.428
19497	3.7118	132.362	41.0695
19498	3.7119	106.324	52.4087
19499	3.7119	129.047	99.9854
19500	3.7121	80.8269	222.318
19501	3.7121	133.755	88.123
19502	3.7121	-2.7881	152.382
19503	3.7122	113.83	128.534
19504	3.7122	127.739	101.247
19505	3.7123	111.762	219.659
19506	3.7124	119.788	173.562
19507	3.7124	138.141	1.6994
19508	3.7125	122.545	94.8691
19509	3.7126	146.493	30.7633
19510	3.7126	164.115	242.255
19511	3.7126	137.68	27.8645
19512	3.7126	125.433	127.858
19513	3.7127	145.613	47.3649
19514	3.7127	116.465	154.974
19515	3.7129	128.225	123.717
19516	3.7131	131.983	72.832
19517	3.7131	3.5852	11.4157
19518	3.7131	103.187	158.524
19519	3.7132	148.321	205.208
19520	3.7132	106.502	169.041
19521	3.7134	109.336	243.884
19522	3.7134	5.9142	125.212
19523	3.7134	122.641	108.227
19524	3.7135	107.425	162.12
19525	3.7135	106.356	99.8575

19526	3.7136	158.006	85.521
19527	3.7136	134.594	30.8457
19528	3.7137	108.611	3.3618
19529	3.7138	132.915	126.526
19530	3.7138	129.244	108.79
19531	3.7138	154.122	39.4406
19532	3.7141	2.4587	162.172
19533	3.7142	89.1319	258.667
19534	3.7142	126.531	11.7571
19535	3.7143	113.829	110.878
19536	3.7143	118.095	36.3108
19537	3.7143	91.6189	107.77
19538	3.7143	127.697	66.2107
19539	3.7144	121.946	107.828
19540	3.7144	78.9482	33.0487
19541	3.7145	150.838	214.012
19542	3.7145	105.28	163.286
19543	3.7146	119.745	279.753
19544	3.7147	127.578	161.639
19545	3.7148	118.059	281.814
19546	3.7149	105.437	328.318
19547	3.715	142.441	96.8653
19548	3.715	-3.2989	59.1279
19549	3.715	125.837	162.133
19550	3.7151	127.273	227.292
19551	3.7152	152.278	52.8603
19552	3.7153	121.388	98.0304
19553	3.7153	112.575	133.695
19554	3.7153	137.321	148.107
19555	3.7154	138.436	10.4603
19556	3.7154	121.463	111.714
19557	3.7155	129.257	74.6746
19558	3.7156	88.3792	163.162
19559	3.7158	125.401	190.101
19560	3.7158	127.8	91.6326
19561	3.7159	105.95	301.672
19562	3.716	119.198	49.8634
19563	3.716	131.156	118.667
19564	3.7161	126.401	102.384
19565	3.7161	138.881	218.388
19566	3.7161	113.598	306.861
19567	3.7162	112.534	171.06
19568	3.7163	121.946	256.876
19569	3.7163	128.571	84.8929
19570	3.7164	112.986	196.334
19571	3.7165	120.846	117.407
19572	3.7165	116.501	216.389
19573	3.7166	128.817	284.247

19574	3.7166	32.7394	269.513
19575	3.7166	98.1938	310.341
19576	3.7167	127.743	118.198
19577	3.7169	120.029	155.013
19578	3.7169	118.279	68.1873
19579	3.717	123.303	103.403
19580	3.717	126.408	216.057
19581	3.717	117.386	264.802
19582	3.7171	97.2546	192.297
19583	3.7173	119.1	100.791
19584	3.7173	112.758	119.11
19585	3.7173	77.1863	28.503
19586	3.7174	93.795	266.85
19587	3.7175	104.648	0.8597
19588	3.7175	103.377	41.5563
19589	3.7175	110.259	141.068
19590	3.7175	128.112	127.607
19591	3.7176	127.56	80.61
19592	3.7176	111.309	153.667
19593	3.7177	142.628	59.7523
19594	3.7178	130.977	339.223
19595	3.7178	83.7866	51.2601
19596	3.7178	132.135	306.73
19597	3.718	144.601	217.053
19598	3.718	122.129	297.854
19599	3.7181	100.667	157.15
19600	3.7182	132.606	148.168
19601	3.7183	129.167	96.4562
19602	3.7185	126.472	280.82
19603	3.7187	145.974	76.4169
19604	3.7187	132.947	206.741
19605	3.7187	91.1308	159.201
19606	3.7188	155.162	95.9294
19607	3.7188	119.815	193.188
19608	3.7188	113.497	58.8813
19609	3.7188	8.7253	317.987
19610	3.7191	126.624	119.857
19611	3.7192	128.909	167.168
19612	3.7193	141.85	317.539
19613	3.7195	154.658	204.152
19614	3.7195	107.079	104.906
19615	3.7195	114.118	329.54
19616	3.7196	115.09	107.899
19617	3.7197	121.405	122.181
19618	3.7198	152.527	148.742
19619	3.7198	148.814	95.185
19620	3.7198	141.297	216.192
19621	3.7198	112.13	78.2051

19622	3.72	159.431	303.259
19623	3.72	104.659	325.936
19624	3.7201	118.556	299.669
19625	3.7201	135.972	60.4729
19626	3.7201	91.2427	238.75
19627	3.7202	139.407	76.695
19628	3.7202	110.064	52.2487
19629	3.7203	98.5053	137.842
19630	3.7203	108.864	94.7698
19631	3.7203	130.015	99.1257
19632	3.7206	167.935	90.8742
19633	3.7207	-5.1127	4.7402
19634	3.7209	78.948	254.075
19635	3.7209	111.76	113.843
19636	3.721	110.747	170.047
19637	3.7211	109.757	176.761
19638	3.7211	122.665	72.6975
19639	3.7213	141.737	257.195
19640	3.7214	124.206	234.919
19641	3.7214	98.3176	309.901
19642	3.7214	98.9095	300.634
19643	3.7215	128.384	170.888
19644	3.7215	113.66	164.359
19645	3.7216	107.413	185.749
19646	3.7217	125.459	324.498
19647	3.7217	117.795	25.5622
19648	3.7218	134.304	1.7402
19649	3.7218	131.197	117.785
19650	3.7218	140.112	150.093
19651	3.7219	144.472	235.527
19652	3.7219	139.956	78.5074
19653	3.7219	112.055	182.712
19654	3.7219	97.4302	141.973
19655	3.722	108.594	79.4599
19656	3.7221	139.793	145.169
19657	3.7221	121.292	65.7003
19658	3.7222	109.228	12.5422
19659	3.7222	119.419	93.6042
19660	3.7222	104.397	284.153
19661	3.7222	125.248	303.456
19662	3.7223	139.561	66.2396
19663	3.7224	104.641	340.294
19664	3.7224	133.527	47.7088
19665	3.7224	133.342	216.223
19666	3.7226	112.629	233.728
19667	3.7227	129.413	190.755
19668	3.7228	116.099	281.861
19669	3.7229	135.094	217.897

19670	3.7231	134.57	136.446
19671	3.7231	91.766	147.712
19672	3.7232	126.038	9.3092
19673	3.7232	138.107	64.6519
19674	3.7233	135.813	43.6397
19675	3.7235	117.606	155.62
19676	3.7237	145.143	38.8624
19677	3.7237	117.023	32.1168
19678	3.7238	143.678	293.205
19679	3.7238	137.612	103.129
19680	3.724	161.866	140.227
19681	3.7241	150.649	54.6337
19682	3.7241	134.107	265.383
19683	3.7241	117.599	100.955
19684	3.7241	129.876	119.173
19685	3.7242	123.028	275.609
19686	3.7242	121.891	108.935
19687	3.7242	91.1143	30.6915
19688	3.7243	161.986	208.066
19689	3.7243	125.681	49.0408
19690	3.7245	129.979	25.6391
19691	3.7245	101.877	199.957
19692	3.7246	132.831	73.8792
19693	3.7247	134.364	105.964
19694	3.7248	133.095	17.6247
19695	3.7248	77.8423	81.1139
19696	3.7249	117.01	59.2307
19697	3.7249	112.352	111.605
19698	3.725	150.288	54.9678
19699	3.725	108.211	314.018
19700	3.725	126.472	308.873
19701	3.725	121.647	40.8062
19702	3.7251	134.613	39.5335
19703	3.7252	102.028	9.0523
19704	3.7252	116.048	33.7574
19705	3.7253	118.029	128.494
19706	3.7256	134.234	101.289
19707	3.7257	157.403	46.784
19708	3.7259	82.345	80.2039
19709	3.726	77.1863	49.4069
19710	3.726	96.8139	184.09
19711	3.726	117.018	123.212
19712	3.7261	145.222	136.188
19713	3.7261	103.019	145.166
19714	3.7262	148.623	56.2279
19715	3.7262	110.502	183.993
19716	3.7263	139.747	186.888
19717	3.7263	123.513	264.499

19718	3.7264	135.831	203.571
19719	3.7265	150.314	48.6337
19720	3.7265	125.543	212.193
19721	3.7265	115.882	78.9422
19722	3.7265	114.165	36.0033
19723	3.7266	140.207	259.948
19724	3.7266	132.028	103.243
19725	3.7266	124.897	153.998
19726	3.7267	149.786	35.2163
19727	3.7267	112.197	75.9305
19728	3.7268	109.104	175.993
19729	3.7268	128.872	293.195
19730	3.7268	105.064	98.3475
19731	3.7269	124.584	184.063
19732	3.7269	111.785	159.189
19733	3.7269	157.44	194.795
19734	3.727	132.449	112.691
19735	3.727	124.064	172.611
19736	3.7272	119.907	95.8221
19737	3.7275	114.2	118.068
19738	3.7275	112.92	144.478
19739	3.7276	95.6553	50.9852
19740	3.7276	114.723	146.521
19741	3.7277	127.944	87.0163
19742	3.7278	110.744	136.641
19743	3.7279	102.015	159.84
19744	3.7279	114.537	78.6171
19745	3.728	105.533	265.97
19746	3.728	125.382	231.7
19747	3.7282	137.294	202.107
19748	3.7282	121.975	139.116
19749	3.7283	93.8946	158.19
19750	3.7283	104.761	127.146
19751	3.7284	115.318	91.2207
19752	3.7284	122.972	219.395
19753	3.7284	146.737	330.28
19754	3.7286	132.545	153.062
19755	3.7287	110.091	169.128
19756	3.7287	120.693	179.3
19757	3.7287	111.34	165.497
19758	3.7287	120.535	328.683
19759	3.7288	124.842	274.781
19760	3.7288	105.97	132.483
19761	3.729	92.7129	214.91
19762	3.7291	104.499	119.937
19763	3.7291	128.962	96.779
19764	3.7295	153.86	43.1229
19765	3.7297	118.697	139.82

19766	3.7298	121.362	132.629
19767	3.7299	147.851	143.836
19768	3.7299	93.3059	137.552
19769	3.7299	120.255	28.1697
19770	3.7301	117.021	196.24
19771	3.7301	121.055	196.334
19772	3.7302	106.463	71.3614
19773	3.7304	96.7727	333.295
19774	3.7304	108.809	131.188
19775	3.7307	154.526	37.5818
19776	3.7307	123.739	84.0819
19777	3.7307	112.607	225.104
19778	3.7308	135.095	210.63
19779	3.7309	128.157	220.661
19780	3.7309	128.467	147.228
19781	3.731	92.6645	27.9202
19782	3.731	131.103	257.775
19783	3.731	122.054	93.7991
19784	3.7311	113.16	268.769
19785	3.7311	96.5283	182.607
19786	3.7313	140.578	202.802
19787	3.7313	114.67	96.3215
19788	3.7313	-2.7668	117.096
19789	3.7314	138.756	82.7405
19790	3.7315	140.76	298.725
19791	3.7316	126.723	183.647
19792	3.7318	124.13	340.643
19793	3.7319	163.022	42.2488
19794	3.7319	124.622	304.385
19795	3.7319	117.987	109.233
19796	3.732	118.997	297.299
19797	3.7321	115.737	174.889
19798	3.7322	130.991	13.0257
19799	3.7322	126.53	208.803
19800	3.7323	110.335	157.715
19801	3.7324	81.677	97.6372
19802	3.7326	118.389	166.411
19803	3.7326	147.22	147.262
19804	3.7327	138.046	124.032
19805	3.7327	109.468	138.017
19806	3.7328	131.561	49.1881
19807	3.7328	127.712	281.728
19808	3.7328	105.616	137.716
19809	3.7329	161.221	46.6483
19810	3.7329	106.494	114.794
19811	3.7329	149.28	334.889
19812	3.733	126.471	231.706
19813	3.7331	151.642	243.69

19814	3.7332	106.456	208.318
19815	3.7332	126.652	224.157
19816	3.7333	152.024	53.7882
19817	3.7333	104.401	334.934
19818	3.7333	130.066	259.576
19819	3.7333	103.448	266.8
19820	3.7334	120.403	253.999
19821	3.7335	122.085	143.769
19822	3.7337	121.906	196.726
19823	3.7338	86.9194	74.0138
19824	3.7339	122.037	82.0316
19825	3.7339	112.613	203.366
19826	3.7339	156.844	150.657
19827	3.7339	109.143	338.146
19828	3.7339	124.376	97.4846
19829	3.734	135.215	317.693
19830	3.7341	140.775	65.569
19831	3.7341	93.9651	308.161
19832	3.7342	141.941	61.4666
19833	3.7343	99.0057	11.4692
19834	3.7345	134.193	195.266
19835	3.7345	131.459	78.5177
19836	3.7345	139.03	327.617
19837	3.7346	121.926	130.82
19838	3.7346	134.06	225.208
19839	3.7347	126.293	97.9418
19840	3.7347	103.719	103.405
19841	3.7349	-2.6997	253.012
19842	3.735	85.9054	33.2741
19843	3.7352	136.334	53.209
19844	3.7352	157.098	43.6237
19845	3.7352	135.079	334.639
19846	3.7353	118.503	195.055
19847	3.7353	157.883	77.5931
19848	3.7354	148.426	29.52
19849	3.7355	127.327	92.3406
19850	3.7356	116.682	96.576
19851	3.7356	123.487	64.5573
19852	3.7357	117.143	125.05
19853	3.7357	109.39	189.312
19854	3.7358	91.7798	155.978
19855	3.7359	125.677	105.049
19856	3.7359	119.072	299.167
19857	3.736	127.544	299.018
19858	3.7362	154.422	52.9175
19859	3.7362	136.28	24.7926
19860	3.7364	131.339	160.422
19861	3.7364	147.897	131.767

19862	3.7365	138.326	259.509
19863	3.7366	119.993	221.488
19864	3.7366	145.886	127.701
19865	3.7367	128.782	59.232
19866	3.7369	163.212	63.8316
19867	3.737	152.788	191.441
19868	3.7371	138.761	184.887
19869	3.7372	120.915	18.8792
19870	3.7372	132.744	138.536
19871	3.7373	133.238	293.242
19872	3.7373	137.024	149.845
19873	3.7373	135.878	122.336
19874	3.7375	107.005	147.297
19875	3.7379	170.606	200.632
19876	3.7379	152.948	205.413
19877	3.7379	119.238	193.974
19878	3.7379	119.993	45.8889
19879	3.7379	116.286	291.54
19880	3.738	111.557	72.6641
19881	3.738	110.737	31.4413
19882	3.7381	107.15	170.177
19883	3.7382	136.624	290.896
19884	3.7382	133.452	106.785
19885	3.7383	111.053	157.671
19886	3.7384	143.772	129.925
19887	3.7385	119.226	121.8
19888	3.7385	140.483	30.3683
19889	3.7386	117.443	115.331
19890	3.7387	126.127	321.601
19891	3.7389	139.481	205.252
19892	3.739	147.682	64.2258
19893	3.7391	134.914	216.089
19894	3.7392	158.584	49.7114
19895	3.7392	142.183	208.261
19896	3.7395	142.976	228.059
19897	3.7397	134.13	16.9677
19898	3.7398	136.046	59.9753
19899	3.74	171.134	22.3525
19900	3.74	98.4674	264.687
19901	3.7401	208.548	314.593
19902	3.7402	145.604	281.647
19903	3.7402	119.426	104.409
19904	3.7403	139.629	243.114
19905	3.7403	107.966	127.445
19906	3.7406	120.051	126.229
19907	3.7407	69.4747	166.035
19908	3.7408	111.097	167.102
19909	3.7409	134.615	30.3244

19910	3.741	121.761	118.118
19911	3.7412	178.103	85.7792
19912	3.7412	129.226	222.266
19913	3.7414	111.054	114.432
19914	3.7415	148.665	96.4015
19915	3.7417	147.135	97.1399
19916	3.7417	103.976	114.408
19917	3.7419	115.762	195.388
19918	3.7419	98.0169	150.408
19919	3.742	113.89	104.611
19920	3.742	127.163	214.728
19921	3.7421	165.042	97.9169
19922	3.7421	117.196	152.722
19923	3.7422	102.843	50.082
19924	3.7422	127.373	68.8201
19925	3.7422	118.682	80.5372
19926	3.7423	89.0853	306.127
19927	3.7423	129.791	218.465
19928	3.7424	115.408	213.235
19929	3.7425	134.558	77.5492
19930	3.7425	112.163	129.756
19931	3.7425	150.275	96.8157
19932	3.7428	101.218	133.523
19933	3.7428	134.272	280.701
19934	3.7429	112.289	252.179
19935	3.7429	116.418	79.6118
19936	3.743	123.661	267.157
19937	3.7431	112.876	81.267
19938	3.7431	109.064	227.273
19939	3.7432	184.084	82.6211
19940	3.7432	143.719	126.893
19941	3.7434	84.604	30.3122
19942	3.7436	132.213	157.352
19943	3.7436	110.72	199.823
19944	3.7436	106.064	128.65
19945	3.7436	-2.6924	25.4169
19946	3.7436	134.705	132.397
19947	3.7437	-2.7985	143.008
19948	3.7437	105.443	214.812
19949	3.7437	143.503	135.196
19950	3.7438	123.091	128.345
19951	3.7439	137.395	53.182
19952	3.7439	128.393	335.719
19953	3.744	122.191	164.983
19954	3.7441	134.676	278.199
19955	3.7442	132.837	116.987
19956	3.7443	132.745	273.97
19957	3.7443	107.772	133.347

19958	3.7444	124.687	291.709
19959	3.7445	144.034	279.153
19960	3.7445	168.472	310.702
19961	3.7445	115.629	87.7609
19962	3.7446	128.95	246.517
19963	3.7446	139.704	204.111
19964	3.7447	127.872	37.8176
19965	3.7448	93.7667	332.523
19966	3.7448	143.958	261.99
19967	3.745	147.072	314.302
19968	3.745	118.336	269.177
19969	3.745	113.712	139.952
19970	3.7451	122.357	12.0612
19971	3.7451	-3.5516	298.969
19972	3.7452	140.811	93.4348
19973	3.7452	140.213	276.305
19974	3.7452	117.862	94.345
19975	3.7453	142.682	121.544
19976	3.7453	96.7294	159.768
19977	3.7454	108.809	173.984
19978	3.7454	116.618	348.192
19979	3.7455	117.321	206.37
19980	3.7455	123.36	293.152
19981	3.7456	135.318	147.869
19982	3.7456	156.006	28.2715
19983	3.7456	136.794	23.1709
19984	3.7457	125.592	30.3477
19985	3.7457	133.077	131.708
19986	3.7457	127.746	114.347
19987	3.7457	144.022	304.419
19988	3.7461	126.046	197.083
19989	3.7462	103.165	108.191
19990	3.7464	147.518	224.459
19991	3.7465	110.248	199.171
19992	3.7466	132.997	112.182
19993	3.7466	110.441	126.452
19994	3.7466	119.887	93.0655
19995	3.7467	160.192	23.7326
19996	3.7467	128.665	56.6833
19997	3.7468	150.921	65.2331
19998	3.7468	101.467	109.924
19999	3.7471	120.026	289.632
20000	3.7471	151.513	292.257
20001	3.7472	143.864	149.687
20002	3.7472	124.559	15.4353
20003	3.7473	152.665	57.0598
20004	3.7474	145.771	132.891
20005	3.7474	114.37	173.26

20006	3.7474	124.261	166.749
20007	3.7479	-5.9849	133.942
20008	3.748	125.897	233.262
20009	3.7481	146.394	64.8075
20010	3.7481	101.868	29.6371
20011	3.7482	121.471	99.5457
20012	3.7483	133.023	249.682
20013	3.7483	118.921	305.672
20014	3.7485	-3.5051	281.316
20015	3.7486	121.029	195.297
20016	3.7486	153.715	48.0733
20017	3.7487	118.555	172.348
20018	3.7489	147.85	277.313
20019	3.749	78.6075	106.47
20020	3.749	76.6632	135.637
20021	3.749	89.3336	161.298
20022	3.749	151.729	116.939
20023	3.7492	90.5559	125.16
20024	3.7493	123.641	88.1544
20025	3.7494	105.984	37.6344
20026	3.7495	149.247	109.864
20027	3.7496	127.201	302.252
20028	3.7497	131.808	74.7015
20029	3.7498	128.624	97.4196
20030	3.7498	118.131	247.404
20031	3.7499	145.443	254.194
20032	3.7501	129.5	132.995
20033	3.7502	141.842	38.6832
20034	3.7502	103.205	325.162
20035	3.7502	122.929	329.815
20036	3.7503	157.033	146.13
20037	3.7504	144.997	281.6
20038	3.7504	111.454	125.822
20039	3.7506	121.064	186.772
20040	3.7507	136.167	294.396
20041	3.7512	116.016	176.962
20042	3.7513	100.071	201.721
20043	3.7513	132.099	117.518
20044	3.7513	123.007	319.083
20045	3.7514	111.94	165.783
20046	3.7514	122.141	33.7022
20047	3.7516	126.646	213.88
20048	3.7518	134.229	295.207
20049	3.7518	126.895	245.557
20050	3.7519	129.414	232.695
20051	3.752	164.065	12.1308
20052	3.752	130.776	326.082
20053	3.7524	120.657	174.242

20054	3.7525	94.9996	83.5145
20055	3.7526	135.282	190.974
20056	3.7526	155.425	44.7777
20057	3.7527	133.631	247.278
20058	3.7527	138.572	75.7542
20059	3.7527	119.034	176.116
20060	3.7528	139.595	7.7817
20061	3.7529	123.311	242.356
20062	3.7529	152.767	324.295
20063	3.7529	129.347	90.4771
20064	3.7529	125.891	89.5546
20065	3.753	125.268	130.191
20066	3.7531	127.542	110.201
20067	3.7531	106.849	2.4504
20068	3.7534	122.369	274.921
20069	3.7536	162.853	37.2272
20070	3.7536	114.72	178.897
20071	3.7536	145.119	156.428
20072	3.7537	127.232	278.704
20073	3.7539	142.318	57.8356
20074	3.7539	114.307	95.3423
20075	3.754	117.541	193.442
20076	3.7541	102.121	316.443
20077	3.7541	139.684	148.11
20078	3.7543	147.775	254.712
20079	3.7544	152.181	32.8441
20080	3.7544	134.945	177.068
20081	3.7546	112.064	144.647
20082	3.7546	132.866	152.887
20083	3.7547	136.949	246.647
20084	3.7547	138.151	67.5484
20085	3.7547	135.976	323.546
20086	3.7548	139.62	75.4366
20087	3.7549	120.62	274.983
20088	3.7552	122.325	39.5741
20089	3.7552	35.3042	69.3472
20090	3.7554	129.152	228.653
20091	3.7556	150.788	61.0021
20092	3.7559	145.377	131.447
20093	3.756	139.644	93.352
20094	3.7561	162.462	52.538
20095	3.7564	159.785	55.974
20096	3.7564	152.746	5.206
20097	3.7564	95.725	271.065
20098	3.7565	124.637	131.693
20099	3.7565	127.497	305.833
20100	3.7567	115.919	164.89
20101	3.7567	133.189	172.662

20102	3.7567	108.254	97.3003
20103	3.7568	110.232	110.848
20104	3.7568	125.477	34.5813
20105	3.7569	128.25	164.253
20106	3.7572	98.3481	96.5961
20107	3.7573	116.365	84.0912
20108	3.7573	117.881	108.464
20109	3.7573	100.234	291.369
20110	3.7574	100.563	260.724
20111	3.7574	98.9776	142.817
20112	3.7575	98.7219	336.898
20113	3.7576	122.339	123.365
20114	3.7577	122.091	231.008
20115	3.7578	65.6753	163.038
20116	3.7578	110.518	183.919
20117	3.7578	99.6032	134.736
20118	3.7578	112.165	109.42
20119	3.7578	46.9202	139.787
20120	3.7581	129.248	99.3204
20121	3.7582	159.416	88.5499
20122	3.7583	140.884	13.0753
20123	3.7583	129.908	163.236
20124	3.7583	113.973	256.905
20125	3.7584	164.881	48.4774
20126	3.7584	97.2488	192.985
20127	3.7584	132.195	317.269
20128	3.7585	151.583	27.9506
20129	3.7585	135.631	180.418
20130	3.7587	140.038	196.013
20131	3.7587	-2.7254	14.951
20132	3.7587	136.578	30.9914
20133	3.7588	162.578	93.604
20134	3.7588	175.73	115.268
20135	3.7589	147.151	283.489
20136	3.7591	127.309	108.205
20137	3.7592	137.088	78.6087
20138	3.7595	144.747	284.596
20139	3.7596	133.591	188.022
20140	3.7596	92.4784	161.247
20141	3.7596	132.722	59.6868
20142	3.7597	116.392	59.0867
20143	3.7598	123.459	76.5575
20144	3.7599	105.374	10.473
20145	3.7601	161.031	201.646
20146	3.7602	151.295	248.13
20147	3.7602	96.4513	135.013
20148	3.7602	126.621	104.873
20149	3.7603	106.778	152.896

20150	3.7605	134.302	171.748
20151	3.7606	130.151	221.155
20152	3.7606	108.406	172.503
20153	3.7608	99.0808	154.369
20154	3.7609	129.74	300.173
20155	3.761	170.485	121.805
20156	3.761	145.199	116.612
20157	3.7612	143.693	233.724
20158	3.7614	-3.5071	108.425
20159	3.7615	87.1614	164.643
20160	3.7616	137.471	88.9043
20161	3.7616	96.3438	144.964
20162	3.7616	99.0379	47.9012
20163	3.7617	127.31	103.508
20164	3.7618	121.417	185.824
20165	3.762	87.959	284.713
20166	3.762	140.868	134.232
20167	3.7621	-2.7607	33.0284
20168	3.7622	126.348	273.014
20169	3.7623	154.489	135.258
20170	3.7623	128.406	73.4017
20171	3.7623	153.74	61.1294
20172	3.7623	122.295	158.77
20173	3.7624	154.364	40.0568
20174	3.7625	114.492	195.243
20175	3.7627	135.2	247.058
20176	3.7627	136.701	266.005
20177	3.7629	140.354	71.5426
20178	3.763	103.956	125.657
20179	3.763	117.486	167.873
20180	3.7631	150.376	319.247
20181	3.7631	138.933	30.9091
20182	3.7633	161.288	37.7498
20183	3.7635	116.582	188.01
20184	3.7635	105.869	255.362
20185	3.7637	-2.7757	125.407
20186	3.7638	136.038	267.315
20187	3.7638	132.742	215.616
20188	3.7639	140.416	60.9751
20189	3.7639	125.112	197.339
20190	3.764	144.909	352.243
20191	3.764	91.565	148.413
20192	3.764	134.221	183
20193	3.764	149.991	145.67
20194	3.7643	124.322	139.035
20195	3.7644	127.08	315.496
20196	3.7645	126.652	156.64
20197	3.7645	124.053	147.01

20198	3.7646	98.4738	217.536
20199	3.7647	141.339	113.614
20200	3.7647	137.076	353.264
20201	3.7647	134.425	16.8572
20202	3.7648	79.6446	4.953
20203	3.7648	132.928	218.421
20204	3.7648	124.945	74.4476
20205	3.7649	146.3	339.378
20206	3.7649	137.179	199.164
20207	3.7649	135.303	42.7773
20208	3.7649	142.611	188.892
20209	3.765	40.8488	48.745
20210	3.7651	136.633	141.457
20211	3.7651	144.779	22.6547
20212	3.7652	166.035	144.754
20213	3.7655	130.135	156.81
20214	3.7656	114.078	299.211
20215	3.7657	108.516	30.5718
20216	3.7658	185.636	314.857
20217	3.7658	120.479	99.4971
20218	3.7659	96.729	170.1
20219	3.7659	134.605	103.165
20220	3.7659	111.542	48.0508
20221	3.766	105.664	85.3618
20222	3.766	77.2507	186.129
20223	3.766	137.683	333.511
20224	3.7662	126.117	53.2804
20225	3.7664	145.803	128.257
20226	3.7666	132.343	276.573
20227	3.7667	108.502	62.654
20228	3.7667	146.898	85.3644
20229	3.7667	117.08	238.412
20230	3.7667	123.664	262.647
20231	3.7668	110.578	157.552
20232	3.767	120.795	337.291
20233	3.7672	149.247	198.942
20234	3.7672	114.373	268.779
20235	3.7673	140.503	49.9392
20236	3.7673	141.027	245.209
20237	3.7673	105.428	178.721
20238	3.7673	85.3453	157.702
20239	3.7674	150.048	111.174
20240	3.7674	143.647	33.5931
20241	3.7674	109.88	104.747
20242	3.7675	139.272	5.9674
20243	3.7677	75.1381	130.188
20244	3.7677	88.9225	38.136
20245	3.7678	142.079	149.199

20246	3.7678	123.282	219.368
20247	3.768	156.802	269.217
20248	3.768	149.107	124.238
20249	3.768	151.652	148.765
20250	3.768	133.446	278.022
20251	3.7681	92.5828	135.993
20252	3.7682	160.169	133.324
20253	3.7684	110.989	179.746
20254	3.7685	122.904	279.095
20255	3.7685	145.533	153.626
20256	3.7686	114.052	331.754
20257	3.7686	130.825	214.31
20258	3.7687	122.209	100.78
20259	3.7688	167.032	42.0329
20260	3.7689	117.712	215.216
20261	3.7689	102.715	176.742
20262	3.7689	114.454	60.2271
20263	3.769	128.63	72.34
20264	3.769	119.083	192.137
20265	3.769	118.693	198.705
20266	3.7691	132.702	151.267
20267	3.7691	104.048	252.496
20268	3.7693	99.1286	306.333
20269	3.7694	105.346	156.814
20270	3.7695	133.336	89.3865
20271	3.7695	135.047	91.8881
20272	3.7697	127.275	348.901
20273	3.7698	100.974	331.283
20274	3.7698	109.673	116.577
20275	3.7699	145.247	97.0168
20276	3.77	127.365	240.726
20277	3.77	111.54	98.8287
20278	3.7701	130.469	229.149
20279	3.7701	132.697	86.6663
20280	3.7702	138.046	274.898
20281	3.7703	117.724	134.437
20282	3.7703	128.509	58.796
20283	3.7703	117.324	74.732
20284	3.7705	124.398	93.6626
20285	3.7706	135.516	64.0413
20286	3.7706	141.947	92.0153
20287	3.7708	-2.834	123.194
20288	3.7709	127.885	165.177
20289	3.771	129.237	296.845
20290	3.771	126.747	122.068
20291	3.7712	-2.8667	141.637
20292	3.7713	106.829	68.1241
20293	3.7714	108.774	170.42

20294	3.7715	134.913	139.671
20295	3.7715	114.356	129.852
20296	3.7715	157.463	19.2512
20297	3.7716	103.164	175.927
20298	3.7717	146.31	101.608
20299	3.7717	108.539	188.406
20300	3.7717	119.05	101.567
20301	3.7718	143.175	110.221
20302	3.7718	108.334	258.932
20303	3.7719	140.052	295.511
20304	3.7719	119.155	170.499
20305	3.7724	143.077	229.11
20306	3.7724	128.635	177.074
20307	3.7724	116.62	63.2881
20308	3.7727	65.3581	79.1804
20309	3.7729	176.791	203.597
20310	3.7729	115.841	151.918
20311	3.7729	141.768	95.6457
20312	3.7731	130.876	222.169
20313	3.7731	110.532	66.922
20314	3.7735	135.419	151.465
20315	3.7735	137.78	43.5566
20316	3.7736	131.254	82.0876
20317	3.7736	88.1787	158.21
20318	3.7737	130.382	221.42
20319	3.7737	127.363	197.322
20320	3.7739	114.481	97.8978
20321	3.774	134.975	44.843
20322	3.774	122.567	134.884
20323	3.774	113.519	302.133
20324	3.774	124.538	64.9519
20325	3.7742	144.088	328.163
20326	3.7743	140.498	110.555
20327	3.7743	127.953	191.047
20328	3.7744	121.815	72.571
20329	3.7745	-2.7137	25.586
20330	3.7745	136.765	41.7361
20331	3.7745	137.242	78.4726
20332	3.7746	133.484	172.728
20333	3.7749	18.5111	77.9419
20334	3.775	146.738	173.284
20335	3.775	129.728	248.452
20336	3.7751	121.151	54.0082
20337	3.7751	139.19	329.034
20338	3.7751	152.477	110.357
20339	3.7752	151.944	229.689
20340	3.7754	134.585	154.176
20341	3.7755	147.447	231.407

20342	3.7755	134.639	99.9191
20343	3.7757	122.12	304.71
20344	3.7757	140.226	276.574
20345	3.7759	131.112	240.081
20346	3.7759	118.526	135.888
20347	3.776	157.157	135.361
20348	3.776	138.4	194.775
20349	3.7761	150.242	12.5172
20350	3.7761	101.453	192.426
20351	3.7761	130.464	137.442
20352	3.7762	136.026	286.376
20353	3.7762	117.783	52.3582
20354	3.7763	133.772	295.328
20355	3.7764	131.411	264.734
20356	3.7764	134.176	209.316
20357	3.7765	130.562	235.823
20358	3.7766	144.668	313.545
20359	3.7766	92.375	81.8731
20360	3.7768	124.153	45.5718
20361	3.7769	155.504	311.361
20362	3.777	95.8219	349.622
20363	3.7771	136.856	104.35
20364	3.7771	124.755	126.733
20365	3.7774	134.212	90.8579
20366	3.7774	101.785	296.528
20367	3.7774	109.524	165.086
20368	3.7775	175.727	143.506
20369	3.7775	109.293	132.714
20370	3.7776	144.085	39.8176
20371	3.7777	145.902	78.9027
20372	3.7777	102.681	51.9044
20373	3.7779	143.861	126.621
20374	3.7779	108.706	109.568
20375	3.778	94.0564	186.203
20376	3.778	119.077	218.892
20377	3.7781	118.889	95.8668
20378	3.7781	111.951	167.221
20379	3.7782	139.25	229.555
20380	3.7783	91.4729	350.275
20381	3.7783	16.5211	267.368
20382	3.7784	166.376	46.3321
20383	3.7784	106.568	101.776
20384	3.7784	124.972	86.3314
20385	3.7786	128.465	86.7549
20386	3.7786	125.857	245.25
20387	3.7786	129.328	111.927
20388	3.7786	153.439	148.284
20389	3.7786	131.586	64.6467

20390	3.7787	122.431	197.228
20391	3.7787	118.884	224.059
20392	3.779	98.721	319.354
20393	3.779	117.129	223.15
20394	3.7792	116.104	91.1252
20395	3.7792	114.343	245.81
20396	3.7793	122.734	167.554
20397	3.7794	155.713	55.6703
20398	3.7796	141.106	300.57
20399	3.7797	98.8173	145.676
20400	3.7798	168.48	240.176
20401	3.7798	110.502	165.201
20402	3.7799	99.1618	98.9983
20403	3.78	124.149	184.003
20404	3.78	133.683	62.0119
20405	3.7801	155.073	92.1429
20406	3.7807	135.231	62.6209
20407	3.7807	133.234	107.829
20408	3.7808	145.518	26.3063
20409	3.7808	116.754	61.1792
20410	3.7809	146.775	292.005
20411	3.7809	141.479	191.425
20412	3.781	140.887	319.547
20413	3.781	131.836	283.099
20414	3.781	135.372	10.1162
20415	3.781	135.976	97.9653
20416	3.7811	113.981	331.052
20417	3.7812	114.618	196.505
20418	3.7812	128.654	111.451
20419	3.7813	125.103	135.478
20420	3.7813	157.399	20.1614
20421	3.7814	162.356	122.303
20422	3.7815	116.564	177.619
20423	3.7815	143.597	55.2839
20424	3.7816	135.826	109.457
20425	3.7816	106.292	178.812
20426	3.7816	135.318	112.551
20427	3.7817	102.147	183.854
20428	3.782	144.35	92.7964
20429	3.782	124.763	223.632
20430	3.782	118.528	253.049
20431	3.7821	119.865	272.758
20432	3.7821	144.499	162.503
20433	3.7822	138.313	286.549
20434	3.7822	116.802	103.212
20435	3.7823	133.443	106.001
20436	3.7823	116.455	135.832
20437	3.7824	93.4583	159.847

20438	3.7825	133.416	41.4476
20439	3.7825	143.574	201.427
20440	3.7826	119.717	70.3074
20441	3.7827	126.331	230.432
20442	3.7828	103.515	26.6036
20443	3.7828	128.226	148.467
20444	3.7828	113.539	244.43
20445	3.783	162.134	69.5403
20446	3.7831	118.855	236.449
20447	3.7831	136.635	336.006
20448	3.7834	179.682	86.0606
20449	3.7835	135.698	174.695
20450	3.7835	118.828	167.656
20451	3.7836	144.675	32.5728
20452	3.7837	131.217	85.7158
20453	3.7837	122.025	168.017
20454	3.7837	190.619	310.453
20455	3.7838	110.28	175.72
20456	3.7839	112.694	217.418
20457	3.784	109.873	106.942
20458	3.784	127.532	40.1335
20459	3.7841	-2.8166	144.896
20460	3.7841	130.685	139.091
20461	3.7841	134.402	174.777
20462	3.7842	120.416	196.562
20463	3.7842	133.989	64.1132
20464	3.7842	151.333	67.1608
20465	3.7843	106.698	172.845
20466	3.7844	128.925	76.372
20467	3.7846	144.424	137.262
20468	3.7848	162.846	353.491
20469	3.7849	134.29	160.556
20470	3.7853	153.811	37.4707
20471	3.7853	128.071	102.872
20472	3.7855	119.578	15.5684
20473	3.7855	-2.8279	139.071
20474	3.7856	105.674	337.093
20475	3.7857	108.432	197.147
20476	3.7858	108.432	245.332
20477	3.7858	136.369	354.216
20478	3.786	131.971	111.955
20479	3.786	112.3	184.173
20480	3.7862	130.692	144.21
20481	3.7862	143.456	152.194
20482	3.7863	143.569	44.5864
20483	3.7864	119.805	76.7198
20484	3.7864	115.926	168.731
20485	3.7866	110.347	104.544

20486	3.7866	123.4	304.462
20487	3.7866	107.807	90.1731
20488	3.7867	167.202	118.433
20489	3.7868	-0.4716	231.013
20490	3.7868	141.516	97.0526
20491	3.7869	129.381	201.562
20492	3.7871	133.137	163.122
20493	3.7871	96.1405	219.402
20494	3.7873	156.201	293.393
20495	3.7873	134.783	33.229
20496	3.7876	129.815	115.699
20497	3.7876	164.404	334.824
20498	3.7876	115.592	67.756
20499	3.7877	164.724	246.487
20500	3.7878	128.532	72.2246
20501	3.7879	124.961	221.176
20502	3.7879	127.679	176.962
20503	3.7879	152.482	215.226
20504	3.788	128.541	57.6632
20505	3.788	78.406	130.912
20506	3.788	124.861	84.64
20507	3.7881	101.737	153.948
20508	3.7882	152.022	117.884
20509	3.7882	100.2	169.41
20510	3.7883	113.544	84.2048
20511	3.7883	121.081	42.5475
20512	3.7884	169.221	250.566
20513	3.7884	155.628	268.345
20514	3.7884	135.668	144.415
20515	3.7884	141.54	47.626
20516	3.7885	101.702	133.164
20517	3.7887	140.173	135.21
20518	3.7887	148.85	95.531
20519	3.7888	118.762	244.952
20520	3.789	131.074	117.61
20521	3.7891	133.779	256.122
20522	3.7892	143.088	203.555
20523	3.7892	148.82	340.062
20524	3.7893	79.648	166.287
20525	3.7893	77.8541	141.865
20526	3.7893	142.783	352.548
20527	3.7894	153.966	84.6505
20528	3.7894	99.2332	80.8065
20529	3.7895	138.765	64.304
20530	3.7895	135.959	280.41
20531	3.7896	125.482	66.7524
20532	3.7898	146.839	70.7793
20533	3.7898	115.535	168.218

20534	3.7898	128.53	241.231
20535	3.7899	121.494	137.557
20536	3.7901	133.593	358.787
20537	3.7901	128.565	97.6665
20538	3.7902	121.324	118.198
20539	3.7903	126.086	71.4924
20540	3.7904	148.46	328.272
20541	3.7904	146.526	108.9
20542	3.7904	135.558	310.814
20543	3.7906	179.514	131.765
20544	3.7906	108.497	169.497
20545	3.7907	146.192	55.9515
20546	3.7907	154.269	322.935
20547	3.7908	171.368	72.4168
20548	3.7908	113.066	261.845
20549	3.7909	133.36	172.628
20550	3.7909	91.7791	192.406
20551	3.791	113.329	99.676
20552	3.791	110.543	137.701
20553	3.7911	162.526	32.9808
20554	3.7911	134.339	235.844
20555	3.7912	114.779	104.251
20556	3.7912	147.209	171.971
20557	3.7913	126.427	140.449
20558	3.7916	134.527	9.144
20559	3.7918	108.649	40.5101
20560	3.7918	132.073	191.201
20561	3.7919	107.565	213.052
20562	3.7919	138.924	20.2064
20563	3.792	100.493	285.305
20564	3.7924	120.674	107.861
20565	3.7924	110.83	168.256
20566	3.7924	54.0797	189.199
20567	3.7926	149.716	50.2661
20568	3.7927	152.505	118.691
20569	3.7927	147.147	76.8426
20570	3.7928	130.546	109.557
20571	3.7929	140.814	350.381
20572	3.7929	140.962	162.928
20573	3.793	154.099	289.21
20574	3.7931	119.524	177.837
20575	3.7932	133.66	349.506
20576	3.7932	157.239	330.989
20577	3.7933	159.727	311.772
20578	3.7933	129.939	147.296
20579	3.7933	105.315	107.974
20580	3.7934	149.935	59.8149
20581	3.7935	190.606	303.101

20582	3.7935	137.648	281.595
20583	3.7936	134.699	59.4798
20584	3.7939	139.992	41.576
20585	3.7939	5.3381	109.927
20586	3.794	156.695	214.956
20587	3.794	123.312	179.093
20588	3.7941	149.184	229.067
20589	3.7941	1.9021	113.105
20590	3.7942	143.123	109.606
20591	3.7942	143.555	169.422
20592	3.7944	117.957	156.28
20593	3.7945	132.6	226.909
20594	3.7945	120.143	181.274
20595	3.7947	152.934	229.43
20596	3.7947	79.4757	117.184
20597	3.7948	107.086	308.689
20598	3.7948	126.091	159.705
20599	3.7949	122.705	58.9076
20600	3.7949	130.533	198.794
20601	3.7949	145.782	38.0307
20602	3.7949	119.011	215.98
20603	3.795	160.521	59.0785
20604	3.795	118.331	78.8239
20605	3.7951	127.172	335.984
20606	3.7952	161.32	210.039
20607	3.7953	132.133	168.635
20608	3.7953	128.296	269.74
20609	3.7954	132.222	102.387
20610	3.7955	148.403	291.036
20611	3.7957	157.388	105.799
20612	3.7957	124.6	196.618
20613	3.7958	116.52	137.426
20614	3.7958	143.518	105.017
20615	3.796	147.119	57.9385
20616	3.796	158.688	344.9
20617	3.7961	149.148	40.7478
20618	3.7961	152.997	178.261
20619	3.7962	126.33	227.18
20620	3.7962	134.002	195.93
20621	3.7968	144.119	262.379
20622	3.7968	145.228	202.037
20623	3.7968	126.561	199.811
20624	3.7968	115.81	140.081
20625	3.7969	125.916	233.165
20626	3.797	169.52	282.926
20627	3.797	129.15	279.24
20628	3.797	126.004	170.078
20629	3.7972	129.18	253.235

20630	3.7974	132.735	78.3383
20631	3.7974	123.068	142.039
20632	3.7975	177.957	40.6213
20633	3.7976	129.372	69.4247
20634	3.7978	140.269	208.305
20635	3.7978	163.387	39.4387
20636	3.7981	169.998	58.5429
20637	3.7981	113.27	151.907
20638	3.7981	131.028	126.545
20639	3.7983	157.72	43.2938
20640	3.7983	98.5609	224.158
20641	3.7983	118.742	64.9463
20642	3.7984	150.046	337.499
20643	3.7985	122.388	335.543
20644	3.7987	138.144	210.79
20645	3.7987	149.699	231.325
20646	3.7987	125.303	324.992
20647	3.7989	147.901	78.7444
20648	3.799	152.634	140.975
20649	3.799	112.985	162.034
20650	3.799	109.992	349.472
20651	3.7991	164.869	285.283
20652	3.7992	149	309.652
20653	3.7992	119.659	100.212
20654	3.7993	196.495	260.407
20655	3.7993	115.681	221.688
20656	3.7994	144.319	318.662
20657	3.7994	142.625	167.584
20658	3.7995	170.038	41.64
20659	3.7995	127.734	34.7225
20660	3.7996	119.017	147.625
20661	3.7997	112.847	177.52
20662	3.7997	116.044	122.02
20663	3.7997	128.684	86.201
20664	3.7997	132.536	206.001
20665	3.7998	144.712	197.089
20666	3.7998	138.786	292.766
20667	3.7999	128.022	158.447
20668	3.7999	152.937	196.448
20669	3.7999	137.809	145.214
20670	3.8001	153.076	108.058
20671	3.8001	134.677	133.538
20672	3.8002	123.387	359.891
20673	3.8003	128.169	77.7625
20674	3.8004	133.521	120.029
20675	3.8005	126.552	94.3423
20676	3.8006	148.147	67.4424
20677	3.8006	135.244	115.022

20678	3.8007	212.093	1.0884
20679	3.8007	117.699	65.6534
20680	3.8008	102.801	142.866
20681	3.8009	126.714	178.842
20682	3.801	131.276	324.472
20683	3.801	105.241	232.145
20684	3.8012	141.097	145.882
20685	3.8012	108.847	145.991
20686	3.8012	158.583	330.461
20687	3.8013	117.553	9.3114
20688	3.8014	71.1444	25.0147
20689	3.8014	142.782	213.993
20690	3.8015	149.326	261.027
20691	3.8015	141.364	129.767
20692	3.8015	132.285	145.885
20693	3.8016	177.225	258.665
20694	3.8017	118.23	69.1545
20695	3.8018	129.439	116.524
20696	3.8018	140.704	218.008
20697	3.8019	114.293	232.426
20698	3.802	86.6731	349.725
20699	3.8021	143.927	150.959
20700	3.8021	152.646	58.3054
20701	3.8022	128.745	109.797
20702	3.8024	144.948	71.2231
20703	3.8025	121.11	242.503
20704	3.8027	134.19	85.832
20705	3.8029	121.872	216.173
20706	3.803	140.658	105.852
20707	3.803	154.615	239.839
20708	3.8031	157.721	60.8529
20709	3.8031	108.713	165.13
20710	3.8032	122.521	194.474
20711	3.8033	126.447	3.0118
20712	3.8034	117.304	100.728
20713	3.8034	146.052	175.69
20714	3.8035	100.905	312.044
20715	3.8036	176.425	111.411
20716	3.8036	140.648	118.478
20717	3.8037	113.039	144.471
20718	3.8038	97.8378	221.482
20719	3.8038	143.621	273.317
20720	3.8039	156.309	245.958
20721	3.8039	160.581	304.25
20722	3.8039	139.603	116.216
20723	3.8039	114.963	105.363
20724	3.8039	185.214	260.752
20725	3.8041	183.508	241.247

20726	3.8041	142.896	114
20727	3.8041	130.589	2.7673
20728	3.8041	128.655	92.8355
20729	3.8042	148.673	223.394
20730	3.8042	113.641	203.368
20731	3.8043	140.689	132.726
20732	3.8043	133.932	114.698
20733	3.8043	149.723	35.7279
20734	3.8044	125.89	282.293
20735	3.8046	137.855	306.848
20736	3.8046	139.53	24.054
20737	3.8047	110.516	242.789
20738	3.8048	184.642	310.857
20739	3.8049	120.576	167.941
20740	3.805	140.781	100.518
20741	3.8052	103.103	111.355
20742	3.8053	142.511	77.1405
20743	3.8055	155.644	113.479
20744	3.8056	76.372	66.3219
20745	3.8057	130.17	280.231
20746	3.8058	135.196	316.567
20747	3.8059	151.608	215.178
20748	3.8061	79.426	21.301
20749	3.8062	84.1997	250.134
20750	3.8063	149.176	78.8361
20751	3.8063	40.4279	122.611
20752	3.8064	187.058	92.5314
20753	3.8064	2.0549	196.814
20754	3.8066	174.735	57.9758
20755	3.8066	126.675	124.131
20756	3.8066	119.831	120.113
20757	3.8067	141.527	312.031
20758	3.8067	87.7788	160.139
20759	3.8068	155.086	223.859
20760	3.8068	121.749	130.167
20761	3.8069	156.415	287.627
20762	3.807	100.304	32.7896
20763	3.807	152.418	237.343
20764	3.8071	137.457	177.459
20765	3.8071	142.94	104.848
20766	3.8071	152.118	58.7035
20767	3.8072	150.591	109.528
20768	3.8074	136.862	31.9066
20769	3.8075	121.256	127.565
20770	3.8076	166.092	287.678
20771	3.8079	112.349	173.436
20772	3.8079	140.535	219.245
20773	3.808	158.86	290.443

20774	3.8081	125.952	161.625
20775	3.8081	63.8945	79.4643
20776	3.8082	152.094	347.258
20777	3.8082	115.188	118.283
20778	3.8082	104.937	159.731
20779	3.8082	134.576	221.073
20780	3.8083	118.911	220.137
20781	3.8083	113.598	188.788
20782	3.8084	116.73	161.131
20783	3.8084	130.661	101.304
20784	3.8084	114.395	223.204
20785	3.8086	170.228	21.7502
20786	3.8086	130.528	217.605
20787	3.8086	133.694	164.823
20788	3.8087	118.941	300.427
20789	3.8088	135.643	139.881
20790	3.809	82.5161	109.978
20791	3.809	90.7755	128.877
20792	3.809	106.176	297.949
20793	3.8091	106.342	109.099
20794	3.8091	137.464	312.383
20795	3.8091	123.762	333.817
20796	3.8092	152.906	257.022
20797	3.8093	141.927	321.324
20798	3.8094	136.651	271.586
20799	3.8095	129.672	157.946
20800	3.8097	150.946	78.715
20801	3.8098	132.799	10.575
20802	3.8098	148.417	109.245
20803	3.8099	151.006	201.507
20804	3.8099	142.953	140.389
20805	3.8101	120.057	233.159
20806	3.8103	121.634	296.182
20807	3.8103	107.765	176.347
20808	3.8103	135.645	80.0001
20809	3.8105	140.079	33.0825
20810	3.8106	116.858	169.421
20811	3.8107	128.243	329.717
20812	3.8107	111.237	162.05
20813	3.8107	65.5633	150.767
20814	3.8108	138.094	275.107
20815	3.8108	201.008	149.42
20816	3.811	126.847	197.816
20817	3.811	115.863	109.403
20818	3.811	130.556	36.0229
20819	3.811	147.012	107.888
20820	3.811	143.761	14.1794
20821	3.8111	147.591	113.688

20822	3.8111	145.962	34.8752
20823	3.8111	171.632	118.965
20824	3.8112	131.734	241.688
20825	3.8113	-4.0926	259.834
20826	3.8113	117.678	11.0154
20827	3.8114	98.6569	170.013
20828	3.8114	146.932	28.6476
20829	3.8115	114.196	250.834
20830	3.8115	147.079	34.5678
20831	3.8117	118.002	64.3258
20832	3.8117	109.703	296.747
20833	3.8117	110.437	112.505
20834	3.8117	133.959	106.137
20835	3.8118	191.112	4.6482
20836	3.8118	115.677	5.2008
20837	3.8119	146.048	22.7195
20838	3.8119	145.738	138.019
20839	3.8119	151.461	331.484
20840	3.812	131.763	114.111
20841	3.8121	132.775	113.038
20842	3.8122	132.712	103.111
20843	3.8123	80.9164	114.966
20844	3.8123	160.864	64.8578
20845	3.8124	132.39	83.8983
20846	3.8125	124.757	99.0355
20847	3.8125	124.054	122.207
20848	3.8126	157.82	320.679
20849	3.8126	127.042	110.249
20850	3.8129	123.598	65.5985
20851	3.813	185.02	223.594
20852	3.8131	149.04	221.647
20853	3.8131	-2.7307	16.7094
20854	3.8133	116.351	130.613
20855	3.8134	178.336	212.597
20856	3.8135	112.447	186.751
20857	3.8136	138.114	150.924
20858	3.8137	140.029	83.1189
20859	3.8138	132.872	279.498
20860	3.8139	144.892	99.0499
20861	3.814	112.547	319.431
20862	3.814	143.178	191.506
20863	3.8141	103.281	161.351
20864	3.8143	170.233	49.1048
20865	3.8143	167.155	161.862
20866	3.8144	147.398	105.918
20867	3.8145	127.386	169.537
20868	3.8145	151.103	287.711
20869	3.8146	137.646	103.251

20870	3.8147	173.53	42.4987
20871	3.8147	165.761	313.405
20872	3.8147	148.518	114.554
20873	3.8147	46.7641	357.641
20874	3.8149	147.407	212.784
20875	3.8149	147.78	108.309
20876	3.8149	112.886	79.9427
20877	3.8149	123.792	121.371
20878	3.8149	122.19	264.535
20879	3.8149	185.66	314.753
20880	3.8149	158.415	35.2383
20881	3.8149	131.833	112.417
20882	3.8152	133.856	330.208
20883	3.8153	147.475	94.6993
20884	3.8153	142.731	314.95
20885	3.8154	155.498	61.349
20886	3.8156	147.547	113.121
20887	3.8159	153.726	20.6732
20888	3.816	124.814	251.719
20889	3.816	115.921	84.9553
20890	3.816	131.273	330.541
20891	3.816	145.79	143.649
20892	3.8161	141.896	139.29
20893	3.8161	114.021	352.246
20894	3.8162	121.079	141.429
20895	3.8164	117.695	113.99
20896	3.8165	87.2809	172.657
20897	3.8166	-2.807	306.452
20898	3.8169	160.452	83.0858
20899	3.817	127.91	272.889
20900	3.817	109.112	76.8299
20901	3.817	166.586	326.532
20902	3.8172	125.555	72.7815
20903	3.8173	120.976	151.779
20904	3.8173	131.496	56.2071
20905	3.8175	116.123	102.619
20906	3.8176	87.1467	190.939
20907	3.8176	124.275	318.195
20908	3.8178	90.851	278.57
20909	3.8178	126.059	290.287
20910	3.8179	113.422	157.638
20911	3.818	116.606	198.275
20912	3.818	129.489	230.501
20913	3.8182	139.918	218.646
20914	3.8183	106.574	174.449
20915	3.8185	149.106	299.284
20916	3.8186	139.397	307.009
20917	3.8186	149.464	87.5745

20918	3.8186	-2.7362	15.375
20919	3.8186	152.177	72.8351
20920	3.8187	105.921	359.457
20921	3.8187	-2.6861	252.522
20922	3.8187	133.62	288.29
20923	3.8188	126.829	261.98
20924	3.8188	136.816	100.487
20925	3.8189	137.987	52.3148
20926	3.819	155.033	95.1177
20927	3.8191	123.381	186.623
20928	3.8191	94.5756	189.761
20929	3.8193	154.012	113.053
20930	3.8198	142.339	133.085
20931	3.8198	157.353	278.083
20932	3.8198	154.786	142.852
20933	3.8199	152.293	31.3998
20934	3.8201	166.223	38.981
20935	3.8201	123.892	55.0048
20936	3.8201	163.221	311.788
20937	3.8202	190.799	11.9432
20938	3.8202	139.221	200.861
20939	3.8203	155.323	130.09
20940	3.8203	137.287	134
20941	3.8203	132.019	208.608
20942	3.8204	132.232	269.931
20943	3.8204	120.072	38.1222
20944	3.8205	144.233	237.885
20945	3.8206	137.73	171.57
20946	3.8206	144.314	212.864
20947	3.8208	151.154	87.4642
20948	3.8208	165.488	148.241
20949	3.8209	124.124	85.2918
20950	3.821	147.418	100.25
20951	3.821	181.046	283.27
20952	3.8212	123.601	166.293
20953	3.8212	154.544	156.863
20954	3.8215	159.347	77.1533
20955	3.8215	110.652	180.784
20956	3.8216	119.656	158.893
20957	3.8217	187.168	248.713
20958	3.8217	138.056	148.433
20959	3.8218	140.464	125.437
20960	3.8218	135.922	148.044
20961	3.8219	138.668	114.614
20962	3.8221	133.343	44.9759
20963	3.8221	134.401	283.948
20964	3.8221	119.725	195.069
20965	3.8222	164.246	274.109

20966	3.8224	127.836	35.5427
20967	3.8227	154.821	65.7893
20968	3.8229	99.385	32.2273
20969	3.8231	145.794	21.1396
20970	3.8234	144.773	199.803
20971	3.8234	137.729	118.009
20972	3.8234	130.583	100.771
20973	3.8235	140.793	71.6742
20974	3.8235	148.03	95.9446
20975	3.8235	126.582	197.211
20976	3.8235	150.703	194.033
20977	3.8237	125.11	72.7442
20978	3.8237	165.804	98.4322
20979	3.8238	118.017	119.65
20980	3.8238	155.85	113.177
20981	3.824	140.179	62.1308
20982	3.8242	96.5035	109.995
20983	3.8243	124.24	189.432
20984	3.8245	143.068	174.598
20985	3.8246	121.32	194.012
20986	3.825	109.7	258.061
20987	3.825	145.274	134.97
20988	3.8252	154.753	145.747
20989	3.8252	116.716	217.002
20990	3.8252	133.344	52.0082
20991	3.8253	151.941	177.166
20992	3.8253	132.69	190.158
20993	3.8253	151.793	203.984
20994	3.8254	142.179	19.7354
20995	3.8254	108.465	9.9895
20996	3.8255	106.376	192.305
20997	3.8255	66.8097	164.546
20998	3.8256	130.51	89.9536
20999	3.8257	175.222	141.921
21000	3.8257	117.165	146.008
21001	3.8259	140.039	63.7977
21002	3.826	130.498	78.7148
21003	3.8261	161.652	67.5101
21004	3.8262	152.648	87.454
21005	3.8263	128.393	117.311
21006	3.8263	111.956	171.885
21007	3.8264	164.243	23.4421
21008	3.8266	147.53	351.12
21009	3.8266	113.335	168.482
21010	3.8266	117.028	347.387
21011	3.8267	125.17	173.278
21012	3.8267	142.543	220.187
21013	3.8268	156.446	89.3754

21014	3.8268	131.193	347.579
21015	3.8269	120.122	112.584
21016	3.8269	154.337	82.8663
21017	3.8269	108.482	101.317
21018	3.8269	140.768	275.093
21019	3.8271	65.6204	154.932
21020	3.8271	144.53	83.8349
21021	3.8272	100.784	165.701
21022	3.8273	159.406	40.1782
21023	3.8273	129.906	195.668
21024	3.8274	138.721	72.8444
21025	3.8274	135.424	72.7441
21026	3.8274	87.9521	61.5774
21027	3.8275	92.743	306.107
21028	3.8275	144.321	71.9894
21029	3.8276	140.996	101.863
21030	3.8276	141.539	11.1171
21031	3.8277	127.536	194.072
21032	3.8278	137.655	198.18
21033	3.8278	115.237	147.496
21034	3.8282	145.834	279.626
21035	3.8283	177.37	34.4213
21036	3.8283	159.268	347.734
21037	3.8283	136.302	146.567
21038	3.8283	138.854	262.423
21039	3.8284	162.313	349.506
21040	3.8284	146.466	244.203
21041	3.8285	146.917	160.2
21042	3.8285	152.668	281.209
21043	3.8285	114.105	290.96
21044	3.8286	156.732	128.719
21045	3.8287	159.175	12.4176
21046	3.8287	115.534	147.516
21047	3.8287	135.36	145.846
21048	3.8288	125.412	92.977
21049	3.8289	162.566	89.5165
21050	3.8289	99.4156	193.894
21051	3.8292	132.954	69.4934
21052	3.8293	142.959	248.701
21053	3.8293	153.509	43.8009
21054	3.8294	134.76	307.227
21055	3.8295	129.809	359.674
21056	3.8296	141.924	225.54
21057	3.8296	138.163	61.4219
21058	3.8298	148.12	149.662
21059	3.8298	123.397	113.124
21060	3.8299	146.029	292.047
21061	3.83	54.3727	354.527

21062	3.8301	136.379	11.2365
21063	3.8301	144.542	113.784
21064	3.8302	137.055	94.4004
21065	3.8303	168.213	49.028
21066	3.8304	143.42	178.396
21067	3.8304	116.412	271.453
21068	3.8304	141.866	113.017
21069	3.8306	179.763	203.498
21070	3.8307	60.0796	151.064
21071	3.8307	126.256	82.3392
21072	3.8309	117.659	71.78
21073	3.831	149.974	74.3451
21074	3.8312	135.555	10.6677
21075	3.8312	122.3	3.1695
21076	3.8313	134.415	27.5332
21077	3.8313	122.646	172.661
21078	3.8315	92.5778	195.411
21079	3.8315	139.433	224.859
21080	3.8316	125.674	147.403
21081	3.8317	107.586	150.496
21082	3.8317	149.013	281.925
21083	3.8319	144.175	151.07
21084	3.8321	116.574	295.938
21085	3.8321	136.477	104.114
21086	3.8322	133.702	7.2664
21087	3.8322	140.448	126.86
21088	3.8322	102.85	258.473
21089	3.8322	179.446	285.486
21090	3.8323	139.063	276.3
21091	3.8324	153.737	135.704
21092	3.8325	115.025	219.821
21093	3.8325	163.002	107.533
21094	3.8326	134.789	111.313
21095	3.8327	-2.7987	132.764
21096	3.8328	140.06	114.592
21097	3.8329	130.806	92.0252
21098	3.8329	174.518	38.986
21099	3.8329	131.828	355.853
21100	3.833	125.243	49.8091
21101	3.833	108.172	179.461
21102	3.833	150.881	35.0573
21103	3.8331	149.352	74.8955
21104	3.8331	134.66	129.118
21105	3.8331	169.037	262.735
21106	3.8332	124.379	313.087
21107	3.8333	193.33	205.517
21108	3.8333	159.408	147.97
21109	3.8335	137.317	217.789

21110	3.8335	155.906	258.788
21111	3.8336	142.95	70.5218
21112	3.8336	138.656	117.295
21113	3.8336	96.3066	358.6
21114	3.8337	129.101	128.585
21115	3.8339	87.0921	78.5227
21116	3.834	150.808	39.7116
21117	3.834	153.644	91.9394
21118	3.834	26.9319	139.943
21119	3.834	76.9674	173.033
21120	3.8341	-4.0483	195.665
21121	3.8342	151.639	251.319
21122	3.8344	137.45	160.877
21123	3.8345	97.0506	114.671
21124	3.8345	137.911	217.234
21125	3.8346	133.523	271.973
21126	3.8347	132.203	3.5996
21127	3.8347	102.022	323.007
21128	3.8347	102.892	5.1449
21129	3.8348	124.842	226.355
21130	3.8349	155.682	33.2358
21131	3.835	171.542	327.033
21132	3.8351	145.03	57.8552
21133	3.8351	143.49	98.7079
21134	3.8351	141.588	207.759
21135	3.8351	163.013	0.4092
21136	3.8352	145.34	151.963
21137	3.8352	182.108	208.167
21138	3.8352	139.407	141.088
21139	3.8353	121.799	141.074
21140	3.8355	139.425	209.12
21141	3.8355	112.919	318.301
21142	3.8356	145.081	358.092
21143	3.8356	136.991	111.787
21144	3.8356	144.126	6.8457
21145	3.8356	150.898	26.3798
21146	3.8357	142.973	91.9376
21147	3.8358	140.396	227.682
21148	3.8358	160.995	107.145
21149	3.8359	113.261	307.168
21150	3.836	183.815	58.0215
21151	3.8361	121.489	307.276
21152	3.8362	158.064	297.715
21153	3.8364	70.2837	12.903
21154	3.8364	109.416	197.668
21155	3.8364	141.191	94.9099
21156	3.8365	157.926	74.3859
21157	3.8365	139.726	107.804

21158	3.8365	135.172	114.338
21159	3.8366	138.224	113.512
21160	3.8366	127.628	80.2098
21161	3.8367	130.378	135.387
21162	3.8367	133.817	70.7853
21163	3.8367	-2.8291	201.055
21164	3.8368	145.494	154.548
21165	3.837	109.441	182.146
21166	3.8371	180.797	96.6151
21167	3.8371	160.937	132.946
21168	3.8372	144.394	272.519
21169	3.8373	122.285	197.751
21170	3.8374	171.334	57.019
21171	3.8374	132.378	315.045
21172	3.8375	138.663	107.349
21173	3.8375	129.463	99.9826
21174	3.8375	148.233	26.2624
21175	3.8376	130.825	64.8185
21176	3.8377	147.477	231.181
21177	3.8377	138.258	321.791
21178	3.8377	158.726	270.897
21179	3.8378	130.274	71.0175
21180	3.8378	87.0463	125.663
21181	3.8379	133.457	28.6236
21182	3.8381	147.231	78.0771
21183	3.8382	183.038	36.3081
21184	3.8383	143.603	168.139
21185	3.8383	123.649	133.625
21186	3.8384	110.355	106.529
21187	3.8384	126.842	110.969
21188	3.8384	146.235	128.696
21189	3.8385	156.829	268.94
21190	3.8385	172.255	117.881
21191	3.8386	179.882	41.8638
21192	3.8386	126.453	108.623
21193	3.8387	145.724	270.877
21194	3.8388	117.837	150.991
21195	3.8389	112.978	22.2609
21196	3.8389	156.59	288.167
21197	3.8389	144.587	43.7992
21198	3.839	87.5503	138.364
21199	3.839	-2.7013	249.886
21200	3.839	138.088	170.394
21201	3.8391	155.678	139.648
21202	3.8393	-2.7165	15.0605
21203	3.8393	144.303	77.674
21204	3.8393	127.513	162.508
21205	3.8393	130.271	338.488

21206	3.8394	137.189	250.694
21207	3.8395	126.884	161.334
21208	3.8397	139.15	299.059
21209	3.8397	150.003	279.399
21210	3.8397	148.985	83.3719
21211	3.8397	129.668	123.307
21212	3.8398	131.033	188.834
21213	3.8398	134.432	117.247
21214	3.8399	174.808	74.1932
21215	3.84	165.069	258.125
21216	3.84	147.756	275.268
21217	3.8401	117.169	118.053
21218	3.8401	150.318	67.7688
21219	3.8402	184.5	89.7843
21220	3.8402	149.255	49.8072
21221	3.8402	163.488	61.9049
21222	3.8402	135.846	175.101
21223	3.8402	127.477	131.12
21224	3.8405	110.375	207.089
21225	3.8405	149.597	141.203
21226	3.8407	150.128	83.7224
21227	3.8407	157.883	200.059
21228	3.8409	35.7182	124.904
21229	3.841	129.362	154.163
21230	3.8412	137.769	211.444
21231	3.8413	134.494	193.023
21232	3.8414	98.5472	137.778
21233	3.8417	158.172	5.3203
21234	3.8418	200.776	54.3163
21235	3.8419	102.004	94.9915
21236	3.8419	86.5639	86.4041
21237	3.8419	145.175	238.691
21238	3.8421	156.27	170.755
21239	3.8422	148.789	104.809
21240	3.8422	123.826	209.235
21241	3.8422	132.209	288.606
21242	3.8423	164.608	51.7703
21243	3.8424	145.144	75.7936
21244	3.8424	125.23	108.424
21245	3.8425	141.303	107.289
21246	3.8426	155.248	349.736
21247	3.8426	155.34	214.016
21248	3.8426	104.433	185.536
21249	3.8427	142.127	78.1074
21250	3.8428	144.713	107.289
21251	3.843	139.711	117.079
21252	3.8432	147.871	193.553
21253	3.8432	115.532	55.1458

21254	3.8432	152.944	109.657
21255	3.8433	128.208	104.694
21256	3.8433	135.961	278.164
21257	3.8433	147.459	220.475
21258	3.8434	161.85	319.973
21259	3.8434	105.561	10.401
21260	3.8434	150.211	292.148
21261	3.8435	102.84	115.292
21262	3.8435	-2.6847	26.7005
21263	3.8435	147.292	217.326
21264	3.8436	122.825	245.592
21265	3.8436	176.39	332.889
21266	3.8438	-2.7386	10.4995
21267	3.8439	152.893	114.333
21268	3.8439	164.811	132.406
21269	3.844	158.734	105.228
21270	3.844	108.754	79.3399
21271	3.8441	186.884	118.209
21272	3.8441	115.678	136.159
21273	3.8441	197.391	314.867
21274	3.8443	158.253	247.727
21275	3.8443	119.949	126.083
21276	3.8444	186.49	68.7687
21277	3.8445	132.365	102.143
21278	3.8448	190.357	124.181
21279	3.8448	127.91	109.013
21280	3.8448	157.758	97.4217
21281	3.845	166.275	40.5524
21282	3.845	152.655	21.8178
21283	3.8451	105.925	189.435
21284	3.8451	243.977	312.266
21285	3.8452	178.72	59.8323
21286	3.8452	158.057	69.1288
21287	3.8454	148.101	93.0666
21288	3.8454	165.431	33.4169
21289	3.8455	160.868	100.988
21290	3.8455	132.346	75.7052
21291	3.8455	138.722	140.817
21292	3.8456	139.44	248.198
21293	3.8458	120.633	65.2394
21294	3.8458	122.938	91.4621
21295	3.8458	154.761	321.136
21296	3.8458	121.062	25.2064
21297	3.8459	176.905	40.6738
21298	3.8459	156.409	42.5587
21299	3.846	155.927	216.428
21300	3.8462	147.331	51.9807
21301	3.8464	150.349	264.711

21302	3.8464	150.109	74.6275
21303	3.8464	109.651	103.379
21304	3.8465	150.268	297.701
21305	3.8465	152.555	166.978
21306	3.8466	166.724	104.479
21307	3.8467	117.514	258.65
21308	3.8468	145.031	172.998
21309	3.8468	133.039	157.422
21310	3.8469	145.668	170.053
21311	3.847	144.509	130.2
21312	3.847	231.741	311.11
21313	3.8473	138.109	134.453
21314	3.8473	115.131	119.456
21315	3.8474	116.563	201.036
21316	3.8474	156.73	49.3802
21317	3.8474	130.722	143.865
21318	3.8475	113.282	223.198
21319	3.8479	148.143	249.209
21320	3.8479	141.649	73.983
21321	3.848	100.584	100.291
21322	3.848	160.427	48.3656
21323	3.8481	159.122	238.89
21324	3.8481	113.473	164.756
21325	3.8481	182.858	293.285
21326	3.8484	150.198	60.4698
21327	3.8485	141.027	44.5766
21328	3.8485	144.67	43.4521
21329	3.8486	130.817	306.663
21330	3.8486	128.547	94.1356
21331	3.8486	113.334	115.126
21332	3.8488	168.405	72.6449
21333	3.8488	154.24	112.337
21334	3.8489	160.641	56.4339
21335	3.8489	164.28	86.4469
21336	3.8489	119.72	310.615
21337	3.8491	118.423	34.8537
21338	3.8495	141.39	203.693
21339	3.8496	66.3935	10.6822
21340	3.8496	116.638	22.1972
21341	3.8499	153.424	350.137
21342	3.8501	167.442	255.047
21343	3.8501	225.627	146.565
21344	3.8502	138.384	316.065
21345	3.8502	141.142	219.806
21346	3.8503	151.101	102.647
21347	3.8504	141.077	145.536
21348	3.8504	147.802	143.037
21349	3.8504	128.381	57.2622

21350	3.8505	156.651	35.1593
21351	3.8507	117.744	187.241
21352	3.8507	142.323	72.2844
21353	3.8507	102.555	27.3872
21354	3.8507	157.422	304.307
21355	3.8508	89.3635	16.3171
21356	3.8508	183.848	198.959
21357	3.8508	151.979	307.645
21358	3.8508	147.89	44.1576
21359	3.8509	143.02	305.331
21360	3.8509	136.834	352.18
21361	3.8509	116.375	92.4538
21362	3.8509	109.182	168.205
21363	3.851	175.945	34.1064
21364	3.8511	127.041	149.35
21365	3.8511	135.061	107.516
21366	3.8514	164.262	58.4332
21367	3.8514	216.015	206.819
21368	3.8515	157.71	129.595
21369	3.8515	145.467	108.383
21370	3.8516	163.605	283.531
21371	3.8519	141.221	307.121
21372	3.8519	108.315	139.832
21373	3.852	134.673	74.3512
21374	3.8521	160.691	259.298
21375	3.8521	171.294	201.119
21376	3.8521	175.807	0.1198
21377	3.8523	140.462	215.937
21378	3.8524	148.661	347.959
21379	3.8524	176.149	251.502
21380	3.8525	127.173	178.62
21381	3.8525	140.196	125.388
21382	3.8525	179.114	222.327
21383	3.8526	153.943	118.575
21384	3.8526	-2.8077	100.173
21385	3.8528	107.701	159.651
21386	3.8529	171.325	137.288
21387	3.853	174.452	110.353
21388	3.853	108.662	271.527
21389	3.8531	133.964	57.596
21390	3.8532	124.968	115.796
21391	3.8533	115.335	133.145
21392	3.8533	143.96	160.249
21393	3.8535	135.323	300.02
21394	3.8536	139.188	283.634
21395	3.8537	124.342	9.7245
21396	3.8539	144.589	86.7371
21397	3.8539	139.897	87.6605

21398	3.8539	126.019	113.795
21399	3.854	156.526	128.345
21400	3.8541	127.925	77.8516
21401	3.8541	144.293	292.342
21402	3.8543	143.182	109.489
21403	3.8544	142.383	66.6209
21404	3.8545	111.531	110.505
21405	3.8545	136.646	130.132
21406	3.8548	131.843	160.177
21407	3.8553	146.614	271.04
21408	3.8554	143.185	71.5172
21409	3.8554	139.689	155.761
21410	3.8555	153.824	147.189
21411	3.8556	148.219	55.693
21412	3.8556	138.657	127.514
21413	3.8556	51.3416	195.495
21414	3.8557	116.904	338.183
21415	3.8558	168.499	257.361
21416	3.8558	131.762	299.714
21417	3.856	146.633	103.92
21418	3.8561	147.089	280.5
21419	3.8561	165.366	123.424
21420	3.8561	140.307	243.844
21421	3.8561	114.716	75.1304
21422	3.8562	228.778	307.61
21423	3.8564	152.929	313.46
21424	3.8566	186.125	285.389
21425	3.8567	136.323	64.9421
21426	3.8568	169.246	241.618
21427	3.8568	153.38	114.593
21428	3.8568	123.749	28.978
21429	3.857	179.285	135.437
21430	3.8571	137.041	114.296
21431	3.8571	153.132	107.386
21432	3.8572	148.9	153.722
21433	3.8573	147.254	240.231
21434	3.8573	144.806	146.028
21435	3.8573	107.427	38.6968
21436	3.8574	160.05	95.6018
21437	3.8574	150.884	138.072
21438	3.8575	152.163	235.194
21439	3.8576	143.104	222.2
21440	3.8576	147.592	306.917
21441	3.8577	162.473	16.5892
21442	3.8577	144.262	65.5574
21443	3.8578	126.286	290.051
21444	3.8578	148.923	128.991
21445	3.8578	153.321	111.059

21446	3.8579	126.87	120.722
21447	3.858	68.1498	4.5756
21448	3.8582	150.259	258.975
21449	3.8583	144.748	304.628
21450	3.8584	124.958	195.99
21451	3.8584	114.391	214.902
21452	3.8586	126.636	162.454
21453	3.8587	106.028	256.003
21454	3.8588	143.594	98.9807
21455	3.8588	153.445	14.1714
21456	3.8588	190.466	306.152
21457	3.8589	125.857	191.1
21458	3.8591	144.136	27.7624
21459	3.8591	120.798	65.064
21460	3.8592	134.91	273.436
21461	3.8593	113.583	219.931
21462	3.8593	123.541	180.482
21463	3.8595	141.371	143.569
21464	3.8595	140.86	85.651
21465	3.8595	134.993	117.715
21466	3.8598	112.984	351.756
21467	3.86	160.212	70.0383
21468	3.86	152.603	134.19
21469	3.8601	163.796	101.637
21470	3.8601	134.894	75.8596
21471	3.8602	181.987	45.543
21472	3.8603	189.129	101.223
21473	3.8603	139.886	154.749
21474	3.8604	131.477	173.829
21475	3.8605	-2.7699	123.34
21476	3.8605	164.546	49.7834
21477	3.8606	140.566	35.8581
21478	3.861	164.223	192.067
21479	3.861	131.355	291.33
21480	3.861	176.521	118.678
21481	3.8611	124.858	152.218
21482	3.8612	165.115	232.029
21483	3.8613	141.248	89.5717
21484	3.8613	135.398	227.531
21485	3.8615	161.711	97.9625
21486	3.8615	142.93	63.2956
21487	3.8616	169.884	59.9126
21488	3.8617	137.033	88.6412
21489	3.8617	157.272	194.059
21490	3.8618	130.131	77.2672
21491	3.8619	127.378	157.145
21492	3.862	146.171	96.285
21493	3.8621	137.23	198.687

21494	3.8621	147.719	24.9154
21495	3.8623	141.107	229.51
21496	3.8623	-3.3077	291.384
21497	3.8624	120.204	169.101
21498	3.8625	125.039	179.377
21499	3.8629	152.547	198.596
21500	3.8629	133.343	138.618
21501	3.863	149.753	137.803
21502	3.863	89.5884	167.061
21503	3.8631	140.642	36.2015
21504	3.8632	128.033	151.709
21505	3.8632	133.3	79.178
21506	3.8632	154.107	161.28
21507	3.8633	177.155	8.1865
21508	3.8633	138.11	249.091
21509	3.8633	148.207	234.057
21510	3.8633	139.4	64.094
21511	3.8635	125.014	287.613
21512	3.8636	113.009	30.2307
21513	3.8637	138.26	142.642
21514	3.8638	129.489	258.794
21515	3.864	126.729	92.7381
21516	3.864	121.163	194.431
21517	3.864	147.211	146.876
21518	3.8641	181.126	254.046
21519	3.8641	153.476	102.508
21520	3.8641	115.772	136.623
21521	3.8642	134.631	169.597
21522	3.8644	185.281	31.4234
21523	3.8644	138.353	193.532
21524	3.8645	164.534	61.8098
21525	3.8646	122.977	121.53
21526	3.8646	146.569	179.838
21527	3.8649	172.524	350.353
21528	3.8649	120.017	106.352
21529	3.8649	100.949	144.839
21530	3.865	164.327	119.01
21531	3.8652	156.06	256.791
21532	3.8652	140.994	111.911
21533	3.8654	168.491	186.837
21534	3.8654	150.821	225.518
21535	3.8654	138.194	221.818
21536	3.8654	150.054	336.909
21537	3.8655	156.087	115.721
21538	3.8655	138.835	299.099
21539	3.8655	138.733	91.8864
21540	3.8655	100.397	348.254
21541	3.8656	131.767	102.431

21542	3.8656	138.626	265.348
21543	3.8656	153.637	88.7477
21544	3.8659	115.984	60.5573
21545	3.8659	122.395	273.861
21546	3.866	143.899	74.2742
21547	3.866	128.245	166.644
21548	3.8661	118.109	168.692
21549	3.8661	122.995	71.6197
21550	3.8662	175.584	75.0372
21551	3.8662	164.059	68.3422
21552	3.8662	95.2073	46.842
21553	3.8665	127.539	255.834
21554	3.8665	150.982	128.048
21555	3.8669	158.771	262.107
21556	3.8669	148.532	100.447
21557	3.867	164.861	197.27
21558	3.867	127.819	149.77
21559	3.8671	160.029	94.3573
21560	3.8672	147.721	120.091
21561	3.8672	121.83	191.328
21562	3.8672	172.668	102.316
21563	3.8673	148.143	178.049
21564	3.8674	171.617	322.649
21565	3.8674	179.907	56.616
21566	3.8674	164.751	240.037
21567	3.8674	140.945	38.5713
21568	3.8675	141.681	61.7727
21569	3.8675	175.284	57.2955
21570	3.8675	157.563	125.882
21571	3.8676	142.37	231.432
21572	3.8677	168.106	42.1248
21573	3.8678	133.813	143.491
21574	3.8678	-3.2119	114.993
21575	3.8679	121.011	252.785
21576	3.8679	185.355	80.3023
21577	3.868	113.355	147.65
21578	3.8683	172.987	152.457
21579	3.8684	159.004	260.234
21580	3.8686	164.8	277.64
21581	3.8686	110.221	169.756
21582	3.8687	124.591	156.153
21583	3.8687	155.004	95.1463
21584	3.8689	160.285	154.596
21585	3.869	167.65	190.748
21586	3.869	137.702	188.308
21587	3.8691	135.091	197.51
21588	3.8691	102.638	256.392
21589	3.8691	128.077	138.416

21590	3.8692	138.214	118.571
21591	3.8693	197.504	53.9084
21592	3.8694	165.724	138.175
21593	3.8695	127.352	130.738
21594	3.8696	123.017	198.744
21595	3.8696	140.257	113.931
21596	3.8697	191.676	242.87
21597	3.8698	125.778	275.619
21598	3.87	157.644	81.5855
21599	3.87	115.961	81.4219
21600	3.87	147.072	25.5508
21601	3.8701	124.519	351.172
21602	3.8702	161.001	120.005
21603	3.8702	133.479	120.251
21604	3.8702	88.3837	140.902
21605	3.8703	145.417	174.492
21606	3.8704	178.334	28.6229
21607	3.8705	133.141	100.786
21608	3.8705	143.547	180.702
21609	3.8705	46.383	264.298
21610	3.8705	98.863	155.836
21611	3.8706	124.017	113.353
21612	3.8707	120.73	165.142
21613	3.8708	154.052	254.845
21614	3.8709	111.468	61.6525
21615	3.871	126.47	116.49
21616	3.871	151.232	157.004
21617	3.871	118.853	17.3015
21618	3.8711	109.666	49.6128
21619	3.8713	143.171	58.9967
21620	3.8714	171.113	56.2316
21621	3.8714	151.718	204.056
21622	3.8715	128.045	51.4073
21623	3.8715	129.945	95.075
21624	3.8715	160.512	106.522
21625	3.8716	145.875	97.368
21626	3.8716	150.624	21.5033
21627	3.8716	146.007	147.453
21628	3.8718	125.421	195.451
21629	3.8718	177.757	79.0114
21630	3.8718	136.91	102.174
21631	3.8719	175.249	282.051
21632	3.8719	145.755	38.9055
21633	3.872	214.598	37.8743
21634	3.872	125.364	2.5461
21635	3.8721	125.256	148.325
21636	3.8721	147.271	261.982
21637	3.8722	141.127	125.082

21638	3.8723	170.13	338.984
21639	3.8723	131.493	236.015
21640	3.8724	167.717	63.1878
21641	3.8725	167.703	102.108
21642	3.8726	96.5326	169.115
21643	3.8726	172.624	326.405
21644	3.8727	149.871	128.125
21645	3.8728	141.633	109.907
21646	3.8729	154.715	88.6286
21647	3.8729	133.189	123.841
21648	3.873	171.537	307.179
21649	3.873	85.9529	0.1732
21650	3.8731	159.154	257.852
21651	3.8731	160.652	223.001
21652	3.8731	148.54	109.343
21653	3.8732	176.139	284.487
21654	3.8733	105.564	194.17
21655	3.8734	138.55	103.878
21656	3.8736	172.705	342.662
21657	3.8736	135.015	124.887
21658	3.8737	129.722	143.066
21659	3.8738	150.913	198.107
21660	3.8738	161.472	230.616
21661	3.8739	186.379	11.7663
21662	3.8739	157.372	136.204
21663	3.874	159.928	117.867
21664	3.8742	178.682	114.115
21665	3.8742	140.86	118.797
21666	3.8743	155.847	86.3412
21667	3.8743	163.448	257.259
21668	3.8743	141.936	258.349
21669	3.8745	144.767	257.007
21670	3.8746	136.331	155.946
21671	3.8746	150.394	94.441
21672	3.8747	120.629	177.181
21673	3.8747	112.244	188.706
21674	3.8747	145.588	50.4666
21675	3.8748	137.952	155.048
21676	3.8748	144.734	93.5087
21677	3.8749	155.223	109.582
21678	3.875	125.193	73.7407
21679	3.8751	176.968	214.082
21680	3.8751	139.882	159.145
21681	3.8752	68.1769	261.297
21682	3.8752	134.503	129.868
21683	3.8753	175.199	58.6839
21684	3.8753	177.704	101.486
21685	3.8753	148.285	35.2959

21686	3.8753	153.518	309.461
21687	3.8754	97.3448	30.7754
21688	3.8755	153.309	263.25
21689	3.8755	138.508	263.321
21690	3.8757	172.61	62.6313
21691	3.8757	216.621	202.214
21692	3.8759	149.585	95.2611
21693	3.876	147.124	148.834
21694	3.876	122.739	107.484
21695	3.8761	167.164	288.892
21696	3.8763	249.268	314.701
21697	3.8763	142.863	292.756
21698	3.8763	120.656	83.2103
21699	3.8764	135.135	247.733
21700	3.8764	153.104	298.126
21701	3.8767	142.058	245.376
21702	3.8769	122.883	8.1011
21703	3.877	148.771	97.6087
21704	3.8771	158.641	283.951
21705	3.8771	160.668	258.693
21706	3.8772	169.173	296.028
21707	3.8772	163.587	346.573
21708	3.8772	160.084	28.8826
21709	3.8772	164.088	312.02
21710	3.8772	154.513	147.973
21711	3.8772	194.907	231.331
21712	3.8773	88.5236	26.7797
21713	3.8773	166.604	51.0031
21714	3.8773	127.036	177.222
21715	3.8774	114.848	187.358
21716	3.8776	175.53	140.675
21717	3.8777	141.654	127.833
21718	3.8778	128.974	94.2392
21719	3.8778	120.939	78.7688
21720	3.878	134.56	165.961
21721	3.878	127.94	168.992
21722	3.8782	149.954	22.4794
21723	3.8786	191.778	91.1992
21724	3.8786	169.241	67.2942
21725	3.8786	-2.8087	132.255
21726	3.8787	181.858	61.8255
21727	3.8787	155.568	260.196
21728	3.8788	169.037	110.019
21729	3.8788	156.989	133.279
21730	3.8789	116.185	252.048
21731	3.879	182.261	108.874
21732	3.879	172.572	330.924
21733	3.8792	140.116	113.93

21734	3.8793	130.639	132.941
21735	3.8794	139.138	306.578
21736	3.8794	135.039	156.361
21737	3.8796	168.322	23.71
21738	3.8796	116.33	197.852
21739	3.8797	194.524	63.3553
21740	3.88	128.641	95.6971
21741	3.8801	147.247	56.1536
21742	3.8802	170.577	37.3175
21743	3.8803	162.188	59.191
21744	3.8804	165.49	263.922
21745	3.8805	150.948	114.725
21746	3.8805	138.423	130.038
21747	3.8805	149.645	167.177
21748	3.8806	159.754	99.3127
21749	3.8806	146.195	232.936
21750	3.8806	172.747	141.973
21751	3.8808	155.132	109.358
21752	3.8809	161.646	44.7688
21753	3.8809	139.961	87.7432
21754	3.8813	138.107	99.3772
21755	3.8813	153.335	131.043
21756	3.8814	167.662	71.4882
21757	3.8814	143.14	274.033
21758	3.8814	101.435	186.448
21759	3.8815	148.036	119.063
21760	3.8816	106.898	40.3746
21761	3.8818	160.721	111.318
21762	3.8818	158.133	88.5034
21763	3.8818	132.93	113.709
21764	3.8818	176.51	67.0631
21765	3.8819	164.723	75.8452
21766	3.882	172.924	82.1382
21767	3.8821	135.354	148.936
21768	3.8821	29.6901	86.839
21769	3.8821	128.07	142.116
21770	3.8823	130.809	73.863
21771	3.8823	161.222	86.0459
21772	3.8823	163.408	284.981
21773	3.8824	131.177	102.38
21774	3.8825	138.582	92.8212
21775	3.8826	194.825	80.5657
21776	3.8827	136.825	199.228
21777	3.8834	182.032	29.2134
21778	3.8834	140.949	141.358
21779	3.8835	123.18	195.61
21780	3.8836	148.795	102.393
21781	3.8838	165.78	108.231

21782	3.8839	137.949	239.508
21783	3.884	145.399	129.234
21784	3.8841	114.102	182.726
21785	3.8842	149.761	184.245
21786	3.8842	106.946	325.326
21787	3.8842	152.481	179.292
21788	3.8843	146.968	82.2666
21789	3.8844	173.081	344.878
21790	3.8846	150.165	317.244
21791	3.8848	147.741	128.44
21792	3.8851	149.886	261.627
21793	3.8854	173.389	114.655
21794	3.8855	179.837	33.2349
21795	3.8856	144.532	194.098
21796	3.8856	147.343	62.1088
21797	3.8856	125.052	129.228
21798	3.8856	172.016	127.748
21799	3.8858	135.628	139.279
21800	3.8858	168.864	313.252
21801	3.8859	178.875	138.915
21802	3.886	145.278	146.565
21803	3.886	125.198	193.155
21804	3.886	158.548	86.1399
21805	3.886	131.709	55.2621
21806	3.8861	168.434	299.053
21807	3.8862	144.316	356.911
21808	3.8863	154.808	140.047
21809	3.8865	127.117	200.608
21810	3.8865	138.978	164.527
21811	3.8866	140.798	263.816
21812	3.8866	166.694	166.833
21813	3.8867	150.294	74.7314
21814	3.8868	177.689	145.742
21815	3.8868	116.394	167.883
21816	3.8869	165.231	205.189
21817	3.8869	135.901	253.416
21818	3.8869	137.021	154.166
21819	3.8869	128.902	305.628
21820	3.887	131.513	88.5964
21821	3.8872	134.916	43.6845
21822	3.8873	158.741	73.1354
21823	3.8874	165.408	30.535
21824	3.8874	150.678	299.063
21825	3.8875	145.81	282.874
21826	3.8876	148.601	68.7078
21827	3.8876	123.09	136.198
21828	3.8877	181.154	149.125
21829	3.8877	142.007	166.09

21830	3.8877	128.832	191.134
21831	3.8877	146.678	96.2233
21832	3.8878	141.714	10.5895
21833	3.8879	144.018	67.0986
21834	3.8882	162.609	177.208
21835	3.8882	168.487	346.727
21836	3.8883	158.254	58.9175
21837	3.8884	139.883	174.26
21838	3.8884	-2.7373	31.7955
21839	3.8885	141.452	60.0291
21840	3.8885	161.823	110.342
21841	3.8886	107.903	176.565
21842	3.8886	90.8318	153.073
21843	3.8887	174.571	143.396
21844	3.8888	183.391	70.3131
21845	3.8888	161.63	38.4115
21846	3.8888	151.836	73.0488
21847	3.8889	162.851	238.866
21848	3.889	128.484	116.848
21849	3.889	98.3644	332.607
21850	3.889	145.346	127.752
21851	3.8892	153.993	222.832
21852	3.8892	141.117	238.275
21853	3.8893	150.621	217.316
21854	3.8895	147.559	97.9123
21855	3.8897	140.274	173.781
21856	3.8898	148.524	99.39
21857	3.8898	188.594	154.269
21858	3.89	171.365	83.5999
21859	3.8901	149.302	298.261
21860	3.8902	157.776	350.102
21861	3.8902	144.869	39.0261
21862	3.8902	147.558	164.767
21863	3.8903	183.6	199.681
21864	3.8903	146.037	93.7693
21865	3.8903	149.545	263.237
21866	3.8903	149.84	107.078
21867	3.8904	172.776	86.526
21868	3.8904	134.611	74.2128
21869	3.8904	119.909	104.242
21870	3.8905	143.178	65.2817
21871	3.8908	118.694	217.426
21872	3.8909	171.368	109.958
21873	3.8909	145.339	45.5365
21874	3.8912	87.7722	154.76
21875	3.8912	177.853	150.566
21876	3.8915	120.733	197.436
21877	3.8915	130.534	117.582

21878	3.8916	143	63.8635
21879	3.8917	40.6331	189.08
21880	3.8918	206.885	89.5961
21881	3.8918	151.843	49.0289
21882	3.8918	148.022	74.4827
21883	3.8919	148.803	274.223
21884	3.8919	125.647	140.263
21885	3.892	160.586	83.9591
21886	3.8922	182.841	77.7052
21887	3.8923	148.398	192.896
21888	3.8923	146.314	70.6471
21889	3.8923	132.158	270.713
21890	3.8923	204.685	289.965
21891	3.8924	158.279	32.3915
21892	3.8925	111.305	118.798
21893	3.8926	196.445	151.342
21894	3.8927	126.646	192.664
21895	3.8927	171.807	11.0576
21896	3.8927	159.521	23.0308
21897	3.8928	139.799	169.951
21898	3.8928	161.036	175.054
21899	3.893	125.207	160.782
21900	3.8931	151.74	137.822
21901	3.8933	133.215	14.5478
21902	3.8935	167.313	71.7298
21903	3.8937	87.2135	281.857
21904	3.8938	143.753	274.455
21905	3.8938	147.524	51.3743
21906	3.894	175.525	53.2423
21907	3.894	141.156	80.1201
21908	3.894	134.116	118.046
21909	3.8941	169.714	282.632
21910	3.8942	118.333	310.373
21911	3.8942	134.275	134.76
21912	3.8942	155.822	116.193
21913	3.8943	155.985	263.54
21914	3.8944	183.454	243.05
21915	3.8944	142.966	213.116
21916	3.8945	60.5226	6.2074
21917	3.8945	144.192	91.513
21918	3.8945	142.623	308.947
21919	3.8946	171.778	27.3965
21920	3.8947	155.982	96.1165
21921	3.8949	143.561	241.337
21922	3.8949	167.602	217.002
21923	3.8949	135.192	129.329
21924	3.895	132.316	296.349
21925	3.8952	153.643	49.2487

21926	3.8952	160.534	320.177
21927	3.8952	150.948	115.249
21928	3.8953	72.3414	9.0996
21929	3.8953	202.63	21.806
21930	3.8955	197.667	71.8219
21931	3.8955	193.644	55.9949
21932	3.8956	154.084	187.065
21933	3.8956	146.336	73.6453
21934	3.8956	142.179	275.737
21935	3.8957	138.044	145.142
21936	3.8958	132.679	51.4195
21937	3.8958	140.013	155.041
21938	3.8958	168.205	233.318
21939	3.8959	164.38	94.8712
21940	3.896	150.53	248.413
21941	3.8962	158.135	18.1955
21942	3.8962	134.32	248.347
21943	3.8962	163.436	85.4957
21944	3.8962	142.878	147.035
21945	3.8965	115.709	19.4972
21946	3.8965	130.86	243.206
21947	3.8966	158.086	236.362
21948	3.8967	143.34	108.588
21949	3.8967	129.972	74.0804
21950	3.8968	122.693	146.819
21951	3.8968	151.148	127.857
21952	3.8968	160.611	67.6617
21953	3.897	153.763	116.546
21954	3.897	128.617	148.426
21955	3.8971	159.643	36.9457
21956	3.8972	131.711	74.6855
21957	3.8974	142.831	134.913
21958	3.8974	135.388	164.782
21959	3.8974	133.073	256.508
21960	3.8974	211.165	319.873
21961	3.8974	173.817	193.506
21962	3.8975	138.027	97.8318
21963	3.8977	181.59	273.406
21964	3.8977	167.62	177.589
21965	3.8978	147.632	53.2635
21966	3.8979	177.009	68.4014
21967	3.8979	153.674	213.406
21968	3.8979	162.908	103.13
21969	3.898	193.155	84.1314
21970	3.8981	205.92	99.6101
21971	3.8981	144.425	13.1368
21972	3.8981	157.418	219.992
21973	3.8983	132.915	296.892

21974	3.8983	117.713	197.697
21975	3.8983	128.936	88.6524
21976	3.8983	137.382	63.9164
21977	3.8985	135.095	156.832
21978	3.8985	138.375	161.577
21979	3.8986	127.351	183.495
21980	3.8988	113.404	310.432
21981	3.8988	155.724	52.861
21982	3.8988	156.284	94.4392
21983	3.899	-2.7452	0.92
21984	3.8991	165.179	84.1078
21985	3.8992	198.689	97.1874
21986	3.8992	133.169	82.4029
21987	3.8994	119.524	68.8387
21988	3.8994	170.756	44.2211
21989	3.8995	140.98	128.31
21990	3.8995	123.158	154.619
21991	3.8995	154.62	123.348
21992	3.8995	177.865	129.797
21993	3.8997	143.813	134.316
21994	3.8998	141.783	277.126
21995	3.8999	120.408	176.675
21996	3.9	184.574	88.3131
21997	3.9002	79.1839	329.639
21998	3.9003	115.493	118.145
21999	3.9003	134.921	44.431
22000	3.9004	157.958	269.52
22001	3.9006	155.507	267.328
22002	3.9007	161.282	301.435
22003	3.9008	128.772	250.753
22004	3.9009	183.263	37.8695
22005	3.9012	161.534	132.755
22006	3.9012	152.719	285.752
22007	3.9012	-2.8311	144.404
22008	3.9013	150.68	96.7418
22009	3.9014	140.09	256.405
22010	3.9014	39.8163	109.385
22011	3.9016	114.32	259.581
22012	3.9016	141.765	294.706
22013	3.9016	145.578	73.5316
22014	3.9017	125.999	164.251
22015	3.9017	151.928	98.1567
22016	3.9018	138.258	224.702
22017	3.9018	115.715	14.7874
22018	3.9018	127.944	176.563
22019	3.902	131.394	134.263
22020	3.9022	81.4746	68.7536
22021	3.9022	106.661	296.834

22022	3.9023	157.498	115.17
22023	3.9024	145.642	64.4713
22024	3.9024	108.004	16.2753
22025	3.9024	174.572	31.8519
22026	3.9025	158.831	88.0713
22027	3.9026	165.185	230.849
22028	3.9026	139.168	279.245
22029	3.9026	127.597	262.491
22030	3.9027	136.554	164.706
22031	3.9028	140.306	147.491
22032	3.9029	65.7947	102.824
22033	3.9029	155.524	106.182
22034	3.903	146.033	114.253
22035	3.9033	156.535	195.009
22036	3.9034	138.779	92.3527
22037	3.9035	158.743	101.468
22038	3.9036	170.134	132.526
22039	3.9037	115.525	304.479
22040	3.9037	165.875	37.2652
22041	3.9037	143.81	253.626
22042	3.9038	147.312	295.743
22043	3.9038	-4.0606	286.201
22044	3.904	172.496	87.4048
22045	3.904	155.281	176.82
22046	3.904	135.758	113.548
22047	3.904	166.6	160.399
22048	3.904	-2.6921	10.447
22049	3.904	165.217	104.837
22050	3.9041	230.858	28.8145
22051	3.9041	158.688	233.041
22052	3.9041	170.906	77.9387
22053	3.9041	138.341	24.5501
22054	3.9042	154.367	158.98
22055	3.9042	153.828	271.855
22056	3.9043	150.759	57.8602
22057	3.9044	129.29	262.263
22058	3.9044	144.682	44.1253
22059	3.9045	169.314	29.673
22060	3.9045	166.614	305.641
22061	3.9045	118.997	160.149
22062	3.9046	179.347	116.381
22063	3.9049	94.2609	167.435
22064	3.9049	175.116	122.305
22065	3.9049	185.377	18.9192
22066	3.9051	142.216	119.855
22067	3.9051	225.559	213.259
22068	3.9052	111.203	219.883
22069	3.9053	100.117	125.116

22070	3.9053	145.982	68.8947
22071	3.9053	161.766	106.978
22072	3.9053	167.276	121.632
22073	3.9056	202.216	307.946
22074	3.9056	188.867	26.609
22075	3.9056	124.72	186.658
22076	3.9057	141.021	192.326
22077	3.9059	133.854	287.89
22078	3.9059	143.395	179.054
22079	3.9061	155.365	197.531
22080	3.9062	153.006	137.138
22081	3.9063	180.021	76.5832
22082	3.9063	159.214	279.502
22083	3.9063	142.12	52.0397
22084	3.9066	201.133	20.4773
22085	3.9066	144.677	161.493
22086	3.9067	162.788	103.31
22087	3.9067	143.768	174.381
22088	3.907	186.435	357.847
22089	3.907	172.577	25.7879
22090	3.907	117.303	351.346
22091	3.9071	189.264	48.057
22092	3.9072	74.3477	198.056
22093	3.9072	177.363	133.959
22094	3.9073	170.542	222.421
22095	3.9074	186.607	134.176
22096	3.9076	176.422	295.182
22097	3.9077	189.499	203.226
22098	3.9078	173.218	48.0446
22099	3.9079	108.868	213.899
22100	3.9079	138.938	249.357
22101	3.9079	132.131	146.238
22102	3.9079	138.465	312.106
22103	3.9081	123.654	166.575
22104	3.9081	123.67	114.451
22105	3.9083	151.283	283.099
22106	3.9084	157.151	293.098
22107	3.9084	148.696	306.072
22108	3.9086	179.471	304.586
22109	3.9089	138.6	140.174
22110	3.9089	152.433	109.257
22111	3.9089	142.383	270.504
22112	3.9091	178.407	126.721
22113	3.9091	170.376	60.317
22114	3.9092	162.113	165.29
22115	3.9092	140.998	108.736
22116	3.9092	171.54	170.278
22117	3.9093	136.717	180.065

22118	3.9093	149.546	47.0105
22119	3.9094	123.326	35.6319
22120	3.9095	154.636	127.135
22121	3.9096	110.321	108.053
22122	3.9097	149.465	337.324
22123	3.9098	161.165	271.507
22124	3.9098	149.418	84.3692
22125	3.91	134.754	182.36
22126	3.9103	157.675	221.292
22127	3.9104	114.202	332.489
22128	3.9104	168.038	114.615
22129	3.9104	143.873	278.445
22130	3.9104	154.718	31.3638
22131	3.9106	182.37	111.726
22132	3.9106	187.976	290.41
22133	3.9108	111.82	324.18
22134	3.9109	189.822	305.214
22135	3.9109	124.24	218.341
22136	3.9109	155.067	157.887
22137	3.911	141.988	12.8563
22138	3.9111	170.52	324.482
22139	3.9112	185.507	37.4746
22140	3.9114	198.266	88.4392
22141	3.9115	144.632	289.137
22142	3.9116	191.377	45.516
22143	3.9116	142.741	295.258
22144	3.9117	130.927	230.44
22145	3.9117	150.948	216.146
22146	3.9118	130.365	219.895
22147	3.9118	173.234	278.628
22148	3.9119	148.061	145.197
22149	3.912	-2.7993	107.013
22150	3.9121	133.867	56.6428
22151	3.9122	190.297	66.8495
22152	3.9123	168.358	203.797
22153	3.9124	140.1	347.506
22154	3.9124	117.697	343.482
22155	3.9125	142.728	104.866
22156	3.9126	166.217	288.607
22157	3.9126	171.709	343.446
22158	3.9127	-3.5334	261.449
22159	3.9128	156.211	155.747
22160	3.9128	190.233	43.9205
22161	3.9129	155.163	70.2528
22162	3.9132	150.26	326.613
22163	3.9133	182.456	60.3795
22164	3.9134	188.562	25.519
22165	3.9134	163.631	69.0309

22166	3.9135	153.818	4.0579
22167	3.9136	159.164	252.192
22168	3.9136	140.699	182.969
22169	3.9136	144.61	136.41
22170	3.9137	152.107	22.2606
22171	3.9137	169.028	80.9217
22172	3.9139	196.74	96.3097
22173	3.9139	142.477	190.731
22174	3.914	138.656	103.64
22175	3.9142	193.666	7.4687
22176	3.9142	205.64	84.3483
22177	3.9143	144.645	162.886
22178	3.9144	165.983	41.5453
22179	3.9144	-2.7311	9.7919
22180	3.9145	146.31	251.43
22181	3.9145	147.771	91.2803
22182	3.9146	158.259	132.561
22183	3.9148	152.851	55.8709
22184	3.9149	135.874	139.223
22185	3.915	188.664	317.197
22186	3.9152	158.968	230.668
22187	3.9153	137.46	267.644
22188	3.9154	125.748	94.6061
22189	3.9154	132.615	351.971
22190	3.9155	185.997	55.127
22191	3.9155	137.367	76.0439
22192	3.9156	131.539	100.334
22193	3.9157	120.374	251.15
22194	3.9157	112.406	82.3404
22195	3.9159	145.008	0.9789
22196	3.916	140.113	164.064
22197	3.9161	144.372	175.636
22198	3.9162	185.836	275.768
22199	3.9162	182.497	44.9721
22200	3.9163	118.064	138.259
22201	3.9163	118.773	37.5077
22202	3.9163	151.765	164.678
22203	3.9164	100.543	39.9434
22204	3.9164	-4.0666	199.564
22205	3.9167	150.797	202.351
22206	3.9168	167.428	307.326
22207	3.9168	157.358	273.87
22208	3.9168	99.6982	14.3875
22209	3.9169	184.886	48.6628
22210	3.9171	149.68	146.233
22211	3.9171	140.398	136.542
22212	3.9171	155.458	34.1281
22213	3.9172	125.61	112.502

22214	3.9172	162.351	44.9383
22215	3.9172	185.009	22.577
22216	3.9174	127.952	126.974
22217	3.9175	127.559	139.052
22218	3.9176	174.098	98.4963
22219	3.9176	164.765	76.822
22220	3.9176	145.65	142.994
22221	3.9177	171.664	80.0527
22222	3.9179	172.952	59.4241
22223	3.9179	149.604	199.815
22224	3.918	74.3563	44.6097
22225	3.9182	177.389	138.942
22226	3.9182	159.777	95.5118
22227	3.9183	194.763	256.821
22228	3.9183	144.53	136.055
22229	3.9183	140.162	158.981
22230	3.9184	126.05	60.2416
22231	3.9185	147.176	181.173
22232	3.9185	149.255	119.115
22233	3.9186	147.97	149.151
22234	3.9187	187.242	114.576
22235	3.9187	151.632	21.9313
22236	3.9188	199.844	256.703
22237	3.9188	142.742	167.125
22238	3.9188	145.971	93.2461
22239	3.9189	144.706	57.0476
22240	3.9189	154.228	163.46
22241	3.919	123.735	215.831
22242	3.919	109.628	210.82
22243	3.919	164.082	65.5218
22244	3.919	161.486	338.965
22245	3.9191	143.448	202.419
22246	3.9192	130.989	126.168
22247	3.9192	141.468	198.266
22248	3.9192	125.686	170.406
22249	3.9193	132.226	112.971
22250	3.9194	129.227	134.169
22251	3.9194	162.041	218.389
22252	3.9195	170.351	81.701
22253	3.9195	155.705	160.727
22254	3.9197	190.351	94.9845
22255	3.9197	169.413	96.149
22256	3.9197	144.949	170.26
22257	3.9197	110.024	98.4987
22258	3.9197	184.521	22.2311
22259	3.9198	124.445	145.295
22260	3.92	181.385	72.3471
22261	3.92	183.369	168.325

22262	3.92	161.498	114.967
22263	3.9201	164.844	195.776
22264	3.9201	149.185	112.422
22265	3.9204	172.607	101.071
22266	3.9206	106.238	141.442
22267	3.9206	132.673	258.35
22268	3.9206	119.71	138.209
22269	3.9207	180.386	77.1864
22270	3.9207	112.635	139.114
22271	3.9209	157.268	302.518
22272	3.9209	142.345	217.527
22273	3.921	130.981	102.431
22274	3.9211	133.997	323.318
22275	3.9211	149.557	118.311
22276	3.9211	151.252	79.7971
22277	3.9211	132.877	304.599
22278	3.9213	125.054	303.251
22279	3.9214	198.936	56.2861
22280	3.9215	169.381	299.463
22281	3.9216	147.971	266.589
22282	3.9217	166.502	153.714
22283	3.9218	125.039	154.262
22284	3.9218	138.945	258.135
22285	3.9219	144.041	152.025
22286	3.9221	200.802	314.402
22287	3.9221	156.35	199.995
22288	3.9224	190.599	70.3749
22289	3.9225	145.672	116.592
22290	3.9225	166.906	91.1769
22291	3.9226	185.272	91.8986
22292	3.9226	163.722	134.435
22293	3.9227	189.822	17.9957
22294	3.9227	198.285	132.914
22295	3.9227	138.008	138.977
22296	3.9228	194.056	88.1244
22297	3.9228	145.338	264.593
22298	3.9228	137.978	98.2921
22299	3.923	143.152	105.379
22300	3.9232	163.172	109.884
22301	3.9234	124.919	144.117
22302	3.9234	160.624	104.084
22303	3.9235	150.451	224.915
22304	3.9235	180.097	120.511
22305	3.9236	155.089	157.633
22306	3.9239	135.979	213.901
22307	3.9241	192.192	45.2715
22308	3.9244	32.632	259.461
22309	3.9244	143.659	176.72

22310	3.9245	154.858	79.8942
22311	3.9246	170.26	346.645
22312	3.9246	126.07	281.142
22313	3.9246	169.34	76.8694
22314	3.9248	133.523	52.41
22315	3.9248	159.377	118.674
22316	3.9249	189.19	40.0226
22317	3.925	145.404	246.109
22318	3.925	111.134	138.833
22319	3.925	136.566	109.904
22320	3.9251	144.821	100.371
22321	3.9251	165.076	268.09
22322	3.9254	187.666	37.602
22323	3.9255	164.418	238.511
22324	3.9256	167.022	294.691
22325	3.9259	179.667	97.1617
22326	3.926	152.811	150.218
22327	3.9261	-4.0927	154.783
22328	3.9262	148.804	183.676
22329	3.9263	118.716	133.508
22330	3.9264	166.296	38.5036
22331	3.9264	156.921	224.932
22332	3.9265	180.558	14.2897
22333	3.9265	167.182	5.1737
22334	3.9265	169.794	41.641
22335	3.9267	120.91	242.858
22336	3.9269	164.711	340.007
22337	3.9269	128.049	150.096
22338	3.9269	166.41	111.132
22339	3.9269	197.076	263.005
22340	3.927	131.372	140.813
22341	3.9272	189.019	247.859
22342	3.9273	140.947	343.088
22343	3.9274	151.237	260.599
22344	3.9274	171.771	105.99
22345	3.9276	144.169	331.427
22346	3.9276	162.215	259.662
22347	3.9277	138.116	195.668
22348	3.9278	160.528	73.5817
22349	3.928	111.913	318.814
22350	3.928	174.542	349.442
22351	3.9281	172.03	237.03
22352	3.9281	152.07	75.4217
22353	3.9281	145.216	153.323
22354	3.9281	175.391	31.2414
22355	3.9282	153.698	274.516
22356	3.9282	136.296	193.398
22357	3.9283	87.2626	29.3596

22358	3.9285	151.603	218.124
22359	3.9285	120.428	17.489
22360	3.9285	164.642	137.702
22361	3.9286	133.279	119.759
22362	3.9286	143.884	187.322
22363	3.9287	194.493	77.8233
22364	3.9287	168.389	266.286
22365	3.9287	151.689	95.9856
22366	3.9288	146.286	346.636
22367	3.9288	164.915	66.009
22368	3.929	126.198	318.296
22369	3.929	128.323	167.77
22370	3.9291	171.426	33.6678
22371	3.9291	177.091	106.319
22372	3.9292	189.29	1.0455
22373	3.9292	191.249	29.849
22374	3.9292	154.035	141.461
22375	3.9292	155.319	90.8267
22376	3.9293	180.462	154.518
22377	3.9293	171.224	81.9447
22378	3.9293	154.723	304.967
22379	3.9293	150.644	41.3704
22380	3.9294	180.714	73.2708
22381	3.9294	161.751	249.834
22382	3.9294	175.004	106.227
22383	3.9294	119.809	55.8691
22384	3.9295	126.978	63.6334
22385	3.9296	195.994	71.3247
22386	3.9297	148.09	37.1894
22387	3.9297	111.807	100.176
22388	3.9297	107.825	329.323
22389	3.9298	140.288	265.462
22390	3.9298	142.256	11.6987
22391	3.9298	118.01	218.938
22392	3.9299	181.052	312.139
22393	3.9299	139.047	311.007
22394	3.9299	157.69	257.731
22395	3.9301	137.222	10.7952
22396	3.9301	128.195	116.718
22397	3.9302	197.978	7.1842
22398	3.9302	144.856	119.384
22399	3.9302	1.0579	123.238
22400	3.9303	108.682	58.346
22401	3.9304	117.124	320.5
22402	3.9304	156.886	51.3583
22403	3.9304	178.514	39.0499
22404	3.9305	150.73	157.389
22405	3.9306	143.509	197.005

22406	3.9306	140.189	204.523
22407	3.9307	156.541	44.1465
22408	3.9308	184.234	33.2569
22409	3.9309	125.007	11.7727
22410	3.931	-0.347	66.8694
22411	3.931	143.254	218.325
22412	3.9311	177.815	140.617
22413	3.9311	179.489	53.6112
22414	3.9311	147.102	84.202
22415	3.9313	239.498	118.73
22416	3.9313	174.675	48.4606
22417	3.9313	154.658	66.0668
22418	3.9314	153.166	154.617
22419	3.9315	155.03	284.112
22420	3.9316	132.581	135.029
22421	3.9317	184.171	157.281
22422	3.9318	124.524	106.18
22423	3.9319	159.655	241.28
22424	3.932	173.711	13.4842
22425	3.932	194.448	82.7327
22426	3.932	156.168	133.85
22427	3.9321	169.307	85.0513
22428	3.9322	166.933	34.8928
22429	3.9324	144.847	87.4062
22430	3.9327	-4.1106	215.726
22431	3.9328	180.084	313.657
22432	3.933	141.608	95.4216
22433	3.9331	169.291	229.614
22434	3.9332	143.974	96.9163
22435	3.9332	166.729	144.478
22436	3.9333	162.142	270.925
22437	3.9335	148.111	198.281
22438	3.9336	165.531	64.4492
22439	3.9336	146.991	96.3687
22440	3.9337	159.036	269.069
22441	3.9339	142.632	85.7593
22442	3.934	159.414	124.183
22443	3.9341	161.088	289.433
22444	3.9341	155.392	245.103
22445	3.9341	172.807	102.527
22446	3.9341	199.635	350.89
22447	3.9342	140.027	144.266
22448	3.9342	154.425	217.912
22449	3.9344	166.682	38.5664
22450	3.9344	153.013	126.304
22451	3.9344	163.405	304.905
22452	3.9348	159.921	157.479
22453	3.9348	154.027	219.829

22454	3.9348	146.966	290.157
22455	3.9349	108.363	152.128
22456	3.9351	157.445	77.2585
22457	3.9353	133.933	268.313
22458	3.9354	172.745	261.795
22459	3.9354	131.475	159.654
22460	3.9357	143.016	133.299
22461	3.9359	192.126	205.344
22462	3.936	151.817	153.479
22463	3.936	159.513	133.599
22464	3.936	-3.889	247.668
22465	3.9361	224.082	22.1683
22466	3.9362	145.908	109.249
22467	3.9362	96.9752	200.645
22468	3.9362	147.047	62.8932
22469	3.9363	165.537	240.11
22470	3.9364	163.562	290.405
22471	3.9364	159.647	98.3196
22472	3.9365	194.22	24.1257
22473	3.9365	183.152	30.4793
22474	3.9366	149.023	216.923
22475	3.9367	149.217	109.368
22476	3.9367	153.195	75.3336
22477	3.9367	159.995	269.428
22478	3.9368	133.475	110.445
22479	3.9369	161.498	296.349
22480	3.9369	168.023	158.565
22481	3.9369	140.607	196.136
22482	3.937	149.628	41.9714
22483	3.937	135.622	84.0693
22484	3.937	157.705	136.336
22485	3.9371	165.851	46.6546
22486	3.9371	126.178	315.463
22487	3.9374	135.702	220.572
22488	3.9374	239.574	318.419
22489	3.9374	169.449	339.334
22490	3.9374	153.626	201.567
22491	3.9375	145.367	282.776
22492	3.9376	92.6021	151.132
22493	3.9377	143.551	37.7133
22494	3.9378	-2.7284	355.115
22495	3.9378	137.927	125.056
22496	3.9379	161.987	306.239
22497	3.9379	155.21	155.814
22498	3.938	130.286	233.332
22499	3.938	120.432	145.78
22500	3.938	167.987	310.849
22501	3.9381	122.237	137.742

22502	3.9382	164.158	127.134
22503	3.9382	147.983	124.505
22504	3.9382	205.534	74.3978
22505	3.9384	169.87	141.609
22506	3.9384	183.572	119.675
22507	3.9385	124.113	253.396
22508	3.9385	173.393	312.097
22509	3.9386	145.256	15.4139
22510	3.9387	167.376	228.376
22511	3.9388	185.35	250.363
22512	3.9388	118.06	125.607
22513	3.9391	184.498	346.051
22514	3.9391	119.14	124.011
22515	3.9392	196.65	44.5333
22516	3.9393	188.645	71.7869
22517	3.9393	146.611	320.895
22518	3.9395	115.46	91.4774
22519	3.9395	183.876	220.743
22520	3.9396	181.491	280.901
22521	3.9396	177.73	86.2544
22522	3.9396	146.985	169.216
22523	3.9397	207.088	11.7429
22524	3.9397	123.948	166.192
22525	3.9399	188.071	58.583
22526	3.9399	126.939	75.601
22527	3.9399	178.128	25.7641
22528	3.94	131.579	117.373
22529	3.9401	169.897	69.662
22530	3.9401	227.562	207.623
22531	3.9402	119.032	123.433
22532	3.9402	179.321	322.189
22533	3.9404	159.82	87.8523
22534	3.9404	203.452	82.8824
22535	3.9405	146.208	285.466
22536	3.9409	120.031	101.957
22537	3.9411	169.267	76.6003
22538	3.9412	116.964	84.9606
22539	3.9412	97.0505	75.9347
22540	3.9412	56.9484	54.197
22541	3.9413	225.551	50.6322
22542	3.9413	171.922	125.512
22543	3.9413	189.693	316.741
22544	3.9414	125.564	179.886
22545	3.9415	192.268	45.3019
22546	3.9415	152.881	301.87
22547	3.9415	149.173	140.126
22548	3.9416	182.841	97.8546
22549	3.9416	196.358	88.5252

22550	3.9418	150.22	10.3623
22551	3.942	177.141	146.671
22552	3.942	186.221	289.818
22553	3.9424	197.239	79.3427
22554	3.9424	115.119	206.579
22555	3.9425	189.197	55.8168
22556	3.9425	145.925	98.9175
22557	3.9426	155.941	125.019
22558	3.9426	156.775	239.453
22559	3.9429	143.602	69.4121
22560	3.943	169.079	5.1001
22561	3.943	157.051	311.929
22562	3.9432	70.5616	261.922
22563	3.9432	182.23	5.3429
22564	3.9433	176.866	106.406
22565	3.9433	158.444	126.477
22566	3.9434	213.6	44.1653
22567	3.9434	122.296	218.504
22568	3.9434	140.913	267.69
22569	3.9434	151.589	118.28
22570	3.9434	147.282	261.995
22571	3.9435	185.177	145.104
22572	3.9436	137.644	80.5303
22573	3.9438	171.124	253.545
22574	3.9439	118.981	197.814
22575	3.9439	145.233	141.934
22576	3.9445	211.22	53.7072
22577	3.9446	134.88	134.355
22578	3.9447	183.934	98.9531
22579	3.9447	194.121	178.7
22580	3.9449	101.521	16.521
22581	3.945	188.718	176.736
22582	3.945	187.388	307.478
22583	3.945	140.403	133.714
22584	3.9452	80.0875	322.351
22585	3.9453	145.957	121.819
22586	3.9455	151.334	41.3089
22587	3.9456	161.781	225.039
22588	3.9457	169.317	236.013
22589	3.9457	174.874	311.872
22590	3.9458	189.837	61.5857
22591	3.9458	106.692	162.083
22592	3.9459	141.789	67.0334
22593	3.9459	156.054	156.194
22594	3.946	169.603	153.793
22595	3.946	136.59	213.242
22596	3.946	-2.7296	5.0722
22597	3.9462	171.469	331.014

22598	3.9462	201	156.683
22599	3.9465	229.753	152.679
22600	3.9466	145.348	223.58
22601	3.9467	181.07	153.296
22602	3.9467	182.813	118.204
22603	3.9468	148.969	105.289
22604	3.9469	152.385	96.5129
22605	3.9469	125.158	137.952
22606	3.947	137.043	183.878
22607	3.9471	157.205	265.593
22608	3.9472	162.215	151.318
22609	3.9474	183.173	178.966
22610	3.9475	159.108	233.984
22611	3.9475	121.766	169.841
22612	3.9476	147.444	73.8831
22613	3.9478	-2.7911	165.42
22614	3.9478	119.857	190.196
22615	3.9479	160.115	293.146
22616	3.9479	150.876	182.557
22617	3.948	163.396	86.7504
22618	3.9481	170.164	264.798
22619	3.9481	142.748	175.078
22620	3.9483	163.396	139.32
22621	3.9484	152.325	291.307
22622	3.9485	197.343	65.5359
22623	3.9486	128.382	104.854
22624	3.9486	134.276	81.5565
22625	3.9487	168.346	69.8956
22626	3.9487	161.076	312.962
22627	3.9488	173.606	8.8599
22628	3.9489	181.715	150.182
22629	3.9489	141.855	59.4141
22630	3.949	16.8214	65.7911
22631	3.949	172.851	112.86
22632	3.9491	178.107	71.3824
22633	3.9494	138.355	65.5722
22634	3.9494	119.03	153.019
22635	3.9495	194.038	50.0495
22636	3.9495	159.272	160.496
22637	3.9495	158.591	217.66
22638	3.9497	185.448	126.512
22639	3.9499	179.231	30.7254
22640	3.9499	168.593	150.275
22641	3.95	142.198	129.789
22642	3.9501	144.939	300.006
22643	3.9501	160.473	226.462
22644	3.9503	161.229	45.2466
22645	3.9504	160.901	254.549

22646	3.9504	168.554	115.283
22647	3.9504	179.302	297.33
22648	3.9505	111.739	30.3413
22649	3.9506	164.074	56.6609
22650	3.9506	-2.7666	108.549
22651	3.9507	113.389	4.4223
22652	3.9507	190.641	56.5641
22653	3.9508	218.664	355.3
22654	3.9509	169.148	268.084
22655	3.951	187.345	144.876
22656	3.9511	135.144	202.406
22657	3.9512	184.42	127.558
22658	3.9513	184.74	67.5165
22659	3.9513	138.946	186.029
22660	3.9514	130.62	295.516
22661	3.9515	172.441	261.495
22662	3.9515	157.59	131.695
22663	3.9516	160.078	229.095
22664	3.9518	144.16	73.2359
22665	3.952	160.71	163.376
22666	3.9522	129.574	225.982
22667	3.9523	172.594	122.952
22668	3.9523	171.352	303.87
22669	3.9524	161.921	117.461
22670	3.9525	168.427	214.276
22671	3.9525	152.637	103.822
22672	3.9525	186.615	58.2064
22673	3.9526	201.014	36.8987
22674	3.9526	164.665	231.66
22675	3.9526	135.426	348.464
22676	3.9527	162.412	102.521
22677	3.9528	165.565	109.16
22678	3.9529	201.777	79.1774
22679	3.9531	156.988	259.037
22680	3.9531	159.781	268.277
22681	3.9532	215.699	32.3456
22682	3.9533	216.507	115.652
22683	3.9534	217.891	313.694
22684	3.9534	150.652	134.235
22685	3.9536	175.464	126.733
22686	3.9537	125.448	180.471
22687	3.9539	154.02	181.887
22688	3.9539	145.863	155.791
22689	3.9539	164.022	145.968
22690	3.954	160.238	131.214
22691	3.954	164.17	147.564
22692	3.9541	191.667	73.8494
22693	3.9542	191.97	275.864

22694	3.9543	163.55	91.6609
22695	3.9544	235.442	242.847
22696	3.9545	158.546	320.754
22697	3.9546	181.178	19.9077
22698	3.9546	157.586	148.409
22699	3.9546	171.681	214.951
22700	3.9547	242.09	308.134
22701	3.9547	169.178	113.423
22702	3.9548	167.385	163.727
22703	3.9549	139.522	307.751
22704	3.955	149.739	93.2734
22705	3.955	151.537	137.249
22706	3.9551	166.762	37.3252
22707	3.9552	163.413	261.586
22708	3.9553	213.228	52.1403
22709	3.9553	168.949	92.8988
22710	3.9553	134.032	326.746
22711	3.9553	202.882	36.3787
22712	3.9553	164.259	152.664
22713	3.9554	185.725	197.935
22714	3.9555	159.493	155.393
22715	3.9555	-2.8067	132.752
22716	3.9555	135.8	110.646
22717	3.9555	175.781	108.581
22718	3.9556	178.456	331.176
22719	3.9556	147.795	171.038
22720	3.9557	107.318	160.913
22721	3.9557	145.77	134.568
22722	3.9558	164.113	135.248
22723	3.956	172.555	349.417
22724	3.956	117.024	162.967
22725	3.956	147.111	257.354
22726	3.9561	169.261	125.683
22727	3.9561	194.273	218.65
22728	3.9562	139.698	128.227
22729	3.9563	253.44	310.982
22730	3.9565	189.85	40.9644
22731	3.9565	162.385	95.3537
22732	3.9565	165.997	302.992
22733	3.9567	165.699	240.361
22734	3.9569	127.323	88.4515
22735	3.957	144.329	95.7532
22736	3.9571	134.664	100.53
22737	3.9571	149.726	203.975
22738	3.9571	134.618	61.0416
22739	3.9571	141.009	130.876
22740	3.9571	157.614	71.9822
22741	3.9572	157.48	76.8888

22742	3.9572	144.406	279.797
22743	3.9572	181.557	100.318
22744	3.9574	160.104	40.4308
22745	3.9574	138.51	164.739
22746	3.9574	147.558	20.3412
22747	3.9578	125.761	121.281
22748	3.9579	171.847	61.9153
22749	3.958	173.104	48.8616
22750	3.958	192.17	63.9925
22751	3.958	222.026	116.863
22752	3.958	135.723	187.727
22753	3.958	125.628	163.651
22754	3.9581	158.836	162.578
22755	3.9582	165.775	228.227
22756	3.9583	160.261	307.187
22757	3.9584	170.451	100.962
22758	3.9584	150.377	15.7816
22759	3.9588	126.423	178.256
22760	3.959	158.272	84.228
22761	3.959	139.386	73.8384
22762	3.9591	194.186	103.717
22763	3.9592	155.199	139.901
22764	3.9592	122.841	117.151
22765	3.9593	194.453	89.9112
22766	3.9594	133.449	26.5026
22767	3.9594	-2.8103	183.644
22768	3.9595	178.881	92.324
22769	3.9596	150.652	260.489
22770	3.9599	196.933	196.669
22771	3.9599	182.378	25.1361
22772	3.9601	167.061	4.7775
22773	3.9601	166.214	144.008
22774	3.9603	127.006	2.5935
22775	3.9604	179.755	302.822
22776	3.9607	168.245	24.5457
22777	3.9608	171.91	191.689
22778	3.9609	182.705	47.4317
22779	3.9609	160.569	263.284
22780	3.961	133.83	178.985
22781	3.9611	200.142	148.261
22782	3.9612	137.946	305.662
22783	3.9613	155.481	349.406
22784	3.9615	164.46	158.386
22785	3.9615	163.581	317.798
22786	3.9615	146.162	54.7975
22787	3.9616	161.45	112.942
22788	3.9622	32.9176	202.142
22789	3.9622	149.115	243.58

22790	3.9622	139.759	50.5029
22791	3.9622	164.119	37.4032
22792	3.9624	156.624	127.099
22793	3.9624	161.731	150.331
22794	3.9624	164.458	254.438
22795	3.9625	159.664	49.8337
22796	3.9625	167.428	43.2045
22797	3.9626	160.808	299.253
22798	3.9627	152.657	81.9751
22799	3.9628	192.43	5.8348
22800	3.9628	182.735	60.0777
22801	3.9629	156.808	91.5929
22802	3.9629	162.062	293.955
22803	3.963	138.977	201.064
22804	3.9632	176.361	41.1969
22805	3.9634	163.187	217.272
22806	3.9636	158.833	242.097
22807	3.9639	146.186	96.5963
22808	3.9639	144.057	48.5519
22809	3.9639	164.805	108.978
22810	3.9641	134.937	211.645
22811	3.9643	138.003	161.314
22812	3.9644	206.533	50.024
22813	3.9645	172.568	118.202
22814	3.9646	198.092	110.927
22815	3.9646	148.428	114.731
22816	3.9646	145.009	90.1218
22817	3.9647	148.769	305.614
22818	3.9647	169.571	98.5492
22819	3.9649	162.783	137.425
22820	3.9649	162.468	119.653
22821	3.965	163.305	124.553
22822	3.9651	223.272	77.1371
22823	3.9653	142.123	114.173
22824	3.9655	145.829	131.41
22825	3.9657	157.082	94.049
22826	3.9657	163.734	115.06
22827	3.9658	171.933	125.882
22828	3.9659	144.752	114.978
22829	3.966	164.949	105.321
22830	3.9661	175.825	26.5034
22831	3.9661	224.9	37.711
22832	3.9661	145.693	290.424
22833	3.9663	195.729	157.511
22834	3.9664	175.374	153.164
22835	3.9664	177.099	74.6972
22836	3.9664	177.599	106.646
22837	3.9664	175.549	45.7698

22838	3.9666	197.075	53.9247
22839	3.9668	184.9	78.7386
22840	3.9669	165.934	69.8845
22841	3.9669	173.508	341.858
22842	3.967	104.033	8.7357
22843	3.967	168.1	71.914
22844	3.9671	199.358	93.092
22845	3.9672	179.157	109.953
22846	3.9674	147.912	24.7949
22847	3.9674	127.825	156.09
22848	3.9675	166.98	341.42
22849	3.9675	198.842	94.5012
22850	3.9676	160.444	72.8092
22851	3.9676	131.651	148.073
22852	3.9676	197.883	101.368
22853	3.9677	181.889	222.486
22854	3.968	-2.8784	32.1125
22855	3.968	164.409	23.3595
22856	3.9681	147.866	123.292
22857	3.9684	174.66	253.186
22858	3.9685	182.646	189.317
22859	3.9686	215.417	316.776
22860	3.9688	142.279	122.06
22861	3.9689	152.418	219.135
22862	3.969	148.92	243.659
22863	3.969	161.749	103.653
22864	3.9692	266.34	315.13
22865	3.9692	177.905	155.386
22866	3.9692	168.512	40.6851
22867	3.9692	218.187	22.4935
22868	3.9693	216.984	4.3161
22869	3.9694	165.189	146.135
22870	3.9694	165.151	40.4729
22871	3.9694	127.746	58.6669
22872	3.9694	214.133	175.336
22873	3.9695	204.68	100.963
22874	3.9695	161.115	239.386
22875	3.9695	152.386	311.838
22876	3.9696	145.253	185.602
22877	3.9696	182.454	231.53
22878	3.9697	130.623	92.3174
22879	3.9698	151.812	179.452
22880	3.9698	161.106	310.909
22881	3.9698	145.85	171.806
22882	3.9699	229.875	85.551
22883	3.9699	161.904	55.1262
22884	3.9699	198.399	202.251
22885	3.9701	175.693	74.9336

22886	3.9702	149.33	266.6
22887	3.9702	173.974	62.876
22888	3.9703	180.762	101.027
22889	3.9705	150.55	197.104
22890	3.971	127.725	179.537
22891	3.9713	192.302	318.243
22892	3.9713	103.554	108.205
22893	3.9713	167.597	140.537
22894	3.9713	142.606	75.2954
22895	3.9714	153.681	87.4897
22896	3.9715	168.179	122.077
22897	3.9716	155.506	88.8222
22898	3.9717	107.925	187.782
22899	3.9719	212.474	38.5022
22900	3.9719	167.191	141.244
22901	3.972	181.614	274.395
22902	3.9722	166.191	113.986
22903	3.9722	128.493	202.232
22904	3.9723	152.383	63.531
22905	3.9723	152.545	69.4477
22906	3.9729	142.969	136.155
22907	3.9729	161.077	104.576
22908	3.9729	179.259	137.463
22909	3.9731	163.579	142.022
22910	3.9731	162.288	61.88
22911	3.9733	108.541	274.625
22912	3.9734	142.372	162.546
22913	3.9734	181.691	301.605
22914	3.9734	148.845	124.582
22915	3.9735	153.305	164.562
22916	3.9735	179.473	102.298
22917	3.9735	130.579	184.587
22918	3.9736	-2.8019	120.164
22919	3.9737	209.575	52.422
22920	3.9737	159.731	74.1268
22921	3.9741	167.757	175.607
22922	3.9742	167.543	116.011
22923	3.9742	180.214	42.2404
22924	3.9745	74.3332	10.7118
22925	3.9747	131.914	292.743
22926	3.9747	167.564	44.0825
22927	3.9747	156.374	265.053
22928	3.9747	195.543	287.609
22929	3.9748	141.92	117.403
22930	3.9749	148.402	132.895
22931	3.9749	162.477	189.101
22932	3.9752	180.363	47.7695
22933	3.9752	233.545	310.624

22934	3.9752	173.432	29.7666
22935	3.9753	166.278	265.717
22936	3.9753	158.224	281.885
22937	3.9753	-2.7327	284.58
22938	3.9755	174.246	232.927
22939	3.9756	164.982	60.7132
22940	3.9756	154.624	267.45
22941	3.9757	201.475	58.7763
22942	3.976	126.072	232.749
22943	3.9761	159.109	144.826
22944	3.9762	122.892	30.745
22945	3.9762	170.629	102.582
22946	3.9763	220.776	318.803
22947	3.9764	173.685	91.5106
22948	3.9765	165.614	136.65
22949	3.9765	230.577	285.272
22950	3.9767	158.808	289.947
22951	3.9769	117.632	163.759
22952	3.9769	141.499	335.661
22953	3.9769	151.629	175.145
22954	3.977	218.855	114.444
22955	3.977	135.821	273.209
22956	3.977	193.915	43.4253
22957	3.9771	133.632	28.0505
22958	3.9774	161.201	257.731
22959	3.9775	168.432	290.604
22960	3.9775	143.028	125.121
22961	3.9777	136.827	293.12
22962	3.9777	143.663	167.334
22963	3.9778	146.076	148.879
22964	3.9779	236.972	358.811
22965	3.9779	172.076	102.231
22966	3.9779	150.444	117.56
22967	3.978	189.831	63.1219
22968	3.9782	157.848	181.237
22969	3.9782	125.242	170.981
22970	3.9783	149.759	143.987
22971	3.9783	158.932	229.514
22972	3.9785	156.1	175.237
22973	3.9785	156.297	104.299
22974	3.9787	175.904	316.086
22975	3.9788	168.153	119.407
22976	3.9788	146.512	236.334
22977	3.9788	171.863	99.1193
22978	3.9788	195.847	261.039
22979	3.9788	179.296	194.95
22980	3.9789	168.119	48.197
22981	3.9789	175.451	157.823

22982	3.979	159.292	84.6404
22983	3.979	156.742	190.638
22984	3.9792	166.092	278.255
22985	3.9792	168.19	314.178
22986	3.9793	156.506	123.178
22987	3.9793	153.701	234.357
22988	3.9794	184.01	259.341
22989	3.9794	-3.2084	150.946
22990	3.9795	196.611	9.5726
22991	3.9795	135.321	242.08
22992	3.9796	188.812	128.663
22993	3.9796	163.93	317.118
22994	3.9796	134.318	175.964
22995	3.9797	178.03	83.8431
22996	3.9797	197.46	67.1149
22997	3.9798	148.072	176.994
22998	3.9798	155.233	65.5788
22999	3.98	167.969	43.6453
23000	3.9801	205.794	194.49
23001	3.9801	175.506	238.359
23002	3.9802	154.209	104.359
23003	3.9802	157.641	217.079
23004	3.9802	149.766	226.039
23005	3.9802	171.438	173.793
23006	3.9802	159.246	103.592
23007	3.9804	164.145	114.657
23008	3.9805	151.023	317.494
23009	3.9808	200.406	22.5783
23010	3.9808	147.653	121.645
23011	3.9809	165.731	111.067
23012	3.9811	150.148	355.611
23013	3.9812	148.579	105.735
23014	3.9813	179.137	287.801
23015	3.9814	183.865	147.527
23016	3.9814	149.855	100.533
23017	3.9815	211.112	74.3691
23018	3.9815	183.252	255.882
23019	3.9816	164.355	15.1792
23020	3.9816	154.837	169.293
23021	3.9818	176.376	194.315
23022	3.9819	189.394	259.83
23023	3.9819	169.775	99.2542
23024	3.9819	185.62	65.3481
23025	3.982	166.802	214.205
23026	3.9821	160.062	78.1554
23027	3.9822	167.688	37.37
23028	3.9823	174.584	299.04
23029	3.9824	187.219	73.0162

23030	3.9824	119.347	250.658
23031	3.9825	200.99	65.8908
23032	3.9827	171.219	132.211
23033	3.9827	137.431	120.439
23034	3.9828	166.946	115.858
23035	3.9828	211.657	25.0002
23036	3.9829	221.376	12.1704
23037	3.9829	166.865	64.2868
23038	3.9829	146.869	135.518
23039	3.983	135.652	7.0638
23040	3.983	193.261	69.7378
23041	3.9832	196.442	74.3622
23042	3.9832	148.21	250.631
23043	3.9832	160.527	111.431
23044	3.9834	186.728	317.163
23045	3.9834	158.439	161.113
23046	3.9834	98.0388	152.445
23047	3.9834	163.571	8.7819
23048	3.9836	163.304	131.869
23049	3.9836	162.783	198.631
23050	3.9837	163.199	36.9972
23051	3.9837	133.324	33.8369
23052	3.9838	162.331	308.788
23053	3.9838	179.667	103.588
23054	3.9839	187.378	67.3419
23055	3.9839	108.614	176.082
23056	3.9841	136.398	193.574
23057	3.9843	194.249	158.531
23058	3.9843	158.538	155.212
23059	3.9843	167.485	129.514
23060	3.9844	173.478	225.059
23061	3.9844	119.007	163.817
23062	3.9844	149.302	99.1546
23063	3.9845	89.9902	136.453
23064	3.9845	-2.792	307.052
23065	3.9846	135.192	70.8944
23066	3.9848	179.288	201.478
23067	3.9848	155.542	122.323
23068	3.9849	171.026	279.032
23069	3.9849	128.644	322.423
23070	3.9849	178.889	93.0909
23071	3.9849	169.458	45.7866
23072	3.985	174.307	119.971
23073	3.985	152.239	262.391
23074	3.9851	167.024	122.12
23075	3.9852	152.657	135.532
23076	3.9853	164.091	98.0298
23077	3.9854	144.965	98.3333

23078	3.9855	160.662	231.387
23079	3.9856	39.2556	358.797
23080	3.9857	155.176	302.772
23081	3.9857	148.076	98.8038
23082	3.9858	142.84	213.563
23083	3.9859	157.093	220.592
23084	3.9859	199.146	205.552
23085	3.9859	171.964	102.821
23086	3.986	160.345	297.912
23087	3.9862	188.003	44.9872
23088	3.9862	152.939	58.0703
23089	3.9862	87.0137	87.1742
23090	3.9863	132.916	192.438
23091	3.9863	160.831	229.938
23092	3.9864	206.786	44.02
23093	3.9864	126.581	192.866
23094	3.9865	160.264	140.431
23095	3.9867	143.047	27.7685
23096	3.987	180.71	98.773
23097	3.9871	178.703	162.564
23098	3.9871	158.594	226.982
23099	3.9871	0.2539	331.358
23100	3.9873	177.77	141.246
23101	3.9874	184.218	84.968
23102	3.9874	182.642	103.665
23103	3.9875	158.626	274.205
23104	3.9876	145.096	155.556
23105	3.9876	151.783	117.098
23106	3.9876	131.124	136.413
23107	3.9876	225.688	64.7557
23108	3.9878	188.083	296.36
23109	3.9878	132.036	191.677
23110	3.9878	183.989	171.124
23111	3.988	194.583	290.541
23112	3.988	160.931	154.226
23113	3.9882	129.992	125.104
23114	3.9882	156.659	156.665
23115	3.9882	170.149	110.615
23116	3.9882	193.634	344.965
23117	3.9883	135.912	252.198
23118	3.9885	132.434	97.8223
23119	3.9887	136.12	87.2939
23120	3.9887	147.534	172.682
23121	3.9888	145.493	328.125
23122	3.989	150.413	153.356
23123	3.9891	147.118	111.324
23124	3.9893	160.099	159.677
23125	3.9893	181.876	66.2884

23126	3.9894	206.001	173.954
23127	3.9894	180.94	81.3752
23128	3.9894	169.492	109.457
23129	3.9894	114.129	126.283
23130	3.9894	141.971	183.664
23131	3.9897	155.143	142.6
23132	3.9897	179.196	20.6339
23133	3.9898	192.347	135.079
23134	3.9898	168.399	67.4141
23135	3.9898	159.65	112.385
23136	3.99	162.887	225.287
23137	3.9902	182.21	117.862
23138	3.9902	160.044	287.757
23139	3.9904	142.783	212.737
23140	3.9904	157.1	150.353
23141	3.9905	213.426	53.1361
23142	3.9905	145.641	163.54
23143	3.9906	145.755	196.354
23144	3.9906	180.801	72.0833
23145	3.9906	154.082	311.092
23146	3.9907	170.244	275.963
23147	3.9909	176.663	232.168
23148	3.9909	175.552	48.0665
23149	3.9911	146.813	181.151
23150	3.9911	161.553	191.99
23151	3.9912	150.286	216.684
23152	3.9912	158.566	241.056
23153	3.9914	147.4	254.978
23154	3.9914	-2.8115	101.517
23155	3.9914	214.195	156.678
23156	3.9915	155.484	114.899
23157	3.9915	171.081	138.735
23158	3.9916	162.923	271.709
23159	3.9919	172.809	67.6348
23160	3.9921	32.536	263.489
23161	3.9921	187.135	284.362
23162	3.9922	168.151	147.826
23163	3.9922	167.484	110.245
23164	3.9924	151.918	78.4705
23165	3.9924	132.834	162.067
23166	3.9924	182.04	46.5044
23167	3.9925	161.068	173.641
23168	3.9925	144.753	115.938
23169	3.9925	166.371	129.214
23170	3.9927	214.873	87.6201
23171	3.9927	199.832	91.9389
23172	3.9928	200.156	160.299
23173	3.9929	168.369	221.671

23174	3.9929	192.161	326.194
23175	3.9929	159.376	172.874
23176	3.9931	155.584	41.5765
23177	3.9932	146.188	106.383
23178	3.9933	170.28	84.4923
23179	3.9934	164.863	269.839
23180	3.9934	184.385	204.498
23181	3.9934	101.097	153.82
23182	3.9935	169.887	153.628
23183	3.9936	114.838	38.9402
23184	3.9937	192.076	172.632
23185	3.9939	131.554	171.415
23186	3.994	183.345	150.299
23187	3.9941	145.429	347.903
23188	3.9943	148.17	167.715
23189	3.9943	147.266	52.187
23190	3.9944	182.613	57.6932
23191	3.9945	119.392	150.665
23192	3.9945	142.113	95.5692
23193	3.9947	204.522	120.943
23194	3.9947	165.372	98.4066
23195	3.9947	181.236	53.9542
23196	3.9948	160.525	36.6921
23197	3.9948	130.201	268.389
23198	3.9949	189.414	21.0514
23199	3.9949	174.677	286.693
23200	3.995	185.826	154.069
23201	3.995	-2.7651	112.031
23202	3.9951	151.964	184.231
23203	3.9951	198.533	82.6972
23204	3.9952	126.055	244.177
23205	3.9952	163.687	90.233
23206	3.9953	191.855	153.896
23207	3.9953	163.028	89.3765
23208	3.9954	163.796	292.2
23209	3.9954	194.523	355.272
23210	3.9955	166.358	177.874
23211	3.9955	167.497	106.103
23212	3.9958	163.652	248.327
23213	3.9959	151.581	107.948
23214	3.9959	100.947	4.8122
23215	3.996	165.158	187.511
23216	3.9962	147.636	33.3793
23217	3.9962	161.606	270.644
23218	3.9964	216.913	100.923
23219	3.9964	146.072	154.036
23220	3.9965	157.591	217.008
23221	3.9965	183.728	109.585

23222	3.9966	128.289	249.387
23223	3.9966	149.174	103.426
23224	3.9966	204.016	19.5612
23225	3.9967	185.108	265.105
23226	3.9968	192.692	266.295
23227	3.9969	167.872	159.615
23228	3.997	15.302	138.313
23229	3.9971	200.14	37.8578
23230	3.9971	168.524	136.136
23231	3.9971	155.102	133.476
23232	3.9972	238.72	202.588
23233	3.9972	161.614	75.5406
23234	3.9972	202.113	177.597
23235	3.9973	162.613	135.051
23236	3.9973	168.309	99.4213
23237	3.9974	152.446	135.701
23238	3.9975	184.467	175.651
23239	3.9975	181.965	35.1537
23240	3.9976	161.792	198.053
23241	3.9976	174.491	130.824
23242	3.9977	177.377	173.421
23243	3.9979	-2.7795	275.981
23244	3.998	-2.8448	145.812
23245	3.9981	180.91	61.6405
23246	3.9981	168.163	138.146
23247	3.9982	173.81	97.6816
23248	3.9982	196.366	155.208
23249	3.9983	166.56	271.029
23250	3.9985	156.921	308.116
23251	3.9985	201.199	97.1942
23252	3.9986	149.352	193.292
23253	3.9988	184.512	316.011
23254	3.9989	159.172	193.972
23255	3.9989	142.425	199.679
23256	3.9989	162.808	220.142
23257	3.9989	146.069	142.397
23258	3.999	126.694	254.993
23259	3.999	159.713	221.064
23260	3.999	188.948	142.585
23261	3.9991	167.178	90.5204
23262	3.9992	199.583	68.8581
23263	3.9994	188.114	269.957
23264	3.9995	161.417	114.786
23265	3.9996	168.023	70.781
23266	3.9996	158.417	119.031
23267	3.9997	170.615	197.569
23268	3.9997	156.317	109.983
23269	3.9998	161.015	63.9347

23270	3.9998	178.948	271.713
23271	3.9998	175.818	74.5072
23272	3.9999	202.427	65.6182
23273	3.9999	166.672	143.697
23274	4	191.302	76.909
23275	4.0001	160.062	131.926
23276	4.0002	136.548	307.468
23277	4.0002	190.791	83.801
23278	4.0002	167.837	156.267
23279	4.0005	178.164	69.9337
23280	4.0006	190.525	219.725
23281	4.0007	190.486	71.3356
23282	4.0007	142.023	221.8
23283	4.0007	224.638	150.885
23284	4.0008	137.067	60.6478
23285	4.0009	179.663	30.0709
23286	4.0009	224.499	30.2087
23287	4.0013	150.583	260.874
23288	4.0014	168.835	170.677
23289	4.0015	187.452	53.8841
23290	4.0015	172.285	71.8853
23291	4.0015	150.928	59.6571
23292	4.0015	-2.9341	194.176
23293	4.0016	179.088	30.4691
23294	4.0017	184.526	38.2709
23295	4.0017	181.738	270.405
23296	4.0017	137.151	266.563
23297	4.0017	223.054	155.022
23298	4.0017	188.323	173.629
23299	4.0018	162.549	245.995
23300	4.0018	151.989	17.086
23301	4.002	167.109	65.5346
23302	4.002	174.905	93.3648
23303	4.002	150.788	49.597
23304	4.0023	159.073	276.058
23305	4.0023	187.106	42.4497
23306	4.0023	164.01	134.155
23307	4.0024	144.643	296.872
23308	4.0024	188.751	111.564
23309	4.0025	175.364	271.578
23310	4.0029	157.179	158.684
23311	4.0029	147.717	225.843
23312	4.003	172.849	179.95
23313	4.003	141.131	156.976
23314	4.0032	198.063	78.8112
23315	4.0033	141.494	213.999
23316	4.0033	153.741	128.901
23317	4.0033	186.181	323.7

23318	4.0034	147.356	172.741
23319	4.0035	158.45	64.052
23320	4.0035	134.43	76.0976
23321	4.0037	153.492	88.4381
23322	4.0038	181.859	124.531
23323	4.0038	162.85	314.879
23324	4.0038	163.67	305.507
23325	4.0038	127.161	69.2015
23326	4.0039	206.256	95.9294
23327	4.004	169.217	103.501
23328	4.004	145.135	188.513
23329	4.004	165.119	225.354
23330	4.004	156.518	176.092
23331	4.0041	169.22	198.949
23332	4.0043	184.408	64.6124
23333	4.0044	150.056	19.3426
23334	4.0045	204.859	21.6861
23335	4.0045	163.061	166.547
23336	4.0046	160.629	114.888
23337	4.0047	182.622	66.9662
23338	4.0047	145.075	143.322
23339	4.0048	129.64	180.764
23340	4.0049	179.168	18.3655
23341	4.0049	233.468	150.194
23342	4.0051	190.738	57.5965
23343	4.0051	181.922	221.908
23344	4.0051	198.394	82.2413
23345	4.0052	173.601	79.81
23346	4.0052	181.113	137.809
23347	4.0052	180.777	141.338
23348	4.0054	112.63	90.6488
23349	4.0054	190.655	334.644
23350	4.0055	162.538	35.9552
23351	4.0056	171.641	39.2561
23352	4.0056	177.308	221.838
23353	4.0057	166.225	196.148
23354	4.0059	212.092	74.0336
23355	4.0059	183.846	109.543
23356	4.0059	152.318	102.397
23357	4.006	205.673	90.5969
23358	4.0061	192.02	72.3764
23359	4.0062	159.27	153.712
23360	4.0063	141.002	231.684
23361	4.0064	177.35	76.824
23362	4.0064	169.253	345.491
23363	4.0066	153.586	245.279
23364	4.0066	158.769	170.344
23365	4.0066	167.227	135.136

23366	4.0068	178.883	104.183
23367	4.0068	157.49	70.5649
23368	4.0068	149.878	117.685
23369	4.0068	158.986	159.288
23370	4.0069	176.127	117.863
23371	4.0069	155.801	318.956
23372	4.007	148.564	178.739
23373	4.0071	190.075	78.9199
23374	4.0072	150.205	180.737
23375	4.0073	170.317	163.076
23376	4.0073	152.81	307.236
23377	4.0074	197.808	299.682
23378	4.0074	176.748	116.98
23379	4.0075	139.023	209.871
23380	4.0075	176.324	296.368
23381	4.0077	133.715	96.6
23382	4.0078	141.303	140.857
23383	4.0079	116.098	280.841
23384	4.0081	160.205	57.2803
23385	4.0083	172.289	253.84
23386	4.0083	147.832	215.02
23387	4.0083	89.8562	106.393
23388	4.0083	141.231	179.638
23389	4.0084	194.641	64.56
23390	4.0084	136.566	203.911
23391	4.0084	124.522	96.7069
23392	4.0084	155.194	172.938
23393	4.0085	168.602	99.2534
23394	4.0085	161.322	185.856
23395	4.0086	135.758	24.7257
23396	4.0086	209.338	303.925
23397	4.0087	158.273	198.19
23398	4.0088	202.45	57.3213
23399	4.0089	169.88	224.586
23400	4.0089	132.964	94.7563
23401	4.0089	159.441	95.3679
23402	4.0091	172.917	23.5987
23403	4.0092	168.11	134.127
23404	4.0094	149.678	132.98
23405	4.0095	167.803	108.657
23406	4.0096	220.228	38.9194
23407	4.0097	190.321	99.1862
23408	4.0097	172.919	194.394
23409	4.0097	160.284	147.952
23410	4.0097	205.832	36.8516
23411	4.0099	183.598	128.815
23412	4.01	209.213	289.183
23413	4.0101	185.255	40.3753

23414	4.0102	154.488	195.768
23415	4.0103	135.091	128.863
23416	4.0103	122.756	73.9003
23417	4.0104	163.116	188.073
23418	4.0105	196.575	275.557
23419	4.0105	211.698	195.145
23420	4.0105	163.151	45.5592
23421	4.0105	127.775	72.5009
23422	4.0106	68.6015	256.808
23423	4.0106	129.606	95.6177
23424	4.0106	173.851	97.1463
23425	4.0107	143.475	252.385
23426	4.0107	148.186	130.258
23427	4.0108	147.08	215.029
23428	4.011	182.612	292.441
23429	4.0112	171.527	102.035
23430	4.0112	194.607	97.4462
23431	4.0118	179.731	90.8501
23432	4.012	146.732	151.414
23433	4.0121	172.085	59.1616
23434	4.0121	169.769	146.97
23435	4.0121	184.714	6.5546
23436	4.0121	177.708	187.02
23437	4.0122	184.631	102.484
23438	4.0123	199.167	45.9105
23439	4.0124	177.857	157.047
23440	4.0125	175.771	80.7466
23441	4.0125	134.293	126.182
23442	4.0126	123.013	38.6328
23443	4.0128	181.084	124.003
23444	4.0129	198.912	82.5814
23445	4.013	183.471	152.061
23446	4.013	304.362	84.2838
23447	4.0131	190.946	109.952
23448	4.0131	162.961	131.256
23449	4.0132	143.172	107.413
23450	4.0132	128.795	125.103
23451	4.0132	140.427	162.022
23452	4.0133	132.242	191.501
23453	4.0133	175.22	77.9355
23454	4.0136	174.485	102.127
23455	4.0136	146.614	102.538
23456	4.0137	221.503	355.179
23457	4.0138	138.541	159.803
23458	4.0139	178.798	0.5707
23459	4.014	140.981	110.786
23460	4.014	166.876	267.321
23461	4.0141	215.35	86.23

23462	4.0142	118.362	320.332
23463	4.0143	144.469	178.826
23464	4.0143	192.925	128.713
23465	4.0144	182.695	20.4021
23466	4.0144	196.203	76.3268
23467	4.0145	222.944	102.72
23468	4.0146	218.192	40.2754
23469	4.0146	162.943	135.735
23470	4.0146	180.598	68.8033
23471	4.0147	178.865	146.105
23472	4.0148	150.874	184.11
23473	4.0149	160.417	347.805
23474	4.0149	154.467	132.676
23475	4.015	169.989	166.836
23476	4.015	176.736	71.0785
23477	4.0153	191.666	226.151
23478	4.0154	140.433	166.969
23479	4.0154	162.189	133.273
23480	4.0156	211.107	103.752
23481	4.0156	170.417	135.72
23482	4.0156	175.642	50.3739
23483	4.0158	195.196	285.225
23484	4.0158	159.619	132.046
23485	4.0159	120.989	1.6359
23486	4.0159	188.004	88.179
23487	4.016	190.128	25.0321
23488	4.016	-4.9938	16.437
23489	4.016	141.168	327.863
23490	4.016	162.999	155.804
23491	4.016	188.424	154.348
23492	4.0161	225.755	97.8874
23493	4.0161	200.012	165.983
23494	4.0164	214.426	11.014
23495	4.0164	210.424	96.8454
23496	4.0165	170.243	118.797
23497	4.0166	132.403	328.939
23498	4.0167	197.312	79.4772
23499	4.0167	177.993	78.657
23500	4.0168	142.98	99.8938
23501	4.0168	158.894	316.413
23502	4.0169	167.993	126.978
23503	4.017	174.216	272.073
23504	4.0173	147.806	270.536
23505	4.0174	138.52	305.516
23506	4.0174	181.845	193.644
23507	4.0175	184.325	296.655
23508	4.0175	211.683	125.732
23509	4.0175	154.455	36.8376

23510	4.0177	146.878	234.425
23511	4.0177	159.61	118.802
23512	4.0178	141.021	258.743
23513	4.0179	185.206	264.981
23514	4.018	155.726	25.4481
23515	4.0182	187.292	331.161
23516	4.0184	177.19	116.8
23517	4.0186	141.644	222.983
23518	4.0187	170.412	82.2807
23519	4.0187	60.8699	252.957
23520	4.0188	141.1	106.934
23521	4.0189	167.073	279.113
23522	4.0189	180.556	104.393
23523	4.019	151.717	236.981
23524	4.019	201.192	25.9722
23525	4.0191	182.643	109.701
23526	4.0191	184.437	25.2096
23527	4.0192	200.17	264.543
23528	4.0193	211.578	264.75
23529	4.0194	199.202	49.6488
23530	4.0195	201.601	108.915
23531	4.0195	156.861	193.95
23532	4.0195	161.667	104.92
23533	4.0196	199.184	223.171
23534	4.0201	133.829	153.711
23535	4.0202	188.818	277.039
23536	4.0204	157.475	132.558
23537	4.0204	160	160.383
23538	4.0204	199.419	324.383
23539	4.0205	130.909	307.219
23540	4.0205	168.741	190.057
23541	4.0205	145.638	69.9289
23542	4.0206	184.38	170.306
23543	4.0206	160.095	118.685
23544	4.0207	182.081	354.119
23545	4.0207	144.91	241.941
23546	4.0207	141.74	180.031
23547	4.0208	165.522	222.855
23548	4.0209	225.963	175.815
23549	4.021	154.352	3.9171
23550	4.0212	140.766	87.0542
23551	4.0212	144.16	34.7378
23552	4.0212	94.9563	110.345
23553	4.0213	155.749	120.424
23554	4.0214	167.133	237.19
23555	4.0215	138.843	279.433
23556	4.0216	196.508	138.53
23557	4.0217	161.695	160.238

23558	4.0218	190.765	38.1166
23559	4.0219	159.478	220.567
23560	4.0221	196.101	183.565
23561	4.0222	200.999	37.096
23562	4.0222	184.785	174.671
23563	4.0224	189.21	238.173
23564	4.0225	211.987	355.335
23565	4.0226	148.783	194.613
23566	4.0226	174.259	76.5926
23567	4.0226	138.573	253.475
23568	4.0227	176.586	309.256
23569	4.023	172.692	48.6474
23570	4.023	183.448	148.109
23571	4.0231	163.951	166.682
23572	4.0232	160.517	115.159
23573	4.0233	193.56	298.066
23574	4.0235	171.967	63.8835
23575	4.0238	155.019	292.56
23576	4.0238	149.644	129.699
23577	4.0238	177.268	1.2451
23578	4.024	153.228	325.681
23579	4.0241	207.268	40.0789
23580	4.0242	194.072	286.391
23581	4.0243	177.747	157.212
23582	4.0244	190.23	84.3418
23583	4.0244	204.309	49.6883
23584	4.0245	167.334	274.353
23585	4.0247	155.106	108.398
23586	4.0249	229.235	44.9663
23587	4.025	184.581	60.3454
23588	4.025	197.552	319.194
23589	4.0251	133.006	40.248
23590	4.0252	212.093	27.7849
23591	4.0253	171.477	346.486
23592	4.0254	176.5	82.8683
23593	4.0254	197.866	31.7869
23594	4.0255	166.963	99.3326
23595	4.0256	200.024	115.237
23596	4.0256	156.91	40.8304
23597	4.0258	145.339	269.641
23598	4.0258	148.599	158.281
23599	4.0259	114.539	37.7147
23600	4.026	164.538	275.181
23601	4.0262	177.263	76.6235
23602	4.0263	161.408	107.157
23603	4.0264	217.503	67.7853
23604	4.0265	150.724	217.474
23605	4.0267	178.287	288.668

23606	4.0267	140.281	198.415
23607	4.0268	175.459	220.991
23608	4.0269	148.454	124.236
23609	4.0269	227.216	94.1912
23610	4.0269	224.423	67.5116
23611	4.0273	157.97	244.264
23612	4.0273	155.015	237.546
23613	4.0273	198.497	116.3
23614	4.0273	197.94	256.375
23615	4.0274	168.696	176.367
23616	4.0275	173.249	79.4002
23617	4.0275	141	117.185
23618	4.0276	137.777	261.602
23619	4.0276	124.777	138.649
23620	4.0276	265.835	201.677
23621	4.0277	203.287	21.2072
23622	4.0278	180.14	69.496
23623	4.0281	205.086	55.5932
23624	4.0281	161.477	150.624
23625	4.0282	150.026	96.5139
23626	4.0282	198.648	71.6062
23627	4.0282	204.612	223.249
23628	4.0284	180.535	111.001
23629	4.0284	161.525	278.662
23630	4.0285	177.751	44.9848
23631	4.0285	162.724	289.487
23632	4.0286	200.22	233.398
23633	4.0286	204.823	217.627
23634	4.0286	154.036	140.083
23635	4.0287	176.467	149.541
23636	4.0288	182.993	96.2925
23637	4.0288	121.634	144.886
23638	4.029	186.716	255.232
23639	4.0291	233.008	45.7355
23640	4.0293	149.709	335.668
23641	4.0293	201.125	99.0548
23642	4.0294	206.6	5.6506
23643	4.0294	183.087	22.37
23644	4.0295	137.414	30.759
23645	4.0295	179.544	132.237
23646	4.0295	196.996	46.5788
23647	4.0297	125.24	159.522
23648	4.0299	172.105	267.38
23649	4.0299	170.722	88.3588
23650	4.0299	191.015	177.653
23651	4.03	193.125	117.368
23652	4.0301	171.75	172.677
23653	4.0301	145.083	104.563

23654	4.0301	147.641	132.017
23655	4.0302	203.017	3.5359
23656	4.0302	121.209	199.644
23657	4.0305	157.886	79.5191
23658	4.0305	126.8	107.876
23659	4.0308	171.215	111.796
23660	4.0308	253.346	208.085
23661	4.0309	177.552	267.4
23662	4.0309	132.621	175.713
23663	4.0309	160.146	217.938
23664	4.031	160.517	198.732
23665	4.0311	179.675	187.129
23666	4.0311	219.197	23.1728
23667	4.0312	190.829	106.188
23668	4.0312	182.624	193.782
23669	4.0312	181.352	90.7968
23670	4.0313	196.359	149.186
23671	4.0313	174.243	115.238
23672	4.0313	137.352	53.5805
23673	4.0315	135.877	120.356
23674	4.0315	204.776	158.888
23675	4.0318	209.62	173.397
23676	4.0318	199.022	216.88
23677	4.0318	202.423	200.485
23678	4.0318	146.469	106.159
23679	4.0318	162.907	119.056
23680	4.0319	168.271	109.202
23681	4.0321	161.95	101.715
23682	4.0326	173.271	266.113
23683	4.0329	201.43	283.728
23684	4.033	230.125	84.0081
23685	4.033	167.101	147.181
23686	4.0331	136.57	145.756
23687	4.0332	216.104	104.139
23688	4.0332	184.821	116.491
23689	4.0332	202.284	116.45
23690	4.0332	168.764	98.8414
23691	4.0333	209.799	78.0978
23692	4.0334	123.596	36.5856
23693	4.0335	165.329	18.5271
23694	4.0337	146.305	123.577
23695	4.034	150.491	128.248
23696	4.0342	174.574	134.304
23697	4.0342	161.424	125.351
23698	4.0345	174.961	20.9843
23699	4.0346	191.522	30.256
23700	4.0347	207.805	315.69
23701	4.0347	138.106	199.509

23702	4.0347	161.718	85.3703
23703	4.0347	146.618	19.5072
23704	4.0348	-4.2086	189.963
23705	4.0349	178.354	45.418
23706	4.035	142.753	172.405
23707	4.0351	164.303	266.831
23708	4.0353	180.109	316.636
23709	4.0356	163.321	264.731
23710	4.0356	190.066	236.57
23711	4.0356	166.174	247.413
23712	4.0357	216.646	35.9516
23713	4.0357	146.71	219.791
23714	4.0357	151.861	37.3301
23715	4.0359	176.875	99.3525
23716	4.0359	-3.614	31.6623
23717	4.036	182.571	1.3143
23718	4.0361	160.729	98.4147
23719	4.0363	180.393	108.657
23720	4.0363	205.07	305.686
23721	4.0363	159.386	167.292
23722	4.0364	186.118	141.982
23723	4.0364	102.442	151.242
23724	4.0364	156.557	158.72
23725	4.0364	182.296	299.048
23726	4.0365	177.463	325.107
23727	4.0366	198.238	329.702
23728	4.0367	194.864	29.752
23729	4.0367	143.583	173.142
23730	4.0367	168.537	229.702
23731	4.0367	157.628	290.616
23732	4.0368	141.748	149.123
23733	4.0372	171.104	83.6696
23734	4.0372	172.269	180.161
23735	4.0373	281.514	285.398
23736	4.0373	162.083	184.509
23737	4.0373	219.193	293.899
23738	4.0376	137.712	233.602
23739	4.0379	166.05	195.35
23740	4.0381	172.043	178.522
23741	4.0381	178.522	280.267
23742	4.0382	221.655	42.1915
23743	4.0383	173.125	330.963
23744	4.0384	208.482	73.8921
23745	4.0384	162.498	153.924
23746	4.0386	169.814	79.2339
23747	4.0386	145.155	34.4101
23748	4.0387	229.414	312.965
23749	4.0388	163.665	100.603

23750	4.0389	214.404	285.033
23751	4.0389	209.419	155.876
23752	4.0391	169.789	152.89
23753	4.0393	141.396	166.061
23754	4.0394	166.16	173.959
23755	4.0394	193.867	75.1337
23756	4.0394	134.409	152.524
23757	4.0394	175.677	119.064
23758	4.0394	164.891	243.035
23759	4.0395	146.044	283.26
23760	4.0395	128.477	314.881
23761	4.0395	168.42	231.062
23762	4.0396	160.862	31.8472
23763	4.0397	160.827	253.147
23764	4.0398	180.466	91.3915
23765	4.0399	177.079	352.382
23766	4.04	148.689	199.877
23767	4.04	160.627	40.1501
23768	4.0402	155.23	60.6551
23769	4.0403	224.736	314.559
23770	4.0403	196.992	152.05
23771	4.0403	142.908	139.501
23772	4.0403	190.948	163.568
23773	4.0404	182.412	290.438
23774	4.0405	182.578	81.7359
23775	4.0405	163.776	111.299
23776	4.0407	196.164	93.4586
23777	4.0408	144.433	102.421
23778	4.041	203.668	29.2936
23779	4.041	168.194	148.33
23780	4.0412	176.246	283.896
23781	4.0413	219.157	95.642
23782	4.0414	169.315	235.879
23783	4.0419	145.896	112.666
23784	4.042	155.004	161.582
23785	4.042	150.062	80.3387
23786	4.0421	155.464	176.24
23787	4.0423	140.229	154.371
23788	4.0424	146.547	206.738
23789	4.0424	188.35	66.7317
23790	4.0426	183.503	158.031
23791	4.0427	115.869	151.612
23792	4.0427	189.204	266.913
23793	4.0428	189.122	71.0135
23794	4.0428	174.741	136.959
23795	4.0429	177.762	66.8882
23796	4.043	255.498	28.2022
23797	4.0431	160.765	135.126

23798	4.0433	224.64	1.3764
23799	4.0433	141.817	180.465
23800	4.0433	139.352	178.036
23801	4.0434	170.156	232.54
23802	4.0435	192.909	174.476
23803	4.0435	234.566	314.021
23804	4.0435	155.65	117.645
23805	4.0435	156.226	106.618
23806	4.0436	173.588	280.004
23807	4.0436	131.88	246.699
23808	4.0437	151.693	128.302
23809	4.0438	224.461	24.3752
23810	4.0438	181.895	139.123
23811	4.044	190.56	59.1122
23812	4.0445	193.909	54.5744
23813	4.0445	198.97	50.1453
23814	4.0446	169.144	76.4595
23815	4.0446	186.231	121.61
23816	4.0447	209.096	120.82
23817	4.0448	173.346	186.065
23818	4.0449	170.488	230.665
23819	4.0451	163.412	270.026
23820	4.0452	189.078	48.8134
23821	4.0455	171.081	150.598
23822	4.0455	148.201	106.519
23823	4.0455	176.705	300.271
23824	4.0455	183.37	98.2718
23825	4.0456	212.391	63.3106
23826	4.0457	212.033	43.262
23827	4.0457	125.742	185.626
23828	4.0457	170.874	222.133
23829	4.0457	204.843	283.044
23830	4.0458	5.7768	237.037
23831	4.0459	236.29	97.2411
23832	4.0459	229.016	4.5949
23833	4.0459	166.198	2.3278
23834	4.046	191.638	250.607
23835	4.046	124.199	14.7169
23836	4.046	217.708	77.267
23837	4.0461	173.87	146.115
23838	4.0462	167.012	237.621
23839	4.0462	121.498	38.7314
23840	4.0463	190.542	66.9266
23841	4.0463	195.819	341.925
23842	4.0464	154.508	107.707
23843	4.0465	193.96	62.5416
23844	4.0465	206.567	241.1
23845	4.0466	205.361	200.313

23846	4.0466	155.239	86.9975
23847	4.0466	167.9	198.424
23848	4.0468	140.64	190.83
23849	4.0469	236.783	43.858
23850	4.0469	179.76	34.9045
23851	4.0469	118.771	65.1936
23852	4.047	145.804	186.582
23853	4.047	213.185	87.9748
23854	4.0471	167.485	106.917
23855	4.0471	176.128	133.356
23856	4.0473	153.559	254.712
23857	4.0474	220.286	249.876
23858	4.0476	206.442	32.1216
23859	4.0476	156.593	316.316
23860	4.0476	176.222	50.5486
23861	4.0477	152.672	151.404
23862	4.0477	171.268	138.349
23863	4.0478	168.452	80.6233
23864	4.0479	153.764	241.005
23865	4.0479	185.951	242.624
23866	4.0481	160.404	224.55
23867	4.0485	61.1887	9.1308
23868	4.0485	167.874	239.252
23869	4.0486	201.698	26.1776
23870	4.0486	169.796	161.93
23871	4.0487	164.37	107.893
23872	4.0489	162.763	80.4236
23873	4.049	185.577	159.617
23874	4.049	162.207	53.9772
23875	4.049	179.816	163.471
23876	4.0491	171.381	262.788
23877	4.0491	174.568	95.2037
23878	4.0492	166.83	266.854
23879	4.0493	182.87	259.586
23880	4.0493	107.076	158.783
23881	4.0493	176.409	18.5357
23882	4.0494	185.812	81.7009
23883	4.0494	140.953	171.139
23884	4.0494	164.979	164.97
23885	4.0495	185.972	150.897
23886	4.0495	159.912	42.1599
23887	4.0498	159.856	255.716
23888	4.0499	171.88	157.73
23889	4.05	205.958	105.932
23890	4.0502	123.985	35.1195
23891	4.0502	133.028	194.001
23892	4.0502	177.325	301.676
23893	4.0503	189.189	110.884

23894	4.0504	176.033	178.369
23895	4.0505	162.753	141.623
23896	4.0505	177.012	176.43
23897	4.0507	157.64	136.069
23898	4.0508	179.983	331.919
23899	4.0508	225.141	19.6947
23900	4.0509	152.874	195.925
23901	4.0509	183.675	108.552
23902	4.0509	176.166	105.551
23903	4.051	212.815	97.1948
23904	4.051	180.572	62.0721
23905	4.0513	153.433	217.069
23906	4.0514	197.226	140.402
23907	4.0514	178.594	283.084
23908	4.0514	134.446	129.476
23909	4.0514	245.502	18.2602
23910	4.0515	167.074	132.621
23911	4.0516	281.52	218.673
23912	4.0516	217.268	339.829
23913	4.0517	183.558	37.4172
23914	4.0517	197.847	120.606
23915	4.0518	209.687	348.912
23916	4.0518	136.344	351.126
23917	4.0518	155.656	163.603
23918	4.0519	182.083	174.809
23919	4.052	198.656	82.0208
23920	4.0522	180.239	194.477
23921	4.0523	148.247	169.459
23922	4.0524	143.993	155.818
23923	4.0524	168.681	128.264
23924	4.0524	175.231	142.976
23925	4.0525	221.49	41.7473
23926	4.0528	219.405	136.575
23927	4.0528	162.329	65.1618
23928	4.0528	234.447	290.184
23929	4.0529	141.135	169.81
23930	4.053	191.974	95.5755
23931	4.053	145.012	169.035
23932	4.053	171.579	87.5611
23933	4.053	196.617	118.466
23934	4.0534	172.589	47.8622
23935	4.0536	180.91	321.182
23936	4.0537	176.49	72.581
23937	4.0538	140.715	320.383
23938	4.0538	152.408	177.946
23939	4.0539	163.683	237.875
23940	4.0539	179.896	146.533
23941	4.054	204.796	115.887

23942	4.0541	161.696	218.436
23943	4.0541	165.37	29.3141
23944	4.0542	187.442	315.614
23945	4.0543	148.506	168.082
23946	4.0545	162.26	27.9435
23947	4.0546	180.946	194.342
23948	4.0546	185.18	97.7588
23949	4.0547	220.07	126.639
23950	4.0547	154.855	42.9694
23951	4.0548	176.429	175.534
23952	4.0548	197.908	52.4207
23953	4.055	174.908	105.218
23954	4.055	160.583	149.77
23955	4.0551	148.197	128.182
23956	4.0552	211.375	82.2216
23957	4.0554	188.781	11.3737
23958	4.0556	165.247	146.047
23959	4.0557	151.15	107.21
23960	4.0558	195.567	187.093
23961	4.0558	178.71	129.681
23962	4.0558	179.54	82.2961
23963	4.0562	55.8871	176.626
23964	4.0562	173.275	275.057
23965	4.0564	219.325	50.8798
23966	4.0564	174.215	116.132
23967	4.0564	185.982	77.6568
23968	4.0564	169.873	298.849
23969	4.0565	193.449	234.426
23970	4.0567	123.93	178.504
23971	4.0568	192.096	95.3531
23972	4.0568	137.57	77.9955
23973	4.0568	165.746	253.672
23974	4.0571	227.882	30.7811
23975	4.0571	167.868	190.334
23976	4.0571	178.568	248.01
23977	4.0572	177.569	127.74
23978	4.0572	168.998	132.251
23979	4.0572	172.559	167.945
23980	4.0573	224.79	40.4347
23981	4.0573	206.888	98.7584
23982	4.0574	147.691	174.698
23983	4.0575	245.594	98.9072
23984	4.0575	213.265	273.098
23985	4.0575	173.967	146.914
23986	4.0576	135.034	317.145
23987	4.0576	227.281	58.9008
23988	4.058	178.421	284.233
23989	4.058	170.502	250.603

23990	4.0582	218.127	89.9904
23991	4.0582	184.071	115.229
23992	4.0582	168.857	76.4703
23993	4.0584	291.284	286.935
23994	4.0584	169.016	219.199
23995	4.0585	157.761	164.396
23996	4.0585	169.888	70.3085
23997	4.0586	46.6334	62.7251
23998	4.0588	209.242	153.983
23999	4.0588	165.013	125.676
24000	4.0588	165.995	32.6177
24001	4.059	169.08	48.4487
24002	4.059	140.625	145.925
24003	4.059	152.054	153.144
24004	4.0591	136.722	260.948
24005	4.0592	251.695	68.3491
24006	4.0592	169.291	194.045
24007	4.0592	120.621	198.787
24008	4.0593	231.703	318.357
24009	4.0595	173.122	149.051
24010	4.0595	180.087	141.804
24011	4.0595	171.781	140.756
24012	4.0595	163.85	338.559
24013	4.0596	179.311	74.316
24014	4.0597	245.392	333.613
24015	4.0597	182.898	59.3791
24016	4.0597	162.648	203.891
24017	4.0599	182.127	268.297
24018	4.06	172.615	155.655
24019	4.06	55.6876	345.205
24020	4.06	122.403	142.478
24021	4.06	155.155	99.0284
24022	4.0603	211.431	80.7624
24023	4.0605	175.474	65.6219
24024	4.0605	157.16	93.072
24025	4.0606	194.84	112.056
24026	4.0606	239.829	264.181
24027	4.0608	219.43	190.935
24028	4.0608	176.518	153.331
24029	4.0608	146.018	173.79
24030	4.0609	140.692	102.373
24031	4.0609	181.293	170.734
24032	4.0611	161.283	260.229
24033	4.0611	197.832	176.101
24034	4.0611	211.343	323.126
24035	4.0612	224.995	284.701
24036	4.0612	171.415	164.08
24037	4.0613	140.446	183.387

24038	4.0613	299.588	245.291
24039	4.0615	159.796	122.48
24040	4.0616	205.304	94.7751
24041	4.0616	166.566	218.716
24042	4.0617	162.554	148.714
24043	4.0617	196.194	87.6431
24044	4.0617	213.581	321.219
24045	4.0619	150.016	165.703
24046	4.0619	125.777	256.731
24047	4.062	156.029	141.494
24048	4.0621	192.131	298.887
24049	4.0621	232.383	78.5476
24050	4.0621	125.73	253.736
24051	4.0621	150.364	131
24052	4.0622	180.992	143.654
24053	4.0623	221.348	4.4972
24054	4.0623	171.308	87.7334
24055	4.0623	170.03	162.027
24056	4.0625	136.587	222.606
24057	4.0626	176.983	153.094
24058	4.0626	145.469	234.984
24059	4.0627	127.76	107.704
24060	4.063	172.998	206.971
24061	4.0631	199.14	74.3154
24062	4.0631	158.374	192.606
24063	4.0632	154.722	125.14
24064	4.0632	178.251	71.2998
24065	4.0634	166.051	251.444
24066	4.0635	160.718	96.987
24067	4.0635	179.392	343.554
24068	4.0637	168.447	94.8632
24069	4.0638	196.534	307.105
24070	4.0638	202.563	24.9913
24071	4.0639	169.189	301.904
24072	4.064	166.302	235.607
24073	4.0641	215.538	50.739
24074	4.0641	180.09	100.69
24075	4.0642	203.749	35.4287
24076	4.0644	151.943	172.897
24077	4.0644	191.627	138.812
24078	4.0644	258.627	308.649
24079	4.0644	178.723	290.207
24080	4.0645	148.79	120.37
24081	4.0645	162.437	26.1511
24082	4.0645	172.976	66.2915
24083	4.0646	191.432	286.561
24084	4.0646	147.431	149.352
24085	4.0646	217.287	86.82

24086	4.0648	190.014	294.841
24087	4.065	215.66	24.4258
24088	4.0653	148.535	147.855
24089	4.0653	232.675	292.16
24090	4.0654	181.772	215.192
24091	4.0655	203.927	168.426
24092	4.0656	163.136	341.308
24093	4.0659	149.444	273.315
24094	4.0659	194.991	137.317
24095	4.066	157.747	63.0244
24096	4.0663	168.616	314.67
24097	4.0665	177.419	31.147
24098	4.0665	161.589	170.59
24099	4.0665	174.903	124.788
24100	4.0666	210.611	98.3751
24101	4.0666	175.314	72.1429
24102	4.0668	191.683	238.532
24103	4.0669	134.317	243.552
24104	4.0669	194.859	295.238
24105	4.067	187.132	296.911
24106	4.0671	171.819	55.8215
24107	4.0671	173.807	224.111
24108	4.0672	190.133	151.948
24109	4.0672	202.245	29.4642
24110	4.0672	196.769	70.3995
24111	4.0673	148.39	93.5984
24112	4.0673	179.968	48.2026
24113	4.0674	171.237	316.29
24114	4.0675	146.832	128.438
24115	4.0675	239.26	207.505
24116	4.0676	106.813	210.618
24117	4.0676	117.527	267.431
24118	4.0676	191.406	304.272
24119	4.068	160.787	136.414
24120	4.0682	170.005	141.233
24121	4.0683	164.344	89.9608
24122	4.0684	152.56	57.9203
24123	4.0684	166.433	221.812
24124	4.0685	179.57	275.968
24125	4.0685	164.452	114.685
24126	4.0686	201.039	131.716
24127	4.0686	183.059	73.886
24128	4.0688	212.269	112.956
24129	4.0691	141.479	90.868
24130	4.0691	129.881	36.1665
24131	4.0692	128.711	311.909
24132	4.0692	136.11	200.256
24133	4.0692	156.935	304.138

24134	4.0694	143.022	94.0152
24135	4.0694	157.734	109.376
24136	4.0695	187.895	245.554
24137	4.0695	149.521	191.269
24138	4.0695	183.543	274.963
24139	4.0695	137.346	174.894
24140	4.0697	132.519	225.38
24141	4.0697	206.965	87.8936
24142	4.0698	179.212	154.445
24143	4.0699	168.071	48.715
24144	4.07	214.365	64.7723
24145	4.0701	207.67	75.5981
24146	4.0701	193.002	68.1465
24147	4.0701	200.527	104.256
24148	4.0701	223.238	83.8109
24149	4.0703	168.926	187.538
24150	4.0703	191.363	30.5373
24151	4.0704	176.201	69.3117
24152	4.0705	121.933	139.118
24153	4.0706	181.263	270.793
24154	4.0707	211.23	44.1832
24155	4.0708	212.808	99.3077
24156	4.0709	140.333	153.544
24157	4.0709	165.779	112.339
24158	4.0711	186.916	254.424
24159	4.0713	228.273	329.602
24160	4.0714	195.74	103.514
24161	4.0717	172.161	179.907
24162	4.0717	63.1959	57.4275
24163	4.0718	196.572	61.7302
24164	4.0718	157.555	110.901
24165	4.0718	172.325	136.089
24166	4.0721	197.333	280.008
24167	4.0721	172.243	190.35
24168	4.0723	191.291	102.674
24169	4.0724	183.019	186.591
24170	4.0724	191.177	117.467
24171	4.0726	221.672	99.7036
24172	4.0727	158.446	113.703
24173	4.0729	221.071	287.923
24174	4.0729	214.477	196.976
24175	4.0729	141.422	105.826
24176	4.0731	211.416	195.852
24177	4.0731	218.285	67.6554
24178	4.0732	190.625	260.818
24179	4.0733	226.303	267.192
24180	4.0733	207.661	41.4693
24181	4.0733	184.156	117.325

24182	4.0733	130.704	196.625
24183	4.0735	165.447	164.696
24184	4.0735	165.139	40.0469
24185	4.0737	145.475	205.062
24186	4.0739	152.012	227.482
24187	4.074	174.958	149.997
24188	4.0742	168.293	261.866
24189	4.0742	206.687	275.35
24190	4.0743	169.031	230.883
24191	4.0743	168.388	129.234
24192	4.0746	139.79	176.61
24193	4.0746	163.554	128.159
24194	4.0746	243.119	178.906
24195	4.0747	186.612	151.086
24196	4.0749	242.03	130.394
24197	4.0749	195.648	193.405
24198	4.075	184.016	131.72
24199	4.075	196.894	152.014
24200	4.0751	172.415	48.8331
24201	4.0753	224.475	117.285
24202	4.0753	235.524	215.417
24203	4.0756	200.024	104.778
24204	4.0757	158.045	150.622
24205	4.0758	151.198	270.506
24206	4.0759	207.218	154.379
24207	4.076	217.791	99.3613
24208	4.0761	201.277	188.676
24209	4.0762	212.654	53.9361
24210	4.0765	170.824	145.308
24211	4.0766	205.181	254.414
24212	4.0767	194.486	5.2406
24213	4.0767	116.321	89.8237
24214	4.0769	185.403	252.81
24215	4.0769	181.086	156.337
24216	4.0769	192.795	109.998
24217	4.077	198.685	169.657
24218	4.077	171.226	166.954
24219	4.077	153.113	124.368
24220	4.0771	213.568	25.688
24221	4.0771	217.582	96.1034
24222	4.0771	201.085	138.231
24223	4.0772	153.678	203.728
24224	4.0772	185.356	61.7423
24225	4.0773	159.67	169.509
24226	4.0775	170.778	101.944
24227	4.0778	185.65	302.183
24228	4.0778	195.193	267.455
24229	4.0779	194.989	284.056

24230	4.0779	204.691	83.3805
24231	4.078	185.887	162.624
24232	4.0783	294.362	312.518
24233	4.0783	203.407	149.646
24234	4.0784	130.132	213.6
24235	4.0784	175.707	38.0781
24236	4.0785	73.4351	123.331
24237	4.0786	168.855	213.721
24238	4.0787	156.171	212.153
24239	4.0787	179.218	140.904
24240	4.0787	180.95	161.649
24241	4.0788	231.454	204.515
24242	4.0789	195.647	26.4765
24243	4.0789	149.717	353.272
24244	4.0789	204.283	20.5694
24245	4.0791	215.9	47.3993
24246	4.0791	205.44	48.746
24247	4.0791	187.831	304.76
24248	4.0792	198.607	17.7715
24249	4.0792	134.284	220.091
24250	4.0793	123.637	11.4259
24251	4.0795	130.375	321.859
24252	4.0797	195.775	141.243
24253	4.0798	177.445	187.097
24254	4.0798	157.036	76.6867
24255	4.0798	175.635	8.6098
24256	4.0799	189.674	32.5885
24257	4.08	166.821	254.155
24258	4.0802	217.881	261.048
24259	4.0803	256.56	242.019
24260	4.0804	169.024	22.518
24261	4.0804	184.545	72.3139
24262	4.0805	194.036	95.4352
24263	4.0806	171.859	198.158
24264	4.0807	154.579	223.759
24265	4.0807	155.357	348.915
24266	4.0807	189.391	290.337
24267	4.0807	130.428	191.317
24268	4.0807	160.175	183.73
24269	4.0809	139.045	270.066
24270	4.0811	236.562	84.0792
24271	4.0811	186.595	41.2402
24272	4.0812	149.243	102.553
24273	4.0814	158.992	108.361
24274	4.0815	167.94	15.3292
24275	4.0819	151.898	174.993
24276	4.0819	150.73	213.083
24277	4.0819	198.922	186.575

24278	4.0821	174.023	248.865
24279	4.0823	120.43	324.125
24280	4.0823	180.872	119.645
24281	4.0823	204.948	63.9249
24282	4.0824	173.946	174.315
24283	4.0827	144.061	192.255
24284	4.0827	225.944	63.6395
24285	4.0828	248.151	68.5444
24286	4.0828	192.81	188.37
24287	4.0829	196.314	83.809
24288	4.0829	181.803	144.495
24289	4.083	188.246	31.7194
24290	4.083	188.653	123.576
24291	4.0832	170.755	162.124
24292	4.0834	151.645	170.707
24293	4.0835	152.087	117.006
24294	4.0835	252.336	291.758
24295	4.0836	231.512	89.6628
24296	4.0838	184.18	54.9524
24297	4.0839	183.526	281.106
24298	4.084	148.115	221.891
24299	4.084	168.128	55.6021
24300	4.084	161.113	31.925
24301	4.084	175.424	175.977
24302	4.0841	187.189	165.191
24303	4.0841	148.862	183.528
24304	4.0845	166.205	188.057
24305	4.0846	156.996	261.377
24306	4.0846	137.565	70.6163
24307	4.0846	182.455	268.718
24308	4.0847	162.077	301.522
24309	4.0847	181.724	274.818
24310	4.0848	186.092	196.175
24311	4.0849	150.583	88.3575
24312	4.085	124.598	164.731
24313	4.0851	131.966	145.471
24314	4.0851	159.131	131.537
24315	4.0854	176.363	145.903
24316	4.0854	160.091	144.359
24317	4.0857	201.892	267.049
24318	4.0859	181.618	195.833
24319	4.0859	162.526	222.827
24320	4.086	134.081	249.97
24321	4.0864	184.583	287.75
24322	4.0864	164.372	129.348
24323	4.0865	163.451	200.33
24324	4.0865	189.525	235.522
24325	4.0866	146.978	86.0055

24326	4.0866	235.246	53.9717
24327	4.0866	229.27	287.521
24328	4.0866	197.976	70.1452
24329	4.0867	215.552	123.03
24330	4.0868	155.198	102.138
24331	4.0868	226.222	82.2358
24332	4.0869	168.497	192.923
24333	4.0869	183.495	246.777
24334	4.0871	225.599	78.15
24335	4.0871	156.185	116.76
24336	4.0873	217.492	118.841
24337	4.0874	207.298	97.1741
24338	4.0875	236.016	90.5263
24339	4.0875	158.606	252.524
24340	4.0877	174.096	99.5512
24341	4.0878	236.925	36.3482
24342	4.0878	170.785	171.822
24343	4.0879	171.058	100.099
24344	4.088	196.9	55.4734
24345	4.088	188.675	285.229
24346	4.0881	189.774	112.443
24347	4.0881	253.467	201.346
24348	4.0882	147.645	99.0184
24349	4.0883	134.766	32.3265
24350	4.0884	147.48	19.3882
24351	4.0884	189.288	150.683
24352	4.0885	249.61	41.0161
24353	4.0886	217.023	65.8948
24354	4.0886	218.917	345.633
24355	4.0886	124.285	205.576
24356	4.0887	139.296	222.588
24357	4.0887	191.946	137.592
24358	4.0889	197.603	200.65
24359	4.089	240.726	132.34
24360	4.089	172.095	123.89
24361	4.0892	193.595	40.083
24362	4.0893	217.359	260.896
24363	4.0893	192.225	103.737
24364	4.0893	192.825	90.1147
24365	4.0894	191.154	118.242
24366	4.0894	166.611	126.337
24367	4.0895	197.297	270.892
24368	4.0895	216.161	33.531
24369	4.0895	194.732	275.432
24370	4.0896	179.304	105.165
24371	4.0898	195.15	90.4332
24372	4.0898	214.8	64.3392
24373	4.0899	203.666	89.8236

24374	4.0899	177.07	284.562
24375	4.0899	195.155	100.325
24376	4.0899	162.388	123.106
24377	4.09	233.83	53.2708
24378	4.09	135.303	252.892
24379	4.09	203.331	282.491
24380	4.09	162.069	277.825
24381	4.0901	209.685	64.7599
24382	4.0901	180.095	120.842
24383	4.0902	182.744	97.9257
24384	4.0902	160.04	117.452
24385	4.0903	196.287	110.413
24386	4.0904	192.254	152.204
24387	4.0904	276.347	291.643
24388	4.0905	155.909	24.6515
24389	4.0907	182.392	118.834
24390	4.0907	201.366	174.393
24391	4.0908	178.375	161.645
24392	4.0909	187.764	187.42
24393	4.0909	177.143	198.33
24394	4.091	184.634	142.984
24395	4.091	164.589	196.59
24396	4.091	151.781	161.462
24397	4.091	151.475	176.64
24398	4.0912	182.749	92.6792
24399	4.0912	161.322	74.084
24400	4.0913	209.234	149.056
24401	4.0913	227.689	234.896
24402	4.0913	183.211	127.569
24403	4.0914	157.469	102.635
24404	4.0914	168.637	310.755
24405	4.0915	169.356	108.983
24406	4.0915	146.56	39.129
24407	4.0917	222.822	12.3299
24408	4.0917	165.642	224.402
24409	4.0918	180.878	259.825
24410	4.0919	177.153	100.575
24411	4.092	192.97	60.3346
24412	4.0921	182.847	259.007
24413	4.0921	164.688	218.172
24414	4.0921	174.712	95.0864
24415	4.0921	189.754	266.472
24416	4.0923	219.266	183.54
24417	4.0924	153.806	177.778
24418	4.0925	212.721	90.2547
24419	4.0927	177.065	220.922
24420	4.0928	183.005	103.485
24421	4.0928	193.552	11.4196

24422	4.0929	193.77	124.498
24423	4.0929	131.116	164.949
24424	4.093	193.358	247.371
24425	4.093	175.81	147.243
24426	4.093	127.552	95.0424
24427	4.0931	237.68	310.418
24428	4.0931	169.995	139.617
24429	4.0932	158.87	127.386
24430	4.0934	208.095	122.886
24431	4.0934	155.24	222.878
24432	4.0935	201.962	256.942
24433	4.0935	147.067	186.666
24434	4.0935	157.191	146.547
24435	4.0937	192.407	122.179
24436	4.0942	215.474	85.4496
24437	4.0943	198.251	116.178
24438	4.0945	165.962	58.43
24439	4.0945	195.576	143.687
24440	4.0945	222.474	161.534
24441	4.0947	201.858	28.4521
24442	4.0947	191.408	222.737
24443	4.0947	151.929	180.776
24444	4.0947	149.064	96.3184
24445	4.0948	152.353	196.192
24446	4.0949	149.83	167.014
24447	4.0949	170.387	167.509
24448	4.095	193.327	193.645
24449	4.0951	191.909	282.919
24450	4.0952	153.174	83.9486
24451	4.0953	222.339	44.8423
24452	4.0953	209.557	49.6386
24453	4.0954	171.277	62.6934
24454	4.0955	171.92	158.566
24455	4.0956	131.609	147.205
24456	4.0956	172.306	89.2683
24457	4.0956	211.998	322.628
24458	4.0957	159.357	25.4478
24459	4.0957	202.367	126.695
24460	4.0958	170.395	336.772
24461	4.0959	85.4797	12.8977
24462	4.0959	165.56	134.835
24463	4.0959	150.834	130.532
24464	4.0961	160.239	168.955
24465	4.0961	192.077	134.337
24466	4.0962	103.16	18.5135
24467	4.0962	208.397	316.643
24468	4.0963	236.968	63.1677
24469	4.0963	168.877	85.598

24470	4.0964	245.712	92.747
24471	4.0965	189.914	31.8922
24472	4.0965	188.251	151.568
24473	4.0966	183.916	143.661
24474	4.0967	182.714	259.214
24475	4.0967	-2.6821	14.8938
24476	4.0967	208.251	100.129
24477	4.0968	201.689	113.103
24478	4.0968	180.946	231.138
24479	4.0968	164.786	317.433
24480	4.0968	232.191	74.5447
24481	4.0969	255.038	359.176
24482	4.0969	180.549	119.766
24483	4.0973	176.822	199.197
24484	4.0973	164.93	292.474
24485	4.0973	216.36	203.131
24486	4.0974	181.918	143.211
24487	4.0974	200.119	136.825
24488	4.0974	181.878	165.011
24489	4.0974	178.951	108.367
24490	4.0975	198.977	74.0127
24491	4.0975	188.978	354.547
24492	4.0977	146.049	151.955
24493	4.0977	211.527	129.682
24494	4.0978	132.588	141.69
24495	4.0978	191.878	287.885
24496	4.0979	212.212	276.281
24497	4.098	195.949	274.469
24498	4.098	243.392	261.657
24499	4.0981	191.958	89.0539
24500	4.0982	182.318	195.676
24501	4.0982	184.182	173.893
24502	4.0983	242.183	44.5046
24503	4.0983	217.004	57.5849
24504	4.0984	189.712	164.37
24505	4.0985	220.127	10.2298
24506	4.0985	198.454	159.438
24507	4.0986	219.713	165.638
24508	4.0987	232.188	287.827
24509	4.0988	170.349	355.511
24510	4.0989	199.371	185.999
24511	4.0989	215.039	86.4803
24512	4.099	163.559	171.148
24513	4.0993	141.715	34.225
24514	4.0993	186.691	265.076
24515	4.0994	239.748	2.1659
24516	4.0994	161.082	128.607
24517	4.0997	132.589	112.651

24518	4.0997	182.19	97.3697
24519	4.0998	174.583	331.886
24520	4.0998	214.642	1.3138
24521	4.0999	175.478	242.468
24522	4.1	161.013	180.876
24523	4.1001	247.935	193.223
24524	4.1003	178.385	241.437
24525	4.1005	249.894	32.4069
24526	4.1005	189.902	48.928
24527	4.1005	197.785	200.452
24528	4.1006	166.232	180.833
24529	4.1006	178.345	141.209
24530	4.1007	140.786	87.2156
24531	4.1009	147.862	143.476
24532	4.101	165.277	266.128
24533	4.1011	150.944	245.66
24534	4.1013	169.49	103.015
24535	4.1015	185.459	35.7178
24536	4.1015	206.716	292.714
24537	4.1016	160.44	222.437
24538	4.1016	184.157	318.223
24539	4.1017	190.197	196.674
24540	4.1019	144.69	134.576
24541	4.1019	240.512	21.9153
24542	4.102	211.575	312.813
24543	4.1021	249.758	83.234
24544	4.1022	153.919	99.0644
24545	4.1022	174.543	34.4144
24546	4.1023	199.602	72.8658
24547	4.1023	191.396	117.982
24548	4.1024	224.649	107.302
24549	4.1024	216.11	128.486
24550	4.1024	216.069	103.91
24551	4.1025	149.379	238.709
24552	4.1025	199.793	359.01
24553	4.1025	-2.7925	294.781
24554	4.1026	155.261	99.8831
24555	4.1026	252.239	45.6397
24556	4.1027	187.843	96.6405
24557	4.1028	197.026	90.837
24558	4.1028	231.681	100.61
24559	4.103	202.794	255.381
24560	4.1031	214.933	92.6144
24561	4.1032	174.661	195.05
24562	4.1042	153.456	163.534
24563	4.1043	151.042	166.142
24564	4.1044	208.78	81.1227
24565	4.1044	178.077	39.8579

24566	4.1044	198.94	169.517
24567	4.1045	186.359	83.5413
24568	4.1045	139.782	141.558
24569	4.1045	191.212	100.938
24570	4.1046	147.933	195.13
24571	4.1047	192.181	13.9978
24572	4.1047	139.762	39.4901
24573	4.1048	171.552	325.005
24574	4.1048	229.711	115.385
24575	4.105	201.157	46.9175
24576	4.1053	132.998	38.4809
24577	4.1054	208.457	92.4057
24578	4.1055	176.926	64.2679
24579	4.1056	161.405	353.588
24580	4.1056	195.365	114.681
24581	4.1056	179.46	23.7181
24582	4.1058	204.211	80.7493
24583	4.1058	168.96	245.386
24584	4.106	208.627	244.817
24585	4.106	196.452	141.945
24586	4.1061	177.241	224.122
24587	4.1061	189.696	76.7057
24588	4.1062	188.328	174.715
24589	4.1062	224.653	99.6286
24590	4.1063	166.181	135.084
24591	4.1063	231.927	239.508
24592	4.1064	189.413	165.445
24593	4.1064	144.24	149.88
24594	4.1064	182.47	268.986
24595	4.1064	216.032	228.582
24596	4.1065	218.587	80.7215
24597	4.1065	183.145	148.159
24598	4.1066	187.465	39.2328
24599	4.1068	231.368	34.6386
24600	4.1069	154.821	201.772
24601	4.107	189.336	148.946
24602	4.1071	137.5	127.186
24603	4.1071	172.747	129.438
24604	4.1071	204.43	51.0084
24605	4.1073	192.836	322.934
24606	4.1074	186.012	237.743
24607	4.1077	171.38	112.501
24608	4.1077	224.586	125.942
24609	4.1078	155.287	261.83
24610	4.1078	198.246	230.651
24611	4.108	226.641	311.744
24612	4.1081	212.742	120.26
24613	4.1081	155.895	200.269

24614	4.1083	141.98	221.224
24615	4.1084	221.653	81.1766
24616	4.1084	171.325	209.895
24617	4.1084	176.992	216.881
24618	4.1084	195.52	96.4118
24619	4.1085	194.216	79.759
24620	4.1085	216.246	142.27
24621	4.1088	226.003	143.787
24622	4.1088	210.017	65.3098
24623	4.1088	148.235	235.065
24624	4.1089	167.239	353.261
24625	4.109	169.334	233.005
24626	4.109	183.301	20.544
24627	4.1091	186.638	154.978
24628	4.1091	208.678	14.1758
24629	4.1093	195.493	152.766
24630	4.1093	162.282	76.05
24631	4.1093	195.058	270.406
24632	4.1095	205.661	81.7162
24633	4.1095	218.021	99.5772
24634	4.1096	170.932	218.488
24635	4.1096	174.672	265.851
24636	4.1097	206.426	60.3009
24637	4.1097	169.525	329.707
24638	4.1098	172.414	87.8068
24639	4.11	182.399	353.877
24640	4.1101	202.026	289.293
24641	4.1101	203.444	174.306
24642	4.1103	183.568	65.8205
24643	4.1103	168.479	106.016
24644	4.1104	207.232	72.416
24645	4.1104	202.956	102.28
24646	4.1107	200.841	298.406
24647	4.1107	226.268	309.829
24648	4.1108	157.357	308.887
24649	4.1108	163.779	82.6435
24650	4.1109	181.981	218.45
24651	4.1109	178.281	149.361
24652	4.111	223.96	293.4
24653	4.1111	169.284	258.242
24654	4.1112	196.262	138.841
24655	4.1112	162.541	257.263
24656	4.1112	206.943	24.6701
24657	4.1114	175.934	262.518
24658	4.1115	214.874	199.084
24659	4.1115	176.29	229.203
24660	4.1116	173.608	141.927
24661	4.1116	194.406	95.7855

24662	4.1116	230.21	91.8348
24663	4.1117	164.271	160.327
24664	4.1118	155.138	143.365
24665	4.1119	218.905	343.188
24666	4.1119	189.063	171.825
24667	4.112	154.003	107.553
24668	4.1121	214.47	141.236
24669	4.1121	192.895	50.309
24670	4.1122	161.188	232.901
24671	4.1122	167.759	136.226
24672	4.1123	210.984	56.2579
24673	4.1126	117.342	178.819
24674	4.1126	232.466	117.938
24675	4.1127	206.096	67.735
24676	4.1129	165.877	187.103
24677	4.1129	187.667	299.766
24678	4.1129	219.126	265.964
24679	4.1129	199.663	280.631
24680	4.113	160.875	46.978
24681	4.1132	177.219	293.198
24682	4.1132	180.267	69.7766
24683	4.1132	191.826	322.052
24684	4.1135	216.776	43.6529
24685	4.1137	190.203	135.78
24686	4.1137	198.103	110.415
24687	4.1138	234.743	94.2887
24688	4.1138	200.284	121.804
24689	4.1139	104.957	221.101
24690	4.1139	-2.7051	9.5904
24691	4.1139	209.294	42.0798
24692	4.114	139.552	344.493
24693	4.114	195.885	167.072
24694	4.114	160.562	149.885
24695	4.1142	222.98	60.8347
24696	4.1142	209.62	156.295
24697	4.1143	187.456	213.437
24698	4.1144	168.932	96.385
24699	4.1145	194.21	309.384
24700	4.1145	154.108	295.181
24701	4.1146	195.302	107.572
24702	4.1147	233.871	229.909
24703	4.1148	200.287	110.911
24704	4.1148	158.452	250.295
24705	4.1149	200.763	225.216
24706	4.1149	134.906	252.657
24707	4.115	163.022	187.515
24708	4.1151	221.322	104.357
24709	4.1151	170.727	158.057

24710	4.1151	244.052	69.1896
24711	4.1154	199.438	170.097
24712	4.1154	149.685	124.782
24713	4.1155	234.599	225.943
24714	4.1156	157.92	72.0127
24715	4.1157	183.142	145.864
24716	4.1158	208.336	97.2151
24717	4.1158	206.177	30.8305
24718	4.1159	137.691	220.727
24719	4.1161	201.245	88.4077
24720	4.1161	197.631	129.81
24721	4.1161	173.343	74.1998
24722	4.1161	230.668	31.2729
24723	4.1162	213.606	112.633
24724	4.1162	159.742	318.561
24725	4.1162	195.501	74.9561
24726	4.1163	182.858	92.9662
24727	4.1163	174.126	159.71
24728	4.1164	212.713	73.291
24729	4.1165	192.244	96.8523
24730	4.1166	153.024	128.464
24731	4.1166	145.971	103.162
24732	4.1167	159.82	347.958
24733	4.1167	142.902	179.193
24734	4.1167	204.14	26.1365
24735	4.1168	175.894	322.949
24736	4.117	221.544	345.256
24737	4.117	177.785	146.003
24738	4.1171	227.392	28.4788
24739	4.1171	144.388	43.9331
24740	4.1173	156.681	168.208
24741	4.1174	197.581	108.034
24742	4.1175	207.024	52.0017
24743	4.1175	180.972	142.953
24744	4.1175	219.868	43.5299
24745	4.1177	161.673	213.893
24746	4.1177	168.387	221.311
24747	4.1178	185.951	34.8687
24748	4.1179	162.629	178.276
24749	4.1179	129.167	199.379
24750	4.118	169.273	30.9213
24751	4.118	204.8	111.467
24752	4.1182	188.457	17.5661
24753	4.1185	203.922	106.085
24754	4.1185	177.07	39.806
24755	4.1186	196.926	203.786
24756	4.1187	246.352	140.865
24757	4.1187	176.006	192.51

24758	4.1188	205.554	103.588
24759	4.1191	206.435	113.639
24760	4.1191	162.92	72.9355
24761	4.1191	127.579	199.953
24762	4.1191	150.972	132.926
24763	4.1192	215.304	201.058
24764	4.1194	180.949	98.4448
24765	4.1195	191.294	217.087
24766	4.1196	198.76	296.629
24767	4.1196	194.474	109.217
24768	4.1196	193.244	92.8092
24769	4.1196	170.12	86.433
24770	4.1199	233.972	52.0956
24771	4.1199	168.336	125.2
24772	4.1199	143.127	12.9376
24773	4.1199	202.677	239.831
24774	4.1202	199.847	172.848
24775	4.1202	220.475	57.5662
24776	4.1203	255.994	47.3956
24777	4.1203	227.207	355.925
24778	4.1203	164.668	128.46
24779	4.1203	218.933	132.217
24780	4.1205	180.721	109.671
24781	4.1206	213.257	304.532
24782	4.1207	207.963	190.961
24783	4.1207	171.915	114.786
24784	4.1209	197.942	148.426
24785	4.1209	213.194	72.7528
24786	4.1209	140.104	205.148
24787	4.121	230.044	275.532
24788	4.121	207.52	110.229
24789	4.1211	91.2264	28.5478
24790	4.1211	165.484	239.818
24791	4.1211	182.644	200.205
24792	4.1211	201.948	300.113
24793	4.1212	185.905	221.52
24794	4.1212	141.618	303.838
24795	4.1212	153.437	257.168
24796	4.1213	87.1023	13.7776
24797	4.1213	167.936	10.2837
24798	4.1213	199.272	88.6036
24799	4.1213	172.56	68.3508
24800	4.1214	-2.7618	6.4953
24801	4.1215	198.68	91.6011
24802	4.1217	175.175	169.384
24803	4.1218	186.055	158.58
24804	4.1224	255.405	66.6774
24805	4.1224	173.749	253.425

24806	4.1225	172.81	124.305
24807	4.1225	193.782	88.0966
24808	4.1226	225.634	153.965
24809	4.1227	183.078	50.5346
24810	4.1227	136.87	174.496
24811	4.1227	167.49	114.708
24812	4.1229	172.74	129.056
24813	4.1229	156.743	49.2466
24814	4.123	151.996	233.508
24815	4.123	167.086	140.668
24816	4.1231	176.513	50.903
24817	4.1234	30.3983	50.1003
24818	4.1236	213.339	114.899
24819	4.1237	192.531	143.639
24820	4.1238	169.698	15.4815
24821	4.1238	203.052	151.57
24822	4.1239	131.877	262.894
24823	4.1241	223.084	143.954
24824	4.1241	177.087	129.706
24825	4.1241	197.174	123.26
24826	4.1241	181.31	90.3329
24827	4.1242	237.879	273.568
24828	4.1242	181.415	116.14
24829	4.1243	164.966	129.016
24830	4.1245	162.504	182.528
24831	4.1246	212.218	175.33
24832	4.1247	95.3546	19.4846
24833	4.1248	185.517	195.757
24834	4.1248	192.414	123.112
24835	4.1248	163.484	35.9094
24836	4.1249	173.804	91.4312
24837	4.1249	176.412	201.643
24838	4.1249	149.766	134.701
24839	4.1251	210.758	45.9726
24840	4.1251	209.889	173.44
24841	4.1252	186.777	56.628
24842	4.1252	174.699	255.818
24843	4.1252	176.681	290.512
24844	4.1253	228.766	53.9432
24845	4.1253	207.423	157.962
24846	4.1254	178.101	251.851
24847	4.1254	184.869	173.475
24848	4.1256	202.255	170.235
24849	4.1256	130.798	103.493
24850	4.1258	257.264	88.884
24851	4.1259	198.667	97.7801
24852	4.126	183.671	220.089
24853	4.1261	201.537	110.314

24854	4.1263	212.585	124.449
24855	4.1263	175.354	38.469
24856	4.1264	158.923	164.308
24857	4.1264	178.886	259.097
24858	4.1265	204.479	161.46
24859	4.1265	203.51	41.8699
24860	4.1266	200.639	227.823
24861	4.1266	203.489	198.794
24862	4.1266	155.129	98.6459
24863	4.1266	236.841	245.747
24864	4.1267	173.262	288.15
24865	4.1267	240.119	210.148
24866	4.1267	159.463	279.349
24867	4.1268	171.108	204.408
24868	4.1268	138.914	213.73
24869	4.1268	238.822	38.4285
24870	4.1269	178.136	73.6496
24871	4.1269	183.957	105.004
24872	4.127	239.519	109.616
24873	4.127	169.513	147.75
24874	4.127	173.484	271.171
24875	4.1271	111.414	157.958
24876	4.1272	196.24	231.805
24877	4.1273	149.17	138.435
24878	4.1274	201.264	89.3573
24879	4.1274	151.824	118.655
24880	4.1275	202.013	190.782
24881	4.1275	197.237	104.352
24882	4.1276	237.68	77.5687
24883	4.1276	177.506	176.656
24884	4.1277	238.831	356.807
24885	4.1279	226.534	117.078
24886	4.1279	202.094	112.217
24887	4.128	205.337	338.168
24888	4.128	173.57	239.33
24889	4.128	212.049	168.114
24890	4.1281	190.985	309.81
24891	4.1281	196.269	299.866
24892	4.1282	112.429	151.68
24893	4.1284	195.724	316.524
24894	4.1284	189.428	97.2756
24895	4.1284	192.39	322.503
24896	4.1285	234.21	79.542
24897	4.1285	152.876	81.5829
24898	4.1285	177.361	149.101
24899	4.1286	173.198	95.6022
24900	4.1287	149.813	223.439
24901	4.1287	210.058	231.202

24902	4.1288	174.76	155.403
24903	4.1288	185.268	152.68
24904	4.1288	204.867	74.2396
24905	4.1289	248.05	55.7731
24906	4.129	230.193	40.8764
24907	4.129	285.766	309.425
24908	4.129	176.216	290.207
24909	4.129	166.016	199.305
24910	4.129	202.17	151.912
24911	4.1291	183.718	272.192
24912	4.1292	200.283	216.219
24913	4.1292	184.112	10.9425
24914	4.1294	188.287	134.95
24915	4.1294	162.727	36.3329
24916	4.1295	182.21	159.749
24917	4.1296	179.019	108.117
24918	4.1296	191.379	131.929
24919	4.1298	203.331	98.514
24920	4.1298	175.489	172.78
24921	4.1299	182.434	150.05
24922	4.1299	168.573	29.6879
24923	4.13	251.752	56.8928
24924	4.1302	188.739	167.51
24925	4.1302	178.925	341.361
24926	4.1302	216.449	65.8468
24927	4.1304	189.672	213.381
24928	4.1304	190.76	215.539
24929	4.1304	226.557	206.817
24930	4.1304	164.84	183.112
24931	4.1305	164.088	222.242
24932	4.1308	264.316	210.429
24933	4.1308	192.906	172.711
24934	4.1308	178.137	78.2262
24935	4.1308	249.043	359.203
24936	4.1309	170.68	291.411
24937	4.1309	202.044	162.195
24938	4.131	165.052	236.312
24939	4.131	162.979	97.0336
24940	4.1311	211.817	266.943
24941	4.1311	230.96	31.8095
24942	4.1315	190.044	120.928
24943	4.1318	190.299	108.072
24944	4.1319	187.545	116.626
24945	4.1319	193.756	58.4097
24946	4.132	186.285	143.183
24947	4.1321	194.854	235.133
24948	4.1321	188.199	117.887
24949	4.1321	161.681	38.5744

24950	4.1322	146.344	142.829
24951	4.1323	169.451	105.507
24952	4.1324	175.525	72.6271
24953	4.1324	243.528	180.547
24954	4.1324	183.863	54.6838
24955	4.1325	178.659	256.676
24956	4.1326	130.199	2.9923
24957	4.1326	230.917	117.944
24958	4.1327	228.405	352.542
24959	4.1327	210.624	53.5004
24960	4.1328	173.866	280.401
24961	4.1328	171.833	99.9638
24962	4.133	165.34	256.381
24963	4.133	202.492	126.564
24964	4.133	214.916	92.1927
24965	4.133	229.246	63.6166
24966	4.1331	190.276	85.406
24967	4.1331	138.614	206.903
24968	4.1332	185.669	156.949
24969	4.1333	178.19	136.305
24970	4.1333	198.39	269.508
24971	4.1335	255.651	58.5041
24972	4.1337	195.328	39.4271
24973	4.1337	134.187	122.746
24974	4.1338	222.219	228.877
24975	4.1338	173.2	217.507
24976	4.1339	169.768	91.9725
24977	4.1339	158.393	90.7284
24978	4.134	207.507	256.972
24979	4.1341	174.074	284.568
24980	4.1341	190.012	133.664
24981	4.1341	163.278	135.161
24982	4.1343	218.772	273.212
24983	4.1343	182.331	73.4403
24984	4.1344	195.998	174.794
24985	4.1345	163.097	271.638
24986	4.1346	215.407	274.815
24987	4.1346	192.684	227.735
24988	4.1346	189.781	129.589
24989	4.1346	170.356	269.698
24990	4.1347	204.851	60.3016
24991	4.1347	209.747	292.662
24992	4.1347	178.849	103.504
24993	4.1349	178.046	153.698
24994	4.1349	163.989	74.5712
24995	4.1349	165.825	146.98
24996	4.135	164.009	222.656
24997	4.1351	191.548	115.095

24998	4.1353	180.037	354.308
24999	4.1353	185.841	241.387
25000	4.1354	167.961	219.391
25001	4.1354	194.658	189.085
25002	4.1354	192.781	154.111
25003	4.1355	150.148	352.046
25004	4.1357	224.264	14.5037
25005	4.1357	172.371	70.4808
25006	4.1358	199.551	312.539
25007	4.1359	183.007	76.7561
25008	4.136	132.67	193.432
25009	4.136	156.925	140.118
25010	4.1362	199.292	345.989
25011	4.1363	175.712	139.255
25012	4.1363	166.9	105.985
25013	4.1364	191.065	256.81
25014	4.1367	193.076	94.9305
25015	4.1368	207.288	209.821
25016	4.1368	233.078	200.06
25017	4.137	164.768	132.102
25018	4.1372	174.02	158.111
25019	4.1373	217.84	100.132
25020	4.1375	191.758	242.34
25021	4.1376	175.095	162.552
25022	4.1378	214.05	307.006
25023	4.1378	262.513	22.9956
25024	4.1381	186.881	70.0818
25025	4.1382	199.867	303.711
25026	4.1382	196.162	63.0281
25027	4.1382	154.988	305.17
25028	4.1385	255.141	84.4878
25029	4.1385	148.98	45.2681
25030	4.1386	130.058	100.531
25031	4.1386	200.829	93.6105
25032	4.1387	149.223	312.913
25033	4.1388	193.043	198.83
25034	4.1388	163.141	130.866
25035	4.1389	140.284	326.12
25036	4.1392	190.848	163.965
25037	4.1392	214.12	1.6263
25038	4.1396	182.284	256.492
25039	4.1396	240.197	64.3681
25040	4.1396	189.536	63.4801
25041	4.1397	246.582	70.1297
25042	4.1397	150.811	78.6684
25043	4.1397	185.942	116.252
25044	4.1397	171.73	127.186
25045	4.1399	191.516	116.008

25046	4.1399	179.288	284.181
25047	4.14	178.353	222.593
25048	4.1401	214.482	49.6429
25049	4.1405	222.63	86.4792
25050	4.1408	231.502	322.819
25051	4.1411	197.378	115.604
25052	4.1411	195.454	115.153
25053	4.1411	203.674	270.969
25054	4.1412	208.569	335.679
25055	4.1413	220.265	100.249
25056	4.1414	-5.5105	22.169
25057	4.1415	189.122	295.178
25058	4.1415	157.882	117.888
25059	4.1415	192.091	170.12
25060	4.1416	208.908	132.327
25061	4.1417	194.465	29.5338
25062	4.1417	208.925	170.626
25063	4.1419	169.709	127.767
25064	4.142	220.489	209.472
25065	4.142	167.97	112.187
25066	4.142	167.07	249.415
25067	4.1423	189.215	251.236
25068	4.1424	184.372	306.236
25069	4.1424	177.031	89.4734
25070	4.1425	223.828	89.4261
25071	4.1425	212.4	103.052
25072	4.1425	182.238	26.9721
25073	4.1426	148.006	135.254
25074	4.1426	115.037	155.549
25075	4.1427	223.607	128.107
25076	4.1428	173.931	216.227
25077	4.1428	209.383	71.5839
25078	4.1429	211.475	80.6251
25079	4.1429	153.667	110.181
25080	4.1429	176.082	67.7805
25081	4.143	211.364	85.4458
25082	4.1432	165.638	191.369
25083	4.1435	182.463	157.813
25084	4.1436	203.436	67.2495
25085	4.1436	214.53	199.174
25086	4.1436	180.332	224.06
25087	4.1437	226.544	25.871
25088	4.1438	199.751	101.185
25089	4.144	171.126	215.942
25090	4.1442	171.195	66.5258
25091	4.1443	193.094	230.2
25092	4.1443	224.513	327.512
25093	4.1445	171.552	58.647

25094	4.1445	267.559	270.233
25095	4.1445	213.477	77.7797
25096	4.1445	-2.8145	121.448
25097	4.1445	163.274	168.549
25098	4.1446	256.765	62.3213
25099	4.1447	154.16	162.861
25100	4.1447	192.079	143.404
25101	4.1447	-2.7155	247.665
25102	4.1447	182.74	46.9385
25103	4.1447	184.89	67.3277
25104	4.1448	205.87	226.509
25105	4.1448	164.39	132.986
25106	4.1449	151.829	161.527
25107	4.145	207.454	99.5212
25108	4.145	188.563	104.995
25109	4.1451	159.98	163.83
25110	4.1451	197.519	204.541
25111	4.1451	167.008	155.671
25112	4.1452	255.701	45.0176
25113	4.1452	163.282	290.674
25114	4.1454	269.396	97.6268
25115	4.1455	207.339	102.973
25116	4.1456	168.458	112.658
25117	4.1458	235.455	120.642
25118	4.1459	224.257	348.056
25119	4.1459	164.349	229.563
25120	4.1461	142.918	109.867
25121	4.1461	206.266	152.281
25122	4.1464	251.557	40.4001
25123	4.1464	194.207	261.159
25124	4.1464	218.76	104.202
25125	4.1466	180.487	131.148
25126	4.1468	209.91	147.32
25127	4.1468	175.214	132.554
25128	4.1468	-2.9086	194.396
25129	4.1469	174.228	55.9654
25130	4.147	180.33	50.8412
25131	4.1471	208.77	48.303
25132	4.1472	220.199	139.726
25133	4.1472	194.396	104.052
25134	4.1472	203.287	68.3571
25135	4.1474	172.338	196.784
25136	4.1474	169.28	299.477
25137	4.1476	150.237	194.176
25138	4.1477	212.096	142.691
25139	4.1477	221.128	115.482
25140	4.1478	195.25	92.0313
25141	4.148	213.584	50.6951

25142	4.1481	186.714	31.6945
25143	4.1481	213.832	111.42
25144	4.1481	174.165	309.994
25145	4.1481	155.393	164.659
25146	4.1482	160.093	155.296
25147	4.1482	168.258	112.009
25148	4.1484	187.454	270.678
25149	4.1484	189.031	285.248
25150	4.1486	206.278	152.122
25151	4.1486	162.315	170.727
25152	4.1486	228.036	27.9101
25153	4.1487	195.591	359.203
25154	4.1487	165.404	138.399
25155	4.1487	193.216	162.316
25156	4.1487	216.615	153.724
25157	4.1488	214.73	39.8519
25158	4.1488	229.16	30.7438
25159	4.1489	245.998	120.442
25160	4.149	196.555	42.0528
25161	4.149	178.198	173.89
25162	4.1491	174.277	177.569
25163	4.1492	179.346	96.703
25164	4.1493	171.068	110.554
25165	4.1495	219.514	75.4811
25166	4.1496	228.523	119.743
25167	4.1496	199.339	110.478
25168	4.1497	189.646	225.423
25169	4.1497	189.053	33.3251
25170	4.1498	162.347	270.146
25171	4.1498	179.087	60.3929
25172	4.1498	188.842	146.118
25173	4.1498	200.441	288.073
25174	4.1499	214.079	200.645
25175	4.15	156.385	120.705
25176	4.15	156.324	222.037
25177	4.1501	191.476	261.485
25178	4.1501	170.229	184.697
25179	4.1501	188.205	132.89
25180	4.1504	195.508	191.73
25181	4.1504	240.982	21.9201
25182	4.1505	200.498	50.2396
25183	4.1506	216.005	69.2919
25184	4.1508	272.713	308.749
25185	4.1508	202.96	115.496
25186	4.1509	232.634	140.569
25187	4.151	143.395	270.42
25188	4.151	190.295	59.8821
25189	4.1511	204.158	40.1086

25190	4.1511	233.24	328.782
25191	4.1512	186.148	124.8
25192	4.1512	175.835	90.9603
25193	4.1513	186.801	134.109
25194	4.1513	136.805	192.082
25195	4.1513	209.2	154.026
25196	4.1513	207.129	273.002
25197	4.1516	204.163	254.564
25198	4.1516	223.671	66.6059
25199	4.1516	149.777	66.5114
25200	4.1517	213.296	281.96
25201	4.1517	225.699	297.762
25202	4.1518	160.22	10.7686
25203	4.1519	206.047	319.761
25204	4.152	242.035	104.711
25205	4.1522	218.844	214.587
25206	4.1523	135.081	220.948
25207	4.1523	-2.733	155.175
25208	4.1523	216.753	343.506
25209	4.1524	211.669	52.4144
25210	4.1525	229.781	96.2762
25211	4.1525	192.788	118.228
25212	4.1527	197.006	295.03
25213	4.1527	188.87	120.778
25214	4.1527	213.249	70.2785
25215	4.1528	152.323	283.178
25216	4.1529	164.762	247.73
25217	4.1531	203.343	161.638
25218	4.1532	159.341	117.659
25219	4.1532	197.67	110.244
25220	4.1533	195.472	281.633
25221	4.1535	225.562	115.416
25222	4.1535	198.12	82.1035
25223	4.1537	209.423	21.4569
25224	4.1537	129.188	133.153
25225	4.1537	182.622	199.319
25226	4.1537	148.531	112.295
25227	4.1538	245.283	42.3675
25228	4.1539	200.583	158.924
25229	4.154	188.484	90.7813
25230	4.1541	192.766	180.943
25231	4.1544	206.424	119.014
25232	4.1544	170.183	151.51
25233	4.1544	212.642	152.583
25234	4.1544	198.977	90.33
25235	4.1547	158.577	37.5408
25236	4.1547	221.176	84.1833
25237	4.1548	275.1	315.559

25238	4.1548	200.642	152.779
25239	4.155	187.39	110.831
25240	4.1551	171.457	212.192
25241	4.1552	242.425	57.8259
25242	4.1552	220.52	126.19
25243	4.1553	122.178	143.968
25244	4.1554	226.471	61.4263
25245	4.1554	198.565	128.64
25246	4.1554	239.238	36.3268
25247	4.1555	186.36	46.2897
25248	4.1555	210.025	292.397
25249	4.1556	205.203	186.636
25250	4.1556	153.581	126.378
25251	4.1558	225.712	84.2727
25252	4.1559	248.11	325.527
25253	4.156	239.273	119.139
25254	4.156	188.303	89.3766
25255	4.156	171.57	222.563
25256	4.156	245.492	100.268
25257	4.1561	205.7	115.435
25258	4.1562	217.475	292.034
25259	4.1564	192.615	109.645
25260	4.1565	113.22	168.593
25261	4.1568	203.471	175.585
25262	4.1569	243.598	261.941
25263	4.1572	251.242	83.7697
25264	4.1572	200.131	233.474
25265	4.1572	185.961	159.778
25266	4.1572	245.298	295.177
25267	4.1573	205.72	273.089
25268	4.1573	168.122	101.106
25269	4.1573	256.36	202.978
25270	4.1575	169.504	257.012
25271	4.1575	205.209	267.825
25272	4.1577	148.446	222.438
25273	4.1577	168.395	319.488
25274	4.1578	247.251	59.6858
25275	4.1579	182.234	147.864
25276	4.158	206.907	156.76
25277	4.1581	172.484	73.8917
25278	4.1582	179.92	186.353
25279	4.1583	183.337	171.142
25280	4.1583	233.941	179.763
25281	4.1586	138.292	82.26
25282	4.1587	217.71	299.16
25283	4.1588	-2.7059	254.561
25284	4.1591	243.462	95.0175
25285	4.1591	229.59	98.3001

25286	4.1591	211.723	16.015
25287	4.1593	244.348	27.3438
25288	4.1596	240.904	22.9414
25289	4.1596	191.264	188.519
25290	4.1596	181.336	169.815
25291	4.1596	194.494	122.103
25292	4.1596	256.066	310.462
25293	4.1597	245.218	149.157
25294	4.1597	173.864	222.265
25295	4.1597	224.673	45.2898
25296	4.1597	160.123	159.274
25297	4.1598	227.863	78.3467
25298	4.1598	232.671	80.2671
25299	4.16	226.284	77.8778
25300	4.1601	205.213	223.787
25301	4.1601	236.09	18.9927
25302	4.1603	233.772	154.995
25303	4.1603	181.333	38.6731
25304	4.1604	174.949	91.4439
25305	4.1604	253.357	203.356
25306	4.1605	159.892	63.4419
25307	4.1606	211.631	97.1198
25308	4.1607	160.969	173.248
25309	4.1608	212.081	182.76
25310	4.1608	192.561	170.969
25311	4.1609	223.753	110.689
25312	4.1611	221.282	22.7959
25313	4.1611	160.048	67.4015
25314	4.1612	190.469	212.125
25315	4.1612	197.804	141.678
25316	4.1613	280.372	140.051
25317	4.1613	146.389	239.907
25318	4.1614	231.298	81.6239
25319	4.1614	159.292	241.776
25320	4.1614	228.22	167.644
25321	4.1615	220.447	277.618
25322	4.1615	195.979	62.2112
25323	4.1617	166.514	63.1026
25324	4.1617	-2.6944	6.2334
25325	4.1617	207.811	135.081
25326	4.1617	243.834	88.5625
25327	4.1618	160.904	113.525
25328	4.1618	175.355	91.5902
25329	4.162	225.232	272.7
25330	4.162	221.305	17.8768
25331	4.1621	261.538	85.1918
25332	4.1621	186.927	173.236
25333	4.1621	195.861	130.364

25334	4.1621	170.04	74.6968
25335	4.1621	247.051	38.3057
25336	4.1622	171.691	295.886
25337	4.1623	199.224	158.035
25338	4.1624	200.336	33.2297
25339	4.1626	208.177	88.3862
25340	4.1628	264.793	203.952
25341	4.1632	186.948	30.664
25342	4.1632	206.387	90.6663
25343	4.1632	189.053	243.379
25344	4.1632	143.764	107.363
25345	4.1633	259.002	53.6575
25346	4.1635	190.368	64.0091
25347	4.1637	219.53	155.857
25348	4.1638	235.67	66.8428
25349	4.1639	208.163	69.8383
25350	4.1639	225.046	56.7224
25351	4.164	269.482	69.7111
25352	4.164	202.918	267.38
25353	4.1641	223.975	59.802
25354	4.1643	239.685	55.7158
25355	4.1643	206.784	255.135
25356	4.1644	198.468	77.042
25357	4.1645	174.094	111.114
25358	4.1646	201.395	24.7633
25359	4.1646	262.407	17.5527
25360	4.1646	281.097	330.189
25361	4.1647	228.81	104.52
25362	4.1647	172.652	43.3873
25363	4.165	217.73	343.365
25364	4.1651	209.996	110.756
25365	4.1652	181.419	127.184
25366	4.1654	170.595	186.677
25367	4.1654	182.293	113.997
25368	4.1655	237.913	216.865
25369	4.1656	169.595	251.257
25370	4.1656	153.114	198.192
25371	4.1657	184.45	243.193
25372	4.1658	198.31	176.342
25373	4.1659	189.605	109.6
25374	4.1659	234.814	151.595
25375	4.1659	249.388	311.743
25376	4.1659	167.328	191.01
25377	4.1659	166.411	334.387
25378	4.1661	210.542	116.236
25379	4.1663	206.569	40.2196
25380	4.1663	166.613	91.0616
25381	4.1665	163.611	317.512

25382	4.1665	182.188	303.568
25383	4.1665	190.216	51.0531
25384	4.1666	-0.5565	100.941
25385	4.1666	172.824	105.122
25386	4.1666	196.219	281.071
25387	4.1666	225.337	129.965
25388	4.1667	186.582	213.641
25389	4.1669	175.546	106.064
25390	4.167	207.313	59.1416
25391	4.1671	219.077	105.941
25392	4.1673	213.578	4.2711
25393	4.1674	178.542	114.912
25394	4.1675	221.311	269.193
25395	4.1675	153.524	165.922
25396	4.1675	148.641	284.119
25397	4.1679	221.167	97.2465
25398	4.1679	200.226	184.431
25399	4.168	169.269	191.612
25400	4.168	183.704	293.596
25401	4.1681	267.952	102.068
25402	4.1681	185.345	153.335
25403	4.1681	182.89	106.061
25404	4.1682	192.017	43.9865
25405	4.1683	202.438	154.729
25406	4.1684	178.934	196.963
25407	4.1684	218.443	334.69
25408	4.1685	201.365	152.292
25409	4.1687	183.625	255.064
25410	4.1687	187.766	110.718
25411	4.1688	177.516	321.438
25412	4.1688	201.789	120.271
25413	4.1689	193.606	161.386
25414	4.169	224.305	154.659
25415	4.169	188.293	121.094
25416	4.1691	175.756	236.975
25417	4.1692	184.72	277.489
25418	4.1692	180.305	124.454
25419	4.1693	197.276	103.457
25420	4.1693	188.228	112.212
25421	4.1693	188.648	31.0188
25422	4.1694	222.822	46.767
25423	4.1694	209.432	134.739
25424	4.1695	219.706	207.479
25425	4.1695	219.686	172.782
25426	4.1696	168.007	183.585
25427	4.1696	239.148	67.8915
25428	4.1697	208.905	234.863
25429	4.1698	247.067	89.5704

25430	4.17	187.38	266.68
25431	4.17	180.558	173.834
25432	4.17	195.086	288.66
25433	4.1701	265.019	307.443
25434	4.1701	174.876	130.31
25435	4.1702	193.946	228.499
25436	4.1702	171.671	94.4886
25437	4.1703	185.405	84.1765
25438	4.1705	187.125	178.09
25439	4.1707	311.119	281.44
25440	4.1707	185.984	201.518
25441	4.1709	182.618	198.264
25442	4.171	166.333	178.482
25443	4.1711	201.276	110.587
25444	4.1711	147.789	279.916
25445	4.1712	207.158	59.0652
25446	4.1714	-3.6132	119.157
25447	4.1715	177.378	20.0679
25448	4.1718	171.938	119.307
25449	4.1719	176.752	33.2112
25450	4.1719	234.92	289.909
25451	4.172	185.413	23.1056
25452	4.172	234.094	34.8473
25453	4.1723	219.979	152.082
25454	4.1725	213.472	80.8456
25455	4.1726	253.986	288.239
25456	4.1726	202.435	225.24
25457	4.1726	226.43	104.898
25458	4.1727	189.338	192.72
25459	4.1727	197.348	122.152
25460	4.1727	166.894	238.417
25461	4.1728	210.377	162.743
25462	4.1731	182.674	110.494
25463	4.1732	188.112	221.34
25464	4.1732	218.213	253.364
25465	4.1733	64.0216	118.362
25466	4.1733	201.535	197.496
25467	4.1734	236.879	66.2116
25468	4.1734	197.053	121.359
25469	4.1734	231.251	137.316
25470	4.1735	157.751	262.032
25471	4.1736	208.552	171.21
25472	4.1737	159.679	72.7929
25473	4.1737	239.511	222.426
25474	4.1739	179.743	118.07
25475	4.174	223.619	284.429
25476	4.1741	298.182	46.9394
25477	4.1741	179.713	146.775

25478	4.1741	-2.801	179.73
25479	4.1742	243.832	88.5712
25480	4.1742	187.128	36.9331
25481	4.1743	190.328	34.8234
25482	4.1743	185.366	54.203
25483	4.1743	198.565	199.239
25484	4.1745	209.276	138.094
25485	4.1746	189.821	115.263
25486	4.1748	186.456	255.531
25487	4.1748	239.822	272.874
25488	4.1749	198.972	141.621
25489	4.1749	183.266	226.379
25490	4.175	208.736	179.495
25491	4.1751	238.454	114.94
25492	4.1751	219.047	295.857
25493	4.1756	208.511	117.575
25494	4.1757	214.941	38.5076
25495	4.176	190.308	239.713
25496	4.1761	184.266	303.481
25497	4.1762	247.078	68.1264
25498	4.1762	156.626	224.046
25499	4.1763	212.803	253.65
25500	4.1765	224.157	135.377
25501	4.1766	228.597	108.585
25502	4.1766	218.997	259.424
25503	4.1767	199.032	130.203
25504	4.1767	217.523	241.191
25505	4.1768	200.691	130.462
25506	4.1768	161.196	118.034
25507	4.1769	225.682	153.097
25508	4.177	190.228	54.7474
25509	4.177	192.652	103.767
25510	4.177	173.85	17.1694
25511	4.1773	200.688	26.0679
25512	4.1776	196.827	110.212
25513	4.1776	207.764	24.5595
25514	4.1779	202.271	79.7055
25515	4.1779	188.528	137.074
25516	4.1784	265.756	39.6118
25517	4.1784	204.931	262.379
25518	4.1784	222.932	120.599
25519	4.1785	247.949	329.845
25520	4.1785	210.511	41.3056
25521	4.1786	230.569	83.6255
25522	4.1789	236.504	133.476
25523	4.1789	150.671	202.546
25524	4.179	159.572	100.328
25525	4.1792	209.891	251.667

25526	4.1792	201.615	62.387
25527	4.1795	-2.6898	1.3413
25528	4.1796	197.833	305.283
25529	4.1797	252.557	83.8771
25530	4.1797	203.383	193.947
25531	4.1797	224.328	244.529
25532	4.1797	179.086	36.9526
25533	4.18	98.8992	24.5091
25534	4.18	220.235	86.1481
25535	4.18	151.698	141.615
25536	4.1801	236.574	70.3604
25537	4.1801	190.674	106.426
25538	4.1803	182.361	66.7011
25539	4.1803	194.29	110.963
25540	4.1805	253.121	46.88
25541	4.1806	232.733	73.487
25542	4.1807	246.706	55.7455
25543	4.1807	160.8	219.256
25544	4.1807	205.503	114.497
25545	4.1807	187.107	141.204
25546	4.1807	189.85	229.196
25547	4.1808	233.467	138.753
25548	4.1808	156.17	232.524
25549	4.1809	223.029	172.703
25550	4.181	217.488	27.3019
25551	4.1811	201.556	110.84
25552	4.1811	229.342	178.273
25553	4.1812	236.354	66.1561
25554	4.1814	233.132	218.111
25555	4.1816	193.525	152.329
25556	4.1816	201.236	263.957
25557	4.1817	171.404	130.522
25558	4.1817	146.694	101.278
25559	4.1819	231.669	227.8
25560	4.1821	94.8508	25.9342
25561	4.1821	186.026	162.391
25562	4.1822	182.581	217.776
25563	4.1822	144.472	213.894
25564	4.1822	231.221	74.9243
25565	4.1823	206.365	80.9571
25566	4.1827	216.399	88.5733
25567	4.1828	178.234	136.058
25568	4.1828	161.596	268.499
25569	4.1828	215.177	157.071
25570	4.1829	200.72	123.918
25571	4.1829	192.239	91.2655
25572	4.1829	197.868	121.306
25573	4.183	202.222	38.2426

25574	4.183	150	104.81
25575	4.1832	217.235	24.2931
25576	4.1832	209.17	177.839
25577	4.1832	188.804	102.792
25578	4.1832	173.242	105.241
25579	4.1832	241.873	252.866
25580	4.1833	269.756	141.668
25581	4.1833	289.14	61.0633
25582	4.1833	220.438	186.804
25583	4.1833	167.549	302.544
25584	4.1834	236.957	162.272
25585	4.1836	174.894	308.073
25586	4.1837	158.746	120.773
25587	4.1838	210.457	253.12
25588	4.1838	201.9	65.2266
25589	4.1839	185.329	154.689
25590	4.184	206.747	216.33
25591	4.184	182.778	17.4967
25592	4.1841	177.582	18.9636
25593	4.1841	209.528	178.641
25594	4.1844	186.393	294.405
25595	4.1844	199.197	124.333
25596	4.1844	181.076	199.289
25597	4.1845	159.77	157.904
25598	4.1845	179.7	84.6535
25599	4.1845	209.752	156.448
25600	4.1845	239.172	179.185
25601	4.1846	189.792	112.841
25602	4.1849	230.845	155.776
25603	4.1849	168.799	64.8773
25604	4.185	194.638	3.0419
25605	4.185	171.101	110.392
25606	4.1852	232.691	76.0457
25607	4.1854	243.687	119.238
25608	4.1854	173.009	66.0481
25609	4.1855	232.741	329.483
25610	4.1855	161.485	351.632
25611	4.1858	234.738	359.992
25612	4.1858	237.714	81.1849
25613	4.1858	219.114	238.188
25614	4.1859	243.433	195.252
25615	4.1859	265.083	171.307
25616	4.186	296.3	289.485
25617	4.1861	220.309	109.729
25618	4.1861	208.707	259.715
25619	4.1861	193.061	186.903
25620	4.1861	190.768	151.25
25621	4.1863	171.343	47.4773

25622	4.1863	174.545	50.4834
25623	4.1863	188.556	103.965
25624	4.1863	216.044	49.6575
25625	4.1864	186.694	184.498
25626	4.1864	107.869	44.2703
25627	4.1864	199.751	148.092
25628	4.1865	160.388	228.073
25629	4.1866	175.743	111.15
25630	4.1867	253.916	81.941
25631	4.1867	200.558	333.511
25632	4.1868	163.782	85.8923
25633	4.1869	267.272	254.244
25634	4.187	224.255	302.264
25635	4.187	182.817	126.407
25636	4.187	230.577	77.142
25637	4.187	205.51	61.549
25638	4.187	247.409	160.256
25639	4.1871	167.093	204.41
25640	4.1873	219.959	247.022
25641	4.1873	197.68	141.022
25642	4.1874	255.113	82.375
25643	4.1875	208.512	134.567
25644	4.1878	189.427	113.609
25645	4.1879	212.964	118.034
25646	4.188	174.785	125.681
25647	4.1881	275.508	359.037
25648	4.1881	227.278	20.8052
25649	4.1883	186.835	149.347
25650	4.1884	244.816	325.561
25651	4.1885	209.706	181.589
25652	4.1886	174.386	58.8372
25653	4.189	232.488	77.7852
25654	4.1891	254.389	91.3559
25655	4.1891	253.802	145.956
25656	4.1892	197.669	131.661
25657	4.1892	186.434	341.67
25658	4.1895	192.389	193.161
25659	4.1896	210.78	121.971
25660	4.1896	244.614	33.045
25661	4.1897	212.16	212.637
25662	4.1897	241.477	82.4206
25663	4.1898	221.836	243.947
25664	4.19	223.306	185.359
25665	4.19	233.615	301.448
25666	4.1901	320.988	313.486
25667	4.1901	204.967	114.007
25668	4.1902	196.745	118.108
25669	4.1903	221.274	91.0305

25670	4.1904	182.27	179.267
25671	4.1904	236.394	178.318
25672	4.1904	280.72	37.3929
25673	4.1906	222.738	135.982
25674	4.1907	200.067	188.733
25675	4.1908	190.129	103.773
25676	4.1909	256.168	283.116
25677	4.1912	230.266	154.467
25678	4.1912	252.009	286.998
25679	4.1913	209.424	232.211
25680	4.1913	206.991	163.316
25681	4.1916	188.519	236.136
25682	4.1916	-2.6979	353.673
25683	4.1917	166.495	259.076
25684	4.1918	184.808	241.595
25685	4.1918	197.863	302.901
25686	4.192	227.3	88.2728
25687	4.192	174.687	107.819
25688	4.1922	210.377	88.9397
25689	4.1922	236.935	295.234
25690	4.1924	285.341	241.421
25691	4.1925	204.82	187.631
25692	4.1926	199.05	298.073
25693	4.1927	198.405	79.2711
25694	4.1927	306.415	312.189
25695	4.1927	177.111	133.196
25696	4.1927	132.991	185.481
25697	4.1927	154.401	206.416
25698	4.1928	191.023	250.933
25699	4.1928	215.321	134.012
25700	4.1928	254.657	289.039
25701	4.1929	205.1	80.9943
25702	4.1929	215.834	297.888
25703	4.1933	376.295	84.3083
25704	4.1933	234.995	179.121
25705	4.1934	245.107	68.316
25706	4.1936	218.163	115.636
25707	4.1937	230.227	312.323
25708	4.1937	193.593	125.258
25709	4.1938	193.401	119.845
25710	4.1938	223.095	320.051
25711	4.1939	234.731	69.0336
25712	4.194	194.783	127.376
25713	4.194	232.318	80.7635
25714	4.1941	272.306	91.1165
25715	4.1942	357.852	309.555
25716	4.1942	231.295	81.6088
25717	4.1943	232.422	277.268

25718	4.1943	222.041	1.2372
25719	4.1944	263.357	346.767
25720	4.1946	184.804	171.14
25721	4.1946	188.334	118.594
25722	4.1947	205.236	75.1884
25723	4.1949	240.379	91.1757
25724	4.1949	217.783	117.444
25725	4.195	216.559	26.7523
25726	4.1953	222.862	99.1407
25727	4.1954	169.028	167.698
25728	4.1955	215.689	42.2313
25729	4.1957	168.681	119.986
25730	4.1957	206.691	129.629
25731	4.1957	210.255	169.643
25732	4.1958	187.485	190.379
25733	4.1961	203.995	166.162
25734	4.1962	188.824	2.4094
25735	4.1962	224.859	37.6902
25736	4.1963	268.889	86.516
25737	4.1963	213.208	90.347
25738	4.1964	190.419	150.477
25739	4.1965	157.311	102.942
25740	4.1967	325.031	133.473
25741	4.1968	205.138	156.605
25742	4.1969	169.986	280.886
25743	4.197	257.048	121.218
25744	4.197	182.703	184.058
25745	4.1971	218.73	340.21
25746	4.1972	190.272	84.4922
25747	4.1972	206.275	174.595
25748	4.1974	250.5	119.8
25749	4.1974	256.054	71.8219
25750	4.1974	218.32	139.827
25751	4.1976	233.834	296.219
25752	4.1978	165.61	99.6594
25753	4.1978	217.415	142.975
25754	4.1979	184.553	123.31
25755	4.198	190.858	186.795
25756	4.198	170.227	142.266
25757	4.198	224.585	89.3459
25758	4.198	140.713	51.6223
25759	4.198	204.831	133.736
25760	4.1981	209.451	71.9437
25761	4.1981	231.709	77.2705
25762	4.1982	168.597	59.4611
25763	4.1983	273.451	46.0449
25764	4.1983	251.278	104.975
25765	4.1984	220.543	144.433

25766	4.1985	252.752	115.281
25767	4.1985	201.991	108.985
25768	4.1985	280.776	242.415
25769	4.1986	185.358	153.62
25770	4.1987	171.929	239.358
25771	4.1988	213.931	220.28
25772	4.1989	171.15	146.383
25773	4.1989	213.93	299.622
25774	4.199	161.185	116.236
25775	4.199	222.185	16.5916
25776	4.1991	259.588	37.7217
25777	4.1991	253.24	106.918
25778	4.1992	188.387	127.001
25779	4.1992	187.876	238.203
25780	4.1992	202.809	296.543
25781	4.1993	288.49	32.8833
25782	4.1993	166.418	23.6549
25783	4.1993	226.722	93.8293
25784	4.1994	205.58	172.586
25785	4.1995	213.291	169.74
25786	4.1997	169.454	144.83
25787	4.1998	223.39	161.489
25788	4.1998	205.869	120.807
25789	4.1999	176.862	48.8328
25790	4.1999	210.139	218.483
25791	4.1999	195.767	221.547
25792	4.2	219.005	248.972
25793	4.2	209.704	169.496
25794	4.2005	179.671	163.862
25795	4.2007	257.14	4.0318
25796	4.2007	178.48	178.451
25797	4.2008	176.21	214.995
25798	4.2009	255.972	53.2893
25799	4.201	224.494	269.89
25800	4.2012	131.793	57.9024
25801	4.2013	214.045	262.461
25802	4.2014	172.767	91.323
25803	4.2016	266.655	71.4449
25804	4.2017	136.958	170.241
25805	4.2017	188.897	96.0094
25806	4.2017	313.544	206.383
25807	4.2018	197.964	126.987
25808	4.2019	173.229	267.692
25809	4.2021	260.372	63.4883
25810	4.2021	201.091	124.976
25811	4.2023	224.857	188.296
25812	4.2024	232.841	120.931
25813	4.2024	194.29	2.5722

25814	4.2025	191.587	170.933
25815	4.2025	135.992	153.557
25816	4.2028	181.878	159.973
25817	4.2028	214.306	180.495
25818	4.2029	209.941	191.648
25819	4.2029	237.315	211.208
25820	4.203	257.599	120.56
25821	4.203	187.619	225.585
25822	4.2031	254.707	92.1273
25823	4.2033	211.448	231.566
25824	4.2035	248.011	49.489
25825	4.2036	241.15	6.9985
25826	4.2036	235.169	173.677
25827	4.2036	182.816	83.0436
25828	4.2036	202.161	110.001
25829	4.2037	219.55	99.6166
25830	4.2042	150.75	211.499
25831	4.2044	324.085	315.572
25832	4.2045	217.232	259.94
25833	4.2046	162.435	222.621
25834	4.2048	217.532	127.133
25835	4.2049	206.589	192.846
25836	4.2049	224.981	199.504
25837	4.2049	270.165	37.1138
25838	4.205	220.207	315.564
25839	4.205	220.349	290.71
25840	4.2051	181.083	50.0226
25841	4.2052	198.097	123.973
25842	4.2054	250.593	204.002
25843	4.2055	170.679	210.752
25844	4.2055	178.179	245.702
25845	4.2055	229.911	126.084
25846	4.2057	219.627	262.266
25847	4.2057	224.559	42.3199
25848	4.2058	197.245	83.9143
25849	4.2058	181.517	197.683
25850	4.206	276.674	43.8625
25851	4.2061	218.701	68.966
25852	4.2063	193.385	141.099
25853	4.2063	181.319	89.7157
25854	4.2064	156.914	180.492
25855	4.2065	202.29	196.468
25856	4.2065	176.675	252.006
25857	4.2067	246.551	351.636
25858	4.2068	200.492	160.452
25859	4.2069	199.79	256.929
25860	4.2069	179.005	87.0717
25861	4.2071	194.944	180.24

25862	4.2071	189.311	134.695
25863	4.2072	183.124	319.273
25864	4.2072	223.169	140.783
25865	4.2073	222.712	117.448
25866	4.2073	186.638	122.193
25867	4.2075	239.682	182.228
25868	4.2075	188.426	318.421
25869	4.2076	224.918	175.698
25870	4.2078	139.59	135.66
25871	4.2078	167.51	116.091
25872	4.2079	178.401	64.5528
25873	4.208	169.829	109.993
25874	4.208	150.421	165.728
25875	4.2081	194.787	113.817
25876	4.2081	181.974	72.3426
25877	4.2081	217.233	42.5372
25878	4.2081	168.403	160.718
25879	4.2082	130.526	186.981
25880	4.2083	192.026	94.2572
25881	4.2083	170.553	37.3517
25882	4.2084	197.309	95.6723
25883	4.2084	184.307	153.533
25884	4.2084	209.887	293.651
25885	4.2085	221.417	61.3478
25886	4.2087	161.115	129.565
25887	4.2087	213.002	19.6955
25888	4.2088	160.372	150.899
25889	4.209	255.103	92.8876
25890	4.2091	197.338	281.937
25891	4.2092	186.726	82.2757
25892	4.2093	226.021	184.699
25893	4.2094	162.136	349.101
25894	4.2095	193.848	4.9045
25895	4.2097	211.661	9.9867
25896	4.2097	137.353	295.847
25897	4.2098	209.281	143.517
25898	4.2099	206.437	181.479
25899	4.2099	188.386	159.401
25900	4.2099	271.243	71.2592
25901	4.21	234.351	84.7609
25902	4.2101	223.072	129.789
25903	4.2102	189.775	46.0997
25904	4.2103	217.001	304.486
25905	4.2105	215.436	85.9415
25906	4.2105	222.724	276.031
25907	4.2106	231.287	232.773
25908	4.2107	246.737	75.7358
25909	4.2107	211.892	256.365

25910	4.2108	208.834	303.16
25911	4.211	265.353	108.923
25912	4.211	238.572	62.3921
25913	4.211	168.796	100.92
25914	4.2111	225.135	208.764
25915	4.2111	165.358	187.736
25916	4.2112	183.322	78.911
25917	4.2113	182.621	102.487
25918	4.2114	227.896	119.693
25919	4.2116	188.747	276.455
25920	4.2117	251.177	85.4003
25921	4.2117	211.489	290.824
25922	4.2118	276.868	150.412
25923	4.2118	215.137	96.3641
25924	4.2118	257.551	40.1363
25925	4.2119	263.282	205.633
25926	4.212	228.667	233.102
25927	4.2122	181.883	198.322
25928	4.2122	192.089	97.4083
25929	4.2122	256.622	122.743
25930	4.2123	200.773	142.814
25931	4.2123	190.213	130.502
25932	4.2125	172.484	164.174
25933	4.2128	244.002	154.756
25934	4.213	238.904	67.1729
25935	4.2132	249.61	40.8092
25936	4.2135	242.116	134.516
25937	4.2136	223.509	148.89
25938	4.2136	275.966	79.6261
25939	4.2136	164.671	194.526
25940	4.2137	222.566	126.324
25941	4.2137	242.29	77.2482
25942	4.2138	162.6	223.862
25943	4.2138	248.767	75.5556
25944	4.2139	219.428	261.785
25945	4.214	209.8	345.571
25946	4.2142	227.705	118.623
25947	4.2143	234.441	318.113
25948	4.2143	228.123	218.298
25949	4.2143	219.154	125.411
25950	4.2144	153.875	125.389
25951	4.2144	278.875	100.979
25952	4.2146	290.941	29.6362
25953	4.2148	141.693	190.919
25954	4.215	184.726	126.119
25955	4.215	191.906	41.5034
25956	4.215	184.675	109.499
25957	4.2151	221.876	332.738

25958	4.2152	230.675	65.7703
25959	4.2153	211.025	147.88
25960	4.2154	175.464	28.2496
25961	4.2154	182.228	172.895
25962	4.2155	208.572	220.298
25963	4.2155	174.117	111.1
25964	4.2157	205.07	94.6758
25965	4.2158	196.402	317.793
25966	4.2159	243.631	169.704
25967	4.216	252.628	49.48
25968	4.2161	256.721	144.487
25969	4.2162	168.125	199.153
25970	4.2163	258.113	34.088
25971	4.2163	187.629	173.597
25972	4.2164	205.059	113.529
25973	4.2165	244.466	67.5474
25974	4.2166	183.766	75.8043
25975	4.2166	210.725	171.212
25976	4.2166	214.68	110.724
25977	4.2167	272.281	315.386
25978	4.2169	235.511	63.9992
25979	4.2169	174.621	90.0349
25980	4.217	185.677	85.9238
25981	4.2171	186.659	87.4973
25982	4.2171	240.266	74.5946
25983	4.2171	193.137	264.351
25984	4.2171	232.848	243.036
25985	4.2172	200.641	21.3693
25986	4.2172	207.831	23.9847
25987	4.2172	210.466	309.653
25988	4.2172	219.048	243.372
25989	4.2172	88.3548	41.963
25990	4.2173	216.353	279.511
25991	4.2175	278.949	16.3843
25992	4.2175	243.154	231.312
25993	4.2175	230.463	230.556
25994	4.2175	211.752	321.23
25995	4.2175	266.881	332.364
25996	4.2176	241.266	117.99
25997	4.2177	185.338	273.613
25998	4.2177	180.185	177.504
25999	4.2177	-2.7142	3.7337
26000	4.2177	236.28	322.722
26001	4.2178	172.08	298.445
26002	4.2178	164.102	156.995
26003	4.2179	180.119	228.722
26004	4.2179	214.136	66.7555
26005	4.2181	247.163	162.812

26006	4.2183	241.785	155.741
26007	4.2183	225.657	100.125
26008	4.2184	213.63	116.931
26009	4.2184	217.905	74.9928
26010	4.2184	161.791	166.154
26011	4.2184	242.872	9.7142
26012	4.2187	244.814	87.3562
26013	4.2187	219.665	120.528
26014	4.2188	201.981	143.486
26015	4.2189	231.352	214.889
26016	4.2189	255.834	287.594
26017	4.219	237.569	81.2426
26018	4.2191	222.958	55.064
26019	4.2191	248.167	322.433
26020	4.2194	210.581	61.4571
26021	4.2194	185.303	127.737
26022	4.2195	215.063	47.4924
26023	4.2196	286.879	50.1135
26024	4.2196	222.909	150.484
26025	4.2197	237.318	145.655
26026	4.2197	224.765	96.435
26027	4.2198	203.099	188.225
26028	4.2199	225.344	177.296
26029	4.2199	181.672	40.4537
26030	4.22	194.286	129.328
26031	4.22	180.974	150.845
26032	4.22	197.113	303.905
26033	4.2201	198.837	114.077
26034	4.2201	253.475	272.951
26035	4.2202	295.075	84.7456
26036	4.2202	194.62	187.048
26037	4.2202	188.442	175.007
26038	4.2202	241.909	154.823
26039	4.2203	180.755	155.824
26040	4.2203	157.256	83.2809
26041	4.2204	241.134	33.532
26042	4.2204	213.883	344.549
26043	4.2205	204.996	58.5121
26044	4.2205	288.706	241.722
26045	4.2206	254.862	88.5273
26046	4.2207	150.1	324.987
26047	4.2207	272.713	49.9903
26048	4.2208	212.989	95.1492
26049	4.2209	202.721	8.6856
26050	4.2209	211.51	55.6789
26051	4.2211	219.706	96.4807
26052	4.2211	227.931	105.228
26053	4.2212	211.199	53.5388

26054	4.2212	210.127	157.321
26055	4.2212	190.478	42.7462
26056	4.2212	239.64	278.858
26057	4.2213	203.495	232.544
26058	4.2215	170.046	187.603
26059	4.2216	185.817	196.735
26060	4.2216	186.009	165.403
26061	4.2217	247.962	99.7112
26062	4.2217	209.586	184.474
26063	4.2218	233.546	142.674
26064	4.2219	252.565	86.8129
26065	4.2219	213.265	217.989
26066	4.222	238.304	148.194
26067	4.2221	207.474	271.253
26068	4.2221	199.456	156.438
26069	4.2222	269.131	28.7685
26070	4.2222	157.531	167.052
26071	4.2222	281.403	315.696
26072	4.2223	137.874	167.6
26073	4.2223	116.37	199.418
26074	4.2223	249.594	166.043
26075	4.2225	219.714	183.566
26076	4.2225	182.671	143.997
26077	4.2225	200.004	7.9313
26078	4.2227	190.474	192.459
26079	4.2228	219.647	162.555
26080	4.2228	182.506	126.601
26081	4.2229	188.501	97.5045
26082	4.2229	222.121	289.867
26083	4.2229	223.87	288.796
26084	4.2229	202.899	166.228
26085	4.2231	240.371	210.263
26086	4.2233	174.089	26.2906
26087	4.2233	226.313	108.575
26088	4.2234	142.072	128.008
26089	4.2235	199.985	124.373
26090	4.2235	232.746	112.153
26091	4.2237	208.122	193.706
26092	4.2237	226.476	172.383
26093	4.2237	220.29	127.467
26094	4.2238	173.351	36.3078
26095	4.2239	208.013	151.046
26096	4.2239	199.568	25.52
26097	4.2241	253.447	150.186
26098	4.2242	213.041	235.177
26099	4.2242	248.122	310.155
26100	4.2243	174.195	191.282
26101	4.2243	288.415	46.9202

26102	4.2244	183.719	222.255
26103	4.2245	201.688	240.671
26104	4.2245	213.11	115.889
26105	4.2246	-4.0263	168.726
26106	4.2248	163.79	310.954
26107	4.2249	34.5677	206.87
26108	4.225	227.791	273.682
26109	4.225	153.201	251.496
26110	4.225	191.482	69.5241
26111	4.2254	217.038	318.742
26112	4.2254	208.739	41.8699
26113	4.2254	189.68	142.912
26114	4.2255	176.03	113.557
26115	4.2256	264.91	65.0388
26116	4.2256	163.944	179.369
26117	4.2256	275.916	56.1732
26118	4.2257	194.473	56.7799
26119	4.2259	299.992	64.5823
26120	4.226	201.432	266.006
26121	4.2262	228.219	94.7159
26122	4.2265	194.041	282.748
26123	4.2267	205.414	77.781
26124	4.2267	213.771	147.724
26125	4.2267	191.996	224.562
26126	4.2268	170.323	306.206
26127	4.2269	200.715	121.756
26128	4.227	247.161	80.4743
26129	4.2272	204.638	120.892
26130	4.2272	158.413	103.848
26131	4.2273	281.221	106.303
26132	4.2273	195.601	46.8612
26133	4.2273	184.429	117.96
26134	4.2273	237.57	290.147
26135	4.2274	224.707	63.8576
26136	4.2274	265.442	45.4167
26137	4.2275	185.437	253.384
26138	4.2275	229.865	23.0923
26139	4.2275	221.471	134.981
26140	4.2276	252.55	78.395
26141	4.2276	223.419	345.847
26142	4.2277	249.282	216.635
26143	4.2277	186.055	358.649
26144	4.2279	235.953	82.4487
26145	4.228	194.071	248.776
26146	4.228	197.305	107.691
26147	4.2281	270.617	328.977
26148	4.2282	234.792	12.2433
26149	4.2282	266.664	144.418

26150	4.2283	179.138	172.46
26151	4.2283	203.14	191.551
26152	4.2284	191.061	164.213
26153	4.2285	205.1	113.69
26154	4.2286	156.536	164.44
26155	4.2287	137.371	192.498
26156	4.2287	247.053	19.6498
26157	4.2288	208.453	53.0218
26158	4.2289	164.606	115.294
26159	4.2289	194.677	199.613
26160	4.2289	167.536	140.783
26161	4.229	201.468	163.354
26162	4.2291	248.166	124.93
26163	4.2292	202.911	72.9715
26164	4.2292	185.063	277.003
26165	4.2295	226.127	80.7488
26166	4.2295	211.086	124.051
26167	4.2296	194.991	64.2676
26168	4.2299	177.819	244.685
26169	4.2299	204.628	127.03
26170	4.2299	173.366	100.128
26171	4.2299	217.645	25.6689
26172	4.2299	-2.7059	251.451
26173	4.2301	232.807	119.439
26174	4.2301	225.446	104.567
26175	4.2304	242.756	316.517
26176	4.2305	251.594	196.023
26177	4.2307	216.149	161.801
26178	4.2307	262.963	71.8855
26179	4.2309	205.309	142.079
26180	4.2312	247.722	113.02
26181	4.2314	237.192	32.885
26182	4.2314	188.684	66.8576
26183	4.2314	244.893	297.942
26184	4.2315	188.42	108.251
26185	4.2315	213.65	44.0797
26186	4.2316	245.864	71.6234
26187	4.2316	225.809	78.5192
26188	4.2316	171.723	124.483
26189	4.2316	196.932	61.9362
26190	4.2318	274.684	42.9846
26191	4.2319	179.568	36.8008
26192	4.2319	213.456	292.189
26193	4.2319	212.128	306.515
26194	4.2319	195.68	6.1726
26195	4.2319	244.871	121.972
26196	4.232	232.524	279.473
26197	4.232	164.615	99.3659

26198	4.232	223.878	107.409
26199	4.2321	198.078	319.557
26200	4.2323	228.75	184.716
26201	4.2323	254.421	84.2435
26202	4.2323	198.305	137.494
26203	4.2323	184.043	67.0151
26204	4.2324	201.047	151.449
26205	4.2327	211.171	178.772
26206	4.2327	225.271	122.39
26207	4.2328	240.281	275.512
26208	4.2329	196.957	255.803
26209	4.2329	234.07	150.031
26210	4.233	221.047	34.1911
26211	4.2331	247.643	23.6727
26212	4.2332	250.426	64.0649
26213	4.2332	253.005	43.9106
26214	4.2333	217.781	85.2717
26215	4.2333	211.244	114.515
26216	4.2334	160.726	129.091
26217	4.2335	180.696	120.306
26218	4.2335	191.212	252.508
26219	4.2335	185.868	129.69
26220	4.2336	188.852	35.8497
26221	4.2336	186.938	155.286
26222	4.2337	219.224	201.721
26223	4.2339	250.568	40.1718
26224	4.2339	196.989	52.3638
26225	4.2339	220.044	109.945
26226	4.2339	247.181	62.4123
26227	4.234	244.294	73.9536
26228	4.234	222.17	316.988
26229	4.234	224.208	49.3379
26230	4.2341	211.38	65.0568
26231	4.2343	249.827	33.1528
26232	4.2343	255.23	123.673
26233	4.2343	214.204	62.2698
26234	4.2344	210.044	102.902
26235	4.2344	209.387	105.069
26236	4.2345	212.709	171.579
26237	4.2347	237.621	75.2711
26238	4.2347	203.279	302.218
26239	4.2347	163.68	87.2954
26240	4.2348	287.063	201.77
26241	4.2348	196.071	348.705
26242	4.235	231.027	123.542
26243	4.2351	221.216	174.653
26244	4.2351	187.945	176.224
26245	4.2351	237.031	150.884

26246	4.2352	198.729	281.074
26247	4.2353	243.196	255.236
26248	4.2354	261.808	31.1497
26249	4.2354	199.425	221.693
26250	4.2355	245.192	102.713
26251	4.2357	273.574	83.7747
26252	4.2358	211.725	22.3255
26253	4.236	242.24	57.8173
26254	4.236	262.683	62.7589
26255	4.2361	209.752	111.246
26256	4.2362	266.588	19.3218
26257	4.2362	243.858	49.3826
26258	4.2362	199.872	183.644
26259	4.2362	175.51	198.329
26260	4.2366	189.423	28.7201
26261	4.2369	211.468	150.332
26262	4.2369	247.179	350.281
26263	4.237	234.394	34.9037
26264	4.237	156.244	249.537
26265	4.237	260.761	159.661
26266	4.2371	174.363	189.495
26267	4.2372	184.313	99.5085
26268	4.2373	188.321	251.824
26269	4.2374	207.332	191.998
26270	4.2374	-3.891	121.434
26271	4.2376	166.368	115.428
26272	4.2378	187.957	126.862
26273	4.2379	242.608	307.902
26274	4.2379	201.322	294.154
26275	4.2379	262.816	218.276
26276	4.238	198.939	70.4192
26277	4.2381	217.93	109.753
26278	4.2382	253.469	334.135
26279	4.2382	212.049	131.574
26280	4.2385	224.401	192.555
26281	4.2385	192.659	216.159
26282	4.2386	273.79	312.396
26283	4.2386	215.744	93.82
26284	4.2389	221.878	171.534
26285	4.2389	215.316	282.876
26286	4.239	227.745	106.192
26287	4.239	245.168	74.613
26288	4.2391	217.962	82.7412
26289	4.2392	238.055	86.0219
26290	4.2392	181.572	41.7512
26291	4.2393	289.191	8.2863
26292	4.2393	256.452	278.116
26293	4.2394	127.07	73.6459

26294	4.2395	194.207	134.934
26295	4.2396	205.915	330.113
26296	4.2397	179.386	140.59
26297	4.2398	193.623	109.447
26298	4.24	186.696	160.018
26299	4.24	218.201	102.227
26300	4.2401	291.052	14.9419
26301	4.2402	294.528	309.768
26302	4.2402	229.184	153.235
26303	4.2402	176.189	165.55
26304	4.2402	215.142	117.643
26305	4.2402	214.953	106.556
26306	4.2403	243.607	290.029
26307	4.2403	191.352	188.602
26308	4.2403	264.513	64.355
26309	4.2405	202.752	132.83
26310	4.2405	166.92	75.8523
26311	4.2406	191.718	178.146
26312	4.2406	180.586	101.381
26313	4.2406	238.128	104.624
26314	4.2406	197.355	349.709
26315	4.2406	252.376	290.248
26316	4.2407	226.784	269.754
26317	4.2407	224.381	174.287
26318	4.2407	241.624	55.6323
26319	4.2407	225.911	168.241
26320	4.2407	209.779	295.11
26321	4.2408	207.266	127.688
26322	4.241	190.089	156.339
26323	4.2411	197.962	222.643
26324	4.2412	273.57	43.8042
26325	4.2414	229.951	137.497
26326	4.2416	209.387	76.864
26327	4.2417	196.799	17.5761
26328	4.2418	247.864	46.3628
26329	4.2418	249.508	163.684
26330	4.2419	106.586	114.39
26331	4.242	244.302	346.425
26332	4.2421	235.177	113.539
26333	4.2422	216.888	128.973
26334	4.2422	251.551	152.008
26335	4.2423	221.18	60.0364
26336	4.2423	200.148	117.568
26337	4.2423	224.624	256.059
26338	4.2424	162.609	262.658
26339	4.2425	239.289	160.35
26340	4.2427	178.575	195.913
26341	4.2427	214.786	44.8388

26342	4.2428	256.665	206.943
26343	4.243	211.195	108.49
26344	4.2431	266.557	356.229
26345	4.2431	203.535	300.768
26346	4.2432	223.431	133.88
26347	4.2433	218.595	62.0265
26348	4.2435	209.11	300.727
26349	4.2436	210.473	90.502
26350	4.2437	192.128	128.511
26351	4.244	222.634	163.36
26352	4.2441	187.947	0.5451
26353	4.2442	233.612	127.799
26354	4.2443	210.442	157.197
26355	4.2443	211.382	342.531
26356	4.2444	198.176	160.313
26357	4.2446	242.554	57.8243
26358	4.2447	227.064	163.544
26359	4.2448	219.29	168.56
26360	4.2448	215.289	245.433
26361	4.2449	276.18	27.43
26362	4.2449	259.246	348.249
26363	4.2451	224.92	101.842
26364	4.2452	206.051	278.559
26365	4.2452	213.394	135.558
26366	4.2453	180.038	265.693
26367	4.2453	257.457	296.368
26368	4.2453	314.406	287.858
26369	4.2453	228.261	60.2701
26370	4.2454	175.075	307.215
26371	4.2455	236.918	163.786
26372	4.2456	226.562	118.129
26373	4.2456	184.816	34.8628
26374	4.2456	140.345	21.4326
26375	4.2456	225.919	168.233
26376	4.2457	273.976	61.2064
26377	4.2457	203.959	125.253
26378	4.2459	321.451	310.67
26379	4.246	207.412	147.734
26380	4.246	208.718	338.985
26381	4.2461	199.894	130.279
26382	4.2464	183.248	45.2342
26383	4.2465	194.964	98.9854
26384	4.2465	239.662	305.422
26385	4.2466	240.574	22.6003
26386	4.2467	193.441	157.668
26387	4.2468	279.324	38.8438
26388	4.2469	323.425	207.336
26389	4.2469	209.698	117.395

26390	4.2471	232.355	221.067
26391	4.2471	199.939	75.3258
26392	4.2472	263.032	117.895
26393	4.2473	212.123	265.721
26394	4.2473	185.451	165.015
26395	4.2475	226.417	102.536
26396	4.2475	183.028	262.525
26397	4.2476	202.691	128.333
26398	4.2478	111.608	41.5507
26399	4.2481	106.464	352.744
26400	4.2483	168.541	49.9313
26401	4.2484	210.329	269.007
26402	4.2486	193.709	146.997
26403	4.2487	-2.7911	49.2253
26404	4.2488	236.942	149.8
26405	4.2488	-2.8353	198.398
26406	4.2491	166.578	124.961
26407	4.2493	278.646	93.1234
26408	4.2494	199.997	238.007
26409	4.2494	212.694	172.234
26410	4.2494	227.204	16.3693
26411	4.2494	239.727	120.56
26412	4.2495	318.949	311.849
26413	4.2495	228.088	56.8483
26414	4.2497	220.059	306.501
26415	4.2497	162.946	281.293
26416	4.2498	295.698	1.0865
26417	4.2498	227.242	48.2918
26418	4.2501	259.125	49.7838
26419	4.2502	231.215	216.74
26420	4.2503	200.692	134.033
26421	4.2504	169.48	340.462
26422	4.2504	191.71	129.374
26423	4.2504	214.412	106.716
26424	4.2505	203.821	117.448
26425	4.2505	234.645	78.9061
26426	4.2507	220.441	136.915
26427	4.2509	194.612	152.05
26428	4.2512	253.15	145.01
26429	4.2513	176.253	166.449
26430	4.2513	232.119	105.988
26431	4.2513	194.652	94.3114
26432	4.2515	208.481	109.434
26433	4.2516	206.783	240.938
26434	4.2517	185.082	34.9887
26435	4.2517	251.92	62.1743
26436	4.2517	195.525	299.221
26437	4.2519	268.018	241.856

26438	4.2519	230.945	274.328
26439	4.252	256.734	138.172
26440	4.2522	201.278	114.861
26441	4.2523	212.613	229.43
26442	4.2523	237.375	268.9
26443	4.2524	254.539	44.176
26444	4.2524	183.745	91.3527
26445	4.2525	236.887	257.534
26446	4.2525	168.572	240.656
26447	4.2525	178.974	67.7617
26448	4.2526	239.612	138.32
26449	4.2527	217.228	305.916
26450	4.2527	192.985	54.4353
26451	4.2531	252.759	237.555
26452	4.2532	114.161	99.9214
26453	4.2534	271.492	94.5974
26454	4.2534	228.819	279.273
26455	4.2537	182.064	85.6561
26456	4.2539	249.581	355.848
26457	4.2541	169.649	165.262
26458	4.2542	223.485	60.7488
26459	4.2542	252.821	282.821
26460	4.2543	215.71	216.284
26461	4.2543	232.243	83.2216
26462	4.2545	262.479	181.159
26463	4.2545	286.722	57.8563
26464	4.2547	217.785	269.181
26465	4.2548	261.077	106.59
26466	4.2549	220.927	172.057
26467	4.255	224.676	218.437
26468	4.2551	198.692	128.927
26469	4.2552	224.367	119.681
26470	4.2553	211.259	36.4172
26471	4.2555	152.752	258.376
26472	4.2556	243.612	17.4455
26473	4.2557	180.299	130.675
26474	4.2559	210.899	271.299
26475	4.2561	278.458	310.132
26476	4.2562	193.243	75.9624
26477	4.2562	197.356	73.532
26478	4.2563	161.805	314.363
26479	4.2564	231.057	271.983
26480	4.2565	218.423	196.788
26481	4.2571	197.041	261.12
26482	4.2575	181.503	231.299
26483	4.2575	189.144	32.5767
26484	4.2575	200.258	161.041
26485	4.2577	194.062	120.614

26486	4.2578	245.036	272.992
26487	4.2579	175.916	130.878
26488	4.2579	327.501	28.258
26489	4.2581	203.273	192.55
26490	4.2582	273.868	232.503
26491	4.2582	192.623	83.0541
26492	4.2583	219.531	125.564
26493	4.2585	215.878	156.545
26494	4.2586	193.636	220.964
26495	4.2586	226.905	130.165
26496	4.2587	209.215	109.677
26497	4.2588	246.478	262.631
26498	4.2588	228.874	272.454
26499	4.2588	270.859	309.089
26500	4.259	245.138	283.053
26501	4.2592	287.056	83.5576
26502	4.2592	245.905	27.9576
26503	4.2592	263.337	349.45
26504	4.2593	247.021	319.875
26505	4.2593	203.822	255.417
26506	4.2594	173.998	189.568
26507	4.2594	183.065	115.007
26508	4.2597	196.898	305.347
26509	4.2597	242.449	63.4591
26510	4.2598	120.456	39.2872
26511	4.2599	231.136	175.877
26512	4.2599	238.259	295.513
26513	4.26	229.082	158.286
26514	4.26	221.487	33.4976
26515	4.2603	232.259	193.186
26516	4.2603	217.422	90.2516
26517	4.2604	228.367	131.254
26518	4.2604	171.852	139.831
26519	4.2605	156.682	292.284
26520	4.2605	218.416	134.403
26521	4.2605	176.771	153.83
26522	4.2605	221.014	119.618
26523	4.2605	224.647	75.9793
26524	4.2607	283.863	316.116
26525	4.2607	225.016	281.555
26526	4.2607	192.57	83.5406
26527	4.2608	226.281	148.224
26528	4.2608	176.385	259.291
26529	4.2609	215.98	170.301
26530	4.2609	243.962	86.7474
26531	4.261	197.788	197.436
26532	4.2612	217.135	134.421
26533	4.2613	235.432	235.379

26534	4.2613	182.617	107.107
26535	4.2614	263.685	309.77
26536	4.2614	207.954	84.9008
26537	4.2615	243.123	281.149
26538	4.2616	174.719	33.7831
26539	4.2617	229.303	38.5594
26540	4.2618	200.691	18.1593
26541	4.2619	242.936	190.961
26542	4.2619	132.319	207.189
26543	4.2619	171.942	151.548
26544	4.2619	251.404	288.156
26545	4.262	177.481	297.084
26546	4.2621	187.563	130.853
26547	4.2624	251.681	58.9381
26548	4.2624	-2.7903	100.334
26549	4.2627	-2.751	156.652
26550	4.263	226.221	275.553
26551	4.263	173.551	225.914
26552	4.2631	269.071	302.212
26553	4.2632	260.937	212.802
26554	4.2632	237.665	321.183
26555	4.2636	265.973	236.446
26556	4.2636	213.329	69.2635
26557	4.2636	255.73	45.4492
26558	4.2637	203.117	19.7925
26559	4.2639	247.493	293.11
26560	4.2639	179.837	186.118
26561	4.2642	251.11	27.4376
26562	4.2643	245.631	354.485
26563	4.2643	185.68	83.2582
26564	4.2643	203.112	298.747
26565	4.2646	209.134	121.925
26566	4.2647	204.651	192.776
26567	4.2649	235.077	117.922
26568	4.2649	164.73	248.926
26569	4.2649	248.17	62.0171
26570	4.265	281.299	261.631
26571	4.2653	233.125	57.942
26572	4.2654	204.621	250.737
26573	4.2656	238.548	184.415
26574	4.2656	224.299	309.358
26575	4.2657	216.475	168.073
26576	4.2658	169.153	211.346
26577	4.266	245.715	88.8749
26578	4.266	224.628	294.765
26579	4.2661	214.633	135.012
26580	4.2661	198.141	270.492
26581	4.2661	260.113	314.751

26582	4.2661	247.665	157.961
26583	4.2663	238.935	131.608
26584	4.2664	274.226	280.967
26585	4.2664	191.032	107.048
26586	4.2664	161.315	278.83
26587	4.2667	183.259	98.21
26588	4.2667	167.795	21.3624
26589	4.2668	207.043	301.414
26590	4.2668	231.432	289.156
26591	4.2668	240.962	107.525
26592	4.267	233.431	129.729
26593	4.267	207.644	121.064
26594	4.2671	257.823	316.921
26595	4.2671	201.586	113.182
26596	4.2672	207.076	95.8004
26597	4.2672	205.771	156.475
26598	4.2673	214.705	118.562
26599	4.2673	173.891	81.8216
26600	4.2674	231.238	64.391
26601	4.2675	255.844	87.9884
26602	4.2676	166.628	103.149
26603	4.2676	187.495	230.385
26604	4.2677	195.833	258.781
26605	4.2677	219.976	138.59
26606	4.2677	213.376	52.5955
26607	4.2677	206.005	158.324
26608	4.2678	191.157	53.8664
26609	4.2678	196.906	130.079
26610	4.2679	186.851	154.9
26611	4.268	235.146	128.543
26612	4.2682	240.369	120.856
26613	4.2683	249.16	76.6034
26614	4.2684	268.044	152.881
26615	4.2686	204.037	123.24
26616	4.2687	234.737	61.5959
26617	4.2687	219.851	20.213
26618	4.2688	184.223	158.426
26619	4.2689	204.506	64.3084
26620	4.2689	264.691	47.3047
26621	4.2689	221.532	56.1357
26622	4.2691	224.968	27.8486
26623	4.2692	195.728	124.333
26624	4.2693	265.499	61.489
26625	4.2694	219.752	91.3541
26626	4.2695	244.998	300.76
26627	4.2695	224.204	126.468
26628	4.2696	216.649	156.603
26629	4.2696	203.099	183.581

26630	4.2696	228.864	173.977
26631	4.2697	286.844	88.5978
26632	4.2697	209.935	48.9222
26633	4.2698	232.512	308.971
26634	4.2699	208.274	251.733
26635	4.2699	196.073	165.655
26636	4.27	251.457	63.6484
26637	4.27	196.479	270.676
26638	4.2702	191.503	162.997
26639	4.2703	221.593	189
26640	4.2704	226.229	33.877
26641	4.2705	225.458	175.134
26642	4.2706	241.626	67.3491
26643	4.2706	195.424	51.1534
26644	4.2707	151.389	126.158
26645	4.2708	273.958	25.5313
26646	4.2709	271.242	53.3976
26647	4.271	171.595	76.7283
26648	4.271	232.672	105.662
26649	4.2713	204.911	240.422
26650	4.2713	212.4	315.705
26651	4.2714	179.564	100.563
26652	4.2716	282.965	35.9211
26653	4.2717	196.139	167.496
26654	4.2718	254.024	149.571
26655	4.2718	222.233	99.5193
26656	4.2719	196.475	86.4955
26657	4.272	241.71	290.243
26658	4.272	229.092	33.3834
26659	4.272	241.78	315.304
26660	4.272	273.106	91.3214
26661	4.2721	192.162	294.688
26662	4.2722	214.566	326.632
26663	4.2722	182.315	164.759
26664	4.2723	216.109	85.9662
26665	4.2724	291.764	63.2653
26666	4.2725	217.751	145.174
26667	4.2725	185.387	131.103
26668	4.2727	119.988	25.4799
26669	4.2727	225.407	155.925
26670	4.2729	201.885	179.529
26671	4.2729	180.635	129.998
26672	4.2733	277.222	304.586
26673	4.2733	197.49	150.399
26674	4.2734	198.985	203.738
26675	4.2734	218.726	142.479
26676	4.2735	197.855	337.034
26677	4.2736	204.333	163.988

26678	4.2737	124.897	189.123
26679	4.274	212.248	103.275
26680	4.2741	262.035	133.718
26681	4.2741	212.751	46.257
26682	4.2741	219.17	296.376
26683	4.2742	301.109	19.2079
26684	4.2742	179.457	158.569
26685	4.2742	211.043	17.1398
26686	4.2742	227.312	150.687
26687	4.2743	262.879	197.895
26688	4.2743	248.759	159.932
26689	4.2744	160.029	137.007
26690	4.2744	253.753	321.583
26691	4.2745	221.491	44.5982
26692	4.2745	212.889	11.9165
26693	4.2745	244.902	290.949
26694	4.2745	289.721	49.0115
26695	4.2746	249.835	66.5636
26696	4.2747	216.566	94.2042
26697	4.2747	248.583	121.296
26698	4.2748	176.649	278.489
26699	4.2749	272.564	71.2356
26700	4.275	-2.8136	193.947
26701	4.275	225.512	53.4328
26702	4.2752	207.893	68.739
26703	4.2752	221.2	298.488
26704	4.2753	228.032	58.4113
26705	4.2753	186.126	72.5971
26706	4.2753	190.052	225.549
26707	4.2753	224.383	290.555
26708	4.2754	237.788	342.792
26709	4.2755	273.599	14.3696
26710	4.2755	238.335	173.18
26711	4.2757	239.901	179.517
26712	4.2757	208.718	124.69
26713	4.2758	216.964	169.345
26714	4.2759	247.942	107.212
26715	4.2759	200.301	98.7884
26716	4.276	186.345	46.1606
26717	4.2761	156.624	353.323
26718	4.2761	202.335	44.5403
26719	4.2762	230.411	35.929
26720	4.2762	247.118	321.902
26721	4.2763	207.894	269.897
26722	4.2763	215.982	165.144
26723	4.2764	187.027	197.058
26724	4.2765	109.992	41.7934
26725	4.2765	250.458	192.575

26726	4.2765	257.15	310.824
26727	4.2766	246.272	290.757
26728	4.2766	214.499	121.4
26729	4.2767	187.801	265.339
26730	4.2769	311.71	23.0293
26731	4.277	175.019	160.803
26732	4.2771	188.047	100.617
26733	4.2771	169.625	105.43
26734	4.2771	214.651	51.2723
26735	4.2772	215.959	142.155
26736	4.2772	236.193	251.049
26737	4.2773	218.711	264.064
26738	4.2774	265.541	236.956
26739	4.2775	213.646	162.937
26740	4.2777	217.023	123.178
26741	4.2777	247.617	63.4249
26742	4.2778	201.655	150.824
26743	4.2778	185.327	139.183
26744	4.2779	255.631	197.918
26745	4.2779	229.252	128.549
26746	4.2779	237.358	160.929
26747	4.278	194.476	85.845
26748	4.2782	190.465	28.0763
26749	4.2783	213.33	189.711
26750	4.2783	246.77	36.8264
26751	4.2783	182.67	359.532
26752	4.2783	270.72	35.4415
26753	4.2784	243.107	56.5628
26754	4.2786	208.013	275.846
26755	4.2786	195.616	180.698
26756	4.2787	218.727	192.707
26757	4.2789	223.326	107.885
26758	4.2789	241.493	301.208
26759	4.279	255.835	107.853
26760	4.2791	269.478	116.515
26761	4.2791	264.572	91.298
26762	4.2792	235.008	91.6519
26763	4.2792	222.387	109.95
26764	4.2793	240.005	45.5808
26765	4.2794	295.781	308.807
26766	4.2794	274.839	199.78
26767	4.2795	215.277	119.116
26768	4.2795	278.744	54.5264
26769	4.2796	164.188	113.591
26770	4.2796	199.499	345.85
26771	4.2797	253.452	136.046
26772	4.2798	253.367	120.944
26773	4.2798	222.185	274.408

26774	4.2799	227.481	316.031
26775	4.2799	163.388	279.193
26776	4.28	209.036	88.4464
26777	4.2801	292.944	271.526
26778	4.2802	232.401	282.576
26779	4.2803	290.893	315.967
26780	4.2803	164.044	162.35
26781	4.2803	216.998	321.392
26782	4.2803	289.878	313.525
26783	4.2804	231.518	123.635
26784	4.2804	257.688	119.464
26785	4.2805	218.546	188.346
26786	4.2807	233.296	106.628
26787	4.281	283.445	72.5404
26788	4.281	248.266	25.1138
26789	4.2813	202.355	82.5622
26790	4.2813	241.19	266.444
26791	4.2814	211.181	81.3667
26792	4.2815	242.596	104.396
26793	4.2817	202.834	182.48
26794	4.2818	235.758	127.037
26795	4.2818	217.777	128.349
26796	4.2818	264.491	119.414
26797	4.2819	247.94	144.355
26798	4.2819	263.724	15.5647
26799	4.282	232.551	136.443
26800	4.2821	235.829	32.9196
26801	4.2822	252.94	219.769
26802	4.2822	234.347	116.474
26803	4.2822	239.235	200.762
26804	4.2823	242.46	233.174
26805	4.2823	269.667	80.7708
26806	4.2823	258.636	38.3801
26807	4.2824	242.893	314.259
26808	4.2825	267.924	289.412
26809	4.2825	215.313	113.599
26810	4.2825	203.061	129.488
26811	4.2825	260.415	51.9691
26812	4.2826	194.941	241.894
26813	4.2826	207.524	20.5832
26814	4.2828	253.091	130.974
26815	4.2828	183.267	59.1336
26816	4.2828	191.511	147.573
26817	4.2829	245.403	235.002
26818	4.2831	227.7	165.688
26819	4.2832	170.043	222.207
26820	4.2832	191.09	77.4509
26821	4.2832	183.622	189.937

26822	4.2832	230.839	87.0097
26823	4.2833	256.903	147.296
26824	4.2833	289.112	141.621
26825	4.2833	235.886	108.309
26826	4.2833	253.325	168.206
26827	4.2834	165.703	163.758
26828	4.2835	290.906	32.5599
26829	4.2836	264.806	70.4939
26830	4.2837	240.891	159.336
26831	4.2839	257.077	224.001
26832	4.2839	-2.8446	125.616
26833	4.2839	216.273	159.078
26834	4.284	221.136	238.235
26835	4.2841	200.769	343.793
26836	4.2842	252.153	148.894
26837	4.2842	223.366	306.373
26838	4.2844	258.272	122.245
26839	4.2845	262.274	51.2863
26840	4.2845	221.909	171.727
26841	4.2846	262.122	343.235
26842	4.2846	190.357	127.963
26843	4.2847	190.244	241.912
26844	4.2849	117.456	39.9107
26845	4.2849	239.013	115.368
26846	4.2849	253.725	124.551
26847	4.285	259.858	99.692
26848	4.285	186.635	184.365
26849	4.285	155.947	74.0452
26850	4.285	243.7	267.145
26851	4.2852	262.411	55.8341
26852	4.2854	232.959	48.0241
26853	4.2854	220.304	135.608
26854	4.2854	241.06	231.504
26855	4.2854	240.694	241.934
26856	4.2857	284.349	37.3936
26857	4.2857	227.084	126.944
26858	4.2859	219.645	126.405
26859	4.2859	227.822	338.294
26860	4.2861	226.158	58.1506
26861	4.2862	201.452	162.254
26862	4.2862	157.546	116.509
26863	4.2862	228.658	112.732
26864	4.2863	223.095	83.4
26865	4.2863	199.824	200.86
26866	4.2865	231.697	184.638
26867	4.2865	255.623	47.3243
26868	4.2866	275.073	53.5452
26869	4.2867	280.302	93.666

26870	4.2867	164.25	138.723
26871	4.2868	196.684	98.9078
26872	4.2869	186.391	350.807
26873	4.287	171.184	69.1059
26874	4.2876	205.802	70.2038
26875	4.2877	276.802	79.0847
26876	4.2879	225.33	215.857
26877	4.288	228.896	128.322
26878	4.2881	196.696	81.1278
26879	4.2882	192.028	104.95
26880	4.2884	311.702	42.0033
26881	4.2884	266.221	148.436
26882	4.2886	196.833	179.704
26883	4.2886	243.273	107.064
26884	4.2887	199.207	142.005
26885	4.2887	164.592	277.709
26886	4.2887	221.753	177.175
26887	4.2887	248.556	100.359
26888	4.2888	217.48	249.24
26889	4.2888	159.687	220.61
26890	4.289	192.355	234.309
26891	4.289	247.916	8.7591
26892	4.289	223.423	269.617
26893	4.2891	279.778	56.9214
26894	4.2891	188.58	231.347
26895	4.2892	226.55	23.5386
26896	4.2892	282.002	36.563
26897	4.2893	207.834	228.826
26898	4.2893	213.628	326.977
26899	4.2894	238.134	243.078
26900	4.2895	188.745	159.208
26901	4.2896	212.328	196.263
26902	4.2896	203.036	220.272
26903	4.2898	206.112	90.4964
26904	4.29	247.732	58.5874
26905	4.2901	218.701	154.734
26906	4.2902	273.271	91.9565
26907	4.2903	200.304	113.525
26908	4.2909	188.323	122.794
26909	4.2909	325.238	309.345
26910	4.2911	284.422	116.071
26911	4.2912	302.318	117.805
26912	4.2912	221.856	118.046
26913	4.2912	261.441	27.9015
26914	4.2914	199.142	29.681
26915	4.2915	254.805	130.587
26916	4.2915	251.88	157.361
26917	4.2916	217.67	90.2756

26918	4.2916	201.25	127.007
26919	4.2917	249.039	278.745
26920	4.2917	176.996	87.1349
26921	4.2918	348.3	356.161
26922	4.2918	245.962	82.6906
26923	4.2918	247.334	74.3165
26924	4.292	234.837	238.42
26925	4.292	188.115	173.686
26926	4.2922	228.067	119.204
26927	4.2923	215.602	107.563
26928	4.2924	233.21	24.0178
26929	4.2925	224.82	111.428
26930	4.2926	198.028	93.3971
26931	4.2927	233.591	22.3796
26932	4.293	260.199	99.8489
26933	4.293	303.183	199.985
26934	4.2931	212.636	291.693
26935	4.2931	260.837	231.405
26936	4.2932	219.742	96.3903
26937	4.2932	243.602	87.9099
26938	4.2934	193.733	261.344
26939	4.2934	216.41	47.9758
26940	4.2935	251.809	5.7468
26941	4.2935	218.471	290.888
26942	4.2938	208.513	172.106
26943	4.2939	214.995	260.311
26944	4.294	188.173	72.3979
26945	4.294	203.651	120.814
26946	4.294	214.058	37.3815
26947	4.294	192.206	157.686
26948	4.294	255.999	63.9888
26949	4.2941	289.658	34.6392
26950	4.2941	218.26	199.746
26951	4.2941	253.464	202.488
26952	4.2941	207.904	331.815
26953	4.2944	309.282	106.642
26954	4.2944	206.438	253.059
26955	4.2944	145.862	247.228
26956	4.2944	229.294	350.231
26957	4.2944	187.159	158.183
26958	4.2945	287.68	35.7035
26959	4.2946	225.769	140.574
26960	4.2946	184.011	94.9091
26961	4.2947	232.961	109.884
26962	4.295	244.637	326.885
26963	4.295	291.529	13.3286
26964	4.2951	196.324	88.4106
26965	4.2952	163.148	88.8104

26966	4.2952	219.926	97.3774
26967	4.2953	280.543	15.5935
26968	4.2954	240.034	171.032
26969	4.2956	247.231	38.194
26970	4.2957	288.686	55.5573
26971	4.2957	198.132	218.169
26972	4.2957	220.674	146.378
26973	4.2958	255.225	103.289
26974	4.2958	261.447	80.7354
26975	4.2959	237.114	135.343
26976	4.296	262.236	139.238
26977	4.296	236.469	56.5145
26978	4.296	163.362	55.4166
26979	4.296	274.727	117.674
26980	4.2962	200.193	226.265
26981	4.2962	202.051	20.1829
26982	4.2963	227.401	32.0838
26983	4.2964	184.49	240.409
26984	4.2964	220.759	79.2471
26985	4.2964	163.172	188.543
26986	4.2964	260.722	152.875
26987	4.2966	187.257	202.759
26988	4.2968	228.163	91.3589
26989	4.2968	245.783	329.312
26990	4.2969	255.109	77.6251
26991	4.2969	275.86	4.4948
26992	4.297	271.644	61.2606
26993	4.297	248.173	272.36
26994	4.2971	186.63	191.293
26995	4.2972	230.957	90.4945
26996	4.2972	214.948	110.16
26997	4.2975	271.548	24.623
26998	4.2976	240.931	130.858
26999	4.2977	234.282	267.932
27000	4.2977	260.395	134.014
27001	4.2978	7.8453	51.9781
27002	4.2979	268.952	64.5971
27003	4.298	208.454	90.3363
27004	4.2982	221.15	154.81
27005	4.2983	245.177	114.044
27006	4.2985	210.486	261.291
27007	4.2987	129.719	159.619
27008	4.2987	228.221	116.587
27009	4.2988	257.521	334.596
27010	4.2988	282.756	61.3924
27011	4.2988	257.448	160.554
27012	4.2989	235.486	17.0456
27013	4.299	204.582	259.451

27014	4.299	215.229	129.37
27015	4.2992	218.571	112.992
27016	4.2994	247.337	240.413
27017	4.2997	322.015	87.7738
27018	4.2997	165.545	40.9185
27019	4.2997	210.989	117.476
27020	4.2998	196.459	29.355
27021	4.2998	228.395	167.76
27022	4.2999	258.865	135.313
27023	4.2999	262.545	86.8271
27024	4.2999	213.124	142.758
27025	4.2999	248.316	170.195
27026	4.3	200.592	161.988
27027	4.3001	170.773	73.0294
27028	4.3001	221.738	291.772
27029	4.3002	279.057	60.608
27030	4.3002	203.501	69.8963
27031	4.3003	211.886	108.952
27032	4.3003	264.244	117.772
27033	4.3003	221.048	233.269
27034	4.3004	186.597	238.61
27035	4.3005	258.153	46.122
27036	4.3005	227.15	112.54
27037	4.3006	227.247	44.3796
27038	4.3009	225.587	122.494
27039	4.301	229.064	256.688
27040	4.301	290.648	332.891
27041	4.3012	221.714	233.754
27042	4.3012	203.773	251.15
27043	4.3013	195.505	194.196
27044	4.3014	253.155	136.567
27045	4.3018	261.104	43.9808
27046	4.3018	217.33	276.473
27047	4.3018	297.075	98.7419
27048	4.3019	281.081	46.3406
27049	4.3019	248.856	236.785
27050	4.3019	246.675	127.203
27051	4.302	247.882	258.041
27052	4.3021	258.444	238.432
27053	4.3022	193.523	163.083
27054	4.3022	233.72	106.695
27055	4.3023	232.678	306.81
27056	4.3024	261.082	125.548
27057	4.3025	233.735	130.258
27058	4.3026	260.091	68.4822
27059	4.3026	280.844	28.9689
27060	4.3026	196.303	202.32
27061	4.3028	252.645	53.6772

27062	4.3029	246.356	59.663
27063	4.3031	223.034	112.577
27064	4.3032	264.834	119.543
27065	4.3032	199.149	121.779
27066	4.3033	208.135	18.3218
27067	4.3034	302.302	37.1714
27068	4.3034	243.863	160.564
27069	4.3034	270.326	124.282
27070	4.3035	211.154	292.936
27071	4.3035	248.575	81.8794
27072	4.3035	188.023	191.364
27073	4.3035	228.273	27.5086
27074	4.3036	251.705	93.0074
27075	4.3037	269.637	136.784
27076	4.3037	205.906	235.328
27077	4.3037	241.02	122.988
27078	4.3038	251.276	62.8086
27079	4.3039	300.231	202.564
27080	4.3041	232.116	227.146
27081	4.3043	273.031	90.8785
27082	4.3043	180.586	178.203
27083	4.3043	286.758	68.7705
27084	4.3044	252.336	343.799
27085	4.3046	203.605	268.723
27086	4.3046	228.091	173.428
27087	4.3046	165.736	343.713
27088	4.3047	226.682	117.203
27089	4.3049	226.051	75.736
27090	4.305	247.919	40.406
27091	4.305	211.377	162.622
27092	4.305	272.699	328.236
27093	4.3051	194.363	112.18
27094	4.3051	208.281	55.2201
27095	4.3052	227.591	116.401
27096	4.3052	273.847	155.041
27097	4.3053	262.227	215.733
27098	4.3053	222.728	71.213
27099	4.3053	254.696	154.948
27100	4.3054	174.356	213.873
27101	4.3054	268.817	216.887
27102	4.3055	191.063	258.845
27103	4.3055	197.899	171.328
27104	4.3055	207.267	124.085
27105	4.3055	279.84	180.847
27106	4.3057	227.915	150.732
27107	4.3058	242.815	253.254
27108	4.3059	275.434	10.9315
27109	4.306	211.229	280.034

27110	4.3061	209.096	15.6493
27111	4.3062	216.977	112.279
27112	4.3063	242.846	50.9942
27113	4.3063	247.574	126.063
27114	4.3063	186.644	169.707
27115	4.3064	236.745	110.06
27116	4.3064	221.509	99.4097
27117	4.3066	230.172	109.909
27118	4.3067	192.019	115.763
27119	4.3069	216.496	161.282
27120	4.3069	243.413	115.422
27121	4.307	257.367	316.793
27122	4.3072	221.537	41.649
27123	4.3072	258.389	294.384
27124	4.3073	227.588	173.58
27125	4.3073	238.507	118.673
27126	4.3074	258.376	98.7047
27127	4.3077	226.086	117.416
27128	4.3078	242.164	192.222
27129	4.3079	219.726	142.447
27130	4.308	217.928	16.9065
27131	4.3082	194.789	161.736
27132	4.3082	223.975	117.571
27133	4.3084	203.535	139.66
27134	4.3085	158.274	290.287
27135	4.3085	256.273	64.8477
27136	4.3085	195.247	155.686
27137	4.3086	275.639	244.144
27138	4.3086	236.743	287.585
27139	4.3088	192.56	129.87
27140	4.3089	241.14	116.84
27141	4.3089	216.517	90.6281
27142	4.3091	223.388	121.794
27143	4.3092	277.612	249.757
27144	4.3092	178.118	116.06
27145	4.3092	222.276	149.077
27146	4.3092	233.017	281.411
27147	4.3093	287.376	42.4072
27148	4.3093	246.367	205.374
27149	4.3097	266.623	71.0958
27150	4.3098	292.814	120.229
27151	4.3099	206.928	296.193
27152	4.3099	203.491	113.84
27153	4.3099	225.434	158.526
27154	4.31	-2.7327	21.2503
27155	4.31	191.108	333.959
27156	4.3101	210.276	113.297
27157	4.3103	239.42	270.47

27158	4.3103	212.26	93.2522
27159	4.3103	231.942	269.992
27160	4.3103	220.798	108.432
27161	4.3104	258.1	24.058
27162	4.3104	189.918	220.74
27163	4.3105	212.631	230.193
27164	4.3105	225.379	129.385
27165	4.3106	215.654	99.8503
27166	4.3106	242.627	84.5692
27167	4.3106	180.5	115.949
27168	4.3107	244.089	129.909
27169	4.3107	243.089	283.79
27170	4.3108	229.842	89.8705
27171	4.311	232.899	81.3471
27172	4.311	173.436	91.6194
27173	4.3111	250.282	50.1278
27174	4.3112	215.079	223.488
27175	4.3113	311.964	76.4364
27176	4.3114	194.823	105.63
27177	4.3114	230.881	7.1611
27178	4.3115	237.283	154.93
27179	4.3116	280.592	321.965
27180	4.3117	296.321	68.8966
27181	4.3117	213.521	186.888
27182	4.3117	313.206	42.7673
27183	4.3118	276.474	308.162
27184	4.3118	207.088	30.5859
27185	4.3119	246.785	99.946
27186	4.3119	234.967	74.7943
27187	4.3121	298.916	46.3225
27188	4.3122	183.916	334.66
27189	4.3124	261.971	16.9088
27190	4.3125	314.829	46.077
27191	4.3125	206.781	156.454
27192	4.3127	218.877	119.947
27193	4.3127	213.643	36.6114
27194	4.3127	294.841	15.0593
27195	4.3128	266.418	151.035
27196	4.3128	277.031	99.6758
27197	4.3128	239.892	81.3644
27198	4.3129	202.563	259.059
27199	4.3129	176.949	161.366
27200	4.313	236.289	36.0878
27201	4.3131	271.148	92.6266
27202	4.3132	257.151	270.696
27203	4.3132	210.03	292.136
27204	4.3132	259.824	172.834
27205	4.3132	253.686	151.424

27206	4.3132	288.08	43.8794
27207	4.3133	314.043	114.068
27208	4.3133	296.071	286.39
27209	4.3134	251.658	77.0865
27210	4.3134	188.973	224.057
27211	4.3134	184.481	168.669
27212	4.3134	240.549	127.855
27213	4.3135	271.629	110.135
27214	4.3135	222.989	202.418
27215	4.3135	230.978	113.78
27216	4.3135	236.86	105.902
27217	4.3136	194.464	105.961
27218	4.3136	236.408	314.402
27219	4.3138	233.635	91.6005
27220	4.3138	243.275	157.381
27221	4.3141	268.729	139.374
27222	4.3145	213.033	223.852
27223	4.3147	172.959	316.554
27224	4.3148	235.628	139.887
27225	4.3148	200.734	131.787
27226	4.3149	235.486	124.558
27227	4.3149	188.354	141.703
27228	4.3153	220.538	94.2477
27229	4.3154	260.064	86.5272
27230	4.3154	270.636	116.8
27231	4.3155	236.541	258.818
27232	4.3157	191.723	123.943
27233	4.3157	188.728	139.691
27234	4.3158	277.918	125.722
27235	4.316	216.057	176.082
27236	4.3161	254.5	157.281
27237	4.3162	234.11	99.3926
27238	4.3162	288.629	116.64
27239	4.3163	218.155	230.348
27240	4.3164	202.572	126.806
27241	4.3164	221.918	253.903
27242	4.3166	159.765	31.1062
27243	4.3166	225.256	193.513
27244	4.3167	268.947	48.8256
27245	4.3167	250.544	113.947
27246	4.3168	185.391	305.481
27247	4.317	254.741	112.657
27248	4.317	235.734	4.9524
27249	4.3171	211.162	119.434
27250	4.3171	246.421	103.098
27251	4.3173	320.988	117.8
27252	4.3173	239.071	130.964
27253	4.3173	253.189	359.402

27254	4.3174	198.244	185.655
27255	4.3174	186.658	138.855
27256	4.3176	206.269	146.045
27257	4.3177	199.074	234.861
27258	4.3177	220.799	117.793
27259	4.3178	203.625	352.24
27260	4.3178	204.006	128.731
27261	4.318	223.097	25.5817
27262	4.3181	259.837	36.6426
27263	4.3184	267.72	87.6287
27264	4.3184	246.646	100.679
27265	4.3185	241.019	249.713
27266	4.3185	178.643	80.3669
27267	4.3186	260.775	248.306
27268	4.3186	205.444	126.488
27269	4.3188	234.899	154.526
27270	4.3188	196.466	165.942
27271	4.3188	304.095	25.5823
27272	4.319	199.88	110.83
27273	4.3191	264.154	296.604
27274	4.3191	217.742	122.838
27275	4.3191	253.672	210.986
27276	4.3191	201.929	146.505
27277	4.3191	237.335	156.516
27278	4.3192	272.107	358.842
27279	4.3192	265.968	88.3994
27280	4.3192	215.682	120.098
27281	4.3194	254.586	19.8515
27282	4.3194	243.254	65.3787
27283	4.3194	219.678	220.366
27284	4.3194	229.514	229.376
27285	4.3195	278.635	359.593
27286	4.3196	180.564	268.317
27287	4.3198	241.143	87.1527
27288	4.3199	224.576	147.57
27289	4.32	260.888	297.789
27290	4.32	244.437	97.4093
27291	4.32	240.118	105.105
27292	4.3201	192.405	74.0299
27293	4.3202	242.669	167.738
27294	4.3204	207.243	53.4426
27295	4.3206	222.022	327.17
27296	4.3206	265.262	166.657
27297	4.3207	304.336	241.501
27298	4.3207	221.388	37.3628
27299	4.3207	300.542	86.9125
27300	4.3208	216.734	28.6434
27301	4.321	158.026	350.254

27302	4.3212	239.218	145.441
27303	4.3212	229.493	42.8747
27304	4.3212	177.428	273.378
27305	4.3213	252.522	297.409
27306	4.3214	226.074	225.9
27307	4.3216	188.89	184.053
27308	4.3218	216.9	277.393
27309	4.322	225.909	150.959
27310	4.322	243.823	160.881
27311	4.3221	160.654	224.106
27312	4.3221	248.238	132.389
27313	4.3222	222.489	234.888
27314	4.3222	257.491	130.073
27315	4.3223	254.789	94.9894
27316	4.3223	204.812	263.661
27317	4.3225	260.756	33.9132
27318	4.3225	184.754	318.269
27319	4.3226	256.989	162.215
27320	4.3227	228.311	195.812
27321	4.3227	202.645	75.5958
27322	4.3227	202.837	111.49
27323	4.3228	305.88	306.786
27324	4.3228	197.076	1.2869
27325	4.3229	232.448	27.7458
27326	4.323	271.692	239.005
27327	4.3231	222.728	291.384
27328	4.3231	207.711	172.568
27329	4.3231	290.148	73.2201
27330	4.3232	271.634	205.265
27331	4.3232	269.042	159.349
27332	4.3234	243.309	178.328
27333	4.3235	289.301	303.929
27334	4.3235	251.612	117.801
27335	4.3236	232.037	73.6342
27336	4.3236	-2.9209	177.326
27337	4.3237	230.051	63.568
27338	4.3238	209.956	136.096
27339	4.3239	235.223	337.214
27340	4.3239	245.295	169.606
27341	4.324	258.281	349.253
27342	4.324	212.009	178.179
27343	4.324	279.195	82.4076
27344	4.3243	216.401	86.9061
27345	4.3244	262.242	144.148
27346	4.3244	-2.6959	1.1001
27347	4.3245	192.363	175.1
27348	4.3245	283.079	137.053
27349	4.3247	225.129	145.317

27350	4.3247	183.899	123.042
27351	4.3249	246.86	270.905
27352	4.3252	250.625	104.585
27353	4.3253	208.31	105.515
27354	4.3255	221.042	280.102
27355	4.3255	215.976	177.259
27356	4.3255	210.383	292.349
27357	4.3255	197.52	92.4046
27358	4.3255	281.098	180.821
27359	4.3256	266.6	27.0199
27360	4.3256	253.384	315.157
27361	4.3257	229.886	49.6553
27362	4.3257	269.282	69.2278
27363	4.3258	187.004	265.783
27364	4.3258	220.365	334.424
27365	4.326	212.477	139.031
27366	4.3261	267.866	37.774
27367	4.3262	288.259	41.698
27368	4.3262	199.849	163.399
27369	4.3263	209.941	158.164
27370	4.3263	205.652	119.549
27371	4.3263	287.846	75.493
27372	4.3263	216.361	174.434
27373	4.3264	301.915	18.4792
27374	4.3264	299.349	45.3203
27375	4.3264	288.223	318.364
27376	4.3266	228.744	194.011
27377	4.3267	273.963	127.671
27378	4.3268	228.57	183.294
27379	4.3268	173.435	164.451
27380	4.3268	185.617	100.829
27381	4.3268	208.662	113.968
27382	4.3269	296.401	342.79
27383	4.3269	212.541	141.042
27384	4.327	224.267	268.218
27385	4.327	251.006	107.862
27386	4.327	240.958	201.9
27387	4.327	192.293	144.321
27388	4.3271	281.243	265.304
27389	4.3271	286.417	36.517
27390	4.3272	300.598	73.8821
27391	4.3272	253.457	69.191
27392	4.3273	210.618	106.903
27393	4.3273	236.04	342.286
27394	4.3274	166.726	173.111
27395	4.3275	197.195	156.12
27396	4.3275	211.405	191.661
27397	4.3276	283.607	251.742

27398	4.3278	190.336	137.615
27399	4.3279	214.749	141.162
27400	4.3279	211.163	56.2082
27401	4.3279	310.138	25.0094
27402	4.328	238.653	98.9586
27403	4.3281	225.246	182.641
27404	4.3281	238.854	60.8044
27405	4.3281	262.03	316.899
27406	4.3283	214.866	342.579
27407	4.3283	276.169	94.5313
27408	4.3284	216.449	155.262
27409	4.3285	257.135	69.5038
27410	4.3287	308.22	16.4164
27411	4.3288	163.967	260.498
27412	4.3291	227.199	92.5064
27413	4.3291	195.284	273.07
27414	4.3292	257.097	57.4985
27415	4.3293	247.231	129.916
27416	4.3294	208.951	168.551
27417	4.3294	205.553	22.4913
27418	4.3295	261.576	50.1045
27419	4.3296	218.855	25.3675
27420	4.3297	252.145	290.117
27421	4.3298	312.201	50.9485
27422	4.3298	236.346	147.418
27423	4.3298	250.136	125.925
27424	4.33	219.823	220.291
27425	4.3302	283.228	120.87
27426	4.3302	260.403	43.9555
27427	4.3303	294.488	69.1697
27428	4.3304	260.147	120.005
27429	4.3304	248.544	50.3633
27430	4.3305	231.559	64.1153
27431	4.3307	304.335	92.8902
27432	4.3307	186.862	160.694
27433	4.3307	239.373	106.942
27434	4.3307	237.811	109.988
27435	4.3308	211.913	229.47
27436	4.3308	305.89	84.1833
27437	4.3309	233.881	112.422
27438	4.3309	301.226	288.773
27439	4.331	337.653	285.119
27440	4.331	245.376	91.1454
27441	4.3311	223.644	113.547
27442	4.3312	213.282	268.852
27443	4.3313	208.394	98.7521
27444	4.3315	170.419	178.276
27445	4.3316	216.799	122.468

27446	4.3317	321.107	133.621
27447	4.3318	226.852	116.574
27448	4.3318	208.527	68.9979
27449	4.3319	267.084	44.285
27450	4.3319	203.534	140.154
27451	4.332	229.842	92.2276
27452	4.3321	241.376	81.9272
27453	4.3322	338.959	52.9054
27454	4.3322	203.836	160.518
27455	4.3323	200.455	23.0778
27456	4.3323	192.391	248.584
27457	4.3324	124.487	171.885
27458	4.3325	218.42	167.912
27459	4.3325	241.99	73.968
27460	4.3327	226.374	85.2596
27461	4.3327	198.25	222.381
27462	4.3329	252.471	152.119
27463	4.333	263.865	164.991
27464	4.333	281.026	226.697
27465	4.3331	232.541	224.344
27466	4.3331	199.595	235.183
27467	4.3332	212.263	129.014
27468	4.3333	227.126	26.0919
27469	4.3333	212.302	112.703
27470	4.3334	165.771	35.4678
27471	4.3334	249.691	85.5296
27472	4.3335	203.099	254.665
27473	4.3338	241.749	129.072
27474	4.3339	213.488	117.784
27475	4.3341	230.84	139.194
27476	4.3342	318.028	287.176
27477	4.3342	199.16	87.7829
27478	4.3344	223.398	145.136
27479	4.3345	223.604	190.888
27480	4.3346	182.708	326.138
27481	4.3347	179.582	152.046
27482	4.3348	178.118	105.593
27483	4.3349	261.296	16.135
27484	4.3349	295.765	16.8158
27485	4.3349	246.719	71.3946
27486	4.3349	205.348	107.802
27487	4.3352	203.795	229.13
27488	4.3352	246.254	336.974
27489	4.3352	272.242	85.914
27490	4.3354	209.625	224.077
27491	4.3354	288.265	45.9583
27492	4.3355	206.938	185.685
27493	4.3356	264.655	3.695

27494	4.3357	276.921	102.059
27495	4.3357	250.236	60.1746
27496	4.3359	223.664	322.502
27497	4.3359	219.811	290.981
27498	4.3359	262.075	88.4115
27499	4.3359	188.825	184.151
27500	4.3359	237.484	316.951
27501	4.336	243.36	107.685
27502	4.3361	227.675	194.622
27503	4.3361	246.055	101.282
27504	4.3362	257.648	89.8401
27505	4.3362	209.522	110.72
27506	4.3362	248.86	230.204
27507	4.3363	261.888	216.406
27508	4.3363	231.9	266.635
27509	4.3363	251.333	230.095
27510	4.3364	257.716	188.912
27511	4.3365	231.227	186.774
27512	4.3367	273.296	136.248
27513	4.3368	209.201	181.677
27514	4.3368	220.595	188.247
27515	4.337	311.427	72.0392
27516	4.3372	256.93	129.37
27517	4.3372	190.128	91.2072
27518	4.3373	247.134	164.049
27519	4.3373	223.702	117.119
27520	4.3374	264.562	138.346
27521	4.3374	114.167	31.4922
27522	4.3375	228.96	283.031
27523	4.3375	238.17	68.9353
27524	4.3376	187.283	349.097
27525	4.3376	188.42	99.7672
27526	4.3376	262.229	198.188
27527	4.3379	264.372	54.4375
27528	4.3379	177.161	124.465
27529	4.3381	250.063	56.6943
27530	4.3382	220.552	165.13
27531	4.3383	191.865	74.9402
27532	4.3386	225.4	132.12
27533	4.3388	233.334	180.802
27534	4.3389	278.574	230.771
27535	4.3393	207.857	223.101
27536	4.3394	229.461	85.3067
27537	4.3395	276.061	86.7209
27538	4.3395	293.385	81.0613
27539	4.3396	195.74	172.188
27540	4.3397	226.836	57.0128
27541	4.3399	270.583	271.835

27542	4.3399	234.633	120.831
27543	4.3401	271.493	205.473
27544	4.3402	210.634	30.3865
27545	4.3403	265.856	109.943
27546	4.3403	201.468	69.1904
27547	4.3403	225.281	136.251
27548	4.3404	298.22	285.969
27549	4.3404	210.161	269.265
27550	4.3404	176.424	201.459
27551	4.3404	202.921	129.106
27552	4.3404	242.25	35.8877
27553	4.3404	210.925	337.833
27554	4.3404	229.072	164.711
27555	4.3405	222.684	233.465
27556	4.3405	-2.7976	246.509
27557	4.3408	179.227	172.141
27558	4.3409	256.672	197.411
27559	4.3411	224.374	83.801
27560	4.3411	297.065	234.833
27561	4.3413	255.118	290.939
27562	4.3414	253.408	252.134
27563	4.3414	207.163	232.406
27564	4.3414	283.327	74.8899
27565	4.3414	251.776	189.835
27566	4.3414	229.367	131.602
27567	4.3415	209.645	115.058
27568	4.3415	173.042	129.472
27569	4.3415	220.216	126.871
27570	4.3415	201.69	140.048
27571	4.3415	218.641	151.922
27572	4.3416	179.989	317.021
27573	4.3418	293.915	306.454
27574	4.3418	243.63	50.8401
27575	4.3419	188.308	129.781
27576	4.3419	177.675	133.666
27577	4.3419	185.688	327.189
27578	4.3421	256.128	17.1032
27579	4.3424	281.868	136.634
27580	4.3425	251.486	117.922
27581	4.3425	216.646	76.9249
27582	4.3426	253.535	131.787
27583	4.3428	185.422	252.484
27584	4.3429	221.377	114.984
27585	4.3431	269.078	31.6015
27586	4.3431	221.428	179.408
27587	4.3432	274.509	38.7528
27588	4.3433	248.274	183.415
27589	4.3433	213.215	99.2874

27590	4.3434	321.142	50.9156
27591	4.3434	228.99	131.415
27592	4.3434	257.85	348.438
27593	4.3435	278.004	123.024
27594	4.3435	161.065	139.911
27595	4.3436	256.727	193.537
27596	4.3436	301.173	179.516
27597	4.3437	208.337	107.493
27598	4.3437	210.712	161.783
27599	4.3437	187.66	253.373
27600	4.3437	251.504	57.0577
27601	4.3438	212.289	156.835
27602	4.3438	242.406	120.252
27603	4.3439	278.385	56.8422
27604	4.3439	217.401	172.832
27605	4.3439	179.762	109.263
27606	4.3444	227.275	129.875
27607	4.3444	250.856	118.828
27608	4.3444	217.284	36.0961
27609	4.3445	251.889	280.744
27610	4.3445	257.776	168.825
27611	4.3445	172.673	39.2224
27612	4.3446	-3.3991	118.962
27613	4.3447	138.076	144.011
27614	4.3447	244.529	103.718
27615	4.3448	271.357	177.559
27616	4.3448	219.939	171.642
27617	4.3449	219.141	70.7145
27618	4.3449	242.422	312.156
27619	4.345	236.221	118.181
27620	4.345	196.64	127.776
27621	4.3451	181.469	27.1333
27622	4.3451	263.618	269.832
27623	4.3453	261.399	48.8198
27624	4.3456	240.25	321.714
27625	4.3457	330.479	23.6524
27626	4.3457	248.007	28.8589
27627	4.3458	214.669	67.7587
27628	4.346	284.383	27.2242
27629	4.346	190.298	120.493
27630	4.3461	232.506	251.469
27631	4.3461	216.17	77.0335
27632	4.3462	242.916	10.9759
27633	4.3464	288.763	41.9662
27634	4.3464	-2.7619	163.756
27635	4.3465	221.007	7.8327
27636	4.3466	195.387	116.919
27637	4.3466	200.799	336.228

27638	4.3467	162.242	150.82
27639	4.3468	328.822	256.936
27640	4.3468	233.953	178.288
27641	4.3468	149.854	265.929
27642	4.3471	274.751	31.1977
27643	4.3471	255.906	161.567
27644	4.3472	228.406	19.1155
27645	4.3472	223.436	105.499
27646	4.3473	278.345	221.661
27647	4.3474	240.157	235.84
27648	4.3476	210.531	209.858
27649	4.3477	224.385	142.221
27650	4.3477	238.314	76.7496
27651	4.3478	248.989	191.498
27652	4.3481	178.666	227.82
27653	4.3483	257.673	166.374
27654	4.3484	249.139	69.6555
27655	4.3484	191.365	39.9783
27656	4.3486	313.86	54.0679
27657	4.3488	335.686	39.9896
27658	4.3488	119.075	122.679
27659	4.3492	229.754	216.689
27660	4.3494	224.144	196.588
27661	4.3495	210.754	113.388
27662	4.3495	197.944	112.969
27663	4.3496	270.289	274.701
27664	4.3497	278.649	143.975
27665	4.3497	225.585	129.336
27666	4.3497	298.292	305.75
27667	4.3497	197.053	257.807
27668	4.3498	193.966	253.608
27669	4.3498	230.56	288.823
27670	4.3499	272.961	68.2911
27671	4.3499	221.826	227.608
27672	4.35	219.849	89.0889
27673	4.35	253.596	50.2664
27674	4.3501	259.216	63.5476
27675	4.3501	206.495	103.617
27676	4.3501	264.637	164.722
27677	4.3501	260.206	5.313
27678	4.3503	226.288	255.09
27679	4.3505	254.809	154.545
27680	4.3507	297.386	63.1288
27681	4.3508	232.574	36.1231
27682	4.3508	194.397	207.609
27683	4.3508	225.689	101.355
27684	4.3509	235.758	103.861
27685	4.351	226.638	149.965

27686	4.351	244.614	110.847
27687	4.351	263.393	31.8068
27688	4.3511	239.485	248.185
27689	4.3512	254.459	169.602
27690	4.3513	186.531	94.5408
27691	4.3514	283.204	75.2024
27692	4.3515	273.972	119.359
27693	4.3515	236.21	37.9415
27694	4.3515	188.413	28.3592
27695	4.3516	233.895	240.286
27696	4.3516	215.791	176.815
27697	4.3517	268.422	61.2805
27698	4.3517	250.257	70.8191
27699	4.3519	213.762	20.2122
27700	4.352	242.793	140.719
27701	4.3521	252.131	227.859
27702	4.3522	211.525	118.788
27703	4.3523	164.139	178.704
27704	4.3524	190.177	163.362
27705	4.3525	213.309	315.048
27706	4.3527	241.793	134.28
27707	4.3527	155.284	219.348
27708	4.3528	267.56	68.6228
27709	4.3528	235.066	342.558
27710	4.3529	210.133	133.7
27711	4.3529	282.559	61.9156
27712	4.353	261.277	72.2365
27713	4.353	225.954	316.851
27714	4.3531	306.212	281.377
27715	4.3531	233.371	230.695
27716	4.3531	225.976	233.865
27717	4.3532	233.552	29.7545
27718	4.3534	230.543	79.7375
27719	4.3535	259.253	42.7238
27720	4.3535	272.763	93.6379
27721	4.3536	238.14	77.5338
27722	4.3538	212.22	54.4425
27723	4.3539	269.067	64.7174
27724	4.3539	305.47	21.324
27725	4.354	217.024	143.089
27726	4.354	207.401	72.2843
27727	4.3541	219.48	198.248
27728	4.3542	252.161	105.603
27729	4.3543	268.352	96.0047
27730	4.3545	195.432	169.727
27731	4.3545	262.365	114.479
27732	4.3547	294.441	60.2053
27733	4.3548	257.125	46.4211

27734	4.3548	216.925	41.863
27735	4.3549	238.238	108.258
27736	4.3549	188.866	246.507
27737	4.3549	234.037	131.731
27738	4.3549	267.25	107.214
27739	4.355	191.9	220.23
27740	4.355	230.779	97.5922
27741	4.3551	299.46	44.8637
27742	4.3551	190.855	166.795
27743	4.3552	279.943	94.196
27744	4.3554	258.589	227.389
27745	4.3555	242.01	220.967
27746	4.3556	280.442	227.211
27747	4.3556	322.335	242.701
27748	4.3557	228.162	359.863
27749	4.3557	190.015	309.016
27750	4.3561	302.298	34.7434
27751	4.3561	267.856	90.0741
27752	4.3562	249.68	172.753
27753	4.3562	286.483	20.6846
27754	4.3563	194.221	84.4975
27755	4.3564	238.846	162.657
27756	4.3564	204.082	243.85
27757	4.3564	172.768	212.158
27758	4.3564	146.196	144.042
27759	4.3565	208.225	118.649
27760	4.3566	257.104	241.341
27761	4.3569	305.109	49.8354
27762	4.357	257.959	78.1477
27763	4.3572	247.911	79.6962
27764	4.3573	287.754	86.778
27765	4.3573	208.616	143.007
27766	4.3574	282.897	114.96
27767	4.3574	196.263	237.684
27768	4.3575	245.712	254.318
27769	4.3575	224.984	146.789
27770	4.3575	201.244	103.22
27771	4.3576	246.725	51.5856
27772	4.3577	218.101	194.055
27773	4.3578	192.436	163.305
27774	4.3579	258.281	165.23
27775	4.358	265.232	74.7957
27776	4.3581	223.323	24.4516
27777	4.3583	274.566	91.9334
27778	4.3583	277.58	15.428
27779	4.3583	184.562	267.004
27780	4.3583	197.74	155.886
27781	4.3583	236.741	105.402

27782	4.3583	259.165	275.897
27783	4.3585	225.467	53.4434
27784	4.3585	255.877	155.187
27785	4.3585	303.265	7.24
27786	4.3586	207.066	224.416
27787	4.3586	295.039	127.515
27788	4.3587	203.796	160.302
27789	4.3587	225.324	83.6737
27790	4.3587	239.275	95.8867
27791	4.3588	211.968	159.359
27792	4.3588	238.536	239.894
27793	4.3588	199.15	90.5518
27794	4.359	252.032	79.1443
27795	4.359	205.171	335.995
27796	4.359	226.41	139.186
27797	4.359	198.935	59.7536
27798	4.359	207.849	269.62
27799	4.3591	315.213	26.9258
27800	4.3591	214.374	107.829
27801	4.3592	241.633	199.525
27802	4.3592	264.792	275.551
27803	4.3593	204.36	17.3785
27804	4.3594	232.454	137.137
27805	4.3595	264.457	92.9345
27806	4.3596	254.684	51.6992
27807	4.3599	259.862	95.4178
27808	4.3599	276.836	66.749
27809	4.3601	64.9494	87.9745
27810	4.3604	242.016	34.8207
27811	4.3604	235.369	98.3042
27812	4.3605	188.636	304.709
27813	4.3605	261.388	115.444
27814	4.3605	205.553	50.4246
27815	4.3605	256.545	33.2509
27816	4.3606	265.916	113.917
27817	4.3606	252.341	340.051
27818	4.3608	249.654	132.225
27819	4.3609	278.64	254.134
27820	4.361	291.561	201.999
27821	4.3611	289.68	299.544
27822	4.3612	235.32	302.488
27823	4.3612	245.401	159.727
27824	4.3615	281.508	84.7001
27825	4.3616	290.713	195.738
27826	4.3616	205.029	163.93
27827	4.3618	234.453	112.997
27828	4.3618	297.926	327.481
27829	4.3619	223.056	76.0481

27830	4.362	213.622	188.968
27831	4.3621	223.429	133.515
27832	4.3621	237.182	15.3081
27833	4.3622	202.603	10.4121
27834	4.3626	202.469	40.4865
27835	4.3627	324.895	253.216
27836	4.363	217.842	234.503
27837	4.3632	319.131	108.699
27838	4.3632	223.632	156.497
27839	4.3633	249.982	86.3013
27840	4.3633	190.268	175.558
27841	4.3634	295.826	38.3371
27842	4.3635	252.189	151.014
27843	4.3635	227.666	115.821
27844	4.3636	164.83	134.327
27845	4.3636	208.257	125.71
27846	4.3636	247.993	309.827
27847	4.3637	293.946	324.301
27848	4.3637	277.473	152.135
27849	4.3638	234.455	169.425
27850	4.3638	268.214	48.1448
27851	4.3638	301.081	122.26
27852	4.3639	284.493	78.9057
27853	4.3639	186.87	226.891
27854	4.3639	239.828	187.914
27855	4.3639	246.431	329.583
27856	4.364	261.921	192.83
27857	4.364	187.775	69.2806
27858	4.3641	289.072	71.0132
27859	4.3642	253.868	315.866
27860	4.3642	180.455	34.0692
27861	4.3644	223.448	111.322
27862	4.3645	221.635	162.752
27863	4.3645	217.903	249.886
27864	4.3645	225.371	89.2322
27865	4.3646	239.908	128.311
27866	4.3647	287.217	314.584
27867	4.3647	205.528	334.232
27868	4.3647	251.216	151.83
27869	4.3649	226.537	156.936
27870	4.365	179.705	145.941
27871	4.3653	-5.7268	62.0807
27872	4.3654	244.042	60.7729
27873	4.3656	341.598	309.356
27874	4.3656	266.958	127.13
27875	4.3657	240.747	160.349
27876	4.3658	207.485	224.05
27877	4.3658	207.629	310.538

27878	4.3658	219.668	295.827
27879	4.3659	266.136	102.753
27880	4.3659	312.988	60.7337
27881	4.366	309.256	147.121
27882	4.3661	237.527	45.2812
27883	4.3661	232.466	302.751
27884	4.3661	228.385	153.157
27885	4.3663	214.31	120.706
27886	4.3665	320.912	16.936
27887	4.3665	250.835	132.898
27888	4.3665	273.79	287.139
27889	4.3666	189.687	207.081
27890	4.3667	184.592	124.088
27891	4.367	203.884	111.26
27892	4.367	248.816	175.263
27893	4.3671	267.046	69.0782
27894	4.3673	198.683	93.437
27895	4.3673	193.71	68.9118
27896	4.3674	257.806	122.327
27897	4.3674	286.37	110.419
27898	4.3674	187.928	270.94
27899	4.3675	279.434	18.7785
27900	4.3676	246.947	127.963
27901	4.3679	262.283	265.377
27902	4.3682	253.019	108.719
27903	4.3682	280.921	68.9736
27904	4.3684	274.06	291.574
27905	4.3685	231.934	153.806
27906	4.3685	235.756	108.202
27907	4.3685	321.023	240.178
27908	4.3686	247.241	249.059
27909	4.3687	191.595	164.488
27910	4.3688	287.772	209.975
27911	4.369	237.14	145.511
27912	4.369	234.061	103.917
27913	4.369	234.224	108.135
27914	4.3691	295.164	64.0948
27915	4.3691	229.185	90.7607
27916	4.3693	212.406	146.424
27917	4.3694	147.298	88.6182
27918	4.3695	278.287	125.764
27919	4.3696	234.859	61.7405
27920	4.37	216.139	266.942
27921	4.37	238.796	279.113
27922	4.37	272.109	229.991
27923	4.3701	266.434	63.4103
27924	4.3702	249.944	143.671
27925	4.3703	257.453	154.588

27926	4.3704	279.43	71.841
27927	4.3705	262.701	9.005
27928	4.3705	187.205	333.9
27929	4.3705	244.552	95.305
27930	4.3707	259.944	171.419
27931	4.3707	268.475	106.525
27932	4.3708	233.351	237.022
27933	4.371	306.734	28.7532
27934	4.3711	259.799	132.699
27935	4.3712	292.237	109.537
27936	4.3712	296.99	18.0976
27937	4.3712	287.121	71.0273
27938	4.3713	244.736	154.28
27939	4.3714	258.857	155.005
27940	4.3714	217.295	2.6536
27941	4.3717	284.46	266.805
27942	4.3718	223.702	257.868
27943	4.3718	303.742	48.4949
27944	4.3719	233.573	129.387
27945	4.372	253.109	26.7851
27946	4.3722	112.929	36.611
27947	4.3722	250.208	233.646
27948	4.3722	217.206	294.004
27949	4.3724	276.412	93.9601
27950	4.3728	218.983	304.193
27951	4.3729	231.597	118.699
27952	4.3729	253.418	53.7813
27953	4.3731	228.661	125.478
27954	4.3731	225.29	165.286
27955	4.3731	266.101	237.721
27956	4.3734	241.306	260.784
27957	4.3734	303.289	316.819
27958	4.3736	292.524	145.338
27959	4.3736	256.457	195.611
27960	4.3736	244.789	218.7
27961	4.3737	207.912	225.313
27962	4.3748	227.89	164.793
27963	4.3749	273.986	177.668
27964	4.3749	201.018	144.17
27965	4.3749	218.837	329.041
27966	4.3752	279.556	76.0386
27967	4.3752	227.684	261.014
27968	4.3752	204.415	106.717
27969	4.3753	287.733	135.876
27970	4.3753	276.78	289.57
27971	4.3754	213.666	223.987
27972	4.3754	227.222	174.271
27973	4.3755	220.198	206.99

27974	4.3756	256.345	317.826
27975	4.3758	283.25	241.228
27976	4.376	226.272	73.4032
27977	4.3762	229.17	140.751
27978	4.3765	190.139	117.464
27979	4.3765	181.147	82.7815
27980	4.3765	221.006	91.3903
27981	4.3766	247.73	187.055
27982	4.3769	229.338	12.2977
27983	4.3769	172.896	45.5502
27984	4.377	278.181	26.547
27985	4.377	232.981	16.8838
27986	4.3772	257.711	199.585
27987	4.3772	192.981	165.487
27988	4.3773	229.453	70.901
27989	4.3774	279.124	115.081
27990	4.3774	221.249	150.351
27991	4.3774	253.744	100.504
27992	4.3775	194.691	125.824
27993	4.3777	259.193	110.456
27994	4.3777	245.04	19.4093
27995	4.3777	283.18	77.0351
27996	4.3778	234.119	134.976
27997	4.3778	256.857	92.8153
27998	4.378	240.541	163.547
27999	4.3781	255.849	113.52
28000	4.3781	221.619	83.5183
28001	4.3782	213.207	111.34
28002	4.3783	257.039	306.47
28003	4.3783	268.815	80.0683
28004	4.3783	308.265	234.296
28005	4.3784	211.915	162.157
28006	4.3786	326.41	359.699
28007	4.3786	261.756	73.01
28008	4.3789	224.698	172.55
28009	4.3791	251.557	111.732
28010	4.3791	232.92	161.905
28011	4.3792	245.343	136.597
28012	4.3793	162.516	230.107
28013	4.3793	192.028	90.654
28014	4.3795	285.246	354.797
28015	4.38	300.8	52.2899
28016	4.38	273.764	93.2216
28017	4.3801	336.917	208.105
28018	4.3801	123.681	355.188
28019	4.3801	185.631	117.497
28020	4.3802	275.513	336.111
28021	4.3803	324.03	42.1494

28022	4.3804	272.979	81.0086
28023	4.3805	310.055	40.1772
28024	4.3805	53.7429	170.496
28025	4.3805	192.827	133.607
28026	4.3806	262.588	34.822
28027	4.3806	232.454	227.571
28028	4.3806	217.672	28.1609
28029	4.3807	225.993	147.723
28030	4.3807	292.434	237.285
28031	4.3808	247.867	201.795
28032	4.3808	271.483	30.887
28033	4.3809	257.779	293.538
28034	4.3809	216.532	102.883
28035	4.381	220.906	175.855
28036	4.381	232.267	183.072
28037	4.3811	191.041	123.452
28038	4.3811	304.461	102.591
28039	4.3812	251.463	186.054
28040	4.3812	254.019	193.444
28041	4.3812	254.453	145.818
28042	4.3813	236.06	178.847
28043	4.3814	254.372	255.374
28044	4.3814	273.497	61.4688
28045	4.3814	232.322	295.067
28046	4.3815	255.154	188.873
28047	4.3815	218.494	161.388
28048	4.3815	263.452	129.635
28049	4.3816	324.612	78.62
28050	4.3816	279.562	209.437
28051	4.3817	259.875	54.9305
28052	4.3818	195.181	322.425
28053	4.382	314.189	107.546
28054	4.382	312.658	81.2789
28055	4.3821	251.217	49.9384
28056	4.3822	294.219	318.438
28057	4.3823	279.904	190.203
28058	4.3823	232.245	225.224
28059	4.3823	202.382	84.3163
28060	4.3823	237.222	250.553
28061	4.3823	265.077	27.2872
28062	4.3823	240.211	111.757
28063	4.3824	301.408	66.8578
28064	4.3825	272.625	319.756
28065	4.3831	208.407	149.998
28066	4.3831	194.583	136.008
28067	4.3831	228.616	89.4158
28068	4.3832	273.789	72.1345
28069	4.3833	262.098	214.944

28070	4.3835	276.769	115.672
28071	4.3839	257.527	352.657
28072	4.384	276.535	107.613
28073	4.3841	262.694	89.7374
28074	4.3841	244.996	164.496
28075	4.3843	173.639	318.717
28076	4.3843	226.692	232.299
28077	4.3845	257.097	122.376
28078	4.3845	271.298	125.078
28079	4.3847	223.138	218.017
28080	4.3847	176.416	263.511
28081	4.3848	258.243	124.751
28082	4.3849	273.354	81.453
28083	4.385	227.558	353.495
28084	4.3854	329.635	196.209
28085	4.3854	239.715	159.715
28086	4.3855	277.967	228.861
28087	4.3856	290.012	94.6235
28088	4.3856	275.726	199.943
28089	4.3856	270.503	39.5966
28090	4.3857	237.982	123.174
28091	4.3857	247.038	137.018
28092	4.3857	309.093	242.577
28093	4.3858	314.366	73.1232
28094	4.3861	271.133	37.0424
28095	4.3861	309.81	28.7225
28096	4.3863	273.174	129.411
28097	4.3864	255.766	131.577
28098	4.3865	225.66	274.732
28099	4.3865	242.797	53.2336
28100	4.3866	241.248	109.531
28101	4.3869	229.216	30.1543
28102	4.387	314.36	71.5833
28103	4.3871	211.094	226.483
28104	4.3871	178.236	179.646
28105	4.3872	313.296	66.4399
28106	4.3873	286.065	115.601
28107	4.3875	248.227	114.785
28108	4.3875	267.167	347.72
28109	4.3876	273.517	182.967
28110	4.3877	296.46	165.013
28111	4.3878	263.513	69.1303
28112	4.3878	207.545	189.709
28113	4.3879	262.063	61.6305
28114	4.3881	224.217	88.0042
28115	4.3881	248.823	246.888
28116	4.3882	276.978	133.855
28117	4.3882	293.386	239.223

28118	4.3884	272.543	308.901
28119	4.3885	235.087	172.947
28120	4.3885	263.464	176.27
28121	4.3886	233.443	77.2114
28122	4.3887	230.627	121.711
28123	4.3888	245.028	107.901
28124	4.3891	266.455	32.1234
28125	4.3892	216.839	130.848
28126	4.3892	256.519	160.897
28127	4.3892	221.006	115.499
28128	4.3892	232.674	283.116
28129	4.3894	304.133	65.2178
28130	4.3895	240.78	219.516
28131	4.3896	262.403	297.806
28132	4.3899	221.562	192.472
28133	4.3899	266.13	42.7753
28134	4.39	294.98	166.872
28135	4.39	252.247	309.266
28136	4.3902	260.387	67.465
28137	4.3902	294.388	53.5259
28138	4.3903	260.101	129.318
28139	4.3905	287.397	64.9077
28140	4.3905	223.958	96.6501
28141	4.3905	279.465	279.429
28142	4.3905	238.585	165.94
28143	4.3906	258.802	264.798
28144	4.3906	201.982	167.603
28145	4.3908	314.605	308.058
28146	4.3908	193.467	304.167
28147	4.3908	450.11	309.79
28148	4.391	300.856	107.702
28149	4.391	255.357	121.625
28150	4.3911	222.678	277.445
28151	4.3914	279.99	292.488
28152	4.3917	274.011	319.042
28153	4.3917	225.545	121.372
28154	4.3918	282.067	96.2016
28155	4.3919	223.939	154.036
28156	4.3919	105.609	141.404
28157	4.3923	230.629	140.921
28158	4.3924	242.817	74.4436
28159	4.3924	206.733	155.789
28160	4.3924	211.365	172.467
28161	4.3925	149.499	209.357
28162	4.3926	270.792	141.54
28163	4.3926	221.13	179.633
28164	4.3926	247.563	160.857
28165	4.3928	239.922	118.716

28166	4.3929	247.167	77.3234
28167	4.3931	249.236	303.206
28168	4.3931	292.015	356.477
28169	4.3932	217.729	114.57
28170	4.3934	244.255	61.3808
28171	4.3934	273.593	23.0312
28172	4.3937	199.117	252.665
28173	4.3937	227.109	85.344
28174	4.3938	297.202	47.2766
28175	4.3939	268.776	278.127
28176	4.3939	235.095	124.04
28177	4.394	213.251	269.412
28178	4.394	211.557	164.02
28179	4.3941	213.238	304.145
28180	4.3941	232.904	114.852
28181	4.3941	242.209	234.88
28182	4.3941	319.302	18.9265
28183	4.3944	304.622	166.247
28184	4.3945	216.313	136.022
28185	4.3945	186.293	281.411
28186	4.3947	240.23	104.591
28187	4.3948	255.887	276.196
28188	4.3949	218.119	128.763
28189	4.3949	311.633	207.626
28190	4.395	221.717	263.151
28191	4.3951	268.433	151.246
28192	4.3952	168.782	193.925
28193	4.3953	184.814	89.4898
28194	4.3954	240.626	173.356
28195	4.3955	252.196	91.8169
28196	4.3958	394.28	243.448
28197	4.3958	250.197	168.37
28198	4.3958	236.935	255.071
28199	4.3959	253.659	354.15
28200	4.3959	257.873	29.2932
28201	4.3959	265.077	348.981
28202	4.3962	262.368	105.334
28203	4.3962	258.988	231.922
28204	4.3962	250.926	139.393
28205	4.3963	330.458	39.0773
28206	4.3964	299.63	188.078
28207	4.3964	216.657	193.545
28208	4.3964	183.487	39.0144
28209	4.3966	181.965	259.174
28210	4.3966	295.656	7.8349
28211	4.3967	272.583	60.656
28212	4.3969	265.614	223.403
28213	4.3969	278.579	232.211

28214	4.3969	227.865	104.644
28215	4.3972	278.277	152.346
28216	4.3973	254.632	185.618
28217	4.3975	272.33	66.0893
28218	4.3976	276.132	192.628
28219	4.3976	238.101	294.566
28220	4.3976	265.497	128.242
28221	4.3977	302.824	220.305
28222	4.3978	289.685	103.11
28223	4.3978	219.348	75.4478
28224	4.3979	311.127	46.1436
28225	4.3979	215.028	256.927
28226	4.398	176.948	201.207
28227	4.3981	251.717	228.921
28228	4.3981	251.951	125.035
28229	4.3981	169.275	89.4199
28230	4.3982	266.302	88.4581
28231	4.3983	207.744	296.84
28232	4.3983	261.243	123.777
28233	4.3985	240.202	188.387
28234	4.3985	268.583	111.457
28235	4.3986	324.326	55.5736
28236	4.3989	241.987	65.2376
28237	4.3989	212.712	3.2146
28238	4.3989	295.161	139.694
28239	4.3992	168.146	170.683
28240	4.3994	283.289	17.1164
28241	4.3994	200.539	170.375
28242	4.3995	266.342	58.474
28243	4.3998	332.439	80.2297
28244	4.4	324.734	33.1594
28245	4.4	282.21	237.37
28246	4.4	251.364	82.7136
28247	4.4001	178.657	156.978
28248	4.4001	204.319	124.915
28249	4.4001	273.006	20.4563
28250	4.4002	228.051	33.6308
28251	4.4003	310.543	57.7063
28252	4.4003	261.96	160.087
28253	4.4004	214.871	220.62
28254	4.4004	306.514	307.992
28255	4.4005	189.593	117.799
28256	4.4006	294.287	78.6241
28257	4.4006	301.197	81.5267
28258	4.4007	222.952	271.034
28259	4.4007	267.577	222.041
28260	4.4007	195.227	161.37
28261	4.4008	337.504	32.5212

28262	4.4012	28.9436	59.4107
28263	4.4013	312.139	245.858
28264	4.4013	240.322	202.641
28265	4.4014	251.43	317.856
28266	4.4016	254.976	224.26
28267	4.4018	261.29	35.0197
28268	4.4019	286.941	138.446
28269	4.4019	228.1	83.0878
28270	4.402	162.035	186.984
28271	4.402	206.556	295.631
28272	4.4021	246.122	161.061
28273	4.4022	198.871	126.127
28274	4.4024	-2.752	9.3187
28275	4.4025	237.99	236.208
28276	4.4026	310.161	43.2304
28277	4.4027	246.02	146.431
28278	4.4028	278.644	81.5839
28279	4.4028	228.227	256.71
28280	4.4029	236.817	284.825
28281	4.403	243.083	189.105
28282	4.403	289.794	103.691
28283	4.4032	265.637	113.085
28284	4.4033	222.874	87.1467
28285	4.4035	258.088	85.2072
28286	4.4035	255.642	135.553
28287	4.4036	269.003	141.802
28288	4.4036	226.259	182.416
28289	4.4037	304.346	61.8202
28290	4.4038	323.925	65.33
28291	4.4039	244.468	155.013
28292	4.4039	250.251	172.294
28293	4.404	294.756	78.3817
28294	4.4041	197.844	80.1562
28295	4.4042	236.363	16.4712
28296	4.4043	270.222	51.5499
28297	4.4044	223.602	144.538
28298	4.4045	238.606	110.406
28299	4.4047	360.261	356.339
28300	4.4047	-2.8877	120.022
28301	4.4047	248.12	269.91
28302	4.4047	254.84	317.953
28303	4.4049	266.044	78.7318
28304	4.4049	236.378	110.374
28305	4.4051	192.886	231.974
28306	4.4051	280.185	50.6252
28307	4.4052	277.145	84.333
28308	4.4052	276.695	75.4887
28309	4.4053	229.175	81.4441

28310	4.4053	250.835	107.776
28311	4.4054	285.312	161.488
28312	4.4055	224.698	29.0907
28313	4.4055	257.116	56.6983
28314	4.4056	194.297	71.0214
28315	4.4057	190.837	269.47
28316	4.4057	249.859	70.7371
28317	4.4057	295.593	177.498
28318	4.4057	296.666	296.763
28319	4.4058	277.104	85.5091
28320	4.4058	221.287	264.636
28321	4.4058	241.61	251.427
28322	4.4058	259.277	112.151
28323	4.4059	290.544	266.131
28324	4.4059	271.562	64.6056
28325	4.406	270.233	76.1026
28326	4.4061	251.006	350.87
28327	4.4061	230.179	123.292
28328	4.4062	256.391	187.916
28329	4.4066	239.618	30.2626
28330	4.4067	274.237	92.2153
28331	4.4068	226.434	184.103
28332	4.4069	242.04	109.804
28333	4.4071	282.683	98.3403
28334	4.4074	326.897	21.1394
28335	4.4076	192.908	167.337
28336	4.4077	271.8	138.139
28337	4.4077	55.5399	100.263
28338	4.4077	293.535	60.6968
28339	4.4077	244.145	111.523
28340	4.4077	260.323	16.9018
28341	4.4078	243.515	124.204
28342	4.4079	207.585	138.438
28343	4.408	186.232	347.897
28344	4.4081	233.308	160.582
28345	4.4083	262.433	18.646
28346	4.4085	218.025	237.168
28347	4.4085	208.294	176.035
28348	4.4087	253.394	179.383
28349	4.4087	193.174	72.9532
28350	4.4088	190.854	8.4936
28351	4.4089	245.574	119.219
28352	4.4089	256.666	165.942
28353	4.4092	302.468	104.986
28354	4.4092	240.71	136.004
28355	4.4092	272.091	58.7697
28356	4.4095	249.117	161.708
28357	4.4099	239.649	272.427

28358	4.4099	217.798	148.288
28359	4.4099	239.019	17.7856
28360	4.4099	182.804	153.864
28361	4.4101	225.166	228.555
28362	4.4102	246.864	237.076
28363	4.4102	248.16	55.8462
28364	4.4103	276.987	216.008
28365	4.4106	263.689	174.649
28366	4.4106	263.267	54.9261
28367	4.4107	227.86	115.465
28368	4.4109	147.31	140.672
28369	4.4109	321.593	5.3986
28370	4.4109	266.316	36.9214
28371	4.4112	251.043	118.372
28372	4.4114	272.417	93.4121
28373	4.4116	279.47	75.5929
28374	4.4116	221.499	252.296
28375	4.412	247.332	257.069
28376	4.412	301.34	60.5921
28377	4.4121	241.594	64.2696
28378	4.4122	257.306	171.518
28379	4.4122	195.539	117.44
28380	4.4123	183.675	151.917
28381	4.4125	214.928	132.314
28382	4.4126	212.999	350.774
28383	4.4128	261.328	332.077
28384	4.413	243.7	215.426
28385	4.4131	256.237	228.455
28386	4.4132	226.046	240.836
28387	4.4132	207.678	219
28388	4.4134	216.943	112.929
28389	4.4137	244.616	218.148
28390	4.4137	205.987	115.329
28391	4.4138	172.18	240.917
28392	4.4138	315.683	314.618
28393	4.414	330.937	30.9923
28394	4.414	276.669	35.0382
28395	4.4142	272.622	78.3171
28396	4.4142	212.788	62.4892
28397	4.4142	187.526	126.517
28398	4.4142	291.35	303.947
28399	4.4145	234.188	210.173
28400	4.4147	268.482	27.4154
28401	4.4152	262.238	200.804
28402	4.4152	281.835	64.0506
28403	4.4153	216.196	102.946
28404	4.4154	248.288	180.883
28405	4.4155	248.546	140.216

28406	4.4158	264.346	80.8905
28407	4.4158	265.867	136.923
28408	4.4158	297.605	87.148
28409	4.4159	375.949	312.266
28410	4.4159	214.799	241.523
28411	4.4159	234.121	119.857
28412	4.416	243.227	72.8237
28413	4.4161	269.355	34.5892
28414	4.4161	315.689	66.225
28415	4.4162	297.39	139.861
28416	4.4162	218.526	156.648
28417	4.4163	201.442	137.617
28418	4.4163	245.988	125.335
28419	4.4165	280.726	252.185
28420	4.4168	159.064	16.9028
28421	4.4168	243.928	130.196
28422	4.4169	244.637	130.124
28423	4.4169	212.034	256.691
28424	4.417	213.367	49.3465
28425	4.4172	250.459	119.378
28426	4.4173	318.18	240.63
28427	4.4174	193.3	110.717
28428	4.4174	241.041	62.7709
28429	4.4174	261.416	159.832
28430	4.4174	277.512	317.768
28431	4.4175	256.275	105.795
28432	4.4177	296.442	85.1569
28433	4.4177	247.19	82.6375
28434	4.4177	255.811	94.0028
28435	4.4177	278.832	118.052
28436	4.4178	262.608	79.6504
28437	4.4179	278.068	278.494
28438	4.418	249.984	76.8503
28439	4.4183	297.511	328.45
28440	4.4184	271.43	94.4278
28441	4.4186	199.709	344.097
28442	4.4186	230.648	257.962
28443	4.4187	296.826	89.9203
28444	4.4187	226.527	86.8586
28445	4.4187	215.497	223.964
28446	4.4187	265.644	189.185
28447	4.4188	269.084	301.563
28448	4.4188	213.656	26.4155
28449	4.4188	241.411	124.798
28450	4.419	256.562	110.034
28451	4.4191	297.898	41.4213
28452	4.4191	-2.7946	97.7716
28453	4.4191	269.729	316.801

28454	4.4192	219.077	189.994
28455	4.4192	309.95	65.6399
28456	4.4192	257.996	110.669
28457	4.4193	286.455	123.175
28458	4.4193	233.764	169.425
28459	4.4194	209.703	184.293
28460	4.4194	225.15	262.824
28461	4.4198	281.223	351.97
28462	4.4198	273.37	234.233
28463	4.4199	294.607	93.2422
28464	4.4201	240.27	23.6364
28465	4.4201	274.694	159.287
28466	4.4202	268.304	64.3721
28467	4.4202	284.401	81.5942
28468	4.4202	300.907	248.706
28469	4.4203	307.983	266.761
28470	4.4203	268.805	52.4123
28471	4.4204	220.514	152.51
28472	4.4205	227.239	38.8939
28473	4.4206	267.301	108.352
28474	4.4207	262.701	232.058
28475	4.4208	312.336	109.759
28476	4.421	346.688	48.1219
28477	4.421	273.895	162.396
28478	4.4213	204.89	126.373
28479	4.4214	309.08	95.8302
28480	4.4214	272.863	129.75
28481	4.4214	293.03	117.091
28482	4.4215	291.206	92.8873
28483	4.4215	265.795	235.218
28484	4.4216	297.574	66.9637
28485	4.4216	226.403	149.996
28486	4.4217	256.066	224.68
28487	4.4217	290.192	97.8495
28488	4.4218	179.598	222.259
28489	4.4218	255.401	85.7063
28490	4.422	221.322	245.806
28491	4.4221	234.183	185.775
28492	4.4221	239.436	178.954
28493	4.4222	346.335	82.5763
28494	4.4222	230.853	149.535
28495	4.4224	210.598	126.127
28496	4.4226	243.966	133.983
28497	4.4228	198.39	166.602
28498	4.4229	308.348	91.9403
28499	4.4231	332.56	68.5377
28500	4.4231	218.063	107.103
28501	4.4231	235.271	333.491

28502	4.4232	262.066	301.971
28503	4.4232	308.777	2.209
28504	4.4233	212.099	167.735
28505	4.4233	305.895	74.6446
28506	4.4233	197.841	119.05
28507	4.4233	218.852	41.8212
28508	4.4234	284.015	258.173
28509	4.4234	252.172	105.635
28510	4.4235	247.475	50.2724
28511	4.4236	222.641	300.698
28512	4.4236	240.257	131.16
28513	4.4236	330.11	74.0155
28514	4.4237	239.006	192.447
28515	4.4237	251.946	122.196
28516	4.4238	189.377	232.406
28517	4.4239	207.915	280.333
28518	4.4239	255.342	159.919
28519	4.4241	229.479	128.366
28520	4.4243	291.127	84.4068
28521	4.4244	277.013	144.94
28522	4.4245	222.46	57.2724
28523	4.4245	194.042	162.362
28524	4.4245	239.503	0.4798
28525	4.4246	290.149	146.12
28526	4.4246	264.591	184.282
28527	4.4247	239.366	297.427
28528	4.4247	308.574	78.0181
28529	4.4247	166.581	184.817
28530	4.4248	247.569	174.568
28531	4.425	224.411	221.814
28532	4.4252	293.543	75.1554
28533	4.4252	244.836	37.8206
28534	4.4253	234.59	226.865
28535	4.4253	281.684	162.384
28536	4.4254	272.576	233.794
28537	4.4254	225.676	139.261
28538	4.4254	247.172	139.497
28539	4.4256	295.641	151.306
28540	4.4257	331.316	259.026
28541	4.4257	216.797	124.084
28542	4.4257	223.82	51.195
28543	4.4258	253.211	119.771
28544	4.4261	225.888	128.967
28545	4.4262	221.787	77.5188
28546	4.4262	270.473	23.094
28547	4.4263	244.566	170.016
28548	4.4263	293.113	23.6412
28549	4.4265	273.895	290.098

28550	4.4265	249.282	132.513
28551	4.4266	225.586	303.921
28552	4.4267	270.045	106.008
28553	4.4267	304.439	228.889
28554	4.4268	344.912	284.509
28555	4.4268	324.518	292.891
28556	4.4269	282.661	65.2762
28557	4.427	185.898	114.938
28558	4.4271	250.855	292.859
28559	4.4271	310.177	75.3305
28560	4.4272	209.5	250.046
28561	4.4273	235.652	85.5637
28562	4.4273	229.126	126.624
28563	4.4273	334.689	51.0762
28564	4.4274	212.058	351.788
28565	4.4276	310.557	305.567
28566	4.4277	257.214	84.3325
28567	4.4277	235.846	113.533
28568	4.4279	405.801	82.6696
28569	4.4281	333.778	18.1957
28570	4.4282	267.877	86.2014
28571	4.4283	275.825	103.904
28572	4.4284	232.104	63.8694
28573	4.4285	260.169	152.606
28574	4.4287	293.704	355.64
28575	4.4289	310.922	335.046
28576	4.4292	191.634	148.243
28577	4.4293	261.226	72.4522
28578	4.4293	194.005	115.176
28579	4.4295	242.003	117.192
28580	4.4298	292.899	86.2464
28581	4.4298	188.569	127.871
28582	4.4299	218.343	334.151
28583	4.4301	225.596	186.363
28584	4.4302	287.734	161.218
28585	4.4303	304.283	290.621
28586	4.4304	241.887	86.4473
28587	4.4306	272.57	178.126
28588	4.4306	198.925	108.893
28589	4.4307	289.201	13.1591
28590	4.4308	344.767	242.888
28591	4.4309	227.911	133.028
28592	4.431	285.645	77.6463
28593	4.4312	319.013	124.172
28594	4.4312	198.435	73.3454
28595	4.4313	252.402	118.922
28596	4.4313	255.559	126.818
28597	4.4314	245.922	220.878

28598	4.4314	225.795	150.645
28599	4.4314	244.8	102.861
28600	4.4315	199.338	269.003
28601	4.4316	294.626	68.5839
28602	4.4317	236.241	25.8429
28603	4.4318	234.976	302.505
28604	4.4318	248.663	111.698
28605	4.4319	258.675	241.541
28606	4.4321	174.73	191.758
28607	4.4321	278.683	21.9261
28608	4.4322	286.333	23.5578
28609	4.4324	269.465	141.039
28610	4.4324	202.943	130.318
28611	4.4325	272.039	141.994
28612	4.4327	236.587	302.633
28613	4.4327	304.737	169.355
28614	4.4329	202.894	111.406
28615	4.433	312.571	346.135
28616	4.4331	241.996	268.884
28617	4.4332	254.579	144.496
28618	4.4332	214.888	103.814
28619	4.4333	262.999	211.17
28620	4.4334	196.155	112.249
28621	4.4335	252.113	159.628
28622	4.4335	236.134	166.941
28623	4.4335	238.09	253.345
28624	4.4336	252.712	172.962
28625	4.4337	276.848	39.3343
28626	4.4338	300.364	115.539
28627	4.4338	231.403	225.101
28628	4.4339	217.86	104.081
28629	4.4339	238.867	119.258
28630	4.4341	235.044	248.117
28631	4.4344	248.939	63.5892
28632	4.4345	319.768	56.5338
28633	4.4346	387.144	129.409
28634	4.4346	276.849	17.6786
28635	4.4347	299.145	152.24
28636	4.4347	237.647	115.903
28637	4.4347	255.524	241.331
28638	4.4348	302.399	301.538
28639	4.4349	234.453	147.864
28640	4.4349	285.441	322.393
28641	4.4351	239.296	30.7325
28642	4.4351	211.481	108.864
28643	4.4353	325.711	31.2517
28644	4.4353	248.528	212.579
28645	4.4354	257.812	179.281

28646	4.4354	267.23	90.4867
28647	4.4354	303.898	341.614
28648	4.4355	281.327	122.182
28649	4.4356	243.636	133.197
28650	4.4356	282.786	225.85
28651	4.4356	302.718	285
28652	4.4357	219.844	94.189
28653	4.436	316.451	81.0276
28654	4.436	216.845	107.893
28655	4.436	263.146	326.256
28656	4.4363	312.188	202.085
28657	4.4363	270.308	144.427
28658	4.4365	251.374	38.8086
28659	4.4365	259.754	84.4153
28660	4.4366	246.557	174.07
28661	4.4366	262.914	86.2451
28662	4.4371	258.435	47.2663
28663	4.4372	251.357	180.133
28664	4.4372	219.14	168.451
28665	4.4373	234.991	107.6
28666	4.4374	253.291	221.389
28667	4.4377	225.714	103.751
28668	4.4377	289.034	12.0285
28669	4.4378	243.418	132.19
28670	4.438	243.097	99.31
28671	4.4381	324.247	209.748
28672	4.4385	202.463	155.144
28673	4.4386	267.974	33.9142
28674	4.4387	275.261	105.248
28675	4.4387	221.277	113.933
28676	4.4387	207.082	112.126
28677	4.439	272.583	219.422
28678	4.4391	252.618	90.1305
28679	4.4392	236.385	105.056
28680	4.4393	119.037	168.467
28681	4.4395	336.854	24.9313
28682	4.4396	242.629	36.1693
28683	4.4396	243.556	120.452
28684	4.4397	282.147	217.55
28685	4.4398	225.404	162.688
28686	4.4399	303.111	65.8171
28687	4.4399	216.194	149.143
28688	4.4399	238.68	183.247
28689	4.4399	203.543	296.687
28690	4.4402	295.558	243.728
28691	4.4403	215.679	117.924
28692	4.4403	228.332	82.287
28693	4.4403	268.781	198.363

28694	4.4405	210.132	249.868
28695	4.4408	323.893	104.397
28696	4.4408	259.055	309.319
28697	4.4409	252.501	187.578
28698	4.4412	333.68	14.9012
28699	4.4412	323.819	328.947
28700	4.4412	247.445	106.794
28701	4.4412	280.969	91.4497
28702	4.4413	303.202	101.265
28703	4.4414	180.252	169.985
28704	4.4415	288.154	320.118
28705	4.4415	245.931	178.844
28706	4.4418	311.156	257.595
28707	4.4419	293.796	67.7688
28708	4.4419	220.099	95.9125
28709	4.442	315.315	63.0754
28710	4.4421	278.056	158.473
28711	4.4423	307.83	274.181
28712	4.4423	233.244	300.964
28713	4.4423	253.608	17.5024
28714	4.4426	236.091	177.053
28715	4.4427	235.405	185.746
28716	4.4427	245.785	76.0329
28717	4.443	168.735	231.805
28718	4.4431	277.898	10.9911
28719	4.4431	266.435	66.5102
28720	4.4434	269.29	49.7905
28721	4.4435	243.359	41.0358
28722	4.4435	290.218	51.5258
28723	4.4436	244.66	116.86
28724	4.4438	347.826	129.418
28725	4.4438	305.355	77.5528
28726	4.4442	221.758	231.621
28727	4.4442	199.25	168.664
28728	4.4442	231.963	149.973
28729	4.4443	257.656	80.7137
28730	4.4443	268.055	112.258
28731	4.4443	308.303	38.4594
28732	4.4445	344.515	36.3841
28733	4.4445	314.766	286.708
28734	4.4448	230.23	188.608
28735	4.4448	266.823	125.428
28736	4.4451	297.09	88.9798
28737	4.4451	252.068	232.597
28738	4.4451	226.848	145.829
28739	4.4453	270.027	359.301
28740	4.4453	254.029	305.568
28741	4.4453	252.93	111.339

28742	4.4453	242.339	235.71
28743	4.4453	243.767	184.226
28744	4.4454	237.404	46.7224
28745	4.4454	218.488	164.148
28746	4.4455	273.114	90.4671
28747	4.4456	293.321	53.5906
28748	4.4458	195.728	39.7373
28749	4.446	273.894	87.175
28750	4.446	270.287	168.992
28751	4.4461	205.474	252.29
28752	4.4463	292.069	306.392
28753	4.4463	195.277	0.0977
28754	4.4464	288.48	119.817
28755	4.4465	291.197	216.415
28756	4.4465	250.171	261.581
28757	4.4465	238.782	121.444
28758	4.4467	243.107	223.111
28759	4.4468	306.139	20.6103
28760	4.4468	240.913	240.078
28761	4.4469	285.502	163.366
28762	4.447	262.382	37.9309
28763	4.447	236.66	203.307
28764	4.4471	241.965	143.423
28765	4.4473	211.361	84.053
28766	4.4473	183.673	159.315
28767	4.4473	223.131	109.988
28768	4.4474	280.037	60.8542
28769	4.4474	213.268	118.21
28770	4.4475	253.241	154.787
28771	4.4475	235.468	170.407
28772	4.4475	217.592	102.551
28773	4.4475	215.452	80.1609
28774	4.4477	289.494	124.469
28775	4.4479	170.087	295.569
28776	4.448	253.94	75.3162
28777	4.4481	239.141	185.073
28778	4.4482	198.211	215.871
28779	4.4482	237.36	96.1623
28780	4.4482	285.14	76.1827
28781	4.4482	235.756	281.796
28782	4.4484	274.394	108.931
28783	4.4485	290.708	324.358
28784	4.4485	322.112	98.0671
28785	4.4485	241.91	303.468
28786	4.4486	317.037	184.295
28787	4.4487	218.409	182.824
28788	4.4487	330.548	27.5672
28789	4.4489	243.945	124.414

28790	4.449	265.981	38.9157
28791	4.4491	290.859	240.276
28792	4.4491	198.074	105.793
28793	4.4491	306.023	56.5384
28794	4.4492	269.611	179.717
28795	4.4494	295.297	120.409
28796	4.4495	332.92	39.0492
28797	4.4495	290.942	7.1598
28798	4.4495	189.735	143.883
28799	4.4495	228.511	4.0485
28800	4.4498	278.27	212.046
28801	4.4498	299.415	77.4952
28802	4.4499	303.169	69.6909
28803	4.45	235.591	180.31
28804	4.45	233.271	223.277
28805	4.45	279.432	73.0528
28806	4.4501	257.604	298.201
28807	4.4502	238.209	176.881
28808	4.4503	282.705	174.679
28809	4.4503	301.983	4.8227
28810	4.4503	235.17	232.907
28811	4.4503	259.233	38.8998
28812	4.4504	348.563	46.874
28813	4.4504	108.858	187.025
28814	4.4505	272.686	219.622
28815	4.4506	264.809	274.931
28816	4.4506	260.322	111.198
28817	4.4509	249.003	167.267
28818	4.451	221.631	279.335
28819	4.451	311.394	206.86
28820	4.4511	194.597	77.9802
28821	4.4513	252.689	96.1985
28822	4.4514	287.075	100.024
28823	4.4517	326.088	49.9624
28824	4.4517	209.224	230.583
28825	4.4518	252.846	20.2984
28826	4.4518	255.108	279.211
28827	4.452	350.556	32.564
28828	4.452	309.406	348.789
28829	4.452	288.539	316.641
28830	4.4522	214.93	174.092
28831	4.4524	281.085	269.129
28832	4.4525	283.72	151.162
28833	4.4525	236.624	46.1421
28834	4.4526	256.411	140.827
28835	4.4526	284.31	108.434
28836	4.4528	267.163	107.214
28837	4.4528	259.286	135.123

28838	4.4528	279.019	171.642
28839	4.4529	254.773	264.575
28840	4.4529	205.352	139.841
28841	4.453	278.612	110.013
28842	4.453	237.872	169.933
28843	4.4531	241.983	103.743
28844	4.4531	297.51	48.54
28845	4.4532	227.127	297.266
28846	4.4532	253.525	194.628
28847	4.4532	253.568	37.8772
28848	4.4532	242.714	66.2527
28849	4.4533	274.35	123.162
28850	4.4535	283.69	21.9808
28851	4.4536	218.911	180.413
28852	4.4536	263.881	198.908
28853	4.4537	340.958	124.578
28854	4.4539	230.317	114.452
28855	4.454	220.877	177.088
28856	4.4543	267.763	265.451
28857	4.4543	267.377	172.252
28858	4.4544	279.92	15.4025
28859	4.4544	261.36	177.3
28860	4.4545	219.193	105.597
28861	4.4546	261.762	155.668
28862	4.4548	276.625	111.746
28863	4.4549	278.18	143.892
28864	4.4549	298.646	79.9045
28865	4.455	211.206	111.86
28866	4.4552	247.783	117.082
28867	4.4552	255.13	208.269
28868	4.4553	279.692	46.9591
28869	4.4553	258.403	70.8783
28870	4.4554	264.919	257.446
28871	4.4555	339.246	329.864
28872	4.4557	230.356	158.048
28873	4.4557	267.133	177.272
28874	4.4559	315.582	65.1046
28875	4.4559	253.453	79.9762
28876	4.4559	173.414	88.7419
28877	4.4559	258.928	163.32
28878	4.456	355.723	73.2692
28879	4.456	227.397	173.179
28880	4.4561	314.545	137.617
28881	4.4563	202.642	125.071
28882	4.4564	338.071	92.6079
28883	4.4565	244.008	137.009
28884	4.4566	262.061	194.5
28885	4.4566	319.335	327.81

28886	4.4568	218.407	19.4244
28887	4.4569	272.957	344.255
28888	4.4569	253.148	230.876
28889	4.4569	252.107	236.237
28890	4.4569	204.348	152.191
28891	4.4572	318.42	54.7582
28892	4.4572	281.747	233.317
28893	4.4573	219.941	29.9287
28894	4.4573	454.191	310.907
28895	4.4575	257.432	242.931
28896	4.4578	290.352	21.1749
28897	4.458	243.692	122.475
28898	4.4581	244.605	153.384
28899	4.4584	115.035	30.8906
28900	4.4584	311.516	65.785
28901	4.4584	260.982	274.705
28902	4.4585	333.976	66.052
28903	4.4587	322.282	82.9078
28904	4.4588	285.01	47.1532
28905	4.4588	195.547	338.541
28906	4.4589	313.635	27.3749
28907	4.459	199.607	214.872
28908	4.459	248.05	129.344
28909	4.4591	217.472	160.411
28910	4.4591	276.015	247.821
28911	4.4593	306.981	279.947
28912	4.4593	255.41	80.618
28913	4.4593	207.354	59.5515
28914	4.4594	281.695	80.8581
28915	4.4594	313.067	239.823
28916	4.4595	205.824	177.68
28917	4.4598	232.139	303.173
28918	4.4598	244.784	160.455
28919	4.4603	341.746	37.1259
28920	4.4604	284.782	86.0827
28921	4.4604	179.138	180.991
28922	4.4606	280.794	317.092
28923	4.4606	232.645	257.474
28924	4.4606	282.407	76.8282
28925	4.4607	320.405	98.2033
28926	4.4608	221.205	67.4722
28927	4.4608	236.018	105.087
28928	4.4608	234.85	191.358
28929	4.4609	219.948	124.81
28930	4.4609	275.619	106.658
28931	4.4611	363.81	202.758
28932	4.4611	280.274	142.193
28933	4.4612	318.018	291.262

28934	4.4613	316.315	83.7687
28935	4.4614	224.15	257.989
28936	4.4614	276.069	19.622
28937	4.4615	308.76	100.909
28938	4.4615	274.615	192.35
28939	4.4615	260.204	129.17
28940	4.4616	-2.8025	141.509
28941	4.4617	263.956	143.586
28942	4.4618	199.593	180.63
28943	4.462	273.223	159.273
28944	4.4621	246.367	109.531
28945	4.4622	237.011	71.8002
28946	4.4623	294.715	80.0675
28947	4.4625	276.423	197.955
28948	4.4627	274.514	277.531
28949	4.4628	277.382	266.092
28950	4.4629	266.675	135.299
28951	4.4629	298.772	74.1684
28952	4.463	272.814	163.669
28953	4.4631	286.571	9.9396
28954	4.4632	256.859	33.9347
28955	4.4633	244.513	158.742
28956	4.4634	248.755	238.078
28957	4.4634	274.734	103.03
28958	4.4636	342.52	72.6561
28959	4.4637	258.379	98.8785
28960	4.464	260.894	176.734
28961	4.464	282.229	133.889
28962	4.4641	277.773	169.768
28963	4.4642	273.66	265.128
28964	4.4642	280.034	258.523
28965	4.4643	250.469	179.107
28966	4.4644	327.975	179.972
28967	4.4645	208.397	158.843
28968	4.4645	232.411	219.86
28969	4.4645	283.446	125.679
28970	4.4647	219.353	261.39
28971	4.4647	179.013	9.7785
28972	4.4647	245.629	104.457
28973	4.4647	281.308	293.37
28974	4.4649	71.4053	16.3949
28975	4.465	308.234	133.624
28976	4.465	287.264	144.947
28977	4.465	213.722	193.287
28978	4.465	275.094	344.842
28979	4.4651	238.358	44.1726
28980	4.4654	310.871	325.762
28981	4.4656	291.191	79.8647

28982	4.4656	222.316	258.637
28983	4.4657	293.712	250.825
28984	4.4657	184.028	157.286
28985	4.4658	252.874	186.175
28986	4.4659	288.63	50.2355
28987	4.4659	188.486	196.704
28988	4.4659	200.788	253.944
28989	4.466	250.523	148.844
28990	4.4664	302.63	97.1357
28991	4.4665	235.689	169.859
28992	4.4668	268.062	154.834
28993	4.4668	226.344	280.954
28994	4.4669	229.03	171.807
28995	4.467	302.112	84.7071
28996	4.4671	199.061	222.742
28997	4.4671	269.752	68.2633
28998	4.4671	329.737	29.2872
28999	4.4672	232.287	158.447
29000	4.4673	259.722	42.6164
29001	4.4673	271.198	93.8835
29002	4.4675	255.899	110.906
29003	4.4677	266.49	40.7728
29004	4.4678	205.801	105.778
29005	4.4678	205.152	139.394
29006	4.4679	310.978	49.1913
29007	4.4681	233.077	120.751
29008	4.4682	200.934	145.624
29009	4.4682	276.51	106.653
29010	4.4682	312.488	113.982
29011	4.4682	317.494	228.517
29012	4.4685	317.947	280.94
29013	4.4686	282.191	235.226
29014	4.4686	267.504	72.2271
29015	4.4686	237.037	79.6546
29016	4.4687	194.092	236.552
29017	4.4687	232.835	147.914
29018	4.4687	286.515	159.682
29019	4.4687	246.492	245.947
29020	4.4688	240.839	172.369
29021	4.469	320.173	106.008
29022	4.469	309.125	49.2691
29023	4.4691	335.152	35.9791
29024	4.4692	304.704	192.013
29025	4.4692	321.913	30.5196
29026	4.4696	335.172	35.2541
29027	4.4696	292.39	236.054
29028	4.4701	219.215	116.462
29029	4.4701	258.764	17.4301

29030	4.4702	245.336	69.7091
29031	4.4703	267.947	172.039
29032	4.4703	257.207	79.9788
29033	4.4704	128.387	23.5348
29034	4.4704	182.641	136.693
29035	4.4705	236.488	148.007
29036	4.4706	241.438	80.0377
29037	4.4706	270.92	57.5664
29038	4.4706	315.201	89.9038
29039	4.4707	217.136	33.5761
29040	4.4707	276.866	75.189
29041	4.4709	302.556	67.0207
29042	4.471	312.423	126.141
29043	4.4712	245.602	88.4331
29044	4.4713	300.138	261.8
29045	4.4713	255.316	151.58
29046	4.4714	295.112	113.81
29047	4.4714	207.775	182.106
29048	4.4715	295.075	162.37
29049	4.4716	255.285	118.615
29050	4.4716	230.76	55.8915
29051	4.4717	268.28	83.5424
29052	4.4717	220.295	196.797
29053	4.4717	254.636	170.45
29054	4.4718	240.134	347.265
29055	4.4718	243.869	185.955
29056	4.4718	260.269	100.76
29057	4.4719	280.143	37.8934
29058	4.4719	267.157	163.698
29059	4.472	223.451	214.781
29060	4.4721	239.599	126.024
29061	4.4722	323.896	254.36
29062	4.4722	270.072	102.229
29063	4.4722	307.848	115.183
29064	4.4722	312.012	101.156
29065	4.4723	249.59	140.89
29066	4.4724	233.457	21.776
29067	4.4724	304.677	75.934
29068	4.4724	309.096	308.559
29069	4.4726	159.853	209.554
29070	4.4726	285.8	172.898
29071	4.4727	102.995	202.923
29072	4.4727	242.817	169.261
29073	4.4727	244.722	284.023
29074	4.4728	237.084	183.746
29075	4.4728	291.105	232.968
29076	4.4729	304.105	240.418
29077	4.473	282.332	64.1165

29078	4.4731	324.093	51.231
29079	4.4733	239.552	315.551
29080	4.4734	297.529	207.206
29081	4.4734	252.318	357.651
29082	4.4734	258.233	87.9804
29083	4.4737	252.599	133.657
29084	4.4738	250.994	291.97
29085	4.4738	271.305	122.747
29086	4.4738	279.776	65.1644
29087	4.474	290.933	201.284
29088	4.474	257.483	152.949
29089	4.474	248.844	124.749
29090	4.4742	344.152	206.849
29091	4.4743	218.495	227.532
29092	4.4743	237.723	138.446
29093	4.4743	284.009	179.255
29094	4.4744	231.302	157.32
29095	4.4744	223.889	339.813
29096	4.4745	196.582	147.054
29097	4.4745	322.134	293.159
29098	4.4746	271.987	155.143
29099	4.4747	267.106	97.294
29100	4.4748	299.916	84.3221
29101	4.4749	238.506	291.554
29102	4.4749	253.535	162.767
29103	4.475	226.594	88.094
29104	4.4752	203.753	78.8494
29105	4.4753	274.343	108.348
29106	4.4753	250.673	108.84
29107	4.4753	307.302	346.974
29108	4.4754	221.172	198.053
29109	4.4756	250.945	81.6184
29110	4.4759	353.178	203.723
29111	4.476	300.371	356.791
29112	4.476	254.187	150.686
29113	4.476	216.969	129.616
29114	4.476	238.73	108.337
29115	4.4761	411.541	310.314
29116	4.4761	305.887	298.672
29117	4.4761	152.216	202.52
29118	4.4761	269.79	85.4856
29119	4.4764	353.769	256.132
29120	4.4764	205.81	171.421
29121	4.4766	337.549	71.7721
29122	4.4766	309.548	93.9782
29123	4.4769	243.22	291.604
29124	4.477	220.445	86.872
29125	4.477	263.747	75.0827

29126	4.477	261.336	20.473
29127	4.477	305.201	95.7951
29128	4.4771	254.26	184.107
29129	4.4772	233.028	129.99
29130	4.4774	323.161	104.929
29131	4.4776	239.786	122.681
29132	4.4778	264.624	84.0577
29133	4.4779	267.098	82.9684
29134	4.4781	243.034	246.95
29135	4.4781	222.28	224.305
29136	4.4782	360.763	50.7252
29137	4.4782	281.111	122.464
29138	4.4782	175.825	136.653
29139	4.4782	263.484	36.0711
29140	4.4783	276.366	202.034
29141	4.4783	294.072	332.489
29142	4.4783	268.641	77.4788
29143	4.4784	210.412	30.3882
29144	4.4784	255.71	60.1993
29145	4.4784	246.394	108.833
29146	4.4785	238.799	258.319
29147	4.4787	259.464	268.658
29148	4.4787	274.546	60.7478
29149	4.4789	247.198	222.543
29150	4.4789	192.007	210.211
29151	4.4792	256.467	251.082
29152	4.4793	243.81	170.83
29153	4.4794	275.571	124.365
29154	4.4797	237.015	172.47
29155	4.4798	228.668	119.184
29156	4.4799	224.88	91.7027
29157	4.48	261.634	154.345
29158	4.4801	269.762	262.833
29159	4.4801	268.781	173.957
29160	4.4801	285.121	69.1342
29161	4.4803	302.809	227.087
29162	4.4803	279.33	233.13
29163	4.4804	220.298	144.774
29164	4.4805	216.905	181.286
29165	4.4805	203.751	291.406
29166	4.4807	274.844	182.616
29167	4.4807	293.852	115.473
29168	4.4809	231.107	226.359
29169	4.4809	221.681	98.2223
29170	4.4809	346.05	47.7944
29171	4.4809	323.952	85.3806
29172	4.481	247.747	175.893
29173	4.4812	286.206	254.595

29174	4.4812	269.61	119.604
29175	4.4813	322.12	194.192
29176	4.4813	259.103	247.515
29177	4.4814	214.99	62.0096
29178	4.4815	269.425	12.2382
29179	4.4815	220.551	101.577
29180	4.4816	278.492	138.421
29181	4.4816	275.945	319.916
29182	4.4816	305.731	319.413
29183	4.4816	303.707	305.717
29184	4.4817	255.432	193.671
29185	4.4817	193.666	150.544
29186	4.4817	286.766	103.226
29187	4.4818	249.978	255.366
29188	4.4819	231.034	230.814
29189	4.482	316.263	22.0607
29190	4.4821	226.9	316.042
29191	4.4822	313.73	82.6906
29192	4.4823	223.697	171.1
29193	4.4823	305.193	89.4887
29194	4.4823	250.761	75.5167
29195	4.4823	258.423	155.377
29196	4.4825	306.291	321.387
29197	4.4826	230.893	181.244
29198	4.4827	56.3213	15.6633
29199	4.4828	307.707	86.1542
29200	4.483	263.695	38.297
29201	4.4831	272.396	303.431
29202	4.4831	239.092	173.526
29203	4.4831	222.209	101.951
29204	4.4831	300.002	288.621
29205	4.4832	339.26	22.4872
29206	4.4832	291.43	123.753
29207	4.4832	291.208	97.333
29208	4.4832	299.915	115.628
29209	4.4835	260.223	181.077
29210	4.4835	267.626	134.749
29211	4.4835	315.542	180.018
29212	4.4836	286.137	141.126
29213	4.4836	256.59	283.191
29214	4.4839	304.495	115.973
29215	4.4839	211.742	119.036
29216	4.484	216.463	271.814
29217	4.4841	320.639	199.669
29218	4.4842	194.726	156.467
29219	4.4843	303.012	92.2771
29220	4.4844	256.177	111.676
29221	4.4844	222.075	133.873

29222	4.4844	252.774	148.026
29223	4.4845	309.717	83.7063
29224	4.4845	269.764	315.062
29225	4.4846	253.484	252.752
29226	4.4847	302.923	300.81
29227	4.4848	251.956	75.6441
29228	4.4848	250.023	96.6542
29229	4.4848	251.558	294.391
29230	4.4849	232.307	229.365
29231	4.4852	225.112	102.569
29232	4.4854	259.249	257.241
29233	4.4855	225.113	217.938
29234	4.4855	269.185	165.966
29235	4.4856	320.182	125.673
29236	4.4857	248.754	2.6697
29237	4.4858	293.047	217.589
29238	4.4858	228.058	107.791
29239	4.4858	296.007	65.1562
29240	4.4858	330.817	67.8946
29241	4.4858	308.195	36.9684
29242	4.486	269.893	171.314
29243	4.486	234.358	92.0268
29244	4.486	289.792	219.369
29245	4.4862	246.282	194.442
29246	4.4862	191.904	199.194
29247	4.4863	305.375	109.112
29248	4.4863	268.432	160.358
29249	4.4863	267.379	170.834
29250	4.4863	241.368	32.8664
29251	4.4866	297.777	190.158
29252	4.4867	256.307	289.099
29253	4.4867	263.744	119.337
29254	4.4869	227.268	239.297
29255	4.4869	244.565	110.97
29256	4.4873	264.299	111.75
29257	4.4875	244.346	122.857
29258	4.4876	296.447	39.8274
29259	4.4877	238.452	144.767
29260	4.4878	317.596	350.536
29261	4.4878	283.831	15.4485
29262	4.4878	295.184	293.153
29263	4.4879	368.702	82.2489
29264	4.488	275.911	330.094
29265	4.4881	239.229	120.359
29266	4.4883	271.859	127.647
29267	4.4888	314.549	45.0034
29268	4.4889	281.888	125.14
29269	4.4891	307.689	48.6557

29270	4.4892	203.072	122.832
29271	4.4893	210.148	260.128
29272	4.4894	276.669	177.948
29273	4.4895	296.54	109.23
29274	4.4898	262.399	191.094
29275	4.4899	310.495	291.615
29276	4.49	246.628	98.9444
29277	4.49	313.238	105.242
29278	4.4901	348.666	63.3289
29279	4.4902	264.507	171.473
29280	4.4902	253.143	174.617
29281	4.4904	236.092	127.491
29282	4.4905	303.449	70.6365
29283	4.4906	335.515	227.105
29284	4.4906	228.311	19.9374
29285	4.4906	327.283	81.0801
29286	4.4906	271.228	356.381
29287	4.4911	307.85	55.9123
29288	4.4912	294.453	64.9292
29289	4.4912	322.313	87.9004
29290	4.4913	235.363	95.5891
29291	4.4915	278.891	68.0049
29292	4.4916	304.979	216.905
29293	4.4917	316.659	33.1151
29294	4.4918	299.19	231.43
29295	4.4919	314.734	319.505
29296	4.4923	320.189	237.064
29297	4.4923	319.396	32.0387
29298	4.4924	257.25	65.0282
29299	4.4925	286.923	36.4013
29300	4.4926	215.599	255.844
29301	4.4927	285.343	132.733
29302	4.4928	311.854	141.136
29303	4.4929	218.655	182.972
29304	4.4929	280.638	7.0607
29305	4.493	314.874	89.8882
29306	4.493	224.778	270.994
29307	4.4931	295.176	328.668
29308	4.4931	295.084	54.7079
29309	4.4932	277.745	37.1084
29310	4.4932	241.44	130.077
29311	4.4935	321.798	133.727
29312	4.4939	295.906	33.2068
29313	4.494	234.747	196.575
29314	4.4941	294.002	61.4195
29315	4.4941	294.052	10.2356
29316	4.4942	289.011	94.0499
29317	4.4942	266.575	273.503

29318	4.4943	275.152	61.3896
29319	4.4943	281.037	41.5363
29320	4.4944	281.586	307.629
29321	4.4946	227.495	194.977
29322	4.4946	246.199	134.087
29323	4.4949	303.32	186.711
29324	4.495	220.064	120.01
29325	4.4952	316.335	75.0136
29326	4.4952	249.707	268.647
29327	4.4953	269.348	104.986
29328	4.4955	231.197	217.82
29329	4.4955	275.029	280.23
29330	4.4955	278.909	115.877
29331	4.4956	291.009	217.526
29332	4.4956	275.963	304.674
29333	4.4957	212.78	187.281
29334	4.4958	327.83	66.6438
29335	4.4958	300.247	269.336
29336	4.4959	287.927	76.3038
29337	4.4962	331.442	65.7195
29338	4.4962	267.856	244.793
29339	4.4963	270.191	35.127
29340	4.4963	294.867	307.452
29341	4.4963	207.265	106.679
29342	4.4964	341.605	16.3466
29343	4.4965	277.913	163.611
29344	4.4965	215.973	220.314
29345	4.4968	281.425	64.6806
29346	4.4968	289.441	134.941
29347	4.497	229.193	342.098
29348	4.4971	239.339	229.212
29349	4.4971	301.934	90.1179
29350	4.4972	320.301	64.0645
29351	4.4973	278.193	20.1832
29352	4.4973	328.711	256.164
29353	4.4973	298.442	26.2495
29354	4.4974	232.856	110.969
29355	4.4975	312.817	125.729
29356	4.4976	239.095	85.1942
29357	4.4977	292.825	117.815
29358	4.4977	276.659	49.0045
29359	4.4977	249.709	100.516
29360	4.4978	256.944	33.2281
29361	4.498	298.797	191.853
29362	4.498	323.444	198.257
29363	4.4981	238.495	145.965
29364	4.4982	253.888	192.682
29365	4.4983	345.664	44.6258

29366	4.4984	177.803	264.345
29367	4.4985	376.029	36.3362
29368	4.4985	226.096	219.224
29369	4.4988	226.898	164.861
29370	4.4988	260.729	154.195
29371	4.4988	236.378	123.994
29372	4.4989	294.219	267.888
29373	4.4991	277.149	90.1645
29374	4.4992	201.709	156.368
29375	4.4994	260.545	27.2339
29376	4.4995	276.417	66.5316
29377	4.4995	279.855	129.643
29378	4.4997	304.626	138.808
29379	4.4997	304.396	103.187
29380	4.4998	273.227	73.2666
29381	4.4998	244.478	138.195
29382	4.4998	226.713	143.315
29383	4.4998	311.395	317.143
29384	4.5001	333.014	145.854
29385	4.5001	189.373	172.484
29386	4.5002	233.112	47.4946
29387	4.5003	264.489	61.0028
29388	4.5003	281.419	231.424
29389	4.5003	299.649	120.146
29390	4.5003	293.37	274.673
29391	4.5004	330.969	68.8822
29392	4.5007	252.47	183.597
29393	4.5007	268.812	38.9785
29394	4.5007	322.858	230.851
29395	4.5008	302.112	39.6065
29396	4.5009	204.715	192.736
29397	4.501	366.723	90.1771
29398	4.501	231.925	73.3415
29399	4.501	274.788	65.9838
29400	4.5011	263.021	83.4964
29401	4.5011	220.284	47.3401
29402	4.5012	250.67	227.502
29403	4.5013	255.117	188.555
29404	4.5013	296.851	27.7782
29405	4.5013	266.772	177.631
29406	4.5013	222.649	252.674
29407	4.5013	246.421	311.436
29408	4.5013	293.051	31.6853
29409	4.5015	200.705	208.537
29410	4.5015	278.249	216.05
29411	4.5015	317.747	31.6889
29412	4.5017	345.136	69.0582
29413	4.5017	193.295	224.217

29414	4.5019	265.198	125.606
29415	4.5019	270.644	181.116
29416	4.502	244.462	328.371
29417	4.5021	239.753	147.7
29418	4.5021	240.126	337.931
29419	4.5022	251.921	121.342
29420	4.5023	274.748	168.46
29421	4.5024	290.58	59.5602
29422	4.5024	287.421	280.734
29423	4.5025	314.325	75.5525
29424	4.5025	267.345	272.934
29425	4.5025	157.991	152.598
29426	4.5026	291.524	105.354
29427	4.5028	284.774	299.942
29428	4.5029	333.798	143.724
29429	4.5029	214.749	130.177
29430	4.5029	267.277	142.526
29431	4.503	197.109	65.4532
29432	4.5031	249.869	231.416
29433	4.5031	264.867	47.029
29434	4.5032	249.269	317.77
29435	4.5032	280.615	88.8427
29436	4.5032	216.162	80.3324
29437	4.5032	240.729	36.3496
29438	4.5033	217.778	222.989
29439	4.5034	245.698	181.697
29440	4.5038	355.518	84.3119
29441	4.5039	350.559	37.079
29442	4.5041	291.656	350.538
29443	4.5042	237.46	63.9851
29444	4.5045	261.367	255.587
29445	4.5046	321.553	70.9834
29446	4.5047	277.999	116.036
29447	4.5047	280.3	262.226
29448	4.5047	256.497	225.37
29449	4.5047	266.837	235.883
29450	4.5048	295.709	65.8466
29451	4.5048	259.29	191.428
29452	4.5049	272.862	349.486
29453	4.505	291.904	111.229
29454	4.505	247.446	73.4808
29455	4.505	296.239	89.019
29456	4.505	334.08	235.606
29457	4.5051	315.422	83.3243
29458	4.5051	309.333	150.901
29459	4.5052	261.714	270.122
29460	4.5053	374.66	21.7274
29461	4.5054	201.343	107.816

29462	4.5058	303.391	217.172
29463	4.5061	313.728	13.1331
29464	4.5062	245.525	224.861
29465	4.5064	283.458	280.231
29466	4.5064	275.028	122.628
29467	4.5064	210.408	241.88
29468	4.5065	300.708	234.998
29469	4.5065	288.525	91.4565
29470	4.5066	359.513	44.6831
29471	4.5066	242.66	221.314
29472	4.5067	271.461	45.9467
29473	4.5069	323.603	74.5326
29474	4.5069	303.575	2.0168
29475	4.507	286.548	101.4
29476	4.507	361.493	289.616
29477	4.5071	349.452	191.123
29478	4.5071	208.653	169.679
29479	4.5071	256.288	236.761
29480	4.5072	282.042	181.045
29481	4.5073	303.254	301.3
29482	4.5073	236.501	138.986
29483	4.5073	272.049	94.8569
29484	4.5075	335.823	44.8492
29485	4.5076	199.672	102.091
29486	4.5078	260.904	232.262
29487	4.5078	243.008	190.648
29488	4.5079	273.768	62.4043
29489	4.508	291.53	269.435
29490	4.5082	248.443	32.0165
29491	4.5085	271.753	139.365
29492	4.5085	314.744	80.1459
29493	4.5086	343.394	66.4638
29494	4.5087	313.622	321.948
29495	4.5089	272.036	247.491
29496	4.509	296.695	302.053
29497	4.509	230.87	303.804
29498	4.5092	246.38	129.661
29499	4.5092	246.115	158.905
29500	4.5092	325.96	137.69
29501	4.5093	242.288	182.676
29502	4.5093	64.7116	129.003
29503	4.5094	295.207	137.944
29504	4.5094	225.193	190.485
29505	4.5094	144.053	34.4539
29506	4.5095	236.422	46.2817
29507	4.5096	370.937	224.548
29508	4.5096	264.073	87.4442
29509	4.5098	285.291	153.471

29510	4.51	301.913	171.93
29511	4.5101	267.342	96.1185
29512	4.5101	301.77	291.704
29513	4.5102	339.89	47.1941
29514	4.5103	327.04	1.5459
29515	4.5103	238.752	124.761
29516	4.5103	315.371	66.7206
29517	4.5104	312.516	61.6912
29518	4.5104	270.821	319.541
29519	4.5106	267.823	103.85
29520	4.5107	413.866	356.896
29521	4.5108	225.078	196.035
29522	4.5108	250.641	146.639
29523	4.5108	265.558	82.7357
29524	4.5109	325.044	149.938
29525	4.5109	328.169	45.295
29526	4.511	296.203	131.851
29527	4.5111	281.007	87.7911
29528	4.5112	290.22	186.466
29529	4.5112	280.22	19.8379
29530	4.5113	248.426	183.409
29531	4.5113	293.885	94.5331
29532	4.5114	253.374	89.0298
29533	4.5114	234.645	99.4622
29534	4.5114	294.067	291.235
29535	4.5114	323.329	29.072
29536	4.5114	312.047	201.372
29537	4.5115	278.235	280.413
29538	4.5116	258.396	195.788
29539	4.5119	283.783	128.984
29540	4.512	310.154	178.326
29541	4.512	228.973	61.8503
29542	4.5121	305.246	272.922
29543	4.5121	322.841	146.274
29544	4.5121	298.284	326.308
29545	4.5121	233.935	303.257
29546	4.5124	352.649	188.717
29547	4.5124	265.828	76.5389
29548	4.5125	315.053	231.891
29549	4.5126	281.899	60.5642
29550	4.5127	311.74	322.786
29551	4.5127	253.955	169.422
29552	4.5128	297.928	275.022
29553	4.5128	339.492	188.001
29554	4.5131	280.841	104.948
29555	4.5132	187.404	222.079
29556	4.5132	310.975	104.836
29557	4.5133	286.685	186.827

29558	4.5135	237.085	276.816
29559	4.5136	317.907	170.522
29560	4.5137	248.9	191.561
29561	4.5138	257.791	48.5594
29562	4.5138	251.853	279.679
29563	4.5139	226.096	121.82
29564	4.5139	264.286	26.4214
29565	4.514	310.648	146.895
29566	4.514	300.847	186.079
29567	4.5143	301.351	103.328
29568	4.5143	299.02	103.682
29569	4.5144	320.97	94.2095
29570	4.5144	269.47	274.752
29571	4.5144	290.714	62.4158
29572	4.5145	289.838	80.2013
29573	4.5146	230.661	255.616
29574	4.5146	224.376	98.7649
29575	4.5149	291.128	45.6731
29576	4.5151	301.797	59.3001
29577	4.5151	289.28	217.028
29578	4.5153	365.708	27.7163
29579	4.5154	329.667	16.276
29580	4.5156	331.007	200.889
29581	4.5157	303.18	290.36
29582	4.5158	292.869	91.086
29583	4.516	252.981	102.792
29584	4.516	298.95	316.014
29585	4.5163	293.042	272.166
29586	4.5163	251.599	106.561
29587	4.5163	37.0602	103.221
29588	4.5163	335.609	80.6104
29589	4.5165	304.976	104.294
29590	4.5165	214.205	227.487
29591	4.5166	249.521	135.111
29592	4.5167	319.325	271.881
29593	4.517	291.235	114.308
29594	4.517	281.418	147.714
29595	4.5171	248.369	160.123
29596	4.5171	217.382	173.036
29597	4.5173	228.003	209.833
29598	4.5173	225.428	115.011
29599	4.5174	310.984	248.825
29600	4.5174	306.498	58.9049
29601	4.5175	277.967	184.163
29602	4.5176	264.841	135.192
29603	4.5176	258.959	53.2925
29604	4.5177	299.605	113.097
29605	4.5178	272.536	161.854

29606	4.518	221.848	0.5096
29607	4.518	-2.7148	257.878
29608	4.5183	-2.7493	247.361
29609	4.5184	248.812	110.271
29610	4.5186	255.793	311.233
29611	4.519	272.369	57.2941
29612	4.5192	244.675	175.87
29613	4.5193	274.574	231.644
29614	4.5194	205.843	314.741
29615	4.5195	263.051	279.835
29616	4.5195	257.87	146.342
29617	4.5195	279.932	276.531
29618	4.5196	251.139	163.731
29619	4.5198	284.835	258.043
29620	4.5199	279.934	197.276
29621	4.52	255.444	324.224
29622	4.5203	327.35	278.47
29623	4.5203	268.504	172.703
29624	4.5203	317.101	234.892
29625	4.5205	258.584	187.592
29626	4.5205	233.581	219.411
29627	4.5206	315.603	124.811
29628	4.5207	9.9543	116.237
29629	4.5209	319.067	66.9991
29630	4.5209	271.466	133.07
29631	4.5209	315.722	331.72
29632	4.5212	240.041	236.823
29633	4.5212	220.144	35.5486
29634	4.5212	269.07	87.562
29635	4.5214	331.572	80.3856
29636	4.5214	172.769	212.674
29637	4.5215	230.171	155.558
29638	4.5217	242.31	134.384
29639	4.5217	291.633	163.09
29640	4.5218	307.135	193.978
29641	4.5221	337.832	38.4327
29642	4.5221	270.274	252.034
29643	4.5221	328.25	32.3602
29644	4.5222	286.554	216.383
29645	4.5223	284.194	37.2499
29646	4.5225	336.943	318.522
29647	4.5225	342.81	332.893
29648	4.5226	184.723	228.466
29649	4.5228	266.211	130.122
29650	4.5229	309.586	73.9189
29651	4.523	304.726	291.025
29652	4.523	277.046	163.523
29653	4.523	220.017	77.4172

29654	4.5231	324.81	17.9903
29655	4.5232	326.49	46.6799
29656	4.5232	184.9	82.3531
29657	4.5232	249.988	330.684
29658	4.5232	266.753	134.43
29659	4.5233	298.87	72.3937
29660	4.5234	266.87	234.871
29661	4.5234	341.603	201.403
29662	4.5235	340.027	63.9472
29663	4.5236	257.854	257.006
29664	4.5236	264.462	134.879
29665	4.5237	252.707	132.65
29666	4.5237	252.048	105.402
29667	4.5238	319.813	258.763
29668	4.524	293.616	147.839
29669	4.5241	284.095	24.342
29670	4.5241	298.873	98.5654
29671	4.5242	336.232	94.012
29672	4.5243	238.229	302.36
29673	4.5243	277.125	105.474
29674	4.5243	219.12	32.6663
29675	4.5245	274.598	64.0691
29676	4.5245	278.259	94.3076
29677	4.5247	291.899	104.6
29678	4.5249	312.147	314.791
29679	4.5251	369.297	252.342
29680	4.5251	296.124	273.255
29681	4.5252	310.555	54.1296
29682	4.5253	273.336	306.748
29683	4.5255	308.46	55.5041
29684	4.5258	297.612	123.717
29685	4.5259	243.001	190.452
29686	4.526	196.668	105.798
29687	4.526	312.147	157.203
29688	4.5261	292.101	102.241
29689	4.5261	260.63	170.041
29690	4.5261	189.457	252.128
29691	4.5261	-2.9877	127.573
29692	4.5261	300.176	281.717
29693	4.5262	278.806	94.3392
29694	4.5262	287.52	135.992
29695	4.5263	295.879	67.647
29696	4.5263	276.803	134.265
29697	4.5264	270.381	287.141
29698	4.5265	242.756	294.501
29699	4.5266	306.203	265.086
29700	4.5266	226.882	123.464
29701	4.5267	204.609	258.862

29702	4.5267	242.869	185.521
29703	4.5267	258.871	171.889
29704	4.5268	324.854	319.117
29705	4.5269	288.88	280.504
29706	4.5269	272.667	270.532
29707	4.5269	383.173	78.439
29708	4.5271	364.929	128.093
29709	4.5271	273.522	33.2478
29710	4.5271	340.802	21.626
29711	4.5272	390.673	221.688
29712	4.5272	253.696	357.78
29713	4.5273	350.88	307.45
29714	4.5274	271.038	24.7409
29715	4.5275	285.971	34.9889
29716	4.5276	264.314	94.6608
29717	4.5277	348.151	15.0371
29718	4.5277	282.851	137.985
29719	4.5277	329.425	41.8687
29720	4.5278	239.524	100.387
29721	4.5278	248.886	179.256
29722	4.5279	-2.7846	103.73
29723	4.5279	320.608	63.7303
29724	4.5282	334.269	92.2867
29725	4.5283	304.787	280.461
29726	4.5285	208.872	164.669
29727	4.5289	223.241	104.922
29728	4.5291	279.483	148.706
29729	4.5292	335.21	81.8193
29730	4.5294	278.448	142.397
29731	4.5295	236.75	126.466
29732	4.5296	257.147	300.343
29733	4.5296	272.797	166.53
29734	4.5296	8.4839	65.1805
29735	4.5298	432.624	309.749
29736	4.5299	348.693	63.059
29737	4.53	276.971	56.3891
29738	4.5301	-2.7834	99.0799
29739	4.5302	357.975	34.2872
29740	4.5302	318.725	22.5151
29741	4.5303	221.969	28.9075
29742	4.5303	296.769	273.802
29743	4.5304	90.4675	143.043
29744	4.5305	322.14	57.1917
29745	4.5305	288.789	132.82
29746	4.5306	252.569	266.757
29747	4.5307	358.998	41.5166
29748	4.5307	350.127	287.624
29749	4.5308	295.261	302.11

29750	4.5309	345.787	319.323
29751	4.5314	325.873	159.572
29752	4.5317	240.026	113.282
29753	4.5318	266.342	122.678
29754	4.532	188.037	54.037
29755	4.5321	309.015	91.7087
29756	4.5321	219.843	212.516
29757	4.5322	232.442	304.165
29758	4.5322	288.396	253.194
29759	4.5322	323.003	272.607
29760	4.5324	236.103	119.141
29761	4.5324	235.039	172.452
29762	4.5325	304.202	166.107
29763	4.5326	262.042	116.275
29764	4.5326	334.774	345.093
29765	4.5328	318.02	130.909
29766	4.5329	296.643	83.3569
29767	4.533	296.904	172.791
29768	4.5331	265.041	233.749
29769	4.5332	357.534	53.8934
29770	4.5332	237.004	133.959
29771	4.5333	270.998	161.858
29772	4.5333	280.739	128.262
29773	4.5334	-2.8309	195.31
29774	4.5335	259.89	132.543
29775	4.5336	286.838	87.4212
29776	4.5336	314.788	176.591
29777	4.5337	293.583	53.1382
29778	4.5338	275.183	93.4681
29779	4.5339	263.256	27.9383
29780	4.5339	211.891	99.5814
29781	4.5339	238.496	222.777
29782	4.534	282.55	235.415
29783	4.534	269.106	153.725
29784	4.5344	282.617	229.57
29785	4.5346	256.683	258.432
29786	4.5348	228.51	249.901
29787	4.5348	252.575	20.2336
29788	4.5348	316.934	67.075
29789	4.5349	250.162	97.4936
29790	4.535	245.888	184.05
29791	4.535	274.556	198.637
29792	4.535	261.633	127.545
29793	4.535	284.303	167.523
29794	4.5351	297.423	154.201
29795	4.5351	236.497	32.5929
29796	4.5352	223.137	172.843
29797	4.5353	272.143	300.344

29798	4.5354	324.322	89.657
29799	4.5354	365.013	35.4945
29800	4.5354	320.436	243.55
29801	4.5355	307.116	188.444
29802	4.5356	293.59	55.4651
29803	4.5357	230.926	335.911
29804	4.5357	298.965	287.614
29805	4.5358	366.693	281.756
29806	4.5358	222.209	238.48
29807	4.5359	334.829	74.0629
29808	4.536	286.001	276.005
29809	4.536	280.437	197.905
29810	4.5363	267.471	70.6089
29811	4.5366	365.974	43.2846
29812	4.5366	257.915	104.628
29813	4.5366	262.197	101.128
29814	4.5367	329.248	258.586
29815	4.5369	288.568	126.465
29816	4.5369	263.597	109.297
29817	4.5373	316.569	36.4184
29818	4.5374	271.177	141.884
29819	4.5375	244.415	130.018
29820	4.5375	221.434	181.346
29821	4.5375	297.659	82.9214
29822	4.5375	273.105	154.726
29823	4.5378	257.646	147.57
29824	4.5379	229.296	153.121
29825	4.538	233.898	176.068
29826	4.5382	361.274	45.6228
29827	4.5384	369.171	59.0627
29828	4.5384	331.037	357.762
29829	4.5385	255.881	154.884
29830	4.5385	294.695	50.8144
29831	4.5387	248.285	279.45
29832	4.5388	297.97	20.5305
29833	4.539	285.006	183.705
29834	4.539	310.06	89.2453
29835	4.5391	294.938	75.7819
29836	4.5391	278.305	140.163
29837	4.5392	297.183	97.5975
29838	4.5394	234.368	212.407
29839	4.5397	321.736	235.489
29840	4.54	346.208	35.0157
29841	4.54	253.927	278.476
29842	4.5401	319.424	206.531
29843	4.5401	218.123	240.053
29844	4.5402	308.495	141.986
29845	4.5402	337.627	53.4279

29846	4.5403	351.301	46.7165
29847	4.5404	235.073	187.909
29848	4.5404	258.075	137.574
29849	4.5404	255.367	252.241
29850	4.5404	254.303	164.285
29851	4.5405	256.953	236.087
29852	4.5405	245.444	167.692
29853	4.5405	267.008	135.285
29854	4.5406	263.511	77.8968
29855	4.5407	303.109	242.63
29856	4.5407	311.523	270.816
29857	4.5409	233.496	113.202
29858	4.541	256.399	236.254
29859	4.5413	243.654	172.898
29860	4.5413	210.155	278.983
29861	4.5413	249.531	322.788
29862	4.5416	329.771	149.704
29863	4.5417	246.106	50.4273
29864	4.5417	193.729	177.014
29865	4.5417	255.318	232.483
29866	4.5418	281.55	267.94
29867	4.5418	276.508	130.864
29868	4.5419	280.055	193.659
29869	4.5419	200.442	180.825
29870	4.5419	220.956	111.381
29871	4.542	278.357	181.978
29872	4.542	283.651	111.484
29873	4.5421	288.361	164.05
29874	4.5421	314.518	140.407
29875	4.5422	366.387	271.193
29876	4.5422	249.548	115.486
29877	4.5422	350.476	97.0834
29878	4.5423	373.304	288.263
29879	4.5423	285.101	176.001
29880	4.5425	305.997	220.075
29881	4.5427	289.77	253.004
29882	4.5428	386.257	310.557
29883	4.5429	333.558	83.3906
29884	4.543	286.36	268.706
29885	4.543	240.851	145.147
29886	4.5431	299.38	90.4921
29887	4.5434	270.318	170.046
29888	4.5435	-2.7392	245.699
29889	4.5436	360.682	46.0286
29890	4.5438	368.311	311.039
29891	4.5439	376.353	269.788
29892	4.544	264.081	229.066
29893	4.544	286.224	109.088

29894	4.5444	463.879	310.048
29895	4.5444	222.318	274.983
29896	4.5449	280.668	128.397
29897	4.5449	232.653	153.86
29898	4.545	270.057	170.095
29899	4.545	313.745	74.1972
29900	4.5451	275.593	113.288
29901	4.5451	262.215	122.851
29902	4.5454	251.983	173.879
29903	4.5455	247.53	306.144
29904	4.5456	211.61	162.112
29905	4.5457	319.262	58.5408
29906	4.5458	259.196	126.029
29907	4.5459	346.859	256.752
29908	4.546	314.137	88.3426
29909	4.5461	251.11	121.162
29910	4.5462	-2.8178	116.214
29911	4.5463	309.907	115.715
29912	4.5463	239.297	144.545
29913	4.5463	174.184	174.986
29914	4.5465	267.967	90.072
29915	4.5465	345.732	38.4191
29916	4.5467	264.562	226.899
29917	4.547	-4.3821	47.4372
29918	4.5471	249.313	355.089
29919	4.5471	335.665	295.79
29920	4.5472	271.535	191.973
29921	4.5472	248.688	87.8749
29922	4.5474	316.855	71.7071
29923	4.5475	246.827	3.4659
29924	4.5475	326.89	72.9597
29925	4.5476	282.142	89.6276
29926	4.5476	301.182	237.004
29927	4.5477	243.037	102.508
29928	4.5478	256.602	107.348
29929	4.5479	308.278	90.9356
29930	4.548	239.474	249.449
29931	4.5482	375.148	46.2635
29932	4.5482	192.752	171.567
29933	4.5483	263.989	65.5743
29934	4.5483	288.677	247.735
29935	4.5486	281.514	79.6449
29936	4.5486	239.185	222.738
29937	4.5486	343.173	238.681
29938	4.5487	283.848	189.569
29939	4.549	278.922	218.023
29940	4.549	395.897	305.446
29941	4.5491	302.74	104.73

29942	4.5491	310.529	33.0996
29943	4.5492	223.917	7.619
29944	4.5493	221.683	122.992
29945	4.5493	332.535	59.3264
29946	4.5493	342.442	182.004
29947	4.5493	344.991	76.5077
29948	4.5494	294.971	36.7715
29949	4.5496	280.779	5.6739
29950	4.5499	275.473	195.03
29951	4.5499	336.672	230.479
29952	4.55	268.196	120.603
29953	4.55	277.812	27.3895
29954	4.5501	336.347	73.3919
29955	4.5502	288.885	177.13
29956	4.5502	330.929	42.0931
29957	4.5503	234.017	176.878
29958	4.5503	288.943	114.945
29959	4.5506	320.661	116.228
29960	4.5508	309.115	101.659
29961	4.5511	279.876	107.941
29962	4.5512	287.895	50.8626
29963	4.5512	283.913	125.412
29964	4.5513	304.625	5.2773
29965	4.5514	231.137	120.2
29966	4.5514	275.415	128.277
29967	4.5515	336.277	262.16
29968	4.5517	303.185	59.0673
29969	4.5517	274.478	226.668
29970	4.5519	309.142	135.941
29971	4.5519	226.698	129.721
29972	4.5519	304.492	155.306
29973	4.5523	268.626	184.891
29974	4.5523	267.276	274.716
29975	4.5524	269.004	227.622
29976	4.5525	298.393	212.718
29977	4.5525	273.555	125.136
29978	4.5526	311.284	153.598
29979	4.5527	367.464	5.6989
29980	4.5527	301.167	253.493
29981	4.5528	311.826	116.163
29982	4.5528	275.39	147.422
29983	4.5529	291.107	137.132
29984	4.553	298.952	66.755
29985	4.553	232.549	176.953
29986	4.5531	379.032	3.3638
29987	4.5531	288.09	77.0757
29988	4.5532	282.426	152.716
29989	4.5532	299.575	14.6978

29990	4.5533	254.588	120.266
29991	4.5533	297.426	337.092
29992	4.5535	369.167	13.0792
29993	4.5535	311.964	81.3633
29994	4.5536	237.796	220.75
29995	4.5536	305.915	113.308
29996	4.5536	276.555	140.294
29997	4.5536	244.058	120.906
29998	4.5536	363.544	291.306
29999	4.5537	287.005	208.309
30000	4.554	304.156	117.337
30001	4.5543	225.513	174.051
30002	4.5544	308.912	130.965
30003	4.5545	282.322	174.652
30004	4.5545	273.346	119.635
30005	4.5545	253.351	133.574
30006	4.5546	319.877	99.0267
30007	4.5547	343.248	120.311
30008	4.5547	280.674	84.6085
30009	4.5547	287.75	202.826
30010	4.5547	289.058	62.7683
30011	4.5549	301.088	176.123
30012	4.5549	245.439	137.373
30013	4.5549	272.234	115.667
30014	4.5549	325.986	37.273
30015	4.555	247.574	121.508
30016	4.555	268.034	176.954
30017	4.555	243.84	135.543
30018	4.5551	242.416	241.275
30019	4.5552	319.515	195.882
30020	4.5552	265.162	231.965
30021	4.5552	-2.8127	189.77
30022	4.5553	325.387	155.858
30023	4.5554	370.551	269.525
30024	4.5554	288.74	68.2074
30025	4.5554	245.391	346.127
30026	4.5556	318.8	40.9421
30027	4.5556	324.934	106.636
30028	4.5559	245.59	162.622
30029	4.556	341.824	24.2774
30030	4.5567	182.442	84.6823
30031	4.5568	218.176	165.605
30032	4.5569	273.77	197.896
30033	4.557	312.172	287.123
30034	4.557	203.091	76.9862
30035	4.5572	269.332	251.224
30036	4.5572	269.107	21.7597
30037	4.5574	318.808	194.839

30038	4.5575	262.372	173.803
30039	4.5575	277.475	123.58
30040	4.5576	298.046	123.337
30041	4.5576	351.954	49.0003
30042	4.5578	244.408	197.718
30043	4.5579	283.506	117.143
30044	4.5579	266.504	55.5166
30045	4.558	181.811	4.0223
30046	4.558	240.285	111.889
30047	4.5581	237.319	197.908
30048	4.5581	275.718	179.28
30049	4.5581	309.068	109.203
30050	4.5581	249.184	279.368
30051	4.5582	224.346	141.316
30052	4.5583	306.954	41.4378
30053	4.5583	321.795	155.857
30054	4.5584	254.063	112.936
30055	4.5585	254.891	106.258
30056	4.5587	245.567	144.297
30057	4.5588	288.977	182.131
30058	4.5589	335.554	61.836
30059	4.5589	345.752	118.438
30060	4.559	318.68	106.194
30061	4.5591	298.977	159.594
30062	4.5591	234.805	26.9845
30063	4.5592	365.402	36.039
30064	4.5593	262.32	73.9366
30065	4.5594	339.007	76.6397
30066	4.5595	227.979	123.718
30067	4.5597	216.42	157.718
30068	4.5597	335.089	285.97
30069	4.5598	312.533	139.982
30070	4.5601	255.994	150.554
30071	4.5602	246.726	168.411
30072	4.5604	276.792	235.644
30073	4.5605	316.942	123.666
30074	4.5605	248.784	153.687
30075	4.5605	329.351	317.07
30076	4.5605	238.048	115.551
30077	4.5606	379.439	252.336
30078	4.5606	342.034	34.0482
30079	4.5609	282.441	323.711
30080	4.5609	282.465	123.407
30081	4.5609	325.678	115.177
30082	4.5609	288.239	296.061
30083	4.561	369.437	97.6898
30084	4.5612	358.497	71.0719
30085	4.5612	301.519	101.608

30086	4.5612	334.149	94.447
30087	4.5613	306.107	110.838
30088	4.5617	267.814	171.35
30089	4.5617	255.47	103.143
30090	4.5618	376.061	29.6774
30091	4.5618	263.794	315.116
30092	4.5619	199.475	196.073
30093	4.5619	268.066	116.194
30094	4.562	315.573	122.502
30095	4.562	316.461	74.9373
30096	4.5621	314.152	79.4797
30097	4.5621	328.378	63.2745
30098	4.5621	283.491	134.908
30099	4.5621	289.052	49.0012
30100	4.5627	230.735	173.297
30101	4.5627	278.415	201.709
30102	4.5627	239.338	129.332
30103	4.5628	191.359	37.8108
30104	4.5628	343.512	278.592
30105	4.5629	327.784	237.34
30106	4.5629	323.071	88.7032
30107	4.563	283.658	100.418
30108	4.563	305.41	60.1407
30109	4.5631	355.317	23.885
30110	4.5631	336.383	305.705
30111	4.5631	313.663	98.254
30112	4.5632	208.108	243.605
30113	4.5633	269.779	63.7443
30114	4.5634	256.389	198.421
30115	4.5639	247.984	202.331
30116	4.5639	352.731	243.617
30117	4.5642	296.113	84.5464
30118	4.5643	313.696	97.2433
30119	4.5644	274.014	251.249
30120	4.5644	295.667	17.7582
30121	4.5645	258.501	36.5217
30122	4.5645	258.995	113.938
30123	4.5645	368.793	25.195
30124	4.5646	319.07	95.1211
30125	4.5646	284.074	122.732
30126	4.5646	228.937	171.646
30127	4.5647	339.894	290.13
30128	4.5649	333.508	248.612
30129	4.5649	307.269	290.138
30130	4.565	258.905	232.952
30131	4.5651	351.3	332.733
30132	4.5651	380.069	47.6712
30133	4.5653	310.209	74.4666

30134	4.5656	384.258	40.592
30135	4.5656	253.689	331.524
30136	4.5657	301.025	13.8113
30137	4.5657	267.76	84.7271
30138	4.5658	324.557	114.623
30139	4.566	262.041	175.755
30140	4.5664	345.967	81.8768
30141	4.5665	282.921	136.555
30142	4.5665	291.964	81.9358
30143	4.5667	350.197	36.3147
30144	4.5668	262.657	180.505
30145	4.5668	346.492	242.263
30146	4.5668	269.776	166.724
30147	4.5668	334.177	47.6332
30148	4.5669	326.54	55.9058
30149	4.5669	296.43	152.436
30150	4.5669	340.292	112.51
30151	4.567	129.585	171.578
30152	4.567	275.133	118.654
30153	4.5671	284.369	248.252
30154	4.5671	251.818	171.719
30155	4.5672	327.586	70.2541
30156	4.5672	248.069	130.376
30157	4.5672	305.502	149.438
30158	4.5674	77.8065	21.6223
30159	4.5674	292.729	110.411
30160	4.5674	266.776	243.662
30161	4.5675	260.096	182.169
30162	4.5676	281.167	95.1803
30163	4.5677	325.916	75.428
30164	4.5677	263.681	79.0173
30165	4.5677	312.052	323.059
30166	4.5678	251.395	113.099
30167	4.568	328.411	146.393
30168	4.568	292.327	283.319
30169	4.568	286.658	70.86
30170	4.5682	330.447	110.268
30171	4.5684	356.465	130.93
30172	4.5684	282.384	121.333
30173	4.5685	242.784	217.361
30174	4.5685	285.991	220.509
30175	4.5686	305.367	236.751
30176	4.5687	341.227	91.9569
30177	4.5687	274.124	69.5833
30178	4.5687	293.185	107.487
30179	4.5687	255.047	195.339
30180	4.5687	310.926	75.2881
30181	4.5688	289.455	348.915

30182	4.5688	259.566	248.595
30183	4.569	269.075	198.489
30184	4.5691	258.778	234.947
30185	4.5692	339.929	208.961
30186	4.5695	270.519	195.768
30187	4.5696	308.301	169.896
30188	4.5696	299.484	127.078
30189	4.5698	332.039	243.757
30190	4.5698	355.854	45.3535
30191	4.5699	246.446	169.508
30192	4.5699	332.841	58.7356
30193	4.57	303.12	128.085
30194	4.5701	285.879	152.378
30195	4.5702	228.885	48.7732
30196	4.5702	303.465	39.7345
30197	4.5703	261.433	193.126
30198	4.5703	329.596	242.377
30199	4.5704	289.934	74.9714
30200	4.5704	304.944	112.472
30201	4.5705	302.743	75.7559
30202	4.5707	312.404	81.7043
30203	4.5709	362.673	269.595
30204	4.5709	225.228	110.612
30205	4.5709	281.04	80.9289
30206	4.571	343.159	82.5624
30207	4.571	303.197	117.723
30208	4.5711	325.328	194.476
30209	4.5712	279.148	39.9396
30210	4.5712	322.062	138.568
30211	4.5714	260.196	320.879
30212	4.5714	237.367	48.4476
30213	4.5714	308.274	81.6628
30214	4.5714	246.943	17.7612
30215	4.5714	301.707	137.259
30216	4.5716	301.583	128.564
30217	4.5716	204.423	120.983
30218	4.5717	286.09	175.069
30219	4.5718	366.511	60.0318
30220	4.5718	237.911	127.242
30221	4.5718	312.34	208.06
30222	4.5718	320.943	85.4325
30223	4.572	265.53	165.075
30224	4.5721	345.441	60.0587
30225	4.5721	299.765	73.1599
30226	4.5721	277.999	237.369
30227	4.5726	270.233	113.859
30228	4.5726	291.275	322.04
30229	4.5727	276.755	25.7362

30230	4.5727	312.489	155.833
30231	4.5729	362.01	3.6518
30232	4.573	277.126	81.8813
30233	4.573	259.359	43.309
30234	4.5732	257.871	229.793
30235	4.5732	231.687	243.54
30236	4.5733	334.663	125.599
30237	4.5733	294.83	129.994
30238	4.5734	229.991	65.4741
30239	4.5734	295.266	174.192
30240	4.5739	326.166	106.036
30241	4.574	271.185	104.16
30242	4.574	286.465	359.114
30243	4.574	308.579	310.948
30244	4.5741	325.894	214.162
30245	4.5741	265.39	88.969
30246	4.5745	-2.7999	138.914
30247	4.5746	300.502	97.011
30248	4.5746	243.381	82.1848
30249	4.5747	252.518	6.5414
30250	4.5748	328.645	136.9
30251	4.5749	280.405	223.816
30252	4.575	236.529	228.891
30253	4.575	292.129	327.711
30254	4.575	255.702	90.3868
30255	4.5751	304.819	127.35
30256	4.5754	324.868	129.134
30257	4.5754	300.093	88.7203
30258	4.5756	323.177	23.5702
30259	4.5756	277.088	178.931
30260	4.5756	309.212	64.6994
30261	4.5756	293.866	183.084
30262	4.5757	335.511	69.6861
30263	4.5757	284.497	239.327
30264	4.5757	205.324	340.805
30265	4.5758	272.331	258.435
30266	4.5759	307.555	175.55
30267	4.5759	352.778	152.041
30268	4.576	292.259	195.491
30269	4.5763	302.436	119.036
30270	4.5767	291.296	268.477
30271	4.5767	252.869	161.071
30272	4.577	302.331	20.5452
30273	4.577	315.227	267.519
30274	4.5771	236.66	143.046
30275	4.5772	291.564	37.4145
30276	4.5776	330.763	172.097
30277	4.578	401.817	70.4702

30278	4.578	298.045	83.8756
30279	4.5781	349.138	67.3282
30280	4.5782	251.734	48.8771
30281	4.5784	221.831	109.645
30282	4.5784	328.653	62.5622
30283	4.5784	323.762	139.92
30284	4.5785	314.151	70.8713
30285	4.5785	323.431	324.311
30286	4.5786	308.876	15.8039
30287	4.5787	283.995	189.576
30288	4.5787	315.604	32.627
30289	4.5787	341.164	16.9691
30290	4.5788	358.149	155.947
30291	4.5788	263.027	124.388
30292	4.5792	318.27	35.8809
30293	4.5793	286.552	265.801
30294	4.5794	331.121	213.009
30295	4.5794	313.91	127.649
30296	4.5795	230.252	65.377
30297	4.5796	285.279	165.673
30298	4.5797	354.447	215.847
30299	4.5798	214.923	343.971
30300	4.5799	371.807	25.5059
30301	4.5799	368.329	28.4055
30302	4.58	315.301	47.9806
30303	4.58	270.191	253.957
30304	4.5802	443.806	19.6587
30305	4.5802	282.616	99.118
30306	4.5803	330.117	222.518
30307	4.5805	327.483	322.682
30308	4.5807	208.524	152.174
30309	4.5807	287.594	28.4502
30310	4.5807	260.404	138.376
30311	4.5807	360.934	26.4751
30312	4.5807	275.029	141.066
30313	4.5808	295.439	44.7414
30314	4.5809	417.742	308.405
30315	4.5809	302.496	92.9463
30316	4.5809	343.902	70.1648
30317	4.581	278.336	262.036
30318	4.5813	363.95	250.083
30319	4.5814	269.404	112.926
30320	4.5816	329.216	75.0367
30321	4.5817	284.363	134.01
30322	4.5818	311.634	71.6657
30323	4.5818	315.516	203.03
30324	4.5819	299.259	193.345
30325	4.5821	323.987	69.0392

30326	4.5822	349.957	25.9326
30327	4.5822	319.548	155.89
30328	4.5824	235.299	222.425
30329	4.5824	299.003	20.9
30330	4.5824	296.959	321.334
30331	4.5826	286.634	158.773
30332	4.5826	298.429	241.84
30333	4.5827	293.375	236.387
30334	4.5827	370.076	128.256
30335	4.5829	301.399	325.583
30336	4.5831	278.022	51.9214
30337	4.5832	346.234	78.7437
30338	4.5833	225.812	252.466
30339	4.5833	319.092	277.095
30340	4.5833	295.452	320.401
30341	4.5834	360.995	146.333
30342	4.5834	331.063	87.0708
30343	4.5834	280.621	179.007
30344	4.5836	293.365	37.0134
30345	4.5837	360.766	34.7816
30346	4.5837	311.281	157.444
30347	4.584	330.727	140.219
30348	4.5842	242.376	116.232
30349	4.5844	315.044	275.454
30350	4.5847	372.888	35.1864
30351	4.5847	264.881	100.849
30352	4.5848	364.191	63.133
30353	4.5851	298.669	130.021
30354	4.5851	322.481	110.04
30355	4.5852	250.701	265.142
30356	4.5852	362.598	82.2035
30357	4.5853	287.952	263.612
30358	4.5854	360.717	28.3997
30359	4.5855	247.338	119.497
30360	4.5855	290.601	127.152
30361	4.5856	306.614	62.4021
30362	4.5856	276.574	248.4
30363	4.5859	288.162	237.195
30364	4.5859	361.064	38.6487
30365	4.5861	283.006	141.617
30366	4.5863	304.176	174.924
30367	4.5863	243.118	109.773
30368	4.5863	313.995	332.187
30369	4.5865	244.03	108.944
30370	4.5865	331.874	71.8485
30371	4.5866	247.334	146.491
30372	4.5868	311.592	85.6867
30373	4.5868	248.512	187.878

30374	4.5869	356.441	79.7041
30375	4.5869	264.905	229.144
30376	4.587	408.264	133.196
30377	4.587	356.494	205.162
30378	4.587	308.346	103.042
30379	4.5871	264.866	134.509
30380	4.5871	261.529	114.974
30381	4.5872	255.172	185.332
30382	4.5873	258.945	334.77
30383	4.5875	319.029	152.284
30384	4.5876	314.933	93.2445
30385	4.5876	330.317	60.4323
30386	4.5877	236.4	238.673
30387	4.5877	311.89	245.841
30388	4.5877	321.452	108.337
30389	4.5878	286.266	101.833
30390	4.5878	335.971	93.901
30391	4.5879	265.528	17.3755
30392	4.5882	335.425	358.965
30393	4.5882	373.156	36.4834
30394	4.5884	223.184	152.907
30395	4.5885	261.869	229.21
30396	4.5888	289.843	18.6634
30397	4.589	282.659	139.753
30398	4.589	320.93	98.6174
30399	4.5891	261.424	121.65
30400	4.5891	238.605	124.051
30401	4.5892	322.603	30.1599
30402	4.5892	278.868	143.535
30403	4.5892	313.404	101.679
30404	4.5893	184.81	283.411
30405	4.5893	297.21	284.786
30406	4.5893	284.88	164.426
30407	4.5894	391.42	64.167
30408	4.5894	275.934	153.086
30409	4.5896	358.625	41.1504
30410	4.5896	382.175	26.029
30411	4.5896	263.831	173.221
30412	4.5897	333.903	236.009
30413	4.5897	332.526	85.6612
30414	4.5897	356.062	128.668
30415	4.5898	250.234	174.965
30416	4.5898	322.309	112.748
30417	4.5898	335.262	37.5241
30418	4.5899	292.799	193.255
30419	4.5899	306.54	123.907
30420	4.59	225.524	225.65
30421	4.5903	378.708	334.559

30422	4.5904	324.872	136.426
30423	4.5905	312.521	233.921
30424	4.5906	300.025	123.607
30425	4.5906	335.319	324.169
30426	4.5906	252.062	199.872
30427	4.5906	350.956	81.5937
30428	4.5907	286.346	258.029
30429	4.5907	252.606	104.84
30430	4.5908	354.297	21.0488
30431	4.5909	311.55	101.856
30432	4.5911	273.702	184.142
30433	4.5911	349.703	111.037
30434	4.5912	348.211	21.4738
30435	4.5913	274.733	318.301
30436	4.5919	343.467	96.5829
30437	4.5919	-2.6869	21.555
30438	4.592	344.507	81.3666
30439	4.592	290.726	228.022
30440	4.592	321.58	31.5514
30441	4.5925	308.028	157.884
30442	4.5925	229.09	160.996
30443	4.5925	281.88	115.077
30444	4.5927	280.858	45.2371
30445	4.5927	285.836	119.262
30446	4.5927	241.11	237.065
30447	4.5928	361.032	83.0669
30448	4.5931	270.414	171.841
30449	4.5932	303.141	136.105
30450	4.5932	304.541	319.303
30451	4.5933	285.236	235.286
30452	4.5933	279.939	346.409
30453	4.5933	-2.7935	106.908
30454	4.5934	206.96	342.908
30455	4.5936	291.657	263.962
30456	4.5937	213.806	126.952
30457	4.5938	306.091	122.971
30458	4.5938	272.213	199.748
30459	4.5938	271.422	168.149
30460	4.5939	289.292	179.679
30461	4.5939	356.724	327.455
30462	4.594	277.737	134.814
30463	4.594	340.122	39.0619
30464	4.594	293.981	168.195
30465	4.5942	294.72	280.56
30466	4.5943	323.047	16.805
30467	4.5943	248.539	166.536
30468	4.5945	295.931	87.6352
30469	4.5947	345.725	256.143

30470	4.5947	348.856	36.5483
30471	4.5949	363.797	49.5914
30472	4.5952	296.746	193.775
30473	4.5953	311.996	319.635
30474	4.5956	333.547	277.88
30475	4.5956	347.653	13.2445
30476	4.5957	324.622	184.349
30477	4.5957	251.435	188.98
30478	4.5957	295.147	86.7906
30479	4.5958	366.983	302.965
30480	4.5959	349.598	55.2517
30481	4.5959	341.048	248.932
30482	4.596	308.329	39.9317
30483	4.5962	279.909	166.639
30484	4.5963	269.007	231.277
30485	4.5966	318.116	116.434
30486	4.5967	220.111	133.369
30487	4.5967	328.119	228.376
30488	4.5968	304.372	161.296
30489	4.597	288.905	302.565
30490	4.5971	256.152	79.1746
30491	4.5973	300.939	186.785
30492	4.5974	394.24	29.13
30493	4.5974	318.758	59.977
30494	4.5974	272.076	266.607
30495	4.5975	216.474	149.446
30496	4.5977	234.085	131.155
30497	4.5978	278.424	82.7577
30498	4.5978	260.447	169.737
30499	4.5978	303.244	106.263
30500	4.5979	270.195	88.8232
30501	4.598	323.004	29.9799
30502	4.5981	304.184	46.0256
30503	4.5982	242.148	131.884
30504	4.5982	310.412	92.9408
30505	4.5986	252.193	119.106
30506	4.5987	334.078	260.715
30507	4.5988	294.357	198.661
30508	4.5988	359.325	14.4151
30509	4.5988	310.36	153.962
30510	4.5989	368.713	117.607
30511	4.599	302.677	38.166
30512	4.5991	289.69	236.4
30513	4.5991	301.477	181.627
30514	4.5991	248.091	175.653
30515	4.5992	301.11	30.4346
30516	4.5993	336.564	100.268
30517	4.5993	368.905	295.349

30518	4.5994	304.264	229.62
30519	4.5994	291.309	74.9785
30520	4.5995	289.974	170.817
30521	4.5995	263.384	121.67
30522	4.5997	332.986	146.983
30523	4.5997	319.336	263.805
30524	4.5998	316.492	88.3105
30525	4.5999	248.548	215.814
30526	4.6	260.935	17.5932
30527	4.6001	238.732	62.6778
30528	4.6004	214.044	241.789
30529	4.6004	263.176	112.544
30530	4.6005	-4.7959	120.675
30531	4.6005	272.518	118.969
30532	4.6007	357.798	301.43
30533	4.6008	338.17	182.354
30534	4.6008	285.082	246.544
30535	4.6008	269.487	236.172
30536	4.6008	380.183	180.231
30537	4.6009	296.318	285.43
30538	4.601	333.165	264.928
30539	4.6012	246.932	176.74
30540	4.6012	283.174	92.0454
30541	4.6012	323.95	241.607
30542	4.6013	307.458	273.858
30543	4.6013	131.461	174.841
30544	4.6013	238.873	155.285
30545	4.6013	302.259	341.469
30546	4.6015	304.535	78.4686
30547	4.6015	309.544	336.338
30548	4.6016	363.53	74.7804
30549	4.6016	259.049	135.778
30550	4.6017	231.307	251.684
30551	4.6018	321.471	26.534
30552	4.6018	293.143	93.6152
30553	4.6019	306.575	300.27
30554	4.602	309.008	106.315
30555	4.602	322.51	112.902
30556	4.602	313.079	193.874
30557	4.602	293.739	299.289
30558	4.6021	352.407	24.7691
30559	4.6024	296.697	159.368
30560	4.6027	312.42	245.527
30561	4.6029	283.623	57.0219
30562	4.603	323.088	57.7082
30563	4.6033	344.062	107.856
30564	4.6033	359.182	87.0246
30565	4.6034	300.965	308.282

30566	4.6034	306.993	36.596
30567	4.6035	323.421	70.1379
30568	4.6035	277.098	186.72
30569	4.6035	272.134	84.8127
30570	4.6035	229.105	321.239
30571	4.6037	265.31	133.175
30572	4.6038	306.669	70.3014
30573	4.6038	201.317	138.106
30574	4.6039	216.436	171.025
30575	4.6039	244.96	346.004
30576	4.604	237.883	277.471
30577	4.6041	312.022	83.4684
30578	4.6042	297.453	346.622
30579	4.6042	209.16	240.581
30580	4.6043	329.356	202.641
30581	4.6044	320.509	204.245
30582	4.6044	255.915	128.103
30583	4.6046	207.462	118.408
30584	4.6046	243.454	91.1246
30585	4.6046	344.158	142.23
30586	4.6048	336.088	26.658
30587	4.6048	269.63	196.29
30588	4.6048	298.588	132.659
30589	4.6049	304.606	164.765
30590	4.605	235.867	14.8052
30591	4.605	288.241	103.189
30592	4.6052	319.744	226.203
30593	4.6054	313.58	85.93
30594	4.6054	252.244	233.681
30595	4.6056	376.641	29.9663
30596	4.6057	257.494	326.147
30597	4.6058	287.521	281.378
30598	4.6058	271.823	112.225
30599	4.6058	311.23	78.0221
30600	4.6058	297.019	154.511
30601	4.6059	222.044	32.734
30602	4.606	254.685	323.168
30603	4.6062	323.569	124.711
30604	4.6063	281.673	230.261
30605	4.6063	271.671	98.9713
30606	4.6063	292.836	73.8174
30607	4.6064	283.501	133.984
30608	4.6065	332.274	88.5427
30609	4.6065	245.624	141.559
30610	4.6066	293.213	318.647
30611	4.6066	300.082	54.4327
30612	4.6066	316.913	100.333
30613	4.6067	290.682	181.898

30614	4.6067	267.793	117.344
30615	4.6069	320.306	109.59
30616	4.6071	300.341	145.859
30617	4.6071	295.062	226.526
30618	4.6072	309.044	311.739
30619	4.6072	306.628	11.7418
30620	4.6072	306.54	263.892
30621	4.6073	282.289	138.022
30622	4.6074	297.977	336.906
30623	4.6077	324.658	283.692
30624	4.6078	304.04	38.4883
30625	4.608	283.729	184.37
30626	4.608	321.08	62.3064
30627	4.6082	351.801	53.5181
30628	4.6082	267.011	191.33
30629	4.6082	293.2	179.169
30630	4.6085	301.397	95.8611
30631	4.6085	286.181	155.07
30632	4.6086	290.344	93.9712
30633	4.6086	308.22	28.1756
30634	4.6086	306.667	28.6387
30635	4.6087	295.191	186.135
30636	4.6087	255.358	219.922
30637	4.6087	331.254	47.3097
30638	4.6089	334.114	71.9472
30639	4.6089	340.816	299.121
30640	4.6091	293.98	293.256
30641	4.6095	247.205	136.35
30642	4.6097	305.546	217.283
30643	4.6097	302.028	203.4
30644	4.6098	395.982	305.929
30645	4.6099	347.014	249.384
30646	4.61	288.717	167.439
30647	4.61	304.249	86.8518
30648	4.61	328.244	28.3326
30649	4.6101	285.644	70.8552
30650	4.6101	281.19	136.183
30651	4.6104	296.469	120.952
30652	4.6105	300.664	86.7455
30653	4.6105	425.213	18.5735
30654	4.6106	243.072	215.668
30655	4.6108	305.174	125.797
30656	4.6109	271.536	139.897
30657	4.6109	316.94	200.692
30658	4.611	383.588	83.1137
30659	4.611	342.142	245.973
30660	4.6112	300.544	290.872
30661	4.6112	276.871	188.513

30662	4.6113	299.121	284.007
30663	4.6114	280.841	105.236
30664	4.6114	335.539	114.503
30665	4.6114	343.977	285.136
30666	4.6114	334.83	123.834
30667	4.6117	370.133	304.786
30668	4.6117	303.773	48.5632
30669	4.6117	263.787	145.952
30670	4.6119	334.674	43.9606
30671	4.6125	292.574	66.6364
30672	4.6125	243.914	222.812
30673	4.6127	394.549	75.7051
30674	4.6128	319.877	163.547
30675	4.6128	297.358	69.0057
30676	4.6128	331.693	152.791
30677	4.6131	247.9	342.049
30678	4.6131	314.042	75.1878
30679	4.6132	324.429	296.421
30680	4.6133	299.306	180.361
30681	4.6134	292.522	9.6477
30682	4.6134	330.618	100.872
30683	4.6135	258.254	184.695
30684	4.6135	277.244	232.282
30685	4.6136	354.28	291.055
30686	4.6137	299.059	231.282
30687	4.6138	328.44	193.573
30688	4.6138	283.986	174.411
30689	4.6138	309.347	229.57
30690	4.6139	335.312	91.1139
30691	4.6139	277.992	19.4559
30692	4.614	361.69	274.334
30693	4.6142	329.298	178.137
30694	4.6143	303.312	281.569
30695	4.6143	331.521	315.446
30696	4.6143	292.452	109.343
30697	4.6143	264.023	136.546
30698	4.6145	332.568	189.401
30699	4.6145	319.973	158.08
30700	4.6145	335.315	207.105
30701	4.6145	277.948	100.585
30702	4.6147	307.286	108.643
30703	4.615	254.598	119.522
30704	4.6151	333.963	23.3175
30705	4.6151	296.97	102.603
30706	4.6151	352.009	65.9091
30707	4.6153	264.343	73.2017
30708	4.6153	259.336	169.889
30709	4.6154	206.641	146.111

30710	4.6154	279.07	128.354
30711	4.6155	344.772	84.185
30712	4.6155	314.534	129.184
30713	4.6155	282.371	30.9297
30714	4.6156	299.683	128.386
30715	4.6157	331.652	173.264
30716	4.6158	320.502	110.918
30717	4.6158	287.169	65.8988
30718	4.616	319.606	136.944
30719	4.616	269.687	227.48
30720	4.616	272.912	186.184
30721	4.616	302.338	9.6418
30722	4.6161	324.557	147.43
30723	4.6161	403.514	58.0138
30724	4.6161	278.204	134.082
30725	4.6161	240.29	142.945
30726	4.6162	364.846	37.773
30727	4.6162	340.679	346.996
30728	4.6162	268.781	182.636
30729	4.6164	323.779	110.152
30730	4.6166	249.759	298.836
30731	4.6167	233.307	148.955
30732	4.6169	325.276	199.994
30733	4.617	233.026	284.341
30734	4.6171	266.381	221.212
30735	4.6171	279.649	70.6776
30736	4.6173	307.468	247.317
30737	4.6173	282.97	38.4351
30738	4.6174	373.14	21.8163
30739	4.6175	324.078	108.849
30740	4.6177	149.479	21.0894
30741	4.6177	298.429	90.0215
30742	4.618	342.416	180.655
30743	4.6181	288.038	103.702
30744	4.6182	317.237	89.6381
30745	4.6183	327.211	193.469
30746	4.6183	346.887	104.69
30747	4.6184	312.475	128.56
30748	4.6186	243.621	111.654
30749	4.6187	331.256	93.1007
30750	4.6188	334.427	266.513
30751	4.6188	304.741	198.32
30752	4.6189	321.223	230.775
30753	4.6189	256.649	256.15
30754	4.6189	414.248	33.2345
30755	4.6191	332.139	318.313
30756	4.6191	315.954	107.711
30757	4.6192	-3.029	324.583

30758	4.6192	321.013	105.216
30759	4.6195	408.488	290.551
30760	4.6195	221.462	109.789
30761	4.6196	281.591	105.689
30762	4.6196	354.766	315.493
30763	4.6199	273.363	161.501
30764	4.62	330.481	341.536
30765	4.62	311.079	54.8268
30766	4.6201	353.904	65.0552
30767	4.6201	335.646	190.151
30768	4.6202	344.179	129.426
30769	4.6203	368.666	40.199
30770	4.6205	339.857	206.689
30771	4.6205	328.653	222.923
30772	4.6206	273.328	110.471
30773	4.621	306.021	117.52
30774	4.6212	336.444	120.566
30775	4.6212	296.532	63.5021
30776	4.6213	320.888	151.73
30777	4.6213	337.128	325.859
30778	4.6215	397.084	69.1425
30779	4.6215	240.259	293.337
30780	4.6217	277.972	179.104
30781	4.6219	347.932	38.919
30782	4.622	338.162	50.713
30783	4.6221	299.959	184.58
30784	4.6222	345.265	39.9355
30785	4.6222	312.44	244.284
30786	4.6222	258.568	129.746
30787	4.6223	346.444	56.9272
30788	4.6224	317.894	231.852
30789	4.6225	304.072	232.135
30790	4.6226	275.824	160.67
30791	4.6226	334.391	95.6825
30792	4.6227	245.305	111.992
30793	4.6229	269.481	45.1068
30794	4.6229	379.132	29.496
30795	4.6231	244.084	184.967
30796	4.6231	329.702	162.767
30797	4.6232	256.918	264.917
30798	4.6232	288.237	96.7678
30799	4.6233	236.894	139.478
30800	4.6235	300.932	101.662
30801	4.6236	344.428	93.8853
30802	4.6236	244.789	180.494
30803	4.6237	258.462	123.178
30804	4.6239	291.95	145.805
30805	4.6244	272.237	48.5089

30806	4.6246	260.918	229.785
30807	4.6249	347.215	151.494
30808	4.625	181.774	137.213
30809	4.6252	276.953	52.6851
30810	4.6253	335.913	22.0753
30811	4.6253	307.447	111.861
30812	4.6253	322.702	216.146
30813	4.6255	292.697	350.327
30814	4.6255	287.726	217.211
30815	4.6255	351.822	2.0001
30816	4.6255	338.626	245.757
30817	4.6256	284.045	128.994
30818	4.6256	212.053	303.287
30819	4.6256	350.963	323.397
30820	4.6257	299.879	242.607
30821	4.626	220.908	228.632
30822	4.626	323.976	142.656
30823	4.6261	232.545	41.772
30824	4.6262	321.162	237.697
30825	4.6263	415.881	218.719
30826	4.6263	315.567	274.798
30827	4.6264	336.374	70.0413
30828	4.6264	320.774	46.3861
30829	4.6264	309.902	137.442
30830	4.6265	324.09	301.051
30831	4.6265	333.344	89.8449
30832	4.6265	319.768	30.5925
30833	4.6265	305.152	334.069
30834	4.6266	341.392	241.217
30835	4.6266	292.663	185.095
30836	4.6267	318.981	231.539
30837	4.6267	364.005	74.1137
30838	4.6267	211.995	338.303
30839	4.6268	325.322	109.353
30840	4.6269	319.817	112.039
30841	4.6269	295.368	177.959
30842	4.6269	319.479	340.203
30843	4.627	312.563	263.507
30844	4.627	310.295	135.579
30845	4.6271	284.711	124.31
30846	4.6272	257.536	277.988
30847	4.6273	332.466	98.9433
30848	4.6273	321.551	275.112
30849	4.6274	245.046	359.698
30850	4.6276	234.78	133.473
30851	4.6278	382.598	27.3216
30852	4.6279	250.604	108.382
30853	4.628	219.824	184.661

30854	4.628	300.161	89.7431
30855	4.6281	326.022	271.636
30856	4.6281	265.614	252.64
30857	4.6284	244.288	127.347
30858	4.6286	296.533	179.283
30859	4.6288	317.53	287.572
30860	4.6288	249.434	189.585
30861	4.629	242.909	195.197
30862	4.6292	231.194	202.217
30863	4.6293	362.114	111.373
30864	4.6293	235.41	79.611
30865	4.6296	307.004	73.9385
30866	4.6296	292.492	159.63
30867	4.6297	294.681	200.738
30868	4.6297	232.938	221.936
30869	4.6297	317.896	80.8255
30870	4.6298	332.159	60.0766
30871	4.6299	277.093	192.017
30872	4.63	257.344	68.216
30873	4.6301	276.352	185.49
30874	4.6302	314.031	184.932
30875	4.6303	314.65	98.0406
30876	4.6303	327.362	235.198
30877	4.6304	334.76	20.212
30878	4.6307	299.745	59.7791
30879	4.6307	237.422	118.223
30880	4.631	388.88	23.2286
30881	4.6311	284.524	19.1707
30882	4.6312	286.451	183.011
30883	4.6313	325.57	98.9485
30884	4.6313	380.01	31.2174
30885	4.6314	304.487	112.33
30886	4.6314	344.969	95.6955
30887	4.6314	262.345	169.838
30888	4.6315	339.743	42.6414
30889	4.6316	320.703	133.466
30890	4.6317	213.653	100.757
30891	4.6321	369.239	138.484
30892	4.6321	359.598	242.993
30893	4.6322	313.858	113.968
30894	4.6322	320.485	324.655
30895	4.6323	310.506	227.341
30896	4.6323	302.699	131.988
30897	4.6326	329.238	113.922
30898	4.6329	373.093	257.36
30899	4.6329	303.956	172.123
30900	4.6329	220.914	178.29
30901	4.6329	277.547	104.514

30902	4.6331	305.047	107.318
30903	4.6332	258.552	227.909
30904	4.6333	368.723	43.0171
30905	4.6337	371.268	34.9606
30906	4.6337	291.077	82.4061
30907	4.6339	323.933	26.0234
30908	4.6339	386.696	65.6479
30909	4.6339	299.883	167.468
30910	4.6339	270.674	244.542
30911	4.6342	323.392	278.134
30912	4.6343	322.296	163.041
30913	4.6343	355.251	239.536
30914	4.6344	224.53	320.235
30915	4.6344	311.162	24.4735
30916	4.6345	270.364	262.904
30917	4.6345	325.726	2.6504
30918	4.6345	314.669	199.86
30919	4.6346	308.861	291.043
30920	4.6346	311.081	123.567
30921	4.6346	204.528	136.599
30922	4.6346	302.342	165.477
30923	4.6349	238.546	19.0326
30924	4.635	342.67	78.6583
30925	4.635	376.977	44.3589
30926	4.635	295.358	151.402
30927	4.635	282.588	141.417
30928	4.6351	388.685	36.4253
30929	4.6351	284.155	351.203
30930	4.6351	304.36	170.595
30931	4.6351	287.31	111.397
30932	4.6351	349.958	164.458
30933	4.6352	367.017	19.3555
30934	4.6352	287.369	176.198
30935	4.6352	331.899	125.693
30936	4.6353	248.158	95.1651
30937	4.6354	311.182	179.719
30938	4.6354	247.272	229.129
30939	4.6356	272.388	213.955
30940	4.6357	237.195	165.275
30941	4.6357	245.786	159.793
30942	4.6357	383.061	50.7009
30943	4.6357	340.457	231.908
30944	4.6358	-4.0779	250.946
30945	4.6359	366.227	79.2991
30946	4.6361	280.662	290.674
30947	4.6363	282.194	80.4416
30948	4.6364	332.862	319.345
30949	4.6364	286.502	98.071

30950	4.6367	354.995	61.2506
30951	4.6369	311.948	302.352
30952	4.6371	319.495	114.531
30953	4.6372	365.192	54.8251
30954	4.6372	273.632	45.7649
30955	4.6373	321.51	147.396
30956	4.6373	270.113	234.275
30957	4.6374	307.514	333.808
30958	4.6375	289.083	196.474
30959	4.6375	320.57	295.358
30960	4.6375	245.274	212.469
30961	4.6376	360.115	270.578
30962	4.6376	233.85	222.414
30963	4.6376	263.304	125.911
30964	4.6377	270.754	234.455
30965	4.6377	318.765	334.351
30966	4.6377	264.668	16.2988
30967	4.6378	253.725	171.261
30968	4.6378	259.9	8.5709
30969	4.6379	274.839	179.384
30970	4.638	332.445	66.3012
30971	4.6381	267.326	258.273
30972	4.6382	341.808	124.166
30973	4.6383	266.638	179.527
30974	4.6383	330.322	101.097
30975	4.6385	299.635	7.7944
30976	4.6386	288.134	141.633
30977	4.6388	339.273	64.2054
30978	4.6389	352.019	325.345
30979	4.639	319.285	220.444
30980	4.639	337.662	79.5869
30981	4.639	247.947	123.238
30982	4.6391	316.383	113.938
30983	4.6391	329.736	24.2379
30984	4.6391	287.37	179.539
30985	4.6393	329.9	60.9377
30986	4.6393	313.363	240.362
30987	4.6394	289.274	44.2526
30988	4.6395	323.265	122.789
30989	4.6396	348.105	94.5148
30990	4.6397	373.925	78.1735
30991	4.6397	336.154	157.76
30992	4.6398	282.003	21.0862
30993	4.64	262.619	226.779
30994	4.64	343.036	87.3106
30995	4.64	306.426	163.795
30996	4.6403	340.307	298.981
30997	4.6403	367.287	72.7609

30998	4.6404	360.953	308.16
30999	4.6404	291.846	184.632
31000	4.6405	285.276	183.932
31001	4.6407	292.715	183.562
31002	4.6407	269.396	47.1705
31003	4.6409	334.57	313.32
31004	4.6409	330.307	95.9498
31005	4.641	303.383	119.146
31006	4.6411	378.44	285.966
31007	4.6413	332.66	231.958
31008	4.6413	228.1	244.327
31009	4.6413	315.35	166.504
31010	4.6413	300.609	138.309
31011	4.6413	355.671	137.062
31012	4.6414	238.937	218.069
31013	4.6414	225.184	161.809
31014	4.6414	254.815	223.371
31015	4.6414	332.392	82.4066
31016	4.6415	264.416	213.854
31017	4.6416	245.865	209.986
31018	4.6416	310.206	61.6894
31019	4.6416	295.153	86.5283
31020	4.6416	259.045	131.312
31021	4.6417	261.937	258.364
31022	4.6418	227.884	147.139
31023	4.6419	420.306	283.01
31024	4.642	290.125	225.612
31025	4.642	323.032	49.9102
31026	4.6422	287.573	68.0239
31027	4.6423	322.011	268.796
31028	4.6424	325.878	64.0773
31029	4.6424	295.142	137.607
31030	4.6424	317.077	111.305
31031	4.6425	384.716	32.3014
31032	4.6425	235.067	89.8574
31033	4.6426	341.08	74.6834
31034	4.6427	253.971	191.606
31035	4.6428	342.791	68.2254
31036	4.6428	283.957	149.426
31037	4.6429	295.737	94.2447
31038	4.643	259.166	169.029
31039	4.643	292.927	156.507
31040	4.6431	267.996	102.387
31041	4.6432	409.228	83.9897
31042	4.6432	329.794	62.0211
31043	4.6433	328.916	80.2818
31044	4.6434	252.349	143.235
31045	4.6435	301.035	122.78

31046	4.6437	255.081	189.055
31047	4.6438	238.19	160.091
31048	4.6438	295.011	232.232
31049	4.6438	249.006	335.311
31050	4.6439	226.442	135.335
31051	4.6439	324.418	259.1
31052	4.6439	365.358	8.9235
31053	4.6441	286.129	134.456
31054	4.6445	370.961	92.8685
31055	4.6445	286.205	135.077
31056	4.6447	310.341	342.536
31057	4.6448	335.264	118.898
31058	4.6448	330.626	22.1119
31059	4.6452	322.852	104.119
31060	4.6452	276.42	219.668
31061	4.6452	305.154	349.136
31062	4.6453	372.366	99.3228
31063	4.6454	292.134	77.1943
31064	4.6456	337.761	58.1408
31065	4.6457	335.54	81.2961
31066	4.6458	281.03	71.0809
31067	4.6461	300.951	72.5822
31068	4.6461	389.677	292.308
31069	4.6462	377.409	83.1676
31070	4.6462	315.649	146.426
31071	4.6466	332.365	98.6778
31072	4.6466	327.669	85.1246
31073	4.6467	278.298	194.41
31074	4.6467	361.091	89.5326
31075	4.6469	325.927	57.3733
31076	4.647	299.205	81.1695
31077	4.647	349.416	128.98
31078	4.6471	318.59	107.523
31079	4.6473	326.707	155.182
31080	4.6473	315.564	125.037
31081	4.6473	408.373	22.0103
31082	4.6474	309.795	99.7916
31083	4.6474	338.596	91.7664
31084	4.6474	369.39	344.955
31085	4.6475	282.539	174.483
31086	4.6476	271.59	325.236
31087	4.6476	314.595	132.523
31088	4.6477	357.031	123.131
31089	4.6477	251.619	269.216
31090	4.6478	295.805	71.9008
31091	4.6478	330.358	192.941
31092	4.6478	316.685	82.4008
31093	4.6478	335.538	49.7291

31094	4.648	241.443	198.444
31095	4.6481	301.669	195.186
31096	4.6481	233.506	198.119
31097	4.6482	322.027	101.815
31098	4.6483	356.866	63.1343
31099	4.6485	372.863	62.1725
31100	4.6486	329.205	114.031
31101	4.6486	270.896	178.592
31102	4.6486	335.337	12.428
31103	4.6486	358.745	335.026
31104	4.6487	336.447	109.382
31105	4.6488	308.536	166.224
31106	4.6489	391.224	8.7527
31107	4.6491	23.3592	57.5031
31108	4.6492	310.803	45.8046
31109	4.6493	292.47	166.908
31110	4.6493	331.647	110.31
31111	4.6494	374.936	247.876
31112	4.6495	267.14	42.7975
31113	4.6495	332.619	72.5212
31114	4.6496	273.861	86.0329
31115	4.6496	290.962	190.459
31116	4.6498	314.979	154.796
31117	4.65	355.238	154.712
31118	4.6501	304.831	176.531
31119	4.6501	332.282	90.1941
31120	4.6502	343.295	207.432
31121	4.6502	285.772	100.798
31122	4.6502	377.104	20.7236
31123	4.6505	342.972	257.771
31124	4.6506	346.165	261.78
31125	4.6506	339.045	173.806
31126	4.6507	322.079	10.4653
31127	4.6508	271.092	306.332
31128	4.6509	281.446	122.739
31129	4.6511	337.065	301.279
31130	4.6511	264.018	176.511
31131	4.6511	378.005	292.394
31132	4.6512	288.306	180.736
31133	4.6512	339.459	279.971
31134	4.6513	372.781	78.6973
31135	4.6513	337.191	70.9882
31136	4.6513	313.271	119.421
31137	4.6514	354.914	94.0559
31138	4.6515	324.805	157.214
31139	4.6516	342.422	52.4846
31140	4.6516	322.598	224.193
31141	4.6516	358.195	46.3997

31142	4.6516	240.168	138.091
31143	4.6517	253.501	200.038
31144	4.6517	268.554	340.19
31145	4.6517	240.276	28.8751
31146	4.6518	282.165	237.956
31147	4.6519	302.996	175.317
31148	4.6521	202.203	140.755
31149	4.6522	373.664	51.4145
31150	4.6522	366.036	84.8506
31151	4.6524	320.161	186.847
31152	4.6524	321.252	119.909
31153	4.6525	306.233	121.416
31154	4.6525	332.258	344.765
31155	4.6526	354.035	216.302
31156	4.6527	234.116	187.335
31157	4.6528	279.475	348.05
31158	4.6528	308.434	253.585
31159	4.6528	271.63	131.056
31160	4.6529	364.329	35.8765
31161	4.653	308.153	26.5913
31162	4.6531	250.077	317.486
31163	4.6531	345.682	247.291
31164	4.6532	348.507	91.1511
31165	4.6532	272.781	251.984
31166	4.6532	273.422	141.726
31167	4.6532	358.45	31.7246
31168	4.6533	314.799	118.226
31169	4.6535	295.472	221.09
31170	4.6538	320.726	237.217
31171	4.6538	333.022	282.618
31172	4.6539	315.582	236.326
31173	4.654	358.533	21.3443
31174	4.6541	327.02	129.079
31175	4.6541	331.899	303.464
31176	4.6542	263.014	120.066
31177	4.6543	328.517	38.9385
31178	4.6543	264.908	168.471
31179	4.6544	288.968	147.437
31180	4.6545	345.069	40.0232
31181	4.6546	237.997	40.263
31182	4.6546	360.065	41.1062
31183	4.6547	284.917	142.462
31184	4.6548	382.415	34.3033
31185	4.6549	380.911	30.6486
31186	4.6549	324.549	275.132
31187	4.6549	269.943	207.736
31188	4.655	401.799	291.737
31189	4.6551	315.561	230.173

31190	4.6551	239.389	178.316
31191	4.6551	295.643	96.6466
31192	4.6551	306.259	98.5586
31193	4.6551	294.893	156.484
31194	4.6552	330.991	245.338
31195	4.6552	271.603	236.16
31196	4.6553	270.78	166.597
31197	4.6553	326.982	116.941
31198	4.6553	319.893	102.332
31199	4.6553	279.134	177.591
31200	4.6555	291.673	301.472
31201	4.6555	321.348	77.1245
31202	4.6556	337.308	340.695
31203	4.6557	309.754	147.092
31204	4.6557	319.455	134.245
31205	4.6557	328.991	80.873
31206	4.6559	352.203	31.1817
31207	4.656	311.76	108.705
31208	4.656	341.36	236.737
31209	4.6561	262.932	181.522
31210	4.6561	346.309	94.3338
31211	4.6562	314.408	215.051
31212	4.6563	365.399	80.7759
31213	4.6563	355.841	96.6449
31214	4.6564	281.116	183.757
31215	4.6564	405.737	22.6418
31216	4.6568	272.154	185.787
31217	4.6568	341.351	297.853
31218	4.657	321.867	336.124
31219	4.657	261.927	136.026
31220	4.6571	378.513	77.6823
31221	4.6571	342.436	80.4016
31222	4.6571	292.564	73.6794
31223	4.6571	265.659	159.034
31224	4.6572	339.618	84.9276
31225	4.6572	331.116	96.9639
31226	4.6572	357.965	71.1306
31227	4.6572	294.001	128.296
31228	4.6573	283.781	241.134
31229	4.6574	303.133	123.175
31230	4.6574	354.807	15.0973
31231	4.6575	285.199	132.636
31232	4.6576	334.277	61.7335
31233	4.6578	304.905	314.376
31234	4.6579	365.916	351.955
31235	4.6582	292.171	102.829
31236	4.6582	322.037	102.757
31237	4.6582	351.988	19.2717

31238	4.6583	283.976	224.399
31239	4.6589	314.048	332.972
31240	4.659	290.621	179.245
31241	4.659	295.218	133.459
31242	4.659	43.961	355.125
31243	4.659	330.2	36.5541
31244	4.6593	279.277	235.673
31245	4.6593	333.246	56.9358
31246	4.6594	279.527	103.067
31247	4.6595	312.985	181.089
31248	4.6595	316.677	136.151
31249	4.6597	290.106	296.937
31250	4.6599	297.453	141.625
31251	4.66	380.343	72.6231
31252	4.66	310.757	134.889
31253	4.6601	306.84	169.328
31254	4.6603	292.862	278.663
31255	4.6607	362.599	36.6924
31256	4.6609	211.616	179.638
31257	4.6611	343.266	254.008
31258	4.6611	302.092	127.009
31259	4.6612	290.639	286.348
31260	4.6614	276.493	223.734
31261	4.6615	343.316	128.31
31262	4.6616	319.108	91.5785
31263	4.6616	321.339	70.8476
31264	4.6617	305.341	152.516
31265	4.6619	274.281	168.381
31266	4.6619	350.837	325.983
31267	4.6619	346.828	156.51
31268	4.6621	333.242	98.2435
31269	4.6621	322.467	275.779
31270	4.6622	320.714	117.675
31271	4.6623	370.165	59.7397
31272	4.6624	278.184	107.044
31273	4.6624	310.65	124.124
31274	4.6626	342.134	43.3576
31275	4.6628	348.167	138.654
31276	4.6631	281.832	108.274
31277	4.6631	289.437	91.7402
31278	4.6633	344.195	109.171
31279	4.6633	351.804	211.57
31280	4.6633	312.583	123.511
31281	4.6634	246.514	92.6502
31282	4.6635	297.753	81.3005
31283	4.6637	265.207	194.054
31284	4.6637	246.499	96.7319
31285	4.6637	274.212	29.8204

31286	4.6639	365.158	70.8655
31287	4.6639	277.73	180.13
31288	4.664	312.692	189.125
31289	4.6641	305.307	218.777
31290	4.6641	245.893	78.5918
31291	4.6642	330.409	133.13
31292	4.6644	337.172	331.501
31293	4.6645	277.261	48.2697
31294	4.6647	336.876	105.845
31295	4.6647	401.523	81.8108
31296	4.6649	266.113	333.785
31297	4.6649	397.06	29.6571
31298	4.665	271.335	252.913
31299	4.6651	286.298	124.884
31300	4.6654	302.808	120.386
31301	4.6655	306.856	71.9034
31302	4.6655	357.399	297.631
31303	4.6656	362.186	66.377
31304	4.6656	313.836	185.267
31305	4.6656	344.526	142.297
31306	4.6658	263.661	161.513
31307	4.6658	311.342	21.5204
31308	4.6659	311.828	237.341
31309	4.6659	328.932	232.412
31310	4.6659	186.574	156.585
31311	4.6659	317.455	254.622
31312	4.6659	384.789	184.293
31313	4.6661	349.318	2.6925
31314	4.6661	267.712	218.137
31315	4.6663	292.935	183.134
31316	4.6664	323.197	273.111
31317	4.6664	351.064	81.8666
31318	4.6665	335.93	127.729
31319	4.6667	246.593	126.174
31320	4.6669	340.826	329.559
31321	4.6669	386.497	30.6401
31322	4.667	304.803	133.085
31323	4.6672	239.197	181.406
31324	4.6673	297.329	230.085
31325	4.6673	260.439	176.758
31326	4.6673	311.784	80.6193
31327	4.6673	339.848	56.693
31328	4.6673	317.506	155.829
31329	4.6674	354.205	64.4188
31330	4.6676	296.25	250.753
31331	4.6676	345.388	159.042
31332	4.6677	312.048	280.291
31333	4.6678	274.76	179.016

31334	4.6681	318.104	78.6621
31335	4.6683	348.194	358.608
31336	4.6683	348.502	259.876
31337	4.6683	315.192	180.673
31338	4.6683	300.595	122.349
31339	4.6684	335.672	61.892
31340	4.6685	319.099	12.4081
31341	4.6686	328.643	350.528
31342	4.6686	326.133	336.665
31343	4.6687	316.584	147.042
31344	4.6688	294.005	110.731
31345	4.6689	342.044	35.6815
31346	4.669	323.532	333.311
31347	4.6691	359.483	169.693
31348	4.6691	285.115	76.57
31349	4.6693	260.882	173.955
31350	4.6693	255.315	134.693
31351	4.6696	379.773	303.988
31352	4.6696	350.132	76.2936
31353	4.6697	354.732	103.416
31354	4.6698	230.74	174.789
31355	4.6698	265.239	125.1
31356	4.67	342.518	158.421
31357	4.6701	259.532	111.154
31358	4.6702	369.44	60.1008
31359	4.6703	237.967	40.0068
31360	4.6704	226.257	183.068
31361	4.6705	302.389	176.315
31362	4.6705	348.454	94.4445
31363	4.6706	337.724	116.09
31364	4.6706	310.308	126.974
31365	4.6707	295.012	278.62
31366	4.6708	389.939	60.2476
31367	4.6709	336.618	116.006
31368	4.6709	363.175	313.981
31369	4.6711	355.121	56.6156
31370	4.6712	344.998	90.7305
31371	4.6712	320.328	259.101
31372	4.6712	247.849	239.487
31373	4.6712	354.767	324.956
31374	4.6713	289.117	133.531
31375	4.6713	266.745	210.945
31376	4.6713	329.424	39.1557
31377	4.6714	315.007	184.824
31378	4.6714	300.811	178.194
31379	4.6719	297.67	125.966
31380	4.6719	270.113	183.41
31381	4.672	293.806	155.868

31382	4.6721	324.804	188.686
31383	4.6722	285.317	82.3378
31384	4.6722	337.018	272.887
31385	4.6722	390.142	162.453
31386	4.6724	336.896	103.576
31387	4.6726	377.972	28.1547
31388	4.6726	405.564	29.2847
31389	4.6726	347.872	146.771
31390	4.6726	371.996	207.623
31391	4.6729	268.281	116.909
31392	4.6731	300.734	111.593
31393	4.6733	355.017	73.8675
31394	4.6734	297.984	229.919
31395	4.6735	308.386	221.578
31396	4.6736	290.141	186.619
31397	4.6739	312.957	124.663
31398	4.674	360.043	227.465
31399	4.674	313.767	200.288
31400	4.6741	451.019	23.5019
31401	4.6742	249.587	204.533
31402	4.6745	400.455	56.0848
31403	4.6746	281.402	187.801
31404	4.6746	230.018	129.364
31405	4.6747	374.91	116.203
31406	4.6748	303.506	117.583
31407	4.6749	302.149	184.851
31408	4.6751	325.682	43.7507
31409	4.6752	354.079	68.658
31410	4.6753	380.397	27.3997
31411	4.6753	372.203	229.59
31412	4.6754	256.687	261.647
31413	4.6754	307.331	96.5908
31414	4.6755	348.21	90.168
31415	4.6755	316.503	105.126
31416	4.6757	402.325	25.9304
31417	4.6757	409.778	23.5564
31418	4.6758	361.641	80.2016
31419	4.6758	309.045	73.9671
31420	4.6758	261.544	249.96
31421	4.676	342.578	109.79
31422	4.676	362.292	82.7459
31423	4.6764	273.307	13.1023
31424	4.6764	338.779	67.7628
31425	4.6764	277.429	104.729
31426	4.6766	260.05	305.194
31427	4.6767	353.55	50.8143
31428	4.6767	300.44	35.8679
31429	4.6767	293.353	175.757

31430	4.6768	322.601	130.788
31431	4.6768	317.733	251.705
31432	4.6769	339.122	103.441
31433	4.6771	314.909	189.263
31434	4.6771	291.497	148.716
31435	4.6771	420.74	23.1141
31436	4.6772	325.724	82.5517
31437	4.6773	362.279	262.078
31438	4.6773	265.75	243.261
31439	4.6773	324.081	129.557
31440	4.6774	300.26	66.568
31441	4.6775	375.957	193.8
31442	4.6775	319.647	177.436
31443	4.6775	272.736	110.967
31444	4.6778	374.038	84.0929
31445	4.6778	322.131	9.3912
31446	4.6779	400.418	299.98
31447	4.6779	239.41	228.011
31448	4.6779	323.316	215.049
31449	4.6779	271.598	99.6838
31450	4.6779	332.923	341.895
31451	4.6781	345.695	178.851
31452	4.6785	325.108	106.747
31453	4.6785	383.468	189.527
31454	4.6785	250.928	120.954
31455	4.6785	293.655	124.079
31456	4.6786	302.228	60.1564
31457	4.6789	279.474	28.4078
31458	4.6789	261.87	135.339
31459	4.6789	311.824	20.602
31460	4.679	376.555	68.1976
31461	4.679	347.37	304.904
31462	4.6791	353.274	72.9648
31463	4.6792	275.263	182.428
31464	4.6792	322.98	112.736
31465	4.6792	333.731	78.8596
31466	4.6793	317.044	23.6665
31467	4.6794	264.122	126.304
31468	4.6795	360.649	65.8489
31469	4.6799	300.603	39.9922
31470	4.6801	259.273	225.038
31471	4.6801	370.361	303.534
31472	4.6802	331.343	94.7981
31473	4.6802	385.18	347.35
31474	4.6803	324.959	108.582
31475	4.6805	303.362	85.0221
31476	4.6808	362.082	71.1571
31477	4.681	303.277	130.901

31478	4.681	386.051	62.7001
31479	4.681	333.219	186.469
31480	4.6814	353.101	19.5719
31481	4.6814	20.9652	116.615
31482	4.6814	318.792	103.366
31483	4.6814	299.122	86.461
31484	4.6817	259.554	134.467
31485	4.682	344.461	109.817
31486	4.6821	363.31	85.5822
31487	4.6823	328.145	342.512
31488	4.6824	421.597	319.156
31489	4.6825	438.932	132.459
31490	4.6827	367.761	118.435
31491	4.6827	299.148	120.943
31492	4.6828	329.099	69.7631
31493	4.6828	341.475	55.9979
31494	4.6828	312.067	215.029
31495	4.6829	326.927	62.6371
31496	4.6829	289.363	215.288
31497	4.6832	302.184	1.5674
31498	4.6833	348.572	245.239
31499	4.6834	395.961	38.5078
31500	4.6834	427.892	134.597
31501	4.6837	310.733	258.908
31502	4.6837	327.043	169.28
31503	4.6837	370.278	325.74
31504	4.6837	348.536	136.6
31505	4.6838	390.45	271.331
31506	4.6838	251.362	188.053
31507	4.6842	299.451	253.545
31508	4.6844	309.584	151.4
31509	4.6844	319.224	135.494
31510	4.6845	289.581	174.142
31511	4.6845	369.833	198.161
31512	4.6846	283.604	78.804
31513	4.6846	287.639	143.011
31514	4.6846	315.477	136.891
31515	4.685	359.788	359.821
31516	4.685	341.127	69.1715
31517	4.6852	332.246	116.833
31518	4.6853	343.471	57.5332
31519	4.6853	347.669	268.61
31520	4.6854	353.855	1.2458
31521	4.6854	288.376	120.861
31522	4.6855	345.147	89.7004
31523	4.6855	348.036	5.7393
31524	4.6856	322.148	352.577
31525	4.6856	298.598	238.23

31526	4.6857	258.132	75.5212
31527	4.686	293.854	195.819
31528	4.6861	306.768	41.6787
31529	4.6862	265.502	170.901
31530	4.6862	331.167	318.443
31531	4.6863	300.889	5.0807
31532	4.6863	362.614	358.292
31533	4.6863	324.803	50.2699
31534	4.6865	246.625	64.03
31535	4.6867	308.283	129.575
31536	4.6867	288.631	184.97
31537	4.687	315.3	195.384
31538	4.687	284.009	116.603
31539	4.687	317.862	320.743
31540	4.6873	318.773	0.0747
31541	4.6873	356.082	86.2226
31542	4.6874	375.265	30.9434
31543	4.6874	371.091	234.991
31544	4.6876	272.121	186.442
31545	4.6877	355.523	59.0309
31546	4.6877	282.313	146.017
31547	4.6879	-2.8057	255.629
31548	4.688	304.62	331.588
31549	4.6881	347.616	120.54
31550	4.6881	345.517	46.1732
31551	4.6882	429.791	69.4773
31552	4.6882	319.27	118.812
31553	4.6884	298.481	316.326
31554	4.6884	336.251	74.6822
31555	4.6885	381.362	252.382
31556	4.6885	314.871	228.888
31557	4.6885	345.605	61.0201
31558	4.6886	245.194	239.543
31559	4.6886	307.949	109.983
31560	4.6887	316.452	127.692
31561	4.6887	348.689	97.4971
31562	4.6887	333.991	128.081
31563	4.6887	285.468	140.618
31564	4.6888	341.769	62.7108
31565	4.6888	303.333	182.214
31566	4.6889	360.18	120.996
31567	4.6889	328.497	252.747
31568	4.689	269.158	178.9
31569	4.6892	245.586	154.779
31570	4.6893	248.332	124.483
31571	4.6895	362.242	36.8223
31572	4.6895	232.677	220.736
31573	4.6898	350.414	268.483

31574	4.6898	332.211	82.9226
31575	4.6899	340.801	139.626
31576	4.6899	229.191	115.02
31577	4.69	273.723	135.037
31578	4.6901	413.71	285.756
31579	4.6901	423.38	49.8506
31580	4.6903	385.674	45.5263
31581	4.6903	312.024	229.969
31582	4.6905	237.191	149.395
31583	4.6905	249.486	159.034
31584	4.6906	329.249	84.0842
31585	4.6906	324.544	226.78
31586	4.6907	185.7	146.71
31587	4.6911	272.569	123.257
31588	4.6911	322.006	100.079
31589	4.6912	286.642	190.484
31590	4.6912	329.065	348.621
31591	4.6913	274.529	23.5693
31592	4.6913	216.196	180.854
31593	4.6913	303.712	164.649
31594	4.6914	351.743	89.4981
31595	4.6914	352.927	233.389
31596	4.6915	282.773	324.705
31597	4.6917	270.307	128.146
31598	4.6917	309.628	92.7491
31599	4.6918	271.293	216.105
31600	4.6919	332.78	157.033
31601	4.692	317.694	100.095
31602	4.692	393.633	209.602
31603	4.6921	300.139	255.545
31604	4.6921	343.468	29.8915
31605	4.6922	317.475	286.621
31606	4.6923	280.997	164.789
31607	4.6925	268.971	164.536
31608	4.6925	335.974	259.208
31609	4.6926	250.585	111.767
31610	4.6927	301.282	148.573
31611	4.6928	323.977	125.776
31612	4.6928	367.962	63.9304
31613	4.6929	436.502	33.8512
31614	4.6929	275.062	178.098
31615	4.6929	319.623	164.587
31616	4.693	235.268	296.815
31617	4.6931	329.53	43.3229
31618	4.6931	344.77	161.837
31619	4.6932	268.628	187.609
31620	4.6933	359.065	298.769
31621	4.6934	285.41	133.322

31622	4.6935	288.77	103.4
31623	4.6935	339.44	27.4565
31624	4.6937	334.471	56.9987
31625	4.6937	344.243	153.49
31626	4.6937	335.777	335.374
31627	4.6938	287.744	123.795
31628	4.6941	361.369	110.176
31629	4.6942	302.713	286.432
31630	4.6945	271.544	241.832
31631	4.6947	295.11	143.187
31632	4.695	374.205	116.746
31633	4.695	356.167	284.748
31634	4.6952	296.969	51.9291
31635	4.6952	295.25	148.618
31636	4.6953	275.964	215.652
31637	4.6953	297.725	272.914
31638	4.6954	328.75	59.0574
31639	4.6954	380.062	292.629
31640	4.6955	367.703	146.857
31641	4.6955	259.934	168.159
31642	4.6955	313.298	83.8769
31643	4.6955	259.811	138.744
31644	4.6957	364.74	173.207
31645	4.6958	359.224	136.637
31646	4.6958	312.815	131.931
31647	4.6958	361.459	80.0269
31648	4.6959	286.225	176.925
31649	4.6959	285.933	232.286
31650	4.696	387.403	34.8088
31651	4.6961	311.535	149.067
31652	4.6961	348.494	116.605
31653	4.6962	349.026	1.7415
31654	4.6963	308.848	192.083
31655	4.6963	224.573	158.668
31656	4.6963	-2.7811	247.705
31657	4.6963	365.093	118.648
31658	4.6965	318.607	77.2417
31659	4.6965	322.588	228.626
31660	4.6966	295.427	132.435
31661	4.6967	286.821	97.8109
31662	4.6968	338.574	142.975
31663	4.6968	353.451	3.1918
31664	4.6968	374.098	129.658
31665	4.697	353.173	61.8439
31666	4.697	306.291	161.893
31667	4.6973	287.147	12.5725
31668	4.6974	301.857	223.513
31669	4.6975	396.105	38.4754

31670	4.6976	355.642	137.344
31671	4.6977	299.959	112.21
31672	4.6978	316.06	289.077
31673	4.6979	341.207	44.463
31674	4.6979	315.164	161.539
31675	4.6981	343.989	8.3166
31676	4.6982	377.894	99.8346
31677	4.6983	364.11	290.754
31678	4.6983	331.482	238.715
31679	4.6984	353.157	107.546
31680	4.6984	341.798	323.055
31681	4.6985	312.935	88.0297
31682	4.6985	289.819	129.315
31683	4.6986	250.456	184.826
31684	4.6986	332.667	191.88
31685	4.6986	312.401	108.105
31686	4.6986	372.573	28.7533
31687	4.6988	329.037	59.9539
31688	4.6988	340.344	112.805
31689	4.6988	367.934	145.287
31690	4.6989	332.489	41.9927
31691	4.6989	280.787	121.202
31692	4.699	278.129	94.608
31693	4.699	344.055	282.102
31694	4.699	294.004	170.926
31695	4.6991	303.338	172.288
31696	4.6992	340.097	251.009
31697	4.6992	363.054	250.152
31698	4.6993	359.573	230.326
31699	4.6993	311.261	12.3526
31700	4.6993	359.902	83.5546
31701	4.6993	307.651	138.515
31702	4.6993	327.114	100.865
31703	4.6994	340.508	132.748
31704	4.6994	331.142	3.7176
31705	4.6999	353.399	123.464
31706	4.7001	378.21	141.642
31707	4.7001	325.855	232.429
31708	4.7001	350.341	135.674
31709	4.7002	275.901	143.247
31710	4.7003	396.158	64.9222
31711	4.7005	257.853	139.893
31712	4.7005	347.546	295.097
31713	4.7006	338.177	57.3743
31714	4.7008	335.734	133.245
31715	4.7011	265.895	157.387
31716	4.7011	290.459	199.263
31717	4.7013	308.6	106.241

31718	4.7013	339.835	79.3493
31719	4.7014	341.93	165.741
31720	4.7015	288.777	190.273
31721	4.7017	264.93	132.537
31722	4.7019	296.5	182.353
31723	4.7021	250.226	230.759
31724	4.7022	251.752	86.4877
31725	4.7024	394.165	290.089
31726	4.7026	318.12	133.758
31727	4.7026	279.879	131.874
31728	4.7027	331.31	295.25
31729	4.7027	316.013	78.2885
31730	4.7028	250.525	131.351
31731	4.7031	302.022	80.0838
31732	4.7032	346.607	119.532
31733	4.7032	325.829	312.661
31734	4.7032	422.65	307.6
31735	4.7034	286.67	183.25
31736	4.7035	362.144	54.9964
31737	4.7035	253.449	145.451
31738	4.7036	417.674	270.675
31739	4.7037	271.31	24.6824
31740	4.7039	313.585	123.213
31741	4.704	365.009	133.021
31742	4.7042	433.443	67.5189
31743	4.7042	413.121	19.7529
31744	4.7042	361.985	124.257
31745	4.7043	329.874	267.886
31746	4.7043	325.522	97.3527
31747	4.7043	300.784	257.07
31748	4.7043	358.779	87.2653
31749	4.7043	323.557	287.59
31750	4.7046	333.473	263.51
31751	4.7046	251.113	119.967
31752	4.7046	331.415	300.575
31753	4.7047	290.467	219.181
31754	4.7049	311.52	127.279
31755	4.705	327.627	115.466
31756	4.705	312.495	277.405
31757	4.7051	339.456	327.44
31758	4.7051	366.15	63.0306
31759	4.7052	318.985	162.674
31760	4.7052	326.495	335.328
31761	4.7056	299.027	119.651
31762	4.7056	302.261	170.953
31763	4.7056	301.224	57.0749
31764	4.7058	347.901	166.864
31765	4.7059	342.261	192.588

31766	4.7059	334.928	95.0067
31767	4.706	308.882	5.1671
31768	4.7062	422.989	33.0776
31769	4.7064	275.619	90.7318
31770	4.7065	149.57	32.9766
31771	4.7066	357.162	66.6832
31772	4.7066	315.812	109.429
31773	4.7067	364.497	119.591
31774	4.7068	276.727	270.854
31775	4.7068	343.965	69.2762
31776	4.7073	300.049	219.373
31777	4.7073	406.265	210.335
31778	4.7074	341.613	82.2518
31779	4.7074	325.918	130.038
31780	4.7075	315.856	169.408
31781	4.7076	348.053	50.9136
31782	4.7077	318.767	72.9328
31783	4.7077	312.525	125.573
31784	4.7077	348.742	66.6675
31785	4.7078	367.47	75.1591
31786	4.7079	249.138	126.122
31787	4.708	404.415	310.918
31788	4.708	309.439	80.2831
31789	4.7081	264.307	202.114
31790	4.7081	315.286	95.0605
31791	4.7081	288.172	142.408
31792	4.7082	274.371	126.072
31793	4.7083	308.915	98.2313
31794	4.7084	314.976	32.7773
31795	4.7084	347.291	121.261
31796	4.7084	287.865	2.1679
31797	4.7086	393.469	46.0083
31798	4.7086	326.851	165.825
31799	4.7087	313.676	139.125
31800	4.7087	292.679	221.64
31801	4.7088	352.604	76.6937
31802	4.709	241.232	182.084
31803	4.709	357.345	178.773
31804	4.7091	320.366	152.156
31805	4.7092	234.972	183.835
31806	4.7094	353.991	124.504
31807	4.7094	318.771	136.328
31808	4.7094	283.704	192.46
31809	4.7095	275.297	152.249
31810	4.7096	336.466	69.3141
31811	4.7096	376.622	22.4968
31812	4.7098	321.401	59.0197
31813	4.7098	375.246	69.0616

31814	4.7098	313.303	61.0416
31815	4.71	289.34	96.383
31816	4.7101	314.433	117.556
31817	4.7101	262.242	60.2055
31818	4.7102	206.619	178.709
31819	4.7103	349.025	290.085
31820	4.7103	337.653	98.2729
31821	4.7105	356.06	268.813
31822	4.7105	352.649	335.429
31823	4.7106	314.651	174.701
31824	4.7107	256.291	74.5311
31825	4.7107	362.968	51.6053
31826	4.7107	322.802	119.49
31827	4.7108	263.714	198.065
31828	4.7109	373.658	145.228
31829	4.7109	277.606	238.602
31830	4.7109	390.721	30.9956
31831	4.7112	269.054	170.73
31832	4.7112	295.853	55.8587
31833	4.7113	273.98	186.099
31834	4.7113	367.928	48.2554
31835	4.7115	310.094	75.9144
31836	4.7116	390.055	53.9716
31837	4.7116	282.33	141.867
31838	4.7116	333.939	145.281
31839	4.7118	306.407	226.391
31840	4.7119	308.622	124.117
31841	4.712	360.528	65.257
31842	4.712	308.642	170.323
31843	4.7121	326.739	198.793
31844	4.7121	259.659	229.686
31845	4.7121	377.451	98.2944
31846	4.7123	287.629	233.299
31847	4.7123	329.326	134.677
31848	4.7123	418.899	74.5204
31849	4.7124	379.384	85.0536
31850	4.7124	327.99	300.214
31851	4.7125	358.948	115.3
31852	4.7126	395.862	117.077
31853	4.7128	340.709	81.5531
31854	4.7129	369.216	71.8982
31855	4.7132	388.881	49.3871
31856	4.7133	331.846	170.715
31857	4.7134	281.467	163.381
31858	4.7134	343.12	82.3205
31859	4.7135	315.874	118.275
31860	4.7135	342.501	186.056
31861	4.7135	335.764	275.654

31862	4.7136	253.136	271.94
31863	4.7138	342.702	0.8322
31864	4.7139	347.987	158.287
31865	4.7139	290.58	190.903
31866	4.714	297.959	115.394
31867	4.7141	386.768	41.9635
31868	4.7142	252.527	230.912
31869	4.7144	310.953	183.202
31870	4.7144	318.042	53.6887
31871	4.7144	280.803	114.579
31872	4.7144	352.065	273.923
31873	4.7145	389.009	306.807
31874	4.7146	370.432	317.928
31875	4.7148	219.006	161.274
31876	4.7148	356.053	142.478
31877	4.7151	371.766	65.6956
31878	4.7151	366.466	11.9632
31879	4.7151	299.497	287.752
31880	4.7152	332.804	30.0264
31881	4.7152	338.167	325.003
31882	4.7153	292.622	88.2054
31883	4.7154	345.824	232.995
31884	4.7154	317.03	175.404
31885	4.7154	280.684	77.949
31886	4.7157	308.958	101.113
31887	4.7158	358.82	314.298
31888	4.716	372.924	68.4859
31889	4.7161	307.476	298.637
31890	4.7162	385.689	81.8479
31891	4.7162	335.365	232.785
31892	4.7162	346.857	120.245
31893	4.7162	286.13	223.934
31894	4.7165	263.403	99.7087
31895	4.7166	296.774	262.02
31896	4.7167	349.863	280.996
31897	4.7167	390.159	75.2233
31898	4.7167	347.98	80.8684
31899	4.7168	353.802	110.848
31900	4.7168	376.646	29.1052
31901	4.7169	296.515	236.279
31902	4.7169	346.318	90.57
31903	4.7169	375.189	128.502
31904	4.7171	279.797	110.138
31905	4.7172	289.85	242.245
31906	4.7173	340.105	228.717
31907	4.7177	299.719	0.8033
31908	4.7179	292.782	232.5
31909	4.718	278.822	183.969

31910	4.718	303.368	224.595
31911	4.7181	341.485	85.743
31912	4.7181	410.017	49.1923
31913	4.7182	305.679	106.074
31914	4.7182	301.614	121.688
31915	4.7182	318.77	34.2233
31916	4.7182	372.61	232.238
31917	4.7184	345.509	119.691
31918	4.7185	347.69	84.9113
31919	4.7185	358.392	75.0202
31920	4.7187	365.078	105.008
31921	4.7188	304.1	171.873
31922	4.7189	388.691	58.4531
31923	4.719	331.414	109.071
31924	4.7192	350.955	115.631
31925	4.7193	388.189	283.689
31926	4.7193	332.898	331.219
31927	4.7193	355.002	314.103
31928	4.7195	384.709	49.6281
31929	4.7196	325.81	62.9137
31930	4.7196	275.288	164.51
31931	4.7197	330.541	232.635
31932	4.7197	227.833	171.449
31933	4.7199	352.367	295.803
31934	4.7201	471.414	315.527
31935	4.7201	304.25	212.218
31936	4.7201	301.242	74.1065
31937	4.7201	450.098	202.243
31938	4.7202	344.245	256.479
31939	4.7203	315.469	195.954
31940	4.7203	307.398	270.488
31941	4.7203	343.52	163.954
31942	4.7205	347.37	21.7298
31943	4.7205	291.316	187.584
31944	4.7211	320.191	192.291
31945	4.7211	269.625	138.459
31946	4.7211	303.31	106.647
31947	4.7211	340.765	132.129
31948	4.7213	381.205	154.748
31949	4.7214	308.092	62.8582
31950	4.7216	387.705	82.2969
31951	4.722	313.616	307.579
31952	4.7221	365.605	153.488
31953	4.7221	373.257	63.9103
31954	4.7221	318.993	335.647
31955	4.7222	209.962	40.9547
31956	4.7222	331.367	188.127
31957	4.7224	320.909	52.2877

31958	4.7224	331.395	115.401
31959	4.7224	339.79	113.638
31960	4.7225	240.379	280.212
31961	4.7226	320.99	300.751
31962	4.7227	343.826	245.485
31963	4.7227	303.452	133.088
31964	4.7228	312.914	200.478
31965	4.7231	316.814	66.6176
31966	4.7231	371.369	294.732
31967	4.7232	340.163	108.563
31968	4.7232	394.361	179.854
31969	4.7233	247.077	118.549
31970	4.7233	255.703	115.714
31971	4.7234	330.111	330.025
31972	4.7235	380.359	67.4477
31973	4.7235	318.251	184.628
31974	4.7236	399.172	36.6562
31975	4.7236	358.15	26.596
31976	4.7237	410.505	32.2
31977	4.7238	337.741	71.3557
31978	4.7239	316.875	121.489
31979	4.7242	334.282	166.551
31980	4.7243	338.281	326.891
31981	4.7244	321.319	175.219
31982	4.7247	302.131	167.235
31983	4.7248	396.904	203.426
31984	4.7248	240.025	212.662
31985	4.7249	364.574	206.178
31986	4.7249	352.816	52.0452
31987	4.725	292.461	152.865
31988	4.725	359.157	151.926
31989	4.7251	335.14	57.7717
31990	4.7252	363.548	47.6682
31991	4.7252	316.973	338.353
31992	4.7254	314.099	109.816
31993	4.7254	372.142	1.9526
31994	4.7254	232.966	340.365
31995	4.7255	345.431	80.8478
31996	4.7255	314.946	122.725
31997	4.7255	295.17	155.359
31998	4.7256	327.434	51.6049
31999	4.7256	328.603	158.1
32000	4.7258	219.075	171.678
32001	4.7259	261.166	140.033
32002	4.7259	355.554	73.5261
32003	4.726	338.488	319.839
32004	4.726	341.544	50.8872
32005	4.726	300.414	113.196

32006	4.7262	329.817	129.405
32007	4.7263	269.461	278.321
32008	4.7264	329.72	162.542
32009	4.7265	431.136	23.4131
32010	4.7265	313.707	273.287
32011	4.7266	316.167	104.695
32012	4.7266	347.791	52.4745
32013	4.7267	318.591	323.007
32014	4.7267	335.522	227.688
32015	4.7267	333.007	80.1994
32016	4.7267	370.295	228.446
32017	4.7268	330.279	300.288
32018	4.7272	357.977	126.767
32019	4.7272	337.928	62.7409
32020	4.7272	322.524	77.7054
32021	4.7273	235.838	196.898
32022	4.7275	388.572	128.401
32023	4.7276	371.178	101.381
32024	4.7277	314.435	214.922
32025	4.7278	366.074	145.732
32026	4.7278	431.565	24.7368
32027	4.728	284.024	145.148
32028	4.7282	363.131	137.915
32029	4.7282	410.476	312.694
32030	4.7283	314.644	107.702
32031	4.7285	320.08	116.45
32032	4.7285	321.42	236.383
32033	4.7285	315.419	98.9083
32034	4.7285	339.666	154.334
32035	4.7288	367.234	84.084
32036	4.729	298.128	78.7302
32037	4.7292	327.456	141.942
32038	4.7293	359.218	174.573
32039	4.7295	406.09	48.4548
32040	4.7295	325.845	350.999
32041	4.7295	213.058	161.049
32042	4.7295	330.339	114.251
32043	4.7295	310.731	102.948
32044	4.7295	350.454	199.846
32045	4.7297	306.308	135.139
32046	4.7298	383.718	191.666
32047	4.7298	387.756	92.2657
32048	4.7299	359.85	58.7792
32049	4.73	407.202	257.334
32050	4.73	343.108	58.6551
32051	4.7301	365.4	54.9815
32052	4.7302	388.136	54.7035
32053	4.7303	351.667	236.512

32054	4.7303	308.131	182.038
32055	4.7304	372.238	279.571
32056	4.7304	373.294	82.5896
32057	4.7304	310.952	111.193
32058	4.7306	395.462	110.187
32059	4.7306	279.112	63.3788
32060	4.7307	339.035	279.952
32061	4.7307	364.179	126.427
32062	4.731	268.715	143.501
32063	4.7312	372.421	93.8153
32064	4.7313	247.129	145.855
32065	4.7313	349.247	91.7065
32066	4.7315	377.223	187.649
32067	4.7316	398.239	68.0811
32068	4.7317	313.183	215.57
32069	4.7317	297.706	89.7798
32070	4.7318	327.295	77.3453
32071	4.7319	306.55	122.604
32072	4.7319	349.926	170.773
32073	4.7319	428.73	244.001
32074	4.7319	385.158	325.136
32075	4.7319	348.841	333.585
32076	4.7321	344.81	332.063
32077	4.7322	442.718	60.0758
32078	4.7323	350.796	149.37
32079	4.7324	350.69	272.167
32080	4.7324	348.597	90.7473
32081	4.7325	305.861	212.619
32082	4.7329	331.279	24.7128
32083	4.7329	287.801	239.265
32084	4.7329	279.484	165.125
32085	4.733	372.481	146.953
32086	4.7333	260.229	91.8295
32087	4.7333	289.592	106.201
32088	4.7333	311.47	331.607
32089	4.7335	366.974	294.004
32090	4.7336	270.415	249.289
32091	4.7338	340.995	137.925
32092	4.7339	339.606	116.692
32093	4.7341	332.183	193.6
32094	4.7342	317.342	133.944
32095	4.7343	279.978	39.4311
32096	4.7344	310.019	195.233
32097	4.7344	276.126	233.368
32098	4.7344	301.331	145.608
32099	4.7344	355.989	55.2498
32100	4.7344	304.362	120.554
32101	4.7347	356.764	56.9439

32102	4.7347	355.031	134.164
32103	4.7347	253.899	139.32
32104	4.7348	380.594	33.9109
32105	4.7348	384.171	139.588
32106	4.7349	329.246	91.3365
32107	4.735	353.403	130.432
32108	4.7351	314.72	173.031
32109	4.7351	315.484	54.1777
32110	4.7351	352.864	75.7185
32111	4.7351	266.528	93.485
32112	4.7352	286.609	131.39
32113	4.7353	318.96	181.151
32114	4.7353	278.071	296.628
32115	4.7353	378.029	29.2274
32116	4.7354	310.897	101.599
32117	4.7355	271.958	241.508
32118	4.7357	432.492	26.6765
32119	4.7357	298.209	111.18
32120	4.7358	358.95	259.938
32121	4.7358	399.312	16.1373
32122	4.7359	305.292	300.137
32123	4.736	346.937	193.767
32124	4.736	356.642	88.5877
32125	4.7361	317.216	44.2762
32126	4.7362	422.372	31.2284
32127	4.7364	366.721	202.488
32128	4.7365	275.065	141.049
32129	4.7365	385.228	76.8158
32130	4.7366	354.944	7.3949
32131	4.7366	376.251	76.6705
32132	4.7366	304.616	173.196
32133	4.7366	295.093	331.563
32134	4.7367	315.594	128.412
32135	4.7368	386.976	69.3833
32136	4.737	299.526	57.0871
32137	4.7371	329.984	77.8205
32138	4.7373	300.877	117.813
32139	4.7375	219.503	114.859
32140	4.7376	302.636	139.396
32141	4.7377	322.669	128.691
32142	4.7377	345.155	93.612
32143	4.7379	320.614	75.7607
32144	4.7379	312.279	120.306
32145	4.7379	307.232	152.493
32146	4.7381	312.491	86.0763
32147	4.7382	312.154	28.5959
32148	4.7383	303.2	251.69
32149	4.7385	290.739	130.085

32150	4.7386	414.801	55.1467
32151	4.7386	378.889	236.243
32152	4.7389	291.985	112.163
32153	4.739	315.848	341.901
32154	4.739	306.806	140.769
32155	4.7391	348.763	43.7463
32156	4.7391	282.799	19.7082
32157	4.7392	200.021	71.2662
32158	4.7393	357.461	309.348
32159	4.7396	322.865	132.917
32160	4.7397	262.574	140.122
32161	4.7397	371.635	290.435
32162	4.7398	451.129	209.474
32163	4.7398	456.864	308.171
32164	4.74	329.37	315.212
32165	4.7404	368.988	256.12
32166	4.7404	300.074	232.156
32167	4.7404	303.256	179.833
32168	4.7404	286.281	137.59
32169	4.7404	357.516	44.8627
32170	4.7405	293.043	180.964
32171	4.7405	310.817	191.291
32172	4.7408	292.713	152.369
32173	4.741	345.908	72.6512
32174	4.741	313.264	78.3666
32175	4.741	344.104	224.134
32176	4.7414	276.488	157.001
32177	4.7415	237.978	104.397
32178	4.7415	379.454	70.4955
32179	4.7416	356.502	247.687
32180	4.7416	312.542	77.8541
32181	4.7416	301.831	320.903
32182	4.7417	323.311	127.93
32183	4.7418	351.069	77.6861
32184	4.7418	437.707	26.8753
32185	4.7419	381.068	115.156
32186	4.7419	294.267	333.88
32187	4.7419	339.249	103.35
32188	4.742	246.152	215.796
32189	4.7421	302.489	176.495
32190	4.7424	319.782	187.567
32191	4.7427	378.45	70.0988
32192	4.7428	341.119	231.657
32193	4.7428	395.773	26.6465
32194	4.7428	413.974	325.008
32195	4.743	323.025	215.601
32196	4.743	345.072	77.241
32197	4.7433	337.267	189.418

32198	4.7433	319.8	121.665
32199	4.7434	379.063	82.2823
32200	4.7435	356.201	40.0806
32201	4.7437	400.373	25.4335
32202	4.7439	377.662	97.1712
32203	4.744	316.493	176.88
32204	4.744	333.565	221.024
32205	4.7441	357.41	306.597
32206	4.7442	379.664	208.914
32207	4.7442	355.794	59.3285
32208	4.7443	362.802	151.494
32209	4.7444	374.801	38.24
32210	4.7444	340.52	79.0661
32211	4.7444	275.109	86.0956
32212	4.7445	336.045	354.813
32213	4.7447	351.054	53.0744
32214	4.7448	352.193	264.289
32215	4.7448	425.952	49.5904
32216	4.7448	342.545	36.7476
32217	4.7448	367.785	78.9271
32218	4.7449	417.214	48.1011
32219	4.7449	306.381	252.126
32220	4.7449	344.673	0.6256
32221	4.7451	388.49	35.0889
32222	4.7451	375.525	7.7158
32223	4.7452	352.674	117.516
32224	4.7452	332.623	350.091
32225	4.7456	268.428	146.359
32226	4.7458	288.7	179.298
32227	4.746	292.001	89.5616
32228	4.746	319.286	44.4387
32229	4.7462	343.396	116.942
32230	4.7464	246.043	128.662
32231	4.7466	385.509	315.897
32232	4.7466	268.574	313.178
32233	4.7467	352.496	125.525
32234	4.7468	372.907	69.2906
32235	4.7468	331.607	233.03
32236	4.747	295.608	223.596
32237	4.7473	346.19	25.4332
32238	4.7474	364.027	67.5683
32239	4.7475	339.398	305.184
32240	4.7476	318.763	304.649
32241	4.7477	274.947	68.8661
32242	4.7478	299.105	122.644
32243	4.7478	282.179	198.515
32244	4.7478	325.221	136.273
32245	4.7479	361.639	219.694

32246	4.748	382.919	118.759
32247	4.748	295.697	144.63
32248	4.748	365.718	26.9289
32249	4.7482	415.416	74.9834
32250	4.7482	362.519	118.239
32251	4.7483	390.66	85.4111
32252	4.7483	256.494	11.2176
32253	4.7485	320.536	92.3188
32254	4.7485	298.037	352.773
32255	4.7485	335.389	139.946
32256	4.7486	298.171	127.642
32257	4.7486	389.503	235.053
32258	4.7487	377.219	188.083
32259	4.7488	281.629	274.499
32260	4.7489	378.934	135.488
32261	4.7491	362.173	103.139
32262	4.7491	270.39	85.6309
32263	4.7492	354.511	197.213
32264	4.7492	355.601	28.4723
32265	4.7492	332.847	253.568
32266	4.7493	371.636	62.9575
32267	4.7494	337.508	100.2
32268	4.7494	355.946	272.012
32269	4.7494	335.917	322.41
32270	4.7495	383.533	52.2543
32271	4.7496	310.977	174.559
32272	4.7496	340.982	115.943
32273	4.7496	384.569	200.393
32274	4.7497	282.827	112.442
32275	4.7497	387.108	126.156
32276	4.7501	336.483	325.102
32277	4.7502	422.184	253.605
32278	4.7503	370.723	177.311
32279	4.7506	402.12	76.1478
32280	4.7508	342.215	232.159
32281	4.7508	353.713	96.3622
32282	4.7509	256.211	242.864
32283	4.7509	415.049	12.4031
32284	4.751	358.767	181.514
32285	4.751	362.701	102.028
32286	4.751	342.709	118.444
32287	4.7512	380.027	359.692
32288	4.7516	343.465	228.149
32289	4.7516	337.824	115.506
32290	4.7517	299.649	254.168
32291	4.7518	289.55	184.748
32292	4.7518	292.071	105.46
32293	4.7522	338.124	108.726

32294	4.7523	358.84	181.077
32295	4.7523	243.754	202.329
32296	4.7523	393.123	74.6219
32297	4.7524	295.942	296.769
32298	4.7525	372.476	93.5104
32299	4.7525	342.021	248.48
32300	4.7526	322.434	351.521
32301	4.7526	278.95	124.362
32302	4.7528	419.488	339.909
32303	4.7529	312.218	220.301
32304	4.753	376.214	148.637
32305	4.7532	285.545	137.72
32306	4.7532	387.351	202.943
32307	4.7533	384.501	139.887
32308	4.7533	318.202	235.938
32309	4.7534	488.506	199.898
32310	4.7534	235.916	190.883
32311	4.7536	312.065	64.9152
32312	4.7536	278.071	64.9708
32313	4.7537	416.07	48.485
32314	4.7537	374.68	72.216
32315	4.7538	315.8	127.607
32316	4.7539	313.89	177.671
32317	4.7539	273.162	117.74
32318	4.754	408.299	48.7836
32319	4.754	367.856	124.365
32320	4.754	314.223	262.346
32321	4.7541	304.538	226.167
32322	4.7542	386.888	91.1735
32323	4.7542	299.622	146.705
32324	4.7543	344.546	92.3345
32325	4.7543	361.086	62.5707
32326	4.7544	298.896	234.324
32327	4.7544	324.864	113.746
32328	4.7547	313.616	64.8565
32329	4.7549	413.311	117.917
32330	4.755	342.946	128.917
32331	4.755	397.912	70.761
32332	4.7551	353.555	133.549
32333	4.7551	370.441	12.7479
32334	4.7553	285.984	95.7659
32335	4.7553	281.076	68.4289
32336	4.7556	418.27	35.164
32337	4.7556	335.264	73.109
32338	4.7557	346.986	10.7474
32339	4.7557	323.04	15.194
32340	4.7561	345.355	171.037
32341	4.7562	361.53	101.263

32342	4.7562	368.245	349.549
32343	4.7566	314.705	100.039
32344	4.7567	315.847	99.8256
32345	4.7569	73.3495	25.0571
32346	4.7569	337.427	93.1946
32347	4.757	457.637	83.2051
32348	4.757	267.014	129.954
32349	4.757	353.965	85.466
32350	4.7571	314.092	47.9779
32351	4.7571	317.531	227.435
32352	4.7572	281.394	182.929
32353	4.7572	373.545	120.29
32354	4.7574	423.464	128.979
32355	4.7574	297.664	133.912
32356	4.7577	293.158	135.125
32357	4.7578	243.73	146.075
32358	4.758	279.617	351.319
32359	4.7582	331.757	90.7821
32360	4.7583	330.699	86.1666
32361	4.7583	365.901	32.3352
32362	4.7584	473.419	286.871
32363	4.7584	375.908	32.7513
32364	4.7585	345.442	120.377
32365	4.7586	354.405	224.872
32366	4.7587	343.484	151.245
32367	4.7588	388.562	187.875
32368	4.7589	328.167	143.068
32369	4.7589	353.856	278.034
32370	4.759	349.549	46.9132
32371	4.7591	432.027	38.1191
32372	4.7593	292.819	248.782
32373	4.7594	415.243	313.082
32374	4.7595	243.893	172.847
32375	4.7596	308.53	59.3846
32376	4.7596	358.746	85.0193
32377	4.7597	336.405	131.859
32378	4.7599	318.462	105.739
32379	4.7599	267.804	128.552
32380	4.7601	294.651	127.505
32381	4.7602	380.085	275.805
32382	4.7603	337.581	187.72
32383	4.7603	348.283	192.788
32384	4.7603	335.477	239.085
32385	4.7604	403.145	59.1531
32386	4.7607	294.619	206.163
32387	4.7608	315.118	109.141
32388	4.7609	369.367	97.2211
32389	4.761	367.815	67.3143

32390	4.761	316.612	145.4
32391	4.7611	325.801	132.673
32392	4.7611	368.575	44.0793
32393	4.7612	402.149	131.094
32394	4.7613	389.388	242.444
32395	4.7614	332.194	135.244
32396	4.7614	282.052	232.249
32397	4.7614	350.084	67.1236
32398	4.7615	263.364	191.432
32399	4.7615	292.801	120.317
32400	4.7616	322.748	96.0864
32401	4.7618	384.22	67.4871
32402	4.762	335.81	79.7074
32403	4.7621	323.041	110.772
32404	4.7622	377.505	287.639
32405	4.7622	311.247	231.947
32406	4.7622	319.556	175.437
32407	4.7625	338.042	162.587
32408	4.7625	335.211	303.751
32409	4.7626	326.366	187.149
32410	4.7626	334.187	251.121
32411	4.7626	342.805	128.253
32412	4.7627	306.064	89.3265
32413	4.7627	353.582	7.161
32414	4.7628	355.68	82.3222
32415	4.7628	335.369	121.595
32416	4.763	272.104	148.402
32417	4.7631	351.765	260.234
32418	4.7632	361.997	53.6647
32419	4.7634	340.914	175.304
32420	4.7637	261.609	142.663
32421	4.7638	382.377	307.331
32422	4.764	372.017	131.738
32423	4.7644	325.063	123.913
32424	4.7644	364.2	65.2183
32425	4.7649	365.886	266.445
32426	4.7649	275.734	242.313
32427	4.7652	-2.7812	79.2972
32428	4.7653	365.063	298.355
32429	4.7654	258.439	221.286
32430	4.7655	327.416	86.0153
32431	4.7656	423.043	38.5728
32432	4.7656	359.585	217.165
32433	4.7656	361.77	157.682
32434	4.7657	434.784	22.6723
32435	4.7661	282.806	152.533
32436	4.7661	399.345	164.154
32437	4.7662	342.284	152.984

32438	4.7663	354.986	282.493
32439	4.7663	294.423	90.5876
32440	4.7663	371.122	96.8545
32441	4.7664	445.569	245.731
32442	4.7664	356.535	88.4884
32443	4.7665	403.698	189.205
32444	4.7666	391.485	124.662
32445	4.7668	322.628	180.527
32446	4.7669	381.829	76.3113
32447	4.767	376.206	265.336
32448	4.767	303.429	94.2233
32449	4.7674	352.011	88.8055
32450	4.7674	250.866	137.874
32451	4.7675	315.916	313.883
32452	4.7676	341.285	131.358
32453	4.7676	386.521	177.021
32454	4.7676	414.927	25.1309
32455	4.7677	324.966	239.169
32456	4.7678	391.265	128.349
32457	4.7678	346.866	236.623
32458	4.7678	367.686	72.6736
32459	4.7679	357.3	192.77
32460	4.7679	332.039	136.312
32461	4.768	358.141	121.021
32462	4.768	362.151	59.4083
32463	4.7681	270.347	184.036
32464	4.7682	367.724	73.1008
32465	4.7684	345.732	42.2429
32466	4.7686	349.733	112.078
32467	4.7687	337.395	75.3023
32468	4.7688	387.631	80.8974
32469	4.7688	302.556	113.655
32470	4.769	330.955	47.2342
32471	4.7691	323.845	181.195
32472	4.7692	351.928	134.033
32473	4.7692	353.755	165.739
32474	4.7692	327.036	286.932
32475	4.7692	311.12	219.392
32476	4.7694	366.282	31.325
32477	4.7694	349.194	83.6016
32478	4.7695	337.394	259.23
32479	4.7696	346.668	257.025
32480	4.7696	292.63	173.253
32481	4.7697	329.586	183.033
32482	4.7698	296.533	172.764
32483	4.7698	324.946	106.594
32484	4.7698	364.423	23.1132
32485	4.7699	350.668	235.964

32486	4.7699	151.437	199.704
32487	4.7699	419	25.1405
32488	4.7701	399.649	45.035
32489	4.7702	350.37	247.978
32490	4.7705	239.786	261.885
32491	4.7705	268.821	323.28
32492	4.7705	424.381	243.399
32493	4.7706	368.242	317.127
32494	4.7706	355.954	88.907
32495	4.7707	372.07	317.952
32496	4.771	332.201	98.6927
32497	4.7711	422.55	50.4036
32498	4.7713	314.341	87.0406
32499	4.7713	322.478	208.011
32500	4.7714	254.685	121.404
32501	4.7715	425.205	91.4374
32502	4.7716	331.78	281.703
32503	4.7716	358.165	29.7793
32504	4.7716	-2.7522	11.6449
32505	4.7717	380.806	91.4987
32506	4.7717	350.335	95.2867
32507	4.7718	243.664	84.9043
32508	4.7719	358.258	292.118
32509	4.772	357.897	160.947
32510	4.7722	354.688	5.8596
32511	4.7723	398.706	326.584
32512	4.7724	388.61	27.2275
32513	4.7724	399.029	41.0778
32514	4.7726	336.433	71.9733
32515	4.773	318.47	151.869
32516	4.7731	399.734	34.2818
32517	4.7732	417.753	30.115
32518	4.7732	343.169	151.589
32519	4.7733	346.902	97.3053
32520	4.7733	317.253	134.337
32521	4.7735	381.098	150.009
32522	4.7737	253.429	318.486
32523	4.7738	297.066	180.304
32524	4.774	439.202	61.2159
32525	4.774	298.152	48.5763
32526	4.774	343.438	138.892
32527	4.774	385.028	233.851
32528	4.7741	240.72	134.859
32529	4.7741	369.061	90.2245
32530	4.7742	386.343	284.221
32531	4.7745	330.131	85.3274
32532	4.7745	352.86	73.8788
32533	4.7745	304.627	290.901

32534	4.7745	320.588	336.351
32535	4.7746	409.247	81.1802
32536	4.7747	330.075	178.139
32537	4.7747	243.482	302.546
32538	4.7747	322.177	191.91
32539	4.775	350.181	95.1966
32540	4.775	382.094	309.805
32541	4.7751	354.941	345.047
32542	4.7753	406.753	272.953
32543	4.7754	252.296	160.226
32544	4.7755	360.945	94.2823
32545	4.7756	323.91	130.055
32546	4.7757	378.214	217.146
32547	4.7758	317.521	230.156
32548	4.7758	338.997	125.94
32549	4.7759	239.474	148.078
32550	4.7761	350.481	107.659
32551	4.7764	391.676	60.7582
32552	4.7766	328.176	260.932
32553	4.7767	339.167	163.446
32554	4.7767	297.769	272.598
32555	4.7767	417.136	21.2842
32556	4.7768	367.809	36.543
32557	4.7769	298.83	138.817
32558	4.7769	370.346	268.133
32559	4.7771	306.709	55.8083
32560	4.7773	423.591	307.526
32561	4.7773	361.946	102.133
32562	4.7773	375.541	20.6348
32563	4.7776	436.032	37.3248
32564	4.7776	417.594	357.95
32565	4.7776	331.099	168.172
32566	4.7777	352.835	75.4693
32567	4.7778	367.965	24.2092
32568	4.7779	418.177	29.844
32569	4.778	379.135	184.162
32570	4.7781	304.717	211.218
32571	4.7782	409.53	84.1447
32572	4.7782	382.658	47.5716
32573	4.7782	349.489	55.8257
32574	4.7783	390.378	58.2394
32575	4.7784	359.054	263.605
32576	4.7784	302.799	231.592
32577	4.7784	348.55	119.633
32578	4.7784	417.476	30.3253
32579	4.7784	393.373	331.714
32580	4.7787	300.092	184.483
32581	4.7788	406.755	92.6692

32582	4.7789	399.19	60.8258
32583	4.7789	370.22	182.681
32584	4.779	363.444	133.045
32585	4.779	342.347	63.1753
32586	4.779	355.332	58.0696
32587	4.7791	373.473	180.757
32588	4.7791	396.579	271.757
32589	4.7792	401.618	51.2377
32590	4.7796	349.752	272.366
32591	4.7797	272.16	155.486
32592	4.7797	279.786	175.205
32593	4.7798	320.297	147.673
32594	4.7798	300.384	156.565
32595	4.7799	340.288	87.9736
32596	4.7801	397.431	1.9161
32597	4.7801	345.118	179.849
32598	4.7802	310.295	64.8133
32599	4.7802	357.434	190.065
32600	4.7803	292.774	171.282
32601	4.7804	317.867	185.62
32602	4.7804	335.585	126.144
32603	4.7805	331.176	331.309
32604	4.7806	334.617	66.3848
32605	4.7807	396.328	83.9708
32606	4.7808	357.954	189.604
32607	4.7808	270.736	168.788
32608	4.781	356.597	71.4976
32609	4.781	300.318	133.846
32610	4.7812	304.182	152.475
32611	4.7813	308.715	125.082
32612	4.7815	368.095	32.7462
32613	4.7815	358.492	63.6445
32614	4.7816	328.23	134.378
32615	4.7817	370.231	151.708
32616	4.7817	365.288	225.759
32617	4.7817	344.433	305.15
32618	4.7818	365.976	176.818
32619	4.7819	343.04	116.815
32620	4.7819	308.533	196.331
32621	4.7819	337.008	318.919
32622	4.782	325.835	188.966
32623	4.7821	273.911	349.474
32624	4.7821	412.757	52.219
32625	4.7821	326.596	144.203
32626	4.7822	244.307	342.062
32627	4.7824	369.838	283.002
32628	4.7825	343.71	205.91
32629	4.7825	426.73	305.613

32630	4.7827	315.37	170.661
32631	4.7828	292.203	111.392
32632	4.7831	352.423	72.1165
32633	4.7832	372.522	256.75
32634	4.7832	315.126	190.577
32635	4.7832	384.543	120.462
32636	4.7834	244.466	207.534
32637	4.7834	372.682	171.347
32638	4.7834	285.37	60.4072
32639	4.7834	348.526	121.507
32640	4.7835	412.472	47.1207
32641	4.7838	405.572	119.921
32642	4.7839	306.986	10.4987
32643	4.784	390.623	55.1605
32644	4.7841	326.147	131.672
32645	4.7842	339.46	54.7794
32646	4.7842	368.964	128.641
32647	4.7846	396.547	73.6554
32648	4.7846	317.288	179.82
32649	4.7849	336.861	40.1503
32650	4.785	410.807	55.6761
32651	4.785	269.857	137.407
32652	4.7851	360.589	284.243
32653	4.7852	253.008	44.4302
32654	4.7853	342.504	133.463
32655	4.7853	353.092	224.798
32656	4.7855	380.66	43.2932
32657	4.7856	351.628	292.148
32658	4.7857	339.081	30.3794
32659	4.7859	359.271	129.961
32660	4.7859	285.244	222.327
32661	4.7859	311.634	159.197
32662	4.7859	359.834	165.299
32663	4.786	274.056	57.5033
32664	4.7861	297.194	240.826
32665	4.7862	367.802	317.957
32666	4.7863	409.804	45.3189
32667	4.7865	290.589	74.9897
32668	4.7867	368.026	64.5484
32669	4.7868	324.331	133.143
32670	4.7869	339.855	38.3609
32671	4.7869	347.482	131.702
32672	4.787	355.808	142.385
32673	4.7871	413.421	81.0869
32674	4.7872	306.398	127.856
32675	4.7872	377.054	209.773
32676	4.7878	300.151	55.8404
32677	4.7881	380.859	42.3729

32678	4.7881	287.542	179.863
32679	4.7881	379.516	23.2635
32680	4.7883	343.865	100.384
32681	4.7883	345.118	27.2642
32682	4.7884	419.577	12.4766
32683	4.7885	433.791	11.7918
32684	4.7888	354.105	140.776
32685	4.7888	307.775	83.1414
32686	4.7889	320.351	249.593
32687	4.7889	263.12	168.017
32688	4.7889	305.581	116.03
32689	4.7889	364.999	164.988
32690	4.7891	377.354	323.727
32691	4.7892	355.98	78.1202
32692	4.7893	387.819	301.256
32693	4.7894	326.558	104.088
32694	4.7895	340.988	334.379
32695	4.7895	283.981	170.02
32696	4.7896	296.396	197.972
32697	4.7897	379.645	130.392
32698	4.7899	399.449	144.166
32699	4.7899	285.834	126.775
32700	4.7899	337.98	154.828
32701	4.79	364.714	80.807
32702	4.7902	271.695	242.835
32703	4.7902	335.803	134.308
32704	4.7903	399.132	32.3677
32705	4.7903	317.519	166.928
32706	4.7905	388.518	145.595
32707	4.7905	370.48	95.6858
32708	4.7905	347.015	171.426
32709	4.7905	274.223	122.976
32710	4.7906	428.951	70.985
32711	4.7907	363.235	241.931
32712	4.7907	372.76	79.3182
32713	4.7907	408.957	27.7945
32714	4.7908	338.107	177.271
32715	4.7908	319.658	191.145
32716	4.7909	395.585	77.6079
32717	4.7909	367.138	4.7917
32718	4.7909	451.764	12.3724
32719	4.791	349.946	78.6552
32720	4.7911	369.722	53.0234
32721	4.7912	288.132	121.386
32722	4.7913	394.868	39.5784
32723	4.7913	383.886	122.863
32724	4.7914	438.556	112.837
32725	4.7914	310.219	168.461

32726	4.7916	378.364	2.8357
32727	4.7916	298.636	153.152
32728	4.7918	391.222	119.744
32729	4.7918	345.854	82.8225
32730	4.7919	324.187	122.4
32731	4.7921	294.522	230.565
32732	4.7922	285.665	164.181
32733	4.7923	375.991	83.3468
32734	4.7923	320.013	130.063
32735	4.7924	381.033	79.1857
32736	4.7926	280.901	227.91
32737	4.7926	379.946	323.937
32738	4.7929	257.158	226.811
32739	4.793	356.994	101.368
32740	4.7931	352.213	130.729
32741	4.7933	460.61	287.911
32742	4.7933	280.63	299.758
32743	4.7933	395.805	53.4588
32744	4.7934	315.761	45.1228
32745	4.7935	350.39	122.906
32746	4.7936	350.934	190.901
32747	4.7937	367.045	177.72
32748	4.7938	435.755	49.3502
32749	4.7938	256.665	162.311
32750	4.7939	321.368	112.105
32751	4.794	333.844	324.561
32752	4.794	404.664	38.6792
32753	4.7943	391.543	83.6023
32754	4.7944	238.068	132.181
32755	4.7944	344.814	296.987
32756	4.7945	361.825	323.148
32757	4.7945	363.905	63.1222
32758	4.7945	359.103	319.415
32759	4.7946	398.084	96.0993
32760	4.7947	389.961	93.1775
32761	4.7947	325.769	262.905
32762	4.7947	247.714	161.688
32763	4.7948	393.773	83.1871
32764	4.795	304.204	239.701
32765	4.7953	259.749	175.741
32766	4.7955	405.474	250.758
32767	4.7957	440.541	36.7893
32768	4.7958	375.624	100.896
32769	4.796	340.12	136.543
32770	4.796	371.265	75.7675
32771	4.7962	364.653	129.126
32772	4.7962	229.193	45.0144
32773	4.7962	351.676	38.6587

32774	4.7962	356.456	81.3484
32775	4.7963	423.63	53.2803
32776	4.7963	362.253	352.156
32777	4.7964	352.736	97.1055
32778	4.7964	376.744	130.867
32779	4.7967	375.982	60.5094
32780	4.7967	311.017	312.583
32781	4.7968	55.6121	66.022
32782	4.7969	358.062	46.8995
32783	4.7971	332.737	52.1871
32784	4.7972	305.971	115.243
32785	4.7973	366.113	61.7245
32786	4.7973	321.071	132.719
32787	4.7975	316.033	126.343
32788	4.7975	303.173	237.634
32789	4.7977	310.428	175.17
32790	4.7978	398.717	76.1045
32791	4.7979	340.387	125.749
32792	4.7981	282.974	28.6588
32793	4.7981	414.819	36.3844
32794	4.7981	239.596	226.754
32795	4.7983	336.6	116.005
32796	4.7984	339.835	153.62
32797	4.7985	369.838	230.217
32798	4.7986	362.039	272.392
32799	4.7986	344.088	189.592
32800	4.7988	346.532	236.272
32801	4.7989	287.719	185.518
32802	4.7989	380.905	65.6329
32803	4.799	405.618	301.57
32804	4.7991	299.322	221.736
32805	4.7991	310.61	19.4792
32806	4.7993	339.13	21.4581
32807	4.7993	356.928	293.804
32808	4.7994	360.999	96.2471
32809	4.7995	259.421	128.578
32810	4.7996	267.242	0.2565
32811	4.7997	372.497	28.1601
32812	4.7998	340.592	120.821
32813	4.7999	289.152	230.366
32814	4.7999	331.179	178.467
32815	4.8	233.329	65.6998
32816	4.8003	277.367	49.9454
32817	4.8006	447.38	45.9454
32818	4.8007	377.746	25.6467
32819	4.8008	396.971	141.566
32820	4.8008	388.854	235.333
32821	4.8008	333.837	333.512

32822	4.8008	329.245	234.515
32823	4.8011	384.576	123.023
32824	4.8012	386.116	65.6363
32825	4.8012	343.177	289.742
32826	4.8013	348.824	69.3967
32827	4.8013	303.055	73.7321
32828	4.8015	355.689	73.2728
32829	4.8015	355.406	135.537
32830	4.8017	382.574	54.5423
32831	4.8017	340.004	102.622
32832	4.8018	287.512	262.543
32833	4.8018	332.472	175.556
32834	4.802	299.717	231.945
32835	4.8021	317.256	184.468
32836	4.8023	330.894	86.5005
32837	4.8024	320.52	12.4053
32838	4.8026	360.598	227.945
32839	4.8028	350.933	125.047
32840	4.8028	278.978	77.291
32841	4.803	308.068	213.079
32842	4.8031	417.845	62.2669
32843	4.8033	338.032	103.189
32844	4.8033	321.238	148.692
32845	4.8033	262.961	223.193
32846	4.8034	294.079	180.194
32847	4.8034	336.277	260.142
32848	4.8035	348.425	301.447
32849	4.8038	318.115	175.154
32850	4.8039	330.529	181.307
32851	4.804	315.05	184.337
32852	4.804	376.1	49.1147
32853	4.8041	362.795	224.51
32854	4.8041	266.204	112.252
32855	4.8042	375.655	92.1549
32856	4.8042	328.974	278.448
32857	4.8042	356.786	184.325
32858	4.8043	316.924	133.905
32859	4.8046	235.569	165.566
32860	4.8047	392.902	97.8054
32861	4.8048	367.026	138.548
32862	4.8048	351.994	185.987
32863	4.805	288.948	231.834
32864	4.805	295.728	180.657
32865	4.8052	326.763	240.25
32866	4.8052	330.445	286.989
32867	4.8053	410.902	236.71
32868	4.8053	315.237	123.006
32869	4.8053	370.731	52.2762

32870	4.8054	387.189	119.976
32871	4.8054	396.744	82.2777
32872	4.8055	278.103	122.473
32873	4.8059	371.466	40.5318
32874	4.8059	350.535	51.1957
32875	4.806	319.543	2.8282
32876	4.8061	349.818	131.877
32877	4.8061	358.364	102.14
32878	4.8061	373.097	88.471
32879	4.8062	399.632	53.7088
32880	4.8063	359.421	280.585
32881	4.8066	394.627	228.187
32882	4.8069	332.928	126.852
32883	4.8071	356.273	322.6
32884	4.8071	377.454	280.602
32885	4.8071	390.605	88.2617
32886	4.8071	356.119	92.3592
32887	4.8071	427.125	286.815
32888	4.8072	346.606	181.494
32889	4.8072	335.023	72.6823
32890	4.8073	243.348	266.427
32891	4.8073	348.51	85.089
32892	4.8073	257.348	26.3974
32893	4.8073	369.06	110.374
32894	4.8074	337.57	102.984
32895	4.8074	304.009	339.322
32896	4.8075	406.782	292.967
32897	4.8075	300.455	126.103
32898	4.8076	314.987	176.866
32899	4.8077	375.496	124.424
32900	4.8077	382.19	65.8559
32901	4.8078	329.26	78.6914
32902	4.8078	364.85	314.51
32903	4.8079	332.058	336.384
32904	4.8081	365.469	122.173
32905	4.8081	313.795	174.907
32906	4.8081	367.706	263.443
32907	4.8081	383.742	110.106
32908	4.8084	335.786	199.981
32909	4.8085	403.592	65.5198
32910	4.8086	406.158	205.922
32911	4.8088	348.039	276.5
32912	4.8089	354.062	303.397
32913	4.8091	306.139	178.21
32914	4.8091	325.006	176.45
32915	4.8092	356.548	136.262
32916	4.8093	424.045	240.615
32917	4.8094	356.662	225.282

32918	4.8094	360.317	237.936
32919	4.8095	370.995	90.4086
32920	4.8096	392.326	83.2018
32921	4.8096	381.657	67.3684
32922	4.8096	358.244	104.431
32923	4.8097	438.126	243.879
32924	4.8098	377.193	86.9373
32925	4.8099	374.966	145.855
32926	4.8101	280.117	300.789
32927	4.8103	334.873	122.021
32928	4.8105	357.322	170.261
32929	4.8105	361.997	231.386
32930	4.8106	391.343	230.633
32931	4.8107	330.682	116.948
32932	4.8108	451.249	80.7455
32933	4.8108	321.666	126.676
32934	4.8112	323.481	258.422
32935	4.8112	350.93	48.3116
32936	4.8113	371.85	80.2321
32937	4.8114	362.263	265.573
32938	4.8114	287.712	142.592
32939	4.8114	267.81	79.0356
32940	4.8115	413.681	62.5385
32941	4.8115	303.879	96.2617
32942	4.8116	342.68	100.651
32943	4.8119	394.461	149.153
32944	4.812	347.873	113.53
32945	4.8123	304.47	101.361
32946	4.8123	495.374	318.903
32947	4.8124	324.994	181.931
32948	4.8126	330.209	177.192
32949	4.8127	325.93	129.449
32950	4.813	318.958	286.74
32951	4.8131	375.267	112.229
32952	4.8131	340.849	290.353
32953	4.8131	384.637	167.055
32954	4.8134	316.588	188.506
32955	4.8135	312.672	128.128
32956	4.8136	283.193	236.69
32957	4.8138	356.789	159.938
32958	4.8139	377.047	124.02
32959	4.814	424.275	100.893
32960	4.8142	296.466	50.6407
32961	4.8143	356.941	291.103
32962	4.8145	385.008	90.4118
32963	4.8145	394.285	138.641
32964	4.8146	469.926	243.986
32965	4.8147	371.791	44.9298

32966	4.8148	356.065	196.518
32967	4.8148	357.142	125.416
32968	4.8148	367.805	206.94
32969	4.8148	377.864	302.884
32970	4.815	414.928	82.1073
32971	4.8151	366.025	250.951
32972	4.8151	325.89	159.962
32973	4.8151	395.324	269.29
32974	4.8152	346.541	92.5842
32975	4.8152	268.903	25.9955
32976	4.8153	318.905	182.957
32977	4.8153	215.43	46.981
32978	4.8153	437.025	198.995
32979	4.8154	324.054	72.9704
32980	4.8155	284.27	323.046
32981	4.8155	373.597	173.327
32982	4.8156	391.672	36.1451
32983	4.8157	305.948	320.306
32984	4.8157	351.928	48.0107
32985	4.8158	336.965	21.7274
32986	4.8161	339.863	226.576
32987	4.8163	426.949	45.1016
32988	4.8164	373.081	67.1531
32989	4.8165	309.072	82.0164
32990	4.8165	298.11	176.344
32991	4.8165	330.219	227.184
32992	4.817	302.33	224.059
32993	4.817	314.385	131.871
32994	4.8171	388.432	193.24
32995	4.8171	307.682	162.061
32996	4.8172	342.177	227.845
32997	4.8173	322.597	20.896
32998	4.8175	313.517	138.838
32999	4.8176	445.404	72.3056
33000	4.8176	344.829	239.245
33001	4.8177	342.272	53.3578
33002	4.8177	239.73	115.191
33003	4.8177	379.763	43.6957
33004	4.8178	363.417	136.46
33005	4.8179	441.45	9.2565
33006	4.8179	426.434	21.8918
33007	4.818	357.342	345.429
33008	4.818	335.438	127.024
33009	4.8181	371.866	117.119
33010	4.8181	364.654	92.4652
33011	4.8181	357.518	74.2255
33012	4.8185	399.202	109.976
33013	4.8185	302.787	196.256

33014	4.8187	342.373	161.747
33015	4.8187	365.855	102.831
33016	4.8189	253.331	164.906
33017	4.819	280.607	137.296
33018	4.819	405.056	23.5725
33019	4.819	333.759	67.1158
33020	4.8191	326.622	101.472
33021	4.8191	292.043	86.7173
33022	4.8192	387.449	47.1935
33023	4.8192	360.192	59.9278
33024	4.8193	418.903	70.0641
33025	4.8193	400.088	109.611
33026	4.8194	359.613	130.761
33027	4.8195	402.95	86.2871
33028	4.8196	378.86	215.008
33029	4.8196	352.924	90.2087
33030	4.8198	385.977	49.3563
33031	4.8199	420.891	83.3199
33032	4.8202	358.058	176.325
33033	4.8203	294.965	228.556
33034	4.8204	391.954	31.3165
33035	4.8205	373.422	342.172
33036	4.8205	280.895	225.165
33037	4.8205	300.009	58.5678
33038	4.8207	345.794	89.3833
33039	4.8208	340.416	164.867
33040	4.8209	363.976	315.934
33041	4.821	397.081	211.302
33042	4.821	427.316	33.3484
33043	4.821	380.457	321.517
33044	4.821	326.996	354.318
33045	4.8211	413.832	302.848
33046	4.8211	306.5	144.619
33047	4.8211	361.379	4.5185
33048	4.8212	405.815	208.269
33049	4.8213	451.684	34.5311
33050	4.8213	336.467	267.56
33051	4.8214	252.532	184.342
33052	4.8214	362.744	77.0287
33053	4.8215	401.918	279.184
33054	4.8215	382.519	151.962
33055	4.8215	341.08	192.586
33056	4.8215	370.516	232.903
33057	4.8216	345.327	124.028
33058	4.8218	183.044	50.9467
33059	4.822	355.531	232.722
33060	4.822	284.21	307.554
33061	4.822	416.346	54.4564

33062	4.8221	304.062	80.2578
33063	4.8222	322.535	177.099
33064	4.8224	350.963	21.1155
33065	4.8225	413.781	70.2388
33066	4.8227	337.405	88.5133
33067	4.8229	366.335	84.2409
33068	4.8233	359.125	287.912
33069	4.8233	349.447	130.972
33070	4.8234	273.416	156.234
33071	4.8235	351.573	125.943
33072	4.8236	290.381	31.7385
33073	4.8236	395.885	72.6089
33074	4.8237	310.732	200
33075	4.8239	329.562	165.335
33076	4.824	373.481	17.2634
33077	4.8241	327.974	295.592
33078	4.8241	315.436	329.913
33079	4.8241	310.353	172.406
33080	4.8241	375.764	122.147
33081	4.8242	422.588	304.291
33082	4.8244	319.839	122.79
33083	4.8245	409.034	289.399
33084	4.8246	302.612	295.338
33085	4.8248	329.195	222.385
33086	4.8248	374.348	28.792
33087	4.825	415.612	131.516
33088	4.825	391.696	134.27
33089	4.8251	343.995	101.621
33090	4.8251	389.309	231.214
33091	4.8253	437.288	119.103
33092	4.8253	339.774	9.5696
33093	4.8254	446.111	119.751
33094	4.8254	390.141	206.623
33095	4.8255	317.438	254.888
33096	4.8258	391.052	36.324
33097	4.8259	333.25	128.774
33098	4.8262	404.732	61.9954
33099	4.8264	430.049	61.7317
33100	4.8264	452.672	66.9968
33101	4.8264	320.727	193.467
33102	4.8264	333.569	133.884
33103	4.8264	370.379	127.171
33104	4.8265	418.284	189.852
33105	4.8265	275.537	294.84
33106	4.8267	385.065	41.0997
33107	4.8269	381.508	84.6116
33108	4.8271	273.576	225.978
33109	4.8272	366.093	86.8715

33110	4.8273	424.551	58.9842
33111	4.8274	344.7	283.127
33112	4.8276	385.987	128.778
33113	4.8277	415.828	186.726
33114	4.8277	377.862	309.635
33115	4.8279	317.626	231.826
33116	4.828	341.125	151.637
33117	4.828	340.328	130.458
33118	4.8281	386.058	18.7375
33119	4.8283	374.808	75.8862
33120	4.8285	358.05	30.2269
33121	4.8285	493.244	311.976
33122	4.8286	385.199	79.3265
33123	4.8286	323.06	277.686
33124	4.8287	357.849	289.538
33125	4.8288	452.911	88.3519
33126	4.8288	361.008	298.293
33127	4.8288	370.095	76.8069
33128	4.8289	407.539	45.1701
33129	4.8289	345.87	175.78
33130	4.829	333.092	172.841
33131	4.8292	355.974	123.524
33132	4.8293	387.679	191.791
33133	4.8294	355.549	127.5
33134	4.8295	406.262	38.2858
33135	4.8298	365.515	143.138
33136	4.8298	301.997	77.3948
33137	4.8298	422.995	31.5784
33138	4.8299	376.463	214.093
33139	4.83	363.719	87.0082
33140	4.83	388.067	168.545
33141	4.8301	370.07	103.627
33142	4.8302	356.082	130.35
33143	4.8302	401.756	156.393
33144	4.8305	350.219	61.6545
33145	4.8306	418.536	80.2922
33146	4.8307	367.197	60.1045
33147	4.8308	312.587	192.882
33148	4.8309	374.599	76.4176
33149	4.8309	368.877	116.119
33150	4.8309	316.105	246.931
33151	4.831	327.479	47.061
33152	4.8311	313.332	111.357
33153	4.8312	345.488	169.74
33154	4.8313	405.821	101.461
33155	4.8314	372.304	116.138
33156	4.8315	386.322	20.9964
33157	4.8315	288.182	181.072

33158	4.8315	344.837	47.0194
33159	4.8317	436.801	195.735
33160	4.8317	306.97	70.6621
33161	4.832	431.456	97.6777
33162	4.8321	318.73	250.752
33163	4.8321	307.711	244.912
33164	4.8322	109.739	187.007
33165	4.8324	393.858	271.097
33166	4.8328	327.912	162.476
33167	4.8328	336.383	104.581
33168	4.833	394.456	91.6999
33169	4.8334	349.227	247.486
33170	4.8334	393.023	85.3597
33171	4.8338	444.248	119.372
33172	4.8338	402.921	69.6
33173	4.8338	266.051	234.043
33174	4.8341	341.531	87.1233
33175	4.8342	296.75	90.3371
33176	4.8342	382.366	73.3265
33177	4.8344	380.9	339.109
33178	4.8347	99.2264	25.8293
33179	4.8347	277.979	222.271
33180	4.8347	370.669	0.227
33181	4.8347	398.132	14.6133
33182	4.8348	336.078	44.6227
33183	4.8348	324.595	32.1894
33184	4.8349	316.161	191.553
33185	4.8352	389.947	91.3835
33186	4.8352	381.593	220.935
33187	4.8354	365.017	266.946
33188	4.8354	334.598	249.046
33189	4.8355	365.808	85.5089
33190	4.8355	333.23	97.5008
33191	4.8356	323.067	156.804
33192	4.8357	321.833	150.524
33193	4.8357	320.973	233.962
33194	4.8357	412.925	75.8837
33195	4.8359	364.513	62.4949
33196	4.8359	372.619	83.1447
33197	4.8359	248.165	341.104
33198	4.836	316.767	143.54
33199	4.836	315.575	189.604
33200	4.8361	379.8	45.0329
33201	4.8361	365.591	120.631
33202	4.8366	393.69	240.512
33203	4.8366	334.963	304.045
33204	4.8368	324.821	185.658
33205	4.8371	453.922	9.6045

33206	4.8371	334.51	134.785
33207	4.8371	337.542	59.2054
33208	4.8372	297.877	169.84
33209	4.8374	437.641	82.6502
33210	4.8377	350.281	66.628
33211	4.8379	358.405	164.441
33212	4.8379	310.595	91.2315
33213	4.838	411.359	242.322
33214	4.8381	396.013	119.952
33215	4.8381	360.583	102.022
33216	4.8382	382.292	103.375
33217	4.8384	434.257	30.4122
33218	4.8384	424.485	39.4502
33219	4.8387	376.713	64.0357
33220	4.8387	410.609	266.629
33221	4.8388	426.962	121.604
33222	4.8388	347.701	183.447
33223	4.8388	327.234	190.315
33224	4.839	381.501	67.577
33225	4.839	357.388	49.3433
33226	4.839	363.881	80.6555
33227	4.839	372.999	299.011
33228	4.8393	317.788	176.243
33229	4.8394	430.838	39.5967
33230	4.8395	345.254	84.6044
33231	4.8396	321.613	128.634
33232	4.8397	399.437	60.3953
33233	4.8398	330.674	62.7561
33234	4.8399	367.732	75.8177
33235	4.8399	276.981	171.637
33236	4.84	282.04	278.355
33237	4.8403	381.102	58.6706
33238	4.8406	397.205	85.1622
33239	4.8409	435.468	70.6406
33240	4.8409	325.373	130.472
33241	4.841	364.329	65.5496
33242	4.8412	300.827	251.936
33243	4.8412	392.026	99.153
33244	4.8413	368.021	103.311
33245	4.8414	353.986	64.6404
33246	4.8418	372.074	165.775
33247	4.8419	326.724	179.956
33248	4.8421	379.467	143.892
33249	4.8421	367.078	82.4897
33250	4.8422	358.739	90.4604
33251	4.8424	332.553	133.954
33252	4.8424	378.74	276.827
33253	4.8425	302.151	234.014

33254	4.8425	334.987	329.097
33255	4.8426	327.426	169.759
33256	4.8427	357.058	60.5558
33257	4.8428	452.376	24.3737
33258	4.843	407.209	110.119
33259	4.8431	347.6	134.747
33260	4.8431	319.598	31.9203
33261	4.8432	384.083	67.5805
33262	4.8432	320.623	79.2857
33263	4.8433	437.227	55.4374
33264	4.8435	363.125	214.734
33265	4.8437	304.752	98.2513
33266	4.8437	352.278	133.109
33267	4.8442	462.955	76.9473
33268	4.8442	380.763	52.9015
33269	4.8443	350.565	136.604
33270	4.8444	355.92	116.195
33271	4.8445	429.323	49.2053
33272	4.8446	279.481	158.753
33273	4.8448	383.397	64.8355
33274	4.8448	358.556	113.691
33275	4.8449	378.25	171.841
33276	4.845	397.284	85.5603
33277	4.8451	385.812	297.904
33278	4.8452	512.504	19.2669
33279	4.8454	284.845	194.483
33280	4.8456	300.575	128.337
33281	4.8457	386.968	61.9521
33282	4.846	344.759	149.43
33283	4.8462	264.495	214.143
33284	4.8463	385.205	110.993
33285	4.8464	441.643	29.315
33286	4.8466	396.026	45.4931
33287	4.8466	367.645	340.215
33288	4.8467	291.781	120.565
33289	4.8468	438.579	63.6634
33290	4.8468	380.597	313.366
33291	4.8473	334.151	340.525
33292	4.8473	350.005	219.101
33293	4.8474	445.318	210.086
33294	4.8476	428.094	94.6776
33295	4.8476	349.127	229.595
33296	4.8477	246.126	120.167
33297	4.8477	302.972	109.223
33298	4.8477	356.003	60.2328
33299	4.8477	419.658	28.2147
33300	4.8478	349.784	256.238
33301	4.8479	321.776	129.807

33302	4.848	295.669	117.27
33303	4.8481	408.87	0.1325
33304	4.8482	416.826	229.838
33305	4.8484	396.159	81.5885
33306	4.8485	403.775	321.863
33307	4.8485	344.112	98.6225
33308	4.8485	339.485	72.8186
33309	4.8486	317.789	135.845
33310	4.8487	315.333	200.314
33311	4.8488	426.001	295.34
33312	4.8489	378.167	89.0768
33313	4.8489	363.219	104.44
33314	4.849	395.636	320.439
33315	4.849	415.09	192.68
33316	4.849	369.575	126.51
33317	4.849	340.549	122.098
33318	4.8492	295.576	202.357
33319	4.8492	259.632	123.952
33320	4.8493	463.103	7.7023
33321	4.8493	285.136	82.7741
33322	4.8495	405.897	66.0313
33323	4.8496	309.966	135.018
33324	4.8497	321.072	48.6976
33325	4.8498	366.731	159.88
33326	4.8498	349.475	177.936
33327	4.8503	314.101	64.8179
33328	4.8504	274.778	103.581
33329	4.8507	251.769	168.641
33330	4.851	375.415	83.9344
33331	4.8512	383.767	135.27
33332	4.8512	332.6	275.215
33333	4.8512	346.93	135.488
33334	4.8515	429.737	56.3168
33335	4.8515	363.087	226.629
33336	4.8516	375.838	90.2669
33337	4.8517	445.092	53.4283
33338	4.8517	426.024	314.414
33339	4.8518	377.359	44.3383
33340	4.8519	275.007	185.639
33341	4.8519	373.068	117.964
33342	4.8521	370.689	136.338
33343	4.8522	385.935	287.307
33344	4.8523	269.412	177.096
33345	4.8524	378.265	165.519
33346	4.8524	404.345	20.6846
33347	4.8525	437.33	23.4942
33348	4.8529	350.013	193.026
33349	4.8529	369.416	41.5342

33350	4.8529	317.611	97.6539
33351	4.8531	385.197	64.5432
33352	4.8531	352.741	155.186
33353	4.8532	276.346	144.05
33354	4.8532	349.984	29.0533
33355	4.8533	415.411	326.832
33356	4.8534	297.087	19.6918
33357	4.8534	376.431	163.728
33358	4.8534	361.86	108.197
33359	4.8534	208.658	135.473
33360	4.8535	314.194	194.56
33361	4.8535	58.5303	330.584
33362	4.8535	411.443	51.6345
33363	4.8537	393.674	288.58
33364	4.8537	433.672	60.0475
33365	4.8539	352.968	96.2881
33366	4.854	358.236	315.699
33367	4.854	389.054	125.084
33368	4.854	342.422	169.863
33369	4.854	359.106	82.7461
33370	4.854	372.023	140.224
33371	4.8541	349.18	69.1216
33372	4.8542	300.685	231.332
33373	4.8544	429.773	188.224
33374	4.8544	360.294	130.221
33375	4.8544	367.751	48.3864
33376	4.8548	381.436	277.323
33377	4.8548	350.307	132.41
33378	4.8549	418.621	158.72
33379	4.8549	376.097	74.4267
33380	4.855	344.324	264.356
33381	4.8552	336.581	97.452
33382	4.8552	375.464	50.0829
33383	4.8553	315.1	210.682
33384	4.8553	309.935	132.234
33385	4.8554	370.243	246.833
33386	4.8558	338.803	197.809
33387	4.8558	423.093	41.0356
33388	4.856	348.966	153.805
33389	4.856	405.625	121.619
33390	4.856	334.47	169.055
33391	4.8561	301.521	257.658
33392	4.8561	361.845	63.8863
33393	4.8563	334.701	29.674
33394	4.8564	345.112	283.316
33395	4.8564	285.873	98.2182
33396	4.8565	404.671	90.5605
33397	4.8566	362.525	305.751

33398	4.8567	431.92	31.1821
33399	4.8567	383.305	163.964
33400	4.8568	411.167	305.087
33401	4.8569	336.888	129.341
33402	4.857	306.792	16.7764
33403	4.8571	373.021	257.13
33404	4.8573	274.152	32.3431
33405	4.8573	372.441	179.903
33406	4.8573	334.237	115.765
33407	4.8573	396.279	72.0893
33408	4.8576	363.877	101.364
33409	4.8577	364.537	246.213
33410	4.8577	377.265	76.6941
33411	4.8578	320.695	78.3986
33412	4.8578	331.234	112.331
33413	4.8579	421.288	289.801
33414	4.8579	282.258	120.152
33415	4.858	362.233	101.589
33416	4.8581	373.291	122.937
33417	4.8581	344.246	136.963
33418	4.8582	330.905	231.293
33419	4.8585	327.129	107.05
33420	4.8586	443.106	28.5087
33421	4.8587	414.561	41.3466
33422	4.8587	315.963	195.107
33423	4.8588	352.261	168.265
33424	4.8588	399.425	32.8644
33425	4.8591	384.476	232.112
33426	4.8592	360.351	88.8099
33427	4.8593	473.344	32.1862
33428	4.8593	383.973	77.0524
33429	4.8594	415.627	42.295
33430	4.8594	316.641	179.589
33431	4.8595	319.961	232.895
33432	4.8596	413.683	81.1093
33433	4.8596	287.029	151.562
33434	4.8596	389.645	74.2452
33435	4.8597	386.311	114.032
33436	4.8598	402.991	318.585
33437	4.8598	316.282	94.9659
33438	4.86	433.452	318.15
33439	4.86	368.503	68.4807
33440	4.86	343.73	282.55
33441	4.8602	425.088	79.2873
33442	4.8604	415.704	121.549
33443	4.8604	261.157	240.439
33444	4.8604	367.957	275.372
33445	4.8605	256.25	20.0725

33446	4.8607	334.938	83.0576
33447	4.8607	369.736	51.4675
33448	4.8607	405.52	332.778
33449	4.8608	396.221	317.822
33450	4.8608	410.822	116.169
33451	4.8612	339.975	161.985
33452	4.8612	349.467	93.8683
33453	4.8614	360.408	251.437
33454	4.8614	285.056	258.746
33455	4.8615	325.256	219.945
33456	4.8615	268.628	52.7917
33457	4.8616	395.569	117.916
33458	4.8616	323.816	186.26
33459	4.8617	333.191	237.139
33460	4.8617	265.492	148.842
33461	4.8617	365.575	131.15
33462	4.8618	349.115	219.71
33463	4.8619	392.848	131.287
33464	4.8619	288.444	86.1551
33465	4.8619	444.92	232.001
33466	4.8621	440.354	24.0179
33467	4.8621	344.538	172.807
33468	4.8622	370.965	157.226
33469	4.8624	394.282	70.0787
33470	4.8626	353.33	75.2123
33471	4.8626	408.468	183.293
33472	4.8627	361.643	208.753
33473	4.8627	327.933	87.3613
33474	4.8627	338.426	193.218
33475	4.8627	380.185	121.024
33476	4.863	393.511	93.7896
33477	4.8631	334.676	121.076
33478	4.8632	393.479	77.2313
33479	4.8632	373.267	251.843
33480	4.8632	271.235	182.866
33481	4.8634	332.096	100.642
33482	4.8637	371.401	187.957
33483	4.8638	379.708	97.3751
33484	4.8639	356.382	96.2235
33485	4.8642	387.761	76.6282
33486	4.8644	371.978	185.939
33487	4.8644	319.991	232.41
33488	4.8644	420.498	64.6638
33489	4.8645	387.085	305.048
33490	4.8646	384.557	194.328
33491	4.8646	364.601	157.156
33492	4.8648	353.366	79.18
33493	4.8649	335.065	204.856

33494	4.8649	368.624	129.082
33495	4.8651	364.74	194.862
33496	4.8652	428.668	289.131
33497	4.8656	318.62	52.6972
33498	4.8657	394.348	109.692
33499	4.8657	371.213	119.843
33500	4.8657	330.009	328.76
33501	4.8659	371.799	124.365
33502	4.866	391.593	86.7317
33503	4.866	354.218	295.21
33504	4.866	341.873	178.357
33505	4.8661	393.823	36.7301
33506	4.8661	354.39	82.1469
33507	4.8664	386.819	136.515
33508	4.8667	351.741	124.474
33509	4.8667	346.584	135.86
33510	4.8668	351.426	221.098
33511	4.8668	397.504	46.4808
33512	4.8669	309.326	218.215
33513	4.8671	370.884	292.273
33514	4.8671	360.735	140.507
33515	4.8672	338.863	113.303
33516	4.8673	384.08	184.661
33517	4.8673	300.145	99.271
33518	4.8673	341.081	146.029
33519	4.8677	374.433	223.706
33520	4.8678	370.813	46.3824
33521	4.8678	383.324	110.45
33522	4.8679	396.108	111.208
33523	4.8679	397.951	318.671
33524	4.868	334.268	240.187
33525	4.868	441.825	91.4258
33526	4.8681	376.786	140.244
33527	4.8681	448.323	10.0724
33528	4.8682	384.842	144.481
33529	4.8683	346.348	236.781
33530	4.8683	384.737	278.562
33531	4.8683	360.914	160.82
33532	4.8684	385.708	158.222
33533	4.8685	273.368	42.4076
33534	4.8687	378.679	227.641
33535	4.8692	287.426	220.208
33536	4.8694	350.323	101.505
33537	4.8694	425.774	108.269
33538	4.8694	361.492	78.4796
33539	4.8695	456.778	207.486
33540	4.8696	429.846	16.9066
33541	4.8697	307.659	306.251

33542	4.8697	343.17	99.9497
33543	4.8698	340.923	109.136
33544	4.8698	344.396	340.516
33545	4.87	379.741	43.183
33546	4.87	377.494	192.532
33547	4.8702	390.899	114.972
33548	4.8702	367.741	65.2032
33549	4.8702	395.762	182.645
33550	4.8703	355.981	192.678
33551	4.8704	408.324	224.443
33552	4.8704	353.632	81.467
33553	4.8706	390.697	131.708
33554	4.8708	436.238	38.5002
33555	4.8708	420.325	29.9823
33556	4.871	362.542	249.024
33557	4.871	443.334	117.638
33558	4.8711	394.328	82.2335
33559	4.8714	313.094	276.118
33560	4.8715	245.707	6.1114
33561	4.8716	363.236	100.92
33562	4.8716	283.272	186.692
33563	4.8717	331.458	26.1877
33564	4.8719	479.976	247.329
33565	4.872	346.584	187.73
33566	4.872	343.535	69.6896
33567	4.8721	425.066	41.464
33568	4.8723	306.255	187.188
33569	4.8723	295.835	168.806
33570	4.8724	281.015	196.78
33571	4.8724	319.552	200.62
33572	4.8725	406.365	26.2708
33573	4.8727	363.625	124.261
33574	4.8727	357.277	64.1419
33575	4.8727	369.028	103.612
33576	4.8728	444.405	27.6355
33577	4.873	326.48	281.901
33578	4.873	349.234	121.95
33579	4.8731	375.867	83.9846
33580	4.8732	389.404	139.771
33581	4.8732	387.164	105.309
33582	4.8734	376.869	263.148
33583	4.8735	356.426	203.137
33584	4.8739	322.511	163.957
33585	4.8742	374.622	344.259
33586	4.8743	330.921	135.165
33587	4.8744	327.475	149.078
33588	4.8745	374.62	82.1836
33589	4.8745	245.42	47.3507

33590	4.8746	424.23	26.7128
33591	4.8747	402.975	76.9537
33592	4.8748	311.781	129.75
33593	4.875	362.422	201.861
33594	4.875	352.39	124.185
33595	4.8751	351.906	285.164
33596	4.8752	410.455	216.076
33597	4.8752	301.557	97.1835
33598	4.8753	290.376	163.675
33599	4.8753	299.926	252.289
33600	4.8753	442.932	162.034
33601	4.8754	362.701	135.745
33602	4.8756	300.532	158.243
33603	4.8757	371.449	107.35
33604	4.8758	327.631	82.8048
33605	4.8758	301.867	319.707
33606	4.876	377.202	131.546
33607	4.876	362.236	110.697
33608	4.8763	351.692	157.32
33609	4.8768	317.765	266.085
33610	4.8769	345.394	40.8139
33611	4.877	271.296	30.6182
33612	4.877	359.301	170.746
33613	4.8771	458.622	314.694
33614	4.8772	338.026	164.734
33615	4.8772	312.868	94.0896
33616	4.8773	399.584	173.049
33617	4.8774	414.77	27.2229
33618	4.8774	410.115	38.02
33619	4.8775	325.597	170.054
33620	4.8776	370.728	229.989
33621	4.8776	322.371	47.3098
33622	4.8777	458.37	80.9286
33623	4.8777	345.415	147.071
33624	4.8777	370.585	224.663
33625	4.8779	316.411	158.033
33626	4.878	373.678	84.1283
33627	4.878	405.307	25.5369
33628	4.8782	432.766	121.228
33629	4.8782	398.001	263.138
33630	4.8783	305.125	85.6789
33631	4.8783	323.124	195.638
33632	4.8786	415.738	70.864
33633	4.8786	376.105	107.506
33634	4.8787	285.888	218.215
33635	4.8789	423.868	122.818
33636	4.879	388.138	34.506
33637	4.879	312.791	94.3601

33638	4.879	324.286	87.2933
33639	4.8791	411.893	82.5801
33640	4.8793	340.162	111.145
33641	4.8794	339.062	170.627
33642	4.8795	375.486	57.8504
33643	4.8795	421.457	64.5382
33644	4.8795	304.815	281.92
33645	4.8796	301.599	190.666
33646	4.8797	453.321	67.6161
33647	4.8798	426.548	196.192
33648	4.8798	417.582	73.6498
33649	4.8798	351.578	22.4482
33650	4.8799	440.594	286.102
33651	4.8801	464.523	133.068
33652	4.8801	332.934	204.05
33653	4.8801	371.971	132.61
33654	4.8803	327.606	88.4108
33655	4.8805	410.944	13.9974
33656	4.8808	390.949	95.6968
33657	4.8808	334.027	106.829
33658	4.8809	396.089	97.1761
33659	4.881	373.922	318.125
33660	4.881	353.823	59.9466
33661	4.8812	403.983	183.05
33662	4.8814	393.074	143.927
33663	4.8817	367.594	198.813
33664	4.8817	289.564	130.787
33665	4.8817	441.806	66.9003
33666	4.8818	353.99	284.167
33667	4.8819	341.803	111.674
33668	4.8821	279.875	180.792
33669	4.8822	309.118	227.546
33670	4.8822	327.854	104.046
33671	4.8826	391.886	291.82
33672	4.8826	322.37	220.328
33673	4.8827	334.186	173.874
33674	4.8827	295.502	232.292
33675	4.8827	312.803	94.3946
33676	4.8827	345.177	148.446
33677	4.8828	344.126	326.829
33678	4.8828	379.948	74.0207
33679	4.8829	416.093	75.3462
33680	4.883	416.067	94.061
33681	4.8831	325.128	123.348
33682	4.8832	391.703	105.613
33683	4.8832	362.582	63.0772
33684	4.8833	393.349	40.2371
33685	4.8835	355.629	256.288

33686	4.8835	377.068	75.0478
33687	4.8836	273.847	86.185
33688	4.8837	415.403	132.099
33689	4.8837	288.317	258.397
33690	4.8837	351.315	130.522
33691	4.8837	427.008	185.079
33692	4.8838	378.892	229.881
33693	4.8839	369.491	156.522
33694	4.884	322.713	135.992
33695	4.8842	350.444	131.593
33696	4.8843	324.293	29.5549
33697	4.8843	398.411	51.9759
33698	4.8845	360.251	124.835
33699	4.8847	431.126	51.0521
33700	4.8847	376.203	93.2251
33701	4.8848	311.909	172.626
33702	4.8848	307.923	141.682
33703	4.8849	434.908	73.4059
33704	4.8849	385.524	123.046
33705	4.8852	367.15	164.798
33706	4.8853	212.874	214.661
33707	4.8854	342.338	41.0983
33708	4.8854	341.113	227.637
33709	4.8855	400.188	138.93
33710	4.8855	363.601	249.216
33711	4.8857	437.729	57.8072
33712	4.8857	381.682	182.765
33713	4.8857	360.9	113.76
33714	4.8858	425.494	192.159
33715	4.8858	376.479	84.1761
33716	4.886	373.527	327.141
33717	4.8861	363.847	252.185
33718	4.8864	344.46	117.422
33719	4.8865	344.037	103.463
33720	4.8866	385.383	24.052
33721	4.8866	329.011	221.249
33722	4.8868	311.997	281.42
33723	4.887	411.639	62.2771
33724	4.887	355.187	184.882
33725	4.8871	478.77	69.3639
33726	4.8872	312.087	218.321
33727	4.8872	421.845	26.6927
33728	4.8872	319.839	152.966
33729	4.8873	387.481	139.551
33730	4.8873	358.253	137.024
33731	4.8874	386.745	86.4112
33732	4.8874	302.782	128.436
33733	4.8875	-2.7199	14.2134

33734	4.8877	401.359	222.292
33735	4.8878	411.746	239.771
33736	4.8879	337.106	287.522
33737	4.8879	389.949	62.7413
33738	4.8879	399.87	114.149
33739	4.888	315.782	143.294
33740	4.888	339.745	355.141
33741	4.8882	380.184	98.2152
33742	4.8883	313.232	132.373
33743	4.8884	423.861	178.884
33744	4.8885	387.273	112.269
33745	4.8885	378.853	43.7826
33746	4.8885	327.342	32.5041
33747	4.8886	359.498	118.807
33748	4.8887	431.624	82.4619
33749	4.8887	345.881	342.915
33750	4.8887	372.393	90.9202
33751	4.8888	404.137	180.258
33752	4.8889	333.968	93.1441
33753	4.8891	330.104	78.245
33754	4.8893	386.988	146.677
33755	4.8893	336.931	51.9643
33756	4.8894	364.47	35.8371
33757	4.8894	389.123	122.734
33758	4.8895	428.399	25.5363
33759	4.8895	341.901	89.9681
33760	4.8901	295.815	243.05
33761	4.8901	361.879	115.826
33762	4.8902	376.776	188.488
33763	4.8902	362.025	118.442
33764	4.8902	421.575	245.039
33765	4.8903	396.8	260.775
33766	4.8904	352.866	128.948
33767	4.8904	303.143	111.575
33768	4.8906	325.27	224.713
33769	4.8907	447.166	42.1237
33770	4.8907	370.971	226.801
33771	4.8908	367.795	98.8152
33772	4.891	338.614	235.35
33773	4.891	318.686	317.113
33774	4.8911	417.778	56.7159
33775	4.8912	452.106	122.751
33776	4.8914	402.637	243.707
33777	4.8914	415.975	129.821
33778	4.8915	421.156	72.6635
33779	4.8916	418.253	246.698
33780	4.8916	345.755	121.993
33781	4.8917	433.612	225.187

33782	4.8918	387.292	120.094
33783	4.8918	412.9	188.684
33784	4.892	437.375	24.255
33785	4.8921	302.614	133.38
33786	4.8922	422.637	117.528
33787	4.8923	380.031	111.845
33788	4.8924	456.403	9.6021
33789	4.8926	378.011	92.1354
33790	4.8926	332.55	142.038
33791	4.8928	285.774	173.324
33792	4.8929	392.773	123.611
33793	4.893	390.745	111.624
33794	4.8934	393.434	267.614
33795	4.8934	357.882	92.3194
33796	4.8935	362.838	116.369
33797	4.8936	369.649	131.592
33798	4.8937	399.785	71.9403
33799	4.8937	324.549	153.655
33800	4.8937	406.849	47.4373
33801	4.8938	382.233	62.1657
33802	4.8938	384.959	223.314
33803	4.8939	283.27	278.486
33804	4.8939	423.416	307.499
33805	4.8941	447.55	99.5956
33806	4.8942	423.199	158.794
33807	4.8942	360.421	71.2003
33808	4.8943	443.236	87.9726
33809	4.8943	432.357	105.657
33810	4.8943	328.407	88.8782
33811	4.8945	377.817	283.337
33812	4.8945	496.716	286.766
33813	4.8947	343.557	54.9407
33814	4.8949	301.867	168.349
33815	4.8949	342.644	90.9991
33816	4.8949	392.334	67.496
33817	4.8952	382.798	248.822
33818	4.8952	348.798	69.0134
33819	4.8952	322.621	69.7869
33820	4.8954	397.999	78.7502
33821	4.8955	312.002	179.435
33822	4.8955	363.634	116.27
33823	4.8955	342.853	32.8719
33824	4.8957	475.397	269.556
33825	4.8957	398.138	105.763
33826	4.8958	431.886	231.823
33827	4.8958	377.654	168.513
33828	4.8959	386.607	38.1029
33829	4.896	347.271	196.157

33830	4.8961	430.746	120.509
33831	4.8962	325.598	218.081
33832	4.8962	407.431	70.0269
33833	4.8963	438.433	203.165
33834	4.8964	410.434	111.524
33835	4.8965	421.972	66.895
33836	4.8968	411.494	332.984
33837	4.897	411.373	5.326
33838	4.8973	368.581	60.2378
33839	4.8975	389.331	115.27
33840	4.8977	316.87	175.047
33841	4.8979	433.236	50.2155
33842	4.8979	348.062	235.315
33843	4.8981	340.806	172.567
33844	4.8982	416.319	122.217
33845	4.8982	383.12	245.052
33846	4.8982	409.299	79.6229
33847	4.8983	341.831	165.469
33848	4.8983	320.38	190.971
33849	4.8984	435.803	99.3647
33850	4.8984	359.094	88.5213
33851	4.8984	413.083	56.581
33852	4.8985	414.108	58.2262
33853	4.8987	397.795	79.8581
33854	4.8988	363.671	123.47
33855	4.8989	348.312	110.268
33856	4.8992	382.487	221.221
33857	4.8992	407.065	185.623
33858	4.8993	337.605	137.569
33859	4.8993	150.671	182.309
33860	4.8993	360.726	128.603
33861	4.8993	407.271	225.469
33862	4.8993	397.675	149.827
33863	4.8995	327.884	162.637
33864	4.8995	327.474	117.771
33865	4.8996	412.985	179.718
33866	4.8996	370.988	40.4041
33867	4.8996	404.227	52.4145
33868	4.8998	303.419	191.458
33869	4.8999	377.63	214.449
33870	4.8999	362.733	312.133
33871	4.9001	469.976	24.7447
33872	4.9002	345.401	76.8036
33873	4.9003	326.759	95.5961
33874	4.9004	372.756	76.8403
33875	4.9004	393.063	150.378
33876	4.9004	371.345	82.2723
33877	4.9005	414.176	60.3039

33878	4.9007	103.313	62.6719
33879	4.9008	384.463	124.377
33880	4.9009	363.737	250.227
33881	4.9009	378.715	278.365
33882	4.901	329.589	109.788
33883	4.901	427.929	31.2124
33884	4.9012	376.114	316.299
33885	4.9012	432.488	11.8005
33886	4.9014	418.488	73.6585
33887	4.9015	345.117	178.051
33888	4.9016	289.215	182.126
33889	4.9017	387.46	77.9637
33890	4.9019	403.237	26.9897
33891	4.902	327.801	179.661
33892	4.9022	424.871	308.419
33893	4.9024	397.867	149.298
33894	4.9025	365.936	41.8149
33895	4.9027	311.444	265.028
33896	4.9028	401.712	69.4076
33897	4.9028	328.778	125.049
33898	4.9028	327.518	131.92
33899	4.903	356.081	171.489
33900	4.903	379.154	34.4139
33901	4.9031	403.467	104.754
33902	4.9031	413.515	75.6173
33903	4.9032	420.231	109.439
33904	4.9033	293.031	171.56
33905	4.9034	347.125	103.982
33906	4.9035	446.898	207.344
33907	4.9036	403.895	107.395
33908	4.9037	407.381	81.8258
33909	4.9039	388.761	86.1235
33910	4.9039	-4.0177	243.077
33911	4.904	391.998	235.846
33912	4.904	305.745	130.654
33913	4.904	273.432	144.826
33914	4.9041	357.558	47.9937
33915	4.9041	405.745	275.51
33916	4.9042	366.643	130.342
33917	4.9043	443.404	92.4316
33918	4.9043	338.836	42.2299
33919	4.9045	359.173	154.116
33920	4.9045	375.144	105.12
33921	4.9047	423.549	235.535
33922	4.9048	302.398	178.238
33923	4.9048	408.97	47.7221
33924	4.905	373.491	113.724
33925	4.905	373.159	32.5347

33926	4.905	419.867	73.2915
33927	4.905	346.477	171.988
33928	4.9051	363.051	121.992
33929	4.9052	400.939	76.4943
33930	4.9055	349.862	188.773
33931	4.9055	396.269	240.425
33932	4.9057	330.113	185.116
33933	4.9057	321.505	125.55
33934	4.9058	412.436	282.052
33935	4.9059	367.793	233.95
33936	4.906	393.502	117.282
33937	4.906	366.914	141.189
33938	4.906	322.745	321.8
33939	4.9062	403.002	254.137
33940	4.9064	346.211	143.557
33941	4.9064	330.938	120.309
33942	4.9065	345.074	296.915
33943	4.9066	379.278	117.986
33944	4.9066	416.526	285.496
33945	4.9068	404.298	359.564
33946	4.9069	423.15	118.686
33947	4.907	369.066	241.806
33948	4.907	394.343	184.032
33949	4.9072	376.46	67.9455
33950	4.9072	440.193	80.056
33951	4.9073	347.903	234.81
33952	4.9073	318.541	139.252
33953	4.9074	367.714	208.013
33954	4.9076	382.105	107.022
33955	4.9078	359.685	95.9565
33956	4.9079	401.389	191.124
33957	4.9081	409.073	89.9323
33958	4.9081	363.743	69.7566
33959	4.9082	396.268	6.6592
33960	4.9082	365.687	60.1722
33961	4.9084	396.065	302.322
33962	4.9084	398.106	133.704
33963	4.9085	388.401	57.7712
33964	4.9085	391.999	75.0175
33965	4.9086	391.588	137.846
33966	4.9086	293.664	161.47
33967	4.9087	352.462	157.653
33968	4.9088	394.611	121.955
33969	4.9088	453.6	207.754
33970	4.9088	388.609	301.128
33971	4.909	339.583	25.773
33972	4.909	386.645	79.9397
33973	4.909	367.678	110.98

33974	4.9091	350.747	156.07
33975	4.9091	357.721	97.3272
33976	4.9091	402.132	119.403
33977	4.9092	350.512	122.035
33978	4.9093	337.798	115.766
33979	4.9093	476.944	39.1482
33980	4.9094	398.716	161.622
33981	4.9095	443.812	84.135
33982	4.9095	391.104	104.903
33983	4.9096	252.066	161.291
33984	4.91	413.955	138.538
33985	4.91	457.551	50.1726
33986	4.9101	343.126	125.456
33987	4.9101	282.279	94.6528
33988	4.9103	461.955	81.7787
33989	4.9105	371.456	143.515
33990	4.9106	322.283	189.411
33991	4.9107	402.225	119.838
33992	4.9107	320.868	109.154
33993	4.9108	365.497	120.978
33994	4.9109	394.565	163.116
33995	4.9109	412.668	62.3953
33996	4.9111	388.766	109.517
33997	4.9112	400.961	230.786
33998	4.9115	439.858	53.4931
33999	4.9116	362.665	222.459
34000	4.9118	351.74	304.203
34001	4.9118	381.788	204.332
34002	4.9118	306.803	84.3695
34003	4.9119	353.416	126.465
34004	4.912	463.415	38.2796
34005	4.912	355.186	323.851
34006	4.9121	328.044	183.987
34007	4.9122	440.454	33.7344
34008	4.9122	385.156	129.119
34009	4.9125	367.65	109.996
34010	4.9126	438.032	297.678
34011	4.9127	317.758	156.081
34012	4.9127	311.94	77.7441
34013	4.9127	399.969	321.733
34014	4.9127	470.299	71.1746
34015	4.9128	387.577	135.305
34016	4.9128	322.736	201.908
34017	4.9128	394	125.37
34018	4.9131	492.166	62.6634
34019	4.9132	435.032	171.799
34020	4.9133	410.328	84.3779
34021	4.9133	300.068	177.232

34022	4.9134	399.73	55.5462
34023	4.9135	458.592	58.2031
34024	4.9135	353.295	246.362
34025	4.9135	408.683	18.8203
34026	4.9136	430.502	288.699
34027	4.9137	331.661	154.051
34028	4.9137	359.858	133.211
34029	4.9138	404.587	121.121
34030	4.9138	427.01	290.238
34031	4.914	415.057	29.0337
34032	4.914	344.577	76.8217
34033	4.9141	445.421	90.8878
34034	4.9143	337.072	112.894
34035	4.9143	383.3	187.781
34036	4.9144	383.475	269.629
34037	4.9145	377.168	172.009
34038	4.9146	339.807	154.387
34039	4.9149	389.166	112.494
34040	4.9149	431.434	153.117
34041	4.9151	414.011	56.3687
34042	4.9152	322.521	222.885
34043	4.9152	366.194	331.738
34044	4.9157	405.235	45.5636
34045	4.9158	382.202	329.113
34046	4.9158	501.69	313.482
34047	4.9159	369.938	122.192
34048	4.9159	385.139	80.6977
34049	4.916	395.927	47.7271
34050	4.916	412.749	79.5884
34051	4.9161	389.777	80.8407
34052	4.9162	409.601	300.443
34053	4.9163	369.089	31.9197
34054	4.9164	411.83	45.0938
34055	4.9164	329.376	176.535
34056	4.9167	386.666	191.963
34057	4.9167	354.411	341.326
34058	4.9168	397.642	275.945
34059	4.917	370.542	93.4784
34060	4.9171	333.101	229.569
34061	4.9171	395.267	68.2008
34062	4.9172	341.758	149.337
34063	4.9172	253.355	72.3092
34064	4.9172	377.273	159.675
34065	4.9172	378.679	335.406
34066	4.9174	374.453	130.867
34067	4.9175	356.705	194.494
34068	4.9176	389.609	132.447
34069	4.9179	395.239	122.096

34070	4.918	452.031	302.746
34071	4.9181	318.704	183.706
34072	4.9182	374.428	335.451
34073	4.9184	347.325	114.893
34074	4.9184	394.602	158.441
34075	4.9186	425.002	81.5471
34076	4.9186	433.587	290.545
34077	4.9188	408.186	150.374
34078	4.919	424.339	12.6046
34079	4.9191	316.14	237.687
34080	4.9191	285.619	179.543
34081	4.9192	420.561	35.0245
34082	4.9195	441.5	212.177
34083	4.9197	492.33	290.147
34084	4.9197	379.926	134.443
34085	4.9199	383.823	166.1
34086	4.9199	355.947	166.925
34087	4.9199	373.248	111.336
34088	4.92	315.516	145.011
34089	4.9202	361.315	155.266
34090	4.9203	411.245	121.097
34091	4.9203	360.892	156.669
34092	4.9205	434.969	229.625
34093	4.9205	353.651	40.7596
34094	4.9209	435.815	187.54
34095	4.9209	333.469	35.8691
34096	4.9209	405.798	74.3871
34097	4.921	456.44	44.4479
34098	4.9211	362.175	121.629
34099	4.9211	308.849	207.044
34100	4.9214	318.216	127.608
34101	4.9216	389.406	135.657
34102	4.9218	379.103	120.888
34103	4.922	348.319	138.861
34104	4.9221	388.566	123.066
34105	4.9221	359.637	164.975
34106	4.9223	406.707	236.301
34107	4.9223	309.661	160.039
34108	4.9224	409.805	202.141
34109	4.9226	436.385	70.3265
34110	4.9226	310.138	138.086
34111	4.9229	438.087	230.516
34112	4.9231	412.306	161.865
34113	4.9231	356.547	353.082
34114	4.9233	242.728	42.4709
34115	4.9233	339.859	60.7851
34116	4.9234	411.702	337.09
34117	4.9235	345.124	87.0296

34118	4.9235	358.698	119.924
34119	4.9238	441.932	349.215
34120	4.9239	383.66	66.987
34121	4.9241	409.963	86.9344
34122	4.9241	313.927	209.984
34123	4.9241	434.344	54.8711
34124	4.9241	358.614	150.076
34125	4.9243	402.208	126.307
34126	4.9245	425.675	69.9672
34127	4.9245	339.898	257.357
34128	4.9245	379.622	141.242
34129	4.9246	374.727	138.207
34130	4.9246	380.728	122.973
34131	4.9246	410.741	124.345
34132	4.9247	247.637	322.28
34133	4.9249	389.986	114.804
34134	4.9249	373.873	260.409
34135	4.925	340.098	338.991
34136	4.9251	337.419	101.547
34137	4.9254	402.588	88.4074
34138	4.9255	363.993	257.477
34139	4.9256	428.134	25.0104
34140	4.9257	419.358	288.345
34141	4.9257	286.23	233.249
34142	4.9257	349.849	97.7193
34143	4.9258	394.2	97.5068
34144	4.926	343.533	10.8086
34145	4.926	376.203	117.815
34146	4.926	370.86	108.867
34147	4.926	338.429	66.6696
34148	4.9261	407.575	89.456
34149	4.9263	327.279	237.427
34150	4.9263	313.87	96.0141
34151	4.9264	406.057	188.03
34152	4.9268	319.673	140.907
34153	4.9269	460.923	46.9407
34154	4.927	319.574	170.872
34155	4.9271	384.866	197.419
34156	4.9272	385.067	270.378
34157	4.9275	369.413	160.773
34158	4.9276	430.658	100.915
34159	4.9277	463.54	22.6403
34160	4.9278	300.42	142.265
34161	4.9279	433.547	256.028
34162	4.928	447.371	213.066
34163	4.9281	417.585	16.429
34164	4.9281	449.168	66.4158
34165	4.9282	357.586	108.802

34166	4.9283	433.653	104.323
34167	4.9283	385.444	138.322
34168	4.9283	367.493	171.157
34169	4.9286	466.136	44.2442
34170	4.9286	390.054	280
34171	4.9286	321.883	75.8241
34172	4.9288	346.387	174.728
34173	4.9289	347.334	7.7801
34174	4.9289	433.485	298.92
34175	4.9291	423.456	194.177
34176	4.9291	313.223	116.792
34177	4.9292	345.086	198.34
34178	4.9295	439.507	173.091
34179	4.9296	337.657	162.419
34180	4.9296	380.088	119.209
34181	4.9297	404.572	191.983
34182	4.9297	371.979	232.183
34183	4.9297	403.252	119.857
34184	4.9299	450.07	18.0697
34185	4.93	358.714	295.227
34186	4.9301	379.055	91.2003
34187	4.9301	426.762	81.227
34188	4.9303	429.536	85.6284
34189	4.9303	462.771	8.4107
34190	4.9304	427.256	71.5152
34191	4.9305	380.295	73.8189
34192	4.9306	374.829	93.644
34193	4.9306	408.207	201.525
34194	4.9307	443.485	63.3937
34195	4.9307	346.035	97.673
34196	4.9308	408.849	114.441
34197	4.9309	439.976	287.449
34198	4.931	353.231	276.25
34199	4.931	482.905	207.102
34200	4.9313	327.098	176.096
34201	4.9313	440.444	267.252
34202	4.9314	322.541	176.695
34203	4.9317	293.028	357.005
34204	4.9317	352.507	62.4285
34205	4.9318	447.815	110.754
34206	4.9318	397.804	106.508
34207	4.9319	334.847	276.654
34208	4.9319	469.346	284.044
34209	4.932	402.759	54.2664
34210	4.9321	393.144	160.817
34211	4.9321	319.207	201.795
34212	4.9321	354.834	95.4488
34213	4.9323	336.178	12.4455

34214	4.9323	400.633	41.3086
34215	4.9324	312.327	124.924
34216	4.9325	430.483	59.9261
34217	4.9325	403.664	272.245
34218	4.9325	279.475	302.432
34219	4.9326	435.291	75.0906
34220	4.9327	454.144	302.069
34221	4.9327	401.459	188.034
34222	4.9327	436.358	53.7693
34223	4.9328	413.892	339.412
34224	4.9331	175.852	100.177
34225	4.9332	163.571	34.8951
34226	4.9332	364.258	128.374
34227	4.9332	325.97	138.507
34228	4.9332	320.598	157.438
34229	4.9334	393.598	85.1212
34230	4.9334	484.934	33.6964
34231	4.9336	392.99	193.191
34232	4.9336	377.858	72.9639
34233	4.9337	-8.6537	66.4892
34234	4.9339	428.653	49.0454
34235	4.934	385.619	129.999
34236	4.9341	439.268	317.25
34237	4.9342	441.062	64.2424
34238	4.9342	379.967	247.029
34239	4.9342	430.732	27.0589
34240	4.9344	420.782	189.825
34241	4.9344	340.017	246.146
34242	4.9345	329.725	92.9336
34243	4.9345	379.798	332.29
34244	4.9346	361.687	212.099
34245	4.9347	319.642	182.573
34246	4.9348	516.092	269.656
34247	4.9348	379.455	108.019
34248	4.9348	385.619	186.848
34249	4.9349	391.579	130.661
34250	4.9349	271.47	280.95
34251	4.935	340.618	149.706
34252	4.935	375.996	216.759
34253	4.935	415.754	38.9522
34254	4.9352	328.409	39.0412
34255	4.9352	446.5	77.8442
34256	4.9352	353.561	142.575
34257	4.9353	427.837	119.5
34258	4.9353	337.969	216.559
34259	4.9353	335.037	140.353
34260	4.9353	368.34	19.2485
34261	4.9353	325.962	125.515

34262	4.9354	377.113	141.092
34263	4.9357	402.077	44.2907
34264	4.9359	358.911	94.7968
34265	4.936	442.005	85.4127
34266	4.936	353.644	340.801
34267	4.9362	408.22	160.949
34268	4.9365	432.428	36.2157
34269	4.9365	367.638	83.1087
34270	4.9368	332.494	199.653
34271	4.9368	309.257	183.377
34272	4.9369	384.936	196.446
34273	4.9369	462.864	96.0994
34274	4.9369	429.233	303.533
34275	4.937	423.457	128.695
34276	4.937	439.976	60.1816
34277	4.937	428.358	72.2798
34278	4.9372	405.964	321.685
34279	4.9373	399.704	228.459
34280	4.9373	319.344	13.7591
34281	4.9374	357.165	84.2399
34282	4.9374	381.837	102.363
34283	4.9376	399.79	341.425
34284	4.9376	403.98	231.418
34285	4.9377	368.474	184.769
34286	4.9377	321.752	102.527
34287	4.9379	365.039	70.9345
34288	4.938	432.588	65.4186
34289	4.938	373.602	60.8195
34290	4.9381	419.931	130.2
34291	4.9382	442.088	28.2414
34292	4.9382	428.308	71.7307
34293	4.9383	352.187	234.262
34294	4.9384	351.577	142.147
34295	4.9385	307.209	316.192
34296	4.9385	443.598	59.0718
34297	4.9385	420.589	272.972
34298	4.9388	350.67	229.611
34299	4.9388	395.327	51.5306
34300	4.939	342.869	148.649
34301	4.9391	306.826	181.375
34302	4.9392	490.35	33.634
34303	4.9394	424.956	177.837
34304	4.9394	435.438	174.245
34305	4.9395	453.622	207.158
34306	4.9395	311.682	236.805
34307	4.9396	364.814	119.999
34308	4.9398	345.735	121.416
34309	4.9401	385.036	320.107

34310	4.9403	480.385	213.482
34311	4.9403	405.45	28.8191
34312	4.9403	451.589	84.8493
34313	4.9405	425.97	298.308
34314	4.9406	361.256	129.245
34315	4.9406	355.546	164.65
34316	4.9406	337.311	156.243
34317	4.9407	411.483	192.335
34318	4.9407	364.059	68.484
34319	4.9409	375.846	129.911
34320	4.941	445.09	199.359
34321	4.9411	341.737	263.117
34322	4.9411	361.813	184.386
34323	4.9412	430.634	270.479
34324	4.9414	393.228	7.3227
34325	4.9414	327.157	173.89
34326	4.9414	410.493	28.3549
34327	4.9414	358.366	138.724
34328	4.9415	391.929	30.2358
34329	4.9417	428.802	255.745
34330	4.9417	409.53	45.5283
34331	4.9418	407.001	217.75
34332	4.9419	444.467	358.643
34333	4.942	399.509	177.655
34334	4.9423	379.669	335.093
34335	4.9424	320.765	217.559
34336	4.9425	399.601	62.799
34337	4.9426	383.007	134.411
34338	4.9427	329.43	179.381
34339	4.9428	414.235	112.289
34340	4.9428	366.971	148.58
34341	4.9428	368.67	127.71
34342	4.9429	351.103	197.684
34343	4.9431	383.837	149.862
34344	4.9431	407.177	138.05
34345	4.9431	320.28	233.287
34346	4.9432	349.89	125.321
34347	4.9433	318.528	119.235
34348	4.9434	456.969	49.0065
34349	4.9435	363.319	193.358
34350	4.9435	407.847	121.625
34351	4.9437	72.1941	55.9775
34352	4.9438	302.401	227.048
34353	4.9438	494.729	208.626
34354	4.9441	353.707	100.366
34355	4.9442	378.951	118.678
34356	4.9447	302.66	158.127
34357	4.9447	402.236	231.247

34358	4.9449	412.862	59.812
34359	4.9449	378.313	254.531
34360	4.9449	365.091	65.7434
34361	4.9449	351.279	257.442
34362	4.9449	407.124	64.6113
34363	4.9449	423.721	325.405
34364	4.945	448.726	348.564
34365	4.9451	337.934	162.347
34366	4.9452	415.053	140.035
34367	4.9454	417.285	39.1677
34368	4.9455	461.238	113.299
34369	4.9457	330.889	179.328
34370	4.9458	377.741	105.374
34371	4.9459	423.196	301.436
34372	4.946	297.507	233.444
34373	4.9461	331.339	88.7586
34374	4.9463	390.996	82.6124
34375	4.9463	394.988	74.8387
34376	4.9463	382.501	139.818
34377	4.9463	372.441	174.804
34378	4.9464	315.679	190.441
34379	4.9464	445.792	32.7718
34380	4.9465	418.083	105.083
34381	4.9465	324.758	180.247
34382	4.9465	415.274	122.953
34383	4.9465	445.855	40.1078
34384	4.9468	288.952	84.5504
34385	4.9468	385.485	88.9921
34386	4.9469	426.433	240.22
34387	4.947	333.628	192.505
34388	4.947	352.258	123.222
34389	4.947	351.1	104.31
34390	4.9471	398.722	102.594
34391	4.9471	477.201	36.6596
34392	4.9472	355.292	227.764
34393	4.9472	324.239	68.0147
34394	4.9472	400.71	124.68
34395	4.9473	320.414	142.441
34396	4.9474	354.76	191.39
34397	4.9474	-2.7758	9.3007
34398	4.9475	383.5	118.657
34399	4.9476	304.815	187.529
34400	4.9481	363.916	227.716
34401	4.9482	346.372	122.564
34402	4.9482	411.363	78.8032
34403	4.9483	316.357	182.808
34404	4.9483	388.004	40.9437
34405	4.9485	378.702	302.032

34406	4.9486	412.707	187.085
34407	4.9486	316.413	117.133
34408	4.9487	417.11	95.0818
34409	4.9488	451.397	63.0144
34410	4.9488	347.224	170.796
34411	4.9489	343.899	81.5543
34412	4.9489	462.166	81.5632
34413	4.949	343.395	53.3575
34414	4.949	416.783	147.108
34415	4.9491	464.202	281.264
34416	4.9491	386.591	36.431
34417	4.9492	345.379	61.3285
34418	4.9494	430.245	124.853
34419	4.9495	432.381	171.389
34420	4.9496	383.26	121.056
34421	4.9499	449.773	102.822
34422	4.9499	406.231	122.823
34423	4.9499	356.873	174.526
34424	4.9499	336.576	195.891
34425	4.9499	408.604	335.505
34426	4.9499	406.281	337.862
34427	4.95	440.715	73.0011
34428	4.95	337.066	157.601
34429	4.9501	297.701	66.2646
34430	4.9501	352.34	303.924
34431	4.9504	336.94	150.993
34432	4.9504	401.977	101.051
34433	4.9506	386.877	199.468
34434	4.9507	438.271	206.065
34435	4.9508	447.321	102.245
34436	4.9509	420.024	150.758
34437	4.951	356.575	181.389
34438	4.951	273.104	184.89
34439	4.9511	343.113	183.18
34440	4.9511	334.306	191.493
34441	4.9512	451.776	259.517
34442	4.9512	284.19	125.328
34443	4.9512	436.508	81.7461
34444	4.9513	383.784	138.682
34445	4.9513	395.47	351.562
34446	4.9514	436.807	34.8732
34447	4.9514	308.828	190.061
34448	4.9514	362.446	164.119
34449	4.9514	362.379	142.467
34450	4.9515	387.308	230.564
34451	4.9515	338.686	92.8485
34452	4.9519	430.385	89.7281
34453	4.952	408.359	78.1091

34454	4.9525	434.137	239.764
34455	4.9526	357.513	220.246
34456	4.9528	402.16	331.829
34457	4.9528	414.69	82.5233
34458	4.9529	325.94	177.782
34459	4.9529	385.934	137.604
34460	4.9531	405.579	101.528
34461	4.9532	419.853	115.934
34462	4.9533	407.563	113.324
34463	4.9534	308.154	148.631
34464	4.9534	323.383	217.699
34465	4.9536	279.209	168.756
34466	4.9537	338.959	84.5112
34467	4.9538	413.956	155.558
34468	4.954	398.888	44.145
34469	4.9541	299.973	163.261
34470	4.9542	390.431	62.8628
34471	4.9543	392.246	114.371
34472	4.9544	368.07	107.5
34473	4.9544	464.071	36.9215
34474	4.9545	406.697	77.7658
34475	4.9546	352.1	54.8084
34476	4.9546	364.556	191.598
34477	4.9547	375.152	258.914
34478	4.9547	351.312	213.9
34479	4.9548	341.865	28.5318
34480	4.9549	399.588	122.811
34481	4.955	373.633	115.212
34482	4.9554	406.581	24.9868
34483	4.9554	489.217	19.7254
34484	4.9555	433.177	85.4736
34485	4.9555	350.037	105.617
34486	4.9556	428.756	100.845
34487	4.9556	406.35	188.625
34488	4.9557	459.824	83.9773
34489	4.9557	343.224	114.374
34490	4.9557	415.041	13.2625
34491	4.9557	451.874	236.201
34492	4.9558	371.32	134.441
34493	4.9558	-2.7922	12.1384
34494	4.9559	404.434	272.133
34495	4.956	347.683	35.0284
34496	4.9561	419.538	241.189
34497	4.9562	397.039	352.752
34498	4.9564	390.873	102.431
34499	4.9565	370.881	326.973
34500	4.9566	366.816	187.443
34501	4.9566	381.463	138.747

34502	4.9569	388.105	137.913
34503	4.957	487.623	119.688
34504	4.957	342.293	122.058
34505	4.9571	394.367	45.8774
34506	4.9572	318.73	165.248
34507	4.9573	391.192	69.9851
34508	4.9573	427.267	319.969
34509	4.9573	431.99	21.6916
34510	4.9574	480.777	9.993
34511	4.9574	313.364	150.884
34512	4.9576	317.5	50.4247
34513	4.9576	324.508	146.011
34514	4.9576	454.028	271.898
34515	4.9577	413.199	110.669
34516	4.958	359.196	108.355
34517	4.958	456.837	332.367
34518	4.9581	369.413	162.871
34519	4.9582	354.41	141.981
34520	4.9582	419.658	59.1964
34521	4.9582	367.497	122.087
34522	4.9583	429.39	131.44
34523	4.9584	394.442	47.1587
34524	4.9584	427.799	292.395
34525	4.9584	463.348	34.8443
34526	4.9584	409.954	30.3859
34527	4.9584	388.468	134.358
34528	4.9589	442.774	120.429
34529	4.959	361.55	42.1564
34530	4.9591	440.964	22.1444
34531	4.9591	405.619	274.87
34532	4.9591	271.838	56.8308
34533	4.9591	412.983	101.287
34534	4.9591	399.011	172.373
34535	4.9592	492.937	318.036
34536	4.9592	374.135	177.981
34537	4.9593	424.489	278.292
34538	4.9593	413.686	81.4952
34539	4.9594	530.888	67.6788
34540	4.9594	376.386	44.9241
34541	4.9594	348.768	109.163
34542	4.9595	363.762	203.172
34543	4.9598	463.142	289.133
34544	4.9599	379.787	169.911
34545	4.96	370.245	118.244
34546	4.96	395.679	317.326
34547	4.9602	350.853	166.632
34548	4.9602	367.081	164.867
34549	4.9602	355.032	111.064

34550	4.9602	418.102	328.775
34551	4.9604	413.976	74.3611
34552	4.9607	273.288	16.4252
34553	4.9607	460.019	353.667
34554	4.9609	342.855	231.907
34555	4.9609	483.823	306.967
34556	4.9612	384.307	129.697
34557	4.9612	465.966	47.4906
34558	4.9612	444.195	185.415
34559	4.9612	435.515	94.8155
34560	4.9613	368.818	224.508
34561	4.9613	318.086	181.944
34562	4.9615	346.694	196.589
34563	4.9618	461.814	90.271
34564	4.9619	330.663	79.8485
34565	4.962	466.949	86.1759
34566	4.962	341.219	76.2505
34567	4.962	448.342	55.0301
34568	4.9621	467.975	24.6485
34569	4.9621	428.046	236.759
34570	4.9624	363.367	171.552
34571	4.9625	353.396	119.953
34572	4.9626	385.446	291.122
34573	4.9626	370.866	106.415
34574	4.9626	269.203	169.128
34575	4.9627	448.944	39.4697
34576	4.9627	438.906	330.822
34577	4.9629	320.711	50.5203
34578	4.963	311.135	134.105
34579	4.963	329.917	133.141
34580	4.9631	422.207	163.907
34581	4.9634	406.737	308.247
34582	4.9634	394.278	96.3937
34583	4.9634	412.334	107.624
34584	4.9635	441.067	189.275
34585	4.9635	371.229	293.22
34586	4.9637	356.833	42.3263
34587	4.9638	399.374	136.252
34588	4.964	368.373	161.067
34589	4.9645	272.457	153.51
34590	4.9645	333.556	131.865
34591	4.9646	449.117	71.6785
34592	4.9648	286.778	160.396
34593	4.9649	432.337	81.2077
34594	4.9649	416.273	187.702
34595	4.9653	405.019	65.0827
34596	4.9653	355.835	321.815
34597	4.9653	401.337	83.888

34598	4.9653	374.828	17.8586
34599	4.9654	426.301	53.6449
34600	4.9654	289.955	215.63
34601	4.9656	345.506	87.1666
34602	4.9657	387.861	67.8153
34603	4.9657	359.699	78.6916
34604	4.9661	443.699	265.601
34605	4.9661	444.373	40.7795
34606	4.9661	383.713	122.605
34607	4.9662	431.326	216.688
34608	4.9662	421.148	100.816
34609	4.9662	376.817	359.284
34610	4.9662	358.932	155.843
34611	4.9664	414.93	72.7155
34612	4.9664	437.811	311.915
34613	4.9664	409.71	271.115
34614	4.9665	349.001	299.683
34615	4.9665	430.129	141.2
34616	4.9665	398.876	36.5098
34617	4.9666	364.296	192.635
34618	4.9666	330.145	133.216
34619	4.9667	342.691	122.894
34620	4.9668	406.383	186.887
34621	4.9671	356.259	254.269
34622	4.9671	338.766	184.71
34623	4.9671	383.879	284.783
34624	4.9671	453.631	125.062
34625	4.9672	380.529	116.076
34626	4.9672	394.562	201.765
34627	4.9674	350.1	195.045
34628	4.9674	404.252	74.3129
34629	4.9676	404.832	168.939
34630	4.9676	317.269	157.654
34631	4.9677	252.111	223.329
34632	4.9678	394.379	177.302
34633	4.9681	373.963	274.908
34634	4.9682	302.42	129.626
34635	4.9683	473.925	303.565
34636	4.9683	403.468	127.221
34637	4.9683	393.942	72.2267
34638	4.9683	404.146	136.606
34639	4.9684	401.208	220.985
34640	4.9685	335.4	136.152
34641	4.9686	432.612	88.0756
34642	4.9688	394.589	129.044
34643	4.9688	396.557	89.6536
34644	4.9688	408.228	141.355
34645	4.9689	531.201	29.5381

34646	4.9691	376.38	193.286
34647	4.9691	456.316	117.608
34648	4.9692	371.331	93.5537
34649	4.9693	453.236	56.0389
34650	4.9693	469.66	18.2116
34651	4.9694	456.572	326.543
34652	4.9694	346.761	199.635
34653	4.9694	466.579	65.7979
34654	4.9696	394.997	109.027
34655	4.9697	316.535	182.517
34656	4.9697	355.002	19.0314
34657	4.9701	354.964	131.701
34658	4.9703	455.839	65.5008
34659	4.9703	498.855	24.0368
34660	4.9703	468.562	26.2509
34661	4.9704	314.077	84.4767
34662	4.9704	415.236	33.3253
34663	4.9705	447.964	47.4086
34664	4.9707	380.47	1.5357
34665	4.9708	507.588	29.1287
34666	4.9708	407.82	118.414
34667	4.9709	318.695	172.169
34668	4.971	417.764	29.4379
34669	4.971	373.025	256.095
34670	4.971	331.29	219.437
34671	4.9712	456.053	40.5459
34672	4.9714	345.147	346.247
34673	4.9715	397.378	103.997
34674	4.9715	326.489	115.2
34675	4.9715	398.968	105.11
34676	4.9717	463.099	43.4157
34677	4.9719	415.416	112.626
34678	4.972	417.862	122.706
34679	4.9722	421.944	72.5463
34680	4.9725	392.008	86.827
34681	4.9725	364.121	23.8916
34682	4.9726	354.293	143.491
34683	4.9727	306.667	179.245
34684	4.9728	366.155	121.985
34685	4.9729	446.034	37.8716
34686	4.9729	378.551	156.794
34687	4.973	408.562	171.089
34688	4.9731	426.679	71.6423
34689	4.9732	435.339	28.5902
34690	4.9732	348.558	57.3751
34691	4.9733	409.465	7.5227
34692	4.9734	394.633	236.293
34693	4.9734	438.644	55.5393

34694	4.9735	320.023	192.386
34695	4.9737	385.903	72.8321
34696	4.9738	392.769	130.866
34697	4.9739	355.523	65.0951
34698	4.9739	348.442	347.425
34699	4.974	421.898	112.071
34700	4.9741	457.564	312.148
34701	4.9742	395.777	102.842
34702	4.9743	479.13	39.6644
34703	4.9743	372.868	29.5978
34704	4.9745	372.175	8.0262
34705	4.9745	304.084	317.856
34706	4.9746	379.571	228.007
34707	4.9747	351.077	151.457
34708	4.9749	387.257	81.3361
34709	4.975	480.046	72.425
34710	4.9751	417.879	131.333
34711	4.9751	487.42	85.6206
34712	4.9751	432.101	86.5798
34713	4.9753	378.39	127.704
34714	4.9755	347.606	67.5965
34715	4.9756	447.44	16.7207
34716	4.9756	404.558	118.091
34717	4.9756	396.013	93.7303
34718	4.9756	385.09	150.467
34719	4.9756	225.523	27.0041
34720	4.9763	-6.1944	113.421
34721	4.9763	414.015	115.749
34722	4.9765	341.004	129.053
34723	4.9766	385.537	127.365
34724	4.9766	439.314	15.2317
34725	4.9767	459.528	187.097
34726	4.9768	428.604	274.267
34727	4.977	371.194	136.726
34728	4.977	418.414	70.6188
34729	4.9772	325.847	95.4748
34730	4.9772	395.487	30.4277
34731	4.9773	339.957	167.303
34732	4.9774	420.109	145.115
34733	4.9779	417.234	42.4811
34734	4.9781	401.767	123.93
34735	4.9782	365.558	236.014
34736	4.9782	363.747	167.87
34737	4.9782	407.339	118.125
34738	4.9783	439.639	299.203
34739	4.9783	477.348	62.3188
34740	4.9785	350.024	139.822
34741	4.9785	423.718	132.395

34742	4.9786	406.331	61.0384
34743	4.9786	369.314	73.2494
34744	4.9788	405.755	10.4003
34745	4.979	333.691	128.246
34746	4.9791	356.951	103.287
34747	4.9792	410.979	278.717
34748	4.9792	486.595	41.6425
34749	4.9792	468.466	63.6245
34750	4.9793	300.272	261.744
34751	4.9793	372.856	141.384
34752	4.9793	463.196	124.202
34753	4.9797	427.447	39.9063
34754	4.9797	422.764	38.288
34755	4.9798	421.997	25.3203
34756	4.9801	401.818	3.9457
34757	4.9802	500.518	65.3426
34758	4.9802	473.968	84.471
34759	4.9803	369.574	153.247
34760	4.9803	422.932	169.706
34761	4.9804	459.384	294.441
34762	4.9805	463.392	100.146
34763	4.9806	388.274	128.785
34764	4.9807	379.83	273.546
34765	4.9807	392.098	48.3263
34766	4.9808	401.615	115.124
34767	4.9811	499.493	52.3629
34768	4.9811	380.737	164.052
34769	4.9814	408.339	56.2656
34770	4.9814	325.334	112.547
34771	4.9814	302.418	101.125
34772	4.9814	412.362	16.1073
34773	4.9815	357.897	187.009
34774	4.9815	417.107	158.86
34775	4.982	312.976	55.7934
34776	4.9821	415.104	77.9963
34777	4.9823	298.161	304.663
34778	4.9823	296.492	109.068
34779	4.9824	397.777	164.675
34780	4.9825	421.588	61.5575
34781	4.9826	373.145	169.836
34782	4.9827	387.754	95.1572
34783	4.9827	398.035	112.799
34784	4.9829	426.363	115.413
34785	4.9829	328.289	255.682
34786	4.9829	366.391	256.377
34787	4.9829	427.991	103.379
34788	4.9834	406.499	217.14
34789	4.9834	370.726	116.691

34790	4.9835	359.952	45.8304
34791	4.9835	393.145	302.553
34792	4.9837	510.188	47.2268
34793	4.984	458.573	37.0405
34794	4.9841	392.669	107.091
34795	4.9843	417.993	107.575
34796	4.9844	367.377	328.308
34797	4.9845	411.757	111.838
34798	4.985	404.275	115.278
34799	4.9851	410.165	174.278
34800	4.9852	369.88	163.555
34801	4.9854	422.05	340.615
34802	4.9855	502.011	311.456
34803	4.9855	402.049	104.485
34804	4.9856	371.948	180.017
34805	4.9856	382.735	135.843
34806	4.9857	301.274	131.921
34807	4.9858	346.613	95.056
34808	4.9859	427.748	121.057
34809	4.9861	425.479	83.6554
34810	4.9861	371.543	192.281
34811	4.9861	466.258	339.863
34812	4.9862	290.323	102.262
34813	4.9862	355.935	237.297
34814	4.9865	437.436	265.054
34815	4.9867	461.384	288.325
34816	4.9867	383.861	100.151
34817	4.9869	436.813	29.6722
34818	4.987	442.927	43.2845
34819	4.987	181.147	57.6296
34820	4.9871	449.555	24.4869
34821	4.9871	398.952	116.979
34822	4.9872	434.359	133.872
34823	4.9873	432.172	185.434
34824	4.9873	436.135	290.179
34825	4.9874	395.501	233.195
34826	4.9874	288.724	92.1908
34827	4.9875	446.496	20.9167
34828	4.9875	365.684	173.518
34829	4.9875	394.759	82.3763
34830	4.9876	500.788	19.9808
34831	4.9877	278.248	215.308
34832	4.9877	343.436	170.687
34833	4.9878	401.434	129.255
34834	4.9881	393.978	133.147
34835	4.9885	426.422	64.8063
34836	4.9885	423.658	126.326
34837	4.9886	400.494	147.135

34838	4.9891	335.047	168.461
34839	4.9891	347.867	181.391
34840	4.9891	491.152	69.3445
34841	4.9892	420.969	58.3307
34842	4.9894	365.178	132.284
34843	4.9895	361.317	263.811
34844	4.9897	417.004	186.449
34845	4.9899	460.131	81.1742
34846	4.9899	406.366	87.0781
34847	4.99	291.365	171.143
34848	4.9901	462.099	79.5003
34849	4.9902	371.544	177.62
34850	4.9903	309.849	23.6338
34851	4.9903	322.729	113.267
34852	4.9903	342.639	322.856
34853	4.9904	498.19	51.944
34854	4.9904	447.158	182.099
34855	4.9904	457.433	70.6073
34856	4.9904	422.363	303.264
34857	4.9904	363.279	179.37
34858	4.9905	334.376	164.281
34859	4.9906	390.32	149.086
34860	4.9906	415.225	26.3729
34861	4.9908	346.506	237.327
34862	4.9908	371.034	169.659
34863	4.9909	479.548	107.065
34864	4.991	487.956	77.3393
34865	4.991	256.122	154.524
34866	4.991	401.313	296.206
34867	4.991	371.163	113.058
34868	4.991	445.994	32.0644
34869	4.9911	514.526	134.567
34870	4.9911	436.283	81.2385
34871	4.9913	444.465	94.3916
34872	4.9913	347.011	158.766
34873	4.9916	474.965	67.6828
34874	4.9917	442.057	92.9258
34875	4.9918	381.713	224.76
34876	4.9919	265.314	306.602
34877	4.9923	446.372	228.098
34878	4.9923	396.162	36.6852
34879	4.9924	466.36	78.3987
34880	4.9924	318.62	237.662
34881	4.9924	395.948	36.4678
34882	4.9924	381.7	174.478
34883	4.9924	421.271	157.568
34884	4.9925	508.392	259.81
34885	4.9926	407.236	295.706

34886	4.9926	359.507	282.482
34887	4.993	437.817	54.6998
34888	4.993	349.857	125.105
34889	4.9931	414.915	240.501
34890	4.9934	513.592	60.7639
34891	4.9934	319.474	182.317
34892	4.9935	314.74	135.269
34893	4.9936	501.961	50.8463
34894	4.9938	375.207	110.594
34895	4.994	359.74	169.371
34896	4.9941	351.859	357.14
34897	4.9942	347.372	213.206
34898	4.9942	344.761	286.781
34899	4.9943	317.403	121.432
34900	4.9944	373.567	197.594
34901	4.9944	340.714	185.364
34902	4.9945	464.82	121.785
34903	4.9946	433.424	29.9455
34904	4.9947	403.213	71.2644
34905	4.9949	392.089	99.5936
34906	4.9951	424.154	345.28
34907	4.9951	409.374	91.0893
34908	4.9951	439.879	32.9826
34909	4.9952	376.595	34.4823
34910	4.9953	368.266	149.108
34911	4.9953	388.221	221.425
34912	4.9954	317.783	271.915
34913	4.9954	360.147	122.589
34914	4.9954	402.802	120.98
34915	4.9956	346.488	189.959
34916	4.9956	311.947	82.0067
34917	4.9958	492.198	59.3339
34918	4.9958	390.162	157.129
34919	4.9958	364.846	119.522
34920	4.996	438.105	30.3123
34921	4.996	456.303	20.7547
34922	4.9961	372.121	116.464
34923	4.9961	448.129	234.669
34924	4.9962	395.665	130.108
34925	4.9962	404.143	122.77
34926	4.9965	458.382	108.083
34927	4.9965	445.48	188.004
34928	4.9965	441.172	65.2205
34929	4.9965	456.511	119.154
34930	4.997	368.477	82.0728
34931	4.997	264.096	119.139
34932	4.9971	397.447	51.9795
34933	4.9972	371.802	131.052

34934	4.9974	388.964	85.2853
34935	4.9974	449.5	100.938
34936	4.9974	452.871	67.1943
34937	4.9974	407.748	85.8091
34938	4.9974	373.388	161.723
34939	4.9975	380.25	166.277
34940	4.9977	501.175	14.9564
34941	4.9977	315.775	121.415
34942	4.9978	445.983	124.722
34943	4.9978	361.437	118.513
34944	4.9981	493.61	27.5681
34945	4.9981	468.997	23.6492
34946	4.9982	458.425	21.5875
34947	4.9983	449.155	36.3489
34948	4.9983	371.512	98.9776
34949	4.9984	401.559	293.29
34950	4.9987	475.544	145.897
34951	4.9988	349.174	31.8548
34952	4.9989	421.33	53.6908
34953	4.9989	308.569	188.609
34954	4.9989	442.753	354.429
34955	4.999	465.354	235.965
34956	4.9991	374.809	130.931
34957	4.9991	406.286	117.768
34958	4.9992	515.719	31.0178
34959	4.9994	437.893	184.1
34960	4.9994	420.932	65.8325
34961	4.9997	447.506	326.335
34962	4.9998	446.437	266.776
34963	4.9998	403.254	86.5098
34964	4.9999	407.568	62.3517
34965	4.9999	275.178	167.65
34966	4.9999	436.724	78.471
34967	5	440.157	93.0989
34968	5.0003	408.871	167.442
34969	5.0004	335.38	172.57
34970	5.0009	440.731	96.3409
34971	5.0009	475.063	358.745
34972	5.0009	411.623	82.634
34973	5.0009	465.545	78.2738
34974	5.0011	413.64	62.6122
34975	5.0011	444.538	106.781
34976	5.0012	221.772	24.524
34977	5.0013	477.254	267.744
34978	5.0013	523.807	37.17
34979	5.0013	392.459	84.5982
34980	5.0013	410.826	27.8937
34981	5.0015	364.738	116.897

34982	5.0016	272.747	135.234
34983	5.0016	448.941	81.4754
34984	5.0016	377.042	94.3198
34985	5.0017	424.394	31.1224
34986	5.0019	374.374	178.89
34987	5.002	462.132	90.0585
34988	5.002	394.045	185.923
34989	5.0022	429.363	359.289
34990	5.0022	410.196	71.8612
34991	5.0024	370.203	227.656
34992	5.0024	459.006	82.2752
34993	5.0024	382.005	295.643
34994	5.0025	455.423	53.1967
34995	5.0026	431.831	255.138
34996	5.0026	381.284	188.421
34997	5.0027	312.26	233.041
34998	5.0028	420.045	213.831
34999	5.003	344.58	157.205
35000	5.0031	664.468	308.499
35001	5.0034	386.658	115.492
35002	5.0034	356.161	60.864
35003	5.0035	380.312	120.202
35004	5.0037	415.055	113.07
35005	5.0038	406.947	55.4119
35006	5.0039	475.434	75.3306
35007	5.0039	390.993	258.432
35008	5.0039	355.428	269.911
35009	5.0039	339.834	164.583
35010	5.0039	443.752	62.171
35011	5.004	468.72	65.3102
35012	5.004	332.017	224.126
35013	5.0041	429.009	124.307
35014	5.0043	506.274	37.3963
35015	5.0046	395.223	252.707
35016	5.0047	486.381	20.88
35017	5.0051	340.175	201.172
35018	5.0052	409.841	38.5171
35019	5.0054	358.377	47.0823
35020	5.0054	409.576	187.498
35021	5.0055	430.65	20.4129
35022	5.0055	343.209	198.986
35023	5.0055	377.814	279.036
35024	5.006	309.515	321.246
35025	5.0062	313.165	23.2977
35026	5.0062	392.761	236.803
35027	5.0062	378.426	260.759
35028	5.0065	456.516	61.379
35029	5.0065	316.868	255.785

35030	5.0066	388.534	169.832
35031	5.0066	337.033	262.951
35032	5.0066	427.93	115.114
35033	5.0066	356.375	140.617
35034	5.0067	407.353	246.329
35035	5.0067	484.93	23.9568
35036	5.007	420.638	122.83
35037	5.007	352.437	81.7864
35038	5.007	398.425	78.6138
35039	5.0072	424.603	9.631
35040	5.0073	438.024	138.927
35041	5.0073	379.269	83.9151
35042	5.0075	450.52	55.224
35043	5.0078	221.126	71.1246
35044	5.0079	340.88	156.726
35045	5.0081	378.53	131.926
35046	5.0081	469.776	83.1201
35047	5.0084	394.544	96.9428
35048	5.0084	394.331	333.952
35049	5.0086	363.979	120.972
35050	5.0087	421.093	46.4571
35051	5.0088	425.129	67.9718
35052	5.0088	370.161	139.719
35053	5.0089	454.916	269.063
35054	5.009	448.196	62.2888
35055	5.0091	168.831	37.1083
35056	5.0091	254.868	86.6335
35057	5.0093	371.339	108.285
35058	5.0094	396.322	236.09
35059	5.0094	357.058	121.708
35060	5.0094	480.144	36.8777
35061	5.0095	501.8	73.3524
35062	5.0095	385.857	149.738
35063	5.0096	412.642	122.729
35064	5.0101	522.366	64.9598
35065	5.0101	389.219	162.642
35066	5.0102	368.379	93.1612
35067	5.0102	476.328	127.757
35068	5.0103	435.347	280.302
35069	5.0104	403.972	116.996
35070	5.0105	309.937	175.927
35071	5.0105	358.92	293.615
35072	5.0107	340.433	55.214
35073	5.0107	424.183	277.595
35074	5.0108	406.386	267.068
35075	5.0111	419.436	133.25
35076	5.0111	444.029	143.872
35077	5.0112	448.149	99.0063

35078	5.0114	427.932	155.415
35079	5.0115	456.087	11.0174
35080	5.0115	366.253	12.7418
35081	5.0118	415.596	107.213
35082	5.012	399.531	76.6863
35083	5.0122	415.364	110.839
35084	5.0122	358.708	117.423
35085	5.0127	427.373	86.8093
35086	5.0127	352.438	117.486
35087	5.0127	373.542	131.143
35088	5.0127	483.421	46.0517
35089	5.0129	351.999	136.472
35090	5.013	455.685	118.961
35091	5.0133	387.044	183.264
35092	5.0133	349.823	153.059
35093	5.0133	437.729	130.31
35094	5.0134	464.909	83.0718
35095	5.0134	445.308	319.506
35096	5.0135	481.665	315.574
35097	5.0136	492.371	78.4773
35098	5.0138	476.822	127.268
35099	5.0139	477.76	209.925
35100	5.0139	387.499	160.295
35101	5.0141	408.801	125.361
35102	5.0141	329.125	174.938
35103	5.0142	127.996	28.3906
35104	5.0144	337.792	331.689
35105	5.0146	415.435	121.774
35106	5.0147	325.536	134.068
35107	5.0147	345.69	124.122
35108	5.0147	469.237	35.6163
35109	5.0148	387.938	263.211
35110	5.015	381.552	172.752
35111	5.015	453.334	55.2798
35112	5.0154	406.185	88.3759
35113	5.0155	417.311	237.034
35114	5.0155	457.559	29.6066
35115	5.0155	410.472	328.341
35116	5.0156	463.604	145.192
35117	5.0156	396.591	52.9952
35118	5.0157	337.198	281.755
35119	5.0157	480.121	23.3698
35120	5.0158	381.652	155.065
35121	5.0158	357.567	133.471
35122	5.0159	390.37	211.443
35123	5.0159	403.185	104.622
35124	5.0163	371.213	315.71
35125	5.0164	486.997	165.003

35126	5.0165	380.732	256.468
35127	5.0166	452.773	49.6921
35128	5.0166	264.784	141.974
35129	5.0166	402.406	234.713
35130	5.0167	329.204	236.061
35131	5.0167	411.265	117.251
35132	5.0167	387.877	121.282
35133	5.0168	351.686	187.579
35134	5.0168	428.269	56.3544
35135	5.0169	461.048	172.43
35136	5.017	400.731	77.8612
35137	5.017	365.133	139.529
35138	5.017	438.079	65.0339
35139	5.0171	464.32	284.382
35140	5.0172	372.187	108.112
35141	5.0172	397.821	110.015
35142	5.0172	381.014	179.153
35143	5.0173	490.324	51.0836
35144	5.0173	441.421	137.48
35145	5.0173	350.334	51.805
35146	5.0174	-2.7893	81.0685
35147	5.0176	414.57	58.6282
35148	5.0177	339.941	95.8875
35149	5.0177	397.3	134.211
35150	5.0178	155.816	51.4167
35151	5.0181	350.885	274.504
35152	5.0181	419	154.77
35153	5.0181	452.834	126.549
35154	5.0182	415.145	64.492
35155	5.0182	363.588	115.426
35156	5.0182	506.214	10.4516
35157	5.0184	429.951	235.615
35158	5.0186	313.727	111.844
35159	5.0187	413.749	78.0061
35160	5.0187	354.608	90.0426
35161	5.0187	409.812	27.7371
35162	5.0187	381.753	239.405
35163	5.0188	414.91	107.57
35164	5.0188	367.618	200.525
35165	5.0188	403.643	322.674
35166	5.0189	388.99	102.568
35167	5.019	489.339	58.3897
35168	5.0195	333.811	117.688
35169	5.0196	358.916	266.417
35170	5.0196	407.503	121.78
35171	5.0197	386.936	148.205
35172	5.0201	501.537	22.6567
35173	5.0201	307.424	78.4146

35174	5.0202	444.479	75.3477
35175	5.0204	479.438	117.369
35176	5.0205	416.283	239.119
35177	5.0206	405.859	295.036
35178	5.0208	418.732	234.881
35179	5.021	427.286	184.374
35180	5.0213	399.167	159.421
35181	5.0214	345.821	235.966
35182	5.0215	194.018	41.1021
35183	5.0217	424.159	193.451
35184	5.0218	139.892	32.0721
35185	5.0218	384.639	110.792
35186	5.0219	433.961	24.1494
35187	5.0219	511.54	4.9362
35188	5.0219	419.967	110.422
35189	5.0219	411.505	172.711
35190	5.0219	397.396	138.229
35191	5.0219	518.84	18.7487
35192	5.0221	377.704	273.531
35193	5.0222	462.247	266.3
35194	5.0223	445.432	93.6688
35195	5.0223	392.586	190.583
35196	5.0223	407.273	153.207
35197	5.0227	382.17	238.142
35198	5.0231	313.217	319.73
35199	5.0231	372.577	82.3311
35200	5.0232	428.913	322.066
35201	5.0232	428.064	121.641
35202	5.0233	393.619	59.6371
35203	5.0233	375.101	44.1665
35204	5.0233	407.473	15.0738
35205	5.0234	418.226	129.719
35206	5.0235	401.449	126.805
35207	5.0238	420.238	55.6414
35208	5.0239	439.557	120.228
35209	5.024	476.22	41.729
35210	5.024	419.586	89.6342
35211	5.0241	398.521	285.717
35212	5.0242	427.452	69.3991
35213	5.0243	488.683	205.582
35214	5.0245	373.26	152.436
35215	5.0246	402.477	166.217
35216	5.0246	330.463	176.949
35217	5.0247	444.51	273.147
35218	5.0247	390.027	227.929
35219	5.0249	395.529	163.275
35220	5.0249	453.789	323.682
35221	5.0251	448.774	228.287

35222	5.0251	446.471	69.3624
35223	5.0252	345.477	159.932
35224	5.0253	402.377	82.771
35225	5.0254	453.332	17.5716
35226	5.0255	345.717	266.585
35227	5.0255	458.779	125.908
35228	5.0256	398.569	123.342
35229	5.0256	405.626	115.589
35230	5.0257	397.977	250.01
35231	5.0257	497.619	334.911
35232	5.0259	423.62	229.636
35233	5.0259	456.438	149.449
35234	5.026	408.771	54.5407
35235	5.0262	370.72	333.175
35236	5.0262	414.914	190.958
35237	5.0262	426.411	122.015
35238	5.0263	314.817	183.362
35239	5.0263	357.514	111.894
35240	5.0264	414.61	303.771
35241	5.0266	502.908	13.1767
35242	5.0267	387.17	104.143
35243	5.0268	393.969	175.901
35244	5.0269	385.509	197.429
35245	5.027	448.524	43.5369
35246	5.0271	339.643	175.098
35247	5.0273	487.052	56.5046
35248	5.0273	472.157	93.8599
35249	5.0274	374.327	190.328
35250	5.0275	450.207	60.2813
35251	5.0276	367.817	84.6031
35252	5.0278	411.408	30.9964
35253	5.0279	467.057	124.725
35254	5.028	302.99	213.785
35255	5.0282	440.414	112.007
35256	5.0282	415.073	139.218
35257	5.0283	389.937	253.159
35258	5.0283	397.583	125.685
35259	5.0284	421.255	333.279
35260	5.0285	351.551	295.682
35261	5.0286	400.381	324.754
35262	5.0286	388.04	296.343
35263	5.0286	385.631	123.34
35264	5.0287	430.343	231.188
35265	5.0287	338.495	123.943
35266	5.0289	369.03	75.4083
35267	5.029	427.869	216.991
35268	5.029	382.948	164.75
35269	5.0291	414.187	260.16

35270	5.0291	400.415	117.357
35271	5.0291	350.594	177.915
35272	5.0291	431.693	238.746
35273	5.0292	383.667	149.608
35274	5.0294	400.597	254.743
35275	5.0294	523.21	318.816
35276	5.0295	377.489	93.8211
35277	5.0295	387.618	102.487
35278	5.0296	412.949	197.505
35279	5.0297	481.227	65.8043
35280	5.0298	418.459	164.212
35281	5.0301	392.151	22.3867
35282	5.0302	475.821	42.0093
35283	5.0303	413.813	318.239
35284	5.0304	341.472	104.082
35285	5.0304	412.012	136.389
35286	5.0305	352.291	170.063
35287	5.0305	361.529	217.376
35288	5.0307	475.303	32.9149
35289	5.0307	348.303	175.729
35290	5.0308	285.359	222.324
35291	5.0309	376.827	59.8934
35292	5.031	398.209	189.804
35293	5.031	438.976	243.246
35294	5.031	392.064	105.153
35295	5.031	479.684	335.806
35296	5.0311	424.568	42.7614
35297	5.0311	364.173	187.872
35298	5.0312	431.223	1.1854
35299	5.0312	374.422	117.146
35300	5.0314	387.851	71.8134
35301	5.0314	430.533	118.31
35302	5.0314	510.315	42.7265
35303	5.0315	425.049	282.639
35304	5.0315	309.381	95.4746
35305	5.0316	407.461	21.0451
35306	5.0318	431.706	84.4871
35307	5.0318	381.349	117.592
35308	5.032	426.725	164.069
35309	5.0321	400.89	118.273
35310	5.0322	425.188	172.566
35311	5.0324	472.245	55.2291
35312	5.0324	422.11	295.101
35313	5.0324	326.087	301.338
35314	5.0324	400.082	119.597
35315	5.0328	461.713	90.4709
35316	5.0328	343.274	180.665
35317	5.0328	429.691	245.341

35318	5.0329	385.058	72.0089
35319	5.0331	487.775	94.002
35320	5.0331	451.438	100.912
35321	5.0334	501.391	256.883
35322	5.0334	363.995	154.623
35323	5.0336	356.241	229.928
35324	5.0338	454.21	233.572
35325	5.0339	393.825	192.988
35326	5.034	418.886	212.948
35327	5.034	446.773	44.5604
35328	5.0342	368.848	236.258
35329	5.0343	119.168	126.98
35330	5.0343	357.777	234.002
35331	5.0345	376.785	261.033
35332	5.0345	424.002	30.9111
35333	5.0346	408.958	85.1693
35334	5.0346	372.568	127.344
35335	5.0347	449.516	291.73
35336	5.0348	409.228	117.361
35337	5.0348	349.624	126.633
35338	5.0351	438.912	80.5579
35339	5.0352	395.58	137.54
35340	5.0352	369.331	143.3
35341	5.0353	458.999	82.6132
35342	5.0353	365.57	244.48
35343	5.0353	285.71	119.477
35344	5.0353	404.089	82.1992
35345	5.0354	363.797	97.5101
35346	5.0355	492.291	56.5983
35347	5.0356	529.63	38.795
35348	5.0356	440.354	82.8381
35349	5.0359	403.66	217.455
35350	5.036	477.422	34.9329
35351	5.0363	424.311	106.598
35352	5.0366	392.284	156.413
35353	5.0367	463.08	195.819
35354	5.037	399.357	214.544
35355	5.0372	417.903	181.625
35356	5.0372	483.114	79.3915
35357	5.0372	392.176	149.406
35358	5.0372	418.24	42.1619
35359	5.0373	311.157	299.106
35360	5.0375	420.226	260.346
35361	5.0378	399.168	315.702
35362	5.038	410.5	116.571
35363	5.038	401.447	106.566
35364	5.0381	353.388	198.66
35365	5.0381	411.046	37.333

35366	5.0384	440.444	77.4997
35367	5.0386	521.433	86.7722
35368	5.0386	437.728	295.634
35369	5.0389	457.766	119.261
35370	5.0392	416.369	140.014
35371	5.0392	415.25	12.9432
35372	5.0393	501.65	59.0495
35373	5.0393	496.041	96.9329
35374	5.0394	485.796	39.03
35375	5.0396	435.734	150.317
35376	5.0397	371.341	104.699
35377	5.0398	379.873	179.529
35378	5.0398	438.423	197.447
35379	5.0398	445.299	143.884
35380	5.0399	435.208	121.268
35381	5.0402	358.554	222.44
35382	5.0405	482.497	310.188
35383	5.0406	402.299	263.992
35384	5.0407	408.103	118.624
35385	5.0408	420.859	358.606
35386	5.0408	434.2	116.517
35387	5.0408	506.157	3.8924
35388	5.0408	422.263	182.534
35389	5.0408	306.295	115.771
35390	5.041	412.047	161.222
35391	5.041	422.364	134.139
35392	5.041	362.062	132.172
35393	5.0411	362.663	102.244
35394	5.0412	391.375	131.354
35395	5.0413	472.622	347.967
35396	5.0414	454.488	156.769
35397	5.0415	404.229	176.228
35398	5.0415	402.672	162.045
35399	5.0417	320.053	128.771
35400	5.0418	328.34	162.273
35401	5.042	404.312	131.515
35402	5.0422	384.838	81.5712
35403	5.0424	371.914	121.179
35404	5.0428	478.315	41.7993
35405	5.0429	397.135	129.652
35406	5.043	408.598	0.0621
35407	5.0432	405.764	127.996
35408	5.0432	443.687	32.3435
35409	5.0433	427.386	179.091
35410	5.0434	330.813	154.14
35411	5.0434	441.787	42.6721
35412	5.0434	419.152	112.101
35413	5.0435	378.277	216.391

35414	5.0435	412.579	27.8944
35415	5.0437	458.589	88.3486
35416	5.0438	392.347	101.578
35417	5.0438	415.602	142.342
35418	5.0439	368.155	88.1745
35419	5.0441	412.124	132.794
35420	5.0441	442.908	79.3158
35421	5.0441	356.118	76.3544
35422	5.0441	417.661	163.134
35423	5.0442	386.391	25.6974
35424	5.0443	368.267	215.847
35425	5.0444	477.488	62.1952
35426	5.0444	405.581	112.24
35427	5.0444	534.951	28.1859
35428	5.0447	417.136	120.327
35429	5.0448	404.226	182.023
35430	5.0449	440.188	116.416
35431	5.0451	531.14	63.196
35432	5.0452	427.589	97.8393
35433	5.0453	410.9	143.224
35434	5.0454	349.426	180.655
35435	5.0455	362.446	181.668
35436	5.0457	389.364	335.38
35437	5.0457	406.342	118.399
35438	5.0457	321.679	240.024
35439	5.0457	348.886	156.453
35440	5.0458	335.056	145.605
35441	5.046	427.281	272.21
35442	5.0462	329.171	284.879
35443	5.0464	409.905	161.857
35444	5.0465	506.184	46.7449
35445	5.0466	447.229	78.9389
35446	5.0467	396.169	146.892
35447	5.0468	441.335	81.421
35448	5.0469	486.472	46.5158
35449	5.0469	416.698	172.98
35450	5.0469	478.748	352.051
35451	5.0469	445.812	77.2121
35452	5.047	273.978	320.235
35453	5.047	383.102	75.9061
35454	5.0471	343.563	126.981
35455	5.0472	396.341	333.956
35456	5.0474	375.312	64.8786
35457	5.0474	419.11	35.2349
35458	5.0475	451.627	39.9552
35459	5.0475	357.139	168.828
35460	5.0476	476.054	52.8372
35461	5.0477	373.966	64.0085

35462	5.0478	363.714	142.9
35463	5.0479	421.267	114.287
35464	5.0479	396.518	137.542
35465	5.0481	478.814	93.3556
35466	5.0483	498.626	26.5808
35467	5.0483	508.945	25.6099
35468	5.0484	388.486	98.2756
35469	5.0484	418.95	112.814
35470	5.0484	388.463	130.049
35471	5.0484	351.033	233.345
35472	5.0485	369.108	123.409
35473	5.0485	391.758	131.904
35474	5.0486	532.901	68.6734
35475	5.0487	302.792	196.32
35476	5.0488	469.224	124.347
35477	5.0489	446.931	189.839
35478	5.0489	405.755	353.065
35479	5.049	320.192	218.284
35480	5.049	501.586	27.7952
35481	5.0491	528.03	91.51
35482	5.0493	320.964	221.252
35483	5.0493	413.226	115.044
35484	5.0494	381.375	259.634
35485	5.0496	376.205	12.3952
35486	5.0497	439.503	263.926
35487	5.0498	409.816	33.6993
35488	5.0498	400.131	145.961
35489	5.05	422.209	326.694
35490	5.0502	484.162	111.549
35491	5.0502	368.706	96.1651
35492	5.0502	299.657	190.798
35493	5.0504	387.257	290.018
35494	5.0504	409.924	101.448
35495	5.0504	436.802	318.713
35496	5.0505	380.538	272.991
35497	5.0507	427.575	243.762
35498	5.0508	391.885	258.72
35499	5.0508	415.091	138.959
35500	5.0508	449.779	40.4359
35501	5.0512	487.201	63.7309
35502	5.0512	431.248	193.336
35503	5.0513	377.128	9.3598
35504	5.0513	423.089	270.199
35505	5.0514	347.226	222.924
35506	5.0516	458.85	199.916
35507	5.0517	422.417	192.236
35508	5.0518	361.804	100.317
35509	5.0518	419.224	120.656

35510	5.0518	402.283	114.818
35511	5.0518	393.29	194.428
35512	5.0519	548.804	56.3996
35513	5.0521	334.383	113.725
35514	5.0522	406.934	121.382
35515	5.0523	401.582	235.828
35516	5.0523	379.465	171.432
35517	5.0523	437.774	41.6777
35518	5.0525	482.437	108.744
35519	5.0525	463.392	221.257
35520	5.0526	445.033	195.975
35521	5.0527	380.349	276.493
35522	5.0529	400.822	49.747
35523	5.053	422.625	143.409
35524	5.0531	366.23	266.1
35525	5.0532	447.445	130.372
35526	5.0532	425.326	135.014
35527	5.0532	406.709	159.08
35528	5.0534	407.071	247.397
35529	5.0534	443.052	31.0991
35530	5.0535	488.433	29.3456
35531	5.0537	417.387	98.9895
35532	5.0537	400.327	61.8356
35533	5.0538	407.244	5.3598
35534	5.0538	461.076	79.7743
35535	5.0539	405.274	192.509
35536	5.0539	424.037	97.6351
35537	5.054	486.535	225.53
35538	5.0542	475.954	50.9118
35539	5.0542	426.596	301.276
35540	5.0543	403.436	22.944
35541	5.0546	347.698	88.0448
35542	5.0548	401.937	145.592
35543	5.0549	490.801	318.996
35544	5.0549	401.019	278.133
35545	5.055	381.314	156.337
35546	5.055	386.59	18.15
35547	5.0551	319.267	131.777
35548	5.0553	347.819	141.95
35549	5.0553	437.247	230.17
35550	5.0554	487.163	301.352
35551	5.0556	466.935	154.812
35552	5.0556	360.279	305.609
35553	5.0557	421.034	135.037
35554	5.0558	444.841	97.3385
35555	5.0558	525.873	13.8274
35556	5.0561	470.796	111.962
35557	5.0561	448.453	297.279

35558	5.0562	376.826	309.355
35559	5.0562	372.087	295.77
35560	5.0564	367.967	250.795
35561	5.0567	424.823	277.203
35562	5.0567	441.658	264.601
35563	5.0567	366.739	217.838
35564	5.0567	488.208	161.497
35565	5.057	407.236	187.586
35566	5.057	403.784	118.482
35567	5.0571	527.977	314.157
35568	5.0573	461.373	20.8025
35569	5.0573	358.719	229.219
35570	5.0573	452.654	28.6053
35571	5.0573	434.761	275.92
35572	5.0574	554.794	70.4117
35573	5.0574	446.868	116.813
35574	5.0574	476.491	206.341
35575	5.0575	524.967	81.1699
35576	5.0576	451.617	104.684
35577	5.0576	382.592	143.629
35578	5.0576	383.522	142.561
35579	5.058	415.968	58.3231
35580	5.0582	463.53	179.712
35581	5.0583	404.453	222.982
35582	5.0585	467.057	80.778
35583	5.0585	367.898	206.89
35584	5.0585	451.785	131.612
35585	5.0586	482.057	135.375
35586	5.0587	375.172	142.365
35587	5.0587	439.593	31.2576
35588	5.0588	418.465	28.7846
35589	5.0588	489.465	51.812
35590	5.0589	493.574	128.432
35591	5.0589	403.607	85.802
35592	5.059	342.934	123.744
35593	5.059	392.63	289.152
35594	5.0591	486.445	82.5467
35595	5.0593	416.838	170.622
35596	5.0594	350.192	232.053
35597	5.0594	415.806	135.803
35598	5.0595	405.136	117.004
35599	5.0597	407.015	192.788
35600	5.0597	429.348	56.8588
35601	5.0599	482.711	78.9994
35602	5.0599	385.844	170.27
35603	5.06	520.81	89.1213
35604	5.0601	393.22	171.186
35605	5.0602	433.98	193.051

35606	5.0605	342.243	227.284
35607	5.0606	459.032	223.112
35608	5.0606	430.335	258.311
35609	5.061	347.95	261.846
35610	5.061	336.293	113.842
35611	5.061	465.35	44.8406
35612	5.0613	505.806	77.2395
35613	5.0613	332.489	147.679
35614	5.0615	432.101	143.568
35615	5.0616	374.632	104.337
35616	5.0619	480.789	8.3132
35617	5.062	522.692	57.5176
35618	5.062	405.073	234.563
35619	5.0621	441.555	124.574
35620	5.0623	484.56	109.185
35621	5.0624	336.283	166.583
35622	5.0624	429.406	33.4812
35623	5.0626	413.546	247.12
35624	5.0626	440.833	60.7768
35625	5.0627	429.256	114.718
35626	5.0627	420.89	85.625
35627	5.0629	421.945	166.98
35628	5.063	454.172	49.215
35629	5.0631	366.51	186.277
35630	5.0631	491.295	289.496
35631	5.0632	471.172	328.723
35632	5.0632	437.881	179.883
35633	5.0636	456.499	60.4436
35634	5.0636	402.483	279.916
35635	5.0637	422.406	324.349
35636	5.0639	428.147	168.023
35637	5.0639	347.169	95.3435
35638	5.064	377.563	75.0658
35639	5.064	394.065	137.862
35640	5.0641	395.27	186.099
35641	5.0643	390.797	101.52
35642	5.0643	441.886	164.966
35643	5.0644	502.691	246.273
35644	5.0646	378.09	238.39
35645	5.0647	329.804	49.1963
35646	5.0649	381.002	139.332
35647	5.0653	421.26	155.201
35648	5.0654	429.622	273.756
35649	5.0655	383.47	162.013
35650	5.0656	398.312	139.238
35651	5.0656	444.553	38.0168
35652	5.0656	407.495	10.4537
35653	5.0656	467.707	24.3035

35654	5.0658	452.53	96.8011
35655	5.0659	482.133	57.2531
35656	5.0662	452.715	208.917
35657	5.0662	492.342	118.724
35658	5.0663	473.408	117.47
35659	5.0664	426.867	265.425
35660	5.0665	429.119	129.771
35661	5.0665	440.96	56.4092
35662	5.0666	458.344	143.418
35663	5.0666	489.174	58.2246
35664	5.0666	456.214	156.115
35665	5.0668	530.749	43.3244
35666	5.0668	464.458	23.6535
35667	5.0669	357.658	120.31
35668	5.0669	460.107	49.3329
35669	5.0672	445.535	72.3739
35670	5.0672	398.469	116.379
35671	5.0674	482.247	90.0052
35672	5.0675	421.055	111.275
35673	5.0675	528.115	20.5608
35674	5.0677	383.904	151.788
35675	5.0682	400.424	257.707
35676	5.0683	446.292	82.5083
35677	5.0684	417.125	179.654
35678	5.0686	519.604	127.626
35679	5.0686	384.823	38.3573
35680	5.0686	384.824	130.751
35681	5.0687	404.601	110.727
35682	5.0689	424.265	10.2212
35683	5.0691	439.699	64.2236
35684	5.0691	360.302	135.303
35685	5.0692	455.238	178.474
35686	5.0696	407.65	114.965
35687	5.0697	409.238	178.46
35688	5.07	371.716	192.638
35689	5.07	421.953	120.159
35690	5.0701	417.255	140.954
35691	5.0702	373.003	251.66
35692	5.0703	395.756	132.582
35693	5.0704	498.132	50.7466
35694	5.0705	501.168	6.0911
35695	5.0706	465.631	269.765
35696	5.0706	283.881	157.107
35697	5.0707	430.206	263.149
35698	5.071	469.701	28.2169
35699	5.071	455.685	322.333
35700	5.071	425.784	58.1434
35701	5.0711	451.064	226.298

35702	5.0711	370.473	238.061
35703	5.0713	473.309	124.623
35704	5.0713	384.372	71.8362
35705	5.0714	438.783	55.2844
35706	5.0714	412.722	24.6493
35707	5.0715	486.647	83.0234
35708	5.0717	475.387	32.4852
35709	5.0717	478.06	27.5758
35710	5.0717	482.056	69.7672
35711	5.0719	388.876	178.204
35712	5.072	530.412	212.996
35713	5.0723	361.27	253.854
35714	5.0727	392.572	85.5028
35715	5.0728	502.387	73.7768
35716	5.0734	412.3	171.984
35717	5.0735	375.149	178.623
35718	5.0738	356.679	130.86
35719	5.074	364.767	63.9346
35720	5.0745	451.043	114.717
35721	5.0747	458.868	46.6666
35722	5.0748	418.29	184.156
35723	5.0749	471.252	70.129
35724	5.0749	430.717	91.9052
35725	5.075	443.947	111.415
35726	5.0751	455.984	27.3335
35727	5.0752	433.044	274.075
35728	5.0753	433.707	48.6108
35729	5.0754	454.448	119.23
35730	5.0754	439.606	68.6667
35731	5.0756	408.908	115.651
35732	5.0756	428.455	80.1307
35733	5.0757	355.35	247.69
35734	5.0758	479.873	162.091
35735	5.0759	443.254	110.896
35736	5.0759	352.226	191.571
35737	5.076	423.664	226.984
35738	5.076	432.215	30.6558
35739	5.0761	403.039	111.565
35740	5.0762	452.026	325.313
35741	5.0763	402.451	234.273
35742	5.0763	288.71	41.4565
35743	5.0764	477.94	62.8659
35744	5.0764	382.349	189.822
35745	5.0764	398.507	117.434
35746	5.0764	384.154	120.299
35747	5.0765	471.015	137.999
35748	5.0766	484.555	90.2564
35749	5.0766	477.841	14.2263

35750	5.0767	505.405	216.046
35751	5.077	518.477	19.8001
35752	5.0771	468.618	65.359
35753	5.0771	360.214	176.935
35754	5.0771	511.84	16.3278
35755	5.0771	405.21	167.372
35756	5.0773	511.563	59.7413
35757	5.0774	458.933	323.665
35758	5.0774	457.566	313.671
35759	5.0774	356.241	218.787
35760	5.0775	430.433	179.891
35761	5.0776	501.345	304.826
35762	5.0776	372.119	154.809
35763	5.0778	407.555	66.8179
35764	5.0778	375.77	177.57
35765	5.0779	408.398	103.288
35766	5.0781	507.16	62.2049
35767	5.0782	203.342	193.504
35768	5.0782	387.925	306.739
35769	5.0782	396.406	135.553
35770	5.0783	428.439	176.756
35771	5.0783	449.662	124.025
35772	5.0785	475.088	71.8021
35773	5.0786	508.799	38.1341
35774	5.0787	343.294	108.846
35775	5.0787	483.673	38.3432
35776	5.0788	359.708	144.276
35777	5.0788	467.115	287.062
35778	5.079	470.315	91.7065
35779	5.079	444.553	99.42
35780	5.0792	367.679	139.756
35781	5.0793	373.389	220.663
35782	5.0794	448.733	343.832
35783	5.0794	501.558	27.6485
35784	5.0796	404.072	153.368
35785	5.0799	446.625	3.3283
35786	5.08	387.995	145.598
35787	5.0801	400.992	33.4428
35788	5.0801	394.589	131.186
35789	5.0803	339.478	215.501
35790	5.0803	334.568	192.49
35791	5.0804	413.396	121.986
35792	5.0808	451.858	24.6621
35793	5.0814	432.8	115.601
35794	5.0815	370.317	111.244
35795	5.0815	446.935	190.284
35796	5.0815	407.654	184.624
35797	5.0815	463.53	131.482

35798	5.0817	420.071	187.853
35799	5.0817	432.676	110.694
35800	5.0819	415.213	103.464
35801	5.082	414.245	294.672
35802	5.082	443.709	232.362
35803	5.0821	393.069	244.504
35804	5.0821	335.296	87.9713
35805	5.0821	420.489	93.9789
35806	5.0823	386.345	134.399
35807	5.0824	465.73	189.428
35808	5.0826	481.484	57.6853
35809	5.0829	440.739	302.3
35810	5.083	504.389	220.15
35811	5.083	358.701	40.5877
35812	5.083	396.846	3.884
35813	5.083	397.841	331.749
35814	5.0831	339.149	173.185
35815	5.0831	486.223	95.4374
35816	5.0832	440.42	112.092
35817	5.0832	343.875	96.3936
35818	5.0835	489.281	36.6314
35819	5.0835	512.901	46.8744
35820	5.0836	438.254	126.366
35821	5.0837	391.984	94.7179
35822	5.0839	434.335	167.022
35823	5.0839	422.085	130.519
35824	5.084	460.978	178.964
35825	5.084	388.272	166.716
35826	5.0841	576.262	68.4991
35827	5.0842	443.218	25.5299
35828	5.0845	479.583	73.556
35829	5.0846	495.262	30.4653
35830	5.0847	435.9	165.499
35831	5.0848	455.417	123.748
35832	5.0849	-2.9436	221.03
35833	5.085	446.137	56.0782
35834	5.085	426.056	25.0663
35835	5.085	468.857	191.64
35836	5.085	390.068	120.229
35837	5.0851	390.188	191.882
35838	5.0852	494.371	21.7762
35839	5.0856	365.566	145.959
35840	5.0857	380.44	266.855
35841	5.0857	371.379	296.435
35842	5.0858	449.542	50.4144
35843	5.0858	393.25	188.976
35844	5.0859	518.636	22.8957
35845	5.086	-2.7843	79.7073

35846	5.0861	405.461	130.372
35847	5.0863	425.645	296.906
35848	5.0865	370.384	150.174
35849	5.0865	529.934	144.66
35850	5.0866	387.24	118.588
35851	5.0866	449.134	124.328
35852	5.0867	403.793	71.395
35853	5.0871	399.695	144.961
35854	5.0871	404.406	166.811
35855	5.0871	381.275	128.137
35856	5.0874	430.723	127.087
35857	5.0874	394.412	28.1749
35858	5.0875	397.564	63.4016
35859	5.0876	361.055	98.9588
35860	5.0876	514.03	39.908
35861	5.0876	529.387	17.7572
35862	5.0877	441.168	122.079
35863	5.0877	408.079	254.076
35864	5.0878	350.544	117.128
35865	5.088	395.466	120.726
35866	5.088	531.345	18.5614
35867	5.0881	545.929	14.5535
35868	5.0882	656.748	207.053
35869	5.0884	389.851	141.575
35870	5.0884	391.972	226.349
35871	5.0886	421.841	98.8531
35872	5.089	448.159	143.683
35873	5.0891	349.457	182.249
35874	5.0892	368.095	118.985
35875	5.0892	564.139	206.055
35876	5.0893	394.788	333.889
35877	5.0894	381.216	197.181
35878	5.0894	519.569	206.599
35879	5.0897	469.048	293.13
35880	5.0897	414.921	123.59
35881	5.0898	425.417	76.8524
35882	5.0899	517.637	61.4797
35883	5.0899	460.388	25.6139
35884	5.09	455.865	71.6227
35885	5.09	412.926	84.1357
35886	5.0902	586.846	202.685
35887	5.0903	459.301	23.2694
35888	5.0903	368.501	178.401
35889	5.0904	422.922	218.612
35890	5.0904	471.953	33.7845
35891	5.0907	542.877	103.522
35892	5.0908	522.138	63.7045
35893	5.0908	504.27	133.941

35894	5.091	309.286	133.545
35895	5.0912	358.793	174.557
35896	5.0913	476.375	225.822
35897	5.0913	349.723	235.174
35898	5.0915	397.619	227.532
35899	5.0916	417.138	130.41
35900	5.0917	436.758	49.469
35901	5.0917	454.134	92.5045
35902	5.0917	468.157	238.122
35903	5.0921	496.244	61.3662
35904	5.0921	387.298	114.925
35905	5.0921	512.263	41.9942
35906	5.0922	382.959	203.997
35907	5.0923	486.506	146.79
35908	5.0924	454.409	24.1422
35909	5.0924	446.666	311.112
35910	5.0925	466.129	84.6194
35911	5.0925	-2.7978	76.706
35912	5.0929	492.471	311.147
35913	5.0931	388.338	98.2843
35914	5.0931	452.727	39.1318
35915	5.0932	347.021	106.515
35916	5.0932	451.92	128.094
35917	5.0932	401.093	18.8798
35918	5.0932	406.458	171.45
35919	5.0933	420.407	148.883
35920	5.0934	507.948	44.4727
35921	5.0935	396.895	156.386
35922	5.0936	438.49	290.745
35923	5.0937	561.551	53.8623
35924	5.0937	487.814	226.383
35925	5.0938	542.018	51.9062
35926	5.0938	457.482	269.736
35927	5.0939	418.719	156.943
35928	5.0939	454.003	87.8125
35929	5.0939	546.099	30.7006
35930	5.094	469.664	4.2931
35931	5.094	451.953	167.849
35932	5.094	422.675	91.5841
35933	5.0941	436.297	178.504
35934	5.0945	496.195	84.1151
35935	5.0946	484.987	45.8213
35936	5.0946	350.616	172.925
35937	5.0946	403.399	46.3991
35938	5.0947	451.099	82.8544
35939	5.0947	452.176	92.5637
35940	5.0947	489.138	17.429
35941	5.0948	492.975	173.428

35942	5.0949	379.009	10.5737
35943	5.0949	425.429	129.281
35944	5.095	367.395	69.4676
35945	5.095	392.75	188.443
35946	5.0951	519.868	241.429
35947	5.0954	416.592	60.1936
35948	5.0955	462.519	121.166
35949	5.0957	458.604	252.144
35950	5.0958	428.138	219.18
35951	5.0959	435.19	119.764
35952	5.0959	448.503	71.9103
35953	5.0964	347.241	192.429
35954	5.0964	381.249	80.5355
35955	5.0965	506.573	79.7398
35956	5.0965	467.096	334.294
35957	5.0966	428.476	179.232
35958	5.0969	356.154	110.281
35959	5.097	347.335	180.969
35960	5.0971	414.416	175.159
35961	5.0971	315.38	148.6
35962	5.0972	496.461	30.0746
35963	5.0973	365.207	149.809
35964	5.0974	422.318	117.809
35965	5.0975	512.681	47.1156
35966	5.0976	415.185	77.7778
35967	5.0977	518.506	58.3772
35968	5.0978	301.566	262.442
35969	5.098	441.427	85.7457
35970	5.098	437.352	122.678
35971	5.0981	508.113	55.5423
35972	5.0981	436.921	132.919
35973	5.0983	417.306	167.693
35974	5.0984	428.921	70.5927
35975	5.0985	488.261	60.0588
35976	5.0985	316.036	79.7062
35977	5.0986	491.865	124.237
35978	5.0986	313.267	186.839
35979	5.0987	407.445	175.901
35980	5.0987	376.847	151.66
35981	5.0988	396.126	58.1203
35982	5.0991	500.851	18.0587
35983	5.0991	466.672	117.17
35984	5.0991	358.037	118.784
35985	5.0991	387.55	141.861
35986	5.0992	475.778	221.278
35987	5.0992	479.373	276.529
35988	5.0994	417.296	93.3741
35989	5.0994	439.459	258.317

35990	5.0994	408.218	321.67
35991	5.0994	525.656	22.558
35992	5.0994	442.335	132.329
35993	5.0996	391.152	324.721
35994	5.0997	395.695	44.3722
35995	5.0998	474.807	107.902
35996	5.0998	426.518	108.765
35997	5.0999	536.85	91.2365
35998	5.1	412.465	196.47
35999	5.1	397.734	133.083
36000	5.1003	381.192	173.823
36001	5.1003	473.434	51.9414
36002	5.1004	444.278	246.461
36003	5.1006	413.13	157.521
36004	5.1006	397.317	281.469
36005	5.1009	440.333	45.8956
36006	5.101	531.602	33.9664
36007	5.1011	386.65	89.8102
36008	5.1012	541.166	79.7845
36009	5.1012	402.526	138.86
36010	5.1015	436.413	114.626
36011	5.1016	413.491	143.555
36012	5.1016	378.386	339.74
36013	5.1017	416.743	294.263
36014	5.1017	423.109	183.268
36015	5.1017	492.421	119.794
36016	5.1018	418.384	193.746
36017	5.102	359.843	287.994
36018	5.1021	456.339	104.379
36019	5.1024	406.375	178.587
36020	5.1025	482.256	112.102
36021	5.1025	372.699	32.4939
36022	5.1026	489.806	56.9123
36023	5.1026	534.233	84.027
36024	5.1026	441.532	39.3689
36025	5.1026	459.353	119.058
36026	5.1027	431.606	120.611
36027	5.1028	452.024	188.323
36028	5.1028	549.24	59.3997
36029	5.103	416.055	85.6412
36030	5.1033	431.961	222.815
36031	5.1033	388.765	174.402
36032	5.1033	437.199	90.6097
36033	5.1036	414.482	137.646
36034	5.1039	388.629	220.119
36035	5.1039	410.261	86.2414
36036	5.1039	543.884	205.067
36037	5.104	465.77	45.8901

36038	5.1041	327.104	180.384
36039	5.1043	480.31	63.8621
36040	5.1043	369.127	161.949
36041	5.1045	400.338	250.04
36042	5.1046	548.816	68.6913
36043	5.1046	345.854	246.874
36044	5.1046	470.78	107.344
36045	5.1046	402.756	74.4493
36046	5.1048	495.211	38.2925
36047	5.1049	479.21	62.7273
36048	5.1049	441.447	53.2726
36049	5.1049	436.746	100.546
36050	5.1049	427.037	154.774
36051	5.105	465.885	98.1264
36052	5.1051	436.687	112.148
36053	5.1052	402.452	256.42
36054	5.1052	409.754	233.152
36055	5.1052	429.693	83.4927
36056	5.1053	343.863	190.764
36057	5.1055	455.829	72.9025
36058	5.1055	175.512	194.749
36059	5.1055	403.239	97.7906
36060	5.1055	326.982	145.474
36061	5.1055	466.289	184.39
36062	5.1057	439.046	111.861
36063	5.1057	501.733	29.3307
36064	5.1059	416.592	180.113
36065	5.106	528.45	61.709
36066	5.1062	356.931	25.4027
36067	5.1063	476.903	49.2181
36068	5.1063	461.052	109.227
36069	5.1064	386.426	96.0507
36070	5.1064	400.512	234.959
36071	5.1065	285.977	175.524
36072	5.1069	448.079	274.456
36073	5.1069	448.661	280.006
36074	5.1071	436.75	202.327
36075	5.1071	391.386	177.747
36076	5.1071	324	251.544
36077	5.1072	404.595	188.985
36078	5.1074	441.862	296.949
36079	5.1074	395.852	289.189
36080	5.1075	460.485	291.75
36081	5.1076	440.701	334.664
36082	5.1077	435.288	119.002
36083	5.1078	482.379	19.2312
36084	5.1081	479.336	56.5676
36085	5.1083	427.737	232.581

36086	5.1084	408.316	141.619
36087	5.1084	433.57	137.421
36088	5.1086	476.222	351.213
36089	5.1086	478.606	128.452
36090	5.1087	302.466	280.386
36091	5.1087	376.394	138.118
36092	5.1087	362.173	77.882
36093	5.1087	672.283	307.205
36094	5.1088	438.191	247.256
36095	5.109	415.342	26.8717
36096	5.1093	401.767	134.255
36097	5.1094	467.846	100.763
36098	5.1095	353.898	229.88
36099	5.1095	441.447	332.845
36100	5.1097	341.654	119.58
36101	5.1097	441.046	326.992
36102	5.1098	454.232	111.886
36103	5.11	424.88	45.2229
36104	5.11	451.461	183.012
36105	5.1103	422.671	4.9867
36106	5.1103	474.82	75.9589
36107	5.1104	466.998	293.967
36108	5.1104	419.342	311.515
36109	5.1104	362.818	151.188
36110	5.1104	376.636	150.52
36111	5.1104	444.883	123.907
36112	5.1104	437.54	47.6964
36113	5.1107	364.325	156.943
36114	5.1108	520.437	305.602
36115	5.111	320.87	23.6384
36116	5.111	411.811	42.2572
36117	5.1111	388.146	160.376
36118	5.1111	423.206	28.6682
36119	5.1112	389.004	90.4173
36120	5.1113	491.191	31.6084
36121	5.1114	431.187	189.009
36122	5.1118	423.485	80.6554
36123	5.1118	381.437	194.416
36124	5.1118	465.136	85.3581
36125	5.1118	485.255	149.495
36126	5.1119	197.979	58.2351
36127	5.112	366.41	217.406
36128	5.1121	446.05	57.776
36129	5.1122	505.382	269.794
36130	5.1124	489.349	42.7736
36131	5.1125	473.117	319.218
36132	5.1129	411.088	149.699
36133	5.113	422.608	71.6996

36134	5.113	437.831	79.1772
36135	5.1134	405.739	82.9693
36136	5.1134	423.236	188.565
36137	5.1135	426.54	121.929
36138	5.1136	408.621	234.274
36139	5.1136	434.039	90.5671
36140	5.1138	384.962	216.671
36141	5.1141	445.574	122.018
36142	5.1142	423.916	277.878
36143	5.1142	378.81	85.7645
36144	5.1143	420.877	39.6895
36145	5.1143	446.599	26.3737
36146	5.1144	420.03	177.735
36147	5.1145	349.662	188.716
36148	5.1145	440.602	158.029
36149	5.1146	359.423	132.354
36150	5.1148	453.573	233.682
36151	5.1148	473.195	58.7707
36152	5.1149	460.519	189.962
36153	5.115	438.484	117.661
36154	5.1151	507.058	42.0277
36155	5.1151	423.767	90.4083
36156	5.1153	489.35	80.8286
36157	5.1158	473.328	227.235
36158	5.1158	383.583	94.8276
36159	5.116	387.754	178.426
36160	5.1161	390.336	103.581
36161	5.1161	455.864	211.319
36162	5.1162	463.155	86.4722
36163	5.1163	449.841	190.258
36164	5.1164	422.31	43.4581
36165	5.1165	417.466	193.231
36166	5.1165	484.806	69.4738
36167	5.1165	436.193	237.546
36168	5.1165	522.042	126.669
36169	5.1166	403.255	142.68
36170	5.1169	428.823	143.12
36171	5.1175	420.858	178.994
36172	5.1177	465.853	148.683
36173	5.1177	521.633	122.39
36174	5.1182	477.15	107.274
36175	5.1182	485.898	293.83
36176	5.1182	393.425	120.975
36177	5.1184	483.233	17.7034
36178	5.1186	552.616	61.2647
36179	5.1188	348.301	194.122
36180	5.1189	549.063	18.0355
36181	5.119	375.156	350.455

36182	5.1193	420.341	304.037
36183	5.1194	431.108	68.1146
36184	5.1194	458.243	324.16
36185	5.1196	411.413	222.068
36186	5.1198	436.338	257.343
36187	5.12	519.989	47.3754
36188	5.1203	398.737	128.333
36189	5.1203	365.169	148.922
36190	5.1206	550.28	36.8324
36191	5.1206	372.301	73.7123
36192	5.1207	469.839	281.441
36193	5.1208	456.779	68.0469
36194	5.121	442.794	140.867
36195	5.1212	543.149	2.9494
36196	5.1213	390.293	50.2492
36197	5.1213	436.815	175.752
36198	5.1214	473.648	211.754
36199	5.1215	443.581	94.8202
36200	5.1216	478.021	266.063
36201	5.1216	504.915	75.5188
36202	5.1216	353.46	43.1173
36203	5.1217	426.566	88.8689
36204	5.1217	466.805	174.725
36205	5.1217	512.106	329.669
36206	5.1218	477.335	43.4781
36207	5.1218	425.904	209.86
36208	5.1219	479.855	174.021
36209	5.1219	380.169	108.043
36210	5.122	293.647	142.81
36211	5.1223	475.285	139.493
36212	5.1223	528.156	49.5286
36213	5.1224	459.901	74.3976
36214	5.1225	460.987	295.129
36215	5.1225	449.098	3.6955
36216	5.1225	375.453	196.429
36217	5.1225	462.572	25.4051
36218	5.1226	516.224	186.594
36219	5.1226	445.335	196.366
36220	5.1226	478.323	83.9512
36221	5.1227	459.731	212.25
36222	5.1228	445.443	20.1139
36223	5.1229	473.79	41.5867
36224	5.1229	500.316	82.3066
36225	5.1234	408.838	137.536
36226	5.1235	443.065	130.272
36227	5.1235	366.662	114.979
36228	5.1236	414.095	24.1747
36229	5.1237	401.732	278.345

36230	5.1237	429.274	218.452
36231	5.124	498.373	202.821
36232	5.124	478.83	239.161
36233	5.1241	486.391	80.7429
36234	5.1246	477.07	70.0928
36235	5.1247	489.574	26.5717
36236	5.1248	424.532	61.5704
36237	5.1249	526.645	58.9144
36238	5.125	400.619	291.157
36239	5.1253	528.725	124.106
36240	5.1253	498.929	283.484
36241	5.1253	380.553	170.302
36242	5.1254	380.895	42.3446
36243	5.1256	487.411	117.992
36244	5.1257	416.169	297.603
36245	5.1257	540.663	24.657
36246	5.1258	552.288	258.733
36247	5.1259	490.439	58.4931
36248	5.1259	399.579	184.458
36249	5.1259	367.257	69.1463
36250	5.126	453.888	83.7183
36251	5.1261	425.457	187.445
36252	5.1261	429.48	208.958
36253	5.1263	424.636	172.528
36254	5.1265	523.841	51.5165
36255	5.1265	348.203	179.302
36256	5.1268	323.823	151.418
36257	5.1269	522.572	58.7802
36258	5.127	409.699	298.26
36259	5.1271	408.132	307.83
36260	5.1272	443.891	271.242
36261	5.1272	432.655	292.401
36262	5.1272	428.2	145.114
36263	5.1274	405.85	131.135
36264	5.1275	449.894	291.633
36265	5.1276	437.52	86.2487
36266	5.1276	321.902	307.026
36267	5.1277	324.706	174.01
36268	5.1278	465.161	87.0581
36269	5.1281	396.569	160.238
36270	5.1281	389.875	191.912
36271	5.1282	389.432	120.679
36272	5.1283	409.047	84.1479
36273	5.1285	357.291	250.288
36274	5.1286	427.272	115.921
36275	5.1288	341.828	225.474
36276	5.1289	399.927	8.1231
36277	5.129	374.196	119.519

36278	5.1291	394.62	56.9736
36279	5.1292	498.829	71.5947
36280	5.1293	426.097	178.992
36281	5.1293	437.025	74.1085
36282	5.1293	442.565	127.859
36283	5.1294	488.085	297.952
36284	5.1295	426.38	87.3275
36285	5.1296	-2.7112	259.402
36286	5.1298	479.326	60.5607
36287	5.13	383.511	234.653
36288	5.1301	480.192	106.235
36289	5.1301	462.459	75.6233
36290	5.1302	392.385	112.835
36291	5.1303	388.74	302.699
36292	5.1303	451.484	101.483
36293	5.1308	364.797	139.811
36294	5.1309	393.972	77.9381
36295	5.1311	406.934	173.702
36296	5.1311	448.299	56.0415
36297	5.1312	396.078	187.825
36298	5.1312	450.389	93.3914
36299	5.1314	483.546	58.337
36300	5.1315	407.44	49.335
36301	5.1322	480.838	272.425
36302	5.1323	422.103	159.755
36303	5.1325	460.622	79.0609
36304	5.1328	420.493	67.274
36305	5.1329	459.44	273.104
36306	5.1329	477.25	52.8686
36307	5.1329	443.077	235.73
36308	5.133	397.331	358.112
36309	5.133	528.038	114.362
36310	5.1334	428.267	29.6803
36311	5.1336	380.684	177.691
36312	5.1337	429.774	94.3564
36313	5.1339	395.39	91.7191
36314	5.134	521.077	66.296
36315	5.1341	374.609	277.085
36316	5.1341	387.57	146.899
36317	5.1343	543.064	28.0807
36318	5.1344	453.696	300.169
36319	5.1345	430.95	86.9338
36320	5.1346	520.244	71.0781
36321	5.1347	529.7	72.7652
36322	5.1348	447.914	97.3809
36323	5.1349	454.757	153.742
36324	5.1355	442.086	227.545
36325	5.1356	429.628	24.6311

36326	5.1357	76.2117	26.2263
36327	5.1357	454.508	297.441
36328	5.1357	387.797	137.774
36329	5.1358	378.042	197.513
36330	5.1359	400.042	30.3579
36331	5.1361	443.192	176.079
36332	5.1361	532.178	58.585
36333	5.1362	354.294	151.228
36334	5.1362	464.527	161.743
36335	5.1363	524.1	24.2496
36336	5.1363	537.932	112.681
36337	5.1364	361.493	113.792
36338	5.1365	459.218	157.761
36339	5.1366	559.384	291.715
36340	5.1367	438.901	118.112
36341	5.1371	461.436	225.209
36342	5.1374	445.836	333.431
36343	5.1374	528.89	37.6438
36344	5.1375	377.241	166.825
36345	5.1375	529.997	40.0821
36346	5.1377	470.542	124.306
36347	5.1377	440.642	3.6232
36348	5.1378	420.144	67.6496
36349	5.1378	476.274	98.4229
36350	5.1378	570.571	207.693
36351	5.1379	457.18	127.692
36352	5.138	478.954	180.045
36353	5.1381	429.758	264.459
36354	5.1382	463.652	58.9059
36355	5.1382	426.129	144.525
36356	5.1384	360.999	155.251
36357	5.1386	448.338	259.962
36358	5.1386	433.897	264.456
36359	5.1388	465.882	109.553
36360	5.1388	494.078	89.9348
36361	5.1389	449.917	327.01
36362	5.1393	463.736	158.504
36363	5.1394	539.515	352.595
36364	5.1394	414.249	129.34
36365	5.1395	521.399	25.7003
36366	5.1397	409.207	322.238
36367	5.1397	444.428	331.81
36368	5.1399	463.011	248.036
36369	5.1399	479.804	25.0084
36370	5.1399	420.253	153.73
36371	5.1399	435.647	250.858
36372	5.14	463.612	321.156
36373	5.14	382.959	35.7853

36374	5.14	407.527	176.243
36375	5.1406	458.177	129.474
36376	5.1407	447.369	43.4633
36377	5.1407	537.496	287.946
36378	5.1408	468.26	324.668
36379	5.1409	386.426	107.625
36380	5.1409	441.212	45.4688
36381	5.1411	489.498	81.825
36382	5.1413	446.21	124.872
36383	5.1414	398.895	169.534
36384	5.1414	429.422	251.297
36385	5.1415	408.719	188.055
36386	5.1416	387.832	301.805
36387	5.1418	405.929	324.277
36388	5.1422	478.931	226.654
36389	5.1422	423.494	91.8699
36390	5.1425	354.551	154.484
36391	5.1427	35.1583	283.808
36392	5.1428	524.357	187.867
36393	5.1431	389.614	97.6439
36394	5.1433	513.302	95.2035
36395	5.1433	537.21	11.4927
36396	5.1436	480.162	69.5226
36397	5.1439	441.245	253.057
36398	5.144	421.416	124.436
36399	5.1441	481.176	229.848
36400	5.1442	434.998	142.248
36401	5.1442	377.662	115.249
36402	5.1443	406.75	233.974
36403	5.1445	461.382	75.9079
36404	5.1445	449.718	49.9752
36405	5.1446	437.227	326.252
36406	5.1447	445.795	119.132
36407	5.1448	441.408	116.638
36408	5.1448	438.891	129.805
36409	5.145	370.215	172.853
36410	5.1451	469.452	62.4905
36411	5.1451	430.55	113.621
36412	5.1453	456.111	13.8763
36413	5.1454	474.234	304.387
36414	5.1455	397.579	160.128
36415	5.1457	388.32	169.03
36416	5.1457	460.149	142.841
36417	5.146	524.348	88.9483
36418	5.1463	500.955	266.516
36419	5.1464	428.965	26.1751
36420	5.1465	438.727	85.8075
36421	5.1466	446.74	176.901

36422	5.1469	435.942	225.206
36423	5.147	461.715	142.853
36424	5.1474	398.519	88.5393
36425	5.1475	432.343	77.1329
36426	5.1475	409.16	115.109
36427	5.1476	438.767	21.9841
36428	5.1479	447.757	79.4346
36429	5.148	447.667	239.579
36430	5.148	394.936	72.5914
36431	5.1482	473.62	255.517
36432	5.1483	414.297	130.918
36433	5.1483	427.382	105.356
36434	5.1483	527.589	249.974
36435	5.1485	484.457	42.7133
36436	5.1486	447.03	258.771
36437	5.1486	517.737	63.7094
36438	5.1486	503.773	127.125
36439	5.1486	455.001	54.1188
36440	5.1487	414.769	215.425
36441	5.1487	478.791	59.0763
36442	5.1489	466.451	268.516
36443	5.1489	378.865	118.537
36444	5.149	495.113	158.502
36445	5.149	442.988	118.313
36446	5.149	494.674	125.393
36447	5.1491	457.995	132.777
36448	5.1492	403.833	52.123
36449	5.1492	492.36	313.914
36450	5.1493	448.499	6.169
36451	5.1494	544.325	56.836
36452	5.1495	455.717	89.6541
36453	5.1495	450.397	83.1068
36454	5.1502	399.326	230.825
36455	5.1503	520.771	22.7067
36456	5.1504	426.353	100.511
36457	5.1505	388.749	35.6655
36458	5.1505	388.041	127.871
36459	5.1506	467.306	296.939
36460	5.1507	408.007	44.0453
36461	5.1508	310.145	125.582
36462	5.1508	510.015	70.8867
36463	5.151	420.073	171.484
36464	5.1511	477.379	60.1366
36465	5.1513	481.107	312.9
36466	5.1513	502.479	125.983
36467	5.1517	475.303	83.4205
36468	5.1518	440.246	156.481
36469	5.1518	419.481	85.8137

36470	5.1519	402.298	287.656
36471	5.1519	390.154	40.6719
36472	5.1521	434.185	123.554
36473	5.1521	467.65	110.192
36474	5.1522	404.24	235.875
36475	5.1523	353.407	135.246
36476	5.1526	387.375	68.2129
36477	5.1527	523.124	118.127
36478	5.1528	478.246	290.858
36479	5.1529	396.987	126.917
36480	5.1529	387.689	104.621
36481	5.1531	501.54	25.081
36482	5.1532	471.863	36.1998
36483	5.1533	434.863	197.273
36484	5.1533	498.24	27.5079
36485	5.1533	471.961	297.099
36486	5.1534	479.044	231.26
36487	5.1534	439.94	135.54
36488	5.1534	452.41	80.7637
36489	5.1534	532.77	58.2324
36490	5.1535	485.636	331.39
36491	5.1536	441.396	75.9094
36492	5.1536	457.163	109.234
36493	5.1537	506.498	146.557
36494	5.1538	498.332	103.592
36495	5.154	421.177	118.957
36496	5.154	425.773	122.016
36497	5.1541	428.742	157.586
36498	5.1542	396.422	237.899
36499	5.1542	472.697	334.272
36500	5.1542	430.404	187.576
36501	5.1544	502.583	113.863
36502	5.1547	502.058	81.2166
36503	5.1547	461.772	161.08
36504	5.1547	487.12	72.0367
36505	5.1548	441.915	122.379
36506	5.1548	374.887	146.184
36507	5.1548	483.918	328.587
36508	5.1551	463.488	218.324
36509	5.1551	412.208	82.0995
36510	5.1551	481.86	285.825
36511	5.1553	349.49	229.77
36512	5.1555	508.768	251.789
36513	5.1556	392.153	295.089
36514	5.1556	349.708	171.331
36515	5.1557	479.345	59.4233
36516	5.1557	436.98	282.145
36517	5.1557	457.378	273.147

36518	5.1558	418.253	70.3457
36519	5.1559	374.981	71.4202
36520	5.156	427.933	175.74
36521	5.156	460.979	160.128
36522	5.1562	498.35	132.848
36523	5.1564	477.526	49.2021
36524	5.1567	397.344	218.741
36525	5.1568	430.139	132.694
36526	5.1568	390.728	160.604
36527	5.1571	424.127	59.978
36528	5.1573	425.716	191.747
36529	5.1574	482.989	163.282
36530	5.1578	517.997	71.9655
36531	5.1578	452.419	37.0554
36532	5.1579	527.073	22.6478
36533	5.1579	444.055	80.4217
36534	5.158	420.8	304.668
36535	5.1581	556.997	10.4842
36536	5.1583	501.329	208.281
36537	5.1584	389.103	26.8947
36538	5.1585	506.873	293.712
36539	5.1586	385.404	158.887
36540	5.1586	402.902	116.376
36541	5.1586	526.277	55.3372
36542	5.1588	477.875	67.7358
36543	5.1588	467.702	104.157
36544	5.1588	522.22	82.4892
36545	5.1591	404.307	335.591
36546	5.1592	441.305	45.962
36547	5.1594	352.629	261.311
36548	5.1598	480.936	60.5432
36549	5.1599	495.699	86.8377
36550	5.16	386.575	132.754
36551	5.16	374.595	177.098
36552	5.16	420.345	11.5336
36553	5.1608	522.508	65.6535
36554	5.1608	380.189	121.08
36555	5.1608	435.198	137.955
36556	5.1609	451.173	127.693
36557	5.161	482.438	235.972
36558	5.1612	481.005	76.2773
36559	5.1612	425.238	119.313
36560	5.1612	427.158	122.509
36561	5.1614	431.53	147.772
36562	5.1614	436.736	324.699
36563	5.1615	391.211	188.329
36564	5.1615	450.476	133.225
36565	5.1617	400.648	80.5271

36566	5.1617	398.488	189.796
36567	5.162	325.232	89.226
36568	5.1621	507.441	299.866
36569	5.1621	565.332	202.208
36570	5.1622	418.584	223.286
36571	5.1623	405.13	25.614
36572	5.1624	368.861	118.392
36573	5.1626	423.954	294.268
36574	5.1627	415.848	337.306
36575	5.163	421.044	74.6036
36576	5.163	467.373	28.9463
36577	5.163	430.217	111.384
36578	5.1634	438.94	166.489
36579	5.1634	419.682	165.431
36580	5.1634	513.909	115.536
36581	5.1635	320.259	212.752
36582	5.1635	459.05	205.16
36583	5.1635	427.063	154.761
36584	5.1635	382.38	115.106
36585	5.1635	427.327	181.533
36586	5.1637	510.67	175.444
36587	5.1639	436.576	115.037
36588	5.1639	414.624	302.078
36589	5.164	419.075	197.162
36590	5.1641	386.837	151.852
36591	5.1642	392.503	110.555
36592	5.1642	348.226	138.036
36593	5.1642	423.825	112.706
36594	5.1643	434.839	335.371
36595	5.1644	421.705	119.199
36596	5.1644	433.431	278.712
36597	5.1644	468.099	167.326
36598	5.1646	537.37	305.079
36599	5.1647	424.164	144.492
36600	5.1648	314.587	99.8161
36601	5.1648	495.507	73.8855
36602	5.1648	419.66	109.777
36603	5.1648	490.82	25.8865
36604	5.165	460.882	58.9545
36605	5.1652	463.222	254.85
36606	5.1653	403.581	249.559
36607	5.1653	453.915	151.881
36608	5.1654	501.46	69.0037
36609	5.1654	519.57	123.807
36610	5.1656	461.139	139.351
36611	5.1658	471.052	68.3448
36612	5.1659	447.715	130.303
36613	5.166	418.597	250.949

36614	5.1661	527.672	32.0968
36615	5.1662	547.217	12.4563
36616	5.1663	468.374	54.1837
36617	5.1665	379.666	209.007
36618	5.1666	488.665	147.605
36619	5.1666	410.267	12.3695
36620	5.1666	496.921	129.878
36621	5.1668	461.452	129.145
36622	5.167	367.684	202.417
36623	5.167	508.202	63.8978
36624	5.1671	558.898	69.1526
36625	5.1671	525.21	28.6386
36626	5.1671	419.251	87.3974
36627	5.1672	502.455	306.812
36628	5.1672	496.551	38.0915
36629	5.1676	486.105	32.2111
36630	5.1676	431.099	92.0116
36631	5.1676	497.334	274.397
36632	5.1676	523.179	51.7261
36633	5.1679	464.534	177.001
36634	5.168	318.834	120.343
36635	5.168	496.53	229.919
36636	5.1681	510.43	292.285
36637	5.1682	408.107	166.692
36638	5.1683	506.689	56.2487
36639	5.1685	422.124	252.196
36640	5.1685	540.806	12.8805
36641	5.1687	479.835	115.649
36642	5.1687	397.425	137.008
36643	5.1688	534.44	78.5492
36644	5.1688	542.319	244.172
36645	5.1688	409.894	192.196
36646	5.1689	503.863	35.8754
36647	5.169	457.379	51.7403
36648	5.169	423.095	123.442
36649	5.1691	495.216	324.321
36650	5.1694	381.707	96.7298
36651	5.1695	450.918	156.519
36652	5.17	499.824	126.272
36653	5.1701	524.126	344.702
36654	5.1701	519.972	229.126
36655	5.1704	537.717	46.7716
36656	5.1704	431.327	232.336
36657	5.1704	475.248	249.494
36658	5.1705	507.239	290.723
36659	5.1709	435.193	120.485
36660	5.1709	411.441	167.195
36661	5.1709	510.287	56.883

36662	5.1711	385.561	194.716
36663	5.1712	525.562	81.1332
36664	5.1715	404.512	119.016
36665	5.1715	435.023	135.373
36666	5.1715	463.167	187.705
36667	5.1717	433.047	157.477
36668	5.1717	468.335	95.2201
36669	5.1718	320.835	150.307
36670	5.1721	536.299	23.0317
36671	5.1723	412.5	125.319
36672	5.1725	189.781	31.0373
36673	5.1725	429.682	283.15
36674	5.1725	461.384	142.523
36675	5.1726	448.043	12.6533
36676	5.1727	442.15	154.153
36677	5.1727	450.714	54.3378
36678	5.1727	412.529	44.4957
36679	5.1727	440.401	55.7513
36680	5.1728	454.209	83.2388
36681	5.1732	489.418	35.9784
36682	5.1732	524.732	211.666
36683	5.1732	391.772	186.001
36684	5.1732	490.737	43.3701
36685	5.1732	470.333	3.9861
36686	5.1735	497.064	97.4926
36687	5.1736	444.644	123.735
36688	5.1736	468.321	93.2409
36689	5.1737	435.66	115.879
36690	5.1738	431.782	144.548
36691	5.1739	378.975	209.878
36692	5.1739	413.184	159.56
36693	5.1739	485.973	65.3756
36694	5.174	438.939	169.331
36695	5.1741	573.952	309.59
36696	5.1742	349.51	258.945
36697	5.1742	438.227	194.836
36698	5.1747	339.381	150.709
36699	5.1748	517.265	83.6967
36700	5.1752	553.488	28.5516
36701	5.1754	470.224	80.5529
36702	5.1754	410.013	120.492
36703	5.1756	458.403	119.738
36704	5.1756	431.67	165.193
36705	5.1756	498.417	124.933
36706	5.1757	528.023	269.607
36707	5.1757	485.526	122.674
36708	5.1757	547.872	81.0992
36709	5.1758	459.023	112.498

36710	5.1762	459.756	117.288
36711	5.1763	472.357	90.7967
36712	5.1764	271.046	50.4748
36713	5.1765	465.594	70.9254
36714	5.1767	477.965	177.53
36715	5.1767	623.737	208.557
36716	5.1769	511.87	121.185
36717	5.1769	379.072	140.257
36718	5.1769	386.158	192.358
36719	5.1769	494.203	91.7032
36720	5.1773	507.242	103.441
36721	5.1774	485.913	228.727
36722	5.1774	379.155	169.624
36723	5.1776	479.785	60.9292
36724	5.1777	393.942	123.787
36725	5.1777	395.965	144.803
36726	5.1778	373.772	155.79
36727	5.1778	426.936	76.0845
36728	5.1778	462.277	93.6291
36729	5.1779	413.753	319.061
36730	5.1779	518.867	125.941
36731	5.1781	426.91	155.083
36732	5.1782	447.21	173.365
36733	5.1782	433.881	167.276
36734	5.1782	423.852	114.236
36735	5.1782	522.558	233.71
36736	5.1782	505.282	124.704
36737	5.1783	480.099	108.513
36738	5.1784	430.005	144.191
36739	5.1785	377.418	137.481
36740	5.1785	477.71	31.4852
36741	5.1785	509.913	211.129
36742	5.1786	482.356	123.144
36743	5.1786	440.028	123.007
36744	5.1786	420.529	78.9748
36745	5.1787	472.722	60.3303
36746	5.1788	459.258	132.599
36747	5.1788	451.382	243.871
36748	5.1789	416.005	177.845
36749	5.1789	385.328	81.6096
36750	5.179	358.361	45.6555
36751	5.1791	450.313	188.533
36752	5.1793	425.417	157.328
36753	5.1793	404.97	154.556
36754	5.1793	421.902	71.6726
36755	5.1794	305.4	85.1456
36756	5.1794	475.006	186.513
36757	5.1796	428.617	183.268

36758	5.1796	445.917	104.917
36759	5.1798	574.018	107.168
36760	5.1798	341.389	219.312
36761	5.1798	429.798	116.037
36762	5.1801	481.759	129.97
36763	5.1803	433.02	144.891
36764	5.1804	443.067	76.4976
36765	5.1804	555.307	242.456
36766	5.1807	459.048	284.507
36767	5.1807	411.702	115.548
36768	5.1807	550.268	200.99
36769	5.1808	489.665	258.36
36770	5.1808	457.209	104.673
36771	5.1808	431.079	95.9207
36772	5.1809	517.099	74.7443
36773	5.1809	443.053	331.467
36774	5.181	133.28	17.9262
36775	5.181	646.25	76.2922
36776	5.1811	504.569	116.677
36777	5.1813	543.022	55.37
36778	5.1813	379.923	277.993
36779	5.1813	444.197	67.0992
36780	5.1814	444.741	228.719
36781	5.1815	429.98	61.52
36782	5.1815	531.551	10.6969
36783	5.1815	362.212	109.956
36784	5.1815	534.017	324.73
36785	5.1817	429.983	83.6506
36786	5.1819	441.239	121.261
36787	5.1819	500.801	22.1739
36788	5.182	447.588	111.73
36789	5.182	471.488	113.268
36790	5.1822	485.472	91.5723
36791	5.1822	475.484	69.0742
36792	5.1822	469.218	135.536
36793	5.1824	460.888	117.075
36794	5.1825	543.077	356.192
36795	5.1825	453.726	51.5714
36796	5.1827	508.093	31.3294
36797	5.1828	481.67	293.282
36798	5.183	480.975	40.4265
36799	5.1831	435.836	94.1217
36800	5.1832	503.61	91.9686
36801	5.1832	433.353	58.2324
36802	5.1833	465.104	153.937
36803	5.1833	276.48	111.149
36804	5.1834	535.96	88.9075
36805	5.1834	382.044	192.171

36806	5.1835	488.426	16.6474
36807	5.1837	492.912	17.6102
36808	5.1837	481.184	77.6584
36809	5.1838	648.491	25.7514
36810	5.1839	390.935	232.655
36811	5.184	352.082	163.201
36812	5.1846	442.258	52.7049
36813	5.1847	466.673	170.454
36814	5.1847	419.577	193.52
36815	5.1847	501.24	0.6529
36816	5.1848	437.841	84.8752
36817	5.1849	360.451	100.816
36818	5.185	416.23	236.205
36819	5.185	490.356	52.6459
36820	5.1853	373.413	173.941
36821	5.1853	430.376	186.718
36822	5.1854	441.407	176.166
36823	5.1855	361.228	52.9369
36824	5.1855	320.773	151.754
36825	5.1859	426.688	253.079
36826	5.186	516.383	267.842
36827	5.1861	448.614	96.0288
36828	5.1862	531.37	20.3324
36829	5.1863	499.393	79.2703
36830	5.1864	449.545	171.868
36831	5.1865	426.699	297.561
36832	5.1865	452.913	124.233
36833	5.1865	469.513	101.762
36834	5.1865	458.961	126.311
36835	5.187	395.229	148.514
36836	5.1871	492.032	126.572
36837	5.1871	472.855	110.591
36838	5.1872	453.035	86.9034
36839	5.1874	479.648	116.809
36840	5.1874	458.806	116.476
36841	5.1875	558.66	82.5405
36842	5.1875	422.855	279.674
36843	5.1875	414.261	227.802
36844	5.1876	471.276	70.646
36845	5.1878	530.246	287.914
36846	5.1878	503.057	180.465
36847	5.1881	351.441	17.8686
36848	5.1881	476.635	140.191
36849	5.1881	498.401	114.076
36850	5.1882	438.544	133.638
36851	5.1884	473.626	201.223
36852	5.1885	509.629	189.796
36853	5.1887	457.757	183.938

36854	5.1887	442.755	114.396
36855	5.1888	445.119	302.376
36856	5.189	481.469	51.9071
36857	5.1891	398.279	224.604
36858	5.1893	446.503	126.376
36859	5.1894	455.726	114.834
36860	5.1894	433.213	54.7423
36861	5.1895	522.01	100.013
36862	5.1895	509.678	206.665
36863	5.1895	388.345	48.4771
36864	5.1896	603.718	49.4159
36865	5.1896	363.811	102.241
36866	5.1896	379.005	223.496
36867	5.1896	486.761	105.874
36868	5.1898	515.12	62.7364
36869	5.1898	366.525	64.8912
36870	5.1899	486.295	89.5297
36871	5.1899	382.481	46.6853
36872	5.19	453.588	155.871
36873	5.19	483.21	76.2908
36874	5.19	521.03	31.6909
36875	5.1901	530.37	1.5081
36876	5.1904	410.483	175.329
36877	5.1905	432.664	126.333
36878	5.1905	455.056	120.664
36879	5.1906	340.737	181.152
36880	5.1907	435.233	218.778
36881	5.1908	441.608	81.4849
36882	5.1909	486.691	59.2689
36883	5.191	497.616	24.7027
36884	5.191	444.305	168.01
36885	5.1912	467.167	135.493
36886	5.1912	467.147	168.913
36887	5.1913	358.041	188.917
36888	5.1913	453.089	20.3955
36889	5.1914	333.784	24.6138
36890	5.1914	404.646	122.525
36891	5.1914	424.721	42.084
36892	5.1914	474.833	73.9454
36893	5.1917	518.181	61.4748
36894	5.1917	480.243	199.61
36895	5.192	367.297	133.549
36896	5.1922	412.798	126.288
36897	5.1922	415.281	220.624
36898	5.1922	504.449	23.9523
36899	5.1922	641.702	81.2671
36900	5.1923	534.691	258.224
36901	5.1923	393.577	174.916

36902	5.1926	465.681	140.834
36903	5.1928	550.693	259.322
36904	5.1928	477.246	110.85
36905	5.1929	412.953	232.173
36906	5.1932	480.178	78.7632
36907	5.1933	467.249	122.274
36908	5.1934	476.084	125.733
36909	5.1935	529.838	111.955
36910	5.1935	523.975	15.7499
36911	5.1937	443.837	118.618
36912	5.1937	413.096	101.145
36913	5.1937	468.677	125.54
36914	5.194	552.109	329.145
36915	5.1941	445.176	172.847
36916	5.1941	465.501	142.893
36917	5.1942	509.733	64.7487
36918	5.1943	417.504	108.024
36919	5.1944	379.743	203.108
36920	5.1948	525.827	59.1104
36921	5.1948	437.018	165.345
36922	5.1948	408.052	71.7089
36923	5.1948	476.81	102.821
36924	5.1949	443.251	182.284
36925	5.195	502.331	226.229
36926	5.195	527.39	355.302
36927	5.1951	542.96	77.7146
36928	5.1953	502.409	75.5212
36929	5.1954	539.578	29.6764
36930	5.1954	445.395	160.318
36931	5.1957	474.748	189.914
36932	5.196	533.164	69.3695
36933	5.196	408.114	263.14
36934	5.1962	477.923	72.3399
36935	5.1963	362.823	115.246
36936	5.1964	379.765	64.0464
36937	5.1964	437.882	189.99
36938	5.1965	469.015	340.033
36939	5.1965	479.948	48.0899
36940	5.1967	479.818	75.6368
36941	5.1968	460.837	238.554
36942	5.1969	467.809	105.996
36943	5.197	402.082	92.6684
36944	5.1971	465.811	146.317
36945	5.1971	440.976	101.566
36946	5.1971	338.754	189.8
36947	5.1974	505.136	233.817
36948	5.1976	162.433	119.062
36949	5.1976	399.903	80.7067

36950	5.1977	434.293	162.543
36951	5.1978	458.092	130.679
36952	5.1979	430.793	193.572
36953	5.1981	408.348	114.757
36954	5.1982	450.611	78.9093
36955	5.1985	375.242	223.068
36956	5.1987	525.684	269.755
36957	5.1987	516.536	269.86
36958	5.1988	503.868	94.372
36959	5.1989	456.293	316.393
36960	5.199	394.17	304.569
36961	5.1993	494.211	117.716
36962	5.1995	386.828	174.682
36963	5.1995	495.803	98.9746
36964	5.1996	552.664	47.8989
36965	5.1996	465.514	119.139
36966	5.1998	335.546	242.823
36967	5.2	450.579	125.35
36968	5.2	449.374	16.6043
36969	5.2	461.882	137.429
36970	5.2	471.288	147.423
36971	5.2001	499.436	271.043
36972	5.2001	424.371	282.035
36973	5.2003	430.544	100.196
36974	5.2003	512.201	103.049
36975	5.2006	448.997	118.685
36976	5.2006	418.776	219.799
36977	5.2009	433.716	44.3325
36978	5.201	459.827	123.723
36979	5.201	487.744	146.718
36980	5.201	498.498	331.389
36981	5.2011	424.859	13.4883
36982	5.2011	513.494	358.375
36983	5.2013	447.476	115.345
36984	5.2014	404.498	139.34
36985	5.2014	494.142	116.544
36986	5.2017	560.118	37.72
36987	5.2017	441.152	305.693
36988	5.2017	470.9	115.604
36989	5.2017	416.439	142.101
36990	5.2018	442.826	101.574
36991	5.2019	417.495	187.365
36992	5.202	502.332	48.517
36993	5.202	469.673	65.0139
36994	5.202	426.891	79.4542
36995	5.2021	478.353	91.4655
36996	5.2021	491.487	116.559
36997	5.2024	504.399	278.969

36998	5.2024	583.973	63.8077
36999	5.2024	396.536	133.642
37000	5.2024	426.121	98.6282
37001	5.2024	427.547	103.993
37002	5.2025	448.171	41.169
37003	5.2026	449.622	136.976
37004	5.2026	459.891	68.9996
37005	5.2028	501.766	183.492
37006	5.2028	524.054	112.452
37007	5.2029	510.435	102.499
37008	5.203	448.473	168.991
37009	5.2031	492.983	41.3369
37010	5.2032	403.847	248.874
37011	5.2033	307.639	184.542
37012	5.2034	485.985	195.097
37013	5.2034	492.089	31.8025
37014	5.2034	429.775	165.614
37015	5.2035	496.813	110.302
37016	5.2036	407.808	106.19
37017	5.2038	448.674	135.283
37018	5.2043	-2.8093	254.894
37019	5.2043	434.652	172.247
37020	5.2044	534.846	74.6334
37021	5.2044	484.274	82.9355
37022	5.2044	482.247	177.891
37023	5.2045	424.138	188.323
37024	5.2045	436.45	13.4834
37025	5.2045	433.349	204.144
37026	5.2046	461.596	121.569
37027	5.2046	406.106	246.623
37028	5.2046	423.31	159.044
37029	5.2046	481.615	231.361
37030	5.2047	508.845	75.1777
37031	5.205	470.152	133.598
37032	5.2051	421.5	177.48
37033	5.2054	520.953	145.841
37034	5.2054	500.22	98.615
37035	5.2054	446.795	139.792
37036	5.2054	451.889	108.522
37037	5.2055	472.726	83.177
37038	5.2056	480.3	186.833
37039	5.2058	420.745	236.222
37040	5.206	444.652	132.252
37041	5.2061	550.498	82.6149
37042	5.2061	478.347	64.0775
37043	5.2062	406.483	42.4705
37044	5.2063	451.779	216.951
37045	5.2063	410.413	99.8283

37046	5.2063	478.564	6.8685
37047	5.2064	464.973	145.39
37048	5.2066	448.894	180.045
37049	5.2066	451.58	135.516
37050	5.2066	430.657	115.785
37051	5.2066	393.422	110.939
37052	5.2067	421.168	122.9
37053	5.2068	176.153	30.7734
37054	5.2068	498.902	148.471
37055	5.2071	520.336	41.5257
37056	5.2073	496.398	90.366
37057	5.2073	476.676	167.573
37058	5.2075	397.503	128.529
37059	5.2075	380.395	154.27
37060	5.2076	477.699	141.464
37061	5.2076	603.65	83.6421
37062	5.2077	453.607	323.761
37063	5.2079	480.845	211.344
37064	5.208	491.369	49.32
37065	5.2082	526.142	225.805
37066	5.2083	471.455	89.4901
37067	5.2084	604.471	241.643
37068	5.209	425.056	46.2216
37069	5.2091	547.75	73.956
37070	5.2091	493.856	291.854
37071	5.2092	441.424	87.841
37072	5.2093	410.663	147.46
37073	5.2094	523.317	45.4368
37074	5.2095	531.322	21.1189
37075	5.2096	439.024	111.625
37076	5.2096	553.746	28.1403
37077	5.2097	406.364	111.256
37078	5.2098	438.303	101.033
37079	5.21	497.927	22.4589
37080	5.2101	419.55	120.312
37081	5.2103	398.814	132.685
37082	5.2104	557.303	20.599
37083	5.2107	564.559	56.0692
37084	5.2108	610.879	73.9917
37085	5.2108	415.546	134.766
37086	5.2108	429.342	103.452
37087	5.2109	380.688	294.603
37088	5.2112	445.455	176.083
37089	5.2114	429.281	283.444
37090	5.2116	469.142	210.25
37091	5.2118	532.192	124.43
37092	5.2119	449.325	218.473
37093	5.2119	463.82	165.897

37094	5.2119	526.457	51.0818
37095	5.2121	371.676	290.114
37096	5.2122	448.223	39.146
37097	5.2123	451.341	276.725
37098	5.2124	496.625	70.1646
37099	5.2125	523.143	102.573
37100	5.2127	430.211	179.799
37101	5.2128	513.384	132.716
37102	5.2133	506.104	73.8007
37103	5.2133	454.65	31.4835
37104	5.2135	438.728	257.189
37105	5.2137	558.142	52.8971
37106	5.2138	471.769	54.1648
37107	5.2139	424.842	155.752
37108	5.214	497.025	101.762
37109	5.2141	476.572	80.3163
37110	5.2146	449.32	281.763
37111	5.2147	465.755	139.163
37112	5.2147	438.594	165.724
37113	5.2148	502.352	118.571
37114	5.2149	572.363	52.4164
37115	5.2149	508.481	49.3018
37116	5.2149	544.382	312.36
37117	5.2155	445.528	28.4558
37118	5.2158	-2.7847	79.5574
37119	5.2159	524.858	99.4291
37120	5.2159	503.368	69.221
37121	5.2161	497.261	271.577
37122	5.2161	415.587	93.1802
37123	5.2162	484.476	181.085
37124	5.2166	454.103	132.496
37125	5.2167	423.694	329.518
37126	5.2168	459.843	261.656
37127	5.2168	466.629	244.052
37128	5.2169	477.835	210.378
37129	5.2173	443.81	115.901
37130	5.2174	546.733	114.154
37131	5.2174	503.623	116.289
37132	5.2174	579.217	357.502
37133	5.2175	548.669	44.7412
37134	5.2175	442.473	106.326
37135	5.2176	473.273	253.616
37136	5.2176	396.796	140.526
37137	5.2177	470.09	139.699
37138	5.2178	409.859	83.4242
37139	5.2179	392.443	86.6726
37140	5.2179	421.053	94.1656
37141	5.218	394.351	132.053

37142	5.2181	485.653	124.155
37143	5.2182	460.819	282.492
37144	5.2184	464.241	322.292
37145	5.2184	519.729	116.395
37146	5.2185	519.579	2.522
37147	5.2185	441.976	91.8121
37148	5.2187	514.019	28.1006
37149	5.2188	495.034	94.1911
37150	5.2192	456.327	119.949
37151	5.2192	462.775	127.674
37152	5.2193	477.416	175.094
37153	5.2193	476.315	357.3
37154	5.2198	473.852	47.541
37155	5.2199	437.861	171.73
37156	5.2199	481.816	96.5575
37157	5.2199	496.795	110.423
37158	5.22	482.035	105.49
37159	5.2201	516.602	322.827
37160	5.2201	444.829	215.45
37161	5.2201	394.455	245.304
37162	5.2201	398.19	97.8015
37163	5.2202	606.539	97.7497
37164	5.2202	408.547	149.316
37165	5.2203	429.583	121.705
37166	5.2204	399.344	193.918
37167	5.2205	526.815	270.037
37168	5.2205	465.068	148.031
37169	5.2207	466.679	109.984
37170	5.2208	419.186	232.48
37171	5.2208	485.503	120.799
37172	5.221	490.309	14.6046
37173	5.2212	467.81	115.579
37174	5.2213	409.854	162.62
37175	5.2214	477.747	111.491
37176	5.2214	563.545	307.645
37177	5.2214	506.156	298.961
37178	5.2215	490.373	172.553
37179	5.2215	435.801	180.257
37180	5.2215	432.533	129.961
37181	5.2216	534.124	122.319
37182	5.2217	394.226	269.022
37183	5.222	428.392	194.468
37184	5.2221	498.533	264.746
37185	5.2222	550.673	48.3224
37186	5.2224	478.309	231.223
37187	5.2225	501.212	58.3976
37188	5.2226	515.567	92.978
37189	5.2226	409.909	72.8859

37190	5.2228	446.761	56.4259
37191	5.2228	464.853	162.113
37192	5.223	465.223	5.0294
37193	5.2231	499.585	49.6575
37194	5.2231	482.504	83.8453
37195	5.2232	372.536	152.584
37196	5.2232	473.028	91.1282
37197	5.2234	426.263	106.987
37198	5.2234	386.807	148.641
37199	5.2234	461.648	126.603
37200	5.2234	457.687	159.075
37201	5.2235	380.786	284.896
37202	5.2235	460.755	232.852
37203	5.2236	410.951	175.843
37204	5.2236	467.452	156.601
37205	5.2236	544.878	125.256
37206	5.2237	452.241	73.6847
37207	5.2238	414.57	17.1106
37208	5.2239	503.312	78.6024
37209	5.2239	394.019	183.882
37210	5.224	457.902	74.4395
37211	5.2241	443.199	124.252
37212	5.2245	-8.393	53.0499
37213	5.2245	518.431	29.0848
37214	5.2246	459.519	26.1637
37215	5.2248	441.52	142.511
37216	5.2248	495.951	112.423
37217	5.2249	433.356	166.505
37218	5.2249	560.2	52.8391
37219	5.225	187.996	32.4715
37220	5.2251	460.416	69.7789
37221	5.2251	413.768	35.9494
37222	5.2252	583.227	61.0548
37223	5.2253	470.117	121.803
37224	5.2254	506.155	303.231
37225	5.2256	431.952	60.4954
37226	5.2257	432.267	150.071
37227	5.2257	364.535	58.7954
37228	5.2258	438.283	224.129
37229	5.2258	545.815	72.3325
37230	5.2258	401.167	166.805
37231	5.2258	477.467	76.9003
37232	5.2259	465.327	38.5835
37233	5.2259	378.245	137.271
37234	5.2259	425.885	107.092
37235	5.226	452.094	89.3835
37236	5.2262	448.746	165.403
37237	5.2264	436.429	135.816

37238	5.2265	484.355	27.8665
37239	5.2266	472.394	223.594
37240	5.2269	472.864	300.071
37241	5.227	539.23	85.7367
37242	5.2271	480.239	216.469
37243	5.2271	472.41	114.186
37244	5.2271	434.791	131.475
37245	5.2272	508.953	271.251
37246	5.2273	487.889	231.466
37247	5.2273	504.844	87.0227
37248	5.2273	451.99	97.988
37249	5.2274	429.574	334.555
37250	5.2275	449.383	255.905
37251	5.2275	456.508	282.162
37252	5.2276	406.422	172.271
37253	5.2277	446.596	35.666
37254	5.2279	456.88	265.35
37255	5.2279	423.732	27.0507
37256	5.2281	445.623	80.1256
37257	5.2281	373.134	127.027
37258	5.2281	494.901	85.6277
37259	5.2282	548.685	115.355
37260	5.2282	538.248	95.2598
37261	5.2283	479.877	268.88
37262	5.2283	436.704	99.1954
37263	5.2284	479.661	41.2071
37264	5.2285	566.673	23.7146
37265	5.2286	493.578	122.173
37266	5.2288	507.152	70.9838
37267	5.2289	507.729	63.9448
37268	5.2289	321.679	181.551
37269	5.229	454.465	217.605
37270	5.2291	509.748	128.086
37271	5.2293	431.042	329.627
37272	5.2293	429.523	314.374
37273	5.2294	522.359	16.6704
37274	5.2296	455.336	109.992
37275	5.2296	365.132	100.014
37276	5.2297	427.349	60.5023
37277	5.2298	549.798	110.364
37278	5.2298	385.257	281.464
37279	5.23	424.512	254.711
37280	5.2301	415.048	144.855
37281	5.2301	386.084	171.696
37282	5.2302	415.888	183.927
37283	5.2302	512.46	359.937
37284	5.2302	449.239	277.988
37285	5.2302	535.072	73.2169

37286	5.2304	507.968	28.5717
37287	5.2305	599.862	69.2842
37288	5.2305	475.639	70.9667
37289	5.2305	574.059	27.2355
37290	5.2306	449.382	248.995
37291	5.2306	420.937	184.676
37292	5.2307	512.692	40.8003
37293	5.2308	405.5	161.044
37294	5.231	491.034	117.904
37295	5.2313	400.964	179.679
37296	5.2314	475.411	130.712
37297	5.2316	473.599	122.663
37298	5.2316	507.302	338.831
37299	5.2317	505.389	131.501
37300	5.2318	497.52	100.965
37301	5.2318	517.175	93.9363
37302	5.2319	434.409	157.95
37303	5.2319	519.367	69.1049
37304	5.2319	476.04	119.617
37305	5.2321	514.672	73.2777
37306	5.2321	490.009	47.7124
37307	5.2323	419.529	97.2133
37308	5.2324	484.53	112.146
37309	5.2326	424.856	221.157
37310	5.233	455.192	119.407
37311	5.233	504.304	129.625
37312	5.2331	480.917	117.779
37313	5.2332	437.518	305.996
37314	5.2333	435.442	183.03
37315	5.2334	568.392	206.914
37316	5.2336	312.611	138.675
37317	5.2336	562.685	57.6428
37318	5.2337	465.159	257.559
37319	5.2338	417.543	166.279
37320	5.2341	541.406	125.531
37321	5.2341	535.217	321.961
37322	5.2343	507.245	35.1605
37323	5.2343	434.559	106.414
37324	5.2343	317.451	181.619
37325	5.2346	510.486	60.819
37326	5.2346	495.287	71.7359
37327	5.2349	487.126	110.087
37328	5.2349	550.231	26.9463
37329	5.235	517.081	0.6562
37330	5.2352	477.139	71.324
37331	5.2353	496.392	219.526
37332	5.2354	488.648	282.065
37333	5.2354	394.71	233.778

37334	5.2355	476.878	40.9552
37335	5.2357	555.874	86.7509
37336	5.2357	388.955	244.192
37337	5.2357	568.041	201.477
37338	5.236	377.327	183.121
37339	5.2361	471.388	18.4016
37340	5.2361	399.956	77.9481
37341	5.2361	477.276	12.4439
37342	5.2362	444.553	60.398
37343	5.2364	451.221	128.241
37344	5.2365	477.859	84.2373
37345	5.2367	444.222	300.908
37346	5.2368	461.515	162.939
37347	5.2368	484.735	164.457
37348	5.2369	480.438	6.3018
37349	5.2369	352.553	176.351
37350	5.237	385.516	192.169
37351	5.2371	476.64	145.701
37352	5.2372	430.438	204.898
37353	5.2373	477.396	184.134
37354	5.2373	431.164	172.446
37355	5.2373	528.241	85.8388
37356	5.2374	583.684	69.3209
37357	5.2374	395.074	87.8119
37358	5.2375	513.575	52.0814
37359	5.2377	428.84	221.133
37360	5.2379	461.246	156.865
37361	5.238	496.952	220.965
37362	5.2381	436.609	337.667
37363	5.2382	445.748	151.939
37364	5.2384	522.802	35.5944
37365	5.2384	444.211	83.0462
37366	5.2387	558.537	206.196
37367	5.2388	441.836	135.743
37368	5.2388	420.1	20.7566
37369	5.2389	482.532	119.152
37370	5.2393	422.147	74.4502
37371	5.2394	424.793	0.7588
37372	5.2395	416.159	257.726
37373	5.2395	432.082	120.645
37374	5.2396	511.554	11.8751
37375	5.2396	454.188	170.992
37376	5.2397	529.231	23.6215
37377	5.2397	502.59	215.636
37378	5.2398	423.993	130.632
37379	5.2399	463.313	317.857
37380	5.2399	450.095	323.811
37381	5.2401	496.489	192.161

37382	5.2401	496.241	108.726
37383	5.2402	475.712	98.2299
37384	5.2404	580.297	109.576
37385	5.2404	400.511	96.8006
37386	5.2404	453.15	8.3207
37387	5.2405	504.831	125.653
37388	5.2407	571.038	55.3148
37389	5.2408	410.741	262.324
37390	5.2409	514.497	96.3408
37391	5.2409	498.462	119.635
37392	5.2409	423.91	9.5826
37393	5.2409	509.442	94.6531
37394	5.241	460.44	124.776
37395	5.241	24.9552	33.127
37396	5.2412	445.118	177.579
37397	5.2412	473.529	169.178
37398	5.2412	371.123	146.372
37399	5.2412	520.193	55.7479
37400	5.2413	557.918	61.2187
37401	5.2413	429.246	45.5138
37402	5.2413	324.588	53.8851
37403	5.2413	440.091	338.022
37404	5.2415	441.032	355.163
37405	5.2416	531.373	81.9443
37406	5.2417	385.514	153.569
37407	5.2418	457.775	328.034
37408	5.2419	516.44	58.5155
37409	5.242	567.185	27.4142
37410	5.2422	553.797	25.5267
37411	5.2423	499.487	143.393
37412	5.2424	526.524	172.548
37413	5.2425	549.362	16.7369
37414	5.2425	445.433	120.305
37415	5.2426	477.8	68.9985
37416	5.2427	399.899	224.648
37417	5.2428	418.002	229.872
37418	5.243	407.724	187.346
37419	5.243	434.824	85.8173
37420	5.243	515.728	39.1904
37421	5.2438	352.733	211.867
37422	5.2438	491.617	73.0644
37423	5.2439	469.836	265.051
37424	5.2445	581.002	11.9382
37425	5.2446	539.535	40.7711
37426	5.2446	440.941	265.735
37427	5.2446	502.699	329.674
37428	5.2452	442.376	286.939
37429	5.2454	502.166	321.545

37430	5.2455	474.62	103.569
37431	5.2458	465.511	95.9388
37432	5.2459	543.212	194.923
37433	5.2459	462.608	134.619
37434	5.2462	546.178	226.235
37435	5.2462	460.695	57.4315
37436	5.2463	411.285	141.771
37437	5.2463	499.344	100.547
37438	5.2463	546.108	67.5659
37439	5.2465	460.802	20.3971
37440	5.2466	459.321	242.909
37441	5.2467	290.359	210.547
37442	5.2468	416.227	256.968
37443	5.2469	393.648	127.192
37444	5.247	487.904	128.241
37445	5.2471	484.297	129.469
37446	5.2472	437.559	196.862
37447	5.2472	375.061	174.738
37448	5.2475	429.291	117.102
37449	5.2475	556.223	39.5572
37450	5.2476	395.83	152.56
37451	5.2477	388.124	133.028
37452	5.2478	431.386	170.916
37453	5.2481	516.495	17.5391
37454	5.2482	503.779	73.7026
37455	5.2482	470.781	144.429
37456	5.2482	463.613	48.7473
37457	5.2483	500.831	108.353
37458	5.2483	505.644	234.489
37459	5.2487	451.314	190.549
37460	5.2488	493.553	42.8581
37461	5.2489	488.352	25.083
37462	5.249	420.295	131.845
37463	5.2491	570.188	242.348
37464	5.2493	435.689	41.4477
37465	5.2494	436.484	195.038
37466	5.2495	497.925	92.6895
37467	5.2495	546.189	19.6133
37468	5.2496	477.549	248.267
37469	5.2497	429.213	186.707
37470	5.2498	575.221	278.483
37471	5.2498	497.248	156.706
37472	5.2498	501.975	90.4763
37473	5.2499	451.517	137.98
37474	5.25	510.298	178.886
37475	5.2501	465.416	272.419
37476	5.2501	452.876	194.431
37477	5.2504	465.276	129.489

37478	5.2505	454.643	118.403
37479	5.2506	526.025	26.4163
37480	5.2506	519.757	28.0562
37481	5.2508	498.627	274.344
37482	5.2511	529.25	313.326
37483	5.2511	451.096	168.644
37484	5.2513	372.55	223.832
37485	5.2513	409.294	117.568
37486	5.2514	422.842	120.567
37487	5.2515	557.502	21.5154
37488	5.2518	537.835	53.9605
37489	5.2518	501.337	38.02
37490	5.2519	503.603	83.3727
37491	5.2519	444.366	165.254
37492	5.2521	475.996	213.984
37493	5.2523	363.114	234.239
37494	5.2525	497.587	6.234
37495	5.2526	630.411	26.4214
37496	5.2526	479.736	48.8571
37497	5.2527	491.685	81.6095
37498	5.2527	526.884	56.8023
37499	5.2528	470.102	50.6124
37500	5.253	472.72	131.43
37501	5.2531	516.218	123.153
37502	5.2531	569.097	55.88
37503	5.2531	538.727	18.5901
37504	5.2532	409.808	274.974
37505	5.2536	516.68	185.991
37506	5.2537	499.792	107.681
37507	5.2537	511.468	96.2731
37508	5.2537	489.623	177.028
37509	5.2538	379.297	220.061
37510	5.254	409.4	65.8895
37511	5.2543	448.074	129.672
37512	5.2543	460.576	110.337
37513	5.2543	530.638	187.567
37514	5.2545	405.608	170.188
37515	5.2545	459.19	327.825
37516	5.2545	486.123	205.431
37517	5.2546	520.8	301.886
37518	5.2547	474.714	31.0738
37519	5.2549	428.408	144.699
37520	5.2549	450.177	296.661
37521	5.2549	451.004	267.418
37522	5.2549	349.757	180.373
37523	5.2549	550.5	30.13
37524	5.2554	431.008	330.713
37525	5.2554	470.693	282.812

37526	5.2556	466.95	52.5334
37527	5.2558	498.808	173.137
37528	5.2558	453.557	52.6405
37529	5.2558	556.517	115.806
37530	5.2559	580.912	47.9341
37531	5.256	504.34	46.6901
37532	5.256	462.393	71.6541
37533	5.2561	485.681	319.371
37534	5.2561	447.262	124.088
37535	5.2562	516.421	174.774
37536	5.2562	417.18	181.457
37537	5.2563	425.572	177.076
37538	5.2565	487.453	53.4994
37539	5.2566	469.763	152.17
37540	5.2566	477.117	132.719
37541	5.2568	440.45	92.4054
37542	5.2568	480.707	80.0186
37543	5.2569	474.024	323.129
37544	5.257	524.05	287.328
37545	5.2571	378.594	222.271
37546	5.2571	451.734	285.53
37547	5.2572	532.628	70.0067
37548	5.2572	389.874	116.427
37549	5.2573	404.669	149.758
37550	5.2575	437.632	73.5203
37551	5.2575	485.887	177.971
37552	5.2575	433.277	84.7152
37553	5.258	517.407	174.634
37554	5.258	374.728	74.4814
37555	5.2581	511.936	121.999
37556	5.2583	463.452	101.973
37557	5.2584	518.401	157.185
37558	5.2584	455.924	196
37559	5.2586	546.133	205.411
37560	5.2591	450.46	84.778
37561	5.2593	518.732	29.8619
37562	5.2594	486.68	297.3
37563	5.2595	461.714	348.19
37564	5.2598	474.282	142.012
37565	5.2599	486.163	188.588
37566	5.2599	469.449	237.933
37567	5.2599	515.493	58.2749
37568	5.2599	546.809	73.3642
37569	5.2602	492.937	175.367
37570	5.2602	444.737	296.357
37571	5.2603	410.618	137.454
37572	5.2603	407.283	119.513
37573	5.2604	-2.853	93.6462

37574	5.2604	594.888	41.864
37575	5.2606	472.828	241.794
37576	5.2606	513.146	86.7106
37577	5.2607	475.715	224.028
37578	5.2607	441.567	192.59
37579	5.2607	392.975	286.097
37580	5.2608	387.143	122.491
37581	5.261	595.453	40.1639
37582	5.2612	460.659	129.249
37583	5.2612	431.767	123.51
37584	5.2613	486.719	133.733
37585	5.2613	456.789	60.994
37586	5.2613	553.086	13.7837
37587	5.2613	540.763	345.122
37588	5.2617	449.571	99.208
37589	5.2618	410.383	136.103
37590	5.262	498.978	283.923
37591	5.2621	374.313	239.834
37592	5.2623	427.378	79.2874
37593	5.2623	509.274	109.605
37594	5.2626	515.687	169.383
37595	5.2627	410.725	194.194
37596	5.2627	477.66	9.6822
37597	5.263	459.875	51.0983
37598	5.263	620.956	14.1676
37599	5.2631	481.13	157.746
37600	5.2633	576.551	49.2129
37601	5.2635	508.691	147.81
37602	5.2635	396.635	138.214
37603	5.264	402.667	184.717
37604	5.2641	484.305	99.6101
37605	5.2642	497.088	84.347
37606	5.2642	423.991	165.333
37607	5.2645	535.779	229.445
37608	5.2645	467.702	265.804
37609	5.2645	463.788	118.626
37610	5.2646	444.353	94.6985
37611	5.2649	457.172	127.576
37612	5.2649	587.251	7.0858
37613	5.265	406.099	338.197
37614	5.265	452.544	76.2156
37615	5.2651	511.641	230.708
37616	5.2653	402.823	230.188
37617	5.2656	541.947	105.326
37618	5.2657	-4.1427	309.559
37619	5.2657	471.735	252.069
37620	5.2657	428.117	182.931
37621	5.2658	608.62	51.6063

37622	5.266	554.532	300.581
37623	5.266	544.999	76.7597
37624	5.266	520.239	198.841
37625	5.2661	477.561	281.363
37626	5.2665	532.826	57.387
37627	5.2665	594.413	230.327
37628	5.2666	457.313	93.6197
37629	5.2666	537.524	90.7401
37630	5.2668	534.598	62.4081
37631	5.2669	624.522	77.7752
37632	5.2669	594.521	24.0822
37633	5.267	526.238	350.976
37634	5.267	482.404	144.771
37635	5.267	506.27	25.2704
37636	5.2671	416.038	231.328
37637	5.2671	399.758	221.029
37638	5.2673	532.197	113.914
37639	5.2676	472.051	129.144
37640	5.2678	445.062	170.073
37641	5.2678	423.637	357.997
37642	5.268	432.228	8.788
37643	5.268	525.72	18.306
37644	5.2681	563.844	119.346
37645	5.2682	555.731	118.89
37646	5.2683	532.688	125.164
37647	5.2684	447.285	35.3589
37648	5.2685	474.013	157.685
37649	5.2685	481.46	156.631
37650	5.2686	473.558	156.884
37651	5.2687	506.695	90.7403
37652	5.2687	460.026	105.57
37653	5.2688	461.213	103.156
37654	5.269	543.1	173.539
37655	5.2691	462.948	182.602
37656	5.2692	423.242	135.616
37657	5.2693	457.295	94.9964
37658	5.2693	494.226	126.478
37659	5.2694	470.219	71.218
37660	5.2697	517.585	83.5479
37661	5.2698	398.045	235.46
37662	5.2698	511.406	31.6964
37663	5.2698	577.426	28.2723
37664	5.2698	556.624	47.6925
37665	5.27	494.665	196.446
37666	5.2701	518.289	95.3318
37667	5.2701	595.903	67.336
37668	5.2702	505.917	67.6463
37669	5.2703	459.284	233.491

37670	5.2704	572.552	68.0261
37671	5.2705	483.787	168.251
37672	5.2707	468.053	253.396
37673	5.2709	537.754	61.1161
37674	5.271	435.987	262.898
37675	5.271	490.55	123.862
37676	5.271	537.093	296.72
37677	5.2711	543.908	356.045
37678	5.2712	413.241	221.211
37679	5.2712	502.979	178.282
37680	5.2715	497.008	64.7997
37681	5.2715	492.927	11.2171
37682	5.2715	567.798	206.054
37683	5.2716	498.228	322.615
37684	5.2716	477.189	123.516
37685	5.2716	483.905	219.044
37686	5.2717	490.345	73.4769
37687	5.2717	443.122	104.63
37688	5.2717	491.049	112.101
37689	5.2718	403.747	218.691
37690	5.272	446.628	93.8027
37691	5.2722	451.908	143.552
37692	5.2722	534.877	84.5351
37693	5.2722	549.995	15.8608
37694	5.2723	470.911	182.28
37695	5.2723	495.13	176.927
37696	5.2724	427.935	305.779
37697	5.2724	561.121	40.9238
37698	5.2724	568.536	100.7
37699	5.2727	508.979	296.948
37700	5.2727	477.455	189.67
37701	5.2728	473.946	314.355
37702	5.2732	489.85	116.376
37703	5.2734	656.309	47.1872
37704	5.2734	557.426	46.2944
37705	5.2735	510.771	24.6456
37706	5.2735	521.356	177.926
37707	5.2735	504.432	118.096
37708	5.2736	573.389	18.5317
37709	5.2738	557.742	299.525
37710	5.274	415.579	145.753
37711	5.2741	475.121	135.567
37712	5.2741	100.088	95.3108
37713	5.2742	454.242	84.8728
37714	5.2744	543.854	61.3187
37715	5.2744	518.687	142.143
37716	5.2745	546.392	77.8863
37717	5.2745	421.351	48.4258

37718	5.2746	524.281	60.4739
37719	5.2748	532.994	17.9531
37720	5.2749	441.824	300.421
37721	5.2749	447.124	121.165
37722	5.2749	434.009	257.069
37723	5.2749	454.297	81.9766
37724	5.275	469.464	96.1862
37725	5.2753	462.788	242.022
37726	5.2753	425.064	173.628
37727	5.2754	441.253	278.417
37728	5.2755	520.995	33.8876
37729	5.2757	550.027	267.903
37730	5.2757	475.841	306.135
37731	5.2757	607.586	242.319
37732	5.2758	461.958	75.7507
37733	5.2759	455.452	193.995
37734	5.2759	444.026	170.104
37735	5.2761	443.785	164.958
37736	5.2761	480.93	287.257
37737	5.2761	535.307	48.5331
37738	5.2763	405.229	167.846
37739	5.2763	633.062	208.763
37740	5.2765	486.118	66.3579
37741	5.2766	413.948	278.934
37742	5.2767	508.586	124.151
37743	5.2767	473.042	281.695
37744	5.2769	386.884	118.639
37745	5.2769	437.137	81.11
37746	5.2771	573.81	40.3766
37747	5.2773	445.789	234.102
37748	5.2775	535.013	59.2763
37749	5.2776	499	120.809
37750	5.2777	491.718	234.432
37751	5.2778	502.255	66.6367
37752	5.2779	501.571	106.705
37753	5.278	488.106	108.968
37754	5.278	585.129	101.705
37755	5.278	499.958	128.799
37756	5.278	504.176	26.5006
37757	5.2782	500.936	140.453
37758	5.2783	473.817	107.246
37759	5.2784	548.416	47.6238
37760	5.2787	548.069	116.412
37761	5.279	462.811	279.64
37762	5.2791	402.235	164.437
37763	5.2792	421.939	220.176
37764	5.2793	461.706	131.196
37765	5.2795	477.229	66.204

37766	5.2795	466.326	232.749
37767	5.2799	499.74	55.6065
37768	5.28	529.547	107.042
37769	5.28	488.622	44.3139
37770	5.28	551.837	46.6231
37771	5.2804	485.07	77.1541
37772	5.2805	440.785	187.055
37773	5.2805	420.04	338.744
37774	5.2805	490.263	26.7087
37775	5.2805	549.482	27.1361
37776	5.2807	611.471	54.9348
37777	5.2809	538.84	75.9473
37778	5.281	484.454	22.5748
37779	5.281	499.166	182.764
37780	5.2811	471.525	255.503
37781	5.2811	472.577	319.363
37782	5.2813	462.873	50.0181
37783	5.2813	516.261	151.061
37784	5.2815	264.147	205.428
37785	5.2815	489.476	118.509
37786	5.2818	515.416	103.119
37787	5.2819	426.579	80.2797
37788	5.2819	411.897	131.885
37789	5.2819	515.039	73.0981
37790	5.2819	484.712	54.1002
37791	5.282	564.871	189.215
37792	5.282	460.149	162.81
37793	5.282	601.974	288.959
37794	5.282	397.669	11.7099
37795	5.2821	426.117	251.028
37796	5.2821	552.748	113.355
37797	5.2822	456.233	222.443
37798	5.2822	432	335.016
37799	5.2824	563.291	318.829
37800	5.2826	449.48	133.042
37801	5.2828	464.184	183.829
37802	5.2829	448.219	132.412
37803	5.2834	488.812	213.705
37804	5.2835	484.161	292.027
37805	5.2836	449.343	93.9489
37806	5.2836	416.953	180.134
37807	5.2836	451.722	332.229
37808	5.2838	518.723	304.652
37809	5.2838	474.776	6.6978
37810	5.2839	475.595	153.808
37811	5.2839	463.582	252.153
37812	5.284	459.825	151.554
37813	5.2841	475.852	151.673

37814	5.2842	484.111	255.803
37815	5.2842	433.235	148.271
37816	5.2843	482.137	281.989
37817	5.2843	465.74	80.239
37818	5.2844	502.754	224.672
37819	5.2847	576.36	42.1448
37820	5.2847	526.603	121.197
37821	5.2847	393.395	109.802
37822	5.285	358.638	87.9366
37823	5.2853	445.002	220.141
37824	5.2856	515.277	341.823
37825	5.2857	503.137	128.263
37826	5.2858	437.315	116.588
37827	5.2858	478.282	115.774
37828	5.2859	551.149	67.7128
37829	5.2859	425.687	186.829
37830	5.2859	475.765	131.299
37831	5.286	480.9	142.499
37832	5.286	436.873	145.903
37833	5.286	412.281	114.275
37834	5.2866	514.71	176.235
37835	5.287	491.047	171.99
37836	5.287	428.139	119.43
37837	5.2871	409.087	147.017
37838	5.2871	438.413	90.9496
37839	5.2871	438.996	115.879
37840	5.2873	418.079	276.225
37841	5.2873	459.243	264.117
37842	5.2878	497.5	269.17
37843	5.2879	547.784	48.9283
37844	5.2882	465.566	269.233
37845	5.2882	457.66	179.517
37846	5.2883	524.399	108.296
37847	5.2883	431.292	184.836
37848	5.2883	411.165	40.0166
37849	5.2884	436.516	97.3938
37850	5.2884	200.73	225.635
37851	5.2884	581.213	39.4215
37852	5.2885	434.005	122.525
37853	5.2886	528.856	217.826
37854	5.2887	551.529	63.3102
37855	5.2888	502.376	60.2759
37856	5.2888	543.27	228.185
37857	5.289	485.484	55.229
37858	5.2891	449.22	143.039
37859	5.2891	461.572	233.968
37860	5.2893	480.209	219.452
37861	5.2894	491.995	296.146

37862	5.2896	539.176	340.9
37863	5.2896	431.377	327.146
37864	5.2896	435.894	114.639
37865	5.2898	468.135	123.792
37866	5.29	557.672	245.445
37867	5.29	481.822	234.512
37868	5.29	492.894	122.734
37869	5.29	467.901	144.639
37870	5.2901	447.809	155.353
37871	5.2904	514.864	110.643
37872	5.2905	473.887	123.643
37873	5.2905	479.137	277.881
37874	5.2906	499.325	266.049
37875	5.2906	455.177	112.688
37876	5.2906	466.697	42.7364
37877	5.2906	574.02	59.2774
37878	5.2909	412.316	77.7498
37879	5.2909	419.285	275.657
37880	5.291	541.219	73.8861
37881	5.2911	444.629	100.247
37882	5.2911	536.574	287.097
37883	5.2912	448.945	114.707
37884	5.2917	508.315	291.497
37885	5.2917	518.289	115.934
37886	5.2919	453.047	80.3757
37887	5.292	527.143	66.5733
37888	5.292	505.137	225.083
37889	5.292	554.361	52.6191
37890	5.292	452.024	2.2635
37891	5.2923	399.839	173.242
37892	5.2924	500.227	183.121
37893	5.2924	510.31	104.031
37894	5.2928	573.376	127.049
37895	5.2928	406.188	227.37
37896	5.2929	478.69	106.392
37897	5.293	457.278	85.1816
37898	5.2931	477.512	111.564
37899	5.2932	513.397	121.111
37900	5.2932	-2.7977	79.8658
37901	5.2932	620.257	84.3627
37902	5.2933	603.196	62.543
37903	5.2933	563.666	29.584
37904	5.2935	528.926	206.847
37905	5.2935	481.314	260.623
37906	5.2935	468.548	281.246
37907	5.2937	571.297	268.311
37908	5.2937	525.626	265.16
37909	5.2937	440.028	195.778

37910	5.2939	469.404	110.133
37911	5.294	424.421	151.649
37912	5.2941	468.514	126.603
37913	5.2944	479.791	61.5791
37914	5.2945	550.422	98.739
37915	5.2945	398.606	149.394
37916	5.2945	462.607	47.5141
37917	5.2946	566.691	14.359
37918	5.2946	489.212	135.073
37919	5.2947	461.767	80.6854
37920	5.2947	507.249	182.613
37921	5.2948	570.118	307.551
37922	5.2948	607.433	31.093
37923	5.2949	438.612	100.515
37924	5.2949	438.57	105.684
37925	5.295	441.001	96.0141
37926	5.295	415.466	118.68
37927	5.295	519.737	32.6649
37928	5.2951	443.813	179.706
37929	5.2953	556.95	34.1226
37930	5.2954	569.155	122.516
37931	5.2955	473.037	282.661
37932	5.2955	481.033	329.623
37933	5.2958	567.842	358.502
37934	5.2959	483.738	186.815
37935	5.2959	558.919	286.421
37936	5.296	639.695	51.2967
37937	5.296	513.99	105.817
37938	5.2962	451.427	187.424
37939	5.2962	532.711	42.8261
37940	5.2963	477.886	49.2056
37941	5.2963	485.416	331.208
37942	5.2965	430.967	160.989
37943	5.2969	526.955	155.062
37944	5.2971	532.484	291.715
37945	5.2971	582.622	95.2583
37946	5.2972	506.363	122.589
37947	5.2973	520.499	265.139
37948	5.2973	578.315	75.0856
37949	5.2973	456.097	183.132
37950	5.2974	-2.6867	6.6663
37951	5.2976	347.269	175.867
37952	5.2977	592.088	36.4721
37953	5.2978	450.39	174.064
37954	5.2981	460.084	157.019
37955	5.2981	545.117	77.2402
37956	5.2981	409.76	230.501
37957	5.2981	569.443	342.495

37958	5.2982	405.76	86.0772
37959	5.2985	569.592	94.6767
37960	5.2985	404.748	178.471
37961	5.2985	444.331	88.1231
37962	5.2986	456.281	51.4214
37963	5.2986	474.084	316.083
37964	5.2987	463.439	177.234
37965	5.2987	489.155	70.489
37966	5.2987	455.932	189.551
37967	5.2988	437.636	174.854
37968	5.2989	463.237	320.687
37969	5.2989	475.771	257.059
37970	5.2989	472.67	60.3722
37971	5.299	465.565	269.419
37972	5.299	497.851	274.033
37973	5.2991	543.953	76.0296
37974	5.2992	480.669	97.4212
37975	5.2995	359.653	96.0479
37976	5.2996	434.209	126.203
37977	5.2996	546.128	123.761
37978	5.2997	501.961	193.853
37979	5.2997	498.475	35.5009
37980	5.2998	513.445	230.702
37981	5.2998	447.285	276.267
37982	5.3001	536.713	146.756
37983	5.3003	495.136	97.1657
37984	5.3004	492.972	106.477
37985	5.3006	442.697	132.714
37986	5.3006	460.974	138.414
37987	5.3007	388.885	107.323
37988	5.3009	488.088	250.235
37989	5.301	476.31	233.859
37990	5.301	497.148	279.501
37991	5.301	418.869	131.261
37992	5.3015	538.094	75.5652
37993	5.3015	483.888	86.1034
37994	5.3017	542.696	73.5674
37995	5.3019	501.152	188.338
37996	5.3019	496.772	129.208
37997	5.3021	457.518	110.229
37998	5.3021	544.123	49.1678
37999	5.3022	569.955	288.051
38000	5.3022	484.39	183.757
38001	5.3023	509.514	280.963
38002	5.3023	482.207	66.0606
38003	5.3024	357.777	232.246
38004	5.3025	513.555	116.379
38005	5.3025	367.815	171.74

38006	5.3026	523.379	60.8108
38007	5.3026	532.53	294.864
38008	5.3026	434.372	139.776
38009	5.3026	442.032	134.56
38010	5.3029	536.764	94.661
38011	5.303	468.988	318.901
38012	5.3031	376.671	301.279
38013	5.3031	564.507	73.4571
38014	5.3034	497.812	233.627
38015	5.3035	522.211	79.2759
38016	5.3036	456.618	50.6903
38017	5.3038	456.727	18.5307
38018	5.3042	582.426	40.8295
38019	5.3043	541.367	319.933
38020	5.3043	384.487	175.121
38021	5.3045	457.642	121.893
38022	5.3045	467.573	81.5566
38023	5.3047	463.119	260.677
38024	5.3048	500.056	155.042
38025	5.3049	446.721	188.461
38026	5.3049	370.217	160.136
38027	5.3051	419.674	108.571
38028	5.3052	461.279	147.349
38029	5.3053	514.032	274.771
38030	5.3053	519.662	224.126
38031	5.3053	463.832	219.755
38032	5.3053	506.263	114.172
38033	5.3055	469.663	254.377
38034	5.3055	538.883	89.2245
38035	5.3057	505.377	108.897
38036	5.3057	528.188	118.554
38037	5.3059	518.17	222.46
38038	5.306	547.646	63.5115
38039	5.3062	492.432	32.6685
38040	5.3062	458.539	192.684
38041	5.3062	577.096	31.9867
38042	5.3063	485.548	192.274
38043	5.3064	452.172	192.67
38044	5.3064	470.404	158.556
38045	5.3065	435.288	194.04
38046	5.3065	524.223	72.3517
38047	5.3068	508.112	175.424
38048	5.3069	490.734	135.744
38049	5.3071	468.915	319.398
38050	5.3074	538.118	187.951
38051	5.3074	446.292	116.02
38052	5.3075	452.385	140.509
38053	5.3077	441.427	46.2709

38054	5.3078	516.061	232.538
38055	5.3079	426.699	192.585
38056	5.3081	526.444	209.944
38057	5.3082	451.728	166.495
38058	5.3082	442.039	261.836
38059	5.3083	510.367	117.415
38060	5.3084	500.662	188.253
38061	5.3084	510.834	98.0093
38062	5.3084	449.887	162.884
38063	5.3085	471.32	133.381
38064	5.3086	485.14	92.1001
38065	5.3086	384.822	60.186
38066	5.3087	477.005	114.168
38067	5.309	465.578	229.632
38068	5.309	517.15	101.815
38069	5.309	542.395	75.9132
38070	5.3091	526.409	60.4958
38071	5.3094	540.675	271.606
38072	5.3095	454.486	180.25
38073	5.3096	566.152	47.5318
38074	5.3096	504.201	204.226
38075	5.3097	408.982	104.231
38076	5.3097	536.788	247.674
38077	5.31	467.776	234.162
38078	5.3101	556.741	37.5431
38079	5.3101	655.762	330.199
38080	5.3102	480.974	133.943
38081	5.3107	516.156	100.194
38082	5.3108	537.503	351.155
38083	5.311	483.536	250.909
38084	5.3112	442.206	246.892
38085	5.3112	512.05	327.626
38086	5.3115	463.482	264.236
38087	5.3115	518.768	123.676
38088	5.3117	498.824	61.8341
38089	5.3119	556.178	188.506
38090	5.312	525.201	8.35
38091	5.3121	517.898	75.8099
38092	5.3121	535.943	57.1214
38093	5.3122	495.314	48.7304
38094	5.3123	384.838	117.803
38095	5.3126	385.356	198.257
38096	5.3129	460.043	93.4469
38097	5.313	465.636	169.971
38098	5.3131	506.198	217.017
38099	5.3132	479.446	215.93
38100	5.3132	470.336	148.762
38101	5.3135	501.875	140.097

38102	5.3135	519.847	206.264
38103	5.3135	478.709	123.266
38104	5.3136	545.972	197.576
38105	5.3138	534.298	37.4175
38106	5.3139	441.839	114.12
38107	5.3139	476.461	163.413
38108	5.314	649.043	207.606
38109	5.3141	567.467	310.185
38110	5.3141	592.672	67.017
38111	5.3142	617.172	61.2682
38112	5.3143	526.937	50.0107
38113	5.3143	530.805	343.247
38114	5.3144	536.716	295.975
38115	5.3144	550.192	330.932
38116	5.3145	590.757	72.428
38117	5.3148	441.018	245.072
38118	5.3148	433.004	169.746
38119	5.3149	598.923	307.962
38120	5.3149	-4.208	90.0162
38121	5.315	495.623	121.145
38122	5.3152	542.722	129.875
38123	5.3153	488.689	83.3867
38124	5.3154	458.525	227.18
38125	5.3154	493.996	70.8771
38126	5.3156	482.487	198.057
38127	5.3159	493.868	134.14
38128	5.3161	495.72	99.3497
38129	5.3161	516.029	102.608
38130	5.3163	436.37	273.512
38131	5.3163	474.476	47.3171
38132	5.3163	537.104	122.873
38133	5.3164	478.817	62.9828
38134	5.3168	519.501	140.421
38135	5.3169	451.935	118.131
38136	5.3169	431.591	75.9277
38137	5.317	512.993	78.7539
38138	5.3171	527.259	109.89
38139	5.3172	532.494	20.8183
38140	5.3172	447.12	94.8499
38141	5.3174	421.982	219.786
38142	5.3175	500.722	280.06
38143	5.3175	439.5	120.107
38144	5.3175	510.883	175.71
38145	5.3175	493.361	322.506
38146	5.3176	491.334	284.502
38147	5.3177	460.228	256.735
38148	5.3177	408.097	94.9669
38149	5.3179	517.918	275.41

38150	5.3179	521.099	167.773
38151	5.318	541.606	44.296
38152	5.3182	631.844	59.9191
38153	5.3182	458.618	143.101
38154	5.3182	526.773	118.655
38155	5.3182	552.743	0.1851
38156	5.3184	429.286	200.892
38157	5.3184	483.298	333.75
38158	5.3188	586.226	49.1998
38159	5.3188	618.3	23.751
38160	5.3188	560.755	158.223
38161	5.3192	464.625	157.427
38162	5.3194	488.349	254.339
38163	5.3194	519.927	17.5664
38164	5.3195	378.686	125.415
38165	5.3195	416.937	177.025
38166	5.3196	559.183	296.507
38167	5.3198	566.354	67.0984
38168	5.3198	438.965	79.5335
38169	5.3199	446.732	152.56
38170	5.32	376.421	219.479
38171	5.32	395.731	134.251
38172	5.32	498.316	8.3795
38173	5.3202	365.353	182.146
38174	5.3202	478.802	110.836
38175	5.3202	401.356	125.403
38176	5.3204	488.218	50.1087
38177	5.3205	500.751	64.8921
38178	5.3207	541.829	64.3254
38179	5.3207	468.232	189.305
38180	5.3207	539.807	300.333
38181	5.3208	533.274	132.232
38182	5.3212	428.717	153.825
38183	5.3213	489.11	75.6906
38184	5.3213	540.376	45.0638
38185	5.3214	560.32	80.6517
38186	5.3214	418.125	232.712
38187	5.3214	556.409	205.996
38188	5.3217	442.348	74.7899
38189	5.3219	494.784	173.807
38190	5.3219	622.183	46.063
38191	5.322	449.104	165.022
38192	5.3221	589.17	73.984
38193	5.3221	539.339	211.368
38194	5.3222	474.244	258.684
38195	5.3223	557.835	169.041
38196	5.3223	534.992	55.2667
38197	5.3225	503.475	203.466

38198	5.3225	447.127	167.433
38199	5.3225	479.195	20.1456
38200	5.3226	531.795	114.073
38201	5.3226	451.678	109.513
38202	5.3226	669.978	10.393
38203	5.3227	520.499	31.629
38204	5.3228	500.685	297.413
38205	5.323	458.724	64.4533
38206	5.3231	465.945	321.708
38207	5.3231	456.019	125.892
38208	5.3231	341.757	305.977
38209	5.3232	510.008	81.1168
38210	5.3232	455.455	326.815
38211	5.3233	439.335	359.567
38212	5.3235	498.774	245.508
38213	5.3235	438.864	123.514
38214	5.3236	554.653	70.0842
38215	5.3236	547.073	129.752
38216	5.3239	600.979	89.69
38217	5.3239	514.931	258.861
38218	5.3239	526.246	72.5747
38219	5.324	515.193	113.796
38220	5.324	500.846	181.023
38221	5.324	539.638	99.1739
38222	5.3241	495.452	67.0453
38223	5.3241	413.132	95.6761
38224	5.3241	440.7	87.7465
38225	5.3241	346.486	168.456
38226	5.3242	477.01	259.792
38227	5.3246	498.648	138.334
38228	5.3246	411.401	228.526
38229	5.3246	442.338	107.84
38230	5.3249	489.176	98.5963
38231	5.3249	425.501	171.046
38232	5.325	439.124	213.471
38233	5.325	472.53	93.566
38234	5.325	444.477	164.557
38235	5.325	472.474	149.477
38236	5.325	558.532	28.9545
38237	5.3251	550.888	3.511
38238	5.3252	558.482	160.152
38239	5.3253	500.727	164.882
38240	5.3254	463.451	143.505
38241	5.3254	366.3	325.43
38242	5.3255	455.684	167.175
38243	5.3255	545.73	37.6183
38244	5.3257	543.156	150.759
38245	5.3257	-2.9434	320.962

38246	5.3257	555.54	52.7773
38247	5.3258	537.689	26.0145
38248	5.326	446.157	186.132
38249	5.326	417.775	192.987
38250	5.3261	590.415	230.001
38251	5.3263	565.126	20.6798
38252	5.3264	483.258	214.159
38253	5.3264	552.472	13.4193
38254	5.3264	603.599	49.3237
38255	5.3266	473.591	121.713
38256	5.3266	458.827	116.258
38257	5.3267	490.285	136.011
38258	5.3269	595.542	35.6949
38259	5.327	417.676	18.8799
38260	5.3271	567.019	145.366
38261	5.3271	493.114	138.548
38262	5.3272	411.739	63.9143
38263	5.3274	508.241	39.1523
38264	5.3276	399.362	125.11
38265	5.3276	515.532	121.791
38266	5.3276	456.106	127.685
38267	5.3277	503.514	21.2613
38268	5.3279	511.266	106.873
38269	5.328	476.306	86.4854
38270	5.3281	427.937	221.27
38271	5.3282	504.064	216.691
38272	5.3284	423.501	271.044
38273	5.3285	487.538	332.615
38274	5.3286	575.161	243.426
38275	5.3289	525.613	300.349
38276	5.3291	486.572	87.0069
38277	5.3292	570.494	7.663
38278	5.3296	562.102	146.444
38279	5.3297	443.349	175.949
38280	5.3297	540.959	92.151
38281	5.3297	533.651	37.9827
38282	5.3298	489.947	154.8
38283	5.3298	441.607	234.65
38284	5.3298	456.401	120.298
38285	5.33	544.586	183.294
38286	5.33	482.293	122.428
38287	5.33	548.585	78.6155
38288	5.3305	486.777	260.731
38289	5.3308	483.585	230.414
38290	5.3308	470.763	80.4159
38291	5.3309	468.614	354.217
38292	5.331	424.222	125.529
38293	5.3311	543.801	84.0706

38294	5.3313	528.176	167.785
38295	5.3313	487.159	107.119
38296	5.3313	459.175	76.9749
38297	5.3313	582.144	58.5417
38298	5.3315	517.007	100.042
38299	5.3316	495.05	265.71
38300	5.332	585.078	88.0067
38301	5.3322	480.767	152.845
38302	5.3324	417.97	184.006
38303	5.3326	495.77	252.936
38304	5.3326	599.405	315.354
38305	5.3326	533.841	207.08
38306	5.3326	484.022	70.8682
38307	5.3328	579.165	61.7727
38308	5.3328	439.129	96.1843
38309	5.3328	424.168	82.8466
38310	5.3329	481.014	154.986
38311	5.3329	523.725	284.599
38312	5.3329	506.649	82.7407
38313	5.333	435.327	306.456
38314	5.333	516.518	60.7083
38315	5.333	481.829	222.673
38316	5.3332	501.214	122.134
38317	5.3333	465.168	152.387
38318	5.3334	494.804	144.234
38319	5.3336	473.633	127.055
38320	5.3336	496.277	86.3057
38321	5.3338	390.018	255.269
38322	5.3339	388.884	205.306
38323	5.3339	430.816	109.593
38324	5.334	480.888	154.913
38325	5.3341	456.627	41.0119
38326	5.3342	322.299	83.2205
38327	5.3342	443.954	29.8748
38328	5.3342	507.656	298.87
38329	5.3343	398.554	178.32
38330	5.3344	572.739	40.8362
38331	5.3345	546.446	34.1824
38332	5.3346	512.626	146.909
38333	5.3347	350.657	135.551
38334	5.3348	414.838	114.506
38335	5.3348	559.642	134.274
38336	5.3349	555.426	117.797
38337	5.335	515.829	25.8669
38338	5.335	536.545	38.2229
38339	5.335	465.862	122.218
38340	5.3351	530.964	7.5309
38341	5.3355	600.265	32.3522

38342	5.3356	511.513	103.474
38343	5.3357	538.031	128.582
38344	5.3358	506.311	60.5262
38345	5.336	598.06	38.8311
38346	5.3361	520.077	177.629
38347	5.3362	572.32	312.715
38348	5.3362	556.268	44.158
38349	5.3365	387.936	106.139
38350	5.3366	582.799	51.7979
38351	5.3366	449.061	192.561
38352	5.3366	523.036	279.659
38353	5.3366	540.847	233.128
38354	5.3367	448.723	48.3861
38355	5.3367	509.765	68.532
38356	5.3367	471.928	124.778
38357	5.3367	567.929	59.2497
38358	5.337	507.363	151.183
38359	5.337	491.401	123.81
38360	5.3372	650.921	132.488
38361	5.3373	499.678	249.723
38362	5.3375	428.013	276.42
38363	5.3377	455.649	123.233
38364	5.3377	479.481	112.752
38365	5.3379	616.854	14.9163
38366	5.3379	564.234	61.7749
38367	5.338	582.422	67.3856
38368	5.3381	517.014	58.1336
38369	5.3382	507.72	109.594
38370	5.3388	443.947	85.8224
38371	5.3389	522.513	189.02
38372	5.3389	482.575	92.2193
38373	5.339	571.107	98.0397
38374	5.3391	513.56	102.405
38375	5.3391	590.304	154.505
38376	5.3392	521.036	174.128
38377	5.3392	513.34	149.857
38378	5.3394	494.468	219.496
38379	5.3396	522.933	157.233
38380	5.3396	482.173	282.873
38381	5.3398	452.016	166.907
38382	5.34	528.762	132.349
38383	5.3401	491.214	134.538
38384	5.3401	510.677	328.39
38385	5.3403	516.764	266.346
38386	5.3404	570.122	62.7406
38387	5.3405	435.684	79.1274
38388	5.3406	552.551	314.712
38389	5.3408	529.601	289.162

38390	5.341	489.631	120.992
38391	5.3411	466.37	75.3351
38392	5.3412	528.492	185.742
38393	5.3412	575.112	45.1858
38394	5.3412	570.928	357.086
38395	5.3414	439.84	267.65
38396	5.3416	512.443	353.25
38397	5.3416	510.86	7.9193
38398	5.3419	440.806	174.888
38399	5.3421	512.094	167.411
38400	5.3421	549.359	357.719
38401	5.3422	550.967	104.523
38402	5.3422	609.944	55.3294
38403	5.3422	502.087	63.3192
38404	5.3423	385.642	109.799
38405	5.3426	587.543	215.625
38406	5.3427	413.034	151.012
38407	5.3428	548.782	323.907
38408	5.3429	518.822	62.1812
38409	5.3432	435.494	183.599
38410	5.3433	497.732	135.05
38411	5.3434	488.148	181.737
38412	5.3435	616.52	15.6736
38413	5.3438	431.885	181.375
38414	5.3439	405.697	219.861
38415	5.3439	557.515	90.257
38416	5.344	435.868	123.056
38417	5.344	488.073	353.04
38418	5.3444	606.534	19.2892
38419	5.345	508.282	304.922
38420	5.345	473.139	231.893
38421	5.345	516.307	139.875
38422	5.345	509.964	164.225
38423	5.3454	431.347	272.853
38424	5.3456	541.153	29.5494
38425	5.3457	531.673	292.265
38426	5.3457	545.63	21.7092
38427	5.3458	555.057	100.899
38428	5.3458	545.54	156.834
38429	5.3458	493.421	44.5414
38430	5.3458	569.269	62.8987
38431	5.3459	374.704	143.848
38432	5.3461	539.565	61.8058
38433	5.3462	435.25	146.407
38434	5.3463	472.54	179.158
38435	5.3466	577.575	64.2525
38436	5.3467	531.694	127.417
38437	5.3469	469.973	25.4815

38438	5.3472	533.687	159.534
38439	5.3472	586.309	352.58
38440	5.3473	591.399	28.8439
38441	5.3477	631.279	204.585
38442	5.3478	515.493	58.4095
38443	5.3479	625.871	70.8524
38444	5.3479	522.181	250.204
38445	5.3479	487.984	111.493
38446	5.3481	520.79	88.8786
38447	5.3482	584.582	128.508
38448	5.3482	481.547	133.071
38449	5.3483	554.799	79.0132
38450	5.3483	489.806	177.21
38451	5.3484	567.777	94.9506
38452	5.3484	466.282	121.12
38453	5.3485	555.142	127.367
38454	5.3486	482.532	281.473
38455	5.3488	540.02	73.4731
38456	5.3489	500.41	96.7371
38457	5.3489	499.417	233.365
38458	5.349	570.124	66.9725
38459	5.349	533.102	226.389
38460	5.349	616.502	96.1943
38461	5.3491	427.165	176.226
38462	5.3491	601.928	28.9132
38463	5.3493	502.123	129.009
38464	5.3495	585.988	80.2636
38465	5.3496	530.943	75.2956
38466	5.3496	519.502	86.8888
38467	5.3498	507.212	123.596
38468	5.3499	441.036	252.425
38469	5.35	538.25	154.377
38470	5.3501	544.018	128.096
38471	5.3503	502.857	253.306
38472	5.3503	523.555	141.615
38473	5.3504	412.541	318.541
38474	5.3505	512.636	331.679
38475	5.3505	559.33	129.358
38476	5.3506	481.838	229.638
38477	5.3506	523.114	334.532
38478	5.3507	498.371	162.422
38479	5.3508	502.404	166.619
38480	5.3509	553.406	45.7991
38481	5.351	480.535	246.293
38482	5.3511	446.115	237.486
38483	5.3512	574.547	147.081
38484	5.3513	521.2	58.0184
38485	5.3513	457.193	182.84

38486	5.3514	562.075	40.206
38487	5.3517	447.411	183.509
38488	5.352	525.338	215.325
38489	5.352	587.259	117.486
38490	5.352	539.015	162.794
38491	5.3521	485.572	199.166
38492	5.3524	479.315	357.977
38493	5.3526	468.166	270.305
38494	5.3527	553.802	120.461
38495	5.3528	512.015	114.008
38496	5.3533	551.624	65.6272
38497	5.3534	469.485	105.328
38498	5.3535	585.484	127.91
38499	5.3535	533.595	271.896
38500	5.3535	446.218	123.164
38501	5.3537	563.229	6.9311
38502	5.3539	543.363	70.6768
38503	5.3539	464.46	64.8633
38504	5.354	577.773	38.1062
38505	5.354	511.407	324.678
38506	5.3541	510.034	112.754
38507	5.3541	582.051	109.192
38508	5.3542	541.459	103.662
38509	5.3543	559.269	226.979
38510	5.3545	617.528	36.2364
38511	5.3545	461.012	116.014
38512	5.3546	436.651	133.088
38513	5.3547	489.914	325.284
38514	5.3548	575.481	109.55
38515	5.3548	479.337	35.0303
38516	5.355	534.981	46.7956
38517	5.3551	475.878	213.243
38518	5.3553	507.435	114.408
38519	5.3553	478.133	78.8853
38520	5.3554	560.637	64.3833
38521	5.3556	505.117	251.319
38522	5.3556	547.966	73.4692
38523	5.3556	532.355	111.827
38524	5.3557	542.51	93.6934
38525	5.3557	646.459	28.6925
38526	5.3559	500.11	282.692
38527	5.3562	457.72	268.587
38528	5.3564	455.338	149.116
38529	5.3565	330.461	26.3552
38530	5.3565	557.953	123.464
38531	5.3567	433.641	119.036
38532	5.3571	530.879	60.4458
38533	5.3571	366.042	157.843

38534	5.3573	574.577	45.6566
38535	5.3573	558.956	104.435
38536	5.3573	502.279	53.5266
38537	5.3576	443.612	77.3931
38538	5.3577	508.211	47.536
38539	5.3578	551.595	38.3853
38540	5.358	463.737	123.928
38541	5.358	444.041	154.214
38542	5.3583	495.941	109.236
38543	5.3583	482.221	107.012
38544	5.3586	420.838	103.8
38545	5.3589	448.996	285.455
38546	5.3589	511.274	90.4153
38547	5.359	547.618	286.715
38548	5.359	504.49	156.338
38549	5.3591	612.936	308.572
38550	5.3591	565.99	67.5149
38551	5.3591	594.342	225.167
38552	5.3593	441.019	233.136
38553	5.3593	446.005	161.515
38554	5.3594	534.337	232.407
38555	5.3594	390.447	182.148
38556	5.3598	649.393	19.7225
38557	5.3599	525.573	119.922
38558	5.36	583.628	84.3187
38559	5.3601	522.291	76.3795
38560	5.3602	523.995	103.438
38561	5.3606	514.168	272.448
38562	5.3606	458.235	92.1017
38563	5.3606	479.393	145.709
38564	5.3608	650.924	204.418
38565	5.361	456.543	117.849
38566	5.3613	424.617	62.8949
38567	5.3614	538.675	231.021
38568	5.3614	513.524	286.105
38569	5.3614	535.306	65.52
38570	5.3615	491.425	143.425
38571	5.3616	478.077	187.407
38572	5.3617	485.264	178.882
38573	5.3617	502.66	88.4869
38574	5.3619	474.497	80.3551
38575	5.3619	508.176	49.9291
38576	5.362	563.072	77.849
38577	5.362	392.278	145.724
38578	5.362	591.683	207.325
38579	5.3621	606.502	54.1867
38580	5.3621	528.975	33.3867
38581	5.3626	610.352	48.0335

38582	5.3627	421.246	185.357
38583	5.363	566.331	248.12
38584	5.3631	317.714	301.63
38585	5.3633	482.837	244.169
38586	5.3634	587.201	129.377
38587	5.3636	496.833	181.294
38588	5.3639	486.227	352.472
38589	5.364	476.401	169.102
38590	5.3641	557.411	78.1224
38591	5.3644	536.28	65.9608
38592	5.3648	570.339	155.041
38593	5.3648	571.987	24.1212
38594	5.365	620.27	51.6557
38595	5.3653	523.59	138.947
38596	5.3655	545.618	171.491
38597	5.3659	572.852	224.445
38598	5.3661	614.025	107.318
38599	5.3661	584.713	64.6204
38600	5.3662	436.915	122.698
38601	5.3663	485.399	316.61
38602	5.3664	573.492	305.264
38603	5.3664	582.266	187.493
38604	5.3666	504.939	177.071
38605	5.367	512.515	158.935
38606	5.367	563.833	52.7684
38607	5.367	514.359	265.051
38608	5.367	454.926	1.9138
38609	5.3671	503.707	20.7165
38610	5.3672	514.179	122.402
38611	5.3672	499.085	117.467
38612	5.3672	598.384	17.4964
38613	5.3673	430.711	124.817
38614	5.3676	378.917	115.043
38615	5.3678	532.503	104.838
38616	5.368	465.592	113.612
38617	5.3681	422.742	76.2832
38618	5.3683	494.47	95.0591
38619	5.3686	562.473	29.2927
38620	5.3687	465.862	117.095
38621	5.3688	565.378	277.498
38622	5.3689	526.993	115.891
38623	5.3689	547.538	31.4258
38624	5.369	525.355	201.129
38625	5.3698	425.531	158.466
38626	5.3698	521.305	103.89
38627	5.3701	547.643	110.283
38628	5.3702	450.761	85.9985
38629	5.3703	572.764	95.7017

38630	5.3704	455.647	107.197
38631	5.3708	436.344	118.846
38632	5.3708	587.731	58.8549
38633	5.3709	637.689	12.8019
38634	5.371	562.81	223.724
38635	5.371	605.322	25.0492
38636	5.3712	427.2	96.2276
38637	5.3713	574.839	349.748
38638	5.3714	458.174	108.545
38639	5.3715	396.768	132.14
38640	5.3716	517.443	123.952
38641	5.3716	547.343	68.5043
38642	5.3716	424.587	25.397
38643	5.3719	436.85	79.7264
38644	5.3721	470.842	140.831
38645	5.3721	500.678	149.144
38646	5.3722	436.199	265.624
38647	5.3723	103.654	29.6025
38648	5.3723	499.661	292.368
38649	5.3723	470.083	19.4648
38650	5.3724	587.432	64.6256
38651	5.3724	554.98	52.1185
38652	5.3725	516.495	134.673
38653	5.3725	556.722	67.4223
38654	5.3726	606.877	9.8973
38655	5.3727	555.798	131.621
38656	5.373	507.454	126.848
38657	5.3731	565.406	114.939
38658	5.3732	432.076	195.543
38659	5.3732	516.888	119.797
38660	5.3734	447.502	129.007
38661	5.3734	540.019	124.508
38662	5.3735	512.69	222.461
38663	5.3735	565.021	120.893
38664	5.3736	535.471	91.9325
38665	5.3736	632.792	53.1421
38666	5.3739	465.352	193.198
38667	5.374	513.232	73.7156
38668	5.3741	660.87	55.6059
38669	5.3744	498.922	103.257
38670	5.3745	493.958	280.768
38671	5.3748	501.995	132.469
38672	5.3754	645.667	22.7666
38673	5.3755	458.929	226.97
38674	5.3756	548.733	100.168
38675	5.3757	473.358	153.953
38676	5.376	592.239	28.9081
38677	5.3761	563.718	82.2683

38678	5.3761	509.175	274.698
38679	5.3761	560.228	46.6297
38680	5.3761	580.389	119.018
38681	5.3762	612.027	41.871
38682	5.3763	417.136	152.119
38683	5.3763	507.423	269.522
38684	5.3763	544.565	83.0683
38685	5.3763	564.474	198.316
38686	5.3764	531.358	58.7201
38687	5.3765	508.426	112.361
38688	5.3767	589.191	359.894
38689	5.3767	530.199	235.096
38690	5.3769	431.573	228.109
38691	5.3769	346.287	152.305
38692	5.377	482.238	23.1718
38693	5.377	513.563	242.153
38694	5.3771	603.767	16.2393
38695	5.3773	463.581	232.046
38696	5.3774	525.288	229.39
38697	5.3775	419.576	255.058
38698	5.3778	387.558	150.017
38699	5.3778	572.553	125.284
38700	5.3778	498.128	213.666
38701	5.3779	531.471	74.7354
38702	5.378	474.609	42.8796
38703	5.3781	525.857	180.082
38704	5.3782	452.463	132.716
38705	5.3783	574.346	316.967
38706	5.3784	495.023	279.604
38707	5.3784	443.851	157.354
38708	5.3786	462.584	357.12
38709	5.3787	567.781	63.2064
38710	5.3788	519.95	47.4281
38711	5.3788	554.266	73.0585
38712	5.3788	566.406	68.6858
38713	5.379	513.462	99.6433
38714	5.3791	532.535	94.5034
38715	5.3791	635.728	37.5798
38716	5.3791	529.406	268.006
38717	5.3793	530.842	253.322
38718	5.3795	448.327	173.843
38719	5.3796	499.618	64.4233
38720	5.3797	597.611	288.164
38721	5.3797	589.141	101.901
38722	5.38	542.168	181.47
38723	5.3801	567.251	300.805
38724	5.3801	598.869	124.222
38725	5.3802	622.289	154.26

38726	5.3804	392.957	174.643
38727	5.3804	609.444	8.7558
38728	5.3805	537.864	282.38
38729	5.3805	580.668	53.9442
38730	5.3806	538.479	194.853
38731	5.3806	464.99	115.779
38732	5.3808	470.214	171.745
38733	5.3809	436.842	157.852
38734	5.3809	621.423	22.3158
38735	5.381	528.917	208.069
38736	5.3811	478.187	228.116
38737	5.3811	514.413	81.2801
38738	5.3813	598.755	57.9264
38739	5.3814	578.644	335.096
38740	5.3821	615.41	51.57
38741	5.3822	526.191	92.5191
38742	5.3823	483.653	151.175
38743	5.3823	488.52	144.247
38744	5.3825	521.177	28.6508
38745	5.3826	478.238	204.066
38746	5.3826	483.953	107.041
38747	5.3827	513.792	234.374
38748	5.383	535.264	109.072
38749	5.383	468.846	124.789
38750	5.3832	561.477	321.079
38751	5.3832	527.255	130.422
38752	5.3834	570.08	188.568
38753	5.3835	674.4	79.1552
38754	5.3835	470.625	104.213
38755	5.3836	553.407	262.071
38756	5.3837	452.591	160.099
38757	5.384	537.227	317.818
38758	5.384	525.672	23.7342
38759	5.3841	537.912	69.65
38760	5.3841	501.509	63.9124
38761	5.3842	432.001	327.977
38762	5.3843	564.296	31.173
38763	5.3844	504.919	251.359
38764	5.3844	495.378	73.6164
38765	5.3844	513.113	124.423
38766	5.3844	616.411	61.8617
38767	5.3845	618.772	42.1124
38768	5.3846	534.897	145.567
38769	5.3848	488.216	110.68
38770	5.3849	550.741	33.1639
38771	5.385	559.136	267.047
38772	5.385	569.964	92.8572
38773	5.385	444.998	115.275

38774	5.3851	418.497	283.332
38775	5.3852	584.468	39.0464
38776	5.3852	433.859	284.773
38777	5.3853	540.53	265.031
38778	5.3853	555.624	152.909
38779	5.3854	445.489	83.8257
38780	5.3855	524.541	126.369
38781	5.3856	664.082	39.4235
38782	5.3856	472.847	8.5444
38783	5.3859	558.795	101.59
38784	5.3861	626.551	65.2611
38785	5.3862	483.139	121.318
38786	5.3863	539.902	84.3294
38787	5.3863	445.237	130.074
38788	5.3864	490.543	86.4166
38789	5.3865	559.74	282.01
38790	5.3867	516.216	135.168
38791	5.3867	646.176	206.992
38792	5.3868	558.288	63.0323
38793	5.3869	478.317	176.613
38794	5.3871	543.243	45.6972
38795	5.3871	549.28	127.373
38796	5.3872	615.487	30.8081
38797	5.3877	575.763	29.7139
38798	5.3877	548.542	137.358
38799	5.3878	593.279	13.2023
38800	5.3878	470.733	247.339
38801	5.3878	478.824	139.004
38802	5.3879	433.777	233.455
38803	5.3879	491.11	59.796
38804	5.3881	483.133	173.808
38805	5.3884	419.628	124.237
38806	5.3884	437.223	134.912
38807	5.3885	520.176	123.447
38808	5.3885	418.521	236.18
38809	5.3885	554.789	59.8357
38810	5.3891	591.053	355.848
38811	5.3891	515.695	215.632
38812	5.3893	641.844	57.9652
38813	5.3893	444.858	146.571
38814	5.3893	612.493	60.4091
38815	5.3894	612.769	65.6404
38816	5.3894	496.686	255.06
38817	5.3895	533.469	108.227
38818	5.3896	644.607	31.5885
38819	5.3897	541.714	124.287
38820	5.3897	623.115	26.0599
38821	5.3898	547.048	266.734

38822	5.3898	545.672	166.731
38823	5.3898	483.277	164.637
38824	5.39	544.908	78.4199
38825	5.3902	521.645	12.9937
38826	5.3905	495.68	112.039
38827	5.3905	576.252	40.8916
38828	5.3908	632.587	204.506
38829	5.3909	459.795	23.7955
38830	5.391	530.991	255.907
38831	5.391	529.462	130.204
38832	5.3912	674.836	85.0046
38833	5.3913	427.229	150.394
38834	5.3914	510.512	123.335
38835	5.3914	522.474	169.071
38836	5.3914	500.773	33.607
38837	5.392	519.606	164.618
38838	5.392	513.671	326.532
38839	5.3921	619.766	55.4982
38840	5.3921	448.643	118.846
38841	5.3927	563.862	244.196
38842	5.3927	514.898	294.989
38843	5.3928	548.917	112.503
38844	5.3928	476.543	233.499
38845	5.3929	569.584	297.559
38846	5.3929	581.178	79.8769
38847	5.3929	509.901	50.0306
38848	5.393	538.539	145.445
38849	5.393	491.984	152.204
38850	5.3933	535.116	48.6015
38851	5.3935	466.651	126.771
38852	5.3937	531.076	38.6585
38853	5.3939	530.561	228.693
38854	5.394	578.87	102.441
38855	5.3941	429.478	159.157
38856	5.3942	513.493	76.4469
38857	5.3942	509.285	174.024
38858	5.3942	483.894	2.5438
38859	5.3943	529.142	244.868
38860	5.3944	480.993	51.5621
38861	5.3947	579.851	249.767
38862	5.3948	424.445	283.183
38863	5.395	542.209	54.3406
38864	5.3951	554.669	93.2619
38865	5.3953	506.264	195.972
38866	5.3954	496.007	280.268
38867	5.3955	592.972	74.3901
38868	5.3955	496.724	71.4476
38869	5.3957	529.619	123.355

38870	5.3957	545.48	142.487
38871	5.3958	471.635	217.647
38872	5.3962	518.846	191.885
38873	5.3962	550.55	276.314
38874	5.3964	489.096	178.382
38875	5.3965	515.139	166.063
38876	5.3966	550.458	89.5721
38877	5.3966	546.937	75.2096
38878	5.3968	637.915	92.6481
38879	5.3968	560.869	64.9229
38880	5.3968	492.128	141.741
38881	5.3969	496.081	14.6222
38882	5.3969	458.663	71.7923
38883	5.3969	497.327	107.027
38884	5.3972	502.828	286.189
38885	5.3973	544.226	83.8322
38886	5.3975	477.675	87.741
38887	5.3975	521.51	287.667
38888	5.3977	520.564	61.9029
38889	5.3977	528.546	234.035
38890	5.3977	534.768	251.216
38891	5.3978	566.081	9.9506
38892	5.3979	618.389	39.8101
38893	5.3979	548.106	224.209
38894	5.398	489.076	195.664
38895	5.398	521.501	119.665
38896	5.398	630.624	30.9893
38897	5.3981	534.193	104.426
38898	5.3981	498.725	130.253
38899	5.3984	509.204	334.216
38900	5.3985	589.514	245.74
38901	5.3985	538.901	113.703
38902	5.3988	489.193	250.067
38903	5.399	513.357	26.5337
38904	5.3992	473.44	23.3293
38905	5.3994	558.058	38.3854
38906	5.3995	489.228	91.6759
38907	5.3998	512.968	259.343
38908	5.3999	624.871	37.0547
38909	5.4001	534.819	59.9577
38910	5.4002	587.12	34.8989
38911	5.4004	469.74	77.9216
38912	5.4007	468.136	87.5503
38913	5.4007	538.17	235.168
38914	5.4008	342.024	78.426
38915	5.4009	550.358	103.829
38916	5.4009	472.351	95.0177
38917	5.4009	516.103	293.876

38918	5.401	568.599	146.652
38919	5.401	543.383	221.536
38920	5.401	453.845	134.655
38921	5.401	667.179	204.269
38922	5.4011	477.12	167.184
38923	5.4012	603.808	332.707
38924	5.4012	535.548	4.7615
38925	5.4012	525.838	131.327
38926	5.4013	491.816	119.147
38927	5.4013	-2.791	76.8414
38928	5.4014	594.355	79.5067
38929	5.4014	508.903	8.4706
38930	5.4014	460.193	165.135
38931	5.4016	541.41	69.6957
38932	5.4016	580.037	56.793
38933	5.4016	541.084	84.2889
38934	5.4018	501.439	52.4223
38935	5.4019	555.751	103.283
38936	5.4019	536.161	121.534
38937	5.402	411.667	175.05
38938	5.402	568.014	123.251
38939	5.4021	506.552	302.904
38940	5.4021	551.117	157.477
38941	5.4022	515.277	16.6362
38942	5.4025	498.852	105.097
38943	5.4026	424.123	179.524
38944	5.4026	468.179	75.4979
38945	5.4027	428.712	110.453
38946	5.4029	488.234	145.795
38947	5.403	447.955	228.939
38948	5.4031	442.742	219.571
38949	5.4033	484.166	107.855
38950	5.4033	478.785	143.873
38951	5.4033	588.724	60.0054
38952	5.4033	605.019	10.7144
38953	5.4034	432.266	134.612
38954	5.4035	429.831	181.578
38955	5.4037	429.076	243.774
38956	5.4037	463.028	215.123
38957	5.4038	497.163	306.629
38958	5.4039	540.725	175.744
38959	5.404	541.166	137.831
38960	5.404	577.862	19.2011
38961	5.4042	508.549	115.551
38962	5.4045	586.281	87.508
38963	5.4045	547.313	80.4236
38964	5.4046	611.555	78.1357
38965	5.4046	558.372	10.1114

38966	5.4046	512.819	117.071
38967	5.4046	638.76	226.546
38968	5.4048	549.256	72.0864
38969	5.4051	521.973	229.046
38970	5.4051	436.736	148.512
38971	5.4053	466.852	129.485
38972	5.4054	478.508	134.648
38973	5.4054	476.473	93.8305
38974	5.4054	483.247	159.278
38975	5.4055	539.589	51.3363
38976	5.4055	413.49	183.766
38977	5.4057	494.092	114.979
38978	5.4058	474.554	165.979
38979	5.4059	574.247	58.3565
38980	5.4059	532.555	187.718
38981	5.406	595.922	113.159
38982	5.4061	615.132	53.9316
38983	5.4066	623.35	74.5195
38984	5.4067	512.967	16.1584
38985	5.4068	482.911	354.951
38986	5.4069	472.045	157.072
38987	5.4069	521.782	62.8148
38988	5.407	481.298	136.29
38989	5.4072	542.105	177.722
38990	5.4074	559.995	54.1935
38991	5.4075	576.126	40.2369
38992	5.4075	501.063	190.75
38993	5.4076	386.438	79.7406
38994	5.4079	515.138	63.5369
38995	5.408	581.768	241.283
38996	5.408	545.714	59.9681
38997	5.408	569.427	97.5576
38998	5.4082	471.687	86.4344
38999	5.4084	535.114	52.4998
39000	5.4085	547.598	125.696
39001	5.4086	548.85	277.248
39002	5.4086	541.982	226.278
39003	5.4087	433.564	232.749
39004	5.4087	499.658	55.1388
39005	5.4089	542.641	99.474
39006	5.4091	457.359	98.2627
39007	5.4091	591.294	303.635
39008	5.4093	516.375	7.7145
39009	5.4095	554.556	209.425
39010	5.4098	592.611	186.11
39011	5.4099	501.887	148.776
39012	5.41	548.154	216.719
39013	5.4101	650.25	78.2043

39014	5.4103	522.903	273.617
39015	5.4104	592.623	27.3635
39016	5.4109	548.098	76.3107
39017	5.411	504.756	311.457
39018	5.4111	577.995	208.142
39019	5.4112	543.647	35.513
39020	5.4113	471.533	193.087
39021	5.4115	521.71	132.749
39022	5.4117	368.719	174.476
39023	5.412	485.348	261.974
39024	5.412	438.881	236.865
39025	5.4121	538.308	178.953
39026	5.4121	671.721	206.135
39027	5.4122	423.388	106.793
39028	5.4122	536.225	150.998
39029	5.4123	554.299	67.0823
39030	5.4124	507.948	215.179
39031	5.4125	530.04	86.8372
39032	5.4129	529.201	240.534
39033	5.4129	561.709	104.735
39034	5.4129	531.043	60.9761
39035	5.4129	633.765	55.9808
39036	5.413	570.881	259.731
39037	5.4131	505.738	261.715
39038	5.4139	624.455	61.6391
39039	5.414	421.905	171.061
39040	5.4141	511.373	280.526
39041	5.4141	574.348	26.8753
39042	5.4143	501.548	317.671
39043	5.4146	542.223	21.6107
39044	5.4149	560.005	61.1317
39045	5.4155	508.786	322.452
39046	5.4155	466.361	127.516
39047	5.4155	416.486	275.767
39048	5.4155	528.339	65.6399
39049	5.4157	532.647	121.934
39050	5.4157	614.508	53.1551
39051	5.416	452.039	231.581
39052	5.4163	555.847	122.251
39053	5.4163	461.145	128.872
39054	5.4164	523.086	129.317
39055	5.4165	532.67	120.189
39056	5.4166	573.507	224.975
39057	5.4166	600.803	37.0805
39058	5.4167	513.026	144.755
39059	5.417	557.379	286.683
39060	5.4171	473.949	220.246
39061	5.4172	527.309	197.807

39062	5.4175	509.509	262.327
39063	5.4176	534.124	53.9315
39064	5.4176	493.546	159.587
39065	5.4177	447.743	172.944
39066	5.4178	557.978	93.5842
39067	5.4179	437.65	158.419
39068	5.4179	498.658	116.494
39069	5.4179	448.832	89.2503
39070	5.4179	569.467	49.637
39071	5.4181	504.498	55.131
39072	5.4182	594.764	61.0769
39073	5.4183	512.191	137.211
39074	5.4184	584.504	74.0691
39075	5.4186	516.966	109.153
39076	5.4187	522.739	171.273
39077	5.4187	506.572	58.1746
39078	5.4188	496.703	146.591
39079	5.4188	424.416	199.022
39080	5.4192	598.454	63.9386
39081	5.4193	513.887	100.425
39082	5.4196	600.619	60.6373
39083	5.4198	531.148	278.503
39084	5.4199	465.219	159.94
39085	5.4201	660.369	72.2999
39086	5.4203	628.694	256.149
39087	5.4203	552.062	52.3078
39088	5.4204	537.288	61.7667
39089	5.4204	483.011	126.511
39090	5.4206	439.319	91.6567
39091	5.4207	419.685	108.086
39092	5.4207	585.583	38.7171
39093	5.4208	513.536	21.4521
39094	5.4209	490.189	78.9787
39095	5.421	589.271	218.513
39096	5.4212	576.803	243.874
39097	5.4212	546.485	51.469
39098	5.4212	432.936	197.257
39099	5.4214	477.972	218.993
39100	5.4214	506.286	99.7319
39101	5.4215	578.87	102.65
39102	5.4216	640.705	70.3453
39103	5.4216	489.432	291.165
39104	5.4218	542.091	144.803
39105	5.4219	508.803	247.272
39106	5.422	539.709	120.228
39107	5.4222	578.226	191.096
39108	5.4223	654.209	28.4412
39109	5.4223	478.658	119.706

39110	5.4223	490.789	132.76
39111	5.4223	519.473	115.243
39112	5.4226	526.582	95.2699
39113	5.4234	470.251	219.19
39114	5.4234	529.232	301.904
39115	5.4236	631.357	204.621
39116	5.4239	494.283	155.775
39117	5.4239	446.968	86.4006
39118	5.424	581.72	59.7971
39119	5.4241	411.956	51.1612
39120	5.4242	302.604	339.051
39121	5.4242	577.008	22.6652
39122	5.4244	474.677	191.266
39123	5.4245	607.48	27.2477
39124	5.4245	567.67	322.611
39125	5.4246	538.076	167.248
39126	5.4246	575.442	76.3538
39127	5.4247	545.398	107.039
39128	5.4247	549.529	113.301
39129	5.4247	484.949	124.229
39130	5.4249	626.639	30.2794
39131	5.4251	562.647	56.8772
39132	5.4251	513.206	36.5176
39133	5.4252	534.609	111.105
39134	5.4254	616.901	230.327
39135	5.4256	599.005	172.321
39136	5.4257	515.075	265.303
39137	5.4257	567.926	131.929
39138	5.4259	620.967	238.566
39139	5.426	591.772	139.983
39140	5.426	587.271	314.85
39141	5.4261	572.791	77.7141
39142	5.4261	612.165	31.2448
39143	5.4262	559.456	73.7085
39144	5.4266	274.623	130.353
39145	5.4267	542.963	272.743
39146	5.4268	499.959	143.235
39147	5.4269	593.162	39.1026
39148	5.427	465.502	25.2666
39149	5.4271	448.92	51.2867
39150	5.4271	518.098	135.066
39151	5.4273	558.709	38.2644
39152	5.4274	634.391	57.4936
39153	5.4278	525.025	194.451
39154	5.4281	607.657	357.454
39155	5.4282	522.66	16.3718
39156	5.4283	510.874	129.027
39157	5.4286	556.957	111.635

39158	5.4286	428.723	79.5774
39159	5.4287	480.339	197.363
39160	5.4288	547.712	61.6594
39161	5.4288	521.375	149.591
39162	5.429	482.366	88.3502
39163	5.429	509.461	193.077
39164	5.4292	457.295	163.347
39165	5.4293	588.842	350.189
39166	5.4294	619.221	127.323
39167	5.4294	661.047	97.0433
39168	5.4294	467.078	135.759
39169	5.4296	636.01	28.1318
39170	5.4296	588.762	223.627
39171	5.4296	472.272	175.804
39172	5.4297	601.002	125.534
39173	5.4298	506.105	151.06
39174	5.4298	517.103	198.33
39175	5.4302	488.115	189.155
39176	5.4303	552.84	135.878
39177	5.4307	300.332	180.571
39178	5.4307	596.613	210.308
39179	5.431	551.476	268.082
39180	5.431	540.024	121.654
39181	5.4311	524.817	353.896
39182	5.4311	540.191	97.065
39183	5.4311	479.018	228.721
39184	5.4313	426.218	28.9582
39185	5.4314	618.87	21.4347
39186	5.4314	549.757	6.3134
39187	5.4314	630.996	142.729
39188	5.4316	668.851	356.79
39189	5.4321	434.35	152.971
39190	5.4322	613.242	37.0913
39191	5.4322	677.316	288.08
39192	5.4324	538.946	190.465
39193	5.4324	620.993	11.7409
39194	5.4325	522.393	221.795
39195	5.4325	558.926	61.4983
39196	5.4326	437.909	186.069
39197	5.4329	577.316	36.4497
39198	5.4331	591.768	352.499
39199	5.4331	588.403	54.301
39200	5.4334	611.439	3.8622
39201	5.4334	455.655	124.853
39202	5.4335	574.889	24.2611
39203	5.4336	444.741	122.697
39204	5.4336	602.481	18.8543
39205	5.4337	512.693	132.688

39206	5.4337	487.348	123.871
39207	5.4337	550.459	163.731
39208	5.4339	529.435	102.711
39209	5.434	539.339	169.354
39210	5.4342	637.277	59.9103
39211	5.4345	608.021	84.335
39212	5.4345	441.136	238.761
39213	5.4345	529.345	87.1044
39214	5.4346	527.529	295.339
39215	5.4347	589.278	72.493
39216	5.4348	636.456	243.969
39217	5.4349	490.447	192.858
39218	5.4351	602.831	288.319
39219	5.4356	565.425	127.7
39220	5.4356	531.312	316.275
39221	5.4359	480.179	229.434
39222	5.4361	687.72	77.3714
39223	5.4361	470.288	185.679
39224	5.4361	517.634	101.843
39225	5.4361	558.447	63.3624
39226	5.4361	513.947	84.4418
39227	5.4363	456.483	94.3767
39228	5.4364	443.293	123.67
39229	5.4365	507.831	268.819
39230	5.4365	491.357	190.84
39231	5.4366	507.845	56.918
39232	5.4367	435.797	264.944
39233	5.4367	500.035	172.725
39234	5.4368	547.184	9.7449
39235	5.4369	600.799	87.7538
39236	5.437	553.652	95.3655
39237	5.4371	491.874	292.172
39238	5.4374	560.116	159.679
39239	5.4378	597.702	73.5845
39240	5.4378	498.706	131.672
39241	5.4378	541.044	174.821
39242	5.4379	548.612	275.944
39243	5.4381	456.872	42.4323
39244	5.4382	627.046	32.3207
39245	5.4384	605.519	99.5194
39246	5.4384	515.361	81.233
39247	5.4384	537.526	85.8749
39248	5.4384	508.159	109.171
39249	5.4384	461.852	122.842
39250	5.4384	578.963	43.4899
39251	5.4386	422.53	182.978
39252	5.4389	549.853	261.737
39253	5.4389	530.228	6.7671

39254	5.439	484.881	106.123
39255	5.4391	533.624	174.897
39256	5.4391	530.253	89.7871
39257	5.4392	567.294	266.651
39258	5.4393	499.139	160.383
39259	5.4393	477.696	119.241
39260	5.4393	459.753	150.171
39261	5.4394	460.18	200.444
39262	5.4394	491.743	105.532
39263	5.4394	524.678	108.984
39264	5.4395	613.194	63.0864
39265	5.4395	582.204	88.8029
39266	5.4396	506.494	114.798
39267	5.4396	604.889	63.4507
39268	5.4401	612.315	72.8354
39269	5.4401	475.201	325.768
39270	5.4402	470.512	148.348
39271	5.4403	602.153	58.2269
39272	5.4403	519.592	97.4014
39273	5.4403	547.725	251.191
39274	5.4405	497.035	38.2363
39275	5.4405	549.332	323.484
39276	5.4405	349.598	304.603
39277	5.4405	507.677	170.671
39278	5.4406	547.26	31.331
39279	5.4407	563.825	235.427
39280	5.4408	512.463	249.322
39281	5.4408	553.375	121.344
39282	5.4409	577.06	350.662
39283	5.441	528.414	231.453
39284	5.4411	491.969	22.3548
39285	5.4413	533.394	56.2555
39286	5.4413	671.852	48.2076
39287	5.4413	659.131	229.913
39288	5.4414	455.337	122.821
39289	5.4414	563.473	25.1205
39290	5.4415	504.601	219.383
39291	5.4415	521.472	29.9084
39292	5.4417	560.68	156.832
39293	5.4419	575.184	323.777
39294	5.4422	572.898	147.583
39295	5.4424	458.301	94.9441
39296	5.4424	425.224	133.583
39297	5.4424	519.968	167.935
39298	5.4425	540.321	296.547
39299	5.4425	576.662	52.1127
39300	5.4426	461.777	323.238
39301	5.4427	613.817	333.125

39302	5.4428	592.907	56.215
39303	5.4429	505.352	57.2285
39304	5.4433	518.031	90.1551
39305	5.4435	535.278	83.0025
39306	5.4436	589.445	101.052
39307	5.4437	521.702	255.497
39308	5.4437	623.744	208.296
39309	5.4439	516.278	104.693
39310	5.4439	476.813	86.8001
39311	5.4439	511.879	115.443
39312	5.4443	569.297	159.365
39313	5.4445	619.335	63.2294
39314	5.4445	520.307	109.907
39315	5.4445	595.045	332.81
39316	5.4453	607.356	305.028
39317	5.4454	529.162	96.2282
39318	5.4456	527.511	238.487
39319	5.4456	598.84	246.335
39320	5.4458	573.113	259.441
39321	5.4458	513.981	104.907
39322	5.446	486.539	136.118
39323	5.4464	545.737	119.773
39324	5.4464	655.921	44.2423
39325	5.4465	540.802	143.81
39326	5.4466	470.121	98.8771
39327	5.4467	529.2	106.069
39328	5.4467	614.509	119.824
39329	5.4471	494.44	137.86
39330	5.4472	505.695	182.185
39331	5.4472	650.358	15.3687
39332	5.4473	600.6	80.2512
39333	5.4473	501.152	277.504
39334	5.4474	579.072	359.403
39335	5.4475	532.713	162.968
39336	5.4475	676.53	70.3375
39337	5.4476	516.378	131.828
39338	5.4477	535.763	131.57
39339	5.4477	511.413	96.4105
39340	5.4477	515.469	138.383
39341	5.4477	521.283	161.198
39342	5.4478	570.67	100.231
39343	5.448	624.368	268.713
39344	5.4482	455.258	178.682
39345	5.4483	488.336	157.686
39346	5.4483	616.529	61.0901
39347	5.4485	632.853	45.9202
39348	5.4486	528.183	249.374
39349	5.4489	511.52	153.734

39350	5.4492	439.973	74.7643
39351	5.4493	548.629	321.469
39352	5.4493	482.184	117.066
39353	5.4493	496.024	183.718
39354	5.4493	598.417	135.285
39355	5.4493	630.73	14.7963
39356	5.4494	557.598	127.094
39357	5.4496	474.86	189.242
39358	5.4498	449.124	176.647
39359	5.4499	461.912	190.943
39360	5.4499	483.758	131.369
39361	5.45	543.718	120.778
39362	5.4502	568.469	53.7522
39363	5.4503	609.409	48.4255
39364	5.4505	554.1	100.191
39365	5.4507	612.708	80.4262
39366	5.451	393.334	221.978
39367	5.451	471.985	126.606
39368	5.4512	573.941	292.344
39369	5.4513	555.07	158.741
39370	5.4513	460.652	63.0354
39371	5.4517	465.102	173.467
39372	5.4519	512.263	240.393
39373	5.4519	550.305	123.598
39374	5.452	482.56	284.233
39375	5.4522	611.744	51.1508
39376	5.4522	408.851	123.294
39377	5.4523	547.546	55.0419
39378	5.4524	589.297	328.385
39379	5.4524	568.844	114.014
39380	5.4524	525.111	40.0945
39381	5.4526	622.162	71.6336
39382	5.4526	538.919	118.235
39383	5.4526	578.661	125.298
39384	5.4526	448.977	14.2153
39385	5.4526	483.488	103.083
39386	5.4526	591.437	30.8268
39387	5.4527	479.808	223.526
39388	5.4528	545.245	50.031
39389	5.4529	549.705	128.804
39390	5.4529	526.71	67.0281
39391	5.4529	532.591	71.3537
39392	5.4532	479.628	128.027
39393	5.4535	575.147	94.9086
39394	5.4536	534.415	325.973
39395	5.4536	529.819	124.584
39396	5.4538	490.933	141.399
39397	5.4538	492.824	172.388

39398	5.4539	564.699	120.667
39399	5.454	598.587	287.5
39400	5.4544	543.85	118.661
39401	5.4547	433.523	177.62
39402	5.455	433.071	226.367
39403	5.455	479.895	198.336
39404	5.4551	573.226	40.4591
39405	5.4552	480.696	80.9235
39406	5.4553	565.469	96.9292
39407	5.4553	578.252	22.1582
39408	5.4554	510.193	53.4719
39409	5.4556	525.602	224.398
39410	5.4557	502.78	108.04
39411	5.4559	557.12	84.1699
39412	5.4561	510.287	145.063
39413	5.4561	456.447	328.292
39414	5.4562	463.133	334.912
39415	5.4567	453.64	196.773
39416	5.4568	462.88	118.088
39417	5.4569	597.786	271.528
39418	5.4571	562.771	198.232
39419	5.4572	574.784	2.9174
39420	5.4574	580.682	23.7145
39421	5.4575	475.683	112.54
39422	5.4581	459.993	170.494
39423	5.4582	573.956	84.6558
39424	5.4584	654.718	31.0765
39425	5.4585	476.614	189.544
39426	5.4587	546.82	111.387
39427	5.4591	473.633	135.605
39428	5.4592	469.968	228.512
39429	5.4592	633.797	4.3854
39430	5.4594	557.292	21.8615
39431	5.4594	562.371	94.637
39432	5.4596	396.974	152.169
39433	5.4597	542.871	133.665
39434	5.4597	538.493	111.134
39435	5.4599	453.946	228.057
39436	5.4599	506.523	202.841
39437	5.46	519.623	37.5364
39438	5.46	680.768	57.4311
39439	5.4602	526.204	238.177
39440	5.4605	581.876	127.947
39441	5.4609	530.899	114.111
39442	5.4611	538.989	159.668
39443	5.4614	569.49	229.89
39444	5.4614	622.272	100.519
39445	5.4616	511.57	254.23

39446	5.4616	564.858	50.1076
39447	5.4618	581.88	54.2546
39448	5.4619	486.541	152.576
39449	5.462	510.757	121.897
39450	5.462	594.018	190.964
39451	5.4621	584.694	265.073
39452	5.4621	586.116	255.501
39453	5.4622	458.865	274.483
39454	5.4627	538.417	299.086
39455	5.4627	598.026	190.621
39456	5.4627	645.245	33.7489
39457	5.4629	608.423	74.1561
39458	5.4629	612.022	76.7465
39459	5.463	601.467	187.019
39460	5.463	571.18	108.507
39461	5.463	570.759	110.427
39462	5.4631	621.308	194.297
39463	5.4631	450.88	283.939
39464	5.4631	545.928	57.1751
39465	5.4632	656.764	13.8949
39466	5.4633	465.786	94.3565
39467	5.4634	516.78	254.541
39468	5.4634	472.807	146.937
39469	5.4635	590.576	248.236
39470	5.4635	636.274	58.4632
39471	5.4637	527.213	11.5072
39472	5.4641	600.469	146.456
39473	5.4642	462.256	179.73
39474	5.4643	598.661	112.348
39475	5.4644	624.663	57.3678
39476	5.4646	584.754	176.941
39477	5.4648	538.994	89.9724
39478	5.4648	519.335	34.0989
39479	5.4648	526.268	194.995
39480	5.4648	514.815	129.201
39481	5.4649	530.692	124.719
39482	5.4649	566.616	143.722
39483	5.4651	544.072	322.865
39484	5.4654	594.074	290.186
39485	5.4656	509.368	172.041
39486	5.4658	587.568	303.305
39487	5.4659	462.587	188.96
39488	5.4662	557.191	66.8475
39489	5.4668	462.681	102.944
39490	5.4668	609.86	238.193
39491	5.467	492.645	172.847
39492	5.467	499.271	267.934
39493	5.4671	548.91	263.23

39494	5.4672	622.425	46.3823
39495	5.4674	520.928	230.365
39496	5.4674	621.17	116.815
39497	5.4675	554.095	48.7385
39498	5.4677	583.361	27.3434
39499	5.4678	436.025	237.313
39500	5.4681	655.963	32.4878
39501	5.4683	498.997	59.9369
39502	5.4683	560.128	149.487
39503	5.4684	522.761	94.9466
39504	5.4684	615.962	53.8921
39505	5.4686	535.174	160.152
39506	5.4686	480.329	166.856
39507	5.4686	474.414	85.5822
39508	5.469	516.172	157.657
39509	5.469	458.697	190.632
39510	5.469	617.485	293.014
39511	5.4691	625.515	237.937
39512	5.4692	581.707	125.588
39513	5.4694	527.917	356.762
39514	5.4694	478.752	65.8589
39515	5.4694	572.36	59.9402
39516	5.4696	626.466	106.504
39517	5.4696	564.477	222.152
39518	5.4696	532.738	62.3757
39519	5.4699	676.642	61.1793
39520	5.4699	562.791	51.4
39521	5.47	673.133	55.19
39522	5.47	487.006	269.435
39523	5.47	578.955	22.3784
39524	5.4704	565.963	195.325
39525	5.4706	656.844	82.0698
39526	5.4709	482.376	238.664
39527	5.4709	504.023	168.079
39528	5.4711	434.101	85.4709
39529	5.4712	545.307	99.9652
39530	5.4712	665.833	53.2259
39531	5.4713	497.645	65.4275
39532	5.4714	601.942	128.02
39533	5.4714	670.005	28.5107
39534	5.4717	571.642	115.811
39535	5.4717	556.175	66.5316
39536	5.4718	607.845	70.9037
39537	5.4718	597.783	116.775
39538	5.4721	546.972	178.352
39539	5.4721	453.266	184.921
39540	5.4721	562.83	103.715
39541	5.4722	616.022	95.471

39542	5.4723	559.383	316.303
39543	5.4723	585.321	108.96
39544	5.4723	563.434	114.471
39545	5.4724	501.245	189.538
39546	5.4724	487.133	130.98
39547	5.4726	539.472	246.593
39548	5.4726	584.197	31.9781
39549	5.4726	543.585	105.792
39550	5.4727	572.602	225.119
39551	5.473	472.107	234.756
39552	5.473	665.216	337.982
39553	5.4731	528.004	214.675
39554	5.4732	522.686	322.484
39555	5.4735	502.721	136.818
39556	5.4736	541.002	57.1084
39557	5.4737	551.019	142.117
39558	5.4739	451.385	97.3679
39559	5.4739	501.908	114.267
39560	5.474	521.558	161.666
39561	5.474	550.59	162.042
39562	5.4741	487.385	275.062
39563	5.4742	526.916	96.46
39564	5.4743	614.118	310.812
39565	5.4745	511.972	285.519
39566	5.4746	606.321	43.7678
39567	5.4747	526.217	63.8508
39568	5.4749	618.098	88.7006
39569	5.475	502.879	79.487
39570	5.475	623.494	116.01
39571	5.4751	616.441	102.603
39572	5.4751	520.655	78.491
39573	5.4752	596.325	286.418
39574	5.4752	561.395	51.3107
39575	5.4753	484.525	218.592
39576	5.4753	553.182	30.2983
39577	5.4753	650.803	45.0977
39578	5.4754	520.626	165.63
39579	5.4755	600.371	300.873
39580	5.4756	593.898	72.6492
39581	5.4758	475.386	192.11
39582	5.476	626.535	18.5227
39583	5.4761	567.443	87.9471
39584	5.4762	462.637	176.215
39585	5.4765	675.799	32.308
39586	5.4765	469.277	173.689
39587	5.4765	522.037	94.4683
39588	5.4766	538.748	110.41
39589	5.4766	529.458	152.62

39590	5.4767	564.113	25.5733
39591	5.477	677.684	317.032
39592	5.477	390.657	145.473
39593	5.4771	530.99	88.1498
39594	5.4772	534.169	223.07
39595	5.4772	515.35	25.2265
39596	5.4773	581.126	226.696
39597	5.4773	578.147	254.138
39598	5.4773	554.693	143.609
39599	5.4775	416.51	178.486
39600	5.4776	560.113	185.071
39601	5.4776	682.24	206.389
39602	5.4777	559.527	128.582
39603	5.4777	596.075	126.485
39604	5.4778	558.224	107.895
39605	5.4778	455.777	192.305
39606	5.4778	516.303	37.6851
39607	5.4781	677.037	32.1446
39608	5.4781	602.805	55.6011
39609	5.4784	525.307	170.911
39610	5.4785	546.749	51.7879
39611	5.4787	612.216	67.6788
39612	5.4788	627.012	40.2175
39613	5.4788	500.452	226.046
39614	5.4789	493.356	254.265
39615	5.479	644.079	101.394
39616	5.479	547.692	92.2856
39617	5.4791	565.213	187.505
39618	5.4791	590.623	16.2028
39619	5.4791	569.791	217.205
39620	5.4791	597.489	64.2462
39621	5.4792	556.301	322.663
39622	5.4792	525.944	258.175
39623	5.4793	610.764	93.6038
39624	5.4794	535.606	103.233
39625	5.4795	572.941	3.7959
39626	5.4797	472.136	199.533
39627	5.4799	582.436	182.473
39628	5.4799	476.07	171.19
39629	5.4801	531.607	290.652
39630	5.4804	479.613	263.413
39631	5.4804	487.144	170.634
39632	5.4805	565.502	114.162
39633	5.4805	588.545	64.8334
39634	5.4807	635.876	150.066
39635	5.4808	465.951	135.128
39636	5.4808	614.136	318.01
39637	5.4808	609.198	255.032

39638	5.4809	459.911	190.885
39639	5.4812	544.23	174.212
39640	5.4813	530.28	176.689
39641	5.4813	556.724	175.767
39642	5.4814	497.736	5.7119
39643	5.4816	549.033	233.225
39644	5.4816	596.508	74.8065
39645	5.4819	545.632	107.823
39646	5.4819	470.642	188.128
39647	5.482	538.817	184.736
39648	5.482	477.624	223.38
39649	5.4821	532.004	150.168
39650	5.4822	562.842	58.7617
39651	5.4822	584.2	147.526
39652	5.4824	563.185	182.66
39653	5.4827	463.447	163.832
39654	5.4829	432.309	123.275
39655	5.483	615.721	288.006
39656	5.4832	512.586	203.453
39657	5.4832	586.079	296.326
39658	5.4833	551.777	108.332
39659	5.4834	544.337	187.628
39660	5.4834	508.201	174.063
39661	5.4834	441.014	164.166
39662	5.4835	501.397	66.946
39663	5.4838	575.568	270.742
39664	5.484	516.079	92.0461
39665	5.4841	641.549	39.1746
39666	5.4843	614.633	69.019
39667	5.4843	532.071	201.29
39668	5.4843	595.735	150.801
39669	5.4844	651.238	15.1475
39670	5.4846	599.892	118.624
39671	5.4846	510.002	181.841
39672	5.4847	605.087	41.7157
39673	5.4847	547.728	228.671
39674	5.4847	573.521	71.1673
39675	5.4848	552.882	260.35
39676	5.4848	650.958	69.8259
39677	5.4849	594.681	109.246
39678	5.4849	607.104	57.9031
39679	5.485	601.501	68.6011
39680	5.4851	544.73	121.035
39681	5.4852	484.891	147.588
39682	5.4852	642.784	167.956
39683	5.4853	539.871	48.4124
39684	5.4855	632.539	123.543
39685	5.4855	617.821	61.3751

39686	5.4856	650.149	258.124
39687	5.4857	503.005	100.566
39688	5.4858	314.099	197.589
39689	5.4858	561.123	276.207
39690	5.4858	555.528	57.2024
39691	5.4861	612.971	83.6514
39692	5.4861	616.021	297.197
39693	5.4862	532.919	156.92
39694	5.4864	559.39	189.897
39695	5.4864	479.892	179.62
39696	5.4865	671.264	48.9181
39697	5.4867	622.133	28.9479
39698	5.4868	542.816	193.135
39699	5.487	539.81	109.383
39700	5.487	509.58	142.899
39701	5.4873	482.448	61.3182
39702	5.4874	423.031	156.655
39703	5.4876	533.191	146.178
39704	5.4879	414.106	27.3721
39705	5.488	593.023	212.763
39706	5.4881	620.422	131.532
39707	5.4881	455.169	168.602
39708	5.4881	553.691	110.001
39709	5.4883	588.13	74.6512
39710	5.4883	485.613	101.12
39711	5.4883	605.559	124.81
39712	5.4886	579.849	156.2
39713	5.4887	592.053	92.0525
39714	5.4887	547.679	81.4628
39715	5.489	575.431	179.682
39716	5.4892	573.606	188.713
39717	5.4893	746.025	42.6205
39718	5.4895	482.972	188.723
39719	5.4895	557.015	124.701
39720	5.4895	548.271	144.928
39721	5.4895	531.928	29.4953
39722	5.4899	512.941	178.24
39723	5.4899	513.765	146.856
39724	5.49	450.146	42.7008
39725	5.4902	568.933	20.2688
39726	5.4903	591.506	23.646
39727	5.4905	530.176	307.224
39728	5.4908	690.149	131.059
39729	5.4908	595.373	44.3554
39730	5.491	567.872	127.469
39731	5.4912	564.51	85.8138
39732	5.4912	397.304	92.5142
39733	5.4914	584.827	189.055

39734	5.4915	586.779	107.974
39735	5.4916	671.515	69.587
39736	5.4917	602.988	71.7157
39737	5.4917	609.861	104.974
39738	5.4918	568.097	62.4483
39739	5.4919	526.222	219.437
39740	5.4919	529.766	221.414
39741	5.492	634.426	71.8874
39742	5.4921	427.783	171.61
39743	5.4921	632.618	63.2046
39744	5.4921	596.409	96.4988
39745	5.4924	559.679	52.0195
39746	5.4925	511.672	158.433
39747	5.4926	581.293	258.128
39748	5.4926	460.442	167.16
39749	5.4926	554.093	133.224
39750	5.4927	620.222	62.0998
39751	5.4928	543.301	358.422
39752	5.4928	493.996	113.18
39753	5.4934	448.388	187.643
39754	5.4936	545.965	142.352
39755	5.4936	514.041	111.426
39756	5.4936	654.6	45.6398
39757	5.4937	535.606	121.298
39758	5.4937	660.937	36.3848
39759	5.4938	479.943	146.036
39760	5.4941	542.173	120.195
39761	5.4941	525.471	143.503
39762	5.4942	565.076	116.788
39763	5.4943	407.011	254.542
39764	5.4943	426.73	109.27
39765	5.4945	561.881	43.4536
39766	5.4945	570.204	106.322
39767	5.4948	497.911	234.877
39768	5.495	636.693	306.336
39769	5.495	503.96	274.997
39770	5.4951	462.404	168.916
39771	5.4953	659.228	50.199
39772	5.4955	218.554	356.178
39773	5.4956	-3.6446	119.729
39774	5.4956	483.041	128.18
39775	5.4957	593.965	16.0963
39776	5.4959	546.401	276.756
39777	5.4961	513.587	218.18
39778	5.4961	453.694	175.541
39779	5.4961	477.322	114.657
39780	5.4963	493.803	21.7709
39781	5.4963	614.889	41.7843

39782	5.4964	536.636	119.933
39783	5.4966	616.485	60.4362
39784	5.4966	498.498	121.579
39785	5.4967	600.838	305.39
39786	5.4967	567.301	156.96
39787	5.4967	437.731	173.472
39788	5.4971	597.914	307.442
39789	5.4971	-2.8419	225.016
39790	5.4972	553.993	279.925
39791	5.4972	469.616	173.194
39792	5.4973	590.647	191.526
39793	5.4976	532.372	249.988
39794	5.4977	677.17	49.2451
39795	5.4977	534.301	216.43
39796	5.4979	640.315	123.784
39797	5.4983	584.508	51.7759
39798	5.4984	572.92	255.371
39799	5.4986	498.903	154.973
39800	5.4988	598.235	57.5327
39801	5.4989	523.744	117.545
39802	5.4992	525.522	80.2147
39803	5.4994	480.737	134.35
39804	5.4995	563.607	58.7282
39805	5.4995	557.326	220.389
39806	5.4996	647.923	4.2331
39807	5.5	488.46	222.894
39808	5.5	538.944	54.1793
39809	5.5001	528.765	122.328
39810	5.5001	522.459	101.084
39811	5.5002	468.912	216.679
39812	5.5002	507.858	134.315
39813	5.5004	589.074	267.082
39814	5.5004	530.99	75.4361
39815	5.5005	473.58	104.598
39816	5.5006	468.547	258.57
39817	5.5006	616.556	50.1171
39818	5.5006	655.709	51.3876
39819	5.5007	643.187	104.72
39820	5.5009	568.504	62.0707
39821	5.501	575.745	75.6803
39822	5.5011	549.277	184.426
39823	5.5012	582.822	123.98
39824	5.5015	538.295	120.682
39825	5.5015	612.344	54.2461
39826	5.5015	674.141	87.068
39827	5.5016	597.919	15.909
39828	5.5017	578.457	285.01
39829	5.5017	476.132	266.871

39830	5.5019	535.497	257.782
39831	5.5019	593.042	59.0515
39832	5.502	601.216	275.363
39833	5.5022	635.42	44.0057
39834	5.5023	433.428	225.415
39835	5.5026	637.832	339.079
39836	5.5028	518.246	37.0861
39837	5.5028	574.447	136.522
39838	5.5029	534.347	63.4612
39839	5.5029	565.498	51.9975
39840	5.5035	507.824	267.493
39841	5.5035	488.257	183.341
39842	5.5037	477.179	176.85
39843	5.5037	532.377	122.631
39844	5.5039	484.582	118.332
39845	5.504	470.968	221.849
39846	5.5042	455.854	144.063
39847	5.5043	613.771	90.1317
39848	5.5043	521.071	179.892
39849	5.5043	600.469	110.221
39850	5.5044	654.473	16.0172
39851	5.5046	544.068	177.143
39852	5.5047	535.192	154.277
39853	5.5047	534.044	100.3
39854	5.5047	549.73	119.571
39855	5.5048	577.661	131.683
39856	5.5048	556.465	264.018
39857	5.5048	516.185	255.81
39858	5.5049	578.516	80.0509
39859	5.5049	677.421	189.457
39860	5.5053	587.396	318.626
39861	5.5053	558.966	216.391
39862	5.5053	614.481	106.085
39863	5.5054	594.872	134.813
39864	5.5054	599.736	190.933
39865	5.5055	658.529	58.1622
39866	5.5059	579.661	276.118
39867	5.5059	594.308	206.411
39868	5.5059	460.473	177.008
39869	5.5059	573.026	253.991
39870	5.5061	571.701	49.1649
39871	5.5062	625.65	64.5965
39872	5.5063	631.197	10.7255
39873	5.5064	513.242	80.9968
39874	5.5064	484.028	46.1768
39875	5.5064	683.269	226.687
39876	5.5065	549.556	218.249
39877	5.5066	514.739	231.148

39878	5.5066	534.396	125.295
39879	5.5067	622.005	82.3781
39880	5.5068	569.1	27.2647
39881	5.5069	600.134	70.2337
39882	5.507	530.292	5.5915
39883	5.507	503.565	106.889
39884	5.5072	486.075	187.539
39885	5.5073	558.096	122.921
39886	5.5074	644.084	125.21
39887	5.5074	533.14	98.9519
39888	5.5075	567.725	58.0883
39889	5.5076	641.88	276.652
39890	5.5076	564.92	184.726
39891	5.5077	544.783	60.6786
39892	5.5077	432.075	181.881
39893	5.5079	493.734	151.049
39894	5.508	545.842	73.5535
39895	5.508	468.568	184.761
39896	5.5081	491.56	115.07
39897	5.5081	581.13	101.223
39898	5.5082	430.785	71.8753
39899	5.5082	575.383	68.5237
39900	5.5082	471.59	121.406
39901	5.5082	564.144	193.996
39902	5.5083	522.24	190.986
39903	5.5085	739.616	309.312
39904	5.5088	532.864	130.688
39905	5.5088	581.172	67.3971
39906	5.5092	551.816	127.764
39907	5.5096	525.874	356.862
39908	5.5096	535.142	166.394
39909	5.5097	659.994	23.5708
39910	5.51	504.074	198.478
39911	5.5102	618.173	66.7944
39912	5.5102	488.583	158.413
39913	5.5104	631.566	43.7974
39914	5.5108	637.072	21.2512
39915	5.5109	576.523	220.513
39916	5.511	514.29	276.253
39917	5.5113	683.02	62.5621
39918	5.5119	556.347	120.887
39919	5.5122	692.225	199.411
39920	5.5124	554.823	171.237
39921	5.5127	549.323	344.812
39922	5.5127	508.076	45.8661
39923	5.5128	550.079	39.0188
39924	5.5128	482.289	138.179
39925	5.513	525.904	170.504

39926	5.513	546.443	247.217
39927	5.5131	605.908	24.8365
39928	5.5132	470.098	219.23
39929	5.5132	540.074	352.131
39930	5.5132	600.428	51.9674
39931	5.5133	593.437	180.833
39932	5.5135	611.648	103.716
39933	5.5139	548.837	136.416
39934	5.5142	593.121	285.097
39935	5.5143	648.273	82.9065
39936	5.5145	532.803	198.916
39937	5.5146	653.847	242.688
39938	5.5146	545.105	322.125
39939	5.5146	532.643	130.1
39940	5.5147	581.779	192.97
39941	5.5148	591.473	168.613
39942	5.5149	585.8	246.627
39943	5.5149	609.77	63.0317
39944	5.5149	544.097	175.744
39945	5.515	608.031	190.499
39946	5.5151	690.889	257.871
39947	5.5152	542.458	259.757
39948	5.5152	75.2941	61.5722
39949	5.5152	538.109	103.46
39950	5.5154	576.409	105.014
39951	5.5154	555.383	235.966
39952	5.5155	546.958	93.3349
39953	5.5157	524.932	176.915
39954	5.5157	637.315	19.5277
39955	5.5157	643.372	19.7234
39956	5.5158	638.547	12.5514
39957	5.5159	545.551	209.717
39958	5.516	582.609	166.406
39959	5.516	442.666	133.975
39960	5.5163	488.784	220.74
39961	5.5163	639.856	17.0301
39962	5.5164	575.331	63.0324
39963	5.5164	671.066	53.3516
39964	5.5166	585.582	277.366
39965	5.5166	507.862	265.044
39966	5.5166	510.029	107.486
39967	5.5167	500.137	160.329
39968	5.5167	546.535	81.6649
39969	5.5167	631.939	118.495
39970	5.5169	521.625	126.504
39971	5.517	549.098	19.6663
39972	5.517	499.929	128.697
39973	5.5172	608.113	15.8946

39974	5.5173	586.915	96.446
39975	5.5175	681.222	29.0979
39976	5.5176	588.04	207.102
39977	5.5177	615.635	124.322
39978	5.5178	540.252	301.361
39979	5.5181	540.572	150.139
39980	5.5181	675.055	237.431
39981	5.5182	450.691	112.026
39982	5.5184	476.816	241.224
39983	5.5189	702.933	26.8906
39984	5.5194	480.862	234.934
39985	5.5195	517.891	188.795
39986	5.5195	472.221	104.023
39987	5.5196	726.644	46.6526
39988	5.5197	453.872	186.453
39989	5.5199	630.824	120.504
39990	5.5199	497.714	233.174
39991	5.5202	566.694	122.913
39992	5.5205	529.31	182.097
39993	5.5205	563.142	72.9293
39994	5.5205	384.13	43.0276
39995	5.5208	532.547	137.711
39996	5.5209	595.259	110.644
39997	5.5209	502.023	85.3137
39998	5.5209	519.683	93.2997
39999	5.5213	556.929	134.659
40000	5.5213	580.802	103.94
40001	5.5214	611.191	36.4579
40002	5.5214	641.628	38.8316
40003	5.5214	531.282	216.669
40004	5.5214	432.826	175.982
40005	5.5218	574.138	53.2486
40006	5.5219	570.318	89.298
40007	5.5221	584.701	79.5705
40008	5.5221	547.397	259.918
40009	5.5223	621.877	84.2234
40010	5.5223	530.036	210.819
40011	5.5223	607.44	104.781
40012	5.5226	511.746	193.226
40013	5.5227	566.489	161.72
40014	5.5229	449.377	169.532
40015	5.523	580.324	230.668
40016	5.523	623.939	104.672
40017	5.5231	551.197	129.373
40018	5.5233	449.869	333.702
40019	5.5233	641.385	198.538
40020	5.5234	645.593	85.2504
40021	5.5236	522.187	265.261

40022	5.5237	504.759	172.279
40023	5.5238	495.658	177.911
40024	5.5239	653.46	55.4459
40025	5.524	554.399	251.78
40026	5.5242	527.204	34.7508
40027	5.5242	504.708	190.53
40028	5.5244	531.81	157.457
40029	5.5244	585.078	7.663
40030	5.5247	559.012	191.941
40031	5.5247	473.83	170.521
40032	5.5248	514.651	72.4634
40033	5.5248	549.268	155.763
40034	5.5251	652.558	59.6955
40035	5.5254	594.819	300.727
40036	5.5254	643.582	30.1127
40037	5.5255	525.741	229.087
40038	5.5257	620.353	94.2091
40039	5.5259	547.574	252.876
40040	5.5259	538.055	212.354
40041	5.5259	578.676	109.018
40042	5.5265	602.283	88.1499
40043	5.5265	603.419	130.778
40044	5.5265	688.456	202.297
40045	5.5266	553.868	62.8715
40046	5.5268	511.92	235.501
40047	5.527	590.404	266.018
40048	5.5271	503.709	354.49
40049	5.5273	648.197	237.213
40050	5.5276	518.053	233.064
40051	5.5276	565.037	143.444
40052	5.5277	620.758	45.6198
40053	5.5278	551.473	184.518
40054	5.5279	660.31	59.8476
40055	5.5279	494.705	132.194
40056	5.5279	626.41	124.924
40057	5.528	556.912	228.641
40058	5.5281	531.684	75.7152
40059	5.5282	648.388	59.849
40060	5.5283	480.129	178.483
40061	5.5283	553.839	225.463
40062	5.5284	641.686	42.7404
40063	5.5285	493.401	205.277
40064	5.5286	471.139	173.82
40065	5.5286	517.366	191.897
40066	5.5288	613.358	58.1574
40067	5.5291	423.849	315.854
40068	5.5293	645.168	80.1734
40069	5.5293	460.568	102.407

40070	5.5295	453.577	75.8101
40071	5.5295	508.008	188.307
40072	5.5296	542.959	324.247
40073	5.5297	589.772	184.887
40074	5.5298	639.182	101.493
40075	5.5299	587.631	330.375
40076	5.5301	559.585	173.164
40077	5.5301	525.46	128.162
40078	5.5303	512.7	288.899
40079	5.5303	569.405	119.894
40080	5.5304	589.078	199.014
40081	5.5305	602.783	317.059
40082	5.5305	541.965	163.123
40083	5.5307	453.088	10.6716
40084	5.531	611.751	323.482
40085	5.531	469.712	220.663
40086	5.5311	567.759	103.811
40087	5.5313	672.729	42.4891
40088	5.5315	526.532	73.0378
40089	5.5315	539.326	60.3884
40090	5.5316	604.449	27.6089
40091	5.5316	640.071	18.6667
40092	5.5318	587.173	245.629
40093	5.5319	452.774	195.193
40094	5.532	538.058	253.42
40095	5.532	543.062	93.8691
40096	5.532	558.364	283.555
40097	5.5322	548.158	77.7673
40098	5.5323	493.243	231.872
40099	5.5324	648.688	72.6488
40100	5.5327	752.046	25.0589
40101	5.533	536.744	180.021
40102	5.533	483.085	26.0206
40103	5.5334	400.3	75.9169
40104	5.5334	651.513	18.1231
40105	5.5335	634.749	95.2403
40106	5.5335	654.84	241.561
40107	5.5335	444.338	154.363
40108	5.5336	491.612	6.9875
40109	5.5337	634.11	356.98
40110	5.5338	512.418	133.006
40111	5.5342	640.783	125.327
40112	5.5342	609.451	112.036
40113	5.5344	546.112	209.855
40114	5.5344	495.185	246.663
40115	5.5345	609.712	65.9829
40116	5.5346	687.426	208.359
40117	5.5347	502.899	221.171

40118	5.5348	507.754	76.4114
40119	5.5348	616.21	267.532
40120	5.5349	496.675	134.969
40121	5.5349	621.051	58.566
40122	5.535	577	75.9035
40123	5.5351	643.668	83.99
40124	5.5354	601.931	326.782
40125	5.5354	596.009	288.78
40126	5.5356	423.481	99.0496
40127	5.5356	573.746	38.7893
40128	5.5357	530.542	39.3037
40129	5.5358	595.121	242.04
40130	5.5362	489.973	70.156
40131	5.5364	532.897	3.5561
40132	5.5364	651.85	75.5244
40133	5.5365	516.534	122.991
40134	5.5366	615.31	21.0108
40135	5.5366	597.607	64.5867
40136	5.5367	625.791	93.397
40137	5.5367	542.665	295.08
40138	5.5368	497.577	146.735
40139	5.5368	625.308	59.3644
40140	5.537	662.561	37.982
40141	5.537	549.24	172.036
40142	5.537	456.915	146.531
40143	5.5371	674.074	132.54
40144	5.5371	658.681	61.2931
40145	5.5371	659.64	53.6822
40146	5.5376	625.702	58.5692
40147	5.5376	742.985	290.919
40148	5.5377	536.706	193.682
40149	5.5378	586.969	53.8846
40150	5.5381	584.868	80.1623
40151	5.5382	562.094	291.809
40152	5.5386	581.407	240.752
40153	5.5388	692.012	56.1846
40154	5.5389	510.271	133.323
40155	5.539	534.007	255.074
40156	5.5391	564.592	39.3112
40157	5.5394	568.661	165.294
40158	5.5397	566.145	154.109
40159	5.5399	593.239	137.825
40160	5.5401	630.951	83.0872
40161	5.5401	618.363	83.5247
40162	5.5401	563.438	116.435
40163	5.5407	552.273	22.7429
40164	5.5409	574.504	190.668
40165	5.5409	545.015	331.174

40166	5.5409	647.744	31.5997
40167	5.5411	665.862	63.6419
40168	5.5414	535.664	179.165
40169	5.5415	562.097	188.011
40170	5.5415	575.44	44.9416
40171	5.5419	481.848	199.248
40172	5.542	561.463	172.409
40173	5.5422	568.677	292.907
40174	5.5422	479.928	104.895
40175	5.5422	657.006	284.484
40176	5.5424	555.147	131.657
40177	5.5425	527.421	135.843
40178	5.5425	479.996	118.14
40179	5.5426	534.493	77.1883
40180	5.5426	541.984	157.843
40181	5.5426	657.297	51.0818
40182	5.5427	562.42	123.141
40183	5.5427	466.969	184.058
40184	5.5428	597.587	8.1766
40185	5.5434	547.629	75.1224
40186	5.5435	586.6	61.6402
40187	5.5437	507.839	276.302
40188	5.5437	388.192	305.18
40189	5.5438	656.61	29.8267
40190	5.544	523.858	264.178
40191	5.5441	521.639	176.721
40192	5.5442	621.22	47.9813
40193	5.5443	608.177	183.628
40194	5.5444	629.96	146.318
40195	5.5444	612.226	114.517
40196	5.5444	562.961	68.2519
40197	5.5445	573.089	29.4615
40198	5.5448	625.346	40.8298
40199	5.5452	547.756	126.696
40200	5.5454	483.546	268.931
40201	5.5456	658.775	56.6224
40202	5.5458	657.256	34.1991
40203	5.5458	617.601	43.7348
40204	5.5459	582.856	259.076
40205	5.5459	577.363	104.391
40206	5.5462	580.829	97.496
40207	5.5466	730.579	29.1938
40208	5.5466	546.98	118.271
40209	5.5468	445.044	278.05
40210	5.547	479.902	132.728
40211	5.547	559.736	264.804
40212	5.5471	641.474	99.3056
40213	5.5471	645.181	100.615

40214	5.5471	686.224	358.972
40215	5.5471	618.519	83.0488
40216	5.5473	720.789	41.936
40217	5.5474	598.018	29.1289
40218	5.5475	592.61	327.324
40219	5.5478	676.357	4.642
40220	5.5479	517.106	246.027
40221	5.5479	598.615	313.6
40222	5.5481	621.535	89.721
40223	5.5482	618.972	324.152
40224	5.5482	488.904	268.667
40225	5.5483	563.984	173.149
40226	5.5484	661.54	24.2025
40227	5.5485	549.174	139.861
40228	5.5489	606.787	170.684
40229	5.5493	581.178	264.929
40230	5.5493	571.283	87.0227
40231	5.5495	573.459	61.5811
40232	5.5498	476.484	69.3461
40233	5.55	611.137	326.375
40234	5.55	530.789	330.688
40235	5.5503	519.3	131.433
40236	5.5504	501.289	220.39
40237	5.5507	608.453	292.664
40238	5.5507	590.24	65.4251
40239	5.5508	591.117	21.9784
40240	5.5509	689.422	76.1657
40241	5.5511	490.425	182.792
40242	5.5512	476.385	146.726
40243	5.5515	707.901	40.0842
40244	5.5516	576.531	91.9608
40245	5.5516	478.23	180.627
40246	5.5521	541.111	65.5637
40247	5.5523	576.406	158.327
40248	5.5524	631.332	318.081
40249	5.5524	511.617	267.87
40250	5.5525	511.908	119.479
40251	5.5525	585.464	139.254
40252	5.5526	629.981	55.9631
40253	5.5526	571.107	274.795
40254	5.5526	634.193	61.0181
40255	5.5526	583.832	313.871
40256	5.5531	529.633	99.4055
40257	5.5531	657.581	344.027
40258	5.5533	532.854	251.243
40259	5.5534	572.416	167.555
40260	5.5535	569.909	174.574
40261	5.5535	485.793	193.065

40262	5.5535	96.5703	121.351
40263	5.5535	523.826	120.328
40264	5.5535	514.952	98.1808
40265	5.5535	579.379	306.108
40266	5.5536	447.689	278.075
40267	5.5537	560.889	130.317
40268	5.5539	536.336	147.314
40269	5.5539	564.188	71.6215
40270	5.554	619.712	87.0109
40271	5.5546	559.853	136.476
40272	5.5548	489.113	75.3079
40273	5.5549	558.871	179.396
40274	5.5549	592.207	110.041
40275	5.5554	474.669	279.297
40276	5.5559	658.134	45.7649
40277	5.556	657.289	254.157
40278	5.5561	624.513	60.4888
40279	5.5563	552.041	117.677
40280	5.5563	563.414	100.066
40281	5.5564	500.111	291.244
40282	5.5564	493.682	58.3872
40283	5.5566	582.32	52.0299
40284	5.5566	571.899	169.722
40285	5.5566	523.641	133.254
40286	5.5567	642.07	17.546
40287	5.5567	604.705	85.1663
40288	5.5567	591.343	97.2058
40289	5.5569	489.885	157.28
40290	5.557	586.514	320.038
40291	5.5571	491.221	12.6198
40292	5.5571	507.319	69.0157
40293	5.5572	680.486	22.8167
40294	5.5572	532.93	65.3186
40295	5.5578	506.986	14.7686
40296	5.558	557.647	189.61
40297	5.5582	569.932	156.232
40298	5.5583	658.781	178.65
40299	5.5585	-7.7484	73.3528
40300	5.5585	670.877	35.8713
40301	5.5586	525.846	321.606
40302	5.5586	525.708	182.669
40303	5.5588	585.46	44.1521
40304	5.5589	611.567	158.981
40305	5.559	516.586	326.253
40306	5.5597	544.419	98.9807
40307	5.5598	-2.7951	79.0815
40308	5.5598	659.632	28.6071
40309	5.5599	598.355	61.8872

40310	5.5604	548.456	307.462
40311	5.5606	527.095	101.702
40312	5.5608	595.445	33.2502
40313	5.5611	579.968	229.815
40314	5.5613	662.267	257.697
40315	5.5614	552.693	170.314
40316	5.5614	556.763	276.568
40317	5.5615	462.311	221.546
40318	5.5616	569.779	228.457
40319	5.5616	476.148	154.887
40320	5.5618	463.793	160.141
40321	5.5618	600.223	104.119
40322	5.5619	538.161	176.014
40323	5.5619	677.133	299.126
40324	5.5622	535.497	197.397
40325	5.5623	586.272	51.6419
40326	5.5624	523.099	119.125
40327	5.5625	530.149	183.638
40328	5.5626	571.211	81.3877
40329	5.5626	470.36	131.017
40330	5.5629	441.324	278.204
40331	5.563	571.619	26.8698
40332	5.5631	570.715	105.937
40333	5.5631	559.041	88.2563
40334	5.5631	606.803	301.005
40335	5.5632	446.746	281.222
40336	5.5633	662.342	24.0086
40337	5.5636	555.636	146.139
40338	5.5638	698.52	15.4425
40339	5.5639	642.983	287.835
40340	5.5642	577.481	235.624
40341	5.5642	444.225	344.933
40342	5.5642	526.204	242.373
40343	5.5642	520.116	194.102
40344	5.5644	536.871	97.2913
40345	5.5645	586.412	114.981
40346	5.5645	554.06	177.253
40347	5.5647	630.438	85.4448
40348	5.5648	548.341	129.467
40349	5.5649	473.635	220.095
40350	5.5649	478.541	23.9113
40351	5.5649	636.727	155.643
40352	5.5652	576.127	51.0563
40353	5.5653	708.976	101.932
40354	5.5653	627.445	10.4941
40355	5.5654	609.626	230.352
40356	5.5654	713.214	203.487
40357	5.5655	634.778	61.0442

40358	5.5657	463.93	187.343
40359	5.5658	613.422	80.5247
40360	5.5658	719.056	55.8065
40361	5.5661	505.53	138.304
40362	5.5665	709.093	48.716
40363	5.5667	557.076	163.632
40364	5.5668	593.207	127.124
40365	5.5672	482.057	173.81
40366	5.5672	622.578	62.5914
40367	5.5676	683.879	60.407
40368	5.568	511.112	159.558
40369	5.5682	711.234	41.3948
40370	5.5683	592.239	19.0757
40371	5.5683	504.418	120.603
40372	5.5684	593.308	35.8395
40373	5.5686	500.375	238.31
40374	5.5687	552.714	74.609
40375	5.5688	550.377	215.644
40376	5.5688	557.485	244.038
40377	5.5689	516.864	191.782
40378	5.5691	718.549	23.8559
40379	5.5692	621.088	104.53
40380	5.5693	541.813	301.831
40381	5.5694	662.721	32.3533
40382	5.5695	533.463	261.207
40383	5.5697	446.796	164.475
40384	5.5698	594.564	274.897
40385	5.5698	570.544	140.308
40386	5.5699	712.876	29.4971
40387	5.5701	584.271	127.788
40388	5.5706	627.769	195.785
40389	5.5707	563.34	177.687
40390	5.5708	473.184	10.5677
40391	5.5713	552.598	89.2194
40392	5.5714	716.616	47.6972
40393	5.5715	465.838	173.492
40394	5.5716	649.151	74.6489
40395	5.5717	645.738	36.4276
40396	5.5718	669.398	64.4136
40397	5.5718	571.091	332.187
40398	5.5718	534.138	68.0655
40399	5.5719	558.281	208.614
40400	5.5721	502.477	119.356
40401	5.5725	664.11	65.91
40402	5.5725	474.026	156.691
40403	5.5727	587.601	113.893
40404	5.5728	667.005	31.648
40405	5.5729	487.908	189.183

40406	5.5729	500.351	140.442
40407	5.573	561.485	196.245
40408	5.573	548.92	90.863
40409	5.5731	513.102	193.467
40410	5.5731	571.999	113.686
40411	5.5732	505.825	220.796
40412	5.5732	593.435	69.3277
40413	5.5733	514.481	196.67
40414	5.5735	616.752	330.72
40415	5.5738	675.683	77.0325
40416	5.5739	489.286	171.231
40417	5.5742	624.631	187.714
40418	5.5742	648.784	72.6326
40419	5.5742	649.47	40.2666
40420	5.5745	596.502	141.121
40421	5.5745	618.283	23.6662
40422	5.5746	666.266	66.3154
40423	5.575	640.049	113.487
40424	5.5751	616.758	141.882
40425	5.5752	499.926	259.997
40426	5.5753	483.908	183.285
40427	5.5753	466.38	107.102
40428	5.5754	619.062	71.8
40429	5.5755	572.95	35.1742
40430	5.5755	542.23	107.614
40431	5.5756	644.397	18.8713
40432	5.5757	541.022	257.393
40433	5.5757	645.437	81.1675
40434	5.5759	585.746	120.053
40435	5.576	604.548	238.852
40436	5.5761	645.84	83.9876
40437	5.5762	597.295	260.394
40438	5.5763	544.505	293.399
40439	5.5763	497.778	172.769
40440	5.5765	661.433	72.6188
40441	5.5765	604.909	129.77
40442	5.5765	568.981	120.476
40443	5.5766	553.914	21.1587
40444	5.5767	650.345	52.6397
40445	5.5767	609.092	292.562
40446	5.577	524.379	83.2325
40447	5.5773	559.121	90.6172
40448	5.5774	594.986	101.56
40449	5.5774	577.701	104.444
40450	5.5777	541.269	164.012
40451	5.5778	632.039	226.101
40452	5.578	477.881	218.17
40453	5.5781	570.222	216.523

40454	5.5781	564.443	119.161
40455	5.5781	624.635	78.3245
40456	5.5782	664.61	72.1833
40457	5.5784	389.41	21.0386
40458	5.5786	619.977	83.1082
40459	5.5786	731.637	21.4765
40460	5.5788	763.459	101.67
40461	5.5789	543.045	144.626
40462	5.5792	645.869	303.582
40463	5.5792	557.657	168.652
40464	5.5794	641.279	58.3833
40465	5.5796	529.714	106.706
40466	5.5797	702.335	63.5468
40467	5.58	564.375	89.14
40468	5.5803	506.039	79.4481
40469	5.5803	639.153	140.279
40470	5.5805	530.96	78.3224
40471	5.5806	553.054	320.785
40472	5.5809	719.859	56.4148
40473	5.5809	576.693	288.453
40474	5.581	592.657	66.8064
40475	5.581	707.039	57.0429
40476	5.5811	605.447	54.4625
40477	5.5813	641.372	65.6294
40478	5.5813	730.108	33.6085
40479	5.5814	606.488	96.4959
40480	5.5816	636.193	33.4297
40481	5.5817	493.238	20.6071
40482	5.5821	594.833	72.7972
40483	5.5821	486.268	138.192
40484	5.5822	606.485	271.052
40485	5.5824	588.44	142.1
40486	5.5826	570.259	80.8621
40487	5.5828	585.587	25.0746
40488	5.5828	561.42	122.858
40489	5.583	607.471	83.1096
40490	5.583	450.611	136.799
40491	5.583	654.428	56.9314
40492	5.5831	517.747	121.348
40493	5.5832	638.988	85.8495
40494	5.5832	407.093	189.692
40495	5.5833	674.051	65.173
40496	5.5835	603.56	294.986
40497	5.5835	496.334	220.901
40498	5.5835	623.833	78.0423
40499	5.5837	649.645	105.885
40500	5.5838	549.751	198.098
40501	5.5839	629.91	53.5475

40502	5.5843	578.677	152.36
40503	5.5845	634.824	91.8379
40504	5.5845	566.702	163.215
40505	5.5847	549.447	109.281
40506	5.5847	663.902	117.368
40507	5.5849	585.497	233.311
40508	5.5849	585.08	280.796
40509	5.5849	587.487	318.37
40510	5.585	707.216	242.627
40511	5.5853	540.81	281.676
40512	5.5853	690.007	26.7998
40513	5.5855	554.499	218.408
40514	5.5855	506.808	171.49
40515	5.5855	650.484	44.1515
40516	5.5857	692.432	33.5634
40517	5.5859	735.531	213.085
40518	5.586	653.247	126.616
40519	5.5862	667.681	68.5509
40520	5.5863	602.903	228.535
40521	5.5864	558.055	151.974
40522	5.5865	569.699	193.806
40523	5.5866	640.247	46.5396
40524	5.5866	557.743	196.497
40525	5.5868	659.493	286.125
40526	5.5869	600.731	73.5906
40527	5.587	663.549	129.39
40528	5.5871	728.385	51.6885
40529	5.5871	533.066	78.7595
40530	5.5873	665.547	297.587
40531	5.5875	681.915	42.1285
40532	5.5876	264.301	205.001
40533	5.5878	513.096	177.441
40534	5.588	631.023	329.078
40535	5.5881	664.545	225.249
40536	5.5881	691.468	44.1374
40537	5.5882	611.972	62.9235
40538	5.5885	575.46	180.038
40539	5.5885	441.594	230.646
40540	5.5886	602.966	89.631
40541	5.5886	435.254	187.861
40542	5.5887	550.619	109.12
40543	5.5889	590.445	94.6533
40544	5.5892	600.214	232.277
40545	5.5894	674.787	60.8412
40546	5.5894	94.9017	58.5117
40547	5.5894	595.292	85.1795
40548	5.5897	643.626	72.5701
40549	5.5897	515.289	49.9982

40550	5.5898	530.277	337.507
40551	5.5899	524.344	220.948
40552	5.5902	726.91	50.1306
40553	5.5903	548.307	287.13
40554	5.5905	624.514	80.1818
40555	5.5905	618.073	54.1144
40556	5.5905	559.991	77.0896
40557	5.5906	663.405	54.2629
40558	5.5908	656.554	25.076
40559	5.5914	489.923	182.326
40560	5.5916	584.679	153.932
40561	5.5917	606.494	66.6741
40562	5.5918	525.336	144.363
40563	5.5919	621.627	44.1513
40564	5.5919	605.219	109.183
40565	5.5919	569.001	197.547
40566	5.5921	599.439	125.226
40567	5.5922	606.233	102.924
40568	5.5922	544.097	105.771
40569	5.5923	513.762	218.607
40570	5.5925	608.147	182.412
40571	5.5925	689.895	73.085
40572	5.5925	594.155	101.793
40573	5.5925	477.191	198.078
40574	5.5927	591.373	104.166
40575	5.5928	628.889	224.658
40576	5.5929	634.793	120.914
40577	5.593	563.351	296.023
40578	5.593	534.599	97.5307
40579	5.5933	567.101	233.675
40580	5.5933	522.216	89.4545
40581	5.5933	605.75	134.397
40582	5.5934	681.698	31.7827
40583	5.5935	522.105	180.83
40584	5.5935	693.717	31.5127
40585	5.5935	652.798	104.021
40586	5.5936	559.043	173.255
40587	5.5937	608.076	87.1991
40588	5.5937	728.02	329.44
40589	5.5941	542.522	152.685
40590	5.5943	702.405	62.631
40591	5.5946	608.606	125.263
40592	5.595	499.76	124.936
40593	5.5951	621.194	54.6545
40594	5.5953	561.813	79.6273
40595	5.5954	591.577	38.4925
40596	5.5957	624.852	111.872
40597	5.5958	664.805	103.133

40598	5.5959	490.618	75.9588
40599	5.5959	598.416	74.1032
40600	5.5959	616.995	299.725
40601	5.596	604.343	273.388
40602	5.596	618.37	122.03
40603	5.596	424.531	173.339
40604	5.5963	584.552	322.696
40605	5.5965	582.561	29.5524
40606	5.5966	577.612	100.72
40607	5.597	555.99	153.722
40608	5.597	501.493	185.176
40609	5.5972	613.402	94.3791
40610	5.5972	636.033	135.017
40611	5.5972	716.957	201.43
40612	5.5974	573.025	52.3576
40613	5.5977	575.068	226.146
40614	5.5978	491.079	249.915
40615	5.5979	564.15	144.253
40616	5.5984	734.464	45.8263
40617	5.5985	525.285	141.665
40618	5.5988	540.845	182.431
40619	5.5988	582.566	163.736
40620	5.5988	585.638	196.46
40621	5.5989	529.363	127.899
40622	5.5989	467.108	70.4543
40623	5.599	756.518	27.9681
40624	5.5992	552.088	220.002
40625	5.5992	626.984	67.8656
40626	5.5993	561.937	179.647
40627	5.5995	638.686	337.708
40628	5.5995	718.312	7.403
40629	5.5999	584.742	286.019
40630	5.6002	573.437	174.771
40631	5.6002	613.296	56.6193
40632	5.6003	658.534	39.988
40633	5.6004	587.999	185.583
40634	5.6005	672.988	94.7097
40635	5.6006	579.333	102.914
40636	5.6007	564.418	158.116
40637	5.6008	614.057	66.3977
40638	5.6008	516.313	146.23
40639	5.6012	687.5	24.6709
40640	5.6013	615.196	86.8402
40641	5.6014	695.211	226.441
40642	5.6015	558.196	118.67
40643	5.6015	526.135	192.887
40644	5.6017	715.977	28.1265
40645	5.6017	665.508	10.7669

40646	5.6017	571.201	282.559
40647	5.6019	507.148	125.982
40648	5.6021	611.921	164.194
40649	5.6022	727.593	57.8711
40650	5.6022	685.629	32.4932
40651	5.6023	660.026	45.3063
40652	5.6024	483.224	176.357
40653	5.603	641.21	33.7625
40654	5.6032	643.445	190.341
40655	5.6032	554.376	72.7356
40656	5.6033	590.326	182.266
40657	5.6033	547.395	185.265
40658	5.6034	667.881	38.5395
40659	5.6035	466.052	257.713
40660	5.6036	713.316	102.184
40661	5.6037	509.866	124.066
40662	5.6037	704.894	40.1385
40663	5.6038	547.094	218.934
40664	5.604	628.188	186.638
40665	5.604	561.658	190.394
40666	5.604	566.255	74.5906
40667	5.6041	560.611	190.738
40668	5.6041	545.723	78.14
40669	5.6046	686.473	32.9369
40670	5.6047	548.677	117.667
40671	5.6047	648.684	190.555
40672	5.6048	533.16	178.203
40673	5.6048	493.726	223.052
40674	5.6049	556.386	43.4438
40675	5.605	642.577	92.88
40676	5.6051	494.272	149.318
40677	5.6051	590.818	186.204
40678	5.6052	619.042	332.172
40679	5.6052	633.088	271.38
40680	5.6053	592.103	355.68
40681	5.6054	680.324	86.2751
40682	5.6055	535.278	115.741
40683	5.6055	488.285	197.078
40684	5.6056	608.803	84.8954
40685	5.6058	573.703	152.437
40686	5.6058	429.421	170.392
40687	5.6059	576.548	29.5773
40688	5.606	453.851	75.3139
40689	5.6062	386.569	257.783
40690	5.6066	714.523	15.606
40691	5.6067	557.855	88.9446
40692	5.6068	609.993	136.838
40693	5.6068	562.02	178.667

40694	5.6068	616.459	93.748
40695	5.607	579.611	128.296
40696	5.6074	587.307	181.675
40697	5.6077	541.787	181.502
40698	5.6077	710.345	15.1909
40699	5.6078	594.37	124.65
40700	5.608	490.182	225.401
40701	5.6081	566.214	287.291
40702	5.6082	705.862	106.177
40703	5.6083	590.569	212.795
40704	5.6084	546.692	223.646
40705	5.6084	590.621	173.018
40706	5.6085	575.853	195.481
40707	5.6086	590.819	142.265
40708	5.6086	657.565	28.1306
40709	5.6087	536.82	59.1767
40710	5.6088	532.476	283.085
40711	5.6089	434.515	179.551
40712	5.609	606.404	50.8564
40713	5.6094	573.907	98.4611
40714	5.6094	505.189	195.35
40715	5.6094	552.413	159.705
40716	5.6095	575.382	147.461
40717	5.6096	572.73	126.644
40718	5.6098	569.802	69.34
40719	5.6098	593.658	117.856
40720	5.61	774.437	26.7671
40721	5.61	494.464	68.0393
40722	5.6103	545.81	44.4009
40723	5.6103	614.919	13.6113
40724	5.6105	567.695	32.8795
40725	5.6109	480.363	145.759
40726	5.6109	690.986	42.5002
40727	5.611	681.246	22.2246
40728	5.6111	601.823	176.155
40729	5.6111	487.239	191.403
40730	5.6111	562.67	144.301
40731	5.6113	568.711	145.66
40732	5.6114	628.849	111.733
40733	5.6116	518.399	72.6146
40734	5.6117	670.367	151.737
40735	5.6117	578.063	159.55
40736	5.6118	575.03	120.924
40737	5.6119	545.664	326.823
40738	5.6119	678.619	46.7633
40739	5.6121	615.605	138.749
40740	5.6123	560.75	54.838
40741	5.6124	609.901	65.5002

40742	5.6126	548.522	275.04
40743	5.6127	606.277	34.4855
40744	5.613	765.938	82.7579
40745	5.6131	627.831	40.193
40746	5.6131	642.321	46.3257
40747	5.6135	597.652	165.925
40748	5.6136	573.45	39.0917
40749	5.6139	554.766	233.342
40750	5.6143	588.622	162.343
40751	5.6143	534.047	184.409
40752	5.6143	675.66	60.4752
40753	5.6143	517.704	37.0189
40754	5.6143	706.983	10.1636
40755	5.6144	691.532	62.381
40756	5.6145	641.567	299.048
40757	5.6147	482.619	297.416
40758	5.6149	791.209	30.1695
40759	5.615	653.446	16.5628
40760	5.6151	485.871	178.453
40761	5.6153	616.512	269.842
40762	5.6153	544.843	331.444
40763	5.6153	470.542	112.813
40764	5.6155	606.904	177.817
40765	5.6159	525.235	146.817
40766	5.616	609.667	8.3378
40767	5.6163	736.032	288.856
40768	5.6164	639.166	76.0156
40769	5.6165	629.79	174.484
40770	5.6165	663.684	82.0851
40771	5.6166	552.162	79.3254
40772	5.6168	603.154	139.737
40773	5.6169	555.935	146.026
40774	5.6172	599.828	61.0834
40775	5.6174	632.66	307.692
40776	5.6175	528.452	186.263
40777	5.6175	601.53	77.3557
40778	5.6177	603.265	41.7603
40779	5.6179	661.715	91.2968
40780	5.6179	550.382	219.929
40781	5.6181	528.098	110.334
40782	5.6182	590.942	100.887
40783	5.6183	596.056	218.969
40784	5.6188	520.854	92.7599
40785	5.6188	524.063	75.0846
40786	5.6192	383.74	24.8017
40787	5.6192	651.792	66.2621
40788	5.6192	558.108	236.14
40789	5.6192	661.008	63.3277

40790	5.6194	691.678	116.933
40791	5.6196	644.879	23.395
40792	5.6196	714.969	107.136
40793	5.6198	535.907	260.796
40794	5.6199	621.722	98.9015
40795	5.6199	527.833	86.8634
40796	5.62	576.324	216.414
40797	5.6201	591.877	177.152
40798	5.6205	566.894	39.3393
40799	5.6205	748.41	44.6561
40800	5.6206	604.348	140.116
40801	5.6207	558.668	281.502
40802	5.621	512.543	171.094
40803	5.621	505.439	264.922
40804	5.621	625.511	80.4881
40805	5.6212	550.927	222.289
40806	5.6212	531.376	154.75
40807	5.6213	513.005	224.359
40808	5.6213	619.05	240.983
40809	5.6217	591.043	92.4833
40810	5.6218	630.637	116.158
40811	5.6218	590.856	27.762
40812	5.6218	705.037	23.7553
40813	5.6219	532.92	178.513
40814	5.6221	655.43	97.2768
40815	5.6221	650.049	285.654
40816	5.6221	595.08	21.236
40817	5.6222	575.497	56.3504
40818	5.6224	696.216	44.2056
40819	5.6227	570.057	281.654
40820	5.623	651.976	102.246
40821	5.6231	613.767	139.877
40822	5.6234	626.28	38.9236
40823	5.6234	686.931	26.797
40824	5.6235	611.965	98.02
40825	5.6236	592.597	119.122
40826	5.6237	585.032	193.858
40827	5.6239	661.446	29.8421
40828	5.6239	574.585	229.331
40829	5.6242	661.732	40.426
40830	5.6242	592.19	231.039
40831	5.6243	527.78	80.1577
40832	5.6243	593.864	182.557
40833	5.6245	592.24	178.9
40834	5.6246	542.392	255.477
40835	5.6246	649.002	96.9902
40836	5.6247	710.599	57.9483
40837	5.6249	590.251	108.331

40838	5.625	726.674	59.9688
40839	5.6252	568.364	135.093
40840	5.6254	608.39	0.2265
40841	5.6257	626.951	15.3373
40842	5.6258	593.562	212.438
40843	5.626	522.35	128.285
40844	5.6261	575.571	176.419
40845	5.6262	526.91	89.898
40846	5.6263	487.293	163.6
40847	5.6263	585.884	82.4761
40848	5.6266	677.649	263.649
40849	5.6267	579.895	174.468
40850	5.6269	598.992	37.0524
40851	5.627	674.666	79.1718
40852	5.6271	576.961	172.344
40853	5.6274	685.254	20.2539
40854	5.6274	499.848	248.809
40855	5.6275	651.473	97.171
40856	5.6275	618.422	202.782
40857	5.6277	645.283	226.403
40858	5.6277	537.375	97.8001
40859	5.6279	640.713	104.116
40860	5.6279	649.107	106.488
40861	5.6279	605.113	139.186
40862	5.6281	567.697	205.867
40863	5.6284	557.427	177.725
40864	5.6287	627.392	208.274
40865	5.6288	647.951	39.4166
40866	5.6288	607.4	347.815
40867	5.6293	438.343	214.816
40868	5.6293	570.07	35.6334
40869	5.6294	545.597	324.33
40870	5.6296	595.87	103.924
40871	5.6297	630.617	177.067
40872	5.6297	507.645	325.382
40873	5.6297	642.563	99.9717
40874	5.6298	632.38	293.302
40875	5.63	568.574	190.806
40876	5.6301	531.659	255.13
40877	5.6301	673.253	102.402
40878	5.6301	675.831	43.4177
40879	5.6303	516.025	322.678
40880	5.6303	619.716	187.952
40881	5.6305	688.132	106.772
40882	5.6306	593.815	39.8698
40883	5.6306	708.376	77.3105
40884	5.6308	502.573	188.657
40885	5.6309	657.419	238.921

40886	5.631	675.161	102.249
40887	5.6311	477.578	233.216
40888	5.6313	596.943	128.182
40889	5.6314	580.704	20.351
40890	5.6321	590.357	183.439
40891	5.6322	586.419	33.8135
40892	5.6323	610.598	276.404
40893	5.6323	634.211	104.332
40894	5.6323	493.569	130.869
40895	5.6325	658.031	154.899
40896	5.6325	547.285	219.396
40897	5.6327	644.873	244.633
40898	5.6329	716.585	62.5362
40899	5.633	617.668	301.669
40900	5.6331	584.57	112.8
40901	5.6332	703.747	62.3025
40902	5.6332	683.285	304.639
40903	5.6332	484.162	199.844
40904	5.6335	670.797	34.5543
40905	5.6338	537.418	140.161
40906	5.6339	733.201	13.9525
40907	5.634	529.976	175.584
40908	5.634	622.042	24.1911
40909	5.634	712.027	208.795
40910	5.6343	618.872	294.406
40911	5.6344	585.085	135.286
40912	5.6346	589.35	82.5493
40913	5.6347	619.864	115.747
40914	5.6351	661.396	50.9623
40915	5.6353	530.364	221.127
40916	5.6354	712.928	32.4215
40917	5.6356	587.834	56.0406
40918	5.6358	540.945	235.103
40919	5.6359	619.201	138.54
40920	5.636	592.153	334.01
40921	5.6361	586.123	133.895
40922	5.6362	614.09	332.051
40923	5.6365	509.006	182.972
40924	5.6367	579.069	223.419
40925	5.6367	649.896	57.2369
40926	5.6367	715.954	31.6248
40927	5.6368	580.674	168.097
40928	5.6369	566.019	108.629
40929	5.6369	634.273	69.4478
40930	5.6371	707.676	54.9931
40931	5.6373	630.83	61.8149
40932	5.6373	576.371	218.558
40933	5.6373	600.384	149.319

40934	5.6376	436.122	134.619
40935	5.6382	555.226	120.791
40936	5.6383	775.071	75.139
40937	5.6385	450.367	184.751
40938	5.6386	551.385	176.331
40939	5.6387	559.711	352.725
40940	5.6387	521.624	134.568
40941	5.639	632.73	288.568
40942	5.6394	606.57	115.825
40943	5.6394	607.765	89.6531
40944	5.6395	438.505	129.526
40945	5.6395	648.846	77.6964
40946	5.6396	616.68	12.5175
40947	5.6397	614.707	135.495
40948	5.6397	696.075	57.0495
40949	5.6398	514.741	190.393
40950	5.6399	669.278	208.515
40951	5.64	599.309	119.017
40952	5.6401	508.372	195.975
40953	5.6405	552.285	182.645
40954	5.6405	579.468	156.248
40955	5.6406	593.558	182.937
40956	5.6406	603.148	58.8061
40957	5.6406	536.653	344.183
40958	5.6408	675.325	58.2637
40959	5.641	638.328	229.888
40960	5.6411	588.623	260.933
40961	5.6412	559.211	165.373
40962	5.6414	648.414	85.5151
40963	5.6415	590.559	132.55
40964	5.6418	563.842	335.967
40965	5.6418	614.765	81.735
40966	5.6418	742.03	48.9885
40967	5.6422	676.541	52.3379
40968	5.6423	599.069	131.548
40969	5.6424	657.154	207.945
40970	5.6424	587.72	146.405
40971	5.6426	612.276	124.437
40972	5.6426	597.238	202.136
40973	5.6429	740.769	19.6029
40974	5.643	616.422	70.731
40975	5.6431	715.33	21.1263
40976	5.6432	649.923	229.044
40977	5.6433	610.375	41.637
40978	5.6437	681.657	37.7659
40979	5.6438	621.91	88.7208
40980	5.6439	725.391	64.2191
40981	5.644	536.05	28.9927

40982	5.644	693.645	208.476
40983	5.6441	543.955	132.574
40984	5.6442	640.535	78.915
40985	5.6443	618.97	142.254
40986	5.6445	689.564	311.657
40987	5.6446	539.265	353.118
40988	5.6448	646.574	60.7863
40989	5.6449	630.54	57.096
40990	5.6449	533.568	126.206
40991	5.6451	532.807	327.775
40992	5.6452	639.309	302.434
40993	5.6452	632.602	174.003
40994	5.6455	579.687	272.479
40995	5.6457	524.451	168.689
40996	5.6459	534.092	66.656
40997	5.6459	624.421	197.495
40998	5.6459	712.612	43.7387
40999	5.646	672.785	48.1043
41000	5.6461	623.327	271.724
41001	5.6464	576.425	256.963
41002	5.6466	752.177	50.6705
41003	5.6468	624.632	89.2941
41004	5.6469	-4.0714	256.115
41005	5.647	595.304	67.7032
41006	5.6472	601.409	146.304
41007	5.6472	540.932	119.208
41008	5.6473	721.284	56.5752
41009	5.648	615.465	325.072
41010	5.648	717.671	33.5307
41011	5.648	557.787	118.452
41012	5.6481	594.858	162.354
41013	5.6481	611.243	103.293
41014	5.6483	604.621	141.989
41015	5.6484	679.613	92.6784
41016	5.6484	547.275	49.1669
41017	5.6485	511.755	264.138
41018	5.6486	579.616	26.3702
41019	5.6486	663.688	282.331
41020	5.6487	643.086	4.2692
41021	5.6487	731.37	45.621
41022	5.6488	636.647	106.155
41023	5.6488	539.922	190.06
41024	5.6489	511.206	177.125
41025	5.6489	710.219	331.836
41026	5.6493	610.387	149.541
41027	5.6493	639.275	192.575
41028	5.6494	494.932	152.928
41029	5.6495	562.1	250.158

41030	5.6497	633.136	118.307
41031	5.6498	683.855	200.206
41032	5.6499	468.128	160.941
41033	5.65	537.602	56.4409
41034	5.6502	617.197	281.831
41035	5.6504	780.102	50.1396
41036	5.6505	531.504	235.278
41037	5.6506	558.612	45.3686
41038	5.6507	712.769	14.3253
41039	5.6508	510.904	182.558
41040	5.6509	573.282	78.4027
41041	5.651	517.041	124.465
41042	5.6512	639.007	49.7174
41043	5.6518	634.443	215.29
41044	5.6519	597.771	75.2052
41045	5.6523	525.684	112.811
41046	5.6523	669.436	354.903
41047	5.6525	609.178	108.153
41048	5.6527	576.045	182.178
41049	5.6527	430.232	65.5251
41050	5.6527	637.633	359.079
41051	5.6527	701.171	199.346
41052	5.6529	485.088	258.095
41053	5.6529	713.856	60.4235
41054	5.653	675.117	242.886
41055	5.6531	677.294	38.0313
41056	5.6531	523.818	172.994
41057	5.6532	552.592	296.723
41058	5.6533	627.219	113.253
41059	5.6534	620.128	217.574
41060	5.6534	580.965	100.528
41061	5.6534	476.728	85.0486
41062	5.6534	615.881	167.797
41063	5.6535	671.53	28.2683
41064	5.6535	698.122	16.1666
41065	5.6537	657.657	155.329
41066	5.6537	585.951	120.44
41067	5.6537	779.763	20.8883
41068	5.6538	525.374	56.99
41069	5.6539	613.885	123.471
41070	5.654	546.354	177.028
41071	5.654	655.345	103.044
41072	5.654	524.786	7.1589
41073	5.6541	602.082	98.123
41074	5.6542	680.343	106.02
41075	5.6543	519.573	31.3138
41076	5.6545	611.153	42.2955
41077	5.6549	622.155	293.402

41078	5.6549	581.803	81.7938
41079	5.6549	702.641	16.244
41080	5.655	740.949	54.4047
41081	5.6551	572.204	138.135
41082	5.6551	619.34	117.807
41083	5.6551	582.724	84.0414
41084	5.6554	605.97	68.2142
41085	5.6555	558.456	78.4446
41086	5.6556	619.488	115.354
41087	5.6557	696.167	67.6478
41088	5.6557	557.642	145.416
41089	5.6558	714.52	62.135
41090	5.6558	641.748	107.592
41091	5.6558	601.289	138.115
41092	5.656	703.646	58.6012
41093	5.6561	575.63	43.0406
41094	5.6562	659.118	76.5998
41095	5.6562	594.78	148.474
41096	5.6563	692.293	114.394
41097	5.6564	601.596	25.4305
41098	5.6565	599.39	160.87
41099	5.6565	507.18	126.832
41100	5.6567	525.224	80.2706
41101	5.6568	458.232	213.828
41102	5.6568	579.184	275.114
41103	5.657	528.662	153.693
41104	5.6576	642.881	54.264
41105	5.6576	589.277	74.2615
41106	5.658	488.297	19.9728
41107	5.6581	651.807	169.638
41108	5.6581	584.723	114.548
41109	5.6582	644.297	75.1676
41110	5.6582	600.107	111.445
41111	5.6589	657.725	35.9808
41112	5.6593	641.676	42.054
41113	5.6593	592.194	164.355
41114	5.6595	648.255	52.9071
41115	5.6597	518.747	111.946
41116	5.6598	611.292	246.062
41117	5.6598	634.261	235.908
41118	5.6602	584.217	242.985
41119	5.6602	506.399	190.038
41120	5.6604	683.354	289.37
41121	5.6605	527.635	278.076
41122	5.6605	578.328	184.468
41123	5.6605	714.534	15.0216
41124	5.6606	613.476	123.794
41125	5.6606	554.325	76.2215

41126	5.6608	661.94	127.146
41127	5.661	688.197	32.2556
41128	5.6611	589.497	113.188
41129	5.6615	591.88	105.444
41130	5.6616	531.295	56.3676
41131	5.6618	793.126	102.104
41132	5.6618	536.019	171.198
41133	5.6619	545.48	219.899
41134	5.662	626.424	293.616
41135	5.6622	554.321	145.688
41136	5.6624	578.217	63.0381
41137	5.6624	610.047	101.782
41138	5.6624	658.724	50.3979
41139	5.6624	541.136	327.87
41140	5.6625	584.37	302.371
41141	5.6626	582.079	219.241
41142	5.6627	552.488	5.2481
41143	5.6627	598.058	283.748
41144	5.6628	687.335	6.4855
41145	5.6629	737.727	48.4267
41146	5.663	598.629	22.9194
41147	5.6631	620.046	182.846
41148	5.6633	616.699	44.5549
41149	5.6634	585.08	253.19
41150	5.6635	640.854	61.8235
41151	5.6636	627.821	343.403
41152	5.6637	659.003	304.019
41153	5.6637	810.452	307.848
41154	5.6637	567.95	64.0442
41155	5.6637	478.309	147.664
41156	5.6638	535.413	179.745
41157	5.6638	691.611	46.8964
41158	5.6641	581.173	179.374
41159	5.6643	647.321	228.205
41160	5.6648	536.79	130.625
41161	5.665	593.088	144.514
41162	5.6651	694.577	129.894
41163	5.6651	661.382	147.624
41164	5.6655	607.289	94.6805
41165	5.6656	639.725	140.057
41166	5.6656	632.508	256.379
41167	5.6657	564.847	52.7639
41168	5.6657	670.543	197.778
41169	5.6659	500.6	252.367
41170	5.6661	520.314	210.205
41171	5.6665	710.904	101.338
41172	5.6667	577.147	148.873
41173	5.667	616.692	196.63

41174	5.6671	423.476	219.968
41175	5.6672	569.139	126.733
41176	5.6672	621.031	23.3925
41177	5.6675	598.256	160.487
41178	5.6677	583.657	304.674
41179	5.668	640.982	106.439
41180	5.6681	512.128	171.408
41181	5.6681	631.356	111.224
41182	5.6682	634.687	110.21
41183	5.6683	639.877	24.9407
41184	5.6684	703.065	20.8041
41185	5.6686	579.414	146.181
41186	5.6686	573.799	121.441
41187	5.669	595.049	299.053
41188	5.6691	617.721	143.132
41189	5.6693	572.989	194.282
41190	5.6694	607.175	140.425
41191	5.6695	567.046	265.776
41192	5.6697	585.175	258.666
41193	5.6697	652.516	92.2697
41194	5.6697	490.822	155.341
41195	5.6697	728.494	42.6149
41196	5.6698	550.94	110.442
41197	5.6698	670.734	73.1966
41198	5.6702	635.098	129.76
41199	5.6702	765.821	51.6926
41200	5.6705	638.454	83.7052
41201	5.6706	671.486	206.775
41202	5.6706	604.226	71.7183
41203	5.6707	671.741	73.9596
41204	5.6707	628.93	308.808
41205	5.6708	644.568	31.4163
41206	5.6708	505.526	102.301
41207	5.6709	619.388	127.897
41208	5.6714	587.766	178.55
41209	5.6716	659.444	48.9927
41210	5.6716	518.874	147.617
41211	5.6717	752.889	229.457
41212	5.6719	655.285	43.6834
41213	5.6719	579.85	222.009
41214	5.672	714.273	88.4747
41215	5.6722	645.167	33.7658
41216	5.6726	719.245	15.1372
41217	5.6727	619.083	95.1754
41218	5.6731	558.132	193.388
41219	5.6733	602.855	173.318
41220	5.6733	608.896	133.549
41221	5.6735	641.246	228.893

41222	5.6736	740.126	257.783
41223	5.6737	492.777	177.421
41224	5.674	634.464	102.159
41225	5.6741	692.107	92.9655
41226	5.6742	691.823	81.229
41227	5.6743	742.29	73.5485
41228	5.6743	701.191	104.894
41229	5.6745	711.701	143.788
41230	5.6747	677.188	228.481
41231	5.6747	565.305	174.441
41232	5.6747	623.728	76.1969
41233	5.6753	621.394	280.317
41234	5.6757	435.376	61.1647
41235	5.6758	579.708	107.483
41236	5.6759	570.235	277.522
41237	5.6759	548.76	221.219
41238	5.676	624.847	249.014
41239	5.6761	663.324	127.232
41240	5.6764	651.811	229.922
41241	5.6766	698.38	27.7008
41242	5.6767	725.531	23.0006
41243	5.6769	560.277	191.81
41244	5.6771	687.362	125.318
41245	5.6772	86.2048	31.5565
41246	5.6772	747.362	31.2202
41247	5.6773	661.248	85.8278
41248	5.6773	572.45	304.61
41249	5.6775	649.047	186.087
41250	5.6775	584.092	171.789
41251	5.6776	606.3	118.558
41252	5.6779	615.49	119.61
41253	5.678	529.25	187.443
41254	5.678	638.584	110.256
41255	5.6781	515.625	189.095
41256	5.6781	634.218	159.936
41257	5.6783	669.645	85.686
41258	5.6783	608.057	172.854
41259	5.6784	609.195	18.5506
41260	5.6785	614.204	114.897
41261	5.6785	771.026	26.8451
41262	5.6786	719.49	30.0089
41263	5.6787	628.965	277.607
41264	5.6788	607.439	113.788
41265	5.6789	682.838	22.6278
41266	5.679	642.869	194.765
41267	5.6791	606.976	236.913
41268	5.6791	645.484	270.808
41269	5.6791	698.7	189.761

41270	5.6792	675.959	83.8987
41271	5.6793	556.126	220.329
41272	5.6794	597.244	279.949
41273	5.6797	590.997	179.035
41274	5.6797	648.178	266.357
41275	5.6797	694.904	27.2932
41276	5.6798	650.634	37.6823
41277	5.6799	658.291	22.4271
41278	5.68	605.631	135.659
41279	5.68	601.394	331.915
41280	5.6801	549.21	129.11
41281	5.6804	652.952	122.47
41282	5.6804	691.159	135.372
41283	5.6807	642.003	325.838
41284	5.681	578.856	249.303
41285	5.6814	570.553	216.474
41286	5.6815	667.834	44.1456
41287	5.6817	657.042	77.218
41288	5.6817	659.799	55.4902
41289	5.6818	613.23	141.562
41290	5.6821	681.585	143.988
41291	5.6821	630.494	114.221
41292	5.6823	535.468	175.859
41293	5.6824	482.881	356.169
41294	5.6828	604.473	227.576
41295	5.6829	636.881	103.704
41296	5.6831	589.897	50.895
41297	5.6831	647.741	110.752
41298	5.6832	552.851	183.74
41299	5.6835	736.385	103.626
41300	5.6835	555.771	64.5107
41301	5.6836	521.205	286.842
41302	5.6836	557.281	126.597
41303	5.6839	677.894	106.754
41304	5.6843	627.67	273.477
41305	5.6844	581.142	222.648
41306	5.6845	571.123	48.6119
41307	5.6846	677.308	27.6946
41308	5.6846	651.08	81.184
41309	5.6847	565.997	175.784
41310	5.6848	686.577	68.036
41311	5.6848	633.149	62.5834
41312	5.6849	697.929	92.034
41313	5.685	591.812	267.605
41314	5.685	668.079	204.383
41315	5.6851	655.541	123.908
41316	5.6851	697.94	50.0453
41317	5.6851	717.22	121.569

41318	5.6853	715.16	83.7113
41319	5.6854	693.928	40.0485
41320	5.6855	757.323	11.5555
41321	5.6856	588.899	160.054
41322	5.6859	679.851	345.74
41323	5.686	567.154	44.1123
41324	5.6861	595.162	300.59
41325	5.6862	569.596	264.878
41326	5.6865	840.916	306.118
41327	5.6866	530.271	140.336
41328	5.687	785.367	70.6665
41329	5.6875	630.374	117.006
41330	5.6875	643.817	68.0546
41331	5.6875	713.83	75.2427
41332	5.6875	664.825	160.62
41333	5.6876	585.011	101.984
41334	5.688	607.972	222.307
41335	5.688	604.96	186.757
41336	5.688	631.873	176.865
41337	5.688	620.623	177.526
41338	5.6884	550.2	183.791
41339	5.6884	602.909	44.7055
41340	5.6889	622.109	74.0857
41341	5.6889	504.23	174.586
41342	5.689	520.485	215.567
41343	5.689	502.745	190.07
41344	5.6891	681.952	69.638
41345	5.6894	608.158	143.384
41346	5.6894	749.195	101.806
41347	5.6894	788.453	204.611
41348	5.6896	590.645	150.493
41349	5.6899	616.674	329.224
41350	5.6901	650.746	108.067
41351	5.6903	605.445	196.793
41352	5.6903	606.46	109.116
41353	5.6904	653.219	46.9268
41354	5.6905	595.522	175.507
41355	5.6905	476.902	163.869
41356	5.6906	609.787	186.973
41357	5.6907	539.176	327.951
41358	5.6908	518.665	131.419
41359	5.6908	653.934	68.1917
41360	5.6909	613.72	228.551
41361	5.6916	751.046	269.238
41362	5.6921	684.984	70.3895
41363	5.6922	605.891	349.03
41364	5.6924	635.198	145.769
41365	5.6924	597.491	234.214

41366	5.6924	-2.9494	316.43
41367	5.6925	538.316	357.538
41368	5.6928	660.109	60.5057
41369	5.6934	563.269	161.612
41370	5.6935	569.06	248.368
41371	5.6936	591.109	52.0975
41372	5.6937	667.013	306.046
41373	5.6939	675.978	305.151
41374	5.694	582.944	149.802
41375	5.6941	649.355	208.89
41376	5.6943	650.571	103.276
41377	5.6943	754.446	58.1047
41378	5.6945	609.833	190.478
41379	5.6947	711.61	26.3717
41380	5.6948	575.369	185.784
41381	5.695	725.65	23.697
41382	5.6952	603.397	150.801
41383	5.6954	698.472	11.3571
41384	5.6956	659.42	43.3189
41385	5.6957	827.282	24.9452
41386	5.6957	677.071	24.2853
41387	5.6958	696.232	153.659
41388	5.6959	520.112	78.4059
41389	5.6963	610.76	284.469
41390	5.6963	753.935	202.299
41391	5.6965	703.514	72.7919
41392	5.6965	715.195	44.1215
41393	5.6966	671.202	144.732
41394	5.6968	584.455	59.1736
41395	5.6968	628.674	184.92
41396	5.6971	674.151	81.8512
41397	5.6976	611.311	100.069
41398	5.6978	547.688	40.6548
41399	5.6979	755.558	81.1981
41400	5.6979	593.457	237.111
41401	5.6981	615.982	74.0409
41402	5.6982	518.494	75.2334
41403	5.6982	691.028	101.924
41404	5.6983	662.247	95.9938
41405	5.6987	627.141	171.853
41406	5.6987	476.48	147.843
41407	5.6991	625.189	130.702
41408	5.6991	644.601	136.29
41409	5.6994	818.374	23.6708
41410	5.6995	679.129	356.746
41411	5.6996	693.907	303.511
41412	5.6998	496.326	198.246
41413	5.6999	658.608	267.587

41414	5.6999	533.652	262.43
41415	5.7	575.006	112.897
41416	5.7002	552.509	171.175
41417	5.7004	679.099	93.5125
41418	5.7005	611.782	120.722
41419	5.7005	616.269	52.9734
41420	5.7006	675.755	46.4339
41421	5.7006	727.416	202.318
41422	5.7007	615.643	52.8935
41423	5.701	537.515	256.264
41424	5.701	540.357	107.455
41425	5.7015	732.618	32.8803
41426	5.7015	603.299	155.647
41427	5.7016	668.633	133.313
41428	5.7017	637.642	5.6885
41429	5.7017	625.473	149.26
41430	5.7019	570.459	193.988
41431	5.7021	754.861	24.7109
41432	5.7022	623.892	186.928
41433	5.7023	645.647	85.7462
41434	5.7024	611.056	295.149
41435	5.7027	659.939	86.1521
41436	5.7028	545.51	173.714
41437	5.7029	751.16	327.527
41438	5.7029	793.464	11.9507
41439	5.7031	624.322	117.455
41440	5.7033	672.467	129.067
41441	5.7033	676.385	160.847
41442	5.7035	624.893	149.746
41443	5.7037	535.048	253.972
41444	5.7038	512.832	219.567
41445	5.7041	663.338	100.201
41446	5.7041	264.374	208.482
41447	5.7042	730.414	22.0581
41448	5.7045	571.124	191.721
41449	5.7045	620.395	325.059
41450	5.7045	527.618	73.1417
41451	5.7045	651.463	55.1916
41452	5.7046	672.02	73.8491
41453	5.7048	559.081	239.113
41454	5.7049	608.837	15.3569
41455	5.705	611.317	225.844
41456	5.705	696.152	35.851
41457	5.7051	741.83	358.09
41458	5.7052	730.701	327.32
41459	5.7052	719.613	32.224
41460	5.7057	696.12	26.2421
41461	5.7059	481.372	175.456

41462	5.7059	527.851	97.2759
41463	5.706	679.373	65.5406
41464	5.7061	579.108	234.347
41465	5.7062	617.694	314.228
41466	5.7065	555.875	183.537
41467	5.7067	612.143	198.643
41468	5.7067	691.589	295.035
41469	5.7069	575.888	165.13
41470	5.707	680.797	322.062
41471	5.7072	558.581	183.785
41472	5.7076	682.982	296.455
41473	5.7078	541.41	189.163
41474	5.7079	696.201	208.465
41475	5.708	648.62	94.8394
41476	5.7081	543.073	182.67
41477	5.7082	674.223	108.155
41478	5.7084	601.899	174.349
41479	5.7084	534.152	168.583
41480	5.7084	595.871	64.1153
41481	5.7085	684.027	67.0751
41482	5.7085	636.104	6.5506
41483	5.7087	722.985	19.5706
41484	5.7087	660.819	174.374
41485	5.7091	579.033	135.225
41486	5.7091	616.801	181.791
41487	5.7091	537.26	44.7639
41488	5.7092	665.348	130.642
41489	5.7092	473.345	352.337
41490	5.7094	570.635	155.604
41491	5.7098	621.033	320.8
41492	5.7099	507.677	221.516
41493	5.7099	752.191	110.459
41494	5.7101	412.89	348.574
41495	5.7103	591.789	37.5643
41496	5.7104	583.634	288.352
41497	5.7104	748.93	102.285
41498	5.7106	564.089	54.5412
41499	5.711	693.113	330.106
41500	5.711	614.628	108.854
41501	5.7111	650.79	198.585
41502	5.7112	644.702	81.8017
41503	5.7116	565.387	142.63
41504	5.7123	607.926	157.93
41505	5.7123	562.315	180.967
41506	5.7126	701.268	131.775
41507	5.713	488.578	153.258
41508	5.7134	599.262	65.4288
41509	5.7135	671.149	287.023

41510	5.7137	566.856	324.836
41511	5.7139	640.584	145.808
41512	5.7141	622.305	31.7886
41513	5.7144	613.72	178.189
41514	5.7151	614.583	171.653
41515	5.7152	632.194	144.927
41516	5.7152	660.477	15.5988
41517	5.7153	538.371	90.679
41518	5.7154	537.949	182.097
41519	5.7157	632.153	265.668
41520	5.7161	729.359	122.022
41521	5.7162	580.354	37.2073
41522	5.7163	552.873	170.899
41523	5.7163	522.556	193.034
41524	5.7164	615.835	169.347
41525	5.7168	637.551	162.78
41526	5.7169	616.498	45.6703
41527	5.7169	517.953	87.1895
41528	5.7169	752.253	56.8935
41529	5.7171	708.687	130.992
41530	5.7171	754.228	23.8844
41531	5.7171	657.456	183.29
41532	5.7172	717.775	72.9205
41533	5.7172	735.6	56.1064
41534	5.7173	614.042	175.45
41535	5.7173	540.937	252.935
41536	5.7173	665.069	52.8539
41537	5.7174	693.997	291.1
41538	5.7175	655.986	182.34
41539	5.7175	663.663	78.0256
41540	5.7176	623.01	36.9789
41541	5.7177	570.271	118.783
41542	5.718	710.781	117.181
41543	5.7181	645.918	87.7456
41544	5.7183	653.746	82.289
41545	5.7187	661.129	161.701
41546	5.7187	517.884	183.29
41547	5.7187	574.82	106.606
41548	5.7187	767.272	50.7871
41549	5.7188	739.901	16.478
41550	5.719	646.85	128.828
41551	5.7192	714.547	36.2904
41552	5.7192	640.589	123.213
41553	5.7194	538.675	172.811
41554	5.7196	-2.8036	78.8279
41555	5.7199	615.9	172.576
41556	5.7199	750.097	60.0633
41557	5.7202	557.151	283.184

41558	5.7203	650.015	297.969
41559	5.7204	579.177	234.565
41560	5.7207	543.457	246.101
41561	5.7208	600.196	182.307
41562	5.7208	530.222	103.813
41563	5.721	652.301	191.031
41564	5.7211	688.933	74.8081
41565	5.7211	665.069	204.949
41566	5.7213	681.177	330.863
41567	5.7213	-4.0515	265.531
41568	5.7214	762.639	54.2053
41569	5.7215	712.659	27.6522
41570	5.7219	598.487	60.9445
41571	5.7219	562.105	71.4828
41572	5.7221	699.403	225.707
41573	5.7222	627.097	26.7649
41574	5.7226	629.527	280.345
41575	5.7226	716.721	57.5017
41576	5.7232	636.602	122.722
41577	5.7232	751.664	211.684
41578	5.7234	572.662	305.12
41579	5.7237	632.061	188.317
41580	5.7237	607.782	124.691
41581	5.7238	521.423	246.393
41582	5.7238	600.607	187.553
41583	5.7241	687.673	334.307
41584	5.7244	607.916	64.8307
41585	5.7244	641.134	128.134
41586	5.7245	633.994	186.633
41587	5.7248	646.637	97.2223
41588	5.7249	638.043	105.281
41589	5.7249	575.252	231.686
41590	5.7249	696.092	103.954
41591	5.7252	686.542	119.067
41592	5.7252	617.012	20.2831
41593	5.7252	668.732	28.6346
41594	5.7258	627.08	225.923
41595	5.726	672.962	103.965
41596	5.7261	604.35	138.635
41597	5.7266	548.298	155.635
41598	5.7268	795.541	103.732
41599	5.7268	591.861	178.047
41600	5.727	670.255	91.37
41601	5.7272	759.915	40.9882
41602	5.7273	567.199	198.108
41603	5.7275	693.115	76.8203
41604	5.7278	694.005	246.738
41605	5.7278	511.956	130.421

41606	5.7281	652.58	75.769
41607	5.7281	623.696	125.608
41608	5.7281	667.534	75.384
41609	5.7281	742.364	14.1899
41610	5.7284	667.163	25.4074
41611	5.7284	579.563	166.468
41612	5.7284	634.481	181.078
41613	5.7285	546.763	193.821
41614	5.729	638.334	127.487
41615	5.7295	717.214	21.8811
41616	5.7297	572.007	155.223
41617	5.7299	627.358	68.4678
41618	5.7301	719.997	247.501
41619	5.7301	725.146	23.8702
41620	5.7302	750.13	40.1643
41621	5.7303	578.604	172.964
41622	5.7303	609.015	160.312
41623	5.7304	627.93	49.8707
41624	5.7305	662.812	66.4613
41625	5.731	406.99	95.0309
41626	5.7315	536.413	223.565
41627	5.7315	656.27	66.8852
41628	5.7316	624.507	192.623
41629	5.7317	598.635	125.266
41630	5.7317	588.199	123.334
41631	5.7319	599.445	174.994
41632	5.732	619.651	245.49
41633	5.7322	586.924	180.286
41634	5.7322	602.396	333.317
41635	5.7323	644.091	81.2984
41636	5.7323	569.375	129.551
41637	5.7326	524.727	324.49
41638	5.7327	634.868	272.32
41639	5.7327	554.604	134.611
41640	5.7328	657.161	84.5277
41641	5.733	587.623	236.438
41642	5.7331	614.268	355.878
41643	5.7332	614.485	95.0434
41644	5.7334	618.374	9.9624
41645	5.7338	646.535	147.42
41646	5.734	598.981	175.02
41647	5.7345	615.798	176.46
41648	5.7347	540.88	167.092
41649	5.7348	734.894	202.354
41650	5.7351	619.865	146.459
41651	5.7351	582.423	241.229
41652	5.7351	629.93	53.2675
41653	5.7354	582.893	267.494

41654	5.7354	583.603	178.299
41655	5.7355	625.892	275.362
41656	5.7355	728.246	229.301
41657	5.7356	591.252	281.83
41658	5.7357	752.389	52.2979
41659	5.7358	612.252	145.613
41660	5.7359	598.088	235.128
41661	5.7361	693.49	88.8785
41662	5.7362	674.895	118.393
41663	5.7362	748.636	26.011
41664	5.7363	637.757	266.314
41665	5.7366	583.963	142.095
41666	5.7367	687.832	35.3715
41667	5.7368	510.929	154.196
41668	5.7368	762.553	72.4109
41669	5.7372	697.054	97.3719
41670	5.7373	616.704	149.118
41671	5.7375	605.236	109.487
41672	5.7376	674.256	68.1727
41673	5.7377	588.975	58.4983
41674	5.7378	681.346	51.4308
41675	5.7379	603.796	13.6556
41676	5.7379	729.317	211.249
41677	5.738	627.316	246.862
41678	5.7382	746.24	48.2476
41679	5.7385	730.37	82.0949
41680	5.7386	648.987	87.5374
41681	5.7389	661.416	58.9044
41682	5.7389	634.07	114.185
41683	5.7391	662.58	207.341
41684	5.7392	495.924	137.67
41685	5.7393	727.499	59.5268
41686	5.7393	616.274	222.465
41687	5.7393	761.584	54.7886
41688	5.7393	736.83	201.485
41689	5.7396	593.279	167.494
41690	5.7396	775.592	28.3909
41691	5.7397	538.577	78.3084
41692	5.7397	560.451	149.409
41693	5.7402	656.95	133.437
41694	5.7402	633.426	176.568
41695	5.7407	648.61	240.876
41696	5.7408	728.703	8.7095
41697	5.7409	543.268	173.911
41698	5.741	655.443	233.821
41699	5.7411	677.892	51.4048
41700	5.7411	693.004	34.7662
41701	5.7412	729.398	102.939

41702	5.7412	714.953	212.415
41703	5.7415	642.465	138.441
41704	5.7416	682.49	359.171
41705	5.7417	544.473	126.87
41706	5.7418	838.888	21.6105
41707	5.7419	765.019	50.7358
41708	5.7419	742.328	14.9525
41709	5.7421	484.136	78.1617
41710	5.7425	748	55.3779
41711	5.7426	535.339	185.266
41712	5.7427	706.554	90.6361
41713	5.7428	724.069	70.0588
41714	5.7429	678.474	195.229
41715	5.743	763.412	20.7252
41716	5.7431	646.552	340.291
41717	5.7433	729.333	338.01
41718	5.7437	553.25	77.3753
41719	5.7438	568.943	113.767
41720	5.7439	508.055	93.5276
41721	5.744	711.788	63.5099
41722	5.7441	438.503	160.312
41723	5.7442	567.669	127.905
41724	5.7444	737.176	28.1517
41725	5.7444	699.31	334.032
41726	5.7445	727.942	32.7708
41727	5.7445	618.788	182.047
41728	5.745	472.148	339.081
41729	5.7452	675.105	72.9314
41730	5.7452	602.857	9.5313
41731	5.7452	551.389	127.609
41732	5.7452	594.972	178.673
41733	5.7454	683.319	160.39
41734	5.7456	717.531	347.672
41735	5.7456	661.107	4.4714
41736	5.7457	593.016	292.773
41737	5.7459	537.682	142.939
41738	5.7459	800.628	9.5896
41739	5.746	565.486	219.677
41740	5.746	-2.849	85.2677
41741	5.7461	555.533	234.919
41742	5.7464	595.791	197.459
41743	5.7465	667.574	29.3685
41744	5.7467	524.886	13.6691
41745	5.7472	623.389	176.973
41746	5.7472	601.402	73.8201
41747	5.7474	691.311	84.5301
41748	5.7476	695.26	103.074
41749	5.748	698.441	249.638

41750	5.748	586.058	70.6561
41751	5.7481	463.133	128.298
41752	5.7481	709.341	275.972
41753	5.7482	700.572	77.2574
41754	5.7483	512.452	176.718
41755	5.7484	725.894	208.769
41756	5.7484	588.763	220.889
41757	5.7488	731.834	22.6314
41758	5.7488	483.163	354.384
41759	5.7489	673.389	242.262
41760	5.7491	644.073	294.089
41761	5.7491	624.683	112.665
41762	5.7492	508.664	262.172
41763	5.7492	699.105	232.519
41764	5.7493	699.978	203.538
41765	5.7497	663.204	9.2038
41766	5.7498	732.397	27.644
41767	5.7498	677.934	77.0671
41768	5.75	612.638	90.4429
41769	5.7501	665.381	179.075
41770	5.7501	531.318	124.102
41771	5.7505	604.014	181.931
41772	5.7508	644.128	12.9128
41773	5.7509	760.122	100.963
41774	5.7511	596.1	190.095
41775	5.7512	595.766	186.193
41776	5.7512	572.487	87.172
41777	5.7512	633.722	175.148
41778	5.7512	556.719	296.74
41779	5.7513	623.939	185.494
41780	5.7514	617.743	159.505
41781	5.7514	624.492	100.077
41782	5.7516	619.557	242.646
41783	5.7517	760.507	77.8066
41784	5.7517	567.546	153.856
41785	5.7518	742.253	32.9493
41786	5.7518	607.568	75.0432
41787	5.7525	650.354	169.32
41788	5.7526	683.09	207.117
41789	5.7526	768.286	227.478
41790	5.7529	496.424	127.696
41791	5.753	750.635	261.515
41792	5.753	643.608	194.34
41793	5.7531	536.205	168.478
41794	5.7533	667.545	129.976
41795	5.7536	779.011	55.203
41796	5.754	579.107	192.522
41797	5.7541	572.997	120.134

41798	5.7542	700.297	105.586
41799	5.7543	592.309	180.952
41800	5.7544	586.027	240.734
41801	5.7547	639.771	96.1052
41802	5.7547	723.011	71.106
41803	5.7548	588.555	186.713
41804	5.7551	709.33	132.161
41805	5.7552	801.381	80.0408
41806	5.7553	594.852	220.229
41807	5.7554	810.78	17.2475
41808	5.7557	546.309	227.238
41809	5.7558	568.971	11.7391
41810	5.756	693.758	75.2223
41811	5.7562	556.024	70.0746
41812	5.7563	723.023	311.191
41813	5.7565	652.082	75.4635
41814	5.7566	606.595	190.725
41815	5.7566	616.374	59.8393
41816	5.7567	590.097	179.451
41817	5.7568	737.404	237.081
41818	5.757	702.524	56.3246
41819	5.7571	627.699	114.909
41820	5.7572	666.493	175.244
41821	5.7575	628.302	231.45
41822	5.7577	554.569	219.183
41823	5.7578	714.032	100.965
41824	5.7579	746.781	50.7591
41825	5.7585	473.405	177.26
41826	5.7585	550.573	259.338
41827	5.7585	730.223	103.776
41828	5.759	556.147	193.689
41829	5.759	569.163	236.961
41830	5.7593	621.646	176.081
41831	5.7593	533.874	180.498
41832	5.7593	726.837	333.286
41833	5.7595	634.061	89.0887
41834	5.7598	777.25	209.77
41835	5.7599	719.641	47.2872
41836	5.76	645.967	93.6343
41837	5.7603	609.601	38.3034
41838	5.7604	692.11	189.163
41839	5.7608	660.228	18.7935
41840	5.7611	758.446	14.0747
41841	5.7613	695.785	115.866
41842	5.7614	672.68	52.9041
41843	5.7622	714.062	351.105
41844	5.7624	679.522	67.5884
41845	5.7625	551.916	151.034

41846	5.7625	631.698	135.316
41847	5.7625	667.912	291.966
41848	5.7627	755.421	94.3878
41849	5.763	656.382	157.323
41850	5.763	613.622	299.764
41851	5.7632	562.221	100.01
41852	5.7635	659.25	107.617
41853	5.7637	614.736	183.235
41854	5.7642	706.83	65.9198
41855	5.7642	787.201	79.7007
41856	5.7645	700.705	132.802
41857	5.7645	762.385	56.1539
41858	5.7646	666.612	72.8929
41859	5.7646	667.766	43.8212
41860	5.7648	609.455	220.858
41861	5.7648	663.384	81.8514
41862	5.7653	666.842	78.5925
41863	5.7654	663.365	107.373
41864	5.7655	569.521	168.401
41865	5.7656	643.761	273.163
41866	5.7657	544.634	127.74
41867	5.7657	601.856	124.297
41868	5.7657	591.613	52.503
41869	5.7658	526.002	31.721
41870	5.766	637.825	18.8383
41871	5.7663	686.268	19.7398
41872	5.7665	615.493	100.522
41873	5.7665	552.414	7.9008
41874	5.7666	662.865	26.7276
41875	5.7666	661.703	104.171
41876	5.7667	706.682	103.201
41877	5.7667	589.721	250.366
41878	5.7668	728.807	323.755
41879	5.7668	652.346	100.283
41880	5.767	-2.8428	259.075
41881	5.767	745.123	57.6276
41882	5.7671	653.149	79.206
41883	5.7672	688.731	5.6678
41884	5.7674	631.506	29.7493
41885	5.7674	703.626	202.194
41886	5.7675	679.093	94.9747
41887	5.7676	551.953	179.929
41888	5.7682	651.489	154.819
41889	5.7682	697.323	230.73
41890	5.7686	656.932	95.2235
41891	5.7686	667.883	40.5731
41892	5.7688	671.757	325.782
41893	5.7688	698.125	28.0009

41894	5.7691	765.536	69.3407
41895	5.7692	837.05	101.375
41896	5.7692	605.118	173.636
41897	5.7692	595.866	165.87
41898	5.7693	620.064	176.774
41899	5.7695	684.156	58.1811
41900	5.7696	571.498	181.822
41901	5.7699	656.447	30.4709
41902	5.7702	544.753	172.2
41903	5.7702	619.707	17.6952
41904	5.7702	644.909	116.233
41905	5.7703	715.047	63.8367
41906	5.7707	784.904	81.1025
41907	5.7707	674.433	107.851
41908	5.7708	557.826	183.797
41909	5.7708	600.452	143.86
41910	5.7715	607.344	73.578
41911	5.7719	759.419	82.8375
41912	5.772	690.85	94.9013
41913	5.7721	629.821	96.3118
41914	5.7724	580.825	140.01
41915	5.7726	662.308	225.479
41916	5.7726	656.151	86.1578
41917	5.7727	671.433	48.33
41918	5.7728	638.925	181.287
41919	5.7728	641.038	190.762
41920	5.7729	500.198	256.313
41921	5.7733	627.199	138.484
41922	5.7733	742.689	72.1744
41923	5.7735	568.887	75.323
41924	5.7735	667.747	43.3581
41925	5.7736	570.976	189.663
41926	5.7736	580.018	12.4052
41927	5.7737	587.522	68.0434
41928	5.7741	529.265	188.81
41929	5.7742	623.257	81.6628
41930	5.7743	609.425	100.269
41931	5.7743	608.256	249.409
41932	5.7744	705.073	125.832
41933	5.7746	654.965	26.3105
41934	5.7746	515.02	159.766
41935	5.7747	685.671	99.5173
41936	5.7747	561.349	202.565
41937	5.7748	628.296	30.5405
41938	5.7751	579.642	92.7512
41939	5.7752	644.503	191.652
41940	5.7753	706.719	3.9185
41941	5.7755	652.585	87.03

41942	5.7756	744.197	347.364
41943	5.7757	715.922	77.2224
41944	5.7758	662.624	61.6587
41945	5.7758	682.416	56.7458
41946	5.7759	727.142	78.7669
41947	5.7759	683.031	72.9526
41948	5.7759	665.5	172.209
41949	5.7762	739.533	39.3838
41950	5.7764	708.991	278.094
41951	5.7765	604.619	72.3802
41952	5.7766	807.434	48.0585
41953	5.7768	761.308	32.9121
41954	5.7769	264.116	204.021
41955	5.7769	649.985	185.434
41956	5.777	574.954	293.69
41957	5.777	517.813	183.607
41958	5.7771	618.173	302.61
41959	5.7772	630.814	253.863
41960	5.7776	669.295	58.0512
41961	5.7776	558.382	173.108
41962	5.7777	668.441	168.98
41963	5.7777	752.897	116.28
41964	5.7778	544.257	120.818
41965	5.7779	629.313	169.608
41966	5.778	636.463	305.148
41967	5.7781	685.997	80.2436
41968	5.7782	710.415	115.861
41969	5.7782	565.431	272.652
41970	5.7784	707.749	300.137
41971	5.7785	752.887	58.5056
41972	5.7787	804.872	45.8904
41973	5.7787	753.441	268.736
41974	5.7787	680.258	128.297
41975	5.7788	723.557	99.0511
41976	5.7789	621.934	194.368
41977	5.779	723.357	19.479
41978	5.7791	625.963	295.352
41979	5.7792	593.815	106.24
41980	5.7793	743.485	9.2502
41981	5.7794	663.542	320.894
41982	5.7794	641.678	194.411
41983	5.7795	671.261	63.6489
41984	5.7797	700.271	112.754
41985	5.7797	685.297	77.4509
41986	5.7799	653.842	94.1535
41987	5.7799	539.309	197.29
41988	5.78	728.891	97.9685
41989	5.78	612.544	188.797

41990	5.7802	560.819	253.417
41991	5.7802	751.653	41.2684
41992	5.7803	636.905	149.886
41993	5.7804	644.407	25.9527
41994	5.7811	711.082	80.0309
41995	5.7811	530.209	189.002
41996	5.7821	657.513	174.078
41997	5.7821	630.955	117.991
41998	5.7825	627.919	154.714
41999	5.7829	701.921	75.7553
42000	5.783	763.439	57.5347
42001	5.7831	683.22	63.8035
42002	5.7832	610.557	107.634
42003	5.7833	531.049	113.832
42004	5.7834	689.457	177.269
42005	5.7835	621.482	162.321
42006	5.7838	638.898	21.9456
42007	5.7842	703.261	205.212
42008	5.7843	651.442	333.225
42009	5.7845	681.013	148.374
42010	5.7847	637.264	251.826
42011	5.7848	601.204	183.409
42012	5.7848	712.982	102.876
42013	5.785	614.202	128.42
42014	5.7853	641.032	60.4212
42015	5.7855	683.677	134.599
42016	5.7856	586.862	193.194
42017	5.7857	663.029	134.649
42018	5.7859	548.713	224.405
42019	5.786	683.233	120.974
42020	5.7861	654.085	160.048
42021	5.7864	762.417	7.6768
42022	5.7866	805.322	94.3289
42023	5.7867	672.429	162.331
42024	5.7867	671.702	39.8833
42025	5.7868	818.877	257.582
42026	5.7868	768.391	40.9705
42027	5.7869	636.219	214.049
42028	5.787	617.525	159.286
42029	5.787	725.407	80.8014
42030	5.7871	667.936	162.992
42031	5.7871	612.497	229.802
42032	5.7871	700.196	245.474
42033	5.7871	587.936	178.656
42034	5.7874	708.378	60.9574
42035	5.7874	553.575	139.491
42036	5.7874	731.832	332.52
42037	5.7875	591.262	266.589

42038	5.7879	597.247	154.248
42039	5.788	562.078	255.991
42040	5.788	649.453	128.826
42041	5.7881	676.802	257.13
42042	5.7881	598.698	170.006
42043	5.7884	579.876	87.4575
42044	5.7885	585.058	264.566
42045	5.7888	644.267	126.904
42046	5.7889	620.352	156.807
42047	5.7889	652.029	109.054
42048	5.7893	771.043	202.575
42049	5.7895	734.855	92.1905
42050	5.7895	765.355	40.5326
42051	5.7896	582.786	8.2337
42052	5.7897	672.641	302.504
42053	5.7899	655.609	161.928
42054	5.79	704.583	35.6222
42055	5.7903	657.329	179.283
42056	5.7905	755.964	356.704
42057	5.7907	662.375	81.8171
42058	5.7909	766.287	52.3175
42059	5.791	771.773	70.0849
42060	5.7911	652.486	165.702
42061	5.7911	653.902	121.251
42062	5.7911	819.326	24.5339
42063	5.7912	686.583	55.4078
42064	5.7913	514.166	133.159
42065	5.7913	649.153	352.217
42066	5.7914	761.704	50.8421
42067	5.7918	620.268	317.73
42068	5.7919	636.474	174.72
42069	5.7925	645.435	163.52
42070	5.7926	650.824	148.42
42071	5.7929	817.941	44.5658
42072	5.793	690.207	339.934
42073	5.7933	596.88	219.257
42074	5.7933	666.01	125.129
42075	5.7933	736.843	31.5903
42076	5.7934	633.167	123.552
42077	5.7934	627.04	228.664
42078	5.7935	628.925	219.696
42079	5.7936	653.669	105.615
42080	5.7936	729.143	56.5981
42081	5.7937	622.752	65.3567
42082	5.7938	714.116	24.5609
42083	5.7939	610.48	137.067
42084	5.7939	618.017	176.351
42085	5.794	698.66	17.3802

42086	5.7945	735.993	282.495
42087	5.7947	543.336	145.771
42088	5.7949	606.71	19.4627
42089	5.795	728.45	146.365
42090	5.795	554.478	178.726
42091	5.7951	778.926	71.8295
42092	5.7952	659.517	68.8448
42093	5.7957	659.343	80.5891
42094	5.7957	762.026	72.6806
42095	5.7958	551.864	218.768
42096	5.7958	599.753	104.556
42097	5.796	710.255	136.775
42098	5.7961	532.318	222.313
42099	5.7963	746.993	20.1854
42100	5.7964	723.329	287.752
42101	5.797	649.69	330.711
42102	5.797	609.78	301.724
42103	5.7971	593.658	177.66
42104	5.7974	796.732	115.981
42105	5.7978	696.198	72.9727
42106	5.7978	680.316	35.1694
42107	5.7981	706.011	199.488
42108	5.7984	694.831	45.9714
42109	5.7985	604.329	251.505
42110	5.7986	761.814	64.8786
42111	5.7988	669.384	265.241
42112	5.7992	647.623	123.029
42113	5.7993	642.974	190.575
42114	5.7993	645.434	181.947
42115	5.7997	650.669	152.473
42116	5.7998	603.866	111.344
42117	5.7998	696.664	50.6577
42118	5.8	630.058	61.3132
42119	5.8003	634.12	148.544
42120	5.8004	741.57	324.115
42121	5.8004	719.686	101.053
42122	5.8008	698.785	171.01
42123	5.8008	690.593	121.87
42124	5.8009	541.201	15.2724
42125	5.801	701.163	277.311
42126	5.801	632.816	23.0328
42127	5.801	412.365	171.044
42128	5.8011	726.218	126.563
42129	5.8014	663.266	127.7
42130	5.8017	721.388	215.899
42131	5.8019	646.853	286.04
42132	5.8021	531.658	223.34
42133	5.8023	741.064	76.8032

42134	5.8026	602.112	222.573
42135	5.8028	490.969	175.714
42136	5.8029	681.739	271.359
42137	5.8029	639.861	32.4879
42138	5.8031	639.855	3.7981
42139	5.8034	584.203	66.9338
42140	5.8035	664.817	266.305
42141	5.8036	491.537	62.9414
42142	5.8036	562.899	147.153
42143	5.8037	647.433	173.692
42144	5.8037	668.284	266.75
42145	5.804	635.986	105.46
42146	5.8041	739.787	126.844
42147	5.8044	602.108	71.8892
42148	5.8045	648.002	111.626
42149	5.8046	628.285	192.359
42150	5.8047	592.715	145.547
42151	5.8049	744.429	261.142
42152	5.8049	748.871	154.251
42153	5.805	709.806	41.6142
42154	5.8051	594.566	307.459
42155	5.8052	517.518	127.049
42156	5.8052	706.914	288.793
42157	5.8052	614.978	96.7695
42158	5.8056	636.267	185.494
42159	5.8059	651.398	195.435
42160	5.8062	732.014	312.68
42161	5.8063	463.138	318.524
42162	5.8063	646.803	319.502
42163	5.8064	741.289	36.8876
42164	5.8067	637.302	164.14
42165	5.8068	774.81	37.4463
42166	5.8068	878.088	260.958
42167	5.8069	593.66	139.753
42168	5.807	630.825	151.537
42169	5.807	744.161	43.8968
42170	5.8074	632.367	221.259
42171	5.8074	565.233	76.7557
42172	5.8075	649.941	131.442
42173	5.8075	760.662	300.102
42174	5.8077	611.43	196.463
42175	5.8079	649.459	158.159
42176	5.8081	708.396	99.6335
42177	5.8081	580.516	119.641
42178	5.8082	656.267	73.9056
42179	5.8082	793.659	46.9784
42180	5.8087	673.19	67.2144
42181	5.8087	540.134	196.744

42182	5.8089	592.12	138.612
42183	5.8093	584.435	218.31
42184	5.8095	743.851	104.592
42185	5.8095	568.484	118.419
42186	5.8097	587.546	234.658
42187	5.8098	602.074	252.954
42188	5.8099	667.369	109.076
42189	5.8099	828.721	48.5536
42190	5.81	672.852	170.95
42191	5.81	-2.8019	266.451
42192	5.81	777.367	57.5104
42193	5.8101	662.249	82.504
42194	5.8102	684.185	120.298
42195	5.8106	646.155	179.118
42196	5.8107	611.334	140.323
42197	5.8109	597.74	43.0864
42198	5.8109	657.572	138.233
42199	5.811	611.466	162.184
42200	5.8113	699.861	208.641
42201	5.8113	672.361	9.0205
42202	5.8114	670.125	285.942
42203	5.8117	792.192	80.3298
42204	5.812	673.128	53.2734
42205	5.812	634.695	202.824
42206	5.8121	630.07	260.766
42207	5.8122	626.956	225.558
42208	5.8122	608.737	176.017
42209	5.8128	508.541	280.508
42210	5.8131	665.447	84.0031
42211	5.8132	586.14	83.4741
42212	5.8132	640.385	51.4031
42213	5.8136	690.874	65.3965
42214	5.814	623.259	159.015
42215	5.8141	801.309	76.6563
42216	5.8141	640.816	54.1405
42217	5.8142	646.297	245.27
42218	5.8142	533.984	113.976
42219	5.8144	562.214	250.641
42220	5.8144	713.759	74.6064
42221	5.8145	596.908	227.702
42222	5.8145	707.405	50.8796
42223	5.8148	708.562	122.735
42224	5.815	615.358	141.154
42225	5.8151	698.027	96.3968
42226	5.8152	745.974	66.54
42227	5.8154	585.299	230.989
42228	5.8157	733.694	358.474
42229	5.8158	763.718	144.61

42230	5.8158	702.317	57.2108
42231	5.816	778.959	73.0149
42232	5.816	672.87	231.868
42233	5.8161	627.632	184.419
42234	5.8161	649.708	69.8191
42235	5.8161	641.369	121.508
42236	5.8161	728.508	99.6213
42237	5.8162	728.593	164.686
42238	5.8162	586.858	189.905
42239	5.8163	624.979	0.627
42240	5.8166	647.495	176.747
42241	5.8167	819.465	258.461
42242	5.8168	586.892	185.007
42243	5.8169	497.262	174.542
42244	5.8171	718.873	168.06
42245	5.8171	721.85	15.2881
42246	5.8177	726.571	352.877
42247	5.8179	733.134	59.9558
42248	5.818	718.432	72.2721
42249	5.8181	688.096	194.213
42250	5.8184	493.658	201.774
42251	5.8184	565.915	104.067
42252	5.8185	653.979	173.73
42253	5.8186	794.377	74.4688
42254	5.8186	684.325	192.695
42255	5.8187	704.43	338.413
42256	5.8187	816.048	25.9039
42257	5.8188	707.335	68.0579
42258	5.8189	719.555	288.036
42259	5.8189	687.672	54.5251
42260	5.8193	844.219	83.5837
42261	5.8195	787.227	11.762
42262	5.8197	708.763	117.095
42263	5.8198	756.79	61.1746
42264	5.82	706.247	123.241
42265	5.8203	641.49	266.001
42266	5.8204	739.605	212.413
42267	5.8206	804.7	40.1501
42268	5.8209	544.781	124.494
42269	5.8214	767.391	63.6903
42270	5.8214	621.447	74.5909
42271	5.8216	695.798	328.761
42272	5.8219	739.381	84.5659
42273	5.8219	694.497	307.575
42274	5.8221	714.64	122.305
42275	5.8224	711.455	47.2428
42276	5.8225	826.428	57.4633
42277	5.8227	658.145	119.063

42278	5.8228	659.317	65.5841
42279	5.823	808.377	202.53
42280	5.8231	622.828	167.577
42281	5.8232	606.829	168.915
42282	5.8232	719.531	74.7745
42283	5.8233	554.824	86.8529
42284	5.8236	559.606	87.7594
42285	5.8238	644.054	14.644
42286	5.8241	668.148	112.345
42287	5.8242	651.981	157.461
42288	5.8242	583.995	152.025
42289	5.8243	650.862	176.115
42290	5.8246	637.203	203.622
42291	5.8247	741.214	79.4647
42292	5.8248	665.474	224.923
42293	5.8254	767.526	252.766
42294	5.8254	656.154	321.332
42295	5.8258	685.23	134.555
42296	5.8265	749.176	72.179
42297	5.8266	693.304	163.931
42298	5.8266	566.482	176.893
42299	5.8266	558.159	184.77
42300	5.8267	701.867	126.098
42301	5.8267	603.049	219.862
42302	5.827	693.826	166.244
42303	5.8273	713.17	228.313
42304	5.8273	665.04	228.208
42305	5.8275	612.887	219.642
42306	5.8276	772.691	28.7166
42307	5.8276	537.089	154.043
42308	5.8277	737.176	17.672
42309	5.8277	680.418	57.2541
42310	5.8279	610.751	198.727
42311	5.8282	664.04	112.474
42312	5.8283	741.602	81.8641
42313	5.8284	731.188	106.937
42314	5.8286	680.741	146.736
42315	5.8289	651.117	177.919
42316	5.8289	659.421	62.4422
42317	5.8294	700.578	140.574
42318	5.8296	726.373	48.6703
42319	5.8297	619.628	255.362
42320	5.8298	607.938	86.2596
42321	5.83	754.948	77.0574
42322	5.83	652.239	191.588
42323	5.83	563.066	179.819
42324	5.8306	822.452	49.1361
42325	5.8307	713.844	329.171

42326	5.8308	651.838	168.609
42327	5.8308	693.981	90.1634
42328	5.831	707.405	282.692
42329	5.8311	590.757	140.891
42330	5.8314	645.069	250.733
42331	5.8314	580.385	225.07
42332	5.8316	616.769	140.472
42333	5.8316	656.137	178.929
42334	5.8317	609.963	252.118
42335	5.8317	648.757	332.106
42336	5.8317	770.409	110.972
42337	5.8318	723.341	183.049
42338	5.8319	413.549	172.961
42339	5.8321	512.303	235.763
42340	5.8323	760.349	202.453
42341	5.8324	614.44	145.246
42342	5.8324	729.819	159.038
42343	5.8324	480.951	58.4377
42344	5.8327	663.29	28.3765
42345	5.8328	723.444	67.2654
42346	5.833	590.33	157.773
42347	5.8331	-2.836	78.0267
42348	5.8334	729.019	74.9589
42349	5.8336	655.809	11.2188
42350	5.8338	666.807	148.932
42351	5.8344	665.89	191.818
42352	5.8346	733.619	107.769
42353	5.8348	679.656	291.86
42354	5.8349	676.676	247.295
42355	5.835	672.744	249.142
42356	5.8351	813.059	142.844
42357	5.8351	688.61	126.957
42358	5.8352	714.7	121.082
42359	5.8352	757.827	36.5279
42360	5.8355	651.481	226.535
42361	5.8355	615.279	151.785
42362	5.8356	806.595	101.539
42363	5.8357	647.277	186.577
42364	5.8358	719.709	126.107
42365	5.8358	737.919	31.7309
42366	5.8358	640.53	18.6098
42367	5.8359	872.506	75.8806
42368	5.836	669.96	262.735
42369	5.836	748.081	35.0781
42370	5.8362	572.111	183.436
42371	5.8362	577.429	177.932
42372	5.8366	659.774	163.308
42373	5.8366	511.403	175.232

42374	5.8367	709.442	215.682
42375	5.8368	703.431	190.689
42376	5.8368	748.185	333.508
42377	5.8368	679.138	226.979
42378	5.8371	747.611	56.2347
42379	5.8372	747.122	132.733
42380	5.8373	751.419	106.172
42381	5.8375	737.881	36.3092
42382	5.8376	744.729	23.0605
42383	5.8377	724.197	297.087
42384	5.8379	682.556	235.217
42385	5.838	749.607	39.0804
42386	5.8381	720.573	249.859
42387	5.8381	692.967	133.312
42388	5.8383	604.654	137.937
42389	5.8386	799.255	64.1257
42390	5.8388	656.043	151.662
42391	5.8388	638.3	27.0393
42392	5.839	691.76	67.4694
42393	5.8391	690.2	216.832
42394	5.8391	652.703	149.487
42395	5.8392	607.196	105.638
42396	5.8393	654.698	261.202
42397	5.8393	755.506	112.496
42398	5.8394	672.482	230.836
42399	5.8394	603.052	120.998
42400	5.8396	594.649	150.47
42401	5.8396	673.38	67.552
42402	5.8396	827.541	57.1352
42403	5.8397	647.383	324.832
42404	5.8398	709.017	112.259
42405	5.8401	680.301	331.829
42406	5.8402	663.422	121.014
42407	5.8402	703.339	218.778
42408	5.8404	582.307	175.548
42409	5.8405	638.226	193.015
42410	5.8406	655.009	145.672
42411	5.8407	658.292	318.325
42412	5.8408	654.907	97.4752
42413	5.8409	695.498	146.036
42414	5.8409	690.218	245.565
42415	5.8411	665.193	32.7832
42416	5.8411	692.292	64.6948
42417	5.8415	720.097	98.941
42418	5.8416	716.687	67.1487
42419	5.842	578.358	321.942
42420	5.842	780.927	305.953
42421	5.8421	706.317	139.641

42422	5.8421	812.849	24.4928
42423	5.8423	654.317	130.211
42424	5.8426	770.778	303.634
42425	5.8426	727.864	17.88
42426	5.8427	629.301	155.435
42427	5.8427	652.752	114.156
42428	5.843	742.978	132.551
42429	5.843	677.635	96.3719
42430	5.8431	778.361	33.7868
42431	5.8431	666.676	225.593
42432	5.8432	842.141	261.649
42433	5.8432	790.318	31.7186
42434	5.8433	756.555	108.873
42435	5.8434	613.613	67.3134
42436	5.8436	744.21	256.313
42437	5.8438	596.741	81.2966
42438	5.8439	692.546	133.697
42439	5.8441	555.251	253.257
42440	5.8441	664.777	145.627
42441	5.8441	594.513	122.016
42442	5.8442	792.237	0.7408
42443	5.8444	618.221	196.729
42444	5.8446	648.738	116.876
42445	5.8446	634.134	238.004
42446	5.8446	580.739	165.818
42447	5.8447	687.81	87.0481
42448	5.845	692.363	127.827
42449	5.8451	677.759	57.9314
42450	5.8451	501.585	173.581
42451	5.8454	601.792	248.751
42452	5.8455	648.178	78.6195
42453	5.8458	751.325	92.2084
42454	5.8459	702.253	41.6638
42455	5.8461	632.343	83.8587
42456	5.8466	754.314	24.9667
42457	5.8468	601.822	163.991
42458	5.8476	654.955	326.519
42459	5.8477	706.112	129.109
42460	5.8481	610.858	67.8813
42461	5.8481	762.708	50.5817
42462	5.8482	746.203	56.2516
42463	5.8483	605.507	99.1882
42464	5.8483	721.436	78.1603
42465	5.8487	792.865	56.0848
42466	5.8488	740.239	99.312
42467	5.8489	709.97	58.13
42468	5.8491	720.016	151.961
42469	5.8492	691.2	287.607

42470	5.8493	705.623	277.736
42471	5.8494	660.907	178.754
42472	5.8495	697.597	134.126
42473	5.8495	722.974	53.6303
42474	5.8495	776.786	113.256
42475	5.8495	714.461	102.013
42476	5.8496	635.124	196.927
42477	5.8496	697.908	135.857
42478	5.8497	686.423	183.691
42479	5.8498	730.645	2.1669
42480	5.8499	631.074	190.664
42481	5.85	588.654	181.079
42482	5.8503	782.331	78.9859
42483	5.8503	717.131	109.631
42484	5.8503	711.02	125.655
42485	5.8505	845.268	304.894
42486	5.8506	866.685	49.4777
42487	5.8506	773.796	60.7127
42488	5.8508	797.14	47.6199
42489	5.8509	667.617	250.12
42490	5.8511	655.781	140.128
42491	5.8512	719.54	231.397
42492	5.8514	629.311	323.19
42493	5.8517	592.529	281.273
42494	5.8518	597.012	188.835
42495	5.8518	762.355	10.7918
42496	5.8519	729.583	11.4938
42497	5.8522	682.389	133.594
42498	5.8522	734.057	24.1468
42499	5.8522	737.74	99.7513
42500	5.8522	793.838	200.953
42501	5.8523	766.797	52.8817
42502	5.8524	757.531	351.9
42503	5.8528	628.568	60.2837
42504	5.8529	661.725	229.859
42505	5.853	635.992	232.565
42506	5.8531	590.052	176.543
42507	5.8532	641.165	44.7094
42508	5.8532	635.667	95.4673
42509	5.8533	723.112	169.239
42510	5.8536	630.778	72.1344
42511	5.8537	763.255	113.777
42512	5.8537	799.528	16.0932
42513	5.8538	680.109	79.7106
42514	5.8539	720.397	108.002
42515	5.854	748.191	132.738
42516	5.854	761.3	313.576
42517	5.854	661.035	111.744

42518	5.8541	541.279	1.9592
42519	5.8543	644.281	159.24
42520	5.8544	641.773	324.689
42521	5.8544	747.383	235.601
42522	5.8545	606.448	150.025
42523	5.8547	567.627	256.579
42524	5.8547	660.368	232.234
42525	5.8548	646.765	53.9699
42526	5.8551	691.786	106.381
42527	5.8551	785.663	189.024
42528	5.8552	736.279	36.1512
42529	5.8562	728.276	300.977
42530	5.8563	692.074	170.995
42531	5.8572	651.904	143.751
42532	5.8573	810.999	102.891
42533	5.8573	675.667	16.4705
42534	5.8574	610.047	130.129
42535	5.8575	646.368	111.088
42536	5.8576	694.28	137.35
42537	5.8578	713.428	110.328
42538	5.8578	583.045	191.407
42539	5.8579	702.42	40.3684
42540	5.858	631.632	162.501
42541	5.8581	685.204	246.985
42542	5.8582	719.42	286.832
42543	5.8587	698	161.821
42544	5.8589	618.793	88.9383
42545	5.8592	713.671	207.48
42546	5.8593	704.161	40.9988
42547	5.8594	599.825	102.985
42548	5.8595	597.391	129.66
42549	5.8596	627.695	117.762
42550	5.8597	720.94	41.7611
42551	5.8598	724.17	173.349
42552	5.8602	847.146	62.0666
42553	5.8602	748.826	334.317
42554	5.8604	715.712	84.9045
42555	5.8606	542.462	110.928
42556	5.8615	804.352	45.4183
42557	5.8616	823.848	47.0532
42558	5.8616	686.975	136.989
42559	5.8618	645.312	53.5945
42560	5.8621	689.468	99.4772
42561	5.8621	631.118	114.064
42562	5.8622	805.741	47.924
42563	5.8623	764.543	21.9759
42564	5.8623	894.187	98.6916
42565	5.8625	638.887	285.689

42566	5.8625	792.542	91.8021
42567	5.8628	798.953	81.9763
42568	5.8629	694.252	270.099
42569	5.8631	796.368	23.5961
42570	5.8633	691.705	26.7081
42571	5.8637	601.501	246.137
42572	5.864	705.075	110.119
42573	5.8641	636.254	174.152
42574	5.8641	585.153	80.1832
42575	5.8643	622.827	119.737
42576	5.8646	669.498	131.765
42577	5.8647	619.46	133.923
42578	5.8647	678.017	322.295
42579	5.8648	604.696	123.932
42580	5.8649	673.433	73.8288
42581	5.8649	483.191	1.068
42582	5.865	720.094	59.3972
42583	5.865	797.441	53.429
42584	5.8651	589.958	219.709
42585	5.8652	708.448	17.5954
42586	5.8653	636.095	140.7
42587	5.8653	669.823	151.704
42588	5.8658	600.767	92.3042
42589	5.8659	872.226	46.2988
42590	5.8661	547.954	191.242
42591	5.8662	694.48	259.722
42592	5.8663	616.301	21.9567
42593	5.8665	594.551	176.968
42594	5.8665	670.898	176.376
42595	5.8669	615.591	178.15
42596	5.8671	628.106	220.064
42597	5.8672	574.198	147.153
42598	5.8672	734.515	224.325
42599	5.8674	669.666	217.211
42600	5.8676	597.635	113.314
42601	5.8676	697.451	180.335
42602	5.8677	703.173	275.809
42603	5.8677	702.519	113.006
42604	5.8678	744.899	45.7032
42605	5.8679	651.352	183.594
42606	5.8679	624.951	128.082
42607	5.8681	590.292	231.245
42608	5.8684	563.828	343.369
42609	5.8685	684.819	103.347
42610	5.8686	770.735	86.127
42611	5.8689	724.558	73.5059
42612	5.8689	787.392	44.9601
42613	5.869	649.502	120.579

42614	5.869	711.178	163.374
42615	5.8693	737.708	66.3632
42616	5.8693	669.809	140.415
42617	5.8695	719.561	67.0695
42618	5.8695	632.097	109.937
42619	5.8695	783.377	235.974
42620	5.8696	715.95	197.049
42621	5.8697	731.89	338.697
42622	5.8697	802.034	283.725
42623	5.87	663.759	252.965
42624	5.87	578.813	169.925
42625	5.8704	769.157	79.1413
42626	5.8709	714.241	124.513
42627	5.871	617.375	156.655
42628	5.871	668.767	135.476
42629	5.8713	615.645	353.968
42630	5.8715	643.693	71.6887
42631	5.8716	753.089	183.02
42632	5.8717	599.836	178.335
42633	5.8717	691.301	196.044
42634	5.8718	179.385	22.5478
42635	5.8718	753.319	270.202
42636	5.8718	862.859	287.793
42637	5.8722	726.441	67.4163
42638	5.8724	663.611	99.1135
42639	5.8725	615.652	164.74
42640	5.8727	629.64	143.632
42641	5.8729	685.203	268.039
42642	5.8731	703.768	9.7121
42643	5.8732	818.393	25.8324
42644	5.8733	639.583	143.697
42645	5.8734	651.476	110.678
42646	5.8736	718.141	271.986
42647	5.8739	670.277	126.811
42648	5.8743	865.496	11.4645
42649	5.8748	771.199	99.5752
42650	5.8749	632.819	183.488
42651	5.8752	824.671	30.028
42652	5.8752	681.822	179.255
42653	5.8753	717.881	163.661
42654	5.8756	751.094	102.125
42655	5.8756	601.264	289.987
42656	5.8756	578.866	184.433
42657	5.8756	566.85	145.959
42658	5.8758	667.652	173.794
42659	5.8758	535.918	127.735
42660	5.876	677.022	109.784
42661	5.8762	725.335	349.893

42662	5.8762	665.838	106.264
42663	5.8762	760.457	23.7658
42664	5.8765	616.465	108.414
42665	5.8765	745.484	297.042
42666	5.8766	681.557	31.8474
42667	5.877	701.939	289.882
42668	5.8771	659.063	238.754
42669	5.8771	675.715	28.8689
42670	5.8773	811.418	287.633
42671	5.878	612.483	121.945
42672	5.8782	782.239	28.7961
42673	5.8783	536.022	72.7757
42674	5.8783	688.133	252.535
42675	5.8784	575.49	184.285
42676	5.8784	718.525	171.658
42677	5.8784	667.889	332.107
42678	5.8786	654.334	250.382
42679	5.8786	781.482	74.2884
42680	5.8787	724.732	230.312
42681	5.8789	532.131	60.9707
42682	5.879	660.559	114.673
42683	5.879	719.626	177.755
42684	5.8791	775.455	116.465
42685	5.8795	663.408	58.1304
42686	5.8797	730.472	211.657
42687	5.8805	602.24	260.908
42688	5.8807	619.431	178.814
42689	5.8807	646.366	107.754
42690	5.8807	821.023	23.3706
42691	5.8809	768.202	45.7653
42692	5.8811	651.617	183.696
42693	5.8817	649.531	299.389
42694	5.8819	519.767	178.538
42695	5.8819	593.224	122.712
42696	5.8822	815.065	21.5969
42697	5.8824	556.933	346.543
42698	5.8825	689.828	28.5411
42699	5.8825	848.174	13.5616
42700	5.8828	585.955	122.09
42701	5.8828	630.805	115.416
42702	5.8829	684.283	100.336
42703	5.883	688.553	236.752
42704	5.883	734.995	128.376
42705	5.883	623.406	193.534
42706	5.8831	611.632	148.139
42707	5.8832	652.912	52.0331
42708	5.8832	772.959	109.588
42709	5.8834	735.145	226.36

42710	5.8835	591.402	180.32
42711	5.8835	755.469	74.9236
42712	5.8835	597.016	7.3911
42713	5.8835	721.573	120.672
42714	5.8835	739.757	103.613
42715	5.884	799.943	206.412
42716	5.8843	715.807	78.4187
42717	5.8846	683.044	354.212
42718	5.8846	783.864	100.672
42719	5.8847	763.3	71.3702
42720	5.8851	730.049	219.315
42721	5.8853	749.37	89.2931
42722	5.8853	649.03	192.209
42723	5.8853	715.744	60.3814
42724	5.8853	683.08	192.139
42725	5.8855	625.557	304.939
42726	5.8855	464.313	177.532
42727	5.8856	671.137	150.627
42728	5.8857	749.447	55.3693
42729	5.8857	569.583	215.468
42730	5.8858	619.376	283.554
42731	5.8858	756.492	26.8742
42732	5.8858	741.875	76.2334
42733	5.886	673.116	131.259
42734	5.886	710.909	234.629
42735	5.8864	630.516	173.899
42736	5.8864	676.499	138.415
42737	5.8866	613.875	264.652
42738	5.8867	642.934	125.231
42739	5.8868	509.042	79.6284
42740	5.8874	698.252	247.61
42741	5.8876	730.992	356.318
42742	5.8878	579.262	245.293
42743	5.8878	582.995	57.564
42744	5.8879	643.234	227.662
42745	5.8879	686.392	143.665
42746	5.888	714.608	21.6213
42747	5.8881	817.725	50.5773
42748	5.8883	669.395	176.435
42749	5.8884	698.66	235.445
42750	5.8885	708.358	172.83
42751	5.8887	646.122	244.648
42752	5.8887	436.792	173.329
42753	5.8888	611.398	295.978
42754	5.8888	746.677	30.1657
42755	5.8888	703.731	301.94
42756	5.889	643.485	222.395
42757	5.8893	653.541	170.367

42758	5.8895	659.27	221.284
42759	5.8895	822.893	26.3779
42760	5.8896	264.108	204.598
42761	5.8896	684.903	322.533
42762	5.8896	761.497	76.3129
42763	5.8896	753.026	207.442
42764	5.8897	723.253	227.506
42765	5.8898	709.907	17.903
42766	5.8899	754.688	74.5226
42767	5.8899	625.528	141.756
42768	5.89	703.36	293.699
42769	5.8901	772.62	42.4981
42770	5.8902	672.924	280.542
42771	5.8903	610.617	114.549
42772	5.8903	665.054	109.878
42773	5.8904	683.495	13.6882
42774	5.8904	803.801	44.8896
42775	5.8905	670.759	231.176
42776	5.8905	632.537	225.201
42777	5.8906	650.715	163.557
42778	5.8907	626.452	124.697
42779	5.8909	714.464	96.9175
42780	5.891	712.072	356.742
42781	5.8913	622.914	303.33
42782	5.8915	809.024	17.1034
42783	5.8919	598.099	137.577
42784	5.8922	656.255	194.144
42785	5.8923	666.894	115.95
42786	5.8924	709.82	116.223
42787	5.8924	670.636	251.856
42788	5.8925	680.198	156.295
42789	5.8927	660.193	87.9909
42790	5.8927	-3.5863	78.3409
42791	5.8928	776.516	306.401
42792	5.8928	645.973	195.396
42793	5.8935	613.435	227.763
42794	5.8936	718.272	40.8187
42795	5.8942	674.287	172.853
42796	5.8944	707.504	228.242
42797	5.8946	855.796	86.8865
42798	5.8946	773.535	38.4516
42799	5.8949	655.821	317.774
42800	5.8951	557.828	221.976
42801	5.8951	636.196	324.258
42802	5.8953	624.055	72.4789
42803	5.8954	665.87	239.221
42804	5.8955	769.583	246.788
42805	5.8955	837.715	291.142

42806	5.8955	739.157	64.3033
42807	5.8955	707.474	183.165
42808	5.8958	814.822	36.5285
42809	5.8958	812.373	42.7654
42810	5.8959	950.876	78.8943
42811	5.896	630.661	309.114
42812	5.8962	700.652	142.331
42813	5.8965	811.039	84.3995
42814	5.8966	698.897	25.4534
42815	5.8966	541.92	171.236
42816	5.8967	785.788	20.6956
42817	5.8967	818.376	44.0262
42818	5.8968	648.358	197.729
42819	5.8969	646.813	240.663
42820	5.8972	743.306	223.5
42821	5.8973	698.006	128.892
42822	5.8975	5.8711	105.549
42823	5.8979	752.512	68.6125
42824	5.8979	708.26	78.6062
42825	5.898	687.324	231.8
42826	5.898	630.19	12.2947
42827	5.8984	702.02	192.438
42828	5.8985	690.168	278.348
42829	5.8985	664.309	109.724
42830	5.8987	854.318	57.6366
42831	5.8988	715.096	31.9082
42832	5.8994	709.777	92.133
42833	5.8995	474.255	347.787
42834	5.8995	458.829	61.5354
42835	5.8996	605.438	146.45
42836	5.8996	796.195	23.3561
42837	5.9003	673.955	246.759
42838	5.9003	852.87	52.5664
42839	5.9004	693.248	195.8
42840	5.9004	743.826	21.9671
42841	5.9004	857.962	33.8676
42842	5.9007	-2.7988	256.296
42843	5.9012	641.852	153.538
42844	5.9012	672.244	282.153
42845	5.9012	585.876	89.6235
42846	5.9013	660.402	162.242
42847	5.9015	691.392	141.979
42848	5.9017	656.356	286.467
42849	5.9017	791.155	49.1027
42850	5.9018	783.952	329.749
42851	5.9021	647.254	290.973
42852	5.9021	707.626	174.752
42853	5.9032	851.024	40.906

42854	5.9034	596.841	141.905
42855	5.9035	779.759	22.9909
42856	5.9036	667.897	97.3147
42857	5.9038	606.012	164.022
42858	5.9039	723.955	72.9827
42859	5.904	683.434	131.045
42860	5.9043	803.18	58.8934
42861	5.9044	759.018	5.1867
42862	5.9046	643.384	233.209
42863	5.9047	711.151	164
42864	5.905	799.267	72.2783
42865	5.9051	697.983	248.36
42866	5.9053	582.829	187.475
42867	5.9059	658.992	35.2947
42868	5.9059	555.57	132.545
42869	5.906	663.587	101.724
42870	5.906	833.549	42.3293
42871	5.9062	773.92	156.789
42872	5.9067	713.071	333.528
42873	5.9067	717.766	124.053
42874	5.9068	847.227	47.8795
42875	5.907	673.875	129.797
42876	5.9071	695.654	294.735
42877	5.9073	769.655	303.012
42878	5.9074	760.338	160.135
42879	5.9076	682.422	111.504
42880	5.9076	762.034	91.2062
42881	5.9079	645.883	287.372
42882	5.9085	-4.0877	262.455
42883	5.9086	783.175	33.8221
42884	5.9088	672.71	233.641
42885	5.9089	696.339	150.702
42886	5.9089	620.056	131.055
42887	5.909	712.975	103.644
42888	5.909	624.217	147.613
42889	5.9092	794.692	22.743
42890	5.9093	805.88	201.49
42891	5.9099	905.754	23.6991
42892	5.9099	733.344	124.613
42893	5.9099	785.754	115.067
42894	5.9103	678.177	251.789
42895	5.9104	805.849	44.7893
42896	5.9106	743.273	315.167
42897	5.9106	665.498	57.5133
42898	5.9107	689.437	132.496
42899	5.9109	601.337	253.185
42900	5.911	653.195	298.463
42901	5.9111	830.921	22.2487

42902	5.9112	664.415	258.863
42903	5.9112	631.865	146.974
42904	5.9117	746.313	53.3904
42905	5.9121	701.93	135.373
42906	5.9123	648.948	233.576
42907	5.9124	692.699	331.122
42908	5.9126	481.49	334.329
42909	5.9129	841.776	257.461
42910	5.9131	786.367	95.535
42911	5.9136	661.048	140.4
42912	5.9137	655.724	51.4156
42913	5.9138	817.079	100.884
42914	5.9139	702.948	300.26
42915	5.9139	793.891	36.0416
42916	5.9142	605.002	134.483
42917	5.9143	689.802	181.534
42918	5.9145	792.445	73.4755
42919	5.9146	557.009	175.529
42920	5.9147	707.773	350.698
42921	5.9149	791.523	19.1223
42922	5.915	773.544	20.6893
42923	5.9152	723.52	27.5981
42924	5.9153	714.272	163.151
42925	5.9156	711.498	132.751
42926	5.9156	785.353	40.7872
42927	5.9159	726.625	136.573
42928	5.9159	602.363	317.424
42929	5.916	789.339	205.939
42930	5.9161	635.181	292.026
42931	5.9162	806.649	244.758
42932	5.9166	692.954	267.609
42933	5.9167	513.096	254.835
42934	5.917	736.562	103.261
42935	5.9173	718.957	137.26
42936	5.9173	780.605	24.223
42937	5.9174	596.364	190.6
42938	5.9174	670.372	142.941
42939	5.9175	676.646	274.398
42940	5.9175	696.124	234.006
42941	5.9175	739.741	74.9008
42942	5.9175	787.641	42.2388
42943	5.9176	714.99	356.261
42944	5.9178	655.013	116.905
42945	5.918	902.387	79.8622
42946	5.9183	646.587	181.047
42947	5.9185	691.027	166.258
42948	5.9185	870.641	242.565
42949	5.9187	696.217	157.794

42950	5.9195	666.783	62.3463
42951	5.9195	686.075	82.9369
42952	5.9196	968.526	204.743
42953	5.9197	738.291	338.555
42954	5.92	801.284	42.0135
42955	5.92	647.836	155.176
42956	5.9201	783.373	16.5522
42957	5.9203	825.66	244.385
42958	5.9205	647.748	144.754
42959	5.9206	644.195	257.488
42960	5.9206	689.614	171.959
42961	5.9208	713.506	100.825
42962	5.9211	665.961	191.142
42963	5.9214	592.354	99.582
42964	5.9218	839.396	34.8161
42965	5.9219	687.003	77.002
42966	5.922	686.767	117.033
42967	5.9221	582.05	131.639
42968	5.9223	825.447	99.1421
42969	5.9225	688.739	182.49
42970	5.9225	843.46	58.5189
42971	5.9226	-3.5162	323.649
42972	5.9226	540.053	213.095
42973	5.9226	742.095	102.366
42974	5.9229	636.527	173.455
42975	5.9229	757.872	85.9041
42976	5.9229	679.857	10.6363
42977	5.923	749.805	26.6869
42978	5.9233	790.949	91.7583
42979	5.9233	791.942	93.9844
42980	5.9234	818.738	16.8328
42981	5.9235	629.472	176.251
42982	5.9236	800.344	77.5321
42983	5.9236	745.97	73.8956
42984	5.9239	569.102	127.195
42985	5.924	828.795	48.1368
42986	5.9243	833.033	32.9664
42987	5.9243	714.058	69.433
42988	5.9244	741.693	272.666
42989	5.9246	627.846	213.928
42990	5.9247	594.696	241.165
42991	5.925	685.802	321.556
42992	5.9251	776.704	358.65
42993	5.9251	749.007	76.1826
42994	5.9253	685.287	92.7466
42995	5.9256	743.031	300.686
42996	5.9257	241.515	230.181
42997	5.9259	788.892	93.11

42998	5.9262	755.178	43.1463
42999	5.9262	715.061	250.564
43000	5.9262	517.863	101.576
43001	5.9268	595.1	94.2204
43002	5.9268	694.796	36.053
43003	5.9269	773.161	46.9885
43004	5.9272	754.76	187.709
43005	5.9272	722.323	152.936
43006	5.9273	745.863	356.826
43007	5.9273	644.441	70.5285
43008	5.9276	785.039	18.4369
43009	5.9278	813.076	118.432
43010	5.9281	622.455	150.054
43011	5.9283	708.939	258.04
43012	5.9284	627.862	50.1761
43013	5.9286	681.013	265.125
43014	5.9287	666.237	159.829
43015	5.9289	802.085	152.301
43016	5.9289	668.635	137.547
43017	5.9291	715.999	301.24
43018	5.9293	716.616	187.305
43019	5.9293	770.978	89.1694
43020	5.9297	604.038	180.997
43021	5.9298	717.619	155.123
43022	5.9302	692.237	217.779
43023	5.9302	583.241	105.122
43024	5.9303	806.83	22.0729
43025	5.9303	614.504	253.66
43026	5.9303	793.17	103.84
43027	5.9305	703.296	101.93
43028	5.9307	796.943	71.4836
43029	5.9308	642.624	293.538
43030	5.9308	811.351	22.0136
43031	5.9312	842.824	33.5132
43032	5.9312	708.1	59.1354
43033	5.9313	652.286	183.126
43034	5.9313	710.002	58.6038
43035	5.9319	654.163	227.036
43036	5.932	720.94	9.6417
43037	5.9321	646.871	137.307
43038	5.9322	648.732	104.078
43039	5.9327	734.209	132.024
43040	5.9327	695.804	329.794
43041	5.9328	612.16	140.142
43042	5.9329	639.984	187.887
43043	5.9329	613.571	62.0346
43044	5.9332	701.342	331.252
43045	5.9333	615.212	173.331

43046	5.9335	657.43	190.7
43047	5.9336	716.101	72.0164
43048	5.9337	697.956	4.7087
43049	5.9337	635.623	100.706
43050	5.9337	733.072	25.1374
43051	5.9338	707.95	81.7208
43052	5.934	722.126	136.622
43053	5.9341	859.244	21.3358
43054	5.9342	897.654	261.05
43055	5.9342	832.873	271.917
43056	5.9344	771.202	98.0482
43057	5.9345	667.112	182.155
43058	5.9346	830.779	21.8987
43059	5.9347	722.332	141.454
43060	5.9347	649.181	116.556
43061	5.9347	603.021	36.5819
43062	5.9349	597.04	182.905
43063	5.9349	852.456	15.1336
43064	5.9355	573.714	158.126
43065	5.9359	797.469	29.9926
43066	5.9359	829.968	106.676
43067	5.9361	548.172	164.246
43068	5.9363	799.82	101.438
43069	5.9365	587.824	328.339
43070	5.9365	688.606	184.341
43071	5.9366	-2.8046	258.256
43072	5.937	765.788	55.1187
43073	5.9373	600.492	94.7471
43074	5.9373	599.2	47.829
43075	5.9373	782.878	316.332
43076	5.9374	774.876	100.277
43077	5.9375	799.028	28.6617
43078	5.9376	735.558	83.059
43079	5.9376	807.013	90.5026
43080	5.9378	770.119	3.0173
43081	5.938	681.515	173.243
43082	5.9382	654.127	247.969
43083	5.9382	655.74	262.491
43084	5.9384	710.557	143.717
43085	5.9384	585.756	133.458
43086	5.9384	802.055	80.8652
43087	5.9386	683.237	97.9389
43088	5.9386	811.951	63.0961
43089	5.9392	832.071	41.3913
43090	5.9393	732.315	71.1002
43091	5.9397	832.249	44.3008
43092	5.9398	727.074	76.3896
43093	5.9401	692.629	179.648

43094	5.9401	591.74	195.979
43095	5.9403	778.764	81.856
43096	5.9403	799.062	51.8687
43097	5.9404	809.291	201.251
43098	5.9408	797.042	103.483
43099	5.941	-2.8603	98.0666
43100	5.9411	732.384	122.581
43101	5.9412	809.673	117.073
43102	5.9413	621.663	220.773
43103	5.9413	756.555	63.1791
43104	5.9413	672.673	162.817
43105	5.9414	690.392	93.8163
43106	5.9414	617.616	284.812
43107	5.9415	803.198	33.8389
43108	5.9418	670.054	158.977
43109	5.9422	758.779	181.97
43110	5.9427	604.213	150.854
43111	5.9428	719.554	67.6104
43112	5.9433	677.238	181.575
43113	5.9435	692.758	181.358
43114	5.9435	655.702	166.057
43115	5.9437	678.227	64.624
43116	5.9439	665.87	99.8315
43117	5.944	820.374	302.18
43118	5.944	599.682	237.977
43119	5.944	657.468	73.9258
43120	5.9441	641.34	220.072
43121	5.9443	603.467	197.85
43122	5.9444	681.414	70.2241
43123	5.9444	666.922	256.534
43124	5.9445	725.044	69.6979
43125	5.9445	773.467	233.121
43126	5.9447	740.974	80.3138
43127	5.9448	644.054	179.979
43128	5.9448	757.179	111.515
43129	5.9449	659.966	196.626
43130	5.9452	683.382	200.689
43131	5.9452	752.212	183.808
43132	5.9461	759.166	102.235
43133	5.9463	703.899	106.178
43134	5.9465	685.274	165.912
43135	5.9468	471.135	141.605
43136	5.9469	638.497	222.138
43137	5.9471	653.148	87.1164
43138	5.9472	722.229	146.228
43139	5.9475	677.13	160.783
43140	5.9477	828.179	45.1876
43141	5.9479	728.161	171.107

43142	5.9479	672.304	226.837
43143	5.9484	688.189	127.301
43144	5.9484	684.312	252.434
43145	5.9485	696.279	117.175
43146	5.9487	633.223	255.691
43147	5.9488	728.464	256.085
43148	5.9491	758.18	124.238
43149	5.9491	659.701	147.848
43150	5.9491	680.88	157.289
43151	5.9491	732.98	107.974
43152	5.9494	661.397	214.23
43153	5.9496	806.416	29.8616
43154	5.9497	633.64	72.0952
43155	5.9498	736.658	152.776
43156	5.9505	803.299	35.9903
43157	5.9507	695.033	111.616
43158	5.9509	775.394	280.35
43159	5.9514	681.965	123.578
43160	5.9514	876.333	11.8805
43161	5.9516	714.912	258.234
43162	5.9518	736.285	146.244
43163	5.9519	749.57	235.246
43164	5.9522	682.873	143.258
43165	5.9524	761.595	63.9251
43166	5.9524	680.12	199.39
43167	5.9525	759.527	82.4501
43168	5.9525	730.605	76.861
43169	5.9528	735.182	179.777
43170	5.9529	905.612	27.8276
43171	5.9529	617.302	188.269
43172	5.9533	699.26	75.336
43173	5.9533	602.741	180.123
43174	5.9534	693.783	238.387
43175	5.9536	723.921	177.948
43176	5.9536	642.164	249.696
43177	5.9538	638.137	290.418
43178	5.9538	613.267	96.0205
43179	5.954	803.538	57.6652
43180	5.954	770.277	140.807
43181	5.9543	791.546	21.3834
43182	5.9545	806.92	98.3204
43183	5.9545	620.933	124.645
43184	5.9546	710.283	142.971
43185	5.9552	837.563	45.6671
43186	5.9556	598.138	228.235
43187	5.9556	593.841	113.388
43188	5.9559	694.135	81.7518
43189	5.956	706.535	134.696

43190	5.956	703.656	132.82
43191	5.9561	642.055	218.609
43192	5.9566	747.761	11.2795
43193	5.9569	695.954	282.588
43194	5.957	743.373	11.8174
43195	5.9571	676.428	297.36
43196	5.9571	582.734	219.82
43197	5.9574	686.351	85.6469
43198	5.9574	766.147	128.799
43199	5.958	609.557	146.861
43200	5.9581	788.313	42.5468
43201	5.9582	644.772	178.593
43202	5.9583	699.187	154.841
43203	5.9583	889.284	24.9339
43204	5.9585	683.47	195.194
43205	5.9586	761.107	110.486
43206	5.9588	616.378	174.373
43207	5.9588	690.107	86.724
43208	5.9588	618.188	52.3177
43209	5.9588	780.685	64.934
43210	5.9591	727.079	112.643
43211	5.9592	561.253	69.2546
43212	5.9595	813.911	259.542
43213	5.9601	678.245	284.81
43214	5.9602	653.621	218.277
43215	5.9602	706.242	318.929
43216	5.9603	801.771	56.411
43217	5.9605	835.424	103.371
43218	5.9605	689.729	190.361
43219	5.9605	569.93	85.1929
43220	5.9609	768.16	18.2511
43221	5.961	687.515	320.632
43222	5.9611	660.926	122.763
43223	5.9611	695.909	195.85
43224	5.9613	699.642	248.258
43225	5.9616	668.37	140.202
43226	5.9616	833.882	330.437
43227	5.9617	742.674	73.538
43228	5.962	661.133	251.666
43229	5.9625	849.087	44.9079
43230	5.9625	708.009	52.8883
43231	5.9626	879.618	43.991
43232	5.9627	767.447	158.109
43233	5.9628	660.845	138.847
43234	5.9629	626.383	185.949
43235	5.9629	830.132	77.1489
43236	5.963	762.266	51.7724
43237	5.9631	697.642	151.757

43238	5.9632	686.739	46.3141
43239	5.9632	631.39	252.542
43240	5.9633	743.656	274.341
43241	5.9634	597.45	186.062
43242	5.9634	928.134	10.5109
43243	5.9635	740.534	254.153
43244	5.9638	746.255	70.5712
43245	5.964	709.879	164.348
43246	5.9642	806.781	32.4239
43247	5.9646	861.415	42.0339
43248	5.9651	702.585	100.216
43249	5.9657	936.329	24.7007
43250	5.9659	815.029	255.536
43251	5.9659	858.84	49.322
43252	5.966	649.993	160.283
43253	5.9662	800.144	270.256
43254	5.9662	557.063	232.096
43255	5.9664	650.627	228.935
43256	5.9665	859.617	42.5922
43257	5.9669	620.574	351.682
43258	5.9673	583.23	184.327
43259	5.9674	816.399	148.289
43260	5.9675	931.867	260.742
43261	5.9676	787.395	200.233
43262	5.9678	629.193	124.13
43263	5.9679	769.208	355.948
43264	5.968	675.289	62.3699
43265	5.968	729.651	10.91
43266	5.9681	712.958	163.417
43267	5.9685	824.571	25.0386
43268	5.9685	740.552	95.9524
43269	5.9685	739.25	177.769
43270	5.9693	804.295	38.8279
43271	5.9694	711.641	232.504
43272	5.9697	690.232	135.016
43273	5.9697	605.23	76.3843
43274	5.9698	797.448	40.2109
43275	5.9698	835.98	95.0227
43276	5.9699	752.368	11.7396
43277	5.9702	913.733	41.0645
43278	5.9707	754.068	77.1997
43279	5.971	820.324	64.1612
43280	5.971	898.709	29.8801
43281	5.9711	735.048	92.8152
43282	5.9712	667.427	120.758
43283	5.9713	910.003	99.8227
43284	5.9713	791.997	87.6504
43285	5.9714	842.963	253.542

43286	5.9714	819.962	110.205
43287	5.9716	638.487	249.594
43288	5.9717	690.505	178.689
43289	5.9718	693.13	177.235
43290	5.9718	706.407	133.519
43291	5.972	727.435	149.914
43292	5.972	672.553	136.578
43293	5.9724	668.756	164.603
43294	5.9724	615.462	182.419
43295	5.9725	648.398	291.342
43296	5.9725	685.909	96.9161
43297	5.9727	652.712	181.8
43298	5.9729	737.924	27.4032
43299	5.9731	689.87	194.939
43300	5.9738	828.749	87.7305
43301	5.9739	732.372	19.732
43302	5.9742	662.037	127.248
43303	5.9743	820.081	98.3269
43304	5.9744	686.313	286.955
43305	5.9746	670.734	157.543
43306	5.9751	813.436	303.475
43307	5.9751	575.512	146.09
43308	5.9753	727.931	43.4567
43309	5.9755	781.344	139.885
43310	5.9755	795.102	20.7406
43311	5.9755	716.949	106.383
43312	5.9756	572.937	220.152
43313	5.9758	689.601	128.416
43314	5.9762	718.069	169.105
43315	5.9762	717.96	78.2852
43316	5.9765	755.242	71.252
43317	5.9766	670.702	179.617
43318	5.9766	602.787	117.128
43319	5.9766	-2.7994	77.7414
43320	5.9767	721.379	42.0047
43321	5.9767	762.583	94.2129
43322	5.9768	673.162	230.478
43323	5.977	821.218	226.236
43324	5.9773	694.201	104.515
43325	5.9774	715.709	132.315
43326	5.9776	798.999	99.0214
43327	5.9782	826.466	258.732
43328	5.9782	686.918	227.48
43329	5.9782	762.523	76.8708
43330	5.9784	820.843	50.6885
43331	5.9787	703.345	293.896
43332	5.9789	685.359	261.044
43333	5.979	687.434	269.779

43334	5.979	561.563	184.973
43335	5.9793	753.103	85.9112
43336	5.9794	637.23	110.745
43337	5.9794	629.102	221.374
43338	5.9794	666.113	23.4851
43339	5.9795	711.019	124.953
43340	5.9798	684.566	144.295
43341	5.9799	734.293	59.7833
43342	5.98	695.676	222.56
43343	5.9801	752.016	30.1495
43344	5.9805	660.163	169.917
43345	5.9806	672.128	195.445
43346	5.9806	913.238	203.213
43347	5.9809	830.582	76.1016
43348	5.9812	845.153	252.794
43349	5.9816	721.217	144.655
43350	5.9818	562.591	156.883
43351	5.982	717.533	134.217
43352	5.982	653.792	123.771
43353	5.9822	650.351	241.654
43354	5.9823	774.413	291.155
43355	5.9823	684.817	145.873
43356	5.9824	624.464	123.358
43357	5.9825	483.194	358.865
43358	5.9825	797.911	205.075
43359	5.9826	730.685	191.812
43360	5.9826	713.776	57.2576
43361	5.9827	696.623	96.4207
43362	5.9829	665.339	63.7748
43363	5.983	763.693	144.939
43364	5.9834	425.509	8.2698
43365	5.9835	733.038	7.6981
43366	5.9837	727.483	50.4012
43367	5.9839	218.119	23.1803
43368	5.9843	742.354	226.547
43369	5.9843	699.1	148.245
43370	5.9843	684.734	263.811
43371	5.9844	771.778	61.0291
43372	5.9849	790.394	100.023
43373	5.9852	720.231	165.684
43374	5.9852	693.33	19.6714
43375	5.9853	734.837	120.539
43376	5.9853	-2.8176	77.4247
43377	5.9857	683.732	69.3001
43378	5.9858	725.631	27.8324
43379	5.9859	880.553	49.5812
43380	5.9861	667.701	92.0797
43381	5.9862	732.13	113.196

43382	5.9864	693.151	157.99
43383	5.9866	728.172	146.764
43384	5.9866	678.741	150.925
43385	5.9866	784.099	96.688
43386	5.9868	665.857	175.07
43387	5.9869	787.488	2.1518
43388	5.9874	768.328	43.5055
43389	5.9877	864.42	23.4954
43390	5.9878	785.524	90.0804
43391	5.9879	794.662	105.175
43392	5.9882	-3.4951	79.6944
43393	5.9886	706.69	120.419
43394	5.9887	767.531	102.855
43395	5.989	819.874	357.347
43396	5.989	892.805	57.6273
43397	5.9893	587.114	217.591
43398	5.9895	676.405	339.951
43399	5.9898	662.458	57.4776
43400	5.9901	821.429	21.5998
43401	5.9901	744.415	173.84
43402	5.9901	722.107	66.8585
43403	5.9901	772.297	101.015
43404	5.9902	691.309	85.5608
43405	5.9903	781.978	58.9062
43406	5.9907	640.656	228.798
43407	5.9912	881.803	44.9712
43408	5.9913	657.66	173.248
43409	5.9916	808.508	50.1863
43410	5.9917	649.401	225.792
43411	5.9918	723.158	248.122
43412	5.992	741.034	173.359
43413	5.9921	655.919	155.859
43414	5.9921	674.105	18.1114
43415	5.9922	762.19	58.9585
43416	5.9926	727.084	304.041
43417	5.9929	631.338	184.426
43418	5.993	649.486	354.132
43419	5.9931	717.273	93.6455
43420	5.9931	706.953	178.416
43421	5.9932	702.876	126.932
43422	5.9935	876.469	259.531
43423	5.9937	823.601	51.7541
43424	5.9944	777.528	40.146
43425	5.9945	808.102	142.279
43426	5.9947	801.077	76.7587
43427	5.9951	858.448	45.414
43428	5.9952	855.042	22.8502
43429	5.9954	792.428	73.0705

43430	5.9957	650.321	48.5452
43431	5.9962	753.214	78.1467
43432	5.9963	822.233	39.8686
43433	5.9964	811.594	127.036
43434	5.9965	755.567	6.2852
43435	5.9967	925.591	260.182
43436	5.997	657.107	265.287
43437	5.9973	612.51	135.936
43438	5.9973	828.048	47.5824
43439	5.9973	792.142	54.7163
43440	5.9974	817.137	120.463
43441	5.9975	711.096	151.354
43442	5.9979	657.755	71.4747
43443	5.9979	812.423	89.3573
43444	5.998	637.374	189.317
43445	5.9982	679.01	178.748
43446	5.9982	634.866	138.182
43447	5.9984	830.164	66.6334
43448	5.9988	760.114	195.549
43449	5.999	683.993	144.954
43450	5.9991	685.179	177.261
43451	5.9993	775.634	186.717
43452	5.9994	789.966	55.5121
43453	5.9995	839.014	44.6902
43454	5.9996	687.897	70.3201
43455	5.9997	731.573	229.99
43456	6.0001	747.811	29.7043
43457	6.0001	854.471	41.3638
43458	6.0002	840.35	53.3157
43459	6.0003	653.222	33.5868
43460	6.0003	646.193	224.087
43461	6.0004	769.67	187.225
43462	6.0006	645.119	155.604
43463	6.0008	634.371	186.632
43464	6.0009	778.185	157.489
43465	6.0013	858.648	169.973
43466	6.0013	685.752	15.8405
43467	6.0013	712.447	295.205
43468	6.0015	711.501	172.085
43469	6.0018	858.431	43.0805
43470	6.0024	773.702	340.238
43471	6.0025	656.58	349.154
43472	6.003	730.404	250.83
43473	6.0031	647.546	189.393
43474	6.0038	674.566	247.555
43475	6.0039	668.241	40.9601
43476	6.0045	596.866	154.287
43477	6.0045	615.391	31.5288

43478	6.0047	688.96	159.757
43479	6.0048	590.84	184.284
43480	6.0055	697.401	229.789
43481	6.0059	818.046	305.245
43482	6.006	853.94	11.2829
43483	6.0062	753.007	127.525
43484	6.0063	731.927	112.622
43485	6.007	741.947	86.3036
43486	6.0071	635.11	173.494
43487	6.0074	562.2	306.164
43488	6.0076	813.407	103.827
43489	6.0077	738.742	75.6291
43490	6.0078	743.924	143.145
43491	6.0079	687.806	112.089
43492	6.0079	706.584	68.3995
43493	6.0079	662.166	163.17
43494	6.008	671.434	159.906
43495	6.008	645.749	266.703
43496	6.0081	671.702	78.7088
43497	6.0083	781.058	30.2997
43498	6.0085	649.413	133.473
43499	6.0085	698.206	95.8486
43500	6.0086	717.101	37.2549
43501	6.0087	899.06	24.4359
43502	6.0089	782.016	305.218
43503	6.0093	780.043	127.232
43504	6.0094	873.364	53.5259
43505	6.0099	786.776	110.143
43506	6.0101	852.493	54.8665
43507	6.0107	658.462	222.972
43508	6.0111	734.756	116.786
43509	6.0111	565.265	73.342
43510	6.0112	767.626	261.081
43511	6.0113	917.826	42.0963
43512	6.0114	761.504	301.097
43513	6.0123	787.915	30.2005
43514	6.0123	724.2	261.569
43515	6.0125	713.026	70.1186
43516	6.0126	736.866	165.083
43517	6.0127	684.11	294.164
43518	6.0127	648.818	197.769
43519	6.0128	708.681	52.7279
43520	6.0129	800.54	216.273
43521	6.0129	735.325	126.362
43522	6.013	754.226	232.54
43523	6.013	843.352	95.4009
43524	6.0131	712.254	229.151
43525	6.0132	832.788	19.3155

43526	6.0133	642.864	180.233
43527	6.0133	619.369	177.026
43528	6.014	696.327	172.545
43529	6.0141	826.114	151.518
43530	6.0142	874.899	10.7853
43531	6.0142	712.918	135.969
43532	6.0145	784.287	129.715
43533	6.0147	791.505	15.0013
43534	6.0147	907.767	20.7828
43535	6.0149	625.65	191.075
43536	6.0151	803.973	334.201
43537	6.0152	807.628	61.9683
43538	6.0157	762.97	6.7667
43539	6.0161	779.682	42.9152
43540	6.0162	770.725	155.004
43541	6.0163	821.064	44.82
43542	6.0164	765.411	171.477
43543	6.0166	758.342	89.9169
43544	6.017	771.25	143.555
43545	6.0171	698.419	159.175
43546	6.0172	746.948	146.366
43547	6.0172	792.917	168.859
43548	6.0172	760.61	296.785
43549	6.0175	779.828	49.6568
43550	6.0182	744.687	102.459
43551	6.0184	776.142	196.129
43552	6.019	779.992	127.661
43553	6.0193	835.934	60.0083
43554	6.0198	635.575	235.298
43555	6.0199	804.412	44.1453
43556	6.0199	847.955	80.8259
43557	6.02	639.395	341.282
43558	6.02	594.128	114.443
43559	6.0201	837.572	41.207
43560	6.0202	792.31	332.479
43561	6.0204	897.381	55.1914
43562	6.0204	794.629	209.886
43563	6.0207	722.318	109.739
43564	6.0207	823.626	90.339
43565	6.0208	605.202	186.42
43566	6.0208	640.066	118.459
43567	6.021	674.173	113.493
43568	6.0212	658.749	36.69
43569	6.0213	855.636	46.993
43570	6.0216	859.372	37.0413
43571	6.0218	688.769	190.087
43572	6.0218	744.974	110.438
43573	6.0219	660.983	195.319

43574	6.0219	751.858	167.379
43575	6.0221	569.602	296.301
43576	6.0221	829.994	96.5393
43577	6.0221	871.194	14.8033
43578	6.0222	736.862	101.127
43579	6.0223	699.615	86.5843
43580	6.0225	828.39	21.4187
43581	6.0226	868.87	257.158
43582	6.0228	851.001	207.308
43583	6.0229	647.807	341.068
43584	6.0229	768.835	53.9301
43585	6.0229	799.276	199.064
43586	6.0229	822.634	81.5451
43587	6.0234	759.229	119.835
43588	6.0235	724.474	177.135
43589	6.0236	755.436	68.8662
43590	6.0237	734.307	188.32
43591	6.0238	747.437	85.6971
43592	6.0241	887.628	8.1817
43593	6.0241	689.266	331.411
43594	6.0242	767.678	67.123
43595	6.0242	785.252	170.932
43596	6.0242	780.373	24.8872
43597	6.0243	736.081	192.341
43598	6.0243	647.451	172.233
43599	6.0245	767.634	88.0053
43600	6.0246	745.029	257.743
43601	6.0248	719.892	146.845
43602	6.0254	712.988	284.925
43603	6.0254	798.338	320.716
43604	6.0255	691.075	187.166
43605	6.0257	679.955	198.987
43606	6.0257	946.325	10.1286
43607	6.0258	694.983	190.873
43608	6.0258	661.816	145.494
43609	6.0258	740.502	104.146
43610	6.0258	785.485	320.532
43611	6.0261	866.872	30.1181
43612	6.0262	870.644	32.9826
43613	6.0262	765.963	199.516
43614	6.0263	723.685	122.779
43615	6.0263	757.826	327.453
43616	6.0264	828.635	228.963
43617	6.0266	586.809	177.423
43618	6.0274	718.614	14.772
43619	6.0279	817.593	88.4852
43620	6.028	793.016	225.011
43621	6.0283	677.587	146.465

43622	6.0284	698.996	230.325
43623	6.0285	771.236	317.851
43624	6.0291	763.638	33.1335
43625	6.0291	887.546	35.0725
43626	6.0292	751.575	126.228
43627	6.0294	772.008	40.2337
43628	6.0294	772.168	80.8223
43629	6.0298	707.873	166.377
43630	6.0298	661.95	84.0345
43631	6.0299	864.265	97.1168
43632	6.0305	718.224	135.747
43633	6.0308	879.428	14.3431
43634	6.031	876.861	88.985
43635	6.0315	684.024	260.189
43636	6.0316	675.017	147.363
43637	6.0316	703.667	215.71
43638	6.0318	761.73	57.6882
43639	6.0319	787.941	61.914
43640	6.0322	598.19	145.359
43641	6.0323	637.226	224.712
43642	6.0325	675.794	17.0512
43643	6.0326	781.126	129.514
43644	6.0326	689.874	159.556
43645	6.033	847.147	102.662
43646	6.0332	615.602	146.089
43647	6.0332	726.254	314.716
43648	6.0332	712.467	148.538
43649	6.0334	672.099	303.604
43650	6.0335	827.048	43.4513
43651	6.0337	752.244	95.3152
43652	6.0337	847.475	57.5107
43653	6.0338	655.815	328.842
43654	6.0339	881.242	289.242
43655	6.0341	732.936	77.6935
43656	6.0342	723.177	333.647
43657	6.0342	708.469	233.358
43658	6.0346	861.301	27.2329
43659	6.0346	811.201	272.195
43660	6.0347	729.754	79.3391
43661	6.0347	687.634	74.7262
43662	6.0351	882.615	41.7001
43663	6.0352	-2.9379	314.823
43664	6.0353	891.063	28.7048
43665	6.0354	666.642	125.538
43666	6.0355	779.188	303.094
43667	6.0356	583.79	182.737
43668	6.0356	662.013	143.38
43669	6.0357	753.54	51.5626

43670	6.0359	806.208	31.9856
43671	6.036	718.934	230.635
43672	6.036	769.212	263.044
43673	6.036	715.499	193.98
43674	6.0363	647.033	219.478
43675	6.0364	726.441	259.424
43676	6.0368	736.181	135.23
43677	6.0372	689.149	220.003
43678	6.0373	729.52	16.8726
43679	6.0374	688.628	204.044
43680	6.0375	732.963	293.267
43681	6.0376	847.6	52.2465
43682	6.0376	796.122	22.4389
43683	6.0376	611.002	135.461
43684	6.0376	835.464	295.673
43685	6.0379	697.696	78.28
43686	6.0379	891.891	42.0442
43687	6.038	780.443	113.413
43688	6.0384	731.229	123.523
43689	6.0385	694.197	84.1392
43690	6.0386	792.98	82.7051
43691	6.0387	718.495	225.547
43692	6.0387	537.561	59.5684
43693	6.0388	835.367	142.371
43694	6.0388	652.653	325.081
43695	6.0389	795.568	61.2671
43696	6.0392	726.974	302.289
43697	6.0392	678.958	221.302
43698	6.0394	761.215	291.673
43699	6.0395	752.635	47.9105
43700	6.0396	688.372	306.877
43701	6.0396	483.184	356.595
43702	6.0396	777.594	107.038
43703	6.0398	713.788	38.0476
43704	6.0399	926.127	40.348
43705	6.0402	660.868	146.585
43706	6.0403	-2.7797	265.63
43707	6.0404	830.232	52.5115
43708	6.0405	761.164	123.903
43709	6.0408	786.548	193.09
43710	6.0408	633.629	163.639
43711	6.0408	821.605	357.319
43712	6.0409	828.765	65.1674
43713	6.0409	611.817	221.638
43714	6.0409	767.678	181.804
43715	6.041	865.962	24.2966
43716	6.041	636.753	65.0989
43717	6.041	774.735	136.75

43718	6.0415	750.971	133.806
43719	6.0417	696.34	250.818
43720	6.042	783.791	232.013
43721	6.0423	751.728	82.8806
43722	6.0423	805.333	23.6809
43723	6.0424	853.371	141.839
43724	6.0424	771.611	130.359
43725	6.0429	833.322	46.9818
43726	6.043	692.44	184.364
43727	6.043	773.68	82.6747
43728	6.043	778.495	160.2
43729	6.0431	853.013	91.799
43730	6.0436	697.498	299.464
43731	6.0436	737.635	11.594
43732	6.0437	945.334	44.0882
43733	6.044	725.716	222.554
43734	6.0443	599.752	202.739
43735	6.0443	712.495	66.2424
43736	6.0447	748.067	273.058
43737	6.045	592.498	180.246
43738	6.0452	735.362	142.753
43739	6.0454	698.559	140.19
43740	6.0454	869.511	44.5781
43741	6.0457	667.098	67.3384
43742	6.0458	765.733	227.407
43743	6.0458	697.983	177.171
43744	6.046	757.591	159.157
43745	6.046	708.907	179.991
43746	6.0462	715.64	227.13
43747	6.0469	872.206	315.265
43748	6.047	625.417	161.011
43749	6.0472	777.793	197.207
43750	6.0475	896	25.1412
43751	6.0475	766.352	13.905
43752	6.0476	618.712	124.972
43753	6.0476	870.372	44.3257
43754	6.0477	766.021	81.8094
43755	6.0478	852.197	98.3035
43756	6.0481	648.629	77.6308
43757	6.0486	742.734	59.1061
43758	6.0486	811.406	173.122
43759	6.0487	667.536	180.647
43760	6.0488	794.912	48.3408
43761	6.0489	624.643	169.943
43762	6.049	820.634	56.1278
43763	6.0494	828.397	57.9436
43764	6.0495	831.051	95.6108
43765	6.0497	749.972	228.176

43766	6.0498	706.756	176.379
43767	6.0501	921.032	78.928
43768	6.0502	724.493	226.43
43769	6.0503	696.522	117.894
43770	6.0503	823.952	30.2537
43771	6.0507	620.628	135.661
43772	6.0508	785.86	65.5282
43773	6.0508	814.018	45.003
43774	6.051	985.73	260.005
43775	6.051	748.356	264.862
43776	6.0511	695.109	53.9234
43777	6.0511	820.943	53.7559
43778	6.0514	731.798	230.829
43779	6.0516	802.115	187.068
43780	6.052	822.768	79.6605
43781	6.0522	587.881	223.152
43782	6.0524	688.563	174.46
43783	6.0527	862.97	52.1369
43784	6.0529	622.34	75.381
43785	6.053	910.469	10.813
43786	6.0538	676.866	299.215
43787	6.0538	694.22	162.667
43788	6.0541	633.332	111.299
43789	6.0542	725.087	224.252
43790	6.0542	809.737	88.2565
43791	6.0543	801.852	42.7627
43792	6.0543	778	171.939
43793	6.0546	760.927	156.791
43794	6.0549	836.963	119.223
43795	6.055	837.37	255.093
43796	6.0551	757.433	142.68
43797	6.0551	750.284	79.8835
43798	6.0552	561.345	357.444
43799	6.0552	786.108	104.237
43800	6.0552	849.764	31.5266
43801	6.0554	264.089	198.108
43802	6.0554	648.058	116.46
43803	6.0555	721.884	162.78
43804	6.0557	-2.856	80.6018
43805	6.056	780.739	138.191
43806	6.0561	-2.8117	78.0937
43807	6.0566	812.673	67.5082
43808	6.0567	630.856	170.987
43809	6.0567	649.717	179.69
43810	6.0568	688.896	191.101
43811	6.0569	713.872	109.303
43812	6.0571	821.814	159.776
43813	6.0571	908.077	47.9901

43814	6.0571	644.789	158.891
43815	6.0572	816.005	49.5001
43816	6.0572	753.383	116.368
43817	6.0572	678.529	147.012
43818	6.0573	859.398	29.1898
43819	6.0573	749.886	72.2686
43820	6.0581	858.498	112.688
43821	6.0583	767.693	87.5574
43822	6.0588	802.956	97.421
43823	6.0589	885.691	51.4942
43824	6.0593	758.3	283.028
43825	6.0595	790.054	49.385
43826	6.0601	795.933	142.946
43827	6.0612	899.952	54.6275
43828	6.0616	677.842	213.793
43829	6.0616	715.279	176.153
43830	6.0616	700.663	142.715
43831	6.0616	715.202	120.493
43832	6.0619	773.161	183.324
43833	6.0622	843.07	50.67
43834	6.0623	671.914	186.298
43835	6.0627	800.189	211.407
43836	6.0628	945.295	61.7025
43837	6.0629	645.229	175.276
43838	6.063	707.414	220.555
43839	6.063	669.767	141.038
43840	6.0632	806.996	63.607
43841	6.0634	900.616	16.208
43842	6.0634	909.534	12.4257
43843	6.0636	646.894	190.983
43844	6.0643	-2.9114	262.404
43845	6.0644	709.104	297.854
43846	6.0644	783.717	46.3771
43847	6.0645	734.767	51.5833
43848	6.0647	884.077	16.5161
43849	6.0648	754.108	305.071
43850	6.0649	727.311	145.751
43851	6.0649	865.543	271.188
43852	6.065	875.914	34.1233
43853	6.0655	744.284	139.376
43854	6.0656	727.941	215.717
43855	6.0657	732.729	173.526
43856	6.0658	675.339	127.547
43857	6.0658	825.422	151.414
43858	6.066	661.184	287.399
43859	6.0661	864.469	88.1045
43860	6.0661	761.517	53.4973
43861	6.0662	744.084	331.19

43862	6.0663	651.112	253.762
43863	6.0667	839.904	44.1134
43864	6.067	727.563	136.634
43865	6.0674	913.289	36.1111
43866	6.0675	827.416	82.7555
43867	6.0675	953.722	82.353
43868	6.0676	863.324	301.87
43869	6.068	699.278	225.817
43870	6.0689	742.475	221.659
43871	6.0689	838.662	35.3401
43872	6.069	742.637	351.195
43873	6.0696	858.35	38.4862
43874	6.0696	645.149	223.519
43875	6.0697	842.897	55.7937
43876	6.0698	785.745	48.8119
43877	6.07	888.771	33.6474
43878	6.0701	670.331	157.12
43879	6.0701	673.814	147.649
43880	6.0707	728.494	103.49
43881	6.0708	774.248	102.72
43882	6.0711	714.776	26.2253
43883	6.0718	677.377	240.181
43884	6.0718	729.136	256.889
43885	6.0719	683.042	140.082
43886	6.0719	809.493	301.316
43887	6.072	676.849	258.596
43888	6.0724	825.658	14.935
43889	6.0725	844.381	26.3582
43890	6.0725	748.267	331.447
43891	6.0725	648.159	17.0792
43892	6.0727	772.224	115.617
43893	6.0728	655.537	189.922
43894	6.0729	742.625	61.6429
43895	6.0729	999.958	241.37
43896	6.073	739.897	112.217
43897	6.0731	667.744	162.688
43898	6.0732	703.432	101.36
43899	6.0736	849.507	130.683
43900	6.0738	705.18	72.8431
43901	6.074	766.869	187.469
43902	6.0742	657.339	281.991
43903	6.0746	629.913	185.483
43904	6.0746	854.634	44.2749
43905	6.0751	703.873	17.1533
43906	6.0751	756.477	39.3418
43907	6.0751	753.477	133.015
43908	6.0752	733.936	55.9175
43909	6.0753	753.355	330.087

43910	6.0756	757.32	317.778
43911	6.0756	689.435	98.6862
43912	6.0761	654.244	172.866
43913	6.0766	1009.1	35.6125
43914	6.0768	763.809	63.3203
43915	6.0768	670.926	129.009
43916	6.0769	837.805	162.051
43917	6.0771	793.278	53.8148
43918	6.0772	926.867	43.5825
43919	6.0775	817.437	108.854
43920	6.0776	816.782	50.8972
43921	6.0777	725.208	178.99
43922	6.078	780.66	60.6109
43923	6.0781	774.538	198.77
43924	6.0782	818.387	72.6671
43925	6.0785	764.753	232.068
43926	6.0789	818.144	41.3963
43927	6.079	627.224	221.724
43928	6.0791	840.228	243.366
43929	6.0791	752.234	112.997
43930	6.0793	765.554	63.9402
43931	6.0795	890.959	138.644
43932	6.0795	767.214	161.055
43933	6.0796	759.731	126.16
43934	6.0798	679.564	190.174
43935	6.0798	731.699	195.723
43936	6.08	850.973	138.528
43937	6.08	641.633	222.69
43938	6.0805	736.113	31.1529
43939	6.0806	809.37	242.752
43940	6.0806	938.257	9.8095
43941	6.0809	644.719	185.616
43942	6.081	955.779	47.1489
43943	6.0811	777.733	236.565
43944	6.0812	775.795	149.573
43945	6.0814	808.579	40.3841
43946	6.0815	667.977	279.615
43947	6.082	996.445	40.056
43948	6.0824	778.108	160.483
43949	6.0826	893.842	89.0031
43950	6.0827	803.258	98.2864
43951	6.0828	872.057	326.345
43952	6.083	747.049	233.614
43953	6.0831	739.055	349.859
43954	6.0831	670.677	228.063
43955	6.0831	820.866	38.7821
43956	6.0834	781.678	36.7712
43957	6.0836	662.241	106.381

43958	6.0839	835.397	168.1
43959	6.0841	688.108	173.367
43960	6.0841	790.572	99.749
43961	6.0841	818.396	46.2471
43962	6.0843	-2.8114	78.747
43963	6.0846	949.437	258.816
43964	6.0846	776.405	35.9887
43965	6.0848	812.031	63.0106
43966	6.0849	736.352	275.541
43967	6.0851	871.818	77.661
43968	6.0852	681.806	269.086
43969	6.0856	627.26	189.332
43970	6.0858	883.525	254.033
43971	6.086	827.696	224.701
43972	6.0862	750.057	171.442
43973	6.0862	908.194	25.4327
43974	6.0864	814.483	110.474
43975	6.0864	758.208	292.698
43976	6.0865	589.189	143.566
43977	6.0868	710.612	156.744
43978	6.0869	869.201	6.8493
43979	6.0872	701.158	136.693
43980	6.0873	668.953	93.9003
43981	6.0874	742.182	28.8003
43982	6.0876	688.238	120.124
43983	6.0881	718.007	18.6445
43984	6.0881	736.925	147.479
43985	6.0881	812.403	28.241
43986	6.0882	581.127	79.3959
43987	6.0883	728.325	84.0188
43988	6.0884	551.257	139.152
43989	6.0884	560.231	151.73
43990	6.0887	669.618	223.728
43991	6.089	830.056	24.3367
43992	6.0896	770.087	101.828
43993	6.0899	761.773	198.546
43994	6.0904	792.296	79.0296
43995	6.0904	707.258	233.744
43996	6.0905	960.27	43.7896
43997	6.091	771.119	277.261
43998	6.0911	780.209	241.824
43999	6.0916	826.389	48.7578
44000	6.0916	728.032	262.826
44001	6.0916	873.039	81.5696
44002	6.0923	660.614	239.859
44003	6.0924	725.381	273.581
44004	6.0924	739.5	94.8671
44005	6.0928	683.568	231.284

44006	6.0931	563.761	307.494
44007	6.0932	800.29	231.107
44008	6.0934	879.956	149.089
44009	6.0935	840.83	4.4822
44010	6.0935	665.259	228.037
44011	6.0939	860.488	120.253
44012	6.094	314.28	195.407
44013	6.094	641.45	330.239
44014	6.0941	797.066	25.7846
44015	6.0942	810.823	172.127
44016	6.0943	648.677	177.61
44017	6.0945	737.186	224.119
44018	6.0947	814.758	12.2702
44019	6.0952	843.79	254.187
44020	6.0955	806.327	145.46
44021	6.0957	723.239	221.711
44022	6.096	779.742	126.098
44023	6.0962	964.369	99.3157
44024	6.0964	788.497	22.4215
44025	6.0967	724.096	264.816
44026	6.0968	684.106	128.374
44027	6.0973	648.774	144.034
44028	6.0974	797.02	113.52
44029	6.0974	703.004	142.271
44030	6.0975	714.576	54.8932
44031	6.0976	734.982	227.149
44032	6.0977	622.272	345.768
44033	6.0987	798.311	153.389
44034	6.0988	783.92	14.1847
44035	6.0989	714.838	61.5809
44036	6.0993	597.184	235.411
44037	6.0995	821.809	81.7675
44038	6.0996	752.292	156.549
44039	6.0996	812.167	231.165
44040	6.0998	812.325	116.139
44041	6.0998	758.678	127.232
44042	6.1001	872.602	27.9965
44043	6.1002	929.526	313.929
44044	6.1002	895.261	58.7422
44045	6.1004	588.185	198.138
44046	6.1004	800.934	64.9347
44047	6.1005	792.581	124.754
44048	6.1006	740.838	158.308
44049	6.101	819.143	98.5522
44050	6.101	818.312	136.42
44051	6.1011	702.197	340.219
44052	6.1011	787.524	64.5633
44053	6.1011	684.303	138.164

44054	6.1015	681.848	188.158
44055	6.1016	763.092	14.4038
44056	6.1017	778.405	163.846
44057	6.1017	711.08	100.673
44058	6.1021	658.57	145.712
44059	6.1023	684.547	286.269
44060	6.1024	671.351	169.992
44061	6.1024	859.809	17.0232
44062	6.1031	923.04	57.6138
44063	6.1032	913.629	13.6051
44064	6.1033	709.376	268.918
44065	6.1033	698.473	165.408
44066	6.1035	641.21	172.365
44067	6.1035	718.357	161.806
44068	6.1038	602.026	10.4619
44069	6.1039	748.239	171.197
44070	6.104	791.033	90.4049
44071	6.1041	762.526	329.576
44072	6.1041	773.905	143.287
44073	6.1041	764.973	254.1
44074	6.1042	650.102	231.292
44075	6.1042	812.494	75.3829
44076	6.1043	651.3	78.9867
44077	6.1043	903.655	58.5006
44078	6.1044	687.069	145.591
44079	6.1045	598.089	294.691
44080	6.1048	750.875	235.983
44081	6.105	687.147	158.728
44082	6.1051	729.188	136.098
44083	6.1052	684.888	129.298
44084	6.1059	799.241	231.988
44085	6.1062	672.994	237.758
44086	6.1063	860.439	28.6691
44087	6.1065	912.659	332.139
44088	6.1066	768.753	246.296
44089	6.1068	745.894	195.929
44090	6.1078	872.98	50.0882
44091	6.1078	713.496	250.663
44092	6.108	734.211	231.216
44093	6.1084	796.203	71.2206
44094	6.1084	915.525	202.038
44095	6.1086	675.351	220.53
44096	6.1087	758.047	7.1431
44097	6.1087	914.077	20.5378
44098	6.1092	860.038	97.3175
44099	6.1095	721.345	221.039
44100	6.1097	712.318	224.031
44101	6.1097	766.274	204.627

44102	6.1098	821.619	293.368
44103	6.1098	790.092	89.3842
44104	6.1098	852.155	62.9406
44105	6.1102	720.002	168.379
44106	6.1103	691.274	183.336
44107	6.1104	774.875	118.643
44108	6.1111	696.479	213.601
44109	6.1115	667.412	113.454
44110	6.1116	629.105	183.028
44111	6.1117	757.334	113.184
44112	6.1117	819.881	50.8291
44113	6.1119	685.083	186.475
44114	6.112	719.158	176.51
44115	6.112	657.678	179.476
44116	6.1122	890.395	28.2149
44117	6.1122	841.041	84.6261
44118	6.1125	782.736	17.5386
44119	6.1125	651.474	113.161
44120	6.1126	731.91	273.201
44121	6.1129	842.145	63.97
44122	6.1133	706.789	137.157
44123	6.1134	646.776	114.452
44124	6.1134	905.358	191.367
44125	6.1138	838.698	27.3405
44126	6.1139	732.074	173.81
44127	6.1139	633.575	196.381
44128	6.114	687.883	21.8544
44129	6.1142	928.661	55.5822
44130	6.1143	753.099	125.32
44131	6.1144	788.491	62.2953
44132	6.1146	889.473	1.5532
44133	6.1147	682.28	176.469
44134	6.1147	679.826	77.2918
44135	6.1148	807.077	181.136
44136	6.1149	725.189	281.551
44137	6.115	755.005	130.686
44138	6.115	752.239	154.964
44139	6.115	640.427	173.96
44140	6.1153	734.238	157.651
44141	6.1156	767.754	90.6167
44142	6.1156	844.911	53.3824
44143	6.1162	766.369	78.3175
44144	6.1163	985.42	30.6491
44145	6.1165	707.712	157.809
44146	6.1165	964.791	36.6612
44147	6.1166	940.067	54.0139
44148	6.1167	449.456	330.677
44149	6.1169	717.58	256.204

44150	6.117	798.671	121.451
44151	6.117	914.507	79.2708
44152	6.1172	921.578	143.964
44153	6.1172	764.254	189.267
44154	6.1176	699.491	125.837
44155	6.1179	854.889	61.3364
44156	6.1186	879.589	209.363
44157	6.1186	836.154	137.612
44158	6.1188	843.011	70.2145
44159	6.1188	913.869	19.1751
44160	6.1194	781.555	64.4621
44161	6.1196	874.948	93.4333
44162	6.1203	734.577	164.967
44163	6.1203	801.966	214.061
44164	6.1204	743.166	112.183
44165	6.1205	956.969	85.9012
44166	6.1205	778.262	266.2
44167	6.1206	903.339	40.3499
44168	6.1206	774.638	75.8788
44169	6.1207	779.916	187.653
44170	6.1207	736.877	224.843
44171	6.1208	918.327	17.6697
44172	6.1208	797.972	297.835
44173	6.1209	822.04	340.135
44174	6.121	748.93	224.385
44175	6.121	748.626	228.972
44176	6.121	878.422	53.6047
44177	6.121	742.006	168.211
44178	6.1211	768.857	80.7262
44179	6.1215	782.787	24.27
44180	6.1217	744.257	228.052
44181	6.1218	865.118	170.482
44182	6.1221	804.042	81.7323
44183	6.1223	778.411	329.816
44184	6.1223	798.463	34.1841
44185	6.1227	650.86	42.3884
44186	6.1228	701.856	172.425
44187	6.1231	922.834	17.673
44188	6.1234	810.132	225.128
44189	6.1235	696.441	61.4151
44190	6.1236	756.403	175.535
44191	6.1237	746.911	161.833
44192	6.1238	885.789	257.45
44193	6.1238	721.326	155.386
44194	6.1241	687.474	158.779
44195	6.1242	784.457	247.304
44196	6.1244	832.975	47.8585
44197	6.1246	861.111	104.623

44198	6.1248	704.81	171.873
44199	6.1248	795.638	161.797
44200	6.1251	823.413	277.435
44201	6.1252	690.79	284.019
44202	6.1252	794.855	12.1498
44203	6.1252	861.413	15.8116
44204	6.1253	725.747	294.78
44205	6.1253	673.518	119.219
44206	6.1262	714.226	192.97
44207	6.1262	902.233	86.7205
44208	6.1262	715.949	82.2667
44209	6.1263	850.336	38.9348
44210	6.1265	743.211	84.2746
44211	6.1267	884.1	103.894
44212	6.1267	763.75	121.301
44213	6.1268	662.594	319.362
44214	6.1269	741.652	283.695
44215	6.127	821.919	156.853
44216	6.1272	752.143	283.277
44217	6.1273	721.278	164.746
44218	6.1276	755.183	248.061
44219	6.1282	805.093	73.5475
44220	6.1283	896.436	47.2421
44221	6.1284	699.184	143.437
44222	6.1285	778.402	31.3506
44223	6.1285	846.645	38.0558
44224	6.1289	772.144	295.96
44225	6.129	814.793	48.9216
44226	6.1291	745.576	134.365
44227	6.1292	775.958	146.734
44228	6.1292	649.857	200.748
44229	6.1295	746.996	250.629
44230	6.1295	1057.61	241.431
44231	6.1296	680.958	283.721
44232	6.1297	941.165	18.3838
44233	6.1297	920.938	189.896
44234	6.1304	753.273	216.06
44235	6.1305	782.05	82.0467
44236	6.1305	764.859	226.66
44237	6.1308	708.477	223.339
44238	6.1311	732.252	317.336
44239	6.1317	951.977	11.5081
44240	6.1318	713.047	245.656
44241	6.1321	559.948	182.622
44242	6.1321	710.322	186.324
44243	6.1321	758.326	288.455
44244	6.1324	805.68	246.347
44245	6.1324	1000.81	240.678

44246	6.1324	872.124	83.0235
44247	6.1326	725.069	122.5
44248	6.1326	802.64	302.943
44249	6.1327	-4.1175	257.956
44250	6.1332	930.487	22.3918
44251	6.1333	-4.7791	58.3482
44252	6.1333	647.631	143.048
44253	6.1335	675.373	136.429
44254	6.1337	637.498	175.124
44255	6.1339	701.166	107.783
44256	6.1339	783.826	109.086
44257	6.1341	709.528	185.92
44258	6.1345	807.197	166.04
44259	6.1345	752.211	159.791
44260	6.1345	798.052	114.242
44261	6.135	740.295	113.563
44262	6.1352	768.772	251.426
44263	6.1353	799.251	142.176
44264	6.1354	707.192	111.915
44265	6.1358	925.506	26.2383
44266	6.1361	793.69	174.768
44267	6.1362	832.007	101.911
44268	6.1363	852.879	30.2018
44269	6.1363	943.165	28.6791
44270	6.1366	854.265	81.053
44271	6.1367	850.385	86.8751
44272	6.1367	815.195	107.655
44273	6.1369	730.541	292.038
44274	6.137	769.209	112.591
44275	6.1371	923.498	255.884
44276	6.1372	766.274	304.305
44277	6.1372	686.103	122.92
44278	6.1374	710.529	358.816
44279	6.1374	742.03	76.4199
44280	6.1375	703.138	243.586
44281	6.1375	878.131	138.707
44282	6.1377	824.196	69.1744
44283	6.1377	903.821	260.639
44284	6.1377	882.944	322.799
44285	6.1379	770.029	229.923
44286	6.138	715.016	233.301
44287	6.1382	587.881	59.4123
44288	6.1382	866.85	38.9225
44289	6.1383	733.389	303.062
44290	6.139	819.545	29.6265
44291	6.1392	866.953	182.224
44292	6.1394	796.566	204.978
44293	6.1394	812.529	127.568

44294	6.1398	816.71	236.242
44295	6.1399	745.558	156.396
44296	6.1402	823.644	298.784
44297	6.1405	689.799	146.848
44298	6.1406	781.699	158.185
44299	6.1407	836.87	244.878
44300	6.1407	842.349	108.152
44301	6.1409	694.506	132.715
44302	6.1411	784.404	153.071
44303	6.1411	913.069	93.979
44304	6.1415	692.005	23.7909
44305	6.1415	704.771	229.579
44306	6.1415	828.54	76.0234
44307	6.1416	750.051	154.234
44308	6.1418	757.911	252.853
44309	6.1418	871.052	105.943
44310	6.142	699.36	230.525
44311	6.1422	782.828	241.734
44312	6.1422	787.819	223.626
44313	6.1422	748.602	150.086
44314	6.1425	797.474	130.049
44315	6.1427	792.635	185.583
44316	6.1427	707.788	147.774
44317	6.1431	874.418	51.9696
44318	6.1431	760.859	356.55
44319	6.1432	816.888	24.7748
44320	6.1433	831.428	271.197
44321	6.1433	733.478	168.833
44322	6.1433	909.777	24.7048
44323	6.1435	830.196	188.718
44324	6.1435	846.182	103.255
44325	6.1437	756.236	156.261
44326	6.1437	630.557	226.353
44327	6.1438	694.367	17.4745
44328	6.1438	848.651	61.9909
44329	6.1442	910.02	348.83
44330	6.1442	657.345	193.459
44331	6.1444	718.888	37.826
44332	6.1445	855.681	93.3161
44333	6.1445	760.811	105.689
44334	6.145	886.478	94.9981
44335	6.1452	784.241	223.59
44336	6.1452	852.622	43.1407
44337	6.1461	749.632	178.532
44338	6.1462	882.504	26.2821
44339	6.1462	834.915	240.408
44340	6.1462	756.452	223.761
44341	6.1463	761.048	169.363

44342	6.1465	644.837	117.294
44343	6.147	809.389	234.296
44344	6.1472	717.962	28.0766
44345	6.1475	774.745	186.397
44346	6.1475	764.488	152.702
44347	6.1477	720.383	192.515
44348	6.1482	699.68	76.1392
44349	6.1483	790.191	234.914
44350	6.1485	666.188	145.693
44351	6.1485	937.58	26.738
44352	6.1487	789.141	266.274
44353	6.1488	718.98	60.1462
44354	6.1493	791.889	153.782
44355	6.1494	754.759	177.704
44356	6.1497	866.736	234.146
44357	6.1501	781.217	173.418
44358	6.1501	719.927	5.4665
44359	6.1503	866.165	261.411
44360	6.1511	788.908	77.036
44361	6.1513	271.789	25.6357
44362	6.1513	692.403	159.936
44363	6.1513	795.953	133.524
44364	6.1515	745.074	199.666
44365	6.1516	779.19	305.245
44366	6.1517	892.463	48.6825
44367	6.152	46.2255	320.63
44368	6.1521	896.119	325.229
44369	6.1522	888.371	58.8758
44370	6.1527	713.825	160.596
44371	6.1528	685.788	236.295
44372	6.153	806.73	3.3295
44373	6.1533	751.935	196.02
44374	6.1533	975.632	39.0748
44375	6.1534	747.428	141.171
44376	6.1535	813.282	183.875
44377	6.1536	743.838	145.55
44378	6.1538	699.446	178.201
44379	6.1541	763.535	251.104
44380	6.1542	789.183	175.973
44381	6.1543	827.331	358.663
44382	6.1543	701.44	253.124
44383	6.1546	764.872	178.796
44384	6.1546	761.229	158.968
44385	6.1547	885.574	94.6691
44386	6.1549	841.754	47.6474
44387	6.1549	796.046	123.396
44388	6.1554	827.775	359.496
44389	6.1555	882.808	18.1116

44390	6.1557	726.246	169.984
44391	6.1559	783.9	70.3835
44392	6.1561	771.382	302.097
44393	6.1561	814.632	86.7673
44394	6.1561	703.621	165.94
44395	6.1561	739.886	147.84
44396	6.1563	743.791	146.187
44397	6.1565	859.63	15.8265
44398	6.1567	792.895	44.8696
44399	6.1568	656.108	221.57
44400	6.1569	583.614	326.856
44401	6.1572	732.464	285.269
44402	6.1574	753.628	223.999
44403	6.1575	786.064	166.353
44404	6.1578	939.459	10.8865
44405	6.1578	808.147	56.237
44406	6.1586	733.224	159.92
44407	6.1586	950.262	48.9377
44408	6.1589	678.905	137.416
44409	6.1592	797.023	359.621
44410	6.1595	756.688	158.638
44411	6.1596	699.857	195.436
44412	6.1596	826.832	226.803
44413	6.1596	890.819	19.6669
44414	6.1598	743.287	246.802
44415	6.1598	821.898	138.036
44416	6.1599	929.743	53.1157
44417	6.1602	790.123	177.648
44418	6.1606	804.883	281.41
44419	6.1608	839.484	171.827
44420	6.1614	791.678	303.658
44421	6.1619	932.161	80.704
44422	6.1622	578.476	180.861
44423	6.1623	779.691	170.756
44424	6.1624	796.545	319.841
44425	6.1624	791.796	134.364
44426	6.1626	813.515	223.724
44427	6.1631	827.13	130.679
44428	6.1631	731.667	152.151
44429	6.1633	891.06	272.5
44430	6.1633	636.47	143.285
44431	6.1641	823.246	271.727
44432	6.1642	861.682	55.0431
44433	6.1643	924.629	35.3356
44434	6.1645	789.964	117.562
44435	6.1645	825.39	109.413
44436	6.1651	685.255	183.623
44437	6.1655	741.548	345.934

44438	6.1656	782.056	348.629
44439	6.1658	746.419	352.654
44440	6.1659	874.044	51.1149
44441	6.166	826.224	48.6383
44442	6.1668	826.653	297.996
44443	6.1669	645.57	144.674
44444	6.1671	314.11	194.002
44445	6.1674	840.515	198.387
44446	6.1676	862.375	198.248
44447	6.1678	710.488	290.989
44448	6.1679	789.173	146.265
44449	6.168	710.665	186.191
44450	6.1682	692.43	221.496
44451	6.1683	790.556	293.237
44452	6.1683	819.545	212.898
44453	6.1685	719.666	252.544
44454	6.1692	785.426	83.2505
44455	6.1693	894.464	68.9196
44456	6.1695	830.586	261.402
44457	6.1695	984.506	47.7372
44458	6.1701	863.729	206.847
44459	6.1702	842.982	134.011
44460	6.1702	790.346	154.768
44461	6.1704	908.918	257.071
44462	6.1707	932.441	46.6294
44463	6.1707	797.8	18.5961
44464	6.171	890.786	36.0508
44465	6.171	935.718	86.8909
44466	6.171	879.214	47.6334
44467	6.1711	734.895	216.133
44468	6.1712	894.783	23.0979
44469	6.1717	841.433	188.288
44470	6.1718	808.783	211.49
44471	6.1719	807.095	41.8739
44472	6.1719	889.545	136.582
44473	6.172	768.847	267.56
44474	6.1721	857.176	47.315
44475	6.1722	712.457	247.36
44476	6.1724	861.053	48.5061
44477	6.1724	929.133	243.482
44478	6.1725	914.755	93.6004
44479	6.1729	731.939	112.279
44480	6.1731	918.904	11.3537
44481	6.1734	759.191	162.319
44482	6.1736	806.032	352.75
44483	6.1739	781.479	73.1817
44484	6.1739	686.038	159.888
44485	6.174	738.893	202.369

44486	6.1742	829.73	358.014
44487	6.1742	827.721	235.312
44488	6.1743	665.091	133.016
44489	6.1744	1021.62	287.83
44490	6.1744	839.129	86.5948
44491	6.1745	687.353	253.449
44492	6.1747	794.767	143.721
44493	6.1747	959.735	6.2027
44494	6.175	856.86	345.747
44495	6.1755	751.116	284.769
44496	6.1757	716.773	152.758
44497	6.1757	964.576	25.8621
44498	6.1758	802.541	79.7282
44499	6.1761	773.018	202.422
44500	6.1761	674.756	160.111
44501	6.1763	747.022	148.732
44502	6.1763	983.712	39.5788
44503	6.1764	782.584	182.255
44504	6.1769	821.399	51.725
44505	6.177	868.851	146.891
44506	6.177	907.172	56.059
44507	6.1772	853.529	99.3018
44508	6.1774	744.85	112.356
44509	6.1778	738.337	36.8944
44510	6.1781	890.08	96.9808
44511	6.1785	787.16	197.485
44512	6.1786	696.547	130.909
44513	6.1788	805.489	10.3778
44514	6.179	826.815	88.8941
44515	6.1791	827.462	141.945
44516	6.1795	831.129	86.4109
44517	6.1796	694.841	104.798
44518	6.1799	739.873	173.963
44519	6.18	607.457	178.537
44520	6.1803	751.406	161.555
44521	6.1804	-2.7901	76.7586
44522	6.1804	891.436	151.736
44523	6.1805	800.484	216.035
44524	6.1806	781.091	115.767
44525	6.1807	707.679	154.486
44526	6.1809	961.967	306.225
44527	6.1811	659.037	218.636
44528	6.1811	698.609	251.63
44529	6.1812	766.209	56.951
44530	6.1813	833.57	192.416
44531	6.1814	887.18	69.0832
44532	6.1814	853.927	138.641
44533	6.1814	665.036	188.466

44534	6.1815	264.209	226.472
44535	6.1817	824.868	79.9335
44536	6.1818	683.994	182.33
44537	6.182	763.164	161.664
44538	6.1822	877.303	33.7228
44539	6.1822	797.806	175.454
44540	6.1825	738.538	190.699
44541	6.1825	758.049	125.301
44542	6.1831	-2.8363	269.604
44543	6.1832	787.864	161.649
44544	6.1832	805.704	79.6432
44545	6.1834	681.404	196.205
44546	6.1834	730.599	181.647
44547	6.1835	897.26	109.571
44548	6.1838	810.601	145.224
44549	6.1843	739.332	97.8984
44550	6.1843	752.786	152.773
44551	6.1844	733.529	140.578
44552	6.1845	757.657	185.214
44553	6.1848	-2.781	77.416
44554	6.1849	977.77	44.5265
44555	6.1851	667.168	241.198
44556	6.1851	847.521	33.0108
44557	6.1857	911.371	163.678
44558	6.1859	930.781	56.5351
44559	6.1864	802.526	153.165
44560	6.1864	732.38	226.881
44561	6.1866	737.944	215.048
44562	6.1867	944.145	36.6551
44563	6.1868	704.061	155.024
44564	6.187	875.326	53.2366
44565	6.1873	692.792	67.4874
44566	6.1874	664.09	126.838
44567	6.1875	738.122	175.286
44568	6.1876	789.754	257.124
44569	6.1876	822.242	144.162
44570	6.1877	682.521	183.296
44571	6.188	702.865	260.288
44572	6.1882	853.222	24.3503
44573	6.1883	698.531	246.845
44574	6.1883	985.416	23.9191
44575	6.1883	982.567	8.9699
44576	6.1886	844.606	336.653
44577	6.1889	774.57	31.4369
44578	6.1889	903.255	100.393
44579	6.1892	769.402	177.077
44580	6.1892	892.48	43.4294
44581	6.1893	941.929	24.1424

44582	6.1894	-4.1332	272.443
44583	6.1898	720.324	233.293
44584	6.1902	804.882	208.426
44585	6.1904	819.07	163.99
44586	6.1907	693.06	194.422
44587	6.1907	814.205	51.7372
44588	6.1909	776.5	59.2758
44589	6.1909	1015.4	45.9881
44590	6.191	803.783	90.7491
44591	6.1911	729.292	328.172
44592	6.1914	934.039	26.5115
44593	6.1915	880.497	116.869
44594	6.1916	818.545	84.8319
44595	6.1919	713.847	121.653
44596	6.1922	819.651	140.565
44597	6.1923	796.816	74.9874
44598	6.1927	949.814	29.3971
44599	6.1927	758.224	73.0956
44600	6.1929	832.668	320.663
44601	6.1929	845.934	63.3774
44602	6.193	907.076	356.894
44603	6.1932	857.352	108.874
44604	6.1935	798.812	231.975
44605	6.1937	734.656	121.899
44606	6.1943	604.1	181.129
44607	6.1949	834.556	17.0632
44608	6.1954	720.024	286.381
44609	6.1956	890.645	103.918
44610	6.1959	841.359	80.4957
44611	6.1961	964.728	72.0293
44612	6.1964	718.285	156.015
44613	6.1964	735.037	87.6269
44614	6.1965	877.615	78.1987
44615	6.1965	655.37	128.74
44616	6.1967	-3.5961	154.862
44617	6.1971	835.896	138.026
44618	6.1973	687.173	231.991
44619	6.1973	830.495	29.368
44620	6.1976	736.803	225.785
44621	6.1983	901.552	108.779
44622	6.1985	817.822	173.786
44623	6.1986	869.173	51.7269
44624	6.1986	798.872	142.504
44625	6.1989	771.323	106.208
44626	6.1989	753.486	173.828
44627	6.199	868.997	98.1527
44628	6.1994	721.56	166.121
44629	6.1994	771.965	252.221

44630	6.1996	775.395	45.7353
44631	6.1997	861.619	49.6483
44632	6.2	938.098	46.0024
44633	6.2005	882.913	44.2692
44634	6.2006	812.445	193.154
44635	6.2006	847.186	109.609
44636	6.2007	555.112	325.78
44637	6.2008	783.448	169.414
44638	6.2009	884.039	54.0121
44639	6.2012	732.591	81.7479
44640	6.2013	953.022	2.4777
44641	6.2015	790.401	167.199
44642	6.2017	906.035	101.924
44643	6.2023	813.674	340.382
44644	6.2024	715.401	250.814
44645	6.2026	819.802	55.5085
44646	6.2027	264.082	201.57
44647	6.2027	928.727	91.3058
44648	6.2028	919.055	284.11
44649	6.2031	723.317	160.344
44650	6.2034	717.585	176.457
44651	6.2034	795.194	108.339
44652	6.2038	890.333	62.1
44653	6.204	856.573	352.075
44654	6.204	910.676	30.8271
44655	6.204	1020.13	37.5132
44656	6.2043	147.304	310.796
44657	6.2045	800.625	50.4348
44658	6.2049	926.037	144.843
44659	6.2054	750.178	222.613
44660	6.2056	891.007	52.7092
44661	6.2057	682.646	10.0265
44662	6.2067	869.822	99.4876
44663	6.2068	774.806	232.313
44664	6.2072	810.21	298.449
44665	6.2073	701.366	176.906
44666	6.2073	803.934	115.487
44667	6.2073	865.427	82.1108
44668	6.2074	699.525	18.4186
44669	6.2076	806.063	248.775
44670	6.2078	788.054	293.8
44671	6.2079	1075.1	81.9679
44672	6.208	818.723	142.514
44673	6.208	806.612	180.006
44674	6.2082	798.645	141.751
44675	6.2087	745.558	179.715
44676	6.2087	807.177	147.012
44677	6.2087	992.161	54.7162

44678	6.2091	788.694	49.0156
44679	6.2091	956.867	18.0316
44680	6.2097	935.777	26.9243
44681	6.2099	789.9	48.9439
44682	6.21	926.241	43.4983
44683	6.2103	947.624	33.2003
44684	6.2103	962.829	25.1266
44685	6.2106	853.624	171.057
44686	6.2106	720.726	179.369
44687	6.2107	789.965	17.0396
44688	6.2108	863.861	359.728
44689	6.2108	702.491	153.565
44690	6.2112	832.621	73.1466
44691	6.2112	923.481	120.412
44692	6.2117	959.855	82.0527
44693	6.2118	893.736	80.8563
44694	6.2118	679.908	176.566
44695	6.2118	933.467	63.663
44696	6.2119	-2.815	273.492
44697	6.212	809.364	69.1919
44698	6.2123	727.706	335.096
44699	6.2125	995.581	258.532
44700	6.2126	743.209	188.535
44701	6.2128	924.4	98.82
44702	6.2139	848.449	84.9159
44703	6.2139	736.013	163.105
44704	6.214	854.253	352.678
44705	6.2142	795.08	188.117
44706	6.2144	695.34	109.063
44707	6.2146	821.817	62.9746
44708	6.2151	866.675	276.432
44709	6.2162	803.169	24.8882
44710	6.2164	879.222	359.645
44711	6.2167	796.392	266.876
44712	6.2167	677.027	336.627
44713	6.2168	932.58	26.6523
44714	6.2169	992.69	22.7023
44715	6.2171	706.861	107.673
44716	6.2171	762.134	195.412
44717	6.2172	728.677	278.171
44718	6.2172	856.143	253.482
44719	6.2173	648.621	189.607
44720	6.2174	773.772	284.292
44721	6.2175	809.936	117.822
44722	6.2179	923.647	44.5044
44723	6.218	875.275	317.776
44724	6.2181	807.249	134.407
44725	6.2182	785.671	71.6093

44726	6.2184	824.786	18.3207
44727	6.2186	943.669	86.685
44728	6.219	768.458	118.8
44729	6.219	712.418	128.146
44730	6.2194	859.054	92.941
44731	6.2197	854.817	290.36
44732	6.22	945.124	26.321
44733	6.22	805.861	19.3059
44734	6.2201	788.073	119.481
44735	6.2202	710.153	149.29
44736	6.2203	973.235	11.2192
44737	6.2205	642.96	181.083
44738	6.2211	833.17	164.049
44739	6.2211	769.495	74.1671
44740	6.2215	771.805	103.592
44741	6.2219	731.938	150.65
44742	6.2224	873.5	184.974
44743	6.2224	797.51	172.441
44744	6.2226	881.477	129.981
44745	6.2226	890.902	14.2001
44746	6.2227	802.934	86.1502
44747	6.2228	957.159	24.6305
44748	6.2228	757.276	60.3621
44749	6.2228	1000.48	12.5232
44750	6.2229	730.935	90.7403
44751	6.2229	758.779	174.779
44752	6.2229	805.844	158.827
44753	6.223	875.753	15.7222
44754	6.223	1017.17	47.3257
44755	6.2232	885.28	94.3182
44756	6.2233	887.532	7.0625
44757	6.2233	796.625	92.5168
44758	6.2233	665.481	300.948
44759	6.2234	842.498	264.123
44760	6.2238	798.771	158.436
44761	6.2238	733.05	99.0734
44762	6.2247	595.171	58.7822
44763	6.225	984.726	244.887
44764	6.2251	791.475	87.123
44765	6.2251	-2.8	255.842
44766	6.2251	844.973	327.252
44767	6.2252	671.694	137.695
44768	6.2253	930.245	65.474
44769	6.2253	903.056	77.8605
44770	6.2254	909.233	303.38
44771	6.2255	887.472	132.572
44772	6.2255	940.377	30.4609
44773	6.2258	906.644	38.3422

44774	6.226	768.85	106.501
44775	6.226	675.239	55.9423
44776	6.2262	995.904	8.424
44777	6.2263	753.161	114.613
44778	6.2263	811.607	37.4871
44779	6.2264	838.706	131.597
44780	6.2264	955.495	54.8864
44781	6.2265	935.839	21.921
44782	6.2268	813.914	231.29
44783	6.2268	706.268	133.273
44784	6.2269	792.571	109.132
44785	6.2274	1005.48	13.4475
44786	6.2276	782.96	261.141
44787	6.2281	781.9	160.802
44788	6.2282	959.586	44.4566
44789	6.2284	907.834	53.2754
44790	6.2287	867.516	58.2545
44791	6.2289	711.46	252.853
44792	6.2292	728.223	36.7971
44793	6.2293	806.603	291.412
44794	6.2295	949.342	16.5908
44795	6.2295	609.772	221.423
44796	6.2295	893.887	96.3722
44797	6.2298	954.989	81.2943
44798	6.2301	752.873	172.863
44799	6.2303	697.081	220.407
44800	6.2303	896.307	52.6089
44801	6.2304	703.677	231.151
44802	6.2308	879.7	31.8054
44803	6.2311	756.278	129.296
44804	6.2312	685.189	195.846
44805	6.2313	840.696	176.926
44806	6.2314	813.007	28.3777
44807	6.2317	892.148	21.699
44808	6.2319	748.856	155.814
44809	6.2319	923.531	92.6604
44810	6.232	620.009	183.063
44811	6.2322	897.292	92.5002
44812	6.2322	657.756	202.177
44813	6.2323	753.53	192.639
44814	6.2324	717.297	186.279
44815	6.2327	824.367	175.969
44816	6.2328	828.054	35.2357
44817	6.2329	766.514	19.7274
44818	6.2333	944.213	12.2572
44819	6.2335	752.794	257.272
44820	6.2337	983.868	87.0206
44821	6.2338	808.477	87.507

44822	6.2341	858.946	88.2009
44823	6.2341	784.767	133.185
44824	6.2341	983.678	51.2521
44825	6.2342	604.934	27.9792
44826	6.2343	672.027	134.786
44827	6.2344	844.908	65.7876
44828	6.2344	770.046	258.319
44829	6.2347	784.796	321.441
44830	6.235	932.558	91.7769
44831	6.2351	840.827	87.076
44832	6.2352	743.826	161.12
44833	6.2352	776.504	155.867
44834	6.2354	886.47	66.438
44835	6.2355	986.864	23.6799
44836	6.2355	598.986	183.342
44837	6.2358	673.193	247.865
44838	6.2358	808.165	78.7931
44839	6.2359	910.646	86.0696
44840	6.236	923.389	103.239
44841	6.2361	724.143	174.849
44842	6.2363	884.458	39.8564
44843	6.2364	817.941	84.2521
44844	6.2366	772.582	167.615
44845	6.2367	676.813	233.373
44846	6.2369	855.661	94.6114
44847	6.2371	696.656	258.888
44848	6.2371	792.35	61.9383
44849	6.2372	884.072	85.0661
44850	6.2376	710.039	140.497
44851	6.238	852.995	75.4668
44852	6.2384	1056.89	259.875
44853	6.2387	822.86	253.141
44854	6.239	909.013	43.9255
44855	6.2391	1007.64	42.92
44856	6.2393	474.717	338.56
44857	6.2395	846.2	228.457
44858	6.2396	932.434	61.4497
44859	6.2401	680.516	115.924
44860	6.2402	812.02	196.847
44861	6.2402	951.678	62.6207
44862	6.2407	964.08	17.1407
44863	6.2408	707.712	229.315
44864	6.2413	694.425	122.213
44865	6.2415	821.435	146.168
44866	6.2421	843.456	327.304
44867	6.2421	885.602	198.972
44868	6.2425	829.912	121.072
44869	6.2427	754.627	129.968

44870	6.2429	856.915	225.615
44871	6.2429	850.517	262.766
44872	6.243	703.193	186.107
44873	6.2434	739.563	81.075
44874	6.2436	808.557	163.994
44875	6.2437	700.217	305.703
44876	6.2437	753.416	163.23
44877	6.2438	966.1	255.849
44878	6.2438	834.553	159.651
44879	6.2439	985.13	11.746
44880	6.244	854.081	171.927
44881	6.2441	704.687	178.753
44882	6.2441	669.801	143.738
44883	6.2444	685.785	155.238
44884	6.2445	845.777	42.739
44885	6.2453	980.752	38.3859
44886	6.2455	709.095	179.502
44887	6.2457	980.195	46.5655
44888	6.2462	882.674	49.6544
44889	6.2463	803.588	136.668
44890	6.2467	628.14	82.9981
44891	6.2468	936.126	332.569
44892	6.247	989.048	54.322
44893	6.247	700.275	181.611
44894	6.247	1053.3199	243.804
44895	6.2471	708.254	300.142
44896	6.2471	920.625	78.7386
44897	6.2471	966.01	50.4283
44898	6.2476	755.495	277.423
44899	6.2478	875.678	97.9784
44900	6.2482	775.258	151.012
44901	6.2483	744.495	155.733
44902	6.2485	836.648	110.094
44903	6.2486	874.908	196.662
44904	6.2487	737.649	183.268
44905	6.2488	790.805	148.464
44906	6.2489	789.463	160.406
44907	6.2489	922.947	29.7099
44908	6.2492	817.705	50.1768
44909	6.2496	727.836	86.2819
44910	6.2501	-4.3462	279.414
44911	6.2502	839.5	17.5475
44912	6.2505	912.684	41.6073
44913	6.2506	740.604	224.573
44914	6.2508	778.24	116.642
44915	6.2512	874.154	82.3469
44916	6.2516	766.817	226.542
44917	6.2518	790.392	191.571

44918	6.252	842.586	147.22
44919	6.2523	921.561	4.1558
44920	6.2523	719.348	148.919
44921	6.2524	785.497	198.137
44922	6.2528	923.677	299.37
44923	6.2528	841.883	32.7533
44924	6.253	693.461	227.073
44925	6.2533	941.151	62.4022
44926	6.2533	821.998	179.206
44927	6.2534	724.19	258.509
44928	6.2538	782.033	143.726
44929	6.2539	982.391	47.2051
44930	6.2541	835.462	121.951
44931	6.2541	694.673	155.513
44932	6.2548	784.217	196.319
44933	6.2549	714.349	171.815
44934	6.255	713.022	179.172
44935	6.255	792.1	127.512
44936	6.2553	956.464	52.3059
44937	6.2554	902.089	38.7869
44938	6.256	1024.04	258.66
44939	6.2562	980.726	259.054
44940	6.2562	812.001	350.319
44941	6.2564	753.793	76.7569
44942	6.2565	760.134	188.953
44943	6.2572	693.859	249.555
44944	6.2574	843.667	133.711
44945	6.2575	795.92	192.06
44946	6.2576	693.035	269.292
44947	6.2576	927.753	37.0694
44948	6.2576	900.211	79.5896
44949	6.2579	460.441	315.469
44950	6.2579	822.352	61.2173
44951	6.2581	786.938	124.25
44952	6.2581	920.607	61.3514
44953	6.2587	881.69	115.601
44954	6.2588	966.024	141.626
44955	6.2593	923.681	25.7005
44956	6.2594	689.958	282.957
44957	6.2599	808.227	265.784
44958	6.2601	-3.2509	328.17
44959	6.2602	972.579	56.3888
44960	6.261	934.683	64.4943
44961	6.261	916.526	63.6121
44962	6.2612	892.661	158.76
44963	6.2615	768.893	74.7924
44964	6.2615	986.933	13.8886
44965	6.2617	803.789	243.799

44966	6.2618	859.559	55.5765
44967	6.2619	803.371	339.039
44968	6.2621	775.332	255.972
44969	6.2623	752.516	187.714
44970	6.2623	845.151	333.832
44971	6.2631	790.217	75.6426
44972	6.2631	911.044	129.042
44973	6.2632	793.794	101.693
44974	6.2632	939.483	94.3329
44975	6.2635	729.874	228.127
44976	6.2638	802.585	122.135
44977	6.2639	928.116	98.0343
44978	6.264	748.997	219.495
44979	6.2643	748.636	184.603
44980	6.2644	775.706	119.16
44981	6.2645	814.195	237.819
44982	6.2645	1029.62	51.3518
44983	6.2646	828.401	236.558
44984	6.2646	789.105	74.1611
44985	6.2647	788.545	64.0833
44986	6.2651	951.049	99.53
44987	6.2653	818.353	192.776
44988	6.2654	823.3	281.681
44989	6.2655	840.82	198.78
44990	6.2659	831.181	59.3673
44991	6.2663	675.964	175.52
44992	6.2663	784.835	8.426
44993	6.2664	858.551	48.4554
44994	6.2668	799.934	189.514
44995	6.2672	721.201	146.44
44996	6.2673	838.051	149.277
44997	6.2674	788.839	274.064
44998	6.2681	947.634	58.0238
44999	6.2683	827.738	224.866
45000	6.2688	782.994	109.929
45001	6.2692	944.224	80.5506
45002	6.2694	742.682	221.047
45003	6.2694	954.592	37.4499
45004	6.2696	1041.99	37.4304
45005	6.2697	942.577	26.1543
45006	6.2698	781.845	242.672
45007	6.27	1015.7	10.4071
45008	6.2703	672.341	255.686
45009	6.2704	710.653	328.844
45010	6.2706	767.844	249.503
45011	6.271	840.442	329.432
45012	6.2712	925.941	195.382
45013	6.2715	901.435	270.533

45014	6.2716	800.949	256.458
45015	6.2719	752.991	212.693
45016	6.2723	970.895	288.131
45017	6.2734	990.842	32.4927
45018	6.2735	759.312	86.911
45019	6.2735	717.974	57.0166
45020	6.2736	1023.96	261.628
45021	6.2737	794.718	307.764
45022	6.2737	938.465	95.2018
45023	6.2737	809.3	232.23
45024	6.2738	873.928	117.774
45025	6.2741	931.825	47.3285
45026	6.2745	894.652	15.0988
45027	6.2745	779.917	235.705
45028	6.2749	942.152	33.7728
45029	6.2751	882.383	137.175
45030	6.2756	799.966	148.43
45031	6.2756	961.005	86.0706
45032	6.2757	768.376	222.186
45033	6.2757	783.413	161.872
45034	6.2765	1086.95	36.4906
45035	6.2765	650.229	146.548
45036	6.277	799.06	304.747
45037	6.277	937.135	302.775
45038	6.2774	770.297	256.029
45039	6.2774	850.463	68.1916
45040	6.2775	925.384	82.6118
45041	6.2776	843.042	218.708
45042	6.2782	264.089	217.617
45043	6.2782	760.631	160.316
45044	6.2786	850.227	154.352
45045	6.2788	944.757	60.2365
45046	6.2788	794.726	174.252
45047	6.2792	807.84	164.296
45048	6.2792	780.51	159.807
45049	6.2794	804.369	237.337
45050	6.2801	961.108	57.8786
45051	6.2802	999.776	267.215
45052	6.2804	232.515	21.1062
45053	6.2804	798.158	110.255
45054	6.2804	641.394	161.922
45055	6.2806	861.347	71.4174
45056	6.2807	750.302	143.733
45057	6.2809	927.212	52.0484
45058	6.2812	922.41	55.6206
45059	6.2814	738.678	151.563
45060	6.2814	828.399	257.562
45061	6.2818	894.026	333.015

45062	6.2821	714.853	141.983
45063	6.2822	790.202	240.621
45064	6.2822	1131.22	81.4212
45065	6.2825	773.497	257.757
45066	6.2828	735.641	188.465
45067	6.2832	786.112	187.419
45068	6.2836	910.572	50.587
45069	6.2836	959.999	21.7804
45070	6.2836	945.255	74.2653
45071	6.2839	921.254	226.231
45072	6.2842	786.421	218.446
45073	6.2843	871.034	191.676
45074	6.2843	784.92	283.205
45075	6.285	938.332	91.7311
45076	6.2856	850.337	69.5739
45077	6.2857	810.577	9.2724
45078	6.2858	923.555	78.7669
45079	6.2861	-2.9377	326.37
45080	6.2862	899.534	32.7078
45081	6.2862	982.272	46.1692
45082	6.2866	795.572	70.19
45083	6.2866	915.602	249.981
45084	6.2869	952.008	33.5483
45085	6.2871	1001.43	61.2708
45086	6.2874	966.745	108.677
45087	6.2879	977.482	45.5651
45088	6.2885	860.651	35.6397
45089	6.2886	761.043	188.976
45090	6.2888	786.691	354.282
45091	6.2891	800.798	163.472
45092	6.2895	904.254	308.071
45093	6.2898	730.27	144.129
45094	6.2909	682.522	1.3708
45095	6.291	957.228	94.9518
45096	6.2911	897.78	318.613
45097	6.2913	735.687	155.376
45098	6.2914	1027.61	14.946
45099	6.292	792.117	280.931
45100	6.292	843	161.119
45101	6.2921	975.508	42.0496
45102	6.2923	843.789	237.722
45103	6.2923	762.858	153.244
45104	6.2926	831.071	226.354
45105	6.2927	756.429	167.196
45106	6.293	870.55	81.2024
45107	6.2936	803.618	289.808
45108	6.2936	648.476	59.9312
45109	6.2938	856.883	193.451

45110	6.2938	767.151	147.991
45111	6.294	924.353	54.8009
45112	6.2942	798.688	49.9469
45113	6.2944	975.186	204.922
45114	6.2945	975.299	56.5388
45115	6.295	804.813	96.1998
45116	6.2951	746.585	114.572
45117	6.2952	922.844	52.2991
45118	6.2954	727.115	187.577
45119	6.2954	729.903	98.1722
45120	6.2955	946.917	101.245
45121	6.2955	859.412	128.242
45122	6.2957	787.768	196.135
45123	6.2959	833.08	113.25
45124	6.2959	855.758	236.501
45125	6.2961	750.512	239.695
45126	6.2961	803.933	93.2574
45127	6.2963	750.364	238.793
45128	6.2965	906.224	73.8344
45129	6.297	933.84	92.7901
45130	6.2971	825.738	76.4059
45131	6.2974	805.806	225.261
45132	6.2974	962.071	205.389
45133	6.2976	935.886	126.622
45134	6.2977	845.854	49.4102
45135	6.2978	874.956	158.731
45136	6.2979	943.023	49.7774
45137	6.2981	883.302	299.515
45138	6.2982	714.695	221.136
45139	6.2986	-2.7932	262.093
45140	6.2989	784.837	125.049
45141	6.2989	1026.99	51.136
45142	6.2991	951.516	191.011
45143	6.2997	850.071	88.6771
45144	6.3001	790.542	265.326
45145	6.3002	931.572	105.418
45146	6.3004	906.857	96.7908
45147	6.3004	902.88	36.6619
45148	6.3022	819.376	163.329
45149	6.3023	829.198	158.421
45150	6.3023	693.902	139.109
45151	6.3023	884.723	336.275
45152	6.3024	797.211	331.852
45153	6.3028	958.698	224.931
45154	6.3029	896.777	40.4996
45155	6.3032	893.769	94.4813
45156	6.3038	760.593	17.4048
45157	6.304	873.42	306.568

45158	6.3041	944.947	109.201
45159	6.3043	703.781	176.053
45160	6.3045	730.806	134.291
45161	6.3047	929.42	85.5846
45162	6.3048	719.534	238.573
45163	6.3048	948.66	243.943
45164	6.305	870.106	50.2301
45165	6.3051	908.975	109.35
45166	6.3052	756.561	170.397
45167	6.3054	803.185	63.4149
45168	6.3055	951.316	181.917
45169	6.3055	825.073	106.896
45170	6.3055	935.876	66.8899
45171	6.3057	745.577	88.3981
45172	6.3059	803.981	264.883
45173	6.306	946.407	75.5969
45174	6.3063	1012.07	54.3554
45175	6.3063	772.984	108.349
45176	6.3067	798.556	274.186
45177	6.3067	780.238	189.975
45178	6.307	789.355	319.181
45179	6.307	955.536	23.4797
45180	6.3071	824.683	146.925
45181	6.3073	946.024	79.0704
45182	6.3074	776.084	98.6059
45183	6.3078	829.882	291.14
45184	6.308	801.488	177.53
45185	6.3085	847.048	162.761
45186	6.3085	656.717	222.668
45187	6.3086	812.359	216.195
45188	6.3089	-2.8182	259.756
45189	6.3091	934.23	51.2641
45190	6.3093	732.038	20.3173
45191	6.3095	882.032	301.307
45192	6.3104	964.062	29.6761
45193	6.3105	887.362	218.575
45194	6.3106	860.444	119.053
45195	6.3111	796.596	181.844
45196	6.3112	729.54	159.385
45197	6.3114	965.621	24.8241
45198	6.3118	950.222	86.1011
45199	6.312	770.486	158.621
45200	6.3126	827.494	67.4085
45201	6.3129	707.499	193.162
45202	6.313	990.622	15.3046
45203	6.3131	806.316	52.1009
45204	6.3133	864.42	75.4817
45205	6.3134	942.248	65.1959

45206	6.3136	732.538	181.731
45207	6.314	893.149	262.307
45208	6.3142	737.949	156.452
45209	6.3142	811.16	92.7132
45210	6.3143	888.953	119.212
45211	6.3143	891.834	301.56
45212	6.3147	776.65	8.7036
45213	6.3147	896.359	162.226
45214	6.3148	820.901	93.2317
45215	6.3148	701.018	333.765
45216	6.3149	844.472	131.256
45217	6.3152	952.32	50.1725
45218	6.3153	805.937	177.144
45219	6.3155	747.087	97.7146
45220	6.3156	1018.25	33.6065
45221	6.3157	1049.65	43.1486
45222	6.3161	1053.6801	84.3481
45223	6.3162	724.87	108.091
45224	6.3164	1041.96	53.1688
45225	6.3164	854.274	117.77
45226	6.3165	876.546	77.7245
45227	6.3166	746.581	237.804
45228	6.3168	1098.84	22.305
45229	6.3172	764.322	114.378
45230	6.3172	941.967	46.14
45231	6.3174	903.751	14.6793
45232	6.3181	927.369	271.985
45233	6.3182	1061.8	95.3573
45234	6.3183	881.958	17.4665
45235	6.3183	730.423	78.4275
45236	6.3187	912.514	82.3226
45237	6.319	768.671	152.024
45238	6.3192	831.347	77.0764
45239	6.3193	697.432	182.983
45240	6.3195	782.804	27.4586
45241	6.3195	693.448	131.499
45242	6.3196	850.598	319.389
45243	6.3197	723.99	157.553
45244	6.3199	850.549	235.994
45245	6.3201	820.158	167.51
45246	6.3206	891.279	4.6545
45247	6.3214	765.358	186.612
45248	6.3216	317.924	359.242
45249	6.3217	780.324	195.959
45250	6.3222	911.879	92.741
45251	6.3222	-2.806	263.141
45252	6.3227	1029.67	53.8598
45253	6.3229	899.353	107.063

45254	6.323	883.566	65.9998
45255	6.3232	918.089	64.795
45256	6.3233	825.293	127.031
45257	6.3233	837.3	65.3181
45258	6.3236	814.189	218.444
45259	6.3236	974.405	75.7975
45260	6.3237	735.2	199.985
45261	6.3239	868.77	172.738
45262	6.3241	783.341	26.288
45263	6.3242	1028.77	77.0871
45264	6.3247	800.935	285.192
45265	6.3249	947.509	24.7563
45266	6.3252	976.565	111.018
45267	6.3255	778.05	78.3473
45268	6.3256	864.302	107.399
45269	6.326	839.462	302.121
45270	6.3263	880.791	77.1572
45271	6.3263	748.609	260.444
45272	6.3263	764.141	253.091
45273	6.3263	860.987	89.086
45274	6.3266	850.047	161.868
45275	6.3266	793.254	291.64
45276	6.3266	620.461	169.254
45277	6.327	901.561	32.5498
45278	6.3276	861.164	49.1847
45279	6.3277	886.47	177.693
45280	6.3279	1012.19	305.822
45281	6.328	953.864	31.9219
45282	6.3287	824.535	111.341
45283	6.3289	1038.25	102.869
45284	6.329	801.123	28.5452
45285	6.3295	581.409	250.017
45286	6.3295	825.1	166.944
45287	6.3299	907.063	140.693
45288	6.3301	885.415	341.755
45289	6.3302	822.87	86.0787
45290	6.3302	877.068	263.849
45291	6.3305	897.743	48.9824
45292	6.3306	865.894	145.481
45293	6.3308	930.428	207.461
45294	6.3311	836.807	148.045
45295	6.3315	317.471	21.9635
45296	6.3315	895.29	95.2556
45297	6.3317	937.182	171.953
45298	6.3319	671.314	181.461
45299	6.3324	837.651	17.4201
45300	6.3324	843.526	122.238
45301	6.3325	1011.13	30.0354

45302	6.3327	729.521	135.363
45303	6.3327	1004.1	48.4234
45304	6.3328	829.174	113.287
45305	6.3328	962.015	100.707
45306	6.333	926.6	34.2716
45307	6.3333	769.07	137.265
45308	6.3336	712.612	144.775
45309	6.3337	867.267	269.651
45310	6.334	802.574	90.8276
45311	6.3346	1024	17.3408
45312	6.3349	1020.09	69.7071
45313	6.335	930.575	100.578
45314	6.3352	852.058	176.757
45315	6.3354	818.201	297.733
45316	6.3356	927.335	47.4462
45317	6.3358	952.69	63.6442
45318	6.336	852.69	180.125
45319	6.3362	828.172	81.7615
45320	6.3368	959.347	101.446
45321	6.3369	856.385	166.127
45322	6.3369	738.285	160.827
45323	6.3372	960.832	117.644
45324	6.3373	932.331	33.6737
45325	6.3376	814.651	126.741
45326	6.3382	841.004	199.372
45327	6.3383	746.802	194.668
45328	6.3383	920.106	305.653
45329	6.3384	831.002	351.346
45330	6.3384	899.53	266.425
45331	6.3385	809.689	261.733
45332	6.3387	711.87	180.577
45333	6.339	849.464	302.213
45334	6.3393	796.267	114.118
45335	6.3395	883.576	248.264
45336	6.3396	947.884	253.892
45337	6.3397	792.434	20.6212
45338	6.3398	837.489	138.302
45339	6.3399	967.653	23.3779
45340	6.3399	1048.88	16.3085
45341	6.3403	875.52	8.3357
45342	6.3408	870.868	72.1696
45343	6.3409	1064.9	48.7138
45344	6.3409	687.723	249.762
45345	6.3412	1014.81	84.1498
45346	6.3412	768.995	162.999
45347	6.3413	832.153	31.314
45348	6.3417	836.542	113.523
45349	6.3417	965.867	217.13

45350	6.3419	820.824	179.606
45351	6.3423	907.312	333.112
45352	6.3423	949.831	302.587
45353	6.3428	856.116	231.155
45354	6.3428	979.285	115.457
45355	6.3429	861.892	99.4399
45356	6.3431	1011.23	87.3748
45357	6.3436	998.911	29.1108
45358	6.3438	1073.34	84.5333
45359	6.3439	792.534	104.852
45360	6.3444	806.203	175
45361	6.3445	779.301	229.942
45362	6.3446	929.647	101.66
45363	6.3449	783.9	187.72
45364	6.3449	919.167	217.64
45365	6.3452	917.41	72.8299
45366	6.3455	995.324	39.0509
45367	6.3455	939.456	130.768
45368	6.3456	1016.43	45.146
45369	6.3457	920.509	96.9554
45370	6.3459	972.696	20.7755
45371	6.3462	849.588	171.172
45372	6.3466	847.159	114.391
45373	6.3466	906.312	74.8669
45374	6.3469	791.787	120.381
45375	6.3469	824.41	72.6104
45376	6.3472	711.156	157.098
45377	6.3472	910.618	56.6838
45378	6.3475	847.597	89.2615
45379	6.3476	802.495	188.517
45380	6.3477	969.925	48.4838
45381	6.3482	844.426	164.347
45382	6.3486	733.554	172.688
45383	6.3486	848.259	136.529
45384	6.3488	969.278	28.2177
45385	6.3489	840.335	359.225
45386	6.349	1007.12	23.37
45387	6.349	764.47	114.364
45388	6.3491	802.813	243.355
45389	6.3493	865.82	136.687
45390	6.3496	753.142	172.8
45391	6.35	836.313	60.3176
45392	6.3501	1063.03	41.9534
45393	6.3509	1017.24	55.4645
45394	6.3514	813.668	131.799
45395	6.3521	833.177	226.966
45396	6.3522	717.499	179.905
45397	6.3524	855.638	133.933

45398	6.3526	911.145	20.2743
45399	6.3527	853.377	59.9355
45400	6.3531	911.48	99.1163
45401	6.3533	954.183	32.0735
45402	6.3541	707.65	146.797
45403	6.3543	822.582	154.648
45404	6.3544	1107.8101	36.4935
45405	6.3546	903.013	165.813
45406	6.3546	855.102	190.06
45407	6.3546	955.639	134.206
45408	6.3553	808.692	256.644
45409	6.3553	851.809	136.144
45410	6.3553	1023.75	23.2636
45411	6.3554	891.236	65.3992
45412	6.3555	737.724	250.632
45413	6.3555	880.64	73.1501
45414	6.3556	723.369	178.734
45415	6.3556	768.111	137.674
45416	6.3556	808.981	252.94
45417	6.3556	974.967	281.638
45418	6.3564	789.121	168.607
45419	6.3565	866.37	9.4001
45420	6.3565	905.885	95.1493
45421	6.3566	767.542	198.081
45422	6.3569	1045.64	240.038
45423	6.3572	726.231	164.051
45424	6.3574	755.575	297.623
45425	6.3577	1106.49	262.348
45426	6.3579	894.503	33.5751
45427	6.3579	835.58	338.06
45428	6.3581	955.625	97.4373
45429	6.3584	848.12	23.5366
45430	6.3585	886.594	74.5672
45431	6.3585	889.889	232.152
45432	6.3586	722.915	86.2389
45433	6.3586	743.856	250.604
45434	6.3586	821.26	161.681
45435	6.3587	800.651	157.435
45436	6.3587	960.891	76.5379
45437	6.3588	932.278	141.768
45438	6.3591	1069.74	99.9086
45439	6.3594	1042.84	260.315
45440	6.3598	839.958	246.825
45441	6.3599	957.387	133.193
45442	6.3602	969.486	60.7857
45443	6.3602	777.454	319.135
45444	6.3602	860.16	102.225
45445	6.3606	745.468	304.238

45446	6.3608	741.286	226.13
45447	6.361	-2.8411	276.17
45448	6.3614	728.489	227.004
45449	6.3614	820.068	359.95
45450	6.3614	763.575	166.29
45451	6.3616	950.455	30.0974
45452	6.3616	892.036	145.302
45453	6.3616	831.697	9.708
45454	6.3616	977.58	48.1158
45455	6.3617	797.263	168.187
45456	6.362	825.673	164.233
45457	6.3621	909.069	21.2343
45458	6.3622	829.347	0.9954
45459	6.3624	871.921	77.1311
45460	6.3626	924.704	261.622
45461	6.3629	878.009	186.981
45462	6.363	835.019	114.835
45463	6.3631	863.508	137.369
45464	6.3636	945.76	28.2997
45465	6.3636	830.215	181.047
45466	6.3637	793.954	176.429
45467	6.3641	966.443	85.572
45468	6.3642	782.033	124.334
45469	6.3646	1041.4	11.887
45470	6.3648	812.984	168.665
45471	6.365	815.114	229.599
45472	6.3654	773.872	156.251
45473	6.3655	828.402	192.312
45474	6.3655	662.968	163.125
45475	6.3656	797.016	174.763
45476	6.3657	825.711	273.505
45477	6.3657	1030.89	12.8435
45478	6.366	934.096	23.5901
45479	6.3662	898.765	39.1979
45480	6.3663	771.213	180.423
45481	6.3664	1030.17	29.1677
45482	6.3665	906.863	315.292
45483	6.3665	1028.22	35.3562
45484	6.3667	754.164	62.8505
45485	6.3669	856.939	127.918
45486	6.367	915.818	7.1684
45487	6.3671	966.166	111.988
45488	6.3672	917.304	24.6569
45489	6.3672	961.007	18.4545
45490	6.3673	967.04	23.1204
45491	6.3679	838.463	297.561
45492	6.368	809.843	231.261
45493	6.368	863.728	37.8135

45494	6.3682	784.426	150.574
45495	6.3683	958.312	25.132
45496	6.3686	905.983	340.039
45497	6.3689	937.391	24.3767
45498	6.3694	940.996	87.139
45499	6.3698	1042.67	14.033
45500	6.3704	911.483	36.0335
45501	6.3704	819.932	142.256
45502	6.3709	685.293	328.358
45503	6.3709	892.542	5.3979
45504	6.371	837.886	7.2879
45505	6.3712	883.672	358.947
45506	6.3715	788.206	83.3771
45507	6.3717	996.853	27.0814
45508	6.3718	830.591	163.706
45509	6.3722	1014.19	34.9103
45510	6.3725	939.381	35.665
45511	6.3726	870.767	162.717
45512	6.3726	839.073	175.384
45513	6.3728	773.597	176.43
45514	6.3729	877.034	179.838
45515	6.3733	748.352	151.688
45516	6.3736	749.63	177.892
45517	6.3737	917.473	58.8497
45518	6.374	-2.8048	77.955
45519	6.3742	907.469	242.911
45520	6.3744	711.659	192.015
45521	6.3745	1061.5601	187.054
45522	6.3747	836.392	122.222
45523	6.3747	762.36	147.097
45524	6.375	864.133	240.014
45525	6.3751	740.131	140.774
45526	6.3756	-2.8071	260.018
45527	6.3768	1005.93	30.4255
45528	6.3769	875.734	148.857
45529	6.377	967.385	115.072
45530	6.3777	780.344	280.109
45531	6.3778	1027.87	1.1674
45532	6.3778	851.717	186.539
45533	6.3779	1003.76	13.9189
45534	6.378	821.813	151.021
45535	6.3787	874.581	313.462
45536	6.3789	-2.829	77.7389
45537	6.379	933.536	112.082
45538	6.3802	909.421	191.04
45539	6.3802	868.469	258.546
45540	6.3802	780.752	145.539
45541	6.3805	781.274	221.91

45542	6.3806	997.511	152.337
45543	6.3806	870.484	81.8445
45544	6.3806	763.277	79.7134
45545	6.3807	809.337	226.267
45546	6.3807	728.789	201.444
45547	6.3807	956.945	110.579
45548	6.3808	820.815	218.938
45549	6.3808	776.086	148.397
45550	6.3808	962.956	118.059
45551	6.3809	780.45	186.235
45552	6.3809	887.449	173.927
45553	6.3811	1043.63	242.449
45554	6.3815	852.143	157.367
45555	6.3815	927.836	95.4745
45556	6.3816	825.465	156.253
45557	6.3818	1032.96	26.4638
45558	6.3821	933.911	276.214
45559	6.3821	892.899	212.605
45560	6.3824	1049.6801	325.43
45561	6.3829	823.615	274.297
45562	6.3829	1031.41	92.4286
45563	6.3832	746.587	167.577
45564	6.3837	834.375	153.426
45565	6.384	828.753	151.115
45566	6.384	877.029	63.1526
45567	6.3841	1134.89	262.669
45568	6.3843	861.156	162.35
45569	6.3843	990.279	283.509
45570	6.3844	1039.85	23.5104
45571	6.3844	1041.08	22.8507
45572	6.3845	873.537	56.9046
45573	6.3847	957.174	100.725
45574	6.3847	775.961	220.23
45575	6.385	941.796	102.574
45576	6.3852	768.272	141.493
45577	6.386	940.184	171.163
45578	6.3864	962.712	58.7733
45579	6.3864	993.191	50.1882
45580	6.3866	789.967	291.464
45581	6.3867	980.405	102.399
45582	6.3867	820.941	303.225
45583	6.3869	889.302	190.46
45584	6.3873	952.449	95.1632
45585	6.3876	935.844	43.1468
45586	6.3878	888.495	102.102
45587	6.388	804.186	184.743
45588	6.3882	874.076	112.96
45589	6.3885	783.049	239.784

45590	6.3886	988.833	26.1681
45591	6.3886	889.879	17.3811
45592	6.3893	1059.79	107.431
45593	6.3896	861.195	10.3334
45594	6.3897	883.536	90.745
45595	6.3899	965.507	77.8434
45596	6.39	816.788	122.202
45597	6.3901	1044.49	24.1347
45598	6.3902	856.51	172.393
45599	6.3903	859.143	131.306
45600	6.3903	1034.86	148.361
45601	6.3904	957.262	73.3673
45602	6.3907	769.545	154.382
45603	6.3907	980.434	53.6576
45604	6.391	859.293	252.177
45605	6.391	1003.82	47.7184
45606	6.3911	1069.4399	43.2485
45607	6.3912	984.781	50.9382
45608	6.3912	788.265	161.25
45609	6.3913	880.793	249.69
45610	6.3913	827.165	164.575
45611	6.3914	975.646	35.6931
45612	6.3914	661.381	71.0025
45613	6.3916	848.256	74.1138
45614	6.3916	934.834	49.932
45615	6.392	1028.87	24.8383
45616	6.392	868.053	81.6514
45617	6.392	900.068	21.1221
45618	6.3922	917.976	81.0543
45619	6.3926	748.252	337.004
45620	6.3929	949.088	191.526
45621	6.3932	869.551	70.1792
45622	6.3936	888.811	140.793
45623	6.3937	871.031	73.5995
45624	6.3939	808.676	56.4772
45625	6.3945	1025.88	25.4611
45626	6.3946	853.372	10.7325
45627	6.3947	755.549	131.562
45628	6.3957	976.867	117.761
45629	6.3961	756.254	255.752
45630	6.3962	833.184	121.456
45631	6.3969	839.2	80.6609
45632	6.397	868.39	9.6383
45633	6.3971	824.488	148.738
45634	6.3975	763.301	222.846
45635	6.3977	995.943	335.853
45636	6.3978	923.075	8.1767
45637	6.3981	885.882	322.223

45638	6.3986	889.926	211.168
45639	6.3986	863.674	62.4913
45640	6.399	972.558	37.2562
45641	6.399	921.174	54.8165
45642	6.3991	890.113	229.15
45643	6.3991	731.793	158.823
45644	6.3992	819.612	164.505
45645	6.3994	795.691	274.521
45646	6.3995	835.31	53.2455
45647	6.3996	-4.1298	78.1043
45648	6.3999	944.662	69.6307
45649	6.3999	825.926	169.26
45650	6.3999	852.204	81.2992
45651	6.4	862.327	75.5549
45652	6.4	907.471	164.36
45653	6.4011	922.292	158.251
45654	6.4012	1068.5601	68.5656
45655	6.4015	722.566	218.951
45656	6.4016	914.824	333.238
45657	6.4016	855.027	36.9221
45658	6.4017	941.194	40.3228
45659	6.4019	1013.18	40.9974
45660	6.4019	908.5	98.3423
45661	6.402	975.38	97.9139
45662	6.4027	946.608	190.202
45663	6.4032	890.7	51.4831
45664	6.4034	869.15	127.514
45665	6.4034	846.773	54.7375
45666	6.4036	898.636	266.258
45667	6.4041	1038.33	79.5212
45668	6.4044	720.847	252.012
45669	6.4045	951.783	91.1416
45670	6.4047	871.717	78.9126
45671	6.4047	1116.65	37.6012
45672	6.4048	987.341	320.934
45673	6.405	989.666	86.1961
45674	6.405	942.518	69.154
45675	6.4053	749.376	251.843
45676	6.4061	801.045	249.997
45677	6.4063	1005.42	77.6263
45678	6.4066	952.691	131.034
45679	6.4068	825.159	142.352
45680	6.4068	903.442	330.77
45681	6.4069	865.914	283.227
45682	6.4071	826.381	239.604
45683	6.4071	816.599	327.846
45684	6.4072	1010.7	15.7946
45685	6.4073	966.216	34.0489

45686	6.4077	897.461	167.248
45687	6.4081	888.341	302.22
45688	6.4081	1000.64	38.3183
45689	6.4084	773.187	115.635
45690	6.4088	827.455	252.823
45691	6.4095	836.301	224.331
45692	6.4097	929.046	133.983
45693	6.4099	936.636	197.627
45694	6.4102	985.913	39.8876
45695	6.4107	913.478	186.33
45696	6.411	1102.67	20.6926
45697	6.4114	908.438	184.459
45698	6.4115	646.256	159.829
45699	6.4115	817.369	195.691
45700	6.4116	860.802	34.6207
45701	6.4118	883.476	167.943
45702	6.4123	838.284	161.331
45703	6.4123	936.416	51.853
45704	6.4125	905.237	69.9811
45705	6.4126	1088.02	95.946
45706	6.4128	908.717	51.1038
45707	6.4129	836.653	169.674
45708	6.413	1014.73	50.9882
45709	6.4131	701.292	54.8207
45710	6.4142	849.925	130.4
45711	6.4145	845.494	261.708
45712	6.4148	799.487	113.979
45713	6.4149	929.129	140.271
45714	6.4152	964.636	81.0859
45715	6.4152	836.876	254.065
45716	6.4153	958.844	102.186
45717	6.4153	859.07	162.367
45718	6.4155	906.959	135.386
45719	6.4156	933.288	77.0928
45720	6.4156	850.137	53.8208
45721	6.4156	1133.51	243.053
45722	6.4157	890.43	80.6399
45723	6.4159	914.003	105.085
45724	6.416	867.352	214.131
45725	6.4161	978.466	276.771
45726	6.4169	808.24	149.696
45727	6.417	766.176	181.717
45728	6.417	901.334	50.3948
45729	6.4176	793.153	97.5868
45730	6.4179	768.869	195.413
45731	6.4181	961.342	170.601
45732	6.4187	894.381	88.0757
45733	6.4194	908.427	208.689

45734	6.4198	744.723	164.283
45735	6.4198	899.472	332.842
45736	6.4198	793.698	66.9261
45737	6.4201	879.179	112.482
45738	6.4205	804.968	168.87
45739	6.4206	1056.35	49.9119
45740	6.4209	800.15	189.762
45741	6.421	834.315	132.906
45742	6.4211	865.535	91.8189
45743	6.4211	481.194	336.785
45744	6.4217	819.079	184.938
45745	6.4218	823.097	145.599
45746	6.4223	947.044	118.588
45747	6.4224	885.18	73.1909
45748	6.4226	475.834	325.853
45749	6.4226	927.294	27.1551
45750	6.4229	989.849	354.477
45751	6.423	841.57	194.666
45752	6.4235	836.552	53.0445
45753	6.4236	845.725	348.471
45754	6.4236	922.633	281.231
45755	6.4239	812.082	252.279
45756	6.424	912.13	257.231
45757	6.4242	922.985	217.662
45758	6.4245	865.089	206.748
45759	6.4247	755.806	218.261
45760	6.4259	687.766	158.023
45761	6.4261	690.421	151.623
45762	6.4268	825.555	233.768
45763	6.4268	969.465	100.412
45764	6.4269	875.754	27.8598
45765	6.4274	847.262	32.0851
45766	6.4274	767.846	226.132
45767	6.4275	810.158	144.333
45768	6.4279	765.948	219.236
45769	6.428	850.865	179.672
45770	6.4282	932.518	91.4494
45771	6.4283	947.875	324.857
45772	6.4283	822.503	331.201
45773	6.4287	1015.31	34.1602
45774	6.4288	771.355	177.865
45775	6.429	815.638	131.912
45776	6.4291	798.809	160.119
45777	6.4292	810.247	149.192
45778	6.4294	1078.1801	20.1009
45779	6.4295	914.382	47.7707
45780	6.4304	1020.73	76.2715
45781	6.4309	858.003	9.4875

45782	6.4311	917.296	97.553
45783	6.4311	1034.55	17.236
45784	6.4312	931.037	332.957
45785	6.4316	880.733	59.6401
45786	6.432	805.614	106.402
45787	6.4323	812.704	187.648
45788	6.4325	466.358	327.107
45789	6.4326	903.158	18.0479
45790	6.4328	903.051	226.783
45791	6.4328	804.635	256.949
45792	6.4333	889.99	299.518
45793	6.4333	906.729	113.516
45794	6.4335	850.994	23.1423
45795	6.4336	939.59	72.482
45796	6.4336	760.544	106.746
45797	6.4337	1023.71	260.997
45798	6.4337	797.033	99.1524
45799	6.4338	939.005	259.567
45800	6.4339	703.336	219.836
45801	6.434	951.612	103.602
45802	6.4341	898.12	147.08
45803	6.435	906.578	78.6464
45804	6.4351	482.547	354.023
45805	6.4352	863.199	109.372
45806	6.4352	755.879	146.809
45807	6.4355	952.333	77.6696
45808	6.4356	790.16	300.715
45809	6.4358	850.813	181.77
45810	6.4361	963.922	96.4684
45811	6.4364	830.059	228.871
45812	6.4365	821.436	67.1834
45813	6.4366	886.542	84.6628
45814	6.4366	811.809	185.15
45815	6.4369	808.351	230.437
45816	6.4369	1023.76	135.355
45817	6.4369	1059.5	84.53
45818	6.4371	1069.91	24.856
45819	6.4373	823.933	147.261
45820	6.4375	894.785	44.1159
45821	6.438	735.011	333.728
45822	6.4381	675.098	59.8222
45823	6.4381	913.319	36.0712
45824	6.4382	918.155	14.4067
45825	6.4383	891.622	38.2638
45826	6.4383	946.843	113.75
45827	6.4387	1172.53	39.7088
45828	6.4387	761.163	156.007
45829	6.4388	1058.72	325.447

45830	6.4389	871.756	112.416
45831	6.4389	865.297	161.809
45832	6.4391	888.943	182.092
45833	6.4391	1017.72	110.139
45834	6.4395	743.276	244.402
45835	6.4397	867.016	51.8644
45836	6.4398	1054.03	302.807
45837	6.4398	856.681	26.4599
45838	6.4399	862.912	146.585
45839	6.4399	864.086	125.613
45840	6.44	911.962	193.926
45841	6.4404	892.637	293.902
45842	6.4404	897.539	233.482
45843	6.4405	866.854	118.763
45844	6.4407	871.515	264.687
45845	6.4408	890.975	176.776
45846	6.4408	952.913	103.358
45847	6.4411	874.883	338.144
45848	6.4413	926.11	181.504
45849	6.4413	949.089	123.786
45850	6.4413	841.174	154.814
45851	6.4416	856.394	143.495
45852	6.4416	833.917	254.504
45853	6.4417	836.083	169.285
45854	6.4419	926.487	193.039
45855	6.442	869.013	124.404
45856	6.442	734.979	331.68
45857	6.4426	886.199	256.472
45858	6.4431	987.81	15.2676
45859	6.4435	832.976	169.895
45860	6.4436	902.073	61.2411
45861	6.4436	948.87	236.113
45862	6.4436	908.824	128.447
45863	6.4438	936.713	115.019
45864	6.4439	862.849	125.563
45865	6.4442	767.429	193.178
45866	6.4443	1035.39	25.1415
45867	6.4445	789.024	199.614
45868	6.445	808.31	253.966
45869	6.445	919.991	24.9136
45870	6.4452	803.703	83.7613
45871	6.4452	716.091	304.78
45872	6.4461	1037.09	27.4282
45873	6.4462	870.869	55.776
45874	6.4466	936.466	40.0672
45875	6.4466	938.364	84.586
45876	6.4467	-2.7964	259.202
45877	6.4469	910.101	47.9419

45878	6.4471	1041.61	94.976
45879	6.4472	943.366	170.576
45880	6.4473	960.562	110.115
45881	6.4474	948.723	28.4985
45882	6.4475	875.672	102.695
45883	6.4479	1057.95	36.2701
45884	6.448	889.644	116.025
45885	6.4481	914.463	254.536
45886	6.4485	1019.73	95.9538
45887	6.4489	978.454	24.5114
45888	6.4489	869.946	302.905
45889	6.4491	893.529	315.884
45890	6.4494	1098.37	6.7363
45891	6.4496	834.383	145.47
45892	6.4497	820.818	153.031
45893	6.4499	885.27	28.0654
45894	6.4502	973.783	140.005
45895	6.4504	719.861	180.404
45896	6.4504	812.583	109.019
45897	6.4506	883.707	73.1473
45898	6.4507	916.354	146.356
45899	6.4507	932.869	299.066
45900	6.4508	922.761	300.248
45901	6.4508	983.321	104.161
45902	6.4509	852.727	55.6555
45903	6.4512	817.946	55.3554
45904	6.4517	948.156	82.9434
45905	6.452	857.113	116.283
45906	6.4524	888.485	249.589
45907	6.4527	747.027	237.781
45908	6.4534	948.627	57.9726
45909	6.4535	940.753	101.575
45910	6.4537	783.622	161.796
45911	6.4537	854.155	114.602
45912	6.4546	1023.43	103.65
45913	6.4548	1004.71	35.5827
45914	6.4549	914.301	102.035
45915	6.4554	620.289	88.6483
45916	6.4556	989.577	21.3462
45917	6.4558	775.659	195.118
45918	6.4566	900.353	299.587
45919	6.4567	922.64	191.647
45920	6.457	1085.83	239.67
45921	6.4574	845.44	245.02
45922	6.4575	831.045	265.388
45923	6.4576	756.151	137.671
45924	6.458	912.284	84.188
45925	6.4581	961.631	157.6

45926	6.4581	948.069	12.9122
45927	6.4581	811.488	140.052
45928	6.4582	872.46	140.324
45929	6.4583	893.297	147.916
45930	6.4584	890.59	178.35
45931	6.4585	758.171	222.409
45932	6.4595	946.059	329.338
45933	6.46	859.989	97.4246
45934	6.4607	1002.14	238.376
45935	6.461	907.286	277.004
45936	6.4613	1005.1	244.896
45937	6.4614	1034.37	21.8113
45938	6.4616	864.791	20.5424
45939	6.4621	871.189	91.9079
45940	6.4623	888.361	19.3538
45941	6.4628	776.517	239.117
45942	6.463	907.941	95.2022
45943	6.4632	1070.95	24.9065
45944	6.4633	888.905	77.6407
45945	6.4634	836.056	167.529
45946	6.4635	899.067	258.116
45947	6.4635	1063.55	108.241
45948	6.464	995.131	48.7239
45949	6.4641	828.975	127.188
45950	6.4642	789.947	255.628
45951	6.4646	923.592	56.793
45952	6.4648	275.532	198.14
45953	6.4653	963.332	29.5615
45954	6.4653	943.15	191.92
45955	6.4666	1064.8199	26.5833
45956	6.4668	974.616	61.4399
45957	6.4671	770.538	179.518
45958	6.4671	741.489	173.949
45959	6.4671	752.977	165.478
45960	6.4672	895.261	129.237
45961	6.4675	953.154	69.6473
45962	6.4676	969.423	88.5998
45963	6.4676	824.322	131.754
45964	6.4679	483.007	345.78
45965	6.4686	857.646	238.048
45966	6.4688	571.537	79.738
45967	6.4689	843.27	171.824
45968	6.4689	845.996	233.215
45969	6.4689	927.964	99.8979
45970	6.4691	787.508	276.988
45971	6.4693	925.553	40.5525
45972	6.4697	835.876	87.0982
45973	6.4697	896.971	106.7

45974	6.4697	1025.1801	50.3496
45975	6.47	1093.14	97.1667
45976	6.4702	832.175	252.915
45977	6.4703	990.178	52.7289
45978	6.4705	902.379	299.208
45979	6.4705	977.02	191.458
45980	6.4705	879.661	130.57
45981	6.4707	1027.45	324.36
45982	6.4707	798.986	296.617
45983	6.471	746.335	153.399
45984	6.4715	998.125	289.935
45985	6.4717	856.022	66.1037
45986	6.4719	766.999	159.449
45987	6.4722	944.658	335.541
45988	6.4722	750.788	178.367
45989	6.4724	970.215	104.699
45990	6.4724	942.308	73.965
45991	6.4724	1028.5601	30.7441
45992	6.4724	961.739	110.359
45993	6.4728	948.187	280.648
45994	6.473	1104.84	88.4046
45995	6.4735	1000.98	36.1245
45996	6.4736	950.21	58.4286
45997	6.4739	950.606	292.952
45998	6.474	989.13	34.0145
45999	6.4742	971.563	153.754
46000	6.4743	1089.6899	21.6609
46001	6.4751	846.014	310.981
46002	6.4751	943.513	124.025
46003	6.4752	980.567	33.6391
46004	6.4753	946.892	62.7911
46005	6.4756	891.292	174.908
46006	6.4759	873.237	66.683
46007	6.4768	796.172	196.427
46008	6.4769	804.229	195.333
46009	6.4775	921.384	275.039
46010	6.4776	970.3	124.545
46011	6.4778	744.14	221.727
46012	6.4779	845.536	141.878
46013	6.4779	966.422	173.087
46014	6.4784	856.579	223.946
46015	6.4786	933.287	22.7591
46016	6.4787	952.323	155.408
46017	6.4788	900.804	28.5549
46018	6.4792	1036.08	301.893
46019	6.4792	883.242	236.973
46020	6.4796	924.008	174.296
46021	6.48	821.229	298.788

46022	6.4801	865.813	175.305
46023	6.4802	688.375	25.3832
46024	6.4802	898.072	13.8414
46025	6.4806	812.783	191.191
46026	6.4807	799.207	231.415
46027	6.4807	701.041	168.899
46028	6.4809	844.121	116.716
46029	6.481	945.913	65.5099
46030	6.4813	872.189	174.916
46031	6.4814	832.145	230.414
46032	6.4817	932.456	287.63
46033	6.4818	1113.55	245.487
46034	6.4821	948.521	189.674
46035	6.4825	837.872	162.849
46036	6.4825	864.951	38.73
46037	6.4827	934.755	190.194
46038	6.4828	828.587	169.324
46039	6.483	843.049	232.208
46040	6.483	827.249	32.8771
46041	6.4833	875.086	194.809
46042	6.4835	1029.5601	120.061
46043	6.484	810.036	235.945
46044	6.4843	717.565	160.016
46045	6.4845	834.232	197.462
46046	6.4846	976.263	94.6097
46047	6.4846	1027.35	16.9658
46048	6.485	998.606	31.8724
46049	6.4854	1155.55	31.5515
46050	6.4854	786.444	183.055
46051	6.4856	890.573	73.6302
46052	6.4857	910.108	111.587
46053	6.4859	842.231	113.518
46054	6.4861	873.036	164.26
46055	6.4866	979.159	227.155
46056	6.4867	963.641	63.8904
46057	6.4873	947.149	269.747
46058	6.4879	1056.97	13.3807
46059	6.4885	868.637	125.303
46060	6.4886	934.238	123.465
46061	6.4888	657.096	158.899
46062	6.4889	963.054	317.128
46063	6.489	723.047	160.546
46064	6.49	957.562	301.137
46065	6.4901	1010.64	2.5387
46066	6.4901	1167.84	45.2451
46067	6.4903	890.128	235.399
46068	6.4905	1093.73	27.4843
46069	6.4907	796.019	176.393

46070	6.4913	768.954	186.9
46071	6.4914	1040.88	27.3447
46072	6.4917	950.721	85.0796
46073	6.4919	814.697	94.2504
46074	6.4922	814.368	182.4
46075	6.4923	264.301	195.885
46076	6.4925	911.58	144.466
46077	6.4926	791.015	146.653
46078	6.4931	922.852	28.7328
46079	6.4932	797.45	132.639
46080	6.4933	1053.2	27.7736
46081	6.4934	980.832	97.6357
46082	6.4935	892.188	45.2067
46083	6.4936	832.88	267.452
46084	6.4942	933.299	259.249
46085	6.4944	828.81	223.804
46086	6.4948	901.959	134.492
46087	6.4949	1053.4	99.7474
46088	6.4949	846.575	189.237
46089	6.4956	1084	17.5051
46090	6.4963	1072.79	285.723
46091	6.4963	943.698	67.068
46092	6.4964	776.701	185.008
46093	6.4965	1076.79	23.5228
46094	6.4966	903.032	148.62
46095	6.4967	1078.4399	52.1903
46096	6.4973	792.114	20.1209
46097	6.4977	967.945	348.19
46098	6.4981	905.416	298.945
46099	6.4982	1001.02	272.447
46100	6.4984	916.882	80.1668
46101	6.4986	962.446	81.2619
46102	6.4987	874.03	58.9997
46103	6.4994	837.513	188.721
46104	6.4998	893.255	274.99
46105	6.5	949.764	166.888
46106	6.5001	923.622	153.306
46107	6.5002	946.372	63.8638
46108	6.5003	1094.66	41.3123
46109	6.5004	1080.28	59.3161
46110	6.5005	1066.98	97.0628
46111	6.5005	1044.48	40.2097
46112	6.5009	1092.71	18.6815
46113	6.5016	886.996	249.573
46114	6.5016	898.926	140.402
46115	6.5018	820.201	178.904
46116	6.502	1050.76	25.7482
46117	6.502	1013.95	58.4791

46118	6.5022	855.394	222.515
46119	6.5024	928.18	77.6752
46120	6.5024	463.04	325.703
46121	6.5026	1059.52	44.4766
46122	6.5033	876.515	152.86
46123	6.5034	968.503	258.23
46124	6.5034	940.083	75.8555
46125	6.5035	903.234	236.967
46126	6.5035	798.536	253.116
46127	6.5038	1017.55	47.9979
46128	6.5041	1036.84	36.3433
46129	6.5042	714.522	184.679
46130	6.5042	1039.84	108.551
46131	6.5051	879.571	73.9943
46132	6.5051	840.513	129.159
46133	6.5052	1079.5	21.6645
46134	6.5057	853.716	267.825
46135	6.5065	897.59	227.665
46136	6.5068	1062.46	26.4468
46137	6.5069	872.025	243.564
46138	6.5076	794.254	184.612
46139	6.5079	945.522	247.365
46140	6.5079	1029.05	345.07
46141	6.5081	1045.26	113.802
46142	6.5081	803.508	194.833
46143	6.5083	923.089	191.472
46144	6.5083	940.012	222.743
46145	6.5087	912.722	136.232
46146	6.5095	919.752	20.5167
46147	6.5096	1177.79	23.578
46148	6.5098	988.918	19.3373
46149	6.5104	876.094	67.7293
46150	6.5104	989.856	10.8195
46151	6.5105	1102.85	113.893
46152	6.5106	1001.1	82.613
46153	6.5107	927.292	257.066
46154	6.5108	863.433	165.285
46155	6.5113	907.67	107.567
46156	6.5116	919.727	189.147
46157	6.5118	1095.39	92.1947
46158	6.512	1101.5	141.691
46159	6.5123	1095.2	83.6734
46160	6.5124	1010.91	26.8437
46161	6.5129	869.622	197.555
46162	6.5129	976.636	202.358
46163	6.5131	739.348	166.385
46164	6.5132	258.795	27.6144
46165	6.5134	882.56	296.627

46166	6.5134	781.099	117.755
46167	6.5136	1095.54	50.0116
46168	6.5146	903.56	155.009
46169	6.515	963.577	111.034
46170	6.5156	1023	115.773
46171	6.5157	876.553	248.21
46172	6.5157	844.839	304.584
46173	6.5157	967.154	71.1804
46174	6.5157	981.37	190.323
46175	6.5162	954.475	97.2689
46176	6.5163	1183.4	13.1078
46177	6.5169	1112.41	259.775
46178	6.5175	1113.59	46.8238
46179	6.5176	1057.5699	44.0529
46180	6.5177	866.532	129.518
46181	6.5178	831.304	178.162
46182	6.5179	879.584	75.5358
46183	6.518	759.822	202.823
46184	6.5184	1029.72	32.623
46185	6.5189	684.375	145.516
46186	6.5192	933.917	26.3519
46187	6.5193	1106.9399	93.039
46188	6.5194	1024.28	47.4051
46189	6.5198	990.349	71.0299
46190	6.5202	766.011	314.695
46191	6.5203	859.631	143.157
46192	6.5203	1059.67	37.9969
46193	6.5206	881.263	166.542
46194	6.5207	951.747	76.7201
46195	6.521	947.338	115.044
46196	6.5213	942.44	81.3463
46197	6.5219	933.429	80.6099
46198	6.5225	1013.42	66.0707
46199	6.5234	923.453	191.435
46200	6.5236	1054.8199	118.464
46201	6.5237	955.664	49.2239
46202	6.5238	914.84	164.175
46203	6.5239	796.454	183.708
46204	6.5239	1041.4	31.3496
46205	6.524	962.277	166.942
46206	6.5241	993.408	169.827
46207	6.5246	854.432	220.543
46208	6.5247	841.883	143.332
46209	6.5248	845.69	255.671
46210	6.5251	849.483	235.003
46211	6.5252	1077.5699	282.903
46212	6.5254	953.323	121.291
46213	6.5259	962.443	45.068

46214	6.526	841.806	163.654
46215	6.5262	957.909	92.8088
46216	6.5263	923.198	186.682
46217	6.5264	1024.84	168.994
46218	6.5266	1017.73	24.7723
46219	6.5268	1100.1	25.5413
46220	6.5269	1001.34	95.4481
46221	6.527	1093.35	31.0627
46222	6.5276	816.887	80.9398
46223	6.5281	979.752	130.964
46224	6.5283	890.628	142.153
46225	6.5285	1177.67	17.0129
46226	6.5286	1045.1801	100.294
46227	6.5287	977.399	38.8007
46228	6.5289	1017.83	34.0636
46229	6.5291	935.873	166.883
46230	6.5295	1175.13	45.323
46231	6.5296	932.681	121.738
46232	6.5297	761.847	250.032
46233	6.5298	985.431	58.6459
46234	6.5301	983.624	158.656
46235	6.5303	1108.65	4.9089
46236	6.5304	1034.02	14.1889
46237	6.5306	935.447	220.86
46238	6.5319	986.723	101.267
46239	6.5326	1207.6801	34.769
46240	6.5328	859.37	59.518
46241	6.5333	942.168	13.2296
46242	6.5334	1023.23	243.036
46243	6.5337	1012.99	133.782
46244	6.5338	989.354	100.039
46245	6.5344	639.744	327.705
46246	6.5345	955.762	27.5568
46247	6.5345	1068.1801	151.514
46248	6.5347	957.514	317.781
46249	6.535	1030.01	99.2995
46250	6.535	1051.85	111.322
46251	6.5353	1152.24	35.6057
46252	6.5359	1020.59	111.161
46253	6.5359	927.045	73.5035
46254	6.5361	882.312	154.534
46255	6.5363	935.541	248.109
46256	6.5366	966.819	158.152
46257	6.5372	814.524	242.795
46258	6.5374	836.295	173.137
46259	6.5375	1006.8	19.1455
46260	6.5377	913.07	158.842
46261	6.5377	911.153	27.0453

46262	6.5384	1054.74	100.04
46263	6.5385	870.82	68.5872
46264	6.5386	1023.56	160.288
46265	6.5391	986.241	90.2468
46266	6.5391	767.388	221.567
46267	6.5392	1084.36	238.866
46268	6.5393	729.185	196.567
46269	6.5398	985.253	3.2212
46270	6.5401	738.5	99.4896
46271	6.5401	952.362	5.0225
46272	6.5401	1144.41	42.7293
46273	6.5415	1002.11	93.8064
46274	6.5416	1033.39	55.7649
46275	6.5419	845.103	253.267
46276	6.5422	1071.85	38.9841
46277	6.5428	973.614	135.848
46278	6.5429	829.281	176.98
46279	6.543	790.81	219.046
46280	6.5431	1100.66	23.7457
46281	6.5431	843.512	314.241
46282	6.5435	988.237	124.589
46283	6.5435	930.059	150.713
46284	6.5443	943.168	198.761
46285	6.5445	925.612	65.2191
46286	6.5456	981.609	89.8802
46287	6.5456	932.69	21.5752
46288	6.5459	984.948	28.9962
46289	6.5461	923.532	316.719
46290	6.5463	992.607	85.7491
46291	6.5464	1122.86	109.029
46292	6.5465	1030.42	150.627
46293	6.5467	883.441	14.713
46294	6.5467	1015.94	51.5802
46295	6.5468	817.683	187.007
46296	6.5469	894.557	111.997
46297	6.5474	1075.6	241.584
46298	6.5476	758.521	180.472
46299	6.5477	809.24	166.314
46300	6.5478	826.3	251.566
46301	6.548	950.026	291.737
46302	6.5481	869.341	219.933
46303	6.5489	1099.84	88.1346
46304	6.549	941.32	217.969
46305	6.5493	873.184	55.4349
46306	6.5503	947.875	61.5772
46307	6.5505	893.26	250.045
46308	6.5507	968.953	192.404
46309	6.5519	1018.43	65.2512

46310	6.5525	1135.36	23.8214
46311	6.5525	840.718	322.543
46312	6.5525	945.42	162.846
46313	6.5525	977.415	242.767
46314	6.5525	888.336	295.329
46315	6.5525	1077.03	24.4475
46316	6.5527	956.546	108.293
46317	6.5527	1114.13	238.305
46318	6.554	776.047	244.481
46319	6.5542	1112.4301	72.6267
46320	6.5543	889.124	257.074
46321	6.5547	875.11	238.31
46322	6.5554	610.822	116.831
46323	6.5555	1014.25	94.8392
46324	6.5561	810.264	167.289
46325	6.5569	976.939	103.702
46326	6.5572	913.316	159.345
46327	6.5572	946.362	18.2659
46328	6.5572	1109.58	237.512
46329	6.5575	964.101	289.788
46330	6.5576	1110.9	191.828
46331	6.5586	932.187	75.7178
46332	6.5587	1186.45	54.5232
46333	6.5588	1032.74	169.594
46334	6.5593	957.065	93.8717
46335	6.5594	952.068	164.474
46336	6.5597	850.025	185.757
46337	6.5598	891.424	154.427
46338	6.5605	976.794	157.509
46339	6.5607	850.325	301.598
46340	6.5616	851.94	140.779
46341	6.5617	800.92	149.654
46342	6.5618	927.595	154.141
46343	6.562	1045.17	13.5008
46344	6.5622	779.44	240.993
46345	6.5623	868.931	144.888
46346	6.5627	920.972	142.933
46347	6.563	777.861	188.528
46348	6.563	916.069	42.4664
46349	6.5631	920.403	99.2402
46350	6.5632	717.613	166.089
46351	6.5632	1089.6899	115.182
46352	6.5632	965.172	177.112
46353	6.5633	899.039	189.22
46354	6.5634	1032.72	271.23
46355	6.5637	853.212	240.526
46356	6.5638	1066.92	5.3205
46357	6.5638	1009.2	25.1533

46358	6.5639	907.191	308.261
46359	6.5644	843.213	167.446
46360	6.5647	1079.39	95.479
46361	6.5656	1029.0699	101.957
46362	6.5658	877.332	51.4501
46363	6.5665	996.949	197.726
46364	6.5667	1050.92	28.2726
46365	6.5667	983.97	200.138
46366	6.5676	795.858	176.239
46367	6.5677	1070.73	255.194
46368	6.568	976.207	83.6993
46369	6.5684	949.124	294.096
46370	6.5685	856.08	155.797
46371	6.5685	1000.25	62.011
46372	6.5686	991.07	98.247
46373	6.569	860.452	174.679
46374	6.569	915.22	101.359
46375	6.5692	863.768	244.028
46376	6.5693	955.321	248.637
46377	6.5693	935.128	9.1253
46378	6.5695	1046.59	48.0468
46379	6.57	973.303	130.732
46380	6.5701	999.36	298.899
46381	6.5704	1090.13	325.267
46382	6.5707	-5.2601	53.563
46383	6.5708	1065.13	111.213
46384	6.5708	1001.21	196.704
46385	6.5712	887.126	97.9129
46386	6.5714	1009.17	101.365
46387	6.5715	882.464	167.921
46388	6.5718	904.357	166.786
46389	6.5722	563.326	49.2017
46390	6.5726	928.01	147.973
46391	6.5731	859.77	237.977
46392	6.5733	947.688	85.9836
46393	6.5734	946.337	260.454
46394	6.5734	963.694	171.792
46395	6.5736	1113.42	25.0827
46396	6.5736	820.626	242.875
46397	6.5743	795.555	162.865
46398	6.5744	924.721	121.113
46399	6.5745	849.483	115.698
46400	6.5754	897.78	248.533
46401	6.5756	725.104	57.445
46402	6.5758	1068.23	11.9491
46403	6.5762	976.508	87.7967
46404	6.5763	895.687	243.936
46405	6.5765	939.634	230.201

46406	6.5765	963.785	324.682
46407	6.5767	885.667	174.83
46408	6.5773	1011.07	138.371
46409	6.5774	825.835	261.046
46410	6.5774	794.271	183.295
46411	6.5775	964.204	21.8805
46412	6.5778	943.988	246.958
46413	6.5778	881.492	261.354
46414	6.5781	908.305	164.157
46415	6.5797	892.898	284.104
46416	6.5802	994.862	165.411
46417	6.5803	1067.64	25.0243
46418	6.5803	1052.76	270.909
46419	6.5806	1114.41	32.2774
46420	6.5807	936.943	39.2694
46421	6.5807	1137.9399	14.6405
46422	6.5808	976.234	149.847
46423	6.5815	962.517	137.098
46424	6.582	857.808	219.807
46425	6.5822	951.147	142.024
46426	6.5829	840.729	243.584
46427	6.5831	978.18	173.834
46428	6.5831	1131.46	61.908
46429	6.5833	1033.74	16.0434
46430	6.5833	864.317	239.738
46431	6.5836	1072.04	277.328
46432	6.5837	1001.97	57.2527
46433	6.5838	973.264	106.539
46434	6.5843	938.998	249.044
46435	6.5844	1037.21	89.5571
46436	6.5847	969.768	20.5378
46437	6.5848	943.347	78.7548
46438	6.585	773.677	175.843
46439	6.5853	1062.55	35.1054
46440	6.5854	1015.95	106.692
46441	6.586	1028.4399	196.121
46442	6.5862	1098.71	26.8028
46443	6.5862	967.909	244.244
46444	6.5866	1131.3101	14.3679
46445	6.5867	1132.42	48.7121
46446	6.587	790.796	150.082
46447	6.5872	984.477	283.734
46448	6.5874	870.052	254.286
46449	6.5877	834.645	98.1444
46450	6.5884	985.619	27.6085
46451	6.5885	854.236	228.859
46452	6.589	830.137	187.129
46453	6.589	1064.87	34.0067

46454	6.5892	954.423	54.7816
46455	6.5895	997.149	107.115
46456	6.5898	942.59	191.874
46457	6.5899	981.487	29.9046
46458	6.5905	921.28	26.0338
46459	6.5914	988.05	94.7992
46460	6.5919	832.146	234.999
46461	6.592	924.523	137.376
46462	6.5924	850.916	239.043
46463	6.5932	984.429	102.954
46464	6.5933	918.657	23.5984
46465	6.5934	1055.86	102.995
46466	6.5938	988.683	18.5202
46467	6.5941	900.814	239.104
46468	6.5945	1112.79	52.0532
46469	6.595	974.784	18.4445
46470	6.5958	1174.97	258.096
46471	6.5958	890.835	261.782
46472	6.596	944.012	167.84
46473	6.5963	917.96	45.0987
46474	6.5967	998.566	75.217
46475	6.5969	969.318	91.8644
46476	6.597	1035.41	266.575
46477	6.5971	1033.97	31.5124
46478	6.5973	747.912	169.844
46479	6.5977	1016.79	47.6129
46480	6.5978	926.264	91.5843
46481	6.5981	1009.44	41.0489
46482	6.5984	965.062	248.054
46483	6.5992	840.723	68.9789
46484	6.5994	872.227	111.036
46485	6.5997	1132.16	50.2192
46486	6.5999	940.033	318.596
46487	6.6	990.991	95.5019
46488	6.6002	1082.5601	22.7663
46489	6.6002	806.56	27.1095
46490	6.601	1046.2	78.862
46491	6.6013	851.948	238.963
46492	6.6016	939.229	278.845
46493	6.6022	937.724	139.31
46494	6.6026	892.87	277.288
46495	6.6027	923.621	159.446
46496	6.6028	946.361	8.731
46497	6.6031	1041.8	99.5861
46498	6.6032	990.835	303.573
46499	6.6035	1091.72	99.5978
46500	6.6036	933.518	283.35
46501	6.6037	1004.62	30.8493

46502	6.6038	1094.33	322.867
46503	6.6039	926.885	172.213
46504	6.6042	1049.64	141.725
46505	6.6044	969.158	83.6337
46506	6.6047	982.448	147.397
46507	6.6052	999.549	263.063
46508	6.6053	924.658	76.2096
46509	6.6058	1119.1	61.5032
46510	6.6058	870.791	232.641
46511	6.6059	889.188	221.935
46512	6.606	975.985	234.035
46513	6.6063	894.998	125.826
46514	6.6064	861.398	96.0331
46515	6.6065	1052.04	73.3713
46516	6.607	1045.92	46.2464
46517	6.6071	901.767	91.1669
46518	6.6072	1021.32	139.921
46519	6.6072	981.823	99.7883
46520	6.6078	944.188	302.324
46521	6.6079	1008.39	266.324
46522	6.608	810.952	24.1756
46523	6.6083	841.68	219.16
46524	6.6086	892.7	56.0805
46525	6.6088	1074.8199	93.8481
46526	6.6088	1024.1899	33.4218
46527	6.6089	1123.05	28.5406
46528	6.6093	1073.3199	76.6153
46529	6.6098	945.621	121.617
46530	6.61	866.154	60.3039
46531	6.6102	1081.12	57.2645
46532	6.6104	888.839	137.327
46533	6.6115	1097.02	107.948
46534	6.6116	788.725	151.66
46535	6.6119	1019.57	169.145
46536	6.6119	1055.05	92.928
46537	6.6121	865.186	246.748
46538	6.6127	698.926	79.4514
46539	6.6128	1119.83	114.73
46540	6.6128	908.388	14.1906
46541	6.6135	1042.1801	78.4979
46542	6.614	716.134	195.711
46543	6.6143	979.648	191.754
46544	6.6143	1095.73	53.3036
46545	6.6144	1032.99	259.031
46546	6.6144	264.403	199.677
46547	6.6146	1185.77	17.031
46548	6.6153	973.274	286.142
46549	6.6154	999.697	152.015

46550	6.6165	1004.06	248.094
46551	6.6165	870.574	231.696
46552	6.6165	901.355	111.918
46553	6.6165	1038.72	130.881
46554	6.6167	965.55	259.837
46555	6.6169	921.955	248.831
46556	6.617	856.226	173.592
46557	6.6172	1159.42	283.409
46558	6.6174	1088.6	160.415
46559	6.6175	934.333	185.264
46560	6.6176	972.291	85.8921
46561	6.6185	986.055	136.619
46562	6.6187	1129.85	31.4623
46563	6.6189	794.505	225.24
46564	6.6189	974.188	118.43
46565	6.6189	924.452	198.455
46566	6.6191	916.423	332.803
46567	6.6195	921.175	189.117
46568	6.6203	1062.05	35.6008
46569	6.6204	875.141	251.206
46570	6.6204	933.78	162.076
46571	6.6205	1008.62	94.5725
46572	6.6208	1040.1899	70.7504
46573	6.6213	1006.49	297.428
46574	6.6215	1021.32	127.755
46575	6.6216	901.142	24.9633
46576	6.6219	1207.9	25.5224
46577	6.6219	1020.83	119.33
46578	6.6223	1021.1	141.413
46579	6.6227	847.117	235.245
46580	6.6229	933.487	111.927
46581	6.6229	1173.95	80.1505
46582	6.6236	1117.75	113.667
46583	6.6239	1043	21.5538
46584	6.6244	997.612	65.3301
46585	6.6244	1129.9399	20.6912
46586	6.625	1095.05	109.646
46587	6.6254	1080.29	79.4646
46588	6.6258	1147.58	16.6267
46589	6.626	907.716	51.3373
46590	6.6263	690.572	117.75
46591	6.6264	1012.71	125.726
46592	6.6264	1092.84	50.724
46593	6.6267	869.349	248.39
46594	6.6268	833.228	183.468
46595	6.6269	941.486	221.556
46596	6.6273	899.711	157.302
46597	6.6276	881.468	220.276

46598	6.6276	982.816	154.953
46599	6.6276	929.42	75.4483
46600	6.6277	1046.26	190.271
46601	6.6279	867.681	193.157
46602	6.628	984.51	102.319
46603	6.6281	990.434	104.074
46604	6.6282	877.868	117.509
46605	6.6283	1071.61	103.221
46606	6.6283	962.915	160.934
46607	6.6288	959.318	177.637
46608	6.6289	1068.16	126.934
46609	6.6291	931.96	119.293
46610	6.6293	986.67	343.118
46611	6.6294	1109.6	44.7948
46612	6.6295	933.637	31.4391
46613	6.6297	916.597	251.972
46614	6.6297	901.202	200.709
46615	6.6301	811.549	189.16
46616	6.6301	933.353	49.0329
46617	6.6303	905.124	18.1195
46618	6.631	204.811	253.075
46619	6.6312	929.057	193.92
46620	6.6322	929.722	34.0262
46621	6.6324	1041.61	23.5199
46622	6.6324	1134.4399	5.2259
46623	6.6325	1017.16	145.293
46624	6.6326	941.117	85.0829
46625	6.6332	1065.62	219.947
46626	6.6333	1026.16	191.29
46627	6.6334	976.748	159.102
46628	6.6337	980.627	67.8999
46629	6.634	1108.99	102.343
46630	6.6346	880.731	167.25
46631	6.6349	841.516	184.036
46632	6.6353	1171.08	54.9366
46633	6.6356	882.94	164.133
46634	6.6357	1080.41	36.4552
46635	6.637	909.78	152.008
46636	6.6371	911.433	114.465
46637	6.6376	1226.1899	21.9445
46638	6.6378	938.409	159.923
46639	6.6378	965.616	106.784
46640	6.6384	1037.3199	80.0689
46641	6.6387	795.301	171.438
46642	6.6388	939.608	170.785
46643	6.6389	931.985	117.718
46644	6.6394	1067.02	104.802
46645	6.6398	963.843	112.602

46646	6.6402	1106.25	181.1
46647	6.6404	993.986	62.7831
46648	6.6405	930.918	64.6713
46649	6.6405	1073.47	233.416
46650	6.6406	896.6	109.444
46651	6.6412	855.603	342.742
46652	6.6412	890.569	197.39
46653	6.6415	1098.42	159.362
46654	6.6418	1016.19	187.97
46655	6.6421	886.12	218.245
46656	6.6422	835.145	221.142
46657	6.6426	1184.35	49.6603
46658	6.6428	835.062	226.742
46659	6.6432	987.028	178.195
46660	6.6432	889.319	24.3329
46661	6.6433	1011.07	207.555
46662	6.6434	1081.71	261.212
46663	6.6436	908.572	87.4469
46664	6.6438	775	148.507
46665	6.6442	866.163	237.43
46666	6.6442	1031.83	101.794
46667	6.6443	1043.65	169.336
46668	6.6444	1036.7	358.461
46669	6.6452	766.811	148.786
46670	6.6456	777.153	162.653
46671	6.6457	930.858	120.668
46672	6.6463	1052.91	230.07
46673	6.6466	757.705	160.729
46674	6.6466	1048.8199	110.324
46675	6.6468	1018.05	324.007
46676	6.6469	937.099	325.964
46677	6.6473	1013.63	61.7303
46678	6.6474	1094.78	88.6148
46679	6.6475	977.167	283.531
46680	6.649	940.097	300.177
46681	6.6491	1022.06	101.758
46682	6.6491	832.381	136.702
46683	6.6498	966.226	157.569
46684	6.6498	947.35	136.345
46685	6.6505	1056.85	30.7788
46686	6.6507	1102.53	223.24
46687	6.6508	924.861	165.293
46688	6.6516	869.798	241.534
46689	6.652	1058.27	191.119
46690	6.6521	889.293	171.644
46691	6.6525	1027.36	166.994
46692	6.6527	845.141	90.5824
46693	6.6527	926.54	169.991

46694	6.6529	1083.86	113.027
46695	6.653	1057.3	82.9641
46696	6.654	1018.89	121.715
46697	6.6544	1023.18	103.032
46698	6.6549	1056.99	111.498
46699	6.655	849.357	147.883
46700	6.6565	985.258	50.8216
46701	6.6567	1119.75	78.6733
46702	6.6568	1089.75	189.348
46703	6.6573	946.676	301.138
46704	6.6574	1077.46	76.6552
46705	6.6578	860.528	220.016
46706	6.6579	880.951	192.302
46707	6.6583	1088.6801	20.0602
46708	6.6588	997.763	79.1817
46709	6.6595	1124.96	18.0353
46710	6.6596	1042.91	303.167
46711	6.6598	1036.63	97.0963
46712	6.6598	1112.15	112.905
46713	6.6599	916.332	297.937
46714	6.6601	1077.0601	76.5682
46715	6.6603	965.372	159.262
46716	6.6604	951.016	224.202
46717	6.661	875.654	170.404
46718	6.6611	927.023	202.479
46719	6.6612	928.898	173.904
46720	6.6612	1001.59	228.87
46721	6.6613	931.922	118.455
46722	6.6618	1138.98	21.7472
46723	6.6629	1139.62	28.1418
46724	6.6633	1133.08	1.0432
46725	6.6635	1035.6899	304.736
46726	6.6637	973.179	124.722
46727	6.6638	963.359	100.386
46728	6.6642	1049.26	150.61
46729	6.6644	872.389	105.219
46730	6.6646	1005.41	100.792
46731	6.665	1050.39	32.0015
46732	6.665	1006.52	298.318
46733	6.6653	1131.73	94.0308
46734	6.6655	893.813	231.919
46735	6.6656	986.97	291.665
46736	6.6656	853.597	161.687
46737	6.6662	743.044	165.089
46738	6.6664	925.455	127.184
46739	6.667	944.903	177.407
46740	6.6673	985.62	70.8563
46741	6.6673	953.876	226.459

46742	6.6674	956.817	28.036
46743	6.6674	874.441	194.452
46744	6.6678	945.404	203.01
46745	6.6679	871.419	179.237
46746	6.6681	824.882	220.196
46747	6.6683	1034.71	78.0621
46748	6.6686	842.602	292.429
46749	6.6687	1100.97	19.1651
46750	6.6688	926.645	153.989
46751	6.6688	992.162	305.605
46752	6.6691	898.23	136.761
46753	6.6692	923.208	233.798
46754	6.6693	1145.3	108.718
46755	6.6694	858.997	111.138
46756	6.6695	883.314	234.844
46757	6.6697	1168.33	47.0103
46758	6.6697	908.54	96.8466
46759	6.6699	946.221	161.51
46760	6.67	978.254	229.383
46761	6.6702	795.403	149.612
46762	6.6704	1129.89	255.726
46763	6.6705	968.159	248.921
46764	6.671	958.134	229.442
46765	6.6711	953.017	172.989
46766	6.6711	999.598	225.632
46767	6.6717	1022.28	99.8251
46768	6.6726	1052.87	85.7672
46769	6.6738	264.287	222.029
46770	6.6743	841.906	256.932
46771	6.6743	957.24	55.0379
46772	6.6744	1048.52	109.029
46773	6.6746	960.158	83.7827
46774	6.6748	976.528	180.319
46775	6.6749	1045.17	65.6262
46776	6.6751	807.105	223.087
46777	6.6754	1051.03	224.711
46778	6.6759	973.611	192.576
46779	6.6765	1178.17	240.002
46780	6.6767	1043.09	40.8536
46781	6.6775	1163.49	22.4128
46782	6.6783	1106.13	320.013
46783	6.6784	943.901	1.7318
46784	6.6787	1108.55	131.214
46785	6.6792	1028.5601	113.322
46786	6.6793	1094.45	83.7541
46787	6.6795	1016.3	76.9949
46788	6.6803	1008.75	94.4789
46789	6.6809	1046.46	354.972

46790	6.6813	1187.28	260.034
46791	6.6813	933.926	156.8
46792	6.6821	1050.88	28.5291
46793	6.6824	1172.99	15.1504
46794	6.6827	878.802	185.561
46795	6.6829	1109.5	252.501
46796	6.683	977.797	192.593
46797	6.6831	886.815	19.3425
46798	6.6831	979.233	133.051
46799	6.6835	978.734	257.725
46800	6.6835	873.119	164.952
46801	6.6838	1063.37	22.9515
46802	6.6841	1077.11	82.1082
46803	6.6843	1039.8101	186.826
46804	6.6844	907.381	116.833
46805	6.6853	1137.3199	22.8338
46806	6.6855	977.014	324.86
46807	6.6864	1103.03	34.9333
46808	6.687	1119.87	30.2478
46809	6.6873	1111.76	7.9782
46810	6.6876	-2.9709	327.839
46811	6.6877	1123.99	20.3874
46812	6.6881	1114.87	334.865
46813	6.6883	961.311	359.586
46814	6.6884	1203.21	257.473
46815	6.6885	1020.51	153.182
46816	6.6889	1023.67	73.0018
46817	6.6889	1057.49	89.9395
46818	6.689	1026.87	297.427
46819	6.689	967.775	27.22
46820	6.6895	1024.8199	107.334
46821	6.6896	733.06	154.198
46822	6.6898	864.692	31.7729
46823	6.6901	1097.95	57.972
46824	6.6906	1157.84	189.379
46825	6.6908	1107.34	41.7473
46826	6.6913	923.214	167.841
46827	6.6914	973.48	28.6839
46828	6.6919	962.79	259.504
46829	6.692	1061.01	90.4322
46830	6.6925	948.009	179.126
46831	6.6926	967.262	58.9961
46832	6.6926	860.603	182.194
46833	6.6926	1210.78	13.2273
46834	6.6928	934.096	241.383
46835	6.6929	921.923	334.189
46836	6.6942	925.342	112.008
46837	6.6946	932.84	250.035

46838	6.6958	974.429	171.28
46839	6.6964	809.334	239.599
46840	6.6976	1016.09	175.545
46841	6.6977	1092.67	95.9821
46842	6.698	923.24	135.171
46843	6.6981	972.417	28.3446
46844	6.6983	998.818	212.796
46845	6.6983	874.889	230.527
46846	6.6983	940.183	88.3453
46847	6.6983	1020.92	93.1626
46848	6.6987	1084.63	88.8318
46849	6.6991	1075.4	84.8012
46850	6.7	1059.27	90.5116
46851	6.7003	-2.9397	329.325
46852	6.7004	957.215	65.6214
46853	6.7008	686.343	67.1606
46854	6.701	1027.55	56.0028
46855	6.7012	1107.9301	259.128
46856	6.7013	1035.97	152.941
46857	6.7013	900.879	260.179
46858	6.7014	1166.92	31.2478
46859	6.7018	1108.3101	33.0021
46860	6.7022	1096.95	106.348
46861	6.7025	957.317	259.973
46862	6.7029	859.676	73.7138
46863	6.7031	883.574	170.674
46864	6.7031	924.869	279.873
46865	6.7035	956.359	61.8275
46866	6.7036	899.857	192.47
46867	6.7039	1066.6899	96.515
46868	6.704	1042.5601	172.245
46869	6.704	1203.61	56.6146
46870	6.7046	1062.74	85.1783
46871	6.7048	1147.72	27.2955
46872	6.7053	111.65	23.9127
46873	6.7059	1000.13	13.584
46874	6.7059	1163.59	281.368
46875	6.706	928.19	297.726
46876	6.7063	1104.8101	14.6069
46877	6.7064	1225.13	26.1946
46878	6.7073	1126.1899	25.9909
46879	6.7075	1232.1	31.8656
46880	6.7075	914.533	179.326
46881	6.7076	1219.16	25.008
46882	6.7077	954.594	138.761
46883	6.7078	1016.75	331.418
46884	6.7081	993.754	139.303
46885	6.7087	947.521	44.198

46886	6.7094	928.641	325.778
46887	6.7094	1113.9301	92.6451
46888	6.7096	936.888	71.4106
46889	6.7102	991.265	117.389
46890	6.7104	904.776	252.729
46891	6.7107	952.025	226.915
46892	6.7107	996.624	225.866
46893	6.7108	992.258	155.585
46894	6.7109	1023.61	77.3588
46895	6.711	932.617	249.343
46896	6.711	1060.46	109.724
46897	6.7114	952.882	163.094
46898	6.7114	1048.84	14.0916
46899	6.7118	1185.58	102.586
46900	6.7118	919.17	251.751
46901	6.712	988.848	168.944
46902	6.7123	978.498	179.628
46903	6.7127	1060.86	31.4513
46904	6.7135	943.192	292.981
46905	6.714	1137.47	1.1305
46906	6.714	895.944	180.741
46907	6.7141	903.644	146.682
46908	6.7143	1127.2	141.645
46909	6.7144	974.317	167.197
46910	6.7144	992.951	91.387
46911	6.7146	1040.3101	59.6343
46912	6.715	1156.64	137.689
46913	6.715	1048.87	53.98
46914	6.7151	831.024	317.327
46915	6.7163	1010.51	124.323
46916	6.7168	1134.7	19.3592
46917	6.7171	1043.97	175.648
46918	6.7175	1121.61	354.268
46919	6.7175	983.644	162.715
46920	6.7177	482.934	353.23
46921	6.7179	1087.62	62.5098
46922	6.7179	1070.77	188.118
46923	6.718	995.868	249.193
46924	6.7183	1031.26	179.716
46925	6.7185	870.451	74.2008
46926	6.7187	1081.14	154.542
46927	6.7188	1088.48	3.6688
46928	6.7188	984.498	52.5575
46929	6.719	1080.22	25.5899
46930	6.7194	941.918	229.141
46931	6.7194	976.189	159.122
46932	6.7194	1225.48	22.3017
46933	6.7195	856.834	214.652

46934	6.7196	1166.13	15.2767
46935	6.7198	1000.21	90.68
46936	6.7199	815.236	161.236
46937	6.7201	1082	164.639
46938	6.7204	976.009	223.765
46939	6.721	-2.8201	268.812
46940	6.7212	1212.03	45.7036
46941	6.7214	1030.48	111.447
46942	6.7215	1014.02	113.955
46943	6.7224	1314.59	83.3238
46944	6.7225	969.002	89.2761
46945	6.7228	1210.02	20.9528
46946	6.7232	813.275	189.187
46947	6.7233	886.377	85.6543
46948	6.7237	877.172	182.353
46949	6.7241	1063.88	229.419
46950	6.7241	941.431	214.363
46951	6.7243	1162.17	51.7549
46952	6.7253	1094.62	204.831
46953	6.7254	909.433	257.085
46954	6.7256	992.898	252.813
46955	6.7257	949.934	135.665
46956	6.726	1093.3	74.576
46957	6.7261	1047.5699	259.444
46958	6.7265	910.449	165.825
46959	6.7266	978.823	172.1
46960	6.7271	936.952	305.659
46961	6.7275	938.953	195.718
46962	6.7277	1236.36	19.7406
46963	6.7278	986.874	113.299
46964	6.7279	1103.95	39.6344
46965	6.7281	929.523	157.485
46966	6.7282	1240.17	23.3106
46967	6.7288	995.595	123.669
46968	6.729	1106.03	139.648
46969	6.7291	1169.89	32.4058
46970	6.7292	1028.3	78.3312
46971	6.7293	975.91	180.229
46972	6.7293	988.63	106.595
46973	6.7297	1090.0601	77.3056
46974	6.7297	1013.32	74.9096
46975	6.7298	1164.75	66.3787
46976	6.7298	967.191	186.784
46977	6.7302	1013.62	109.838
46978	6.7309	942.756	86.8453
46979	6.7311	977.492	23.1246
46980	6.7314	1174.55	93.4717
46981	6.7315	1047.61	71.4054

46982	6.7315	838.401	284.511
46983	6.7317	649.895	90.7562
46984	6.7319	1149.6801	0.8213
46985	6.7319	965.292	331.331
46986	6.7319	1111.91	189.163
46987	6.732	930.652	168.459
46988	6.7321	1215.39	87.5125
46989	6.7324	1090.1	96.5562
46990	6.7325	1101.23	140.248
46991	6.7325	947.854	23.8433
46992	6.733	1206.4301	24.2822
46993	6.733	1127.24	25.3086
46994	6.7335	1115.9301	7.3098
46995	6.7339	952.914	240.809
46996	6.734	681.35	61.6597
46997	6.7344	926.92	196.906
46998	6.7347	1011.71	188.644
46999	6.7348	1178.4399	98.9828
47000	6.7348	924.707	75.8685
47001	6.7352	1151.78	21.4525
47002	6.7353	1079.67	190.49
47003	6.7355	-2.9593	325.505
47004	6.7357	781.14	178.284
47005	6.7358	1079.76	301.726
47006	6.736	1078.78	16.4458
47007	6.7361	962.978	2.8601
47008	6.7362	962.681	314.406
47009	6.7364	1049.85	23.9619
47010	6.7367	935.33	166.365
47011	6.7368	1251.16	33.5016
47012	6.737	1048.09	73.4498
47013	6.7372	1018.89	82.7189
47014	6.7377	1026.97	64.1622
47015	6.7379	939.044	215.262
47016	6.7379	1104.3199	5.9471
47017	6.7382	1083.51	84.5874
47018	6.7383	1193.1801	101.94
47019	6.739	994.315	135.933
47020	6.7391	983.747	244.492
47021	6.7395	906.549	179.738
47022	6.74	701.95	160.924
47023	6.7404	473.773	325.831
47024	6.7406	773.505	195.922
47025	6.7409	970.655	116.625
47026	6.741	-4.1136	265.278
47027	6.7421	1252.5699	113.893
47028	6.7423	1014.42	276.629
47029	6.7425	1084.75	150.035

47030	6.7429	1021.57	229.898
47031	6.743	1210.23	6.9408
47032	6.7432	935.145	257.283
47033	6.7432	1094.3	16.3133
47034	6.7433	966.409	91.3394
47035	6.7434	1037.17	74.8242
47036	6.744	1052.77	95.8532
47037	6.7442	886.992	165.687
47038	6.7442	1221.55	342.595
47039	6.7446	1150.1899	55.1508
47040	6.7448	943.541	102.092
47041	6.7449	914.64	260.385
47042	6.746	815.753	156.328
47043	6.746	1060.75	54.0032
47044	6.7463	1030.75	50.6549
47045	6.7463	1052.37	276.231
47046	6.7465	1099.6	39.983
47047	6.7466	1080.96	31.0192
47048	6.7471	1017.12	63.754
47049	6.7478	1065.62	93.8633
47050	6.7478	1003.92	98.813
47051	6.7482	946.591	256.116
47052	6.7484	1122.24	53.0796
47053	6.7487	1187.98	34.4153
47054	6.7488	964.215	125.991
47055	6.7496	1311.9301	40.4468
47056	6.7501	873.514	293
47057	6.7501	992.616	151.136
47058	6.7504	717.921	159.494
47059	6.7504	980.967	203.707
47060	6.7509	1066.3	187.873
47061	6.7514	1001.33	199.827
47062	6.7517	959.652	113.659
47063	6.7519	1097.84	74.1998
47064	6.7526	1001.22	24.0645
47065	6.7533	927.867	14.7682
47066	6.7534	1050.23	98.6563
47067	6.7535	924.865	220.955
47068	6.754	1206.35	7.5128
47069	6.754	1188.47	38.8175
47070	6.7541	1010.75	156.81
47071	6.7543	1183.76	25.4938
47072	6.7549	1073.15	81.1473
47073	6.7551	916.837	102.372
47074	6.7552	905.293	179.178
47075	6.7554	898.749	283.456
47076	6.7554	1014.15	35.8931
47077	6.7555	1044.12	181.905

47078	6.7558	954.82	271.52
47079	6.7559	999.881	250.849
47080	6.7559	994.418	96.5806
47081	6.7562	1065.13	83.2558
47082	6.7565	1088.84	51.1996
47083	6.757	1178.88	12.9226
47084	6.7573	1318.79	41.6127
47085	6.7579	937.9	141.653
47086	6.7585	1152.73	204.343
47087	6.7589	1104.76	232.369
47088	6.7593	989.185	80.41
47089	6.7594	1145.96	240.639
47090	6.7595	903.19	140.851
47091	6.76	1115.02	15.9406
47092	6.7604	920.23	42.711
47093	6.7607	1102.54	190.715
47094	6.7608	1202.8	28.783
47095	6.7617	1048.99	29.2963
47096	6.7621	1067.76	82.2723
47097	6.7622	883.473	183.992
47098	6.7623	893.238	166.641
47099	6.7624	942.891	175.163
47100	6.7639	1043.12	297.78
47101	6.7642	1094.49	3.5916
47102	6.7645	1050.49	221.284
47103	6.7645	1033.9399	111.717
47104	6.7646	1076.3	319.812
47105	6.7651	1055.6801	222.274
47106	6.7656	965.41	161.234
47107	6.7661	937.392	251.86
47108	6.7663	974.742	119.42
47109	6.7665	1031.23	170.76
47110	6.7665	999.227	111.867
47111	6.7666	1038.71	87.6698
47112	6.7668	1054.0699	243.117
47113	6.7671	938.837	337.627
47114	6.7673	1211.02	342.523
47115	6.7674	991.888	150.168
47116	6.7674	1065.34	36.7429
47117	6.7677	909.37	243.37
47118	6.7682	789.944	251.823
47119	6.7685	955.033	96.1558
47120	6.7685	954.048	114.146
47121	6.7688	991.868	124.689
47122	6.7696	1035.41	83.729
47123	6.7702	1130.7	15.1063
47124	6.7702	1214.0601	14.2531
47125	6.7704	1066.16	261.399

47126	6.7706	1215.21	80.565
47127	6.7709	887.497	145.086
47128	6.771	1066.13	47.7284
47129	6.7711	1011.06	64.7624
47130	6.7715	972.516	249.28
47131	6.7716	1053.77	352.543
47132	6.7716	1069.99	32.3488
47133	6.7718	990.408	150.521
47134	6.7718	729.291	153.039
47135	6.7719	1206.96	21.132
47136	6.772	1061.9301	306.502
47137	6.772	867.582	129.678
47138	6.7723	1111.78	303.202
47139	6.7729	1294.99	26.1589
47140	6.7733	1059.03	112.967
47141	6.7736	1009.74	25.7297
47142	6.7737	1136.23	135.654
47143	6.774	887.729	245.381
47144	6.7745	967.208	51.1843
47145	6.7748	1161.96	253.478
47146	6.7758	1059.73	95.6288
47147	6.7764	1240.5601	33.0036
47148	6.7773	1139.26	86.4646
47149	6.7775	1269.88	288.088
47150	6.7775	986.379	165.186
47151	6.7778	1229.3199	78.855
47152	6.7781	1144.66	42.5134
47153	6.7784	1188.26	27.8304
47154	6.7788	1027.36	23.9387
47155	6.7789	1048.62	30.5597
47156	6.7795	943.669	291.596
47157	6.7796	1005.56	298.115
47158	6.78	1131.41	128.444
47159	6.7805	959.498	201.67
47160	6.7805	1050.24	87.9616
47161	6.7806	1109.23	277.564
47162	6.7809	1054.8101	11.3109
47163	6.781	1133.88	22.4499
47164	6.7815	886.483	192.476
47165	6.7818	1254.0699	21.7209
47166	6.782	1043.99	120.176
47167	6.7822	1195.75	50.6869
47168	6.7823	1004.89	146.19
47169	6.7823	1002.34	55.1642
47170	6.7828	994.677	302.307
47171	6.783	946.872	165.638
47172	6.7832	1155.87	94.4448
47173	6.7835	1051.91	7.4184

47174	6.7839	1262.36	288.616
47175	6.784	1088.34	103.81
47176	6.7853	1042.65	99.4882
47177	6.7856	1071.74	77.1772
47178	6.7859	874.586	253.331
47179	6.7862	1093.5	66.4469
47180	6.7866	982.508	300.321
47181	6.7868	987.351	110.553
47182	6.7869	790.76	93.3564
47183	6.7874	1279.64	48.29
47184	6.7878	1074.42	262.77
47185	6.7879	1150.64	33.2443
47186	6.7882	1065.0601	68.4308
47187	6.7883	1185.97	18.2058
47188	6.7888	939.572	3.3193
47189	6.7898	1111.41	259.279
47190	6.7904	1230.21	27.5484
47191	6.7914	881.148	183.235
47192	6.7916	889.209	237.979
47193	6.7916	1062.36	67.0579
47194	6.7917	1016.45	232.082
47195	6.7922	1121.71	123.851
47196	6.7925	968.029	83.4638
47197	6.7933	958.968	291.784
47198	6.794	1034.4301	81.4483
47199	6.7941	1101.0699	186.978
47200	6.7946	1035.33	76.171
47201	6.7947	1012.24	117.624
47202	6.7947	1078.74	32.5931
47203	6.7949	936.03	236.071
47204	6.7949	982.684	85.7785
47205	6.7952	1166.7	87.4933
47206	6.7958	1118.49	3.2724
47207	6.7958	1062.35	118.478
47208	6.7963	945.285	134.341
47209	6.7965	1128.47	27.9435
47210	6.7967	1023.82	35.0359
47211	6.7968	1083.24	92.088
47212	6.797	1118.97	128.47
47213	6.7971	1021.89	266.421
47214	6.7973	937.037	230.305
47215	6.7978	1047.3101	18.3961
47216	6.7999	944.744	91.5011
47217	6.8002	1083.23	122.164
47218	6.8002	1205.54	138.928
47219	6.8003	978.738	189.246
47220	6.8003	927.091	252.497
47221	6.8007	1095.51	48.4742

47222	6.8008	1033.65	16.2597
47223	6.8012	1092.7	29.9193
47224	6.8014	1113.12	90.0195
47225	6.8016	986.882	174.637
47226	6.8026	1203.5699	99.0881
47227	6.8031	916.694	163.806
47228	6.8032	1078.4	54.7878
47229	6.8035	851.03	50.7557
47230	6.8036	996.159	254.017
47231	6.8039	999.344	85.8802
47232	6.8041	1076.7	151.118
47233	6.8041	1166.24	99.6237
47234	6.8042	974.76	9.6224
47235	6.8043	1076.02	32.0157
47236	6.805	969.608	221.63
47237	6.8052	1039.5699	223.076
47238	6.8053	986.498	218.982
47239	6.8059	1057.7	192.899
47240	6.806	1196.8101	99.0968
47241	6.8062	1160.89	72.6922
47242	6.8064	1014.72	193.129
47243	6.8067	1103.23	49.2724
47244	6.8073	1233.23	68.0646
47245	6.8074	-3.2922	304.201
47246	6.808	965.705	299.749
47247	6.8081	1037.9399	57.1103
47248	6.809	949.38	214.434
47249	6.8092	962.206	234.029
47250	6.8095	1107.55	67.1418
47251	6.8096	984.685	24.5729
47252	6.8097	1052.45	296.141
47253	6.8098	1131.9301	322.075
47254	6.8102	1116.79	160.758
47255	6.8107	946.204	249.555
47256	6.8117	1036.24	50.6221
47257	6.8122	971.324	264.153
47258	6.8125	1126.58	234.529
47259	6.8128	1074.25	147.898
47260	6.8128	1094.84	101.338
47261	6.813	1053.41	263.946
47262	6.8131	914.437	246.35
47263	6.8132	1169.11	17.8879
47264	6.8133	1281.29	48.4407
47265	6.8135	1217.4301	129.858
47266	6.8138	923.909	237.466
47267	6.8144	891.512	222.422
47268	6.8145	1262.67	19.2508
47269	6.8148	1235.53	37.3996

47270	6.8153	877.339	151.056
47271	6.8155	981.504	245.261
47272	6.8155	-2.8117	265.458
47273	6.8157	978.355	214.561
47274	6.8166	930.426	173.725
47275	6.8167	1012.86	345.083
47276	6.8171	1129.96	86.2488
47277	6.8171	1020.12	164.272
47278	6.8172	991.807	75.7445
47279	6.8172	1077.89	103.492
47280	6.8175	971.392	2.494
47281	6.8177	1025.88	251.636
47282	6.8178	893.221	90.5768
47283	6.8192	1001.43	224.213
47284	6.8193	1233.36	22.3231
47285	6.8201	1096.6801	265.08
47286	6.8211	1017.45	64.4113
47287	6.8211	918.411	239.144
47288	6.8214	1182.3199	285.011
47289	6.8215	1305.88	89.7939
47290	6.8217	1073.36	101.561
47291	6.8225	925.059	188.057
47292	6.8227	1031.79	112.414
47293	6.8227	929.104	24.0463
47294	6.823	932.084	218.024
47295	6.823	1086.8	86.0127
47296	6.8233	1064.3199	14.5288
47297	6.8236	1019.79	147.266
47298	6.8237	1048.0601	104.806
47299	6.8238	1069.33	95.2624
47300	6.8245	1201.77	17.3215
47301	6.8245	1245.41	28.9305
47302	6.8247	743.938	150.533
47303	6.8256	964.046	129.2
47304	6.8261	1182.6899	23.1164
47305	6.8262	1000.01	163.484
47306	6.8262	1113.99	147.496
47307	6.8271	1087.87	150.035
47308	6.8273	960.971	319.19
47309	6.8273	956.259	172.95
47310	6.8274	943.212	151.165
47311	6.828	958.229	226.44
47312	6.8282	1046.6899	113.902
47313	6.8285	1096.76	46.1317
47314	6.8287	983.698	320.549
47315	6.8287	1164.0699	79.3171
47316	6.8291	1201.33	20.7213
47317	6.8291	1020.72	93.6045

47318	6.8292	900.285	167.235
47319	6.8293	1106.61	100.007
47320	6.8295	908.611	227.849
47321	6.8296	936.18	7.0351
47322	6.83	919.69	221.246
47323	6.8304	1202.5699	108.501
47324	6.8309	1205.73	29.1942
47325	6.831	1046.25	194.953
47326	6.831	1074.73	98.9016
47327	6.8313	963.623	200.51
47328	6.8314	945.137	242.78
47329	6.8315	978.532	148.226
47330	6.8317	1000.11	176.943
47331	6.8318	985.351	100.03
47332	6.8326	1175.39	259.438
47333	6.8331	1211.1	26.3331
47334	6.8335	1138.09	21.2144
47335	6.8338	1266.66	284.583
47336	6.8341	1163.95	333.168
47337	6.8342	1090.74	87.206
47338	6.8343	903.104	123.259
47339	6.8343	802.422	20.757
47340	6.8344	943.011	162.433
47341	6.835	1079.4399	78.7585
47342	6.835	907.905	33.2435
47343	6.8359	888.767	221.756
47344	6.836	1061.02	291.296
47345	6.8362	1042.49	35.9941
47346	6.8363	1056.28	161.975
47347	6.8369	1050.67	76.2955
47348	6.837	1032.1	162.175
47349	6.8371	483.067	322.976
47350	6.8376	981.467	338.133
47351	6.8378	974.744	157.102
47352	6.8379	1059.9399	247.737
47353	6.8382	1238.16	92.2142
47354	6.8385	1061.52	127.65
47355	6.8388	988.357	152.623
47356	6.8389	1193.3	34.4774
47357	6.8393	1024.47	69.5814
47358	6.8395	1165.74	25.9677
47359	6.8396	946.287	170.419
47360	6.8396	1199.46	41.0905
47361	6.8405	1136.9	144.966
47362	6.8407	952.581	17.8129
47363	6.8408	945.999	248.682
47364	6.8413	1130.65	47.1401
47365	6.8415	1087	95.1325

47366	6.8416	1061.38	63.2661
47367	6.8421	1042.65	173.3
47368	6.8422	1025.78	248.009
47369	6.8426	1024.48	44.6034
47370	6.8426	1052.65	97.9318
47371	6.843	1075.01	204.343
47372	6.8431	906.923	136.026
47373	6.8433	962.06	304.195
47374	6.8433	1115.34	26.1079
47375	6.8435	965.311	117.167
47376	6.8435	1053.17	162.671
47377	6.8446	1064.83	159.725
47378	6.8451	1244.01	17.0372
47379	6.8452	1250.12	25.4096
47380	6.8458	947.077	215.159
47381	6.8461	1039.5	121.755
47382	6.8464	1041.72	24.0643
47383	6.8465	1064.59	56.0978
47384	6.8465	1223.52	108.494
47385	6.8469	1009.99	76.4156
47386	6.847	1103.39	31.3687
47387	6.8473	1086.46	61.8617
47388	6.848	923.646	174.747
47389	6.8481	914.34	198.299
47390	6.8481	1052.78	41.3208
47391	6.8482	1242.4301	12.7059
47392	6.8485	1073.23	300.65
47393	6.8492	1178.75	194.09
47394	6.8493	959.444	136.567
47395	6.8499	1154.46	253.639
47396	6.8507	1130.52	104.072
47397	6.8508	1183.11	82.8842
47398	6.8511	1007.05	259.662
47399	6.8518	1152.72	98.5586
47400	6.8519	997.003	96.2075
47401	6.8522	1156.22	293.95
47402	6.8523	1088.9	90.9414
47403	6.8527	1069.39	237.333
47404	6.8532	1271.6899	22.9321
47405	6.8534	1027.22	71.3681
47406	6.8538	887.521	256.911
47407	6.8541	1183.63	22.492
47408	6.8544	1151.9399	240.377
47409	6.8544	1059.01	72.0628
47410	6.8545	480.784	80.889
47411	6.8553	953.414	236.401
47412	6.8555	1015.86	45.6111
47413	6.8564	1060.1801	183.498

47414	6.8564	1040.77	50.3368
47415	6.8566	1070.75	281.622
47416	6.8571	1021.87	311.859
47417	6.8573	1007.05	76.6328
47418	6.8573	1127.11	135.601
47419	6.8574	1078.52	302.75
47420	6.8582	1088.55	77.9669
47421	6.8586	1068.03	121.703
47422	6.8591	-2.8296	266.815
47423	6.8595	949.938	228.07
47424	6.8602	972.286	65.7914
47425	6.8607	1062.92	179.636
47426	6.8609	1139.9399	257.923
47427	6.8609	913.187	186.292
47428	6.8609	1013.91	160.577
47429	6.8609	1074.5699	279.809
47430	6.8616	1053.51	252.248
47431	6.8617	1085.89	99.0479
47432	6.8621	1094.1801	81.3855
47433	6.8629	985.367	81.0433
47434	6.863	1081.02	63.0476
47435	6.8632	893.581	254.264
47436	6.8632	997.217	213.197
47437	6.8633	1140.8	35.4892
47438	6.8636	1032.5	178.146
47439	6.8636	964.873	47.9815
47440	6.8643	1114.9301	187.337
47441	6.8644	1049.87	50.391
47442	6.8647	1015.79	296.085
47443	6.8649	1154.0601	25.8937
47444	6.8657	981.036	17.2991
47445	6.8658	1039.36	17.3645
47446	6.8664	981.857	54.7854
47447	6.8669	1163.62	124.085
47448	6.8674	1168.6899	14.8067
47449	6.8682	1158.48	116.473
47450	6.8686	1028.64	277.697
47451	6.8689	1223.49	17.5761
47452	6.8694	1295.46	22.275
47453	6.8699	949.99	183.562
47454	6.8699	-4.2049	270.117
47455	6.8701	1061.5601	190.745
47456	6.8701	976.839	74.8389
47457	6.8702	958.102	300.647
47458	6.8704	1078.8	39.271
47459	6.8705	1007.4	296.846
47460	6.8705	1045.58	50.8421
47461	6.8706	989.741	165.389

47462	6.8707	1039.6899	149.149
47463	6.8707	882.586	69.1141
47464	6.8714	991.459	81.4077
47465	6.8715	1022.11	118.311
47466	6.8719	998.771	85.5834
47467	6.8724	1255.4301	14.2372
47468	6.8724	983.59	178.589
47469	6.8727	1251.08	43.7731
47470	6.8733	785.448	66.1782
47471	6.8737	1041.35	108.468
47472	6.8737	932.198	184.341
47473	6.8738	1131.3199	118.467
47474	6.8742	1018.61	110.772
47475	6.8744	717.636	157.331
47476	6.8751	980.53	164.885
47477	6.8755	1152.61	17.799
47478	6.8756	1056.25	347.973
47479	6.8763	959.353	318.733
47480	6.8777	843.501	334.137
47481	6.878	1168.21	42.5014
47482	6.8783	1114.83	31.9992
47483	6.8792	958.793	71.1322
47484	6.8799	904.293	178.652
47485	6.88	1269.99	22.5898
47486	6.8801	1102.51	219.276
47487	6.8803	1106.65	326.261
47488	6.8807	1248.65	33.2213
47489	6.8813	1092.02	90.7402
47490	6.8822	1026.8101	248.111
47491	6.8823	1058.86	121.381
47492	6.8825	964.952	169.958
47493	6.8827	940.813	198.162
47494	6.8831	1104.95	151.556
47495	6.8834	1002.77	255.457
47496	6.8837	842.273	253.436
47497	6.884	746.829	171.813
47498	6.884	1170.17	9.8417
47499	6.8841	1095.08	339.462
47500	6.885	927.818	221.38
47501	6.8851	1130.8199	252.233
47502	6.8851	1104.47	33.7835
47503	6.8852	1056	232.256
47504	6.8858	1220.28	138.667
47505	6.886	1093.08	248.238
47506	6.8861	987.8	303.062
47507	6.8862	889.526	251.779
47508	6.8866	1104.21	66.6775
47509	6.8868	1097.8101	174.543

47510	6.8868	1066.4301	102.212
47511	6.8874	973.718	266.249
47512	6.8876	1125.23	15.9233
47513	6.8878	995.969	59.9615
47514	6.8879	1091.14	302.617
47515	6.8882	1005.39	115.507
47516	6.8883	1120.58	33.3562
47517	6.8885	1129.0699	73.376
47518	6.8886	1372.83	12.7065
47519	6.8887	1076.37	198.813
47520	6.8891	1243.66	100.86
47521	6.8892	728.646	156.723
47522	6.8894	1135.54	109.723
47523	6.8897	1243.7	31.4776
47524	6.8898	1114.8199	25.0786
47525	6.8899	1139.88	84.0996
47526	6.8899	968.56	159.155
47527	6.8911	924.727	247.981
47528	6.8912	1264.8	113.694
47529	6.8913	886.87	170.763
47530	6.8915	1128.88	254.41
47531	6.8917	1102.92	191.537
47532	6.8921	1293.37	11.7682
47533	6.8922	1151.6801	280.232
47534	6.8926	965.619	238.714
47535	6.894	1189.38	29.2231
47536	6.8941	943.337	124.189
47537	6.8941	1088.28	35.6609
47538	6.8949	1199.58	29.7506
47539	6.8949	1015.83	153.134
47540	6.8953	929.905	147.142
47541	6.8954	1148.87	58.8425
47542	6.8958	999.222	201.611
47543	6.8959	1028.46	236.814
47544	6.8962	1097.01	112.608
47545	6.8962	952.031	238.126
47546	6.8962	1224.2	59.2818
47547	6.8963	925.056	247.931
47548	6.8969	1116.5699	32.8627
47549	6.8972	1229.22	145.155
47550	6.8975	1054.85	257.356
47551	6.8977	1037.75	9.8253
47552	6.898	1129.37	142.771
47553	6.8982	1236.88	23.6801
47554	6.8991	968.581	199.614
47555	6.8993	985.735	136.455
47556	6.8994	1061.45	262.159
47557	6.8994	1191.16	227.073

47558	6.8996	1238.66	99.6893
47559	6.9004	949.007	173.531
47560	6.9004	978.681	186.603
47561	6.9005	1059.6	196.831
47562	6.9013	1002.97	251.7
47563	6.9014	1232.62	15.2275
47564	6.9019	1160.1	20.1326
47565	6.9019	1246.0699	27.6497
47566	6.9026	1155.96	295.982
47567	6.9042	1105.02	164.591
47568	6.9043	1030.97	4.7329
47569	6.9048	1121.01	356.313
47570	6.9048	1033.21	170.073
47571	6.9054	925.442	183.028
47572	6.9056	1021.96	353.552
47573	6.9056	1005.43	260.85
47574	6.906	1124.3199	76.7521
47575	6.9061	1171.88	81.4829
47576	6.9068	1074.89	163.949
47577	6.9069	1255.47	24.9156
47578	6.9069	1008.29	56.0483
47579	6.9074	977.766	27.6135
47580	6.9079	1036.15	235.868
47581	6.9079	1064.51	97.0485
47582	6.9082	1043.15	163.962
47583	6.9087	942.81	227.366
47584	6.909	1120.13	19.9874
47585	6.909	1124.1801	104.475
47586	6.9092	1195.27	183.894
47587	6.9098	982.545	188.216
47588	6.9103	1095.11	196.717
47589	6.9108	953.6	301.095
47590	6.9109	1232.8199	75.2822
47591	6.911	1020.71	354.153
47592	6.9111	1036.37	150.25
47593	6.9114	1157.54	126.16
47594	6.9115	1128.03	58.1843
47595	6.9117	1254.99	48.8812
47596	6.912	1293.66	21.2473
47597	6.9123	1067.8199	159.031
47598	6.9128	1104.49	150.157
47599	6.9134	1208.9301	14.8048
47600	6.9137	967.541	217.975
47601	6.9137	1056.53	50.6317
47602	6.9138	964.285	239.34
47603	6.914	1219.05	238.608
47604	6.9142	1113.22	73.378
47605	6.9144	1003.37	26.0381

47606	6.9145	1368.36	259.504
47607	6.9147	1320.3101	23.1683
47608	6.9148	991.023	154.591
47609	6.9148	1056.0699	163.9
47610	6.9154	1200.03	0.8316
47611	6.9158	970.809	254.223
47612	6.9159	1002.84	166.811
47613	6.9161	1050.11	164.809
47614	6.9165	1183.01	135.435
47615	6.917	1024.84	163.003
47616	6.917	1114.8	85.3098
47617	6.9175	1264.48	0.8972
47618	6.9175	1035.58	259.642
47619	6.9179	1068.38	215.189
47620	6.9192	1062.66	89.0218
47621	6.9194	1248.3199	22.9772
47622	6.9194	1338.9	16.3558
47623	6.9196	997.724	162.936
47624	6.9198	924.559	247.234
47625	6.92	1068.1	87.2145
47626	6.9206	928.789	100.775
47627	6.921	1116.04	53.1746
47628	6.921	1065.86	164.658
47629	6.9214	1026.95	23.3636
47630	6.9216	1077.0601	300.504
47631	6.9228	1027.35	135.668
47632	6.9229	1243.3199	132.161
47633	6.923	1040.65	259.254
47634	6.9232	1117.99	197.422
47635	6.9233	1161.34	79.8915
47636	6.924	946.076	237.146
47637	6.9242	1091.92	205.594
47638	6.9242	1083.05	218.012
47639	6.9242	1336.1	29.3974
47640	6.9243	1216.89	16.8985
47641	6.9245	918.93	258.216
47642	6.9257	1106.24	178.446
47643	6.9263	1100.29	164.532
47644	6.928	1115.78	17.0934
47645	6.9284	1061.12	152.608
47646	6.9289	1089.01	54.0739
47647	6.9294	1110.98	233.617
47648	6.9294	1198.53	109.734
47649	6.93	944.476	169.257
47650	6.9318	1241.27	76.8985
47651	6.9323	1048.35	96.2957
47652	6.9327	1221.53	244.408
47653	6.9349	1174.01	17.2756

47654	6.936	1224.51	8.2133
47655	6.9361	1182.36	32.5743
47656	6.9369	1064.79	230.678
47657	6.937	1068.65	358.293
47658	6.9373	968.827	153.196
47659	6.9382	1145.9301	207.695
47660	6.9386	1132.27	133.227
47661	6.9387	1032.1899	188.921
47662	6.9391	855.217	154.076
47663	6.9392	1173.09	87.5759
47664	6.9394	1167.58	166.622
47665	6.94	1079.8	124.429
47666	6.9401	1246.91	11.2332
47667	6.9409	962.619	222.979
47668	6.9414	1235.23	17.4434
47669	6.9415	1255.38	21.9046
47670	6.9418	1119.59	113.518
47671	6.9422	1108.39	248.879
47672	6.9424	1125.34	220.707
47673	6.9425	1065.96	258.061
47674	6.9437	973.418	136.999
47675	6.9437	1111.04	297.866
47676	6.9437	1066.4	14.9378
47677	6.9437	1139.65	158.858
47678	6.944	1024.45	18.153
47679	6.9443	1120.0601	110.347
47680	6.9444	1128.77	329.501
47681	6.9447	1005.21	278.77
47682	6.9451	1134.76	86.1608
47683	6.9457	1042.17	150.652
47684	6.9462	1065.41	97.1159
47685	6.9464	1010.02	59.7208
47686	6.9465	1191.26	43.7134
47687	6.9467	1074.1	299.317
47688	6.9472	1181.74	236.941
47689	6.9478	1051.62	141.352
47690	6.9479	994.915	10.2882
47691	6.9481	1201.62	66.8181
47692	6.9483	991.237	292.629
47693	6.9486	1069.11	299.72
47694	6.9487	1079.9	22.8351
47695	6.9488	1033.13	180.63
47696	6.9489	1028.1	108.468
47697	6.9501	1043.6	235.178
47698	6.9502	1070.98	193.908
47699	6.9512	951.466	318.573
47700	6.9513	1114.1899	294.924
47701	6.9516	977.315	260.661

47702	6.9523	1027.02	2.1002
47703	6.9528	955.789	164.364
47704	6.9529	1017.37	60.4643
47705	6.953	1067.4399	305.169
47706	6.9534	1044.04	170.599
47707	6.9536	-2.8446	6.3753
47708	6.9538	1269.86	69.4431
47709	6.9544	1115.86	116.91
47710	6.9548	1098.49	145.905
47711	6.9549	1144.0699	135.43
47712	6.9553	1045.85	164.547
47713	6.9563	733.682	158.561
47714	6.9567	942.011	223.032
47715	6.9573	1262.95	52.4522
47716	6.9574	1046.64	299.052
47717	6.9575	1129.23	30.9122
47718	6.9577	1245.0601	109.658
47719	6.9577	1143.6	102.017
47720	6.9578	986.365	181.089
47721	6.958	1252.5	20.8412
47722	6.9582	1024.09	238.033
47723	6.9588	1045.0601	193.709
47724	6.9592	964.386	109.716
47725	6.9592	1170.41	237.869
47726	6.9593	952.461	164.103
47727	6.9595	1158.0699	150.674
47728	6.9598	1131.92	149.648
47729	6.9602	961.203	185.274
47730	6.9606	1422.72	28.2412
47731	6.9607	1077.86	283.915
47732	6.9609	1007.26	227.517
47733	6.961	1002.83	185.825
47734	6.9619	801.175	67.3417
47735	6.9629	1158.09	304.178
47736	6.9631	932.792	251.848
47737	6.9633	930.239	41.8943
47738	6.9638	1061.98	166.004
47739	6.9644	1034.76	157.486
47740	6.965	1303.49	47.445
47741	6.9652	1206.83	300.837
47742	6.9652	1115.58	324.731
47743	6.9654	1242.11	18.8233
47744	6.9655	1097.03	20.9724
47745	6.9656	1103.5699	105.68
47746	6.9657	1518.64	257.151
47747	6.9658	1020.74	263.355
47748	6.966	1145.91	35.9309
47749	6.9662	1014.49	238.015

47750	6.9666	1193.65	86.2571
47751	6.9667	1154.41	80.2707
47752	6.9669	1117.58	66.1903
47753	6.9681	1053.54	24.6034
47754	6.9686	1350.89	107.491
47755	6.969	1052.52	99.6645
47756	6.969	1121.9301	197.331
47757	6.9697	1188.39	27.764
47758	6.9699	1267.49	34.9938
47759	6.9699	1107.28	109.039
47760	6.9699	1115.52	98.0351
47761	6.9701	1001.63	252.176
47762	6.9701	1078.48	84.2089
47763	6.9703	1011.68	242.7
47764	6.9704	988.204	179.19
47765	6.9706	1258.6899	22.4253
47766	6.9708	907.719	248.789
47767	6.971	1191.74	18.2002
47768	6.971	1067.08	261.301
47769	6.9712	1253.8199	101.779
47770	6.9712	1182.99	98.9251
47771	6.9712	1297.67	45.1437
47772	6.9715	1097.73	33.7324
47773	6.972	1123.1801	105.923
47774	6.9723	1115.87	29.5824
47775	6.9725	1057.35	157.858
47776	6.9737	1298.87	270.138
47777	6.9739	1077.3199	325.157
47778	6.9741	1134.8199	5.4376
47779	6.9744	1130.8199	323.224
47780	6.9745	967.724	251.315
47781	6.9748	1177.01	359.043
47782	6.975	1326.91	21.5289
47783	6.9751	1084.87	108.902
47784	6.9752	1290.5699	10.6225
47785	6.9759	814.464	24.4013
47786	6.9759	1268.88	265.249
47787	6.9763	1042.92	12.8679
47788	6.9765	945.321	252.01
47789	6.9765	1299.63	21.7607
47790	6.9776	1063.87	146.967
47791	6.9778	1133.14	194.917
47792	6.9779	1037.3	250.621
47793	6.9782	1193.84	288.877
47794	6.979	1215.77	42.4151
47795	6.9806	1172.3199	167.046
47796	6.9825	967.682	254.856
47797	6.9826	943.365	256.032

47798	6.9836	1237.6	98.4193
47799	6.9836	1199.64	152.253
47800	6.9837	1299.12	94.4344
47801	6.9842	1032.64	163.032
47802	6.9844	1063.9301	176.752
47803	6.9848	1271.6801	188.877
47804	6.985	1082.92	106.926
47805	6.985	991.587	188.947
47806	6.9852	810.479	156.982
47807	6.9854	1102.88	266.645
47808	6.9858	482.8	333.815
47809	6.9865	1069.22	151.152
47810	6.9868	1054.92	132.034
47811	6.9871	1065.7	57.5167
47812	6.9873	1203.04	102.915
47813	6.9875	1304.1801	105.282
47814	6.988	1401.6801	18.7502
47815	6.9881	1081.53	26.1343
47816	6.9895	1129.17	3.5064
47817	6.9902	1242	24.2305
47818	6.9912	1196.71	25.3563
47819	6.9912	1054.6899	195.716
47820	6.9915	1193.74	280.327
47821	6.9917	1170.64	52.6201
47822	6.9921	1010.62	87.4307
47823	6.9923	1228.98	104.058
47824	6.9924	1021.62	171.14
47825	6.9924	1295.28	24.7181
47826	6.9932	1226.4	79.9863
47827	6.9937	550.135	76.7939
47828	6.9937	1201.09	120.414
47829	6.994	1139	168.665
47830	6.994	1208.6899	25.6428
47831	6.9941	1131.39	99.8426
47832	6.9951	1026.54	156.913
47833	6.9951	1286.96	29.7873
47834	6.9953	1081.1899	346.438
47835	6.9954	779.943	155.291
47836	6.9957	1286.13	31.4101
47837	6.9957	1141.79	305.066
47838	6.9961	954.62	164.573
47839	6.9962	993.569	151.953
47840	6.9968	1064.6899	105.001
47841	6.9968	1080.9	74.7632
47842	6.9969	1208.34	72.4838
47843	6.9974	1047.42	262.505
47844	6.9981	999.672	299.659
47845	6.9982	1082.4399	237.248

47846	6.9985	983.064	187.314
47847	7.0002	949.547	305.469
47848	7.0004	1098.41	222.884
47849	7.0006	837.45	344.448
47850	7.0008	1147.4399	25.7037
47851	7.0009	1138.66	143.967
47852	7.001	1054.66	29.968
47853	7.0012	1043.79	198.675
47854	7.0012	482.699	336.609
47855	7.0021	1081.38	252.466
47856	7.0023	990.428	253.523
47857	7.0031	1063.59	226.209
47858	7.0034	1054.39	248.804
47859	7.0042	948.517	185.744
47860	7.0051	1116.6801	35.4523
47861	7.0054	1113.9	162.881
47862	7.0057	1137.1801	91.2525
47863	7.0059	1066.72	95.446
47864	7.006	1222.62	296.444
47865	7.0062	1162.55	27.0596
47866	7.0064	982.037	64.2978
47867	7.0065	1139.62	146.476
47868	7.007	1100.13	299.914
47869	7.0072	1205.05	76.87
47870	7.0074	1295.97	77.2959
47871	7.0077	1121.14	252.279
47872	7.0078	1028.62	173.85
47873	7.0079	1228.66	255.135
47874	7.0084	1036.0601	177.507
47875	7.0089	1166.39	232.152
47876	7.009	1015.89	165.024
47877	7.0092	1019.79	234.188
47878	7.01	1060.61	72.5955
47879	7.0102	1264.0699	22.5572
47880	7.0107	1104.95	319.645
47881	7.0113	1092.08	106.918
47882	7.0126	1162.6	143.828
47883	7.0127	1148.8	147.015
47884	7.0127	1216.02	64.783
47885	7.0128	1158.4301	93.678
47886	7.013	1151.6801	22.8218
47887	7.0133	1119.47	192.485
47888	7.0143	1180.5	142.013
47889	7.0146	1124.9399	64.7732
47890	7.0147	1201.8199	25.1344
47891	7.0153	1003.95	166.841
47892	7.0153	1206.27	0.4946
47893	7.0156	1154.61	109.918

47894	7.0156	1251.29	17.8246
47895	7.0158	1284.59	96.2405
47896	7.0162	1235.83	25.6887
47897	7.0163	1120.46	297.87
47898	7.0174	1181.55	133.087
47899	7.018	1154.97	74.3038
47900	7.0194	973.822	248.703
47901	7.0196	1199.45	184.292
47902	7.0196	1159.78	3.2104
47903	7.0198	994.007	165.658
47904	7.02	1077.75	31.62
47905	7.0228	1017.59	226.617
47906	7.0228	1201.59	37.6625
47907	7.0229	1125.53	297.279
47908	7.0237	1068.6801	266.683
47909	7.0238	1044.3101	217.696
47910	7.0255	1277.62	343.438
47911	7.0255	1366.12	16.0109
47912	7.0258	1188.5	280.822
47913	7.026	1143.54	226.948
47914	7.0262	1286.29	184.269
47915	7.0264	1151.85	47.6287
47916	7.0268	235.301	18.0894
47917	7.0271	1131.05	153.156
47918	7.0278	1293.37	19.4386
47919	7.0281	1294.51	88.7686
47920	7.0286	1167.35	140.788
47921	7.0287	1099.66	150.732
47922	7.0288	1158.76	22.5833
47923	7.0292	1121.79	108.594
47924	7.0301	1005.28	234.867
47925	7.0301	1076.77	193.132
47926	7.0303	979.831	251.94
47927	7.0305	967.22	222.661
47928	7.0305	1037.23	242.57
47929	7.0308	1120.92	148.346
47930	7.0328	1116.5	106.262
47931	7.033	592.489	348.347
47932	7.0333	949.44	140.689
47933	7.0344	1110.42	164.35
47934	7.0348	-2.9348	289.622
47935	7.0359	1015.64	25.6386
47936	7.0362	1070.4399	149.158
47937	7.0365	1122.46	52.8884
47938	7.0366	1089.96	26.1394
47939	7.037	1279	231.119
47940	7.0371	1066.5	302.754
47941	7.0372	1224.9399	75.3675

47942	7.0377	1057.66	101.16
47943	7.0378	1109.8101	78.1569
47944	7.038	1089.61	26.6249
47945	7.0382	1023.88	232.359
47946	7.0395	1231.1	174.45
47947	7.0404	1022.8	137.717
47948	7.0407	1235.84	27.0354
47949	7.0411	995.822	249.686
47950	7.0415	1335.8	24.7858
47951	7.0425	1043.71	250.517
47952	7.0426	1185.27	37.5656
47953	7.0429	1126.96	193.35
47954	7.0429	1306.16	31.7977
47955	7.0431	1201.22	23.5864
47956	7.0436	-2.8196	271.174
47957	7.0446	1059.99	203.29
47958	7.0459	1037.35	181.664
47959	7.0465	1043.15	2.8797
47960	7.0465	1132.0601	37.4015
47961	7.0465	1075.85	98.5175
47962	7.0467	1046.89	143.81
47963	7.0468	1101.51	262.282
47964	7.0468	1117.05	21.7266
47965	7.047	1372.85	342.322
47966	7.0471	1063.8101	106.761
47967	7.0485	1054.38	111.421
47968	7.0486	984.031	168.479
47969	7.0491	1090.9301	121.326
47970	7.0495	1287.98	276.754
47971	7.0495	1227.51	155.549
47972	7.0496	891.186	82.0939
47973	7.0504	1145.3101	152.051
47974	7.0515	1299.24	268.184
47975	7.0517	964.215	252.295
47976	7.0523	908.309	269.553
47977	7.0523	1039.13	115.518
47978	7.0533	1229.1801	14.8624
47979	7.0537	1244.41	253.355
47980	7.054	1325.3101	33.2797
47981	7.0542	1361.95	96.168
47982	7.0548	1060.13	35.4996
47983	7.0549	847.062	296.651
47984	7.0555	1127.48	59.93
47985	7.0559	1261.0601	91.7927
47986	7.0562	1095.88	65.3823
47987	7.0565	880.138	44.3283
47988	7.0569	1077.21	148.871
47989	7.0598	960.511	176.985

47990	7.0599	1172.92	74.582
47991	7.0601	1007.69	246.979
47992	7.0602	1072.03	251.929
47993	7.0606	1228.67	173.538
47994	7.0607	1097.13	280.207
47995	7.0609	1103.28	166.19
47996	7.0615	1255.04	238.158
47997	7.0616	1051.7	85.2725
47998	7.0617	1070.92	128.496
47999	7.0626	1295.53	280.8
48000	7.0628	1213.87	30.6032
48001	7.0633	1048.97	229.239
48002	7.0637	1093.75	329.026
48003	7.0638	1197.89	259.739
48004	7.0647	1343.88	181.664
48005	7.0649	1555.08	43.0602
48006	7.0653	1216.5699	37.0512
48007	7.0667	1175.9	47.266
48008	7.0669	1085.9301	193.782
48009	7.0669	1153.74	81.6283
48010	7.0678	1232.45	120.42
48011	7.0681	1116.12	52.0182
48012	7.0682	1089.6899	115.073
48013	7.0684	1035.4301	172.954
48014	7.0691	1033.8101	173.641
48015	7.0693	1176.17	197.198
48016	7.0693	1030.8101	329.435
48017	7.0694	1354.6801	258.91
48018	7.0699	1115.35	292.265
48019	7.0702	1069.11	159.486
48020	7.0706	1197.37	27.0633
48021	7.0708	1143.05	23.7939
48022	7.0718	1173.13	139.11
48023	7.0722	1368.95	94.8472
48024	7.0733	991.719	182.079
48025	7.0745	1093.39	70.9307
48026	7.0749	1223.62	69.7964
48027	7.0751	887.334	226.076
48028	7.0752	1145.08	221.51
48029	7.076	1051.26	359.063
48030	7.0763	1050.58	32.0645
48031	7.0768	1314.1801	29.368
48032	7.0771	1111.5699	173.997
48033	7.0772	1157.53	216.478
48034	7.0773	1104.4399	192.312
48035	7.0774	1162.72	116.524
48036	7.0781	1060.95	93.519
48037	7.0785	1158.97	35.9546

48038	7.0786	1182.59	112.213
48039	7.0792	1051.42	184.642
48040	7.0793	1192.05	0.3247
48041	7.0793	1247.12	54.4007
48042	7.0794	1254.42	69.8972
48043	7.0801	1134.77	224.594
48044	7.0802	1086.26	249.804
48045	7.0802	1325.1	262.974
48046	7.081	1337.51	17.2391
48047	7.0814	1137.13	18.8046
48048	7.0814	1069.17	196.732
48049	7.0819	1149.45	347.589
48050	7.082	1189.04	140.493
48051	7.082	954.634	177.817
48052	7.0834	1118.67	144.918
48053	7.0846	1166.96	254.481
48054	7.0848	984.007	238.543
48055	7.0854	1114.79	164.13
48056	7.0863	964.438	293.575
48057	7.0865	1272.45	18.4775
48058	7.087	1329.08	139.322
48059	7.0875	1032.89	170.006
48060	7.0877	1266.96	28.178
48061	7.0879	1150.23	259.226
48062	7.0885	1144.58	78.851
48063	7.0887	1152.39	83.7092
48064	7.0893	954.206	186.779
48065	7.0895	1275.79	224.923
48066	7.0905	988.636	108.166
48067	7.0906	1082.42	150.013
48068	7.0912	1371.09	21.8595
48069	7.0922	1287.64	60.1728
48070	7.0927	1244.65	149.978
48071	7.0928	1388.64	101.773
48072	7.0932	1195.4301	18.3392
48073	7.0934	1178.15	20.2464
48074	7.0939	1240.39	188.584
48075	7.0948	1261.35	83.1791
48076	7.0975	967.929	162.613
48077	7.099	1214.42	112.333
48078	7.1	1180.12	55.7225
48079	7.101	1082.38	164.364
48080	7.1012	1050.33	165.142
48081	7.1012	1086.89	148.284
48082	7.1013	1081	14.9254
48083	7.1016	1054.66	49.26
48084	7.1018	1331.36	138.937
48085	7.1021	981.281	180.572

48086	7.1023	1125.92	137.503
48087	7.1026	1174.79	7.052
48088	7.1034	1239.4399	93.8449
48089	7.1036	1301.58	29.4809
48090	7.1049	1381.59	22.169
48091	7.1051	1275.97	343.071
48092	7.1051	1084.46	234.974
48093	7.1051	1110.75	151.876
48094	7.1052	1254.0601	74.827
48095	7.1054	1381.17	25.3282
48096	7.1067	1369.75	142.024
48097	7.1071	1226.99	150.44
48098	7.1075	1163.9301	165.255
48099	7.1077	1403.03	69.3943
48100	7.1079	1311.67	19.2752
48101	7.1079	1360.15	24.7143
48102	7.1092	1064.5601	20.5704
48103	7.1096	1137.92	105.699
48104	7.1097	1279.55	23.7273
48105	7.1098	1156.26	44.7859
48106	7.111	1393.42	46.1482
48107	7.1111	1037.6801	244.993
48108	7.1111	1077.17	259.124
48109	7.1111	992.293	266.703
48110	7.1119	1070.75	291.488
48111	7.1121	1043.23	179.828
48112	7.1124	1053.98	149.275
48113	7.1126	1217.9399	83.3464
48114	7.1127	1026.79	143.523
48115	7.1127	1088.7	101.191
48116	7.1127	1379.5601	70.3135
48117	7.113	1132.8	29.8445
48118	7.1132	1056.72	56.6091
48119	7.1154	1004.26	256.413
48120	7.1167	1126.01	219.912
48121	7.1171	1233.24	328.83
48122	7.1173	1137.78	224.79
48123	7.1173	1374.59	77.0597
48124	7.1178	1217.1	79.0899
48125	7.1178	1218.04	28.5503
48126	7.1182	1066.92	84.6701
48127	7.1193	1226.85	164.074
48128	7.1194	1111.87	173.058
48129	7.1198	1017.05	252.866
48130	7.121	1278.52	104.511
48131	7.1218	1152.3199	114.099
48132	7.1223	1131.47	186.339
48133	7.1224	1292.95	79.2097

48134	7.1224	1012.16	157.608
48135	7.1226	1198.53	54.419
48136	7.1229	1061.01	105.74
48137	7.123	1069.42	270.645
48138	7.1241	1260.4399	12.9608
48139	7.1247	1135.5699	149.481
48140	7.1255	1428.33	30.7539
48141	7.1261	1128.09	252.629
48142	7.1266	994.637	161.506
48143	7.1268	1252.5601	12.6003
48144	7.1279	993.367	293.42
48145	7.1284	1092.13	171.739
48146	7.1288	1150.79	302.878
48147	7.1298	1191.02	11.355
48148	7.1298	1189.55	15.5736
48149	7.1309	1401.63	13.5923
48150	7.1321	1343.22	184.547
48151	7.1323	1145.51	252.105
48152	7.1329	1122.14	305.897
48153	7.1344	1220.88	13.5166
48154	7.1347	1272.96	344.288
48155	7.1358	1214.26	275.032
48156	7.1358	1136.39	215.165
48157	7.1358	1143.1801	162.085
48158	7.1365	1199.79	171.089
48159	7.1377	972.963	249.917
48160	7.1382	961.208	332.561
48161	7.1383	1136.35	215.814
48162	7.1384	1177.27	82.3846
48163	7.1384	1128.3	263.314
48164	7.1389	1033.59	180.214
48165	7.1389	1118.27	292.188
48166	7.1391	1233.85	186.304
48167	7.1391	1349.46	68.8583
48168	7.1395	1066.42	131.867
48169	7.1398	1062.86	248.771
48170	7.1403	1094.53	238.754
48171	7.1404	915.123	56.129
48172	7.1405	1337.8	23.7274
48173	7.1421	1087.03	93.6582
48174	7.1427	1048.52	59.2608
48175	7.1432	1313.15	22.4871
48176	7.1434	1083.05	223.459
48177	7.1436	1208.97	76.0295
48178	7.1438	1294.8101	99.9297
48179	7.1443	1192.5	38.7594
48180	7.1445	1026.62	46.0458
48181	7.1449	1319.14	117.512

48182	7.1454	1432.96	46.5105
48183	7.1456	1072.23	260.139
48184	7.1458	1294	22.3096
48185	7.1466	482.815	334
48186	7.147	1359.51	21.9935
48187	7.147	1210.97	63.442
48188	7.1474	1147.27	89.2467
48189	7.1475	1161.52	74.1114
48190	7.148	1305.62	35.8067
48191	7.148	1324.36	54.2427
48192	7.1483	1446.7	96.8309
48193	7.1484	1068.03	162.653
48194	7.1492	1227.8199	53.4672
48195	7.1494	1267.38	245.305
48196	7.1495	1297.62	31.5012
48197	7.1503	1083.24	217.459
48198	7.1507	1069.95	249.226
48199	7.1508	1301.7	59.0514
48200	7.151	1287.05	189.117
48201	7.151	978.909	110.916
48202	7.1513	1306.29	12.4356
48203	7.1515	1131.48	95.4566
48204	7.1516	1259.5601	62.7755
48205	7.152	1114.5601	171.583
48206	7.1521	1149.42	271.199
48207	7.1525	1202.88	76.7948
48208	7.1526	994.529	301.802
48209	7.1536	1371.9399	20.442
48210	7.1537	1194.14	53.6792
48211	7.154	1165.76	326.912
48212	7.1558	1159.5601	44.9555
48213	7.156	1304.22	189.979
48214	7.1564	1265.11	311.812
48215	7.1572	1161.73	28.715
48216	7.1576	924.251	175.517
48217	7.1579	1267.6	20.1607
48218	7.1582	1259.75	54.3006
48219	7.1601	1398.3	69.7812
48220	7.1602	1131.88	121.576
48221	7.1604	1245.5601	129.548
48222	7.1605	1247.98	28.1464
48223	7.1618	1076.67	269.358
48224	7.1621	1406.24	19.9201
48225	7.1623	1352.33	79.4354
48226	7.1627	1238.48	108.113
48227	7.1631	1332.58	201.219
48228	7.1632	1160.41	29.7688
48229	7.164	1207.79	218.818

48230	7.1644	1186.49	156.548
48231	7.1646	1092.14	301.012
48232	7.1653	1209.8101	80.9297
48233	7.1656	1140.99	198.753
48234	7.1656	1192.85	15.6187
48235	7.1664	1165.01	78.8491
48236	7.1664	1024.36	249.137
48237	7.1672	1440.9399	11.4647
48238	7.1672	1217.54	339.962
48239	7.1677	1302.3101	75.7654
48240	7.1688	1158.16	247.421
48241	7.169	1128.23	303.454
48242	7.1691	1072.39	232.227
48243	7.1696	1190.2	22.2801
48244	7.1698	1108.87	150.197
48245	7.1704	1254.8	297.257
48246	7.1705	1138.54	77.6696
48247	7.1708	1255.1	126.136
48248	7.1712	1016.4	253.059
48249	7.172	1271.3101	43.4859
48250	7.172	1154.72	74.9568
48251	7.1723	1330.02	65.1561
48252	7.173	1148.39	81.879
48253	7.173	967.8	343.192
48254	7.173	1266.9	48.4722
48255	7.1753	1306.41	200.751
48256	7.1754	887.413	19.938
48257	7.176	1202.73	29.2047
48258	7.1767	1079.02	13.333
48259	7.1769	1245.0601	232.036
48260	7.1769	1160.79	38.1797
48261	7.1774	1041.02	252.378
48262	7.1777	1137.42	165.728
48263	7.1779	1072.46	83.5997
48264	7.1783	1293.74	223.287
48265	7.1789	1105.95	278.048
48266	7.1789	1090.62	21.7944
48267	7.1798	1127.66	260.225
48268	7.1807	1071.89	141.01
48269	7.1808	1076.92	219
48270	7.181	1069.21	164.194
48271	7.181	1137.5699	139.266
48272	7.1811	1125.79	216.162
48273	7.1813	1199.48	110.751
48274	7.1815	1059.26	48.412
48275	7.1816	1380.4301	30.0349
48276	7.1818	1067.71	183.736
48277	7.1822	1201.97	52.0204

48278	7.1827	1240.63	150.928
48279	7.1827	1132.9	260.041
48280	7.1829	1177.01	126.058
48281	7.1829	1094.45	226.316
48282	7.183	1315.01	229.057
48283	7.1833	1231.08	258.47
48284	7.1834	1196.54	53.933
48285	7.1842	1085.37	196.157
48286	7.1845	1210.58	94.7106
48287	7.1848	1093.47	291.651
48288	7.1851	1346.4301	16.7336
48289	7.1851	1089.62	132.373
48290	7.1858	1265.12	258.25
48291	7.1859	1265.96	73.6973
48292	7.1863	1106.61	350.278
48293	7.1866	1109.49	305.363
48294	7.1867	1092.54	173.365
48295	7.187	1169.37	142.82
48296	7.1873	1114.0699	197.291
48297	7.1879	1068.5699	174.684
48298	7.1879	1217.22	106.191
48299	7.1881	1102.3	102.971
48300	7.1882	1062.4301	303.874
48301	7.1883	1138.4	66.4897
48302	7.1891	1384.0601	73.4035
48303	7.1891	1281.08	174.442
48304	7.1892	1277.24	61.194
48305	7.1892	1150.15	134.96
48306	7.1901	1197.6899	274.819
48307	7.191	1085.05	248.229
48308	7.1919	1090.83	108.477
48309	7.192	1313.74	228.144
48310	7.1936	1142.38	171.001
48311	7.1938	1272.0699	140.713
48312	7.194	-2.9525	328.046
48313	7.1942	1135.14	125.665
48314	7.1945	1176.1	196.679
48315	7.1947	1159.42	79.9616
48316	7.1953	1113.4301	87.014
48317	7.1962	1466.84	39.6131
48318	7.1973	1413.36	25.0577
48319	7.1979	1356.21	28.8804
48320	7.1979	1238.16	22.6626
48321	7.1982	1003.56	195.558
48322	7.1983	1185.55	119.467
48323	7.1983	1028.08	299.142
48324	7.1985	1196.77	83.8589
48325	7.1985	1164.78	34.0818

48326	7.1986	1144.08	95.141
48327	7.1991	930.794	123.274
48328	7.2001	1032.66	200.492
48329	7.2002	1444.41	81.4375
48330	7.2003	1283.12	113.772
48331	7.2013	1205.05	25.5762
48332	7.2017	1093.49	194.015
48333	7.2023	1002.9	251.901
48334	7.2024	1495.9301	74.8141
48335	7.203	483.049	348.547
48336	7.2033	1091.15	48.3699
48337	7.2034	1373.84	1.2242
48338	7.2036	1101.73	38.0255
48339	7.2036	1169.73	145.607
48340	7.2038	1309.29	23.7157
48341	7.2046	1006.46	106.07
48342	7.205	1187.34	86.3589
48343	7.2053	1133	192.413
48344	7.2055	1438.42	37.1261
48345	7.2061	1203.99	31.2787
48346	7.2066	1208.39	32.2132
48347	7.2072	1411.0699	15.0613
48348	7.2076	1250.72	141.095
48349	7.2082	1352.5601	20.1873
48350	7.2082	1267.89	76.7032
48351	7.2085	1438.76	39.0943
48352	7.2092	746.561	174.256
48353	7.2092	1163.49	294.657
48354	7.21	1108.97	119.683
48355	7.21	1230.09	49.6313
48356	7.2106	1442.29	20.1849
48357	7.2108	1308.91	183.503
48358	7.2109	1062.99	162.214
48359	7.211	1102.75	170.414
48360	7.2112	1289.51	97.8694
48361	7.2114	1256.41	277.366
48362	7.2115	1166.54	241.662
48363	7.212	1180.89	254.082
48364	7.2137	1281.33	175.757
48365	7.2137	1206.41	144.763
48366	7.2146	1282.98	83.4666
48367	7.2147	1119.48	164.204
48368	7.2149	887.41	65.4112
48369	7.215	1344.34	49.418
48370	7.2154	1243.16	233.564
48371	7.216	1382.04	334.682
48372	7.2162	1146.15	41.1818
48373	7.2164	1182.8101	145.135

48374	7.2165	1391.01	24.4411
48375	7.2166	1243.23	150.19
48376	7.2166	863.285	54.2521
48377	7.2168	1326.46	195.533
48378	7.2177	1278.55	97.2615
48379	7.218	1114.58	61.3349
48380	7.2182	1144.25	37.2002
48381	7.2187	1397.84	12.441
48382	7.2194	1267.39	16.7683
48383	7.2194	1243.38	115.636
48384	7.2195	1080.9399	195.297
48385	7.2201	1176.9301	221.463
48386	7.2202	1134.84	83.6577
48387	7.2205	1289.66	155.907
48388	7.2206	1275.28	268.923
48389	7.2211	1384.53	260.314
48390	7.2225	1278.5	295.317
48391	7.2228	1230.34	61.7103
48392	7.2238	1436.54	142.647
48393	7.2245	1165.7	192.07
48394	7.225	1135.99	91.7347
48395	7.2253	1264.95	202.882
48396	7.2258	1253.41	12.502
48397	7.2259	1192.35	39.4464
48398	7.2264	1225.53	141.928
48399	7.2268	1212.89	29.8462
48400	7.2268	1255.79	112.551
48401	7.2271	1169.24	49.9582
48402	7.2279	1275.6	27.447
48403	7.228	1118.8101	146.866
48404	7.2294	1445.26	27.1613
48405	7.231	1209.53	41.1527
48406	7.2312	1330.48	140.358
48407	7.2313	1370.46	23.417
48408	7.2315	1265.1899	184.762
48409	7.2317	1191.5699	224.652
48410	7.2319	1288.15	243.969
48411	7.2327	929.932	54.3143
48412	7.2333	1340.14	27.3575
48413	7.2333	1007.46	133.362
48414	7.2334	1108.95	75.0248
48415	7.2339	1110.78	250.677
48416	7.2345	998.165	253.897
48417	7.2348	1282.38	173.383
48418	7.2358	1194.83	142.515
48419	7.2361	1127.0699	179.574
48420	7.2365	1217.54	156.647
48421	7.2367	1142	84.2536

48422	7.2368	1171.0601	3.7854
48423	7.2371	1277.8199	13.1562
48424	7.2372	1207.4	126.905
48425	7.2379	1151.66	145.086
48426	7.2391	1134.66	150.962
48427	7.2392	1085.6	155.095
48428	7.2399	1274.38	151.226
48429	7.2405	1019.75	258.06
48430	7.2413	1437.1801	78.233
48431	7.2415	1389.92	19.47
48432	7.2422	1212.85	305.258
48433	7.2429	964.974	256.327
48434	7.2441	1222.1801	160.232
48435	7.2443	1121.24	109.182
48436	7.2449	1282.3101	258.577
48437	7.2449	1138.5699	340.749
48438	7.2451	1268.1801	77.6886
48439	7.2455	1357.74	282.827
48440	7.2463	1077.84	32.9344
48441	7.2466	1202.9399	75.8472
48442	7.2466	1051.26	188.109
48443	7.2469	1301.1801	143.209
48444	7.2472	800.529	24.5981
48445	7.2475	1324.95	92.0428
48446	7.2476	1081.36	108.539
48447	7.2477	1239.47	32.9985
48448	7.2481	1115.02	239.666
48449	7.2486	1092.0601	182.394
48450	7.2488	1213.9399	194.128
48451	7.2489	1277.74	294.862
48452	7.249	1244.73	78.3411
48453	7.2492	1186.55	32.9358
48454	7.2499	1444.12	225.684
48455	7.25	1186.22	177.111
48456	7.2508	1137.28	99.1525
48457	7.2512	1285.47	12.146
48458	7.2524	1249.36	118.012
48459	7.253	1339.51	38.9737
48460	7.2532	1432.97	227.76
48461	7.2534	1103.4	214.58
48462	7.254	1121.79	60.3317
48463	7.2541	1324.67	121.396
48464	7.2542	1238.54	49.0772
48465	7.2543	1172.34	126.7
48466	7.2544	1241.66	264.261
48467	7.2547	1174.28	91.8163
48468	7.2555	106.844	22.8889
48469	7.2562	1344.47	21.2099

48470	7.2563	1244.47	78.9106
48471	7.2564	1041.9399	43.1464
48472	7.2567	1143.47	99.2345
48473	7.2568	959.966	297.702
48474	7.2569	1265.48	14.1145
48475	7.2571	1219.67	80.6055
48476	7.2574	1087.3101	269.048
48477	7.2581	1128.08	171.089
48478	7.2582	1393.99	27.2832
48479	7.2582	1230.3	193.975
48480	7.2583	1167.36	154.987
48481	7.2589	1085.88	237.784
48482	7.2589	1136.58	172.165
48483	7.2598	1205.02	75.7843
48484	7.2602	1276.27	69.7405
48485	7.2605	1242.49	148.53
48486	7.2608	1364.98	93.2229
48487	7.2611	1136.6801	108.78
48488	7.2613	1360.3199	17.6911
48489	7.2617	1170.66	33.0159
48490	7.2619	1130.77	105.402
48491	7.2623	1363.52	98.1084
48492	7.2627	1072.5601	168.184
48493	7.2628	1113.01	215.432
48494	7.263	1321.24	223.819
48495	7.2634	1256.28	231.849
48496	7.264	1411.83	91.9728
48497	7.2645	1222.17	250.781
48498	7.2653	1037.88	271.426
48499	7.2659	1118.8	94.571
48500	7.2661	1243.83	82.7302
48501	7.2663	1265.02	181.873
48502	7.2665	1140.89	167.5
48503	7.2666	1147.25	50.8992
48504	7.2666	1318.9	228.221
48505	7.2675	1142.0601	281.875
48506	7.2677	1188.59	346.59
48507	7.2678	1114.33	49.1023
48508	7.2679	1448.1	12.4464
48509	7.2682	1221.47	76.7808
48510	7.2682	1149.13	83.402
48511	7.2688	1187.74	189.295
48512	7.2697	1242.16	142.441
48513	7.2699	1096.75	4.2273
48514	7.2703	1326.65	71.9935
48515	7.2706	1247.75	279.762
48516	7.2708	1446.71	22.4386
48517	7.2717	1257.9301	54.9087

48518	7.2722	1092.5699	21.5851
48519	7.2726	1231.75	79.8733
48520	7.2731	1201.71	299.825
48521	7.2744	844.811	80.7845
48522	7.2745	1349.17	23.9036
48523	7.2745	1072.83	261.021
48524	7.2751	1129.98	72.9255
48525	7.2751	1108.96	132.412
48526	7.2753	1306.8199	358.716
48527	7.2753	1154.48	225.642
48528	7.2758	1407.65	24.8362
48529	7.2769	1242.01	146.956
48530	7.2772	1413.3	28.7627
48531	7.2775	1236.34	4.061
48532	7.2778	1166.67	35.8048
48533	7.2787	1171.71	247.471
48534	7.279	1032.01	172.525
48535	7.2795	1119.0601	249.236
48536	7.2798	1211.54	13.803
48537	7.2811	1175.3199	164.77
48538	7.2815	1321.87	37.7015
48539	7.2822	1147.96	301.55
48540	7.2823	1191.25	262.01
48541	7.2824	1202.0699	28.4633
48542	7.2827	1238.25	223.867
48543	7.2831	1391.28	29.6887
48544	7.2832	1359.33	68.7256
48545	7.2837	1250.71	321.159
48546	7.2838	1204.9399	152.617
48547	7.2842	1145.13	14.528
48548	7.2864	1177.4	242.622
48549	7.2864	1077.42	301.099
48550	7.2868	1140.65	265.799
48551	7.287	1386.36	78.8007
48552	7.287	1110.38	76.9393
48553	7.2873	1123.38	153.468
48554	7.2874	1097.16	85.5523
48555	7.2875	1225.7	114.687
48556	7.2878	1452.36	98.2405
48557	7.2882	1132.28	171.785
48558	7.2883	1288.1801	52.0231
48559	7.2883	1234.1899	108.298
48560	7.2883	1187.77	77.7983
48561	7.2895	1239.26	314.313
48562	7.29	1296.24	141.772
48563	7.2901	1090.66	237.078
48564	7.2905	1279.11	314.716
48565	7.2912	1220.91	220.958

48566	7.2915	1284.72	255.416
48567	7.2915	1466.23	20.5418
48568	7.2916	1175.61	80.9138
48569	7.2932	1151.29	194.396
48570	7.2932	1297.15	76.8525
48571	7.294	1120.48	256.313
48572	7.2943	1141.8199	97.8543
48573	7.2948	1193.88	22.1908
48574	7.2948	1185.52	97.1516
48575	7.2949	1228.48	34.2108
48576	7.2959	1074.84	163.198
48577	7.2964	1368.54	37.8478
48578	7.2966	1084.13	119.048
48579	7.2975	1162.75	197.236
48580	7.2988	1330.87	27.9121
48581	7.2999	1366.96	106.106
48582	7.3003	1306.8101	43.0138
48583	7.3006	1300.23	221.836
48584	7.3009	1154.16	194.608
48585	7.301	1045.29	184.553
48586	7.3022	1195.47	194.316
48587	7.3026	855.546	80.8497
48588	7.303	1391.14	18.5951
48589	7.3032	1403.05	114.614
48590	7.3032	1115.35	193.943
48591	7.3033	445.291	47.4891
48592	7.3034	1041.52	117.315
48593	7.3042	1290.51	113.391
48594	7.3045	918.378	191.709
48595	7.3053	1168.87	146.03
48596	7.3054	1421.3199	68.6296
48597	7.306	1224.52	103.544
48598	7.3069	1129.46	179.312
48599	7.3071	1132.9399	173.559
48600	7.3072	1365.51	136.866
48601	7.3076	1220.13	58.6617
48602	7.3077	1165.51	296.231
48603	7.3077	1349.92	156.885
48604	7.3079	1343.46	23.0472
48605	7.3082	1378.29	21.2492
48606	7.3082	1060.67	181.459
48607	7.3083	1357.28	119.848
48608	7.3089	1056.3101	115.09
48609	7.3094	1151.7	150.889
48610	7.3099	1178.26	64.396
48611	7.3103	1205.64	68.4829
48612	7.3103	1140.98	163.548
48613	7.3111	1179.37	171.993

48614	7.3111	1213.01	153.245
48615	7.3114	1373.38	87.3905
48616	7.3114	1446.9399	99.272
48617	7.3117	1214.59	96.1962
48618	7.3133	1447.72	24.1461
48619	7.3135	1335.5699	147.28
48620	7.3135	1234.62	78.8509
48621	7.3144	869.603	55.9282
48622	7.3146	1401.53	25.1985
48623	7.3146	1091.09	234.036
48624	7.3146	1281.38	19.3374
48625	7.3156	1305.1801	151.426
48626	7.3161	1102.87	220.172
48627	7.3164	1188.3	166.306
48628	7.3164	957.037	118.772
48629	7.3171	1104.59	106.092
48630	7.3178	1261.53	145.288
48631	7.3179	1264.99	22.8024
48632	7.3183	1109.2	244.354
48633	7.3187	1157.62	152.15
48634	7.3191	918.368	192.925
48635	7.3198	1361.41	29.9415
48636	7.3199	1177.35	329.443
48637	7.321	1353.35	33.5017
48638	7.3228	1088.6899	235.584
48639	7.3245	1193.0699	249.109
48640	7.3247	1169.61	108.035
48641	7.3249	1337.92	187.003
48642	7.3249	1031.51	133.786
48643	7.3255	1276.61	215.154
48644	7.3255	1180.1801	173.643
48645	7.3263	1265.38	273.09
48646	7.3276	1308.96	29.2563
48647	7.3277	1075.0699	15.2813
48648	7.3279	1174.2	172.827
48649	7.328	1540.8	21.1835
48650	7.3288	1325.16	304.208
48651	7.329	1157.15	3.9024
48652	7.3293	1130.4399	5.0443
48653	7.3302	1523.22	24.8703
48654	7.3305	1045.88	252.765
48655	7.3311	1151.3101	48.2435
48656	7.3313	1208.27	117.624
48657	7.3314	1104.39	164.882
48658	7.3316	1345.41	7.4941
48659	7.3318	1361.99	40.8487
48660	7.3334	1255.78	118.935
48661	7.3335	1168.13	82.9631

48662	7.3337	1216.74	80.7112
48663	7.3343	1047.17	250.193
48664	7.3344	1190.4399	217.901
48665	7.3348	1268.83	33.942
48666	7.3358	1307.99	53.4949
48667	7.3364	1216.01	18.0774
48668	7.3369	1243.25	294.231
48669	7.3378	1263.55	234.984
48670	7.3378	1431.53	111.329
48671	7.3382	1077.05	24.3495
48672	7.3384	1267.17	336.004
48673	7.3384	1111.4	214.924
48674	7.3386	1412.09	34.8051
48675	7.3396	1291.4301	34.4285
48676	7.3396	1147.45	135.753
48677	7.341	1117.67	186.636
48678	7.3412	1277.29	346.482
48679	7.3414	1500.09	23.1404
48680	7.3418	1481.89	24.7131
48681	7.3423	1402.6899	34.5352
48682	7.3443	1348.42	32.547
48683	7.3444	1306.3101	82.4666
48684	7.3444	1311.2	48.6909
48685	7.3449	1363.41	153.271
48686	7.3449	1122.75	101.416
48687	7.3455	1074.6801	135.846
48688	7.3456	1424.84	73.4924
48689	7.3458	1265.11	327.433
48690	7.3459	1264.61	217.567
48691	7.3465	1099.1801	218.486
48692	7.3472	1125.83	219.921
48693	7.3473	1235.6801	93.9382
48694	7.3478	1206.98	169.006
48695	7.3479	1130.59	34.4034
48696	7.3487	1148.04	76.5272
48697	7.3491	1331.02	146.165
48698	7.3494	1169.8	191.536
48699	7.3496	1451.0699	12.0685
48700	7.3497	1414.29	142.442
48701	7.35	1200.8101	75.9173
48702	7.3502	1166.4	260.118
48703	7.3512	596.674	310.559
48704	7.3527	1231.97	124.539
48705	7.3527	1430.0699	34.5326
48706	7.3533	1330.08	13.6846
48707	7.3534	1284.86	18.8251
48708	7.354	1351.39	121.327
48709	7.3548	1262.24	22.5133

48710	7.3555	1091.6899	303.192
48711	7.3566	1364.38	27.9644
48712	7.3574	1058.04	87.4485
48713	7.3575	1313.61	284.86
48714	7.3577	1318.05	224.828
48715	7.3583	1018.91	78.4763
48716	7.3586	1457.2	96.505
48717	7.3586	1114.92	16.0136
48718	7.3588	1178.5601	61.3207
48719	7.3591	1142.08	214.871
48720	7.3593	1186.64	313.287
48721	7.3594	1339.5	29.2258
48722	7.36	1032.67	28.4915
48723	7.3601	1191.5601	152.475
48724	7.3604	1224.71	3.3496
48725	7.3608	918.349	192.084
48726	7.3613	1144.88	109.398
48727	7.3614	1206.25	67.0225
48728	7.362	1219.1801	198.724
48729	7.3627	1270.71	171.602
48730	7.3636	1253.21	299.191
48731	7.364	1390.91	89.1178
48732	7.3643	1239.55	22.6868
48733	7.3646	1446.1801	22.4308
48734	7.3649	1395.77	23.5429
48735	7.3655	1279.41	91.6795
48736	7.3674	1364.2	318.948
48737	7.3677	1335.96	28.9891
48738	7.3679	1185.15	191.014
48739	7.3684	1235.15	154.94
48740	7.3687	1252.4	331.805
48741	7.3688	1277.59	13.6153
48742	7.3689	1292.54	102.152
48743	7.3689	1172.2	193.568
48744	7.3699	1402.75	25.9766
48745	7.3701	1291.03	118.867
48746	7.3701	1227	178.878
48747	7.3703	1298.5601	176.946
48748	7.3705	1371.15	36.5035
48749	7.3705	1027	314.173
48750	7.3715	1312.37	71.9838
48751	7.3716	1270.1	67.6335
48752	7.3717	1168.77	264.719
48753	7.3727	1323.0601	22.0361
48754	7.3734	1493.54	80.6762
48755	7.3738	1214.13	85.0486
48756	7.3748	1430.47	65.6669
48757	7.3748	1246.9399	226.319

48758	7.3754	1366.53	184.572
48759	7.3756	1305.12	30.0232
48760	7.376	1265.54	148.677
48761	7.3767	1512.95	19.0357
48762	7.3772	1406.45	43.7859
48763	7.3776	1201.72	115.985
48764	7.3777	1201.85	275.23
48765	7.3781	1134.59	105.341
48766	7.3781	1167.9301	76.9195
48767	7.3786	1226.3101	195.078
48768	7.3792	1085.08	254.63
48769	7.3798	1337.14	241.214
48770	7.3802	1322.02	76.4049
48771	7.3803	1395.78	23.6634
48772	7.3806	1200.84	177.714
48773	7.3807	1268.73	83.0831
48774	7.3808	1130.54	250.285
48775	7.3811	1132.58	170.492
48776	7.3814	1331.28	77.4694
48777	7.3818	1160.86	96.5051
48778	7.3826	1425.33	26.792
48779	7.383	1193.03	58.4757
48780	7.3836	1247.37	74.6931
48781	7.3839	1293.6801	77.9783
48782	7.3846	1373.4399	96.2047
48783	7.3846	1274.73	30.1798
48784	7.3858	1149.9	154.638
48785	7.386	1283.5	23.9344
48786	7.3867	1585.05	15.5589
48787	7.3868	1387.62	113.973
48788	7.3896	1072.61	195.307
48789	7.3896	1231.66	80.3517
48790	7.3902	1159.8	165.574
48791	7.3918	1161.59	94.2021
48792	7.393	1103.13	196.892
48793	7.3944	1256.61	86.9176
48794	7.395	1397.3199	82.8479
48795	7.3954	651.961	50.796
48796	7.3964	646.475	46.6288
48797	7.3965	1341.95	7.5249
48798	7.3982	1254.22	78.3102
48799	7.3988	1342.7	72.9709
48800	7.3994	1269.3101	314.917
48801	7.3995	1317.71	73.1778
48802	7.4	1226.15	86.0517
48803	7.4001	1215.76	141.448
48804	7.4003	1486.76	17.4469
48805	7.4004	1428.13	232.086

48806	7.4005	1187.5699	156.82
48807	7.4006	1349.86	13.0536
48808	7.401	1192.9399	72.7528
48809	7.4013	1347	14.0768
48810	7.4021	1363.11	114.501
48811	7.4026	1121.4	174.266
48812	7.4037	1149.62	221.67
48813	7.4038	1221.28	141.064
48814	7.404	1340.58	34.9112
48815	7.4047	1224.14	13.8292
48816	7.4051	1215.84	177.332
48817	7.4053	1458.65	29.0839
48818	7.4055	1233.67	15.569
48819	7.4077	1111.08	198.272
48820	7.4082	1223.08	272.496
48821	7.4083	1196.8	310.352
48822	7.4093	1195.9301	273.928
48823	7.4096	1357.45	251.932
48824	7.4097	1280.65	229.848
48825	7.4103	1291.95	234.213
48826	7.4111	1208.87	38.4635
48827	7.4113	1114.09	31.8938
48828	7.4114	1139.63	44.9414
48829	7.4118	1262.78	240.922
48830	7.4119	1196.0699	111.387
48831	7.4121	1161.34	254.031
48832	7.4125	1292.86	258.287
48833	7.4126	1231.64	41.6025
48834	7.4127	1550.52	33.8535
48835	7.4132	1283.65	35.8622
48836	7.4151	1175.53	293.923
48837	7.4162	1147.21	307.236
48838	7.4162	1116.6	182.034
48839	7.4166	1107.28	252.004
48840	7.417	1285.65	33.9306
48841	7.4178	1137.4399	116.51
48842	7.4178	1283.91	55.7584
48843	7.4179	-3.1643	7.0658
48844	7.418	1425.1	128.042
48845	7.4181	1411.6801	134.429
48846	7.4186	1319.67	21.9714
48847	7.419	1286.97	16.9366
48848	7.4191	1349.05	75.5308
48849	7.4196	1165.9301	28.1833
48850	7.4196	1376.55	222.161
48851	7.4198	1373.0601	77.224
48852	7.4198	1357.14	12.9069
48853	7.4208	482.724	330.623

48854	7.4211	1300.79	119.36
48855	7.4212	1226.35	188.18
48856	7.4216	1231.46	2.3156
48857	7.422	1141.05	184.161
48858	7.4224	1284.03	331.276
48859	7.4233	1323.24	23.5891
48860	7.4236	1163.29	79.0378
48861	7.4245	1096.49	172.146
48862	7.4246	1342.0699	127.62
48863	7.4263	1183.47	170.536
48864	7.4263	1501.5	226.314
48865	7.4268	1451.01	284.028
48866	7.427	1163.6899	298.225
48867	7.427	1131.55	128.104
48868	7.4274	1274.04	145.173
48869	7.4275	1374.8199	97.1995
48870	7.4277	1102.87	115.644
48871	7.4278	1141.77	308.092
48872	7.4287	1150.38	170.966
48873	7.4299	1199.3101	165.04
48874	7.43	1361.92	152.488
48875	7.4313	1245.42	200.032
48876	7.4328	1481.79	25.1299
48877	7.4329	1179.46	249.439
48878	7.4335	1073.23	141.619
48879	7.4342	1429.77	90.7547
48880	7.4345	1141.76	249.623
48881	7.4349	1527	97.3926
48882	7.4354	1269.1899	149.764
48883	7.4367	1371.77	1.2247
48884	7.4372	1194.21	32.2616
48885	7.4372	1354.97	12.8722
48886	7.4372	1220.27	295.664
48887	7.4376	1188.83	132.221
48888	7.4389	1242.54	225.979
48889	7.439	1032.14	47.9075
48890	7.4392	1359.46	91.3476
48891	7.4404	1198.96	159.595
48892	7.4407	1216.27	225.958
48893	7.4412	1288.72	77.9481
48894	7.4416	1547.01	105.275
48895	7.4422	1097.3101	24.0709
48896	7.443	1122.71	85.4755
48897	7.4444	1312.75	321.737
48898	7.4447	1407.52	281.686
48899	7.4465	1343.88	345.29
48900	7.4473	1173.27	217.991
48901	7.4473	1228.45	172.791

48902	7.4475	1283.65	302.186
48903	7.448	1232.64	112.397
48904	7.449	1218.8199	32.8663
48905	7.4493	1243.89	20.2248
48906	7.4496	1482.95	25.406
48907	7.4498	1138.04	165.651
48908	7.4505	1262.0601	97.6349
48909	7.4511	1297.4	71.9752
48910	7.4525	1274.45	12.5741
48911	7.4528	1369.1	25.1571
48912	7.4535	1431.59	151.123
48913	7.454	1240.47	145.98
48914	7.4548	809.792	18.1025
48915	7.4549	1104.83	218.75
48916	7.4583	1347.3199	181.028
48917	7.4587	1242.84	96.9043
48918	7.4598	1474.27	12.5653
48919	7.4601	1191.8199	2.0574
48920	7.4608	1578.15	20.0016
48921	7.4612	1187.6899	164.843
48922	7.4615	1343.36	40.3518
48923	7.4618	1375.63	56.1454
48924	7.4621	1273.73	79.3177
48925	7.4621	1145.63	57.3305
48926	7.4622	1227.6	301.439
48927	7.4649	1462.41	45.064
48928	7.4663	1219.42	192.977
48929	7.4667	1139.48	23.9568
48930	7.4668	1183.3101	252.829
48931	7.4668	1184.08	196.118
48932	7.4669	1369.33	72.6863
48933	7.4673	1413.03	20.9391
48934	7.4678	1361.79	109.818
48935	7.4693	1099.58	116.987
48936	7.4709	1355.21	256.793
48937	7.4722	1164.6801	103.094
48938	7.4725	1212.03	134.495
48939	7.4732	1286.11	274.705
48940	7.4736	1390.33	188.156
48941	7.4743	1300.53	124.141
48942	7.4745	1303.28	82.1988
48943	7.4751	1136.37	83.0848
48944	7.4754	1198.33	223.595
48945	7.4763	1338.9301	257.481
48946	7.4765	1292.47	150.272
48947	7.4768	1429.85	21.5544
48948	7.477	1207.8	182.031
48949	7.4771	1386.4	140.827

48950	7.4776	1240.3	191.585
48951	7.4779	1363.76	183.199
48952	7.4779	1377.37	12.2346
48953	7.4805	1314.26	24.0765
48954	7.4818	1365.8	318.09
48955	7.4825	1488.5601	20.9936
48956	7.4839	1243.4	89.6036
48957	7.4847	1219.65	70.7776
48958	7.485	1438.64	115.647
48959	7.4852	1466.61	102.219
48960	7.4853	1444.6801	22.7837
48961	7.4853	1204.61	97.5886
48962	7.4857	1226.12	178.301
48963	7.4859	1337.65	93.2347
48964	7.4861	1311.08	14.172
48965	7.4864	1128.04	250.649
48966	7.4873	1326.59	275.847
48967	7.4875	1391.27	42.5084
48968	7.4875	1179.5699	195.042
48969	7.4877	1218.52	306.019
48970	7.4882	1278.78	38.9514
48971	7.489	1057.23	252.988
48972	7.489	1175.42	41.3023
48973	7.4893	1432.6	233.558
48974	7.4896	1310.0699	279.889
48975	7.4898	1444.45	100.331
48976	7.4899	1280.15	43.4069
48977	7.4902	1335.73	77.7169
48978	7.4905	1291.1801	285.867
48979	7.4906	1464.67	252.134
48980	7.4912	1326.8	152.747
48981	7.4914	1216.8101	10.9347
48982	7.4933	1389.33	248.825
48983	7.4953	1217.79	261.048
48984	7.4957	1357.42	13.6779
48985	7.4958	1167.39	164.474
48986	7.4964	1482.8101	288.528
48987	7.4968	1354.38	142.877
48988	7.4975	1477.35	107.223
48989	7.4976	1313.6899	325.469
48990	7.4981	1472.59	20.7075
48991	7.4983	1326.03	67.9591
48992	7.4986	1499.1801	58.6364
48993	7.499	1235.86	248.676
48994	7.499	1235.0601	181.977
48995	7.5002	1348.3	191.568
48996	7.5009	1121.54	76.5817
48997	7.5017	1178.3199	6.4774

48998	7.5017	1431.83	127.926
48999	7.5025	1348.98	225.991
49000	7.5046	1299.67	278.054
49001	7.5048	1258.8	218.812
49002	7.5048	1442.61	11.7235
49003	7.5052	1200.74	228.463
49004	7.5054	1289.67	198.046
49005	7.5057	1136.83	139.027
49006	7.5071	1214.21	24.0185
49007	7.5072	1190.76	75.9515
49008	7.5074	1317.4301	76.4033
49009	7.5076	1189.49	144.143
49010	7.5079	1307.26	194.447
49011	7.5084	900.403	58.165
49012	7.5086	1434.21	1.05
49013	7.5087	1081.5	136.404
49014	7.509	1320.4	121.324
49015	7.5102	1213.04	147.993
49016	7.5105	1170.38	106.99
49017	7.5107	1173.36	58.0274
49018	7.5112	1241.62	238.55
49019	7.5113	1360.64	77.72
49020	7.5118	1269.76	169.779
49021	7.5119	1186.8101	235.675
49022	7.512	1197.48	298.625
49023	7.5121	1185.08	196.343
49024	7.5129	1377.76	30.2935
49025	7.5152	1251.26	32.3429
49026	7.516	1280.67	57.3863
49027	7.5162	1244	157.52
49028	7.5165	1426.83	34.3467
49029	7.517	1265.65	77.6992
49030	7.5176	1248.11	250.037
49031	7.5181	1444.55	37.8502
49032	7.5185	1302.27	162.324
49033	7.5187	1279.6899	32.4918
49034	7.5198	1391.64	254.349
49035	7.5199	1297.45	21.1832
49036	7.5203	1524.11	19.8082
49037	7.521	1265.6899	137.93
49038	7.5216	1581.58	334.985
49039	7.5219	1184.17	175.832
49040	7.522	1215.88	68.1763
49041	7.5223	1229.8199	19.2517
49042	7.5231	1224.4399	29.5323
49043	7.5233	1501.29	22.4613
49044	7.5236	1268.6899	189.899
49045	7.5243	1168.4399	2.7605

49046	7.5246	1310.55	98.0583
49047	7.5273	1236	176.265
49048	7.5273	1297.45	265.533
49049	7.5276	1586.4399	253.221
49050	7.5283	1311.11	2.1457
49051	7.5285	1469.14	6.1624
49052	7.529	1182.4399	186.664
49053	7.5294	1332.78	267.695
49054	7.5296	1296.4399	82.3239
49055	7.5298	1413.53	102.001
49056	7.5304	1281.53	31.3296
49057	7.5306	1450.78	229.017
49058	7.5319	1294.7	136.592
49059	7.5323	1317.83	18.6412
49060	7.5331	1197.61	235.242
49061	7.5335	1305.16	57.0903
49062	7.5354	1210.1801	259.051
49063	7.5365	1290.74	90.5037
49064	7.5367	1195.17	293.976
49065	7.5376	1266.78	173.513
49066	7.5381	1272.73	140.72
49067	7.5392	1504.76	149.908
49068	7.5396	1385.75	184.87
49069	7.5396	1335.9301	216.817
49070	7.5397	1270	358.543
49071	7.5397	1439.3101	150.522
49072	7.5397	1522.1	27.2822
49073	7.54	1419.25	96.3491
49074	7.5403	1339.3199	71.557
49075	7.5406	1379.01	78.4143
49076	7.541	1502.0601	22.6984
49077	7.5413	1356.7	14.2437
49078	7.542	1330.5601	280.971
49079	7.542	1406.0699	26.4208
49080	7.5432	1287.88	177.031
49081	7.5434	1138.04	183.462
49082	7.5434	1274.1801	253.666
49083	7.5434	1248.97	82.4748
49084	7.5437	1138.33	244.124
49085	7.5439	1417.98	106.462
49086	7.5473	1463.8101	129.422
49087	7.5483	918.4	191.424
49088	7.5486	1172.52	235.993
49089	7.5494	1584.13	258.613
49090	7.5499	1281.88	55.3635
49091	7.55	1334.47	277.554
49092	7.5504	1573.14	59.6667
49093	7.5505	1182.73	194.586

49094	7.5511	1227.53	236.996
49095	7.5512	1316.92	217.728
49096	7.5517	1338.55	222.463
49097	7.5527	1242.92	14.3228
49098	7.5531	1256.48	190.558
49099	7.5532	1209.84	199.911
49100	7.5547	1243.76	26.8158
49101	7.555	1268.59	73.5756
49102	7.5551	1217.65	2.6416
49103	7.5556	1244.48	255.482
49104	7.5556	1199.7	68.2124
49105	7.5558	1394.34	190.525
49106	7.5571	1387.4301	177.861
49107	7.5572	1353.55	79.0747
49108	7.5574	1248.66	22.4511
49109	7.5581	1431.47	128.8
49110	7.5584	1180.28	317.101
49111	7.5587	1371.61	55.8465
49112	7.5597	1282.01	83.6917
49113	7.5598	1462.75	100.52
49114	7.5613	1400.8199	124.621
49115	7.5619	1330.24	24.1371
49116	7.563	1171.41	196.413
49117	7.5633	1452.64	26.8352
49118	7.5633	1313.53	144.099
49119	7.5634	1362.33	288.808
49120	7.5637	1168.33	307.064
49121	7.5643	1363.67	22.1834
49122	7.5643	1094.58	34.9085
49123	7.5649	1059.95	31.7534
49124	7.5654	1427.36	30.6148
49125	7.5669	1464.49	222.943
49126	7.567	1239.51	173.384
49127	7.567	1190.52	18.5927
49128	7.5677	1283.27	16.2519
49129	7.5683	1333.1899	70.3556
49130	7.5683	1397.61	111.831
49131	7.5685	1405.64	159.943
49132	7.5687	1387.7	105.288
49133	7.5688	1252.83	34.5453
49134	7.5688	1228.49	153.665
49135	7.5696	1315.64	3.2131
49136	7.5701	1621.99	13.7663
49137	7.5708	1364.99	222.829
49138	7.5711	1238.34	149.207
49139	7.5728	1329.54	163.354
49140	7.573	1387.4	71.4669
49141	7.5735	1223.26	200.9

49142	7.5742	1268.88	170.812
49143	7.5742	1423.92	55.9479
49144	7.5744	1552.4	24.2104
49145	7.5755	1295.77	23.3341
49146	7.5757	1241.65	0.1503
49147	7.5761	1239.73	156.866
49148	7.5761	1262.28	22.4827
49149	7.5764	1501.27	113.373
49150	7.5768	1349.48	86.9394
49151	7.5779	1380.66	70.1497
49152	7.578	1397.36	46.1918
49153	7.5784	1296.4	108.331
49154	7.579	1357.62	106.299
49155	7.5797	1128.89	196.561
49156	7.5801	1526.97	112.753
49157	7.5803	1172.0601	31.4947
49158	7.5806	1299.53	138.706
49159	7.5807	1387.85	12.564
49160	7.5821	1337.22	84.3111
49161	7.5827	1342.33	30.7361
49162	7.5828	1587.1801	8.583
49163	7.5837	1285.5	199.216
49164	7.5842	1319.4301	117.815
49165	7.5843	1178.28	246.92
49166	7.5847	1383.75	46.9438
49167	7.5857	1582.63	74.675
49168	7.5868	1375.24	221.988
49169	7.5871	1193.36	253.863
49170	7.5872	1248.05	191.912
49171	7.5875	1416.73	295.427
49172	7.5878	1224.47	217.132
49173	7.5882	1425.02	232.451
49174	7.5883	1641.7	42.5915
49175	7.5903	1354.73	83.7603
49176	7.5909	1247.48	199.226
49177	7.591	1333.65	95.9705
49178	7.5912	918.249	191.134
49179	7.5922	1688.9399	42.3971
49180	7.5926	1320.71	75.8497
49181	7.5932	1377.21	109.998
49182	7.5944	1350.1899	192.001
49183	7.5955	1336.8	15.8988
49184	7.5965	1263.5	239.428
49185	7.5968	1288.6	118.234
49186	7.5968	1586.46	100.819
49187	7.5976	1281.35	230.648
49188	7.5993	1527.53	100.375
49189	7.5996	1132.65	196.029

49190	7.6021	1345.85	234.409
49191	7.6022	1308.53	13.4517
49192	7.6025	1336.71	142.991
49193	7.6027	1395.61	186.033
49194	7.603	617.752	48.411
49195	7.6035	1157.29	303.464
49196	7.6036	1328	51.2084
49197	7.6037	1033.05	123.011
49198	7.606	1414.55	28.7978
49199	7.6062	1154.95	79.3876
49200	7.6072	1424.49	82.5085
49201	7.6073	1535.24	23.4348
49202	7.6076	1353.37	114.714
49203	7.608	1068.99	65.9366
49204	7.6091	1254.84	84.7709
49205	7.6097	1350.76	93.7357
49206	7.6101	1298.75	256.025
49207	7.6107	1541.39	28.5551
49208	7.6113	1238.0699	17.8755
49209	7.612	1303.76	111.183
49210	7.6123	1252.3	225.299
49211	7.6143	1384.53	12.3576
49212	7.6145	1366.83	304.299
49213	7.615	918.669	192.62
49214	7.6151	1128.5699	257.123
49215	7.6152	1258.11	197.602
49216	7.6174	1501.9	113.636
49217	7.6201	1378.12	35.5099
49218	7.6201	1384.26	76.1254
49219	7.6202	1309.4	162.977
49220	7.6205	1309.27	176.586
49221	7.621	1424.05	358.752
49222	7.622	1285.36	299.986
49223	7.6226	1369.7	53.2921
49224	7.6228	1301.83	20.298
49225	7.6233	1358.15	172.552
49226	7.6238	1310.17	248.785
49227	7.6247	1197.13	255.994
49228	7.6248	1407.51	226.791
49229	7.6252	1507.74	80.4475
49230	7.6271	1228.84	170.138
49231	7.6286	1589.05	29.0633
49232	7.6287	1385.85	194.491
49233	7.6287	1278.77	170.401
49234	7.6299	1244.73	249.429
49235	7.6302	1321.88	14.3588
49236	7.6304	1431.84	86.8395
49237	7.6307	1303.62	146.127

49238	7.6315	1485.78	29.7625
49239	7.6324	1275.65	169.601
49240	7.6327	1355.9301	117.766
49241	7.6332	1358.7	42.24
49242	7.6335	1387.8101	104.966
49243	7.6338	1427.33	27.3653
49244	7.6344	1213.52	157.889
49245	7.6349	1452.15	96.676
49246	7.6368	1299.05	303.143
49247	7.6368	1374.23	143.846
49248	7.6377	1407.9399	138.979
49249	7.6409	1526.89	20.0805
49250	7.6413	1576.14	255.27
49251	7.6414	1397.12	74.3724
49252	7.6415	1213.34	254.548
49253	7.6417	1373.09	28.2326
49254	7.6445	1431.52	258.814
49255	7.6448	1368.53	19.7023
49256	7.6451	1291.9399	235.579
49257	7.6453	1476.1899	15.075
49258	7.6453	1523.7	113.899
49259	7.6454	1285.09	147.39
49260	7.6459	1468.28	105.413
49261	7.646	1402.22	8.2749
49262	7.6464	1476.58	99.5774
49263	7.6466	1458.15	136.718
49264	7.6468	1363.3199	98.1293
49265	7.6471	1531.51	59.633
49266	7.6481	1534.64	15.2746
49267	7.65	1457.1801	110.178
49268	7.6506	1265.91	197.103
49269	7.6507	1346.21	20.3834
49270	7.651	1330.9	126.236
49271	7.6515	1416.53	72.6872
49272	7.6519	1436.39	61.3481
49273	7.6526	1340.21	15.3441
49274	7.6533	1394.3199	85.0832
49275	7.6535	1325.34	319.087
49276	7.6539	1459.7	28.0991
49277	7.655	1006.93	63.7901
49278	7.6553	1519.76	99.0198
49279	7.6554	1584.62	17.128
49280	7.6565	1204.99	145.877
49281	7.6575	918.335	191.734
49282	7.6583	1386.01	15.472
49283	7.6596	1442.9301	23.4983
49284	7.6597	1208.4301	32.2837
49285	7.6606	1203.77	18.9367

49286	7.6613	918.333	190.516
49287	7.662	1272.5699	199.693
49288	7.6631	1413.6801	185.26
49289	7.6632	1450.17	46.9557
49290	7.6637	1206.8	257.278
49291	7.6642	1344.0699	176.883
49292	7.6654	1333.17	254.024
49293	7.6658	1463.49	69.633
49294	7.6663	1272.6	83.2205
49295	7.6665	1492.78	122.427
49296	7.6666	1396.26	224.368
49297	7.6668	1274.3199	14.8276
49298	7.6685	1249.2	273.546
49299	7.6686	1362.6801	86.4492
49300	7.6687	1278.38	180.968
49301	7.669	1584.09	21.4527
49302	7.669	1510.75	22.9601
49303	7.6713	1519.89	28.2927
49304	7.6718	1208.45	85.1205
49305	7.672	1352.49	269.243
49306	7.6722	1295.36	54.928
49307	7.6727	1649.4	48.2686
49308	7.6731	1182.99	86.1323
49309	7.6732	1467.85	135.026
49310	7.6736	1427.03	280.914
49311	7.6738	1459.04	275.832
49312	7.675	1270.11	77.9734
49313	7.6753	1333.21	108.092
49314	7.6757	652.382	47.7017
49315	7.6757	1250.91	169.793
49316	7.6762	1294.17	273.023
49317	7.6764	1348.49	67.1256
49318	7.6765	1308.38	46.7073
49319	7.6773	1287.78	192.986
49320	7.678	1355.83	164.814
49321	7.6792	1430.64	33.3833
49322	7.6804	1464.5601	69.7396
49323	7.6831	1277.02	235.955
49324	7.6846	1341.9399	223.02
49325	7.6851	1366.83	21.3721
49326	7.6852	1372.29	99.7512
49327	7.6859	1342.1899	116.12
49328	7.6865	1399.02	64.2568
49329	7.6866	1320.59	11.7509
49330	7.6868	1311.9399	17.7733
49331	7.6872	1322.89	142.265
49332	7.6889	1261.88	25.045
49333	7.6891	1364.61	225.795

49334	7.6894	1452.8101	16.8958
49335	7.69	1476.42	287.993
49336	7.6905	1299.3	30.0826
49337	7.6908	1420.14	247.891
49338	7.6913	1119.36	26.4314
49339	7.6921	1241.87	60.0219
49340	7.6923	1418.45	52.0871
49341	7.6933	1368.99	13.6286
49342	7.6934	1343.97	194.743
49343	7.6947	1298.0601	248.963
49344	7.6947	1297.03	181.69
49345	7.6953	1275.7	0.607
49346	7.6958	1259.97	138.876
49347	7.6961	1422.62	92.2146
49348	7.6968	1378.9301	94.5305
49349	7.6986	1489.11	320.608
49350	7.6997	1332.34	29.7257
49351	7.7003	1423.33	123.646
49352	7.7013	1359.8	65.4586
49353	7.7026	1574.4	34.3707
49354	7.703	1498.4399	138.008
49355	7.7052	1312.4301	235.642
49356	7.7055	1421.8199	22.935
49357	7.7059	1527.1899	151.381
49358	7.706	1480.2	185.447
49359	7.7082	1203.62	296.883
49360	7.7083	1401.53	72.6152
49361	7.7086	1299.62	359.855
49362	7.7089	1277.85	12.5635
49363	7.709	1257.52	175.917
49364	7.7091	1404.54	77.7712
49365	7.7092	1486.63	186.977
49366	7.7094	1342.86	248.703
49367	7.71	1477.08	135.003
49368	7.7119	1393.91	216.861
49369	7.7122	1172.84	350.846
49370	7.7124	1592.64	22.9555
49371	7.7128	1229.9399	215.071
49372	7.7135	1323.0699	188.221
49373	7.7136	1233.63	166.605
49374	7.7146	1386.3	146.425
49375	7.7148	1316.3	160.987
49376	7.7162	1552.36	336.775
49377	7.7165	1461.42	74.1978
49378	7.7166	1428.08	283.163
49379	7.7171	1386.88	108.348
49380	7.7172	1493.77	74.8676
49381	7.7174	1470.17	69.1739

49382	7.7176	1479.09	22.5664
49383	7.718	1602.98	23.2778
49384	7.7182	1318.58	11.8825
49385	7.719	1273.14	186.52
49386	7.7198	1390.25	77.0898
49387	7.7198	1426.4399	122.888
49388	7.7199	1377.65	119.887
49389	7.7228	1353.96	128.59
49390	7.7234	1385.96	24.6377
49391	7.7235	1132.51	4.1893
49392	7.7238	1236.55	255.364
49393	7.7244	1142.97	147.433
49394	7.7266	1365.67	36.986
49395	7.7287	1355.97	283.91
49396	7.7297	1374.9399	257.279
49397	7.7309	1231.96	57.9927
49398	7.7311	1258.62	185.458
49399	7.7312	1339.42	44.2872
49400	7.7314	1381.14	255.071
49401	7.7322	918.341	191.961
49402	7.7324	1386.34	30.2629
49403	7.7324	1458.75	104.87
49404	7.7325	1361.21	171.717
49405	7.7328	1253.41	317.383
49406	7.7329	1468.1801	93.1817
49407	7.7329	1172.11	251.867
49408	7.7348	1501.48	242.895
49409	7.7349	1540.35	23.3733
49410	7.735	1477.95	144.01
49411	7.7352	1237.13	36.2507
49412	7.7362	1297.11	27.8403
49413	7.7368	1282.6801	82.9183
49414	7.7368	1490.4399	58.8592
49415	7.737	1311.91	178.873
49416	7.7375	1290	255.63
49417	7.7384	1263.8	240.052
49418	7.7395	1445.13	37.0964
49419	7.7397	1525.39	31.9838
49420	7.7401	1587.87	70.0956
49421	7.7402	1217.0699	53.8524
49422	7.7402	1330.8199	217.73
49423	7.7408	1543.2	23.0335
49424	7.7433	1324.45	154.358
49425	7.7436	1338.64	160.609
49426	7.7437	1453.2	31.44
49427	7.7441	1198.5699	250.027
49428	7.7454	1129.9399	357.535
49429	7.7462	1328.29	102.972

49430	7.7464	1536.88	4.8378
49431	7.7474	1411.16	183.933
49432	7.7494	1431.76	126.47
49433	7.7498	1227.88	173.248
49434	7.7499	1563.49	163.053
49435	7.7502	1280.1801	145.074
49436	7.7509	1361.15	159.772
49437	7.7512	1263.6	222.448
49438	7.7512	1327.11	78.1014
49439	7.7515	1342.46	11.8762
49440	7.7516	1365.9	1.2762
49441	7.752	1436.46	320.813
49442	7.7531	1486.61	32.831
49443	7.7538	1279.1899	196.715
49444	7.7551	1540.78	62.7028
49445	7.7551	1577.35	17.5
49446	7.7555	1259.45	159.259
49447	7.7556	1508.61	37.7845
49448	7.7567	1349.3199	169.106
49449	7.7569	1362.42	13.3789
49450	7.7572	1319.48	127.795
49451	7.7578	1496.4	277.811
49452	7.7582	1510.21	275.57
49453	7.7597	1435.35	103.143
49454	7.7599	1588.77	98.6526
49455	7.7615	1498.1801	270.699
49456	7.762	1291.87	78.6641
49457	7.7631	1313.03	176.293
49458	7.7632	1458	231.317
49459	7.7637	1632.88	257.031
49460	7.7639	1532.76	354.062
49461	7.765	1337.87	227.102
49462	7.7653	1444.22	90
49463	7.7654	1264.37	160.452
49464	7.7656	1475.12	279.34
49465	7.7658	1482.48	180.908
49466	7.7658	1492.13	339.901
49467	7.7662	1324.1801	52.5916
49468	7.7664	1584.49	99.5024
49469	7.7671	1458.47	22.6596
49470	7.7674	1448.73	82.9742
49471	7.7676	1362.47	164.407
49472	7.7678	1399.6801	71.3274
49473	7.7685	1298.3199	235.842
49474	7.7688	1372.17	98.0262
49475	7.7693	1376.64	226.758
49476	7.7694	1453.26	77.435
49477	7.7703	1353.3199	7.8853

49478	7.7709	1295.34	257.979
49479	7.7734	1332.51	254.188
49480	7.775	1639.9301	251.55
49481	7.7755	1253.09	216.564
49482	7.7755	1274.11	273.291
49483	7.7759	1369.47	36.0856
49484	7.7764	1440.59	71.1401
49485	7.7775	1402.3	84.8386
49486	7.7781	1475.61	23.0737
49487	7.7789	1410.91	190.601
49488	7.779	1209.6	250.334
49489	7.7798	1395.6801	68.0046
49490	7.7803	1279.9301	174.431
49491	7.7807	1397.4399	246.988
49492	7.7808	1341.59	191.899
49493	7.7809	1466.9399	72.7872
49494	7.7814	1382.66	171.323
49495	7.7817	1335.15	170.601
49496	7.7822	1613.0601	25.2932
49497	7.7828	1336.63	19.8319
49498	7.7833	922.109	190.559
49499	7.7834	1301.9399	234.692
49500	7.7844	1302.58	159.718
49501	7.7848	1371.25	91.3572
49502	7.785	1283.75	41.6288
49503	7.785	1406.9399	185.009
49504	7.7869	1476.04	277.651
49505	7.788	1603.47	15.7946
49506	7.7882	1265.48	77.8972
49507	7.7888	1499.11	23.219
49508	7.789	1380.91	162.634
49509	7.7895	1355.21	276.208
49510	7.7898	1392.76	264.178
49511	7.7905	1632.53	101.819
49512	7.7906	1363.04	293.9
49513	7.7913	1292.5699	177.679
49514	7.7943	1244.23	236.181
49515	7.7945	1438.79	134.785
49516	7.7957	1364.3	12.7181
49517	7.7968	1454.72	328.18
49518	7.7979	1308.78	236.65
49519	7.798	1025.64	87.5195
49520	7.7986	1378.37	83.2408
49521	7.7986	1258.3101	196.436
49522	7.7987	1559.46	7.8083
49523	7.7988	1446.64	182.146
49524	7.7994	1344.45	221.159
49525	7.7995	1317.14	217.413

49526	7.8003	1313.5	176.431
49527	7.8015	1505.21	90.501
49528	7.8029	1481.09	26.5881
49529	7.8032	652.391	51.3184
49530	7.8035	1384.7	107.656
49531	7.8037	1436.74	34.8586
49532	7.8044	1194.89	83.9371
49533	7.8044	1268.6	82.3683
49534	7.8057	1398.17	255.504
49535	7.8057	1309.84	236.781
49536	7.8059	1375.46	177.448
49537	7.8066	1294.24	175.622
49538	7.8067	1387.87	87.3162
49539	7.8108	1443.21	187.59
49540	7.811	1370.27	67.0471
49541	7.8125	1454.5601	187.332
49542	7.8127	1427.3199	325.597
49543	7.8131	1344.64	251.567
49544	7.8133	1562.17	137.103
49545	7.8144	1473.48	231.815
49546	7.8151	1310.09	147.751
49547	7.817	1334.73	78.2461
49548	7.8173	1325.48	185.004
49549	7.8181	1463.22	83.654
49550	7.8196	652.017	46.4608
49551	7.8198	1481.9301	29.5996
49552	7.8202	1425.21	186.078
49553	7.8209	1628.46	25.3234
49554	7.8211	1412.39	324.033
49555	7.8212	1552.0601	61.3432
49556	7.8232	1196.05	34.2643
49557	7.8234	1413.21	85.4543
49558	7.8239	1500.17	22.9416
49559	7.8258	1348.13	348.915
49560	7.827	1242.65	250.157
49561	7.8279	1214.4	174.845
49562	7.828	1407.48	85.7763
49563	7.8281	1261.54	160.193
49564	7.8286	1319.6899	176.549
49565	7.8287	1389.95	227.835
49566	7.8287	1432.29	234.262
49567	7.829	1416.3101	125.382
49568	7.8295	1456.76	79.4495
49569	7.8316	1265.13	147.22
49570	7.8359	1359.63	177.84
49571	7.8397	1272.17	49.1807
49572	7.8401	1409.41	247.54
49573	7.8419	1635.37	102.65

49574	7.8423	-3.9993	9.2511
49575	7.8432	1401.1	119.683
49576	7.8433	1318.28	248.843
49577	7.8437	1513.87	103.997
49578	7.844	1121.52	53.5684
49579	7.8453	918.32	189.887
49580	7.8455	1415.13	37.2006
49581	7.8461	1328.26	104.46
49582	7.8461	1431.15	119.716
49583	7.8468	1550.79	82.2454
49584	7.8478	1506.64	135.289
49585	7.8492	1339.3	173.478
49586	7.8494	1322.83	75.9153
49587	7.8512	1271.21	28.6775
49588	7.8517	1425.3199	113.301
49589	7.8535	1365.86	22.1227
49590	7.8537	1162.6899	137.549
49591	7.854	1467.73	79.2123
49592	7.854	1133.74	71.4268
49593	7.855	1326.3199	29.3014
49594	7.856	1354.48	217.023
49595	7.8561	1600.01	24.4807
49596	7.8566	1361.33	21.1979
49597	7.857	1506.99	30.8655
49598	7.8573	1511.79	25.2417
49599	7.8584	1408.34	18.1824
49600	7.8588	1518.33	84.2854
49601	7.8589	1302.46	314.091
49602	7.8595	1361.62	247.599
49603	7.8596	1486	220.224
49604	7.8599	1377.66	30.3867
49605	7.8612	1664.6899	25.2205
49606	7.8617	1526.25	26.8224
49607	7.8628	-3.662	7.5263
49608	7.863	1342.67	77.8897
49609	7.8637	1517.25	145.852
49610	7.8639	1382.1	81.9057
49611	7.8644	1399.66	217.291
49612	7.8646	1393.85	29.2918
49613	7.8647	1465.3	85.0933
49614	7.8647	1307.04	15.7846
49615	7.8654	1321.03	196.546
49616	7.8658	1352.28	147.18
49617	7.8678	1328.4	107.98
49618	7.8678	1422.53	66.5867
49619	7.8683	1230.5699	251.526
49620	7.869	1224.74	196.912
49621	7.8691	1387.1801	23.402

49622	7.8702	1405.9301	22.2458
49623	7.8704	1318.01	195.591
49624	7.8716	1475.01	258.693
49625	7.8719	454.106	44.5989
49626	7.8725	1295.08	84.6344
49627	7.8728	1394.41	272.563
49628	7.8731	1364.34	47.0028
49629	7.8738	1537.71	100.149
49630	7.8744	1385.42	174.493
49631	7.8744	1385.96	235.169
49632	7.8759	1652.34	18.0606
49633	7.8767	1301.64	20.4435
49634	7.8777	1370.96	32.372
49635	7.878	1575.71	81.1081
49636	7.8782	1344.98	216.37
49637	7.8789	1386.98	306.089
49638	7.8791	1356.3101	43.7663
49639	7.8792	483.018	329.368
49640	7.88	1442.97	87.3353
49641	7.8803	-3.2664	2.8134
49642	7.8803	1511.53	230.449
49643	7.8806	1367.04	80.8887
49644	7.8812	1385.75	309.686
49645	7.8841	1464.85	26.9578
49646	7.8848	1359.8101	74.6537
49647	7.8851	1418.85	0.536
49648	7.8852	1381.4	151.2
49649	7.8854	1342.64	75.1982
49650	7.8865	1247.74	85.2972
49651	7.8867	1587.98	286.544
49652	7.8867	1488.0601	229.451
49653	7.8874	1342.28	17.5098
49654	7.8897	1445.3199	233.991
49655	7.8909	1400.13	270.308
49656	7.8912	1476.5	143.969
49657	7.8916	1282.35	250.903
49658	7.8919	1575.27	21.4546
49659	7.8924	483.087	337.499
49660	7.8935	1352.4301	76.067
49661	7.8939	1471.1899	2.4432
49662	7.8964	1454.87	109.77
49663	7.8972	1385.63	145.299
49664	7.8974	1409.6	11.7555
49665	7.8975	1323.9399	28.1157
49666	7.8988	1338.46	5.9453
49667	7.899	1370.4301	83.4314
49668	7.8993	1508.91	353.365
49669	7.8995	1413.95	84.4693

49670	7.9019	1563.4399	62.0494
49671	7.9019	1377.78	250.482
49672	7.902	1521.8	103.841
49673	7.9039	1547.76	100.559
49674	7.904	1575.33	96.7576
49675	7.9043	1541.63	23.3746
49676	7.9044	1347.77	85.276
49677	7.9048	1550.21	19.9022
49678	7.9051	1324.08	162.194
49679	7.9058	1349.71	258.906
49680	7.906	1291.4	26.2254
49681	7.9066	1501.63	85.9592
49682	7.9067	1353.74	237.283
49683	7.9071	1422.22	78.7996
49684	7.9072	1660.88	75.5545
49685	7.9086	1310.7	256.457
49686	7.9088	1455.7	91.9406
49687	7.9092	1438.6801	191.551
49688	7.9093	1257.37	148.494
49689	7.9097	1384.8199	67.7448
49690	7.9104	1483.55	297.528
49691	7.9108	1361.4399	176.965
49692	7.9113	1579.47	92.8052
49693	7.9114	1354.47	196.299
49694	7.9117	1265.42	256.844
49695	7.9126	1318.26	19.9164
49696	7.9142	1647.37	29.5037
49697	7.915	1420.61	37.0141
49698	7.9156	1430.77	93.0301
49699	7.9179	1467.1899	47.6523
49700	7.9183	1495.53	84.7775
49701	7.9184	1451.25	42.7148
49702	7.9187	1442.24	44.005
49703	7.9198	1504.42	25.335
49704	7.9202	1351.04	26.091
49705	7.9226	1381.9	161.226
49706	7.9227	1394.46	22.8361
49707	7.9248	1481.64	287.684
49708	7.9253	1387.3	74.4466
49709	7.9265	1418.16	175.55
49710	7.9272	1298.58	55.3792
49711	7.9278	1500.88	99.7857
49712	7.9291	1267.2	106.233
49713	7.9306	1424.84	106.153
49714	7.9306	1613.23	61.1284
49715	7.9309	1435.16	124.735
49716	7.931	1646.8101	99.0429
49717	7.9322	1353.22	357.58

49718	7.9328	1327.23	34.6628
49719	7.9335	1323.52	160.425
49720	7.9357	1497.26	76.2051
49721	7.9361	1565.27	96.6835
49722	7.9372	1516.14	181.7
49723	7.9372	1358.85	84.391
49724	7.9383	1182.52	6.4904
49725	7.9384	1465.6	116.307
49726	7.9384	1329.97	255.761
49727	7.9387	1373.6899	67.8353
49728	7.9406	1381.17	174.206
49729	7.9409	1442.25	282.562
49730	7.9412	1232.4399	172.748
49731	7.9413	1383.8199	236.018
49732	7.9446	1369.27	316.925
49733	7.9448	1372.09	86.839
49734	7.9449	1549.8	25.0484
49735	7.9481	1433.29	80.7553
49736	7.9491	1336.78	256.813
49737	7.9492	1450.1899	285.664
49738	7.9495	1608.8	3.7028
49739	7.9498	1343.5	190.452
49740	7.9505	1529.5601	122.072
49741	7.9507	1348.9	235.992
49742	7.9508	1271.04	322.705
49743	7.9518	1390.34	224.451
49744	7.9526	1363.6	313.173
49745	7.9526	1369.84	192.197
49746	7.9528	1408.41	276.164
49747	7.9528	1375.8199	176.368
49748	7.9532	1422.53	194.164
49749	7.9532	1659.2	11.0052
49750	7.9537	1312.62	155.22
49751	7.9537	1432.58	48.4247
49752	7.9551	1467.92	129.531
49753	7.9563	1711.29	18.748
49754	7.9565	1386.5601	178.023
49755	7.9565	1577.04	223.835
49756	7.9581	1419.15	17.5069
49757	7.9616	1396.3199	106.032
49758	7.9645	1431.25	162.61
49759	7.9648	1332.26	220.979
49760	7.9652	1465.21	34.9905
49761	7.9661	1539.96	142.373
49762	7.9662	1352.23	149.258
49763	7.9668	1556.67	17.3458
49764	7.967	1550.8101	102.669
49765	7.9678	1516.4301	227.411

49766	7.9683	1343.1899	85.7711
49767	7.9685	1424.74	71.0182
49768	7.9708	1300.87	190.097
49769	7.9715	1460.11	295.005
49770	7.9719	1529.23	310.909
49771	7.9723	1613.09	20.9668
49772	7.9724	1495.3101	232.463
49773	7.9725	1495.9301	137.871
49774	7.9725	1605.4399	29.7397
49775	7.9727	1628.4	25.1303
49776	7.973	1319.5699	186.275
49777	7.9737	1599.08	24.2153
49778	7.9737	1581.5601	230.762
49779	7.9751	1200.16	5.6028
49780	7.9753	1177.67	7.0751
49781	7.9765	1629.61	187.638
49782	7.9766	1665.78	253.372
49783	7.9768	1376.1899	335.608
49784	7.9785	1401.29	332.295
49785	7.9787	1325.61	196.581
49786	7.9808	1466.92	101.751
49787	7.9823	1506.77	85.0744
49788	7.9823	1610.66	21.2789
49789	7.9826	1582.74	104.334
49790	7.9828	1316.23	142.753
49791	7.9834	1395.23	26.0859
49792	7.9853	1549.62	116.83
49793	7.9857	1401.3101	107.166
49794	7.986	1568.24	22.728
49795	7.9865	1258.04	257.344
49796	7.9886	1155.13	188.819
49797	7.9893	1423.59	85.8707
49798	7.9894	1434.27	91.4936
49799	7.9897	1384.9301	220.594
49800	7.9897	1486.04	217.926
49801	7.9899	1399.01	215.75
49802	7.9923	1481.75	296.663
49803	7.9927	1465.88	235.157
49804	7.993	1414.6	346.905
49805	7.9933	1339.75	250.518
49806	7.9939	1402.9	48.1172
49807	7.994	1465.4399	258.444
49808	7.9951	1426	81.4128
49809	7.9951	1603.89	24.8339
49810	7.9955	1482.73	73.3504
49811	7.9955	1421.83	79.524
49812	7.9955	1570.39	75.0915
49813	7.9967	1148.79	152.951

49814	7.9971	1497.72	30.8666
49815	7.9979	1592.5699	26.5841
49816	7.999	1262.61	19.4331
49817	8.0003	1511.15	235.198
49818	8.0006	1555.63	31.1424
49819	8.0024	1512.36	113.069
49820	8.0038	1300.58	17.9812
49821	8.005	1311.75	111.937
49822	8.0054	1413.65	28.5778
49823	8.0057	1629.49	25.5559
49824	8.0064	1624.2	22.276
49825	8.0066	1391.75	74.4216
49826	8.0078	1481.37	232.15
49827	8.0081	1527.16	79.1706
49828	8.0086	1499.04	75.961
49829	8.0097	1685.01	100.893
49830	8.0106	1328.66	180.75
49831	8.0119	1456.25	55.0493
49832	8.0142	1392.0601	56.066
49833	8.0162	1587.24	16.0487
49834	8.017	1501.6801	78.3665
49835	8.018	1520.29	5.8453
49836	8.0192	1325.9399	249.978
49837	8.0202	1364.48	175.284
49838	8.0218	1387.28	75.3007
49839	8.0229	1451.76	347
49840	8.024	1527.6801	81.9865
49841	8.0251	1394.46	193.484
49842	8.0262	1478.67	259.446
49843	8.027	1639.97	22.5577
49844	8.0287	1626.6801	15.823
49845	8.0295	1407.5	67.558
49846	8.0299	1560.21	105.963
49847	8.03	1611.8	187.122
49848	8.0308	1535.75	280.59
49849	8.0325	1270.4	193.57
49850	8.0335	1530.95	259.37
49851	8.0336	1463.04	37.1776
49852	8.0351	1417.3199	86.5665
49853	8.0358	1323.42	218.29
49854	8.0391	1518.12	231.895
49855	8.04	1369.92	296.393
49856	8.0402	1505.46	85.39
49857	8.0404	1501.55	98.2159
49858	8.0411	1653.87	113.812
49859	8.0418	1340.02	257.492
49860	8.0422	1668.71	17.8392
49861	8.0425	1430.48	5.0526

49862	8.0428	1649.25	19.4903
49863	8.0435	1577.58	32.7526
49864	8.0443	1531.33	143.134
49865	8.0448	1369.02	188.663
49866	8.0465	1522	85.1304
49867	8.0467	1449.42	73.6491
49868	8.0479	1755.5601	271.2
49869	8.048	571.138	49.7647
49870	8.0484	1589.47	77.4626
49871	8.0485	1280.17	142.065
49872	8.0493	1333.24	167.577
49873	8.0531	1563.27	90.9041
49874	8.0533	1500.46	111.399
49875	8.0537	1318.48	259.772
49876	8.0548	1461.3	81.0131
49877	8.0553	1625.5601	23.4162
49878	8.0553	1542.53	193.329
49879	8.0555	1245.1	82.9299
49880	8.0561	1367.79	235.296
49881	8.0585	1354.66	86.9685
49882	8.0589	1583.27	220.82
49883	8.0592	1582.89	38.0369
49884	8.0598	1388.87	139.111
49885	8.0629	1558.4	98.7471
49886	8.064	1331.8101	26.6099
49887	8.0642	1284.37	188.596
49888	8.0643	1414.58	185.159
49889	8.0655	1394.79	257.642
49890	8.0665	1473.5	147.373
49891	8.0667	1739.5699	10.7605
49892	8.0671	1448.28	218.211
49893	8.0681	1376.37	184.521
49894	8.0687	1421.74	304.462
49895	8.0718	1612.16	287.532
49896	8.0721	1417.84	13.1881
49897	8.0727	1344.84	55.9142
49898	8.0728	1403.8101	157.523
49899	8.0729	1579.84	312.454
49900	8.073	1584.13	20.2675
49901	8.0732	1451.9	165.071
49902	8.0735	1502.25	16.9643
49903	8.0746	1636.25	99.7074
49904	8.0751	1460.7	29.2545
49905	8.0753	1610.05	7.682
49906	8.0766	1182.35	257.625
49907	8.0768	1391.23	180.491
49908	8.078	1545.1	78.7451
49909	8.0794	1408.37	86.2106

49910	8.0806	1579.09	16.7014
49911	8.0815	1358.11	160.897
49912	8.0829	1456.71	305.054
49913	8.0848	1623.67	96.7119
49914	8.0851	1440.5601	32.9587
49915	8.0853	1209.45	83.3832
49916	8.0863	1424.47	254.34
49917	8.0873	1626.83	20.7242
49918	8.0875	1458.4301	265.399
49919	8.0886	1533.72	68.4194
49920	8.0902	1458.6	112.193
49921	8.0904	918.333	193.108
49922	8.0917	1336.85	237.761
49923	8.0917	1101.6801	308.346
49924	8.0929	1577.65	221.814
49925	8.0931	1390.39	72.5387
49926	8.0935	1436.36	80.5339
49927	8.0935	1314.33	80.5389
49928	8.0954	1663.5	96.3341
49929	8.0969	1473.4	33.2226
49930	8.0978	1633.6	127.931
49931	8.0982	1320.66	196.102
49932	8.099	1578.88	76.3105
49933	8.0996	1311.63	218.661
49934	8.1001	1434.2	34.7188
49935	8.1019	1461.54	26.7859
49936	8.1019	1235.85	38.8154
49937	8.1021	1516.3	301.314
49938	8.1079	1402.67	79.4189
49939	8.1088	1472.33	229.594
49940	8.109	1321.78	28.3402
49941	8.1126	1476.21	198.788
49942	8.1129	1546.38	309.07
49943	8.1132	1456.5	72.391
49944	8.1142	1414.84	60.9226
49945	8.1144	1412.1801	258.45
49946	8.1171	1389.51	17.8795
49947	8.1175	1410.17	101.505
49948	8.1179	1616.49	230.224
49949	8.118	1554.02	98.6071
49950	8.1191	1527.72	69.2786
49951	8.1192	1717.33	30.7935
49952	8.1196	1572.6801	23.8212
49953	8.1197	1572.6801	84.5431
49954	8.1206	1436.08	239.073
49955	8.1214	1565.08	332.191
49956	8.1218	1483.6	126.081
49957	8.1218	1451.77	251.1

49958	8.1218	1495.92	19.9911
49959	8.1228	1472.64	149.491
49960	8.1242	1539.64	83.7345
49961	8.1244	1519.77	68.514
49962	8.1246	1415.48	42.315
49963	8.1264	1546.53	78.6654
49964	8.1273	1617.45	21.5223
49965	8.1293	1583.41	84.6192
49966	8.132	1579.47	157.301
49967	8.1327	1642.17	22.6893
49968	8.1331	1365.83	81.4663
49969	8.1333	1596.04	20.4556
49970	8.1334	1484.1899	30.645
49971	8.1336	1384.26	238.972
49972	8.1338	1429	109.455
49973	8.1354	1531.72	234.697
49974	8.1359	1279.73	141.857
49975	8.1361	1344.09	189.596
49976	8.1363	1572.4399	28.6701
49977	8.1369	1468.34	72.1306
49978	8.1371	1397	238.499
49979	8.1372	1471.63	187.874
49980	8.1399	1435.38	192.923
49981	8.1407	1448.84	220.774
49982	8.1409	1595.4399	26.979
49983	8.1416	1497.26	233.692
49984	8.1417	823.274	2.3192
49985	8.1431	1402.35	251.083
49986	8.1449	1386.75	161.103
49987	8.1463	1372.64	238.559
49988	8.1467	1398.14	71.9436
49989	8.1472	1409.83	18.5519
49990	8.1484	1458.5	227.661
49991	8.1495	1468.52	335.659
49992	8.1499	1504.95	86.3204
49993	8.1514	1398.0601	113.272
49994	8.1517	1787.53	22.715
49995	8.1518	1458.79	88.0271
49996	8.1522	1489.26	24.2867
49997	8.1522	1487.72	12.5403
49998	8.1527	1394.71	21.1883
49999	8.1538	1673.79	33.1262
50000	8.1556	1463.88	312.441
50001	8.1557	1431.5	78.538
50002	8.1576	1617.59	69.8713
50003	8.1608	1429.28	25.622
50004	8.161	1692.9399	283.305
50005	8.1657	1528.5	226.94

50006	8.1657	1454.3199	78.5437
50007	8.1673	1431.77	72.476
50008	8.1681	1617.5699	8.4865
50009	8.1682	1350.85	148.06
50010	8.1695	1732.6899	19.9522
50011	8.1701	1705.6899	21.0877
50012	8.1727	1424.75	175.391
50013	8.1729	1616.12	225.202
50014	8.1762	1418.33	167.958
50015	8.1765	1596.84	36.0975
50016	8.1772	1557.83	245.611
50017	8.1784	-4.2403	137.359
50018	8.179	1436.7	12.6784
50019	8.1791	1644	25.521
50020	8.1796	1758.16	97.8027
50021	8.1813	1616.23	296.766
50022	8.1826	1458.52	22.5471
50023	8.1828	1181.95	261.477
50024	8.1828	1411.23	216.017
50025	8.1833	1232.62	193.652
50026	8.1856	1380.84	189.028
50027	8.1874	1508.59	52.1405
50028	8.1877	1418.73	216.877
50029	8.1881	1445.1899	74.212
50030	8.1882	1452.67	96.5282
50031	8.1885	1487.6801	191.988
50032	8.1894	1374.23	237.494
50033	8.1906	1712.54	289.459
50034	8.1908	1308.25	187.873
50035	8.1912	1642.9301	4.5454
50036	8.1934	1672.17	33.7
50037	8.1948	1694.52	2.0159
50038	8.1958	1606.39	224.982
50039	8.1963	1324.62	193.75
50040	8.1979	1467.28	32.3158
50041	8.1981	1506.01	108.839
50042	8.1991	1370.99	83.7895
50043	8.2001	1491.73	50.2748
50044	8.2013	1466.34	134.888
50045	8.202	1469.58	343.529
50046	8.2032	1685.0699	24.8296
50047	8.2035	1472.41	20.6238
50048	8.2037	1515.65	71.0275
50049	8.2065	1431.75	349.665
50050	8.2078	1512.3	158.603
50051	8.2089	1702.7	70.3893
50052	8.2091	1430.53	75.9179
50053	8.2094	1408.09	102.264

50054	8.2119	1543.35	94.1695
50055	8.212	1505.24	277.273
50056	8.2126	1461.47	188.926
50057	8.2131	1368.96	88.385
50058	8.2134	1527.14	160.449
50059	8.2135	1564.66	134.762
50060	8.2141	1447.71	159.231
50061	8.2154	1695.17	23.5014
50062	8.2154	1669.6	57.6011
50063	8.2158	1685.54	30.1469
50064	8.216	1467.34	110.135
50065	8.2165	1473.74	15.7392
50066	8.2177	1492.6801	82.8158
50067	8.2202	1553.87	338.038
50068	8.2203	1474.3	49.6841
50069	8.2206	497.33	44.4355
50070	8.2218	1437.7	132.099
50071	8.222	1519.41	75.0377
50072	8.2224	1489.87	349.66
50073	8.223	1453.6	33.2213
50074	8.2239	1400.4	236.575
50075	8.2268	1510.24	33.595
50076	8.228	1374.48	64.5032
50077	8.2283	1352.9301	7.9383
50078	8.2295	1592.3	98.3225
50079	8.231	1522.5699	85.9021
50080	8.2322	1491.29	79.5909
50081	8.2323	1490.7	0.2918
50082	8.2323	1587.6	29.7288
50083	8.2326	1479.87	270.462
50084	8.2329	1470.01	131.93
50085	8.2348	1687.92	23.2791
50086	8.2368	1481.27	224.433
50087	8.2406	1620.45	347.006
50088	8.242	1608.48	98.3318
50089	8.2433	1704.73	25.1201
50090	8.2438	1476.8199	42.2241
50091	8.2444	1489.6	144.531
50092	8.2461	1560.76	36.0366
50093	8.2476	1679.58	26.1471
50094	8.2483	1537.15	332.082
50095	8.2493	1544.09	220.93
50096	8.2497	1441.42	169.208
50097	8.2504	1379.12	188.155
50098	8.2506	1508.3199	80.7358
50099	8.2507	1563.36	161.572
50100	8.2508	1676.62	230.211
50101	8.2512	1612.52	223.146

50102	8.2564	1518.46	21.4321
50103	8.2566	1512.74	85.5665
50104	8.2566	1744.96	30.799
50105	8.2568	1549.41	279
50106	8.2575	1657.47	69.8552
50107	8.2575	1491.77	86.4128
50108	8.2576	430.275	46.7051
50109	8.2591	1337.63	186.602
50110	8.2592	1470.1899	253.849
50111	8.2597	1657.59	21.0589
50112	8.2602	1543.74	24.6545
50113	8.2603	1579.1801	15.5671
50114	8.2612	1392.6899	4.3253
50115	8.2639	1600.59	289.089
50116	8.2645	1484.5	92.9855
50117	8.2647	1537.3199	83.5675
50118	8.2655	868.434	28.0902
50119	8.2659	1379.76	305.14
50120	8.2671	1460.2	248.411
50121	8.2678	441.323	49.9116
50122	8.2681	1517.28	40.5443
50123	8.2696	1631.12	22.2161
50124	8.2723	1617.78	28.4625
50125	8.2723	1369.6	7.3249
50126	8.2731	1526.89	88.3302
50127	8.2738	1620.74	220.028
50128	8.2739	1451.62	184.908
50129	8.2741	1382.7	141.877
50130	8.2763	1585.21	112.694
50131	8.2763	1510.04	297.728
50132	8.2769	1424.7	316.884
50133	8.2796	1442.3	132.049
50134	8.2806	1451.16	151.554
50135	8.2808	1659.4	6.3492
50136	8.2821	1512.58	84.2376
50137	8.283	1341.11	23.2834
50138	8.2833	1545.1899	24.7893
50139	8.2846	1398.42	30.9626
50140	8.2875	1435.6	324.966
50141	8.288	1567.37	30.6641
50142	8.2918	1704.73	16.3056
50143	8.2922	1658.34	22.4026
50144	8.2925	1488.38	83.118
50145	8.2929	1451.28	73.574
50146	8.2946	1402.72	36.8014
50147	8.2947	1658.16	257.066
50148	8.2967	1777.52	22.2176
50149	8.2974	1651.83	28.098

50150	8.2978	1368.4399	25.9038
50151	8.2992	1533.14	69.1547
50152	8.2997	1427.73	300.413
50153	8.3004	1560.47	72.347
50154	8.3014	1521.34	277.611
50155	8.3044	1490.65	159.273
50156	8.3059	1401.86	85.6007
50157	8.3066	1622.61	329.112
50158	8.3078	1640.63	98.6335
50159	8.3079	1571.7	77.7359
50160	8.308	1384.42	186.517
50161	8.3082	1442.3101	81.0746
50162	8.3088	1455.1	72.2104
50163	8.3094	1414.7	18.8834
50164	8.3114	1489.13	109.001
50165	8.3115	1542.55	25.3526
50166	8.3147	1420.26	43.5497
50167	8.3151	1347.36	34.1084
50168	8.3152	1673.55	32.2418
50169	8.3163	1666.99	97.9952
50170	8.3185	1534.95	234.637
50171	8.3199	1712.29	19.0246
50172	8.3205	1462.9399	169.714
50173	8.3228	1464.04	186.859
50174	8.323	1500.05	320.903
50175	8.3239	1447.1801	224.15
50176	8.3241	1408.66	72.9289
50177	8.3242	1475.88	72.4416
50178	8.3297	1715.01	74.3335
50179	8.3334	1620.15	100.876
50180	8.3338	1434.52	5.0337
50181	8.3342	1641.38	99.0326
50182	8.3343	1571.47	350.141
50183	8.3349	1391.78	186.435
50184	8.3353	1439.03	186.666
50185	8.3355	1517.48	359.195
50186	8.3365	1623.58	297.681
50187	8.3369	1635.02	74.873
50188	8.338	1276.17	28.6827
50189	8.3382	1625.83	260.993
50190	8.3383	1524.17	309.686
50191	8.3396	1529.16	179.129
50192	8.3397	1500.12	33.3188
50193	8.3419	1546.59	70.397
50194	8.3426	1450.08	222.866
50195	8.3427	1417.92	20.862
50196	8.3449	1573.08	32.7339
50197	8.3483	1469.42	22.5907

50198	8.349	1519.86	70.9994
50199	8.349	1586.91	234.931
50200	8.3496	1628.28	87.5342
50201	8.3499	1597.1899	68.787
50202	8.3503	1603.4301	7.2202
50203	8.3511	1607.33	26.7482
50204	8.3517	1580.55	225.291
50205	8.3521	1592.77	113.863
50206	8.3532	1512.34	311.108
50207	8.3535	1182.05	256.512
50208	8.3536	1619.7	345.806
50209	8.3537	1271.9399	23.7797
50210	8.356	1652.12	27.7928
50211	8.3565	1669.5601	268.519
50212	8.3566	1611.88	22.9666
50213	8.3586	1731.87	224.443
50214	8.3591	1446.78	278.015
50215	8.3593	1602.8	42.6439
50216	8.3594	1370.78	9.0981
50217	8.3613	1521.6899	86.5301
50218	8.3629	1519.26	337.917
50219	8.3648	1377.03	6.4132
50220	8.3663	1686.17	100.202
50221	8.3663	1529.49	84.7264
50222	8.3664	1456.95	27.7022
50223	8.3671	1732.14	222.907
50224	8.3685	1399.58	252.372
50225	8.372	1685.21	269.825
50226	8.3739	1774.66	30.6305
50227	8.3759	1436.3199	161.594
50228	8.3759	1414.75	16.0233
50229	8.3788	1532.92	18.2701
50230	8.3823	1351.23	87.6921
50231	8.3824	1710.13	19.3266
50232	8.3826	1225.02	196.115
50233	8.383	1676.39	271.66
50234	8.3833	1746.4	19.0814
50235	8.3835	1508.96	77.6436
50236	8.3835	1709.7	18.0374
50237	8.3846	1414.76	252.77
50238	8.3849	1543.92	281.048
50239	8.385	1573.15	92.8543
50240	8.3854	1619.48	278.957
50241	8.3863	1504.36	24.6928
50242	8.3864	1500.89	78.0582
50243	8.3868	1589.8	263.103
50244	8.3882	1498.8199	233.492
50245	8.3909	1456.71	29.0821

50246	8.391	1542.23	164.872
50247	8.3918	1383.54	220.444
50248	8.3923	1553.8	348.607
50249	8.3939	1528.9301	90.8205
50250	8.3941	1371.33	48.7418
50251	8.3956	1680.0699	28.8914
50252	8.3963	1706.58	15.3101
50253	8.3968	1617.36	84.684
50254	8.3992	1764.6899	17.8631
50255	8.3994	1543.8199	91.7783
50256	8.3998	1495.28	224.543
50257	8.401	1697.6801	25.1707
50258	8.4012	1514.1	140.996
50259	8.4015	1638.51	75.8825
50260	8.4024	1686.62	24.147
50261	8.4029	1375.01	37.1143
50262	8.4029	1693.14	20.2531
50263	8.4035	1519.61	25.3521
50264	8.406	1570.38	35.561
50265	8.4079	1464.02	35.4189
50266	8.4086	1513.88	315.004
50267	8.4108	1501.26	23.5275
50268	8.4118	1653.48	329.22
50269	8.412	1639.42	124.524
50270	8.4122	1610.45	220.583
50271	8.4124	1472.5699	26.136
50272	8.4126	1787.05	285.5
50273	8.4156	1597.59	84.342
50274	8.4158	1686.54	98.2932
50275	8.4165	1476.37	19.4329
50276	8.4169	1762.4399	252.27
50277	8.4176	430.387	50.0754
50278	8.4181	1762.2	8.1536
50279	8.4182	1739.2	25.0262
50280	8.4187	1570.96	18.5181
50281	8.4211	1595.45	187.915
50282	8.4216	1575.15	42.7479
50283	8.4242	483.083	340.76
50284	8.4249	1671.47	234.52
50285	8.4279	1655.17	79.8126
50286	8.4285	1614.01	73.1341
50287	8.4297	1537.12	19.8616
50288	8.4298	1740.97	36.2728
50289	8.4299	1614.25	219.773
50290	8.4308	1627.17	21.8653
50291	8.4308	1662	34.1987
50292	8.4313	1524.85	280.335
50293	8.4322	1719.5601	28.4072

50294	8.4336	1649.09	278.757
50295	8.4337	1421.24	253.139
50296	8.4339	1599.28	75.5697
50297	8.4346	1403.96	147.858
50298	8.4374	1572.25	31.2816
50299	8.4379	1753.38	164.869
50300	8.4382	1658.1801	288.649
50301	8.4394	1499.8199	359.654
50302	8.4402	-6.0553	59.1857
50303	8.441	1465.6801	2.5503
50304	8.4411	1597.55	326.073
50305	8.446	1336.64	28.9323
50306	8.446	1362.41	148.365
50307	8.4465	1553.83	48.4702
50308	8.4488	1520.15	45.6446
50309	8.4493	1563.39	147.099
50310	8.4513	1454.97	236.692
50311	8.4525	1619.28	49.6193
50312	8.4529	1594.0699	86.7372
50313	8.4537	1679.83	113.451
50314	8.454	1349.59	27.8309
50315	8.4559	1579.62	192.591
50316	8.4563	1411.38	33.8024
50317	8.4571	1531.22	32.7685
50318	8.4572	1633.75	231.977
50319	8.4593	1701.17	26.9108
50320	8.4607	1451.88	169.257
50321	8.463	1611.7	26.0602
50322	8.467	1573.85	332.213
50323	8.469	1544.2	216.92
50324	8.4702	1581	147.007
50325	8.4702	1717.1899	303.171
50326	8.4722	1677.24	27.126
50327	8.4741	1487.89	50.4178
50328	8.4773	1420.97	39.2104
50329	8.4782	1594.2	252.472
50330	8.4785	1581.1801	102.303
50331	8.481	1678.9301	15.1359
50332	8.4825	1594.6801	77.2773
50333	8.4827	1495.13	281.661
50334	8.4836	1659.84	39.0101
50335	8.4859	1837.4	269.039
50336	8.4868	1644.9301	82.8296
50337	8.4879	1620.6899	278.044
50338	8.4896	1696.01	221.352
50339	8.4914	1660.24	322.101
50340	8.4922	1797.1	25.0786
50341	8.4923	1458.38	252.91

50342	8.4927	1551.34	33.8755
50343	8.4929	1254.64	26.8971
50344	8.4933	1672.01	288.22
50345	8.494	1496.45	161.895
50346	8.4942	1511.83	152.663
50347	8.4948	1656.5	23.0223
50348	8.4953	1501.85	47.6858
50349	8.4988	1723.89	259.027
50350	8.5001	1540.8101	64.0303
50351	8.5035	1751.63	265.532
50352	8.5047	1606.98	100.154
50353	8.5049	1660.5601	43.0121
50354	8.5077	1681.8	21.2629
50355	8.5085	1834.74	266.445
50356	8.5085	1678.9399	7.8341
50357	8.5101	1774.83	358.979
50358	8.5112	1688.76	290.727
50359	8.5123	1526.98	24.4102
50360	8.5128	430.309	49.1461
50361	8.5148	1522.22	157.752
50362	8.5154	1507.39	24.8733
50363	8.5159	1612.9301	90.169
50364	8.5163	1701.87	94.0992
50365	8.5169	1356.91	182.341
50366	8.5172	1708.26	22.6109
50367	8.5177	1590.73	66.1163
50368	8.5179	1756.1899	27.7322
50369	8.5179	1549.54	224.257
50370	8.5204	1752.28	73.996
50371	8.5204	1665.49	253.61
50372	8.5213	1799.62	284.215
50373	8.5218	1467.7	1.3448
50374	8.5219	1468.88	15.7495
50375	8.5225	1782.25	28.6125
50376	8.5226	1607.63	65.6726
50377	8.5234	1604.16	75.7404
50378	8.5244	1664.7	24.5416
50379	8.5255	1557.3	158.656
50380	8.5265	1506.67	162.468
50381	8.5273	1533.96	15.2974
50382	8.5275	1667.71	24.4356
50383	8.5277	1734.2	16.3154
50384	8.528	1441.64	221.928
50385	8.529	1536.02	144.454
50386	8.532	1181.99	254.665
50387	8.5334	1576.5	21.4062
50388	8.5334	1625.73	84.6219
50389	8.5349	1563.6	23.9549

50390	8.5356	511.336	45.8968
50391	8.539	1550.45	316.599
50392	8.5398	1487.48	248.441
50393	8.54	1714.3199	19.5954
50394	8.5411	1645.1	90.3121
50395	8.5414	1587.6	320.743
50396	8.5436	1680.03	33.7343
50397	8.55	1440.4399	277.183
50398	8.5525	1541.14	280.359
50399	8.5533	1666.8	33.7848
50400	8.5559	1545.12	345.936
50401	8.5563	1684.9301	26.6866
50402	8.5575	1800.89	273.429
50403	8.5585	1635.98	219.961
50404	8.5589	1746.01	256.645
50405	8.563	1595.27	88.8172
50406	8.5667	1506.24	18.0849
50407	8.5678	1658.36	75.5055
50408	8.5682	980.95	255.437
50409	8.5683	1692.17	230.837
50410	8.5689	1645.14	75.5553
50411	8.5689	1658.22	221.144
50412	8.569	1742.85	20.6289
50413	8.5692	1525.26	305.99
50414	8.5731	1561.83	250.038
50415	8.5734	1553.27	218.42
50416	8.5748	1702.58	33.2385
50417	8.5749	1502.92	182.461
50418	8.5819	1674.7	65.5198
50419	8.583	1695.6	79.0626
50420	8.5838	1557.54	13.5172
50421	8.5855	1627.34	26.2988
50422	8.5867	1668.61	30.3675
50423	8.5871	1671.75	83.2866
50424	8.5873	1625.73	14.6878
50425	8.5881	1805.9399	114.229
50426	8.5882	1565.84	58.3486
50427	8.589	1555.51	11.566
50428	8.5919	1586.61	144.283
50429	8.5977	1573.12	270.078
50430	8.604	1499.61	32.9385
50431	8.6068	1638.1899	79.8277
50432	8.6074	1573.04	289.749
50433	8.6089	1651.76	174.856
50434	8.6105	1714.9	22.7442
50435	8.6116	1462.6801	16.535
50436	8.6129	1786.61	17.7602
50437	8.6145	1559.42	159.853

50438	8.6183	1672.25	34.5676
50439	8.6191	1797.09	101.049
50440	8.6205	1756.78	80.9062
50441	8.6208	1668.38	217.927
50442	8.6228	1510.71	83.0707
50443	8.6232	1669.39	223.8
50444	8.6256	1662.96	96.4486
50445	8.6258	1565.0699	13.0192
50446	8.6262	1535.9	164.932
50447	8.6287	1421.22	285.097
50448	8.629	1666.49	31.6267
50449	8.6306	1483.16	33.2064
50450	8.6325	1698.67	66.7161
50451	8.6338	1505.78	251.559
50452	8.6351	1607.8101	184.766
50453	8.6368	353.46	31.5006
50454	8.6386	1410.6899	329.552
50455	8.6402	1627.96	3.8803
50456	8.6403	1613.64	219.609
50457	8.6407	1686.66	260.15
50458	8.6416	1415.04	180.798
50459	8.642	1572.11	164.189
50460	8.642	1552.66	58.1228
50461	8.6421	1592.13	328.323
50462	8.6434	1665.63	327.204
50463	8.6455	1696.45	258.061
50464	8.6457	1769.47	291.434
50465	8.6475	341.112	37.7556
50466	8.6485	1798.28	223.668
50467	8.6487	1587.09	48.8129
50468	8.6491	1765.84	222.143
50469	8.6491	1524.47	75.4129
50470	8.6528	1517.55	236.673
50471	8.6543	1687.11	225.597
50472	8.655	1704.46	334.208
50473	8.6558	1779.71	295.418
50474	8.6561	1665.83	164.017
50475	8.6563	1530.77	138.949
50476	8.6583	1744.75	122.96
50477	8.6585	1689.99	218.92
50478	8.6586	1558.64	281.492
50479	8.6588	1648.17	257.95
50480	8.6614	1517.0601	25.4457
50481	8.6623	1462.35	216.424
50482	8.6628	1643.0601	21.7771
50483	8.6629	1181.85	260.944
50484	8.6629	1645.9	60.6929
50485	8.6632	1654.74	105.14

50486	8.6651	1721.74	11.951
50487	8.6661	1425.6899	148.199
50488	8.6673	1703.3199	17.5284
50489	8.6692	1753.8199	1.0377
50490	8.6716	1639.48	278.177
50491	8.6731	1537.58	47.7666
50492	8.6748	1715.5	16.9176
50493	8.6748	1772.33	229.245
50494	8.6749	1465.1801	218.097
50495	8.6755	1553.0601	14.0485
50496	8.6759	1636.8	22.2078
50497	8.6791	1632.83	136.205
50498	8.6817	1778.12	19.2837
50499	8.6821	1689.97	83.6599
50500	8.685	1777.08	73.452
50501	8.6867	1578.1	23.4408
50502	8.6876	1764.41	221.973
50503	8.6908	1760.09	27.0967
50504	8.6911	1801.3199	21.8438
50505	8.6913	1690.21	291.279
50506	8.6927	1555.4	84.6935
50507	8.6948	441.274	45.3051
50508	8.7021	1749.35	4.291
50509	8.703	1662.34	332.525
50510	8.7036	1750.5601	300.718
50511	8.7037	1598.38	235.277
50512	8.7049	1716.62	217.393
50513	8.7053	1680.12	86.0872
50514	8.7063	1619.72	182.419
50515	8.7069	1608.91	323.909
50516	8.7069	1604.28	20.2158
50517	8.7093	714.089	33.6072
50518	8.7094	1749.63	259.908
50519	8.7101	1516.35	239.103
50520	8.7112	1802.71	221.561
50521	8.7142	1783.25	16.9919
50522	8.7144	1656.16	60.7695
50523	8.7145	1797.35	16.2353
50524	8.7149	1650.9399	8.8825
50525	8.7152	1714.0601	217.163
50526	8.7157	1597.95	308.248
50527	8.7163	1532.6801	73.7814
50528	8.7189	1562.79	331.586
50529	8.719	1516.33	107.476
50530	8.7212	1619.4399	32.7755
50531	8.7245	1403.4	181.251
50532	8.7255	1621.5699	81.756
50533	8.7352	1659.72	278.936

50534	8.7353	1587.51	323.438
50535	8.7354	1618.71	357.76
50536	8.7364	1536.63	187.803
50537	8.7366	1579.17	276.277
50538	8.7376	1678.0699	34.4636
50539	8.739	1760.8101	219.95
50540	8.7414	1750.59	81.2876
50541	8.7429	1797.1899	223.7
50542	8.7461	1773.79	29.8628
50543	8.7478	1620.72	49.1481
50544	8.7479	1705.72	25.9856
50545	8.7485	1605.8199	73.9717
50546	8.7489	1733.86	74.8627
50547	8.7497	1671.64	77.3459
50548	8.7511	1771.04	98.8011
50549	8.7514	1704.0601	147.606
50550	8.7515	1782.05	73.6605
50551	8.7523	1696.46	7.6368
50552	8.7546	1814.71	19.5903
50553	8.7563	1734.8199	254.743
50554	8.7568	1182.11	5.4498
50555	8.7576	1623.17	78.5751
50556	8.7591	1826.25	273.544
50557	8.7604	1824.52	74.9272
50558	8.7607	1511.52	237.43
50559	8.7631	1580	249.505
50560	8.764	1761.48	65.2527
50561	8.7644	1782.91	299.27
50562	8.7656	1613.08	251.799
50563	8.7671	1660.84	233.919
50564	8.77	1731.41	22.1503
50565	8.7721	1560.8101	14.9435
50566	8.7738	1667.28	153.63
50567	8.7748	1762.51	273.952
50568	8.7763	1683.23	226.603
50569	8.7764	339.695	38.73
50570	8.7771	1760.78	129.656
50571	8.7774	1570.47	67.6639
50572	8.7782	1634.24	55.5228
50573	8.7804	1780.39	89.9438
50574	8.7822	1535.9399	47.798
50575	8.7844	1555.84	36.0016
50576	8.7874	1514.09	88.5013
50577	8.7883	1624.15	221.389
50578	8.7886	1653.16	296.535
50579	8.7888	1701.49	296.286
50580	8.7902	1673.61	235.484
50581	8.7933	1537.24	82.5104

50582	8.7953	1726.3101	346.037
50583	8.7986	1663.83	77.2442
50584	8.7987	1738.26	273.625
50585	8.801	1718.0601	224.977
50586	8.8015	1706.12	40.5237
50587	8.8083	781.745	82.4938
50588	8.8093	419.372	52.3861
50589	8.8098	1559.61	29.566
50590	8.811	1582.85	36.1185
50591	8.8124	1766.75	22.5554
50592	8.8129	1617.73	250.361
50593	8.8134	1609.1	257.629
50594	8.8139	1731.15	323.949
50595	8.8144	1731.29	24.0132
50596	8.8148	1787.35	31.3417
50597	8.8148	1784.1899	113.552
50598	8.8151	1761.8101	276.671
50599	8.8151	1654.62	32.1072
50600	8.8154	1583.25	25.2667
50601	8.8159	1791.1	143.074
50602	8.817	1693.6899	223.531
50603	8.8184	1479.4	29.0768
50604	8.8189	541.76	47.6927
50605	8.819	1716.8101	262.085
50606	8.8201	1693.41	21.7705
50607	8.827	1612.87	81.4265
50608	8.8283	1602.2	25.2451
50609	8.8333	1798	24.1707
50610	8.8344	1768.87	113.338
50611	8.8354	1701.39	228.458
50612	8.8354	1671.34	76.4902
50613	8.8366	1612.97	316.034
50614	8.8372	1674.34	86.7799
50615	8.8377	1689.64	278.479
50616	8.8427	1685.73	278.987
50617	8.8434	1672.72	97.3305
50618	8.8435	1829.51	223.712
50619	8.8461	1721.05	226.551
50620	8.85	1629.53	19.4187
50621	8.8512	1697.03	218.506
50622	8.8521	1535.79	211.105
50623	8.8539	1687.85	299.181
50624	8.8541	1842.76	2.5177
50625	8.8548	1805.4301	257.178
50626	8.8561	1733.86	337.591
50627	8.8562	1728.79	25.2894
50628	8.8584	1669.0699	319.434
50629	8.859	1608.99	136.531

50630	8.8595	1565.55	23.1142
50631	8.8601	1593.3199	255.85
50632	8.8609	1713.41	25.708
50633	8.861	1718.95	166.958
50634	8.8626	1574.0601	40.8987
50635	8.8637	1676.1801	60.6817
50636	8.8693	1182.28	256.358
50637	8.8723	1767.3199	166.159
50638	8.8726	1519.91	23.0521
50639	8.8737	1741.78	257.581
50640	8.8764	1694.25	322.454
50641	8.8783	1731.14	22.1784
50642	8.8785	1614.34	82.3886
50643	8.8792	1597.01	1.8048
50644	8.881	1716.76	324.883
50645	8.8823	1828.75	230.207
50646	8.8833	1648.4399	28.4413
50647	8.8836	1431.3101	180.592
50648	8.8838	1715.63	66.6686
50649	8.8842	1542.03	31.9677
50650	8.8851	1674.09	221.612
50651	8.886	1672.73	77.7839
50652	8.8885	1644.85	34.2678
50653	8.8901	1703.22	152.617
50654	8.8907	1635.8199	17.8403
50655	8.8933	1804.13	224.251
50656	8.8948	1703.91	279.787
50657	8.8973	1597.22	19.4831
50658	8.9023	1515.67	13.7905
50659	8.9031	1719.4	225.643
50660	8.9037	1806.25	27.0263
50661	8.9038	1722.0699	31.2627
50662	8.9043	1769.5699	275.117
50663	8.9045	1681.71	14.934
50664	8.9055	1742.38	39.8236
50665	8.9074	1625.6899	148.5
50666	8.909	1721.86	100.497
50667	8.9092	1676.36	13.6109
50668	8.9107	1833.05	229.946
50669	8.913	1645.47	266.433
50670	8.9132	1580.75	1.8041
50671	8.9135	482.859	349.031
50672	8.9136	1182.23	261.373
50673	8.914	1609.63	223.414
50674	8.917	1583.97	292.918
50675	8.9185	1695.29	75.4982
50676	8.9197	1650.75	126.305
50677	8.9201	1558.4399	320.108

50678	8.921	1670.04	221.613
50679	8.9226	1818.89	71.4793
50680	8.924	1674.9	346.033
50681	8.9256	1782.27	224.204
50682	8.926	1722.76	79.031
50683	8.9264	1783.89	273.172
50684	8.9284	1767.61	232.55
50685	8.9312	1651.78	30.5067
50686	8.9316	1654.09	13.5752
50687	8.9338	1810.51	93.9071
50688	8.9347	1735.89	44.8367
50689	8.9371	1732.65	24.5667
50690	8.9382	1804.49	230.004
50691	8.9392	1525.0601	85.5648
50692	8.9405	1743.1	298.013
50693	8.9429	1337.16	25.6157
50694	8.944	1770.83	32.0825
50695	8.9458	1660.95	252.671
50696	8.9463	1636.72	250.021
50697	8.9508	1182.37	7.2596
50698	8.9529	1682.25	248.445
50699	8.953	1727.87	259.663
50700	8.9539	1767.36	8.2514
50701	8.9547	1742.63	220.975
50702	8.955	1797.71	171.327
50703	8.9578	1759.97	268.548
50704	8.9578	1830.71	78.0781
50705	8.9586	1720.09	324.536
50706	8.963	1691.02	7.8726
50707	8.9653	1756.41	330.214
50708	8.9669	1783.02	26.8829
50709	8.9678	1650.5	18.0997
50710	8.9689	1571.52	36.9164
50711	8.9691	1759.9301	297.314
50712	8.9748	1840.41	28.2235
50713	8.9749	1182.26	254.732
50714	8.9752	1819.1899	220.268
50715	8.9756	1728.77	17.8028
50716	8.9759	1778.42	88.5296
50717	8.9766	1619.5	4.7497
50718	8.9772	1795.41	22.3033
50719	8.9774	1736.83	222.405
50720	8.9788	1805.36	85.9228
50721	8.9802	1790.14	27.2152
50722	8.9831	1667.42	281.24
50723	8.9843	1755.4	77.6421
50724	8.9854	1790.3199	292.304
50725	8.9861	1670.5601	92.4632

50726	8.9893	1768.12	66.1988
50727	8.99	1707.62	280.218
50728	8.9927	1590.61	327.57
50729	8.9952	1755.52	24.4692
50730	8.998	1652.37	10.7224
50731	8.9983	1726.05	46.9021
50732	8.9996	1764.91	232.981
50733	9.0017	1667.47	251.708
50734	9.0032	1653.8	5.1486
50735	9.0038	1718.6899	179.415
50736	9.0041	1128.21	32.493
50737	9.0064	1725.9301	170.276
50738	9.0072	1644.46	20.2835
50739	9.0072	1788.54	26.0226
50740	9.008	1743.78	22.9076
50741	9.0101	1561.99	75.9814
50742	9.0134	584.181	47.3221
50743	9.0145	1797.34	36.7904
50744	9.0158	1791.21	220.161
50745	9.016	1746.87	90.4545
50746	9.0188	1803.4301	119.683
50747	9.021	1791.46	25.5505
50748	9.0224	1727.53	250.381
50749	9.0232	1781.53	225.527
50750	9.0234	1758	44.5018
50751	9.0262	1839.37	258.543
50752	9.0274	1805.15	88.2205
50753	9.0285	1800.92	144.193
50754	9.0332	1745.4	8.6261
50755	9.0336	1695.6899	80.3841
50756	9.0341	1723.33	328.658
50757	9.0385	870.197	27.027
50758	9.0416	1795.38	291.094
50759	9.0419	1826.16	121.504
50760	9.0422	1781.29	302.083
50761	9.043	1648.5601	326.38
50762	9.0432	1790.85	275.96
50763	9.044	1823.15	79.6024
50764	9.045	1765.76	168.655
50765	9.0455	1795.23	23.7846
50766	9.0456	1812.09	20.5936
50767	9.054	1752.22	101.377
50768	9.0561	1735.13	19.5273
50769	9.064	1732.8199	75.2168
50770	9.0652	1740.08	253.467
50771	9.0663	1823.46	82.8192
50772	9.0671	1756.8199	29.4325
50773	9.0676	1780.91	140.437

50774	9.0693	1730.78	79.909
50775	9.07	1742.6801	357.686
50776	9.0701	1789.86	62.3938
50777	9.0706	1747.1	256.579
50778	9.0712	1770.0601	5.6598
50779	9.0742	1732.97	4.0625
50780	9.0749	1839.49	224.174
50781	9.0754	1779.13	278.813
50782	9.0757	1566.36	75.4534
50783	9.0773	1751.29	341.597
50784	9.0775	1712.91	315.935
50785	9.0777	1772.35	225.643
50786	9.0779	1729.12	78.9352
50787	9.0801	1784.33	8.682
50788	9.0806	1831.34	121.156
50789	9.0817	1729	332.156
50790	9.0825	1618.98	44.2133
50791	9.0826	1575.04	24.8872
50792	9.0843	1797.23	232.739
50793	9.0867	1765.9301	227.691
50794	9.0884	1842.02	114.16
50795	9.0889	1835.02	258.561
50796	9.0914	1825.9399	189.119
50797	9.0928	1751.9399	352.35
50798	9.0939	1555.7	3.101
50799	9.0958	1746.4301	42.2865
50800	9.0998	1182.21	6.7593
50801	9.104	1540.66	23.7162
50802	9.1077	1795.5601	291.477
50803	9.1105	1823.34	135.338
50804	9.1112	1810.3	180.978
50805	9.1151	1811.37	173.032
50806	9.1165	1815.3	283.618
50807	9.117	1822.34	264.097
50808	9.1176	1700.6	217.785
50809	9.1242	1680.66	47.8962
50810	9.1248	1654.2	81.5591
50811	9.1267	1538.0601	20.9694
50812	9.127	1789.83	85.5827
50813	9.1271	1797.86	177.788
50814	9.1277	1746	194.573
50815	9.128	1648.28	17.9761
50816	9.1291	1822.78	255.57
50817	9.1295	1598.64	312.832
50818	9.1296	1829.34	177.391
50819	9.1319	1817.76	26.5359
50820	9.1335	1767.9301	10.592
50821	9.1351	1744.34	6.3963

50822	9.1357	1723.55	25.2954
50823	9.1363	1811.52	27.9663
50824	9.1379	1753.28	21.5138
50825	9.1384	1494.29	27.5705
50826	9.1393	1834.26	20.5532
50827	9.1413	1749.83	345.291
50828	9.1438	1819.71	23.5783
50829	9.1441	1455.72	25.7649
50830	9.1448	1664.41	27.2949
50831	9.1455	1681.74	22.6191
50832	9.1469	1655.02	324.102
50833	9.147	1753.23	33.7253
50834	9.1491	1763.1801	48.7188
50835	9.1503	1809.75	176.208
50836	9.1505	1739.9	142.486
50837	9.1525	1829.5699	77.7427
50838	9.1533	1610.9	36.5333
50839	9.1542	1819.71	295.742
50840	9.1548	1743.54	90.4287
50841	9.1551	1842.64	28.9623
50842	9.1564	1633.99	85.169
50843	9.1565	1729.8101	250.862
50844	9.1575	1834.46	19.2846
50845	9.1576	1640.67	37.2855
50846	9.1578	1650.67	306.598
50847	9.158	1654.79	81.1384
50848	9.158	1801.51	10.0375
50849	9.165	851.826	318.739
50850	9.1675	1821.12	256.284
50851	9.1677	1833.99	276.876
50852	9.1684	1769.46	78.6407
50853	9.1686	1693.14	15.6566
50854	9.1691	1317.73	354.522
50855	9.172	1696.98	237.942
50856	9.1726	1778.48	78.1956
50857	9.174	1776.55	32.0365
50858	9.1762	1832.15	167.316
50859	9.1777	1789.77	231.408
50860	9.1786	1833.17	180.318
50861	9.1788	741.926	34.8225
50862	9.1805	1814.36	13.9641
50863	9.1815	1741.66	85.2103
50864	9.1837	1841.98	225.835
50865	9.1879	1799.48	30.18
50866	9.1888	1759.8	288.853
50867	9.1889	1783.49	28.3047
50868	9.1959	1796.4301	78.1194
50869	9.1968	1836.48	17.9451

50870	9.1987	1817.15	94.089
50871	9.2022	1736.4301	33.0607
50872	9.2036	1778.85	238.169
50873	9.2072	1665.28	285.919
50874	9.2086	1802.14	84.2131
50875	9.2102	1797.52	27.7521
50876	9.2206	1744.87	79.5208
50877	9.2235	1754.96	216.482
50878	9.224	1765.28	17.6214
50879	9.2253	1800.79	156.537
50880	9.2293	1819.67	19.6197
50881	9.2298	1810.1801	227.06
50882	9.2302	1698.64	29.7598
50883	9.2316	1790.62	301.16
50884	9.2344	1733.4	50.3767
50885	9.2367	1681.25	95.064
50886	9.2391	1662.4399	18.5466
50887	9.2411	1703.21	13.5475
50888	9.2442	1764.47	24.5495
50889	9.2471	1729.26	32.4038
50890	9.2482	440.969	51.0596
50891	9.2488	1795.65	24.7142
50892	9.2515	1799.63	79.1672
50893	9.2526	1783.84	250.981
50894	9.254	1803.53	17.1997
50895	9.2543	1796.74	23.8136
50896	9.2545	1839.8101	170.524
50897	9.2615	1629.0601	79.1215
50898	9.2639	1551.1801	24.894
50899	9.2642	1817.74	224.541
50900	9.267	1800.85	26.5803
50901	9.2743	700.407	23.966
50902	9.2761	1709.13	326.202
50903	9.2808	1715.83	11.3097
50904	9.2811	1792.66	25.0606
50905	9.2823	1758.62	343.005
50906	9.2861	1750.8101	48.6457
50907	9.2863	1796.8199	139.855
50908	9.2864	1794.34	230.482
50909	9.288	1724.41	22.2999
50910	9.2889	1640.09	4.9585
50911	9.2923	1755.88	330.673
50912	9.2927	1745.9399	27.9263
50913	9.2933	1770.51	22.3621
50914	9.2943	1842.1801	113.813
50915	9.2953	1812.34	89.1876
50916	9.2963	1700.76	195.341
50917	9.2981	1805.86	218.075

50918	9.2997	1825.28	224.326
50919	9.3004	1834.78	268.781
50920	9.3006	822.177	31.872
50921	9.3015	1832.63	17.41
50922	9.3061	657.256	50.1825
50923	9.3062	1662.12	280.089
50924	9.3082	1830.9399	221.039
50925	9.3096	1839.77	167.67
50926	9.3111	1656.99	32.1007
50927	9.3218	1741.34	75.1356
50928	9.3222	1784.76	77.8758
50929	9.3258	1804.76	251.968
50930	9.3261	719.726	31.9036
50931	9.3267	1517.28	26.9817
50932	9.3279	1841.72	31.5009
50933	9.336	1837.63	222.845
50934	9.3377	1182.3	257.69
50935	9.3457	1778.83	323.806
50936	9.3468	1717.37	319.202
50937	9.3479	1710.11	74.9676
50938	9.3485	1654.28	27.68
50939	9.351	1771.42	141.353
50940	9.3514	1733.1801	88.3667
50941	9.3545	1814.1801	134.314
50942	9.3598	1692.27	313.046
50943	9.3628	1821.88	251.622
50944	9.3629	1825.65	25.8387
50945	9.3653	1712.58	351.192
50946	9.3654	1729.88	325.325
50947	9.3658	1703.11	331.699
50948	9.3681	1631.8101	18.4311
50949	9.3682	1707.9399	21.317
50950	9.3687	1842.02	112.705
50951	9.3717	1800.14	233.347
50952	9.3723	1803.0699	10.8033
50953	9.3736	1720.97	100.568
50954	9.3766	1760.9	19.4147
50955	9.379	1739.09	32.9468
50956	9.3822	1573.55	21.6563
50957	9.3852	1708.17	30.9935
50958	9.39	1784.86	253.953
50959	9.392	1791.48	18.3028
50960	9.3945	1524.76	313.938
50961	9.3985	1834.34	20.7842
50962	9.4012	1738.1	29.777
50963	9.4015	1729.65	85.5683
50964	9.4039	1765.8101	315.383
50965	9.4101	1815.72	78.331

50966	9.4169	1755.9301	146.007
50967	9.4178	1627.37	55.4775
50968	9.4191	1832.59	218.991
50969	9.421	1733.48	6.8931
50970	9.4272	1830.52	28.1139
50971	9.4307	1693.4399	85.6516
50972	9.4319	1792.3101	83.4899
50973	9.4355	1823.27	134.209
50974	9.4392	1842.73	168.563
50975	9.4403	1832.6899	92.0896
50976	9.4415	1726.05	29.9167
50977	9.4419	1695.39	84.3847
50978	9.4449	1744.95	307.398
50979	9.4472	1702.52	24.2395
50980	9.4474	1611.79	9.1842
50981	9.4503	1654.33	66.9831
50982	9.4512	1840.21	180.027
50983	9.4521	1181.97	256.557
50984	9.4599	1793.0699	8.0196
50985	9.4612	1793.3	6.1747
50986	9.4628	1771.04	30.9801
50987	9.4636	1816.51	78.8007
50988	9.465	1839.12	225.993
50989	9.4669	1787.46	77.9026
50990	9.4724	1643.6899	19.5117
50991	9.4727	1717.46	30.4575
50992	9.4794	1182.11	5.8309
50993	9.483	1552.48	259.398
50994	9.4883	1759.97	296.478
50995	9.4898	1842.61	169.149
50996	9.4953	1818.71	9.6912
50997	9.5005	1835.5699	221.195
50998	9.5006	1756.12	81.9797
50999	9.5024	1727.67	31.3719
51000	9.5028	441.102	52.9642
51001	9.5031	1731.36	26.9232
51002	9.5044	1825.55	100.494
51003	9.507	1814.27	218.234
51004	9.5096	1666.8199	288.216
51005	9.51	1833.85	19.5838
51006	9.5124	1839.46	275.308
51007	9.5163	1528.88	17.5746
51008	9.5164	1746.21	5.838
51009	9.519	1630.53	74.8215
51010	9.5209	1797.08	95.0119
51011	9.5215	1679	25.072
51012	9.5222	1794.41	33.8918
51013	9.5249	1807.16	218.728

51014	9.526	1744.66	296.296
51015	9.5271	1702.13	16.5915
51016	9.5287	1826.54	222.253
51017	9.5355	1808.26	136.525
51018	9.5361	1840.1	223.72
51019	9.5372	1785.63	143.088
51020	9.5382	1842.47	224.797
51021	9.5392	1842.47	187.05
51022	9.5396	1841.9301	137.59
51023	9.5404	1823.91	22.4636
51024	9.5423	1491.12	82.6449
51025	9.5426	1491.96	37.7271
51026	9.5493	1772.16	10.0276
51027	9.5502	1757.09	28.5621
51028	9.5531	1799.02	344.733
51029	9.5558	1841.4301	13.708
51030	9.5563	1791.0699	194.381
51031	9.5574	1781.78	83.8786
51032	9.5584	1829.4399	90.1426
51033	9.5606	1842.64	187.144
51034	9.5616	1821.65	20.5358
51035	9.5643	1633.86	78.1351
51036	9.5685	1836.9	222.369
51037	9.5789	1823.36	296.551
51038	9.5814	1760.85	93.7542
51039	9.5815	1816.3199	19.41
51040	9.5874	671.905	30.2508
51041	9.5893	1181.83	256.685
51042	9.5897	1812.62	235.849
51043	9.5897	1711.95	26.278
51044	9.6004	1812.73	9.8439
51045	9.6022	1817.6801	80.5211
51046	9.6056	1809.98	218.638
51047	9.6058	1796.95	77.46
51048	9.6133	1820.62	324.901
51049	9.6146	1829.36	4.6611
51050	9.6159	1840.0699	63.1728
51051	9.6186	1347.46	23.7205
51052	9.6193	1788.38	80.019
51053	9.6288	1799.36	12.8444
51054	9.6331	1705.42	17.6153
51055	9.6342	1819	6.7119
51056	9.6345	1655.21	312.93
51057	9.6427	1785.87	31.1357
51058	9.646	1836.05	226.968
51059	9.65	1836.59	358.875
51060	9.6512	1723.6899	40.9446
51061	9.6539	1842.8	49.1278

51062	9.6569	1813.26	7.4869
51063	9.6606	1571.3	315.89
51064	9.6624	1831.49	25.3335
51065	9.6633	1801.6801	140.282
51066	9.6683	654.79	31.2096
51067	9.6683	1784.04	36.1904
51068	9.6686	1759.62	33.9182
51069	9.6765	1837.3	19.2932
51070	9.679	1835.86	11.2177
51071	9.6793	1842.37	19.4779
51072	9.6805	1784.5	77.7481
51073	9.6823	1829.83	254.328
51074	9.6892	1826.6801	144.664
51075	9.6895	1824.01	22.7939
51076	9.6912	1772.6899	77.6717
51077	9.6919	1841.86	227.882
51078	9.6962	1842.6	225.861
51079	9.6982	1796.16	43.5443
51080	9.699	1817.25	79.804
51081	9.699	1784.42	61.3497
51082	9.7003	1805.34	85.9447
51083	9.7065	1182.1801	260.623
51084	9.7101	1800.61	218.767
51085	9.7103	1816.6801	73.511
51086	9.7129	1837.99	93.3341
51087	9.7186	1802.16	345.117
51088	9.7211	1815.04	9.4691
51089	9.7213	1811.98	253.682
51090	9.7245	1842.21	84.1845
51091	9.7257	1832.22	253.547
51092	9.7365	1814.25	24.4227
51093	9.7368	1839.45	234.538
51094	9.737	1833.6	79.6416
51095	9.737	1836.14	322.461
51096	9.7388	1822.62	25.9007
51097	9.7406	1842.38	223.1
51098	9.7411	1817.67	251.414
51099	9.7526	1772.58	30.7735
51100	9.7594	651.841	50.0333
51101	9.7602	1839.25	220.377
51102	9.7602	1822.97	33.2929
51103	9.7643	1837.84	26.5079
51104	9.7671	1803.78	324.554
51105	9.7683	1827.03	14.434
51106	9.7714	1842.64	225.694
51107	9.7786	1726.58	22.2187
51108	9.7906	1801.0699	29.1556
51109	9.8042	1817.47	347.997

51110	9.8045	1807.0601	78.2675
51111	9.805	1787.66	29.2037
51112	9.8269	1821.39	31.181
51113	9.8298	1831.7	46.5389
51114	9.8319	1834.8	16.1328
51115	9.8395	1741.95	250.778
51116	9.8413	1841.79	83.4961
51117	9.8452	1777.14	58.7403
51118	9.8457	1772.1899	32.1142
51119	9.8498	1835.16	79.8015
51120	9.852	1776.72	13.9634
51121	9.8588	1817.3	10.0709
51122	9.8621	291.033	34.1798
51123	9.863	1794.62	19.2448
51124	9.8674	1685.21	308.245
51125	9.869	1839.87	79.221
51126	9.8778	1817.87	140.341
51127	9.8787	1839.55	18.0775
51128	9.8798	1836.98	255.308
51129	9.8883	1796.76	323.997
51130	9.8886	1769.16	81.3874
51131	9.8968	1814.54	8.7966
51132	9.8977	1842.37	223.391
51133	9.9003	1803.5699	318.565
51134	9.9159	1760.25	86.8948
51135	9.9234	1839.78	220.319
51136	9.9289	1679.38	50.886
51137	9.9339	1707.5	34.7388
51138	9.9379	1838.29	38.9014
51139	9.9392	1658.95	17.8133
51140	9.9431	1830.8	252.327
51141	9.9433	1830.87	65.0116
51142	9.9436	1818.51	323.176
51143	9.9459	1818.9	295.167
51144	9.9503	1762.52	73.2735
51145	9.9536	1769.89	20.9595
51146	9.9561	1838.66	77.0072
51147	9.9566	1800.91	66.4828
51148	9.9584	1755.17	23.8517
51149	9.9621	1799.0699	13.8447
51150	9.9667	1631.02	276.791
51151	9.9687	1783.04	80.9607
51152	9.972	1807.53	33.723
51153	9.9749	1682.5	313.795
51154	9.9937	1730.23	47.6255
51155	9.9992	1842.02	84.588
51156	10.0057	1842.52	188.336
51157	10.0069	1837.08	76.6429

51158	10.0085	1819.01	356.149
51159	10.0151	1839.62	223.287
51160	10.0379	1820.77	26.1538
51161	10.0403	1840.29	254.675
51162	10.0436	1836.6	22.6293
51163	10.044	1839.92	46.7049
51164	10.0456	1594.03	31.465
51165	10.0548	1770.14	347.837
51166	10.0586	1661.9301	36.5308
51167	10.0639	1829.97	85.4229
51168	10.0655	1776.52	73.7658
51169	10.071	1837.4399	19.7295
51170	10.0749	1740.36	101.994
51171	10.0846	1840.83	255.434
51172	10.092	1703.9301	314.892
51173	10.0953	1787.02	29.826
51174	10.0989	1792.37	75.5738
51175	10.1083	1835.26	219.464
51176	10.1138	1790.39	295.829
51177	10.114	1419.87	337.044
51178	10.1157	1842.79	24.8155
51179	10.1209	1819.1801	26.2268
51180	10.1249	1749.3199	26.5899
51181	10.125	1836.55	96.2457
51182	10.1286	1837.6801	24.6781
51183	10.1318	1763.02	32.6172
51184	10.1334	1803.88	26.5252
51185	10.1375	1803.25	10.0206
51186	10.1408	376.163	42.9592
51187	10.149	1837.8	101.085
51188	10.1522	1840.4	82.6213
51189	10.1541	1839.1801	29.6754
51190	10.1732	1582.95	20.4276
51191	10.1788	1753.51	22.0598
51192	10.1821	1773.04	302.026
51193	10.1823	648.853	32.8399
51194	10.188	1822.4	31.3325
51195	10.1894	1841.62	219.612
51196	10.1997	1807.64	35.3274
51197	10.2008	1835.0601	21.0628
51198	10.2055	1804.47	299.815
51199	10.2097	1820.09	125.279
51200	10.2127	1817.13	7.8405
51201	10.2243	1809.47	27.6939
51202	10.251	1710.79	254.951
51203	10.2591	1789.42	31.8743
51204	10.2643	1799.1899	351.908
51205	10.2643	1809.13	251.525

51206	10.2854	1755.72	101.319
51207	10.2904	1818.23	110.111
51208	10.2916	1737.25	26.9658
51209	10.2921	1693.6801	31.7921
51210	10.3029	1795.22	14.4812
51211	10.3052	1796.8	30.7086
51212	10.3092	1811.2	30.6954
51213	10.3192	513.054	355.006
51214	10.3194	1808.3101	316.705
51215	10.3336	1781.88	83.2148
51216	10.3344	1820.84	35.9402
51217	10.3355	1819.26	31.6591
51218	10.3413	1842.08	63.5647
[truncated: 18,218 more chars]
